# Supplementary material for: A telomere-to-telomere genome assembly of koi carp (Cyprinus carpio) using long reads and Hi-C technology
Source: Gigascience. 2025 Aug 29;14:giaf087. doi: 10.1093/gigascience/giaf087 (PMC12395963; doi:10.1093/gigascience/giaf087)
Supplement: giaf087_Supplemental_Files [file giaf087_supplemental_files.zip › Revised Supplementary Table.pdf]

Supplementary Table S1. Summary of the data sequenced by multiple technologies.

Type	Reads Number	Total length (bp)	Genome depth*	N50 length of reads (bp)
ONT ultra-long reads	8,483,369	252,593,380,752	156.18	59,113
PacBio HiFi reads	14,177,272	223,462,120,509	138.17	15,820
Illumina short reads	467,017,946	70,052,691,900	43.31	150
Hi-C reads	1,461,710,676	219,256,601,400	135.57	150

* Calculated with the estimated genome size of 1.6 Gb.

Supplementary Table S2. ***k*** **-mer analysis.**

Kmer	Corrective K-mer Number	Peak Depth	Genome Size	Heterozygous Ratio (%)
17	56,607,066,906	35	1,617,344,768	0.45

Supplementary Table S3. The statistics of initial assembly.

Assembly method	Genome size	Contig N50 (Mb)	Contig number	GC (%)
NextDenovo (ONT)	1,996,759,600	12.79	527	37.20
Hifiasm (mixed assembly)	1,584,964,294	29.44	295	37.30

Supplementary Table S4. The statistics of the anchored chromosome length.

Chromosome	Size	GC content (%)	Gap number
A1	37,363,433	36.95	1
A2	35,276,186	37.06	4
A3	39,467,364	38.29	0
A4	39,627,451	37.96	1
A5	43,340,186	37.10	0
A6	31,526,527	36.79	1
A7	47,545,056	37.48	0
A8	27,724,135	36.91	0
A9	32,589,843	37.20	1
A10	24,985,029	37.15	0
A11	26,363,430	37.03	1
A12	27,790,684	36.95	0
A13	30,220,085	37.06	0
A14	27,810,301	37.31	1
A15	28,031,689	37.50	0
A16	30,707,738	37.18	0
A17	27,689,367	37.00	0
A18	30,594,132	37.36	0
A19	27,496,436	37.07	0
A20	28,563,607	37.22	0
A21	24,242,226	36.93	0
A22	20,682,367	37.40	0
A23	25,889,515	37.46	0
A24	25,066,367	36.86	0
A25	20,930,763	36.74	0
B1	37,444,583	36.86	2
B2	31,458,436	37.00	1
B3	48,957,380	37.86	0
B4	34,063,379	38.04	1
B5	37,834,669	36.81	0
B6	31,834,917	36.78	0
B7	44,250,487	37.36	0
B8	30,401,772	36.98	0
B9	33,844,207	37.00	0
B10	24,405,108	36.80	0
B11	28,037,105	37.87	1
B12	28,284,847	36.86	0
B13	29,404,086	37.20	1
B14	29,011,138	37.06	0
B15	28,314,851	37.29	0
B16	33,679,505	37.18	0
B17	29,436,892	36.87	0
B18	30,900,996	37.16	0
B19	29,988,930	37.07	2
B20	29,173,131	37.17	2
B21	29,326,853	37.09	1
B22	39,792,300	36.97	0
B23	27,936,248	37.23	0
B24	25,681,486	36.60	0
B25	23,776,895	36.47	1

Supplementary Table S5. The identified telomeres in CC 4.0 genome assembly.

Chromosomes	Length	Ends identified with telomeres	Total counts of telomere repeats at the left end	Total counts of telomere repeats at the right end
A1	37,392,995	both	1,098	1,023
A2	32,411,761	left	988	30
A3	39,515,933	left	1,356	6
A4	39,657,765	right	89	1,687
A5	43,375,910	both	1,134	1,333
A6	31,543,561	both	933	1,083
A7	47,587,856	both	1,178	973
A8	27,729,046	both	802	1,170
A9	32,625,473	both	1,700	934
A10	25,003,137	left	168	63
A11	28,046,457	both	1,329	1,258
A12	27,830,011	both	959	1,016
A13	29,427,277	both	976	859
A14	27,825,823	both	1,445	894
A15	28,023,325	both	282	1,283
A16	30,726,694	left	1,439	37
A17	27,720,301	both	1,151	1,407
A18	30,615,592	right	75	1,023
A19	27,522,268	left	1,108	76
A20	28,771,360	both	622	935
A21	24,270,653	left	594	74
A22	20,702,622	left	1,300	10
A23	25,925,873	both	1,111	1,286
A24	25,362,794	both	1,078	1,121
A25	20,952,439	both	171	1,159
B1	37,487,474	both	1,031	1,272
B2	31,462,806	both	1,448	1,245
B3	49,015,229	both	1,112	539
B4	34,083,675	both	123	157
B5	37,870,630	both	636	1,446
B6	31,850,965	right	73	313
B7	44,348,951	both	363	1,537
B8	30,447,840	both	792	1,349
B9	33,877,285	both	2,048	1,361
B10	24,421,637	both	329	1,447
B11	26,384,604	both	693	882
B12	28,308,551	left	327	39
B13	30,246,702	left	832	21
B14	29,023,126	both	696	387
B15	28,341,433	right	63	1,439
B16	33,708,850	both	361	505
B17	29,452,226	both	337	1,643
B18	30,958,292	both	1,068	159
B19	28,142,417	right	29	1,138
B20	29,194,876	both	1,419	908
B21	29,355,585	both	1,788	1,310
B22	39,823,555	both	1,337	1,442
B23	27,965,732	right	99	1,192
B24	25,707,214	left	1,613	26
B25	23,812,905	right	90	1,371

Supplementary Table S6. General statistics of repeats in CC 4.0 assembly.

Item	Repeat Size	% of genome
Tandem repeats	121,176,538	7.79
Interspersed repeats	620,467,484	39.88
Total	696,414,897	44.76

Note: Some elements may partially overlap with another element domain.

Supplementary Table S7. The summary of interspersed repeat contents in CC 4.0 assembly.

Type	Repbase TEs		TE protiens		*De novo*		Combined TEs	
	Length (bp)	% in genome	Length (bp)	% in genome	Length (bp)	% in genome	Length (bp)	% in genome
DNA	210,528,456	13.53	29,590,595	1.90	320,617,611	20.61	403,583,491	25.94
LINE	63,724,921	4.10	54,985,051	3.53	135,507,440	8.71	173,469,599	11.15
SINE	6,956,258	0.45	0	0.00	10,399,039	0.67	16,731,955	1.08
LTR	49,988,622	3.21	41,613,179	2.67	157,797,873	10.14	175,020,590	11.25
Unknown	0	0.00	0	0.00	1,749,830	0.11	1,749,830	0.11
Total	317,262,253	20.39	125,947,359	8.10	564,272,319	36.27	620,467,484	39.88

Note: This statistical table does not contain Tandem Repeats, some elements may partly include another element domain.
*Combined: the non-redundant consensus of all repeat prediction/classification methods employed.
LINE, long interspersed nuclear elements; SINE, short interspersed nuclear elements; LTR, long terminal repeat.

Supplementary Table S8. Non-coding RNAs in CC 4.0 assembly.

Type		Copy	Average length (bp)	Total length (bp)
**miRNA**		4,026	89	359,360
**tRNA**		24,096	76	1,829,538
**rRNA**				
	18S	38	288	10,963
	28S	82	208	17,025
	5.8S	8	110	879
	5S	7,566	112	844,333
**snRNA**				
	CD-box	263	130	34,284
	HACA-box	133	154	20,495
	splicing	2,853	137	390,505

Supplementary Table S9. Summary of gene function annotation.

Item	Count	Percentage (%)
All	50,187	100.00
Annotation	49,362	98.36
KEGG	42,930	85.54
NR	49,075	97.78
Swissprot	44,531	88.73
GO	33,776	67.30
TrEMBL	49,066	97.77
Interpro	48,488	96.61

All, all annotated genes; Annotation, gene products could be annotated by at least one of the databases; KEGG, Kyoto Encyclopedia of Genes and Genomes; NR, Non-Redundant Protein Sequence Database; Swissport, Swiss-Prot Protein Knowledgebase; GO, Gene Ontology; TrEMBL, Translation of European Molecular Biology Laboratory; Interpro, Integrative Protein Signature Database

Supplementary Table S10. The gene expression matrix.

Gene ID	Fin	Scale	Symbol	Description	Refseq zebrafish id
B23G0250	11,479.89	933.17	KRT8	Keratin, type II cytoskeletal 8	NM_131156.2
A21G0110	8,803.60	5,446.02	RPL37	60S ribosomal protein L37	NM_001002069.4
B17G0058	8,176.05	186.90	LOC113083305	A0A6P6NME8_CARAU oleosin-B4-like isoform X1	
B9G0013	5,898.04	3,736.61	RPL31	60S ribosomal protein L31	NM_001040052.2
A3G1115	5,712.29	1,566.01	RPS2	40S ribosomal protein S2	XM_005163871.5
B20G0925	5,673.08	2,813.53	RPS29	40S ribosomal protein S29	NM_212953.1
B24G0523	5,484.13	5,643.10	LIPO	Lipocalin	NM_213634.1
A23G0220	5,334.39	316.43	KRT8	Keratin, type II cytoskeletal 8	NM_131156.2
A13G0549	5,325.12	1,862.54	SH2D4B	SH2 domain-containing protein 4B	XM_005156807.5
A20G0429	5,307.95	1,967.26	RPS7	40S ribosomal protein S7	NM_200752.1
B5G0895	4,918.19	1,799.24	RPL12	60S ribosomal protein L12	NM_201584.4
A24G0464	4,593.72	1,521.67	LIPO	Lipocalin	NM_213634.1
B11G0792	4,307.20	2,175.75			NM_001243178.1
A7G0322	4,205.68	2,811.33	RPS20	40S ribosomal protein S20	NM_213204.1
B13G0358	4,025.11	1,261.32			XM_005156807.5
B20G0514	3,953.74	1,639.92	RPS7	40S ribosomal protein S7	NM_200752.1
B5G0881	3,807.83	1,704.42	RPL7A	60S ribosomal protein L7a	NM_200047.1
B19G0136	3,691.38	206.33	K1C1	Keratin, type I cytoskeletal 50 kDa	NM_001003445.1
B19G0420	3,684.86	1,553.58	RPL14	60S ribosomal protein L14	NM_001002866.2
B23G0265	3,656.21	2,209.45	RPS21	40S ribosomal protein S21	NM_201191.1
A2G0553	3,637.51	1,332.84	RPS8	40S ribosomal protein S8	NM_214793.1
B2G0745	3,613.54	1,514.58	RPS8	40S ribosomal protein S8	NM_214793.1
B6G0120	3,583.57	2,013.52	RPS27A	Ubiquitin-40S ribosomal protein S27a	NM_200502.2
B14G0247	3,516.60	1,883.88	RPL39	60S ribosomal protein L39	NM_001002078.3
A19G0465	3,493.38	1,804.20	RPL11	60S ribosomal protein L11	NM_001002139.1
A3G0800	3,470.94	1,213.37	RPL3	60S ribosomal protein L3	NM_001001590.1
A21G0674	3,453.24	1,741.27	RPS14	40S ribosomal protein S14	NM_200026.1
B7G0284	3,430.89	950.63	RPS20	40S ribosomal protein S20	NM_213204.1
A17G0464	3,426.91	1,471.59	RPL13A	60S ribosomal protein L13a	NM_212784.1
B11G0165	3,411.47	181.13	DAZAP1	DAZ-associated protein 1	NM_213523.2
B3G0596	3,360.64	3,236.97	ACTBB	Actin, cytoplasmic 2	NM_181601.5
B7G0151	3,290.87	1,180.47	RPL13	60S ribosomal protein L13	NM_198143.1
B6G0159	3,233.92	1,543.67	RPSA	40S ribosomal protein SA	NM_201052.1
A19G0462	3,192.93	1,320.97	EEF1A	Elongation factor 1-alpha	NM_131263.1
B7G0981	3,138.74	1,085.42	RPLP2	60S acidic ribosomal protein P2	NM_001100436.2
A9G0015	3,094.76	916.80	RPL31	60S ribosomal protein L31	NM_001040052.2
A19G0031	3,049.88	2,417.47	RPS18	40S ribosomal protein S18	NM_001423550.1
B3G0804	3,047.96	1,953.84	RPL23	60S ribosomal protein L23	NM_200732.1
B1G0001	2,991.03	1,929.18	RPL24	60S ribosomal protein L24	NM_173235.3
A12G0141	2,938.42	1,812.92	RPL38	60S ribosomal protein L38	NM_001002486.2
B22G0967	2,933.01	1,391.06	RPL32	60S ribosomal protein L32	NM_001040043.3
A8G0460	2,887.86	1,060.66	RPL10A	60S ribosomal protein L10a	NM_199636.1
B21G0421	2,849.26	1,009.05	RPL23A	60S ribosomal protein L23a	NM_001001593.2
A14G0684	2,827.20	72.17	LYG	Lysozyme g	NM_001002706.1
B1G0740	2,774.74	1,476.58	RPS6	40S ribosomal protein S6	NM_001003728.1
A11G0703	2,763.25	1,514.60	DIRAS1	GTP-binding protein Di-Ras1	NM_001110121.1
A2G0605	2,703.22	1,641.04	RPL7	60S ribosomal protein L7	NM_213644.2
A10G0282	2,702.08	2,220.70	RPS25	40S ribosomal protein S25	NM_200815.1
A11G0755	2,606.58	1,860.64	HOXC13B	Homeobox protein Hox-C13b	NM_001105526.1
A22G0633	2,586.93	1,041.60	RPL32	60S ribosomal protein L32	NM_001040043.3
A20G0038	2,577.24	1,833.41	RPS29	40S ribosomal protein S29	NM_212953.1
A5G0610	2,573.02	1,087.96	RPL7A	60S ribosomal protein L7a	NM_200047.1
A19G0853	2,572.46	930.92	RPLP1	60S acidic ribosomal protein P1	NM_200029.1
B23G0657	2,509.47	181.22	CSL3	L-rhamnose-binding lectin CSL3	

B3G0640	2,485.50	1,159.06	RPS11	40S ribosomal protein S11	NM_213377.1
A6G0857	2,466.85	1,297.06	RPS26	40S ribosomal protein S26	NM_200742.1
B2G0787	2,461.82	1,554.67	RPS28	40S ribosomal protein S28	NM_213034.3
B17G0435	2,429.41	877.33	RPL13A	60S ribosomal protein L13a (Fragment)	NM_212784.1
B7G0379	2,426.28	1,061.75	RPS4X	40S ribosomal protein S4, X isoform	NM_001005589.1
B6G0951	2,414.00	758.09	RPS10	40S ribosomal protein S10	NM_201146.1
A14G0497	2,386.16	1,013.40	RPL26	60S ribosomal protein L26	NM_213113.2
A1G1087	2,381.06	1,148.36	RPS3A	40S ribosomal protein S3a	NM_200059.2
B2G0065	2,366.68	826.01	UBA52	Ubiquitin-60S ribosomal protein L40	NM_001037113.2
A16G0647	2,362.86	629.02	S100A10	Protein S100-A10	NM_213003.1
B21G0286	2,353.97	1,029.77	RPS14	40S ribosomal protein S14	NM_200026.1
B11G0037	2,346.65	1,238.59	HDHD5	Haloacid dehalogenase-like hydrolase domain-containing 5	NM_001105526.1
A19G0050	2,330.08	1,639.30	RPL15	60S ribosomal protein L15	NM_001003447.1
A9G0045	2,306.05	646.84			NM_001130697.1
A6G0940	2,289.37	996.91	RPS10	40S ribosomal protein S10	NM_201146.1
A5G0052	2,283.78	1,206.50	RPLP0	60S acidic ribosomal protein P0	NM_131580.2
A8G0078	2,247.88	1,002.75	RPL6	60S ribosomal protein L6	NM_001328586.1
B7G0900	2,214.55	168.58	TXN	Thioredoxin	NM_001002461.1
B18G0507	2,197.89	1,017.36	RPL4-B	60S ribosomal protein L4-B	NM_213107.1
A23G0233	2,189.96	844.65	RPS21	40S ribosomal protein S21	NM_201191.1
A2G0513	2,179.29	1,559.10	RPS28	40S ribosomal protein S28	NM_213034.3
B12G0130	2,157.40	2,449.03	RPL38	60S ribosomal protein L38	NM_001002486.2
B3G1064	2,111.11	1,056.83	RPL19	60S ribosomal protein L19	NM_213208.1
B1G0562	2,105.05	743.95	TPT1	Translationally-controlled tumor protein homolog	NM_198140.1
A23G0463	2,095.23	906.43			NM_001423401.1
A9G0298	2,071.14	1,307.20	RPL8	60S ribosomal protein L8	NM_200713.1
B2G0974	2,063.25	762.30	RPL5	60S ribosomal protein L5	NM_199756.2
A11G0505	2,033.72	1,833.72	SPOPLB	Speckle-type POZ protein-like B	NM_001037677.2
A3G0798	2,014.82	916.34	RPL27	60S ribosomal protein L27	NM_199724.1
B2G0691	1,984.63	1,066.11	RPL7	60S ribosomal protein L7	NM_213644.2
A6G0146	1,957.21	717.35	RPSA	40S ribosomal protein SA	NM_201052.1
B23G0366	1,948.86	1,261.73	RPL10	60S ribosomal protein L10	XM_068216691.1
B14G0502	1,935.50	724.50	RPL26	60S ribosomal protein L26	NM_213113.2
B19G0245	1,931.38	1,159.87	RPS27	40S ribosomal protein S27	NM_001159528.1
A6G0855	1,896.93	668.98	RPLP2	60S acidic ribosomal protein P2	NM_212743.1
B3G1298	1,881.38	792.64	RPS2	40S ribosomal protein S2	XM_005163871.5
B13G0940	1,870.18	7,652.00			NR_145818.1
A18G0723	1,854.59	913.73	RPS3	40S ribosomal protein S3	NM_201153.1
B14G0655	1,843.86	1,397.82	CD74	H-2 class II histocompatibility antigen gamma chain	NM_131590.2
A7G0199	1,819.55	1,199.30	RPL13	60S ribosomal protein L13	NM_198143.1
B18G0772	1,795.54	786.02	RPS3	40S ribosomal protein S3	NM_201153.1
B16G0448	1,762.30	828.55	RPS9	40S ribosomal protein S9	NM_200852.3
A6G0514	1,757.60	912.75	RPL5	60S ribosomal protein L5	NM_001002106.1
B3G1008	1,741.62	608.78	RPS15A	40S ribosomal protein S15a	NM_212762.1
A18G0085	1,740.50	878.29	RPL35A	60S ribosomal protein L35a	NM_001002487.1
A21G0118	1,698.85	1,022.91	RPL28	60S ribosomal protein L28	NM_201061.2
B5G1188	1,695.30	700.17	RPL36A	60S ribosomal protein L36a	NM_001202510.1
A1G1072	1,691.31	648.21	RPL9	60S ribosomal protein L9	XM_005170907.5
A24G0396	1,686.36	742.20	RPL21	60S ribosomal protein L21	NM_001002155.1
B5G0053	1,684.34	823.19	RPLP0	60S acidic ribosomal protein P0	NM_131580.2
B14G0804	1,650.42	140.38	TYBA	Thymosin beta-a	
B10G0632	1,642.94	587.66	RPS25	40S ribosomal protein S25	NM_200815.1
B19G0069	1,641.20	571.70	RPLP1	60S acidic ribosomal protein P1	NM_200029.1
A1G0407	1,638.35	1,013.86	RPS6	40S ribosomal protein S6	NM_001003728.1
B18G0077	1,630.38	826.99	RPL35A	60S ribosomal protein L35a	NM_001002487.1

B23G0315	1,613.69	616.02	NACA	Nascent polypeptide-associated complex subunit alpha	NM_001193352.1
B14G0522	1,612.54	708.81	GNB2L1	Guanine nucleotide-binding protein subunit beta-2-like 1	NM_131444.1
B15G0788	1,611.56	679.97	RPS5	40S ribosomal protein S5	NM_173232.1
A19G0305	1,585.68	840.38	RPL30	60S ribosomal protein L30	NM_200028.2
A12G0360	1,577.18	7.90	ROHU_031284	A0A498LWG7_LABRO Uncharacterized protein	
A11G0746	1,575.94	583.99	UBE2V1	Ubiquitin-conjugating enzyme E2 variant 1	NM_001105526.1
B3G0963	1,563.82	440.13	RPL27	60S ribosomal protein L27	NM_199724.1
B6G0869	1,523.67	607.02	RPLP2	60S acidic ribosomal protein P2	NM_212743.1
B6G0510	1,507.45	606.12	RPL5	60S ribosomal protein L5	NM_001002106.1
B9G0079	1,506.24	1,658.95	TYB11	Thymosin beta-11	NM_001130697.1
A23G0515	1,496.80	642.74	RPS26	40S ribosomal protein S26	NM_200025.1
A3G0552	1,496.78	626.67	RPS11	40S ribosomal protein S11	NM_213377.1
A7G0817	1,476.09	709.20	RPS13	40S ribosomal protein S13	NM_001002079.1
A7G0337	1,469.51	950.86	RPL34	60S ribosomal protein L34	NM_201122.1
A25G0777	1,466.66	817.59	RPL27A	60S ribosomal protein L27a	
B8G0210	1,454.40	437.80	PPIA	Peptidyl-prolyl cis-trans isomerase	NM_212758.2
B22G0133	1,451.90	769.87	RPL36	60S ribosomal protein L36	NM_212952.2
A6G0109	1,442.97	677.82	RPS27A	Ubiquitin-40S ribosomal protein S27a	NM_200502.2
A15G0159	1,418.33	563.31	RPS5	40S ribosomal protein S5	NM_173232.1
A20G0034	1,410.91	592.98	FAU	FAU ubiquitin-like and ribosomal protein S30	
B8G0577	1,396.50	1,123.86	RPL10A	60S ribosomal protein L10a	NM_199636.1
A14G0516	1,387.35	492.01	GNB2L1	Guanine nucleotide-binding protein subunit beta-2-like 1	NM_131444.1
B19G0646	1,352.71	527.53	RPL30	60S ribosomal protein L30	NM_200028.2
A7G0400	1,345.94	610.96	RPS4X	40S ribosomal protein S4, X isoform	NM_001005589.1
A3G0903	1,344.73	1,028.81	RPL19	60S ribosomal protein L19	NM_213208.1
B11G0439	1,297.47	903.26	ADNP	Activity-dependent neuroprotector homeobox protein	NM_001037677.2
A2G1162	1,263.20	550.75	UBA52	Ubiquitin-60S ribosomal protein L40	NM_001037113.2
B16G0695	1,233.51	198.56	S10I	Ictacalcin	
B11G0117	1,228.72	664.86	DTNBP1	Dysbindin	NM_001001819.2
A21G0530	1,227.77	556.40	RPL23A	60S ribosomal protein L23a	NM_001001593.2
B19G0479	1,219.01	861.17	EEF1A	Elongation factor 1-alpha	NM_131263.1
B9G0327	1,211.98	714.36	RPL8	60S ribosomal protein L8	NM_200713.1
B11G0101	1,207.57	706.46	X-ELEMENT\ORF2	Probable RNA-directed DNA polymerase from transposon X-element	NM_001110121.1
B1G0469	1,206.25	144.15	TYB11	Thymosin beta-11	
B1G0401	1,186.04	571.94	RPS3A	40S ribosomal protein S3a	NM_200059.2
B8G0997	1,180.85	358.05	RPL6	60S ribosomal protein L6	NM_001328586.1
B8G0083	1,178.90	616.52	YBX1	Y-box-binding protein 1	NM_131620.1
B4G0041	1,172.21	433.74	RPL18A	60S ribosomal protein L18a	NM_201060.1
B23G0544	1,146.34	461.41	RPS26	40S ribosomal protein S26	NM_200025.1
B7G0809	1,136.54	475.92	RPS13	40S ribosomal protein S13	NM_001002079.1
B7G0315	1,133.34	1,061.36	RPL34	60S ribosomal protein L34	NM_201122.1
B13G0856	1,121.61	70.32	LYST	Lysosomal-trafficking regulator	
A1G0569	1,121.41	530.45	TPT1	Translationally-controlled tumor protein homolog	NM_198140.1
B1G0383	1,103.60	598.40	RPL9	60S ribosomal protein L9	XM_005170906.5
A1G0654	1,103.08	222.79	TYB11	Thymosin beta-11	
B16G0921	1,101.47	476.41	RPL18	60S ribosomal protein L18	NM_001003432.2
B20G0927	1,100.36	528.06	FAU	Ubiquitin-like protein FUBI	

A13G0083	1,094.77	161.33	ATE1	Arginyl-tRNA--protein transferase 1	NM_001256207.1
A3G0665	1,085.17	579.27	RPL23	60S ribosomal protein L23	NM_200732.1
B19G0947	1,062.19	60.44	SPACA4	Sperm acrosome membrane-associated protein 4	
B23G0970	1,025.61	451.12	PFN1	Profilin-1	NM_001305588.1
A23G0887	1,024.34	406.54	PFN1	Profilin-1	NM_001305588.1
B19G0915	1,022.57	405.56	RPL15	60S ribosomal protein L15	NM_001003447.1
A14G0117	1,001.55	1,092.34	SPARC	SPARC	NM_001001942.1
A18G0470	995.08	486.51	RPL4	60S ribosomal protein L4	NM_213107.1
A7G1156	994.05	745.84	FRIH	Ferritin, heavy subunit	XM_017356903.3
B25G0001	947.04	619.22	RPL27A	60S ribosomal protein L27a	
B6G0871	938.93	517.15	RPS26	40S ribosomal protein S26	NM_200742.1
B14G0108	912.06	754.35	SPARC	SPARC	XM_068213159.1
A21G0142	892.55	295.23	RPL35	60S ribosomal protein L35	NM_173233.2
B19G0952	885.58	349.59	RPS18	40S ribosomal protein S18	
B19G0477	885.34	523.96	RPL11	60S ribosomal protein L11	NM_001002139.1
A11G0166	882.75	615.10	K1C1	Keratin, type I cytoskeletal 50 kDa	NM_001001819.2
B21G0603	878.60	398.98	RPL17	60S ribosomal protein L17	NM_001319650.1
A1G0680	873.22	230.10	RPL24	60S ribosomal protein L24	NM_173235.3
A14G0776	864.73	172.57	TYBA	Thymosin beta-a	
A16G0848	857.90	383.48	RPL18	60S ribosomal protein L18	NM_001003432.2
A23G0592	856.15	399.63	RPS12	40S ribosomal protein S12	NM_001282177.1
A3G0500	854.74	900.82	ACTBB	Actin, cytoplasmic 2	NM_181601.5
A19G0019	851.45	976.90	NDK	Nucleoside diphosphate kinase	NM_001362265.1
B21G0806	849.40	313.41	RPL35	60S ribosomal protein L35	NM_173233.2
B11G0166	829.33	183.42	RPS15	40S ribosomal protein S15	
B20G0815	821.37	312.20	FOS	Protein c-Fos	NM_205569.1
A16G0078	821.27	733.87	HMGN2	Non-histone chromosomal protein HMG-17	NM_001142543.2
A7G1154	816.67	316.90	RPLP2	60S acidic ribosomal protein P2	NM_001100436.2
A20G0068	804.47	25.46	GFUS	GDP-L-fucose synthase	NM_001008620.1
A16G0640	803.19	390.94	RPS27	40S ribosomal protein S27	NM_200765.1
B18G0843	802.01	786.18	NMRK2	Nicotinamide riboside kinase 2	NM_198877.1
B16G0689	801.41	675.80	RPS27	40S ribosomal protein S27	NM_200765.1
A5G0424	798.03	156.50	ANXA1	Annexin A1	
A4G0740	787.67	855.11	B2M	Beta-2-microglobulin	NM_001159768.1
A23G0286	782.67	292.22	NACA	Nascent polypeptide-associated complex subunit alpha	NM_173264.2
B3G0503	782.40	25,961.17	HBA	Hemoglobin subunit alpha	NM_131257.3
A16G0410	780.32	414.40	RPS9	40S ribosomal protein S9	NM_200852.3
B19G0838	777.68	135.14	FTT-2	14-3-3-like protein 2	NM_212757.2
B14G0232	767.89	770.44	SLC25A6	ATP translocase 3	NM_173247.1
A17G0554	765.08	24.36	G5714_016907	A0A7J6C4B8_9TELE Uncharacterized protein	
B23G0622	762.10	329.09	RPS12	40S ribosomal protein S12	NM_001282177.1
B16G0677	760.85	198.38	APOEB	Apolipoprotein Eb	NM_131098.2
B8G1028	755.59	24.07	LOC113106698	A0A6P6PUF1_CARAU C-C motif chemokine	
A13G0174	747.88	337.11	ADGRB3	Adhesion G protein-coupled receptor B3	NM_001017593.2
B16G0670	747.16	444.21	RPS19	40S ribosomal protein S19	NM_200750.1
A16G0628	730.51	268.36	H2DFI5	H2DFI5_CYPCA Apolipoprotein E	NM_131098.2
B11G0130	723.40	394.78	UBE2J2	Ubiquitin-conjugating enzyme E2 J2	
A9G0046	717.56	957.44	TYB11	Thymosin beta-11	NM_001130697.1
B2G0052	715.25	648.85	EEF2	Elongation factor 2	NM_200458.2
A19G0435	705.67	78.37	FABP3	Fatty acid-binding protein, heart	NM_152961.3
A21G0333	696.86	370.64	RPL17	60S ribosomal protein L17	NM_001319650.1

A14G0648	682.15	794.16	CD74	H-2 class II histocompatibility antigen gamma chain	NM_131590.2
A4G1012	678.12	277.73	RPL18A	60S ribosomal protein L18a	NM_201060.1
A16G0622	674.04	386.64	RPS19	40S ribosomal protein S19	NM_200750.1
B19G0813	671.28	76.68	S100A13	Protein S100-A13	NM_001080161.2
A17G0424	669.48	295.70	G5714_017041	A0A7J6C5D6_9TELE Uncharacterized protein	
A16G0732	659.76	453.40	COX6B1	Cytochrome c oxidase subunit 6B1	NM_200506.3
A14G0233	658.60	474.77	SLC25A6	ATP translocase 3	NM_173247.1
B17G0340	644.55	4.83	G5714_016907	A0A7J6C4B8_9TELE Uncharacterized protein	
B14G0128	642.44	355.74	EEF1G	Elongation factor 1-gamma	NM_173263.1
A8G0805	636.46	348.22	PPIA	Peptidyl-prolyl cis-trans isomerase	NM_001328424.1
A6G0274	632.15	242.21	EEF1B	Elongation factor 1-beta	NM_199949.2
A24G0293	630.56	161.86	RPL22L1	60S ribosomal protein L22-like 1	NM_001045335.2
A8G0927	629.05	807.56	YBX1	Y-box-binding protein 1	NM_131620.1
B12G0809	623.75	199.98	COL1A1	Collagen alpha-1(I) chain	XM_005155976.5
B6G0288	613.78	145.38	EEF1B	Elongation factor 1-beta	NM_199949.2
B13G0264	604.27	72.80	MRPS26	28S ribosomal protein S26, mitochondrial	
B24G0445	602.10	262.63	RPL21	60S ribosomal protein L21	NM_001002155.1
A6G0592	595.70	426.70	SERBP1	Plasminogen activator inhibitor 1 RNA-binding protein	XM_009302506.3
A4G0545	595.68	273.63	RPS16	40S ribosomal protein S16	NM_001076732.2
A19G0157	579.85	62.61	S100A14	Protein S100-A14	
B7G0205	577.43	282.07	CSNK1G1	Casein kinase I isoform gamma-1	NM_001008635.1
A3G0850	576.48	269.02	RPS15A	40S ribosomal protein S15a	NM_212762.1
A11G0179	564.97	166.44			
A22G0084	564.90	502.35	RPL36	60S ribosomal protein L36	
A19G0519	554.17	195.73	RPL14	60S ribosomal protein L14	NM_001002866.2
B14G0182	543.69	310.07	CFL2	Cofilin-2	NM_213641.2
B5G1085	537.04	132.26	ANXA1	Annexin A1	
A22G0265	534.89	71.11	KRT17	Keratin, type I cytoskeletal 17	NM_001002383.1
B4G0538	522.53	380.47	RPS16	40S ribosomal protein S16	NM_001076732.2
A6G0027	519.79	174.91	ATF4	Cyclic AMP-dependent transcription factor ATF-4	
B16G0713	518.98	43.91	S100A16	Protein S100-A16	
B3G0008	517.85	37.11	EMP2	Epithelial membrane protein 2	
A19G0388	516.90	615.51	COL1A2	Collagen alpha-2(I) chain	XM_068215040.1
B3G0502	511.95	12,889.11	HBB	Hemoglobin subunit beta	NM_131020.3
A5G0688	505.41	133.30	TAGLN	Transgelin	NM_201576.1
B10G0459	504.37	304.66	HSPA8	Heat shock cognate 71 kDa protein	NM_001110403.1
B4G0336	503.76	496.62	B2M	Beta-2-microglobulin	NM_001159768.1
B21G0285	501.18	448.87	CD74	HLA class II histocompatibility antigen gamma chain	NM_131372.2
A21G0676	500.41	591.48	CD74	H-2 class II histocompatibility antigen gamma chain	NM_131372.2
A8G0864	497.21	1,404.66	B2M	Beta-2-microglobulin	
B2G1023	496.05	78.56	PTMAB	Prothymosin alpha-B	NM_001077304.1
B25G0863	494.09	241.56	RSL24D1	Probable ribosome biogenesis protein RLP24	NM_212993.1
B14G0729	487.42	5.60	SPINK1	Serine protease inhibitor Kazal-type 1	
B21G0823	487.14	287.96	RPL28	60S ribosomal protein L28	NM_201061.2
A19G0396	477.51	167.90	SEM1	26S proteasome complex subunit SEM1	NM_199593.3
B23G0656	477.40	0.00	CSL1	L-rhamnose-binding lectin CSL1	

B11G0271	469.30	80.63	PTP4A1	K7DYB3_DANRE Protein tyrosine phosphatase 4A1	NM_131245.2
A20G0089	464.18	233.67	HSP90AB1	Heat shock protein HSP 90-beta	NM_131310.3
A14G0660	462.94	234.97	RAN	GTP-binding nuclear protein Ran	NM_131309.3
A14G0187	457.30	557.00	CFL2	Cofilin-2	NM_213641.2
A5G0329	455.25	198.48	RPL36A	60S ribosomal protein L36a	NM_001202510.1
A8G0745	452.55	1,348.44	H2-AA	H-2 class II histocompatibility antigen, A-B alpha chain	NM_131695.1
A25G0516	452.01	81.16	ANXA2	Annexin A2	NM_181761.2
B7G0099	450.92	174.16	MLRN	Myosin regulatory light chain 2, smooth muscle minor isoform	NM_214699.2
B6G0443	450.85	106.51	GLUL	Glutamine synthetase	NM_182866.3
B15G0889	449.51	84.46	LOC113114638	A0A6P6QUZ1_CARAU Uncharacterized protein	
A16G0650	446.69	31.45	G5714_016148	A0A7J6C9K5_9TELE Uncharacterized protein	
B3G0793	444.82	587.75	MUCM	Ig mu chain C region membrane-bound form	
B25G0903	444.01	275.73	RPS17	40S ribosomal protein S17	NM_001013455.1
B1G0152	443.60	521.12	ACTBA	Actin, cytoplasmic 1	NM_131031.2
A6G0531	439.98	132.75	PTMAA	Prothymosin alpha-A	
B1G0421	439.51	46.98	FABP2	Fatty acid-binding protein, intestinal	NM_131431.1
A19G0126	432.56	75.16	FTT-2	14-3-3-like protein 2	NM_212757.2
B5G0750	432.53	108.50	CXCL13	C-X-C motif chemokine 13	
A1G0833	424.65	370.71	ACTBA	Actin, cytoplasmic 1	NM_131031.2
A24G0292	423.68	646.24	EIF5A	Eukaryotic translation initiation factor 5A-1	NM_213185.1
B11G0485	421.68	187.38	EIF4G3	Eukaryotic translation initiation factor 4 gamma 3	NM_001351705.1
A9G0883	420.77	63.76	G5714_002636	A0A7J6D7B7_9TELE Uncharacterized protein	
B7G0979	420.34	499.18	FRIH	Ferritin, heavy subunit	NM_131585.1
B5G0676	420.13	75.72	AQP3	Aquaporin-3	NM_213468.1
B18G0176	419.89	77.84	TRY2	Trypsin	
A23G0337	417.11	186.14	RPL10	60S ribosomal protein L10	NM_200027.1
B5G0857	416.40	290.13	BTF3	Transcription factor BTF3	NM_001431121.1
B19G0102	416.03	2,365.61	HA1F	Class I histocompatibility antigen, F10 alpha chain	
A14G0136	415.39	247.21	EEF1G	Elongation factor 1-gamma	NM_173263.1
A20G0146	407.21	252.64	FOS	Protein c-Fos	NM_205569.1
B22G1074	401.41	101.10	PFN2	Profilin-2	NM_201044.1
B6G0968	392.20	15.51	PKP1	Plakophilin-1	XM_001338263.7
B1G1167	388.71	74.20	KRT14	Keratin, type I cytoskeletal 14	
B3G0965	385.05	264.20	RPL3	60S ribosomal protein L3	NM_001001590.1
A2G0256	382.76	133.89	PTMAB	Prothymosin alpha-B	NM_001098730.1
B8G0150	376.40	548.76	B2M	Beta-2-microglobulin	
A25G0815	373.13	239.60	RPS17	40S ribosomal protein S17	NM_001013455.1
A19G0794	371.21	59.66	K1C1	Keratin, type I cytoskeletal 50 kDa	NM_001017824.2
A9G0300	368.82	27.82	LADD	Ladderlectin	XM_003199234.5
B3G0504	364.12	13,305.08	HBB	Hemoglobin subunit beta	NM_131020.3
B25G0320	353.93	139.92	ANXA2	Annexin A2	NM_181761.2
B16G0089	353.69	304.13	HMGN2	Non-histone chromosomal protein HMG-17	NM_001142543.2
B22G0564	353.63	47.76	KRT17	Keratin, type I cytoskeletal 17	NM_001002383.1
A9G0074	347.65	54.19	DAPL1-B	Death-associated protein-like 1-B	
B21G0875	346.12	119.75	GNB2L1	Guanine nucleotide-binding protein subunit beta-2-like 1	NM_131444.1

A15G0215	344.38	44.53	G5714_015330	A0A7J6CCI7_9TELE Uncharacterized protein	
B6G0024	343.64	182.69	ATF4	Cyclic AMP-dependent transcription factor ATF-4	
B19G0555	338.70	350.27	COL1A2	Collagen alpha-2(I) chain	NM_182968.2
A19G0717	337.75	31.07	S100A11	Protein S100-A11	
B17G0668	335.16	142.17	CRIP1	Cysteine-rich protein 1	NM_001166582.2
A5G0544	324.09	335.77	ATP5MG	ATP synthase subunit g, mitochondrial	NM_199757.1
A18G0597	321.99	116.33	COTL1	Coactosin-like protein	
B19G0220	319.93	11.45	S100A13	Protein S100-A13	
A16G0030	315.98	217.17	PABPC1A	Polyadenylate-binding protein 1A	XM_005169771.5
B6G0590	314.01	118.46	SERBP1	Plasminogen activator inhibitor 1 RNA-binding protein	XM_009302506.3
B3G0919	313.04	18.33	ACTPC	DELTA-actitoxin-Afr1a	NM_001293671.1
B16G0697	312.44	310.98	S100A1	Protein S100-A1	NM_213003.1
B9G0524	312.40	204.40	ITM2B	Integral membrane protein 2B	NM_212976.1
A2G0073	308.78	40.04	DSP	Desmoplakin	NM_001423756.1
B6G0527	306.18	143.54	PTMAA	Prothymosin alpha-A	
B25G0490	305.88	44.33			
A7G0601	304.95	436.37	HCEA	High choriolytic enzyme 1	NM_001077779.2
A3G0657	304.25	143.60	IER2	Immediate early response gene 2 protein	NM_001142583.3
B15G0890	303.16	15.20	LOC113114640	A0A6P6QUZ6_CARAU splicing factor U2af large subunit B-like	
B3G0489	301.82	183.25	EIF3G	Eukaryotic translation initiation factor 3 subunit G	NM_200999.1
A15G0654	301.55	141.04	ZFP36L1	mRNA decay activator protein ZFP36L1	NM_001089404.2
B24G0211	297.92	212.69	CEBPD	enhancer-binding protein delta	NM_131887.1
B20G0817	297.21	123.71	JDP2	Jun dimerization protein 2	XM_005160857.4
A21G0587	294.87	265.41	COX8B	Cytochrome c oxidase subunit 8B, mitochondrial	
A3G0156	293.34	174.50	JUNB	Transcription factor JunB	NM_212750.1
A3G1066	290.79	119.91	DDX5	Probable ATP-dependent RNA helicase DDX5	NM_212612.2
B25G0381	288.82	143.11	COX5A	Cytochrome c oxidase subunit 5A, mitochondrial	NM_001024403.2
A8G0496	288.23	256.44	GSTM3	Glutathione S-transferase Mu 3	NM_212676.1
A10G0194	284.46	184.40	COX5B	Cytochrome c oxidase subunit 5B, mitochondrial	NM_001002163.1
A1G0334	284.28	108.28	ATP5MK	ATP synthase membrane subunit K, mitochondrial	
A22G0562	283.32	66.62	CAPN2	Calpain-2 catalytic subunit	NM_001018227.1
A4G0652	283.16	93.08	LDHBA	L-lactate dehydrogenase B-A chain	NM_131247.1
A1G0784	280.78	81.40	TBA	Tubulin alpha chain	NM_200691.1
A5G0637	277.97	172.90	BTF3	Transcription factor BTF3	NM_001076558.2
A11G0330	276.86	49.35	MAF1	Repressor of RNA polymerase III transcription MAF1 homolog	NM_131245.2
B1G0633	276.68	90.22	TXN	Thioredoxin	NM_200023.1
B6G0136	274.39	311.36	TBA	Tubulin alpha chain	NM_200185.1
B17G0197	273.10	27.53	LGALS3	Galectin-3	NM_214693.3
B19G0546	272.43	122.38	SEM1	26S proteasome complex subunit SEM1	NM_199593.3
A11G0020	270.88	145.75	ASPSCR1	Tether containing UBX domain for GLUT4	NM_001243178.1
A13G0069	270.72	123.77	FBLN7	Fibulin-7	
B14G0434	268.00	484.24	LECG	Galactose-specific lectin nattectin	

A3G0170	267.74	155.95	SUMO2	Small ubiquitin-related modifier 2	NM_001003422.2
A20G0672	267.69	34.37	DSG2	Desmoglein-2	NM_001256637.1
A18G0215	267.45	168.06	COX41	Cytochrome c oxidase subunit 4 isoform 1, mitochondrial	
B20G0274	266.88	31.15	DSC1	Desmocollin-1	NM_001287083.1
B23G0945	262.92	201.93	HMGB2	High mobility group protein B2	NM_001004674.1
B13G0213	262.52	306.19	TTL	Tubulin--tyrosine ligase	NM_200867.1
B24G0070	261.83	373.08	IGLC3	Ig lambda-3 chain C region	XM_068217596.1
B1G0513	260.57	32.95	ITGA6	Integrin alpha-6	NM_001013448.1
B11G0125	260.08	44.64	MAPK14B	Mitogen-activated protein kinase 14B	NM_213470.1
A3G0754	259.15	11.30	ACTPB	DELTA-actitoxin-Afr1c	NM_001386552.1
B21G0862	258.52	114.75	NSA2	Ribosome biogenesis protein NSA2 homolog	NM_199568.2
A22G0050	258.13	210.96	BTG2	Protein BTG2	NM_130922.2
B19G0106	258.02	339.49	HA1B	BOLA class I histocompatibility antigen, alpha chain BL3-7	NM_131705.1
B13G0259	256.17	309.36	BBOF1	Basal body-orientation factor 1	
B1G0597	255.80	142.77	HMGB1	High mobility group protein B1	NM_001037424.2
A1G0304	254.88	115.69	MDH1	Malate dehydrogenase, cytoplasmic	NM_001309523.2
B12G0396	252.24	87.49	CAPG	Macrophage-capping protein	NM_001001594.2
A14G0668	251.12	154.13	EGR1	Early growth response protein 1	NM_131248.1
A25G0591	250.69	72.58	PHLDA2	Pleckstrin homology-like domain family A member 2	NM_001020596.2
B14G0130	249.23	118.35	AHNAK	Neuroblast differentiation-associated protein AHNAK	XM_005173182.5
B13G0467	249.17	27.28	TIAL1	Nucleolysin TIAR	XM_692149.10
B13G0886	245.56	116.32	PTK7	Inactive tyrosine-protein kinase 7	NM_200548.1
A2G0420	244.36	56.70	PTGS2	H synthase 2	NM_153657.1
A6G0201	244.24	559.99	ITM2B	Integral membrane protein 2B	NM_199646.1
A6G0127	243.78	168.05	TBA	Tubulin alpha chain	NM_001326595.1
B3G1160	242.64	153.93	DDX5	Probable ATP-dependent RNA helicase DDX5	NM_001309525.1
B1G0199	240.81	146.00	EIF3F	Eukaryotic translation initiation factor 3 subunit F	NM_001200009.1
B22G0639	240.43	80.66	FAM32AL	Protein FAM32A-like	NM_001002203.1
B11G0541	240.24	302.13	STX6	Syntaxin-6	NM_001007281.2
A25G0457	237.85	127.33	COX5A	Cytochrome c oxidase subunit 5A, mitochondrial	NM_001024403.2
B21G0635	237.34	100.29	ARPC3-A	3 complex subunit 3-A	NM_001002114.1
A10G0832	236.72	253.30	HNRNPDL	Heterogeneous nuclear ribonucleoprotein D-like	NR_137312.1
B25G0268	236.54	48.95	PKP3	Plakophilin-3	NM_001045280.1
B9G0784	236.39	216.22	BZW1B	eIF5-mimic protein 2-B	NM_213092.1
B19G0319	235.20	44.96	G5714_018815	A0A7J6C008_9TELE Uncharacterized protein	XM_005159376.3
B24G0330	234.64	144.55	EIF5A	Eukaryotic translation initiation factor 5A-1	NM_213185.1
B23G0562	234.27	132.37	MYL6B	Myosin light chain 6B	XM_021469755.2
A11G0264	234.06	51.61	STIMATE	Store-operated calcium entry regulator STIMATE	NM_213523.2
B6G0910	233.86	90.76	CX6C1	Cytochrome c oxidase subunit 6C-1	
A16G0539	233.49	10.09			NM_001080576.1
B10G0731	232.39	235.81	UBC	Polyubiquitin-C	NM_001077804.2
B19G0703	231.12	47.97	AGR2	Anterior gradient protein 2 homolog	NM_001012481.2
B7G0733	230.18	74.48	SERF2	Small EDRK-rich factor 2	

A19G0241	230.15	173.62	EIF3I	Eukaryotic translation initiation factor 3 subunit I	NM_212990.1
B15G0281	230.05	367.07	ZFP36	mRNA decay activator protein ZFP36	NM_001089404.2
B6G0709	230.01	53.03	TOMM6	Mitochondrial import receptor subunit TOM6 homolog	NM_001303056.1
A12G0377	228.80	175.72	CALMA	Calmodulin-alpha (Fragment)	NM_214736.1
B23G0249	227.68	149.69	KRT8	Keratin, type II cytoskeletal 8	NM_200080.2
B3G0877	224.12	162.76	FRIM	Ferritin, middle subunit	NM_001109854.1
A16G0076	223.56	28.45	SERPINB1	Leukocyte elastase inhibitor	XM_002665065.7
B13G0742	223.25	294.36	ZAX	Homeobox protein zampogna	NM_199996.2
A9G0306	223.12	13.20	LECG	Galactose-specific lectin nattectin	
A10G0655	221.97	15.91	PPP1R3CB	Protein phosphatase 1 regulatory subunit 3C-B	
A11G0259	221.77	34.67	QRICH1	Transcriptional regulator QRICH1	NM_131177.1
B18G0376	221.23	164.09	CAPRIN1	Caprin-1	XM_068214751.1
A16G0524	219.72	169.40	SEC61B	Protein transport protein Sec61 subunit beta	NM_001002527.3
A7G0257	219.51	113.79	RPS17	40S ribosomal protein S17	NM_200845.1
A16G0176	218.17	280.19	GAPDH-2	Glyceraldehyde-3-phosphate dehydrogenase 2	NM_213094.2
B14G0668	217.44	252.20	RAN	GTP-binding nuclear protein Ran	NM_131309.3
B11G0172	215.43	35.33	MCC	Colorectal mutant cancer protein	NM_131177.1
A12G0793	214.78	878.68	COL1A1	Collagen alpha-1(I) chain	XM_005155976.5
B18G0082	214.51	140.24	EIF3M	Eukaryotic translation initiation factor 3 subunit M	NM_001024735.1
B4G0416	213.49	107.58	DCN	Decorin	NM_131697.1
B5G0805	213.21	123.04	TAGLN2	Transgelin-2	NM_201576.1
B3G0891	212.89	181.57	FRIM	Ferritin, middle subunit	NM_001109854.1
A24G0107	211.51	566.22	IGK	Immunoglobulin kappa light chain	XM_068217596.1
B8G0335	211.13	156.29	COX6A	Cytochrome c oxidase subunit 6A, mitochondrial	
B5G0826	211.07	83.89	CAPNS1	Calpain small subunit 1	NM_001017899.2
A5G0228	210.52	96.89	A0A1V0D965	A0A1V0D965_CYPCA XBP1s	NM_131874.1
A22G0398	209.55	163.26	CIRBP-B	Cold-inducible RNA-binding protein B	
A10G0129	209.13	181.83	PPIA	Peptidyl-prolyl cis-trans isomerase	NM_212758.2
B1G0810	208.96	118.44	LOC107675992	A0A671KMW6_9TELE C-X-C motif chemokine 11-like	
B7G1130	207.85	57.81	RNF182	E3 ubiquitin-protein ligase RNF182	NM_001386727.1
A16G0645	207.30	280.73	S10I	Ictacalcin	
A25G0115	206.88	100.48	PPIB	Peptidyl-prolyl cis-trans isomerase B	NM_213019.1
A11G0540	205.81	88.99	LOC113110910	A0A6P6QBP1_CARAU uncharacterized protein LOC113110910	NM_001351705.1
B14G0570	204.19	174.11	TUBB4B	Tubulin beta-4B chain	NM_198809.2
A9G0738	203.90	308.40	BZW1A	eIF5-mimic protein 2-A	XM_005167390.5
A9G0760	203.06	25.13	DBI	Acyl-CoA-binding protein	NM_199608.2
A10G0658	203.06	149.89	CCL19	C-C motif chemokine 19	NM_001302701.1
A6G0955	201.89	7.98	PKP1	Plakophilin-1	XM_001338263.7
B20G0878	201.13	203.35	HSP90AB1	Heat shock protein HSP 90-beta	NM_131310.3
A8G0443	201.11	122.21	PPDPFA	Pancreatic progenitor cell differentiation and proliferation factor A	NM_200079.1

B16G0043	200.98	146.02	PABPC1A	Polyadenylate-binding protein 1A	XM_005169771.5
B10G0456	200.68	153.55	H2A	Histone H2A	NM_001005967.1
B14G0540	200.59	20.08	STING1	Stimulator of interferon genes protein	XM_005157121.5
B12G0370	200.30	140.34	CALM1	Calmodulin-1	NM_214736.1
B11G0629	199.24	194.31	LOC107751248	A0A673FSA5_9TELE Uncharacterized LOC107751248	XM_005155917.2
B19G0466	198.27	134.97	UQCRB	Cytochrome b-c1 complex subunit 7	
B9G0056	197.17	25.83	LOC107566575	A0A672NL81_SINGR Cell surface glycoprotein 1-like	XM_021479062.2
A14G0141	197.13	70.72	TSC22D3	TSC22 domain family protein 3	NM_001348480.1
A10G0154	197.02	331.14	UBC	Polyubiquitin-C	NM_001077804.2
A23G0532	195.69	120.95	MYL6	Myosin light polypeptide 6	NM_213638.1
B8G1027	194.95	57.37	LOC113106698	A0A6P6PUF1_CARAU C-C motif chemokine	
A21G0071	194.87	119.84	GNB2L1	Guanine nucleotide-binding protein subunit beta-2-like 1	NM_131444.1
B6G0935	194.74	140.05	RBM5	RNA-binding protein 5	NM_001100138.3
B8G0135	193.46	93.36	FKBP1A	Peptidyl-prolyl cis-trans isomerase FKBP1A	NM_199945.2
A8G0190	192.92	68.01	PIM1	threonine-protein kinase pim-1	NM_001077391.1
A13G0178	192.80	229.47	LOC113071541	A0A6P6MVR8_CARAU interferon alpha-inducible protein 27-like protein 2A isoform X1	NM_199996.2
B4G0415	190.98	54.92	LUM	Lumican	NM_001002059.1
B25G0238	190.08	99.12	PHLDA2	Pleckstrin homology-like domain family A member 2	NM_001020596.2
A20G0475	190.02	142.49	CHAC1	Glutathione-specific gamma-glutamylcyclotransferase 1	NM_001110126.2
A24G0453	189.96	146.06	SEC61G	Protein transport protein Sec61 subunit gamma	NM_001002499.3
A7G0688	189.10	5.76	ITLN	Intelectin	NM_001076581.1
B21G0401	188.86	82.96	BANF1	Barrier-to-autointegration factor	NM_205562.1
A25G0066	187.80	82.88	TMEM258	Transmembrane protein 258	
B3G0884	186.72	21.76	FRIM	Ferritin, middle subunit	NM_001109854.1
B2G1167	185.75	79.38	PFN2	Profilin-2	XM_005171152.3
A6G0221	185.19	131.60	ATP5MC3	ATP synthase F(0) complex subunit C3, mitochondrial	XM_005165783.5
B4G0014	184.71	154.00	OST4	Dolichyl-diphosphooligosaccharide--protein glycosyltransferase subunit 4	
B8G0465	184.53	92.02	TBAT	Tubulin alpha chain, testis-specific	NM_212772.1
A1G0539	184.33	105.00	HMGB2	High mobility group protein B2	NM_001037424.2
A10G0131	184.12	129.99	PPIA	Peptidyl-prolyl cis-trans isomerase	NM_212758.2
B21G0089	183.60	16.84	DKC1	ACA ribonucleoprotein complex subunit DKC1	
A20G0886	183.05	119.67	AMD1	A0A7N6A9C3_ANATE Adenosylmethionine decarboxylase 1	NM_001161601.1
B10G0037	182.56	103.18	REEP5	Receptor expression-enhancing protein 5	NM_200058.1
B20G0711	182.49	21.90	FABP7	Fatty acid-binding protein, brain	NM_214807.1
A21G0734	182.01	170.74	COX7B	Cytochrome c oxidase subunit 7B, mitochondrial	NM_001130602.1

B3G1658	181.30	11.24	LOC113100217	A0A6P6PGU6_CARAU uncharacterized protein LOC113100217	
B9G0333	180.97	112.32	ATP5PO	ATP synthase subunit O, mitochondrial	NM_001003843.1
B22G0721	180.35	228.14	CIRBP-B	Cold-inducible RNA-binding protein B	
B2G0060	178.83	106.01	GPX4	Phospholipid hydroperoxide glutathione peroxidase	NR_144467.1
A21G0080	178.75	62.95	NSA2	Ribosome biogenesis protein NSA2 homolog	NM_199568.2
A8G0077	178.74	83.75	CTP	Dynein light chain 1, cytoplasmic	NM_213024.2
B22G0029	178.68	74.98	SRSF3	arginine-rich splicing factor 3	
B3G0938	177.90	167.18	CORO1C	Coronin-1C	NM_201114.1
B3G0969	177.38	81.30	RAC2	Ras-related C3 botulinum toxin substrate 2	NM_001002061.1
A8G0044	177.18	18.97	LOC107568102	A0A672MHF1_SINGR C-C motif chemokine 28-like	
B6G0233	175.97	240.37	ATP5MC3	ATP synthase F(0) complex subunit C3, mitochondrial	XM_005165783.5
A13G0123	174.76	207.08	ADTRP	Androgen-dependent TFPI-regulating protein	NM_131883.2
A20G0671	173.42	16.24	DSC3	Desmocollin-3	NM_001287083.1
B9G0150	173.08	75.13	ARPC2-A	3 complex subunit 2-A	NM_212699.1
A25G0167	172.87	2.86	MUC2	Mucin-2 (Fragments)	XM_021470904.1
A17G0517	171.54	5.47	MAL	Myelin and lymphocyte protein	
B21G0711	170.76	56.67	NOP56	Nucleolar protein 56	NM_201217.2
A6G0658	170.67	112.01	KRT18	Keratin, type I cytoskeletal 18	NM_200568.1
B9G0003	170.18	107.48	ACTR3	Actin-related protein 3	NM_001003944.1
A3G0773	170.16	22.79	CLDN4	Claudin-4	NM_131768.2
A7G0435	170.07	36.73	HYPK	Huntingtin-interacting protein K	NM_001127470.1
B16G0701	169.45	28.54	LOC113116478	A0A6P6R3X3_CARAU uncharacterized protein LOC113116478	
A8G0892	167.91	77.28	FKBP1A	Peptidyl-prolyl cis-trans isomerase FKBP1A	NM_199945.2
A18G0555	167.59	17.67	ENDOUC	Uridylate-specific endoribonuclease C	NM_001044974.1
B17G0607	166.89	80.09	YWHAZ	delta	NM_201484.1
A4G1036	166.84	58.71	OST4	Dolichyl-diphosphooligosaccharide--protein glycosyltransferase subunit 4	
B24G0569	166.74	85.70	PSME1	Proteasome activator complex subunit 1	NM_131375.2
A14G0375	165.94	181.62	TOMM5	Mitochondrial import receptor subunit TOM5 homolog	NM_001166115.2
A6G0899	164.65	84.59	CX6C1	Cytochrome c oxidase subunit 6C-1	
B2G0890	163.95	10.68	PRG4	Proteoglycan 4	NM_212753.2
A18G0505	163.53	666.72	MT2	Metallothionein-2	NM_131075.1
A3G1011	162.54	75.79	ATP5MC2	ATP synthase F(0) complex subunit C2, mitochondrial	NM_213028.1
B8G0824	162.44	33.25	ITGB4	Integrin beta-4	NM_001024386.1
B3G0255	162.26	69.37	SUMO2	Small ubiquitin-related modifier 2	NM_001003422.2
A10G0595	162.14	37.04	NPM1	Nucleophosmin	NM_199428.2
B5G0953	161.12	99.61	ATP5MG	ATP synthase subunit g, mitochondrial	NM_199757.1
B2G0148	161.08	90.99	EEF1D	Elongation factor 1-delta	XM_017358747.3
A5G1049	160.88	0.70	UPK2	Uroplakin-2	

A1G0277	160.65	81.00	JUNB	Transcription factor JunB	NM_213556.3
A4G0666	160.14	94.25	BTG1	Protein BTG1	NM_200020.1
B20G0647	160.00	20.64	ABRACL	Costars family protein ABRACL	
A21G0220	159.48	29.71	CAST	Calpastatin	XM_009295209.4
B11G0240	159.43	68.14	CAMK4	calmodulin-dependent protein kinase type IV	NM_001003762.1
A6G0507	159.07	13.32	TP63	Tumor protein 63	XM_068221639.1
A3G1029	158.91	92.23	ATF4	Cyclic AMP-dependent transcription factor ATF-4	NM_001103192.2
A23G0591	158.08	3.53	SH3BGRL3	SH3 domain-binding glutamic acid-rich-like protein 3	NM_001045409.2
B25G0812	157.46	137.55	SLC25A3	Phosphate carrier protein, mitochondrial	NM_213722.1
A22G0327	156.99	100.59	KLF2	Krueppel-like factor 2	NM_131856.3
A3G0007	156.68	14.12	EMP2	Epithelial membrane protein 2	
B14G0521	156.31	112.33	CLK4	Dual specificity protein kinase CLK4	XM_003199840.5
A19G0703	156.14	40.55	LOC107599498	A0A672P2Q6_SINGR Uncharacterized LOC107599497	XM_009294050.4
B22G1056	156.05	94.70	NCL	Nucleolin	XM_005170547.5
A23G0219	155.64	271.24	ION3	Intermediate filament protein ON3	NM_200080.2
A6G0717	155.60	176.54	H1-10	Histone H1.10	NM_199276.1
B4G0571	154.93	103.76	ATP5F1C	ATP synthase subunit gamma, mitochondrial	NM_200041.2
B5G1111	154.69	19.43			
A6G0897	154.08	90.52	YWHABA	alpha-A	NM_001082798.1
B24G0154	152.72	101.79	HIS3.3A	Histone H3.3A	NM_201101.1
A3G0727	152.71	98.93	FRIM	Ferritin, middle subunit	NM_001113659.1
A13G0037	152.51	109.21	DNAJC12	DnaJ homolog subfamily C member 12	XM_068212834.1
B21G0336	151.21	30.26	NHP2	ACA ribonucleoprotein complex subunit 2-like protein	
A14G0755	151.08	54.03	PLS3	Plastin-3	NM_001002326.1
A15G0358	150.72	78.72	CAPNS1	Calpain small subunit 1	NM_001109706.1
A10G0452	150.63	163.32	HSPA8	Heat shock cognate 71 kDa protein	NM_001110403.1
B15G0368	150.37	24.75	POU2AF3	POU Class 2 homeobox-associating factor 3	NM_001386216.1
B23G0618	149.98	94.44	HMGN3	High mobility group nucleosome-binding domain-containing protein 3	NM_001243173.1
B1G0967	148.97	29.73	EXOSC1	Exosome complex component CSL4	NM_001145567.1
B6G0662	148.49	136.96	ION3	Intermediate filament protein ON3	NM_131509.2
A8G0568	148.29	44.70	TBAT	Tubulin alpha chain, testis-specific	NM_212772.1
A5G0827	147.46	78.89	CTSL	Procathepsin L	XM_005165268.3
A5G0675	147.42	2.34	NCCRP1	F-box only protein 50	NM_130921.2
A8G0572	147.32	129.11	CEBPB	enhancer-binding protein beta	NM_131884.2
A21G0305	147.15	89.68	ARPC3-B	3 complex subunit 3-B	NM_001002114.1
B24G0331	147.03	28.24	RPL22L1	60S ribosomal protein L22-like 1	NM_001045335.2
B14G0249	146.66	103.89	NDUFA1	NADH dehydrogenase [ubiquinone] 1 alpha subcomplex subunit 1	
B6G0237	146.41	65.82	CYC	Cytochrome c	NM_001002068.1
A21G0829	146.13	17.47	DKC1	ACA ribonucleoprotein complex subunit DKC1	
B11G0805	145.80	131.53			NM_001326405.1

B1G0043	145.66	65.72	ATP5PF	ATP synthase-coupling factor 6, mitochondrial	
A16G0729	144.84	44.16	TRPV6	Transient receptor potential cation channel subfamily V member 6	XM_005173468.3
B22G1098	144.77	314.60	LOC113039727	A0A6P6J6F5_CARAU uncharacterized protein LOC113039727	
A21G0521	144.56	32.20	CLDN4	Claudin-4	NM_131762.2
A21G0043	144.35	12.27	CGN	Cingulin	XM_021468944.2
A16G0184	143.95	4.62	UPK1A	Uroplakin-1a	NM_001040242.1
A14G0394	143.39	423.12			
B7G0985	143.29	31.53	POLR2L	DNA-directed RNA polymerases I, II, and III subunit RPABC5	NM_001159825.3
B8G0538	143.22	114.75	ATP5PB	ATP synthase F(0) complex subunit B1, mitochondrial	NM_001005960.1
A18G0346	142.93	48.16	CD82	CD82 antigen	NM_212663.1
B25G0751	142.87	104.78	PPIB	Peptidyl-prolyl cis-trans isomerase B	NM_213019.1
A12G0455	142.35	140.43	TEF	Thyrotroph embryonic factor	NM_001431066.1
A24G0177	141.79	92.42	CEBPD	enhancer-binding protein delta	NM_131887.1
A25G0639	141.78	183.27	IGLC7	Immunoglobulin lambda constant 7	XM_068217596.1
A3G0755	141.59	3.03	ACTP1	DELTA-sagatoxin-Srs1a	NM_001293671.1
B13G0432	141.53	45.20	SLC1A4	Neutral amino acid transporter A	NM_001017870.1
B20G0222	140.82	60.77	JUN	Transcription factor Jun	NM_199987.1
A3G0963	140.81	111.03	NDUFA4	Cytochrome c oxidase subunit NDUFA4	NM_213417.2
A14G0515	140.68	102.95	CLK4	Dual specificity protein kinase CLK4	XM_003199840.5
A6G0879	140.46	33.10	MYCA	Transcriptional regulator Myc-1	NM_001126109.1
B10G0381	140.12	80.95	SOD1	Superoxide dismutase [Cu-Zn]	NM_131294.1
A21G0246	140.12	109.19	SET	Protein SET	NM_201468.1
A20G0144	139.56	141.39	JDP2	Jun dimerization protein 2	XM_005160857.4
B23G0724	139.52	148.27	HNRNPA1	Heterogeneous nuclear ribonucleoprotein A1	NM_200104.2
A23G0276	139.45	158.57	PPDPFB	Pancreatic progenitor cell differentiation and proliferation factor B	NM_200008.1
B14G0375	139.39	96.03	ADHX	Alcohol dehydrogenase class-3	NM_131849.2
A5G0518	139.36	17.12	A0A673J3E8	A0A673J3E8_9TELE Thy-1 cell surface antigen	NM_001313770.1
B19G0504	138.84	48.41	FABP3	Fatty acid-binding protein, heart	NM_152961.3
A11G0035	138.00	121.15	PFKL	ATP-dependent 6-phosphofructokinase, liver type	NM_001326405.1
B2G0758	137.11	24.14	EIF5A	Eukaryotic translation initiation factor 5A-1	NM_213262.1
A4G0767	136.84	65.97	GDI2	Rab GDP dissociation inhibitor beta	NM_199655.1
B17G0339	136.77	2.61	G5714_016907	A0A7J6C4B8_9TELE Uncharacterized protein	
A24G0416	136.61	28.75	FAM162B	Protein FAM162B	NM_001089343.2
B18G0634	136.58	90.50	COTL1	Coactosin-like protein	
A15G0018	136.57	35.94	HIS3.3A	Histone H3.3A	NM_001366916.1
A19G0692	136.50	102.24	RPS27	40S ribosomal protein S27	NM_001159528.1
A7G1356	135.44	16.47	NOP10	ACA ribonucleoprotein complex subunit 3	
B21G0210	134.82	52.85	NOP16	Nucleolar protein 16	NM_001002534.1
A19G0612	134.78	147.73	TPM3	Tropomyosin alpha-3 chain	NM_201492.3
A3G1180	134.23	84.04	ARHGDIA	Rho GDP-dissociation inhibitor 1	NM_213461.1

B7G0410	133.88	35.70	HYPK	Huntingtin-interacting protein K	NM_001127470.1
A4G0964	133.42	109.28	NAP1L1	Nucleosome assembly protein 1-like 1	XM_021474937.2
A23G0338	133.04	48.89	FLNA	Filamin-A	XM_021473622.2
A22G0683	132.90	117.38	NCL	Nucleolin	
A12G0243	132.60	92.84	SRSF2	arginine-rich splicing factor 2	NM_213382.1
B20G0468	132.15	126.08	CHAC1	Glutathione-specific gamma-glutamylcyclotransferase 1	NM_001110126.2
B20G0223	132.01	84.22	PRDX6	Peroxiredoxin-6	NM_200805.1
A2G0503	131.68	48.50	TRNAU1APL	tRNA selenocysteine 1-associated protein 1-like	NM_001029966.2
A2G1158	131.65	83.98	JUND	Transcription factor JunD	NM_001128342.1
B2G0129	131.47	43.35	CD59	CD59 glycoprotein	
A3G0828	131.39	74.56	ARHGDIB	Rho GDP-dissociation inhibitor 2	NM_001423647.1
A20G0720	131.30	67.90	JUN	Transcription factor Jun	NM_199987.1
A7G0447	131.18	58.86	CD44	CD44 antigen	
B6G0504	131.15	8.05	TP63	Tumor protein 63	XM_068221639.1
A5G0962	131.13	117.97	EDF1	Endothelial differentiation-related factor 1 homolog	NM_200745.1
A9G0552	130.93	25.40	COX17	Cytochrome c oxidase copper chaperone	
A8G0744	130.93	597.81	HB24	H-2 class II histocompatibility antigen, E-Q beta chain	NM_001007167.1
A20G0285	130.78	37.92	CCL8	C-C motif chemokine 8	NM_001030173.1
A3G0343	130.28	104.59	ATP5PD	ATP synthase subunit d, mitochondrial	NM_200702.1
B3G0967	130.11	142.24	EIF3D	Eukaryotic translation initiation factor 3 subunit D	NM_200016.1
A9G0437	129.73	144.76	ITM2B	Integral membrane protein 2B	NM_212976.1
A11G0566	129.39	70.20	BICD2	Protein bicaudal D homolog 2	XM_068224238.1
B19G0733	129.29	109.44			
A9G0114	128.91	92.09	ARPC2-A	3 complex subunit 2-A	NM_212699.1
A1G0188	128.62	27.63	ZGC:136864	UPF0390 protein zgc136864	NM_001040044.2
B5G0636	128.52	103.48	COX7C	Cytochrome c oxidase subunit 7C, mitochondrial	NM_199995.1
B1G0816	128.45	111.99	ATP5MK	ATP synthase membrane subunit K, mitochondrial	
A9G0014	128.41	140.01	LCP1	Plastin-2	NM_131320.3
A20G0035	128.05	76.89	FKBP3	Peptidyl-prolyl cis-trans isomerase FKBP3	NM_001077770.1
B21G0719	128.03	64.59	GLRX	Glutaredoxin-1	
A19G0246	127.99	35.39	AGR2	Anterior gradient protein 2 homolog	NM_001012481.2
A8G0872	127.99	56.82	LOC113039697	A0A6P6J0E3_CARAU small serum protein 2-like isoform X2	
B5G0085	127.83	49.17	LIPO	Lipocalin	NM_001076726.2
B5G0818	127.63	71.33	NCCRP1	F-box only protein 50	NM_130921.2
A17G0289	127.53	180.29	CTSB	Cathepsin B	NM_213336.2
A18G0152	127.51	78.32	SNRPD2	Small nuclear ribonucleoprotein Sm D2	NM_001017582.1
B2G1210	127.26	140.01	SERBP1	Plasminogen activator inhibitor 1 RNA-binding protein	XM_001338162.9
B1G0849	127.20	85.41	MDH1	Malate dehydrogenase, cytoplasmic	NM_001309523.2
A18G0738	126.68	63.51	HAAF	amebocyte aggregation factor	
A3G0407	126.67	4,036.77	HBB	Hemoglobin subunit beta	NM_001013027.1
A6G0871	126.63	85.35	ATP5F1EP2	ATP synthase subunit epsilon-like protein, mitochondrial	
B7G1095	126.34	31.62	SNRPF	Small nuclear ribonucleoprotein F	NM_001003881.3
B6G0230	126.12	68.27	OLA1	Obg-like ATPase 1	XM_021476786.2

B17G0780	126.02	44.41	TRB	M1-specific T cell receptor beta chain	
B2G0009	125.40	153.36	ROHU_002699	A0A498NX11_LABRO Uncharacterized protein	
B16G0200	125.37	4.46	UPK1A	Uroplakin-1a	NM_001040242.1
B1G0993	125.36	39.19	GNG5	G(O) subunit gamma-5	NM_001200040.1
B16G0170	125.30	52.25	RBM8A	RNA-binding protein 8A	NM_001013345.1
B18G0146	125.25	30.78	SNRPD2	Small nuclear ribonucleoprotein Sm D2	NM_001017582.1
A10G0705	125.21	36.28	SUB1	Activated RNA polymerase II transcriptional coactivator p15	NM_001020540.3
A8G0384	125.15	72.30	MAGOH	Protein mago nashi homolog	NM_001017700.1
A3G0166	124.97	15.25	CYGB1	Cytoglobin-1	NM_152952.2
B21G0194	124.84	18.54	POF1B	Protein POF1B	XM_068216131.1
A16G0612	124.79	83.85	YWHABA	alpha-A	NM_199562.1
A13G0080	124.67	46.47	RTASE	Probable RNA-directed DNA polymerase from transposon BS	
A1G1059	124.20	60.91	ANXA5	Annexin A5	NM_181757.2
B21G0244	123.75	69.75	SERPINF1	Pigment epithelium-derived factor	NM_001004539.1
B5G0812	123.67	85.91			NM_200003.1
A7G0831	123.39	61.35	EIF4A1	Eukaryotic initiation factor 4A-I	NM_198366.2
B20G0326	122.83	50.83	ATP5MJ	ATP synthase subunit ATP5MJ, mitochondrial	NM_001135972.3
B16G0250	122.75	39.64	FBL	rRNA 2'-O-methyltransferase fibrillarin	NM_213002.1
B19G0482	122.69	63.44	CAP1	Adenylyl cyclase-associated protein 1	NM_199909.2
B3G0622	122.45	198.60	ALDOA	Fructose-bisphosphate aldolase A	XM_005164116.5
B10G0694	121.75	120.89	COX5B	Cytochrome c oxidase subunit 5B, mitochondrial	NM_001002163.1
B15G0915	121.69	44.36	EIF3K	Eukaryotic translation initiation factor 3 subunit K	NM_001017583.1
B14G0825	121.43	62.25	LOC113113531	A0A6P6QQC3_CARAU non-histone chromosomal protein HMG-14A-like	NM_001161336.1
B3G0591	121.25	52.76	ARPC1A	3 complex subunit 1A	NM_201343.2
B11G0015	120.86	59.90	NPFF	Pro-FMRFamide-related neuropeptide FF	NM_199576.1
B19G0609	120.57	52.71	ZNF706	Zinc finger protein 706	NM_001003731.2
B5G1317	120.55	49.91	H2AZ2	Histone H2A.V	
B21G0696	120.26	91.90	SET	Protein SET	NM_201468.1
A12G0323	119.96	33.00			NM_199644.2
B14G0338	119.61	70.81	UQCRQ	Cytochrome b-c1 complex subunit 8	NM_001002495.3
B24G0402	119.05	26.10	RRS1	Ribosome biogenesis regulatory protein homolog	NM_200062.1
B3G0590	118.78	105.25	ARPC1B	3 complex subunit 1B	NM_213156.1
B7G0564	118.55	79.09	MDK-A	Midkine-A	NM_131070.2
A2G1032	118.23	89.81	MBNL1	Muscleblind-like protein 1	NM_001100050.3
A11G0679	117.78	102.62	B3GALT6	Beta-1,3-galactosyltransferase 6	XM_005155917.2
B21G0834	117.67	97.34	ATP5F1A	ATP synthase subunit alpha, mitochondrial	NM_001077355.1
B22G0668	116.44	35.87	IER5	Immediate early response gene 5 protein	NM_001007197.1
B16G0802	116.42	31.98	G5714_016248	A0A7J6C9V0_9TELE Uncharacterized protein	
B22G0726	116.36	156.07	BSG	Basigin	NM_198142.2
B2G0087	115.87	22.64	CCL25	C-C motif chemokine 25	
B5G1276	115.18	30.46	RANBP1	Ran-specific GTPase-activating protein	NM_212766.2

B25G0402	114.76	84.28	PDIA3	Protein disulfide-isomerase A3	NM_001199737.1
B19G0927	114.74	313.04	HA1F	Class I histocompatibility antigen, F10 alpha chain	NM_001110118.1
B4G0133	114.72	36.51	CD9	CD9 antigen	XM_068219577.1
B23G0969	114.47	278.35	ENO3	Beta-enolase	NM_214723.2
B20G0593	114.43	38.62	IVNS1ABPA	Influenza virus NS1A-binding protein homolog A	NM_199279.2
A24G0503	114.38	61.57	PSME1	Proteasome activator complex subunit 1	NM_131375.2
A11G0637	114.15	43.20	KCNAB2	Voltage-gated potassium channel subunit beta-2	NM_201004.2
A19G0936	113.87	111.22	ATP5IF1A	ATPase inhibitor A, mitochondrial	NM_001089521.1
A20G0887	113.86	91.79	AMD1	S-adenosylmethionine decarboxylase proenzyme	NM_001161601.1
A17G0120	113.60	38.94	TRB	M1-specific T cell receptor beta chain	
A19G0820	113.10	75.56	PSMB7	Proteasome subunit beta type-7	NM_131677.2
B19G0926	112.74	503.40	H2-L	H-2 class I histocompatibility antigen, L-D alpha chain	NM_001083545.1
B15G0891	112.46	6.73	DPX16_3625	A0A3N0XR32_ANAGA Uncharacterized protein	
B9G0338	112.41	101.51	LECG	Galactose-specific lectin nattectin	
A8G0083	112.40	77.29	SET	Protein SET	NM_201475.1
A3G0493	112.37	105.70	ARPC1B	3 complex subunit 1B	NM_213156.1
B3G0798	112.27	102.55	IER2	Immediate early response gene 2 protein	
B3G0681	112.23	20.85	ARPC1A	3 complex subunit 1A	XM_005164011.5
B13G0720	111.78	26.48	LOC113068807	A0A6P6MLP7_CARAU interferon alpha-inducible protein 27-like protein 2A	XM_068212999.1
A2G0670	111.60	13.03	LOC107718023	A0A673NE78_9TELE Cystatin-like protein	
A8G0688	111.58	92.03	COX6A	Cytochrome c oxidase subunit 6A, mitochondrial	
B23G0644	111.25	120.51	CD63	CD63 antigen	NM_199543.1
A16G0326	110.94	169.87	PLAC8L1	PLAC8-like protein 1	
B2G0605	110.90	47.71	CCR9	C-C chemokine receptor type 9	NM_001244716.1
A14G0331	110.77	44.40	UQCRQ	Cytochrome b-c1 complex subunit 8	
B23G0040	110.76	55.27	LOC107564799	A0A672NXC7_SINGR Cellular nucleic acid-binding protein-like	
A9G0115	110.69	33.14	KRT18	Keratin, type I cytoskeletal 18	XM_009304971.3
B13G0509	110.42	153.99	FGF8A	Fibroblast growth factor 8	
B8G0651	110.18	51.57	MAGOH	Protein mago nashi homolog	NM_001017700.1
B17G0226	110.16	129.81	NFKBIA	NF-kappa-B inhibitor alpha	NM_199629.1
A25G0045	110.15	98.97	SLC25A3	Phosphate carrier protein, mitochondrial	NM_213722.1
A13G0674	109.72	71.40	TLR4	Toll-like receptor 4	NM_214759.1
A5G0852	109.56	135.67	COX7C	Cytochrome c oxidase subunit 7C, mitochondrial	NM_199995.1
B4G0930	109.54	101.25	CCT2	T-complex protein 1 subunit beta	NM_201455.1
B7G0419	109.51	34.68	CD44	CD44 antigen	
A9G0128	109.22	9.57	AGR3	Anterior gradient protein 3	NM_001099232.2
B3G0599	108.99	99.14	ATP5MF	ATP synthase subunit f, mitochondrial	
A5G0255	108.41	37.77	RANBP1	Ran-specific GTPase-activating protein	NM_212766.2
A21G0694	108.07	20.82	SLCO2B1	Solute carrier organic anion transporter family member 2B1	NM_001037678.2

A14G0250	108.02	43.41	NDUFA1	NADH dehydrogenase [ubiquinone] 1 alpha subcomplex subunit 1	
A25G0435	107.90	51.17	PDIA3	Protein disulfide-isomerase A3	NM_001199737.1
B13G0794	107.86	143.72	ZBTB8OS	Protein archease	NM_131883.2
A14G0533	107.86	28.99	DDIT4L	DNA damage-inducible transcript 4-like protein	NM_001258317.1
B17G0204	107.54	68.98	PSMA3	Proteasome subunit alpha type-3	NM_001030187.1
A11G0468	107.46	88.04	CFLAR	CASP8 and FADD-like apoptosis regulator	NM_201188.1
B10G0236	107.45	11.77	PPP1R3CB	Protein phosphatase 1 regulatory subunit 3C-B	
A17G0693	107.37	51.61	PSMA3	Proteasome subunit alpha type-3	NM_001030187.1
B5G1174	107.27	61.27	LOC113048829	A0A6P6K3U0_CARAU non-histone chromosomal protein HMG-14A-like	NM_001080033.2
A11G0211	106.15	108.95	PLPPR3	Phospholipid phosphatase-related protein type 3	NM_001024429.2
A8G0499	105.87	104.71	ATP5PB	ATP synthase F(0) complex subunit B1, mitochondrial	NM_001005960.1
B24G0342	105.62	31.30	COPS9	COP9 signalosome complex subunit 9	
B11G0196	105.62	119.55	ZRSR2	U2 small nuclear ribonucleoprotein auxiliary factor 35 kDa subunit-related protein 2	NM_001024429.2
A2G0169	105.49	45.25	GNG5	G(O) subunit gamma-5	NM_214721.2
A1G0216	105.16	63.28	SMDT1	Essential MCU regulator, mitochondrial	
B14G0524	105.04	57.10	HNRNPAB	B	NM_212587.3
A4G0999	104.92	135.96	MTPN	Myotrophin	NM_212972.2
A21G0029	104.13	60.47	OAZ1B	Ornithine decarboxylase antizyme 2	NM_194432.3
B21G0405	103.64	60.07	PRDX5	Peroxiredoxin-5, mitochondrial	
B13G0691	103.50	79.31	CNIH1	Protein cornichon homolog 1	NM_199585.3
B2G0953	103.16	80.69	GLUL	Glutamine synthetase	NM_181559.2
B8G0475	102.73	59.58	CAPZA1	F-actin-capping protein subunit alpha-1	NM_001006663.1
A20G0131	102.71	19.71	RAB10	Ras-related protein Rab-10	NM_001002566.1
B14G0689	102.67	77.95	EGR1	Early growth response protein 1	NM_131248.1
B2G0640	102.37	162.49	ROHU_024221	A0A498LJX8_LABRO Uncharacterized protein	
B3G1208	102.18	57.64	ATF4	Cyclic AMP-dependent transcription factor ATF-4	NM_001103192.2
A2G0937	101.95	93.47	GTPBP4	GTP-binding protein 4	NM_199851.1
B6G0729	101.89	66.23	H1-10	Histone H1.10	NM_199276.1
B2G0132	101.81	32.84	DAD1	Dolichyl-diphosphooligosaccharide--protein glycosyltransferase subunit dad1	
B22G0320	101.77	34.71	SMC5	Structural maintenance of chromosomes protein 5	
B3G0432	101.33	82.95	ATP5PD	ATP synthase subunit d, mitochondrial	NM_200702.1
A22G0497	101.27	78.57	PTPRC	Receptor-type tyrosine-protein phosphatase C	XM_068216640.1
B22G0083	101.11	108.96	BTG2	Protein BTG2	NM_130922.2
B7G0825	100.91	102.16	EIF4A1	Eukaryotic initiation factor 4A-I	NM_198366.2
A10G0327	100.89	173.41	HMGB1	High mobility group protein B1	NM_199555.3
A9G0370	100.76	7.11	MID1IP1A	Mid1-interacting protein 1A	NM_131335.1
A18G0547	100.70	74.83			NM_213486.1

A22G0459	100.53	113.35	OAZ1A	Ornithine decarboxylase antizyme 1	NM_194264.2
B5G0086	99.83	30.19	CYTB	Cystatin-B	
B16G0184	99.50	79.39	FXYD1	Phospholemman	NM_001256212.1
B21G0140	99.49	95.38	HMGB3	High mobility group protein B3	NM_001017769.1
B1G0927	99.47	83.72	CCT4	T-complex protein 1 subunit delta	NM_200583.1
A14G0536	99.08	67.56	UBE2D2	Ubiquitin-conjugating enzyme E2 D2	NM_200959.3
B8G0205	99.02	75.34	YWHABB	alpha-B	NM_213145.1
B16G0658	98.97	85.52	YWHABA	alpha-A	NM_199562.1
B17G0801	98.92	45.88	FOSL2	Fos-related antigen 2	NM_001082998.2
A24G0123	98.91	161.53	HIS3.3A	Histone H3.3A	NM_201101.1
A17G0625	98.76	41.32	COL10A1	Collagen alpha-1(X) chain	NM_001083827.1
A21G0024	98.63	27.02	KLF4	Krueppel-like factor 4	NM_001113483.1
A11G0205	98.10	50.09	SID1	E9QH29_DANRE Secreted immunoglobulin domain 1	NM_200694.1
A14G0060	97.71	134.07	CCNG1	Cyclin-G1	NM_199481.1
A6G0926	97.70	73.02	RBM5	RNA-binding protein 5	NM_001100138.3
A22G0516	97.61	13.94	RGS21	Regulator of G-protein signaling 21	XM_001923553.8
B3G1229	97.51	42.33	ATP5MC2	ATP synthase F(0) complex subunit C2, mitochondrial	NM_213028.1
B17G0434	97.31	18.74	ATP5IF1B	ATPase inhibitor B, mitochondrial	NM_001044859.1
B10G0577	97.16	130.03	HMGB1	High mobility group protein B1	NM_199555.3
A2G0656	97.00	33.80	TRB	M1-specific T cell receptor beta chain	
B20G0814	96.95	91.82	TMED10	Transmembrane emp24 domain-containing protein 10	NM_214698.2
A3G0494	96.87	61.32	ARPC1A	3 complex subunit 1A	NM_201343.2
A10G0284	96.61	95.98	LOC113054513	A0A6P6KUA8_CARAU high mobility group nucleosome-binding domain-containing protein 3-like	NM_001386720.1
A13G0781	96.57	95.37	G5714_013141	A0A7J6CDU4_9TELE Uncharacterized protein	NM_213128.1
B2G1224	96.50	95.27	HIGD1A	HIG1 domain family member 1A, mitochondrial	NM_200100.2
B21G0779	96.43	91.35	TBCA	Tubulin-specific chaperone A	NM_201054.1
B11G0779	96.13	158.26	NLRC3	Protein NLRC3	XM_009305712.4
A7G1202	96.01	0.47	CXCL8	Interleukin-8	
B6G0335	95.93	47.73	PSMB3	Proteasome subunit beta type-3	NM_001129823.1
B5G1121	95.79	43.26	EIF4A1	Eukaryotic initiation factor 4A-I	NM_201510.2
B2G0069	95.75	88.76	JUND	Transcription factor JunD	NM_001128342.1
B4G0119	95.52	10.84	COA6	Cytochrome c oxidase assembly factor 6 homolog	NM_001327831.1
A7G1005	95.51	14.40	RNF183	E3 ubiquitin-protein ligase RNF183	
B14G0401	95.38	241.70			
A9G0026	95.35	10.38	LOC113052885	A0A6P6KPN9_CARAU protein TsetseEP-like	
A17G0137	95.28	72.41	NPC2	NPC intracellular cholesterol transporter 2	NM_173224.1
B20G0902	95.23	46.94	EEF1D	Elongation factor 1-delta	XM_017352493.3
A23G0218	95.16	163.17	KRT18	Keratin, type I cytoskeletal 18	NM_178437.2
A12G0044	95.15	19.75	DDIT4	DNA damage-inducible transcript 4 protein	NM_200107.2
A18G0014	95.07	7.97	LGALS4	Galectin-4	XM_005173704.5
A19G0424	94.86	43.53	CFAD	Counting factor associated protein D	NM_001037120.2

A3G0806	94.78	44.25	RAC1	Ras-related C3 botulinum toxin substrate 1	NM_001002061.1
B3G0476	94.72	376.98	HEBP2	Heme-binding protein 2	
B6G0072	94.26	28.26	TGM5	Protein-glutamine gamma-glutamyltransferase 5	
B16G0059	94.26	37.95	GRINA	Protein lifeguard 1	
A20G0332	94.18	51.43	SF3B6	Splicing factor 3B subunit 6	NM_001002067.1
A19G0919	93.98	66.51	GPD1	Glycerol-3-phosphate dehydrogenase [NAD(+)], cytoplasmic	NM_214753.2
A1G0529	93.79	61.79	SPCS3	Signal peptidase complex subunit 3	NM_212948.1
A9G0303	93.76	57.46	ATP5PO	ATP synthase subunit O, mitochondrial	NM_001003843.1
A20G0743	93.54	65.87	NFKBIA	NF-kappa-B inhibitor alpha	NM_213184.2
A12G0712	93.52	8.45	DLX3B	Homeobox protein Dlx3b	NM_131322.2
A1G0266	93.27	168.26	A0A673IEN9	A0A673IEN9_9TELE Ubiquitin-like domain-containing protein	
A14G0563	93.04	132.53	TUBB4B	Tubulin beta-4B chain	NM_198809.2
A5G0103	93.01	43.84	OSTF1	Osteoclast-stimulating factor 1	NM_212857.2
B11G0484	92.75	0.92	CSTF1	Cleavage stimulation factor subunit 1	
B1G1112	92.67	31.96	SMC5	Structural maintenance of chromosomes protein 5	
A17G0703	92.58	27.03	LGALS3	Galectin-3	NM_214693.3
A14G0116	92.30	41.67	G3BP1	Ras GTPase-activating protein-binding protein 1	XM_005173102.5
A7G1035	91.97	27.33	SNRPF	Small nuclear ribonucleoprotein F	NM_001003881.3
A14G0241	91.97	24.90			
A16G0646	91.95	49.93	S100B	Protein S100-B	NM_001326451.1
B1G0537	91.77	92.67	CD99L2	CD99 antigen-like protein 2	
B3G0344	91.74	39.38	ELOF1	Transcription elongation factor 1 homolog	NM_200386.1
B13G0749	91.63	34.33	K02A2.6	Uncharacterized protein K02A2.6	
A13G0795	91.61	96.03	RTASE	Probable RNA-directed DNA polymerase from transposon BS	NR_155507.1
B6G0908	91.33	62.50	YWHABA	alpha-A	NM_001082798.1
B25G0788	91.23	65.57	TMEM258	Transmembrane protein 258	
A10G0489	91.14	24.18	FAU	FAU ubiquitin-like and ribosomal protein S30	NM_001017866.2
B22G0201	91.11	65.38	TMA7	Translation machinery-associated protein 7	NM_001159834.3
B17G0611	91.08	110.98	CTSB	Cathepsin B	NM_213336.2
A6G0979	91.02	37.04	SNRPN	Small nuclear ribonucleoprotein-associated protein N	NM_205667.1
B1G0655	90.86	82.12	PAIP2B	Polyadenylate-binding protein-interacting protein 2B	NM_200766.1
B22G0861	90.61	32.15	CAPN2	Calpain-2 catalytic subunit	NM_001018227.1
B25G0694	90.30	4.62	MUC2	Mucin-2 (Fragments)	XM_068217494.1
A6G0836	89.92	34.93	CAPZA1	F-actin-capping protein subunit alpha-1	NM_212697.1
B11G0137	89.91	60.10	PRSS2	Anionic trypsin-2	NM_213031.3
B19G0884	89.87	71.68	STMN1	Stathmin	NM_001040375.1
B16G0843	89.75	11.02	CRABP2	Cellular retinoic acid-binding protein 2	NM_182859.1
B12G0464	89.73	24.67	RSL1D1	Ribosomal L1 domain-containing protein 1	NM_001326710.1

B21G0364	89.48	44.51	COX8B	Cytochrome c oxidase subunit 8B, mitochondrial	
A8G0420	89.47	35.36	CSDE1	Cold shock domain-containing protein E1	XM_068222882.1
B14G0360	89.34	17.33	MID1IP1L	Mid1-interacting protein 1-like	
A13G0228	89.34	124.35	CD274	Programmed cell death 1 ligand 1	NM_199585.3
B9G0703	89.18	33.11	ECRG4A	Augurin-A	NM_001017697.1
A7G1226	89.06	92.16	PNP	Purine nucleoside phosphorylase	
A21G0880	88.91	62.72	CCT6A	T-complex protein 1 subunit zeta	NM_201290.1
A22G0351	88.80	28.40	IER5	Immediate early response gene 5 protein	NM_001007197.1
B22G0767	88.79	50.87	OAZ1A	Ornithine decarboxylase antizyme 1	NM_194264.2
A6G0390	88.78	71.17	P4HB	Protein disulfide-isomerase	NM_213364.3
B21G0722	88.63	46.13	CAST	Calpastatin	XM_068215911.1
B7G1196	88.61	10.07	CT027	UPF0687 protein C20orf27 homolog	NM_001436955.1
A20G0386	88.56	38.56	ERH	Enhancer of rudimentary homolog	NM_001271817.1
A6G0737	88.30	222.42	FKBP5	Peptidyl-prolyl cis-trans isomerase FKBP5	NM_213149.2
A14G0186	88.25	24.82	PRSS23	Serine protease 23	NM_001423236.1
B23G0421	88.24	37.54	RPL22	60S ribosomal protein L22	
B6G0087	88.24	70.12	UQCRH	Cytochrome b-c1 complex subunit 6, mitochondrial	
B3G0581	87.80	59.37	EIF3B	Eukaryotic translation initiation factor 3 subunit B	NM_001290093.1
A10G0876	87.75	66.94	HINT1	Adenosine 5'-monophosphoramidase HINT1	NM_001005593.2
A3G0631	87.75	4.90	DLX4A	Homeobox protein Dlx4a	NM_131300.2
A19G0288	87.63	159.40	FABP3	Fatty acid-binding protein, heart	NM_001004682.1
A14G0324	87.58	33.37	CSNK1A1	Casein kinase I isoform alpha	XM_009291086.4
B17G0442	87.40	31.51	ZNF593	Zinc finger protein 593	NM_200817.2
B19G0603	87.31	53.89	AK2	Adenylate kinase 2, mitochondrial	XM_005159648.4
A6G0430	87.08	14.35	GLUL	Glutamine synthetase	NM_182866.3
B20G0198	86.94	76.99	NFKBIA	NF-kappa-B inhibitor alpha	NM_213184.2
B12G0642	86.71	37.84	SNU13	NHP2-like protein 1	NM_199535.2
A13G0685	86.60	97.21	TET3	Methylcytosine dioxygenase TET3	NM_200867.1
B3G1123	86.58	74.66	NDUFA4	Cytochrome c oxidase subunit NDUFA4	NM_213417.2
A19G0817	86.37	58.74	HA1B	BOLA class I histocompatibility antigen, alpha chain BL3-7	NM_131705.1
B4G0304	86.05	68.35	GDI2	Rab GDP dissociation inhibitor beta	NM_199655.1
A2G0678	86.05	87.67	CCR9	C-C chemokine receptor type 9	NM_001244716.1
B12G0234	86.02	61.88	SRSF2	arginine-rich splicing factor 2	NM_213382.1
B4G0418	85.93	82.97	BTG1	Protein BTG1	NM_200020.1
B10G0455	85.72	84.57	HIST2H2L	Histone H2B 3	NM_200117.1
B10G0745	85.69	82.95	H2AZ1	Histone H2A.Z	NM_153644.1
B22G0972	85.57	68.63	LSM3	U6 snRNA-associated Sm-like protein LSm3	NM_001386830.1
A6G0081	85.40	59.68	UQCRH	Cytochrome b-c1 complex subunit 6, mitochondrial	
B10G0296	85.30	36.81	NPM1	Nucleophosmin	NM_199428.2
A9G0330	85.07	15.00	HSPE1	10 kDa heat shock protein, mitochondrial	
A21G0550	84.93	71.12	PRDX5	Peroxiredoxin-5, mitochondrial	NM_001024406.1

A1G0838	84.84	19.36			NM_199602.2
B5G0051	84.71	10.27	PLA2G4C	Cytosolic phospholipase A2 gamma	NM_001204308.2
A25G0124	84.68	44.72	PSMA4	Proteasome subunit alpha type-4	NM_214697.1
A24G0466	84.33	80.94	EIF2S3	Eukaryotic translation initiation factor 2 subunit 3	NM_212711.3
A12G0336	84.32	58.99	CHMP2A	Charged multivesicular body protein 2a	NM_213517.1
B8G0399	84.25	472.70	SEPP1A	Selenoprotein Pa	NM_178297.4
B21G0949	84.12	68.27	OAZ1B	Ornithine decarboxylase antizyme 2	NM_194432.3
A19G0344	84.11	61.04	NDUFS5	NADH dehydrogenase [ubiquinone] iron-sulfur protein 5	NM_001020633.1
A2G1091	84.09	43.24	EEF1D	Elongation factor 1-delta	XM_017358747.3
A8G0008	84.01	42.91	TMED7	Transmembrane emp24 domain-containing protein 7	NM_001313710.1
A1G0035	83.96	58.34	UBL5	Ubiquitin-like protein 5	
B9G0364	83.88	128.33	SF3B1	Splicing factor 3B subunit 1	XM_005167788.5
A15G0415	83.73	124.83	LOC107744792	A0A673GT44_9TELE Hypoxia-inducible factor 1-alpha-like	NM_200405.1
A21G0232	83.68	18.89	NOP56	Nucleolar protein 56	NM_201217.2
B8G0873	83.68	49.38	PIM1	threonine-protein kinase pim-1	NM_001077391.1
A14G0137	83.24	76.36	AHNAK	Neuroblast differentiation-associated protein AHNAK	XM_005173182.5
B11G0164	83.22	5.41	GAMT	Guanidinoacetate N-methyltransferase	XM_005155731.5
A25G0556	83.03	13.37	PKP3	Plakophilin-3	NM_001045280.1
A16G0115	82.94	43.08	EIF3HA	Eukaryotic translation initiation factor 3 subunit H-A	NM_001003763.2
B22G0693	82.90	70.24	HNRNPM	Heterogeneous nuclear ribonucleoprotein M	NM_001256631.1
A17G0193	82.78	5.84	CAPN13	Calpain-13	XM_017351848.3
B19G0135	82.65	231.33	EIF1B	Eukaryotic translation initiation factor 1b	NM_199588.3
A10G0634	82.51	37.03	PBDC1	Protein PBDC1	
B21G0558	82.29	42.76	EIF4EBP2	Eukaryotic translation initiation factor 4E-binding protein 2	NM_199645.1
B20G0273	81.75	28.08	DSG2	Desmoglein-2	NM_001256637.1
B14G0695	81.66	57.62	LYG	Lysozyme g	NM_001002706.1
A25G0217	81.36	213.37	CCL24	C-C motif chemokine 24	
A1G0029	81.13	15.31	MFAP4	Microfibril-associated glycoprotein 4	NM_001386618.1
A10G0095	80.99	43.38	TMED2	Transmembrane emp24 domain-containing protein 2	XM_021479108.2
B11G0583	80.90	41.08	TRAIP	E3 ubiquitin-protein ligase TRAIP	NM_201004.2
A5G0810	80.89	14.41	AQP3	Aquaporin-3	NM_213468.1
A6G0600	80.89	26.67	RGS5	Regulator of G-protein signaling 5	NM_199962.2
B9G0633	80.85	37.18	ITGAV	Integrin alpha-V	NM_001033721.1
B3G0220	80.84	29.49	TXN2	Thioredoxin, mitochondrial	NM_205641.1
A15G0093	80.76	82.40	ACTN4	Alpha-actinin-4	XM_021481336.2
A24G0274	80.75	33.74	LOC107729645	A0A673I049_9TELE C-C motif chemokine 8-like	
A1G0875	80.49	64.84	EIF3F	Eukaryotic translation initiation factor 3 subunit F	NM_001200009.1
A15G0743	80.32	72.62	LOC113098369	A0A6P6PEB8_CARAU non-histone chromosomal protein HMG-14A-like	NM_001309644.1

A8G0735	80.31	96.15	H2-EB1	H-2 class II histocompatibility antigen, I-E beta chain	NM_001007206.2
B20G0627	80.26	37.79	SF3B6	Splicing factor 3B subunit 6	NM_001002067.1
B21G0245	80.09	46.97	SLCO2B1	Solute carrier organic anion transporter family member 2B1	NM_001037678.2
A5G0681	80.02	80.52	HIS3.3A	Histone H3.3A	NM_200003.1
A16G0725	80.02	107.24	TPI1B	Triosephosphate isomerase B	NM_153668.4
B18G0213	79.91	43.23	COX41	Cytochrome c oxidase subunit 4 isoform 1, mitochondrial	
B9G0310	79.84	45.57	MPC2	Mitochondrial pyruvate carrier 2	NM_212592.1
A22G0599	79.68	10.51	ABI3BP	Target of Nesh-SH3	XM_068216228.1
A5G0411	79.51	68.46	EWSR1	RNA-binding protein EWS	NM_212630.1
B7G0341	79.46	66.14	CCT7	T-complex protein 1 subunit eta	NM_173248.1
B15G0536	79.29	31.46	EPAS1	Endothelial PAS domain-containing protein 1	NM_200405.1
B24G0248	79.23	37.41	EIF1B	Eukaryotic translation initiation factor 1b	NM_200303.2
A15G0434	79.22	55.56	YWHAE	14-3-3 protein epsilon	NM_212605.2
B23G0248	79.19	206.80	KRT18	Keratin, type I cytoskeletal 18	NM_178437.2
B17G0378	79.17	27.61	MTLN	Mitoregulin	XM_068218872.1
B14G0542	79.05	53.72	UBE2D2	Ubiquitin-conjugating enzyme E2 D2	NM_200959.3
A7G0004	78.96	68.57	DCTN6	Dynactin subunit 6	
B2G0561	78.87	35.02	TRA	M1-specific T cell receptor alpha chain	
A14G0748	78.55	55.04	RGCC	Regulator of cell cycle RGCC	NM_001386477.1
B4G0087	78.54	75.56	NAP1L1	Nucleosome assembly protein 1-like 1	XM_021474937.2
B2G0701	78.51	8.03	G5714_001849	A0A7J6DDA9_9TELE Uncharacterized protein	XM_021467462.2
A20G0443	78.34	57.97	YWHAZ	delta	NM_201513.1
A17G0434	78.31	10.73	KRTCAP3	Keratinocyte-associated protein 3	XM_005158499.5
B7G1099	78.15	45.96	LAMP1	Lysosome-associated membrane glycoprotein 1	
B19G0805	78.04	137.54	TXNIP	Thioredoxin-interacting protein	NM_200087.1
B4G0943	78.02	39.96	G5714_002636	A0A7J6D7B7_9TELE Uncharacterized protein	
A6G0518	77.79	79.45	ATNB233	potassium-transporting ATPase subunit beta-233	NM_131668.3
A20G0364	77.52	26.64	ARPC5-A	3 complex subunit 5-A	XM_068215400.1
B2G0452	77.46	48.56	CCNL1	Cyclin-L1	NM_199740.1
A25G0222	77.39	223.38	CCL13	C-C motif chemokine 13	
B21G0059	77.27	48.91	PPP2CA	threonine-protein phosphatase 2A catalytic subunit alpha isoform	NM_200911.3
B21G0183	77.24	117.42	COX7B	Cytochrome c oxidase subunit 7B, mitochondrial	NM_001130602.1
A23G0588	77.21	37.05	HMGN3	High mobility group nucleosome-binding domain-containing protein 3	NM_001243173.1
B9G0813	77.03	54.36	DBI	Acyl-CoA-binding protein	
B16G1102	77.01	20.16	RRP15	RRP15-like protein	
A21G0451	76.89	125.36	C1QB	Complement C1q subcomponent subunit B	
B6G0471	76.76	83.87	HDLBP	Vigilin	XM_005165939.5
B21G0874	76.74	92.42	HNRNPAB	B	NM_213302.1
B5G0639	76.55	48.38	HAPLN1	Hyaluronan and proteoglycan link protein 1	NM_001007790.2
B18G0818	76.44	62.27	COX5A	Cytochrome c oxidase subunit 5A, mitochondrial	NM_001305577.1
B1G0876	76.25	45.13	JUNB	Transcription factor JunB	NM_213556.3

B7G1275	76.18	2.45	FRRS1	Putative ferric-chelate reductase 1	
B16G0193	76.15	210.59	GAPDH-2	Glyceraldehyde-3-phosphate dehydrogenase 2	NM_213094.2
A9G0790	76.14	29.02	ATP5MC3	ATP synthase F(0) complex subunit C3, mitochondrial	NM_201176.1
B4G0429	76.08	147.80	LDHBA	L-lactate dehydrogenase B-A chain	NM_131247.1
A5G1278	75.98	26.62	GIMAP9	GTPase IMAP family member 9	XM_009299019.4
A3G0717	75.97	0.18			
B3G0855	75.88	18.03	RRAS2	Ras-related protein R-Ras2	NM_001005931.2
A6G0656	75.75	51.12	KRT8	Keratin, type II cytoskeletal 8	NM_131509.2
B15G0515	75.73	109.70	YWHAE	14-3-3 protein epsilon	NM_212605.2
A11G0656	75.71	121.72	PTPRG	Receptor-type tyrosine-protein phosphatase gamma	NM_200632.2
A23G0612	75.63	65.31	CD63	CD63 antigen	NM_199543.1
B24G0502	75.63	61.18	SEC61G	Protein transport protein Sec61 subunit gamma	NM_001002499.3
A20G0066	75.34	23.05	EEF1D	Elongation factor 1-delta	NM_001030147.2
A22G0108	75.32	34.76	CALR	Calreticulin	
A19G0556	75.32	20.96	GSDME	Gasdermin-E	
B10G0586	75.29	54.91	UCP2	Mitochondrial uncoupling protein 2	NM_131176.1
B18G0844	75.24	25.17	CDC42SE2	CDC42 small effector protein 2	XR_658791.4
B22G0608	75.23	36.76	BZW1A	eIF5-mimic protein 2-A	NM_199708.1
B24G0690	75.16	56.33	KLF6	Krueppel-like factor 6	NM_201461.2
B10G0418	74.96	27.19	FAU	FAU ubiquitin-like and ribosomal protein S30	
A20G0062	74.90	18.77	DSP	Desmoplakin	NM_001423427.1
A11G0009	74.82	64.42	HNRNPLL	Heterogeneous nuclear ribonucleoprotein L-like	XM_009305712.4
B16G0155	74.77	60.80	LOC107756903	A0A673MWB8_9TELE Uncharacterized LOC107756903	XM_009292662.4
A3G0877	74.62	33.36	RAB5C	Ras-related protein Rab-5C	NM_201501.1
A11G0295	74.62	36.50	LUM	Lumican	NM_001003762.1
A18G0588	74.51	43.81	HSBP1	Heat shock factor-binding protein 1	NM_200754.2
B10G0630	74.51	50.72	LOC113110020	A0A6P6Q8F9_CARAU non-histone chromosomal protein HMG-14A-like	NM_001386720.1
B22G0642	74.45	59.44	KLF2	Krueppel-like factor 2	NM_131856.3
A21G0106	74.44	50.83	ATP5F1A	ATP synthase subunit alpha, mitochondrial	NM_001077355.1
A11G0771	74.41	44.55	EDN3	Endothelin-3	NM_199576.1
A15G0020	74.34	28.78	EIF3K	Eukaryotic translation initiation factor 3 subunit K	XM_017350953.3
A10G0408	74.29	115.41	MDH2	Malate dehydrogenase, mitochondrial	NM_213131.1
A10G0122	74.27	29.08	DENR	Density-regulated protein	NM_001002697.2
A25G0340	74.20	103.32	CPA1	Carboxypeptidase A1	NM_199271.1
B23G0333	74.19	52.35	SSR4	Translocon-associated protein subunit delta	NM_001002082.1
A1G0159	74.14	40.61	NDUFB7	NADH dehydrogenase [ubiquinone] 1 beta subcomplex subunit 7	
B8G0458	73.84	75.95	CEBPB	enhancer-binding protein beta	NM_131884.2
A2G0797	73.60	48.79	CCNL1	Cyclin-L1	NM_199740.1
B6G0749	73.48	246.11	FKBP5	Peptidyl-prolyl cis-trans isomerase FKBP5	NM_213149.2
B16G0149	73.34	33.40	A0A673MWB5	A0A673MWB5_9TELE Transmembrane protein 176l.2	NM_001386366.1

A23G0034	73.23	16.06			NM_001244736.1
B11G0509	73.14	75.45	PLEKHA3	Pleckstrin homology domain-containing family A member 3	XM_691072.9
A24G0109	73.12	19.93	PXN1	Pentraxin fusion protein	
B15G0931	73.08	45.19	LOC107710511	A0A673LFR8_9TELE T-cell surface glycoprotein CD3 delta chain-like	
B7G0630	73.05	9.46	TPPP3	Tubulin polymerization-promoting protein family member 3	NM_201335.2
A6G0226	73.05	73.74	CYC	Cytochrome c	NM_001002068.1
A1G0278	72.94	174.44	PRDX1	Peroxiredoxin-1	NM_001002468.2
A8G0853	72.88	0.48	NKX3-1	Homeobox protein Nkx-3.1	NM_001386325.1
A24G0468	72.73	70.52	CCT5	T-complex protein 1 subunit epsilon	NM_212613.1
A2G0721	72.73	23.51	G5714_001966	A0A7J6DDM8_9TELE Uncharacterized protein	
A20G0147	72.59	31.38	TMED10	Transmembrane emp24 domain-containing protein 10	NM_214698.2
B6G0522	72.56	44.39	NDUFB5	NADH dehydrogenase [ubiquinone] 1 beta subcomplex subunit 5, mitochondrial	NM_001105596.1
A5G0667	72.54	17.35	CAPNS1	Calpain small subunit 1	NM_001017899.2
B15G0185	72.47	44.50	LOC113115337	A0A6P6QZK5_CARAU non-histone chromosomal protein HMG-14A-like	NM_001309644.1
B16G0157	72.43	40.54	RPZ	Protein rapunzel	NM_001110124.1
B3G1069	72.41	116.20	ATP6V0C	V-type proton ATPase 16 kDa proteolipid subunit c	NM_001105136.2
B13G0350	72.33	29.98	TRIML1	Probable E3 ubiquitin-protein ligase TRIML1	
B18G0002	72.28	55.83	TALDO1	Transaldolase	NM_199687.1
B24G0528	72.24	58.97	CCT5	T-complex protein 1 subunit epsilon	NM_212613.1
A4G0498	72.21	54.20	NDUFA12	NADH dehydrogenase [ubiquinone] 1 alpha subcomplex subunit 12	
B16G0217	72.13	70.80	PSMA2	Proteasome subunit alpha type-2	NM_001024441.1
A3G0719	72.09	21.31	SI:DKEY-16P21.8	X1WCS1_DANRE Si:dkey-16p21.8	NR_170131.1
A21G0678	72.03	35.47	POMP	Proteasome maturation protein	NM_001003424.2
A12G0666	72.01	23.23	SNU13	NHP2-like protein 1	NM_199535.2
B20G0861	71.90	125.63	COX7A2	Cytochrome c oxidase subunit 7A2, mitochondrial	
B24G0733	71.75	86.63	TC1A	Transposable element Tc1 transposase	
A19G0458	71.69	32.44	CAP1	Adenylyl cyclase-associated protein 1	NM_199909.2
A15G0275	71.68	15.17	POSTN	Periostin	NM_001077786.1
A9G0005	71.67	53.26	ACTR3	Actin-related protein 3	NM_001003944.1
A6G0666	71.63	32.87	PFDN5	Prefoldin subunit 5	NM_001111195.1
A18G0229	71.57	40.65	PDCD5	Programmed cell death protein 5	NM_201177.1
A23G0391	71.52	22.97	RPL22	60S ribosomal protein L22	
A14G0694	71.46	21.51	SELENOT2	Selenoprotein T2	NM_001098487.2
A23G0339	71.42	18.31	LOC107712726	A0A673LX88_9TELE Emerin-like	NM_001386483.1
A3G1055	71.38	57.07	ST13	Hsc70-interacting protein	NM_199769.2
B9G0103	71.36	48.98	DAPL1-B	Death-associated protein-like 1-B	
A9G0752	71.33	49.60	MARCO	Macrophage receptor MARCO	XM_068223528.1
B6G0155	71.29	31.16	PA2G4	Proliferation-associated protein 2G4	NM_001002070.2

B22G0006	71.18	18.39			
B5G0081	71.00	134.69	SAT1	Diamine acetyltransferase 1	NM_001002169.2
B11G0550	70.98	53.04	FAM126B	Protein FAM126B	NM_205708.1
A11G0114	70.89	16.86	NDUFS7	NADH dehydrogenase [ubiquinone] iron-sulfur protein 7, mitochondrial	NM_001198743.1
A21G0072	70.86	51.41	HNRNPAB	B	NM_213302.1
A8G0274	70.84	20.77	MRPL55	39S ribosomal protein L55, mitochondrial	NM_001111179.1
A5G0229	70.78	97.56	XBP1	X-box-binding protein 1	NM_131874.1
B6G0428	70.44	27.88	SUMO3	Small ubiquitin-related modifier 3	
A19G0821	70.36	57.94	PSMB9-A	Proteasome subunit beta type-9	NM_131391.2
B24G0525	70.22	65.49	EIF2S3	Eukaryotic translation initiation factor 2 subunit 3	NM_212711.3
B5G0925	70.22	97.15	ISCU	Iron-sulfur cluster assembly enzyme ISCU, mitochondrial	
A9G0334	70.21	81.74	SF3B1	Splicing factor 3B subunit 1	XM_005167788.5
A19G0072	70.06	35.44	STMN1	Stathmin	NM_001040375.1
A20G0513	70.05	2.71	FOXQ1	Forkhead box protein Q1	NM_212907.2
B8G0203	69.93	36.99	CHMP4C	Charged multivesicular body protein 4c	NM_213457.1
B4G0005	69.91	29.42	C11ORF98	Uncharacterized protein C11orf98	
A13G0639	69.87	80.35	ATRN	Attractin	
B9G0329	69.86	11.27	LADD	Ladderlectin	
A3G1181	69.84	87.13	ALYREF	THO complex subunit 4	NM_001105108.1
A5G0915	69.76	17.25	HINT2	Adenosine 5'-monophosphoramidase HINT2	NM_001083572.2
A10G0085	69.65	37.26	UBE2L3	Ubiquitin-conjugating enzyme E2 L3	NM_001002072.2
A10G0859	69.62	58.63	REEP5	Receptor expression-enhancing protein 5	NM_200058.1
B11G0770	69.59	16.49	TRAPPC10	Trafficking protein particle complex subunit 10	NM_001017757.2
A13G0082	69.54	69.30	TMEM63B	CSC1-like protein 2	
A7G1055	69.36	41.47	LOC113052273	A0A6P6KJP8_CARAU cilia- and flagella-associated protein 251-like	
A4G0538	69.07	16.04	LLPH	Protein LLP homolog	NM_001103140.2
B13G0630	68.80	389.94	VTCN1	V-set domain-containing T-cell activation inhibitor 1	NM_001020532.1
A21G0376	68.78	19.80	EIF4EBP2	Eukaryotic translation initiation factor 4E-binding protein 2	NM_199645.1
B3G0876	68.65	170.32	FRIM	Ferritin, middle subunit	NM_001109854.1
B14G0805	68.45	26.45	PRPS1	Ribose-phosphate pyrophosphokinase 1	NM_001076568.2
B5G1138	68.34	28.18	EIF1AX	Eukaryotic translation initiation factor 1A, X-chromosomal	NM_001006082.2
A8G0608	68.33	33.27	SNRPD3	Small nuclear ribonucleoprotein Sm D3	NM_001002081.1
A5G0393	68.33	26.90	EIF4A1	Eukaryotic initiation factor 4A-I	NM_201510.2
B11G0613	68.27	45.29	LOC113055722	A0A6P6L311_CARAU uncharacterized protein LOC113055722	NM_001002073.2
A3G0504	68.24	28.33	BUD31	Protein BUD31 homolog	NM_001003860.1
A19G0552	68.12	49.82	CBX3	Chromobox protein homolog 3	XM_005173769.4
B10G0667	68.01	40.18	ANXA4	Annexin A4	NM_181764.3
B6G0217	67.97	289.48	ITM2B	Integral membrane protein 2B	NM_199646.1
B2G0628	67.97	17.41	IGK	Immunoglobulin kappa light chain	

B22G0723	67.78	41.47	ATP5F1D	ATP synthase subunit delta, mitochondrial	NM_199968.1
A17G0465	67.78	54.27	ATP5IF1B	ATPase inhibitor B, mitochondrial	NM_001044859.1
B10G0662	67.78	27.15	PCNA	Proliferating cell nuclear antigen	NM_131404.2
B3G1708	67.63	4.90	LOC107713007	A0A673HMF0_9TELE Uncharacterized LOC107713007	
A1G0808	67.53	33.38	PCNP	PEST proteolytic signal-containing nuclear protein	NM_001326716.1
A19G0380	67.53	10.63	FAM133	Protein FAM133	NM_001080087.1
B20G0876	67.52	24.81	EIF2S1	Eukaryotic translation initiation factor 2 subunit 1	NM_131800.2
B7G0642	67.48	7.51	ESRP2	Epithelial splicing regulatory protein 2	XM_017356901.3
B9G0255	67.47	35.83	NIFK	MKI67 FHA domain-interacting nucleolar phosphoprotein	NM_173288.2
B21G0333	67.45	74.81	SNX12	Sorting nexin-12	XM_068215813.1
B13G0006	67.44	36.14	CCDC88A	Girdin	NM_200520.1
A22G0402	67.43	41.78	ATP5F1D	ATP synthase subunit delta, mitochondrial	NM_199968.1
A13G0079	67.42	22.40	SLC29A1	Equilibrative nucleoside transporter 1	
B5G0071	67.42	32.96	C1QBP	Complement component 1 Q subcomponent-binding protein, mitochondrial	NM_001017858.2
A19G0422	67.37	51.67	PSMB4	Proteasome subunit beta type-4 (Fragment)	NM_001017801.1
A24G0427	67.33	34.69	GARS1	Glycine--tRNA ligase	NM_001386453.1
A20G0362	67.31	72.81	IVNS1ABPA	Influenza virus NS1A-binding protein homolog A	NM_199279.2
B15G0916	67.28	75.65	HIS3.3A	Histone H3.3A	NM_001366916.1
B11G0336	67.03	55.46	ROHU_016730	A0A498NIF2_LABRO Uncharacterized protein	NM_001012304.1
A24G0418	66.94	22.20	CYTB	Cystatin-B	
A3G1056	66.90	35.63	RBX1	E3 ubiquitin-protein ligase RBX1	NM_001083544.1
A23G0138	66.90	24.28	PKP1	Plakophilin-1	XM_021469887.2
B3G0858	66.88	53.57	PRMT1	Protein arginine N-methyltransferase 1	NM_200650.2
B19G0323	66.88	60.28	SSR2	Translocon-associated protein subunit beta	NM_001001399.2
B23G0055	66.88	65.52	SRSF6	arginine-rich splicing factor 6	XM_005161922.5
B13G0857	66.80	53.72	ZBED1	E3 SUMO-protein ligase ZBED1	
A3G0006	66.76	51.99	CD9	CD9 antigen	NM_200552.1
A4G0657	66.65	49.73	STMP1	Short transmembrane mitochondrial protein 1	NM_001302634.1
B1G0796	66.57	14.86	CBR1	Carbonyl reductase [NADPH] 1	NM_194406.1
A13G0126	66.51	13.68	BTAF1	TATA-binding protein-associated factor 172	NM_001007059.1
B20G0281	66.44	36.18	PSMA7	Proteasome subunit alpha type-7	NM_213166.1
B20G0834	66.20	32.99	RAB10	Ras-related protein Rab-10	NM_001002566.1
A19G0607	66.19	44.14	SSR2	Translocon-associated protein subunit beta	NM_001001399.2
A12G0788	66.15	23.54	LSM12A	Protein LSM12 homolog A	NM_213148.1
A15G0784	66.09	38.35	EIF4H	Eukaryotic translation initiation factor 4H	NM_205695.2
B14G0107	66.08	38.50	G3BP1	Ras GTPase-activating protein-binding protein 1	XM_005173102.5
A13G0221	66.08	24.59	MAP3K9	Mitogen-activated protein kinase kinase kinase 9	NM_181765.2

B7G1231	66.01	187.23	PNP	Purine nucleoside phosphorylase	NM_213311.2
B2G0968	65.96	48.32	GSN	Gelsolin	NM_213090.2
B10G0398	65.93	29.32	FHL1	Four and a half LIM domains protein 1	NM_199217.1
A11G0407	65.91	40.02	MAP1LC3A	1B light chain 3A	NM_001012304.1
A2G0896	65.90	8.96	CCL20	C-C motif chemokine 20	
A18G0801	65.84	28.57	NMES1	Normal mucosa of esophagus-specific gene 1 protein	NM_001198748.1
B3G1240	65.83	555.52	COL1A1	Collagen alpha-1(I) chain	NM_199214.1
B4G0584	65.83	38.73	NDUFA12	NADH dehydrogenase [ubiquinone] 1 alpha subcomplex subunit 12	NM_001033103.2
A9G0017	65.79	46.35	PDCL3	Phosducin-like protein 3	NM_205721.1
B21G0989	65.66	59.81	KLF4	Krueppel-like factor 4	NM_001113483.1
A14G0781	65.50	38.04	HSPA9	Stress-70 protein, mitochondrial	NM_201326.2
B3G0991	65.41	41.22	ARHGDIB	Rho GDP-dissociation inhibitor 2	NM_001423647.1
B5G1036	65.40	39.77	H1	Histone H1	NM_001017660.2
B14G0336	65.38	83.37	HNRNPA0	Heterogeneous nuclear ribonucleoprotein A0	NM_001281721.1
B14G0534	65.38	40.58	HARS	Histidine--tRNA ligase	NM_001302256.1
B21G0057	65.36	25.53	CDKN2AIPNL	CDKN2AIP N-terminal-like protein	
A14G0329	65.24	51.61	HNRNPA0	Heterogeneous nuclear ribonucleoprotein A0	NM_001281721.1
B2G0785	65.22	44.33	CNN2	Calponin-2	NM_213349.1
A4G0934	65.10	53.95	NDUFA5	NADH dehydrogenase [ubiquinone] 1 alpha subcomplex subunit 5	
B3G0005	65.02	80.51	CD9	CD9 antigen	
B6G0597	64.78	29.45	RGS5	Regulator of G-protein signaling 5	NM_199962.2
B2G1112	64.77	97.66	RGS3	Regulator of G-protein signaling 3	
B5G1031	64.42	32.37	TCIM	Transcriptional and immune response regulator	NM_001013472.2
B18G0692	64.04	51.42	ARF4	ADP-ribosylation factor 4	NM_199275.1
B5G1134	64.03	75.32	GABARAP	Gamma-aminobutyric acid receptor-associated protein	NM_001013260.1
B14G0332	64.00	66.73	CSNK1A1	Casein kinase I isoform alpha	XM_009291090.4
B23G0486	63.99	32.57	PA2G4	Proliferation-associated protein 2G4	NM_212641.2
A8G0811	63.93	34.48	YWHABB	alpha-B	NM_213145.1
A13G0208	63.84	82.71	CNIH1	Protein cornichon homolog 1	XM_005156643.5
B13G0384	63.83	13.40	A0A673GDE4	A0A673GDE4_9TELE Zgc:193505	XM_682373.10
A23G0462	63.80	31.79	PA2G4	Proliferation-associated protein 2G4	NM_212641.2
B24G0152	63.79	10.88	CG050	Uncharacterized protein C7orf50 homolog	
A15G0706	63.76	66.61	NDUFC2	NADH dehydrogenase [ubiquinone] 1 subunit C2	NM_001013517.1
A19G0795	63.73	181.34	EIF1B	Eukaryotic translation initiation factor 1b	NM_199588.3
B19G0605	63.53	26.82	NDUFS5	NADH dehydrogenase [ubiquinone] iron-sulfur protein 5	NM_001020633.1
A7G0077	63.42	12.61	PYCARD	Apoptosis-associated speck-like protein containing a CARD	

B9G0593	63.23	45.60	DPX16_19648	A0A3N0XKS2_ANAGA Cyclic AMP receptor-like protein A (Fragment)	NR_186846.1
A2G0515	63.21	77.26	CNN2	Calponin-2	NM_213349.1
B13G0922	63.17	42.03			
B5G1145	63.11	10.05	GBGT1	Globoside alpha-1,3-N-acetylgalactosaminyltransferase 1	XM_005165129.5
A14G0039	63.08	110.46	ROHU_017146	A0A498NHH0_LABRO Ubiquitin 1	
B6G0886	62.75	46.48	AHCY-B	Adenosylhomocysteinase B	NM_199218.1
A19G0653	62.72	67.03	U2AF2	Splicing factor U2AF 65 kDa subunit	NM_205689.1
B2G1160	62.66	54.82	AP2M1A	AP-2 complex subunit mu-A	NM_001291355.1
A5G0469	62.65	30.87	H1	Histone H1	NM_001017660.2
B25G0873	62.59	38.35	TWF1	Twinfilin-1	NM_199725.1
A15G0857	62.50	142.76	TAGLN	Transgelin	NM_001045467.1
B13G0714	62.28	57.66	KCNH5	Potassium voltage-gated channel subfamily H member 5	
B5G0109	62.24	27.12	OSTF1	Osteoclast-stimulating factor 1	NM_212857.2
B22G0550	62.24	39.54	MID1IP1B	Mid1-interacting protein 1-B	
B3G1311	62.20	40.43	IGL1	Immunoglobulin lambda-1 light chain	
A20G0461	62.17	72.71	SRSF5	arginine-rich splicing factor 5	NM_001002610.1
B6G0450	62.15	30.26	GSN	Gelsolin	NM_178131.3
B3G1377	62.14	9.86	G5714_002981	A0A7J6D8K1_9TELE Uncharacterized protein	
A21G0360	62.06	66.75	ATP5ME	ATP synthase subunit e, mitochondrial	NM_001099427.1
B4G0306	62.04	16.89	NET1	Neuroepithelial cell-transforming gene 1 protein	XM_021475011.2
B7G0673	61.96	44.68	ANP32A	Acidic leucine-rich nuclear phosphoprotein 32 family member A	NM_199986.1
B6G0208	61.83	15.38	NOP58	Nucleolar protein 58	NM_001009889.2
B1G0736	61.61	0.04	LOC113100281	A0A6P6PJA3_CARAU uncharacterized protein LOC113100281	XM_021476836.2
B16G0688	61.60	12.55	RAB13	Ras-related protein Rab-13	NM_201329.1
A3G0484	61.59	63.31	EIF3B	Eukaryotic translation initiation factor 3 subunit B	NM_001290093.1
B23G0306	61.53	31.40	TPD52L2	Tumor protein D54	XM_005162032.3
A14G0716	61.27	81.37	DUSP1	Dual specificity protein phosphatase 1	NM_213067.2
A2G0043	61.26	9.80	FOXQ1	Forkhead box protein Q1	NM_001243344.1
A19G0473	61.16	43.30	UQCRB	Cytochrome b-c1 complex subunit 7	
A1G1128	61.15	17.34	ANP32B	Acidic leucine-rich nuclear phosphoprotein 32 family member B	NM_212603.2
B2G1051	61.05	79.85	SERP1	Stress-associated endoplasmic reticulum protein 1	NM_001002573.3
A5G0207	61.05	33.44	SNRNP27	U6.U5 small nuclear ribonucleoprotein 27 kDa protein	NM_001002703.2
A10G0109	61.04	9.02	ELOVL7	Elongation of very long chain fatty acids protein 7	NM_199778.1
B2G1086	60.96	52.28	EIF4A2	Eukaryotic initiation factor 4A-II	NM_213451.2
A19G0792	60.94	0.47	K1C1	Keratin, type I cytoskeletal 50 kDa	XM_001342753.9

A5G0714	60.93	14.10	MPZL2	Myelin protein zero-like protein 2	NM_212763.2
B14G0069	60.92	53.74	NDFIP1L	NEDD4 family-interacting protein 1-like	NM_199666.1
A13G0169	60.91	28.81	FRMD6	FERM domain-containing protein 6	
A14G0591	60.73	93.48	GSTP1	Glutathione S-transferase P	NM_131734.3
B2G0688	60.69	72.12	ELOC	Elongin-C	NM_001002440.2
A5G0677	60.66	28.35	SRSF7	arginine-rich splicing factor 7	NM_205673.1
B8G0195	60.63	92.52	TBB1	Tubulin beta-1 chain	NM_001037410.2
B16G0454	60.63	43.76	CDC42	Cell division control protein 42 homolog	NM_199865.3
B16G0343	60.51	3.83	BNIP2	adenovirus E1B 19 kDa protein-interacting protein 2	NM_001128394.1
A10G0455	60.50	56.40	H2A	Histone H2A	NM_001005967.1
B11G0665	60.50	27.16	CCDC50	Coiled-coil domain-containing protein 50	NM_001002297.1
A14G0518	60.49	53.57	HNRNPAB	B	NM_212587.3
B24G0467	60.47	35.12	FAM162B	Protein FAM162B	NM_001089343.2
B8G0422	60.46	16.59	SNRPD3	Small nuclear ribonucleoprotein Sm D3	NM_001002081.1
A19G0164	60.42	204.08	TXNIP	Thioredoxin-interacting protein	NM_200087.1
B7G0298	60.40	63.99	CHMP5	Charged multivesicular body protein 5	NM_200380.1
B20G0629	60.18	16.36	PLA2G7	Platelet-activating factor acetylhydrolase	NM_213189.2
A3G0907	60.17	45.78	LASP1	LIM and SH3 domain protein 1	NM_212636.1
B24G0469	60.16	20.93	CYTB	Cystatin-B	NR_133011.1
B5G0195	60.12	27.98	PES	Pescadillo	NM_131030.3
A5G0194	60.06	34.94	UBE2L3	Ubiquitin-conjugating enzyme E2 L3	NM_001082797.1
A25G0344	60.00	13.58			
B20G0398	59.96	34.73	AIRC	Multifunctional protein ADE2	XM_068215380.1
B23G0199	59.95	32.71	NAA10	N-alpha-acetyltransferase 10	NM_213334.1
B21G0830	59.95	137.94	RPL37	60S ribosomal protein L37	NM_001002069.4
A21G0820	59.89	57.76	NDUFA2	NADH dehydrogenase [ubiquinone] 1 alpha subcomplex subunit 2	NM_001024420.1
A12G0467	59.86	24.22	TOMM22	Mitochondrial import receptor subunit TOM22 homolog	NM_001001724.2
A9G0600	59.81	18.03	TRA2B	Transformer-2 protein homolog beta	NM_201197.1
A4G0531	59.71	21.74	NDUFB2	NADH dehydrogenase [ubiquinone] 1 beta subcomplex subunit 2, mitochondrial	
B10G0453	59.70	12.63			
A2G0208	59.66	49.06	G5714_021119	A0A7J6BQS2_9TELE Uncharacterized protein	
B25G0559	59.51	14.06	PARVA	Alpha-parvin	NM_001002872.2
B24G0389	59.47	40.29	EIF1AX	Eukaryotic translation initiation factor 1A, X-chromosomal	NM_001025453.1
A5G0146	59.47	19.51	IER3	Radiation-inducible immediate-early gene IEX-1	
A2G0456	59.36	34.28	SELENOF	Selenoprotein F	NM_178294.5
B18G0147	59.24	12.36	VASP	Vasodilator-stimulated phosphoprotein	XM_021473340.2
B7G0259	59.17	70.14	CDH1	Cadherin-1	XM_068222149.1
A21G0720	59.16	14.11	POF1B	Protein POF1B	XM_068218980.1
B14G0150	59.09	39.51	TSTD1	Thiosulfate:glutathione sulfurtransferase	
A16G0417	59.09	41.87	CDC42	Cell division control protein 42 homolog	NM_199865.3

A6G0474	59.01	59.35	HDLBP	Vigilin	XM_005165939.5
A19G0932	59.00	29.37	PLAAT4	Phospholipase A and acyltransferase 4	
A14G0204	58.99	37.52	HPRT1	Hypoxanthine-guanine phosphoribosyltransferase	NM_212986.2
B10G0593	58.98	10.67	DHRS13	reductase SDR family member 13	NM_001004641.1
A23G0886	58.97	136.35	ENO3	Beta-enolase	NM_214723.2
A10G0079	58.79	13.67	OCLN	Occludin	NM_212832.2
A22G0432	58.78	41.23	MICOS13	MICOS complex subunit MIC13	NM_001044873.1
A7G0544	58.73	25.32	PUF60B	Poly(U)-binding-splicing factor PUF60-B	NM_001430966.1
A2G0257	58.71	24.73	PHB2	Prohibitin-2	NM_199681.2
A14G0737	58.69	48.13	OGT	UDP-N-acetylglucosamine--peptide N-acetylglucosaminyltransferase 110 kDa subunit	NM_001018106.1
B24G0235	58.68	73.38	A0A673JSD5	A0A673JSD5_9TELE Chemerin	
B25G0158	58.62	95.05	IGLC7	Immunoglobulin lambda constant 7	XM_068217596.1
B25G0146	58.62	95.05	IGLC7	Immunoglobulin lambda constant 7	XM_068217596.1
B25G0139	58.62	95.05	IGLC7	Immunoglobulin lambda constant 7	XM_068217596.1
B23G0368	58.57	61.76	FLNA	Filamin-A	XM_021473622.2
B2G1134	58.56	17.56	EBNA1BP2	Probable rRNA-processing protein EBP2	NM_001003840.1
A20G0116	58.55	8.17	LOC107745905	A0A673FLS6_9TELE Cell wall protein RBR3-like	
B12G0096	58.53	27.44	MRPL38	39S ribosomal protein L38, mitochondrial	NM_212945.1
A3G0816	58.50	64.08	A0A672PBF1	A0A672PBF1_SINGR Si:ch211-76l23.4	XM_001334914.9
A16G0229	58.42	34.54	FBL	rRNA 2'-O-methyltransferase fibrillarin	NM_213002.1
A2G0344	58.39	21.84	GSN	Gelsolin	NM_213090.2
A4G0145	58.37	0.00	AEP1	Aerolysin-like protein	
B2G0843	58.36	50.60	RAB2A	Ras-related protein Rab-2A	NM_201454.2
B10G0024	58.35	29.00	PPP2CB	threonine-protein phosphatase 2A catalytic subunit beta isoform	NM_213293.1
B6G1037	58.23	4.47	SCEL	Sciellin	
A21G0143	58.17	42.78	UQCR10	Cytochrome b-c1 complex subunit 9	NM_001302478.1
B17G0857	58.16	55.15	COX7A2	Cytochrome c oxidase subunit 7A2, mitochondrial	NM_001032728.1
B16G0130	57.97	76.71	EIF3HA	Eukaryotic translation initiation factor 3 subunit H-A	NM_001003763.2
A15G0608	57.93	74.13	SRSF1A	arginine-rich splicing factor 1A	XM_005157415.5
A3G0772	57.93	67.53	CORO1CA	Coronin-1C-A	NM_201114.1
A25G0775	57.88	57.83	PSMA1	Proteasome subunit alpha type-1	NM_001003427.1
A20G0794	57.86	15.81	EZR	Ezrin	NM_001030285.2
A14G0328	57.85	51.27	HNRNPA0	Heterogeneous nuclear ribonucleoprotein A0	XM_068213169.1
B7G0816	57.77	18.87	CD82	CD82 antigen	NM_212661.2
A13G0345	57.77	56.36	CEFIP	Cardiac-enriched FHL2-interacting protein	NM_199820.2
B24G0271	57.73	43.69	CNN3	Calponin-3	NM_199753.1
B3G1174	57.60	40.07	RBX1	E3 ubiquitin-protein ligase RBX1	NM_001083544.1
B8G0689	57.53	27.25	NPL	N-acetylneuraminate lyase	

B10G0304	57.53	37.92	CLDN7B	Claudin-7-B	NM_131637.2
B2G0798	57.52	17.60	TRNAU1APL	tRNA selenocysteine 1-associated protein 1-like	NM_001029966.2
A23G0075	57.46	41.56	TCP1	T-complex protein 1 subunit alpha	NM_131230.1
B18G0619	57.41	7.13	PECR	Peroxisomal trans-2-enoyl-CoA reductase	NM_001099999.1
B11G0038	57.41	45.40	RAB43	Ras-related protein Rab-43	NM_201078.1
B12G0038	57.41	59.76	NDUFB8	NADH dehydrogenase [ubiquinone] 1 beta subcomplex subunit 8, mitochondrial	
A5G0324	57.36	75.44	MSN	Moesin	NM_001004296.1
B6G0348	57.34	1.73	GJC1	Gap junction gamma-1 protein	XM_021476749.2
B19G0857	57.20	36.90	SRSF4	arginine-rich splicing factor 4	NM_199574.1
B2G0848	57.16	37.45	SELENOF	Selenoprotein F	NM_178294.5
B22G0513	57.12	33.43	LOC107587000	A0A672NRX4_SINGR Uncharacterized LOC107587000	XM_001332531.9
A10G0034	57.10	15.06	PDLIM5	PDZ and LIM domain protein 5	NM_200196.1
B19G0250	57.09	23.78	MRPS21	28S ribosomal protein S21, mitochondrial	
A15G0019	57.06	80.14	HIS3.3A	Histone H3.3A	NM_001366916.1
A21G0556	56.95	132.56	CALM1	Calmodulin-1	NM_199570.1
B16G0610	56.93	31.72			XM_001340824.8
A3G0762	56.90	48.52	ELOB	Elongin-B	NM_001142954.1
A24G0423	56.87	44.63	NKTR	NK-tumor recognition protein	XM_001338036.7
B16G0696	56.84	14.33	S100B	Protein S100-B	NM_001326451.1
A22G0021	56.83	35.37	NECAP2	Adaptin ear-binding coat-associated protein 2	NM_200722.1
A25G0336	56.81	32.58	DUSP6	Dual specificity protein phosphatase 6	NM_194380.2
B7G0264	56.79	48.36	CDH1	Cadherin-1	XM_017357097.3
B11G0604	56.69	79.52	CYB561D1	Probable transmembrane reductase CYB561D1	NM_001018120.2
A2G0522	56.60	61.90	IFI30	Gamma-interferon-inducible lysosomal thiol reductase	NM_001006057.1
B11G0192	56.58	48.40	KLHL26	Kelch-like protein 26	NM_001328594.1
B13G0289	56.53	40.68	SI:CH211-233A24.2	Transmembrane protein KIAA1109 homolog	NM_199611.3
A21G0860	56.49	39.91	PPP2CA	threonine-protein phosphatase 2A catalytic subunit alpha isoform	NM_200911.3
A20G0100	56.43	55.01	NDUFB1	NADH dehydrogenase [ubiquinone] 1 beta subcomplex subunit 1	NM_001386380.1
B20G0697	56.30	19.71	CCDC25	Coiled-coil domain-containing protein 25	NM_200388.1
B3G1163	56.21	12.79	IL2RB	Interleukin-2 receptor subunit beta	
A14G0114	56.17	5.07	SLC25A48	Solute carrier family 25 member 48	NM_001002367.1
A5G1063	56.14	58.17	PPP2R1B	threonine-protein phosphatase 2A 65 kDa regulatory subunit A beta isoform	NM_001312917.1
B11G0406	56.12	42.00	REM1	GTP-binding protein REM 1	NM_201188.1
A13G0451	56.11	13.51	CALHM1	Calcium homeostasis modulator protein 1	NM_212791.2
A19G0443	56.10	49.79	LOC113062109	A0A6P6LVL9_CARAU lysosomal-associated transmembrane protein 5-like	
A5G0849	56.08	86.84	HAPLN1	Hyaluronan and proteoglycan link protein 1	NM_001313730.1

A11G0589	56.08	77.63			NM_212679.2
B9G0512	56.06	7.45	CAVIN2	Caveolae-associated protein 2	NM_001277070.1
A1G0485	56.04	55.22	PAIP2B	Polyadenylate-binding protein-interacting protein 2B	NM_200766.1
B2G0077	56.04	51.07	KLF2	Krueppel-like factor 2	NM_131857.2
B16G0228	55.99	85.31	MS4A4A	Membrane-spanning 4-domains subfamily A member 4A	NM_001199721.1
B19G0234	55.97	11.22	NUGGC	Nuclear GTPase SLIP-GC	
B15G0062	55.94	63.02	TAF15	TATA-binding protein-associated factor 2N	NM_001079973.1
B13G0348	55.93	39.88	FAM204A	Protein FAM204A	NM_178307.2
A4G0668	55.90	39.98	LUM	Lumican	NM_001002059.1
B19G0381	55.89	41.02	CBX3	Chromobox protein homolog 3	XM_005173769.4
A22G0034	55.86	45.29	CAPZB	F-actin-capping protein subunit beta	NM_199935.1
B10G0088	55.72	38.09	IER3IP1	Immediate early response 3-interacting protein 1	
B22G0047	55.71	69.10	LOC107698070	A0A671KE05_9TELE Ribonuclease inhibitor-like	XM_068216562.1
B22G0020	55.68	35.73	CAPZB	F-actin-capping protein subunit beta	NM_199935.1
B17G0513	55.62	25.95	ID3-A	DNA-binding protein inhibitor ID-3-A	NM_152967.1
A24G0206	55.60	31.97	A0A671NDD7	A0A671NDD7_9TELE Chemerin	NM_001386533.1
A1G0034	55.51	86.73	PIN1	Peptidyl-prolyl cis-trans isomerase NIMA-interacting 1	NM_200748.1
B9G0532	55.43	54.86	ARGLU1A	Arginine and glutamate-rich protein 1-A	NM_213216.2
B6G0991	55.34	6.16	TFAP2C	Transcription factor AP-2 gamma	NM_001008576.2
B20G0868	55.32	57.07	NDUFB1	NADH dehydrogenase [ubiquinone] 1 beta subcomplex subunit 1	NM_001386380.1
B13G0592	55.30	36.32	SERTAD4	SERTA domain-containing protein 4	XM_005156542.5
A20G0257	55.24	16.13	CCDC25	Coiled-coil domain-containing protein 25	NM_200388.1
B12G0062	55.20	31.79	ANXA11	Annexin A11	NM_181766.3
B16G0494	55.06	28.66	CCT3	T-complex protein 1 subunit gamma	NM_173250.1
A3G1067	55.05	95.72	DDX5	Probable ATP-dependent RNA helicase DDX5	NM_001309525.1
A21G0019	55.05	102.17	BHMT	Betaine--homocysteine S-methyltransferase 1	NM_001012480.2
B21G0943	55.03	114.45	TNFRSF14	Tumor necrosis factor receptor superfamily member 14	NM_001430990.1
B2G0735	54.91	21.21	U2SURP	U2 snRNP-associated SURP motif-containing protein	NM_001197074.1
B2G0736	54.87	49.58	TMEM59	Transmembrane protein 59	XM_005163400.5
B3G0533	54.76	53.76	HIS3.3A	Histone H3.3A	NM_212996.1
B2G1022	54.52	35.64	PHB2	Prohibitin-2	NM_199681.2
B9G0360	54.51	20.52	HSPE1	10 kDa heat shock protein, mitochondrial	NM_131526.2
A17G0025	54.40	26.69	DLST	Dihydrolipoyllysine-residue succinyltransferase component of 2-oxoglutarate dehydrogenase complex, mitochondrial	NM_201487.1
B25G0160	54.38	150.16	KV3A9	Ig kappa chain V-III region MOPC 63	XM_068217596.1
A1G0199	54.28	13.34	EXOSC1	Exosome complex component CSL4	NM_001145567.1

B20G0528	54.23	34.79	SOD2	Superoxide dismutase [Mn], mitochondrial	NM_199976.1
B14G0539	54.23	16.01	DDIT4L	DNA damage-inducible transcript 4-like protein	NM_001258317.1
B20G0662	54.21	35.37	ATF3	Cyclic AMP-dependent transcription factor ATF-3	NM_200964.1
B21G0281	54.21	39.20	POMP	Proteasome maturation protein	NM_001003424.2
B8G0189	54.17	39.86	TGOLN2	Trans-Golgi network integral membrane protein 2	
B6G0936	54.14	57.55	RBM5	RNA-binding protein 5	NM_001100138.3
B8G0204	54.13	37.56	EIF2S2	Eukaryotic translation initiation factor 2 subunit 2	
A11G0533	54.06	13.20	CAMKV	CaM kinase-like vesicle-associated protein	
A2G0236	54.05	154.86	SERP1	Stress-associated endoplasmic reticulum protein 1	NM_001002573.3
B9G0737	54.05	51.63	FSTL1	Follistatin-related protein 1	NM_001039621.1
B23G0947	53.92	3.13	ROHU_031861	A0A498LJA4_LABRO Macrophage mannose receptor 1-like protein	
B7G0850	53.84	62.12	HMGB3	High mobility group protein B3	NM_001122836.1
A13G0811	53.83	71.49	CSMD1	CUB and sushi domain-containing protein 1	NM_213133.1
A10G0808	53.80	11.35	CST3	Cystatin-C	
B24G0188	53.70	26.93	ARF1	ADP-ribosylation factor 1	NM_201480.1
A17G0403	53.68	72.03			NM_213351.1
A6G0319	53.64	37.62	PSMB3	Proteasome subunit beta type-3	NM_001129823.1
B13G0941	53.62	124.24			XR_011016480.1
B13G0944	53.62	124.24			XR_011016480.1
B13G0946	53.62	124.24			XR_011016480.1
A5G0204	53.61	34.55	H2AZ2	Histone H2A.V	NM_153644.1
B10G0193	53.59	17.03	FEL	Fish-egg lectin	NM_001109856.2
B12G0598	53.56	18.49	HTRA1B	Serine protease HTRA1B	NM_001111182.1
A6G0644	53.41	53.51	EIF4B	Eukaryotic translation initiation factor 4B	XM_005166014.5
A22G0326	53.40	31.66	FAM32AL	Protein FAM32A-like	NM_001002203.1
A7G0685	53.39	52.51	ANP32A	Acidic leucine-rich nuclear phosphoprotein 32 family member A	NM_199986.1
A5G0801	53.36	42.89	CXCL11.6	C-X-C motif chemokine 11-6	
B16G0513	53.33	79.76	CTSS	Cathepsin S	NM_001024409.2
A13G0311	53.33	17.99	DPX16_15969	A0A3N0YTW3_ANAGA Uncharacterized protein	NM_001013484.2
B23G0674	53.24	71.82	DAZAP2	DAZ-associated protein 2	NM_199793.2
A21G0482	53.23	13.67	ANAPC13	Anaphase-promoting complex subunit 13	NM_001302786.1
B8G0983	53.22	8.47	FUT9	4-galactosyl-N-acetylglucosaminide 3-alpha-L-fucosyltransferase 9	NM_001007454.2
A16G0615	53.17	39.96	FXYD6	FXYD domain-containing ion transport regulator 6	NM_001201417.1
B22G0624	53.11	13.85	RRP36	Ribosomal RNA processing protein 36 homolog	NM_001079944.1
A5G0079	53.09	66.47	SAT1	Diamine acetyltransferase 1	NM_001002169.2
B18G0322	53.09	53.54	SSR3	Translocon-associated protein subunit gamma	NM_200053.1
A13G0445	53.04	2.67	PKD2L1	Polycystic kidney disease 2-like 1 protein	XM_692149.10
A8G0871	53.02	63.08	LOC113039697	A0A6P6J0E3_CARAU small serum protein 2-like isoform X2	
B11G0801	52.97	10.67			

B3G0899	52.95	75.77	SNRNP70	U1 small nuclear ribonucleoprotein 70 kDa	NM_001003875.2
A17G0292	52.93	77.89	YWHAZ	delta	NM_201484.1
B10G0800	52.87	18.68	UBE2L3	Ubiquitin-conjugating enzyme E2 L3	NM_001002072.2
B11G0479	52.86	37.57	PTPDC1	Protein tyrosine phosphatase domain-containing protein 1	NM_200716.1
A1G0650	52.85	22.78	U2AF1	Splicing factor U2AF 35 kDa subunit	NM_177479.2
B23G0184	52.85	24.05	PTGES3	Prostaglandin E synthase 3	NM_001002137.2
B9G0011	52.79	110.67	LCP1	Plastin-2	NM_131320.3
A14G0471	52.78	24.61	CLTB	Clathrin light chain B	
B16G0581	52.68	5.50	ESRP1	Epithelial splicing regulatory protein 1	NM_001080576.1
B7G0910	52.64	41.59	MMP14	Matrix metalloproteinase-14	NM_194416.1
B9G0691	52.63	24.37	COA5	Cytochrome c oxidase assembly factor 5	NM_001161497.2
B5G0660	52.62	55.33	CTSL	Procathepsin L	XM_005165268.3
A16G0330	52.61	11.05	CD2AP	CD2-associated protein	NM_001037432.2
B15G0417	52.57	13.33	SPINT2	Kunitz-type protease inhibitor 2	
A16G0200	52.54	22.05	PSMA2	Proteasome subunit alpha type-2	NM_001024441.1
B16G0491	52.53	19.09	LMNA	Lamin-A	NM_152971.1
A8G0812	52.53	52.23	EIF2S2	Eukaryotic translation initiation factor 2 subunit 2	
A14G0743	52.52	2.76	ZNF185	Zinc finger protein 185	NM_001077536.1
A21G0138	52.46	105.17	CCNI	Cyclin-I	NM_213221.1
A3G0526	52.45	51.71	ALDOA	Fructose-bisphosphate aldolase A	NM_194377.2
B8G0618	52.36	80.14	CSDE1	Cold shock domain-containing protein E1	XM_068222882.1
A23G0689	52.31	101.31	HNRNPA1	Heterogeneous nuclear ribonucleoprotein A1	NM_200104.2
B8G0207	52.29	13.10	MAK16	Protein MAK16 homolog	NM_173239.2
A5G0380	52.28	16.46	EIF1AX	Eukaryotic translation initiation factor 1A, X-chromosomal	NM_001006082.2
A5G0614	52.26	28.42	PTGES	Prostaglandin E synthase	NM_001014828.2
B23G0687	52.25	4.74	IL17RD	Interleukin-17 receptor D	XM_683049.8
A2G0608	52.24	30.73	ELOC	Elongin-C	NM_001002440.2
A11G0002	52.21	31.34	IL1RAPL1B	Interleukin-1 receptor accessory protein-like 1-B	NM_001386341.1
B6G0032	52.12	28.39	CUTA	Protein CutA homolog	
B13G0086	52.09	81.04	A0A673JH75	A0A673JH75_9TELE Si:ch73-52p7.1	NM_213133.1
B3G0012	52.04	53.11	WBP2	WW domain-binding protein 2	
B8G0540	51.97	37.58	GSTM3	Glutathione S-transferase Mu 3	NM_001162851.1
A11G0601	51.90	19.11	ZGC:112255	Uncharacterized protein C1orf50 homolog	NM_205708.1
A10G0225	51.88	36.51	PCNA	Proliferating cell nuclear antigen	NM_131404.2
B12G0448	51.87	42.47	TEF	Thyrotroph embryonic factor	NM_001431066.1
B9G0359	51.83	35.58	MOB4	MOB-like protein phocein	NM_001003439.1
B18G0572	51.81	50.62	EIF4G2	Eukaryotic translation initiation factor 4 gamma 2	NM_001013443.2
B14G0756	51.78	60.62	OGT	UDP-N-acetylglucosamine--peptide N-acetylglucosaminyltransferase 110 kDa subunit	NM_001018106.1
A10G0047	51.77	27.49	SRSF9	arginine-rich splicing factor 9	NM_001312899.1
B13G0216	51.75	25.37	SPATA48	Spermatogenesis-associated protein 48	XM_068212865.1
A15G0005	51.68	10.48	A8CVD2	delta	
A8G0809	51.65	13.45	MAK16	Protein MAK16 homolog	NM_173239.2
A3G0586	51.65	7.04	PPL	Periplakin	NM_001137666.1

A16G0233	51.63	35.78	FLI1	Retroviral integration site protein Fli-1 homolog	NM_001008780.1
B17G0502	51.59	2.41	G5714_017064	A0A7J6C5I5_9TELE Uncharacterized protein	NM_001327925.1
B11G0222	51.50	37.14	ARRDC2	Arrestin domain-containing protein 2	XM_068224100.1
A2G0654	51.44	12.26	NOL7	Nucleolar protein 7	
A19G0348	51.41	43.40	PTP4A2	Protein tyrosine phosphatase type IVA 2	NM_001270540.1
B2G1119	51.41	71.62	GNG5	G(O) subunit gamma-5	NM_214721.2
B19G0062	51.41	0.21	LOC113062012	A0A6P6LS26_CARAU AAC-rich mRNA clone AAC11 protein-like	
A9G0250	51.36	12.45	G5714_009955	A0A7J6CT59_9TELE Ig-like domain-containing protein	
A15G0376	51.33	18.91	PITPNA	Phosphatidylinositol transfer protein alpha isoform	NM_200935.1
B6G0596	51.27	18.52	RGS4	Regulator of G-protein signaling 4	NM_199274.1
B17G0703	51.25	4.92	CAPN1	Calpain-1 catalytic subunit	NM_001089383.1
A8G0685	51.22	23.27	PRKAB1	5'-AMP-activated protein kinase subunit beta-1	NM_001328554.1
A23G0157	51.21	34.47	PTGES3	Prostaglandin E synthase 3	NM_001002137.2
A3G0559	51.15	42.25	ARF1	ADP-ribosylation factor 1	NM_201504.1
B2G1128	51.15	62.69	CZIB	CXXC motif containing zinc binding protein	NM_001128685.2
B24G0474	51.12	45.42	NKTR	NK-tumor recognition protein	XM_001338036.7
B8G0338	51.06	25.72	PRKAB1	5'-AMP-activated protein kinase subunit beta-1	NM_001328554.1
A11G0145	51.01	46.36	KLHL26	Kelch-like protein 26	NM_001033102.1
A3G0589	51.00	13.76	SMIM22	A0A673IES3_9TELE Small integral membrane protein 22	NM_001271773.2
A7G0408	51.00	32.07	GOT2A	Aspartate aminotransferase, mitochondrial	NM_199989.1
A17G0270	50.94	52.79	SNAP23	Synaptosomal-associated protein 23	XM_005158725.5
B25G0497	50.93	46.70	DUSP6	Dual specificity protein phosphatase 6	NM_194380.2
A3G0870	50.90	37.51	CNP	2',3'-cyclic-nucleotide 3'-phosphodiesterase	XM_068218823.1
B13G0791	50.84	7.66	GLO1	Lactoylglutathione lyase	NM_001007059.1
B20G0108	50.81	34.53	SNW1	SNW domain-containing protein 1	NM_001002864.1
A24G0235	50.80	26.93	F3	Tissue factor	NM_001245967.1
A8G0722	50.79	64.68			
A11G0062	50.77	30.64	INHBB	Inhibin beta B chain	NM_001037402.2
A19G0240	50.73	52.86	HDAC1-B	Probable histone deacetylase 1-B	XM_005159592.5
B9G0151	50.71	10.27	KRT18	Keratin, type I cytoskeletal 18	XM_009304971.3
A24G0001	50.66	66.04	SERPINB1	Leukocyte elastase inhibitor	
A10G0220	50.66	80.27	ANXA4	Annexin A4	NM_181764.3
B18G0076	50.53	67.36	ABCC5	ATP-binding cassette sub-family C member 5	XM_017351905.3
A10G0882	50.52	17.07	YD286	Glutaredoxin-like protein C5orf63 homolog	
B15G0222	50.51	8.22	TTMP	TPA-induced transmembrane protein	
B6G0611	50.50	6.67	TRAF3IP2	E3 ubiquitin ligase TRAF3IP2	XM_002662486.6
B16G0981	50.48	42.05	CTNNB1	Catenin beta-1	NM_131059.2
A18G0231	50.47	17.34	CD151	CD151 antigen	XM_009293634.4
B3G1067	50.47	41.86	LASP1	LIM and SH3 domain protein 1	NM_212636.1

B19G0215	50.46	22.80	MRPL24	Probable 39S ribosomal protein L24, mitochondrial	NM_001002401.1
B3G1081	50.38	38.52	PHB1	Prohibitin 1	NM_201297.1
A6G0142	50.35	27.22	PA2G4	Proliferation-associated protein 2G4	NM_001002070.2
B1G0852	50.32	25.81	DPY30	Protein dpy-30 homolog	NM_001002438.2
B16G0182	50.14	7.38	FXYD11	FXYD domain-containing ion transport regulator 11	NM_001282018.1
B12G0644	50.11	23.97	RAC1	Ras-related C3 botulinum toxin substrate 1	NM_199771.1
B10G0111	50.07	23.55	MAMDC2	MAM domain-containing protein 2	XM_017358019.3
A21G0636	50.06	16.51	STIM1	Stromal interaction molecule 1	XM_005161288.5
A3G1230	50.05	76.76	SOCS3	Suppressor of cytokine signaling 3	NM_199950.1
A11G0266	49.98	15.93	PRKCD	Protein kinase C delta type	NM_001077454.1
B1G1003	49.93	17.02	NDUFB7	NADH dehydrogenase [ubiquinone] 1 beta subcomplex subunit 7	
A18G0362	49.88	33.07	SEC11A	Signal peptidase complex catalytic subunit SEC11A	NM_001002521.2
B20G0877	49.87	36.89	RBM25	RNA-binding protein 25	XM_009295006.4
A15G0573	49.87	8.61	POU2AF3	POU Class 2 homeobox-associating factor 3	NM_001386216.1
A5G0525	49.79	10.44	TIMM8A	Mitochondrial import inner membrane translocase subunit Tim8 A	
A20G0310	49.77	21.78	RPL7L1	60S ribosomal protein L7-like 1	
B3G0093	49.76	0.48	A0A672LGG2	A0A672LGG2_SINGR AAA domain-containing protein	XM_068222460.1
A18G0370	49.71	52.89	CTSH	Pro-cathepsin H	NM_212688.1
A5G1349	49.67	46.74	STIP1	Stress-induced-phosphoprotein 1	NM_001007766.2
A14G0144	49.67	23.67	PIN4	Peptidyl-prolyl cis-trans isomerase NIMA-interacting 4	NM_001020553.1
B2G0858	49.60	37.44	SEPTIN2	Septin-2	NM_001301360.1
B21G0029	49.54	15.72	HPGD	15-hydroxyprostaglandin dehydrogenase [NAD(+)]	NM_200327.2
B21G0904	49.53	27.62	NARS	Asparagine--tRNA ligase, cytoplasmic	XM_691656.9
B25G0742	49.51	26.93	PSMA4	Proteasome subunit alpha type-4	NM_214697.1
A24G0156	49.47	19.55	ARF1	ADP-ribosylation factor 1	NM_201480.1
A22G0252	49.46	10.54	MID1IP1B	Mid1-interacting protein 1-B	NM_200832.2
B5G0256	49.45	17.33	SELENOM	Selenoprotein M	NM_178286.4
B2G1052	49.43	27.43	SELENOT1A	Thioredoxin reductase-like selenoprotein T1a	NM_178290.6
B24G0213	49.43	45.11	UBE2V2	Ubiquitin-conjugating enzyme E2 variant 2	NM_213515.1
A19G0101	49.41	55.80	SH3BGRL3	SH3 domain-binding glutamic acid-rich-like protein 3	NM_200278.2
A16G0292	49.39	62.34	EIF3EA	Eukaryotic translation initiation factor 3 subunit E-A	NM_200839.1
A3G0013	49.37	29.08	RTASE	Probable RNA-directed DNA polymerase from transposon BS	
A22G0004	49.33	26.28	RHOA	Transforming protein RhoA	NM_001045350.1
A4G0990	49.32	33.60	SLC38A2	Sodium-coupled neutral amino acid symporter 2	NM_001045104.1
B15G0104	49.32	51.46	EIF4H	Eukaryotic translation initiation factor 4H	NM_001197328.1
B9G0725	49.30	19.90	CBS	Cystathionine beta-synthase	NM_001014345.3
A2G0561	49.26	25.23	U2SURP	U2 snRNP-associated SURP motif-containing protein	NM_001197074.1

A5G1115	49.22	9.87	ANXA1	Annexin A1	NM_001017605.1
B11G0636	49.22	44.01	YOD1	Ubiquitin thioesterase OTU1	NM_001005938.1
A10G0168	49.21	38.67	ARRDC3	Arrestin domain-containing protein 3	NM_001004605.1
A22G0315	49.18	9.73	GPC1	Glypican-1	NM_001037373.2
B3G0022	49.10	39.24	NDUFA6	NADH dehydrogenase [ubiquinone] 1 alpha subcomplex subunit 6	NM_200968.1
B5G0022	49.09	14.34	ASS1	Argininosuccinate synthase	NM_001004603.1
A2G1101	49.09	28.10	DAD1	Dolichyl-diphosphooligosaccharide--protein glycosyltransferase subunit dad1	NM_001100439.1
B8G0387	49.05	21.81	EPGN	Epigen	
B2G0102	49.03	18.02	TWSG1A	Twisted gastrulation protein homolog 1-A	NM_131797.3
A3G0716	49.02	62.27	PRMT1	Protein arginine N-methyltransferase 1	NM_200650.2
B20G0294	48.96	41.83	TDH	L-threonine 3-dehydrogenase, mitochondrial	NM_213245.1
A1G0573	48.93	7.38	KLF5	Krueppel-like factor 5	XM_001344880.9
B19G0014	48.92	83.65	GSTA	Glutathione S-transferase A	
B8G0998	48.87	35.19	CTP	Dynein light chain 1, cytoplasmic	NM_213024.2
B19G0944	48.84	23.66			
A12G0676	48.80	20.50	REEP3	Receptor expression-enhancing protein 3	NM_200161.1
A3G0646	48.78	23.75	GNG5	G(O) subunit gamma-5	
A23G0057	48.73	10.88	G5714_023017	A0A7J6BPU2_9TELE Uncharacterized protein	NM_213168.3
B5G1021	48.73	39.87	VAMP5	Vesicle-associated membrane protein 5	
A22G0044	48.70	33.21	SRSF3	arginine-rich splicing factor 3	NM_001309473.1
A25G0234	48.69	12.17	OLFM4	Olfactomedin-4	XM_017354242.3
B9G0407	48.65	50.46	SAP18	Histone deacetylase complex subunit SAP18	NM_200720.2
B17G0169	48.63	54.85	PSMC1	26S proteasome regulatory subunit 4	NM_200033.1
A7G1034	48.62	48.18	LAMP1	Lysosome-associated membrane glycoprotein 1	XM_021471872.2
B3G1074	48.61	27.20	JPT2	Jupiter microtubule associated homolog 2	NM_199575.2
A2G0560	48.60	43.58	TMEM59	Transmembrane protein 59	XM_005163400.5
B3G0239	48.57	52.18	JUNB	Transcription factor JunB	NM_212750.1
B21G0004	48.53	28.53	SAR1B	GTP-binding protein SAR1b	NM_001024377.2
A15G0604	48.47	30.45	TXNDC17	Thioredoxin domain-containing protein 17	
B14G0428	48.43	5.03	FGFBP2	Fibroblast growth factor-binding protein 2	
B13G0100	48.42	57.76	ARFGEF3	Brefeldin A-inhibited guanine nucleotide-exchange protein 3	NR_155507.1
A13G0399	48.40	111.88	CNNM2	Metal transporter CNNM2	
A24G0516	48.39	30.13	PDCD6	Programmed cell death protein 6	NM_200950.1
B2G1205	48.38	25.32	ARF1	ADP-ribosylation factor 1	NM_201452.2
B3G1422	48.38	55.41	SOCS3	Suppressor of cytokine signaling 3	
A12G0324	48.34	27.61	ARL4D	ADP-ribosylation factor-like protein 4D	NM_199644.2
A3G0196	48.32	26.36	EP300	Histone acetyltransferase p300	NM_001365186.1
A3G0397	48.30	43.22	EIF3G	Eukaryotic translation initiation factor 3 subunit G	NM_200999.1

A23G0647	48.26	17.69	SARS1	Serine--tRNA ligase, cytoplasmic	NM_001003882.2
A8G0521	48.23	56.62	RHOAB	Rho-related GTP-binding protein RhoA-B	NM_212749.2
A4G0709	48.22	16.78	YAF2	YY1-associated factor 2	NM_001045268.1
B11G0175	48.14	0.97	SLC1A3	Excitatory amino acid transporter 1	
B13G0074	48.06	0.00	PROKR1	Prokineticin receptor 1	
A1G0622	48.06	8.52			
B19G0485	48.01	9.82	FAM83H	Protein FAM83H	NM_001365994.1
A17G0647	47.95	16.97	RTRAF	RNA transcription, translation and transport factor protein	NM_200270.2
B20G0858	47.94	40.60	NAA20	N-alpha-acetyltransferase 20	NM_001014329.2
A5G0342	47.92	36.75	LOC113091318	A0A6P6NV94_CARAU non-histone chromosomal protein HMG-14A-like	NM_001080033.2
B6G0648	47.91	35.81	EIF4B	Eukaryotic translation initiation factor 4B	XM_068221655.1
B25G0163	47.91	18.26	IGLC7	Immunoglobulin lambda constant 7	XM_068217596.1
B5G0711	47.84	61.27	VCP	Transitional endoplasmic reticulum ATPase	NM_201481.1
A25G0649	47.81	7.34	KV3A9	Ig kappa chain V-III region MOPC 63	XM_068217596.1
B16G0825	47.78	24.65	GRWD1	Glutamate-rich WD repeat-containing protein 1	NM_001003509.1
A10G0528	47.75	46.38	CCT8	T-complex protein 1 subunit theta	NM_201062.1
A8G0962	47.70	50.18	SELENOK	Selenoprotein K	NM_001004681.3
B25G0370	47.70	42.38	FNBP4	Formin-binding protein 4	NM_001305621.1
B3G0291	47.64	32.02	RNPS1	RNA-binding protein with serine-rich domain 1	NM_199761.2
B1G0065	47.63	51.38	CD48	CD48 antigen	
B13G0228	47.62	21.25	VDAC2	Voltage-dependent anion-selective channel protein 2	NM_001077315.1
B23G0130	47.59	32.54	RHOAC	Rho-related GTP-binding protein RhoA-C	NM_213350.1
A21G0619	47.55	9.59	NHP2	ACA ribonucleoprotein complex subunit 2-like protein	
A7G1213	47.49	20.46	PFDN2	Prefoldin subunit 2	NM_001045289.1
B23G0579	47.45	23.80	G5714_022550	A0A7J6BPC1_9TELE TAR DNA-binding protein 43	NM_001362353.1
B1G0443	47.44	22.12	ANP32B	Acidic leucine-rich nuclear phosphoprotein 32 family member B	NM_212603.2
A8G0558	47.34	23.77	CAPZA1	F-actin-capping protein subunit alpha-1	NM_001006663.1
B6G0242	47.33	55.64	SUMO3L	Small ubiquitin-related modifier 3-like	NM_213124.2
B21G0553	47.32	6.20	LIFR	Leukemia inhibitory factor receptor	XM_009295324.4
B13G0470	47.30	36.52	MCMBP	Mini-chromosome maintenance complex-binding protein	NM_001014303.1
B1G0968	47.25	44.11	PGAM1	Phosphoglycerate mutase 1	NM_201300.2
A24G0585	47.22	44.33	CAVIN1	Caveolae-associated protein 1	NM_001114549.2
B14G0651	47.20	13.28	ETF1	Eukaryotic peptide chain release factor subunit 1	NM_001111171.1
A7G0133	47.18	35.94	MLRN	Myosin regulatory light chain 2, smooth muscle minor isoform	NM_214699.2
A19G0560	47.17	42.99	TRA2A	Transformer-2 protein homolog alpha	NM_200416.1

A17G0429	47.16	23.87	SRRM1	arginine repetitive matrix protein 1	NM_001365281.1
A6G0929	47.15	59.04	GNB1	G(T) subunit beta-1	NM_213481.1
A7G0201	47.15	36.29	KARS1	Lysine--tRNA ligase	NM_001002386.2
A22G0017	47.14	16.46	MFAP2	Microfibrillar-associated protein 2	
A17G0551	47.13	13.00			NM_199985.2
A12G0782	47.12	13.23	VPS25	Vacuolar protein-sorting-associated protein 25	NM_001089362.2
A17G0064	47.11	44.10	COX7A2	Cytochrome c oxidase subunit 7A2, mitochondrial	
A8G0257	47.01	37.85	NDUFB11	NADH dehydrogenase [ubiquinone] 1 beta subcomplex subunit 11, mitochondrial	NM_001044981.1
B5G1080	47.00	77.90	ZFAND5	AN1-type zinc finger protein 5	NM_213039.1
A10G0456	47.00	30.54	HIST2H2L	Histone H2B 3	NM_200117.1
A6G0122	46.99	17.03	G5714_006664	A0A7J6CWZ4_9TELE Uncharacterized protein	
B1G0745	46.89	9.43	DPX16_21626	A0A3N0YJL8_ANAGA Uncharacterized protein	
A2G0198	46.87	41.78	EIF4A2	Eukaryotic initiation factor 4A-II	NM_213451.2
B5G1127	46.82	29.68	G5714_005984	A0A7J6D2J8_9TELE Uncharacterized protein	NM_001002300.1
A14G0008	46.82	4.24			
B11G0492	46.81	7.06	EPHB2	Ephrin type-B receptor 2	XM_003199503.6
B23G0455	46.79	21.16	DDOST	Dolichyl-diphosphooligosaccharide--protein glycosyltransferase 48 kDa subunit	NM_213093.1
A23G0642	46.77	36.08	SNRPG	Small nuclear ribonucleoprotein G	NM_001004660.3
A21G0453	46.76	123.54	C1QA	Complement C1q subcomponent subunit A	
A3G0760	46.73	84.67	ARL6IP1	ADP-ribosylation factor-like protein 6-interacting protein 1	NM_201112.1
B17G0766	46.68	64.98	NPC2	NPC intracellular cholesterol transporter 2	NM_001128719.1
B13G0700	46.67	33.03	CDKL1	Cyclin-dependent kinase-like 1	NM_181765.2
B12G0172	46.67	29.04	TIMM23	Mitochondrial import inner membrane translocase subunit Tim23	NM_001105598.1
A18G0290	46.66	48.50	SSR3	Translocon-associated protein subunit gamma	NM_200053.1
B2G0770	46.65	54.90	GYG1	Glycogenin-1	NM_213510.1
A12G0045	46.62	38.02	ANAPC16	Anaphase-promoting complex subunit 16	NM_001017878.2
B8G0992	46.61	37.76	SET	Protein SET	NM_201475.1
B9G0330	46.58	14.10	LADD	Ladderlectin	NM_001045141.3
A6G0902	46.55	19.97	EIF6	Eukaryotic translation initiation factor 6	NM_200944.1
B10G0504	46.50	58.03	MDH2	Malate dehydrogenase, mitochondrial	NM_213131.1
B21G0593	46.48	16.20	GZMK	Granzyme K	
B25G0676	46.41	32.75	PSMD13	26S proteasome non-ATPase regulatory subunit 13	NM_200948.1
B7G1155	46.40	29.05	FIS1	Mitochondrial fission 1 protein	NM_001310843.2
A3G1002	46.40	452.39	COL1A1	Collagen alpha-1(I) chain	NM_199214.1
A23G0817	46.37	24.10	ABCE1	ATP-binding cassette sub-family E member 1	NM_213051.2
A2G0087	46.34	103.48	SERBP1	Plasminogen activator inhibitor 1 RNA-binding protein	XM_001338162.9

A16G0392	46.32	19.51	RWDD1	RWD domain-containing protein 1	NM_001002535.2
B3G0918	46.28	8.92	ACTPB	DELTA-actitoxin-Afr1c	NM_001386552.1
B10G0446	46.17	79.67	CREBZF	ATF bZIP transcription factor	XM_009305487.4
B6G0515	46.14	118.37	ATNB233	potassium-transporting ATPase subunit beta-233	NM_131668.3
B9G0434	46.14	28.28	UCHL3	Ubiquitin carboxyl-terminal hydrolase isozyme L3	NM_001024405.1
B16G0957	46.08	7.96	GRHL2	Grainyhead-like protein 2 homolog	NM_001326533.1
A7G1188	46.05	21.06	SNRPA1	U2 small nuclear ribonucleoprotein A'	NM_001007383.2
A20G0293	45.98	25.86	ATF3	Cyclic AMP-dependent transcription factor ATF-3	NM_200964.1
B21G0906	45.98	24.59	TXNL1	Thioredoxin-like protein 1	NM_201138.2
B18G0814	45.97	95.94	PKM	Pyruvate kinase PKM	XM_009293364.3
B13G0105	45.93	27.55	CSMD1	CUB and sushi domain-containing protein 1	NM_213151.1
A21G0902	45.92	40.00	IRF1	Interferon regulatory factor 1	NM_205747.2
A8G0953	45.92	5.97	BICD2	Protein bicaudal D homolog 2	XM_009304324.4
B14G0731	45.91	95.11	DUSP1	Dual specificity protein phosphatase 1	NM_213067.2
A22G0033	45.87	16.71	NBL1	Neuroblastoma suppressor of tumorigenicity 1	NM_207097.1
A13G0429	45.86	29.49	BCL11A	leukemia 11A	NM_001007161.1
A17G0599	45.75	46.88	SLIRP	SRA stem-loop-interacting RNA-binding protein, mitochondrial	NM_001305612.1
B19G0524	45.71	64.57	ANP32E	Acidic leucine-rich nuclear phosphoprotein 32 family member E	NM_213277.1
A19G0917	45.70	55.85	MCL1	Induced myeloid leukemia cell differentiation protein Mcl-1	
B14G0708	45.69	34.19	SELENOT2	Selenoprotein T2	NM_001098487.2
B10G0589	45.68	32.53	RAB6A	Ras-related protein Rab-6A	NM_001328529.1
B9G0595	45.62	9.17	MID1IP1A	Mid1-interacting protein 1A	NM_131335.1
B16G0408	45.59	67.79	PNRC2	K7DY92_DANRE Proline-rich nuclear receptor coactivator 2	NM_001130192.1
A20G0664	45.57	25.20	PSMA7	Proteasome subunit alpha type-7	NM_213166.1
B21G0484	45.56	44.97	BIRC2	Baculoviral IAP repeat-containing protein 2	NM_194395.4
B19G0358	45.54	36.83	TXNL4A	Thioredoxin-like protein 4A	NM_001005953.1
B12G0190	45.54	44.00	TIMP2	Metalloproteinase inhibitor 2	NM_182874.1
A9G0580	45.54	48.06	CXCR4-B	C-X-C chemokine receptor type 4-B	NM_131834.1
B14G0376	45.54	16.54	GAR1	ACA ribonucleoprotein complex subunit 1	
A20G0655	45.50	41.12	EIF5	Eukaryotic translation initiation factor 5	NM_199591.2
B19G0296	45.50	295.12	G5714_018834	A0A7J6C031_9TELE Uncharacterized protein	
B4G0544	45.47	7.61	LLPH	Protein LLP homolog	NM_001103140.2
A1G0954	45.39	11.44			
A16G0728	45.34	183.95	CD209D	CD209 antigen-like protein D	NM_001326572.1
A21G0768	45.33	50.82	HMGB3	High mobility group protein B3	NM_001017769.1
B20G0565	45.32	54.02	ERH	Enhancer of rudimentary homolog	NM_001271817.1
A16G0648	45.26	34.77	KRTCAP2	Keratinocyte-associated protein 2	NM_001045362.1
A5G1283	45.23	19.67	MOB1A	MOB kinase activator 1A	NM_199914.1

B1G0429	45.22	31.99	NDUFB6	NADH dehydrogenase [ubiquinone] 1 beta subcomplex subunit 6	
B22G0003	45.20	26.05	RHOA	Transforming protein RhoA	NM_001045350.1
B2G1251	45.20	8.03	LAMA3	Laminin subunit alpha-3	XM_690939.9
A22G0321	45.18	36.48	SAFB2	Scaffold attachment factor B2	NM_214683.1
B19G0839	45.11	21.12	ZNF706	Zinc finger protein 706	NM_001198891.2
A13G0175	45.11	16.44	GPR4	G-protein coupled receptor 4	NM_001017593.2
A12G0407	45.10	41.07	CAPG	Macrophage-capping protein	NM_001001594.2
B3G0427	45.06	4.69	ZNF750	Zinc finger protein 750	XM_068216914.1
A9G0696	45.05	22.58	FSTL1	Follistatin-related protein 1	NM_001039621.1
B24G0550	45.04	31.18	PABPN1-B	Polyadenylate-binding protein 2-B	NM_213259.2
B3G0664	45.00	27.14	GSPT1	Eukaryotic peptide chain release factor GTP-binding subunit ERF3A	NM_198806.1
A20G0649	44.99	14.03	TDH	L-threonine 3-dehydrogenase, mitochondrial	NM_213245.1
A13G0299	44.97	28.34	MTRES1	Mitochondrial transcription rescue factor 1	NM_001020574.2
B6G0393	44.97	50.28	P4HB	Protein disulfide-isomerase	NM_213364.3
A2G0808	44.94	8.56	GADD45B	Growth arrest and DNA damage-inducible protein GADD45 beta	NM_001012386.2
B8G0275	44.91	26.13	RBM39	RNA-binding protein 39	NM_001014370.1
A8G0925	44.90	28.67	NDUFA7	NADH dehydrogenase [ubiquinone] 1 alpha subcomplex subunit 7	
A21G0054	44.86	120.90	CR032	UPF0729 protein C18orf32 homolog	
A13G0466	44.84	37.66	SLK	threonine-protein kinase	XM_068212823.1
B6G0036	44.82	28.26	GRAP2	GRB2-related adapter protein 2	
A6G0292	44.79	37.62	GCA	Grancalcin	NM_001005585.1
A14G0251	44.72	31.25	NKAPL	NKAP-like protein	NM_001003414.1
A18G0373	44.70	131.27	IDH2	Isocitrate dehydrogenase [NADP], mitochondrial	NM_199564.1
B17G0296	44.69	27.23	SLIRP	SRA stem-loop-interacting RNA-binding protein, mitochondrial	NM_001305612.1
B16G0430	44.67	15.33	RWDD1	RWD domain-containing protein 1	NM_001002535.2
B1G0553	44.65	21.92	KCTD12	POZ domain-containing protein KCTD12	NM_001034019.2
A21G0107	44.65	4.04	THBS4B	Thrombospondin-4-B	NM_173226.1
A20G0243	44.64	77.72	FABP7	Fatty acid-binding protein, brain	NM_214807.1
A1G0232	44.59	39.83	CCT4	T-complex protein 1 subunit delta	NM_200583.1
A18G0134	44.57	68.58	RNF7	RING-box protein 2	NM_001012498.2
B23G0305	44.57	85.07	PPDPFB	Pancreatic progenitor cell differentiation and proliferation factor B	NM_200008.1
A9G0864	44.56	50.47	PCBP2	Poly(rC)-binding protein 2	NM_201192.1
A18G0443	44.53	7.69	BCL2L13	Bcl-2-like protein 13	NM_001044891.2
B10G0709	44.52	16.61	TMEM167A	Protein kish-A	NM_001023581.2
B3G0826	44.50	24.57	CD2BP2	CD2 antigen cytoplasmic tail-binding protein 2	NM_200961.1
B24G0456	44.49	17.93	NAA50	N-alpha-acetyltransferase 50	XM_005162691.5
A2G0861	44.46	19.78	MMP14	Matrix metalloproteinase-14	NM_194414.1
B5G0079	44.41	44.64	PRDX4	Peroxiredoxin-4	NM_001089425.1
B5G0707	44.41	22.23	RIOK2	threonine-protein kinase RIO2	NM_213554.2
A11G0186	44.38	56.43	RBBP7	Histone-binding protein RBBP7	NM_213031.3

B16G0216	44.35	10.66	MRPL32	39S ribosomal protein L32, mitochondrial	
A7G0446	44.31	18.13	APIP	Methylthioribulose-1-phosphate dehydratase	NM_001004679.1
A20G0527	44.23	8.51	GTF2H5	General transcription factor IIH subunit 5	NM_001134362.2
A21G0878	44.20	22.70	LOC113073439	A0A6P6N0L9_CARAU HMG domain-containing protein 3-like isoform X2	
B1G0308	44.16	16.90	CRIP1	Cysteine-rich protein 1	NM_001161335.2
A8G0472	44.11	38.46	MMP9	Matrix metalloproteinase-9	NM_213123.1
A2G0142	44.05	11.98	IMP3	U3 small nucleolar ribonucleoprotein protein IMP3	NM_001291357.1
B6G0513	44.04	62.31	DPT	Dermatopontin	NM_001030085.3
A16G0119	43.95	28.28	SEPTIN7	Septin-7	XM_068213826.1
A19G0341	43.95	17.06	ZNF706	Zinc finger protein 706	NM_001003731.2
B18G0357	43.94	78.87	FOSB	Protein FosB	XM_005173641.5
A23G0117	43.91	25.21	TAF11	Transcription initiation factor TFIID subunit 11	XM_068216727.1
B14G0682	43.91	19.06	CTBP1	C-terminal-binding protein 1	NM_001040390.1
A16G0937	43.89	53.63	NDUFB9	NADH dehydrogenase [ubiquinone] 1 beta subcomplex subunit 9	NM_001017880.1
A7G0436	43.88	30.60	MFAP1A	Microfibrillar-associated protein 1A	NM_201017.1
A18G0665	43.84	3.49	CERS2	Ceramide synthase 2	XM_005158997.5
B23G0727	43.83	19.16	COPZ1	Coatomer subunit zeta-1	NM_131508.1
B19G0260	43.81	43.22	NDUFV1	NADH dehydrogenase [ubiquinone] flavoprotein 1, mitochondrial	NM_001003747.1
A19G0262	43.81	19.73	RBBP4	Histone-binding protein RBBP4	NM_212595.1
A5G0078	43.78	44.70	PRDX4	Peroxiredoxin-4	NM_001089425.1
A14G0011	43.78	7.78	LYAR	Cell growth-regulating nucleolar protein	NM_200679.3
B17G0638	43.75	31.52	EIF2S1	Eukaryotic translation initiation factor 2 subunit 1	NM_199569.1
B21G0638	43.72	33.56	VPS29	Vacuolar protein sorting-associated protein 29	NM_200037.2
A20G0878	43.71	55.33	LYRM2	LYR motif-containing protein 2	NM_001159831.2
A25G0236	43.66	3.13	OLFM4	Olfactomedin-4	XM_017354130.3
B12G0652	43.65	17.10	REEP3	Receptor expression-enhancing protein 3	NM_200161.1
B8G0968	43.64	3.07	SRRD	SRR1-like protein	NM_001045427.1
A5G0384	43.63	177.54	GABARAP	Gamma-aminobutyric acid receptor-associated protein	NM_001013260.1
A6G0108	43.58	92.51	RTN4	Reticulon-4	NM_001079912.2
B7G0904	43.55	28.04	NEDD8	NEDD8	NM_201321.2
A3G0378	43.55	92.57	PET100	Protein PET100 homolog, mitochondrial	NM_001303057.1
B5G0522	43.50	66.46	EDF1	Endothelial differentiation-related factor 1 homolog	NM_200745.1
B22G0245	43.50	384.34	LOC113079894	A0A6P6NIG4_CARAU uncharacterized protein LOC113079894	XM_068216604.1
A18G0097	43.44	27.97	ARFGAP2	ADP-ribosylation factor GTPase-activating protein 2	XM_005159248.4
A4G0149	43.41	31.73	LOC113067422	A0A6P6MHX5_CARAU uncharacterized protein LOC113067422 isoform X2	
A9G0447	43.40	6.55	CAVIN2	Caveolae-associated protein 2	NM_001277070.1
B7G0339	43.37	33.49	PAIP2B	Polyadenylate-binding protein-interacting protein 2B	NM_200766.1

A5G0772	43.36	21.09	SUB1	Activated RNA polymerase II transcriptional coactivator p15	NM_001002505.1
A22G0375	43.32	42.84	HNRNPM	Heterogeneous nuclear ribonucleoprotein M	NM_001256631.1
A16G0295	43.31	7.81			
B15G0026	43.24	52.92	LXN	Latexin	XM_021481398.2
B21G0088	43.24	21.36	DKC1	ACA ribonucleoprotein complex subunit DKC1	NM_001033107.2
B20G0288	43.22	47.12	EIF5	Eukaryotic translation initiation factor 5	NM_199591.2
B11G0144	43.21	27.51	GLT8D1	Glycosyltransferase 8 domain-containing protein 1	NM_200696.1
A22G0694	43.20	13.82	DNAJC19	Mitochondrial import inner membrane translocase subunit TIM14	NM_200761.1
B4G0386	43.17	21.73	AKR1B1	Aldo-keto reductase family 1 member B1	NM_001077247.1
A10G0141	43.17	25.02	H2AZ1	Histone H2A.Z	NM_153644.1
B7G0685	43.17	71.60	CD81	CD81 protein	NM_131518.2
B4G0512	43.14	27.38	FAM3C	Protein FAM3C	NM_212725.1
B15G0226	43.14	23.89	NDUFC2	NADH dehydrogenase [ubiquinone] 1 subunit C2	
A16G0098	43.12	72.35	NR1D2	Nuclear receptor subfamily 1 group D member 2	NM_001130592.2
A25G0021	43.08	3.19	ADAMTS20	A disintegrin and metalloproteinase with thrombospondin motifs 20	XM_017354517.1
A22G0565	43.06	4.24	CAPN2	Calpain-2 catalytic subunit	NM_001003485.2
B10G0375	43.03	52.11	CCT8	T-complex protein 1 subunit theta	XM_005155366.4
B18G0598	43.01	23.88	ENDOUC	Uridylate-specific endoribonuclease C	NM_001044974.1
A3G0101	43.00	6.22	GIMAP8	GTPase IMAP family member 8	XM_068217815.1
B1G0129	42.96	1.10	SMC5	Structural maintenance of chromosomes protein 5	
B10G0773	42.90	10.05	ELOVL7	Elongation of very long chain fatty acids protein 7	NM_199778.1
A5G0741	42.89	16.78	CXCL13	C-X-C motif chemokine 13	
B2G1257	42.89	2.18	FOXQ1	Forkhead box protein Q1	NM_001243344.1
B19G0282	42.86	4.28	TMEM238	Transmembrane protein 238	
B3G0223	42.85	30.36	DDX39A	ATP-dependent RNA helicase DDX39A	NM_212977.2
B12G0688	42.82	5.78	DLX3B	Homeobox protein Dlx3b	NM_131322.2
A22G0143	42.79	14.16	SI:DKEY-222F2.8	Cytochrome c oxidase assembly protein COX14 homolog	
A4G0013	42.77	4.71	A0A673J997	A0A673J997_9TELE Uncharacterized protein	
B7G1195	42.76	49.58	CNFN	Cornifelin homolog	NM_001327905.1
B16G0903	42.75	6.11	HOXA13B	Homeobox protein Hox-A13b	NM_131194.2
A18G0141	42.74	32.97	CCNL1	Cyclin-L1	NM_001089363.1
B13G0116	42.74	32.51	PDE10A	cAMP and cAMP-inhibited cGMP 3',5'-cyclic phosphodiesterase 10A	NM_001077581.1
B21G0961	42.74	6.05	RAD23B	UV excision repair protein RAD23 homolog B	XR_011007963.1
B8G0825	42.70	30.92	ITGB4	Integrin beta-4	XM_005166790.5
B1G1171	42.69	50.28	AKAP8L	A-kinase anchor protein 8-like	NM_213219.2
B8G0571	42.69	13.59	MAPK14A	Mitogen-activated protein kinase 14A	XM_005167061.5
B6G0883	42.62	34.68	ATP5F1EP2	ATP synthase subunit epsilon-like protein, mitochondrial	

A3G1127	42.58	36.67	IGLL1	Immunoglobulin lambda-like polypeptide 1	XM_021474765.2
A3G1173	42.56	16.43	RAB37	Ras-related protein Rab-37	NM_001017715.1
A3G0683	42.54	39.33	CD2BP2	CD2 antigen cytoplasmic tail-binding protein 2	NM_200961.1
B15G0192	42.54	33.72	NOP53	Ribosome biogenesis protein NOP53	NM_001079946.2
B18G0691	42.54	51.52	ARF4	ADP-ribosylation factor 4	NM_199275.1
A24G0129	42.45	25.78	H1-0	Histone H1.0	NM_199552.1
B5G0503	42.43	42.08	RAB14	Ras-related protein Rab-14	NM_201495.1
B17G0606	42.43	5.20	GRHL1	Grainyhead-like protein 1 homolog	XM_001923728.6
A7G0584	42.38	14.19	MDK-A	Midkine-A	NM_131070.2
A21G0452	42.36	135.19	C1QC	Complement C1q subcomponent subunit C	NM_001005976.2
B5G1299	42.36	61.75	XBP1	X-box-binding protein 1	NM_131874.1
A3G0734	42.36	31.83	SNRNP70	U1 small nuclear ribonucleoprotein 70 kDa	NM_001003875.2
B9G0048	42.34	33.19	A0A673FPH7	A0A673FPH7_9TELE NLRC4_HD2 domain-containing protein	XM_021468229.2
B12G0433	42.33	13.84	XRCC6	X-ray repair cross-complementing protein 6	NM_199904.2
B18G0711	42.33	14.33	CD9	CD9 antigen	XM_005158981.5
B7G0726	42.30	47.13	SLTM	SAFB-like transcription modulator	NM_213333.2
B19G0227	42.26	11.26	MYZAP	Myocardial zonula adherens protein	NM_001082986.1
B24G0593	42.26	25.85	CDV3	Protein CDV3 homolog	NM_212721.1
B3G0925	42.25	70.90	ARL6IP1	ADP-ribosylation factor-like protein 6-interacting protein 1	NM_201112.1
A4G0872	42.23	6.42	UTP20	Small subunit processome component 20 homolog	XM_009300013.4
B11G0539	42.20	25.64			NM_001002097.1
A18G0174	42.19	87.76	NR13	Anti-apoptotic protein NR13	NM_194398.3
B9G0361	42.17	26.00	HSPD1	60 kDa heat shock protein, mitochondrial	NM_181330.3
B22G0635	42.12	34.35	SAFB2	Scaffold attachment factor B2	NM_214683.1
B16G0514	42.12	30.28	CTSK	Cathepsin K	NM_001017778.1
B20G0152	42.07	26.73	ARHGAP20	Rho GTPase-activating protein 20	NM_001365334.1
B14G0818	42.02	14.09	SH2D1A	SH2 domain-containing protein 1A	
B20G0549	41.99	34.85	SNX3	Sorting nexin-3	NM_001037106.2
B23G0173	41.98	18.24	CSRP1	Cysteine and glycine-rich protein 1	NM_205567.1
B18G0626	41.98	23.71	HSBP1	Heat shock factor-binding protein 1	NM_200754.2
B6G0901	41.97	18.52	BLCAP	Bladder cancer-associated protein	NM_131493.2
A12G0098	41.95	22.63	BCCIP	Protein BCCIP homolog	NM_001013475.2
A9G0163	41.93	7.25	GULP1	PTB domain-containing engulfment adapter protein 1	XM_068223305.1
A3G1148	41.93	39.72	I3	Brain protein I3	NM_001200039.1
A9G0531	41.93	89.86	TXNDC9	Thioredoxin domain-containing protein 9	
A13G0160	41.89	12.54	SFRP5	Secreted frizzled-related protein 5	
A9G0603	41.87	32.25	SUMO3L	Small ubiquitin-related modifier 3-like	NM_001002677.2
B13G0566	41.87	21.75	G5714_013544	A0A7J6CJH3_9TELE Palmitoyltransferase	NM_001013460.3

B1G1098	41.87	84.38	GGACT.2	Gamma-glutamylaminecyclotransferase B	
B11G0536	41.87	41.48	TTBK1	Tau-tubulin kinase 1	NM_212679.2
B19G0335	41.85	26.25	IGKC	Immunoglobulin kappa constant	
A23G0048	41.85	31.30	SRSF6	arginine-rich splicing factor 6	XM_005161922.5
B19G0331	41.85	26.25	IGKC	Immunoglobulin kappa constant	
A5G0983	41.80	8.46	GSN	Gelsolin	NM_001089344.1
B3G1045	41.75	21.26	STK17A	threonine-protein kinase 17A	XM_017354592.3
B3G1299	41.73	23.27	KDELR2	ER lumen protein-retaining receptor 2	NM_200103.1
A14G0556	41.71	9.75	TRMT112	Multifunctional methyltransferase subunit TRM112-like protein	NM_001020752.1
B5G0982	41.64	20.91	A0A671S9V8	A0A671S9V8_9TELE Thy-1 cell surface antigen	
A12G0607	41.63	21.44	PSME2	Proteasome activator complex subunit 2	NM_131374.1
B19G0587	41.62	21.62	ILF2	Interleukin enhancer-binding factor 2 homolog	NM_213236.2
A2G0795	41.60	18.29	PAK2	threonine-protein kinase PAK 2	NM_001002717.1
B20G0429	41.59	1.80	FOXQ1	Forkhead box protein Q1	NM_212907.2
B13G0612	41.53	52.22	VTI1B	Vesicle transport through interaction with t-SNAREs homolog 1B	NM_001001946.4
A2G0959	41.51	44.72	ITGB1-A	Integrin beta-1-A	NM_001034987.1
B11G0632	41.50	15.10	HP1BP3	Heterochromatin protein 1-binding protein 3	NM_001006030.1
B6G0643	41.49	49.38	BIN2	Bridging integrator 2	NM_199796.1
B14G0452	41.49	15.92	MIF	Macrophage migration inhibitory factor	NM_001043321.1
B8G1058	41.46	15.16	CDO1	Cysteine dioxygenase type 1	NM_200741.1
A15G0590	41.46	21.74	MRPL28	39S ribosomal protein L28, mitochondrial	NM_001024399.1
B12G0060	41.42	12.56	PPIF	Peptidyl-prolyl cis-trans isomerase F, mitochondrial	NM_001328424.1
B6G0861	41.33	12.76	SLC25A22	Mitochondrial glutamate carrier 1	XM_017356461.2
B24G0160	41.31	41.50	H1-0	Histone H1.0	NM_199552.1
B3G1037	41.30	31.24	RAB5C	Ras-related protein Rab-5C	NM_201501.1
A25G0154	41.29	60.75	MDK-A	Midkine-A	NM_131716.1
A18G0539	41.29	53.09	CTSD	Cathepsin D	NM_131710.2
A22G0638	41.26	24.38	LSM3	U6 snRNA-associated Sm-like protein LSm3	NM_001386830.1
A10G0329	41.26	30.34	UBL3	Ubiquitin-like protein 3	NM_212686.2
B5G1038	41.24	49.47	CYB561A3A	Lysosomal membrane ascorbate-dependent ferrireductase CYB561A3	XM_068221038.1
A8G0952	41.14	85.16	GNB1	G(T) subunit beta-1	NM_212609.1
B15G0750	41.07	36.89	APLP2	Amyloid beta precursor like protein 2	XM_001342885.9
B13G0481	41.04	36.08	REL	Proto-oncogene c-Rel	NM_001007161.1
B5G0475	41.03	68.61	HNRNPUL1	Heterogeneous nuclear ribonucleoprotein U-like protein 1	XM_003198712.6
B18G0122	41.03	65.03	TC1A	Transposable element Tc1 transposase	
A1G0145	41.03	2.94	C3	Complement C3 (Fragment)	XM_009295246.3
B18G0024	41.03	65.03	TC1A	Transposable element Tc1 transposase	
A24G0015	41.03	29.43	VAPA	Vesicle-associated membrane protein-associated protein A	NM_199918.2

A24G0408	41.01	18.41	NAA50	N-alpha-acetyltransferase 50	NM_001003623.1
B23G0905	41.00	51.30	CCN1	CCN family member 1	
B10G0157	40.98	21.95	ANXA3	Annexin A3	NM_001007302.2
A25G0038	40.95	17.68	SCAMP2	Secretory carrier-associated membrane protein 2	NM_213378.2
A16G0240	40.89	27.08	PSMC4	26S proteasome regulatory subunit 6B	NM_199750.2
A20G0306	40.88	10.26	ABRACL	Costars family protein ABRACL	
A21G0733	40.88	57.12	PGK1	Phosphoglycerate kinase 1	NM_213387.1
B8G0758	40.84	18.20	SMIM15	Small integral membrane protein 15	NM_001159946.2
B1G0431	40.83	18.40	MTAP	S-methyl-5'-thioadenosine phosphorylase	NM_200554.1
A5G1277	40.80	9.73	GIMAP5	GTPase IMAP family member 5	XM_021471486.2
B15G0929	40.79	17.12	G5714_015513	threonine protein kinase	NM_200541.1
B16G0244	40.73	40.63	HERPUD2	Homocysteine-responsive endoplasmic reticulum-resident ubiquitin-like domain member 2 protein	NM_200188.1
A21G0040	40.73	16.57	TXNL1	Thioredoxin-like protein 1	NM_201138.2
B3G0601	40.65	27.06	BUD31	Protein BUD31 homolog	NM_001003860.1
A2G0720	40.63	41.80	DPX16_18227	A0A3N0YE48_ANAGA Uncharacterized protein	
A5G0910	40.62	46.84	SCARB2	Lysosome membrane protein 2	NM_173259.1
B22G1116	40.62	13.83	RBBP9	Serine hydrolase RBBP9	NM_001042748.1
B24G0478	40.61	23.03	GARS1	Glycine--tRNA ligase	NM_001386453.1
A4G0866	40.58	26.56	IFRD1	Interferon-related developmental regulator 1	NM_001076555.1
B3G0554	40.58	32.43	LITAF	Lipopolysaccharide-induced tumor necrosis factor-alpha factor homolog	
B19G0824	40.56	25.17	CHTOP	Chromatin target of PRMT1 protein	NM_199546.1
A17G0224	40.53	20.05	ZFP36L1	mRNA decay activator protein ZFP36L1	NM_001077153.2
A8G0640	40.50	8.16	EPGN	Epigen	NM_001386684.1
B19G0721	40.47	38.29	NDUFS6	NADH dehydrogenase [ubiquinone] iron-sulfur protein 6, mitochondrial	NM_001004651.1
A20G0033	40.35	34.63	PRPF39	Pre-mRNA-processing factor 39	NM_001004520.1
A4G0829	40.33	42.38	NFYB	Nuclear transcription factor Y subunit beta	NM_001017565.1
B2G0572	40.33	18.37	MRPS14	28S ribosomal protein S14, mitochondrial	NM_001045358.1
A5G1300	40.31	55.23	PSAP	Prosaposin	
A6G0890	40.26	18.85	BLCAP	Bladder cancer-associated protein	NM_131493.2
A6G0380	40.25	10.52	NOL11	Nucleolar protein 11-like	NM_001030063.1
B7G0496	40.25	78.30	DNAJA2	DnaJ homolog subfamily A member 2	NM_213493.1
B18G0747	40.22	11.22	NIP7	60S ribosome subunit biogenesis protein NIP7 homolog	NM_001020590.1
B6G0278	40.22	25.58	TMBIM1	Protein lifeguard 3	NM_001005992.2
B5G0718	40.17	28.56	SUB1	Activated RNA polymerase II transcriptional coactivator p15	NM_001002505.1
A23G0261	40.16	40.77	SDCBP	Syntenin-1	NM_212691.2
B5G1074	40.07	19.98	KLF9	Krueppel-like factor 9	
B1G1095	39.99	34.11	GGACT.3	Gamma-glutamylaminecyclotransferase C	
B23G0819	39.96	22.74	PFDN4	Prefoldin subunit 4	NM_001386509.1

B8G0623	39.95	62.48	CD40	Tumor necrosis factor receptor superfamily member 5	
B24G0269	39.95	37.11	F3	Tissue factor	
B16G0134	39.94	35.58	SEPTIN7	Septin-7	XM_068213827.1
B2G0882	39.94	50.76	RNF2	E3 ubiquitin-protein ligase RING2	NM_131213.2
A2G0972	39.93	45.47	SDHC	Succinate dehydrogenase cytochrome b560 subunit, mitochondrial	NM_001003523.2
A18G0802	39.90	55.82	NMRK2	Nicotinamide riboside kinase 2	NM_198877.1
A8G0819	39.88	53.35	TBB1	Tubulin beta-1 chain	NM_001037410.2
A5G1109	39.88	21.52	HNRNPK	Heterogeneous nuclear ribonucleoprotein K	NM_212991.3
B8G0864	39.86	22.00	BCAS2	Pre-mRNA-splicing factor SPF27	NM_001007774.1
A20G0839	39.85	7.15	PLEKHG3	Pleckstrin homology domain-containing family G member 3	XM_017352680.3
B7G0330	39.83	14.86	BOLA2	BolA-like protein 2	
B1G1090	39.83	49.28	GGACT.3	Gamma-glutamylaminecyclotransferase C	
A23G0103	39.82	33.29	RHOAC	Rho-related GTP-binding protein RhoA-C	NM_213350.1
B12G0632	39.78	35.85	PSMC5	26S proteasome regulatory subunit 8	NM_001003740.1
B24G0421	39.77	34.11	DAP	Death-associated protein 1	NM_131572.2
A1G0265	39.76	4.42	CCDC86	Coiled-coil domain-containing protein 86	NM_001145359.1
A9G0023	39.74	11.56	SEPTIN10	Septin-10	NM_001017557.1
A19G0678	39.74	34.16	CLK2	Dual specificity protein kinase CLK2	NM_001044879.2
B13G0562	39.73	37.03	G5714_013541	A0A7J6CEX8_9TELE DUF4585 domain-containing protein	XM_005156611.3
B7G0269	39.72	14.20	CAMK2D1	calmodulin-dependent protein kinase type II delta 1 chain	XM_021477468.2
B9G0260	39.71	20.23	HACD2	Very-long-chain (3R)-3-hydroxyacyl-CoA dehydratase 2	NM_199861.2
A9G0329	39.68	26.60	MOB4	MOB-like protein phocein	NM_001003439.1
A16G0659	39.66	10.46	S100A16	Protein S100-A16	NM_001326482.1
A17G0091	39.66	22.65	GNMT	Glycine N-methyltransferase	NM_212816.1
A17G0671	39.63	48.19	NFKBIA	NF-kappa-B inhibitor alpha	NM_199629.1
B8G0966	39.63	1.30	DHX37	Probable ATP-dependent RNA helicase DHX37	
A7G0718	39.62	13.45	EIF3JB	Eukaryotic translation initiation factor 3 subunit J-B	NM_213502.1
A24G0179	39.61	28.94	UBE2V2	Ubiquitin-conjugating enzyme E2 variant 2	NM_213515.1
A11G0593	39.61	74.03	KLF15	Krueppel-like factor 15	NM_001002097.1
A1G0454	39.60	4.16			XM_068221592.1
B19G0280	39.58	23.82	U2AF2	Splicing factor U2AF 65 kDa subunit	NM_205689.1
A19G0275	39.58	39.86	C6ORF62	Uncharacterized protein C6orf62	NM_001020637.1
A3G0796	39.53	20.05	GRN	Progranulin	NM_001001949.3
B14G0760	39.53	3.33	ZNF185	Zinc finger protein 185	NM_001077536.1
A6G0232	39.52	5.19	PTTG1IP	Pituitary tumor-transforming gene 1 protein-interacting protein	NM_001326362.1
B11G0044	39.50	37.48	ACOT8	Acyl-coenzyme A thioesterase 8	NM_001002698.1
B13G0466	39.49	30.10	TIAL1	Nucleolysin TIAR	NM_001145601.1
A10G0589	39.49	22.29	CLDN7B	Claudin-7-B	NM_131637.2

B2G0565	39.49	8.02	LAMC2	Laminin subunit gamma-2	XM_003197884.6
B21G0060	39.46	57.04	SKP1	S-phase kinase-associated protein 1	NM_200743.1
B4G0069	39.45	12.89	UBE2N	Ubiquitin-conjugating enzyme E2 N	NM_200342.1
A7G0036	39.45	46.84	PPP1CB	threonine-protein phosphatase PP1-beta catalytic subunit	XM_695376.8
A7G1384	39.38	96.41	EPD	Ependymin	NM_001293702.1
B16G1123	39.33	17.80	PLEC	Plectin	XM_009292039.4
A10G0579	39.29	109.59	KDM6B	Lysine-specific demethylase 6B	NM_001030178.2
A3G0909	39.29	37.53	ATP6V0C	V-type proton ATPase 16 kDa proteolipid subunit c	NM_001105136.2
A8G0073	39.28	57.25	VDAC2	Voltage-dependent anion-selective channel protein 2	XM_005166746.4
A19G0819	39.26	28.75	PSMB6L-B	Proteasome subunit beta type-6-B like protein	NM_131676.2
A7G0823	39.26	21.53	CD82	CD82 antigen	NM_212661.2
B21G0153	39.25	28.90	DYNLL2	Dynein light chain 2, cytoplasmic	NM_001030000.2
A11G0077	39.25	23.31	ACYP2	Acylphosphatase-2	NM_001024407.2
A18G0736	39.23	25.64	SERP1	Stress-associated endoplasmic reticulum protein 1	
A5G1082	39.23	61.49	ATP6V1G1	V-type proton ATPase subunit G 1	NM_199934.1
A8G0638	39.22	4.94	LIPG	Endothelial lipase	NM_200128.1
B20G0481	39.22	24.01	MAX	Protein max	XM_005160619.5
A6G0875	39.22	15.80	EIF2S2	Eukaryotic translation initiation factor 2 subunit 2	NM_212675.1
A19G0127	39.17	93.57	PABPC1A	Polyadenylate-binding protein 1A	NM_200882.1
A9G0526	39.14	22.79	UCHL3	Ubiquitin carboxyl-terminal hydrolase isozyme L3	NM_001024405.1
A19G0143	39.12	32.77	CHTOP	Chromatin target of PRMT1 protein	NM_199546.1
B19G0001	39.08	48.03	MCL1	Induced myeloid leukemia cell differentiation protein Mcl-1 homolog	
B6G0986	39.06	22.36	NCOA5	Nuclear receptor coactivator 5	XM_005166160.5
B10G0124	39.05	38.32	COPS4	COP9 signalosome complex subunit 4	NM_205556.1
B22G0807	39.04	62.79	PTPRC	Receptor-type tyrosine-protein phosphatase C	XM_068216640.1
B24G0318	39.04	19.77	ZMYND11	Zinc finger MYND domain-containing protein 11	XM_021470178.2
B12G0483	39.02	94.69	AQP8	Aquaporin-8	NM_001004661.1
A5G0335	38.99	23.49	VMA21	Vacuolar ATPase assembly integral membrane protein vma21	NM_001202424.1
B8G0619	38.99	11.53	RASSF8	Ras association domain-containing protein 8	
A21G0161	38.98	77.15	TBCA	Tubulin-specific chaperone A	NM_201054.1
A14G0731	38.94	23.43	CANX	Calnexin	NM_213448.1
B3G1362	38.94	24.19	ARHGDIA	Rho GDP-dissociation inhibitor 1	NM_213461.1
B24G0347	38.86	18.37	TFRC	Transferrin receptor protein 1	NM_001009918.2
A16G0068	38.86	0.36	SERPINB1	Leukocyte elastase inhibitor	XM_002665065.7
B14G0470	38.84	22.57	CTNNA1	Catenin alpha-1	NM_131456.2
B14G0770	38.84	13.75	PLS3	Plastin-3	NM_001002326.1
B7G0602	38.84	25.92	CEBPG	enhancer-binding protein gamma	NM_131886.2
B25G0719	38.79	16.04	ARPP19	cAMP-regulated phosphoprotein 19	NM_200972.1
B2G0623	38.79	27.94	MYCB	Transcriptional regulator Myc-B	NM_200172.1

A12G0669	38.77	8.72	MYOF	Myoferlin	NM_200875.2
A6G0734	38.74	12.29	CRACR2A	EF-hand calcium-binding domain-containing protein 4B	
B3G1334	38.73	22.61	AIMP2	Aminoacyl tRNA synthase complex-interacting multifunctional protein 2	NM_213338.1
B13G0770	38.70	14.09	MLH1	DNA mismatch repair protein Mlh1	
B23G0799	38.64	52.63	MICOS10	MICOS complex subunit MIC10	
A7G0495	38.62	32.28	UQCRFS1	Cytochrome b-c1 complex subunit Rieske, mitochondrial	NM_001103194.1
B21G0805	38.61	31.70	UQCR10	Cytochrome b-c1 complex subunit 9	NM_001302478.1
A2G0152	38.61	44.98	LEPROT	Leptin receptor gene-related protein	NM_001017787.1
A4G0871	38.59	22.11	ARL1	ADP-ribosylation factor-like protein 1	NM_001002473.1
B12G0672	38.57	25.72	UBE2D4	Ubiquitin-conjugating enzyme E2 D4	NM_199664.1
B3G0725	38.56	27.85	GNG10	G(O) subunit gamma-10	
A2G0083	38.55	18.57	THOC1	THO complex subunit 1	NM_201324.1
B10G0067	38.54	32.63	VAMP8	Vesicle-associated membrane protein 8	NM_001293183.1
A14G0325	38.52	150.03	SPINK1	Serine protease inhibitor Kazal-type 1	
B3G0358	38.52	6.47	IRLD	threonine-protein kinase irlD	
B16G0088	38.49	4.12	SERPINB1	Leukocyte elastase inhibitor	XM_002665065.7
A16G0461	38.46	39.94	MCL1	Induced myeloid leukemia cell differentiation protein Mcl-1 homolog	NM_194394.2
A17G0451	38.41	20.83	WDR43	WD repeat-containing protein 43	NM_001018166.1
B7G0772	38.33	119.58	LOC107674451	A0A671NJY6_9TELE Metallothionein	NM_001201469.1
A23G0838	38.26	55.37	CCN1	CCN family member 1	
B23G0422	38.25	14.65	RCC2	Protein RCC2 homolog	XM_005162162.5
B10G0066	38.25	49.33	HNRNPDL	Heterogeneous nuclear ribonucleoprotein D-like	NR_137312.1
B10G0431	38.23	12.81	PTRH2	Peptidyl-tRNA hydrolase 2, mitochondrial	NM_001020535.2
B22G0285	38.23	24.12	LOC113039424	A0A6P6J4H5_CARAU uncharacterized protein LOC113039424	XM_021470864.2
A2G1017	38.16	20.20	PRPF38B	Pre-mRNA-splicing factor 38B	NM_213489.1
A17G0384	38.16	19.20	ID3	DNA-binding protein inhibitor ID-3	NM_152967.1
A3G1220	38.15	25.93	SYNGR2	Synaptogyrin-2	NM_001320463.1
A18G0809	38.14	23.26	KHDC4	KH homology domain-containing protein 4	
A10G0273	38.13	17.63	REXO2	Oligoribonuclease, mitochondrial	NM_200744.2
B7G0202	38.13	6.66	ANO1	Anoctamin-1	XM_009303471.4
A13G0527	38.10	9.14	A0A673GLC2	A0A673GLC2_9TELE Zgc:193505	XM_682373.10
A5G0382	38.09	28.62	PDHA1	Pyruvate dehydrogenase E1 component subunit alpha, somatic form, mitochondrial	XM_068221129.1
B24G0084	38.05	29.53	B0495.8	Uncharacterized protein B0495.8	NM_001002116.1
A14G0354	37.96	32.58	MID1IP1L	Mid1-interacting protein 1-like	
A15G0202	37.96	17.67	PSENEN	Gamma-secretase subunit PEN-2	

A11G0689	37.95	7.07	A0A672M2L7	A0A672M2L7_SINGR Uncharacterized protein	NM_001017657.1
A5G1340	37.93	15.70	RSRC2	serine-rich coiled-coil protein 2	NM_213280.1
B19G0863	37.93	40.21	SH3BGRL3	SH3 domain-binding glutamic acid-rich-like protein 3	NM_200278.2
A13G0690	37.92	17.34	MARCHF8	E3 ubiquitin-protein ligase MARCHF8	NM_213110.1
B5G0830	37.91	10.39	MICU2	Calcium uptake protein 2, mitochondrial	NM_001017539.2
B4G0116	37.91	40.63	NDUFA5	NADH dehydrogenase [ubiquinone] 1 alpha subcomplex subunit 5	
A5G1255	37.90	77.82	AK1	Adenylate kinase isoenzyme 1	NM_001003993.1
B18G0589	37.90	29.17	UBE2N	Ubiquitin-conjugating enzyme E2 N	NM_213486.1
A24G0281	37.89	26.76	ZMYND11	Zinc finger MYND domain-containing protein 11	NM_001077583.3
B8G0261	37.89	10.44	PDLIM2	PDZ and LIM domain protein 2	NM_001326402.1
B6G0890	37.89	22.63	CYC1	Cytochrome c1, heme protein, mitochondrial	NM_001037393.2
A6G0193	37.86	33.41	SUMO1	Small ubiquitin-related modifier 1	NM_213159.1
B14G0335	37.84	35.95	HNRNPA0	Heterogeneous nuclear ribonucleoprotein A0	XM_068213169.1
A22G0124	37.80	20.69	NMRK2	Nicotinamide riboside kinase 2	NM_131693.2
A15G0344	37.78	11.51	LOC113059002	A0A6P6LHP9_CARAU lymphocyte antigen 6D-like	NM_001386226.1
A23G0419	37.77	11.63	HES4-B	Transcription factor HES-4-B	XM_005162143.5
B4G0657	37.77	23.18	RAB21	Ras-related protein Rab-21	NM_001076665.1
B20G0275	37.76	34.11	C1QL4	Complement C1q-like protein 4	
B9G0492	37.75	22.47	COL6A3	Collagen alpha-3(VI) chain	XM_017357806.3
A16G0450	37.75	32.22	CCT3	T-complex protein 1 subunit gamma	NM_173250.1
A7G0497	37.75	22.08	CIAO2B	Cytosolic iron-sulfur assembly component 2B	NM_001002449.1
B8G1059	37.74	42.50	TMED7	Transmembrane emp24 domain-containing protein 7	NM_001313710.1
B10G0263	37.73	31.43	GOLGA7	Golgin subfamily A member 7	NM_001105599.1
A7G0175	37.72	69.86	GABARAP	Gamma-aminobutyric acid receptor-associated protein	NM_001386387.1
B6G0664	37.71	22.41	KRT18	Keratin, type I cytoskeletal 18	NM_200568.1
A18G0770	37.71	97.33	PKM	Pyruvate kinase PKM	NM_199333.1
B12G0452	37.70	26.73	PDAP1	28 kDa heat- and acid-stable phosphoprotein	NM_212632.2
B22G0154	37.68	24.97	CALR	Calreticulin	NM_131047.2
A3G1117	37.67	25.39	KDELR2	ER lumen protein-retaining receptor 2	NM_200103.1
A19G0048	37.67	36.96	UBE2E1	Ubiquitin-conjugating enzyme E2 E1	NM_001103116.1
B5G1102	37.67	52.86	EWSR1	RNA-binding protein EWS	NM_212630.1
A7G0944	37.64	29.64	RBM4.1	RNA-binding protein 4.1	NM_213317.3
A6G0148	37.62	22.02	MYH9	Myosin-9	XM_005165695.5
B13G0052	37.61	6.62	ROHU_026187	A0A498MBN6_LABRO Nuclease HARBI1	
B25G0330	37.60	58.83	RPS27L	40S ribosomal protein S27-like	NM_001172680.1
B7G0520	37.60	27.03	PUF60B	Poly(U)-binding-splicing factor PUF60-B	NM_001430966.1
A17G0136	37.57	26.58	GSKIP	GSK3-beta interaction protein	
B12G0813	37.53	20.10	LSM12A	Protein LSM12 homolog A	NM_213148.1
B12G0826	37.51	47.30	EIF3C	Eukaryotic translation initiation factor 3 subunit C	XM_005155999.5

B6G0613	37.49	64.16	ZRANB2	Zinc finger Ran-binding domain-containing protein 2	NM_001328210.1
B1G0527	37.47	24.45	AS3MT	Arsenite methyltransferase	NM_001039839.1
B12G0583	37.44	42.86	PSME2	Proteasome activator complex subunit 2	NM_131374.1
A1G0677	37.43	35.17	SUGT1	Protein SGT1 homolog	NM_001007361.1
A13G0316	37.42	19.87	SIX4	Homeobox protein SIX4	XM_005156542.5
B16G0357	37.36	15.60	MRPS15	28S ribosomal protein S15, mitochondrial	NM_001002485.2
A18G0341	37.36	46.22	CAPRIN1	Caprin-1	XM_068214751.1
A13G0721	37.36	48.92	AHNAK2	Protein AHNAK2	
A24G0073	37.35	34.97	LUC7L3	Luc7-like protein 3	XM_005162898.4
B3G0836	37.33	18.02	RAB35	Ras-related protein Rab-35	XM_021471455.2
B24G0309	37.32	26.88	LOC113042374	A0A6P6J9X0_CARAU uncharacterized protein LOC113042374 isoform X1	
A6G0502	37.28	28.57	LSM2	U6 snRNA-associated Sm-like protein LSm2	NM_131496.2
A7G0905	37.28	36.06	TXN	Thioredoxin	NM_001002461.1
A13G0480	37.27	39.94	CEP68	Centrosomal protein of 68 kDa	NM_001017870.1
A9G0499	37.27	14.57	RND3	Rho-related GTP-binding protein RhoE	NM_001002591.1
B10G0789	37.24	48.43	TMED2	Transmembrane emp24 domain-containing protein 2	NM_199548.2
B24G0700	37.23	21.11	GYG1	Glycogenin-1	NM_001002062.1
A20G0843	37.21	5.16	SPINT1	Kunitz-type protease inhibitor 1	NM_001111223.1
A8G0926	37.20	10.54	PPIH	Peptidyl-prolyl cis-trans isomerase H	NM_201205.1
B6G0500	37.18	23.68	LSM2	U6 snRNA-associated Sm-like protein LSm2	NM_131496.2
B2G0008	37.16	44.72	NDUFA13	NADH dehydrogenase [ubiquinone] 1 alpha subcomplex subunit 13	
A16G0366	37.15	27.96	AKIRIN1	Akirin-1	NM_001007186.2
A7G1000	37.15	41.30	CNFN	Cornifelin	NM_001327748.1
B24G0527	37.13	74.52	CMBL	Carboxymethylenebutenolidase homolog	NM_001109832.1
A25G0723	37.10	41.15	GPI	Glucose-6-phosphate isomerase	NM_144763.2
A22G0221	37.08	24.26	CCER1	Coiled-coil domain-containing glutamate-rich protein 1	XM_001332531.9
A3G0603	37.08	36.92	SCPEP1	Retinoid-inducible serine carboxypeptidase	NM_200192.1
A24G0207	37.07	9.28	A0A673GST2	A0A673GST2_9TELE Chemerin	NM_001128775.1
B5G0883	37.04	10.14	SURF6	Surfeit locus protein 6 homolog	XM_005171804.5
A25G0495	37.03	34.36	CIART	Circadian-associated transcriptional repressor	XM_695543.10
A12G0772	37.01	45.35	EIF3C	Eukaryotic translation initiation factor 3 subunit C	XM_005155999.5
A12G0657	36.95	44.94	PSMC5	26S proteasome regulatory subunit 8	NM_001003740.1
A21G0805	36.94	31.83	NDFIP1L	NEDD4 family-interacting protein 1-like	XM_005173872.3
B25G0167	36.91	56.54	IGKV3-11	Immunoglobulin kappa variable 3-11	XM_068217596.1
A13G0081	36.91	10.44	SLC39A9-B	Zinc transporter ZIP9-B	
A5G0598	36.89	18.53	RPL12	60S ribosomal protein L12	NM_201584.4
B16G0142	36.84	2.71	RIPK1	threonine-protein kinase 1	XM_002665035.7
A3G0931	36.80	26.56	NUPR1	Nuclear protein 1	
A4G0512	36.80	26.30	ATP5F1C	ATP synthase subunit gamma, mitochondrial	NM_200041.2

B25G0347	36.77	55.44	DLD	Dihydrolipoyl dehydrogenase, mitochondrial	NM_201506.1
B19G0257	36.77	24.19	CLK2	Dual specificity protein kinase CLK2	NM_001044879.2
B9G0621	36.75	31.10	DARS1	Aspartate--tRNA ligase, cytoplasmic	NM_001077611.1
B8G0690	36.74	27.04	NPL	N-acetylneuraminate lyase	NM_207051.2
A1G0806	36.74	31.94	LANCL1	Glutathione S-transferase LANCL1	NM_001009891.2
A10G0012	36.73	53.66	ETS2	Protein C-ets-2	NM_001023580.1
A5G0445	36.72	9.48	AK6	Adenylate kinase isoenzyme 6	NM_001002118.2
B5G0082	36.72	21.41	APOO	MICOS complex subunit MIC26	
A2G0228	36.71	32.00	COPE	Coatomer subunit epsilon	NM_001007364.1
B2G0884	36.70	13.89	ARPC5-A	3 complex subunit 5-A	NM_194378.4
B18G0008	36.70	24.43	CTTN	Src substrate cortactin	NM_001423896.1
A14G0255	36.69	21.04	LAMP2	Lysosome-associated membrane glycoprotein 2	XM_009291116.4
A13G0890	36.66	19.50	RANBP2	E3 SUMO-protein ligase RanBP2 (Fragment)	
B8G0258	36.65	1.07	G2E3	M phase-specific E3 ubiquitin-protein ligase	
B15G0759	36.61	16.15	PSENEN	Gamma-secretase subunit PEN-2	
B5G0240	36.60	15.08	ARL6IP4	ADP-ribosylation factor-like protein 6-interacting protein 4	NM_001020758.1
A3G0502	36.58	23.01	ATP5MF	ATP synthase subunit f, mitochondrial	NM_001303129.1
A14G0371	36.57	10.37	GAR1	ACA ribonucleoprotein complex subunit 1	NM_200975.2
B13G0362	36.57	16.04	TSPAN14	Tetraspanin-14	NM_001017833.1
B7G0470	36.55	40.68	UQCRFS1	Cytochrome b-c1 complex subunit Rieske, mitochondrial	NM_001103194.1
A5G0629	36.55	51.21	AIF1L	Allograft inflammatory factor 1-like	NM_198870.1
B21G0191	36.54	18.99	KLF5	Krueppel-like factor 5	NM_001039927.2
A21G0816	36.52	21.96	FUNDC2	FUN14 domain-containing protein 2	NM_001005954.1
A16G0236	36.52	17.42	PYCARD	Apoptosis-associated speck-like protein containing a CARD	
B6G0119	36.48	40.99	RTN4	Reticulon-4	NM_001079912.2
A5G0839	36.46	32.57	LOC113091252	beta receptor 1-like	
B19G0298	36.43	99.11	LOC113120150	A0A6P6RKR0_CARAU uncharacterized protein LOC113120150	
A18G0652	36.41	28.62	ARF5	ADP-ribosylation factor 5	NM_199876.1
B5G0013	36.41	24.57	RNF185	E3 ubiquitin-protein ligase RNF185	NM_213037.1
A3G0474	36.41	18.93	PSMG3	Proteasome assembly chaperone 3	NM_001002529.1
A14G0216	36.39	41.24	RBMX	RNA-binding motif protein, X chromosome	NM_212598.1
A14G0645	36.34	12.86	ETF1	Eukaryotic peptide chain release factor subunit 1	NM_001111171.1
B2G1225	36.34	14.32	DSP	Desmoplakin	NM_001423755.1
B7G0835	36.33	88.51	SERPINE1	Plasminogen activator inhibitor 1	XM_068222132.1
A9G0683	36.32	54.97	U2AF1	Splicing factor U2AF 35 kDa subunit	NM_177479.2
B19G0659	36.32	31.98	FUCA1	Tissue alpha-L-fucosidase	NM_001003559.2
B8G0967	36.29	10.45	CLUH	Clustered mitochondria protein homolog	XM_068223061.1
A5G0461	36.27	46.56	CD53	Leukocyte surface antigen CD53	NM_200371.1
B5G0980	36.24	16.08	F11R	Junctional adhesion molecule A	NM_001004667.3

A2G0661	36.22	27.93	CYRIB	CYFIP-related Rac1 interactor B	NM_200070.3
A13G0099	36.22	33.39	CHP1	Calcineurin B homologous protein 1	XM_005156487.5
B12G0505	36.22	4.76	BAIAP2L1	Brain-specific angiogenesis inhibitor 1-associated protein 2-like protein 1	NM_198922.1
A8G0453	36.17	11.94	SLC38A3	Sodium-coupled neutral amino acid transporter 3	NM_001002648.1
B19G0619	36.17	4.74	ELP2	Elongator complex protein 2	XM_005159658.5
B6G0939	36.14	43.65	GNB1	G(T) subunit beta-1	NM_213481.1
A5G0580	36.12	16.76	ARPC5L	3 complex subunit 5-like protein	NM_001128686.2
B1G0134	36.11	9.17	FAM83G	Protein FAM83G	XM_005170874.5
A7G0599	36.11	20.61	MTCH2	Mitochondrial carrier homolog 2	NM_131382.1
A3G0788	36.05	27.10	NUCB1	Nucleobindin-1	XM_005163951.5
B4G0491	36.01	6.04	G5714_004825	A0A7J6D5W7_9TELE Uncharacterized protein	
A8G0112	35.97	12.86	SEPTIN2	Septin-2	XM_005172457.5
A15G0153	35.96	16.60	LOC113080893	A0A6P6NKT2_CARAU uncharacterized protein LOC113080893 isoform X1	XM_068213786.1
B1G0209	35.92	13.71	MPC2	Mitochondrial pyruvate carrier 2	NM_001004662.3
A8G0108	35.91	3.73	CLUH	Clustered mitochondria protein homolog	XM_068223061.1
B19G0599	35.90	33.12	PTP4A2	Protein tyrosine phosphatase type IVA 2	NM_001270540.1
A3G1095	35.90	39.21	IL6ST	Interleukin-6 receptor subunit beta	NM_001365786.1
B12G0767	35.89	3.55	WFIKKN2	WAP, Kazal, immunoglobulin, Kunitz and NTR domain-containing protein 2	XM_694147.6
B11G0752	35.89	39.30	OPN5	Opsin-5	NR_155546.1
A9G0331	35.88	18.26	HSPD1	60 kDa heat shock protein, mitochondrial	NM_181330.3
A3G0330	35.85	0.36	HSD17B2	Estradiol 17-beta-dehydrogenase 2	NM_001004556.2
B20G0243	35.84	65.60	PLPP3	Phospholipid phosphatase 3	XM_003200678.6
A14G0444	35.81	28.28	MIF	Macrophage migration inhibitory factor	NM_001043321.1
B5G1249	35.80	9.82	ISG15	Ubiquitin-like protein ISG15	
A23G0173	35.73	29.55	NAA10	N-alpha-acetyltransferase 10	NM_213334.1
A1G0995	35.73	10.98	CRIP3	Cysteine-rich protein 3	NM_001161335.2
B18G0510	35.73	29.61	RAB11A	Ras-related protein Rab-11A	NM_001007359.1
B8G0520	35.69	22.27	EFHD2	EF-hand domain-containing protein D2	NM_001326493.1
B17G0473	35.69	16.85	SRRM1	arginine repetitive matrix protein 1	NM_001365281.1
B19G0500	35.69	22.27	PUM1	Pumilio homolog 1	NM_001277119.2
A16G0965	35.65	20.63	SRI	Sorcin	XM_068213916.1
A16G0338	35.62	9.06	RAB39A	Ras-related protein Rab-39A	NM_001017683.2
B11G0313	35.59	25.67	LOC107696829	A0A671T1P0_9TELE Uncharacterized LOC107696829	NM_212732.2
A3G1198	35.58	33.29	LUC7L3	Luc7-like protein 3	NM_001080591.1
A6G0041	35.56	21.88	GRAP2	GRB2-related adapter protein 2	NM_200678.2
A16G0435	35.56	8.83	G5714_015956	A0A7J6C8R9_9TELE Uncharacterized protein	XM_017351376.3
B15G0037	35.55	73.59	TAGLN	Transgelin	NM_001045467.1
A5G0403	35.53	21.62	TIA1	Cytotoxic granule associated RNA binding protein TIA1	XM_005165110.5
A4G1038	35.51	19.13	MRPS10	Probable 28S ribosomal protein S10, mitochondrial	NM_001159988.1

A1G0555	35.51	25.15	LSM6	U6 snRNA-associated Sm-like protein LSm6	NM_001002077.2
B21G0399	35.46	71.82	CALM1	Calmodulin-1	NM_199570.1
A5G0042	35.45	31.59	PPP1CC-A	threonine-protein phosphatase PP1-gamma catalytic subunit A	XM_695376.8
A11G0424	35.44	26.92	GUCA2A	Guanylin	NM_198367.1
B7G0018	35.42	27.61	PPP1CB	threonine-protein phosphatase PP1-beta catalytic subunit	XM_695376.8
B14G0117	35.42	14.49	TMED9	Transmembrane emp24 domain-containing protein 9	XM_005173178.4
B5G1223	35.39	7.16	LIMK2	LIM domain kinase 2	NM_001002651.1
B18G0380	35.38	30.95	CD82	CD82 antigen	NM_212663.1
B24G0704	35.36	0.06	F13A1	Coagulation factor XIII A chain	XM_681557.9
A10G0103	35.34	16.85			XM_003199277.6
A19G0417	35.34	1.32	A0A673JMX7	A0A673JMX7_9TELE Si:ch211-191i18.2	
A19G0721	35.33	9.01	MRPL24	Probable 39S ribosomal protein L24, mitochondrial	
B5G1132	35.33	18.15	TP53	Cellular tumor antigen p53	NM_001271820.1
A17G0293	35.30	4.19	GRHL1	Grainyhead-like protein 1 homolog	XM_001923728.6
B12G0329	35.29	82.37	AUTS2	Autism susceptibility gene 2 protein homolog	XM_005156304.5
B6G0189	35.28	32.60			
A12G0150	35.28	93.22	PMP22	Peripheral myelin protein 22	NM_001040355.2
B16G0333	35.28	6.23	G5714_015832	A0A7J6C6N8_9TELE Uncharacterized protein	
A7G1167	35.26	17.59			
A3G0300	35.26	12.32	CCDC137	Coiled-coil domain-containing protein 137	
A24G0210	35.24	10.54	RBIS	Ribosomal biogenesis factor	
B4G0330	35.23	59.49	MGST1	Microsomal glutathione S-transferase 1	NM_001002215.2
B6G0209	35.22	22.41	SUMO1	Small ubiquitin-related modifier 1	NM_213159.1
B13G0209	35.15	18.12	TRMT61A	tRNA (adenine(58)-N(1))-methyltransferase catalytic subunit TRMT61A	NM_213110.1
A2G0165	35.15	35.01	PRDX1	Peroxiredoxin-1	NM_001013471.2
B17G0454	35.13	8.52	GJB3	Gap junction beta-3 protein	NM_001017685.1
A4G0761	35.11	55.45	CYB5R3	NADH-cytochrome b5 reductase 3	NM_212685.1
A23G0691	35.04	23.78	COPZ1	Coatomer subunit zeta-1	NM_131508.1
B20G0686	35.03	11.81	DNAJC5	DnaJ homolog subfamily C member 5	XM_005160813.5
A20G0059	35.02	21.48	PSMG4	Proteasome assembly chaperone 4	
B9G0589	35.01	22.46	TFG	Protein TFG	NM_200015.2
A3G0804	35.01	19.94	EIF3D	Eukaryotic translation initiation factor 3 subunit D	NM_200016.1
A18G0708	34.99	27.33	PDCD10A	Programmed cell death protein 10-A	NM_200555.2
A25G0817	34.97	16.10			
B6G0378	34.96	38.25	UQCRC1	Cytochrome b-c1 complex subunit 1, mitochondrial	NM_200820.1
A25G0042	34.96	37.34	METAP2	Methionine aminopeptidase 2	NM_199640.1
A6G0877	34.92	29.70	CYC1	Cytochrome c1, heme protein, mitochondrial	NM_001037393.2
A11G0341	34.92	12.15	MXRA8	Matrix remodeling-associated protein 8	NM_001003429.2
B7G0302	34.92	37.90	OSTC	Oligosaccharyltransferase complex subunit ostc	NM_200269.2

A4G0919	34.89	48.46	CD9	CD9 antigen	XM_005158981.5
B6G0093	34.88	12.10	PPIG	Peptidyl-prolyl cis-trans isomerase G	NM_213464.1
A4G0780	34.86	21.99	CCDC59	Thyroid transcription factor 1-associated protein 26 homolog	NM_001045054.1
A6G1030	34.82	52.89	DDX3Y	ATP-dependent RNA helicase DDX3Y	XM_068221646.1
A5G0077	34.82	23.39	SRRT	Serrate RNA effector molecule homolog	NM_173238.2
A8G0669	34.81	4.28	RFLNA	Refilin-A	NM_001007031.2
B6G0727	34.77	20.95	ARPC4	3 complex subunit 4	NM_205537.1
B23G0525	34.77	34.50	PTGES3	Prostaglandin E synthase 3	NM_213170.1
A11G0226	34.76	40.55	ADORA1	Adenosine receptor A1	
A6G0263	34.74	21.43	A0A672M0A6	A0A672M0A6_SINGR Si:ch73-71c20.5	NM_001326565.1
A16G0796	34.74	13.81	CCDC12	Coiled-coil domain-containing protein 12	NM_001003589.2
B2G0310	34.73	23.77	GTPBP4	GTP-binding protein 4	NM_199851.1
A7G0712	34.72	12.75	CARS1	Cysteine--tRNA ligase, cytoplasmic	NM_001118900.1
A13G0206	34.70	32.86	G5714_013662	A0A7J6CFK9_9TELE DH domain-containing protein	
B15G0749	34.69	5.12	ST14	Suppressor of tumorigenicity 14 protein homolog	XR_011006502.1
B22G0476	34.67	5.93	CD244	Natural killer cell receptor 2B4	XM_068216631.1
B3G0313	34.66	22.06	PAM16	Mitochondrial import inner membrane translocase subunit tim16	NM_200804.2
B14G0141	34.63	13.67	NXT2	NTF2-related export protein 2	NM_001312912.1
B1G0370	34.60	21.24	ANXA5	Annexin A5	NM_181757.2
B5G0992	34.53	10.75	ENTPD2	Ectonucleoside triphosphate diphosphohydrolase 2	NM_001004643.2
A8G0760	34.52	25.26	RBM39	RNA-binding protein 39	NM_001014370.1
A7G1065	34.51	38.08	RAB1A	Ras-related protein Rab-1A	NM_201142.1
A19G0878	34.50	16.55	TOMM7	Mitochondrial import receptor subunit TOM7 homolog	
A6G0865	34.49	21.24	NPEPL1	Probable aminopeptidase NPEPL1	NM_001017688.1
A6G0970	34.46	27.79	NCOA5	Nuclear receptor coactivator 5	XM_005166160.5
B15G0356	34.38	8.93	G5714_014994	A0A7J6C9L2_9TELE Ig-like domain-containing protein	XM_068213625.1
A10G0464	34.36	42.63	CREBZF	ATF bZIP transcription factor	XM_009305485.4
A6G0231	34.36	37.53	SUMO3L	Small ubiquitin-related modifier 3-like	NM_213124.2
B3G1155	34.36	23.60	GRB2	Growth factor receptor-bound protein 2	NM_213035.1
A24G0486	34.35	38.63	PABPN1-B	Polyadenylate-binding protein 2-B	NM_001105132.2
A19G0569	34.34	35.65	RAB5A	Ras-related protein Rab-5A	NM_201485.1
A18G0309	34.33	5.55	PPP1R13L	RelA-associated inhibitor	XM_005173643.5
B20G0641	34.31	21.21	SRP9	Signal recognition particle 9 kDa protein	
B15G0604	34.28	12.25	SERPINH1	Serpin H1	NM_131204.2
B18G0800	34.27	13.26	PIGBOS1	A0A6P6RBK2_CARAU protein PIGBOS1	NM_001308439.1
B15G0013	34.21	27.64	NIPSNAP2	Protein NipSnap homolog 2	NM_131034.1
A14G0320	34.15	4.38	LOC107676263	A0A671QF11_9TELE Vasohibin-2-like	
A15G0529	34.14	12.32	ATPI1	Actinia tenebrosa protease inhibitors	
A6G0702	34.11	40.06	TSPO	Translocator protein	NM_001006032.2
B23G0973	34.10	13.30	PSMB6	Proteasome subunit beta type-6	NM_131152.1

B16G0791	34.09	22.52	GABARAPL1	Gamma-aminobutyric acid receptor-associated protein-like 1	NM_001002707.2
A1G0649	34.08	8.20	CBS	Cystathionine beta-synthase	NM_001111232.1
B20G0202	34.08	30.86	CALMA	Calmodulin-alpha (Fragment)	NM_200082.1
B6G0691	34.02	30.84	IP6K2	Inositol hexakisphosphate kinase 2	NM_001002389.1
B8G0517	34.00	35.51	RHOAB	Rho-related GTP-binding protein RhoA-B	NM_212749.2
A16G0738	33.99	11.81	MFAP2	Microfibrillar-associated protein 2	NM_001386410.1
B16G0460	33.97	27.98	CD40	Tumor necrosis factor receptor superfamily member 5	
B1G0746	33.96	32.15	DDX39A	ATP-dependent RNA helicase DDX39A	NM_001328342.1
A14G0225	33.92	1.11	SOX3	Transcription factor Sox-3	NM_001001811.2
A13G0671	33.91	81.60	TMEM150A	Transmembrane protein 150A	NM_001077315.1
A20G0241	33.91	74.86	SERINC1	Serine incorporator 1	NM_199727.1
B19G0043	33.90	7.20	MR1	Major histocompatibility complex class I-related gene protein	
A13G0741	33.88	17.16	STPG4	Protein STPG4	NM_213119.2
B16G0453	33.86	32.92	MLF2	Myeloid leukemia factor 2	NM_001025516.1
A23G0664	33.85	15.98	TMEM18	Transmembrane protein 18	
A22G0691	33.84	20.60	TTC14	Tetratricopeptide repeat protein 14	NM_001014314.2
A10G0609	33.84	30.33	ETF1	Eukaryotic peptide chain release factor subunit 1	XM_021479101.2
B5G0877	33.84	14.74	PTGES	Prostaglandin E synthase	NM_001014828.2
B2G0377	33.82	20.07	NEDD8	NEDD8	NM_001002557.1
B1G0687	33.82	12.19	TIMM10	Mitochondrial import inner membrane translocase subunit Tim10	NM_001002678.3
A22G0271	33.81	18.74	CCNT2	Cyclin-T2	NM_212586.1
B20G0447	33.79	21.56	A0A671NBQ1	A0A671NBQ1_9TELE Si:dkeyp-55f12.3	
B19G0942	33.76	5.41	DDAH2	N(G),N(G)-dimethylarginine dimethylaminohydrolase 2	NM_001100065.2
B18G0143	33.75	14.27	POLR2I	DNA-directed RNA polymerase II subunit RPB9	NM_001006013.3
A8G0889	33.74	21.02	PLP2	Proteolipid protein 2	NM_001161445.1
B16G0946	33.74	7.27	G5714_016379	A0A7J6C8H2_9TELE Uncharacterized protein	
B1G0966	33.74	4.08	KNOP1	Lysine-rich nucleolar protein 1	XM_682043.9
B3G1654	33.72	29.99	HB2D	DLA class II histocompatibility antigen, DR-1 beta chain	
B20G0093	33.71	4.58	SPINT1	Kunitz-type protease inhibitor 1	NM_001111223.1
B3G0979	33.69	64.11	A0A671SUM3	A0A671SUM3_9TELE Si:ch211-76l23.4	XM_001334914.9
A12G0073	33.68	11.84	ADK	Adenosine kinase	NM_198802.1
B11G0510	33.68	60.14	RPGR	X-linked retinitis pigmentosa GTPase regulator	XM_068224238.1
A9G0581	33.68	30.34	DARS1	Aspartate--tRNA ligase, cytoplasmic	NM_001077611.1
B14G0503	33.68	22.76	ATP6V0E1	V-type proton ATPase subunit e 1	
B4G0411	33.67	29.54	ROHU_026581	A0A498MEJ3_LABRO Uncharacterized protein	
A2G0644	33.63	547.56	ROHU_024221	A0A498LJX8_LABRO Uncharacterized protein	
A3G0261	33.62	13.59	ELOF1	Transcription elongation factor 1 homolog	NM_200386.1

B2G0778	33.61	55.11	IFI30	Gamma-interferon-inducible lysosomal thiol reductase	
B7G0587	33.61	33.67	NDUFS3	NADH dehydrogenase [ubiquinone] iron-sulfur protein 3, mitochondrial	NM_001017755.1
A24G0003	33.58	36.10	WWP1	NEDD4-like E3 ubiquitin-protein ligase WWP1	XM_068217284.1
B8G0820	33.58	29.97	CCDC124	Coiled-coil domain-containing protein 124	NM_200565.2
A19G0709	33.54	10.55	MYZAP	Myocardial zonula adherens protein	NM_001082986.1
A9G0364	33.53	27.10	TRIM39	E3 ubiquitin-protein ligase TRIM39	
A23G0869	33.52	15.31	HMGB2	High mobility group protein B2	NM_001004674.1
B13G0921	33.51	49.81			NM_001127335.2
A5G1015	33.49	13.19	BCKDK	[3-methyl-2-oxobutanoate dehydrogenase [lipoamide]] kinase, mitochondrial	NM_213060.2
A11G0177	33.49	10.27	SOCS7	Suppressor of cytokine signaling 7	XM_017358271.3
B13G0095	33.48	26.17	BMP5	Bone morphogenetic protein 5	NM_213529.2
A6G0873	33.48	54.68	AHCY-B	Adenosylhomocysteinase B	NM_199218.1
B2G1060	33.47	32.49	COPE	Coatomer subunit epsilon	NM_001007364.1
A21G0245	33.46	58.86	SPTAN1	Spectrin alpha chain, non-erythrocytic 1	NM_001098488.1
B18G0127	33.45	26.45	RNF7	RING-box protein 2	NM_001012498.2
A15G0149	33.43	24.37	LOC113080893	A0A6P6NIC1_CARAU uncharacterized protein LOC113080893 isoform X2	XM_068213786.1
B15G0073	33.42	13.92	CLDN4	Claudin-4	NM_131762.2
B4G0490	33.42	59.97	RAP1B	Ras-related protein Rap-1b	NM_199533.1
B2G0967	33.42	23.53	WLS	Protein wntless homolog	NM_213146.3
A15G0328	33.42	48.72	LGALS9	Galectin-9	NM_001001817.2
A9G0110	33.40	10.81	PHGDH	D-3-phosphoglycerate dehydrogenase	NM_199577.2
A14G0437	33.39	8.51	SIMC1	SUMO-interacting motif-containing protein 1	XM_001922548.6
A1G0413	33.37	0.27	G5714_000532	A0A7J6DHB1_9TELE Uncharacterized protein	XM_021476859.2
B10G0702	33.37	28.60	RAB7	Ras-related protein rab7	NM_001005591.1
A17G0861	33.37	15.12	SPINT1	Kunitz-type protease inhibitor 1	NM_213152.1
A10G0293	33.32	59.33	MMP13	Collagenase 3	NM_001290479.1
B3G0350	33.28	67.97	NFIL3	Nuclear factor interleukin-3-regulated protein	NM_001002218.2
B11G0639	33.28	2.77	ETNK1	Ethanolamine kinase 1	NM_001002369.1
B10G0882	33.24	21.73	SRSF9	arginine-rich splicing factor 9	NM_001312899.1
B23G0445	33.24	12.11	HES4-B	Transcription factor HES-4-B	XM_005162143.5
B7G0406	33.23	21.64	IQGAP1	Ras GTPase-activating-like protein IQGAP1	NM_001308966.1
A25G0533	33.21	21.92	COPS2	COP9 signalosome complex subunit 2	NM_001002055.1
B20G0295	33.19	7.03	PINX1	TERF1-interacting telomerase inhibitor 1	NM_001013265.2
A20G0334	33.12	13.81	CYRIA	CYFIP-related Rac1 interactor A	NM_001324495.1
B1G1001	33.11	25.10	DNAJB1	DnaJ homolog subfamily B member 1	NM_199773.2
B3G0487	33.10	10.42	PPAN	Suppressor of SWI4 1 homolog	NM_201463.1
A2G0289	33.07	23.65	LOC113073559	A0A6P6MZQ7_CARAU uncharacterized protein LOC113073559	

B17G0480	33.06	74.95	PPP1CB	threonine-protein phosphatase PP1-beta catalytic subunit	NM_001004527.4
B19G0880	33.06	57.40	SERINC1	Serine incorporator 1	NM_001045182.1
A7G1359	33.05	21.58	CYLD	Ubiquitin carboxyl-terminal hydrolase CYLD	XM_002662593.5
B3G0595	33.04	19.56	FSCN1	Fascin	NM_001076560.1
A6G0042	33.04	4.18	ADAP1	Arf-GAP with dual PH domain-containing protein 1	NM_001002715.1
A15G0831	33.04	31.67	TAF15	TATA-binding protein-associated factor 2N	NM_001079973.1
B21G0012	33.02	13.36	LOC113038186	A0A6P6ITW9_CARAU uncharacterized protein LOC113038186 isoform X2	
A24G0378	33.01	11.51	DAP	Death-associated protein 1	NM_131572.2
B6G0095	33.01	11.81	SSB	Lupus La protein	NM_199547.1
B5G0684	33.01	30.47	CXCL11.6	C-X-C motif chemokine 11-6	
A11G0650	33.00	28.47	APBA1	Amyloid-beta A4 precursor protein-binding family A member 1	NM_001004601.2
B22G0014	32.98	75.89	ROHU_001730	A0A498L9M4_LABRO Ubiquitin	
B3G1179	32.96	8.76			XM_009299414.4
B22G0067	32.95	8.29	LAMB3	Laminin subunit beta-3	XM_695716.11
B8G0660	32.94	17.59	DOHH	Deoxyhypusine hydroxylase	NM_199563.2
A21G0844	32.94	28.08	SEPT8A	Septin-8-A	NM_001115117.1
A15G0214	32.93	24.38	A0A672MAG5	A0A672MAG5_SINGR FXYD domain-containing ion transport regulator	NM_001386220.1
B6G0672	32.92	46.56	PFDN5	Prefoldin subunit 5	NM_001111195.1
A8G0390	32.91	18.84	CRY1	Cryptochrome-1	NM_131791.2
B7G0156	32.88	18.01	PSMD7	26S proteasome non-ATPase regulatory subunit 7	NM_199789.1
B18G0613	32.81	5.91	MAPK12	Mitogen-activated protein kinase 12	NM_131407.1
A3G0171	32.80	20.55	JPT1	Jupiter microtubule associated homolog 1	NM_205613.1
B2G0622	32.79	22.33	CYRIB	CYFIP-related Rac1 interactor B	NM_200070.3
B10G0152	32.75	22.75	NDUFA8	NADH dehydrogenase [ubiquinone] 1 alpha subcomplex subunit 8	NM_200727.1
B7G0949	32.71	34.64	VIMP	Selenoprotein S	NM_001045334.3
A8G0293	32.68	15.16	SMIM15	Small integral membrane protein 15	NM_001159946.2
A22G0250	32.68	58.60	ATP6AP2	Renin receptor	XM_009296094.4
A8G0828	32.68	33.61	DBNL	Drebrin-like protein	NM_001020700.2
B20G0625	32.68	24.07	CYRIA	CYFIP-related Rac1 interactor A	NM_001324495.1
A10G0523	32.68	30.66	SOD1	Superoxide dismutase [Cu-Zn]	
A5G0791	32.67	36.78	CENPV	Centromere protein V	XM_002662055.6
B11G0746	32.59	14.15	RPL37A	60S ribosomal protein L37a	NM_001037402.2
A7G0860	32.57	74.35	HMGB3	High mobility group protein B3	NM_001122836.1
B7G1079	32.56	20.91	LOC113105527	A0A6P6PQ04_CARAU high mobility group nucleosome-binding domain-containing protein 5-like	
A5G0613	32.56	26.63	USP20	Ubiquitin carboxyl-terminal hydrolase 20	XM_009301458.4
A24G0310	32.56	6.09	CCL20	C-C motif chemokine 20	
A11G0740	32.56	24.56	ANKRD52	threonine-protein phosphatase 6 regulatory ankyrin repeat subunit C	NM_001002698.1

B2G0082	32.56	7.39	CIB3	Calcium and integrin-binding family member 3	XM_003197965.5
B1G1163	32.52	20.97	PIN1	Peptidyl-prolyl cis-trans isomerase NIMA-interacting 1	NM_200748.1
B19G0837	32.51	84.33	PABPC1A	Polyadenylate-binding protein 1A	NM_200882.1
A19G0450	32.50	25.65	WASF2	Actin-binding protein WASF2	NM_001328190.1
A13G0558	32.49	55.85	TRIML1	Probable E3 ubiquitin-protein ligase TRIML1	NM_178307.2
B11G0186	32.49	22.41	GADD45B	Growth arrest and DNA damage-inducible protein GADD45 beta	NM_212610.1
B16G0560	32.48	27.39	LOC107689894	A0A671LWJ8_9TELE Uncharacterized LOC107689894	XM_702438.9
A13G0111	32.46	16.25	ABLIM1	Actin-binding LIM protein 1	NM_001320391.1
B23G0069	32.46	8.75	LOC113040983	HMG-Y-like isoform X1	NM_213168.3
A4G0799	32.45	45.02	HSP90B1	Endoplasmin	NM_198210.2
A25G0508	32.44	13.06	KLF13	Krueppel-like factor 13	NM_001076772.1
A7G1040	32.41	17.91	IL13RA1	Interleukin-13 receptor subunit alpha-1	
B6G0537	32.41	2.18	TTC22	Tetratricopeptide repeat protein 22	XM_021471776.2
B7G1185	32.38	15.10	G5714_008380	A0A7J6CVI7_9TELE Uncharacterized protein	XM_002662815.5
A16G0156	32.37	21.16	RBM8A	RNA-binding protein 8A	NM_001013345.1
B22G0698	32.37	18.95	ARHGAP45	Rho GTPase-activating protein 45	XM_068216451.1
B25G0284	32.37	22.81	EIF3JA	Eukaryotic translation initiation factor 3 subunit J-A	NM_201214.2
B2G0006	32.36	21.08	SUGP1	SURP and G-patch domain-containing protein 1	XM_003197980.6
B11G0526	32.35	33.70	UGT1A6	UDP-glucuronosyltransferase 1-6	NM_001001818.2
B14G0824	32.35	28.75	SH3BGRL	Adapter SH3BGRL	
B5G1047	32.33	20.58	IMP4	U3 small nucleolar ribonucleoprotein protein IMP4	NM_001002719.1
B2G1249	32.32	15.22	POLR2D	DNA-directed RNA polymerase II subunit RPB4	NM_001002317.2
B22G0010	32.30	27.48	SDHB	Succinate dehydrogenase [ubiquinone] iron-sulfur subunit, mitochondrial	NM_001098740.1
B14G0750	32.30	69.50	SQSTM1	Sequestosome-1	NM_001312913.1
A25G0521	32.29	118.83	TPM3	Tropomyosin alpha-3 chain	XM_068217345.1
B13G0154	32.28	25.82	UBR2	E3 ubiquitin-protein ligase UBR2	NM_213119.2
B13G0444	32.28	37.64	G5714_013432	A0A7J6CEW4_9TELE Cilia- and flagella-associated protein 43	XM_005156798.5
A25G0211	32.28	36.66	MAP1LC3B	1B light chain 3B	NM_199604.1
B14G0256	32.27	15.11	MCTS1	Malignant T-cell-amplified sequence 1	NM_201071.1
B3G0928	32.26	40.45	ELOB	Elongin-B	NM_001142954.1
B1G0165	32.22	22.85	NDUFAB1	Acyl carrier protein, mitochondrial	NM_001003418.1
B6G0499	32.19	24.58	INPP5D	Phosphatidylinositol 3,4,5-trisphosphate 5-phosphatase 1	XM_021471767.2
A18G0548	32.18	55.37	UBE2N	Ubiquitin-conjugating enzyme E2 N	NM_213486.1
A1G0434	32.17	4.50	DPX16_16247	A0A3N0XK22_ANAGA Uncharacterized protein	

A24G0348	32.14	22.89	EIF1AX	Eukaryotic translation initiation factor 1A, X-chromosomal	NM_001025453.1
B3G0602	32.10	18.07	PDAP1	28 kDa heat- and acid-stable phosphoprotein	NM_200210.1
B3G0276	32.08	19.88	MAP2K4	Dual specificity mitogen-activated protein kinase kinase 4	XM_009299178.4
A9G0678	32.05	15.98	UBE2A	Ubiquitin-conjugating enzyme E2 A	NM_199719.1
B19G0835	32.04	17.06	SPAG1A	Sperm-associated antigen 1A	NM_001089406.2
A17G0159	32.04	23.49	KTN1	Kinectin	NM_001309329.1
A10G0738	32.04	24.68	NDUFA8	NADH dehydrogenase [ubiquinone] 1 alpha subcomplex subunit 8	NM_200727.1
A19G0861	32.02	4.78			XM_009301169.4
B3G1206	32.02	22.13	ADSL	Adenylosuccinate lyase	NM_199899.2
A19G0737	32.01	21.51	CSNK2A1	Casein kinase II subunit alpha	NM_001002164.1
B20G0338	32.00	2.54	SYNE1	Nesprin-1	NM_001365331.1
B19G0569	31.97	12.24	ZMYM4	Zinc finger MYM-type protein 4	NM_001044320.2
A14G0242	31.96	13.67	GDPD2	Glycerophosphoinositol inositolphosphodiesterase GDPD2	XM_068218761.1
A1G0508	31.96	29.68	TXN	Thioredoxin	NM_200023.1
B1G0976	31.91	20.11	KHSRP	Far upstream element-binding protein 2	NM_001089428.2
A19G0937	31.90	35.03	HNRNPR	Heterogeneous nuclear ribonucleoprotein R	NM_213426.1
B18G0365	31.89	26.74	TBCB	Tubulin-folding cofactor B	NM_212775.1
B20G0263	31.83	1.34	MEP1B	Meprin A subunit beta	NM_001076621.2
A14G0754	31.82	21.54	MAGT1	Magnesium transporter protein 1	NM_199700.1
B13G0120	31.81	15.77	MICU1	Calcium uptake protein 1, mitochondrial	NM_201147.2
B23G0914	31.81	25.31	SYAP1	Synapse-associated protein 1	NM_200942.1
B3G1066	31.79	100.02	ARL5B	ADP-ribosylation factor-like protein 5B	NM_200846.1
B9G0432	31.79	17.89	LMO7	LIM domain only protein 7	XM_068223383.1
A3G0621	31.78	17.68	SMARCE1	SNF-related matrix-associated actin-dependent regulator of chromatin subfamily E member 1	XM_005163989.5
A6G0373	31.77	3.23	EVPL	Envoplakin	NM_001118897.1
B16G0162	31.77	24.90	RPZ	Protein rapunzel	
B2G0229	31.77	9.49	FAM102B	Protein FAM102B	NM_001423874.1
A21G0577	31.74	3.88	CFB	Complement factor B	NM_001083858.1
B5G1234	31.74	20.19	ISCU	Iron-sulfur cluster assembly enzyme ISCU, mitochondrial	NM_001003632.2
B4G0193	31.74	36.01	IFRD1	Interferon-related developmental regulator 1	NM_001076555.1
B2G0022	31.74	35.00	VAPA	Vesicle-associated membrane protein-associated protein A	XM_068223760.1
A15G0381	31.73	10.64	RILPL1	RILP-like protein 1	XM_001344147.8
B21G0338	31.72	22.91	LCP2	Lymphocyte cytosolic protein 2	
A4G0690	31.71	26.56	PSMC2	26S proteasome regulatory subunit 7	NM_200966.1
B11G0190	31.70	25.31	LSM4	U6 snRNA-associated Sm-like protein LSm4	NM_200694.1
B16G0502	31.70	5.26	TNFAIP8L2B	Tumor necrosis factor, alpha-induced protein 8-like protein 2 B	NM_200374.1
B25G0639	31.69	26.13	CCL3	C-C motif chemokine 3	
B16G0263	31.68	26.17	PSMC4	26S proteasome regulatory subunit 6B	NM_199750.2

A14G0342	31.64	20.55	ELF1	ETS-related transcription factor Elf-1	NM_131159.2
A6G0662	31.62	19.24	ATF1	Cyclic AMP-dependent transcription factor ATF-1	NM_199723.1
B23G0300	31.61	14.16	COMMD7	COMM domain-containing protein 7	NM_001003577.1
B13G0829	31.60	49.65	RTASE	Probable RNA-directed DNA polymerase from transposon BS	NM_199718.2
A5G1077	31.59	3.33	A0A671SE50	A0A671SE50_9TELE Si:ch211-202f5.3	XM_003198691.6
B14G0135	31.58	30.26	TSC22D3	TSC22 domain family protein 3	NM_001348480.1
A11G0438	31.55	22.20	LOC113111089	A0A6P6QDA1_CARAU DNA helicase	NM_131842.2
A19G0897	31.53	23.43	LOC113062514	A0A6P6LWJ6_CARAU uncharacterized protein LOC113062514 isoform X3	
B5G0242	31.52	40.96	MOB1A	MOB kinase activator 1A	NM_199914.1
B24G0081	31.52	29.73	IGLC3	Ig lambda-3 chain C region	XM_068217596.1
B9G0404	31.50	15.49	COX17	Cytochrome c oxidase copper chaperone	
B16G0358	31.50	47.80	PLAC8L1	PLAC8-like protein 1	
A25G0117	31.49	33.91	SNX1	Sorting nexin-1	NM_001128671.2
A18G0610	31.47	47.82	FAM107B	Protein FAM107B	NM_001328191.1
A16G0381	31.45	41.16	PNISR	serine-rich protein PNISR	NM_001083837.1
A8G0465	31.44	11.09	MAPK14A	Mitogen-activated protein kinase 14A	XM_005167061.5
B11G0355	31.42	29.55	PRELP	Prolargin	NM_198367.1
A6G0250	31.42	50.27	CG057	Uncharacterized protein C7orf57 homolog	
A19G0093	31.42	35.99	NUDC	Nuclear migration protein nudC	NM_200919.1
A23G0788	31.41	24.28	SCP2	Sterol carrier protein 2	NM_001006093.2
A6G0701	31.40	14.98	UQCC2	Ubiquinol-cytochrome-c reductase complex assembly factor 2	NM_200758.2
A3G0441	31.38	27.16	UBE2IB	SUMO-conjugating enzyme UBC9-B	NM_131833.2
B5G1172	31.38	35.26	CHMP1B	Charged multivesicular body protein 1b	XM_009301289.4
A22G0062	31.37	4.50	LAMB3	A0A6P6J4S5_CARAU laminin subunit beta-3	XM_695716.11
A21G0830	31.35	23.95	DKC1	ACA ribonucleoprotein complex subunit DKC1	NM_001033107.2
B1G0427	31.35	30.04	PDCD4	Programmed cell death protein 4	XM_005159885.5
A6G0606	31.35	0.51	RHCG1	Ammonium transporter Rh type C 1	NM_001109843.1
B16G0322	31.33	27.83	EIF3EA	Eukaryotic translation initiation factor 3 subunit E-A	NM_200839.1
B20G0228	31.29	13.65	MYOC	Myocilin	NM_001015062.2
A2G1141	31.26	27.05	CCL25	C-C motif chemokine 25	
A17G0259	31.26	21.62	ID2	DNA-binding protein inhibitor ID-2	NM_201291.1
B20G0016	31.24	17.85	AKIRIN2	Akirin-2	NM_213294.2
B21G1006	31.24	72.10	G5714_021098	A0A7J6BVR6_9TELE Uncharacterized protein	
A11G0168	31.23	28.87	KRT42	Keratin, type I cytoskeletal 42	NM_213127.1
B11G0667	31.21	17.12	FAM83E	Protein FAM83E	NM_197934.1
A17G0271	31.20	23.84	CCDC28A	Coiled-coil domain-containing protein 28A	XM_005158720.5
A13G0708	31.17	15.49	KLC1	Kinesin light chain 1	NM_001115133.1
B18G0835	31.16	21.43	DUT	Deoxyuridine 5'-triphosphate nucleotidohydrolase, mitochondrial	XM_021467638.2

A3G1041	31.14	6.34	SP110	Sp110 nuclear body protein	
B18G0385	31.10	23.32	SMAP	Small acidic protein	NM_200194.1
B14G0173	31.07	10.65	MRPL11	39S ribosomal protein L11, mitochondrial	NM_001002556.1
B22G0735	31.07	27.58	MICOS13	MICOS complex subunit MIC13	XM_068218990.1
B9G0218	31.06	79.20	SLC40A1	Solute carrier family 40 member 1	NM_131629.1
B21G0471	31.05	5.10	ZBTB16A	Zinc finger and BTB domain-containing protein 16-A	NM_199635.1
A2G1018	31.05	6.57	FAM102B	Protein FAM102B	NM_001423874.1
B9G0253	31.04	21.11	DDX18	ATP-dependent RNA helicase DDX18	NM_001003411.1
B5G1153	31.03	18.92	CWC15	Protein CWC15 homolog	NM_001002050.2
A4G0764	31.03	10.50	NET1	Neuroepithelial cell-transforming gene 1 protein	XM_021475011.2
A9G0131	31.01	59.03	ITGB2	Integrin beta-2	XM_680920.8
A23G0754	30.99	25.32	SIKE1	Suppressor of IKBKE 1	XR_011008508.1
A1G0847	30.99	20.48	NDUFAB1	Acyl carrier protein, mitochondrial	NM_001003418.1
A2G0174	30.99	28.93	RGS3	Regulator of G-protein signaling 3	
A3G0832	30.98	26.34	USP7	Ubiquitin carboxyl-terminal hydrolase 7	XM_021473871.2
B5G0205	30.98	15.26	UBE2L3	Ubiquitin-conjugating enzyme E2 L3	XM_005165039.5
B20G0620	30.97	23.18	HNRNPU	Heterogeneous nuclear ribonucleoprotein U	NM_001033595.2
B19G0438	30.97	21.18	SDC2	Syndecan-2	NM_173223.2
A8G0455	30.97	34.56	NT5DC2	5'-nucleotidase domain-containing protein 2	NM_001077143.2
A5G0389	30.97	29.63	LOC107592292	arginine repetitive matrix protein 1-like	NM_001002300.1
B15G0920	30.95	26.97	PSMA6	Proteasome subunit alpha type-6	NM_131795.2
A7G0941	30.94	28.55	SF1	Splicing factor 1	XM_009303075.3
B19G0914	30.94	20.63	UBE2E1	Ubiquitin-conjugating enzyme E2 E1	NM_001103116.1
A18G0532	30.93	33.62	EIF4G2	Eukaryotic translation initiation factor 4 gamma 2	NM_001013443.2
B9G0430	30.92	17.99	GATD3	Glutamine amidotransferase-like class 1 domain-containing protein 3, mitochondrial	NM_001126455.1
A10G0830	30.91	45.52	HNRNPD	Heterogeneous nuclear ribonucleoprotein D0	XM_021479184.2
B2G0075	30.91	43.80	CALR	Calreticulin	NM_201465.3
B16G0372	30.91	32.46	TAF12	Transcription initiation factor TFIID subunit 12	NM_198368.1
A8G0880	30.91	30.87	HNRNPK	Heterogeneous nuclear ribonucleoprotein K	NM_212994.2
B13G0096	30.87	110.98	LRRC1	Leucine-rich repeat-containing protein 1	
A16G0007	30.86	10.49	MALSU1	Mitochondrial assembly of ribosomal large subunit protein 1	NM_001326615.1
A21G0760	30.81	79.32	DNTS_008897	A0A553MVU5_9TELE Uncharacterized protein	XR_659427.4
B8G0798	30.80	18.25	NDUFB11	NADH dehydrogenase [ubiquinone] 1 beta subcomplex subunit 11, mitochondrial	NM_001044981.1
B23G0438	30.79	63.30	ENO1	Alpha-enolase	XM_005162152.5
A11G0355	30.78	9.93	TIMM17A	Mitochondrial import inner membrane translocase subunit Tim17-A	XM_001923555.8

A7G0702	30.78	28.63	SPI1	Transcription factor PU.1	XM_005168848.5
A1G0585	30.77	18.08	CHMP2B	Charged multivesicular body protein 2b	NM_212904.2
A14G0676	30.75	755.92	ALDOB	Fructose-bisphosphate aldolase B	NM_194367.3
B21G0101	30.75	34.88	FUNDC1	FUN14 domain-containing protein 1	
B24G0200	30.74	12.71	PAK1IP1	p21-activated protein kinase-interacting protein 1-like	XM_068217035.1
A18G0414	30.71	8.31	NUTF2	Nuclear transport factor 2	NM_001006000.3
B5G1045	30.70	37.59	CD53	Leukocyte surface antigen CD53	NM_200371.1
A3G0887	30.65	19.19	STK17A	threonine-protein kinase 17A	XM_017354592.3
A3G1114	30.63	10.66	NDUFB10	NADH dehydrogenase [ubiquinone] 1 beta subcomplex subunit 10	NM_200730.1
A19G0418	30.63	33.62	ANP32E	Acidic leucine-rich nuclear phosphoprotein 32 family member E	NM_213277.1
B1G0712	30.62	134.21	LOC107664542	A0A671KCL4_9TELE Uncharacterized protein	NM_001145239.1
B21G0810	30.59	56.11	CCNI	Cyclin-I	NM_213221.1
A13G0279	30.58	11.02	GOLGA5	Golgin subfamily A member 5	NM_001007289.1
B19G0943	30.57	13.74	LY6L	A0A6P6RFZ5_CARAU lymphocyte antigen 6L isoform X1	
A3G0017	30.55	11.77	RRP7A	Ribosomal RNA-processing protein 7 homolog A	NM_001017579.1
A10G0204	30.55	18.40	RHOF	Rho-related GTP-binding protein RhoF	NM_001020642.1
A3G0616	30.54	22.68	NDUFAB1	Acyl carrier protein, mitochondrial	NM_001305570.1
A12G0076	30.54	27.53	EXOC7	Exocyst complex component 7	XM_005172977.5
B6G0420	30.54	11.32	LOC107754866	A0A673IAY1_9TELE Uncharacterized LOC107754866	NM_001098259.2
A13G0443	30.51	24.94	BAG3	BAG family molecular chaperone regulator 3	NM_001014303.1
A8G0791	30.48	22.35	AEBP1	Adipocyte enhancer-binding protein 1	XM_005167251.5
B8G0559	30.48	39.99	LAMTOR5	Ragulator complex protein LAMTOR5	
B10G0269	30.48	5.42	PLPP5	Phospholipid phosphatase 5	NM_001002211.2
A11G0734	30.47	18.99	INPP1	Inositol polyphosphate 1-phosphatase	XM_021479603.2
A19G0895	30.45	10.84	G5714_024648	A0A7J6BHK8_9TELE IG domain-containing protein	
B23G0099	30.45	58.94	TCP1	T-complex protein 1 subunit alpha	NM_131230.1
B3G0180	30.43	17.69	POLR2F	DNA-directed RNA polymerases I, II, and III subunit RPABC2	NM_001002544.2
A6G0030	30.40	12.45	RPS19BP1	Active regulator of SIRT1	NM_001386402.1
B17G0013	30.39	12.61	WARS1	Tryptophan--tRNA ligase, cytoplasmic	NM_200772.1
B16G0666	30.35	289.65	FLT4	Vascular endothelial growth factor receptor 3	
A13G0598	30.31	0.10	OGA	Protein O-GlcNAcase	
A6G0261	30.31	10.52	CXCR4-B	C-X-C chemokine receptor type 4-B	NM_131882.3
B8G0586	30.28	17.43	WAS	Actin nucleation-promoting factor WAS	NM_199938.1

B9G0480	30.28	43.78	NDUFA10	NADH dehydrogenase [ubiquinone] 1 alpha subcomplex subunit 10, mitochondrial	NM_199578.1
A9G0593	30.27	18.32	ZC3H15	Zinc finger CCCH domain-containing protein 15	NM_199888.1
A5G0067	30.27	12.08	C1QBP	Complement component 1 Q subcomponent-binding protein, mitochondrial	NM_001017858.2
B20G0673	30.23	22.19	CCL8	C-C motif chemokine 8	NM_001030173.1
A12G0403	30.22	14.84	BTN3A3	Butyrophilin subfamily 3 member A3	XM_009306726.4
A15G0687	30.22	14.02	AP2S1	AP-2 complex subunit sigma	NM_213155.2
A11G0662	30.20	53.22	NEK4	threonine-protein kinase Nek4	NM_001002073.2
A6G0815	30.19	7.94	PTGIS	Prostacyclin synthase	NM_001111160.1
A13G0798	30.19	16.70	MICU1	Calcium uptake protein 1, mitochondrial	NM_001003437.2
A25G0678	30.16	21.24	IGLC7	Immunoglobulin lambda constant 7	XM_068217596.1
B23G0365	30.14	8.55	DNASE1L3	Deoxyribonuclease gamma	NM_213153.2
B8G0624	30.13	8.24	TNFRSF14	Tumor necrosis factor receptor superfamily member 14	XM_068223135.1
B6G0879	30.05	36.45	CTSZ	Cathepsin Z	NM_001006043.1
B21G0190	30.03	6.68	KLF8	Krueppel-like factor 8	XM_068215909.1
A15G0011	30.02	27.87	GMFG	Glia maturation factor gamma	NM_001040047.1
A11G0056	30.02	31.26	CLDN34	Claudin-34	NR_155546.1
B2G1208	30.01	20.36	PIK3R1	Phosphatidylinositol 3-kinase regulatory subunit alpha	NM_001291329.1
B6G0913	30.00	13.70	EIF6	Eukaryotic translation initiation factor 6	NM_200944.1
B9G0283	29.98	18.52	G5714_009955	A0A7J6CT59_9TELE Ig-like domain-containing protein	
B23G0666	29.96	38.36	CHI3L1	Chitinase-3-like protein 1	
A16G1011	29.95	9.66	RRP15	RRP15-like protein	
A1G0682	29.94	7.93	NFKBIZ	NF-kappa-B inhibitor zeta	
B5G1184	29.93	24.60	NONO	Non-POU domain-containing octamer-binding protein	XM_005165105.5
A19G0938	29.91	26.61	DNAJC8	DnaJ homolog subfamily C member 8	NM_001045306.1
B25G0305	29.89	45.23	ETFA	Electron transfer flavoprotein subunit alpha, mitochondrial	NM_198909.1
B15G0245	29.86	9.66	RHOU	Rho-related GTP-binding protein RhoU	NM_001017784.3
B18G0200	29.85	23.18	TC1A	Transposable element Tc1 transposase	
A6G0440	29.85	5.26	GSN	Gelsolin	NM_178131.3
A16G0698	29.85	24.09	RABAC1	Prenylated Rab acceptor protein 1	NM_214780.1
B18G0778	29.84	16.81	CLNS1A	Methylosome subunit pICln	NM_131424.2
B22G0756	29.84	20.11	MAP2K2	Dual specificity mitogen-activated protein kinase kinase 2	NM_001037391.2
B1G0007	29.84	8.62	PRSS23	Serine protease 23	
A3G0001	29.83	12.42	DDX47	Probable ATP-dependent RNA helicase DDX47	NM_001020686.1
A17G0511	29.82	21.50	YIPF4	Protein YIPF4	NM_212891.1
A20G0419	29.81	28.11	WTAP	Pre-mRNA-splicing regulator WTAP	NM_199853.1
A19G0197	29.79	71.18	CSNK2B	Casein kinase II subunit beta	NM_131187.1
B16G0258	29.77	23.15	CASPA	Caspase a	NM_131505.2
B3G0218	29.76	49.05	MYH9	Myosin-9	XM_005163701.5
A19G0021	29.76	24.12	UTP18	U3 small nucleolar RNA-associated protein 18 homolog	

A2G0422	29.76	29.15	ARPC5-A	3 complex subunit 5-A	NM_194378.4
B12G0099	29.73	15.64	BCCIP	Protein BCCIP homolog	NM_001013475.2
A2G0715	29.72	45.92	DHX9	ATP-dependent RNA helicase A	NM_001201444.1
A18G0792	29.71	19.79	MYEF2	Myelin expression factor 2	NM_001030284.2
B14G0693	29.68	63.64	LYG	Lysozyme g	NM_001002706.1
B17G0674	29.67	30.87	ZFP36L1	mRNA decay activator protein ZFP36L1	NM_001077153.2
B11G0447	29.66	32.96	MBD1	Methyl-CpG-binding domain protein 1	NM_205608.1
B18G0391	29.65	71.56	TSPAN3	Tetraspanin-3	NM_001025538.2
B22G1175	29.64	15.46	LOC113044317	A0A6P6JKE6_CARAU uncharacterized protein LOC113044317 isoform X3	
A2G1113	29.64	2.16	IL6ST	Interleukin-6 receptor subunit beta	XM_068213294.1
B7G0203	29.61	9.93	FADD	FAS-associated death domain protein	
B24G0612	29.59	15.97	G5714_023602	A0A7J6BLP3_9TELE Uncharacterized protein	XM_021473748.2
B18G0842	29.57	42.21	BLOC1S6	Biogenesis of lysosome-related organelles complex 1 subunit 6	NM_001080697.2
B12G0367	29.57	17.16	CRIPT	Cysteine-rich PDZ-binding protein	NM_001017893.1
A16G0093	29.55	47.67	ETFB	Electron transfer flavoprotein subunit beta	NM_212998.2
B6G0376	29.54	28.05	RAE1	mRNA export factor	NM_200998.1
B2G1056	29.53	20.56	TXNDC12	Thioredoxin domain-containing protein 12	
A5G0778	29.53	59.93	VCP	Transitional endoplasmic reticulum ATPase	NM_201481.1
B8G0123	29.51	26.34	HNRNPK	Heterogeneous nuclear ribonucleoprotein K	NM_212994.2
A19G0219	29.50	13.32	RGS8	Regulator of G-protein signaling 8	
B12G0408	29.50	12.72	MRPL27	39S ribosomal protein L27, mitochondrial	
A7G0349	29.50	19.34	CCT7	T-complex protein 1 subunit eta	NM_173248.1
A21G0827	29.50	10.93	PDLIM4	PDZ and LIM domain protein 4	NM_001042696.2
A5G0366	29.49	15.40	CWC15	Protein CWC15 homolog	NM_001002050.2
A21G0856	29.48	28.11	VDAC1	Voltage-dependent anion-selective channel protein 1	NM_001001404.1
B5G1048	29.46	53.73	G5714_005913	A0A7J6D2C5_9TELE Uncharacterized protein	
A3G0994	29.45	40.00	COA3A	Cytochrome c oxidase assembly factor 3 homolog, mitochondrial	NM_001204250.2
B6G0858	29.44	15.28	LOC107596800	A0A672QKA4_SINGR Uncharacterized LOC107596800	NM_001386781.1
A19G0340	29.44	24.37	PSMA2	Proteasome subunit alpha type-2	NM_001128674.1
B16G0507	29.43	44.99	MCL1	Induced myeloid leukemia cell differentiation protein Mcl-1 homolog	NM_194394.2
A1G0811	29.42	15.36	ARGLU1B	Arginine and glutamate-rich protein 1-B	NM_200162.1
B17G0048	29.41	16.43	SPINT1	Kunitz-type protease inhibitor 1	NM_213152.1
B21G0478	29.38	24.04	RCSD1	CapZ-interacting protein	NM_001045422.1
A18G0106	29.37	20.17	PSMD8	26S proteasome non-ATPase regulatory subunit 8	NM_001002131.2
B10G0816	29.37	9.24	MYO3B	Myosin-IIIb	XM_679943.8
B13G0008	29.36	18.08	PLSCR1	Phospholipid scramblase 1	NM_001327785.1
A20G0803	29.36	5.48	CA131	Uncharacterized protein C1orf131 homolog	NM_001130794.1

B2G0883	29.34	17.84	IVNS1ABPB	Influenza virus NS1A-binding protein homolog B	NM_201483.1
B21G0466	29.34	15.59	ANAPC13	Anaphase-promoting complex subunit 13	NM_001302785.1
A7G0834	29.34	14.39	MBLAC1	Metallo-beta-lactamase domain-containing protein 1	
A6G0847	29.34	10.66	SLC25A22	Mitochondrial glutamate carrier 1	XM_017356461.2
A7G0486	29.34	18.32	TP53INP1	Tumor protein p53-inducible nuclear protein 1	NM_001386724.1
A5G0612	29.33	24.72	C9ORF78	Telomere length and silencing protein 1 homolog	NM_001005945.1
B15G0179	29.33	30.95	CHCHD2	Coiled-coil-helix-coiled-coil-helix domain-containing protein 2	NM_200767.1
B10G0228	29.32	13.19	DRG1	Developmentally-regulated GTP-binding protein 1	NM_200038.1
A18G0113	29.29	40.59	APLP2	Amyloid beta precursor like protein 2	XM_005159251.5
B21G0215	29.28	12.66	MRPL22	39S ribosomal protein L22, mitochondrial	NM_001122621.1
B20G0012	29.28	15.71	RPF2	Ribosome production factor 2 homolog	NM_214748.1
A11G0667	29.24	2.00	RPN1	Dolichyl-diphosphooligosaccharide--protein glycosyltransferase subunit 1	XM_684926.10
A12G0470	29.24	7.18	RSL1D1	Ribosomal L1 domain-containing protein 1	
B14G0374	29.23	10.75	EIF4E1A	Eukaryotic translation initiation factor 4E-1A	NM_131733.1
B5G0141	29.21	16.50	KCNV2	Potassium voltage-gated channel subfamily V member 2	NM_001128740.1
A18G0321	29.21	87.15			XM_005173642.5
B20G0416	29.20	17.17	GTF2H5	General transcription factor IIH subunit 5	NM_001134362.2
A11G0277	29.19	21.55	PRICKLE2	Prickle-like protein 2	NM_001083573.1
B8G0669	29.19	14.57	MOB3A	MOB kinase activator 3A	NM_199716.1
A5G0564	29.19	14.55	DPM2	Dolichol phosphate-mannose biosynthesis regulatory protein	NM_001122846.2
A17G0910	29.17	10.20	NEMF	Ribosome quality control complex subunit NEMF	
B12G0013	29.16	24.55	LHPP	Phospholysine phosphohistidine inorganic pyrophosphate phosphatase	NM_001098781.1
B25G0815	29.14	65.33	METAP2	Methionine aminopeptidase 2	NM_199640.1
B9G0620	29.14	52.50	CXCR4-B	C-X-C chemokine receptor type 4-B	NM_131834.1
B21G0499	29.10	11.10	IL7R	Interleukin-7 receptor subunit alpha	NM_001113507.2
B7G0721	29.10	9.31	MPHOSPH10	U3 small nucleolar ribonucleoprotein protein MPP10	NM_001305543.1
B6G0374	29.09	50.42	DDX17	Probable ATP-dependent RNA helicase DDX17	NM_001326391.1
B14G0118	29.09	24.47	LMAN2	Vesicular integral-membrane protein VIP36	NM_001081747.2
A3G0283	29.08	17.20	TUFM	Elongation factor Tu, mitochondrial	NM_001013505.2
B7G0710	29.08	15.97	EIF3JB	Eukaryotic translation initiation factor 3 subunit J-B	NM_213502.1

B19G0519	29.07	21.04	PSMB4	Proteasome subunit beta type-4 (Fragment)	NM_001017801.1
B25G0237	29.05	12.13	NAP1L4	Nucleosome assembly protein 1-like 4	XM_005170675.5
B3G0680	29.04	42.49	PRKAR1A	cAMP-dependent protein kinase type I-alpha regulatory subunit	NM_001009989.3
A25G0381	29.03	3.15	LOC113093989	A0A6P6P321_CARAU uncharacterized protein LOC113093989	
A19G0521	29.03	91.70	FUCL4	Fucolectin-4	
B6G0475	29.01	12.87	BOKA	Bcl-2-related ovarian killer protein homolog A	NM_001003612.2
B13G0283	28.99	16.93	DNAAF9	Dynein axonemal assembly factor 9	NM_001128739.1
B1G0616	28.98	9.06	SOD3	Extracellular superoxide dismutase [Cu-Zn]	XM_068221511.1
A21G0555	28.98	26.48	SLC3A2	4F2 cell-surface antigen heavy chain	NM_201514.2
B17G0234	28.97	29.35	PNN	Pinin	NM_001172570.1
A6G0341	28.96	15.84	RHEB	GTP-binding protein Rheb	NM_001076748.2
B6G0757	28.96	16.40	WDR82	WD repeat-containing protein 82	NM_001166268.1
B14G0552	28.95	3.56	LOC107660157	A0A671MKE9_9TELE Eukaryotic translation initiation factor 1A domain-containing protein	
A22G0215	28.94	12.96	PDHB	Pyruvate dehydrogenase E1 component subunit beta, mitochondrial	NM_213154.1
A13G0813	28.92	11.69	POL	RNA-directed DNA polymerase from mobile element jockey	NM_178303.3
B19G0067	28.90	15.39	SNRPD1	Small nuclear ribonucleoprotein Sm D1	NM_173252.2
A20G0404	28.89	26.18	CD164	Sialomucin core protein 24	
A3G1211	28.89	16.78	UQCRC2	Cytochrome b-c1 complex subunit 2, mitochondrial	NM_001002657.2
A10G0788	28.87	15.65	PUM3	Pumilio homolog 3	NM_001353848.1
A2G0074	28.86	27.01	HIGD1A	HIG1 domain family member 1A, mitochondrial	NM_200100.2
A1G0020	28.85	9.40	MVB12A	Multivesicular body subunit 12A	NM_200484.1
A10G0874	28.83	29.67	PPP2CB	threonine-protein phosphatase 2A catalytic subunit beta isoform	NM_213293.1
A13G0852	28.83	10.66	A0A673JH75	A0A673JH75_9TELE Si:ch73-52p7.1	
B25G0420	28.82	19.33	GNAI1	Guanine nucleotide-binding protein G(i) subunit alpha-1	NM_198805.2
B10G0022	28.80	19.84	HINT1	Adenosine 5'-monophosphoramidase HINT1	NM_001005593.2
A15G0414	28.79	73.81	HIF1A	Hypoxia-inducible factor 1-alpha	XM_005157572.5
A25G0408	28.78	16.71	CSK	Tyrosine-protein kinase CSK	NM_001077599.1
A16G0288	28.77	9.07	ENY2	Transcription and mRNA export factor ENY2	NM_001002427.2
B1G0377	28.76	14.58	GTF2E2	General transcription factor IIE subunit 2	NM_212731.1
A4G0971	28.75	9.34	YEATS4	YEATS domain-containing protein 4	NM_001002752.2
A23G0546	28.75	8.02			XM_021469994.2
A9G0297	28.75	32.14	DDX3Y	ATP-dependent RNA helicase DDX3Y	XM_021478733.2

B3G1655	28.73	38.31	HLA-DPA1	HLA class II histocompatibility antigen, DP alpha 1 chain	
A23G0840	28.73	30.34	ZGC:65873	UPF0690 protein C1orf52 homolog	NM_200542.2
B24G0516	28.71	21.58	COMMD3	COMM domain-containing protein 3	NM_001008734.2
A6G0352	28.70	48.21	DDX17	Probable ATP-dependent RNA helicase DDX17	NM_001326391.1
B21G0015	28.69	39.31	DDX46	Probable ATP-dependent RNA helicase DDX46	XM_009295737.4
B3G0905	28.68	42.11	LIMD2	LIM domain-containing protein 2	NM_001328061.1
A8G0736	28.65	25.41	HLA-DPA1	HLA class II histocompatibility antigen, DP alpha 1 chain	NM_001007205.2
A13G0040	28.65	30.32	MYPN	Myopalladin	NM_001127335.2
B2G0382	28.64	12.32	MNCB-2990	Uncharacterized protein C14orf119 homolog	NM_001244794.1
A13G0456	28.64	12.13	CHUK	Inhibitor of nuclear factor kappa-B kinase subunit alpha	NM_001007372.1
B1G0011	28.61	16.19	GPA33	Cell surface A33 antigen	NM_001115105.2
B25G0827	28.60	15.88	SCAMP2	Secretory carrier-associated membrane protein 2	NM_213378.2
B12G0320	28.59	10.26	PSME3	Proteasome activator complex subunit 3	NM_131376.2
B1G0893	28.57	10.11	CCDC86	Coiled-coil domain-containing protein 86	NM_001145359.1
A4G0593	28.56	65.10	RAP1B	Ras-related protein Rap-1b	NM_199533.1
B14G0407	28.56	39.06	SLU7	Pre-mRNA-splicing factor SLU7	NM_001007367.1
A15G0787	28.56	11.38	ATP1B3	potassium-transporting ATPase subunit beta-3	NM_131670.1
B23G0176	28.55	25.29	TTN	Titin	XM_021469889.2
B9G0576	28.54	0.00			XM_068223346.1
B4G0711	28.53	19.87	SCGN	Secretagogin	NM_001005776.1
B3G0699	28.53	9.74	NDUFAB1	Acyl carrier protein, mitochondrial	NM_001305570.1
A18G0714	28.52	25.19	TC1A	Transposable element Tc1 transposase	
B2G0313	28.52	3.53	TRIM16	Tripartite motif-containing protein 16	NM_001045270.2
B21G0120	28.50	6.39	IL4/13A	13A PE=2 SV=1	
A20G0921	28.49	20.53	SMIM8	Small integral membrane protein 8	
A3G1155	28.48	22.41	AIMP2	Aminoacyl tRNA synthase complex-interacting multifunctional protein 2	NM_213338.1
B9G0199	28.46	30.93	COL5A2	Collagen alpha-2(V) chain	NM_001145782.1
B14G0241	28.46	7.27	GDPD2	Glycerophosphoinositol inositolphosphodiesterase GDPD2	XM_021472855.2
A17G0089	28.44	11.21	FCF1	rRNA-processing protein FCF1 homolog	NM_001017601.1
A19G0727	28.43	2.43	RAB25	Ras-related protein Rab-25	NM_001008641.2
A12G0171	28.42	12.23	CHMP6	Charged multivesicular body protein 6	NM_001003874.1
A15G0625	28.37	6.66	RRP8	Ribosomal RNA-processing protein 8	XM_021472910.2
B1G0041	28.35	2.90	C3AR1	C3a anaphylatoxin chemotactic receptor	
B2G1206	28.35	17.38	GUK1	Guanylate kinase	NM_001316896.1
B16G0931	28.33	12.43	NOP2	Probable 28S rRNA (cytosine-C(5))-methyltransferase	NM_001301359.1

A10G0476	28.31	12.10	PTRH2	Peptidyl-tRNA hydrolase 2, mitochondrial	NM_001020535.2
B21G0284	28.30	7.58	SLC35A4	SLC35A4 upstream open reading frame protein	
B21G0062	28.29	32.73	VDAC1	Voltage-dependent anion-selective channel protein 1	NM_001001404.1
B16G0796	28.29	42.42	COX6B1	Cytochrome c oxidase subunit 6B1	NM_200506.3
A3G0145	28.28	18.91	DDX39A	ATP-dependent RNA helicase DDX39A	NM_212977.2
B7G0507	28.28	12.61	PLXDC2	Plexin domain-containing protein 2	NM_001146305.2
A25G0404	28.28	0.59	KCTD15L	POZ domain-containing protein kctd15-like	XM_005163052.3
A23G0143	28.26	24.00	CSRP1	Cysteine and glycine-rich protein 1	NM_205567.1
A2G0599	28.25	8.81	CNPY1	Protein canopy-1	NM_001039497.2
A17G0228	28.23	4.71	CRIP1	Cysteine-rich protein 1	NM_001166582.2
B1G0063	28.22	66.39	ATP1A1	potassium-transporting ATPase subunit alpha-1	NM_131689.1
A19G0490	28.21	32.50	VPS28	Vacuolar protein sorting-associated protein 28 homolog	NM_200590.1
B18G0173	28.19	28.77	GNB5B	Guanine nucleotide-binding protein subunit beta-5b	NM_200746.1
A4G0640	28.19	43.83	PAH	Phenylalanine-4-hydroxylase	NM_200551.1
B10G0191	28.18	11.51	SUB1	Activated RNA polymerase II transcriptional coactivator p15	NM_001020540.3
B13G0208	28.17	12.00	CKB	Creatine kinase B-type	NM_001033106.1
A23G0660	28.16	9.33	ACP1	Low molecular weight phosphotyrosine protein phosphatase	NM_001014302.2
B12G0330	28.16	27.59	CHMP2A	Charged multivesicular body protein 2a	NM_213517.1
B13G0919	28.15	31.07			
B1G0120	28.14	32.11	ARGLU1B	Arginine and glutamate-rich protein 1-B	NM_200162.1
A5G0609	28.14	14.94	SURF1	Surfeit locus protein 1	NM_001080173.1
B13G0053	28.13	19.26	DEGS1	Sphingolipid delta(4)-desaturase DES1	NM_199981.1
B19G0217	28.12	12.86	MRPL9	39S ribosomal protein L9, mitochondrial	NM_001100044.2
B17G0792	28.11	6.27	G5714_004337	A0A7J6D568_9TELE Uncharacterized protein	
B11G0329	28.11	31.44	ZMYND8	Protein kinase C-binding protein 1	NM_199686.2
A16G0467	28.11	50.93	CTSS	Cathepsin S	NM_001024409.2
B2G1143	28.10	21.02	LEPROT	Leptin receptor gene-related protein	NM_001017787.1
B12G0541	28.09	14.83	PCGF5	Polycomb group RING finger protein 5	NM_001045235.1
B20G0928	28.08	32.05	PRPF39	Pre-mRNA-processing factor 39	NM_001004520.1
B9G0035	28.08	17.62	LOC113109178	A0A6P6Q954_CARAU NACHT, LRR and PYD domains-containing protein 12-like isoform X3	XM_068215350.1
A7G0740	28.05	17.33	SERF2	Small EDRK-rich factor 2	NM_001160335.2
B17G0001	28.04	26.25	VWA5A	von Willebrand factor A domain-containing protein 5A	XM_068217480.1
A16G0591	28.03	9.81	A0A673JIC7	A0A673JIC7_9TELE DNAX-activation protein 10	

A1G0540	28.02	8.47	GALNT7	N-acetylgalactosaminyltransferase 7	NM_001020641.1
A14G0456	28.02	83.40	TNIP1	TNFAIP3-interacting protein 1	NM_001079952.1
B17G0760	28.01	16.69	CHMP3	Charged multivesicular body protein 3	NM_213320.1
A18G0153	27.99	10.95	VASP	Vasodilator-stimulated phosphoprotein	XM_005173679.5
B8G1045	27.98	3.10	SRP19	Signal recognition particle 19 kDa protein	
B19G0667	27.97	30.46	TPD52	Tumor protein D52	XM_017352246.3
B18G0331	27.97	8.92	G5714_017932	A0A7J6C2N9_9TELE Uncharacterized protein	XM_068214925.1
B8G0870	27.97	8.86			
B21G1003	27.96	26.44	BRIX1	Ribosome biogenesis protein BRX1 homolog	NM_001172556.1
A19G0102	27.95	31.36	STX12	Syntaxin-12	NM_001002051.1
A16G0860	27.94	9.71	NOP2	Probable 28S rRNA (cytosine(4447)-C(5))-methyltransferase	NM_001301359.1
A25G0010	27.91	28.75	CIAO2A	Cytosolic iron-sulfur assembly component 2A	NM_213027.1
B1G0650	27.90	18.72	LRPAP1	Alpha-2-macroglobulin receptor-associated protein	NM_201306.2
A10G0278	27.88	25.34	STT3A	Dolichyl-diphosphooligosaccharide--protein glycosyltransferase subunit STT3A	NM_201458.2
A2G0663	27.86	13.68	UBTF-B	Nucleolar transcription factor 1-B	XM_068223429.1
B11G0766	27.85	22.53	ASPSCR1	Tether containing UBX domain for GLUT4	XM_021479605.2
B17G0583	27.84	8.28	ITPKA	Inositol-trisphosphate 3-kinase A	NM_001326476.1
B16G0827	27.84	23.56	ATG12	Ubiquitin-like protein ATG12	NM_001246200.1
B4G0003	27.83	12.16	PHTF2	Protein PHTF2	NM_001002305.1
B3G1364	27.80	27.81	ALYREF	THO complex subunit 4	NM_001105108.1
B21G0400	27.79	29.62	SLC3A2	4F2 cell-surface antigen heavy chain	NM_201514.2
A18G0699	27.78	7.47	NIP7	60S ribosome subunit biogenesis protein NIP7 homolog	NM_001020590.1
B1G0936	27.78	5.98	LTV1	Protein LTV1 homolog	XM_021470934.2
B12G0136	27.76	59.57	PMP22	Peripheral myelin protein 22	
B9G0848	27.72	13.51	ATP5MC3	ATP synthase F(0) complex subunit C3, mitochondrial	NM_201176.1
A12G0556	27.71	24.77	HEXIM1	Protein HEXIM1	NM_001098389.1
B6G0888	27.71	18.55	EIF2S2	Eukaryotic translation initiation factor 2 subunit 2	NM_212675.1
A21G0056	27.70	61.31	CR032	UPF0729 protein C18orf32 homolog	
B19G0827	27.68	40.56	SEPTIN7	Septin-7	XM_068215054.1
A8G0721	27.67	25.16	NPEPL1	Probable aminopeptidase NPEPL1	NM_001076620.1
A8G0620	27.63	2.11	VGLL4	Transcription cofactor vestigial-like protein 4	
B22G0523	27.62	14.21	SHISA5	Protein shisa-5	
A19G0248	27.56	14.48	MRPS18B	28S ribosomal protein S18b, mitochondrial	NM_001017759.2
A8G0369	27.53	30.54	MKNK2	threonine-protein kinase 2	NM_194402.1
B6G0885	27.52	14.07			
B20G0772	27.51	32.97	GPCPD1	Glycerophosphocholine phosphodiesterase GPCPD1	NM_001110523.1

B9G0039	27.50	12.89	LOC113109221	A0A6P6Q9B1_CARAU NACHT, LRR and PYD domains-containing protein 12-like	XM_021468229.2
A3G0705	27.49	28.95	NOSIP	Nitric oxide synthase-interacting protein	NM_001007434.1
B16G0192	27.49	16.41	SYNDIG1L	Synapse differentiation-inducing gene protein 1-like	NM_001386350.1
B10G0018	27.49	40.40	ALDH7A1	Alpha-aminoadipic semialdehyde dehydrogenase	NM_212724.2
B17G0639	27.48	36.93	ID2	DNA-binding protein inhibitor ID-2	NM_201291.1
B4G0011	27.47	21.97	LOC113086299	A0A6P6NS05_CARAU collagen alpha-1(II) chain-like isoform X2	
A1G0022	27.47	21.80	AKAP8L	A-kinase anchor protein 8-like	XM_017352826.3
B19G0964	27.47	11.58	UBE2M	NEDD8-conjugating enzyme Ubc12	XM_001920635.9
A3G0915	27.46	16.02	JPT2	Jupiter microtubule associated homolog 2	NM_199575.2
A1G0143	27.45	13.45	DENND1B	DENN domain-containing protein 1B	XM_005168402.5
B2G0238	27.45	1.62	NLRP1	NACHT, LRR and PYD domains-containing protein 1 homolog	XM_068213264.1
A13G0546	27.43	12.49	ELOVL6	Elongation of very long chain fatty acids protein 6	NM_001017833.1
A9G0590	27.43	33.93	CRYBA2	Beta-crystallin A2	XM_005167477.3
B9G0336	27.42	8.12	LADD	Ladderlectin	
A12G0796	27.41	15.91	DCXR	L-xylulose reductase	NM_001017619.1
A1G0453	27.40	4.29	CTNND1	Catenin delta-1	XM_068221592.1
A6G0716	27.40	43.28	ARPC4	3 complex subunit 4	NM_205537.1
A2G0518	27.39	13.49	DPX16_18405	A0A3N0YF14_ANAGA Uncharacterized protein	
B17G0476	27.39	6.83	RTN4IP1	Reticulon-4-interacting protein 1 homolog, mitochondrial	NM_200352.2
A4G0079	27.38	19.76	LOC113048991	A0A6P6K6T2_CARAU uncharacterized protein LOC113048991 isoform X2	
A22G0325	27.38	9.75	MRPL54	39S ribosomal protein L54, mitochondrial	
B16G0129	27.37	32.52	RAD21A	Double-strand-break repair protein rad21 homolog A	NM_199595.1
B10G0079	27.37	34.78	CST3	Cystatin-C	
A14G0125	27.33	15.97	TMED9	Transmembrane emp24 domain-containing protein 9	XM_005173178.4
A25G0786	27.29	22.02	CHMP1A	Charged multivesicular body protein 1a	NM_200563.3
A5G0291	27.28	24.23	ISCU	Iron-sulfur cluster assembly enzyme ISCU, mitochondrial	NM_001003632.2
B3G1119	27.28	26.99	MIEN1	Migration and invasion enhancer 1	NM_194417.3
A16G0763	27.27	16.77	CDC42EP5	Cdc42 effector protein 5	NM_001326377.1
B2G1072	27.27	32.68	FUBP1	Far upstream element-binding protein 1	XM_009297514.4
A21G0302	27.26	21.42	VPS29	Vacuolar protein sorting-associated protein 29	NM_200037.2
B23G0126	27.26	15.46	CDH26	Cadherin-like protein 26	NM_001423486.1
B5G0063	27.26	20.69	SUPT4H1	Transcription elongation factor SPT4	NM_001002509.1
A21G0188	27.26	12.79	SDAD1	Protein SDA1 homolog	NM_173230.1

B14G0003	27.26	65.19	COPS6	COP9 signalosome complex subunit 6	NM_001017768.1
A4G0980	27.23	8.58	UBE2N	Ubiquitin-conjugating enzyme E2 N	NM_200342.1
B23G0392	27.20	27.37	DPM1	Dolichol-phosphate mannosyltransferase subunit 1	NM_001003596.1
A14G0426	27.20	16.04	QDPR	Dihydropteridine reductase	NM_001110469.1
B22G0011	27.18	31.99	SSU72	RNA polymerase II subunit A C-terminal domain phosphatase SSU72	NM_200728.1
B5G0507	27.18	14.36	SURF4	Surfeit locus protein 4	NM_001002470.2
A3G0498	27.17	14.08	FSCN1	Fascin	NM_001076560.1
A3G0399	27.15	7.51	S1PR2	Sphingosine 1-phosphate receptor 2	NM_001159970.1
B20G0434	27.14	33.00	SERPINB1	Leukocyte elastase inhibitor	NM_001045171.2
A7G0245	27.12	6.19	TPCN2	Two pore channel protein 2	NM_001077722.2
A16G0420	27.11	31.45	PTPN6	Tyrosine-protein phosphatase non-receptor type 6	NM_199960.1
B5G0365	27.10	2.60	ANXA1	Annexin A1	NM_001017605.1
A17G0473	27.09	12.39	MDH1	Malate dehydrogenase, cytoplasmic	NM_001355555.1
A23G0333	27.09	13.06	SMIM4	Small integral membrane protein 4	
A17G0098	27.07	31.60	FOSL2	Fos-related antigen 2	NM_001082998.2
A19G0140	27.06	16.21	SEPTIN7	Septin-7	XM_068215054.1
A23G0844	27.05	27.38	SYAP1	Synapse-associated protein 1	NM_200942.1
B6G0957	27.04	22.12	SNRPC	U1 small nuclear ribonucleoprotein C	NM_173251.2
A12G0183	27.04	13.22	TIMM23	Mitochondrial import inner membrane translocase subunit Tim23	NM_001105598.1
A17G0035	27.02	23.09	SYNCRIP	Heterogeneous nuclear ribonucleoprotein Q	XM_009293300.4
B1G0413	27.00	8.54	ARFIP1	Arfaptin-1	XM_005170891.5
B6G0996	27.00	18.86	SNRPN	Small nuclear ribonucleoprotein-associated protein N	NM_205667.1
A21G0800	26.99	15.00	YIPF5	Protein YIPF5	NM_200295.1
A12G0656	26.99	15.10	SMARCD2	SNF-related matrix-associated actin-dependent regulator of chromatin subfamily D member 2	XM_687657.7
A10G0317	26.98	18.41	UCP2	Mitochondrial uncoupling protein 2	NM_131176.1
A8G0009	26.97	30.89	POL	LINE-1 retrotransposable element ORF2 protein	
A14G0498	26.95	10.36	ATP6V0E1	V-type proton ATPase subunit e 1	
A24G0238	26.95	8.13	TLCD4-B	TLC domain-containing protein 4-B	XM_695466.9
A12G0784	26.94	56.46	COA3A	Cytochrome c oxidase assembly factor 3 homolog, mitochondrial	NM_001114916.2
A19G0801	26.93	6.69	CEACAM5	Carcinoembryonic antigen-related cell adhesion molecule 5	
A8G0717	26.91	85.19	DUSP2	Dual specificity protein phosphatase 2	NM_001003451.1
B9G0363	26.90	84.47	SF3B1	Splicing factor 3B subunit 1	XM_679219.6
B16G0034	26.89	15.53	CMC1	COX assembly mitochondrial protein homolog	
A21G0461	26.87	35.41	BIRC2	Baculoviral IAP repeat-containing protein 2	NM_194395.4

A13G0797	26.86	7.83	NOLC1	Nucleolar and coiled-body phosphoprotein 1	NM_001362344.1
B25G0390	26.86	17.95	UBE2Q2	Ubiquitin-conjugating enzyme E2 Q2	NM_001328199.1
B17G0298	26.86	7.55	SPTLC2	Serine palmitoyltransferase 2	NM_001020619.2
B2G0886	26.85	15.22	PTGS2	H synthase 2	NM_153657.1
B6G0614	26.85	42.17	LOC113101395	A0A6P6PL10_CARAU Zinc finger Ran-binding domain-containing protein 2	
B23G0502	26.83	10.75	ADA	Adenosine deaminase	NM_001002646.1
B16G0698	26.83	17.11	KRTCAP2	Keratinocyte-associated protein 2	NM_001045362.1
A17G0686	26.82	18.89	SCCPDH	Saccharopine dehydrogenase-like oxidoreductase	NM_001114436.2
B6G0419	26.81	13.16	SEPTIN9	Septin-9	NM_001327975.1
A12G0454	26.80	10.03	PHF5A	PHD finger-like domain-containing protein 5A	NM_173266.1
A22G0647	26.79	16.13	MANF	Mesencephalic astrocyte-derived neurotrophic factor	NM_001076629.1
A10G0827	26.76	6.72	LYRM7	Complex III assembly factor LYRM7	
B19G0530	26.75	19.58	LOC107706172	A0A673HMS6_9TELE Uncharacterized LOC107706172	NM_001430907.1
B25G0203	26.75	13.42	KV3A9	Ig kappa chain V-III region MOPC 63	XM_068217596.1
B13G0715	26.74	34.31	PPP2R5E	threonine-protein phosphatase 2A 56 kDa regulatory subunit epsilon isoform	
A7G0214	26.74	6.13	LOC107552988	A0A672P006_SINGR Membrane-spanning 4-domains subfamily A member 10-like	
B14G0599	26.73	36.42	TC1A	Transposable element Tc1 transposase	XM_068220572.1
A16G1026	26.73	28.70	DCTN3	Dynactin subunit 3	
B5G0024	26.72	10.81	SURF2	Surfeit locus protein 2	
A1G0332	26.72	10.98	RNF122	RING finger protein 122	NM_001114727.2
B5G1237	26.71	13.69			
B10G0281	26.71	22.82	ETF1	Eukaryotic peptide chain release factor subunit 1	XM_021479101.2
A20G0313	26.70	9.89	SRP9	Signal recognition particle 9 kDa protein	
A10G0703	26.70	46.91	THAP6	THAP domain-containing protein 6	
B13G0114	26.69	25.77	PRR18	Proline-rich protein 18	NM_213128.1
B17G0509	26.68	19.66	RPS6KA	Ribosomal protein S6 kinase 2 alpha	XM_068214461.1
B20G0317	26.67	36.83	HIF1A	Hypoxia-inducible factor 1-alpha	NM_001310042.1
A13G0547	26.64	28.81	MIXL1	Homeobox protein MIXL1	NM_212803.1
B24G0065	26.62	21.66	IGLC3	Ig lambda-3 chain C region	XM_068217596.1
B2G0610	26.61	17.34	PUF60	Poly(U)-binding-splicing factor PUF60	XM_005163501.5
A3G0222	26.60	25.42	CYP2K1	Cytochrome P450 2K1	XM_005163733.5
B3G1091	26.57	29.36	NUPR1	Nuclear protein 1	
B3G0024	26.55	7.05	NOL12	Nucleolar protein 12	NM_200290.1
A20G0374	26.54	20.95	PHPT1	14 kDa phosphohistidine phosphatase	NM_001030119.2
A19G0037	26.52	11.67	DDAH2	N(G),N(G)-dimethylarginine dimethylaminohydrolase 2	NM_001100065.2
B17G0332	26.48	22.98	SMNDC1	Survival of motor neuron-related-splicing factor 30	NM_212601.2

A12G0575	26.47	22.83	BECN1	Beclin-1	NM_200872.1
B12G0044	26.44	19.48	ANAPC16	Anaphase-promoting complex subunit 16	NM_001017878.2
B22G0628	26.43	3.01	GPC1	Glypican-1	NM_001037373.2
A23G0702	26.43	34.65	TSPAN31	Tetraspanin-31	NM_200281.1
A18G0427	26.43	24.45	GOT2A	Aspartate aminotransferase, mitochondrial	NM_213379.1
A18G0755	26.43	18.30	SS18L2	SS18-like protein 2	
B7G1070	26.42	29.56	RAB1A	Ras-related protein Rab-1A	NM_201142.1
B15G0180	26.42	31.48	HNRNPL	Heterogeneous nuclear ribonucleoprotein L	NM_001423736.1
B18G0254	26.42	23.87	CYBA	Cytochrome b-245 light chain	NM_200579.1
A11G0537	26.40	15.11	ITPR1	Inositol 1,4,5-trisphosphate receptor type 1	NM_200716.1
B3G1407	26.40	15.11	SYNGR2	Synaptogyrin-2	NM_001320463.1
B15G0596	26.40	36.28	CAPNS1	Calpain small subunit 1	NM_001109706.1
A3G0578	26.39	29.94	GSPT1	Eukaryotic peptide chain release factor GTP-binding subunit ERF3A	NM_198806.1
A19G0716	26.39	26.21	S100A1	Protein S100-A1	
B15G0845	26.38	23.03	CAPN9	Calpain-9	XM_068213501.1
A6G0828	26.38	9.32	PADI2	Protein-arginine deiminase type-2	XM_005172097.5
B21G0091	26.37	49.30	ROHU_036320	A0A498N6H3_LABRO Uncharacterized protein	
B13G0290	26.35	13.43	SI:CH211-233A24.2	Transmembrane protein KIAA1109 homolog	XM_005156958.5
A20G0109	26.35	4.74	NAA20	N-alpha-acetyltransferase 20	NM_001014329.2
A13G0163	26.33	15.69	MARVELD1	MARVEL domain-containing protein 1	
A5G0332	26.32	8.92	TNFSF10	Tumor necrosis factor ligand superfamily member 10	NM_001013283.2
A6G0406	26.32	13.03	UTP6	U3 small nucleolar RNA-associated protein 6 homolog	NM_001111207.1
A20G0881	26.31	52.59	PCMT	Protein-L-isoaspartate(D-aspartate) O-methyltransferase	NM_131465.3
A18G0084	26.30	52.66	ABCC5	ATP-binding cassette sub-family C member 5	XM_017351905.3
A22G0498	26.28	29.50	PTPRC	Receptor-type tyrosine-protein phosphatase C	
A13G0661	26.26	9.88	POMK	Protein O-mannose kinase	NM_212992.2
A21G0888	26.26	17.88	LARP1	La-related protein 1	XM_001920867.9
B21G0149	26.25	156.18	ALDOC	Fructose-bisphosphate aldolase C	NM_194384.1
B14G0206	26.24	55.41	FHL1	Four and a half LIM domains protein 1	XM_009291101.4
B11G0331	26.24	25.13	PFKFB2	fructose-2,6-bisphosphatase 2	NM_212869.1
A5G1316	26.23	30.35	SPAG7	Sperm-associated antigen 7 homolog	NM_201125.1
B11G0468	26.23	25.97	DYNLRB1	Dynein light chain roadblock-type 1	NM_001313772.1
B7G0250	26.20	18.38	APRT	Adenine phosphoribosyltransferase	
A7G0251	26.20	12.94	BAX	Apoptosis regulator BAX	NM_001076585.1
A5G1018	26.20	21.77	POLR2J	DNA-directed RNA polymerase II subunit RPB11	
B4G0389	26.20	33.62	PSMC2	26S proteasome regulatory subunit 7	NM_200966.1
B20G0588	26.19	3.27	PTGS2	H synthase 2	NM_001025504.2
A5G0429	26.18	23.05	ZFAND5	AN1-type zinc finger protein 5	XM_021476057.2
A1G0402	26.18	21.22	DDX39A	ATP-dependent RNA helicase DDX39A	NM_001328342.1

A14G0727	26.16	11.45	PRPF19	Pre-mRNA-processing factor 19	NM_201467.1
A21G0448	26.16	42.17	A0A672LSS5	A0A672LSS5_SINGR Grass carp reovirus (GCRV)-induced gene 21	NM_001245989.1
B13G0496	26.15	19.71	CGAS	Cyclic GMP-AMP synthase	NM_001002478.3
A15G0366	26.14	17.46	EML1	Echinoderm microtubule-associated protein-like 1	NM_001030235.1
B16G1050	26.12	12.50	SRI	Sorcin	NM_200373.1
A24G0487	26.12	5.37	NGDN	Neuroguidin	NM_198373.2
A4G1015	26.12	21.95	RTCB	RNA-splicing ligase RtcB homolog	NM_213103.1
B7G0810	26.12	30.98	NUCB2	Nucleobindin-2	NM_201493.1
A12G0074	26.11	20.10	VCL	Vinculin	NM_001317752.1
B7G1058	26.11	60.44	RTN3	Reticulon-3	XM_021477447.2
A21G0859	26.10	16.14	SKP1	S-phase kinase-associated protein 1	NM_200743.1
B21G0006	26.10	28.96	IRF1	Interferon regulatory factor 1	NM_205747.2
B3G1297	26.10	46.03	NDUFB10	NADH dehydrogenase [ubiquinone] 1 beta subcomplex subunit 10	NM_200730.1
A6G0355	26.10	53.07	UQCRC1	Cytochrome b-c1 complex subunit 1, mitochondrial	NM_200820.1
A8G0446	26.08	16.78	MAPRE1	EB family member 1	NM_199864.2
A5G0739	26.08	42.21	A0A673FSX0	A0A673FSX0_9TELE Si:dkey-58f10.14	
B20G0490	26.07	27.76	EMC7	ER membrane protein complex subunit 7	NM_001025515.2
A4G0414	26.06	2.48	SCGN	Secretagogin	NM_001005776.1
B5G0158	26.05	7.57	IER3	Radiation-inducible immediate-early gene IEX-1	NM_001080038.1
A21G0387	26.04	19.91	LOC113064245	A0A6P6M1Y4_CARAU uncharacterized protein LOC113064245	NR_131904.1
A7G0969	26.03	13.21	ROHU_024072	A0A498MNW3_LABRO Transmembrane 4 L6 family member 5-like protein	NR_125779.1
B10G0210	26.00	12.46	STOML2	Stomatin-like protein 2, mitochondrial	NM_201031.1
A23G0776	26.00	14.44	PFDN4	Prefoldin subunit 4	NM_001386509.1
B10G0093	25.99	6.09	G5714_010694	A0A7J6CMY6_9TELE EGF-like domain-containing protein	
A13G0368	25.98	12.66	ACOD	Acyl-CoA desaturase	
B21G0302	25.98	14.12	CLK4	Dual specificity protein kinase CLK4	NM_001329908.1
A5G1233	25.98	18.61	EXOSC2	Exosome complex component RRP4	NM_001017572.2
A20G0711	25.97	9.34	ITPA	Inosine triphosphate pyrophosphatase	NM_001099986.2
A3G1132	25.97	29.67	IGLL1	Immunoglobulin lambda-like polypeptide 1	XM_021474765.2
B14G0349	25.95	22.73	ELF1	ETS-related transcription factor Elf-1	NM_131159.2
B1G0040	25.94	6.47	CENPQ	Centromere protein Q	NM_001328008.1
B6G0066	25.93	37.00	MYL9	Myosin regulatory light polypeptide 9	NM_213212.1
A24G0576	25.92	17.82	BLOC1S5	Biogenesis of lysosome-related organelles complex 1 subunit 5	NM_001030066.2
B19G0179	25.91	18.28	THRAP3	Thyroid hormone receptor-associated protein 3	NM_214815.1
A15G0698	25.90	16.73	TRAPPC4	Trafficking protein particle complex subunit 4	NM_200764.1

B19G0407	25.88	12.96	MRPL3	39S ribosomal protein L3, mitochondrial	XM_005159511.3
B23G0508	25.85	26.40	ATP6AP1	V-type proton ATPase subunit S1	NM_173265.2
B14G0067	25.85	15.65	HSPA4	Heat shock 70 kDa protein 4	NM_199857.1
B3G1301	25.83	44.42	TOB1	Protein Tob1	NM_198363.1
A1G0519	25.83	16.01	CDK2AP2	Cyclin-dependent kinase 2-associated protein 2	NM_001009898.1
A23G0477	25.82	14.19	ADA	Adenosine deaminase	NM_001002646.1
A2G0717	25.81	8.75	LAMC2	Laminin subunit gamma-2	XM_003197884.6
A12G0224	25.81	24.53	CSNK1DB	Casein kinase I isoform delta-B	NM_213250.1
B7G1055	25.79	12.19	MAP4K2	Mitogen-activated protein kinase kinase kinase kinase 2	NM_001040358.1
B21G0076	25.79	17.55	STK26	threonine-protein kinase 26	NM_001127477.1
A9G0440	25.77	11.76	ESD	S-formylglutathione hydrolase	NM_001017796.2
A7G0529	25.77	17.74			
A22G0225	25.76	17.96	BTG1	Protein BTG1	NM_001007375.2
B1G0286	25.76	10.59	CASP3	Caspase-3	NM_131877.3
B14G0492	25.76	20.98	IGFBP7	Insulin-like growth factor-binding protein 7	
B6G0748	25.75	21.90	SRSF3	arginine-rich splicing factor 3	NM_001328122.2
B7G0913	25.74	23.70	ACIN1	Apoptotic chromatin condensation inducer in the nucleus	NM_001305582.1
A17G0053	25.74	31.59	ODC1	Ornithine decarboxylase	NM_131801.2
B20G0532	25.74	23.20	PDIA6	Protein disulfide-isomerase A6	NM_197933.2
A1G0289	25.74	13.95	ACTR2A	Actin-related protein 2-A	NM_213499.1
A22G0198	25.73	2.57	LOC113092180	A0A6P6NXU6_CARAU uncharacterized protein LOC113092180	XM_001336832.9
B14G0563	25.71	10.72	TRMT112	Multifunctional methyltransferase subunit TRM112-like protein	NM_001020752.1
B2G0616	25.71	26.51	ABCF2	ATP-binding cassette sub-family F member 2	NM_201315.2
B17G0175	25.70	41.11	SPTSSA	Serine palmitoyltransferase small subunit A	NM_001093727.1
A15G0588	25.70	14.17	ITGAE	Integrin alpha-E	XM_021474336.2
B10G0781	25.69	17.39	PLK2	threonine-protein kinase PLK2	XM_003199277.6
B2G0034	25.67	45.10	FCER1G	High affinity immunoglobulin epsilon receptor subunit gamma	
A3G0595	25.67	1.37	ARPC1A	3 complex subunit 1A	XM_005164011.5
B22G0527	25.67	4.51	SHISA5	Protein shisa-5	
A1G0867	25.66	42.04	LADD	Ladderlectin	XM_002660367.5
A18G0689	25.64	8.89	GCSH	Glycine cleavage system H protein, mitochondrial	NM_001013457.1
A18G0444	25.63	0.00			
B9G0272	25.63	34.50	RALB	Ras-related protein Ral-B	NM_001003649.1
A12G0667	25.62	23.53	RAC1	Ras-related C3 botulinum toxin substrate 1	NM_199771.1
A5G0647	25.62	12.29	ERLIN2	Erlin-2	NM_001128415.1
A25G0541	25.61	16.54	EIF3JA	Eukaryotic translation initiation factor 3 subunit J-A	NM_201214.2
A24G0272	25.59	15.14	CCL11	Eotaxin	
A4G0019	25.59	22.57	CCT2	T-complex protein 1 subunit beta	NM_201455.1
B17G0879	25.56	8.61	ADI1	Acireductone dioxygenase	
B18G0047	25.55	63.88	LOC113106585	A0A6P6PTL5_CARAU uncharacterized protein LOC113106585	
A23G0100	25.51	10.73	CDH26	Cadherin-like protein 26	XM_017353725.3

A9G0748	25.51	29.14	NDUFB3	NADH dehydrogenase [ubiquinone] 1 beta subcomplex subunit 3	
A5G1008	25.48	61.41	HNRNPUL1	Heterogeneous nuclear ribonucleoprotein U-like protein 1	XM_003198712.6
B19G0591	25.47	12.65	PEF1	Peflin	NM_001003643.1
A11G0590	25.47	67.34	CDK18	Cyclin-dependent kinase 18	NM_212679.2
A17G0196	25.46	19.45	ARF6	ADP-ribosylation factor 6	NM_001161375.1
B7G0694	25.46	25.13	SPI1	Transcription factor PU.1	NM_198062.2
B5G0259	25.45	40.81	MAT2A	S-adenosylmethionine synthase isoform type-2	XM_017356170.3
B21G0693	25.45	14.73	SNRPD3	Small nuclear ribonucleoprotein Sm D3	NM_199760.1
B22G0514	25.45	7.14	NOL8	Nucleolar protein 8	NM_001080202.1
B21G0763	25.42	12.69	PSMB7	Proteasome subunit beta type-7	XM_021468901.2
A13G0201	25.42	10.94	MAP4K5	Mitogen-activated protein kinase kinase kinase kinase 5	XM_068212999.1
A23G0348	25.42	35.77	TKT	Transketolase	NM_198070.3
A17G0260	25.41	19.32	EIF2S1	Eukaryotic translation initiation factor 2 subunit 1	NM_199569.1
A9G0301	25.40	24.25	LADD	Ladderlectin	XM_003199234.5
B3G0522	25.40	64.43	ICAM2	Intercellular adhesion molecule 2	NM_001098252.2
B8G0863	25.39	5.93	DENND2C	DENN domain-containing protein 2C	XM_001919051.8
B21G0531	25.39	40.15	CBR1	Carbonyl reductase [NADPH] 1	NM_194379.2
A16G0831	25.37	2.01	HOXA13B	Homeobox protein Hox-A13b	NM_131194.2
A13G0851	25.35	21.06	IFNGR1L	Interferon gamma receptor 1-like	NM_199981.1
B6G0711	25.35	32.82	TSPO	Translocator protein	NM_001006032.2
A14G0523	25.34	27.20	HBEGF	Proheparin-binding EGF-like growth factor	NM_001111226.1
B24G0008	25.33	17.83	VAPA	Vesicle-associated membrane protein-associated protein A	NM_199918.2
A18G0433	25.33	22.90	ARPIN	Arpin	XR_002456007.2
B10G0503	25.32	14.42	SKA2	Spindle and kinetochore-associated protein 2	NM_001199675.1
B14G0244	25.31	31.51	RAB6A	Ras-related protein Rab-6A	XM_005173246.5
A12G0694	25.30	12.11	UBE2D4	Ubiquitin-conjugating enzyme E2 D4	NM_199664.1
B24G0155	25.30	13.30	ZGC:112185	UPF0488 protein C8orf33 homolog	NM_001013454.2
A15G0875	25.29	10.19	PSPH	Phosphoserine phosphatase	NM_001033099.1
B23G0833	25.28	13.80	SCP2	Sterol carrier protein 2	NM_001006093.2
B15G0578	25.28	16.80	PITPNA	Phosphatidylinositol transfer protein alpha isoform	NM_200935.1
A5G0976	25.27	13.02	SURF4	Surfeit locus protein 4	NM_001002470.2
A5G0577	25.27	25.89	PPP6C	threonine-protein phosphatase 6 catalytic subunit	NM_201005.1
B23G0842	25.27	51.13	SVEP1	Sushi, von Willebrand factor type A, EGF and pentraxin domain-containing protein 1	
B2G0805	25.26	10.03	SLC12A7	Solute carrier family 12 member 7	XM_009298533.4
B6G0679	25.24	26.52	MYL6	Myosin light polypeptide 6	XM_005166077.5
B2G0412	25.24	11.49	SDR39U1	Epimerase family protein SDR39U1	NM_001076578.1
A24G0350	25.23	22.48	BCLAF3	BCLAF1 and THRAP3 family member 3	NM_001430902.1
B14G0528	25.23	21.71	HBEGF	Proheparin-binding EGF-like growth factor	NM_001111226.1

A21G0073	25.23	2.09	ZBTB7C	Zinc finger and BTB domain-containing protein 7C	XM_003200810.6
A12G0583	25.22	35.91	ELOB	Elongin-B	
B14G0478	25.21	7.64	CLTB	Clathrin light chain B	XR_011006284.1
A3G0872	25.20	29.89	DNAJC7	DnaJ homolog subfamily C member 7	XM_009299335.4
B20G0604	25.20	8.34	SLC25A24	Calcium-binding mitochondrial carrier protein SCaMC-1	NM_001004606.1
A18G0575	25.20	5.45	MAPK12	Mitogen-activated protein kinase 12	NM_131407.1
B3G0994	25.19	1.24	LITAF	Cell death-inducing p53-target protein 1 homolog	
A18G0202	25.19	17.13	GOLGB1	Golgin subfamily B member 1	XM_021467827.2
B25G0854	25.19	9.39	CIAO2A	Cytosolic iron-sulfur assembly component 2A	NM_213027.1
B17G0893	25.19	25.01	SYNCRIP	Heterogeneous nuclear ribonucleoprotein Q	XM_068214328.1
B23G0324	25.17	27.90	NUCKS1	Nuclear ubiquitous casein and cyclin-dependent kinase substrate 1	NM_001099239.1
A4G0841	25.16	7.44	LOC113058903	A0A6P6LE90_CARAU uncharacterized protein LOC113058903	
A9G0333	25.15	57.78	SF3B1	Splicing factor 3B subunit 1	XM_679219.6
A19G0828	25.11	33.40	SDHA	Succinate dehydrogenase [ubiquinone] flavoprotein subunit, mitochondrial	NM_200910.1
B20G0406	25.10	32.84	EXOC1	Exocyst complex component 1	XM_005160556.4
B5G0591	25.10	3.90	THBS4B	Thrombospondin-4-B	NM_001114424.2
A1G0247	25.10	13.25	ZNF330	Zinc finger protein 330	NM_213373.1
B13G0617	25.10	20.24	TC2N	Tandem C2 domains nuclear protein	NM_001007777.1
B3G0256	25.09	21.93	JPT1	Jupiter microtubule associated homolog 1	
B8G0030	25.09	12.10	FGD3	FYVE, RhoGEF and PH domain-containing protein 3	NM_131556.2
A24G0629	25.08	22.54	KLF6	Krueppel-like factor 6	NM_201461.2
B3G1030	25.07	29.13	CNP	2',3'-cyclic-nucleotide 3'-phosphodiesterase	XM_068218823.1
A19G0675	25.07	20.24	NDUFV1	NADH dehydrogenase [ubiquinone] flavoprotein 1, mitochondrial	NM_001003747.1
B1G1155	25.06	12.10	TMED1	Transmembrane emp24 domain-containing protein 1	NM_001080640.1
A3G1134	25.05	14.15	SCO1	Protein SCO1 homolog, mitochondrial	
B14G0045	25.05	12.69	SSRP1	FACT complex subunit SSRP1	NM_212802.2
A14G0257	25.04	9.21	MCTS1	Malignant T-cell-amplified sequence 1	NM_201071.1
B8G0249	25.03	18.18	UBAP2	Ubiquitin-associated protein 2	XM_005167271.5
B22G1075	25.03	30.72	RNF13	E3 ubiquitin-protein ligase RNF13	NM_201044.1
B6G0770	25.03	44.29	GPX1	Glutathione peroxidase 1	
A16G0690	25.02	6.06	INO80E	INO80 complex subunit E	NM_205636.2
A8G0013	25.00	16.04	COMMD10	COMM domain-containing protein 10	
B18G0630	25.00	4.69	GAN	Gigaxonin	XM_003200434.6
A21G0123	25.00	7.89	PLAUR	Urokinase plasminogen activator surface receptor	NM_001386334.1
B15G0520	25.00	5.98	G5714_015135	A0A7J6CA12_9TELE Uncharacterized protein	XR_011006460.1
A23G0672	24.99	10.49	RARGA	Retinoic acid receptor gamma-A	XM_068216665.1

A25G0634	24.99	0.00	IGLC7	Immunoglobulin lambda constant 7	XM_068217596.1
A20G0774	24.99	7.88	NT5C1A	Cytosolic 5'-nucleotidase 1A	XM_685092.10
B19G0860	24.98	5.51	CCN2	CCN family member 2	XM_005173770.5
A12G0325	24.98	20.20	TMEM106B	Transmembrane protein 106B	NM_001128676.1
A5G0385	24.98	13.85	GPS2	G protein pathway suppressor 2	NM_001431107.1
A1G0422	24.97	9.75	SMOX	Spermine oxidase	NM_001328186.1
A13G0611	24.97	24.47	METAP1	Methionine aminopeptidase 1	NM_199611.3
A23G0484	24.96	75.14	CFH	Complement factor H	
A6G0024	24.96	6.14	RBFOX2	RNA binding protein fox-1 homolog 2	NM_214746.2
A5G0763	24.94	34.45	NDUFS4	NADH dehydrogenase [ubiquinone] iron-sulfur protein 4, mitochondrial	NM_001020676.1
A3G0633	24.92	12.76	LOC107753314	A0A673NCA9_9TELE Uncharacterized LOC107753314	
A12G0610	24.92	24.76	GSP-2	threonine-protein phosphatase PP1-beta	NM_212710.2
B14G0139	24.92	10.28	PIN4	Peptidyl-prolyl cis-trans isomerase NIMA-interacting 4	NM_001020553.1
B1G1201	24.90	30.82	CDC37	Hsp90 co-chaperone Cdc37	NM_201038.1
A15G0096	24.90	50.79	SERPINE2	Glia-derived nexin	NM_200184.1
B2G0230	24.90	15.15	PRPF38B	Pre-mRNA-splicing factor 38B	NM_213489.1
A6G0165	24.89	14.54	DAB2IP	Disabled homolog 2-interacting protein	XM_005165747.5
B25G0483	24.89	5.88	BRAWNIN	Protein brawnin	NM_001135573.2
A14G0146	24.88	12.27	NXT2	NTF2-related export protein 2	
A17G0437	24.87	13.20	PPIE	Peptidyl-prolyl cis-trans isomerase E	NM_001017678.1
A3G1120	24.87	40.41	TOB1	Protein Tob1	NM_198363.1
A1G0484	24.87	5.72	DOK1	Docking protein 1	XM_682484.9
B5G0038	24.86	41.93	HSPA5	Endoplasmic reticulum chaperone BiP	NM_213058.1
B6G0981	24.86	10.32	DNTTIP1	Deoxynucleotidyltransferase terminal-interacting protein 1	XM_002662552.7
B7G0845	24.85	16.69	IQCJ-SCHIP1	IQCJ-SCHIP1 readthrough transcript protein	XM_001338781.7
A17G0328	24.84	34.48	YY1	Transcriptional repressor protein YY1	NM_212617.2
B8G0415	24.84	18.80	SMARCB1A	SNF-related matrix-associated actin-dependent regulator of chromatin subfamily B member 1-A	NM_001007296.1
B22G1171	24.83	35.71	G5714_021418	A0A7J6BQX2_9TELE Uncharacterized protein	XM_017353481.3
B3G0842	24.82	18.07	LOC113047390	A0A6P6JXI1_CARAU cell wall protein DAN4-like	
B22G0695	24.82	160.42	GPX4	Phospholipid hydroperoxide glutathione peroxidase	NM_001346537.1
A20G0626	24.81	31.77	HIF1A	Hypoxia-inducible factor 1-alpha	NM_001310042.1
A10G0809	24.80	2.49	RASSF6	Ras association domain-containing protein 6	NM_001045339.1
A3G0959	24.79	18.97	MIEN1	Migration and invasion enhancer 1	NM_194417.3
B9G0368	24.76	11.43	ITGBL1	Integrin beta-like protein 1	NM_001024072.1
A22G0195	24.75	20.32	STRIP1	Striatin-interacting protein 1 homolog	NM_213521.2
A22G0406	24.75	80.60	BSG	Basigin	NM_001312882.1
A9G0319	24.74	13.61	ERCC1	DNA excision repair protein ERCC-1	NM_001103138.1

B2G0693	24.72	21.95	DNAJB6	DnaJ homolog subfamily B member 6	NM_001002353.1
B2G0214	24.71	18.59			NM_001100050.3
A6G0192	24.71	21.68	NOP58	Nucleolar protein 58	NM_001009889.2
A21G0608	24.70	20.70	TTC1	Tetratricopeptide repeat protein 1	NM_001007382.2
B5G1151	24.69	22.67	KAT5	Histone acetyltransferase KAT5	NM_001002365.1
A10G0075	24.68	9.37	DDX56	Probable ATP-dependent RNA helicase DDX56	NM_001003876.1
B16G0213	24.67	12.58	RNF144B	E3 ubiquitin-protein ligase RNF144B	NM_201137.2
A5G1275	24.67	37.39	YTHDC1	YTH domain-containing protein 1	XM_021476130.2
B19G0127	24.67	15.31	CLEC4E	C-type lectin domain family 4 member E	NM_001177451.1
A1G0474	24.67	20.81	NDUFS8	NADH dehydrogenase [ubiquinone] iron-sulfur protein 8, mitochondrial	NM_213139.1
B10G0683	24.67	9.84	RHOF	Rho-related GTP-binding protein RhoF	NM_001020642.1
B25G0425	24.66	17.24	CSK	Tyrosine-protein kinase CSK	NM_001077599.1
A3G1113	24.66	20.66	MSRB1	Methionine-R-sulfoxide reductase B1-A	NM_178288.5
B3G1019	24.65	2.96	GDPD1	Lysophospholipase D GDPD1	
B18G0179	24.64	22.98	ARPP19	cAMP-regulated phosphoprotein 19	NM_200630.1
B13G0009	24.64	11.39	PFLUV_G00278250	A0A6A5DYC2_PERFL CxC2 domain-containing protein	NM_199954.1
B12G0584	24.63	12.39	EMC9	ER membrane protein complex subunit 9	NM_200126.1
B14G0747	24.62	35.83	CANX	Calnexin	NM_213448.1
B10G0051	24.61	34.60	IL6ST	Interleukin-6 receptor subunit beta	NM_001113504.2
B22G1059	24.60	8.18	POLR2H	DNA-directed RNA polymerases I, II, and III subunit RPABC3	NM_001017600.1
A19G0282	24.60	20.11	TPD52	Tumor protein D52	XM_005159628.5
B20G0090	24.58	8.63	CEBPZ	enhancer-binding protein zeta	NM_001017745.3
B3G0939	24.58	26.45	GSP-2	threonine-protein phosphatase PP1-beta	NM_214811.2
B21G0108	24.58	37.87	NDFIP1L	NEDD4 family-interacting protein 1-like	NM_001002503.2
B6G0667	24.58	23.33	METTL7B	Thiol S-methyltransferase METTL7B	
A17G0144	24.57	13.39	CHMP3	Charged multivesicular body protein 3	NM_213320.1
B6G0477	24.57	10.90	DTYMK	Thymidylate kinase	
A11G0195	24.57	17.43	TMEM275	Transmembrane protein 275	NM_205558.1
A7G0200	24.56	17.61	GCSH	Glycine cleavage system H protein, mitochondrial	NM_001002579.1
A16G0779	24.56	18.70	CRABP2	Cellular retinoic acid-binding protein 2	NM_182859.1
A6G0089	24.55	17.85	SSB	Lupus La protein	NM_199547.1
A20G0300	24.55	12.16	VTA1	Vacuolar protein sorting-associated protein VTA1 homolog	NM_213140.1
B24G0054	24.54	19.38	A0A673G3H5	A0A673G3H5_9TELE AAA domain-containing protein	XM_068217268.1
A11G0036	24.54	19.72	VTXB	Verrucotoxin subunit beta	NM_001083563.2
A11G0511	24.53	28.35	ATP13A3	Polyamine-transporting ATPase 13A3	NM_205608.1
A7G1187	24.52	25.96	VIMP	Selenoprotein S	NM_001045334.3
A2G0870	24.52	22.30	NEDD8	NEDD8	NM_001002557.1

B23G0688	24.51	15.42	SARS1	Serine--tRNA ligase, cytoplasmic	NM_001003882.2
B17G0744	24.51	15.17	KTN1	Kinectin	XM_005158886.3
A25G0766	24.50	32.22	GABARAPL2	Gamma-aminobutyric acid receptor-associated protein-like 2	NM_205723.1
A13G0413	24.49	5.95	ASRGL1	L-asparaginase	NM_001002478.3
B1G0670	24.48	22.84	NDUFS8	NADH dehydrogenase [ubiquinone] iron-sulfur protein 8, mitochondrial	NM_213139.1
A15G0152	24.48	7.91	G5714_015377	A0A7J6CB12_9TELE Uncharacterized protein	
B13G0527	24.48	1.29	MYOF	Myoferlin	
B6G0526	24.48	10.97	PHB2	Prohibitin-2	NM_001002681.2
A24G0312	24.48	29.04	SH3KBP1	SH3 domain-containing kinase-binding protein 1	XM_002666661.7
B5G0308	24.47	11.37	RSRC2	serine-rich coiled-coil protein 2	NM_213280.1
A23G0063	24.47	23.20	RNF114	E3 ubiquitin-protein ligase RNF114	NM_001001828.4
B8G0070	24.47	16.48	QARS1	Glutamine--tRNA ligase	NM_001423662.1
B10G0267	24.46	14.49	TACC1	Transforming acidic coiled-coil-containing protein 1	XM_688981.10
A5G0245	24.46	16.38	ASCC2	Activating signal cointegrator 1 complex subunit 2	NM_200442.1
B2G0797	24.46	7.16	MRPL34	39S ribosomal protein L34, mitochondrial	NM_001199680.2
A20G0213	24.45	4.87	GPR31	12-(S)-hydroxy-5,8,10,14-eicosatetraenoic acid receptor	XM_005170200.5
A13G0005	24.44	8.86	LCLAT1	Lysocardiolipin acyltransferase 1	NM_200642.2
B18G0406	24.44	42.55	CTSH	Pro-cathepsin H	NM_212688.1
B23G0884	24.43	8.96	LOC113041222	A0A6P6JC75_CARAU uncharacterized protein LOC113041222	
B18G0452	24.41	9.90	NUTF2	Nuclear transport factor 2	NM_001006000.3
B2G0571	24.41	10.35	CACYBP	Calcyclin-binding protein	NM_212887.1
A24G0363	24.41	12.31	SGK3	threonine-protein kinase Sgk3	NM_001110467.1
A4G0667	24.40	62.95	DCN	Decorin	NM_131697.1
A23G0618	24.40	12.51	DGKA	Diacylglycerol kinase alpha	NM_001045315.1
B25G0647	24.34	34.69	MAP1LC3B	1B light chain 3B	NM_199604.1
A7G0655	24.34	2.18	ESRP2	Epithelial splicing regulatory protein 2	XM_005168987.5
A14G0470	24.32	43.51	HIGD2A	HIG1 domain family member 2A	NM_001017641.1
A14G0513	24.31	46.71	P33MONOX	Putative monooxygenase p33MONOX	XM_068213297.1
B12G0354	24.31	59.98	GLUD1	Glutamate dehydrogenase, mitochondrial	NM_199545.4
A12G0453	24.31	30.67	ACO2	Aconitate hydratase, mitochondrial	NM_198908.1
A6G0618	24.31	21.10	CTH	Cystathionine gamma-lyase	NM_212604.3
A11G0339	24.31	22.28	DYNLRB1	Dynein light chain roadblock-type 1	NM_200291.1
A1G0457	24.29	18.91	TIMM10	Mitochondrial import inner membrane translocase subunit Tim10	NM_001002678.3
B5G0048	24.28	8.69	RCL1	RNA 3'-terminal phosphate cyclase-like protein	NM_001003865.1
B17G0499	24.27	15.45	PGRMC2	Membrane-associated progesterone receptor component 2	NM_213104.1
A23G0277	24.27	21.06	TPD52L2	Tumor protein D54	XM_021469744.2

B9G0726	24.27	24.73	U2AF1	Splicing factor U2AF 35 kDa subunit	NM_177479.2
A19G0572	24.27	7.87	NUTF2	Nuclear transport factor 2	NM_001003598.2
B3G0362	24.25	17.53	ILF3	Interleukin enhancer-binding factor 3 homolog	XM_005163741.4
B19G0663	24.24	136.08	FABP3	Fatty acid-binding protein, heart	NM_001004682.1
A5G0195	24.23	37.92	CCL19	C-C motif chemokine 19	
B11G0663	24.21	23.43	PER3	Period circadian protein homolog 3	
A3G0452	24.21	12.20	NSMCE1	Non-structural maintenance of chromosomes element 1 homolog	
B8G0965	24.20	0.33	DHX37	Probable ATP-dependent RNA helicase DHX37	NM_001083004.2
A8G0813	24.20	37.46	CHMP4C	Charged multivesicular body protein 4c	NM_213457.1
B7G0846	24.20	17.84	RNASEKA	Ribonuclease kappa-A	NM_001045405.2
B7G1219	24.18	8.29	ACTR2B	Actin-related protein 2-B	NM_001007772.1
B6G1048	24.15	16.36	DDX3Y	ATP-dependent RNA helicase DDX3Y	XM_068221646.1
B2G1036	24.15	17.63			NM_001003449.1
A9G0779	24.13	30.75	NFE2L2	Nuclear factor erythroid 2-related factor 2	NM_182889.1
A18G0813	24.12	14.62	HDGFL3	Hepatoma-derived growth factor-related protein 3	NM_001012372.1
B6G0690	24.11	15.32	SEC61AL1	Protein transport protein Sec61 subunit alpha-like 1	NM_153659.2
A2G0672	24.10	16.51	PUF60	Poly(U)-binding-splicing factor PUF60	XM_009298622.4
B16G0599	24.08	10.82	MRPL17	39S ribosomal protein L17, mitochondrial	NM_001004683.1
B17G0725	24.06	30.82	ALDH6A1	Methylmalonate-semialdehyde dehydrogenase [acylating], mitochondrial	NM_001002374.2
B18G0520	24.06	17.96	AARS1	Alanine--tRNA ligase, cytoplasmic	NM_001044310.2
A20G0145	24.06	5.20	FOS	Protein c-Fos	XM_687741.10
B16G0420	24.04	36.14	PNISR	serine-rich protein PNISR	NM_001083837.1
A16G0719	24.04	20.61	USP5	Ubiquitin carboxyl-terminal hydrolase 5	NM_214755.2
B4G0555	24.03	20.53	GNAI1	Guanine nucleotide-binding protein G(i) subunit alpha-1	NM_200971.1
B6G0779	24.03	15.28	RBM5	RNA-binding protein 5	
B18G0840	24.00	22.11	NMES1	Normal mucosa of esophagus-specific gene 1 protein	
A23G0889	24.00	11.74	PSMB6	Proteasome subunit beta type-6	NM_131152.1
A15G0462	23.99	6.96	ERCC2	General transcription and DNA repair factor IIH helicase subunit XPD	NM_200926.2
B7G0478	23.98	8.03	NAE1	NEDD8-activating enzyme E1 regulatory subunit	NM_200499.1
A20G0076	23.98	20.62	BROX	BRO1 domain-containing protein BROX	NM_001007427.2
A5G0454	23.98	5.17	RNF181	E3 ubiquitin-protein ligase RNF181	NM_200306.1
B1G0540	23.96	12.22	CHMP2B	Charged multivesicular body protein 2b	NM_212904.2
B16G0079	23.94	11.99	SRSF10	arginine-rich splicing factor 10	XM_017351572.3
A16G0570	23.94	25.15	DERL1	Derlin-1	NM_213444.2
B19G0105	23.94	80.28	HA12	RT1 class I histocompatibility antigen, AA alpha chain	NM_131705.1

A9G0350	23.93	6.44	GPR183A	G-protein coupled receptor 183-A	
B11G0762	23.92	25.14	SYNGR2	Synaptogyrin-2	XM_009305746.4
B3G0639	23.90	32.01	BAX	Apoptosis regulator BAX	NM_131562.2
B19G0930	23.90	18.90	PSMB7	Proteasome subunit beta type-7	
A3G0062	23.89	19.65	NEK1	threonine-protein kinase Nek1	XM_017356939.3
B21G0017	23.89	26.42	TXNDC15	Thioredoxin domain-containing protein 15	
A11G0112	23.89	14.13	ARID3A	AT-rich interactive domain-containing protein 3A	NM_001002355.1
A5G0472	23.89	8.16	SFRP1	Secreted frizzled-related protein 1	NM_205585.2
B8G0663	23.88	23.30	NFIC	Nuclear factor 1 C-type	XM_005166939.5
A15G0525	23.88	13.84	RNASET2	Ribonuclease T2	NM_001386227.1
A7G0879	23.88	18.38	NAA40	N-alpha-acetyltransferase 40	NM_001017632.1
A22G0515	23.86	42.35	RGS1	Regulator of G-protein signaling 1	XM_001332065.9
B3G0237	23.85	21.86	TRIR	Telomerase RNA component interacting RNase	NR_170156.1
A21G0223	23.84	16.65	GLRX	Glutaredoxin-1	NM_001005942.1
B13G0684	23.83	17.78	PACS2	Phosphofurin acidic cluster sorting protein 2	XM_005156639.5
B25G0397	23.82	15.77	API5	Apoptosis inhibitor 5	NM_199540.2
A10G0299	23.81	14.39	USP25	Ubiquitin carboxyl-terminal hydrolase 25	XM_005169157.5
B22G0924	23.81	11.00	COX19	Cytochrome c oxidase assembly protein COX19	NM_001110540.2
B14G0245	23.81	40.47	SEPTIN6	Septin-6	NM_212626.2
B18G0398	23.81	29.99	SEC11A	Signal peptidase complex catalytic subunit SEC11A	NM_001002521.2
A16G0145	23.80	13.05	RPZ	Protein rapunzel	NM_001145238.1
B14G0161	23.79	25.76	FGL1	Fibrinogen-like protein 1	XM_001923551.7
B25G0704	23.78	11.22	DHTKD1	2-oxoadipate dehydrogenase complex component E1	NM_001008619.1
B1G0677	23.78	21.60	POLD4	DNA polymerase delta subunit 4	NM_001256200.1
A5G0459	23.77	6.40	IMP4	U3 small nucleolar ribonucleoprotein protein IMP4	NM_001002719.1
A19G0130	23.76	17.38	POLR2K	DNA-directed RNA polymerases I, II, and III subunit RPABC4	NM_001199106.1
B17G0505	23.76	3.74	GRHL3	Grainyhead-like protein 3 homolog	XM_068214602.1
A16G0203	23.76	8.56	ATAD2	ATPase family AAA domain-containing protein 2	
B24G0278	23.75	11.85	SNX7	Sorting nexin-7	XM_005162844.5
B8G0945	23.74	12.59	PPP2R2A	threonine-protein phosphatase 2A 55 kDa regulatory subunit B alpha isoform	XM_682011.10
B19G0140	23.73	1.40	KRT13	Keratin, type I cytoskeletal 13	NM_001082882.1
A6G0838	23.73	34.43	RHOAD	Rho-related GTP-binding protein RhoA-D	NM_201150.1
B17G0452	23.72	13.23	WDR43	WD repeat-containing protein 43	NM_001018166.1
A9G0225	23.72	17.25	STAM2	Signal transducing adapter molecule 2	NM_001007369.2
A6G0706	23.71	1.97			
B25G0842	23.70	31.75	FBLN1	Fibulin-1	NM_131042.2
A6G0576	23.70	15.49	HIBN	N2	NM_199782.2
B16G0009	23.69	26.06	KHDC4	KH homology domain-containing protein 4	XM_005169775.5
A3G0395	23.65	9.35	PPAN	Suppressor of SWI4 1 homolog	NM_201463.1
B14G0497	23.65	4.02	EXOSC3	Exosome complex component RRP40	

A18G0651	23.64	41.49			
A3G1229	23.64	25.80	CYTH1	Cytohesin-1	NM_214682.2
B4G0184	23.64	13.96	ARL1	ADP-ribosylation factor-like protein 1	NM_001002473.1
A21G0279	23.64	19.70	SFSWAP	Splicing factor, suppressor of white-apricot homolog	NM_201168.2
A14G0778	23.62	23.52	PRPS1	Ribose-phosphate pyrophosphokinase 1	NM_001076568.2
A16G0311	23.62	4.95	BNIP2	adenovirus E1B 19 kDa protein-interacting protein 2	NM_001128394.1
B7G0616	23.62	8.93			XM_001922501.8
A3G0120	23.60	6.62	POLR3H	DNA-directed RNA polymerase III subunit RPC8	NM_001002476.1
B6G0851	23.60	41.57	CAPZA1	F-actin-capping protein subunit alpha-1	NM_212697.1
B19G0923	23.60	13.04	CD6	T-cell differentiation antigen CD6	
B13G0326	23.59	38.16	SDCCAG8	Serologically defined colon cancer antigen 8 homolog	NM_198804.1
A19G0258	23.58	11.27	MFSD2AB	Sodium-dependent lysophosphatidylcholine symporter 1-B	NM_001003570.2
B15G0209	23.58	5.79	LPAR6	Lysophosphatidic acid receptor 6	NM_001080055.2
B8G0031	23.57	10.58	SLC35E2A	Solute carrier family 35 member E2A	NM_001436596.1
B21G0816	23.57	438.12	PLAUR	Urokinase plasminogen activator surface receptor	XM_068216177.1
B19G0861	23.56	22.03	STX12	Syntaxin-12	NM_001002051.1
A15G0722	23.56	5.20	LPAR6	Lysophosphatidic acid receptor 6	
A15G0559	23.55	2.49	ZBTB16A	Zinc finger and BTB domain-containing protein 16-A	XM_693182.7
B7G0204	23.55	7.32	PACSIN3	Protein kinase C and casein kinase II substrate protein 3	NM_200989.1
A8G0344	23.54	42.63	PLIN2	Perilipin-2	NM_001386314.1
B10G0256	23.54	11.97	PBDC1	Protein PBDC1	NM_001002704.2
A19G0371	23.53	36.68	SFPQ	Splicing factor, proline- and glutamine-rich	XR_222585.4
B6G0985	23.53	23.68	CTSA	Lysosomal protective protein	NM_200550.1
A5G1330	23.53	12.24	UTP14A	U3 small nucleolar RNA-associated protein 14 homolog A	XM_005165628.5
A25G0259	23.52	13.19	POLR2C	DNA-directed RNA polymerase II subunit RPB3	
A17G0402	23.51	65.26	CALM1	Calmodulin-1	NM_213351.1
A12G0075	23.51	2.89	PLAU	Urokinase-type plasminogen activator	NM_001252350.1
A3G0711	23.51	5.25	PRRG4	Transmembrane gamma-carboxyglutamic acid protein 4	
B19G0142	23.50	0.18	K1C1	Keratin, type I cytoskeletal 50 kDa	XM_001342753.9
A2G1040	23.50	28.04	PFKP	ATP-dependent 6-phosphofructokinase, platelet type	XM_068218052.1
A8G0749	23.50	14.88	PQBP1	Polyglutamine-binding protein 1	NM_001002435.1
A14G0681	23.50	2.68	TMEM109	Transmembrane protein 109	XM_003199892.5
B3G1105	23.48	3.97	ITGB3	Integrin beta-3	NM_001037235.1
A8G0116	23.47	28.49	UFD1	Ubiquitin recognition factor in ER-associated degradation protein 1	NM_001002451.1

A16G0331	23.44	13.71	THRAP3	Thyroid hormone receptor-associated protein 3	XM_068214170.1
B21G0152	23.44	42.89	DNTS_008897	A0A553MVU5_9TELE Uncharacterized protein	XR_659427.4
B14G0008	23.44	8.65	LYAR	Cell growth-regulating nucleolar protein	NM_200679.3
A16G0458	23.42	2.57	TNFAIP8L2B	Tumor necrosis factor, alpha-induced protein 8-like protein 2 B	NM_200374.1
A9G0550	23.41	18.58	SAP18	Histone deacetylase complex subunit SAP18	
A12G0520	23.41	7.26	EIF2AK1	Eukaryotic translation initiation factor 2-alpha kinase 1	XM_021480133.2
A22G0134	23.40	2.32	LOC113080564	A0A6P6NK80_CARAU uncharacterized protein LOC113080564	NM_001423391.1
A25G0033	23.39	20.43	CIB1	Calcium and integrin-binding protein 1	XM_003201379.5
A1G0185	23.39	9.69	LGALSL	Galectin-related protein	
A16G0361	23.39	9.17	UTP11	Probable U3 small nucleolar RNA-associated protein 11	NM_199998.1
B23G0462	23.38	15.51	SZRD1	SUZ domain-containing protein 1	NM_001030076.2
A9G0592	23.37	23.34	ITGAV	Integrin alpha-V	NM_001033721.1
B3G1617	23.36	46.69	F10	Coagulation factor X	NM_001327792.1
A18G0171	23.36	10.07	TMOD3	Tropomodulin-3	XM_068214761.1
B8G0160	23.35	0.55	NKX3-1	Homeobox protein Nkx-3.1	NM_001386325.1
B18G0134	23.34	17.66	CCNL1	Cyclin-L1	NM_001089363.1
B22G0300	23.34	18.53	A0A672K534	A0A672K534_SINGR Uncharacterized protein	XM_005161637.5
A8G0075	23.34	7.30	POLB	DNA polymerase beta	NM_001003879.2
A4G0547	23.32	7.96	PAWR	PRKC apoptosis WT1 regulator protein	NM_001006015.2
B1G0689	23.30	9.78	CTNND1	Catenin delta-1	XM_009293418.4
A9G0527	23.30	10.14	LMO7	LIM domain only protein 7	XM_068223383.1
A14G0230	23.30	33.46	UBE2A	Ubiquitin-conjugating enzyme E2 A	NM_201273.1
B25G0295	23.30	21.21	COPS2	COP9 signalosome complex subunit 2	NM_001002055.1
A2G0813	23.29	18.02	ELAVL1	ELAV-like protein 1	XM_017357655.2
B8G0946	23.29	26.21	BNIP3L	adenovirus E1B 19 kDa protein-interacting protein 3-like	NM_001012242.1
A10G0626	23.28	20.21	R3HCC1L	Coiled-coil domain-containing protein R3HCC1L	XM_068224067.1
B14G0441	23.28	8.53	PPID	Peptidyl-prolyl cis-trans isomerase D	NM_001002065.1
A19G0816	23.28	29.92	TAPBP	Tapasin	NM_130974.3
A15G0379	23.26	20.52	MYO1C	Unconventional myosin-Ic	NM_001317189.1
B9G0004	23.26	30.19	NMI	N-myc-interactor	
B22G0776	23.24	110.16	REX1BD	Required for excision 1-B domain-containing protein	NM_001256159.1
A10G0627	23.24	22.35	GOLGA7	Golgin subfamily A member 7	NM_001105599.1
A16G0639	23.22	10.14	RAB13	Ras-related protein Rab-13	NM_201329.1
B8G0098	23.20	17.96	MFN2	Mitofusin-2	NM_001128254.2
A6G0162	23.20	13.81	ILF3	Interleukin enhancer-binding factor 3 homolog	XM_068221651.1
B4G0246	23.19	6.52	LMF2	Lipase maturation factor 2	NM_001142563.1
A4G0911	23.16	12.56	APEX1	DNA-(apurinic or apyrimidinic site) endonuclease	NM_001328578.1
A16G0102	23.16	20.96	RAB5A	Ras-related protein Rab-5A	NM_200970.1

B16G0844	23.15	7.36	ROHU_019516	A0A498N575_LABRO Sister chromatid cohesion protein DCC1	XM_021466876.2
B23G0528	23.14	30.62	STAT5B	Signal transducer and activator of transcription 5B	NM_001130590.1
A13G0657	23.14	7.83	CCDC176	Basal body-orientation factor 1	XM_009307490.4
A14G0118	23.13	13.73	ATOX1	Copper transport protein ATOX1	
A11G0090	23.13	18.15	IL17RD	Interleukin-17 receptor D	NM_001243179.1
B3G0817	23.13	16.60	CASP6	Caspase-6	XM_068216927.1
A23G0489	23.13	66.02	TMEM269	Transmembrane protein 269	NM_001195247.1
B3G0444	23.12	6.11	BAIAP2	Brain-specific angiogenesis inhibitor 1-associated protein 2	NM_001040245.2
A19G0456	23.11	3.44	FAM83H	Protein FAM83H	NM_001365994.1
B23G0927	23.11	7.38	IGSF10	Immunoglobulin superfamily member 10	
A3G0354	23.11	7.19	BAIAP2	Brain-specific angiogenesis inhibitor 1-associated protein 2	NM_001040245.2
B20G0502	23.10	50.38	YWHAQ	14-3-3 protein theta	NM_201513.1
A15G0050	23.10	9.63	IWS1	Protein IWS1 homolog	XM_005157821.5
B22G0500	23.10	11.43	BLOC1S1	Biogenesis of lysosome-related organelles complex 1 subunit 1	NM_001044350.2
B13G0808	23.08	18.06	BSDC1	BSD domain-containing protein 1	NM_001320391.1
B22G1047	23.07	13.82	DNAJC19	Mitochondrial import inner membrane translocase subunit TIM14	NM_200761.1
A14G0017	23.06	38.35	WDR1	WD repeat-containing protein 1	NM_201039.1
B6G0238	23.04	7.01	CCR7	C-C chemokine receptor type 7	NM_001098743.1
B16G0751	23.03	3.38	ENDOD1	Endonuclease domain-containing 1 protein	NM_001328123.1
A13G0176	23.03	9.86	PHF3	PHD finger protein 3	NM_001017593.2
B7G1282	23.02	39.35	EMC4	ER membrane protein complex subunit 4	NM_205658.1
A14G0176	23.02	12.01	PFDN6	Prefoldin subunit 6	NM_200513.1
B16G0324	23.02	36.25	LOC107705228	A0A673LCQ1_9TELE CD83 antigen-like	
B13G0708	23.01	16.54	TMX1	Thioredoxin-related transmembrane protein 1	XM_068212817.1
B19G0235	23.00	7.97	LOC113119985	A0A6P6RIP7_CARAU nuclear GTPase SLIP-GC-like isoform X2	
A6G0599	22.99	16.33	RGS4	Regulator of G-protein signaling 4	NM_199274.1
A6G0804	22.99	4.96	CENPS	Centromere protein S	
B14G0255	22.98	23.32	CUL4B	Cullin-4B	NM_001122844.1
B22G0032	22.98	9.62	ELF3	ETS-related transcription factor Elf-3	XM_002666100.4
B2G0385	22.97	17.99	MMP14	Matrix metalloproteinase-14	NM_194414.1
B11G0053	22.96	12.21	NR1D1	Nuclear receptor subfamily 1 group D member 1	XM_021479603.2
B13G0782	22.93	11.51	SMOC2	SPARC-related modular calcium-binding protein 2	XM_005169512.5
B19G0497	22.93	46.83	LOC113119309	A0A6P6RGB2_CARAU uncharacterized protein LOC113119309	
A19G0069	22.93	24.34	PSMB7	Proteasome subunit beta type-7	NM_001328552.2
B17G0868	22.92	23.37	ODC1-A	Ornithine decarboxylase 1	NM_131801.2
B3G0671	22.92	10.76	PPL	Periplakin	XM_005164103.5
A4G0501	22.92	8.90	ACOT4	Peroxisomal succinyl-coenzyme A thioesterase	XM_005164835.4
B19G0965	22.92	21.94	KPNB1	Importin subunit beta-1	NM_001037702.3

A1G0970	22.90	10.81	CASP3	Caspase-3	NM_131877.3
B19G0490	22.89	11.01	WASF2	Actin-binding protein WASF2	NM_001328190.1
B6G1040	22.88	368.73	WBP4	WW domain-binding protein 4	
A3G0194	22.87	12.81	MAP2K4	Dual specificity mitogen-activated protein kinase kinase 4	XM_068216307.1
A9G0699	22.85	33.10	PDXK	Pyridoxal kinase	
A7G0360	22.85	11.58	RWDD4	RWD domain-containing protein 4	NM_001320090.1
B2G0433	22.84	28.27	COPA	Coatomer subunit alpha	NM_001001941.2
A1G0783	22.84	20.74	NDFIP2	NEDD4 family-interacting protein 2	NM_213347.2
A2G0862	22.84	24.95	ACIN1	Apoptotic chromatin condensation inducer in the nucleus	XM_021471260.2
B2G0611	22.83	37.20	G5714_001922	A0A7J6DDQ8_9TELE Uncharacterized protein	
B7G0411	22.82	22.45	MFAP1A	Microfibrillar-associated protein 1A	NM_201017.1
B19G0313	22.82	22.00	PSMD4	26S proteasome non-ATPase regulatory subunit 4	NM_001002112.2
B9G0537	22.81	9.08	NEPRO	Nucleolus and neural progenitor protein	NM_001017650.2
A1G0919	22.81	34.81	CLTA	Clathrin light chain A	NM_213045.1
B3G0491	22.80	10.07	S1PR2	Sphingosine 1-phosphate receptor 2	NM_001159970.1
A5G0351	22.80	12.61	PRPS1	Ribose-phosphate pyrophosphokinase 1	NM_001359894.1
B19G0752	22.79	49.96	CSNK2B	Casein kinase II subunit beta	NM_131187.1
A12G0361	22.78	37.56	GLUD1	Glutamate dehydrogenase, mitochondrial	NM_199545.4
A13G0691	22.78	22.04	VDAC2	Voltage-dependent anion-selective channel protein 2	NM_001033106.1
B7G0839	22.78	10.50	NECTIN3	Nectin-3	
A4G1041	22.78	13.06	TMEM181	Transmembrane protein 181	
B21G0363	22.77	9.81	OTUB1	Ubiquitin thioesterase OTUB1	NM_001002411.2
A1G0274	22.76	11.46	RAD23A	UV excision repair protein RAD23 homolog A	NM_001003739.1
B13G0435	22.76	9.16	RTASE	Probable RNA-directed DNA polymerase from transposon BS	NM_200814.1
B24G0404	22.76	17.31	SGK3	threonine-protein kinase Sgk3	NM_001110467.1
B12G0588	22.75	8.85	PELO	Protein pelota homolog	NM_201136.1
B10G0764	22.74	20.46	KMT5AA	N-lysine methyltransferase KMT5A-A	NM_001045349.2
A3G0645	22.72	2.80	CLPP	ATP-dependent Clp protease proteolytic subunit, mitochondrial	NM_001020684.1
B5G0072	22.72	8.82	ZNHIT3	Zinc finger HIT domain-containing protein 3 (Fragment)	NM_200273.1
B2G0592	22.70	10.14	VWC2L	von Willebrand factor C domain-containing protein 2-like	NM_001013486.2
A22G0373	22.69	25.58	RB11B	Ras-related protein Rab-11B	NM_214770.1
A14G0055	22.68	5.63	SLC43A1	Large neutral amino acids transporter small subunit 3	NM_001327986.1
A10G0756	22.68	15.82	CDC37L1	Hsp90 co-chaperone Cdc37-like 1	
A21G0169	22.67	29.60	F2RL1	Proteinase-activated receptor 2	NM_001098778.1
A3G1057	22.67	43.56	CTSD	Cathepsin D	NM_200031.1
B22G0583	22.66	47.34	STAT1	beta	NM_131480.1
B8G0654	22.66	50.27	ALDH9A1A	4-trimethylaminobutyraldehyde dehydrogenase A	XM_005166774.4

B4G0545	22.66	27.88	HCLS1	Hematopoietic lineage cell-specific protein	NM_001045006.1
B8G0454	22.65	19.29	G5714_009131	A0A7J6CRB4_9TELE Ig-like domain-containing protein	
B3G0469	22.65	13.69	PET100	Protein PET100 homolog, mitochondrial	
B16G0940	22.64	13.02	M6PR	Cation-dependent mannose-6-phosphate receptor	NM_213205.1
A2G1044	22.63	10.24	LPAR6	Lysophosphatidic acid receptor 6	
B1G1096	22.63	7.93	GGT1	Glutathione hydrolase 1 proenzyme	XM_009295754.3
B11G0734	22.63	5.53	GNB1	G(T) subunit beta-1	XM_005155659.5
B3G1396	22.63	10.63	SNU13	NHP2-like protein 1	NM_199535.2
B17G0570	22.62	16.41	TPP1	Tripeptidyl-peptidase 1	NM_001128798.1
A22G0201	22.62	6.63	LOC113077525	A0A6P6N9E6_CARAU natural killer cell receptor 2B4-like isoform X2	XM_068216396.1
A6G0848	22.62	23.62	TSPAN2	Tetraspanin-2	NM_001018150.1
B12G0214	22.62	40.93	CSNK1DB	Casein kinase I isoform delta-B	NM_213250.1
A16G0196	22.61	15.63	RNF144B	E3 ubiquitin-protein ligase RNF144B	XM_005173527.5
B13G0805	22.61	34.67	COL6A6	Collagen alpha-6(VI) chain	
B13G0614	22.60	31.58	ZFYVE26	Zinc finger FYVE domain-containing protein 26	XM_021480549.2
B9G0326	22.59	36.39	DDX3Y	ATP-dependent RNA helicase DDX3Y	XM_021478733.2
A6G0976	22.58	7.47	TFAP2C	Transcription factor AP-2 gamma	NM_001008576.2
A12G0837	22.58	1.38	WFIKKN2	WAP, Kazal, immunoglobulin, Kunitz and NTR domain-containing protein 2	XM_694147.6
B25G0234	22.57	12.76	MRPL23	39S ribosomal protein L23, mitochondrial	NM_001002052.2
A5G1291	22.56	13.99	PSAT1	Phosphoserine aminotransferase	NM_199819.2
A16G0974	22.55	8.31	NLRC3	NLR family CARD domain-containing protein 3	XM_068221903.1
B6G0818	22.54	40.86	TARDBP	TAR DNA-binding protein 43	XR_002458992.2
A5G0982	22.53	27.83	RAB14	Ras-related protein Rab-14	NM_201495.1
B2G0100	22.53	23.62	NDUFV2	NADH dehydrogenase [ubiquinone] flavoprotein 2, mitochondrial	NM_200747.1
A25G0311	22.53	10.79	SETD6	N-lysine methyltransferase setd6	
B25G0562	22.52	6.80	FAR1	Fatty acyl-CoA reductase 1	XM_005174315.5
A5G0474	22.52	26.85	TCIM	Transcriptional and immune response regulator	NM_001013472.2
A20G0656	22.51	37.79	EIF5	Eukaryotic translation initiation factor 5	NM_199591.2
A5G1087	22.51	13.53	TIA1	Cytotoxic granule associated RNA binding protein TIA1	NM_212628.1
A23G0392	22.51	15.16	RCC2	Protein RCC2 homolog	XM_005162162.5
A12G0685	22.50	31.60	ANK3	Ankyrin-3	XM_021480248.2
A22G0007	22.50	12.36	NDUFAF3	NADH dehydrogenase [ubiquinone] 1 alpha subcomplex assembly factor 3	
A13G0477	22.49	7.36	LGALSL-B	Galectin-related protein B	NM_200814.1
B24G0112	22.48	1.79	EPS8L1	Epidermal growth factor receptor kinase substrate 8-like protein 1	XM_005170799.5
A25G0732	22.48	19.42	SPI1	Transcription factor PU.1	NM_001430957.1

B11G0709	22.48	11.98	LOC107557405	A0A672SXM8_SINGR Uncharacterized LOC107557405	NM_201023.1
B3G0717	22.47	14.01	SEPTIN9	Septin-9	NM_001423665.1
B8G0668	22.46	18.43	MKNK2	threonine-protein kinase 2	NM_194402.1
B2G0338	22.46	16.48	ZNF622	Cytoplasmic 60S subunit biogenesis factor ZNF622	XM_017352038.3
B6G0363	22.44	18.62	PSMD12	26S proteasome non-ATPase regulatory subunit 12	NM_201578.1
A1G0520	22.44	17.52	AIP	AH receptor-interacting protein	NM_214712.1
B11G0618	22.44	1.40	TKT	Transketolase	XM_684926.10
B10G0230	22.43	34.29	SLC20A1B	Sodium-dependent phosphate transporter 1-B	XM_005155625.5
A18G0756	22.43	26.04	LSM14A	Protein LSM14 homolog A	XM_005158974.4
B1G0466	22.42	9.49	GK	Glycerol kinase	NM_001431101.1
B22G0412	22.42	14.22	LOC113099126	A0A6P6PGX0_CARAU CD48 antigen-like	XM_021469671.2
A10G0176	22.42	9.83	TMEM167A	Protein kish-A	NM_001023581.2
B8G0497	22.41	36.06	PRKAR2A	cAMP-dependent protein kinase type II-alpha regulatory subunit	NM_001077370.2
A16G0852	22.41	13.82	TNFRSF22	Tumor necrosis factor receptor superfamily member 22	
A23G0410	22.40	8.88	CA6	Carbonic anhydrase 6	XM_021473673.2
B18G0581	22.40	46.21	CTSD	Cathepsin D	NM_131710.2
A15G0004	22.40	7.87	CD3E	T-cell surface glycoprotein CD3 epsilon chain	
A12G0036	22.39	0.00	A0A674BC41	A0A674BC41_SALTR Uncharacterized protein	
B1G0096	22.38	22.36	ATIC	Bifunctional purine biosynthesis protein ATIC	NM_001082796.2
B8G0183	22.38	24.33	COX5B	Cytochrome c oxidase subunit 5B, mitochondrial	NM_001386398.1
B3G0270	22.38	29.09	HLF	Hepatic leukemia factor	NM_001077334.2
B18G0539	22.38	160.03	MT1	Metallothionein-1	NM_131075.1
A21G0855	22.36	17.65	KCT2	Keratinocyte-associated transmembrane protein 2	XM_003200909.6
B21G0634	22.36	19.21	UBE2G1	Ubiquitin-conjugating enzyme E2 G1	NM_213530.1
A23G0430	22.36	15.14	DDOST	Dolichyl-diphosphooligosaccharide--protein glycosyltransferase 48 kDa subunit	NM_213093.1
B22G0570	22.35	12.74	CCNT2	Cyclin-T2	NM_212586.1
A5G0282	22.35	92.58	UBIQP	Polyubiquitin	
B16G0409	22.35	63.28	PNRC2	Proline-rich nuclear receptor coactivator 2	NM_001130192.1
B15G0361	22.33	15.49	RPS6KB1	Ribosomal protein S6 kinase beta-1	NM_213076.1
B5G0816	22.33	10.34	SRSF7	arginine-rich splicing factor 7	NM_205673.1
B6G0617	22.32	22.84	CTH	Cystathionine gamma-lyase	NM_212604.3
B14G0817	22.31	11.33	POLR1D	DNA-directed RNA polymerases I and III subunit RPAC2	
A3G0859	22.31	19.43	MAPK1	Mitogen-activated protein kinase 1	NM_201507.1
A18G0697	22.30	31.44	NOB1	RNA-binding protein NOB1	XM_678662.10
B15G0847	22.30	40.75	ACTN4	Alpha-actinin-4	XM_068213466.1
B24G0171	22.28	18.41	UBE2I	SUMO-conjugating enzyme UBC9	NM_131351.2
B15G0887	22.28	27.37	IWS1-A	Protein IWS1 homolog A	XM_005157821.5
B14G0394	22.27	16.89	UBXN1	UBX domain-containing protein 1	NM_212607.1
A19G0524	22.27	22.85	CTNNB1	Catenin beta-1	NM_001001889.1

B8G0306	22.27	29.61	RNF10	RING finger protein 10	NM_001123255.1
A20G0531	22.27	9.76	CHURC1	Protein Churchill	NM_001007380.1
A8G0452	22.27	12.75	WAS	Actin nucleation-promoting factor WAS	NM_199938.1
A7G0619	22.25	13.74	CEBPG	enhancer-binding protein gamma	NM_131886.2
A16G0538	22.25	5.76	ESRP1	Epithelial splicing regulatory protein 1	XM_005158027.5
A16G0709	22.25	14.22	PAFAH1B3	Platelet-activating factor acetylhydrolase IB subunit alpha1	NM_201058.1
A1G0576	22.24	19.72	KCTD12	POZ domain-containing protein KCTD12	NM_001034019.2
B3G0912	22.23	16.47	CCDC47	PAT complex subunit CCDC47	NM_001004551.1
B13G0727	22.23	26.76	FAM135A	Protein FAM135A	NM_001025502.1
A15G0359	22.22	20.20	TMEM87A	Transmembrane protein 87A	XM_068213563.1
A22G0427	22.22	2.26	BDMD1	Duodenase-1	XM_068218989.1
A11G0087	22.21	27.37	LOC107557405	A0A672SXM8_SINGR Uncharacterized LOC107557405	
B14G0782	22.21	15.50	NUP62	Nuclear pore glycoprotein p62	XM_005157152.5
A10G0013	22.20	25.50	MPZL1	Myelin protein zero-like protein 1	NM_001257340.1
A15G0804	22.19	13.57	TGFB1	Transforming growth factor beta-1 proprotein	NM_182873.1
A13G0772	22.18	5.98	PPP2R5D	threonine-protein phosphatase 2A 56 kDa regulatory subunit delta isoform	NM_201147.2
A11G0410	22.17	4.42	ACAD9	Complex I assembly factor ACAD9, mitochondrial	NM_001080582.1
B3G0573	22.17	3.76	NUDT1	Oxidized purine nucleoside triphosphate hydrolase	NM_213418.1
B5G0131	22.17	5.80	MYBBP1A	Myb-binding protein 1A-like protein	NM_001002042.2
A20G0552	22.16	14.16	MRPL19	39S ribosomal protein L19, mitochondrial	NM_001003544.2
B1G0734	22.16	0.42	ROHU_003313	A0A498NV41_LABRO Flocculation FLO11-like protein	XM_021476859.2
B12G0587	22.15	20.72	PPP1CA	threonine-protein phosphatase PP1-alpha catalytic subunit	NM_212710.2
B9G0821	22.15	2.61			
A6G0517	22.14	39.50	DPT	Dermatopontin	NM_001030085.3
B3G1410	22.14	35.91	LGALS3BPA	Galectin-3-binding protein A	NM_001040041.1
B4G0249	22.14	13.61	LSM8	U6 snRNA-associated Sm-like protein LSm8	NM_001302466.1
A6G0264	22.13	34.40	TMBIM1	Protein lifeguard 3	NM_001005992.2
A14G0094	22.13	22.63	DPF2	Zinc finger protein ubi-d4	NM_212696.2
A16G0169	22.12	11.25	FXYD1	Phospholemman	
B7G0094	22.12	16.35	PSMD9	26S proteasome non-ATPase regulatory subunit 9	XM_005169066.5
A11G0775	22.12	12.76	ARFGAP1	ADP-ribosylation factor GTPase-activating protein 1	
A7G0203	22.10	19.44	PSMD7	26S proteasome non-ATPase regulatory subunit 7	NM_199789.1
A19G0016	22.09	24.03	UBE2M	NEDD8-conjugating enzyme Ubc12	XM_001920635.9
B25G0066	22.09	27.62	GPI	Glucose-6-phosphate isomerase	NM_144763.2
B2G0562	22.08	27.36	G5714_001965	A0A7J6DDP6_9TELE Uncharacterized protein	
A5G0788	22.07	7.18	G5714_005595	A0A7J6D1G7_9TELE Uncharacterized protein	

B16G0596	22.06	39.75	CD79A	B-cell antigen receptor complex-associated protein alpha chain	NM_001326470.1
A5G0913	22.06	9.81	BLVRB	Flavin reductase (NADPH)	
A2G0837	22.06	4.91	LTB4R	Leukotriene B4 receptor 1	NM_001308970.2
A16G0250	22.05	0.43			
A11G0268	22.05	9.63	DIDO1	Death-inducer obliterator 1	XM_005155747.4
A19G0125	22.04	8.54	ZNF706	Zinc finger protein 706	NM_001198891.2
A4G0100	22.03	9.81	RPZ	Protein rapunzel	
A16G0726	22.03	45.12	LENG8	Leukocyte receptor cluster member 8 homolog	NM_001089519.1
B23G0029	22.03	15.77	HM13	Minor histocompatibility antigen H13	XM_005161890.5
A2G0424	22.02	19.03	IVNS1ABPB	Influenza virus NS1A-binding protein homolog B	NM_201483.1
B10G0186	22.02	35.75	PLPP1	Phospholipid phosphatase 1	XM_692415.10
B19G0724	22.01	10.09	MED10	Mediator of RNA polymerase II transcription subunit 10	NM_001017699.1
B5G1008	22.00	12.26	NIBAN2	Protein Niban 2	NM_001326382.1
A16G0854	22.00	14.93	LOC113060153	A0A6P6LMU6_CARAU uncharacterized protein LOC113060153	
B18G0801	21.99	14.12	RAB27A	Ras-related protein Rab-27A	NM_001030257.1
B19G0710	21.99	36.33	HDAC1-B	Probable histone deacetylase 1-B	XM_005159592.5
A2G0461	21.99	27.53	RAB2A	Ras-related protein Rab-2A	NM_201454.2
A1G0476	21.98	13.82	KDM2A	Lysine-specific demethylase 2A	NM_001081692.2
A16G0666	21.97	20.71	MYZAP	Myocardial zonula adherens protein	NM_001080001.1
A25G0341	21.97	11.27	TES	Testin	NM_205720.2
B11G0661	21.96	15.91	CAMTA1	Calmodulin-binding transcription activator 1	XM_021479696.2
B14G0109	21.96	13.83	ATOX1	Copper transport protein ATOX1	
A8G0975	21.96	9.71	FGD3	FYVE, RhoGEF and PH domain-containing protein 3	NM_131556.2
A20G0472	21.96	4.16	RHOV	Rho-related GTP-binding protein RhoV	NM_001012250.2
B7G0148	21.96	18.91	CYB5A	Cytochrome b5	NM_212876.1
A19G0506	21.96	23.45	PSMB2	Proteasome subunit beta type-2	
B22G0518	21.96	9.99	ASPN	Asporin	NM_131713.2
A8G0231	21.95	12.47	DENND2D	DENN domain-containing protein 2D	NM_001430991.1
A13G0437	21.92	6.64	RET	Proto-oncogene tyrosine-protein kinase receptor Ret	NM_001004530.1
B3G0645	21.92	36.76	ARF1	ADP-ribosylation factor 1	NM_201504.1
A22G0039	21.92	12.26	BYSL	Bystin	NM_201106.1
B9G0475	21.92	7.13	ARL4C	ADP-ribosylation factor-like protein 4C	NM_213248.1
B11G0400	21.92	11.81	MAF1	Repressor of RNA polymerase III transcription MAF1 homolog	NM_001004595.1
A1G0432	21.91	15.80	BDH2	reductase SDR family member 6	XM_005160068.5
A8G0170	21.90	7.49	ABT1	Activator of basal transcription 1	NM_001020547.1
A5G0689	21.90	7.98	IL13RA2	Interleukin-13 receptor subunit alpha-2	NM_001113731.3
A24G0552	21.90	13.49	CCR9	C-C chemokine receptor type 9	XM_021473752.2
B11G0717	21.89	19.16	ACYP2	Acylphosphatase-2	NM_212748.1
A2G0067	21.89	9.55	EXOSC4	Exosome complex component RRP41	NM_200739.3
A1G0570	21.88	19.48	GTF2F2	General transcription factor IIF subunit 2	NM_001002127.2

B8G0596	21.88	28.93	PPDPFA	Pancreatic progenitor cell differentiation and proliferation factor A	NM_200079.1
B4G0422	21.88	9.44	PKP2	Plakophilin-2	NM_001113433.1
A25G0147	21.87	8.15	ARPP19	cAMP-regulated phosphoprotein 19	XM_009297776.4
A19G0249	21.85	10.17	ABCF1	ATP-binding cassette sub-family F member 1	NM_213186.1
A1G1075	21.85	20.46	SMIM14	Small integral membrane protein 14	
B8G0407	21.84	1.71	VGLL4	Transcription cofactor vestigial-like protein 4	NM_001079998.1
B2G0441	21.83	10.11	GADD45B	Growth arrest and DNA damage-inducible protein GADD45 beta	NM_001012386.2
B14G0807	21.83	7.01	RHOG	Rho-related GTP-binding protein RhoG	
B19G0195	21.83	24.10	CSNK2A1	Casein kinase II subunit alpha	NM_001002164.1
B3G1510	21.82	14.76	TC1A	Transposable element Tc1 transposase	
A19G0082	21.80	18.29	SERINC1	Serine incorporator 1	NM_001045182.1
A25G0566	21.80	20.13	KRAS	GTPase KRas	NM_001362494.1
B24G0214	21.78	11.49	SNAI2	Zinc finger protein SNAI2	NM_001008581.1
A9G0826	21.78	12.73	PRPF40A	Pre-mRNA-processing factor 40 homolog A	NM_198356.1
A12G0606	21.77	14.79	LSM5	U6 snRNA-associated Sm-like protein LSm5	NM_001100438.2
A1G0428	21.77	22.26	SUCLG1	GDP-forming] subunit alpha, mitochondrial	NM_001002577.2
B24G0069	21.76	35.33	IGLC3	Ig lambda-3 chain C region	XM_068217596.1
B24G0063	21.76	35.33	IGLC3	Ig lambda-3 chain C region	XM_068217596.1
B22G0080	21.76	14.63	CRY1	Cryptochrome-1	XM_009295768.3
A3G0924	21.75	35.54	MVP	Major vault protein	XM_005163803.5
A5G0386	21.74	19.13	TP53	Cellular tumor antigen p53	NM_001271820.1
B20G0499	21.73	13.01	RRM2	Ribonucleoside-diphosphate reductase subunit M2	NM_131450.3
A22G0635	21.73	12.04	SEC13	Protein SEC13 homolog	NM_213335.1
B3G0716	21.73	27.80	LOC107601242	A0A672PI05_SINGR Uncharacterized LOC107601242	
A3G1024	21.73	21.83	CBX1	Chromobox protein homolog 1	NM_199746.2
B13G0837	21.72	2.47	ITGA9	Integrin alpha-9	XM_068212987.1
A15G0866	21.72	34.85	TC1A	Transposable element Tc1 transposase	
A20G0339	21.72	19.06	HNRNPU	Heterogeneous nuclear ribonucleoprotein U	NM_001033595.2
A16G0254	21.71	13.36	CMTM6	CKLF-like MARVEL transmembrane domain-containing protein 6	
B10G0126	21.69	10.61	INIP	SOSS complex subunit C	NM_200219.2
A8G0347	21.69	8.03	CA021	Uncharacterized protein C1orf21 homolog	NM_001004593.1
B2G1110	21.68	10.59	RGS18	Regulator of G-protein signaling 18	
A13G0786	21.68	20.77	PHF10	PHD finger protein 10	XM_005157040.5
B5G0483	21.67	5.66	HPGD	15-hydroxyprostaglandin dehydrogenase [NAD(+)]	
B22G0084	21.67	11.59	ZNF76	Zinc finger protein 76	XM_009295760.4
B20G0437	21.67	16.87	CDCA4	Cell division cycle-associated protein 4	NM_001008580.1
A19G0179	21.65	11.75	RPA3	Replication protein A 14 kDa subunit	

B4G0936	21.64	9.27	DPX16_1179	A0A3N0XUS6_ANAGA Zinc finger protein 569	XM_021468269.2
A7G0959	21.64	14.30	TMEM256	Transmembrane protein 256	
A16G0482	21.64	4.79	GPATCH4	G patch domain-containing protein 4	NM_001199720.1
B12G0008	21.63	22.12	PARG	Poly(ADP-ribose) glycohydrolase	XR_011017502.1
B4G0433	21.62	42.24	ETNK1	Ethanolamine kinase 1	XM_009300314.4
A24G0531	21.62	4.64	LOC107718923	A0A673K0T8_9TELE Yae1 domain-containing protein 1-like	NM_213142.1
B11G0567	21.62	10.93	TDO2B	Tryptophan 2,3-dioxygenase B	NM_001386332.1
A23G0668	21.62	25.11	TUBA1C	Tubulin alpha-1C chain	NM_194388.2
A20G0744	21.60	19.22	PSMA6	Proteasome subunit alpha type-6	NM_001002589.1
A22G0213	21.59	4.86	HTD2	Hydroxyacyl-thioester dehydratase type 2, mitochondrial	NM_001020692.2
A23G0550	21.59	11.03	G5714_022550	A0A7J6BPC1_9TELE TAR DNA-binding protein 43	NM_001362353.1
A9G0164	21.59	39.91	TFPI	Tissue factor pathway inhibitor	
A6G0326	21.59	1.96	GJC1	Gap junction gamma-1 protein	XM_021476749.2
A7G0579	21.58	13.22	SLC8B1	calcium exchanger protein	XM_021477930.2
B16G0224	21.57	12.89	DBF4	Protein DBF4 homolog A	NM_001135135.1
B18G0105	21.57	17.00	PSMD8	26S proteasome non-ATPase regulatory subunit 8	NM_001002131.2
B11G0608	21.56	13.55	LDLRAD2	Low-density lipoprotein receptor class A domain-containing protein 2	
A10G0759	21.56	15.14	PTBP3	Polypyrimidine tract-binding protein 3	XM_009305172.4
A13G0779	21.56	35.47	CST7	Cystatin-F	NM_001077581.1
B5G0687	21.55	18.75	FLOT2A	Flotillin-2a	NM_213075.2
B23G0328	21.55	23.60	HSD17B10	3-hydroxyacyl-CoA dehydrogenase type-2	NM_001006098.1
B13G0262	21.55	6.45	BTN2A1	Butyrophilin subfamily 2 member A1	NM_214769.1
A19G0855	21.54	15.11	SNRPD1	Small nuclear ribonucleoprotein Sm D1	NM_173252.2
B25G0749	21.53	28.01	SNX1	Sorting nexin-1	NM_001128671.2
B7G0159	21.53	29.32	SWAP70	Switch-associated protein 70	NM_001362396.1
B14G0127	21.53	11.27	POLR2G	DNA-directed RNA polymerase II subunit RPB7	NM_199669.1
B12G0024	21.50	26.12	FH	Fumarate hydratase, mitochondrial	NM_200963.1
B21G0055	21.49	35.85	IK	Protein Red	XM_068215781.1
B16G0058	21.49	2.89	SMPD5	Sphingomyelin phosphodiesterase 5	NM_001077615.1
B15G0778	21.49	10.92	LOC113071909	A0A6P6MXB4_CARAU uncharacterized protein LOC113071909	
B20G0921	21.48	21.89	PPP2R5C	threonine-protein phosphatase 2A 56 kDa regulatory subunit gamma isoform	XM_005160898.5
B16G0826	21.48	10.94	CDC42EP5	Cdc42 effector protein 5	NM_001326377.1
A2G0274	21.47	12.32	CWC25	Pre-mRNA-splicing factor CWC25 homolog	NM_213462.3
B11G0114	21.46	33.57	SMIM7	Small integral membrane protein 7	NM_001199912.1
A11G0399	21.46	26.23	PPCS	Phosphopantothenate--cysteine ligase	NM_199686.2
A1G0715	21.45	17.00	GAS6	Growth arrest-specific protein 6	NM_199978.1

A11G0581	21.45	28.85	SARG	Specifically androgen-regulated gene protein	NM_001001818.2
A17G0906	21.44	29.64	DYNC1H1	Cytoplasmic dynein 1 heavy chain 1	NM_001042745.1
B16G0618	21.43	5.28	MYLIPB	E3 ubiquitin-protein ligase MYLIP-B	NM_001080022.2
A18G0012	21.43	9.48	RAP2A	Ras-related protein Rap-2a	NM_001001729.2
A19G0704	21.42	11.05	NUGGC	Nuclear GTPase SLIP-GC	XM_017352166.3
B5G0040	21.42	51.72	GNG10	G(O) subunit gamma-10	NM_001205026.2
B21G0654	21.42	19.82	IDH3B	Isocitrate dehydrogenase [NAD] subunit beta, mitochondrial	XM_017352767.3
A9G0371	21.42	27.85	ROHU_018720	A0A498N7Z7_LABRO Cyclic AMP receptor A	
B7G0779	21.41	30.30	LOC113049489	A0A6P6K5R6_CARAU vicilin-like seed storage protein At2g18540 isoform X1	NM_001386784.1
A21G0261	21.41	6.03	NIBAN2	Protein Niban 2	NM_001080586.1
B10G0551	21.41	2.12	ADGRG2	Adhesion G-protein coupled receptor G2	XM_009305521.4
B6G0231	21.41	18.15	WIPF1	WASL-interacting protein family member 1	XM_068221901.1
A13G0557	21.40	43.75	HABP2	Hyaluronan-binding protein 2	NM_001328090.1
A8G0239	21.39	15.80	CCDC124	Coiled-coil domain-containing protein 124	NM_200565.2
B4G0369	21.39	24.25	YAF2	YY1-associated factor 2	NM_001045268.1
B2G0845	21.38	10.18	TOX	Thymocyte selection-associated high mobility group box protein TOX	NM_001256694.1
A10G0685	21.38	8.24	FAM136A	Protein FAM136A	NM_213400.1
B14G0742	21.38	12.81	PRPF19	Pre-mRNA-processing factor 19	NM_201467.1
B17G0493	21.37	8.17	MRPL57	Ribosomal protein 63, mitochondrial	NM_200775.1
B9G0428	21.37	12.79	MRPL30	39S ribosomal protein L30, mitochondrial	
A20G0445	21.36	10.11	RRM2	Ribonucleoside-diphosphate reductase subunit M2	NM_131450.3
B21G0442	21.34	7.18	MRPS12	28S ribosomal protein S12, mitochondrial	NM_001386273.1
B25G0900	21.34	4.77			
B22G0968	21.33	10.03	SEC13	Protein SEC13 homolog	NM_213335.1
B21G0020	21.33	8.69	MACROH2A1	Core histone macro-H2A.1	NM_001040361.1
B5G1185	21.33	10.23	CASP8	Caspase-8	
B18G0205	21.33	10.51	CMC2	COX assembly mitochondrial protein 2 homolog	NM_001282139.1
A23G0623	21.33	11.26	TAF8	Transcription initiation factor TFIID subunit 8	XM_005162303.5
B25G0585	21.32	24.03	COQ9	Ubiquinone biosynthesis protein COQ9, mitochondrial	NM_001098746.1
A5G0817	21.31	9.27	SMN1	Survival motor neuron protein 1	NM_131191.1
B6G0152	21.31	15.08	FKBP11	Peptidyl-prolyl cis-trans isomerase FKBP11	NM_001012249.2
B4G0267	21.30	31.74	HSP90B1	Endoplasmin	NM_198210.2
A10G0465	21.29	5.82	SYTL2	Synaptotagmin-like protein 2	XM_009305477.4
B5G0021	21.29	20.15	FUBP3	Far upstream element-binding protein 3	NM_001007776.1
B17G0592	21.29	36.30	LIN52	Protein lin-52 homolog	NM_001077796.1
B6G0980	21.28	21.21	UBE2C	Ubiquitin-conjugating enzyme E2 C	NM_001037691.2
A7G1008	21.28	13.75	ACAP2	Arf-GAP with coiled-coil, ANK repeat and PH domain-containing protein 2	NM_001080579.1

B3G1095	21.27	7.67	LOC113046879	A0A6P6JXF2_CARAU uncharacterized protein LOC113046879 isoform X1	
A10G0734	21.27	9.33	ANXA3	Annexin A3	NM_001007302.2
B1G0407	21.26	3.43	LOC107745861	A0A673N030_9TELE Transmembrane protein 154-like	XM_005170905.4
A12G0371	21.25	6.93	COX7A2L	Cytochrome c oxidase subunit 7A-related protein, mitochondrial	
B16G1112	21.25	17.35	RPA2	Replication protein A 32 kDa subunit	NM_131711.2
B10G0317	21.25	157.44	PCOLCE2	Procollagen C-endopeptidase enhancer 2	NM_001030181.3
A5G0521	21.24	25.50	F11R	Junctional adhesion molecule A	NM_001004667.3
A3G1177	21.22	9.66	SI:CH211-110E21.3	E7FCK4_DANRE Si:ch211-110e21.3	NM_001302615.1
A3G1072	21.21	17.13	GRB2	Growth factor receptor-bound protein 2	NM_213035.1
A8G0235	21.21	11.06	ITGB4	Integrin beta-4	NM_001024386.1
A25G0292	21.21	13.34	ATP6V1E1	V-type proton ATPase subunit E 1	NM_001365440.1
B20G0123	21.20	19.35	PAPOLA	Poly(A) polymerase alpha	NM_205758.2
A15G0210	21.20	6.36	ST14	Suppressor of tumorigenicity 14 protein homolog	XR_011006502.1
B8G0056	21.20	12.44	BICD2	Protein bicaudal D homolog 2	XM_009304324.4
A20G0544	21.20	10.90	AIRC	Multifunctional protein ADE2	XM_068215380.1
B7G1210	21.19	16.21	TRMT10B	tRNA methyltransferase 10 homolog B	XM_017356915.3
A3G0162	21.19	3.52	EVPL	Envoplakin	XM_001922038.7
B14G0259	21.18	23.26	SASH3	SAM and SH3 domain-containing protein 3	XM_685351.10
A17G0695	21.18	12.07	TIMM9	Mitochondrial import inner membrane translocase subunit Tim9	NM_001159911.2
A2G0816	21.17	20.12	COPA	Coatomer subunit alpha	NM_001001941.2
A20G0092	21.17	24.72	ATP6V1D	V-type proton ATPase subunit D	NM_001167954.1
A5G1339	21.15	10.62	A0A3B4BVC7	A0A3B4BVC7_PYGNA Arginine and serine rich coiled-coil 2	NM_213280.1
B20G0017	21.14	24.10	SYNCRIP	Heterogeneous nuclear ribonucleoprotein Q	XM_009294527.2
B7G1126	21.13	18.63	ACAP1	Arf-GAP with coiled-coil, ANK repeat and PH domain-containing protein 1	NM_001080579.1
A2G0851	21.13	16.20	HNRNPC	C2	NM_213079.2
A12G0665	21.11	32.73	UQCRC2	Cytochrome b-c1 complex subunit 2, mitochondrial	NM_001001589.1
A3G0506	21.10	9.67	SHMT1	Serine hydroxymethyltransferase, cytosolic	NM_201046.1
B13G0476	21.09	20.44	HGSNAT	Heparan-alpha-glucosaminide N-acetyltransferase	NM_001004529.2
A5G1038	21.08	16.58	SCIMP	A0A6P6K8H4_CARAU SLP adapter and CSK-interacting membrane protein	
A2G1063	21.08	16.17	CDC34	Ubiquitin-conjugating enzyme E2 R1	NM_001002688.1
B2G0920	21.08	47.66	CRJ1A	Crystallin J1A	NM_001193469.1
A4G0340	21.08	19.08	LOC113048991	A0A6P6K6T2_CARAU uncharacterized protein LOC113048991 isoform X2	

A2G0235	21.08	21.15	SELENOT1A	Thioredoxin reductase-like selenoprotein T1a	NM_178290.6
B22G0707	21.07	10.31	RFXANK	DNA-binding protein RFXANK	NM_001130641.1
B24G0638	21.07	12.50	TXNDC5	Thioredoxin domain-containing protein 5	NM_213016.2
B21G0107	21.05	56.58	MPP1	55 kDa erythrocyte membrane protein	NM_214692.2
B23G0396	21.04	8.88	ARHGEF3	Rho guanine nucleotide exchange factor 3	XM_005162074.5
A23G0646	21.04	2.63	IL17RD	Interleukin-17 receptor D	XM_683049.8
A25G0023	21.04	32.75	TCB1	Transposable element Tcb1 transposase	XM_021471168.2
A23G0019	21.03	13.50	HM13	Minor histocompatibility antigen H13	XM_005161890.5
A6G0524	21.02	12.28	ACTL6A	Actin-like protein 6A	NM_173240.1
A5G0192	21.02	10.03	SDF2L1	Stromal cell-derived factor 2-like protein 1	NM_001003730.1
A16G0114	21.02	23.26	RAD21A	Double-strand-break repair protein rad21 homolog A	NM_199595.1
A15G0103	21.02	40.63	NLRP3	NACHT, LRR and PYD domains-containing protein 3	XM_021470830.2
A2G0126	21.01	44.91	PFN2	Profilin-2	XM_005171152.3
A3G0824	21.01	11.12	CARHSP1	Calcium-regulated heat stable protein 1	NM_200562.2
A21G0858	21.00	24.55	TCF7L2	Transcription factor 7-like 2	XM_068215845.1
A7G1038	20.99	23.96	IL13RA1	Interleukin-13 receptor subunit alpha-1	
A21G0249	20.98	8.88	SNRPD3	Small nuclear ribonucleoprotein Sm D3	NM_199760.1
A20G0388	20.98	2.82	MYCN	N-myc proto-oncogene protein	NM_212614.2
B10G0353	20.96	15.14	PAXBP1	PAX3- and PAX7-binding protein 1	NM_001080183.2
B6G0919	20.96	71.85	SDC4	Syndecan-4	NM_001048149.2
A7G1072	20.95	15.57	EML3	Echinoderm microtubule-associated protein-like 3	XM_003198926.6
A16G0830	20.94	10.52	HIBADH	3-hydroxyisobutyrate dehydrogenase, mitochondrial	NM_201160.1
A21G0559	20.94	21.05	FKBP2	Peptidyl-prolyl cis-trans isomerase FKBP2	NM_001004677.2
B22G0755	20.93	7.64	ZBTB7A	Zinc finger and BTB domain-containing protein 7A	XM_005170531.5
A5G0800	20.93	12.05	FLOT2A	Flotillin-2a	XM_005165263.5
A16G0887	20.92	6.20	GRHL2	Grainyhead-like protein 2 homolog	NM_001326533.1
B21G0197	20.91	4.89	RBMX2	RNA-binding motif protein, X-linked 2	NM_001025166.1
A7G0903	20.90	22.03	PTGR1	Prostaglandin reductase 1	NM_001008651.1
B9G0201	20.90	23.70	TFPI	Tissue factor pathway inhibitor	NM_001309803.1
A19G0212	20.90	8.12	POLR1H	DNA-directed RNA polymerase I subunit RPA12	
B4G0059	20.90	25.46	SLC38A2	Sodium-coupled neutral amino acid symporter 2	NM_001045104.1
A7G0953	20.89	20.28	G5714_008380	A0A7J6CVI7_9TELE Uncharacterized protein	XM_002662815.5
A12G0836	20.89	24.24	TOB1	Protein Tob1	NM_212974.1
A8G0302	20.87	15.48	MRPS36	Alpha-ketoglutarate dehydrogenase component 4	NM_001113608.1
B19G0967	20.86	17.59	PSMD3	26S proteasome non-ATPase regulatory subunit 3	NM_200572.1
B3G0961	20.86	20.94	IFI35	Interferon-induced 35 kDa protein homolog	NM_001386657.1

A8G0193	20.85	15.49	MAPK13	Mitogen-activated protein kinase 13	XM_001337797.9
A2G0884	20.85	23.61	COPB2	Coatomer subunit beta'	NM_001001940.1
B5G1186	20.84	7.88	AIFM1	Apoptosis-inducing factor 1, mitochondrial	XM_005165127.4
A5G1147	20.84	20.41	TMEM272	Transmembrane protein 272	
A1G1114	20.84	17.21	PDCD4	Programmed cell death protein 4	XM_005159885.5
B8G0211	20.83	17.42	OGDH	2-oxoglutarate dehydrogenase complex component E1	XM_005172468.5
A6G0805	20.83	64.27	TARDBP	TAR DNA-binding protein 43	XR_002458992.2
A6G0508	20.83	9.94	TBC1D23	TBC1 domain family member 23	XM_005165941.5
A8G0011	20.82	84.49	CDO1	Cysteine dioxygenase type 1	NM_200741.1
B8G0899	20.82	8.07	TSR2	Pre-rRNA-processing protein TSR2 homolog	NM_173284.3
A16G0351	20.81	20.26	TCEA3	Transcription elongation factor A protein 3	XM_009292451.4
A1G1058	20.80	47.70	UCHL1	Ubiquitin carboxyl-terminal hydrolase isozyme L1	NM_201477.1
A19G0467	20.80	17.71	ELOA	Elongin-A	NM_213455.1
B24G0398	20.79	13.19	NDUFB4	NADH dehydrogenase [ubiquinone] 1 beta subcomplex subunit 4	
B3G0713	20.78	18.86	LOC107601242	A0A672PI05_SINGR Uncharacterized LOC107601242	NM_001423737.1
A16G1041	20.77	7.35	MRPL51	39S ribosomal protein L51, mitochondrial	
B11G0134	20.77	20.15	DDX19B	ATP-dependent RNA helicase DDX19B	
A7G1042	20.77	32.95	NDUFS2	NADH dehydrogenase [ubiquinone] iron-sulfur protein 2, mitochondrial	NM_001020645.2
B14G0011	20.77	30.14	WDR1	WD repeat-containing protein 1	NM_201039.1
A6G0775	20.77	16.92	TCTA	T-cell leukemia translocation-altered gene protein homolog	NM_001002585.2
B5G0393	20.76	30.40	ATP6V1G1	V-type proton ATPase subunit G 1	NM_199934.1
A5G0019	20.76	0.13	ASS1	Argininosuccinate synthase	NM_001004603.1
B15G0694	20.75	10.03	LSR	Lipolysis-stimulated lipoprotein receptor	XM_005157631.5
B5G0668	20.75	16.18	MCCC2	Methylcrotonoyl-CoA carboxylase beta chain, mitochondrial	NM_212927.1
B25G0817	20.74	24.93	CIB1	Calcium and integrin-binding protein 1	
B12G0360	20.73	15.92	ZFP36L2-A	mRNA decay activator protein ZFP36L2-A	NM_207055.2
B13G0626	20.73	5.63	CCDC28B	Coiled-coil domain-containing protein 28B	
B12G0076	20.72	17.97	EXOC7	Exocyst complex component 7	XM_005172976.5
B5G0346	20.72	17.97	TRAF2	TNF receptor-associated factor 2	XM_005165499.5
B16G0114	20.72	30.56	RAB5A	Ras-related protein Rab-5A	NM_200970.1
B1G0039	20.71	8.13	MRPL16	39S ribosomal protein L16, mitochondrial	NM_001007764.2
B6G0180	20.70	16.85	DAB2IP	Disabled homolog 2-interacting protein	XM_005165747.5
A14G0037	20.69	12.98	DELE1	DAP3-binding cell death enhancer 1	
A20G0463	20.69	13.23	MAX	Protein max	XM_005160619.5
A4G0440	20.68	17.91	RAB21	Ras-related protein Rab-21	NM_001076665.1
B19G0711	20.68	86.56	MARCKSL1B	MARCKS-related protein 1-B	NM_213223.1
A1G0819	20.67	4.17	FAM83G	Protein FAM83G	XM_005170874.5

B12G0163	20.67	16.12	CHMP6	Charged multivesicular body protein 6	NM_001003874.1
B19G0450	20.67	25.07	VPS28	Vacuolar protein sorting-associated protein 28 homolog	XM_009294451.4
B25G0491	20.66	40.21	CAV1	Caveolin-1	NM_212651.1
B1G0348	20.65	20.61	MED28	Mediator of RNA polymerase II transcription subunit 28	NM_001007770.1
B3G0395	20.64	6.53	MRPL12	39S ribosomal protein L12, mitochondrial	NM_001017696.1
B21G0803	20.64	4.81	A33	Zinc-binding protein A33	
B23G0388	20.63	26.32	TAF13	Transcription initiation factor TFIID subunit 13	NM_001004665.1
B4G0139	20.62	11.51	APEX1	DNA-(apurinic or apyrimidinic site) endonuclease	NM_001328578.1
B6G0653	20.62	65.41	STAT1	beta	NM_001271801.1
B5G0428	20.61	30.97	NRGN	Neurogranin	NM_001302620.1
B2G0659	20.61	22.51	MLRN	Myosin regulatory light chain 2, smooth muscle minor isoform	NM_001130589.2
B6G0141	20.61	17.01	ATG3	Ubiquitin-like-conjugating enzyme ATG3	NM_200022.1
A13G0121	20.60	7.91	LRRFIP2	Leucine-rich repeat flightless-interacting protein 2	NM_001256652.1
B13G0797	20.60	10.96	RUNX3	Runt-related transcription factor 3	NM_001256652.1
B8G0186	20.60	23.60	DBNL	Drebrin-like protein	XM_005167337.5
A14G0482	20.60	21.97	UBE2D2	Ubiquitin-conjugating enzyme E2 D2	NM_199952.2
A3G0906	20.59	60.49	ARL5B	ADP-ribosylation factor-like protein 5B	NM_200846.1
A3G1227	20.59	7.83	IGL1	Immunoglobulin lambda-1 light chain	
B3G1071	20.58	20.93	MRPS34	28S ribosomal protein S34, mitochondrial	NM_001007376.2
A19G0906	20.57	19.24	GSTA	Glutathione S-transferase A	
A11G0408	20.57	32.33	EFCC1	EF-hand and coiled-coil domain-containing protein 1	NM_001044993.1
B4G0141	20.57	19.55	G5714_005088	A0A7J6D757_9TELE Uncharacterized protein	NM_001025503.1
B16G0398	20.57	8.93	GNL2	Nucleolar GTP-binding protein 2	NM_213224.1
A7G0235	20.56	38.48	IPO7	Importin-7	NM_200905.3
A6G0526	20.56	10.07	NDUFB5	NADH dehydrogenase [ubiquinone] 1 beta subcomplex subunit 5, mitochondrial	NM_001105596.1
A11G0190	20.54	17.26	BTF3L4	Transcription factor BTF3 homolog 4	
B22G1054	20.54	3.10	ALPI	Intestinal-type alkaline phosphatase	
A9G0124	20.52	3.85			NM_001005956.2
B16G0474	20.52	21.43	CSNK2A1	Casein kinase II subunit alpha	NM_131252.1
A1G0190	20.50	12.09	KHSRP	Far upstream element-binding protein 2	NM_001089428.2
B9G0773	20.50	0.52	SCEL	Sciellin	NM_001005304.1
B13G0887	20.49	8.71	PTK7	Inactive tyrosine-protein kinase 7	NM_001017621.1
A16G0828	20.49	47.84	TAX1BP1B	Tax1-binding protein 1 homolog B	NM_212664.2
A11G0358	20.49	21.35	PHF20	PHD finger protein 20	NM_201303.1
B12G0447	20.49	10.53	PHF5A	PHD finger-like domain-containing protein 5A	NM_173266.1
A2G0711	20.48	16.44	CACYBP	Calcyclin-binding protein	NM_212887.1
B2G0631	20.48	6.45	NOL7	Nucleolar protein 7	

A3G0198	20.48	29.29	BTN3A1	Butyrophilin subfamily 3 member A1	XM_021472967.2
B23G0630	20.48	8.83	MYB	Transcriptional activator Myb	NM_131266.2
B11G0048	20.48	3.59	MYBL2	Myb-related protein B	
A22G0513	20.48	13.72	UCHL5	Ubiquitin carboxyl-terminal hydrolase isozyme L5	NM_213084.1
A18G0162	20.46	4.73	SEMA6D	Semaphorin-6D	XM_068214767.1
B3G0101	20.46	9.26	GIMAP7	GTPase IMAP family member 7	
A14G0106	20.46	18.91	MATR3	Matrin-3	XM_068213213.1
B7G0774	20.45	8.52	TRADD	Tumor necrosis factor receptor type 1-associated DEATH domain protein	NM_131607.2
B21G0796	20.45	14.82	CDK9	Cyclin-dependent kinase 9	NM_212591.2
B5G0046	20.45	6.04	PPP1CC-A	threonine-protein phosphatase PP1-gamma catalytic subunit A	XM_695376.8
B7G0201	20.44	9.35	BAX	Apoptosis regulator BAX	NM_001076585.1
B1G0117	20.43	8.83	PCNP	PEST proteolytic signal-containing nuclear protein	NM_001326716.1
A3G0104	20.43	9.54	GIMAP7	GTPase IMAP family member 7	XM_068217815.1
B23G0699	20.43	6.96	ACP1	Low molecular weight phosphotyrosine protein phosphatase	NM_001244782.1
B16G0948	20.42	5.30	G5714_016378	A0A7J6CA68_9TELE Uncharacterized protein	
B20G0945	20.41	11.01	ZNF410	Zinc finger protein 410	XM_005170226.5
B1G0719	20.41	23.71	SUCLG1	GDP-forming] subunit alpha, mitochondrial	NM_001002577.2
A21G0763	20.41	128.40	ALDOC	Fructose-bisphosphate aldolase C	NM_194384.1
B2G0067	20.40	26.17	UPF1	Regulator of nonsense transcripts 1	NM_213474.1
B17G0425	20.39	18.21	MDH1	Malate dehydrogenase, cytoplasmic	NM_001355553.1
B24G0062	20.39	33.61	IGKV4-1	Immunoglobulin kappa variable 4-1	NM_001045408.1
A10G0744	20.39	8.63	URM1	Ubiquitin-related modifier 1	
B2G0760	20.38	7.33	TNIK	TRAF2 and NCK-interacting protein kinase	XM_005163390.5
B10G0513	20.37	46.84	IL2RG	Cytokine receptor common subunit gamma	NM_001128271.1
B7G0130	20.37	44.14	GABARAP	Gamma-aminobutyric acid receptor-associated protein	NM_001386387.1
B21G0311	20.36	7.97	CRCP	DNA-directed RNA polymerase III subunit RPC9	NM_001045373.2
B10G0717	20.36	21.67	ARRDC3	Arrestin domain-containing protein 3	NM_001004605.1
B17G0181	20.36	23.60	SRP54	Signal recognition particle 54 kDa protein	NM_200988.2
B7G0080	20.35	11.62	MRPL21	39S ribosomal protein L21, mitochondrial	
B15G0060	20.35	26.35	SBDS	Ribosome maturation protein SBDS	
A10G0619	20.33	14.15	LETM2	LETM1 domain-containing protein LETM2, mitochondrial	XM_001339351.9
A15G0578	20.32	12.87	RPS6KB1	Ribosomal protein S6 kinase beta-1	NM_213076.1
B19G0481	20.32	54.70	PPT1	Palmitoyl-protein thioesterase 1	XM_017352221.3
B25G0333	20.32	13.41	IDH3A	Isocitrate dehydrogenase [NAD] subunit alpha, mitochondrial	NM_200951.2
B8G0374	20.31	38.40	HPD	4-hydroxyphenylpyruvate dioxygenase	NM_001003742.1
A24G0309	20.30	11.96	TFRC	Transferrin receptor protein 1	NM_001009918.2

B16G0932	20.30	7.67	GTF3C6	General transcription factor 3C polypeptide 6	
B1G0860	20.30	6.86	ESF1	ESF1 homolog	NM_199745.2
A4G0734	20.29	23.45	TMBIM4	Protein lifeguard 4	NM_213138.2
B23G0245	20.28	5.73	FOXP1B	Forkhead box protein P1-B	XM_005161959.4
B23G0597	20.28	11.63	LOC107708822	A0A673MLE6_9TELE Coiled-coil domain-containing protein 50-like	NM_001430812.1
B5G0749	20.28	36.87	CNOT6L	CCR4-NOT transcription complex subunit 6-like	XM_005165315.5
B18G0409	20.28	87.56	IDH2	Isocitrate dehydrogenase [NADP], mitochondrial	NM_199564.1
B17G0258	20.28	14.84	STYX-A	tyrosine-interacting protein A	NM_001024390.1
B18G0804	20.28	0.52	HAAF	amebocyte aggregation factor	
B5G0894	20.27	12.02	POLE3	DNA polymerase epsilon subunit 3	NM_200801.1
B2G0044	20.27	12.43	MAP2K2	Dual specificity mitogen-activated protein kinase kinase 2	XM_021478261.2
B22G0064	20.27	8.51	LOC113040334	A0A6P6J320_CARAU proline-rich extensin-like protein EPR1	XM_009303330.4
A5G0273	20.24	11.23	GLTP	Glycolipid transfer protein	NM_001099988.1
B11G0158	20.24	36.90	PEX11G	Peroxisomal membrane protein 11C	NM_001007775.2
A10G0662	20.24	8.47	DRG1	Developmentally-regulated GTP-binding protein 1	NM_200038.1
B21G0973	20.23	0.11	BRIX1	Ribosome biogenesis protein BRX1 homolog	NM_001172556.1
A6G0348	20.23	10.65	THUMPD3	tRNA (guanine(6)-N2)-methyltransferase THUMP3	XM_068221753.1
A12G0327	20.23	13.73	PSME3	Proteasome activator complex subunit 3	NM_131376.2
B9G0043	20.23	52.51	NLRC3	Protein NLRC3	XM_009295700.4
B21G0668	20.21	8.20	PHPT1	14 kDa phosphohistidine phosphatase	NM_001003566.2
A4G0485	20.20	0.62	B4GALNT3	Beta-1,4-N-acetylgalactosaminyltransferase 3	XM_684908.10
B18G0170	20.20	15.21	TMOD3	Tropomodulin-3	XM_068214761.1
A25G0606	20.20	64.95	LDHA	L-lactate dehydrogenase A chain	NM_131246.1
B10G0635	20.19	19.33	STT3A	Dolichyl-diphosphooligosaccharide--protein glycosyltransferase subunit STT3A	NM_201458.2
B2G0732	20.19	19.95	ASPH	asparaginyl beta-hydroxylase	XM_005163382.5
A6G0762	20.18	19.01	EMC3	ER membrane protein complex subunit 3	NM_213505.2
A18G0172	20.18	21.18	LEO1	RNA polymerase-associated protein LEO1	NM_207067.1
B3G0953	20.18	13.63	NUCB1	Nucleobindin-1	XM_005163951.5
A21G0121	20.17	15.87	PDCL	Phosducin-like protein	NM_199647.2
A17G0449	20.17	2.57	GJB3	Gap junction beta-3 protein	NM_001017685.1
B12G0209	20.17	15.33	CYBC1	Cytochrome b-245 chaperone 1 homolog	
B5G1133	20.17	16.04	GPS2	G protein pathway suppressor 2	NM_001431107.1
A8G0277	20.17	11.97	DNTTIP2	Deoxynucleotidyltransferase terminal-interacting protein 2	NM_001145786.1
A12G0429	20.16	12.01	SNF8	Vacuolar-sorting protein SNF8	NM_001007412.1
B19G0360	20.16	7.22	NUTF2	Nuclear transport factor 2	NM_001003598.2
B8G0884	20.16	29.88	BCAP31	B-cell receptor-associated protein 31	NM_200092.1
B19G0745	20.15	15.36	NELFE	Negative elongation factor E	XM_005170027.5

A1G0026	20.14	12.01	MFAP4	Microfibril-associated glycoprotein 4	XM_009296176.4
A7G1192	20.14	15.64	CT027	UPF0687 protein C20orf27 homolog	NM_001436955.1
A6G0478	20.14	3.78	BOKA	Bcl-2-related ovarian killer protein homolog A	XM_005165921.5
A5G0467	20.14	16.73	CYB561A3A	Lysosomal membrane ascorbate-dependent ferrireductase CYB561A3	XM_068221038.1
A5G0955	20.12	9.28	PPIL3	Peptidyl-prolyl cis-trans isomerase-like 3	NM_001002146.1
B18G0161	20.12	6.29	SEMA6D	Semaphorin-6D	XM_068214767.1
A21G0622	20.12	39.99	SNX12	Sorting nexin-12	NM_001145893.1
A19G0236	20.11	7.59	MACO1A	Macoilin-1	NM_200319.2
B14G0048	20.11	8.25	MED19A	Mediator of RNA polymerase II transcription subunit 19-A	NM_001076591.1
B16G0812	20.11	8.09	PITPNC1	Cytoplasmic phosphatidylinositol transfer protein 1	XM_005157890.5
A19G0931	20.11	15.64	ENPP2	phosphodiesterase family member 2	XM_017352386.1
A16G0775	20.10	11.84	RNF115	E3 ubiquitin-protein ligase RNF115	NM_001080073.1
B21G0600	20.09	7.81	MRPS18C	28S ribosomal protein S18c, mitochondrial	NM_001045190.2
A3G0116	20.09	14.28	PLBD1	Phospholipase B-like 1	NM_001130791.1
A1G0500	20.09	16.51	NAA15	N-alpha-acetyltransferase 15, NatA auxiliary subunit	NM_203321.2
A17G0670	20.09	10.84	PSMA6	Proteasome subunit alpha type-6	NM_153655.2
B22G0922	20.08	8.42	ABI3BP	Target of Nesh-SH3	XM_068216228.1
A7G1144	20.07	11.73	SPG21	Maspardin	NM_213395.1
A3G0596	20.07	32.77	PRKAR1A	cAMP-dependent protein kinase type I-alpha regulatory subunit	NM_001009989.3
A11G0174	20.06	7.97	WIPF2	WASL-interacting protein family member 2	NM_213470.1
A7G0908	20.06	20.64	NEDD8	NEDD8	NM_001002557.1
B8G0057	20.06	44.72	GNB1	G(T) subunit beta-1	NM_212609.1
B19G0626	20.06	22.97	ELMO1	Engulfment and cell motility protein 1	XM_068215106.1
B16G0901	20.05	55.70	TAX1BP1B	Tax1-binding protein 1 homolog B	NM_212664.2
B19G0770	20.04	10.88	RPA3	Replication protein A 14 kDa subunit	
B14G0838	20.03	22.21	COMMD5	COMM domain-containing protein 5	
B23G0686	20.03	19.04	FAM50A	Protein FAM50A	NM_001017636.1
B16G0840	20.03	13.34	RNF115	E3 ubiquitin-protein ligase RNF115	NM_001080073.1
A2G0197	20.03	9.48	DCUN1D1	DCN1-like protein 1	NM_199772.1
B22G0417	20.03	6.35	LOC113078163	A0A6P6NAM5_CARAU uncharacterized protein LOC113078163	XM_021469541.2
A11G0045	20.03	14.97	RBM34	RNA-binding protein 34	NM_001008732.1
B4G0871	20.02	6.79	MS4A15	Membrane-spanning 4-domains subfamily A member 15	XM_003199992.6
A7G0126	20.00	10.10			
B6G0448	20.00	12.75	DR1	Protein Dr1	NM_001077314.2
A23G0819	19.99	11.90	LOC113065423	A0A6P6M6V6_CARAU uncharacterized protein LOC113065423 isoform X2	XM_009297139.4

B23G0033	19.99	11.66	NSMCE3	Non-structural maintenance of chromosomes element 3 homolog	NM_001282090.1
B15G0430	19.98	18.51	ALDH3A2	Aldehyde dehydrogenase family 3 member A2	XM_009291620.4
A8G0368	19.97	16.25	MOB3A	MOB kinase activator 3A	NM_199716.1
B22G0231	19.96	39.08	G5714_021281	A0A7J6BS23_9TELE Uncharacterized protein	
B17G0468	19.96	11.08	LCK	Tyrosine-protein kinase Lck	NM_001040328.1
B23G0705	19.96	34.24	TUBA1C	Tubulin alpha-1C chain	NM_194388.2
A17G0518	19.95	1.12	MTLN	Mitoregulin	XM_068218872.1
B3G0460	19.95	6.77	CHCHD5	Coiled-coil-helix-coiled-coil-helix domain-containing protein 5	
A3G0277	19.95	15.79	ILF3	Interleukin enhancer-binding factor 3 homolog	XM_005163741.4
B18G0325	19.94	20.80	LOC113118501	A0A6P6RCM1_CARAU galaxin-like isoform X2	XM_068214925.1
B23G0363	19.94	13.99	SMIM4	Small integral membrane protein 4	
B14G0482	19.93	6.42	DOK3	Docking protein 3	
B7G0849	19.92	24.36	ARR1	Arrestin red cell isoform 1	NM_201124.1
B5G0052	19.91	1.98	PLA2G4C	Cytosolic phospholipase A2 gamma	XM_068221055.1
B2G1025	19.91	10.06	NCBP2AS2	Protein NCBP2AS2	
B16G0458	19.91	28.57	PTPN6	Tyrosine-protein phosphatase non-receptor type 6	NM_199960.1
B1G0474	19.91	10.18	U2AF1	Splicing factor U2AF 35 kDa subunit	NM_177479.2
A16G0474	19.90	10.45	ISG20L2	Interferon-stimulated 20 kDa exonuclease-like 2	NM_001103193.2
B3G0269	19.90	10.22			
A11G0685	19.90	32.60	KIF21B	Kinesin-like protein KIF21B	NM_001005938.1
B16G1117	19.89	15.33	BCKDHB	2-oxoisovalerate dehydrogenase subunit beta, mitochondrial	NM_001080653.1
B22G0361	19.88	66.51	G5714_021418	A0A7J6BQX2_9TELE Uncharacterized protein	XM_068216422.1
A13G0799	19.87	114.47	MCU	Calcium uniporter protein, mitochondrial	
B15G0932	19.87	40.85	CD3E	T-cell surface glycoprotein CD3 epsilon chain	
A19G0274	19.86	5.49	ACOT13	Acyl-coenzyme A thioesterase 13	XM_005159635.5
B11G0063	19.85	11.38	SLC2A10	Solute carrier family 2, facilitated glucose transporter member 10	NM_001002123.1
A16G0699	19.84	21.82	DEDD2	DNA-binding death effector domain-containing protein 2	NM_131602.2
A13G0650	19.84	5.75	LOXL3B	Lysyl oxidase homolog 3B	NM_131863.2
B7G1227	19.84	19.23	SF3B2	Splicing factor 3B subunit 2	NM_001105277.3
B14G0339	19.84	6.69	TIMM8A	Mitochondrial import inner membrane translocase subunit Tim8 A	
B24G0566	19.83	6.44	ELOC	Elongin-C	NM_001004673.1
B8G0389	19.82	18.96	LIPG	Endothelial lipase	NM_200128.1
B14G0578	19.82	5.64			NM_001305661.1
A14G0135	19.82	11.69	POLR2G	DNA-directed RNA polymerase II subunit RPB7	NM_199669.1
B14G0546	19.82	11.21	LOC107758528	A0A673KTD6_9TELE SH2 domain-containing protein 4A-like	XM_068213189.1

B21G0457	19.81	6.09	G5714_020566	A0A7J6BU60_9TELE IF rod domain-containing protein	
B10G0204	19.81	7.42	LOX	Protein-lysine 6-oxidase	NM_207089.1
A21G0289	19.80	6.45	PTPRA	Receptor-type tyrosine-protein phosphatase alpha	NM_131888.2
B4G0847	19.80	3.06	ALOX5	Polyunsaturated fatty acid 5-lipoxygenase	NM_001045331.1
A4G0528	19.79	32.73	GNAI1	Guanine nucleotide-binding protein G(i) subunit alpha-1	NM_200971.1
A10G0509	19.79	28.39	FHL1	Four and a half LIM domains protein 1	NM_199217.1
B14G0123	19.78	30.43	TGFBI	Transforming growth factor-beta-induced protein ig-h3	NM_182862.1
A2G0150	19.78	10.92	LOC107709350	A0A673L822_9TELE Phosphodiesterase	
B24G0076	19.78	57.43	IGKV4-1	Immunoglobulin kappa variable 4-1	NM_001045408.1
A15G0689	19.76	7.05	RHOU	Rho-related GTP-binding protein RhoU	NM_001017784.3
A20G0648	19.75	6.25	PINX1	TERF1-interacting telomerase inhibitor 1	NM_001013265.2
A21G0701	19.75	16.03	CNOT8	CCR4-NOT transcription complex subunit 8	XM_005173877.4
B19G0099	19.74	18.67	BRD2	Bromodomain-containing protein 2	NM_001270571.1
B9G0093	19.74	12.37	IL1R1	Interleukin-1 receptor type 1	
B7G0625	19.73	65.77	MMP2	72 kDa type IV collagenase	NM_198067.1
A13G0436	19.73	10.10	HGSNAT	Heparan-alpha-glucosaminide N-acetyltransferase	NM_001004529.2
B9G0467	19.72	13.14	RND3	Rho-related GTP-binding protein RhoE	NM_001002591.1
B16G0924	19.72	23.73	TNFRSF1A	Tumor necrosis factor receptor superfamily member 1A	NM_213190.1
A13G0796	19.72	15.81	MRPS6	28S ribosomal protein S6, mitochondrial	NM_199715.1
A12G0437	19.72	12.30	GNA13	Guanine nucleotide-binding protein subunit alpha-13	NM_001012243.3
A17G0536	19.69	27.44	ATP6V1B2	V-type proton ATPase subunit B, brain isoform	NM_182878.1
A14G0061	19.68	5.43	NUDCD2	NudC domain-containing protein 2	NM_001003539.1
B5G0247	19.67	28.93	ALDH2	Aldehyde dehydrogenase, mitochondrial	NM_200490.1
A16G0727	19.67	75.33	GABARAPL1	Gamma-aminobutyric acid receptor-associated protein-like 1	NM_001002707.2
A1G0727	19.66	7.46	MRPL39	39S ribosomal protein L39, mitochondrial	
A16G0554	19.65	25.61	ARHGEF1	Rho guanine nucleotide exchange factor 1	XM_017351352.3
A5G0203	19.65	15.34	PURB	Transcriptional activator protein Pur-beta	NM_001160159.1
A18G0140	19.65	14.56	SELENOT1B	Thioredoxin reductase-like selenoprotein T1b	NM_178292.5
B5G0310	19.64	9.44	ZCCHC8	Zinc finger CCHC domain-containing protein 8	NM_001083818.1
B16G0508	19.63	15.43	ENSA	Alpha-endosulfine	NM_001256731.1
B22G1050	19.63	21.28	PSMD1	26S proteasome non-ATPase regulatory subunit 1	NM_201184.2
A13G0049	19.63	3.36	B3GALNT2	UDP-GalNAc:beta-1,3-N-acetylgalactosaminyltransferase 2	NM_001089399.2

A17G0374	19.62	18.46	KDM1A	Lysine-specific histone demethylase 1A	XM_005158783.5
A20G0020	19.61	18.56	ZNF410	Zinc finger protein 410	XM_005170226.5
B18G0354	19.61	14.73	CLASRP	arginine rich protein	NM_001044329.1
B7G0739	19.60	7.50	TMED3	Transmembrane emp24 domain-containing protein 3	NM_001128668.1
A20G0698	19.60	48.99	PLPP3	Phospholipid phosphatase 3	XM_001919526.7
A15G0239	19.60	8.19	DHX16	Pre-mRNA-splicing factor ATP-dependent RNA helicase DHX16	XM_068213477.1
A18G0467	19.60	13.74	SNAPC5	snRNA-activating protein complex subunit 5	XM_005159085.5
B20G0527	19.58	14.97	WTAP	Pre-mRNA-splicing regulator WTAP	NM_199853.1
A5G0570	19.58	23.92	ISCU	Iron-sulfur cluster assembly enzyme ISCU, mitochondrial	
A10G0163	19.58	18.13	CLDN5	Claudin-5	NM_001006044.2
B2G0306	19.58	16.19	ESYT2-A	Extended synaptotagmin-2-A	XM_005171397.5
A6G0680	19.57	14.30	SEC61AL1	Protein transport protein Sec61 subunit alpha-like 1	NM_153659.2
A25G0661	19.57	13.55	KV3A9	Ig kappa chain V-III region MOPC 63	XM_068217596.1
A21G0554	19.56	17.21	BANF1	Barrier-to-autointegration factor	NM_205562.1
B3G0995	19.56	21.00	USP7	Ubiquitin carboxyl-terminal hydrolase 7	XM_021473871.2
A14G0224	19.56	11.18	ATP11C	Phospholipid-transporting ATPase IG	XM_009291180.4
A25G0530	19.56	16.11	SQOR	Sulfide:quinone oxidoreductase, mitochondrial	XM_021470490.2
B22G0539	19.56	6.96	TIMM13	Mitochondrial import inner membrane translocase subunit Tim13	NM_001002465.2
A10G0849	19.55	13.23	IL6ST	Interleukin-6 receptor subunit beta	NM_001365484.1
B7G0826	19.55	3.52			
A6G0995	19.54	17.29	DCTN2	Dynactin subunit 2	XM_005166169.4
A12G0798	19.54	11.00	LRRC45	Leucine-rich repeat-containing protein 45	XM_002663878.6
A18G0068	19.54	16.17	PLD3	5'-3' exonuclease PLD3	XM_003200490.6
A21G0615	19.51	16.29	ZMAT2	Zinc finger matrin-type protein 2	NM_212681.1
A8G0488	19.51	23.52	SYP	Synaptophysin	XM_017357386.2
A21G0793	19.51	4.26	A0A672T947	A0A672T947_SINGR Interleukin 13	NM_001199905.1
B22G0065	19.50	7.06	WDR77	Methylosome protein 50	NM_200534.1
A9G0238	19.49	21.75	RALB	Ras-related protein Ral-B	NM_001003649.1
A19G0107	19.49	22.11	SRSF4	arginine-rich splicing factor 4	NM_199574.1
A16G0468	19.49	9.00	CTSK	Cathepsin K	NM_001017778.1
A16G0270	19.49	6.47	CD163	Scavenger receptor cysteine-rich type 1 protein M130	XM_017351540.3
B23G0621	19.48	2.23	SH3BGRL3	SH3 domain-binding glutamic acid-rich-like protein 3	
B19G0671	19.48	4.52	SLC7A6	Y+L amino acid transporter 2	NM_001100022.1
B15G0514	19.48	14.30	CRK	Adapter molecule crk	NM_001003628.2
A7G0799	19.48	17.50	SLC7A6OS	Probable RNA polymerase II nuclear localization protein SLC7A6OS	NM_001007344.2
A23G0542	19.48	7.04	SRM	Spermidine synthase	NM_201034.2
A14G0247	19.48	6.05	SOWAHD	Ankyrin repeat domain-containing protein SOWAHD	XM_001339829.7
A22G0490	19.47	2.73	SEMA4E	Semaphorin-4E	XM_005170470.5
B3G1175	19.46	32.76	ST13	Hsc70-interacting protein	NM_199769.2

B14G0213	19.45	38.82	RBMX	RNA-binding motif protein, X chromosome	XR_011006078.1
A3G0851	19.45	5.54	A0A672K3G1	A0A672K3G1_SINGR Si:ch211-156b7.4	XR_045227.7
B20G0634	19.43	9.12	CD2AP	CD2-associated protein	NM_001008583.2
B14G0812	19.43	26.31	RRAGA	Ras-related GTP-binding protein A	
B20G0364	19.42	28.51	FRYL	Protein furry homolog-like	XM_009294673.4
B15G0632	19.41	12.37	WSB1	WD repeat and SOCS box-containing protein 1	NM_199633.1
A8G0627	19.41	72.37	SEPP1A	Selenoprotein Pa	NM_178297.4
A5G1296	19.40	6.62	SELENOM	Selenoprotein M	NM_178286.4
B5G0283	19.40	27.69	SPAG7	Sperm-associated antigen 7 homolog	NM_201125.1
B19G0870	19.39	26.68	NUDC	Nuclear migration protein nudC	NM_200919.1
B8G0784	19.39	10.61	ABL2	Tyrosine-protein kinase ABL2	NM_001122705.1
A21G0900	19.38	17.11	SAR1B	GTP-binding protein SAR1b	NM_001024377.2
B7G0828	19.38	7.28	MPDU1	Mannose-P-dolichol utilization defect 1 protein	NM_001002130.1
B20G0213	19.38	6.37	COA7	Cytochrome c oxidase assembly factor 7	NM_001030084.1
B1G0277	19.38	15.50	VPS37A	Vacuolar protein sorting-associated protein 37A	NM_199990.1
B10G0849	19.37	20.73	PSMG1	Proteasome assembly chaperone 1	NM_001002714.1
B2G0351	19.37	4.69	CCL20	C-C motif chemokine 20	
B8G0799	19.36	45.45	RHOAA	Rho-related GTP-binding protein RhoA-A	NM_213137.1
A3G0539	19.35	21.15	RETSAT	All-trans-retinol 13,14-reductase	NM_001015061.2
B1G1031	19.35	0.88	ADGRE3	Adhesion G protein-coupled receptor E3	XM_021477530.2
A20G0205	19.35	20.41	SNX9	Sorting nexin-9	XM_021468448.2
B2G0159	19.34	14.49	CUL1	Cullin-1	NM_199659.2
A21G0897	19.34	10.24	CAMLG	Guided entry of tail-anchored proteins factor CAMLG	
A18G0204	19.34	10.81	GINS2	DNA replication complex GINS protein PSF2	NM_001024439.1
A14G0201	19.34	13.29	CAB39	Calcium-binding protein 39	XM_068213185.1
A22G0363	19.33	15.57	ATPAF1	ATP synthase mitochondrial F1 complex assembly factor 1	NM_001017907.2
A17G0393	19.33	3.04	GRHL3	Grainyhead-like protein 3 homolog	XM_068214602.1
A5G0776	19.32	43.87	EEF2	Elongation factor 2	XM_021476571.2
B23G0260	19.31	19.59	ADRM1B	Proteasomal ubiquitin receptor ADRM1	XM_005161962.5
A15G0223	19.31	6.03	RHBDD1	Rhomboid-related protein 4	NM_001017614.1
B23G0710	19.30	13.26	RARGA	Retinoic acid receptor gamma-A	XM_068216665.1
B21G0351	19.30	9.26	PFDN1	Prefoldin subunit 1	NM_001013344.1
B16G0498	19.30	5.04			
B11G0281	19.30	8.70	ECT2	Protein ECT2	NM_001003429.2
B23G0341	19.30	28.49	NSFL1C	NSFL1 cofactor p47	XM_009296732.2
B1G0714	19.29	26.46	DPX16_16247	A0A3N0XK22_ANAGA Uncharacterized protein	
A19G0555	19.29	7.45	OSBPL3	Oxysterol-binding protein-related protein 3	NM_001004646.1
B6G0933	19.28	16.13	GNAI2	Guanine nucleotide-binding protein G(i) subunit alpha-2	NM_199842.1
A6G0697	19.28	6.51	PRKCD	Protein kinase C delta type	NM_214708.1
B11G0045	19.26	8.54	SRSF6	arginine-rich splicing factor 6	
B3G0999	19.25	16.15	EMP2	Epithelial membrane protein 2	XM_068216679.1

A13G0616	19.24	6.50	FAM241A	Uncharacterized protein FAM241A	NM_001128739.1
B16G0718	19.24	50.50	MYZAP	Myocardial zonula adherens protein	NM_001080001.1
B14G0588	19.24	8.01	CASP3	Caspase-3	
B6G0995	19.23	7.67	DDX27	Probable ATP-dependent RNA helicase DDX27	NM_001002869.1
A5G0782	19.23	3.62	RIOK2	threonine-protein kinase RIO2	NM_213554.2
A3G0592	19.23	23.15	GNA13	Guanine nucleotide-binding protein subunit alpha-13	NM_001013263.2
A17G0525	19.23	33.68	HTRA1A	Serine protease HTRA1A	NM_001002219.1
B2G1150	19.22	13.70	CMPK	UMP-CMP kinase	NM_213109.2
B8G0148	19.22	17.92	RAB7	Ras-related protein rab7	NM_001002178.1
B19G0437	19.22	6.12	TPMT	Probable thiopurine S-methyltransferase	NM_001002569.2
B16G0105	19.20	24.65	ETFB	Electron transfer flavoprotein subunit beta	NM_212998.2
B15G0044	19.20	7.30	NMD3	60S ribosomal export protein NMD3	NM_001013569.2
B20G0948	19.19	18.51	YY1	Transcriptional repressor protein YY1	XM_005170229.5
A2G0134	19.19	32.69	AP2M1A	AP-2 complex subunit mu-A	NM_001291355.1
B11G0607	19.18	12.39	USP48	Ubiquitin carboxyl-terminal hydrolase 48	NM_001202432.2
B7G0791	19.18	13.72	SLC7A6OS	Probable RNA polymerase II nuclear localization protein SLC7A6OS	NM_001007344.2
B7G0154	19.17	51.64	KARS1	Lysine--tRNA ligase	NM_001002386.2
B21G0353	19.17	16.87	HNRNPH2	Heterogeneous nuclear ribonucleoprotein H2	NM_212589.2
A13G0241	19.16	20.59	PSME4A	Proteasome activator complex subunit 4A	NM_001145579.1
A20G0389	19.16	16.73	DDX1	ATP-dependent RNA helicase DDX1	NM_001197324.1
A13G0362	19.16	25.30	PPA1	Inorganic pyrophosphatase	XM_021480667.2
B3G1252	19.15	6.04	ARHGAP27	Rho GTPase-activating protein 27	XM_017354750.3
B19G0692	19.14	10.73	MFSD2AB	Sodium-dependent lysophosphatidylcholine symporter 1-B	NM_001003570.2
A23G0901	19.14	19.44	G3BP2	Ras GTPase-activating protein-binding protein 2	NM_213374.2
A2G0121	19.12	20.92	VPS4B	Vacuolar protein sorting-associated protein 4B	XM_009298075.4
B10G0575	19.12	22.92	UBL3	Ubiquitin-like protein 3	NM_212686.2
B16G0488	19.11	5.50	ZGC:113036	Uncharacterized protein C18orf19 homolog B	NM_001013340.1
B10G0041	19.11	40.90	TPM	Tropomyosin	XM_005155493.5
A8G0030	19.11	12.46	SRP19	Signal recognition particle 19 kDa protein	NM_213343.1
B22G1103	19.11	8.57	WWTR1	WW domain-containing transcription regulator protein 1	NM_001037696.1
B16G0179	19.10	18.31	DNASE2	Deoxyribonuclease-2-alpha	NM_001114738.1
A14G0208	19.10	13.89	MMGT1	ER membrane protein complex subunit 5	NM_205599.1
B22G1035	19.10	14.24	NLRC3	NLR family CARD domain-containing protein 3	XM_021478497.2
A11G0270	19.09	28.93	NCOA3	Nuclear receptor coactivator 3	NM_001007775.2
B9G0170	19.09	2.59	AGR3	Anterior gradient protein 3	
B7G0619	19.09	13.81	AKTIP	AKT-interacting protein	NM_001320326.1
A10G0384	19.09	7.75	EXOSC8	Exosome complex component RRP43	NM_001002865.1

A1G0267	19.09	16.68	A0A672LDL2	A0A672LDL2_SINGR Ubiquitin-like domain-containing protein	
B25G0183	19.08	2.25	IGKV1-9	Immunoglobulin kappa variable 1-9	NM_001045408.1
B19G0892	19.07	12.82	CTPS1	CTP synthase 1	NM_199211.2
A23G0634	19.06	46.33	DAZAP2	DAZ-associated protein 2	NM_199793.2
B6G0055	19.06	4.69	TRIM39	E3 ubiquitin-protein ligase TRIM39	NM_001100041.2
B22G0193	19.06	9.99	CD274	Programmed cell death 1 ligand 1	NM_001128805.1
B23G0882	19.06	10.73	ABCE1	ATP-binding cassette sub-family E member 1	NM_213051.2
A11G0599	19.05	8.88	RTL1	Retrotransposon-like protein 1	NM_001082795.1
A2G0691	19.05	27.19	ATP6V0B	V-type proton ATPase 21 kDa proteolipid subunit c"	NM_199561.2
A3G0735	19.05	15.09	RNF113A	E3 ubiquitin-protein ligase RNF113A	NM_001004536.1
A21G0669	19.04	0.32	RGS14	Regulator of G-protein signaling 14	XM_021473436.2
B25G0542	19.04	21.11	NDUFA9	NADH dehydrogenase [ubiquinone] 1 alpha subcomplex subunit 9, mitochondrial	NM_001013459.2
B21G0487	19.03	16.89	ANGPTL5	Angiopoietin-related protein 5	XM_068218969.1
A12G0247	19.03	0.30	G5714_012430	A0A7J6CGQ6_9TELE Uncharacterized protein	
B11G0337	19.03	8.40	PRICKLE2	Prickle-like protein 2	NM_001044990.2
B2G0712	19.02	2.80	RNF152	E3 ubiquitin-protein ligase rnf152	NM_001014358.2
A6G0337	19.02	13.33	PSMD12	26S proteasome non-ATPase regulatory subunit 12	NM_201578.1
B11G0136	19.02	9.63	TRY3	Trypsin-3 (Fragment)	NM_001089408.1
B6G0629	19.01	9.39	NIPA2	Magnesium transporter NIPA2	XM_005165975.5
B8G0593	19.00	14.23	MAPRE1	EB family member 1	NM_199864.2
B15G0027	19.00	3.36	MLF1	Myeloid leukemia factor 1	NM_001326374.1
B15G0850	18.99	24.24	CTSC	Dipeptidyl peptidase 1	NM_214722.1
B15G0335	18.98	7.30	TXNDC17	Thioredoxin domain-containing protein 17	
A4G0910	18.98	9.55	OSGEP	tRNA N6-adenosine threonylcarbamoyltransferase	XM_021475013.2
A9G0226	18.98	7.94	HACD2	Very-long-chain (3R)-3-hydroxyacyl-CoA dehydratase 2	NM_199861.2
B4G0075	18.97	12.00	YEATS4	YEATS domain-containing protein 4	NM_001002752.2
B16G0289	18.96	4.28	GBP	GSK-3-binding protein	NM_131442.1
B19G0031	18.96	3.56	LOC113075715	A0A6P6N572_CARAU uncharacterized protein LOC113075715	
B13G0611	18.96	19.37	ARG2-A	Arginase, non-hepatic 1	XM_017358739.3
B9G0797	18.95	16.33	NDUFB3	NADH dehydrogenase [ubiquinone] 1 beta subcomplex subunit 3	
B10G0314	18.95	117.64	KDM6B	Lysine-specific demethylase 6B	NM_001030178.2
B19G0101	18.95	26.65	PSMB8	Proteasome subunit beta type-8	NM_131392.3
B12G0772	18.92	10.86	KDELR2	ER lumen protein-retaining receptor 2	NM_212695.1
B8G1001	18.92	13.09	POLB	DNA polymerase beta	NM_001003879.2
A22G0095	18.90	6.88	MR1	Major histocompatibility complex class I-related gene protein	
B23G0118	18.90	14.73	STXBP3	Syntaxin-binding protein 3	NM_213471.1

A3G1062	18.90	13.92	UBE2I	SUMO-conjugating enzyme UBC9	
B13G0669	18.90	17.40	MYCL1A	Protein L-Myc-1a	NM_001354552.1
A20G0460	18.89	19.32	SUSD6	Sushi domain-containing protein 6	XR_011007672.1
B3G1328	18.89	16.46	I3	Brain protein I3	
A25G0235	18.89	26.15	OLFM4	Olfactomedin-4	XM_001335347.8
B2G0752	18.88	16.95	PTBP1	Polypyrimidine tract-binding protein 1	NM_001020477.1
B24G0670	18.88	10.57	PDSS1	All trans-polyprenyl-diphosphate synthase PDSS1	NM_001017656.1
B4G0492	18.88	11.22	IMPDH1B	Inosine-5'-monophosphate dehydrogenase 1b	XM_005164717.5
B11G0503	18.88	27.31	CHD5	Chromodomain-helicase-DNA-binding protein 5	NM_001024389.1
B7G0382	18.87	13.60	PHKA1	Phosphorylase b kinase regulatory subunit alpha, skeletal muscle isoform	XM_009303454.4
B5G0849	18.87	12.07	PTGER4	Prostaglandin E2 receptor EP4 subtype	NM_001128367.1
B19G0502	18.87	9.08	SNRNP40	U5 small nuclear ribonucleoprotein 40 kDa protein	NM_200322.1
B18G0043	18.86	22.34	ETS1	c-ets-1	NM_001017558.1
B8G0357	18.86	4.46	RFLNA	Refilin-A	NM_001007031.2
A19G0800	18.86	39.07	CD209C	CD209 antigen-like protein C	NM_001177451.1
A12G0771	18.84	15.62	PPP4CB	threonine-protein phosphatase 4 catalytic subunit B	NM_001111168.1
A13G0384	18.84	14.37	MYOF	Myoferlin	
A17G0912	18.84	14.63	LRR1	Leucine-rich repeat protein 1	
A16G0658	18.84	16.85	CCPG1	Cell cycle progression protein 1	
A22G0227	18.84	22.68	SHISA5	Protein shisa-5	NM_001128696.1
A4G0448	18.83	12.96	NUP50	Nuclear pore complex protein Nup50	XM_068219518.1
B10G0402	18.82	12.10	HTATSF1	HIV Tat-specific factor 1	NM_201203.1
B23G0309	18.82	17.15	GID8B	Glucose-induced degradation protein 8-B homolog	NM_001386504.1
B8G0311	18.81	21.12	DUSP2	Dual specificity protein phosphatase 2	NM_001003451.1
B17G0350	18.81	7.47	UBE2D4	Ubiquitin-conjugating enzyme E2 D4	NM_201110.1
A4G0648	18.80	36.18	ETNK1	Ethanolamine kinase 1	XM_009300314.4
B5G0862	18.80	21.63	HEXB	Beta-hexosaminidase subunit beta	XM_005171791.5
B5G1030	18.79	24.36	ADAM28	Disintegrin and metalloproteinase domain-containing protein 28	NM_001076718.1
B3G1214	18.79	16.21	CBX1	Chromobox protein homolog 1	NM_199746.2
B24G0001	18.78	16.53	WWP1	NEDD4-like E3 ubiquitin-protein ligase WWP1	XM_068217284.1
B19G0959	18.78	2.54	TNXB	Tenascin-X	XM_068215342.1
A5G0678	18.78	10.06	HNRNPL	Heterogeneous nuclear ribonucleoprotein L	XM_021476043.2
A14G0738	18.77	2.77	PKP3	Plakophilin-3	
B7G0603	18.77	33.01	CEBPA	enhancer-binding protein alpha	NM_131885.2
B9G0044	18.76	17.69	LOC113109173	A0A6P6Q537_CARAU NACHT, LRR and PYD domains-containing protein 12-like isoform X1	XM_021468229.2
B17G0227	18.76	17.28	PSMA6	Proteasome subunit alpha type-6	NM_153655.2
B10G0791	18.74	1.50	DDX55	ATP-dependent RNA helicase DDX55	NM_173229.2

B13G0536	18.74	25.91	CUEDC2	CUE domain-containing protein 2	XM_005156904.5
B20G0900	18.74	20.38	GFUS	GDP-L-fucose synthase	NM_001003528.1
A25G0644	18.74	14.79	IGLC7	Immunoglobulin lambda constant 7	XM_068217596.1
B20G0455	18.73	88.36	SERPINA10	Protein Z-dependent protease inhibitor	NM_001045071.2
A5G1070	18.72	2.27	BICDL2	BICD family-like cargo adapter 2	NM_001193591.1
B8G0108	18.72	12.90	RER1	Protein RER1	NM_200675.2
A12G0530	18.71	45.22	CH25H	Cholesterol 25-hydroxylase-like protein	NM_001008652.1
A21G0545	18.71	12.50	STX5	Syntaxin-5	NM_199630.2
B1G0303	18.71	7.21	KLF3	Krueppel-like factor 3	
A3G0045	18.70	19.49	NITR	Q8AXN8_CYPCA Novel immune type receptor	XM_068214260.1
A6G0766	18.69	2.79	MET	Hepatocyte growth factor receptor	XM_068222037.1
A20G0269	18.69	6.52	CAD	CAD protein	NM_001308825.1
B15G0725	18.69	14.77	ZGC:66022	Mitochondrial fission factor homolog B	XM_005157755.5
B22G0391	18.68	2.17	ROHU_008728	A0A498M8C6_LABRO Terminal uridylyltransferase 4-like protein	
A16G0337	18.68	30.79	TAF12	Transcription initiation factor TFIID subunit 12	NM_198368.1
A16G0619	18.68	35.98	FLT4	Vascular endothelial growth factor receptor 3	
B9G0545	18.67	13.12	RAB5B	Ras-related protein Rab-5B	NM_212885.1
A10G0792	18.67	37.06	LOC113054048	A0A6P6KV56_CARAU dentin sialophosphoprotein-like	NM_001002308.1
B21G0716	18.67	10.50	NUDT2	Bis(5'-nucleosyl)-tetraphosphatase [asymmetrical]	NM_001002323.1
B14G0413	18.67	10.10	NAA15	N-alpha-acetyltransferase 15, NatA auxiliary subunit	NM_200646.2
A23G0259	18.67	5.06	RTASE	Probable RNA-directed DNA polymerase from transposon BS	XR_011015637.1
A13G0505	18.66	10.16	ASRGL1	L-asparaginase	
A10G0325	18.66	18.83	USPL1	SUMO-specific isopeptidase USPL1	NM_001003880.1
B23G0503	18.66	11.34	CCN2	CCN family member 2	NM_001199101.2
B9G0720	18.66	8.55	ANKRD10	Ankyrin repeat domain-containing protein 10	NM_001077377.1
A7G0802	18.65	8.39	DENND2B	DENN domain-containing protein 2B	NM_001002327.1
B13G0753	18.65	11.88	DKK1	Dickkopf-related protein 1	
A13G0515	18.65	15.97	TSPAN15	Tetraspanin-15	XM_005156779.5
A1G0752	18.65	53.16	ATP1A1	potassium-transporting ATPase subunit alpha-1	NM_131689.1
A1G0275	18.64	26.66	NFIX	Nuclear factor 1 X-type	NM_001366171.1
B11G0227	18.64	14.49	WDR18	WD repeat-containing protein 18	
A15G0326	18.64	26.50	WSB1	WD repeat and SOCS box-containing protein 1	NM_199633.1
B10G0855	18.62	34.19	VASP	Vasodilator-stimulated phosphoprotein	
B5G0664	18.62	10.67	TARS1	Threonine--tRNA ligase 1, cytoplasmic	NM_001122786.1
A10G0881	18.61	11.04	LMNB1	Lamin-B1	XM_005155556.5
B7G0386	18.61	15.24	GOT2A	Aspartate aminotransferase, mitochondrial	NM_199989.1
A5G0053	18.61	27.81	NUPR2	Nuclear protein 2	

A13G0610	18.61	8.58	LRIT3	Leucine-rich repeat, immunoglobulin-like domain and transmembrane domain-containing protein 3	XM_005156958.5
B3G0705	18.60	15.39	SMARCE1	SNF-related matrix-associated actin-dependent regulator of chromatin subfamily E member 1	XM_009299517.4
A16G0749	18.59	16.63	PITPNC1	Cytoplasmic phosphatidylinositol transfer protein 1	XM_005157890.5
B2G0372	18.58	20.73	PSMB5	Proteasome subunit beta type-5	NM_131151.1
B6G0102	18.58	11.14	SLC25A12	glutamate antiporter SLC25A12, mitochondrial	NM_212782.1
B23G0226	18.57	6.75	G5714_022879	A0A7J6BPH9_9TELE Uncharacterized protein	NM_001386475.1
A6G0354	18.57	15.17	RAE1	mRNA export factor	NM_200998.1
B6G0843	18.56	10.50	PADI2	Protein-arginine deiminase type-2	XM_005172097.5
B12G0150	18.55	5.51	GPRC5C	G-protein coupled receptor family C group 5 member C	NM_001109713.1
B9G0887	18.54	10.30	G5714_010268	A0A7J6CRH7_9TELE Uncharacterized protein	
B23G0575	18.53	4.60			XM_021469994.2
A10G0133	18.53	23.96	PARG	Poly(ADP-ribose) glycohydrolase	XM_001338221.8
B4G0432	18.53	27.04	STRAP	Serine-threonine kinase receptor-associated protein	NM_200304.1
B25G0393	18.53	9.21	PIM3	threonine-protein kinase pim-3	NM_001034978.2
B12G0245	18.52	4.54	TCF7L2	Transcription factor 7-like 2	XM_068224491.1
B19G0577	18.52	7.31	COL16A1	Collagen alpha-1(XVI) chain	XM_009294358.4
B10G0759	18.51	9.52	DENR	Density-regulated protein	NM_001002697.2
A3G1061	18.51	9.63	MPST	3-mercaptopyruvate sulfurtransferase	NM_001089346.1
B23G0085	18.51	20.60	MAFB	Transcription factor MafB	NM_131015.3
A5G0299	18.51	6.88	LIMK2	LIM domain kinase 2	NM_001002651.1
A5G0018	18.51	28.86	FUBP3	Far upstream element-binding protein 3	NM_001007776.1
A2G0052	18.50	10.63	POLR2D	DNA-directed RNA polymerase II subunit RPB4	NM_001002317.2
A13G0294	18.50	4.92	SLC66A3	Solute carrier family 66 member 3	NM_001007777.1
A22G0016	18.49	10.71	MRPL20	39S ribosomal protein L20, mitochondrial	
A21G0464	18.48	20.74	PCF11	Pre-mRNA cleavage complex 2 protein Pcf11	NM_001007307.1
A17G0651	18.47	16.34	PTP4A2	Protein tyrosine phosphatase type IVA 2	NM_001005583.2
B18G0108	18.47	9.74	ST14	Suppressor of tumorigenicity 14 protein homolog	XM_005159249.4
A6G0509	18.47	16.32	TOMM70	Mitochondrial import receptor subunit TOM70	NM_200002.1
B16G0069	18.46	13.32	BRD9	Bromodomain-containing protein 9	NM_200275.1
A5G0522	18.46	44.52	F11R	Junctional adhesion molecule A	NM_001004667.3
B9G0242	18.46	15.73	SNX4	Sorting nexin-4	NM_001014346.1
A20G0387	18.46	16.61	SMEK1	threonine-protein phosphatase 4 regulatory subunit 3	NM_001044809.1
A19G0762	18.45	13.07	MEAF6	Chromatin modification-related protein MEAF6	NM_001003756.1
B17G0495	18.45	47.07	CALM1	Calmodulin-1	NM_213351.1

B12G0199	18.44	11.71	LLGL2	LLGL scribble cell polarity complex component 2	NM_212582.1
B5G0108	18.44	11.25	PXN	Paxillin	NM_201588.2
B3G0691	18.44	36.68	SUZ12A	Polycomb protein suz12-A	NM_001003529.2
B8G0964	18.43	0.72	DHX37	Probable ATP-dependent RNA helicase DHX37	NM_001083004.2
B7G1233	18.43	10.12	LOC113105444	A0A6P6PNH4_CARAU T-cell surface antigen CD2-like	
B8G0777	18.42	8.55	DNTTIP2	Deoxynucleotidyltransferase terminal-interacting protein 2	NM_001145786.1
B23G0586	18.42	9.06	LZIC	Protein LZIC	NM_001002598.1
A20G0422	18.41	13.50	HEBP2	Heme-binding protein 2	
B1G0442	18.41	5.81	CORO2A	Coronin-2A	XM_005159876.5
A4G0979	18.41	16.38	MRPL42	39S ribosomal protein L42, mitochondrial	
A12G0201	18.40	25.42	TIMP2	Metalloproteinase inhibitor 2	NM_182874.1
A2G0093	18.39	12.05	ARF1	ADP-ribosylation factor 1	NM_201452.2
B11G0217	18.39	10.53	MAST3	threonine-protein kinase 3	
B20G0399	18.39	5.85	PPAT	Amidophosphoribosyltransferase	NM_001082877.2
A1G0053	18.38	28.63	NLRC3	NLR family CARD domain-containing protein 3	XM_068220504.1
B6G0706	18.38	14.62	PRKCD	Protein kinase C delta type	NM_214708.1
B2G0660	18.38	14.85	BAG1	BAG family molecular chaperone regulator 1	NM_001098736.2
B1G0885	18.38	8.39	RAB3D	GTP-binding protein Rab-3D	NM_001145597.1
B17G0882	18.37	3.78	PAPLN	Papilin	XM_021467499.2
A11G0724	18.37	12.93	MMP19	Matrix metalloproteinase-19	NM_001002123.1
A5G0432	18.37	6.37	ABHD17B	beta hydrolase domain-containing protein 17B	XM_021478400.2
B21G0184	18.36	49.03	PGK1	Phosphoglycerate kinase 1	NM_213387.1
B9G0200	18.36	7.23	GULP1	PTB domain-containing engulfment adapter protein 1	NM_001423684.1
B25G0572	18.35	15.95	HIPK3	Homeodomain-interacting protein kinase 3	NM_001144047.1
B6G0644	18.35	15.92	SLC48A1B	Heme transporter hrg1-A	NM_200006.1
B6G0798	18.35	5.96	BDH2	reductase SDR family member 6	NM_001013450.1
B7G0480	18.33	14.75	DYNC1LI2	Cytoplasmic dynein 1 light intermediate chain 2	NM_001017669.2
A21G0564	18.33	12.59	RBM4.1	RNA-binding protein 4.1	NM_205654.2
B23G0654	18.32	15.46	TAF8	Transcription initiation factor TFIID subunit 8	XM_005162303.5
A16G0447	18.32	8.28	LMNA	Lamin-A	NM_152971.1
A4G0579	18.32	14.55	LAMTOR4	Ragulator complex protein LAMTOR4	NM_001044950.1
A10G0180	18.31	7.00	ZCCHC9	Zinc finger CCHC domain-containing protein 9	NM_198370.1
A19G0690	18.31	10.83	CERS2	Ceramide synthase 2	NM_153671.2
A8G0825	18.30	4.81	TGOLN2	Trans-Golgi network integral membrane protein 2	
A13G0744	18.30	19.12	EPCAM	Epithelial cell adhesion molecule	NM_213057.2
B6G0216	18.29	14.81	ABI2	Abl interactor 2	XM_005165772.5
B19G0199	18.29	20.86	HSF1	Heat shock factor protein 1	NM_001313736.1
B3G0610	18.29	10.98	DRG2	Developmentally-regulated GTP-binding protein 2	NM_212638.1
B20G0256	18.28	27.63	IGFBP1	Insulin-like growth factor-binding protein 1	NM_173283.4
A12G0643	18.28	17.62	DNAJC9	DnaJ homolog subfamily C member 9	NM_001002433.1
B20G0813	18.28	8.73	EIF2B2	Translation initiation factor eIF-2B subunit beta	NM_212903.1

B9G0045	18.28	22.15	NLRC3	Protein NLRC3	XM_068222163.1
B23G0581	18.27	38.86	PGD	6-phosphogluconate dehydrogenase, decarboxylating	NM_213453.2
A2G0481	18.27	15.07	RIOK1	threonine-protein kinase RIO1	NM_212995.1
A3G0237	18.25	11.63	CORO7	Coronin-7	XM_003201631.6
A16G0081	18.25	3.49			
A10G0575	18.23	21.56	PER1	Period circadian protein homolog 1	XM_005172625.5
A21G0656	18.23	7.01	CLK4	Dual specificity protein kinase CLK4	NM_001329908.1
B1G0733	18.23	0.35	G5714_000532	A0A7J6DHB1_9TELE Uncharacterized protein	XM_021476836.2
B5G0359	18.22	29.54	PIK3R1	Phosphatidylinositol 3-kinase regulatory subunit alpha	NM_001281844.1
A21G0602	18.22	46.48	PFDN1	Prefoldin subunit 1	NM_001013344.1
B10G0409	18.21	7.22	ZNRD2	Protein ZNRD2	NM_001006010.1
B8G0470	18.21	29.61	RAP1A	Ras-related protein Rap-1A	NM_001002152.1
B6G0174	18.20	13.80	GCDH	Glutaryl-CoA dehydrogenase, mitochondrial	NM_200886.1
B1G0473	18.19	10.49	GEMIN8	Gem-associated protein 8	NM_001007317.1
A6G0720	18.17	15.76	RUVBL1	RuvB-like 1	NM_173835.4
B4G0237	18.16	24.95	NFYB	Nuclear transcription factor Y subunit beta	NM_001017565.1
A8G0125	18.16	12.01	PPP2R2A	threonine-protein phosphatase 2A 55 kDa regulatory subunit B alpha isoform	XM_682011.10
B8G0060	18.16	9.50	MRPS25	28S ribosomal protein S25, mitochondrial	NM_001017590.1
A23G0653	18.15	6.84			
A5G1282	18.15	9.54	MTHFD2	cyclohydrolase, mitochondrial	NM_001002181.1
A9G0648	18.14	19.52	IUNH	Inosine-uridine preferring nucleoside hydrolase	NM_001256625.1
A22G0079	18.14	13.88	PAK2	threonine-protein kinase PAK 2	NM_001025456.1
A21G0179	18.14	10.90	PSMB7	Proteasome subunit beta type-7	XM_021468901.2
B19G0233	18.13	8.32	NUGGC	Nuclear GTPase SLIP-GC	XM_005159395.5
B24G0350	18.12	24.33	SH3KBP1	SH3 domain-containing kinase-binding protein 1	XM_002666661.7
B1G1166	18.12	17.99	GPR108	Protein GPR108	XM_068220886.1
B4G0050	18.12	48.42	MTPN	Myotrophin	NM_212972.2
A3G0189	18.11	13.86	HLF	Hepatic leukemia factor	NM_001077334.2
B13G0109	18.11	11.89	CSMD1	CUB and sushi domain-containing protein 1	XM_005157040.5
B8G0061	18.10	16.23	NR2C2	Nuclear receptor subfamily 2 group C member 2	NM_001123294.1
A10G0760	18.10	20.93	HSDL2	Hydroxysteroid dehydrogenase-like protein 2	NM_199599.2
B9G0665	18.09	7.15	G5714_010504	A0A7J6CQP6_9TELE Uncharacterized protein	
B4G0312	18.08	36.16	CRY1	Cryptochrome-1	NM_001077297.2
B24G0274	18.07	13.49	PTBP2	Polypyrimidine tract-binding protein 2	NM_001328429.1
A16G0903	18.06	18.60	CTNNB1	Catenin beta-1	NM_131059.2
A13G0791	18.05	10.58	C1D	Nuclear nucleic acid-binding protein C1D	NM_213151.1
B20G0738	18.05	3.99	HCAR2	Hydroxycarboxylic acid receptor 2	
B1G0081	18.04	8.44	IPO5	Importin-5	XM_009305394.3
B5G0975	18.04	10.82	TIMM8A	Mitochondrial import inner membrane translocase subunit Tim8 A	
B11G0683	18.03	8.21	MRPS16	28S ribosomal protein S16, mitochondrial	XM_005155658.5

B22G0019	18.03	8.05	NBL1	Neuroblastoma suppressor of tumorigenicity 1	NM_207097.1
A13G0771	18.03	10.07	MEA1	Male-enhanced antigen 1	XM_005169517.5
B11G0367	18.02	8.85	TMEM183A	Transmembrane protein 183A	NM_001044324.2
A18G0127	18.02	14.83	TTC36	Tetratricopeptide repeat protein 36	
B17G0798	18.02	23.57	MRPL33	39S ribosomal protein L33, mitochondrial	
B1G0159	18.02	20.20	ATP5MK	ATP synthase membrane subunit K, mitochondrial	XR_002456862.2
B13G0247	18.01	5.54	MAT1A	S-adenosylmethionine synthase isoform type-1	NM_131863.2
A7G0844	18.01	19.30	RTASE	Probable RNA-directed DNA polymerase from transposon BS	XM_068220297.1
A13G0385	18.01	13.58	MYOF	Myoferlin	NM_212576.1
B22G0946	18.00	18.23	STXB	Stonustoxin subunit beta	XM_005161821.5
A23G0267	17.99	20.31	FBXO6	F-box only protein 6	NM_001111202.1
A12G0367	17.99	7.03	ZFP36L2-A	mRNA decay activator protein ZFP36L2-A	NM_207055.2
B19G0514	17.99	6.47	CRTAP	Cartilage-associated protein	NM_001001406.1
B8G0530	17.99	18.78	SLC38A5	Sodium-coupled neutral amino acid transporter 5	XM_001344713.6
A7G1395	17.99	8.71	P5436	UPF0696 protein C11orf68 homolog	NM_200448.2
B9G0473	17.99	11.91	CCNT2	Cyclin-T2	NM_199828.2
B1G0946	17.99	10.87	SMDT1	Essential MCU regulator, mitochondrial	
B22G0507	17.99	22.81	PDHB	Pyruvate dehydrogenase E1 component subunit beta, mitochondrial	NM_213154.1
A1G0999	17.98	5.50	EXOSC9	Exosome complex component RRP45	NM_001006077.1
A14G0362	17.98	3.79	SLC7A11	glutamate transporter	XM_009291228.4
A2G0084	17.97	11.45	USP14	Ubiquitin carboxyl-terminal hydrolase 14	NM_199973.1
A10G0569	17.97	10.45	MED11	Mediator of RNA polymerase II transcription subunit 11	NM_001160035.1
A24G0567	17.96	3.08	TFAP2A	Transcription factor AP-2-alpha	NM_001319158.1
A7G0522	17.96	38.69	DNAJA2	DnaJ homolog subfamily A member 2	NM_213493.1
B3G0950	17.96	32.08	AKT1S1	Proline-rich AKT1 substrate 1	XM_687419.10
A12G0060	17.96	13.71	PPIF	Peptidyl-prolyl cis-trans isomerase F, mitochondrial	NM_001037122.2
A9G0743	17.96	4.24			
B8G1010	17.95	17.16	SNAP29	Synaptosomal-associated protein 29	NM_001256256.1
B3G0544	17.95	10.64	KCTD5	POZ domain-containing protein KCTD5	NM_207049.1
B22G1166	17.95	10.24	LOC113044317	A0A6P6JKE6_CARAU uncharacterized protein LOC113044317 isoform X3	
B16G0308	17.94	15.92	TGFBR2	TGF-beta receptor type-2	NM_182855.3
A3G0312	17.93	8.71	MRPL12	39S ribosomal protein L12, mitochondrial	NM_001017696.1
A12G0469	17.93	14.01	GSPT1	Eukaryotic peptide chain release factor GTP-binding subunit ERF3A	NM_001003992.1
A6G0794	17.93	23.04	PPP4R2B	threonine-protein phosphatase 4 regulatory subunit 2-B	NM_001110371.2
A21G0025	17.93	30.64	RAD23B	UV excision repair protein RAD23 homolog B	NM_200564.2
B15G0078	17.92	10.14	PPP5C	threonine-protein phosphatase 5	XM_005157376.5

B18G0381	17.91	5.37	TP53I11	Tumor protein p53-inducible protein 11	XM_021467658.2
A17G0688	17.91	8.44	TFB2M	Dimethyladenosine transferase 2, mitochondrial	NM_001113617.2
A3G0080	17.91	6.33	CCDC134	Coiled-coil domain-containing protein 134	XM_003198004.6
A13G0398	17.90	16.06	NT5C2	Cytosolic purine 5'-nucleotidase	NM_200907.1
A8G0476	17.89	7.80	LAMTOR5	Ragulator complex protein LAMTOR5	NM_001328073.1
A23G0066	17.88	9.75	RAB5IF	Respirasome Complex Assembly Factor 1	NM_199850.2
B23G0729	17.88	8.27	CSAD	Cysteine sulfinic acid decarboxylase	NM_001007348.2
B14G0329	17.88	252.82	LECT2	Leukocyte cell-derived chemotaxin-2	NM_001048055.1
A20G0770	17.87	38.64	ZFP36L1	mRNA decay activator protein ZFP36L1	NM_199649.2
B13G0121	17.87	13.67	NOLC1	Nucleolar and coiled-body phosphoprotein 1	XM_005169517.5
A3G0128	17.87	29.32	A0A673JLK6	A0A673JLK6_9TELE Uncharacterized protein	
A6G0953	17.87	8.39	TEAD3	Transcriptional enhancer factor TEF-5	XM_005166141.5
B14G0143	17.85	22.22	XIAP	E3 ubiquitin-protein ligase XIAP	NM_194396.2
B3G1082	17.84	21.80	MVP	Major vault protein	NM_201325.2
B11G0712	17.83	7.52	LOC113055708	A0A6P6L039_CARAU uncharacterized protein LOC113055708	NM_001002160.2
A22G0586	17.83	10.55	VTCN1	V-set domain-containing T-cell activation inhibitor 1	
B6G0853	17.83	20.62	RHOAD	Rho-related GTP-binding protein RhoA-D	NM_201150.1
B21G0607	17.82	17.72	CRKL	Crk-like protein	NM_213538.1
A13G0193	17.82	24.97	FRMD6	FERM domain-containing protein 6	NM_001025502.1
B14G0728	17.82	14.08	ISK1L	Trypsin inhibitor ClTI-1	
B7G1071	17.82	7.32	PELI3	E3 ubiquitin-protein ligase pellino homolog 3	XM_002662799.7
A6G0059	17.81	28.82	MYL9	Myosin regulatory light polypeptide 9	NM_213212.1
B13G0098	17.81	3.51	ELOVL5	Elongation of very long chain fatty acids protein 5	NM_131604.3
A19G0357	17.81	29.50	PEF1	Peflin	NM_001003643.1
A21G0617	17.81	7.28	LCP2	Lymphocyte cytosolic protein 2	
A25G0465	17.80	18.22	FNBP4	Formin-binding protein 4	NM_001305621.1
A19G0733	17.80	4.45	HGH1	Protein HGH1 homolog	NM_001002522.2
B5G1192	17.80	37.40	BTNL2	Butyrophilin-like protein 2	XM_068218224.1
A2G0628	17.80	27.41	MLRN	Myosin regulatory light chain 2, smooth muscle minor isoform	NM_001130589.2
B22G0287	17.79	14.70	LOC113040362	A0A6P6J343_CARAU uncharacterized protein LOC113040362 isoform X2	XM_005161637.5
A22G0226	17.79	12.21	SHISA5	Protein shisa-5	
A2G0666	17.79	24.31	ABCF2	ATP-binding cassette sub-family F member 2	NM_201315.2
B3G0789	17.79	0.23	MUCM	Ig mu chain C region membrane-bound form	
B1G0607	17.78	7.44	SPCS3	Signal peptidase complex subunit 3	NM_212948.1
B1G0561	17.78	14.04	GTF2F2	General transcription factor IIF subunit 2	NM_001002127.2

B21G0933	17.78	27.90	APOL3	Apolipoprotein L3	
A19G0553	17.78	6.69	NFE2L1	Endoplasmic reticulum membrane sensor NFE2L1	XM_005173768.5
B6G0747	17.77	23.31	CRACR2A	EF-hand calcium-binding domain-containing protein 4B	XM_068222028.1
B14G0186	17.76	9.57	SLC7A3	Cationic amino acid transporter 3	XM_001342926.8
A11G0297	17.76	22.04	IL20	Interleukin-20	NM_200928.1
B10G0365	17.75	6.93	TIMM10B	Mitochondrial import inner membrane translocase subunit Tim10 B	NM_001017829.1
A23G0617	17.74	21.19	TAMALIN	General receptor for phosphoinositides 1-associated scaffold protein	NM_001039924.1
B2G0286	17.74	12.06	ITGB1	Integrin beta-1	NM_001034979.1
A2G0875	17.73	32.96	PSMB5	Proteasome subunit beta type-5	NM_131151.1
B21G0492	17.73	36.79	A0A673NE64	A0A673NE64_9TELE Grass carp reovirus (GCRV)-induced gene 2d	NM_001245989.1
B21G0397	17.73	12.22	FKBP2	Peptidyl-prolyl cis-trans isomerase FKBP2	NM_001004677.2
A3G0031	17.72	1.22	GIMAP5	GTPase IMAP family member 5	NM_001386434.1
B6G0892	17.72	27.68	MYCA	Transcriptional regulator Myc-A	XM_068221744.1
B10G0419	17.72	2.71	DMAC1	Distal membrane-arm assembly complex protein 1	
B8G0812	17.72	19.90	LONRF1	LON peptidase N-terminal domain and RING finger protein 1	NM_001277234.1
B22G0132	17.72	7.61	LAML2	Lamin-L(II)	NM_131002.2
B8G1002	17.71	18.03	VDAC2	Voltage-dependent anion-selective channel protein 2	NM_213246.1
B19G0893	17.70	21.43	CITED3	p300-interacting transactivator 3	NM_001044982.2
B18G0081	17.70	8.76	PRRG4	Transmembrane gamma-carboxyglutamic acid protein 4	NM_001017637.2
A7G0923	17.70	9.46	VBP1	Prefoldin subunit 3	NM_001020624.1
A21G0551	17.69	23.40	NXF1	Nuclear RNA export factor 1	XM_001923926.6
B2G0852	17.68	11.24	GTF2B	Transcription initiation factor IIB	NM_199697.1
B16G0954	17.67	20.44	PSMD4	26S proteasome non-ATPase regulatory subunit 4	NM_001017624.2
B2G0593	17.67	3.79	CCDC24	Coiled-coil domain-containing protein 24	NM_001326378.1
A5G0595	17.67	24.16	CRAT	Carnitine O-acetyltransferase	NM_001005587.1
B7G1109	17.66	150.67	UBC	Polyubiquitin-C	
B5G0055	17.66	25.16	NUPR1	Nuclear protein 1	
A14G0768	17.66	43.56	GATD3	Glutamine amidotransferase-like class 1 domain-containing protein 3, mitochondrial	
B9G0817	17.65	5.59	FARP1	FERM, ARHGEF and pleckstrin domain-containing protein 1	XM_005172506.5
A10G0496	17.65	14.39	EHBP1L1	EH domain-binding protein 1-like protein 1	XM_021472282.2
A11G0745	17.64	4.00	CSNK2A1	Casein kinase II subunit alpha	NM_201078.1
A19G0346	17.64	39.93	AK2	Adenylate kinase 2, mitochondrial	NM_212596.1
A16G0014	17.64	8.40	SLC39A1	Zinc transporter ZIP1	
B8G0598	17.63	14.61	TPD52L2	Tumor protein D54	XM_005167010.5
A6G0540	17.63	0.83	TTC22	Tetratricopeptide repeat protein 22	XM_021471776.2
B18G0426	17.63	22.15	CHD2	Chromodomain-helicase-DNA-binding protein 2	XM_009293623.4

A6G0371	17.63	30.32	ACOX1	Peroxisomal acyl-coenzyme A oxidase 1	NM_001005933.2
A5G1132	17.62	12.33	TRAF2	TNF receptor-associated factor 2	XM_005165499.5
A21G0725	17.62	3.26	KLF5	Krueppel-like factor 5	NM_001039927.2
A8G0381	17.62	13.87	ALDH9A1A	4-trimethylaminobutyraldehyde dehydrogenase A	XM_005166774.4
B7G1203	17.61	27.53	F13A1	Coagulation factor XIII A chain	NM_001077154.1
A25G0273	17.61	13.91	HIPK3	Homeodomain-interacting protein kinase 3	NM_001144047.1
B3G0883	17.61	4.76	FRIM	Ferritin, middle subunit	NM_001109854.1
A1G0056	17.61	23.51	NLRC3	NLR family CARD domain-containing protein 3	XM_068222163.1
B8G0826	17.60	23.53	ITGB4	Integrin beta-4	XM_005166790.5
B11G0549	17.60	6.56	G5714_011730	A0A7J6CJP4_9TELE Uncharacterized protein	NM_200667.2
A13G0692	17.60	20.87	NEFM	Neurofilament medium polypeptide	
B18G0785	17.60	24.45	PEPD	Xaa-Pro dipeptidase	NM_198912.1
B1G0865	17.60	31.11	ACTR2A	Actin-related protein 2-A	NM_213499.1
A8G0775	17.60	1.93	G2E3	M phase-specific E3 ubiquitin-protein ligase	
A22G0047	17.59	15.19	ELF3	ETS-related transcription factor Elf-3	XM_002666100.4
A21G0842	17.58	13.03	STK26	threonine-protein kinase 26	NM_001127477.1
B2G0665	17.58	12.58	TCEA1	Transcription elongation factor A protein 1	NM_199994.2
B20G0094	17.58	11.34	PLEKHG3	Pleckstrin homology domain-containing family G member 3	XM_017352680.3
A6G0275	17.57	30.20	NDUFS1	NADH-ubiquinone oxidoreductase 75 kDa subunit, mitochondrial	NM_001007765.1
A1G0001	17.57	34.33	CDC37	Hsp90 co-chaperone Cdc37	NM_201038.1
B7G0836	17.57	16.26	AP1S1	AP-1 complex subunit sigma-1A	NM_001328014.1
B16G0758	17.57	11.98	RABAC1	Prenylated Rab acceptor protein 1	NM_214780.1
B15G0448	17.55	13.39	SDHDB	Succinate dehydrogenase [ubiquinone] cytochrome b small subunit B, mitochondrial	NM_001004004.2
B16G0412	17.55	4.71	PRSS35	Inactive serine protease 35	NM_001002212.1
B16G0745	17.55	48.58	FLOT1	Flotillin-1	NM_201456.1
A21G0298	17.55	11.41	TNFRSF22	Tumor necrosis factor receptor superfamily member 22	NM_131840.2
B7G0991	17.54	12.99	SPG21	Maspardin	NM_213395.1
A6G0831	17.54	14.62	SLC2A1	Solute carrier family 2, facilitated glucose transporter member 1	XM_002662528.6
B25G0168	17.54	4.26	IGKV1-12	Immunoglobulin kappa variable 1-12	NM_001045408.1
B11G0681	17.54	15.20	ERRFI1	ERBB receptor feedback inhibitor 1	
B17G0880	17.53	10.00	PSEN1	Presenilin-1	NM_131024.1
A9G0251	17.52	23.19	APP	Amyloid-beta A4 protein	XM_068223277.1
B13G0567	17.52	16.07	A0A672RMH5	A0A672RMH5_SINGR Palmitoyltransferase	NM_213389.1
B6G0277	17.52	7.56	LOC107748580	A0A673K600_9TELE DNA replication licensing factor MCM6-like	NM_001326565.1
A6G0131	17.52	13.50	ATG3	Ubiquitin-like-conjugating enzyme ATG3	NM_200022.1
A3G0521	17.52	9.09	EPN2	Epsin-2	XM_681373.9
A19G0286	17.51	7.79	MRPL53	39S ribosomal protein L53, mitochondrial	

B19G0688	17.51	12.69	RBBP4	Histone-binding protein RBBP4	NM_212595.1
B9G0721	17.51	26.24	UBE2A	Ubiquitin-conjugating enzyme E2 A	NM_199719.1
A13G0800	17.51	51.11	OIT3	Oncoprotein-induced transcript 3 protein	XM_005157050.5
A16G0665	17.50	7.06	LOC113067422	A0A6P6MHX5_CARAU uncharacterized protein LOC113067422 isoform X2	
B20G0521	17.49	25.74	HEBP2	Heme-binding protein 2	
A5G0435	17.48	17.23	KLF13	Krueppel-like factor 13	NM_001128729.1
A2G1131	17.48	18.37	NDUFV2	NADH dehydrogenase [ubiquinone] flavoprotein 2, mitochondrial	NM_200747.1
A4G1020	17.48	9.28	ETV6	Transcription factor ETV6	NM_131832.2
B5G1136	17.48	12.34	PDHA1	Pyruvate dehydrogenase E1 component subunit alpha, somatic form, mitochondrial	NM_213393.1
B12G0196	17.48	7.08	MRPL43	39S ribosomal protein L43, mitochondrial	NM_001002428.2
B5G0607	17.48	9.13	FAM169A	Soluble lamin-associated protein of 75 kDa	NM_001045367.1
B7G0165	17.46	25.44	LYVE1	Lymphatic vessel endothelial hyaluronic acid receptor 1	
A13G0087	17.46	16.14	BSDC1	BSD domain-containing protein 1	
B24G0311	17.44	7.97	LOC113079873	A0A6P6NFV4_CARAU uncharacterized protein LOC113079873 isoform X1	
B10G0187	17.44	13.11	MTREX	Exosome RNA helicase MTR4	NM_214768.1
A19G0122	17.44	19.87	PSMG2	Proteasome assembly chaperone 2	NM_213032.1
B16G1051	17.43	14.26	NT5C3	Cytosolic 5'-nucleotidase 3	XM_068213888.1
B17G0747	17.43	17.31	AHSA1	Activator of 90 kDa heat shock protein ATPase homolog 1	NM_212602.1
A3G0254	17.43	7.15	GADD45GIP1	Growth arrest and DNA damage-inducible proteins-interacting protein 1	
B25G0561	17.42	1.88	DKK3	Dickkopf-related protein 3	NM_001089545.1
A12G0173	17.41	18.01	CAMK2G	calmodulin-dependent protein kinase type II subunit gamma	XM_005156363.5
B1G1178	17.41	10.58	PARN	Poly(A)-specific ribonuclease PARN	NM_001320404.1
B21G0833	17.41	2.10	THBS4B	Thrombospondin-4-B	NM_173226.1
B19G0173	17.39	7.01	FHL3	Four and a half LIM domains protein 3	NM_001099978.1
A8G0501	17.39	8.30	G5714_009203	A0A7J6CS57_9TELE Uncharacterized protein	
A3G0342	17.39	6.99	METRNL	Meteorin-like protein	NM_212985.1
A14G0584	17.39	11.13	CASP3	Caspase-3	
A3G0445	17.38	11.77	PDPK1	3-phosphoinositide-dependent protein kinase 1	XM_068217664.1
B7G1228	17.38	22.82	CAPN1	Calpain-1 catalytic subunit	NM_213459.1
B2G1248	17.38	9.82	ABCF3	ATP-binding cassette sub-family F member 3	XM_017351221.3
B14G0808	17.37	17.82	HSPA9	Stress-70 protein, mitochondrial	
B16G0757	17.37	3.89			
A21G0726	17.37	6.60	KLF8	Krueppel-like factor 8	NM_001080003.2
A25G0031	17.37	3.64			XM_021470581.2
A18G0018	17.37	23.91	UBE2Q2	Ubiquitin-conjugating enzyme E2 Q2	XM_005173691.5
A11G0658	17.36	13.61	FAM107A	Actin-associated protein FAM107A	

B16G0010	17.36	2.01			NM_200629.2
A8G0862	17.35	3.54	ACSL6	Long-chain-fatty-acid--CoA ligase 6	XM_002663239.7
A20G0827	17.35	17.50	SPTLC2	Serine palmitoyltransferase 2	NM_001114741.1
A13G0070	17.35	60.00	TMEM87A	Transmembrane protein 87A	NM_001327772.1
A21G0620	17.35	43.01	GRPEL2	GrpE protein homolog 2, mitochondrial	NM_001288663.1
A4G0758	17.35	30.83	CRY1	Cryptochrome-1	NM_001077297.2
A11G0183	17.34	19.32	KRT15	Keratin, type I cytoskeletal 15	
B3G1193	17.33	10.82	SP140L	Nuclear body protein SP140-like protein	XM_678217.8
A8G0159	17.31	9.01	CXXC1	CXXC-type zinc finger protein 1	NM_200599.1
B23G0629	17.28	13.62	HBS1L	HBS1-like protein	NM_001308548.1
B7G0948	17.28	21.69	SNRPA1	U2 small nuclear ribonucleoprotein A'	NM_001007383.2
A15G0094	17.28	8.09	CAPN9	Calpain-9	NM_212939.1
B14G0490	17.28	28.47	UBE2D2	Ubiquitin-conjugating enzyme E2 D2	NM_199952.2
B3G1745	17.27	16.69	GBP1	Guanylate-binding protein 1	XM_001920619.8
B10G0282	17.26	21.51	FBXW11B	F-box and WD repeat domain-containing 11-B	NM_213504.2
B11G0514	17.25	10.73	GRPR	Gastrin-releasing peptide receptor	NM_212642.1
A16G0889	17.24	7.97	ID4	DNA-binding protein inhibitor ID-4	NM_001039990.1
A5G1337	17.24	5.44			
A10G0621	17.24	6.63	PLPP5	Phospholipid phosphatase 5	NM_001002211.2
B21G0335	17.24	30.66	GRPEL2	GrpE protein homolog 2, mitochondrial	
B5G0007	17.23	16.50	JUN	Transcription factor Jun	NM_001431134.1
B16G0147	17.23	20.61	LAMTOR2	Ragulator complex protein LAMTOR2	NM_001003549.2
B13G0526	17.23	27.76	MYOF	Myoferlin	NM_212576.1
B1G0674	17.21	122.90	LEC	Lectin	
A8G0500	17.21	20.24	TRAF3IP3	TRAF3-interacting JNK-activating modulator	XM_003199090.6
B7G0248	17.21	9.41	TRAPPC2L	Trafficking protein particle complex subunit 2-like protein	NM_001003506.2
A2G0335	17.21	19.09	GFI1	Zinc finger protein Gfi-1	XM_068224140.1
A17G0253	17.20	5.97	IAH1	Isoamyl acetate-hydrolyzing esterase 1 homolog	NM_001025469.1
B3G0286	17.19	5.55	G5714_003873	A0A7J6DC07_9TELE Uncharacterized protein	
A1G0252	17.18	20.62	PATL1	Protein PAT1 homolog 1	NM_001083028.1
B22G0017	17.18	18.35	CCNL2	Cyclin-L2	
B3G0846	17.18	10.85	RCN1	Reticulocalbin-1	NM_001002158.1
A21G0329	17.18	14.98	CRKL	Crk-like protein	NM_213538.1
A16G0569	17.17	11.66	TOMM40	Mitochondrial import receptor subunit TOM40 homolog	NM_199614.1
A18G0445	17.17	30.65	A0A672MM88	A0A672MM88_SINGR BH3-interacting domain death agonist	
B2G0454	17.17	19.18	PAK2	threonine-protein kinase PAK 2	NM_001002717.1
B12G0207	17.16	46.93	SLC9A3R1	H(+) exchange regulatory cofactor NHE-RF1	NM_212707.2
B1G0003	17.15	6.71	NFKBIZ	NF-kappa-B inhibitor zeta	
B22G0209	17.14	18.63	SI:DKEY-222F2.8	Cytochrome c oxidase assembly protein COX14 homolog	
B16G0750	17.14	7.77	INO80E	INO80 complex subunit E	NM_205636.2
A20G0590	17.14	1.33			
A23G0640	17.14	25.44	CS	Citrate synthase, mitochondrial	NM_199598.1
A6G0614	17.13	1.18	TRAF3IP2	E3 ubiquitin ligase TRAF3IP2	XM_002662486.6
B7G0657	17.13	5.95	FIBIN	Fin bud initiation factor	NM_001017873.2

B22G0691	17.12	21.44	RB11B	Ras-related protein Rab-11B	NM_214770.1
B23G0284	17.12	29.19	HCK	Tyrosine-protein kinase HCK	XM_068216859.1
A3G0799	17.12	17.64	SLC25A39	Probable mitochondrial glutathione transporter SLC25A39	XM_005163930.5
B3G0959	17.12	11.70	HSD17B14	17-beta-hydroxysteroid dehydrogenase 14	NM_001003521.2
A23G0124	17.11	4.68	SLC25A22	Mitochondrial glutamate carrier 1	NM_001003448.1
A11G0064	17.11	8.15	LOC113110633	A0A6P6QBG7_CARAU titin-like isoform X1	
A3G0823	17.11	27.64	ABAT	4-aminobutyrate aminotransferase, mitochondrial	NM_201498.2
B2G1127	17.10	11.52	UCK2B	Uridine-cytidine kinase 2-B	NM_199764.2
B19G0111	17.10	9.52	ZNF384	Zinc finger protein 384	XM_005159417.5
A23G0106	17.10	1.39			XM_009296563.4
B14G0496	17.09	14.80	B4GALT1	Beta-1,4-galactosyltransferase 1	
B14G0571	17.09	18.22	AUP1	Lipid droplet-regulating VLDL assembly factor AUP1	XM_005173083.5
B22G0128	17.08	8.20	PIGX	Phosphatidylinositol-glycan biosynthesis class X protein	
A14G0577	17.08	10.85	GNPDA2	Glucosamine-6-phosphate isomerase 2	XM_679055.8
A8G0973	17.07	16.69	CDK11B	Cyclin-dependent kinase 11B	XM_068222936.1
B13G0766	17.07	19.89	BTAF1	TATA-binding protein-associated factor 172	NM_199907.1
A7G0763	17.07	11.74	GTF2A2	Transcription initiation factor IIA subunit 2	NM_001020605.1
B19G0309	17.07	4.99	TOMM40	Mitochondrial import receptor subunit TOM40 homolog	NM_212959.1
B8G0800	17.06	9.29	LOC107755292	A0A673HFZ6_9TELE Nudix hydrolase 24, chloroplastic-like	NM_001045397.1
B1G1085	17.06	5.69	CASPB	Caspase b	
A16G0111	17.05	8.91	GPSM3	G-protein-signaling modulator 3	
B8G0679	17.04	14.58	LPXN	Leupaxin	XM_684147.10
B1G0376	17.04	12.23	QDPR	Dihydropteridine reductase	NM_001020698.1
B13G0092	17.04	7.92	DST	Dystonin	NM_001080635.1
A13G0660	17.03	12.24	GRXCR1	Glutaredoxin domain-containing cysteine-rich protein 1	NM_199432.1
B13G0765	17.01	38.46	BTAF1	TATA-binding protein-associated factor 172	XM_005156494.5
A20G0366	17.01	5.04	PTGS2	H synthase 2	NM_001025504.2
B6G0103	17.01	9.28	UNC50	Protein unc-50 homolog	NM_200247.1
A3G0417	17.01	12.04	PRKCSH	Glucosidase 2 subunit beta	NM_201053.1
A3G1182	17.01	6.73	MCRIP1	Mapk-regulated corepressor-interacting protein 1	NM_001045471.2
A16G0368	17.00	22.98	PNRC2	K7DY92_DANRE Proline-rich nuclear receptor coactivator 2	NM_001130192.1
A12G0162	17.00	9.07	GPRC5C	G-protein coupled receptor family C group 5 member C	NM_001109713.1
B23G0593	16.99	7.28	AURKAIP1	Aurora kinase A-interacting protein	NM_001105695.1
B21G0045	16.98	6.58	SH3PXD2B	SH3 and PX domain-containing protein 2B	XM_005170288.4
B6G0917	16.98	12.87	UQCC1	Ubiquinol-cytochrome-c reductase complex assembly factor 1	NM_001076755.1
B25G0196	16.98	3.41	IGKV3-15	Immunoglobulin kappa variable 3-15	XM_068217596.1
A16G0717	16.98	14.34	LOC107598455	Leu-3	

A18G0798	16.98	7.30	DTWD1	tRNA-uridine aminocarboxypropyltransferase 1	NM_001005951.2
A1G0074	16.98	0.52	SAMHD1	Deoxynucleoside triphosphate triphosphohydrolase SAMHD1	
B18G0756	16.97	19.34	PDCD10A	Programmed cell death protein 10-A	NM_200555.2
B1G0875	16.96	29.81	PRDX1	Peroxiredoxin-1	NM_001002468.2
B22G0634	16.96	4.62	ADAM10	Disintegrin and metalloproteinase domain-containing protein 10	XM_684373.10
B17G0018	16.96	20.98	THBS1	Thrombospondin-1	XM_002666957.6
B1G0097	16.95	34.21	FN1	Fibronectin	XM_009305554.4
B6G1029	16.95	35.83	PRELID3B	PRELI domain containing protein 3B	NM_199734.1
B21G0182	16.95	27.41	PGRMC1	Membrane-associated progesterone receptor component 1	NM_001007392.1
B12G0492	16.95	10.82	MSRB1	Methionine-R-sulfoxide reductase B1-A	
A22G0300	16.94	7.51	BZW1A	eIF5-mimic protein 2-A	NM_199708.1
A19G0575	16.94	11.63	CNDP2	Cytosolic non-specific dipeptidase	NM_214704.2
B17G0268	16.94	17.84	COL10A1	Collagen alpha-1(X) chain	NM_001083827.1
A10G0474	16.94	18.55	BTG3	Protein BTG3	NM_001326708.1
A3G0923	16.94	18.83	PHB1	Prohibitin 1	NM_201297.1
A22G0199	16.94	5.62	G5714_021488	A0A7J6BR36_9TELE Ig-like domain-containing protein	XM_001336832.9
B9G0608	16.94	8.97	PARP4	Protein mono-ADP-ribosyltransferase PARP4	XM_017357877.2
A14G0799	16.94	19.13	SH2D1A	SH2 domain-containing protein 1A	
A15G0876	16.94	24.23	NIPSNAP2	Protein NipSnap homolog 2	NM_131034.1
A12G0020	16.94	2.62	MTR	Methionine synthase	NM_198072.1
B23G0291	16.93	68.93	SDCBP	Syntenin-1	NM_212691.2
A12G0200	16.93	9.87	CYTH1	Cytohesin-1	XM_068224523.1
B18G0109	16.93	48.17	APLP2	Amyloid beta precursor like protein 2	XM_005159251.5
B18G0556	16.92	17.85	BCL2L14	Apoptosis facilitator Bcl-2-like protein 14	
B12G0460	16.92	14.23	TOMM22	Mitochondrial import receptor subunit TOM22 homolog	NM_001001724.2
B12G0641	16.92	28.39	UQCRC2	Cytochrome b-c1 complex subunit 2, mitochondrial	NM_001001589.1
B18G0532	16.92	15.09	PSME3IP1	PSME3-interacting protein	XM_068214729.1
B15G0064	16.91	22.24	CUL3	Cullin-3	NM_001190485.1
B21G0319	16.91	21.73	RHOG	Rho-related GTP-binding protein RhoG	NM_200040.2
A16G0044	16.90	9.25	GRINA	Protein lifeguard 1	NM_001308611.1
B1G0818	16.90	16.59	RNF122	RING finger protein 122	NM_001114727.2
A8G0573	16.90	12.10	PEDS1	Plasmanylethanolamine desaturase	NM_001006052.1
B5G0724	16.90	8.01	DTX3	Probable E3 ubiquitin-protein ligase DTX3	NM_001123062.1
B5G0918	16.90	40.95	PPP6C	threonine-protein phosphatase 6 catalytic subunit	NM_201005.1
A13G0343	16.90	7.20	WDFY4	WD repeat- and FYVE domain-containing protein 4	NM_001013460.3
A21G0848	16.90	7.25	ZCCHC10	Zinc finger CCHC domain-containing protein 10	
A16G0332	16.88	7.88	MAP7D1	MAP7 domain-containing protein 1	XM_021466720.2

B17G0382	16.88	11.77	MRPS5	28S ribosomal protein S5, mitochondrial	NM_001076596.1
B8G0069	16.88	5.37	G5714_008821	A0A7J6CQF3_9TELE Uncharacterized protein	
A5G0453	16.88	13.20	TMEM150A	Transmembrane protein 150A	NM_001045410.1
B14G0248	16.87	20.63	UPF3B	Regulator of nonsense transcripts 3B	NM_200954.1
B8G0483	16.87	10.18	RIMKLB	Beta-citrylglutamate synthase B	NM_001004554.2
A6G0661	16.87	21.73	METTL7B	Thiol S-methyltransferase METTL7B	
B1G0052	16.87	12.82	G5714_001198	A0A7J6DIR5_9TELE Ig-like domain-containing protein	
A15G0380	16.87	20.35	PRPF8	Pre-mRNA-processing-splicing factor 8	NM_200976.2
B13G0227	16.86	2.82	NEFM	Neurofilament medium polypeptide	XM_684462.10
B6G0766	16.85	19.66	PARP3	Protein mono-ADP-ribosyltransferase PARP3	NM_200501.1
A8G0447	16.85	9.50	CF226	Uncharacterized protein C6orf226 homolog	
B2G0909	16.84	8.35	CNN3	Calponin-3	NM_001024073.2
B7G0338	16.84	11.57	NAGK	N-acetyl-D-glucosamine kinase	XM_005166546.4
A13G0756	16.84	7.23	EML4	Echinoderm microtubule-associated protein-like 4	NM_200605.1
A7G1023	16.83	14.64	ZNF638	Zinc finger protein 638	XM_021477756.2
B10G0196	16.83	35.36	EWSR1	RNA-binding protein EWS	NM_001115138.2
A1G1094	16.83	10.00	TMEM131L	Transmembrane protein 131-like	XM_688724.9
A7G0598	16.82	15.91	PSMC3	26S proteasome regulatory subunit 6A	XM_005168962.5
A14G0643	16.81	7.93			NM_201310.1
A15G0675	16.81	6.33	SLC7A1	High affinity cationic amino acid transporter 1	NM_001328209.1
B13G0256	16.80	26.20	POMK	Protein O-mannose kinase	NM_200549.1
A8G0590	16.79	23.10	TSPAN36	Tetraspanin-36	
A6G0216	16.79	17.18	OLA1	Obg-like ATPase 1	NM_214700.2
B20G0933	16.79	16.11	ARF6	ADP-ribosylation factor 6	NM_199993.1
A16G0652	16.79	7.24	EFNA1	Ephrin-A1	NM_200783.3
A23G0226	16.79	23.60	MAPRE1	EB family member 1	NM_213640.1
A3G0060	16.79	18.77	GIMAP7	GTPase IMAP family member 7	
B6G0130	16.78	14.17	SEC23A	Protein transport protein Sec23A	XM_017356444.3
A23G0725	16.77	36.92	TNFRSF14	Tumor necrosis factor receptor superfamily member 14	
B17G0498	16.77	12.34	LARP1	La-related protein 1	XM_691468.9
B12G0782	16.77	9.02	MED9	Mediator of RNA polymerase II transcription subunit 9	XM_002667433.7
B20G0829	16.76	5.14			
A19G0872	16.75	1.75	SP8B	Transcription factor Sp8	NM_213241.2
B11G0696	16.75	5.19	FLNB	Filamin-B	NM_001198743.1
A20G0719	16.75	11.10	PRDX6	Peroxiredoxin-6	
B5G1053	16.75	10.09	RNF181	E3 ubiquitin-protein ligase RNF181	NM_200306.1
A5G0726	16.75	9.33	LOC107661208	A0A671S6C6_9TELE RBR-type E3 ubiquitin transferase	NM_001386836.1
B3G0937	16.75	3.86	CLDN4	Claudin-4	NM_131768.2
A1G0054	16.74	44.79	NLRC3	Protein NLRC3	XM_068220504.1
A7G0254	16.74	8.52	FADD	FAS-associated death domain protein	
B16G0780	16.74	11.89	LOC107700193	A0A671T0K2_9TELE Tyrosine-protein kinase-like otk	
B9G0851	16.74	15.27	WIPF1	WASL-interacting protein family member 1	

A20G0766	16.74	16.25	PSMC6	26S proteasome regulatory subunit 10B	NM_001003832.1
B10G0697	16.73	15.01	ACTR1B	Beta-centractin	NM_213372.1
B14G0096	16.72	22.63	MATR3	Matrin-3	XM_068213212.1
B1G0761	16.71	8.72	TRAPPC5	Trafficking protein particle complex subunit 5	NM_001002482.1
A15G0845	16.71	7.79	BUD23	Probable 18S rRNA (guanine-N(7))-methyltransferase	NM_001082879.1
B3G0907	16.70	1.13	DPX16_16969	A0A3N0XCQ7_ANAGA Uncharacterized protein	
B7G0437	16.70	6.82	TRAF6	TNF receptor-associated factor 6	XM_068222300.1
B9G0493	16.69	38.82	COL6A3	Collagen alpha-3(VI) chain	XM_068218546.1
A17G0233	16.69	11.85	HADHA	Trifunctional enzyme subunit alpha, mitochondrial	NM_001089437.1
A3G0138	16.68	63.40	MYH9	Myosin-9	XM_005163701.5
A20G0494	16.68	8.15	A0A673K2J5	A0A673K2J5_9TELE Si:dkeyp-55f12.3	NM_001025552.2
A15G0551	16.68	8.96	VPS26B	Vacuolar protein sorting-associated protein 26B	NM_213375.1
B25G0129	16.68	2.00	IGKV4-1	Immunoglobulin kappa variable 4-1	XM_068217596.1
B3G0692	16.68	17.17	CRLF3	Cytokine receptor-like factor 3	NM_001017817.2
B5G0203	16.66	9.01	SDF2L1	Stromal cell-derived factor 2-like protein 1	NM_001003730.1
B9G0216	16.66	13.25	STAT4	Signal transducer and activator of transcription 4	XM_005167880.5
A24G0011	16.66	49.60	SSR1	Translocon-associated protein subunit alpha	NM_201327.1
A3G0361	16.66	13.27	SLC27A1	Long-chain fatty acid transport protein 1	NM_001013537.1
B16G0362	16.66	9.87	SH3KBP1	SH3 domain-containing kinase-binding protein 1	XM_005158311.5
B14G0685	16.65	4.87	LOC113113595	A0A6P6QTK0_CARAU uncharacterized protein LOC113113595	XM_021472768.2
B5G0370	16.65	12.99	HNRNPK	Heterogeneous nuclear ribonucleoprotein K	NM_212991.3
A22G0284	16.65	28.55	STAT1	Signal transducer and activator of transcription 1	NM_131480.1
A19G0670	16.64	16.56	LOC113062158	A0A6P6LSW3_CARAU chromosomal protein D1-like	XM_701324.10
A3G1090	16.64	9.13	KRI1	Protein KRI1 homolog	NM_001002041.1
A23G0458	16.63	22.66	IDH3G	Isocitrate dehydrogenase [NAD] subunit gamma 1, mitochondrial	NM_001243172.1
A16G0874	16.63	6.73	LOC107656710	A0A671P5J6_9TELE Protein CROWDED NUCLEI 3-like	NM_001326612.1
A6G0700	16.63	9.51	TOMM6	Mitochondrial import receptor subunit TOM6 homolog	
A7G0255	16.62	7.98	PACSIN3	Protein kinase C and casein kinase II substrate protein 3	NM_200989.1
B12G0422	16.62	11.80	STX4	Syntaxin-4	NM_200221.1
B23G0680	16.62	34.17	CS	Citrate synthase, mitochondrial	NM_199598.1
B25G0130	16.61	0.00	IGKV1-12	Immunoglobulin kappa variable 1-12	NM_001045408.1
A16G0009	16.61	5.83	ZBTB7B	Zinc finger and BTB domain-containing protein 7B	NM_200629.2
A17G0662	16.61	23.93	PNN	Pinin	NM_001172570.1
A23G0739	16.60	0.32	TGM1	Protein-glutamine gamma-glutamyltransferase K	XM_001331878.8
B19G0017	16.59	5.53	G5714_019159	A0A7J6C2I9_9TELE Uncharacterized protein	

A13G0113	16.58	39.14	SMOC2	SPARC-related modular calcium-binding protein 2	NM_200372.1
A8G0698	16.57	1.62			
B12G0773	16.57	8.40	METTL9	Protein-L-histidine N-pros-methyltransferase	XM_005169429.4
A19G0730	16.57	3.06	CDCP1	CUB domain-containing protein 1	
B4G0399	16.56	7.90	CHCHD3	MICOS complex subunit MIC19	XM_009300263.4
B9G0497	16.54	5.22	RPE	Ribulose-phosphate 3-epimerase	NM_001243424.1
A9G0376	16.53	10.36	TFG	Protein TFG	NM_200015.2
B1G0580	16.52	14.48	LSM6	U6 snRNA-associated Sm-like protein LSm6	NM_001002077.2
A17G0391	16.52	11.37	CNKSR1	Connector enhancer of kinase suppressor of ras 1	XM_009293086.4
B2G0957	16.52	8.72	PTPRF	Receptor-type tyrosine-protein phosphatase F	XM_068212951.1
B8G0780	16.52	5.17	MRPL55	39S ribosomal protein L55, mitochondrial	NM_001111179.1
B25G0339	16.51	24.29	CIART	Circadian-associated transcriptional repressor	XM_695543.10
B13G0548	16.51	18.66	PALD1	Paladin	XM_021480667.2
A15G0583	16.51	17.00	CLTC	Clathrin heavy chain 1	NM_001194978.1
B22G0719	16.51	7.77	GZMG	Granzyme G	
B11G0379	16.50	10.50			NM_001002875.1
A19G0294	16.50	21.78	FUCA1	Tissue alpha-L-fucosidase	NM_001003559.2
A1G0735	16.50	9.91	GART	Trifunctional purine biosynthetic protein adenosine-3	NM_131617.1
A12G0328	16.50	5.93	SKAP1	Src kinase-associated phosphoprotein 1	NM_001080011.2
A14G0079	16.50	10.48	HSPA4	Heat shock 70 kDa protein 4	NM_199857.1
B19G0412	16.49	21.86	CTNNB1	Catenin beta-1	NM_001001889.1
B20G0860	16.49	5.29	COL12A1	Collagen alpha-1(XII) chain	XM_017352646.3
A15G0842	16.49	17.82	SRPRB	Signal recognition particle receptor subunit beta	NM_001002572.1
B2G0455	16.49	6.79	ING5	Inhibitor of growth protein 5	XM_005171273.5
B24G0671	16.48	13.64	ABI1	Abl interactor 1	XM_009297228.4
B3G1172	16.48	32.92	CTSD	Cathepsin D	NM_200031.1
A20G0043	16.47	12.06	PPP2R5C	threonine-protein phosphatase 2A 56 kDa regulatory subunit gamma isoform	XM_005160898.5
B4G0736	16.47	1.72	MS4A4A	Membrane-spanning 4-domains subfamily A member 4A	XM_009301043.4
B4G0466	16.47	13.73	PLXNC1	Plexin-C1	XM_068220763.1
A16G1031	16.46	10.46	PLEC	Plectin	XM_009292043.4
A15G0501	16.46	9.53	SDHDB	Succinate dehydrogenase [ubiquinone] cytochrome b small subunit B, mitochondrial	NM_001004004.2
A19G0708	16.45	9.14	UBAP2L	Ubiquitin-associated protein 2-like	NM_001083066.1
B13G0107	16.45	19.56	LIN1	LINE-1 reverse transcriptase homolog	XM_068212887.1
B2G0308	16.44	11.48	LACTB2	Endoribonuclease LACTB2	NM_212884.1
A16G0188	16.44	8.47	DCAF13	DDB1- and CUL4-associated factor 13	NM_200129.1
B14G0223	16.44	1.58	SOX3	Transcription factor Sox-3	NM_001001811.2
B4G0031	16.44	8.46	ETV6	Transcription factor ETV6	NM_131832.2
A3G0368	16.43	6.18	PGLS	6-phosphogluconolactonase	NM_001004117.3
B19G0095	16.43	16.55	SDHA	Succinate dehydrogenase [ubiquinone] flavoprotein subunit, mitochondrial	NM_200910.1
A15G0435	16.42	11.15	CRK	Adapter molecule crk	NM_001003628.2
B11G0793	16.42	12.28			NM_199896.2

B16G0146	16.42	3.93	RAB25	Ras-related protein Rab-25	NM_001029971.1
B16G0298	16.42	3.96	CD163	Scavenger receptor cysteine-rich type 1 protein M130	XM_021466988.2
B21G0667	16.42	7.22	NOC4L	Nucleolar complex protein 4 homolog	NM_001024397.1
A3G0513	16.42	8.80	DRG2	Developmentally-regulated GTP-binding protein 2	NM_212638.1
A3G0658	16.40	7.83			NM_001423471.1
A25G0664	16.39	10.03	IGLC7	Immunoglobulin lambda constant 7	XM_068217596.1
A21G0886	16.39	1.75	HPGD	15-hydroxyprostaglandin dehydrogenase [NAD(+)]	NM_200327.2
A14G0274	16.39	4.66	ITK	TSK	NM_131104.2
A24G0028	16.38	25.78	NUB1	NEDD8 ultimate buster 1	XM_009297673.4
B20G0880	16.38	15.00	NFKBIE	NF-kappa-B inhibitor epsilon	NM_001080089.1
B8G0833	16.38	13.74	DENND2D	DENN domain-containing protein 2D	NM_001430991.1
A22G0130	16.37	6.27	NCL1	Nicalin-1	NM_207057.1
B2G0436	16.37	25.28	ELAVL1	ELAV-like protein 1	XM_017357655.2
A25G0487	16.36	24.73	DLD	Dihydrolipoyl dehydrogenase, mitochondrial	NM_201506.1
B16G0465	16.36	128.47	A0A672JXG5	A0A672JXG5_SINGR Uncharacterized protein	NM_001386419.1
B23G0978	16.36	10.51	EIF4E2	Eukaryotic translation initiation factor 4E type 2	NM_200759.2
B22G0697	16.35	9.75	POLR2E	DNA-directed RNA polymerases I, II, and III subunit RPABC1	NM_001003564.2
B23G0125	16.34	1.52	CDH26	Cadherin-like protein 26	XM_017353725.3
A18G0447	16.34	4.42	AGK	Acylglycerol kinase, mitochondrial	NM_199880.1
B21G0755	16.34	14.05	TCB1	Transposable element Tcb1 transposase	XM_021470094.2
A2G0815	16.34	15.54	PEX19	Peroxisomal biogenesis factor 19	NM_001017399.1
B8G0149	16.33	18.87	UBL4A	Ubiquitin-like protein 4A	NM_200300.1
B14G0094	16.33	2.70	A0A672HHI8	A0A672HHI8_SALFA ADAM metallopeptidase domain 9	
B7G0754	16.33	9.28	VPS13C	Intermembrane lipid transfer protein VPS13C	
B8G0828	16.33	6.67	SAP30BP	SAP30-binding protein	NM_199565.2
B11G0243	16.32	25.30	SLC39A3	Zinc transporter ZIP3	NM_200928.1
A21G0337	16.31	8.60	MRPS18C	28S ribosomal protein S18c, mitochondrial	NM_001045190.2
B19G0096	16.31	17.09	RXRBA	Retinoic acid receptor RXR-beta-A	XM_009294022.3
A10G0693	16.30	15.37	MAT2A	S-adenosylmethionine synthase isoform type-2	NM_001290080.1
B1G0244	16.29	11.08	ZGC:77739	UPF0462 protein C4orf33 homolog	NM_200896.2
B2G0121	16.29	16.78	YJU2	Splicing factor YJU2	NM_001110833.1
B7G0543	16.29	7.83	GRPEL1	GrpE protein homolog 1, mitochondrial	NM_001037384.2
A19G0735	16.28	14.88	HSF1	Heat shock factor protein 1	XM_009293954.4
A6G0772	16.27	13.40			NM_205566.1
A16G0657	16.27	11.05			XM_005169756.5
B19G0003	16.27	7.03	TNFAIP8L2A	Tumor necrosis factor, alpha-induced protein 8-like protein 2 A	NM_001045301.1
B24G0530	16.27	3.06			NM_001386530.1
B23G0650	16.27	10.19	DGKA	Diacylglycerol kinase alpha	NM_001045315.1
A3G0652	16.26	28.76	MUCM	Ig mu chain C region membrane-bound form	XM_068217859.1

A18G0090	16.26	17.48	EIF3M	Eukaryotic translation initiation factor 3 subunit M	NM_001024735.1
A6G0456	16.26	24.23	GSN	Gelsolin	NM_178131.3
A24G0237	16.25	25.17	CNN3	Calponin-3	NM_199753.1
B7G1056	16.25	11.47	MAP4K2	Mitogen-activated protein kinase kinase kinase kinase 2	NM_001040358.1
B1G0869	16.25	17.98	PPP3R1	Calcineurin subunit B type 1	NM_001004553.1
A1G0409	16.24	14.62	DNAJA1	DnaJ homolog subfamily A member 1	NM_199662.1
B6G0912	16.24	23.44	ESYT1	Extended synaptotagmin-1	XM_694639.10
B1G1179	16.24	9.49	THUMPD1	THUMP domain-containing protein 1	NM_205657.2
A10G0191	16.24	12.05	ACTR1B	Beta-centractin	NM_213372.1
B3G0205	16.24	42.25	TEF	Thyrotroph embryonic factor	NM_001020661.2
A11G0099	16.23	13.75	APPL1	DCC-interacting protein 13-alpha	NM_201023.1
B13G0409	16.23	18.89	PCNX2	Pecanex-like protein 2	
B25G0761	16.22	11.84	PARVG	Gamma-parvin	NM_001089538.1
B11G0774	16.22	7.63	SOS1	Son of sevenless homolog 1	NM_001077777.2
A5G1039	16.21	9.90	DERL2	Derlin-2	NM_001024395.1
A22G0015	16.21	6.09	ATAD3	ATPase family AAA domain-containing protein 3	NM_205703.1
A6G0522	16.21	8.53	IUNH	Inosine-uridine preferring nucleoside hydrolase	NM_001030089.2
A8G0118	16.21	16.89	TMEM127	Transmembrane protein 127	NM_001020524.1
A23G0632	16.21	14.58	TFCP2	Transcription factor CP2	XM_005162355.5
B15G0780	16.20	10.21	STIM1	Stromal interaction molecule 1	XM_005173393.5
B14G0562	16.20	3.59	TGFB2	Transforming growth factor beta-2 proprotein	XM_683088.6
A19G0887	16.20	11.21	TGFBR2	TGF-beta receptor type-2	XM_009293931.4
B23G0282	16.20	18.66	TM9SF4	Transmembrane 9 superfamily member 4	NM_200510.2
B16G0952	16.19	90.33	ECM1	Extracellular matrix protein 1	XM_005173500.5
B13G0908	16.19	26.98			NM_212700.1
A1G1117	16.18	39.82	MTAP	S-methyl-5'-thioadenosine phosphorylase	NM_200554.1
B10G0850	16.18	15.47	MPZL1	Myelin protein zero-like protein 1	NM_001257340.1
B20G0244	16.18	10.27	BLOC1S2	Biogenesis of lysosome-related organelles complex-1 subunit 2	NM_001033101.2
B5G0206	16.18	31.55	CCL19	C-C motif chemokine 19	
B2G1228	16.18	3.31	SSX2IP	Afadin- and alpha-actinin-binding protein	NM_200835.2
A5G0927	16.17	1.66	SLC16A13	Monocarboxylate transporter 13	NM_001029972.2
B2G0422	16.17	12.60	DAPK3	Death-associated protein kinase 3	XR_011006884.1
B15G0366	16.17	5.23	ADAMTS8	A disintegrin and metalloproteinase with thrombospondin motifs 8	XM_021472930.2
B7G0078	16.16	8.54	SLC29A2	Equilibrative nucleoside transporter 2	
A20G0418	16.16	49.73	SOD2	Superoxide dismutase [Mn], mitochondrial	NM_199976.1
B9G0875	16.16	8.67	PRPF40A	Pre-mRNA-processing factor 40 homolog A	NM_198356.1
B11G0109	16.15	20.03	CS025	UPF0449 protein C19orf25 homolog	XM_005168060.5
B22G0865	16.15	1.56	CAPN2	Calpain-2 catalytic subunit	NM_001003485.2
A4G1033	16.15	28.03	MAPRE3	EB family member 3	NM_001002170.1
A25G0461	16.15	12.32	NFYB	Nuclear transcription factor Y subunit beta	NM_001013322.2

B2G0747	16.15	18.02	ACADM	Medium-chain specific acyl-CoA dehydrogenase, mitochondrial	NM_213010.2
A11G0362	16.14	7.85	MYH7B	Myosin-7B	NM_001048235.1
B19G0416	16.14	6.73	CCK	Cholecystokinin	
A7G0141	16.13	23.40	PPP1R15A	Protein phosphatase 1 regulatory subunit 15A	
B20G0010	16.13	18.57	YPEL5	Protein yippee-like 5	NM_001326453.1
A11G0663	16.13	23.89	SPCS1	Signal peptidase complex subunit 1	NM_131584.2
B18G0504	16.12	6.92	SNAPC5	snRNA-activating protein complex subunit 5	XM_005159085.5
B16G0792	16.12	38.69	CD209D	CD209 antigen-like protein D	
B7G0984	16.12	9.28	SDHAF2	Succinate dehydrogenase assembly factor 2, mitochondrial	NM_001082864.1
A8G0795	16.11	8.56	LSM1	U6 snRNA-associated Sm-like protein LSm1	NM_001003551.1
B16G0703	16.11	6.89	EFNA1	Ephrin-A1	NM_200783.3
A21G0896	16.11	17.28	DDX46	Probable ATP-dependent RNA helicase DDX46	XM_009295737.4
B14G0113	16.10	13.17	GM2A	Ganglioside GM2 activator	NM_001017831.2
B15G0893	16.10	18.69	PSMD2	26S proteasome non-ATPase regulatory subunit 2	NM_200546.1
A7G0915	16.10	14.96	ACIN1	Apoptotic chromatin condensation inducer in the nucleus	NM_001305582.1
B13G0651	16.10	11.28	SLX4IP	Protein SLX4IP	NM_001128820.2
A10G0710	16.10	36.17	PLPP1	Phospholipid phosphatase 1	XM_692415.10
B17G0230	16.09	16.86	FAM177A1	Protein FAM177A1	NM_001105707.1
B1G0405	16.08	9.58	DCTPP1	dCTP pyrophosphatase 1	
A14G0260	16.08	11.93	SASH3	SAM and SH3 domain-containing protein 3	XM_685351.10
A10G0552	16.08	14.28	PAXBP1	PAX3- and PAX7-binding protein 1	NM_001080183.2
A3G0747	16.07	16.29	DCAF7	DDB1- and CUL4-associated factor 7	NM_200069.1
A12G0781	16.07	15.47	PAGR1	PAXIP1-associated glutamate-rich protein 1	
B22G0537	16.07	7.98	LSM7	U6 snRNA-associated Sm-like protein LSm7	NM_001048006.1
A16G0357	16.06	9.34	SNIP1	Smad nuclear interacting protein 1	NM_001025470.1
A25G0501	16.06	21.37	IDH3A	Isocitrate dehydrogenase [NAD] subunit alpha, mitochondrial	NM_200951.2
A14G0388	16.05	14.85	EHD1	EH domain-containing protein 1	NM_212874.2
A4G0753	16.05	4.56	MRPS33	28S ribosomal protein S33, mitochondrial	NM_001002306.2
A16G0452	16.04	4.20	TMEM79	Transmembrane protein 79	
B2G0059	16.04	138.10	GPX4	Phospholipid hydroperoxide glutathione peroxidase	NM_001030070.2
A8G0382	16.04	12.31	TMCO1	Calcium load-activated calcium channel	NM_001002575.1
A21G0569	16.03	30.98	SART1	U6.U5 tri-snRNP-associated protein 1	NM_001002673.1
B3G0712	16.03	1.65	DLX4A	Homeobox protein Dlx4a	NM_131300.2
B17G0115	16.03	10.39	DNAJC5	DnaJ homolog subfamily C member 5	
B9G0107	16.02	24.92	CD302	CD302 antigen	
A22G0560	16.02	11.31	SDE2	Replication stress response regulator SDE2	XM_009296385.4
A15G0523	16.02	21.58	CHP1	Calcineurin B homologous protein 1	NM_199836.2

B8G0104	16.02	8.20	TC1A	Transposable element Tc1 transposase	
B12G0305	16.01	8.10	PSMD11B	26S proteasome non-ATPase regulatory subunit 11B	NM_199592.1
B5G1046	16.01	11.19	ARAF	threonine-protein kinase A-Raf	XM_005171818.5
B14G0768	16.01	20.08	THOC2	THO complex subunit 2	XM_005157134.5
B24G0153	16.00	3.56	CCSMST1	Protein ccsmst1	
A17G0009	16.00	6.60	DDX24	ATP-dependent RNA helicase DDX24	XM_003200406.5
A2G0254	15.97	14.54	NCBP2AS2	Protein NCBP2AS2	
B19G0597	15.97	9.12	TRAPPC3	Trafficking protein particle complex subunit 3	NM_001003601.1
B5G0210	15.96	0.00			XM_068221580.1
B6G0257	15.96	8.32	MARCHF7	E3 ubiquitin-protein ligase MARCHF7	XM_005165760.5
A6G0374	15.96	15.31	SRP68	Signal recognition particle subunit SRP68	NM_001005401.3
B19G0573	15.96	25.85	SFPQ	Splicing factor, proline- and glutamine-rich	XR_222585.4
B13G0541	15.96	6.88	TRMT2B	tRNA (uracil-5-)-methyltransferase homolog B	
B15G0252	15.96	4.08	TNFRSF19	Tumor necrosis factor receptor superfamily member 19	NM_001044904.1
B4G0441	15.96	9.80	NUP37	Nucleoporin Nup37	NM_001002619.1
B2G1243	15.95	20.38	SS18	Protein SSXT	NM_199744.2
B19G0211	15.94	6.81	GABPB2	GA-binding protein subunit beta-2	NM_001099977.2
B16G0902	15.93	9.04	HIBADH	3-hydroxyisobutyrate dehydrogenase, mitochondrial	NM_201160.1
B13G0226	15.93	26.34	IFIT1	Interferon-induced protein with tetratricopeptide repeats 1	NM_214759.1
B10G0420	15.93	12.96	UQCC3	Ubiquinol-cytochrome-c reductase complex assembly factor 3	
B5G0325	15.93	14.37	STIP1	Stress-induced-phosphoprotein 1	NM_001007766.2
A1G0753	15.92	34.56	ATP1A1	potassium-transporting ATPase subunit alpha-1	NM_131686.1
B11G0128	15.92	6.16	ROHU_014258	A0A498NH34_LABRO Homeodomain-interacting kinase 1-like protein	NM_001351798.1
B7G1135	15.92	80.51	CNFN	Cornifelin	NM_001327748.1
B1G0617	15.91	9.77	CDK2AP2	Cyclin-dependent kinase 2-associated protein 2	NM_001009898.1
B24G0615	15.91	14.78	PRPF4B	threonine-protein kinase PRP4 homolog	XM_068217049.1
B3G0441	15.91	5.72	FQN60_003873	A0A5J5CVK8_9PERO Uncharacterized protein	
B10G0540	15.91	3.34	RIPK4	threonine-protein kinase 4	XM_068223759.1
B6G1052	15.90	11.22	AAMP	Angio-associated migratory cell protein	
B20G0150	15.89	7.55	EZR	Ezrin	NM_001030285.2
A2G0022	15.89	9.49	SIRT5	NAD-dependent protein deacylase sirtuin-5, mitochondrial	XM_068213385.1
A15G0349	15.88	25.37	SERPINH1	Serpin H1	NM_131204.2
B6G0772	15.87	21.57	EMC3	ER membrane protein complex subunit 3	NM_213505.2
A6G0356	15.87	7.03	NPRL2	GATOR complex protein NPRL2	NM_001045847.2
A23G0112	15.87	3.84	TWIST1	Twist-related protein 1	NM_001017820.1

B19G0700	15.87	8.00	MRPS18B	28S ribosomal protein S18b, mitochondrial	NM_001017759.2
A7G1209	15.86	7.27	AADAT	alpha-aminoadipate aminotransferase, mitochondrial	XM_681142.10
A3G0295	15.85	10.96	SUN1	SUN domain-containing protein 1	NM_001018135.2
A13G0264	15.85	10.11	CFL2	Cofilin-2	NM_001002418.1
B2G0596	15.85	1.87	TC1A	Transposable element Tc1 transposase	XM_021470094.2
B1G0735	15.85	0.09	LOC113089746	A0A6P6NTV1_CARAU uncharacterized protein LOC113089746	XM_021476859.2
B5G0286	15.84	23.15	A0A673LZZ1	A0A673LZZ1_9TELE SGNH_hydro domain-containing protein	XM_068219418.1
A8G0886	15.84	20.46	NFU1	NFU1 iron-sulfur cluster scaffold homolog, mitochondrial	NM_001122708.1
A15G0104	15.84	16.24	NLRC3	Protein NLRC3	XM_017352412.3
A22G0575	15.84	23.87	MCRIP2	MAPK regulated corepressor interacting protein 2	NM_001045127.3
B21G0799	15.83	9.89	DCK	Deoxycytidine kinase	NM_001007056.1
B1G0248	15.83	8.49	ELF2	ETS-related transcription factor Elf-2	XM_005159830.5
B16G0850	15.83	6.97	MRPL13	39S ribosomal protein L13, mitochondrial	NM_001020534.1
B25G0666	15.83	6.87	CK024	Uncharacterized protein C11orf24 homolog	XM_685812.10
B11G0728	15.83	8.37	CLASP1-A	CLIP-associating protein 1-A	NM_001024407.2
B17G0589	15.83	3.04	CINP	Cyclin-dependent kinase 2-interacting protein	NM_001202484.2
A11G0207	15.82	9.00	WDR18	WD repeat-containing protein 18	NM_001328594.1
A14G0316	15.82	14.94	RNF130	E3 ubiquitin-protein ligase RNF130	NM_001020465.2
B14G0068	15.80	25.21	GNPDA1	Glucosamine-6-phosphate isomerase 1	NM_001017867.1
B7G1063	15.80	15.04	MTA2	Metastasis-associated protein MTA2	NM_214695.1
B24G0298	15.80	18.39	SH3GLB1	Endophilin-B1	XM_009297515.4
A17G0396	15.80	1.60	G5714_017064	A0A7J6C5I5_9TELE Uncharacterized protein	NM_001327925.1
B25G0475	15.79	10.75	AEN	Apoptosis-enhancing nuclease	
B3G0170	15.79	25.10	GIMAP8	GTPase IMAP family member 8	NM_001386434.1
B8G0331	15.79	2.26	GBGT1	Globoside alpha-1,3-N-acetylgalactosaminyltransferase 1	
A4G0842	15.79	19.77	ADIPOR2	Adiponectin receptor protein 2	NM_001025506.2
B24G0011	15.77	2.30	TOP1	DNA topoisomerase 1	NM_001001838.1
A7G0748	15.77	11.59	TMED3	Transmembrane emp24 domain-containing protein 3	NM_001128668.1
B13G0826	15.77	12.69			NM_001002712.2
B1G0441	15.76	12.24	USO1	General vesicular transport factor p115	NM_200155.1
B11G0429	15.75	3.29	SLA2	Src-like-adapter 2	NM_001327743.1
A15G0292	15.75	29.00	HMGB1	High mobility group protein B1	NM_001099251.2
B17G0343	15.75	1.37			NM_199985.2
B6G0287	15.75	8.76	ZDBF2	DBF4-type zinc finger-containing protein 2	NM_001326473.1
A20G0740	15.75	26.89	CALMA	Calmodulin-alpha (Fragment)	NM_200082.1
A19G0268	15.75	6.60	SCIN	Scinderin	NM_001045118.2
A19G0047	15.74	27.75	SF3A3	Splicing factor 3A subunit 3	NM_001004289.1

B8G0551	15.74	19.04	AHCYL1	S-adenosylhomocysteine hydrolase-like protein 1	NM_201293.2
B6G0990	15.74	32.79	RTF2	Replication termination factor 2	XM_005168778.3
A8G0062	15.74	5.48			
B22G0066	15.74	5.34	G5714_021137	A0A7J6BQ33_9TELE Uncharacterized protein	
A3G0723	15.74	11.33	LOC113045795	A0A6P6JQX5_CARAU uncharacterized protein LOC113045795 isoform X1	
B16G0145	15.73	4.54	RAB11A	Ras-related protein Rab-11A	NM_200123.1
B4G0039	15.73	35.49	CREBL2	cAMP-responsive element-binding protein-like 2	NM_001002702.2
A18G0010	15.72	6.66	CTTN	Src substrate cortactin	NM_001423896.1
A20G0006	15.72	2.47	PLCB1	1-phosphatidylinositol 4,5-bisphosphate phosphodiesterase beta-1	XM_068215585.1
B18G0806	15.72	17.41	SERP1	Stress-associated endoplasmic reticulum protein 1	
A8G0294	15.71	14.28	NDUFAF2	NADH dehydrogenase [ubiquinone] 1 alpha subcomplex assembly factor 2	NM_001113650.1
B22G0050	15.71	39.25	NLRP3	NACHT, LRR and PYD domains-containing protein 3	XM_068216562.1
A19G0224	15.71	8.31	NSUN2	RNA cytosine C(5)-methyltransferase NSUN2	NM_199711.1
A14G0757	15.71	6.90	NUP62	Nuclear pore glycoprotein p62	NM_001113596.1
A1G0403	15.70	0.98	G5714_000521	A0A7J6DGR0_9TELE Uncharacterized protein	
A5G0769	15.69	13.44	GOLPH3	Golgi phosphoprotein 3	XM_005165301.5
B10G0541	15.68	5.13	NSUN5	28S rRNA (cytosine-C(5))-methyltransferase	XM_009305396.3
A8G0528	15.67	22.33	IMPDH2	Inosine-5'-monophosphate dehydrogenase 2	XM_021478184.2
A1G0436	15.67	44.62	LOC107556796	A0A672Q7Z5_SINGR Translation initiation factor IF-2-like	NM_001145239.1
A16G0282	15.67	11.84	K1143	Uncharacterized protein KIAA1143 homolog	NM_212698.2
A19G0528	15.66	8.39	MRPL3	39S ribosomal protein L3, mitochondrial	XM_005159511.3
B2G1318	15.66	38.05	TCB1	Transposable element Tcb1 transposase	
A3G0042	15.66	38.05	TCB1	Transposable element Tcb1 transposase	
B20G0484	15.66	58.39			NM_001044755.1
B1G1101	15.66	2.20	SAMHD1	Deoxynucleoside triphosphate triphosphohydrolase SAMHD1	XM_068220881.1
B1G0582	15.66	38.05	TCB1	Transposable element Tcb1 transposase	
B22G0367	15.66	38.05	TCB1	Transposable element Tcb1 transposase	
A7G0265	15.66	38.05	TCB1	Transposable element Tcb1 transposase	
A8G0804	15.65	14.89	OGDH	2-oxoglutarate dehydrogenase complex component E1	XM_005172469.5
A8G0021	15.65	18.11	SNX2	Sorting nexin-2	NM_001037698.1
B14G0466	15.65	13.82	DCTN4	Dynactin subunit 4	NM_001017782.2
B20G0695	15.64	45.99	CLU	Clusterin	NM_200802.2
B17G0196	15.64	9.69	FBXO34	F-box only protein 34	NM_001305546.1
A3G0902	15.64	15.37	DIG-1	Mesocentin	XM_005163846.5
B15G0548	15.64	22.34	RPA1	Replication protein A 70 kDa DNA-binding subunit	NM_199811.2

B10G0366	15.63	6.09	LOC113109802	A0A6P6Q7E7_CARAU uncharacterized protein LOC113109802	XM_021479349.2
A25G0805	15.63	4.25	GIMAP8	GTPase IMAP family member 8	
B25G0073	15.63	10.81	CLPX	ATP-dependent Clp protease ATP-binding subunit clpX-like, mitochondrial	XM_002666958.7
A16G0884	15.63	20.17	PSMD4	26S proteasome non-ATPase regulatory subunit 4	NM_001017624.2
A22G0637	15.62	27.45	SLC6A6	Sodium- and chloride-dependent taurine transporter	NM_001037661.1
A8G0343	15.62	18.65	ZFAND5	AN1-type zinc finger protein 5	XM_005166804.5
B19G0184	15.62	8.74	SMIM13	Small integral membrane protein 13	NM_001135579.2
A22G0620	15.62	20.61	PDCD4	Programmed cell death protein 4	NM_198978.1
A7G0859	15.62	16.23	ARR1	Arrestin red cell isoform 1	NM_201124.1
B7G0622	15.61	8.19	IRX3	Iroquois-class homeodomain protein irx-3	NM_001320125.1
B14G0739	15.61	15.48	IMMT	MICOS complex subunit MIC60	NM_001001401.1
B18G0073	15.61	17.86	AP2M1B	AP-2 complex subunit mu-B	NM_201026.2
B21G0159	15.61	1.60	G5714_020301	A0A7J6BU62_9TELE Uncharacterized protein	
A7G0208	15.60	8.75	ADM	Pro-adrenomedullin	NM_001326709.1
B22G0820	15.60	10.44	RGS1	Regulator of G-protein signaling 1	XM_001332065.9
B4G0122	15.59	9.21	SSBP1	Single-stranded DNA-binding protein, mitochondrial	NM_001017806.1
A5G0484	15.59	11.64	VAMP5	Vesicle-associated membrane protein 5	
A19G0324	15.59	21.45	ELMO1	Engulfment and cell motility protein 1	XM_068215106.1
B22G1114	15.58	10.03	NCBP2	Nuclear cap-binding protein subunit 2	NM_173249.1
A25G0358	15.58	6.60	SAMM50A	Sorting and assembly machinery component 50 homolog A	NM_200142.2
A24G0566	15.58	12.70	TMEM14C	Transmembrane protein 14C	XM_005162574.5
A9G0166	15.58	11.36	G5714_009872	A0A7J6CN90_9TELE CARD domain-containing protein	
A5G0771	15.57	14.16	ZFR	Zinc finger RNA-binding protein	NM_199558.2
B21G0014	15.57	6.94	SAP30L	Histone deacetylase complex subunit SAP30L	NM_214703.1
A5G1274	15.56	12.96	UGT2A1	UDP-glucuronosyltransferase 2A1	NM_001191050.1
B7G1112	15.56	10.23	CPRAS1	Circularly permutated Ras protein 1	XM_001922896.7
B15G0327	15.55	56.77	SRSF1A	arginine-rich splicing factor 1A	XM_005157415.5
B12G0147	15.55	8.86	MAP2K6	Dual specificity mitogen-activated protein kinase kinase 6	NM_001312870.1
B1G0835	15.55	18.12	HADH	Hydroxyacyl-coenzyme A dehydrogenase, mitochondrial	NM_001003515.1
A1G0468	15.55	17.12	POLD4	DNA polymerase delta subunit 4	NM_001256200.1
A18G0735	15.55	12.73	EIF2A	Eukaryotic translation initiation factor 2A	NM_001025170.2
B5G0012	15.54	15.07	MBD2	Methyl-CpG-binding domain protein 2	NM_212768.1
B2G0394	15.54	12.08	HNRNPC	C2	XM_017358046.3
A7G0886	15.54	20.46	OTUB1	Ubiquitin thioesterase OTUB1	NM_001002500.2
B7G0106	15.54	45.76	PPP1R15A	Protein phosphatase 1 regulatory subunit 15A	
A12G0795	15.53	11.63	RFNG	Beta-1,3-N-acetylglucosaminyltransferase radical fringe	NM_001001830.2

B7G0555	15.53	10.77	LSP1	Lymphocyte-specific protein 1	NM_001423832.1
A18G0726	15.53	11.11	AAMDC	Mth938 domain-containing protein	NM_001020764.1
B3G0998	15.53	8.94	ATF7IP2	Activating transcription factor 7-interacting protein 2	XM_009299462.4
A8G0484	15.53	14.78	NFYA	Nuclear transcription factor Y subunit alpha	XM_009303999.4
B6G0425	15.53	51.93	LOC107704012	A0A671K1Y7_9TELE Uncharacterized LOC107704012	
B19G0644	15.52	22.59	MTDH	Protein LYRIC	NM_001007135.2
B7G0759	15.52	9.80	GTF2A2	Transcription initiation factor IIA subunit 2	NM_001020605.1
A20G0503	15.52	19.78	CDCA4	Cell division cycle-associated protein 4	NM_001008580.1
A12G0510	15.52	3.27	BAIAP2L1	Brain-specific angiogenesis inhibitor 1-associated protein 2-like protein 1	XM_009306593.4
A8G0256	15.51	52.48	RHOAA	Rho-related GTP-binding protein RhoA-A	NM_213137.1
A13G0120	15.51	25.73	MRPL14	39S ribosomal protein L14, mitochondrial	NM_001083833.1
A1G0869	15.51	16.29	RNF175	RING finger protein 175	NM_001328343.1
B25G0374	15.50	11.82	NFYB	Nuclear transcription factor Y subunit beta	NM_001013322.2
A2G0712	15.50	17.65	PLK3	threonine-protein kinase PLK3	NM_201308.2
B5G0901	15.50	6.75	NTMT1	N-terminal Xaa-Pro-Lys N-methyltransferase 1	NM_212898.1
A3G0947	15.50	7.81	METTL2A	tRNA N(3)-methylcytidine methyltransferase METTL2	NM_001017902.1
A18G0668	15.49	16.68	CD9	CD9 antigen	
A4G0719	15.49	7.35	IRF5	Interferon regulatory factor 5	NM_001327817.1
B11G0775	15.49	15.64	GEMIN6	Gem-associated protein 6	
A25G0583	15.48	16.90	MOB2	MOB kinase activator 2	NM_001430951.1
A24G0577	15.48	8.30	TXNDC5	Thioredoxin domain-containing protein 5	NM_213016.2
A5G0693	15.48	13.37	WDR44	WD repeat-containing protein 44	NM_001100038.2
A5G0738	15.47	9.29	ZNHIT1	Zinc finger HIT domain-containing protein 1	NM_001017401.1
A20G0779	15.47	11.50	YLPM1	YLP motif-containing protein 1	XM_003200679.6
A17G0316	15.46	7.72	ITPKA	Inositol-trisphosphate 3-kinase A	NM_001326476.1
A8G0233	15.45	11.24	SAP30BP	SAP30-binding protein	NM_199565.2
B16G1086	15.45	0.81	E1301_TTI014240	A0A5A9N3G5_9TELE Uncharacterized protein	
B14G0569	15.45	2.05	LOXL3A	Lysyl oxidase homolog 3A	XM_005173089.5
A10G0660	15.45	14.92	SLC20A1B	Sodium-dependent phosphate transporter 1-B	XM_005155625.5
B14G0736	15.44	11.99	PRELID1	PRELI domain-containing protein 1, mitochondrial	NM_200366.2
A5G0602	15.44	35.50	TUBB4B	Tubulin beta-4B chain	NM_213490.1
A24G0510	15.43	14.12	FEN1	Flap endonuclease 1	NM_001328507.1
A14G0764	15.43	0.54	GLOD5	Glyoxalase domain-containing protein 5	
A9G0810	15.42	10.02	HAT1	Histone acetyltransferase type B catalytic subunit	NM_001004572.2
B5G0388	15.42	14.50	TIA1	Cytotoxic granule associated RNA binding protein TIA1	NM_212628.1
B9G0145	15.41	6.93	PHGDH	D-3-phosphoglycerate dehydrogenase	NM_199577.2
B7G0778	15.41	12.31	VRK3	threonine-protein kinase VRK3	NM_001013568.3

A11G0419	15.41	9.05	CASS4	Cas scaffolding protein family member 4	NM_001198741.1
B15G0742	15.41	2.75	NLRC3	NLR family CARD domain-containing protein 3	XM_021470829.2
A6G0764	15.41	32.95	IP6K2	Inositol hexakisphosphate kinase 2	NM_201470.1
B19G0642	15.41	45.06	AZIN1	Antizyme inhibitor 1	NM_001007159.2
A1G0941	15.41	11.27	FIP1L1	Pre-mRNA 3'-end-processing factor FIP1	NM_001006042.1
A3G0774	15.41	30.83	GSP-2	threonine-protein phosphatase PP1-beta	NM_214811.2
A3G0600	15.41	28.87	CSNK1D	Casein kinase I isoform delta	NM_199583.1
B21G0272	15.41	6.19	NSRP1	Nuclear speckle splicing regulatory protein 1	NM_001077430.2
A8G0442	15.40	4.34	PTK6	Protein-tyrosine kinase 6	XM_677686.9
B22G0274	15.40	2.29	RNASEL3	Ribonuclease-like 3	
A14G0332	15.40	4.06	TIMM8A	Mitochondrial import inner membrane translocase subunit Tim8 A	NM_001003637.3
A1G0799	15.40	0.13	GTF3C3	General transcription factor 3C polypeptide 3	NM_001256242.1
A23G0600	15.40	5.13	MYB	Transcriptional activator Myb	NM_001309822.1
B17G0765	15.39	17.71	ISCA2	Iron-sulfur cluster assembly 2 homolog, mitochondrial	NM_001327901.1
A12G0834	15.39	8.41	DAGLB	Diacylglycerol lipase-beta	
A7G1353	15.38	21.85	SUPT16H	FACT complex subunit SPT16	NM_001097584.2
B15G0532	15.38	4.38	TLCD2	TLC domain-containing protein 2	NM_001110382.1
B21G0819	15.38	15.29	PDCL	Phosducin-like protein	NM_199647.2
A1G0055	15.37	40.84	NLRC3	Protein NLRC3	XM_068222163.1
B16G0941	15.35	6.62	ARHGEF5	Rho guanine nucleotide exchange factor 5	XM_003200169.6
B2G0767	15.35	12.03	PCCB	Propionyl-CoA carboxylase beta chain, mitochondrial	NM_212925.1
A19G0229	15.34	6.37	MRPL36	39S ribosomal protein L36, mitochondrial	
B8G0112	15.34	13.67	GOLM1	Golgi membrane protein 1	NM_200279.2
B1G0058	15.34	4.11			
B22G0078	15.33	12.24	DENND2D	DENN domain-containing protein 2D	NM_001007346.1
A8G0318	15.33	11.73	DIO1	Type I iodothyronine deiodinase	
B14G0193	15.33	14.49	MBNL3	Muscleblind-like protein 3	XM_009291129.4
A7G0129	15.33	12.42	PSMD9	26S proteasome non-ATPase regulatory subunit 9	
A1G0251	15.32	13.75	SMARCA5	SNF-related matrix-associated actin-dependent regulator of chromatin subfamily A member 5	NM_001081629.2
A15G0043	15.32	15.15	PSMD2	26S proteasome non-ATPase regulatory subunit 2	NM_200546.1
A9G0607	15.31	4.10	ANKZF1	Ankyrin repeat and zinc finger domain-containing protein 1	XM_068223557.1
A20G0380	15.31	8.05	OLFML2B	Olfactomedin-like protein 2B	XM_005160712.5
B7G0728	15.30	18.45	ADAM10	Disintegrin and metalloproteinase domain-containing protein 10	NM_001159314.2
A8G0571	15.30	12.58	PTPN1	Tyrosine-protein phosphatase non-receptor type 1	NM_001313686.1
A3G1052	15.30	5.19	A0A4W5LP95	A0A4W5LP95_9TELE Uncharacterized protein	XM_009299414.4
A16G0783	15.30	7.03	MRPL13	39S ribosomal protein L13, mitochondrial	NM_001020534.1

B15G0540	15.29	6.26	LOC107743657	threonine-protein kinase TAO1-like	
A20G0670	15.29	19.68	TCB1	Transposable element Tcb1 transposase	XM_021471168.2
A19G0432	15.29	21.27	PDCD6IP	Programmed cell death 6-interacting protein	XM_009294414.4
A18G0578	15.29	13.10	URI1	Unconventional prefoldin RPB5 interactor	NM_001030145.2
B21G0963	15.28	0.85	NOP14	Nucleolar protein 14	NM_001003831.1
B4G0071	15.28	9.93	PWP1	Periodic tryptophan protein 1 homolog	NM_200292.1
B21G0481	15.27	15.42	PCF11	Pre-mRNA cleavage complex 2 protein Pcf11	NM_001007307.1
A9G0222	15.27	6.15	NIFK	MKI67 FHA domain-interacting nucleolar phosphoprotein	NM_173288.2
B11G0203	15.26	19.10	CHRNA4	Neuronal acetylcholine receptor subunit alpha-4	NM_199939.1
B5G1168	15.26	20.93	UPRT	Uracil phosphoribosyltransferase homolog	NM_207091.2
A24G0361	15.26	5.09	RRS1	Ribosome biogenesis regulatory protein homolog	NM_200062.1
B9G0854	15.25	11.57	CIR1	Corepressor interacting with RBPJ 1	XM_021478817.2
A19G0682	15.25	6.66	MRPS21	28S ribosomal protein S21, mitochondrial	
A5G0495	15.24	8.65	NIBAN2	Protein Niban 2	NM_001326382.1
A5G0555	15.24	14.27	ATP2A2	endoplasmic reticulum calcium ATPase 2	XM_692016.10
A1G0704	15.24	13.85	PCID2	PCI domain-containing protein 2	XM_005168000.5
A5G0029	15.24	23.24	DDRGK1	DDRGK domain-containing protein 1	XM_021476728.2
A25G0312	15.24	14.11	SETD6	N-lysine methyltransferase setd6	NM_199600.1
B14G0423	15.24	8.80	TMEM33	Transmembrane protein 33	NM_213663.1
B10G0447	15.23	16.10	TMEM126A	Transmembrane protein 126A	NM_001328432.1
B22G0796	15.23	4.89	CDC34	Ubiquitin-conjugating enzyme E2 R1	NM_200958.1
A9G0336	15.23	11.53	PIKFYVE	1-phosphatidylinositol 3-phosphate 5-kinase	NM_001127305.1
A3G0009	15.22	6.35			
B25G0816	15.21	40.31	METAP2	Methionine aminopeptidase 2	
A10G0631	15.21	16.65	PPP3CC	threonine-protein phosphatase 2B catalytic subunit gamma isoform	XM_005155313.5
A21G0206	15.21	5.12	DNAJC21	DnaJ homolog subfamily C member 21	
B9G0635	15.20	12.54	ZC3H15	Zinc finger CCCH domain-containing protein 15	NM_199888.1
A13G0861	15.20	12.81	HSP30	Heat shock protein 30	
A16G0662	15.20	11.41			
A14G0519	15.19	12.25	EIF4EBP3L	Eukaryotic translation initiation factor 4E-binding protein 3-like	
B4G0004	15.19	8.49	PRR5	Proline-rich protein 5	XM_005168503.5
A3G0671	15.19	7.08	RARA	Retinoic acid receptor alpha	XM_068217050.1
B3G1212	15.18	10.07	COPZ1	Coatomer subunit zeta-1	NM_131507.2
B3G1009	15.18	4.27	A0A673NN90	A0A673NN90_9TELE Si:ch211-156b7.4	XR_045227.7
B21G0304	15.18	27.21	MAT2B	Methionine adenosyltransferase 2 subunit beta	XM_005161395.5
A16G0294	15.18	21.50	LOC107756238	A0A673IL05_9TELE CD83 antigen-like	
B5G0587	15.17	19.04	LNPEP	Leucyl-cystinyl aminopeptidase	XM_005165469.5

B10G0836	15.17	6.28	RIOX2	Ribosomal oxygenase 2	
A21G0348	15.17	14.57	FYB1	FYN-binding protein 1	XM_009295371.4
B11G0013	15.16	9.09			NM_214737.2
B11G0248	15.16	13.27	KRT42	Keratin, type I cytoskeletal 42	NM_001386309.1
B20G0414	15.16	9.26	NRBP1	Nuclear receptor-binding protein	NM_001045189.2
A1G0178	15.15	15.46	LUC7L3	Luc7-like protein 3	NM_001089547.2
B14G0412	15.15	14.90	NDUFC1	NADH dehydrogenase [ubiquinone] 1 subunit C1, mitochondrial	
A16G0099	15.15	8.20	UBE2E2	Ubiquitin-conjugating enzyme E2 E2	NM_001003494.1
B5G0078	15.14	7.77	SRRT	Serrate RNA effector molecule homolog	NM_173238.2
A11G0683	15.14	6.02	SRPK1	SRSF protein kinase 1	NM_001006030.1
B3G0948	15.14	22.42	EMC10	ER membrane protein complex subunit 10	NM_001163918.1
A7G0781	15.14	3.35	LOC107700405	A0A671MIE0_9TELE Uncharacterized LOC107700405	
B11G0544	15.14	5.48	TRMT1	tRNA (guanine(26)-N(2))-dimethyltransferase	NM_001118895.1
A5G1117	15.14	11.66	SREK1	lysine-rich protein 1	NM_001003613.2
B5G0842	15.14	10.67	OPHN1	Oligophrenin-1	XM_068218245.1
A5G0043	15.13	4.94			
A16G0881	15.13	16.36	ECM1	Extracellular matrix protein 1	XM_005173500.5
B14G0597	15.13	12.60	GSTP1	Glutathione S-transferase P	NM_131734.3
A6G0353	15.13	8.51	GGA1	ADP-ribosylation factor-binding protein GGA1	NM_001004000.2
B1G0915	15.13	8.31	LOC107598558	A0A672NXS3_SINGR Interleukin	NM_001039565.2
A17G0146	15.12	8.50	MRPL35	39S ribosomal protein L35, mitochondrial	NM_001076725.1
B2G0020	15.12	23.57	EPB41L3	Band 4.1-like protein 3	NM_214812.1
A23G0441	15.12	15.18	SDHB	Succinate dehydrogenase [ubiquinone] iron-sulfur subunit, mitochondrial	NM_001098740.1
B8G0462	15.11	16.26	PTPN1	Tyrosine-protein phosphatase non-receptor type 1	NM_001313686.1
B1G0892	15.11	19.60	ROHU_011231	A0A498LL17_LABRO Ubiquitin-like domain-containing protein	
B5G1198	15.11	8.56	LAS1L	Ribosomal biogenesis protein LAS1L	NM_001126400.1
A22G0379	15.11	22.05	ARHGAP45	Rho GTPase-activating protein 45	XM_068216451.1
B10G0363	15.10	11.16	ILK	Integrin-linked protein kinase	NM_200571.1
B15G0509	15.10	35.66	TAPBPL	Tapasin-related protein	NM_001083830.1
A2G0960	15.10	14.41	ITGB1	Integrin beta-1	NM_001034979.1
B8G1051	15.10	14.87	SNX2	Sorting nexin-2	NM_001037698.1
A18G0139	15.10	5.08	TSEN34	tRNA-splicing endonuclease subunit Sen34	NM_001386748.1
B18G0480	15.09	22.54	A0A671SV47	A0A671SV47_9TELE BH3-interacting domain death agonist	
A22G0716	15.09	8.58	WWTR1	WW domain-containing transcription regulator protein 1	NM_001037696.1
A21G0531	15.09	3.32	TLCD1	TLC domain-containing protein 1	NM_001006071.1
A10G0884	15.09	6.93	PRRC1	Protein PRRC1	NM_001007060.2
B13G0151	15.09	23.58	POL	Retrovirus-related Pol polyprotein from transposon 412	NM_213057.2

				A0A672MUK5_SINGR	
B13G0431	15.08	17.26	LOC107552940	Centrosomal protein of 68 kDa-like	NM_001001841.3
B16G0194	15.08	9.15	TMEM147	Transmembrane protein 147	NM_001002484.1
A6G0549	15.08	28.61	AK4	Adenylate kinase 4, mitochondrial	NM_213299.1
A15G0111	15.08	6.79	FARSB	Phenylalanine--tRNA ligase beta subunit	NM_001007768.1
B2G0280	15.07	5.17	ARHGAP12	Rho GTPase-activating protein 12	XM_005171385.5
A4G0966	15.06	4.88	KRR1	KRR1 small subunit processome component homolog	NM_001045325.2
B22G0023	15.06	12.45	DSTYK	threonine and tyrosine protein kinase	XM_005168270.5
B23G0056	15.06	49.28	ARL6IP5	PRA1 family protein 3	NM_001002517.2
A12G0833	15.05	4.61	KDELR2	ER lumen protein-retaining receptor 2	NM_212695.1
A1G0705	15.05	20.86	CUL4B	Cullin-4B	NM_201027.2
A13G0673	15.05	5.54	TRIM16	Tripartite motif-containing protein 16	XM_684462.10
B24G0209	15.05	16.86	MAPRE2	EB family member 2	XM_009297591.4
B13G0755	15.05	10.90	IPMK	Inositol polyphosphate multikinase	
A10G0007	15.05	15.49	TTC3	E3 ubiquitin-protein ligase TTC3	XM_005172576.5
B2G0041	15.05	24.89	SF3A2	Splicing factor 3A subunit 2	NM_201043.2
A8G0054	15.04	5.13			
A25G0105	15.04	25.62	NANS	Sialic acid synthase	NM_001007420.1
B2G0702	15.04	6.19	SKT	Sickle tail protein	XM_021467462.2
B5G0445	15.04	5.86	PRKRIP1	PRKR-interacting protein 1 homolog	NM_001017896.2
A10G0454	15.04	8.95	ZPR1	Zinc finger protein ZPR1	NM_213108.1
A20G0637	15.04	9.41	EIF2B4	Translation initiation factor eIF-2B subunit delta	NM_001014361.2
B18G0094	15.03	17.73	ARFGAP2	ADP-ribosylation factor GTPase-activating protein 2	NM_001037430.1
A13G0348	15.03	13.37	CXCL12	Stromal cell-derived factor 1	NM_001002536.1
A17G0723	15.02	22.51	SNX6	Sorting nexin-6	NM_001030217.2
A5G0313	15.02	4.18	SH2D1A	SH2 domain-containing protein 1A	NM_001114691.1
A11G0697	15.02	12.13	TMEM38A	Trimeric intracellular cation channel type A	XM_005168061.5
B1G0409	15.01	13.85	TMEM131L	Transmembrane protein 131-like	XM_688724.9
A14G0421	15.01	20.16	FGFBP2	Fibroblast growth factor-binding protein 2	
A15G0550	15.01	7.18	THYN1	Thymocyte nuclear protein 1	NM_200657.2
B3G0553	15.01	12.30	UBFD1	Ubiquitin domain-containing protein UBFD1	XM_005164210.5
B20G0446	15.00	6.21	TMEM251	Transmembrane protein 251	NM_001033746.2
B17G0634	15.00	11.70	FUT8	Alpha-(1,6)-fucosyltransferase	NM_001361235.1
B3G0688	14.99	21.41	SCPEP1	Retinoid-inducible serine carboxypeptidase	NM_200192.1
B1G0557	14.98	2.02	KLF5	Krueppel-like factor 5	XM_001344880.9
B23G0264	14.98	7.20	ACSS2	Acetyl-coenzyme A synthetase, cytoplasmic	NM_001328353.1
A25G0608	14.98	8.72	TSG101	Tumor susceptibility gene 101 protein	
A21G0180	14.97	19.66	NEK6	threonine-protein kinase Nek6	XM_068215992.1
B2G0811	14.96	5.01	GORASP1	Golgi reassembly-stacking protein 1	XM_005171230.5
B17G0194	14.96	6.55	JMJD7	Bifunctional peptidase and (3S)-lysyl hydroxylase Jmjd7	NM_001017615.1

B3G1710	14.96	9.14	HLA-DPA1	HLA class II histocompatibility antigen, DP alpha 1 chain	NM_131695.1
B13G0454	14.96	6.97	PLAU	Urokinase-type plasminogen activator	NM_001007372.1
A4G0675	14.96	21.01	COPG2	Coatomer subunit gamma-2	NM_130994.1
A4G0537	14.95	28.52	HCLS1	Hematopoietic lineage cell-specific protein	NM_001045006.1
B17G0615	14.95	15.89	SMYD2A	N-lysine methyltransferase SMYD2-A	NM_001013550.1
B1G0830	14.95	8.31	AIMP1	Aminoacyl tRNA synthase complex-interacting multifunctional protein 1	NM_001045851.1
A12G0046	14.95	8.86	ASCC1	Activating signal cointegrator 1 complex subunit 1	
A2G1086	14.95	3.64	XCR1	Chemokine XC receptor 1	
A1G0182	14.95	22.46	RAB1	Ras-related protein ORAB-1	NM_001326521.1
A20G0793	14.94	15.44	ARHGAP20	Rho GTPase-activating protein 20	NM_001365334.1
A21G0307	14.94	12.41	ATP2A2	endoplasmic reticulum calcium ATPase 2	XM_005161196.5
B14G0525	14.94	17.98	EIF4EBP3L	Eukaryotic translation initiation factor 4E-binding protein 3-like	NM_199683.1
B6G0359	14.94	8.66	RHEB	GTP-binding protein Rheb	NM_001076748.2
B14G0085	14.94	6.23	DPF2	Zinc finger protein ubi-d4	NM_212696.2
B22G0977	14.94	3.52	A0A673HQC6	A0A673HQC6_9TELE IG domain-containing protein	
B16G0315	14.93	7.94	EBAG9	Receptor-binding cancer antigen expressed on SiSo cells	NM_201094.1
A13G0523	14.93	6.23	PSD	PH and SEC7 domain-containing protein 1	XM_068213070.1
A12G0835	14.92	17.09	SPAG9	C-Jun-amino-terminal kinase-interacting protein 4	XM_068224849.1
B7G0572	14.92	13.20	ARHGAP1	Rho GTPase-activating protein 1	NM_001017781.1
B11G0094	14.92	2.47	CTBS	Di-N-acetylchitobiase	
A7G0842	14.92	22.13	SERPINE1	Plasminogen activator inhibitor 1	XM_068222132.1
B17G0045	14.91	5.76	CEP170B	Centrosomal protein of 170 kDa protein B	XM_021467254.2
A16G0325	14.91	5.97	MRPS15	28S ribosomal protein S15, mitochondrial	NM_001002485.2
A17G0760	14.91	5.37	STX11	Syntaxin-11	NM_212910.1
A19G0244	14.90	8.32	KHDRBS1	KH domain-containing, RNA-binding, signal transduction-associated protein 1	NM_213235.1
A11G0464	14.90	28.21	G5714_011752	A0A7J6CJ91_9TELE Uncharacterized protein	NM_001276281.1
B1G0304	14.89	8.51	TBC1D1	TBC1 domain family member 1	XM_005168369.5
B16G0973	14.88	0.26	ZG16	Zymogen granule membrane protein 16	
B22G0135	14.88	13.38	FTSJ3	pre-rRNA 2'-O-ribose RNA methyltransferase FTSJ3	NM_001305468.1
B9G0896	14.88	13.91	PLBD2	Putative phospholipase B-like 2	
A4G0649	14.88	17.31	STRAP	Serine-threonine kinase receptor-associated protein	NM_200304.1
B4G0496	14.88	12.96	SND1	Staphylococcal nuclease domain-containing protein 1	NM_182865.3
B16G1041	14.87	10.40	MBP	Myelin basic protein	NM_001271459.1
B4G0425	14.87	6.58	GOLT1B	Vesicle transport protein GOT1B	XM_005164773.5
A1G0525	14.87	4.52	IRF2	Interferon regulatory factor 2	NM_001326712.1
B18G0479	14.87	4.42	BCL2L13	Bcl-2-like protein 13	NM_001044891.2
B1G0656	14.86	2.46	DOK1	Docking protein 1	XM_682484.9

A10G0178	14.86	9.57	SSBP2	Single-stranded DNA-binding protein 2	XM_009305684.4
B13G0709	14.85	6.57	FRMD6	FERM domain-containing protein 6	NM_001145619.1
B22G0098	14.85	3.77	LOC107700627	A0A671SSW9_9TELE Gastrula zinc finger protein XlCGF57.1-like	XM_021469384.2
B9G0605	14.85	8.64	ACP6	Lysophosphatidic acid phosphatase type 6	NM_001110380.1
B2G0058	14.85	14.49	ARHGAP45	Rho GTPase-activating protein 45	XM_009298940.4
B3G0982	14.85	24.40	SEPTIN9	Septin-9	XM_009299472.4
B2G0906	14.85	6.99	HCCS	Holocytochrome c-type synthase	NM_001002498.2
A11G0088	14.85	10.91	A0A672SYR9	A0A672SYR9_SINGR Cystatin domain-containing protein	NM_212748.1
A6G0530	14.84	12.87	PHB2	Prohibitin-2	NM_001002681.2
A7G0736	14.84	14.83	ADAM10	Disintegrin and metalloproteinase domain-containing protein 10	NM_001159314.2
A8G0672	14.84	20.67	RASSF2	Ras association domain-containing protein 2	NM_001004676.2
A17G0891	14.83	25.07	THBS1	Thrombospondin-1	XM_002666957.6
A7G0488	14.83	7.71	SI:CH211-238A12.2	Protein C19orf12 homolog	
B9G0837	14.83	35.21	NFE2L2	Nuclear factor erythroid 2-related factor 2	XM_005172511.5
B21G0626	14.83	21.03	GSN	Gelsolin	XM_021468752.2
B14G0183	14.83	11.28	PRSS23	Serine protease 23	NM_001423236.1
A1G0571	14.83	8.91	GPALPP1	GPALPP motifs-containing protein 1	NM_001003473.1
B4G0368	14.83	12.27	PPHLN1	Periphilin-1	XM_021475141.2
B2G0234	14.82	11.60	GPSM2	G-protein-signaling modulator 2	XM_021479206.2
A6G0921	14.81	10.68	FAM212AB	PAK4-inhibitor inka2	NM_199788.1
A2G0963	14.81	18.77	KIF5B	Kinesin-1 heavy chain	XM_005171408.5
B3G0040	14.81	5.53	ERMP1	Endoplasmic reticulum metallopeptidase 1	XM_005173860.5
B13G0410	14.81	10.77	PCNX2	Pecanex-like protein 2	XM_021480668.2
B2G0168	14.81	13.54	BLVRA	Biliverdin reductase A	NM_001076601.1
B23G0128	14.80	1.07	CDH26	Cadherin-like protein 26	NM_001423486.1
A16G0492	14.80	6.88	S100A16	Protein S100-A16	NM_001366340.1
B5G0657	14.80	3.71	KANK1	KN motif and ankyrin repeat domain-containing protein 1	NM_001365257.1
A3G0333	14.79	12.30	DNM2	Dynamin-2	XM_021470874.2
B11G0270	14.79	14.87	PTP4A1	Protein tyrosine phosphatase type IVA 1	XM_687846.10
A1G1078	14.79	16.06	PDS5A	Sister chromatid cohesion protein PDS5 homolog A	XM_068214606.1
A22G0054	14.78	11.71	TMEM263	Transmembrane protein 263	XM_005168276.5
A1G0722	14.78	13.87	BLZF1	Golgin-45	NM_001144803.1
A17G0177	14.77	37.00	ALDH6A1	Methylmalonate-semialdehyde dehydrogenase [acylating], mitochondrial	NM_001002374.2
B17G0826	14.77	9.56	FCF1	rRNA-processing protein FCF1 homolog	NM_001017601.1
B16G0898	14.76	1.32			
A24G0214	14.75	5.03	EIF1B	Eukaryotic translation initiation factor 1b	NM_200303.2
A18G0742	14.74	11.98	RAB27A	Ras-related protein Rab-27A	NM_001030257.1
A7G0567	14.74	35.45	CCL3	C-C motif chemokine 3	
A6G0735	14.74	12.31	RAB13	Ras-related protein Rab-13	XM_068222028.1
B8G0509	14.74	20.52	IMPDH2	Inosine-5'-monophosphate dehydrogenase 2	NM_201464.1

B2G0315	14.73	21.70	TMEM71	Transmembrane protein 71	NM_001130853.3
A6G0087	14.73	8.79	PPIG	Peptidyl-prolyl cis-trans isomerase G	NM_213464.1
B23G0369	14.73	10.75	LOC107671981	A0A671Q3C8_9TELE Emerin-like	
B24G0582	14.73	10.50	PDCD6	Programmed cell death protein 6	NM_200950.1
B2G1045	14.72	6.73	EGFR	Epidermal growth factor receptor	NM_194424.1
A11G0141	14.72	7.63	PDE4D	cAMP-specific 3',5'-cyclic phosphodiesterase 4D	NM_197934.1
A2G0602	14.72	17.72	DNAJB6	DnaJ homolog subfamily B member 6	NM_001002353.1
A20G0239	14.72	24.78	GJA1	Gap junction alpha-1 protein	NM_131038.1
B7G0871	14.72	11.67	NAA40	N-alpha-acetyltransferase 40	NM_001017632.1
A6G0020	14.72	26.34	HMOX	Heme oxygenase	NM_205671.1
B21G0873	14.70	1.41	ZBTB7C	Zinc finger and BTB domain-containing protein 7C	XM_003200810.6
A15G0091	14.70	9.78	CTSC	Dipeptidyl peptidase 1	NM_214722.1
A12G0824	14.70	11.80	COPS3	COP9 signalosome complex subunit 3	NM_199685.1
A16G0260	14.70	22.45	DEK	Protein DEK	NM_001045276.2
B8G0021	14.69	11.73	MBD4	Methyl-CpG-binding domain protein 4	
A23G0905	14.69	6.96	NFXL1	NF-X1-type zinc finger protein NFXL1	XM_068216961.1
B25G0338	14.69	11.00	DAPK3	Death-associated protein kinase 3	XM_679634.9
A14G0341	14.69	5.40	TNMD	Tenomodulin	NM_001114413.1
B19G0088	14.69	15.28	RNF5	E3 ubiquitin-protein ligase RNF5	XM_003200504.6
B25G0327	14.69	26.64	KLF13	Krueppel-like factor 13	NM_001076772.1
B5G0900	14.68	4.81	IER5L	Immediate early response gene 5-like protein	NM_199754.1
B3G0569	14.68	7.68	PSMG3	Proteasome assembly chaperone 3	
B14G0350	14.68	14.89	TAF7	Transcription initiation factor TFIID subunit 7	NM_173260.1
B7G1092	14.68	12.76	IL13RA1	Interleukin-13 receptor subunit alpha-1	NM_001110837.1
B8G0549	14.66	2.02	NGRN	Neugrin	NM_001045253.1
B1G0783	14.65	11.49	KPNA3	Importin subunit alpha-4	NM_201320.2
B6G0576	14.65	9.47	AKR1A1B	Aldo-keto reductase family 1 member A1-B	NM_001017640.1
A23G0626	14.65	50.56	TCB1	Transposable element Tcb1 transposase	
A16G0567	14.64	18.20	DDX6	Probable ATP-dependent RNA helicase ddx6	XM_001340824.8
B8G0670	14.64	8.36	ZAP70	Tyrosine-protein kinase ZAP-70	NM_001020589.1
B1G1002	14.64	14.74	TECR	Very-long-chain enoyl-CoA reductase	XM_021479665.2
A3G0598	14.63	18.51	CSN1	COP9 signalosome complex subunit 1	XM_021471590.2
A25G0672	14.63	14.90	IGLC7	Immunoglobulin lambda constant 7	XM_068217596.1
A16G0773	14.62	8.45	DAP3	28S ribosomal protein S29, mitochondrial	NM_001098737.1
B8G1000	14.62	0.31	DLC-1	Dynein light chain 1, cytoplasmic	
B4G0357	14.62	7.84			NM_201087.1
B3G0974	14.61	36.63	LEG	Beta-galactoside-binding lectin	
B4G0536	14.61	7.79	PAWR	PRKC apoptosis WT1 regulator protein	NM_001006015.2

A6G0392	14.61	16.02	MCRIP1	Mapk-regulated corepressor-interacting protein 1	
A5G1120	14.61	28.64	PIK3R1	Phosphatidylinositol 3-kinase regulatory subunit alpha	NM_001281844.1
A3G0015	14.60	6.70	FMC1	Protein FMC1 homolog	
A13G0727	14.59	8.13	MACROH2A2	Core histone macro-H2A.2	NM_001145594.1
B12G0585	14.59	25.92	IRF8	Interferon regulatory factor 8	NM_205710.2
A7G1394	14.58	15.17	RELA	Transcription factor p65	XM_068222214.1
A6G0749	14.58	3.92	HEMK1	MTRF1L release factor glutamine methyltransferase	NM_001114419.1
A13G0676	14.57	8.85	TLR4	Toll-like receptor 4	NM_001002084.1
A20G0126	14.57	13.05	HADHB	Trifunctional enzyme subunit beta, mitochondrial	NM_200019.1
B20G0515	14.57	8.07	RNASEH1	Ribonuclease H1	NM_001285828.1
B8G0869	14.57	9.85	MAPK13	Mitogen-activated protein kinase 13	XM_001337797.9
B8G0431	14.56	5.89	ZNG1	Zinc-regulated GTPase metalloprotein activator 1	NM_213253.1
B22G0880	14.56	14.19			
B7G0794	14.56	9.80	DENND2B	DENN domain-containing protein 2B	NM_001002327.1
A20G0559	14.55	14.91	MAP3K7	Mitogen-activated protein kinase kinase kinase 7	XM_068215443.1
B3G0543	14.55	12.60	PDPK1	3-phosphoinositide-dependent protein kinase 1	XM_068217664.1
A6G1018	14.55	3.65	NUFIP1	FMR1-interacting protein NUFIP1	
B4G0508	14.55	9.37	LAMTOR4	Ragulator complex protein LAMTOR4	NM_001044950.1
B17G0433	14.55	14.23	ZMPSTE24	CAAX prenyl protease 1 homolog	NM_199892.1
A2G1027	14.55	8.57	ACSL3	Fatty acid CoA ligase Acsl3	NM_001145932.1
B7G0819	14.55	17.78	PHF23B	PHD finger protein 23B	NM_199844.1
B7G0660	14.55	4.85	TIPIN	TIMELESS-interacting protein	NM_001003452.2
B19G0937	14.55	6.35	NT5C3	Cytosolic 5'-nucleotidase 3	
A10G0889	14.54	9.56	ISOC1	Isochorismatase domain-containing protein 1	NM_001079981.1
A6G0548	14.54	14.68	JAK1	Tyrosine-protein kinase JAK1	NM_131073.1
B13G0385	14.54	8.13	GLUD1	Glutamate dehydrogenase 1, mitochondrial	XM_682373.10
B6G0845	14.54	12.47	SUN2	SUN domain-containing protein 2	XM_001919656.7
A3G0435	14.53	13.32	HIS3.3A	Histone H3.3A	NM_212996.1
B24G0707	14.52	5.18	LYRM4A	LYR motif-containing protein 4A	NM_001386617.1
B25G0398	14.52	5.16	HSD17B12A	Very-long-chain 3-oxoacyl-CoA reductase-A	NM_200881.1
A25G0711	14.52	6.37	DUS2	tRNA-dihydrouridine(20) synthase [NAD(P)+]-like	XM_005163191.5
A10G0608	14.52	34.24	FBXW11B	F-box and WD repeat domain-containing 11-B	NM_213504.2
B16G0389	14.51	14.97	TCEA3	Transcription elongation factor A protein 3	XM_068213923.1
A16G0134	14.51	11.44	UBQLN4	Ubiquilin-4	NM_213356.2
A8G0882	14.51	9.62	GKAP1	G kinase-anchoring protein 1	NM_213072.2
B8G0535	14.51	12.57	LOC113107597	A0A6P6PX35_CARAU uncharacterized protein LOC113107597 isoform X1	
B5G0658	14.50	15.41	FBP1	Fructose-1,6-bisphosphatase 1	NM_199942.2
B14G0811	14.50	11.90	HDAC3	Histone deacetylase 3	NM_200990.1
B7G0635	14.50	22.64	ATP6V0D1	V-type proton ATPase subunit d 1	NM_199620.1

B1G0045	14.50	2.91	CYYR1	Cysteine and tyrosine-rich protein 1	
A2G0231	14.49	8.18	GIPC1	PDZ domain-containing protein GIPC1	NM_200930.1
A10G0094	14.48	5.96	DDX55	ATP-dependent RNA helicase DDX55	NM_173229.2
A20G0148	14.48	14.85	EIF2B2	Translation initiation factor eIF-2B subunit beta	NM_212903.1
A5G0539	14.48	8.81	A0A673JF07	A0A673JF07_9TELE DUF4430 domain-containing protein	NM_001252649.1
B8G0744	14.48	11.07	SKI	Ski oncogene	NM_130935.3
A24G0176	14.48	17.60	MAPRE2	EB family member 2	XM_009297591.4
B17G0906	14.47	23.31	DLST	Dihydrolipoyllysine-residue succinyltransferase component of 2-oxoglutarate dehydrogenase complex, mitochondrial	NM_201487.1
B6G0314	14.47	14.93	MRPS9	28S ribosomal protein S9, mitochondrial	NM_001045396.1
A7G0111	14.46	12.27	SLC29A2	Equilibrative nucleoside transporter 2	XM_068222803.1
A23G0216	14.45	12.62	EIF4B	Eukaryotic translation initiation factor 4B	NM_001099237.2
A9G0800	14.44	17.60	CDCA7	Cell division cycle-associated protein 7	
B5G1279	14.44	13.57	PGAM5	threonine-protein phosphatase PGAM5, mitochondrial	NM_001007323.2
B7G0607	14.44	9.86	BRD7	Bromodomain-containing protein 7	NM_213366.2
A9G0568	14.43	6.64	PARP4	Protein mono-ADP-ribosyltransferase PARP4	XM_017357877.2
B9G0074	14.43	23.96	TRAPPC2	Trafficking protein particle complex subunit 2	NM_001320115.1
A17G0635	14.43	6.91	STYX-A	tyrosine-interacting protein A	NM_001024390.1
B3G0726	14.42	7.02	CLPP	ATP-dependent Clp protease proteolytic subunit, mitochondrial	NM_001020684.1
A23G0301	14.42	16.96	SSR4	Translocon-associated protein subunit delta	NM_001002082.1
A14G0459	14.42	14.97	DCTN4	Dynactin subunit 4	NM_001017782.2
A25G0731	14.41	12.37	CLPX	ATP-dependent Clp protease ATP-binding subunit clpX-like, mitochondrial	XM_002666958.7
A11G0041	14.41	8.54	MAP1LC3C	1B light chain 3C	NM_001017757.2
A1G0645	14.41	4.31	ANKRD10	Ankyrin repeat domain-containing protein 10	NM_200980.1
A19G0302	14.41	17.82	STK3	threonine-protein kinase 3	NM_199672.1
A11G0008	14.41	18.19			NM_001114412.1
A19G0947	14.41	14.45	MYCBP	c-Myc-binding protein	
B12G0722	14.40	3.49	TRPM4	Transient receptor potential cation channel subfamily M member 4	NM_001282153.1
B19G0316	14.40	0.53	G5714_018817	A0A7J6C0N1_9TELE Uncharacterized protein	
B22G0009	14.40	8.64	ATAD3	ATPase family AAA domain-containing protein 3	NM_205703.1
B5G0234	14.40	19.86	YTHDC1	YTH domain-containing protein 1	XM_021476130.2
B3G1188	14.39	4.58	SP110	Sp110 nuclear body protein	NM_001017912.2
B6G1032	14.39	11.62	CCNDBP1	Cyclin-D1-binding protein 1 homolog	
B9G0179	14.38	13.52	NCKAP1	Nck-associated protein 1	XM_005167921.5

A19G0410	14.38	4.14	LOC107731497	A0A673JQ34_9TELE Uncharacterized LOC107731497	
B1G0385	14.37	8.75	UBE2K	Ubiquitin-conjugating enzyme E2 K	NM_001008611.2
B9G0006	14.37	14.95	UBXN4	UBX domain-containing protein 4	NM_213041.3
A16G0128	14.37	3.12	CSNK2A2	Casein kinase II subunit alpha'	XM_002665035.7
A19G0042	14.36	13.86	CRAT	Carnitine O-acetyltransferase	NM_001004594.2
B25G0837	14.36	10.32	CDKN1B	Cyclin-dependent kinase inhibitor 1B	NM_212792.2
A1G0298	14.36	3.75	SRD5A2	3-oxo-5-alpha-steroid 4-dehydrogenase 2	NM_001017703.1
A13G0130	14.36	25.12	MARCHF5	E3 ubiquitin-protein ligase MARCHF5	XR_002459806.2
A16G0869	14.36	5.09	ARHGEF5	Rho guanine nucleotide exchange factor 5	XM_005161483.5
A6G0908	14.36	19.52	SDC4	Syndecan-4	NM_001048149.2
A6G1014	14.35	25.71	PRELID3B	PRELI domain containing protein 3B	NM_199734.1
B8G0893	14.34	9.89	UXT	Protein UXT	NM_001004671.1
B21G0113	14.34	6.25	YIPF5	Protein YIPF5	NM_200295.1
B16G0127	14.33	13.76	GPSM3	G-protein-signaling modulator 3	XM_017351561.3
A21G0600	14.33	14.36	HNRNPH2	Heterogeneous nuclear ribonucleoprotein H2	NM_212589.2
B22G0088	14.32	0.81	LOC113067423	A0A6P6MGK2_CARAU zinc finger protein 585A-like isoform X2	XM_017356204.3
A12G0246	14.32	1.58	CPN1	Carboxypeptidase N catalytic chain	NM_212770.1
B14G0204	14.32	19.19	MMGT1	ER membrane protein complex subunit 5	NM_205599.1
A12G0108	14.32	8.86	PTPRE	Receptor-type tyrosine-protein phosphatase epsilon	XM_003199651.6
A1G0707	14.32	5.79	GRTP1A	Growth hormone-regulated TBC protein 1-A	NM_213402.1
A11G0571	14.32	11.30	SSTR2	Somatostatin receptor type 2	NM_214705.1
A8G0633	14.31	10.93	C7	Complement component C7	XM_009304071.4
B19G0753	14.31	8.39	PDIA3	Protein disulfide-isomerase A3	NM_001003517.2
B19G0493	14.31	22.03	YRK	Proto-oncogene tyrosine-protein kinase Yrk	NM_001365553.1
A12G0459	14.31	9.35	PDAP1	28 kDa heat- and acid-stable phosphoprotein	NM_212632.2
B19G0328	14.31	4.34	IGKC	Immunoglobulin kappa constant	
B5G0962	14.30	4.51	H2AX	Histone H2AX	NM_001386371.1
B14G0515	14.29	19.13	NIPSNAP3A	Protein NipSnap homolog 3A	NM_200264.3
B10G0201	14.29	27.87	MAT2A	S-adenosylmethionine synthase isoform type-2	NM_001290080.1
B15G0451	14.29	13.38	PPP2R1B	threonine-protein phosphatase 2A 65 kDa regulatory subunit A beta isoform	NM_001005590.1
B16G0255	14.29	15.95	FLI1	Retroviral integration site protein Fli-1 homolog	XM_005158200.4
B2G0982	14.28	11.23	MRPL37	39S ribosomal protein L37, mitochondrial	NM_001082855.2
A7G1127	14.28	7.12	MRPS11	28S ribosomal protein S11, mitochondrial	NM_001044797.1
B13G0923	14.27	0.78			
A7G0810	14.27	16.26	COPB1	Coatomer subunit beta	NM_001002013.1
B19G0849	14.27	4.83	RNMT	mRNA cap guanine-N7 methyltransferase	NM_001045000.1

B16G0865	14.26	5.19	CCDC12	Coiled-coil domain-containing protein 12	NM_001003589.2
B2G0387	14.26	18.47	GIMAP8	GTPase IMAP family member 8	
A5G1135	14.26	9.48	TMEM141	Transmembrane protein 141	NM_001076598.1
B14G0214	14.26	9.35	TM9SF2	Transmembrane 9 superfamily member 2	NM_001423226.1
B20G0838	14.25	21.64	HADHB	Trifunctional enzyme subunit beta, mitochondrial	NM_200019.1
A23G0483	14.25	10.23	ATP6AP1	V-type proton ATPase subunit S1	NM_173265.2
B24G0172	14.25	4.35	IL9R	Interleukin-9 receptor	
A23G0549	14.24	41.43	TARDBP	TAR DNA-binding protein 43	XM_021469761.2
B7G0296	14.24	9.55	MRPL15	39S ribosomal protein L15, mitochondrial	NM_001003435.1
B13G0331	14.24	8.72	ABCG8	ATP-binding cassette sub-family G member 8	NM_001030276.2
A1G0023	14.24	13.73	LPAR1-A	Lysophosphatidic acid receptor 1-A	NM_001320133.1
A2G1146	14.23	14.35	RAB8A	Ras-related protein Rab-8A	NM_001089562.2
A12G0252	14.23	3.82	ZDHHC6	Palmitoyltransferase ZDHHC6	NM_001204157.1
B10G0049	14.23	4.00	ST8SIA4	CMP-N-acetylneuraminate-poly-alpha-2,8-sialyltransferase	
A19G0185	14.23	4.19	ASNS	Asparagine synthetase [glutamine-hydrolyzing]	NM_201163.3
A25G0629	14.23	10.33	IGLC7	Immunoglobulin lambda constant 7	XM_068217596.1
A2G0369	14.22	12.85	EIF4G1	Eukaryotic translation initiation factor 4 gamma 1	NM_001080200.1
A10G0032	14.22	27.75	TC1A	Transposable element Tc1 transposase	
A2G0710	14.22	9.00	MRPS14	28S ribosomal protein S14, mitochondrial	NM_001045358.1
A7G0158	14.21	12.51	DHX15	ATP-dependent RNA helicase DHX15	NM_001115141.1
A16G0276	14.21	5.53	HINT3	Adenosine 5'-monophosphoramidase HINT3	NM_001326468.1
B8G0960	14.21	9.53	SEPTIN2	Septin-2	XM_005172457.5
B13G0018	14.21	6.74	LOC113093330	A0A6P6P1N7_CARAU gastrula zinc finger protein XlCGF26.1-like isoform X1	XM_021480917.2
A14G0504	14.21	9.71	SMU1	WD40 repeat-containing protein SMU1	NM_200199.1
B25G0641	14.20	16.14	CA5B	Carbonic anhydrase 5B, mitochondrial	XM_005163076.5
A8G0618	14.20	14.84	SNRNP200	U5 small nuclear ribonucleoprotein 200 kDa helicase	NM_001123257.1
A21G0014	14.19	8.32	PLGRKT	Plasminogen receptor (KT)	NM_001033098.2
B15G0241	14.19	23.06	CALM1	Calmodulin-1	NM_182967.1
A3G0642	14.19	9.80	YIPF1	Protein YIPF1	NM_001003500.1
B1G0329	14.18	5.04	TMA16	Translation machinery-associated protein 16	
B19G0765	14.18	10.04	ASNS	Asparagine synthetase [glutamine-hydrolyzing]	NM_201163.3
B15G0099	14.17	8.10			
B21G0241	14.17	12.26	MED31	Mediator of RNA polymerase II transcription subunit 31	NM_001002417.2
A5G0368	14.17	11.28	KAT5	Histone acetyltransferase KAT5	XM_009301297.4
A14G0098	14.17	37.51	CXCL14	C-X-C motif chemokine 14	NM_131627.1
A6G0346	14.16	12.84	C24G6.8	Probable peptidyl-tRNA hydrolase 2	NM_001326569.1

A6G0945	14.15	11.44	SNRPC	U1 small nuclear ribonucleoprotein C	NM_173251.2
A19G0827	14.15	16.74	RXRBA	Retinoic acid receptor RXR-beta-A	XM_009294022.3
A19G0818	14.15	31.75	PSMB8	Proteasome subunit beta type-8	NM_131392.3
B13G0203	14.15	16.85	TNFAIP2	Tumor necrosis factor alpha-induced protein 2	NM_200218.1
B10G0158	14.14	5.20	PAQR3	Progestin and adipoQ receptor family member 3	XR_011016939.1
A21G0709	14.14	9.23	UIMC1	BRCA1-A complex subunit RAP80	XM_003200891.6
A2G0195	14.14	16.76	DNTS_033861	A0A553Q641_9TELE Uncharacterized protein	
A11G0134	14.14	8.44	NDUFA11	NADH dehydrogenase [ubiquinone] 1 alpha subcomplex subunit 11	NM_173258.1
A7G1216	14.14	5.03	ACTR2	Actin-related protein 2	NM_001007772.1
A10G0783	14.14	32.49	ZFAND5	AN1-type zinc finger protein 5	NM_200949.1
A18G0105	14.13	16.08	NAPA	Alpha-soluble NSF attachment protein	NM_199766.1
A16G0731	14.13	16.62	SLC2A3	Solute carrier family 2, facilitated glucose transporter member 3	XM_002667123.6
B10G0380	14.13	3.76	TIAM1	Rho guanine nucleotide exchange factor TIAM1	XM_001924009.8
B12G0535	14.13	4.45	SLC16A12B	Monocarboxylate transporter 12-B	NM_001145814.1
A8G0936	14.13	7.19	SULT1ST3	Cytosolic sulfotransferase 3	NM_183348.2
B9G0812	14.13	7.84	ZGC:101851	UPF0538 protein C2orf76 homolog	
A19G0086	14.13	9.62	PHACTR4A	Phosphatase and actin regulator 4A	NM_001423797.1
A5G0318	14.12	9.00	ASB12	Ankyrin repeat and SOCS box protein 12	NM_001126376.1
B7G1110	14.12	13.32	ZNF638	Zinc finger protein 638	XM_021477756.2
B20G0483	14.12	62.86	SRSF5	arginine-rich splicing factor 5	NM_001002610.1
B5G0526	14.12	5.95	RXRAB	Retinoic acid receptor RXR-alpha-B	XM_009301955.4
A2G0091	14.12	14.15	GUK1	Guanylate kinase	NM_001316896.1
A24G0606	14.12	6.30	PDSS1	All trans-polyprenyl-diphosphate synthase PDSS1	XM_005162505.4
A20G0415	14.12	10.43	PDIA6	Protein disulfide-isomerase A6	NM_197933.2
B5G0251	14.12	11.05	PSAT1	Phosphoserine aminotransferase	NM_199819.2
A14G0026	14.11	17.85	LEPROTL1	Leptin receptor overlapping transcript-like 1	NM_001002390.2
A17G0864	14.11	9.13	SRP14	Signal recognition particle 14 kDa protein	NM_001033741.1
A11G0257	14.11	4.11	TWF2	Twinfilin-2	XM_005155759.5
B17G0844	14.11	3.93	MOCS3	Adenylyltransferase and sulfurtransferase MOCS3	XM_068214335.1
A16G1024	14.10	11.85	BCKDHB	2-oxoisovalerate dehydrogenase subunit beta, mitochondrial	NM_001080653.1
B7G0073	14.10	11.94	A0A673LZZ1	A0A673LZZ1_9TELE SGNH_hydro domain-containing protein	XM_068219418.1
B10G0642	14.10	2.93	REXO2	Oligoribonuclease, mitochondrial	NM_200744.2
A9G0529	14.10	7.43	GATD3	Glutamine amidotransferase-like class 1 domain-containing protein 3, mitochondrial	NM_001126455.1
B19G0508	14.10	19.89	PDCD6IP	Programmed cell death 6-interacting protein	XM_009294414.4

B15G0695	14.09	20.56	TMEM106B	Transmembrane protein 106B	
A25G0058	14.09	8.65	MORC2A	ATPase MORC2A	NM_001003994.1
B25G0570	14.09	18.90	SPON1	Spondin-1	NM_131517.1
A23G0435	14.09	4.29	EPHA2	Ephrin type-A receptor 2	XM_005162209.5
A25G0414	14.09	24.13	GNAI1	Guanine nucleotide-binding protein G(i) subunit alpha-1	NM_198805.2
A13G0472	14.09	7.38	ZNF511	Zinc finger protein 511	NM_001077379.1
B6G0099	14.09	11.60	GORASP2	Golgi reassembly-stacking protein 2	NM_200703.2
B3G0847	14.08	16.76	NOSIP	Nitric oxide synthase-interacting protein	NM_001007434.1
B6G0395	14.08	4.99	MCRIP1	Mapk-regulated corepressor-interacting protein 1	
B23G0437	14.08	2.29	CA6	Carbonic anhydrase 6	XM_021473673.2
B2G0782	14.08	15.16	ROHU_000628	A0A498P4L0_LABRO Uncharacterized protein	
A21G0662	14.08	1.79	G5714_020420	A0A7J6BTR3_9TELE Uncharacterized protein	XM_017352953.3
A6G0438	14.08	10.36	DR1	Protein Dr1	NM_001077314.2
B9G0400	14.07	18.85	KLHL13	Kelch-like protein 13	XM_001923378.8
B6G0846	14.07	27.22	SLC2A1	Solute carrier family 2, facilitated glucose transporter member 1	XM_002662528.6
A3G1106	14.06	11.60	ARHGAP17	Rho GTPase-activating protein 17	XM_009299322.4
B10G0065	14.06	11.58	ENOPH1	Enolase-phosphatase E1	NM_001002226.1
A7G1071	14.06	14.21	MTA2	Metastasis-associated protein MTA2	NM_214695.1
B8G0063	14.06	9.70	WDR46	WD repeat-containing protein 46	
A3G0380	14.05	19.95	STXBP2	Syntaxin-binding protein 2	NM_213289.1
B10G0012	14.05	6.05	PRRC1	Protein PRRC1	NM_001007060.2
B21G0022	14.05	12.78	LARP1	La-related protein 1	XM_001920867.9
B5G0420	14.05	12.97	RTASE	Probable RNA-directed DNA polymerase from transposon BS	XM_068214189.1
A6G0867	14.05	15.81	CTSZ	Cathepsin Z	NM_001006043.1
A2G0902	14.05	0.76	CCL20	C-C motif chemokine 20	
A8G0903	14.05	14.86	RER1	Protein RER1	XM_068222900.1
A17G0438	14.05	4.52	KBTBD11	Kelch repeat and BTB domain-containing protein 11	XM_068214591.1
A14G0546	14.05	5.56	EIF1AD	Probable RNA-binding protein EIF1AD	NM_200468.1
B5G0364	14.05	16.20	SREK1	lysine-rich protein 1	NM_001003613.2
A3G0388	14.04	7.76	NOTCH3	Neurogenic locus notch homolog protein 3	NM_131549.2
B18G0809	14.04	5.14	DCUN1D5	DCN1-like protein 5	
B23G0071	14.03	10.92	NUDT3	Diphosphoinositol polyphosphate phosphohydrolase 1	NM_201145.2
B3G0023	14.02	18.48	TRIOBP	TRIO and F-actin-binding protein	NM_001327809.1
A9G0332	14.02	32.54	COQ10B	Coenzyme Q-binding protein COQ10 homolog B, mitochondrial	NM_001017747.1
A7G0273	14.02	13.04	ENOSF1	Mitochondrial enolase superfamily member 1	NM_001076742.2
B19G0705	14.01	10.17	KHDRBS1	KH domain-containing, RNA-binding, signal transduction-associated protein 1	NM_213235.1
B16G0342	14.01	10.72	CDC42SE1	CDC42 small effector protein 1	NM_200060.3
B20G0703	14.01	8.41	HDDC2	5'-deoxynucleotidase HDDC2	NM_001045231.1

A10G0653	14.01	4.27	PPP2R2A	threonine-protein phosphatase 2A 55 kDa regulatory subunit B alpha isoform	NM_001309438.1
B14G0098	14.01	13.45	MATR3	Matrin-3	XM_068213213.1
A3G0794	14.01	10.89	BCAT1	Branched-chain-amino-acid aminotransferase, cytosolic	NM_001309527.1
A15G0010	14.01	12.02	PAF1	RNA polymerase II-associated factor 1 homolog	NM_001024453.1
B6G0276	14.01	9.70	MCM6	DNA replication licensing factor MCM6	NM_001082849.1
B25G0739	14.00	6.38	WDR61	WD repeat-containing protein 61	NM_200853.1
A9G0476	14.00	1.54	ESYT3	Extended synaptotagmin-3	NM_001123233.1
B8G0182	14.00	9.31	GINS4	DNA replication complex GINS protein SLD5	NM_001003546.2
A5G0217	14.00	3.79	TBX1	T-box transcription factor TBX1	XM_005168611.5
B5G0721	14.00	11.33	GOLPH3	Golgi phosphoprotein 3	XM_005165301.5
A8G0515	14.00	10.11	TAF10	Transcription initiation factor TFIID subunit 10	
B8G0280	13.99	3.51	RENBP	N-acylglucosamine 2-epimerase	NM_001126443.1
A21G0468	13.99	14.29	RCSD1	CapZ-interacting protein	
B6G0671	13.99	7.48	LARP4	La-related protein 4	XM_009302582.4
B18G0433	13.99	9.03	CBFB	Core-binding factor subunit beta	XM_009293530.4
B17G0353	13.99	18.42	PYGB	Glycogen phosphorylase, brain form	NM_212809.2
B19G0006	13.99	10.61	DUS3L	tRNA-dihydrouridine(47) synthase [NAD(P)(+)]-like	NM_200674.2
A14G0753	13.98	16.23	THOC2	THO complex subunit 2	XM_005157134.5
B6G0405	13.98	8.76	RAB11FIP4B	Rab11 family-interacting protein 4B	NM_001128825.1
B5G0262	13.98	16.60	PSAP	Prosaposin	NM_001171066.1
B19G0541	13.98	4.95	CHRAC1	Chromatin accessibility complex protein 1	NM_001013293.2
A6G0019	13.98	16.35	HMGXB4	HMG box-containing protein 4	
B25G0025	13.98	18.14	WWP2	NEDD4-like E3 ubiquitin-protein ligase WWP2	NM_001099448.1
B20G0875	13.98	24.40	ATP6V1D	V-type proton ATPase subunit D	NM_001167954.1
B1G0891	13.97	6.13	G5714_000396	A0A7J6DH91_9TELE Ubiquitin-like domain-containing protein	
B13G0637	13.96	3.38	SNX5	Sorting nexin-5	
B10G0259	13.96	12.71	PPP3CC	threonine-protein phosphatase 2B catalytic subunit gamma isoform	XM_005155313.5
A4G0851	13.96	14.90	NUDT5	ADP-sugar pyrophosphatase	NM_001002086.1
A18G0031	13.96	11.01	YIF1B	Protein YIF1B	NM_001007334.1
A25G0696	13.96	9.83	AFG3L1	AFG3-like protein 1	XM_005163207.5
A7G0772	13.96	8.12	APH1B	Gamma-secretase subunit Aph-1b	NM_200115.1
A1G0279	13.95	6.65	SNRPB2	U2 small nuclear ribonucleoprotein B"	NM_001256190.1
B1G0564	13.94	4.52	BTLA	B- and T-lymphocyte attenuator	XM_068220914.1
A14G0367	13.94	5.44	EIF4E1A	Eukaryotic translation initiation factor 4E-1A	NM_131733.1
A11G0416	13.94	9.36	BARX1	Homeobox protein BarH-like 1	NM_001002741.2
A1G0368	13.93	10.95	KPNA3	Importin subunit alpha-4	NM_201320.2
A24G0460	13.93	9.41	BMI1A	Polycomb complex protein BMI-1-A	NM_194366.1
B9G0041	13.92	6.65	LOC113051471	A0A6P6KFC7_CARAU uncharacterized protein LOC113051471	

A5G0060	13.92	11.85	SUPT4H1	Transcription elongation factor SPT4	NM_001002509.1
B1G0737	13.92	5.62	DTX4	E3 ubiquitin-protein ligase DTX4	XM_002660524.6
B3G1384	13.92	22.02	LUC7L3	Luc7-like protein 3	NM_001080591.1
B19G0332	13.92	5.15	K-BAS	Ig kappa-b4 chain C region	
A18G0042	13.92	5.48	GEMIN7	Gem-associated protein 7	
B8G0996	13.92	3.94	MRPL40	39S ribosomal protein L40, mitochondrial	
A4G0906	13.92	8.57	CCDC167	Coiled-coil domain-containing protein 167	
B2G0425	13.91	8.57	PRKCI	Protein kinase C iota type	NM_131855.2
A2G1147	13.91	17.96	TPM4	Tropomyosin alpha-4 chain	NM_199675.2
B1G1089	13.91	57.69	NLRP3	NACHT, LRR and PYD domains-containing protein 3	XM_068220664.1
A19G0707	13.91	15.80	HAX1	HCLS1-associated protein X-1	NM_001002337.1
A21G0288	13.91	14.18	IDH3B	Isocitrate dehydrogenase [NAD] subunit beta, mitochondrial	XM_017352767.3
A23G0498	13.90	25.91	PTGES3	Prostaglandin E synthase 3	NM_213170.1
A5G0134	13.90	9.37	KCNV2	Potassium voltage-gated channel subfamily V member 2	
B7G0755	13.90	10.15	RORAB	Nuclear receptor ROR-alpha B	XM_009303216.4
B23G0689	13.90	24.48	CHCHD6A	MICOS complex subunit mic25a	XM_005162311.4
B20G0176	13.89	45.68	ZFP36L1	mRNA decay activator protein ZFP36L1	NM_199649.2
A20G0194	13.89	42.33	LAPTM4A	Lysosomal-associated transmembrane protein 4A	NM_214685.2
A23G0521	13.89	4.54	ACVR1B	Activin receptor type-1B	XM_005162224.5
A8G0772	13.88	4.37	PDLIM2	PDZ and LIM domain protein 2	NM_001326402.1
B17G0895	13.88	12.12	ACYP1	Acylphosphatase-1	
B23G0443	13.87	4.97	PLEKHN1	Pleckstrin homology domain-containing family N member 1	XM_692642.9
A24G0526	13.87	11.64	CDV3	Protein CDV3 homolog	NM_212721.1
B3G0840	13.87	13.56	LOC113047152	A0A6P6JX50_CARAU mucin-5AC-like	
A2G0843	13.86	10.56	ZNRF1	E3 ubiquitin-protein ligase ZNRF1	NM_212983.1
A7G0443	13.86	9.16	HSD17B12B	Very-long-chain 3-oxoacyl-CoA reductase-B	NM_199613.1
A7G1076	13.86	15.26	RTN3	Reticulon-3	XM_021477447.2
A19G0759	13.86	7.65	EVA1B	Protein eva-1 homolog B	NM_001114461.1
B11G0350	13.84	6.47	CDH4	Cadherin-4	NM_001198741.1
A3G0839	13.84	21.83	NUBP1	Cytosolic Fe-S cluster assembly factor nubp1	NM_001017538.2
B10G0038	13.83	26.01	DCP2	m7GpppN-mRNA hydrolase	NM_200152.1
B11G0788	13.83	46.10			NM_201287.1
A7G0656	13.83	2.97	ESRP2	Epithelial splicing regulatory protein 2	XM_017356901.3
B7G0561	13.82	9.43	ACP2	Lysosomal acid phosphatase	NM_001013337.2
B8G0529	13.82	6.56	WAS	Actin nucleation-promoting factor WAS	XM_694827.9
A6G0525	13.82	5.75	MRPL47	39S ribosomal protein L47, mitochondrial	
B19G0945	13.82	32.65	CLIC1	Chloride intracellular channel protein 1	NM_212682.1
B9G0578	13.82	10.51	RNASEH2B	Ribonuclease H2 subunit B	NM_200271.1
B5G0382	13.82	5.91	AATF	Protein AATF	XM_005165555.5
B15G0588	13.82	6.95	EML1	Echinoderm microtubule-associated protein-like 1	NM_001030235.1

A8G0614	13.82	11.24	SMARCB1A	SNF-related matrix-associated actin-dependent regulator of chromatin subfamily B member 1-A	NM_001007296.1
B3G1294	13.81	1.81	HEXD	Hexosaminidase D	XM_009299336.4
A7G1313	13.81	13.51	HNRNPC	Heterogeneous nuclear ribonucleoprotein C	XM_021477445.2
B9G0597	13.81	11.17	SRPX	Sushi repeat-containing protein SRPX	NM_001014326.2
B12G0497	13.81	7.38	ATPAF2	ATP synthase mitochondrial F1 complex assembly factor 2	NM_001082821.1
B16G0739	13.80	21.07	COPS7A	COP9 signalosome complex subunit 7a	NM_001305596.1
B24G0307	13.80	27.10	LOC113042371	A0A6P6JF00_CARAU eotaxin-like	
B3G0911	13.80	12.43	DCAF7	DDB1- and CUL4-associated factor 7	NM_200069.1
B14G0674	13.80	20.67	CTBP1	C-terminal-binding protein 1	NM_131714.3
B13G0931	13.79	13.56			NM_200001.2
B17G0690	13.79	22.15	EPRS1	proline--tRNA ligase	NM_001288652.1
B6G0240	13.79	7.93	COQ7	5-demethoxyubiquinone hydroxylase, mitochondrial	NM_001083011.1
A5G0449	13.79	16.68	ERAP1	Endoplasmic reticulum aminopeptidase 1	XM_009301453.4
A10G0314	13.79	17.22	RAB6A	Ras-related protein Rab-6A	NM_001328529.1
B13G0652	13.79	2.59	MKKS	Molecular chaperone MKKS	NM_001002418.1
B5G0833	13.79	14.35	IGBP1	Immunoglobulin-binding protein 1	XM_005165297.5
A12G0391	13.79	27.75	MAP3K8	Mitogen-activated protein kinase kinase kinase 8	XM_687085.8
B10G0417	13.79	7.95	MRPL49	39S ribosomal protein L49, mitochondrial	NM_001386320.1
A21G0501	13.79	23.15	PPME1	Protein phosphatase methylesterase 1	NM_199937.1
B22G0528	13.79	8.16			
B3G1365	13.78	9.21	MCRIP1	Mapk-regulated corepressor-interacting protein 1	NM_001045471.2
B21G0937	13.78	47.51	TNFRSF14	Tumor necrosis factor receptor superfamily member 14	NM_001430990.1
B23G0860	13.78	4.43	G5714_022276	A0A7J6BMP8_9TELE Uncharacterized protein	
B1G0260	13.77	10.84	FIP1L1	Pre-mRNA 3'-end-processing factor FIP1	XM_009306697.3
B12G0427	13.77	7.54	GNA13	Guanine nucleotide-binding protein subunit alpha-13	NM_001012243.3
A24G0291	13.76	6.02	TNIK	TRAF2 and NCK-interacting protein kinase	XM_005162778.5
B2G0435	13.76	15.05	TSPAN3	Tetraspanin-3	NM_001305572.1
A12G0042	13.76	21.52	ACOD	Acyl-CoA desaturase	NM_198815.2
A14G0293	13.76	15.66	CLINT1	Clathrin interactor 1	NM_001003412.1
B13G0517	13.76	10.09	PCGF6	Polycomb group RING finger protein 6	NM_001327924.1
B12G0459	13.76	27.09	MAFF	Transcription factor MafF	NM_200336.1
B14G0185	13.75	18.08	UBL3	Ubiquitin-like protein 3	NM_212856.1
B19G0842	13.75	14.15	PTPN2	Tyrosine-protein phosphatase non-receptor type 2	NM_212654.1
B7G1008	13.75	8.51	MRPS11	28S ribosomal protein S11, mitochondrial	NM_001044797.1
B8G0164	13.74	14.64	CHMP7	Charged multivesicular body protein 7	NM_200781.1
B8G0644	13.74	16.97	CRY1	Cryptochrome-1	NM_001313822.1

A4G1025	13.74	7.16	NAGA	Alpha-N-acetylgalactosaminidase	NM_001037388.2
B16G0436	13.74	16.33	CNOT3	CCR4-NOT transcription complex subunit 3	XM_005158349.5
B17G0514	13.74	8.00	MSRA	Mitochondrial peptide methionine sulfoxide reductase	XM_009293138.4
A22G0222	13.74	5.53	CENPP	Centromere protein P	NM_001003555.3
B17G0122	13.73	6.20	AFG1L	AFG1-like ATPase	NM_001199767.1
A3G0714	13.73	39.09	AP2A1	AP-2 complex subunit alpha-1	XM_068219099.1
B17G0177	13.73	18.92	SNX6	Sorting nexin-6	NM_001030217.2
B3G0353	13.73	21.18	SLC44A2	Choline transporter-like protein 2	NM_198871.1
B3G1041	13.72	25.10	STAT3	Signal transducer and activator of transcription 3	NM_131479.1
A16G0194	13.72	12.78	OXR1	Oxidation resistance protein 1	XM_021466737.2
B4G0391	13.72	5.07	TSPAN33	Tetraspanin-33	NM_001002617.2
B2G0944	13.72	18.43	EIF4G1	Eukaryotic translation initiation factor 4 gamma 1	NM_001080200.1
A17G0116	13.72	4.17	SMOC1	SPARC-related modular calcium-binding protein 1	XM_021467094.2
B22G0683	13.72	18.94	RABGAP1	Rab GTPase-activating protein 1	XM_021469587.2
B18G0174	13.71	1.87	TRY3	Trypsin-3 (Fragment)	
A16G0740	13.70	16.27	EPN2	Epsin-2	XM_068213928.1
A1G0008	13.70	11.45	ITGAM	Integrin alpha-M	NM_001386625.1
A24G0083	13.70	6.39	NUBP2	Cytosolic Fe-S cluster assembly factor nubp2	NM_001037114.2
A3G0276	13.70	10.81	FARSA	Phenylalanine--tRNA ligase alpha subunit	NM_001045295.2
B10G0578	13.70	4.82	USPL1	SUMO-specific isopeptidase USPL1	NM_001003880.1
A5G1284	13.70	7.65	EIF2B1	Translation initiation factor eIF-2B subunit alpha	XM_009302036.4
B7G0281	13.69	12.31	CHCHD7	Coiled-coil-helix-coiled-coil-helix domain-containing protein 7	
B8G0032	13.69	11.88	CDK11B	Cyclin-dependent kinase 11B	XM_068222936.1
A4G0721	13.68	14.27	CALUA	Calumenin-A	NM_001317392.1
B3G1438	13.68	25.25	RAC1	Ras-related C3 botulinum toxin substrate 1	NM_001039818.1
B5G0080	13.67	27.35	SAT1	Diamine acetyltransferase 1	NM_001030199.1
B9G0021	13.67	10.31	SEPTIN10	Septin-10	NM_001017557.1
B6G0059	13.67	7.35	NTAN1	Protein N-terminal asparagine amidohydrolase	NM_200851.1
A14G0249	13.67	13.21	UPF3B	Regulator of nonsense transcripts 3B	NM_200954.1
B10G0082	13.67	6.61	CNTFR	Ciliary neurotrophic factor receptor subunit alpha	NM_001098242.2
B16G0406	13.67	14.45	AKIRIN1	Akirin-1	NM_001007186.2
B7G1133	13.66	10.41	ACADVL	Very long-chain specific acyl-CoA dehydrogenase, mitochondrial	XM_068222373.1
B19G0618	13.66	4.63	ELP2	Elongator complex protein 2	NM_001044925.1
A10G0717	13.66	15.82			
B16G0148	13.65	13.69	UBQLN4	Ubiquilin-4	NM_213356.2
A18G0095	13.65	8.97	PEX16	Peroxisomal membrane protein PEX16	NM_001025169.2
B5G1033	13.64	7.08	SFRP1	Secreted frizzled-related protein 1	NM_205585.2
A25G0496	13.64	8.67	DAPK3	Death-associated protein kinase 3	XM_679634.9
A21G0849	13.63	22.00	HSPA4	Heat shock 70 kDa protein 4	NM_214716.1

B23G0786	13.63	0.02	TGM1	Protein-glutamine gamma-glutamyltransferase K	XM_009297092.4
A14G0733	13.63	15.84	SQSTM1	Sequestosome-1	NM_001312913.1
B10G0707	13.63	10.23	SSBP2	Single-stranded DNA-binding protein 2	XM_009305684.4
B13G0734	13.63	8.75	SFRP5	Secreted frizzled-related protein 5	NM_001002373.1
A16G0335	13.63	6.46	CITED3	p300-interacting transactivator 3	NM_200078.2
B6G0603	13.63	0.84	RHCG1	Ammonium transporter Rh type C 1	NM_001109843.1
B3G1053	13.62	9.53	HRAS	GTPase HRas	NM_200258.1
B5G0691	13.62	10.68	TRPV1	Transient receptor potential cation channel subfamily V member 1	XM_005165327.5
B1G0242	13.62	34.18	CLTA	Clathrin light chain A	NM_213045.1
A13G0068	13.62	6.65	ZC3H6	Zinc finger CCCH domain-containing protein 6	NM_001017621.1
B20G0741	13.62	6.19	DUSP23	Dual specificity protein phosphatase 23	
B3G0310	13.62	7.80	DNAJA3	DnaJ homolog subfamily A member 3, mitochondrial	XM_005168471.5
B25G0245	13.62	14.00	MOB2	MOB kinase activator 2	NM_001002364.2
B3G0641	13.62	15.90	MAP3K14	Mitogen-activated protein kinase kinase kinase 14	XM_068219258.1
B16G0652	13.61	36.19	B915	Maternal B9.15 protein	XM_003200187.6
B23G0762	13.61	4.93	PLEKHG5	Pleckstrin homology domain-containing family G member 5	XM_068216936.1
A18G0621	13.61	7.85	RBM28	RNA-binding protein 28	NM_200321.1
B5G0838	13.60	7.20	STEEP1	STING ER exit protein	NM_001004659.1
A22G0088	13.60	6.42	GRAMD2B	GRAM domain-containing protein 2B	
B18G0678	13.60	15.24	PRPF18	Pre-mRNA-splicing factor 18	XM_021467629.2
B3G0821	13.60	18.88	KAT7	Histone acetyltransferase KAT7	NM_001076584.1
B18G0845	13.60	19.87	SPPL2A	Signal peptide peptidase-like 2A	NM_001037439.1
B5G0329	13.60	10.62	KAT5	Histone acetyltransferase KAT5	NM_001013309.3
B19G0372	13.59	7.92	TRA2A	Transformer-2 protein homolog alpha	NM_200416.1
A5G0126	13.59	5.69	MYBBP1A	Myb-binding protein 1A-like protein	NM_001002042.2
A7G0403	13.59	5.88	PHKA1	Phosphorylase b kinase regulatory subunit alpha, skeletal muscle isoform	XM_017357095.3
B22G0578	13.59	3.94	SLX9	Ribosome biogenesis protein SLX9 homolog	NM_001045366.1
A12G0366	13.59	15.11	PPM1B	Protein phosphatase 1B	XM_005156264.5
A19G0401	13.59	8.61	CHRAC1	Chromatin accessibility complex protein 1	NM_001013293.2
A1G0884	13.58	33.46	ATNB233	potassium-transporting ATPase subunit beta-233	NM_131671.1
B12G0775	13.58	7.48	POLR3E	DNA-directed RNA polymerase III subunit RPC5	XM_068224531.1
B20G0857	13.58	9.30	CRNKL1	Crooked neck-like protein 1	NM_200946.1
B1G0974	13.57	24.18	CRTAC1	Cartilage acidic protein 1	NM_001080178.1
B11G0743	13.57	8.16	STRN	Striatin	
A17G0400	13.57	8.35	LARP1	La-related protein 1	XM_691468.9
B7G0077	13.57	10.03	SLC29A2	Equilibrative nucleoside transporter 2	XM_068222803.1
B5G0295	13.57	8.91	B4GALT4	Beta-1,4-galactosyltransferase 4	NM_001366733.1
A21G0219	13.55	6.21	CD8A1	A8HDP0_CYPCA CD8 alpha 1	NM_001040049.1
B5G0678	13.55	8.20	GRSF1	G-rich sequence factor 1	XM_005165334.3
B7G0351	13.55	5.64	RWDD4	RWD domain-containing protein 4	NM_001320090.1

A1G1012	13.55	5.68	POLR1E	DNA-directed RNA polymerase I subunit RPA49	
B20G0867	13.54	6.00	RIOX1	Ribosomal oxygenase 1	NM_001089388.1
B10G0384	13.54	67.77	POSTN	Periostin	XM_002663548.6
B17G0218	13.53	14.04	GPN1	GPN-loop GTPase 1	NM_001003633.1
B3G1395	13.53	15.68	UQCRC2	Cytochrome b-c1 complex subunit 2, mitochondrial	NM_001002657.2
A3G0524	13.53	8.56	PRPSAP2	Phosphoribosyl pyrophosphate synthase-associated protein 2	NM_213496.1
A20G0061	13.53	14.06	DSP	Desmoplakin	NM_001423427.1
B19G0380	13.53	3.41	NFE2L1	Endoplasmic reticulum membrane sensor NFE2L1	XM_005173768.5
B17G0407	13.52	16.73	PTEN	Phosphatidylinositol 3,4,5-trisphosphate 3-phosphatase and dual-specificity protein phosphatase PTEN	NM_200708.2
B22G0299	13.52	11.52	LOC113080588	A0A6P6NHE2_CARAU uncharacterized protein LOC113080588 isoform X1	XM_021470864.2
B13G0395	13.52	7.64	HK1	Hexokinase-1	NM_200566.1
B11G0348	13.51	32.14	PIM1	threonine-protein kinase pim-1	NM_200189.2
B10G0848	13.51	36.99	ETS2	Protein C-ets-2	NM_001023580.1
B7G0872	13.51	8.06			XM_068222601.1
B21G0962	13.51	0.00	KLF4	Krueppel-like factor 4	NM_001113483.1
A16G0358	13.51	6.35	GNL2	Nucleolar GTP-binding protein 2	NM_213224.1
A7G0801	13.49	14.33	TMEM9B	Transmembrane protein 9B	NM_199732.2
A6G0227	13.49	9.61	CCR7	C-C chemokine receptor type 7	NM_001098743.1
B12G0431	13.49	8.59	DESI1	Desumoylating isopeptidase 1	NM_200208.1
A21G0150	13.49	3.74	CDK9	Cyclin-dependent kinase 9	NM_212591.2
B5G0671	13.48	2.87	RTKN	Rhotekin	XM_005165339.5
A20G0534	13.47	7.29	MIDEAS	Mitotic deacetylase-associated SANT domain protein	XM_005160589.5
B7G0184	13.47	40.86	IPO7	Importin-7	NM_200905.3
B11G0407	13.47	27.14	RBM39	RNA-binding protein 39	NM_214739.1
B21G0452	13.46	3.02	ST14	Suppressor of tumorigenicity 14 protein homolog	XM_009295332.4
A5G0534	13.46	10.43	ARCN1	Coatomer subunit delta	NM_201459.1
B14G0207	13.46	10.57	MAP7	Ensconsin	XM_001922471.7
A15G0854	13.45	13.47	KPNA4	Importin subunit alpha-3	NM_201305.1
B6G0732	13.45	9.86	RUVBL1	RuvB-like 1	NM_173835.4
B2G0966	13.45	10.73	GNG12	G(O) subunit gamma-12	NM_213312.2
B16G0111	13.45	64.31	NR1D2	Nuclear receptor subfamily 1 group D member 2	NM_001130592.2
B6G0823	13.45	3.24	FAM107A	Actin-associated protein FAM107A	XR_002459113.2
B9G0315	13.45	16.87	CCDC80	Coiled-coil domain-containing protein 80	NM_001007198.1
A10G0532	13.45	10.74	CHORDC1	Cysteine and histidine-rich domain-containing protein 1	NM_200339.1
A5G0793	13.44	7.72	EMC6	ER membrane protein complex subunit 6	
A25G0116	13.44	5.20	SNX24	Sorting nexin-24	NM_001045374.2
B15G0682	13.44	48.38	POSTN	Periostin	NM_001077786.1
B19G0047	13.43	8.05	FAM126A	Hyccin	NM_205630.1
B17G0702	13.43	12.46	ARF6	ADP-ribosylation factor 6	NM_001161375.1
B4G0569	13.43	8.80	GATA3	Transcription factor GATA-3	NM_131211.1
B21G0404	13.43	18.41	NXF1	Nuclear RNA export factor 1	XM_001923926.6
B1G0905	13.43	59.86	A0A672K994	A0A672K994_SINGR Uncharacterized protein	
B19G0252	13.43	9.64	SCAMP3	Secretory carrier-associated membrane protein 3	NM_001007345.1

A2G1140	13.43	14.79	RANBP3	Ran-binding protein 3	NM_200690.1
A7G0169	13.43	2.36	USP53	Inactive ubiquitin carboxyl-terminal hydrolase 53	XM_017357133.3
B22G0956	13.43	6.86	G5714_021923	A0A7J6BUC6_9TELE Ig-like domain-containing protein	XR_002456444.2
B7G1286	13.42	19.47	SUPT16H	FACT complex subunit SPT16	NM_001097584.2
A24G0223	13.42	14.78	RSU1	Ras suppressor protein 1	XM_021470373.2
A16G0958	13.42	15.49	MBP	Myelin basic protein	NM_001271459.1
A8G0803	13.41	3.24	LOC107722987	A0A673LC95_9TELE Disintegrin and metalloproteinase domain-containing protein 9-like	
B22G0521	13.41	8.31	BTG1	Protein BTG1	NM_001007375.2
A24G0141	13.41	19.25	UBE2I	SUMO-conjugating enzyme UBC9	NM_131351.2
A23G0300	13.41	11.40	SEPHS3	Selenide, water dikinase 3	NM_001004295.2
A7G0177	13.40	6.80	ELP5	Elongator complex protein 5	NM_001080067.2
A9G0141	13.40	10.98	ITPRID2	Protein ITPRID2	XM_021479016.2
A10G0887	13.39	3.56	SLC27A6	Long-chain fatty acid transport protein 6	XM_003199319.6
A9G0805	13.39	21.63	PDK1	[Pyruvate dehydrogenase (acetyl-transferring)] kinase isozyme 1, mitochondrial	XM_678484.7
B3G0875	13.39	13.83	FRIM	Ferritin, middle subunit	NM_001109854.1
A20G0032	13.38	10.24	ARF6	ADP-ribosylation factor 6	NM_199993.1
B16G0804	13.38	16.69	EPN2	Epsin-2	NM_001328116.1
A6G0506	13.37	12.57	TPRG1L	Tumor protein p63-regulated gene 1-like protein	NM_001089528.2
A17G0456	13.37	16.83	MTFR1L	Mitochondrial fission regulator 1-like	NM_199884.1
B19G0517	13.37	24.61	CFAD	Counting factor associated protein D	NM_001017633.2
A14G0258	13.36	6.50	C1GALT1C1	C1GALT1-specific chaperone 1	NM_199667.3
B8G0395	13.36	17.10	OXCT1	Succinyl-CoA:3-ketoacid coenzyme A transferase 1, mitochondrial	NM_001007291.1
B6G0235	13.36	13.88	MTX2	Metaxin-2	NM_212575.2
A16G0682	13.36	6.71	ZNF384	Zinc finger protein 384	XM_009292250.4
A5G0599	13.36	7.63	POLE3	DNA polymerase epsilon subunit 3	NM_200801.1
A24G0058	13.35	11.60	LOC113068780	A0A6P6MMY6_CARAU uncharacterized protein LOC113068780	XM_068217267.1
B1G0856	13.35	7.26	A0A673IGG2	A0A673IGG2_9TELE Uncharacterized protein	
B5G0969	13.35	10.25	LOC107717871	A0A673KVJ4_9TELE Uncharacterized LOC107717871	
B19G0221	13.34	22.09	LOC107693314	A0A671PC20_9TELE Nucleolar protein 9-like	
B2G0218	13.33	5.99	AP1S3	AP-1 complex subunit sigma-3	NM_001004635.1
B4G0225	13.33	13.85	ADIPOR2	Adiponectin receptor protein 2	NM_001025506.2
B20G0099	13.32	7.74	TRAF3IP2	E3 ubiquitin ligase TRAF3IP2	XM_005173802.5
A10G0117	13.32	9.43	KMT5AA	N-lysine methyltransferase KMT5A-A	NM_001045349.2
A3G0554	13.32	12.98	MAP3K14	Mitogen-activated protein kinase kinase kinase 14	XM_068219258.1
B19G0297	13.32	3.08	BANF1	Barrier-to-autointegration factor	XR_011007520.1
A25G0126	13.32	10.90	WDR61	WD repeat-containing protein 61	NM_200853.1
B16G0611	13.32	8.33	TOMM40	Mitochondrial import receptor subunit TOM40 homolog	XM_017351318.3

A11G0659	13.32	13.69	CCDC3	Coiled-coil domain-containing protein 3	XM_003201652.6
A3G0534	13.31	9.29	NMT1	Glycylpeptide N-tetradecanoyltransferase 1	NM_001020480.1
B7G0909	13.31	11.32	LRP10	Low-density lipoprotein receptor-related protein 10	XM_683767.10
A6G0619	13.31	11.36	NIT2	Omega-amidase NIT2	NM_205611.3
B8G0088	13.31	77.53	KBP	Glutamate receptor U1	NM_001144802.1
B9G0900	13.31	9.55	RRP1	Ribosomal RNA processing protein 1 homolog A	XM_068223520.1
A13G0863	13.30	5.07	HSP30	Heat shock protein 30	XM_003199759.6
B19G0377	13.30	8.35	OSBPL3	Oxysterol-binding protein-related protein 3	NM_001004646.1
B3G1077	13.30	7.55	TMEM11	Transmembrane protein 11, mitochondrial	NM_001017400.1
B11G0486	13.30	10.26	ECE1	Endothelin-converting enzyme 1	NM_001326447.1
B20G0822	13.30	12.94	FEZ2	Fasciculation and elongation protein zeta-2	NM_001077432.1
B22G0520	13.30	8.73	IPPK	Inositol-pentakisphosphate 2-kinase	NM_001014324.1
B1G0987	13.30	9.21	LUC7L3	Luc7-like protein 3	NM_001089547.2
A20G0880	13.30	6.86	NUP43	Nucleoporin Nup43	NM_212892.1
B24G0597	13.30	6.45	YAE1	Protein YAE1 homolog	NM_213142.1
B21G0723	13.29	5.71	CD8A2	A8HDP2_CYPCA CD8 alpha 2	NM_001040049.1
B21G0386	13.29	22.51	SART1	U6.U5 tri-snRNP-associated protein 1	NM_001002673.1
A20G0021	13.29	35.33	CIPC	CLOCK-interacting pacemaker	
B5G0625	13.29	12.25	ARRDC3	Arrestin domain-containing protein 3	NM_001080029.1
B16G0771	13.29	15.97	PAFAH1B3	Platelet-activating factor acetylhydrolase IB subunit alpha1	NM_201058.1
B6G0693	13.28	3.01	LOC113094658	A0A6P6P4H1_CARAU collagen alpha-1(VII) chain-like isoform X2	XM_068222026.1
B3G0431	13.28	7.13	METRNL	Meteorin-like protein	NM_212985.1
B4G0025	13.28	10.90	NAGA	Alpha-N-acetylgalactosaminidase	NM_001037388.2
B13G0185	13.28	8.47	EPAS1	Endothelial PAS domain-containing protein 1	NM_194412.2
B21G0314	13.28	5.37	ALOXE3	Hydroperoxide isomerase ALOXE3	NM_001076710.2
B20G0049	13.28	32.67	AMD1	S-adenosylmethionine decarboxylase proenzyme	NM_001161601.1
A3G0272	13.28	16.74	IRE-1	endoribonuclease ire-1	
A15G0231	13.28	12.08	AGFG1	Arf-GAP domain and FG repeat-containing protein 1	XM_021481344.2
A7G0283	13.28	2.53	A0A672K7H1	A0A672K7H1_SINGR Integrase catalytic domain-containing protein	
A2G0574	13.28	14.80	UROD	Uroporphyrinogen decarboxylase	NM_131347.1
B12G0394	13.27	9.46	MFSD8	Major facilitator superfamily domain-containing protein 8	NM_001045048.1
B13G0796	13.27	4.20	CHP1	Calcineurin B homologous protein 1	NM_001045456.1
A5G0269	13.27	7.36	MMAB	Corrinoid adenosyltransferase MMAB	NM_001082809.1
A25G0062	13.26	9.96	CHID1	Chitinase domain-containing protein 1	NM_200057.1
A9G0280	13.26	22.34	ME3	NADP-dependent malic enzyme, mitochondrial	NM_001089356.1

B3G1191	13.26	2.20	G5714_003132	A0A7J6D8M6_9TELE SAND domain-containing protein	
A5G0283	13.26	4.25	PXMP2	Peroxisomal membrane protein 2	NM_001004607.1
A5G0264	13.26	13.85	ACACA	Acetyl-CoA carboxylase 1	
A21G0637	13.25	19.36	RHOG	Rho-related GTP-binding protein RhoG	NM_200040.2
B15G0542	13.25	8.42	TSR1	Pre-rRNA-processing protein TSR1 homolog	XM_005157568.5
B3G0345	13.25	36.63	TMEM205	Transmembrane protein 205	XM_005168478.4
A3G0553	13.25	11.08	BAX	Apoptosis regulator BAX	NM_131562.2
B4G0036	13.25	15.98	EPS8	Epidermal growth factor receptor kinase substrate 8	XM_009299922.4
A6G0202	13.24	9.82	SUCLA2	Succinate--CoA ligase [ADP-forming] subunit beta, mitochondrial	NM_213026.1
B12G0002	13.24	17.66	GHITM	Growth hormone-inducible transmembrane protein	
A20G0453	13.24	8.20	AVEN	Cell death regulator Aven	NM_001045292.2
B14G0302	13.24	5.51	RNASEH2C	Ribonuclease H2 subunit C	NM_001161377.1
A5G0005	13.24	21.91	JUN	Transcription factor Jun	NM_001431134.1
A18G0073	13.24	8.10	KCNK6	Potassium channel subfamily K member 6	NM_001030074.2
B23G0874	13.24	14.77	CTNNBL1	Beta-catenin-like protein 1	NM_200866.1
B1G0804	13.23	8.05	STN1	CST complex subunit STN1	NM_200389.1
B17G0381	13.23	5.13	MAL	Myelin and lymphocyte protein	NM_001004625.1
B8G0585	13.23	11.87	SLC38A3	Sodium-coupled neutral amino acid transporter 3	NM_001002648.1
A5G0377	13.23	9.92	GBGT1	Globoside alpha-1,3-N-acetylgalactosaminyltransferase 1	XM_005165128.5
B3G0102	13.23	18.31	GIMAP5	GTPase IMAP family member 5	NM_001128253.1
B3G0099	13.23	18.31	GIMAP5	GTPase IMAP family member 5	NM_001128253.1
A22G0387	13.22	2.93	NR2C2AP	Nuclear receptor 2C2-associated protein	NM_001045327.2
B15G0627	13.22	15.92	LGALS9	Galectin-9	NM_001001817.2
A11G0421	13.22	11.54	EIF4G3	Eukaryotic translation initiation factor 4 gamma 3	NM_213539.1
B12G0012	13.22	39.60	OAT	Ornithine aminotransferase, mitochondrial	NM_001317170.1
A18G0221	13.22	12.75			
A4G0576	13.21	11.97	FAM3C	Protein FAM3C	NM_212725.1
A3G0454	13.21	12.52	LOC107568934	A0A672K614_SINGR Interleukin-4 receptor subunit alpha-like	
A11G0191	13.21	10.24	ZFYVE9	Zinc finger FYVE domain-containing protein 9	NM_198365.1
A12G0062	13.21	14.62	ANXA11	Annexin A11	
A23G0665	13.21	7.45	CCNT1	Cyclin-T1	NM_001256611.1
B20G0384	13.20	15.26	MAP3K7	Mitogen-activated protein kinase kinase kinase 7	XM_068215443.1
B22G0891	13.20	23.02	ICOSLG	ICOS ligand	
A19G0684	13.20	12.21	SCAMP3	Secretory carrier-associated membrane protein 3	NM_001007345.1
A19G0051	13.20	26.08	NR1D2	Nuclear receptor subfamily 1 group D member 2	NM_131065.1
A4G0875	13.20	15.30	LYRM5A	LYR motif-containing protein 5A	
A1G0966	13.20	14.71	ASAH1	Acid ceramidase	NM_200577.1
B22G0638	13.20	7.72	MRPL54	39S ribosomal protein L54, mitochondrial	

B11G0534	13.19	5.34	PGAP4	Post-GPI attachment to proteins factor 4	XM_021479878.2
A24G0216	13.18	3.03	ITPRID1	Protein ITPRID1	XM_005162860.5
B19G0509	13.18	14.96	UBP1	Upstream-binding protein 1	NM_001114574.2
B23G0683	13.18	5.31	SNRPG	Small nuclear ribonucleoprotein G	NM_001004660.3
A8G0658	13.18	3.34	FZD10	Frizzled-10	NM_130917.2
B16G1119	13.17	14.20	DCTN3	Dynactin subunit 3	NM_001002220.1
A19G0200	13.17	8.42	NEU1	Sialidase-1	
B12G0695	13.16	7.39	NOC3L	Nucleolar complex protein 3 homolog	NM_001002863.1
A10G0567	13.16	4.69	BAP18	Chromatin complexes subunit BAP18	NM_001008605.1
A11G0696	13.16	5.46	SMIM7	Small integral membrane protein 7	XM_021479914.2
B21G0103	13.16	5.45	BRCC3	Lys-63-specific deubiquitinase BRCC36	NM_001423851.1
B11G0785	13.16	3.90			XM_005155673.5
A23G0476	13.16	3.59	PKIG	cAMP-dependent protein kinase inhibitor gamma	NM_001309188.1
B2G1190	13.15	0.00	CDC73	Parafibromin	NM_200348.2
A19G0445	13.14	7.68	SESN2	Sestrin-2	NM_001079975.2
A24G0071	13.14	12.94	IGKV4-1	Immunoglobulin kappa variable 4-1	NM_001045408.1
A8G0351	13.14	10.07	NOTCH2	Neurogenic locus notch homolog protein 2	NM_001115094.2
A5G0984	13.14	10.00	TTF1	Transcription termination factor 1	XM_005168641.5
A23G0365	13.14	8.62	ARHGEF3	Rho guanine nucleotide exchange factor 3	XM_005162074.5
B21G0394	13.14	9.76	BAD	Bcl2-associated agonist of cell death	XM_005161364.5
B16G0367	13.14	6.77	CITED3	p300-interacting transactivator 3	NM_200078.2
B18G0647	13.13	21.99	FAM107B	Protein FAM107B	NM_001328191.1
A6G0746	13.13	8.13	WDR82	WD repeat-containing protein 82	NM_001166268.1
A25G0505	13.13	17.54	RPS27L	40S ribosomal protein S27-like	
A15G0137	13.12	11.46	G5714_015384	A0A7J6CAR3_9TELE Single-pass membrane and coiled-coil domain-containing protein 4	
B16G0371	13.12	25.65	CTPS1	CTP synthase 1	NM_001111247.1
A19G0624	13.12	9.45	LIPE	Hormone-sensitive lipase	NM_001316725.1
B2G1154	13.11	14.86	CA021	Uncharacterized protein C1orf21 homolog	NM_001291356.1
A16G0875	13.11	5.23	LOC107707940	A0A673JL54_9TELE FK506-binding protein 3-like	
A3G0374	13.11	8.90	PBX1	Pre-B-cell leukemia transcription factor 1	NM_131447.1
A18G0487	13.11	18.34	SF3B3	Splicing factor 3B subunit 3	NM_213503.1
B7G1188	13.11	0.59	CNFN-B	Cornifelin homolog B	
A18G0769	13.11	14.49	DHDH	Trans-1,2-dihydrobenzene-1,2-diol dehydrogenase	NM_001005600.2
A5G0187	13.10	16.29	PPIL2	RING-type E3 ubiquitin-protein ligase PPIL2	NM_200991.2
B3G0683	13.10	12.56	CSN1	COP9 signalosome complex subunit 1	NM_001077759.1
B14G0621	13.10	14.18	SRP72	Signal recognition particle subunit SRP72	XM_017359078.3
B15G0530	13.10	9.29	CLUH	Clustered mitochondria protein homolog	NM_001077560.1
B3G0881	13.10	0.75	FRIM	Ferritin, middle subunit	NM_001109854.1

A17G0399	13.10	12.26	PGRMC2	Membrane-associated progesterone receptor component 2	NM_213104.1
B1G0072	13.10	12.76	CD2	T-cell surface antigen CD2	
A12G0775	13.10	8.18	NIF3L1	NIF3-like protein 1	
A24G0635	13.10	10.99	NCK1	Cytoplasmic protein NCK1	XM_068217135.1
A17G0820	13.10	4.10	CRLS1	Cardiolipin synthase (CMP-forming)	NM_212931.1
B10G0061	13.09	9.68	LOC107715885	A0A673IQ52_9TELE Uncharacterized LOC107715885	NM_001020495.2
A16G0651	13.09	4.53	SLC50A1	Sugar transporter SWEET1	NM_001012497.2
A11G0219	13.09	8.82			NM_201113.1
A16G1042	13.08	18.55	ARNT	Aryl hydrocarbon receptor nuclear translocator	XM_005157852.5
B7G0196	13.08	4.61	CCND1	S-specific cyclin-D1	NM_131025.4
B19G0953	13.08	11.00	RNF2-A	E3 ubiquitin-protein ligase RING2-A	
A14G0148	13.08	15.19	XIAP	E3 ubiquitin-protein ligase XIAP	NM_194396.2
A19G0099	13.07	16.59	NLRC3	NLR family CARD domain-containing protein 3	
B17G0210	13.07	9.44	TFB2M	Dimethyladenosine transferase 2, mitochondrial	NM_001113617.2
B11G0266	13.07	10.60	KRT15	Keratin, type I cytoskeletal 15	NM_001365237.1
B1G1161	13.07	18.14	VAV1	Proto-oncogene vav	XR_011014280.1
A24G0540	13.07	6.31	GIMAP4	GTPase IMAP family member 4	NM_001366027.1
A11G0044	13.07	7.21	TOMM20B	Mitochondrial import receptor subunit TOM20 homolog B	NM_001006072.1
A1G0057	13.07	8.30	TMED1	Transmembrane emp24 domain-containing protein 1	NM_001080640.1
A12G0627	13.06	8.55	HTRA1B	Serine protease HTRA1B	NM_001111182.1
B20G0205	13.06	15.95	TMEM30A	Cell cycle control protein 50A	NM_205560.3
A4G0587	13.06	10.18	SND1	Staphylococcal nuclease domain-containing protein 1	NM_182865.3
A12G0580	13.06	11.88	MED1	Mediator of RNA polymerase II transcription subunit 1	XM_021480458.2
A1G0293	13.05	5.64	NDUFAF5	Arginine-hydroxylase NDUFAF5, mitochondrial	NM_001082894.2
A3G0809	13.05	10.64	GRAP2	GRB2-related adaptor protein 2	NM_001017756.2
A5G0803	13.05	8.56	CXCL11.6	C-X-C motif chemokine 11-6	
B7G1137	13.04	7.12	ABR	Active breakpoint cluster region-related protein	XM_021471891.2
B11G0014	13.04	12.03	SLC11A2	Natural resistance-associated macrophage protein 2	NM_214690.1
A19G0633	13.04	17.51	USF2	Upstream stimulatory factor 2	NM_001122785.1
A21G0887	13.04	5.45	WDR55	WD repeat-containing protein 55	NM_001003871.2
B12G0783	13.04	12.02	COPS3	COP9 signalosome complex subunit 3	NM_199685.1
B1G0182	13.04	8.77	PMM2	Phosphomannomutase 2	NM_200084.1
B8G0396	13.04	33.39	RIMOC1	RAB7A-interacting MON1-CCZ1 complex subunit 1	NM_001128229.1
A25G0768	13.03	16.06	WWP2	NEDD4-like E3 ubiquitin-protein ligase WWP2	XM_005163171.5
A3G0421	13.03	16.07	TYK2	Non-receptor tyrosine-protein kinase TYK2	XM_005164236.5
A5G0823	13.03	8.53	TARS1	Threonine--tRNA ligase 1, cytoplasmic	NM_001122786.1
B14G0628	13.03	14.78	MORC3	MORC family CW-type zinc finger protein 3	NM_001320211.1
B5G0677	13.02	5.35	MOB1B	MOB kinase activator 1B	NM_200200.1

A5G0592	13.02	7.58	NTMT1	N-terminal Xaa-Pro-Lys N-methyltransferase 1	NM_212898.1
A23G0447	13.02	13.96	CTDSP2	Carboxy-terminal domain RNA polymerase II polypeptide A small phosphatase 2	NM_199544.1
B19G0625	13.02	8.18	RP9	Retinitis pigmentosa 9 protein	XM_009294316.4
B2G0829	13.01	13.22	DNAJC1	DnaJ homolog subfamily C member 1	NM_001077535.1
B11G0808	13.01	8.49			NM_001309488.1
B1G0894	13.01	12.50	CLDND1	Claudin domain-containing protein 1	NM_001161597.1
A5G0652	13.01	11.41	YIPF6	Protein YIPF6	NM_001002096.1
A3G1226	13.01	3.70	IGK	Immunoglobulin kappa light chain	
A24G0391	13.00	10.88	FLT3	Receptor-type tyrosine-protein kinase FLT3	
B23G0738	13.00	10.21	AGAP2	Arf-GAP with GTPase, ANK repeat and PH domain-containing protein 2	XM_009297047.4
A13G0621	13.00	23.64	SI:CH211-233A24.2	Transmembrane protein KIAA1109 homolog	NM_001083577.1
B5G0964	13.00	10.45	ARCN1	Coatomer subunit delta	NM_201459.1
A12G0526	12.99	7.96	LIPA	cholesteryl ester hydrolase	NM_213404.1
B23G0469	12.99	12.81	KANSL2	KAT8 regulatory NSL complex subunit 2	XM_068216740.1
A23G0620	12.99	9.00	RNF41	E3 ubiquitin-protein ligase NRDP1	NM_213516.1
A12G0152	12.99	7.08	COX10	Protoheme IX farnesyltransferase, mitochondrial	NM_001166259.1
A13G0487	12.99	10.08	CCSAP	Centriole, cilia and spindle-associated protein	NM_001128728.1
B11G0104	12.99	6.44	ADGRL4	Adhesion G protein-coupled receptor L4	NM_001003590.1
A1G0072	12.98	10.68	SAMHD1	Deoxynucleoside triphosphate triphosphohydrolase SAMHD1	XM_009295834.4
B14G0013	12.98	10.38	CLNK	Cytokine-dependent hematopoietic cell linker	
B10G0096	12.97	11.28	PARM1	Prostate androgen-regulated mucin-like protein 1 homolog	
B14G0254	12.97	9.13	LAMP2	Lysosome-associated membrane glycoprotein 2	NM_001013533.1
B4G0488	12.97	3.37	G5714_004828	A0A7J6D6G3_9TELE Uncharacterized protein	
B19G0333	12.97	11.23	IGKV1-6	Immunoglobulin kappa variable 1-6	NM_001045408.1
B5G0943	12.97	7.51	CYB5D2	Neuferricin	NM_001102674.1
B10G0132	12.96	34.50	AK3	GTP:AMP phosphotransferase AK3, mitochondrial	NM_213130.2
A3G0883	12.96	23.13	STAT3	Signal transducer and activator of transcription 3	NM_131479.1
B2G0408	12.96	12.47	ZNRF2	E3 ubiquitin-protein ligase ZNRF2	NM_212983.1
A23G0438	12.95	21.99	SZRD1	SUZ domain-containing protein 1	NM_001030076.2
B19G0054	12.95	4.00	POLR1F	DNA-directed RNA polymerase I subunit RPA43	NM_001007153.1
B4G0583	12.95	5.51	TMCC3	Transmembrane and coiled-coil domain protein 3	NM_001044813.2
B11G0646	12.94	11.17	MFSD4A	Major facilitator superfamily domain-containing protein 4A	NM_001080647.1

A9G0416	12.94	16.53	IDH1	Isocitrate dehydrogenase [NADP] cytoplasmic	XM_005167655.5
A3G1094	12.94	9.23	SMARCA4	Transcription activator BRG1	NM_181603.1
B8G0955	12.93	12.71	CIAO1	Probable cytosolic iron-sulfur protein assembly protein ciao1	NM_200147.2
A15G0651	12.93	5.83	TRAPPC6B	Trafficking protein particle complex subunit 6b	NM_001305574.1
A25G0724	12.92	15.68	LSM14A	Protein LSM14 homolog A	XM_021470461.2
A19G0173	12.92	10.31	PTP4A3	Protein tyrosine phosphatase type IVA 3	NM_213181.1
A13G0286	12.92	5.62	ZFYVE26	Zinc finger FYVE domain-containing protein 26	NM_131606.2
A19G0239	12.92	74.18	MARCKSL1B	MARCKS-related protein 1-B	NM_213223.1
B5G0137	12.91	7.00	AGPAT9L	Glycerol-3-phosphate acyltransferase 3-like	NM_001099450.1
B10G0750	12.91	27.14	NUDCD3	NudC domain-containing protein 3	
A25G0440	12.91	9.89	API5-A	Apoptosis inhibitor 5-A	NM_199540.2
A14G0661	12.91	6.52	STX3	Syntaxin-3	NM_200543.1
A18G0485	12.91	7.09	AARS1	Alanine--tRNA ligase, cytoplasmic	NM_001044310.2
B11G0601	12.91	10.50	PSMA5	Proteasome subunit alpha type-5	NM_001020669.1
B19G0909	12.91	18.24	SF3A3	Splicing factor 3A subunit 3	NM_001004289.1
B10G0873	12.90	10.76	WDR70	WD repeat-containing protein 70	XM_068223880.1
A15G0409	12.90	5.45	TSR1	Pre-rRNA-processing protein TSR1 homolog	XM_005157568.5
B7G0332	12.89	17.85	G5714_007802	A0A7J6CUG4_9TELE Uncharacterized protein	
B4G0209	12.89	8.61	CDC123	Cell division cycle protein 123 homolog	NM_200721.1
B1G0571	12.89	7.71	LOC113044462	A0A6P6JKV9_CARAU uncharacterized protein LOC113044462 isoform X2	XM_068218019.1
A16G0222	12.89	13.80	HERPUD2	Homocysteine-responsive endoplasmic reticulum-resident ubiquitin-like domain member 2 protein	NM_200188.1
B20G0543	12.89	22.01	CD164	Sialomucin core protein 24	
A12G0012	12.89	8.64	BUB3	Mitotic checkpoint protein BUB3	XM_005169505.5
A16G0691	12.88	0.79	LOC107671188	A0A671P3D7_9TELE Uncharacterized LOC107671188	NM_001328123.1
A3G0282	12.88	15.18	HSPBP1	Hsp70-binding protein 1	NM_200075.2
B10G0693	12.88	0.86	DBNL	Drebrin-like protein	NM_001002201.1
A1G0239	12.87	7.83	COMMD1	COMM domain-containing protein 1	XM_005160149.5
B7G0676	12.87	9.05	ITLN	Intelectin	NM_001076581.1
A20G0112	12.87	13.34	XRN2	5'-3' exoribonuclease 2	NM_001001944.2
B22G0580	12.87	10.11	CNOT9	CCR4-NOT transcription complex subunit 9	NM_214760.2
B17G0085	12.86	10.96	NLRP3	NACHT, LRR and PYD domains-containing protein 3	XM_021473072.2
B15G0370	12.86	3.18	NFKBIZ	NF-kappa-B inhibitor zeta	NM_001020720.1
B2G0570	12.86	33.67	PLK3	threonine-protein kinase PLK3	NM_201308.2
A15G0544	12.86	36.62	HSPA8	Heat shock cognate 71 kDa protein	NM_001200012.1
B13G0508	12.85	14.21	SLC2A9	Solute carrier family 2, facilitated glucose transporter member 9	NM_001013462.3
B3G0517	12.85	20.16	PRKCSH	Glucosidase 2 subunit beta	XM_005164223.5

A10G0736	12.85	9.85	RBM18	Probable RNA-binding protein 18	NM_200793.1
B16G0838	12.85	8.17	DAP3	28S ribosomal protein S29, mitochondrial	NM_001098737.1
B3G1626	12.85	17.01	RTASE	Probable RNA-directed DNA polymerase from transposon BS	XM_068214194.1
B3G0643	12.84	11.28	GOSR2	Golgi SNAP receptor complex member 2	NM_199688.1
A8G0570	12.84	8.74	ADIPOR1	Adiponectin receptor protein 1	NM_213500.1
A2G1160	12.84	12.03	UPF1	Regulator of nonsense transcripts 1	NM_213474.1
A3G0891	12.83	16.06	HRAS	GTPase HRas	NM_200258.1
A5G0818	12.83	7.12	MCCC2	Methylcrotonoyl-CoA carboxylase beta chain, mitochondrial	NM_212927.1
A23G0093	12.83	9.23	STXBP3	Syntaxin-binding protein 3	NM_213471.1
B1G0611	12.82	3.21	DCTD	Deoxycytidylate deaminase	NM_001017639.1
A3G0322	12.82	7.13	ASF1BA	Histone chaperone asf1b-A	NM_207063.1
B3G0514	12.82	3.41	DOCK7	Dedicator of cytokinesis protein 7	XM_068219443.1
B18G0063	12.82	17.25	PLD3	5'-3' exonuclease PLD3	XM_003200490.6
B8G0124	12.81	4.80	GKAP1	G kinase-anchoring protein 1	NM_213072.2
B15G0260	12.81	20.31	SLC1A5	Neutral amino acid transporter B(0)	NM_001190755.1
B5G1079	12.81	20.46	GDA	Guanine deaminase	NM_001020674.1
A21G0303	12.81	5.18	FAM216A	Protein FAM216A	NM_001386287.1
B1G0006	12.81	19.27	HIKESHI	Protein Hikeshi	
A21G0719	12.81	10.31	SLC25A14	Brain mitochondrial carrier protein 1	XM_017352756.3
B14G0519	12.81	32.86	P33MONOX	Putative monooxygenase p33MONOX	XM_068213297.1
B9G0583	12.80	4.32	CMSS1	Protein CMSS1	NM_001006048.2
B24G0632	12.80	3.95	TFAP2A	Transcription factor AP-2-alpha	NM_001319158.1
A8G0938	12.80	15.02	QARS1	Glutamine--tRNA ligase	NM_001423662.1
B12G0112	12.80	9.06	GLRX3	Glutaredoxin 3	NM_001005950.1
B19G0608	12.79	5.73	PSMA2	Proteasome subunit alpha type-2	NM_001128674.1
B19G0210	12.79	8.06	SF3B4	Splicing factor 3B subunit 4	NM_153661.4
B4G0924	12.79	25.98	PACSIN2	Protein kinase C and casein kinase substrate in neurons protein 2	NM_207069.2
A7G1225	12.79	11.37	SF3B2	Splicing factor 3B subunit 2	NM_001105277.3
B5G0009	12.79	17.63	HOOK3	Protein Hook homolog 3	XM_017356208.3
B5G0282	12.78	23.18	SLC25A11	malate carrier protein	NM_001002099.1
A2G0141	12.78	15.86	SEC22BA	Vesicle-trafficking protein SEC22b-A	NM_213384.1
B22G0630	12.78	2.80	HSH2D	Hematopoietic SH2 domain-containing protein homolog	
B16G0637	12.78	10.54	ZNF576	Zinc finger protein 576	NM_001114425.2
B15G0609	12.78	1.43	G5714_015224	Ly6 domain-containing protein	NM_001386226.1
B24G0532	12.78	17.49	A0A673G6R0	A0A673G6R0_9TELE Si:dkey-118j18.2	XM_002666623.6
A21G0033	12.77	19.40	ADD1	Alpha-adducin	XM_021468846.2
B6G0308	12.77	12.09	GCA	Grancalcin	NM_001005585.1
B21G0097	12.77	12.65	NDUFA2	NADH dehydrogenase [ubiquinone] 1 alpha subcomplex subunit 2	NM_001024420.1
A8G0901	12.76	19.24	GOLM1	Golgi membrane protein 1	
B25G0884	12.76	4.46	A0A672LGL4	A0A672LGL4_SINGR Ubiquitin-like domain-containing protein	
B3G1254	12.76	6.59	IFNA3	Interferon a3	NM_207640.2
B14G0765	12.76	40.58	RGCC	Regulator of cell cycle RGCC	NM_001386477.1

B15G0480	12.76	4.77	PHYKPL	5-phosphohydroxy-L-lysine phospho-lyase	NM_001037557.2
A9G0385	12.75	7.11	RNASEH2B	Ribonuclease H2 subunit B	NM_200271.1
A22G0368	12.75	18.55	RABGAP1	Rab GTPase-activating protein 1	XM_021469587.2
A24G0689	12.75	8.46	TMX3	Protein disulfide-isomerase TMX3	NM_001045561.1
B21G0180	12.75	5.10	MRPS17	28S ribosomal protein S17, mitochondrial	NM_001020559.2
A21G0894	12.75	13.35	TXNDC15	Thioredoxin domain-containing protein 15	
B16G0198	12.74	5.53	RBM42	RNA-binding protein 42	NM_001003850.1
A12G0335	12.74	14.09	AUTS2	Autism susceptibility gene 2 protein	XM_003199613.6
A13G0519	12.74	18.67	DNA2	nuclease DNA2	XM_068224906.1
B18G0343	12.74	6.58	RASGRP1	RAS guanyl-releasing protein 1	XM_005173658.5
A8G0167	12.74	4.35	TSR2	Pre-rRNA-processing protein TSR2 homolog	NM_173284.3
A14G0463	12.74	15.58	CTNNA1	Catenin alpha-1	NM_131456.2
A25G0577	12.73	4.78	FTH1-B	Ferritin heavy chain B	NM_001126392.1
A6G0255	12.73	11.14	STK24	threonine-protein kinase 24	XM_005165833.5
B23G0732	12.73	23.36	LIMA1	LIM domain and actin-binding protein 1	NM_131664.1
A4G0504	12.72	15.32	RBM17	Splicing factor 45	XM_005164814.5
B20G0653	12.72	8.02	VTA1	Vacuolar protein sorting-associated protein VTA1 homolog	XM_005160655.4
B3G0265	12.72	9.19	PCTP	Phosphatidylcholine transfer protein	
A15G0405	12.72	14.13	RPA1	Replication protein A 70 kDa DNA-binding subunit	NM_199811.2
B4G0328	12.72	32.12	MGST1	Microsomal glutathione S-transferase 1	NM_001002215.2
B13G0655	12.72	7.75	WDR27	WD repeat-containing protein 27	NM_001161599.1
A22G0120	12.71	9.90	CHERP	Calcium homeostasis endoplasmic reticulum protein	XM_068216257.1
B3G1649	12.71	1.83	ROHU_014069	A0A498MIS4_LABRO Uncharacterized protein	
A16G0320	12.71	15.23	DECR1	2,4-dienoyl-CoA reductase [(3E)-enoyl-CoA-producing], mitochondrial	NM_001002444.2
B1G1159	12.71	2.18	TRIP10	Cdc42-interacting protein 4 homolog	NM_200224.1
A8G0413	12.70	9.92	TNFRSF14	Tumor necrosis factor receptor superfamily member 14	XM_021478552.2
B18G0522	12.70	12.00	SF3B3	Splicing factor 3B subunit 3	NM_213503.1
A10G0486	12.70	19.01	EHD1	EH domain-containing protein 1	NM_001004578.1
B7G1062	12.70	11.72	EML3	Echinoderm microtubule-associated protein-like 3	XM_005172201.5
A11G0691	12.70	6.84	CDH26	Cadherin-like protein 26	NM_201158.1
B6G0062	12.69	38.38	CHCHD6A	MICOS complex subunit mic25a	
A25G0821	12.69	10.38	PUS7	Pseudouridylate synthase 7 homolog	XM_068217353.1
B1G0155	12.69	15.43	HAPSTR1	HUWE1-associated protein modifying stress responses	NM_199602.2
A14G0012	12.69	6.89	TMEM128	Transmembrane protein 128	
A8G0976	12.69	1.75	YTX2	Transposon TX1 uncharacterized 149 kDa protein	
B11G0168	12.68	0.09	DDA1	DET1- and DDB1-associated protein 1	NM_001017588.2
B6G0574	12.68	12.37	NASP	Nuclear autoantigenic sperm protein	NM_199782.2

B16G0461	12.68	22.69	MASP2	Mannan-binding lectin serine protease 2	NR_027750.1
B22G0700	12.67	3.62	TJP3	Tight junction protein ZO-3	NM_001328340.1
A18G0457	12.67	6.74	MORF4L1	Mortality factor 4-like protein 1	NM_001002604.2
B14G0079	12.67	0.50	MSXA	Homeobox protein MSH-A	NM_001030269.1
B14G0086	12.67	40.61	HNRNPA0	Heterogeneous nuclear ribonucleoprotein A0	NM_212645.2
B20G0677	12.66	17.50	COQ8A	Atypical kinase COQ8A, mitochondrial	NM_001002728.2
A6G0809	12.66	0.68	FAM3C	Protein FAM3C	NM_001366242.1
A9G0189	12.66	15.55	ORMDL1	ORM1-like protein 1	NM_200093.1
B3G1072	12.66	14.77	NME3	Nucleoside diphosphate kinase 3	NM_001362268.1
A5G1076	12.66	17.11	PISD	Phosphatidylserine decarboxylase proenzyme, mitochondrial	XM_017356088.2
B21G0346	12.66	6.59	TTC1	Tetratricopeptide repeat protein 1	NM_001007382.2
A2G0563	12.66	6.67	ASPH	asparaginyl beta-hydroxylase	XM_005163382.5
B20G0546	12.66	52.79	SESN1	Sestrin-1	NM_001002660.1
A5G1053	12.66	11.15	SLC37A2	Glucose-6-phosphate exchanger SLC37A2	NM_213014.2
A8G0538	12.65	20.00	PRKAR2A	cAMP-dependent protein kinase type II-alpha regulatory subunit	NM_001077370.2
B5G0981	12.65	17.11	USF1	Upstream stimulatory factor 1	NM_213194.1
A2G1009	12.65	1.67	CCL20	C-C motif chemokine 20	
A12G0068	12.64	9.47	COMTD1	Catechol O-methyltransferase domain-containing protein 1	
B14G0509	12.64	13.64	SMU1	WD40 repeat-containing protein SMU1	NM_200199.1
A11G0162	12.64	10.18	KRT42	Keratin, type I cytoskeletal 42	XM_694917.8
B16G0820	12.64	15.15	JOSD2	Josephin-2	NM_200151.2
A8G0504	12.64	9.42	STK38	threonine-protein kinase 38	NM_213456.1
A19G0242	12.64	10.28	TXLNA	Alpha-taxilin	NM_001099230.1
A14G0082	12.62	12.29	NR3C1	Glucocorticoid receptor	NM_001020711.3
A5G0333	12.62	11.07	NONO	Non-POU domain-containing octamer-binding protein	XM_005165105.5
B9G0136	12.62	28.89	RDH7	Retinol dehydrogenase 7	NM_199609.1
A12G0521	12.61	13.59	USP42	Ubiquitin carboxyl-terminal hydrolase 42	XM_005169463.4
B6G0361	12.61	20.50	PPP1R12C	Protein phosphatase 1 regulatory subunit 12C	XM_005166225.5
B14G0791	12.61	8.47	GLOD5	Glyoxalase domain-containing protein 5	
A2G0910	12.60	15.29	ZNF622	Cytoplasmic 60S subunit biogenesis factor ZNF622	XM_017352038.3
B16G0259	12.60	22.16	PYCARD	Apoptosis-associated speck-like protein containing a CARD	
A17G0914	12.60	19.35	KLHDC1	Kelch domain-containing protein 1	
A21G0661	12.60	17.18	P33MONOX	Putative monooxygenase p33MONOX	NM_001039994.3
A7G1170	12.60	8.74	MESD	LRP chaperone MESD	XM_005166364.5
B17G0233	12.60	9.95	GEMIN2	Gem-associated protein 2	NM_001017608.2
B8G0816	12.60	14.20	JAK2	Tyrosine-protein kinase JAK2	XM_002663087.7
A20G0820	12.59	18.29	LGALS8	Galectin-8	XM_003200667.6
B5G0221	12.59	466.86	AK1	Adenylate kinase isoenzyme 1	XM_009302056.4
B23G0466	12.59	21.04	SDHB	Succinate dehydrogenase [ubiquinone] iron-sulfur subunit, mitochondrial	
B20G0411	12.58	17.72	CHURC1	Protein Churchill	NM_001007380.1
B14G0266	12.58	78.78	CLIC2	Chloride intracellular channel protein 2	NM_001002561.2

A13G0084	12.57	18.64			XM_687830.8
A14G0126	12.57	6.09	LMAN2	Vesicular integral-membrane protein VIP36	XM_005173172.5
B1G0779	12.57	8.31	CDADC1	Cytidine and dCMP deaminase domain-containing protein 1	XM_005170988.5
A18G0303	12.57	6.13	ITPKC	Inositol-trisphosphate 3-kinase C	NM_001305471.1
A19G0309	12.57	33.64	AZIN1	Antizyme inhibitor 1	NM_001007159.2
A21G0260	12.57	17.32	SLC31A2	Probable low affinity copper uptake protein 2	
A23G0677	12.56	2.23	HOXC13A	Homeobox protein Hox-C13a	NM_131543.1
A17G0317	12.56	13.27	IVD	Isovaleryl-CoA dehydrogenase, mitochondrial	NM_201491.1
B21G0783	12.56	6.43			
B19G0699	12.56	11.53	ABCF1	ATP-binding cassette sub-family F member 1	NM_213186.1
B10G0619	12.56	8.93	SERPINH1	Serpin H1	NM_001110374.2
B5G0596	12.55	6.94	LHFPL2A	LHFPL tetraspan subfamily member 2a protein	XM_021476148.2
A6G0862	12.55	14.73	VAPB	Vesicle-associated membrane protein-associated protein B	XM_021476783.2
A7G1001	12.55	5.70	OR131-2	Odorant receptor 131-2	XM_005172217.5
B12G0429	12.55	11.94	MRTFA	Myocardin-related transcription factor A	XM_068224603.1
B3G0089	12.55	8.43	STXB	Stonustoxin subunit beta	XM_021477861.2
A17G0704	12.55	13.02	FBXO34	F-box only protein 34	NM_001305546.1
A19G0232	12.55	9.69	SOX4	Transcription factor SOX-4	NM_213122.2
B2G0216	12.55	13.54	CUL3B	Cullin-3-B	NM_199691.2
B7G0823	12.54	2.08	TNK2	Activated CDC42 kinase 1	NM_001080017.2
B14G0007	12.54	6.14	STX18	Syntaxin-18	NM_001024425.1
B2G0191	12.54	9.89	A0A671R6K1	A0A671R6K1_9TELE IG domain-containing protein	
A19G0913	12.54	8.39	TNFAIP8L2A	Tumor necrosis factor, alpha-induced protein 8-like protein 2 A	NM_001045301.1
A3G0485	12.54	7.55	GNA12	Guanine nucleotide-binding protein subunit alpha-12	NM_001013277.2
B8G0994	12.53	6.05	GLE1	mRNA export factor GLE1	XM_005166747.5
A19G0226	12.53	6.16	MED10	Mediator of RNA polymerase II transcription subunit 10	NM_001017699.1
A25G0574	12.53	6.54	CALD1	Caldesmon	XM_003201460.6
A16G0353	12.53	10.99	ZDHHC18A	Palmitoyltransferase ZDHHC18-A	NM_001077563.1
B11G0527	12.52	2.69	CFLAR	CASP8 and FADD-like apoptosis regulator	NM_001079988.1
A3G0795	12.52	15.43	IFI35	Interferon-induced 35 kDa protein homolog	NM_001386657.1
A11G0389	12.52	15.27	MON1B	Vacuolar fusion protein MON1 homolog B	XM_017358331.3
A5G0102	12.52	8.90	PXN	Paxillin	NM_201588.2
A14G0537	12.51	11.41	CISD1	CDGSH iron-sulfur domain-containing protein 1	NM_001014305.1
A9G0180	12.51	11.86	STAT4	Signal transducer and activator of transcription 4	XM_009304880.4
A10G0096	12.51	9.30	PTPN11	Tyrosine-protein phosphatase non-receptor type 11	NM_199846.1
B13G0398	12.51	17.10	VPS26A	Vacuolar protein sorting-associated protein 26A	XM_068224923.1
A23G0417	12.50	6.07	PLEKHN1	Probable pleckstrin homology domain-containing family N member 1	XM_692642.9

A3G0625	12.50	12.53	PSMD11A	26S proteasome non-ATPase regulatory subunit 11A	NM_001166238.2
B9G0648	12.50	5.07	ANKZF1	Ankyrin repeat and zinc finger domain-containing protein 1	XM_068223557.1
B14G0460	12.50	28.63	RMND5B	E3 ubiquitin-protein transferase RMND5B	NM_200774.1
A20G0828	12.50	9.84	SNW1	SNW domain-containing protein 1	NM_001002864.1
B19G0209	12.50	1.48	RAB25	Ras-related protein Rab-25	NM_001008641.2
A9G0750	12.49	6.52	INSIG2	Insulin-induced gene 2 protein	NM_212804.1
B21G0787	12.49	5.47	MRPS2	28S ribosomal protein S2, mitochondrial	
A15G0570	12.49	12.43	ATF5	Cyclic AMP-dependent transcription factor ATF-5	XM_021466358.2
A15G0538	12.49	4.94	ALCAMA	CD166 antigen homolog A	NM_212634.3
B6G0785	12.48	13.46	ARL8BA	ADP-ribosylation factor-like protein 8B-A	NM_205566.1
B8G0858	12.47	10.13	PLEKHA2	Pleckstrin homology domain-containing family A member 2	NM_001128363.1
B9G0220	12.47	7.72	ASNSD1	Asparagine synthetase domain-containing protein 1	NM_200095.2
A25G0287	12.47	3.18	RASSF10	Ras association domain-containing protein 10	XM_005174361.5
B21G0909	12.47	13.10	WDR91	WD repeat-containing protein 91	XM_068215824.1
B5G1257	12.47	7.53	GLTP	Glycolipid transfer protein	NM_001099988.1
B8G0991	12.47	16.57	SLC25A25A	Calcium-binding mitochondrial carrier protein SCaMC-2-A	NM_213257.1
A4G1021	12.47	14.11	PARP12	Protein mono-ADP-ribosyltransferase PARP12	NM_001327797.2
A1G0049	12.47	39.80	NLRC3	NLR family CARD domain-containing protein 3	NM_001328438.1
A5G0331	12.46	7.46	AIFM1	Apoptosis-inducing factor 1, mitochondrial	XM_005165127.4
A7G0278	12.46	9.66	SIRT3	NAD-dependent protein deacetylase sirtuin-3, mitochondrial	NM_001080174.1
B22G0199	12.46	7.65	MCM2	DNA replication licensing factor mcm2	NM_173257.2
A11G0091	12.45	10.34	GIMAP8	GTPase IMAP family member 8	NM_001328387.1
B2G1259	12.45	8.57	DUSP22B	Dual specificity protein phosphatase 22-B	NM_001017742.2
A5G1074	12.45	4.60	A0A672LVR7	A0A672LVR7_SINGR Zgc:193711	NM_001128753.1
A10G0864	12.45	5.31	SLC38A9	Sodium-coupled neutral amino acid transporter 9	NM_001079999.1
A3G0737	12.44	22.84	DDX42	ATP-dependent RNA helicase DDX42	NM_001037805.2
A2G0171	12.44	5.00	PPM1L	Protein phosphatase 1L	NM_001077600.1
A14G0211	12.44	12.14	MAP7	Ensconsin	XM_001922471.7
A11G0557	12.44	12.66	G5714_011669	A0A7J6CL58_9TELE Uncharacterized protein	XM_021479620.2
A4G1005	12.44	14.85	RPAP3	RNA polymerase II-associated protein 3	NM_200204.2
A7G0911	12.44	19.07	LRP10	Low-density lipoprotein receptor-related protein 10	XM_683767.10
B20G0179	12.44	11.65	PSMC6	26S proteasome regulatory subunit 10B	NM_001003832.1
B11G0547	12.43	7.78	SIPA1L2	Signal-induced proliferation-associated 1-like protein 2	XM_009306191.4
B20G0124	12.43	2.88	BCL11B	leukemia 11B	XM_002667860.7
B8G0518	12.43	7.07	TMEM115	Transmembrane protein 115	NM_001033745.1

B15G0307	12.43	5.62	RRP8	Ribosomal RNA-processing protein 8	XM_021472910.2
A16G1043	12.43	20.58	CERS2	Ceramide synthase 2	XM_688576.8
B11G0341	12.43	14.85	THOC7	THO complex subunit 7 homolog	NM_001080582.1
B12G0362	12.42	12.94	MTA3	Metastasis-associated protein MTA3	XM_068224467.1
A11G0708	12.42	14.09	TTLL7	Tubulin polyglutamylase TTLL7	
B14G0660	12.42	10.06	PDGFRB	Platelet-derived growth factor receptor beta	XM_021481027.2
A7G1039	12.41	16.34	IL13RA1	Interleukin-13 receptor subunit alpha-1	
A22G0240	12.41	13.96	LSM7	U6 snRNA-associated Sm-like protein LSm7	NM_001048006.1
A17G0719	12.41	19.05	SRP54	Signal recognition particle 54 kDa protein	NM_200988.2
B5G1146	12.40	19.98	RNF128	E3 ubiquitin-protein ligase RNF128	NM_212615.1
A13G0763	12.40	9.90	LOC113057249	A0A6P6L6X8_CARAU zinc finger protein 135-like	NM_001082827.2
A9G0062	12.40	4.23	IL1R1	Interleukin-1 receptor type 1	
A19G0494	12.40	7.60	RPRD1A	Regulation of nuclear pre-mRNA domain-containing protein 1A	XM_068215337.1
B24G0564	12.40	14.26	STAU2	Double-stranded RNA-binding protein Staufen homolog 2	XM_021470116.2
A22G0012	12.39	9.54	FBLIM1	Filamin-binding LIM protein 1	NM_001077303.1
B21G0666	12.39	3.93	PUS1	Pseudouridylate synthase 1 homolog	NM_001008603.2
B18G0078	12.39	11.60	CSTF3	Cleavage stimulation factor subunit 3	NM_213053.2
A2G1129	12.39	6.85	TWSG1A	Twisted gastrulation protein homolog 1-A	NM_131797.3
B3G0702	12.39	11.58	RBBP6	E3 ubiquitin-protein ligase RBBP6	NM_001193674.1
B1G0812	12.39	2.82	MSXB	Homeobox protein MSH-B	NM_131260.1
A14G0256	12.38	23.53	CUL4B	Cullin-4B	NM_001122844.1
A1G0284	12.38	39.18	PPP3R1	Calcineurin subunit B type 1	NM_001004553.1
A1G0787	12.38	4.47	ATIC	Bifunctional purine biosynthesis protein ATIC	NM_001082796.2
B23G0578	12.38	39.58	TARDBP	TAR DNA-binding protein 43	XM_021469761.2
A4G0138	12.38	0.00			
A21G0635	12.38	2.25	SLC6A7	Sodium-dependent proline transporter	NM_001080580.1
B13G0686	12.38	8.38	DESI2	Deubiquitinase DESI2	XM_009307225.4
B12G0189	12.37	8.64	CYTH1	Cytohesin-1	XM_005169466.5
B24G0468	12.37	4.92	MIX23	Protein MIX23	NM_200807.1
A4G0834	12.37	3.92	SAMM50B	Sorting and assembly machinery component 50 homolog B	NM_001007343.2
A4G0609	12.37	7.90	CDKN1B	Cyclin-dependent kinase inhibitor 1B	NM_001013268.1
B11G0810	12.37	7.60			NM_212662.2
B2G0620	12.37	18.35	UBTF	Nucleolar transcription factor 1	XM_068223429.1
B5G1128	12.37	17.73	ROHU_016493	A0A498NK44_LABRO Serine arginine repetitive matrix 1-like protein	NM_001002300.1
A13G0143	12.36	11.51	PRKG1	cGMP-dependent protein kinase 1	NM_001431065.1
A7G0347	12.36	28.93	PAIP2B	Polyadenylate-binding protein-interacting protein 2B	NM_200766.1
A15G0567	12.36	11.62	ARCN1	Coatomer subunit delta	NM_201993.1

B11G0264	12.36	9.72	K1C1	Keratin, type I cytoskeletal 50 kDa	NM_200467.1
A22G0380	12.35	2.56	TJP3	Tight junction protein ZO-3	NM_001328340.1
A24G0431	12.35	10.54	ACAA1B	3-ketoacyl-CoA thiolase B, peroxisomal	NM_001002207.1
B23G0340	12.35	5.81	EMP3	Epithelial membrane protein 3	NM_001025508.1
B3G1238	12.34	1.99	RAPGEFL1	Rap guanine nucleotide exchange factor-like 1	XM_001920985.7
B22G0548	12.34	15.33	ATP6AP2	Renin receptor	NM_213023.2
A14G0611	12.34	6.72	SRP72	Signal recognition particle subunit SRP72	XM_017359078.3
B2G0047	12.34	34.61	MBD3	Methyl-CpG-binding domain protein 3	XM_005168451.5
A7G0617	12.34	7.22	CNEP1R1	Nuclear envelope phosphatase-regulatory subunit 1	NM_001017874.1
A25G0789	12.33	24.04	CD44	CD44 antigen	
B14G0797	12.33	35.22	GATD3	Glutamine amidotransferase-like class 1 domain-containing protein 3, mitochondrial	NM_001386233.1
A16G0679	12.33	14.55	COPS7A	COP9 signalosome complex subunit 7a	NM_001305596.1
A12G0488	12.32	15.42	AQP8	Aquaporin-8	NM_001004661.1
B24G0006	12.32	45.25	SSR1	Translocon-associated protein subunit alpha	NM_201327.1
A10G0072	12.32	8.73	ASH2L	Ash2 histone methyltransferase complex subunit ASH2	NM_001110105.2
A5G1219	12.32	12.22	WBP1	WW domain-binding protein 1	NM_205634.1
A3G1147	12.32	7.99	BAIAP2L1	Brain-specific angiogenesis inhibitor 1-associated protein 2-like protein 1	NM_001017830.1
B1G0873	12.32	3.83	SNRPB2	U2 small nuclear ribonucleoprotein B"	NM_001256190.1
B18G0717	12.32	9.25	CALUB	Calumenin-B	NM_201082.1
A14G0168	12.31	7.93	CNOT7	CCR4-NOT transcription complex subunit 7	NM_001077255.1
A2G0163	12.31	5.53	CZIB	CXXC motif containing zinc binding protein	NM_001128685.2
A9G0431	12.30	23.37	ARGLU1A	Arginine and glutamate-rich protein 1-A	NM_213216.2
B2G0861	12.30	9.80	THAP4	Peroxynitrite isomerase THAP4	
B8G0337	12.30	26.98	CLEC4E	C-type lectin domain family 4 member E	XM_068223181.1
B22G0452	12.29	21.95	NLRP12	NACHT, LRR and PYD domains-containing protein 12	XM_068216562.1
B25G0228	12.29	13.43	SPTY2D1	Protein SPT2 homolog	XM_005170676.5
A22G0317	12.29	1.96	HSH2D	Hematopoietic SH2 domain-containing protein homolog	NM_001017541.2
B11G0675	12.29	10.14	MIIP	Migration and invasion-inhibitory protein	NM_173258.1
A7G0827	12.29	11.14	PLSCR1	Phospholipid scramblase 1	NM_212866.1
A2G0372	12.28	17.69	PPP1R7	Protein phosphatase 1 regulatory subunit 7	NM_001037386.2
B10G0625	12.28	21.86	ACAT1	Acetyl-CoA acetyltransferase, mitochondrial	NM_001003746.1
B16G0156	12.28	15.49	RPZ	Protein rapunzel	NM_001017910.1
B7G0416	12.28	11.12	HSD17B12B	Very-long-chain 3-oxoacyl-CoA reductase-B	NM_199613.1
A16G0131	12.28	8.96	RAB11A	Ras-related protein Rab-11A	NM_200123.1
B10G0403	12.28	0.65	LOC113109832	A0A6P6Q7H7_CARAU transcription cofactor vestigial-like protein 1	
A3G0920	12.27	10.84	NATD1	Protein NATD1	

A11G0772	12.27	10.33	ZNF831	Zinc finger protein 831	NM_214690.1
A2G0040	12.27	7.20	RIPK1	threonine-protein kinase 1	NM_001043350.1
B23G0798	12.27	14.57	SIKE1	Suppressor of IKBKE 1	XR_011008508.1
A5G0834	12.27	27.78	SMARCA2	Probable global transcription activator SNF2L2	NM_001044775.2
A2G0903	12.27	3.33	CCL20	C-C motif chemokine 20	
B5G0809	12.27	15.46	NFKBIE	NF-kappa-B inhibitor epsilon	NM_001128795.1
B6G0336	12.27	4.87	G5714_006841	A0A7J6CXH4_9TELE Uncharacterized protein	XM_001344897.6
A8G0613	12.27	6.91	DERL2	Derlin-2	NM_200903.2
B2G0363	12.27	30.15	COPB2	Coatomer subunit beta'	NM_001001940.1
A7G0078	12.26	6.62	NLRP1	NACHT, LRR and PYD domains-containing protein 1 homolog	
A4G0856	12.26	16.68	LOC107743829	A0A673K627_9TELE Uncharacterized LOC107743829	
B12G0527	12.26	3.47	ANKRD22	Ankyrin repeat domain-containing protein 22	NM_200274.1
B1G0794	12.26	7.26	CRYZL1	Quinone oxidoreductase-like protein 1	NM_001002633.1
B19G0251	12.26	5.95	CRABP2	Cellular retinoic acid-binding protein 2	NM_001320394.1
B2G1211	12.26	5.54	MUL1A	Mitochondrial ubiquitin ligase activator of nfkb 1-A	XM_005171126.5
B15G0384	12.25	7.38	ZBTB16A	Zinc finger and BTB domain-containing protein 16-A	XM_693182.7
B12G0321	12.25	5.23	SKAP1	Src kinase-associated phosphoprotein 1	NM_001080011.2
A11G0388	12.25	3.19	BRPF3	Bromodomain and PHD finger-containing protein 3	XM_068224340.1
B15G0061	12.25	13.71	POR	NADPH--cytochrome P450 reductase	NM_001320439.1
B14G0377	12.24	3.31	LINGO2	Leucine-rich repeat and immunoglobulin-like domain-containing nogo receptor-interacting protein 2	XM_021481255.2
A21G0814	12.24	11.35	BRCC3	Lys-63-specific deubiquitinase BRCC36	NM_001423851.1
B9G0546	12.24	8.79	DBR1	Lariat debranching enzyme	NM_199653.1
A1G0242	12.24	12.28	ELMOD2	ELMO domain-containing protein 2	NM_001204132.1
A4G0209	12.23	4.22	A0A672NKX7	A0A672NKX7_SINGR Uncharacterized protein	XM_003201487.6
A21G0536	12.22	18.72			
A23G0644	12.22	7.97	TMED4	Transmembrane emp24 domain-containing protein 4	NM_001002134.2
B1G0752	12.22	22.18	LOC107676841	A0A671NN24_9TELE Uncharacterized LOC107676841	NM_001145709.1
A9G0147	12.22	7.21	UBE2E3	Ubiquitin-conjugating enzyme E2 E3	NM_200921.3
A5G0349	12.22	10.42	UPRT	Uracil phosphoribosyltransferase homolog	NM_207091.2
B6G0197	12.22	3.38			NM_001020695.2
B13G0600	12.21	17.60	RDH14	Retinol dehydrogenase 14	NM_001346275.1
A23G0643	12.21	20.75	PLPBP	Pyridoxal phosphate homeostasis protein	NM_001126409.2
B4G0113	12.21	4.77	WASL	Actin nucleation-promoting factor WASL	NM_001083006.1

B13G0473	12.20	8.40	CSGALNACT2	Chondroitin sulfate N-acetylgalactosaminyltransferase 2	NM_131272.2
A13G0148	12.20	32.49	HPS1	BLOC-3 complex member HPS1	XM_005156494.5
A8G0705	12.20	5.14	GPX8	Probable glutathione peroxidase 8	NM_200222.1
B17G0281	12.20	0.71	G5714_016855	A0A7J6C605_9TELE Uncharacterized protein	
A6G0001	12.20	1.56	SOWAHC	Ankyrin repeat domain-containing protein SOWAHC	XM_009299786.4
B14G0583	12.20	7.44	GNPDA2	Glucosamine-6-phosphate isomerase 2	XM_679055.8
B3G1131	12.20	19.93	CDIPT	CDP-diacylglycerol--inositol 3-phosphatidyltransferase	NM_207088.1
B12G0359	12.20	12.30	PPM1B	Protein phosphatase 1B	XM_005156264.5
A14G0300	12.20	8.01	MRPL18	39S ribosomal protein L18, mitochondrial	XM_005157321.4
B21G0058	12.19	12.81	UBE2B	Ubiquitin-conjugating enzyme E2 B	NM_001002747.1
B22G0866	12.19	4.94	B3GALT2	Beta-1,3-galactosyltransferase 2	NM_001128796.1
A15G0327	12.19	6.04	KSR1	Kinase suppressor of Ras 1	XM_021466453.2
A6G0267	12.19	18.00	CASP8	Caspase-8	NM_131510.2
A8G0624	12.18	14.37	CREB3L3	Cyclic AMP-responsive element-binding protein 3-like protein 3	XM_005167154.4
A8G0630	12.18	15.63	RIMOC1	RAB7A-interacting MON1-CCZ1 complex subunit 1	NM_001128229.1
B15G0574	12.18	15.45	PRPF8	Pre-mRNA-processing-splicing factor 8	NM_200976.2
A7G0710	12.18	6.40	LIN7C	Protein lin-7 homolog C	NM_001362514.1
A18G0080	12.18	13.64	AP2M1B	AP-2 complex subunit mu-B	NM_201026.2
A3G0899	12.18	14.25	EFTUD2	116 kDa U5 small nuclear ribonucleoprotein component	NM_200508.2
B11G0174	12.17	2.97	TAX1BP3	Tax1-binding protein 3	XM_005155759.5
B19G0100	12.17	18.56	TAP2	Antigen peptide transporter 2	NM_001006594.1
A23G0304	12.17	12.90	SRGAP3	SLIT-ROBO Rho GTPase-activating protein 3	XM_684637.8
A14G0564	12.17	12.82	AUP1	Lipid droplet-regulating VLDL assembly factor AUP1	XM_005173083.5
A20G0308	12.17	3.82	PTRHD1	Putative peptidyl-tRNA hydrolase PTRHD1	
B5G0800	12.17	17.10	U2SURP	U2 snRNP-associated SURP motif-containing protein	NM_001089532.2
B17G0369	12.17	6.81	PLEKHA1	Pleckstrin homology domain-containing family A member 1	NM_213436.1
A20G0122	12.17	4.39	PREP	Prolyl endopeptidase	NM_001020753.1
B7G0628	12.17	7.00	SLC12A4	Solute carrier family 12 member 4	XM_686199.8
B9G0533	12.16	5.01	EFNB2A	Ephrin-B2a	NM_131023.1
A8G0695	12.16	1.80	CUSTOS	Protein CUSTOS	
B20G0471	12.16	1.81	RHOV	Rho-related GTP-binding protein RhoV	NM_001012250.2
B6G0788	12.16	10.36	TCTA	T-cell leukemia translocation-altered gene protein homolog	NM_001002585.2
A7G0306	12.16	11.29			
A2G1055	12.16	7.00	SLAMF9	SLAM family member 9	XM_068213188.1
A14G0659	12.16	6.54	TBC1D10B	TBC1 domain family member 10B	XM_005173146.5
B15G0360	12.15	15.34	VMP1	Vacuole membrane protein 1	NM_207060.1
B18G0716	12.15	4.31	UTP15	U3 small nucleolar RNA-associated protein 15 homolog	XM_005158982.5

A21G0505	12.15	4.94	MRPS12	28S ribosomal protein S12, mitochondrial	NM_001386273.1
B16G0933	12.15	4.39	EMG1	Ribosomal RNA small subunit methyltransferase NEP1	NM_001040337.2
B22G0883	12.15	19.16	XPNPEP1	Xaa-Pro aminopeptidase 1	XM_021469308.1
A20G0724	12.15	1.35	G5714_019433	A0A7J6BWD5_9TELE Uncharacterized protein	
B2G0417	12.14	7.41	MYO9B	Unconventional myosin-IXb	XM_017351952.2
A8G0832	12.14	7.24	PPP1R3C	Protein phosphatase 1 regulatory subunit 3C	NM_213180.1
A10G0628	12.14	10.66	SFRP1	Secreted frizzled-related protein 1	
B6G0903	12.14	22.98	PSMF1	Proteasome inhibitor PI31 subunit	NM_001002547.2
B4G0649	12.13	9.00	NUP50	Nuclear pore complex protein Nup50	XM_068219518.1
B3G0484	12.13	9.45	TRIP10	Cdc42-interacting protein 4 homolog	XM_017354074.3
B19G0303	12.12	5.44	OLAH	S-acyl fatty acid synthase thioesterase, medium chain	NM_001039932.2
B17G0138	12.12	12.53	STX11	Syntaxin-11	NM_212910.1
A2G1004	12.12	12.19	EIF4E2	Eukaryotic translation initiation factor 4E type 2	XM_021480199.2
B9G0617	12.11	7.28	SPOPLA	Speckle-type POZ protein-like A	NM_001013447.1
A5G0118	12.11	2.80	SLC1A3	Excitatory amino acid transporter 1	XM_009301170.3
A16G0794	12.11	7.94	NGFR	Tumor necrosis factor receptor superfamily member 16	XM_695893.9
B12G0432	12.11	17.16	XRCC6	X-ray repair cross-complementing protein 5	XM_068224528.1
B3G0416	12.11	13.63	DNM2	Dynamin-2	XM_021470874.2
A13G0058	12.10	13.40	LOC107676303	A0A671QFL9_9TELE Microtubule-associated protein 10-like	
A16G0274	12.10	3.59	TRMT11	tRNA (guanine(10)-N2)-methyltransferase homolog	NM_200216.3
A14G0410	12.10	4.13	SETD7	Histone-lysine N-methyltransferase SETD7	NM_001002456.1
B10G0143	12.10	7.47	SARDH	Sarcosine dehydrogenase, mitochondrial	NM_001326686.1
B5G0471	12.10	14.35	AP2B1	AP-1 complex subunit beta-1	NM_199919.2
A11G0415	12.10	6.59	PTPDC1	Protein tyrosine phosphatase domain-containing protein 1	NM_001080005.1
B22G1087	12.09	12.25	PACS2	Phosphofurin acidic cluster sorting protein 2	XM_009296534.4
B15G0577	12.09	12.17	SLC43A2	Large neutral amino acids transporter small subunit 4	NM_001008585.1
A17G0743	12.09	3.70	KRTCAP3	Keratinocyte-associated protein 3	XM_068214526.1
B22G0714	12.08	14.74	GATAD2A	Transcriptional repressor p66-alpha	XM_005170445.5
A8G0184	12.08	11.10	PTPN18	Tyrosine-protein phosphatase non-receptor type 18	NM_001013452.2
B1G0988	12.08	9.11			NM_001111238.2
A22G0644	12.08	12.01	LOC113065107	A0A6P6M692_CARAU CD276 antigen homolog isoform X1	
A19G0400	12.08	2.77	G5714_018605	A0A7J6BZG4_9TELE Uncharacterized protein	
B23G0283	12.07	31.01	TM9SF4	Transmembrane 9 superfamily member 4	NM_200510.2

B9G0568	12.07	9.41	FDX1	Adrenodoxin, mitochondrial (Fragment)	XM_001922687.5
A5G0083	12.07	35.24	TSTD1	Thiosulfate:glutathione sulfurtransferase	NM_001326449.1
B13G0623	12.07	11.39	NUSAP1	Nucleolar and spindle-associated protein 1	NM_001145584.1
B8G0521	12.07	14.78	TAF10	Transcription initiation factor TFIID subunit 10	NM_001044811.2
A21G0796	12.07	7.91	LARS1	Leucine--tRNA ligase, cytoplasmic	XM_693187.7
A22G0671	12.07	10.29	PFN2	Profilin-2	NM_201466.2
B2G1108	12.06	12.70	RGS16	Regulator of G-protein signaling 16	
B10G0536	12.06	6.96	ALG5	Dolichyl-phosphate beta-glucosyltransferase	NM_001045354.2
B2G1047	12.06	12.09	PTER	Phosphotriesterase-related protein	
A1G0052	12.06	49.06	NLRC3	Protein NLRC3	XM_068220504.1
A1G0843	12.06	15.44	PRKCBB	Protein kinase C beta type	NM_201029.1
B11G0655	12.05	9.77	AP5B1	AP-5 complex subunit beta-1	NM_201450.1
B18G0220	12.05	5.94	RTEL1	Regulator of telomere elongation helicase 1	NM_001013310.2
B1G0127	12.05	0.00	IFI44	Interferon-induced protein 44	XM_001345921.9
A25G0192	12.05	12.50	AP3S2	AP-3 complex subunit sigma-2	NM_001002539.1
A19G0758	12.04	16.42	THRAP3	Thyroid hormone receptor-associated protein 3	NM_214815.1
A3G1004	12.04	2.59	RAPGEFL1	Rap guanine nucleotide exchange factor-like 1	XM_001920985.7
B1G1160	12.04	3.38	BCAR3	Breast cancer anti-estrogen resistance protein 3 homolog	XM_002665063.7
B3G1333	12.04	0.75	PRVU	Parvalbumin, thymic CPV3	NM_182937.2
B21G0991	12.04	15.00	NOP14	Nucleolar protein 14	NM_001003831.1
A18G0320	12.04	72.65	FOSB	Protein FosB	XM_005173641.5
A20G0282	12.04	13.05	COQ8A	Atypical kinase COQ8A, mitochondrial	NM_001002728.2
A18G0527	12.03	16.34	A0A672PQ34	A0A672PQ34_SINGR Uncharacterized protein	
A14G0416	12.03	7.48	TMEM33	Transmembrane protein 33	NM_213663.1
B23G0280	12.02	7.58	POFUT1	GDP-fucose protein O-fucosyltransferase 1	NM_205718.3
A20G0397	12.02	8.94	SNX3	Sorting nexin-3	NM_001037106.2
B3G1289	12.01	12.49	ARHGAP17	Rho GTPase-activating protein 17	XM_009299322.4
B3G0585	12.01	19.90	CYP3A40	Cytochrome P450 3A40	NM_001077548.1
A1G0263	12.01	8.85	CLDND1	Claudin domain-containing protein 1	NM_001161597.1
A22G0690	12.01	11.80	PSMD1	26S proteasome non-ATPase regulatory subunit 1	NM_201184.2
B17G0462	12.01	4.29	CLN8	Protein CLN8	
A10G0356	12.01	2.54	LOC107728898	A0A673FSK3_9TELE Cytochrome c oxidase assembly factor 4 homolog, mitochondrial-like	NM_001200036.1
A3G0614	12.00	12.43	DCTN5	Dynactin subunit 5	NM_001002497.1
A21G0435	12.00	5.42	IL7R	Interleukin-7 receptor subunit alpha	NM_001113507.2
A8G0417	12.00	7.35	APEX2	DNA-(apurinic or apyrimidinic site) endonuclease 2	NM_200146.1
B23G0460	11.99	6.30	EPHA2	Ephrin type-A receptor 2	XM_005162209.5
A14G0760	11.99	9.71	ZDHHC15B	Palmitoyltransferase ZDHHC15B	NM_001077781.1
B4G0255	11.99	6.35	DCLRE1C	Protein artemis	NM_001045101.1

A4G0523	11.99	13.79	MDM2	E3 ubiquitin-protein ligase Mdm2	NM_131364.3
A3G0271	11.99	48.51	SLC44A2	Choline transporter-like protein 2	NM_198871.1
A10G0500	11.99	7.47	ZNRD2	Protein ZNRD2	NM_001006010.1
B25G0453	11.99	0.49	MX2	Interferon-induced GTP-binding protein Mx2	XM_690470.10
B13G0275	11.97	6.02	POL	Retrovirus-related Pol polyprotein from transposon 297	NM_001145694.1
B9G0033	11.97	25.70	NLRC3	Protein NLRC3	XM_068222163.1
B4G0714	11.97	1.89	A0A673H118	A0A673H118_9TELE Uncharacterized protein	XM_692772.10
A6G0121	11.97	15.99	SEC23A	Protein transport protein Sec23A	XM_017356444.3
A6G0761	11.96	15.65	USP4	Ubiquitin carboxyl-terminal hydrolase 4	XM_002662510.7
A21G0010	11.96	5.89	DTWD2	tRNA-uridine aminocarboxypropyltransferase 2	XM_021468936.2
A25G0315	11.96	10.46	ELMO3	Engulfment and cell motility protein 3	XM_009297958.4
B3G1106	11.96	10.18	METTL2A	tRNA N(3)-methylcytidine methyltransferase METTL2	NM_001017902.1
A25G0261	11.96	11.50	CIAPIN1	Anamorsin	NM_001003738.1
A6G1017	11.96	17.66	CCNDBP1	Cyclin-D1-binding protein 1 homolog	NM_001089365.1
A19G0194	11.96	9.96	INTS3	Integrator complex subunit 3	NM_001044932.1
B8G0746	11.95	4.61	DIMT1	Probable dimethyladenosine transferase	NM_001003556.3
B16G0790	11.95	37.04	LENG8	Leukocyte receptor cluster member 8 homolog	NM_001089519.1
A3G0764	11.95	8.00	LOC113046264	A0A6P6JTH6_CARAU uncharacterized protein LOC113046264	XM_021474111.2
B16G0961	11.95	11.32	PTPN2	Tyrosine-protein phosphatase non-receptor type 2	NM_200466.2
A18G0302	11.93	6.21	SNRPA	U1 small nuclear ribonucleoprotein A	NM_199671.1
A18G0774	11.93	18.79	COX5A	Cytochrome c oxidase subunit 5A, mitochondrial	NM_001305577.1
B25G0478	11.93	6.18	SAMM50A	Sorting and assembly machinery component 50 homolog A	NM_200142.2
A6G0872	11.93	11.59	CHMP4B	Charged multivesicular body protein 4b	NM_200195.1
A2G0622	11.93	9.00	TCEA1	Transcription elongation factor A protein 1	NM_199994.2
A19G0013	11.93	25.37	KPNB1	Importin subunit beta-1	NM_001037702.3
B2G0965	11.93	24.85	GADD45A	Growth arrest and DNA damage-inducible protein GADD45 alpha	NM_200576.2
B4G0556	11.93	11.51	NUP107	Nuclear pore complex protein Nup107	NM_001030167.1
B7G0925	11.93	6.39	VBP1	Prefoldin subunit 3	NM_001020624.1
B12G0827	11.92	14.88	PPP4CB	threonine-protein phosphatase 4 catalytic subunit B	NM_001111168.1
B18G0255	11.92	12.53	DEF8	Differentially expressed in FDCP 8 homolog	NM_001328206.1
B16G0086	11.91	0.85	SERPINB1	Leukocyte elastase inhibitor	NM_001109730.1
A15G0131	11.91	7.24	SPSB4	SPRY domain-containing SOCS box protein 4	NM_001003474.1

A16G0713	11.91	8.04	CXCR3.2	C-X-C chemokine receptor type 3-2	NM_001007314.1
A8G0773	11.91	7.76	SORBS3	Vinexin	NM_001430979.1
B25G0150	11.91	3.46	IGKV1-27	Immunoglobulin kappa variable 1-27	XM_068217596.1
A24G0501	11.91	7.64	ELOC	Elongin-C	XM_068217058.1
B10G0151	11.91	2.79	KRT18	Keratin, type I cytoskeletal 18	NM_001044910.1
A7G0351	11.91	6.29	PLCB3	1-phosphatidylinositol 4,5-bisphosphate phosphodiesterase beta-3	XM_005166533.5
B2G0614	11.90	10.44	TMUB1	Transmembrane and ubiquitin-like domain-containing protein 1	NM_213298.2
B1G0529	11.90	7.11	NT5C2	Cytosolic purine 5'-nucleotidase	NM_001079966.2
A4G0788	11.90	8.84	ING3	Inhibitor of growth protein 3	NM_200937.2
A3G0337	11.90	20.39	PLPP3	Phospholipid phosphatase 3	NM_001079978.1
B19G0473	11.90	20.67	LYPLA2	Acyl-protein thioesterase 2	NM_200749.2
A13G0762	11.90	18.70	QKI	Protein quaking	NM_001089525.2
B21G0322	11.90	11.24	RRM1	Ribonucleoside-diphosphate reductase large subunit	NM_131455.2
B20G0853	11.89	15.91	XRN2	5'-3' exoribonuclease 2	NM_001001944.2
A3G0999	11.89	5.77	LSM12B	Protein LSM12 homolog B	NM_214817.1
B7G0995	11.89	3.04	LOC113105653	A0A6P6PPG6_CARAU uncharacterized protein LOC113105653	NM_001386649.1
A10G0800	11.88	26.30	IER3IP1	Immediate early response 3-interacting protein 1	
A10G0315	11.88	9.62	MRPL48	39S ribosomal protein L48, mitochondrial	NM_001013281.1
B17G0858	11.88	17.70	TMEM30A	Cell cycle control protein 50A	NM_212776.2
B16G0055	11.87	1.78			
A4G0596	11.87	6.43	A0A673I7K2	A0A673I7K2_9TELE BCL2 interacting killer	NM_001045038.2
B3G1130	11.87	10.01	MAZ	Myc-associated zinc finger protein	XM_005171594.5
B21G0660	11.87	8.39	SMARCB1A	SNF-related matrix-associated actin-dependent regulator of chromatin subfamily B member 1-A	XM_005161146.4
B9G0744	11.87	13.86	ATP5PF	ATP synthase-coupling factor 6, mitochondrial	NM_001256697.2
A24G0430	11.86	4.25	PLCD1	1-phosphatidylinositol 4,5-bisphosphate phosphodiesterase delta-1	NM_001109700.1
A3G0355	11.86	1.34			
B8G0152	11.85	4.62	ACSL6	Long-chain-fatty-acid--CoA ligase 6	XM_002663239.7
B7G0529	11.85	5.14	NOM1	Nucleolar MIF4G domain-containing protein 1	NM_001328060.1
A8G0007	11.85	9.45	FEM1C	Protein fem-1 homolog C	NM_198145.2
B5G1327	11.84	5.97	DDX54	ATP-dependent RNA helicase DDX54	NM_173268.1
B1G0281	11.84	40.64	ASAH1	Acid ceramidase	NM_200577.1
A7G0184	11.84	7.87	USP10	Ubiquitin carboxyl-terminal hydrolase 10	XM_680529.10
A14G0084	11.84	6.51	SH3RF2	E3 ubiquitin-protein ligase SH3RF2	XM_068219324.1
A23G0193	11.84	14.21	MRGBP	MORF4L-binding protein	NM_200736.1
A18G0728	11.83	2.00	CLNS1A	Methylosome subunit pICln	NM_131424.2
B2G1085	11.83	6.52	TBL1XR1	WD repeat-containing protein TBL1XR1	NM_001326540.1
A3G0236	11.83	8.07	DNAJA3	DnaJ homolog subfamily A member 3, mitochondrial	NM_201313.1

A20G0916	11.83	7.52	AKIRIN2	Akirin-2	NM_213294.2
B16G0360	11.83	18.02	STK40	threonine-protein kinase 40	XM_688183.10
A20G0755	11.82	15.57	GLRX5	Glutaredoxin-related protein 5, mitochondrial	NM_213021.1
A4G0358	11.82	15.25	NLRC3	NLR family CARD domain-containing protein 3	NM_001328438.1
A9G0404	11.82	13.51			XM_068223331.1
A20G0536	11.82	17.21	EXOC1	Exocyst complex component 1	NM_199597.1
A1G0161	11.82	17.90	DNAJB1	DnaJ homolog subfamily B member 1	NM_199773.2
A9G0846	11.81	13.61	LBP	Lipopolysaccharide-binding protein	
A2G0452	11.81	7.28	GTF2B	Transcription initiation factor IIB	NM_199697.1
B20G0377	11.81	13.75	GINM1	Glycoprotein integral membrane protein 1	NM_001079836.1
B20G0754	11.80	8.47	TTC32	Tetratricopeptide repeat protein 32	NM_001025520.1
A13G0482	11.80	16.36	AGA	N(4)-(beta-N-acetylglucosaminyl)-L-asparaginase	NM_200621.1
B16G0971	11.80	2.54	ZG16	Zymogen granule membrane protein 16	
B8G0954	11.80	11.12	TMEM127	Transmembrane protein 127	NM_001020524.1
B6G0909	11.80	7.59	TOMM34	Mitochondrial import receptor subunit TOM34	NM_199638.2
A19G0352	11.80	14.72	TRAPPC3	Trafficking protein particle complex subunit 3	NM_001003601.1
A13G0386	11.80	21.03	MYOF	Myoferlin	NM_001001840.3
B8G0550	11.80	9.36	NFYA	Nuclear transcription factor Y subunit alpha	XM_009303999.4
B8G1026	11.79	17.04	SLC35D2	GDP-mannose transporter	
A2G0218	11.79	16.77	FUBP1	Far upstream element-binding protein 1	XM_009297514.4
A5G1093	11.79	15.66	ACAC	Acetyl-CoA carboxylase	NM_001271308.1
A15G0582	11.79	10.16	VMP1	Vacuole membrane protein 1	XM_005157413.5
B22G0071	11.79	10.34	DEF6	Differentially expressed in FDCP 6 homolog	XM_003200920.6
B5G0728	11.79	7.16	NDUFS4	NADH dehydrogenase [ubiquinone] iron-sulfur protein 4, mitochondrial	NM_001020676.1
A22G0421	11.79	1.10	GZMA	Granzyme A	XM_009296345.3
A5G1067	11.79	4.49	UFC1	Ubiquitin-fold modifier-conjugating enzyme 1	NM_001003650.1
A25G0592	11.79	7.50	NAP1L4	Nucleosome assembly protein 1-like 4	XM_005170675.5
B2G0220	11.79	9.37	ACSL3	Fatty acid CoA ligase Acsl3	NM_001145932.1
B14G0315	11.79	1.14	PITX2	Pituitary homeobox 2	NM_130975.2
B1G0903	11.79	7.33	LOC113044075	A0A6P6JIJ7_CARAU uncharacterized protein LOC113044075	
A8G0726	11.78	5.74	ARPC5L	3 complex subunit 5-like protein	NM_200578.2
B1G0306	11.78	7.82	PGM2	Phosphopentomutase	NM_212886.1
B1G0018	11.78	4.13	SETDB2	Histone-lysine N-methyltransferase SETDB2	
A2G1111	11.78	9.68	YJU2	Splicing factor YJU2	NM_001110833.1
A13G0229	11.78	9.32	ZP3	Zona pellucida sperm-binding protein 3	XM_009307235.4
A5G0593	11.78	4.13	IER5L	Immediate early response gene 5-like protein	NM_199754.1

B22G0748	11.78	12.20	HMG20B	SNF-related matrix-associated actin-dependent regulator of chromatin subfamily E member 1-related	NM_001020551.2
A4G0982	11.77	9.90	DNAJC2	DnaJ homolog subfamily C member 2	NM_212811.1
A19G0191	11.77	15.75	MYLIPA	E3 ubiquitin-protein ligase MYLIP-A	NM_199983.2
B2G1037	11.77	34.53	RAB18B	Ras-related protein Rab-18-B	NM_001003449.1
B22G1044	11.77	12.69	MCCC1	Methylcrotonoyl-CoA carboxylase subunit alpha, mitochondrial	NM_001044915.1
A13G0053	11.76	12.61	IRF2BP2A	Interferon regulatory factor 2-binding protein 2-A	NM_212700.1
A14G0403	11.76	13.25	ELF2	ETS-related transcription factor Elf-2	NM_001004116.1
A2G0429	11.76	5.01	PRRX1	Paired mesoderm homeobox protein 1	NM_214734.1
A5G1033	11.76	3.15	UPK3B	Uroplakin-3b	
B6G0182	11.76	43.68	MYH11	Myosin-11	NM_001024448.1
A14G0053	11.76	3.56	SSRP1	FACT complex subunit SSRP1	NM_212802.2
B13G0440	11.75	11.70	ADAM8	Disintegrin and metalloproteinase domain-containing protein 8	NM_001077379.1
B16G0759	11.75	28.03	DEDD2	DNA-binding death effector domain-containing protein 2	NM_131602.2
B25G0792	11.75	5.82	GATD1	Glutamine amidotransferase-like class 1 domain-containing protein 1	
B4G0043	11.75	12.26	XPOT	Exportin-T	NM_001044852.1
B14G0699	11.75	18.12	FMR1-A	Fragile X messenger ribonucleoprotein 1 homolog A	NM_152963.1
B9G0217	11.74	6.62	WDR75	WD repeat-containing protein 75	NM_200090.1
B23G0247	11.74	13.21	EIF4B	Eukaryotic translation initiation factor 4B	NM_001099237.2
B23G0450	11.74	5.55	ARHGEF7	Rho guanine nucleotide exchange factor 7	XM_005162207.4
A23G0645	11.73	10.58	FAM50A	Protein FAM50A	NM_001017636.1
A16G0781	11.73	9.13	DEPTOR	DEP domain-containing mTOR-interacting protein	XM_005173523.5
B5G0440	11.73	7.76	DERL2	Derlin-2	NM_001024395.1
B8G0676	11.73	14.50	ACSBG2	Long-chain-fatty-acid--CoA ligase ACSBG2	NM_001126379.1
A20G0737	11.73	13.93	TMEM30A	Cell cycle control protein 50A	NM_205560.3
A1G0147	11.73	11.85	C3	Complement C3 (Fragment)	XM_002660577.7
A11G0381	11.73	13.74	MDFIC2	MyoD family inhibitor domain-containing protein 2	NM_001328581.1
B25G0668	11.72	15.92	PPP6R3	threonine-protein phosphatase 6 regulatory subunit 3	XM_009297814.4
B12G0336	11.72	9.04	HEXD	Hexosaminidase D	NM_001077167.2
A14G0235	11.72	6.21	LNX2	Ligand of Numb protein X 2	NM_212940.2
A4G0730	11.72	6.76	DYRK2	Dual specificity tyrosine-phosphorylation-regulated kinase 2	XM_005164640.5
B9G0380	11.72	8.87	GPR183A	G-protein coupled receptor 183-A	NM_001099241.1
B5G1200	11.72	6.71	ZC4H2	Zinc finger C4H2 domain-containing protein	NM_199642.1
A24G0461	11.72	10.78	MLLT10	Protein AF-10	XM_017353908.3
B1G0622	11.72	7.08	A0A673MTP3	A0A673MTP3_9TELE Ciliary neurotrophic factor	NM_001145632.1

B2G0157	11.72	7.82	VIM	Vimentin	NM_200773.1
B19G0321	11.71	21.39	DAPK2	Death-associated protein kinase 2	XM_021468288.2
A20G0394	11.71	10.47	SEC63	Translocation protein SEC63 homolog	NM_001002588.1
B23G0139	11.71	3.52	TWIST1	Twist-related protein 1	NM_001017820.1
B23G0818	11.71	6.71	LOC113041264	A0A6P6J5J0_CARAU neurofilament heavy polypeptide-like isoform X9	XM_068216764.1
B13G0834	11.71	8.76	HFE	Hereditary hemochromatosis protein homolog	NM_001013445.1
A24G0417	11.71	5.95	MIX23	Protein MIX23	NM_200807.1
A2G0011	11.70	3.19	TNS3	Tensin-3	XM_068213326.1
A3G0607	11.70	3.21	SUZ12A	Polycomb protein suz12-A	NM_001003529.2
B1G1185	11.70	20.65	CYP3A27	Cytochrome P450 3A27	NM_001037438.2
B21G0332	11.70	14.01	SLC7A3	Cationic amino acid transporter 3	NM_001007329.2
A5G0367	11.70	9.38	FAM76B	Protein FAM76B	NM_199932.1
A16G0133	11.70	14.19	LAMTOR2	Ragulator complex protein LAMTOR2	
B2G0380	11.70	13.61	DHRS1	reductase SDR family member 1	NM_001002205.1
A25G0189	11.70	13.09	PPP6R3	threonine-protein phosphatase 6 regulatory subunit 3	NM_199684.2
A16G0434	11.69	27.81	CSNK2A1	Casein kinase II subunit alpha	NM_131252.1
B2G0434	11.68	9.94	PEX19	Peroxisomal biogenesis factor 19	NM_001017399.1
A19G0601	11.68	2.28	IGKC	Immunoglobulin kappa constant	
A1G1122	11.68	8.97	PPA2	Inorganic pyrophosphatase 2, mitochondrial	XM_005159884.4
B1G0038	11.68	6.92	TRMT10C	tRNA methyltransferase 10 homolog C	NM_001007332.2
B6G0773	11.68	8.51	SEC61AL1	Protein transport protein Sec61 subunit alpha-like 1	NM_201577.1
B15G0014	11.67	11.37	MED17	Mediator of RNA polymerase II transcription subunit 17	NM_001077574.1
B5G0241	11.67	6.20	MTHFD2	cyclohydrolase, mitochondrial	NM_001002181.1
A14G0149	11.67	16.99	STAG2	Cohesin subunit SA-2	XM_005173193.5
B4G0382	11.67	5.37	CMASA	N-acylneuraminate cytidylyltransferase A	NM_001040252.2
A9G0623	11.66	6.19	LOC113053615	A0A6P6KSY4_CARAU uncharacterized protein LOC113053615	
B9G0174	11.66	27.23	ITGB2	Integrin beta-2	XM_680920.8
B1G0932	11.66	11.23	LALBA	Alpha-lactalbumin	
A25G0094	11.65	8.19	PARVG	Gamma-parvin	NM_001089538.1
A4G0488	11.65	14.00	ZC3HAV1	Zinc finger CCCH-type antiviral protein 1	NM_001014325.1
A3G0492	11.65	17.77	WIPI2	WD repeat domain phosphoinositide-interacting protein 2	XM_005164125.5
A3G0458	11.65	8.42	UBFD1	Ubiquitin domain-containing protein UBFD1	XM_005164210.5
A3G1222	11.65	17.71	LGALS3BPB	Galectin-3-binding protein B	NM_212873.1
B5G0794	11.64	10.76	LTBP3	Latent-transforming growth factor beta-binding protein 3	XM_005165333.5
A25G0568	11.64	3.78	NT5C1B	Cytosolic 5'-nucleotidase 1B	XM_702238.10
A3G0748	11.64	8.66	CCDC47	PAT complex subunit CCDC47	NM_001004551.1
A15G0728	11.64	16.36	ECH1	Delta(3,5)-Delta(2,4)-dienoyl-CoA isomerase, mitochondrial	NM_001006068.1
B14G0688	11.63	9.43	SMIM19	Small integral membrane protein 19	NM_001025535.2
A6G0018	11.63	0.81	GIMAP4	GTPase IMAP family member 4	
A13G0304	11.63	6.17	NT5C1A	Cytosolic 5'-nucleotidase 1A	NM_001077718.1

B23G0332	11.63	8.37	SEPHS3	Selenide, water dikinase 3	NM_001004295.2
B13G0434	11.63	11.18	LGALSL	Galectin-related protein	XM_005156720.5
A16G0404	11.63	5.67	MBOAT7	Lysophospholipid acyltransferase 7	NM_200537.1
B20G0234	11.62	5.94	TMED5	Transmembrane emp24 domain-containing protein 5	NM_200403.1
B19G0612	11.62	87.14	NDRG1	Protein NDRG1	NM_213348.3
A13G0054	11.62	8.59	EGLN1	Egl nine homolog 1	NM_213484.1
B24G0391	11.62	12.52	BCLAF3	BCLAF1 and THRAP3 family member 3	
A9G0086	11.62	3.37	COBLL1	Cordon-bleu protein-like 1	NM_001365214.1
B12G0364	11.62	8.07	COX7A2L	Cytochrome c oxidase subunit 7A-related protein, mitochondrial	
A7G0767	11.62	25.44	RAB8B	Ras-related protein Rab-8B	NM_001099259.1
B14G0555	11.62	10.44			NM_212677.1
B13G0943	11.62	43.18			
B10G0809	11.61	14.51	YKT6	Synaptobrevin homolog YKT6	NM_201092.1
B20G0048	11.61	38.15	AMD1	A0A7N6A9C3_ANATE Adenosylmethionine decarboxylase 1	NM_001161601.1
A14G0195	11.61	11.93	RAP2C	Ras-related protein Rap-2c	NM_001007055.1
A8G0673	11.60	7.40	SLC23A2	Solute carrier family 23 member 2	XM_021472134.2
A5G0414	11.60	7.68	MRPL41	39S ribosomal protein L41, mitochondrial	
A16G0861	11.60	4.43	EMG1	Ribosomal RNA small subunit methyltransferase NEP1	NM_001040337.2
B15G0511	11.60	8.29	TMEM199	Transmembrane protein 199	NM_001105597.1
B14G0021	11.60	14.13	LEPROTL1	Leptin receptor overlapping transcript-like 1	
B3G1164	11.59	19.25	UBE2I	SUMO-conjugating enzyme UBC9	
B3G0656	11.59	11.65	LOC107721488	A0A673GLR1_9TELE Receptor activity-modifying protein 1-like	
B16G0185	11.59	5.09	G5714_015700	A0A7J6C684_9TELE Uncharacterized protein	
A18G0473	11.59	22.21			
B22G0130	11.59	7.72	IMPACT	Protein IMPACT	NM_001005595.1
A22G0466	11.58	19.81	REX1BD	Required for excision 1-B domain-containing protein	NM_001256159.1
B5G0413	11.58	46.31	PPP2R1B	threonine-protein phosphatase 2A 65 kDa regulatory subunit A beta isoform	NM_001312917.1
A2G0145	11.58	8.92	CMPK	UMP-CMP kinase	NM_213109.2
B12G0522	11.58	10.41	ATAD1B	Outer mitochondrial transmembrane helix translocase	NM_001024421.2
B17G0261	11.58	3.91	SYTL2	Synaptotagmin-like protein 2	XM_688977.9
A21G0588	11.57	11.54	OTUB1	Ubiquitin thioesterase OTUB1	NM_001002411.2
B13G0616	11.57	11.31	PLEK2	Pleckstrin-2	
A14G0448	11.56	19.70	PPP2CA	threonine-protein phosphatase 2A catalytic subunit alpha isoform	NM_001017886.1
A15G0042	11.56	5.01	EIF4G1	Eukaryotic translation initiation factor 4 gamma 1	XM_068217716.1
A7G0591	11.56	5.26	SPSB3	SPRY domain-containing SOCS box protein 3	XM_002662848.6
A23G0500	11.56	20.42	STAT5B	Signal transducer and activator of transcription 5B	NM_001130590.1
B6G0503	11.56	7.22	TPRG1L	Tumor protein p63-regulated gene 1-like protein	NM_001089528.2

B23G0212	11.55	8.88	EXOSC10	Exosome component 10	NM_201089.2
B6G0774	11.55	24.97	IP6K2	Inositol hexakisphosphate kinase 2	NM_201470.1
B18G0738	11.55	8.03	GCSH	Glycine cleavage system H protein, mitochondrial	NM_001013457.1
A19G0426	11.55	6.34	FKBP9	Peptidyl-prolyl cis-trans isomerase FKBP9	NM_001003520.1
B2G0144	11.55	3.75	THEM6	Protein THEM6	NM_001122704.1
B18G0621	11.55	7.95	UTP4	U3 small nucleolar RNA-associated protein 4 homolog	NM_213282.2
B23G0742	11.54	17.65	UBE2J2	Ubiquitin-conjugating enzyme E2 J2	NM_001089535.1
A14G0400	11.54	9.97	SLU7	Pre-mRNA-splicing factor SLU7	NM_001007367.1
B25G0026	11.54	20.93	GABARAPL2	Gamma-aminobutyric acid receptor-associated protein-like 2	NM_205723.1
B16G0511	11.53	20.68	CTSS	Cathepsin S	XM_009292324.4
B8G0260	11.53	9.17	SORBS3	Vinexin	NM_001430979.1
B5G1197	11.53	34.18	MSN	Moesin	NM_001004296.1
B13G0361	11.53	21.21	SH2D4B	SH2 domain-containing protein 4B	NM_212803.1
A1G0728	11.53	2.35	JAM2A	Junctional adhesion molecule 2A	
B1G0083	11.53	17.19	GGACT.2	Gamma-glutamylaminecyclotransferase B	
B5G0841	11.53	10.56	YIPF6	Protein YIPF6	NM_001002096.1
A6G0417	11.53	12.81	SEPTIN9	Septin-9	NM_001327975.1
A13G0637	11.52	8.23			NM_214769.1
B18G0413	11.52	9.71	SERF2	Small EDRK-rich factor 2	NM_001172661.1
B10G0062	11.52	6.44	SEC31A	Protein transport protein Sec31A	NM_199530.2
A24G0597	11.52	1.35	A0A3P9LKB4	A0A3P9LKB4_ORYLA SWIM-type domain-containing protein	
A2G0082	11.52	17.01	THOC1	THO complex subunit 1	NM_201324.1
A1G0463	11.52	4.25	TMX2B	Thioredoxin-related transmembrane protein 2-B	NM_001002113.1
A17G0390	11.51	22.71	RPS6KA	Ribosomal protein S6 kinase 2 alpha	NM_001077775.2
A6G0409	11.51	9.20	RNF135	E3 ubiquitin-protein ligase RNF135	XM_021474084.2
A5G0923	11.51	7.84	MFSD10	Major facilitator superfamily domain-containing protein 10	NM_001017667.1
A4G0958	11.50	5.09	DUS4L	20b) synthase [NAD(P)+]-like	NM_001003490.2
A17G0505	11.50	5.59	FAM98A	Protein FAM98A	XM_021473146.2
B6G0543	11.50	21.58	PDE4B	cAMP-specific 3',5'-cyclic phosphodiesterase 4B	XM_068218329.1
B25G0481	11.50	5.58	TDG	T mismatch-specific thymine DNA glycosylase	NM_001020751.1
B13G0484	11.50	3.58	HARBI1	Putative nuclease HARBI1	NM_001045138.1
A13G0248	11.50	11.52	POL	Retrovirus-related Pol polyprotein from transposon 17.6	NM_001354552.1
A4G1007	11.49	15.25	TBK1	threonine-protein kinase TBK1	NM_001044748.2
B23G0636	11.49	7.36	ORMDL2	ORM1-like protein 2	NM_001040048.1
B3G1421	11.49	22.22	CYTH1	Cytohesin-1	XM_017353258.3
B4G0314	11.49	13.86	ENDOUC	Uridylate-specific endoribonuclease C	
B15G0630	11.49	36.78	LGALS9	Galectin-9	
A13G0050	11.49	4.01	GGPS1	Geranylgeranyl pyrophosphate synthase	XM_009299148.4
B23G0170	11.49	8.68	LAD1	Ladinin-1	NM_001386486.1

A16G0279	11.49	8.84	STT3B	Dolichyl-diphosphooligosaccharide--protein glycosyltransferase subunit STT3B	NM_001328161.1
B22G0181	11.49	8.05	MYDGF	Myeloid-derived growth factor	NM_001002480.2
B7G0388	11.49	6.69	CTSA	Lysosomal protective protein	NM_001386652.1
B18G0374	11.48	26.62	ACO	1-aminocyclopropane-1-carboxylate oxidase	NM_001102635.1
A14G0736	11.48	25.61	OGT	UDP-N-acetylglucosamine--peptide N-acetylglucosaminyltransferase 110 kDa subunit	NM_001018106.1
A1G0464	11.48	6.93	MED19B	Mediator of RNA polymerase II transcription subunit 19-B	NM_001003416.2
B22G0419	11.48	6.32	LOC113099136	A0A6P6PEW8_CARAU uncharacterized protein LOC113099136	XM_021469541.2
A7G1147	11.47	15.46	ARIH1	E3 ubiquitin-protein ligase arih1	NM_212923.1
B4G0030	11.47	10.72	PARP12	Protein mono-ADP-ribosyltransferase PARP12	NM_001327797.2
A11G0160	11.47	8.63	HYLS1	Centriolar and ciliogenesis-associated protein HYSL1	NM_001080647.1
A20G0320	11.46	9.92	CD2AP	CD2-associated protein	NM_001008583.2
A21G0543	11.46	18.09	ADSS2	Adenylosuccinate synthetase isozyme 2	NM_173237.2
B4G0471	11.46	4.07	SCO2	Protein SCO2 homolog, mitochondrial	
A16G0683	11.46	22.89	GNL1	Guanine nucleotide-binding protein-like 1	NM_001077253.1
A18G0331	11.45	5.97	YAP1	Transcriptional coactivator YAP1	NM_001139480.1
B7G0391	11.45	10.97	KATNB1	Katanin p80 WD40 repeat-containing subunit B1	XM_005166505.5
B2G0877	11.45	11.43			XM_005171207.5
A25G0489	11.44	15.57	COMMD4	COMM domain-containing protein 4	NM_001320175.1
B3G1295	11.44	20.34	HAGH	Hydroxyacylglutathione hydrolase, mitochondrial	NM_200043.3
A25G0363	11.44	19.28	EDIL3	EGF-like repeat and discoidin I-like domain-containing protein 3	XM_005163016.5
B1G0384	11.44	12.62	UGDH	UDP-glucose 6-dehydrogenase	NM_001110402.1
A2G0176	11.44	7.44	RGS18	Regulator of G-protein signaling 18	NM_001077307.1
B1G1029	11.44	10.43	LOC113115393	A0A6P6QZD6_CARAU structural maintenance of chromosomes protein 5-like	
B14G0002	11.43	7.81	MCM7-A	DNA replication licensing factor mcm7-A	NM_212569.1
A1G0294	11.43	5.04	ESF1	ESF1 homolog	NM_199745.2
A21G0132	11.43	0.96	LOC113063770	A0A6P6LZI5_CARAU urokinase plasminogen activator surface receptor-like	
A23G0768	11.43	5.32			
B10G0788	11.43	22.60	PTPN11	Tyrosine-protein phosphatase non-receptor type 11	NM_199846.1
B18G0349	11.42	13.56	BCKDHA	2-oxoisovalerate dehydrogenase subunit alpha, mitochondrial	NM_001024419.1
A23G0796	11.42	14.67	CSMD1	CUB and sushi domain-containing protein 1	XM_009297128.4
B25G0640	11.42	9.06	UBA2	SUMO-activating enzyme subunit 2	XM_009297858.4

B16G0876	11.42	12.38	RXRBB	Retinoic acid receptor RXR-beta-B	XM_021466617.2
B22G0505	11.42	22.44	HTD2	Hydroxyacyl-thioester dehydratase type 2, mitochondrial	NM_001020692.2
B12G0361	11.42	3.84	HCAR3	Hydroxycarboxylic acid receptor 3	XM_021472553.2
B3G0723	11.42	9.26	YIPF1	Protein YIPF1	NM_001003500.1
B1G0637	11.42	13.74	NAA15	N-alpha-acetyltransferase 15, NatA auxiliary subunit	NM_203321.2
A23G0415	11.42	6.58	NOC2L	Nucleolar complex protein 2 homolog	XM_017353600.3
B3G1709	11.41	22.31	HB24	H-2 class II histocompatibility antigen, E-Q beta chain	
B8G0963	11.41	0.62	DHX37	Probable ATP-dependent RNA helicase DHX37	NM_001083004.2
B5G0851	11.41	4.21	PTCD2	Pentatricopeptide repeat-containing protein 2, mitochondrial	XM_005171851.5
A1G1067	11.41	6.33	GTF2E2	General transcription factor IIE subunit 2	NM_212731.1
B14G0410	11.41	11.53	ELF2	ETS-related transcription factor Elf-2	NM_001004116.1
B2G0088	11.41	13.22	RANBP3	Ran-binding protein 3	NM_200690.1
A7G0605	11.41	6.70	NDUFS3	NADH dehydrogenase [ubiquinone] iron-sulfur protein 3, mitochondrial	NM_001017755.1
A11G0596	11.41	6.68			NM_001118895.1
A12G0759	11.41	4.44	PRSS33	Serine protease 33	NM_001002596.1
B16G1146	11.41	60.17	NLRP3	NACHT, LRR and PYD domains-containing protein 3	XM_003200109.6
A6G0763	11.41	13.60	SEC61AL1	Protein transport protein Sec61 subunit alpha-like 1	NM_201577.1
B4G0070	11.40	6.37	MRPL42	39S ribosomal protein L42, mitochondrial	
B22G0626	11.40	2.27	RPP21	Ribonuclease P protein subunit p21	NM_001003530.2
A1G0683	11.40	7.83	EED	Polycomb protein eed	NM_001017766.2
A10G0683	11.40	4.00	STOML2	Stomatin-like protein 2, mitochondrial	NM_201031.1
B6G1049	11.40	6.15	FUNDC1	FUN14 domain-containing protein 1	
B4G0408	11.40	14.38	COPG2	Coatomer subunit gamma-2	NM_130994.1
B6G0874	11.40	18.81	VAPB	C	XM_021476783.2
B6G0432	11.39	8.48	MIF4GDA	MIF4G domain-containing protein A	XM_005166179.5
B7G0581	11.39	8.51	MTCH2	Mitochondrial carrier homolog 2	NM_131382.1
B9G0285	11.39	7.68	GABPA	GA-binding protein alpha chain	XM_005167853.5
B23G0784	11.39	0.06	TGM1	Protein-glutamine gamma-glutamyltransferase K	XM_001331878.8
B25G0322	11.39	7.79	BNIP2	adenovirus E1B 19 kDa protein-interacting protein 2	XM_009298160.4
B17G0002	11.39	4.69	POLE2	DNA polymerase epsilon subunit 2	
A5G0144	11.39	8.84	PHETA1	Sesquipedalian-1	XM_688552.8
A13G0134	11.38	7.26	CDC42EP3	Cdc42 effector protein 3	XM_005169512.5
A11G0163	11.38	17.66	KRT15	Keratin, type I cytoskeletal 15	NM_001199912.1
B12G0108	11.38	19.86	PTPRE	Receptor-type tyrosine-protein phosphatase epsilon	XM_003199652.6
A10G0001	11.38	8.32	TIPRL	TIP41-like protein	NM_001328426.1

A25G0299	11.37	11.04	NDUFA9	NADH dehydrogenase [ubiquinone] 1 alpha subcomplex subunit 9, mitochondrial	NM_001013459.2
B6G0092	11.37	10.20	PPIG	Peptidyl-prolyl cis-trans isomerase G	NM_213464.1
B3G1568	11.37	47.77	LOC113047096	A0A6P6JWG1_CARAU uncharacterized protein LOC113047096	XM_068222460.1
B12G0636	11.37	5.55	TUT1	Speckle targeted PIP5K1A-regulated poly(A) polymerase	NM_001030188.1
B14G0837	11.37	13.88	FAM199X	Protein FAM199X	NM_001003766.2
B20G0713	11.37	44.57	SERINC1	Serine incorporator 1	NM_199727.1
B2G0167	11.36	3.17	COA1	Cytochrome c oxidase assembly factor 1 homolog	XM_017352357.3
A1G0482	11.36	2.74	CD5	T-cell surface glycoprotein CD5	
A10G0490	11.36	8.52	MRPL49	39S ribosomal protein L49, mitochondrial	NM_001386320.1
A23G0701	11.35	9.28	AGAP2	Arf-GAP with GTPase, ANK repeat and PH domain-containing protein 2	XM_009297046.4
A17G0448	11.35	8.05	HMGCL	Hydroxymethylglutaryl-CoA lyase, mitochondrial	NM_201215.1
B16G1022	11.35	11.89	NDUFB9	NADH dehydrogenase [ubiquinone] 1 beta subcomplex subunit 9	NM_001017880.1
B21G0132	11.35	11.25	TCERG1	Transcription elongation regulator 1	XM_009295682.4
A18G0350	11.34	5.91	SMAP	Small acidic protein	NM_200194.1
A17G0273	11.34	23.83	XDH	oxidase	XM_683891.8
A11G0096	11.34	5.77	IL17RD	Interleukin-17 receptor D	NM_001002160.2
B16G1133	11.34	19.82	ARNT	Aryl hydrocarbon receptor nuclear translocator	XM_005157852.5
A5G0383	11.34	6.36	CTDNEP1A	CTD nuclear envelope phosphatase 1A	NM_001007309.1
B19G0262	11.33	20.69	LOC113062158	A0A6P6LSW3_CARAU chromosomal protein D1-like	XM_701324.10
A22G0096	11.33	2.55	MR1	Major histocompatibility complex class I-related gene protein	
A2G0356	11.33	3.53	PTPRF	Receptor-type tyrosine-protein phosphatase F	XM_068212965.1
B23G0120	11.33	1.27	TGM1	Protein-glutamine gamma-glutamyltransferase K	XM_009296576.4
B7G0968	11.33	11.70	IL16	Pro-interleukin-16	XM_021477711.2
B17G0637	11.32	8.20	EIF2S1	Eukaryotic translation initiation factor 2 subunit 1	NM_199569.1
A18G0038	11.32	8.27	SHKBP1	SH3KBP1-binding protein 1	XM_001920900.8
A18G0788	11.32	7.14	LARP6	La-related protein 6	NM_199903.1
A11G0776	11.32	8.12	ELMO2	Engulfment and cell motility protein 2	XM_021479925.2
A12G0215	11.32	16.79	SLC9A3R1	H(+) exchange regulatory cofactor NHE-RF1	NM_212707.2
A19G0278	11.31	4.91	SLC7A7	Y+L amino acid transporter 1	NM_001100022.1
B13G0128	11.31	11.50	MEIS1	Homeobox protein Meis1	XM_005169532.5
A18G0429	11.31	23.78	CHKA	Choline kinase alpha	XM_005159105.5
B22G0983	11.31	8.65	MANF	Mesencephalic astrocyte-derived neurotrophic factor	NM_001076629.1
A20G0426	11.31	13.02	CEP43	Centrosomal protein 43	XM_068215412.1
A8G0591	11.31	3.58	MAPK4	Mitogen-activated protein kinase 4	XM_005167152.5
B13G0681	11.30	7.94	ACTN1	Alpha-actinin-1	XM_005156652.4

A19G0129	11.30	9.09	SPAG1A	Sperm-associated antigen 1A	NM_001089406.2
A20G0595	11.30	6.90	LNX1	E3 ubiquitin-protein ligase LNX	XM_005160506.5
A4G0043	11.29	25.16	MS4A8	Membrane-spanning 4-domains subfamily A member 8	
B9G0066	11.29	17.09	TBL1X	WD repeat-containing protein TBL1X	NM_001082994.1
B6G0022	11.29	3.60	RBFOX2	RNA binding protein fox-1 homolog 2	NM_214746.2
B15G0601	11.29	2.46	LOC107588402	A0A672MT73_SINGR Uncharacterized LOC107588402	
B4G0140	11.29	3.84	OSGEP	tRNA N6-adenosine threonylcarbamoyltransferase	NM_001017751.2
B17G0860	11.29	4.17	POGK	Pogo transposable element with KRAB domain	
B18G0151	11.29	10.60	CIPC	CLOCK-interacting pacemaker	NM_001045055.1
A3G0453	11.28	23.52	LOC107568934	A0A672K614_SINGR Interleukin-4 receptor subunit alpha-like	
A14G0469	11.28	13.28	FAF2	FAS-associated factor 2	NM_001128680.1
A5G1084	11.28	5.30	PJA2	E3 ubiquitin-protein ligase Praja-2	NM_001105116.2
A20G0885	11.28	11.54	PPIL4	Peptidyl-prolyl cis-trans isomerase-like 4	NM_001128330.1
B16G0124	11.28	21.86	TAP1	Antigen peptide transporter 1	XM_002665007.4
B21G0532	11.28	20.63	TGFB1	Transforming growth factor beta-1 proprotein	XM_687246.9
A11G0027	11.28	7.69	TK1	Thymidine kinase, cytosolic	XM_021479676.2
B19G0851	11.28	9.02	CTHRC1	Collagen triple helix repeat-containing protein 1	NM_001003765.3
A16G0584	11.27	11.09	TBC1D31	TBC1 domain family member 31	XM_068214126.1
A1G1100	11.27	14.44	PLRG1	Pleiotropic regulator 1	NM_213440.1
B16G0293	11.27	8.09	TP53INP1	Tumor protein p53-inducible nuclear protein 1	NM_001080036.1
A7G0642	11.26	7.30	SLC12A4	Solute carrier family 12 member 4	XM_686199.8
B9G0157	11.26	26.60			
A17G0869	11.26	12.81	SIVA1	Apoptosis regulatory protein Siva	
A15G0790	11.26	5.13	RNF168	E3 ubiquitin-protein ligase rnf168	NM_199855.1
A23G0411	11.25	57.81	ENO1	Alpha-enolase	XM_005162152.5
B20G0298	11.25	4.02	LCK	Proto-oncogene tyrosine-protein kinase LCK	NM_001030220.1
A3G0674	11.25	3.60	CASP6	Caspase-6	
A20G0399	11.25	21.57			NM_131085.1
B19G0565	11.24	55.58	GRN1	Granulin-1	
A16G0291	11.24	11.93	EMC2	ER membrane protein complex subunit 2	NM_213544.1
B22G1078	11.24	8.22	ARMH3	Armadillo-like helical domain-containing protein 3	NM_200619.2
B25G0584	11.24	7.84	CIAPIN1	Anamorsin	
A13G0309	11.24	2.84	SERTAD4	SERTA domain-containing protein 4	XM_001338200.8
A25G0529	11.24	11.83	CTDSPL2A	CTD small phosphatase-like protein 2-A	NM_001077544.1
B9G0354	11.24	9.67	DNAJB11	DnaJ homolog subfamily B member 11	NM_198821.1
A8G0109	11.24	4.08	DHX37	Probable ATP-dependent RNA helicase DHX37	NM_001083004.2
A10G0542	11.24	15.57	ILK	Integrin-linked protein kinase	NM_200571.1

B24G0396	11.24	3.53	G5714_023412	A0A7J6BL67_9TELE Uncharacterized protein	XM_021473784.2
A5G0131	11.23	3.79	AGPAT9L	Glycerol-3-phosphate acyltransferase 3-like	NM_001099450.1
B21G0546	11.23	5.86	A0A671R606	A0A671R606_9TELE Si:ch211-191j22.3	NR_131904.1
B25G0341	11.23	9.08	USP3	Ubiquitin carboxyl-terminal hydrolase 3	NM_001199871.1
B22G1057	11.23	5.82	B3GNT7	UDP-GlcNAc:betaGal beta-1,3-N-acetylglucosaminyltransferase 7	XM_009296510.4
B11G0651	11.23	5.95	EPHA8	Ephrin type-A receptor 8	XM_009305794.4
A5G0288	11.23	10.99	CORO1CA	Coronin-1C-A	NM_201295.1
A12G0549	11.22	10.08	SVIL	Supervillin	XM_009306542.4
B8G1057	11.22	25.61	AP3S1	AP-3 complex subunit sigma-1	NM_001005964.1
A21G0317	11.22	9.24	WDR5	WD repeat-containing protein 5	NM_213099.1
A5G0462	11.22	7.15	LMAN2L	VIP36-like protein	NM_001076733.1
B4G0201	11.22	12.37	LTA4H	Leukotriene A-4 hydrolase	NM_213286.1
B22G0859	11.21	7.79	MRPS28	28S ribosomal protein S28, mitochondrial	
A16G0964	11.21	14.07	NT5C3A	Cytosolic 5'-nucleotidase 3A	NM_199560.2
A24G0383	11.21	5.96	ABHD10	Palmitoyl-protein thioesterase ABHD10, mitochondrial	NM_001386473.1
A16G0824	11.20	17.22	CPVL	Probable serine carboxypeptidase CPVL	NM_199278.1
A19G0927	11.20	4.50	TATDN1	Deoxyribonuclease TATDN1	NM_001002213.1
A3G0693	11.20	6.44	RAB35	Ras-related protein Rab-35	XM_021471455.2
A10G0576	11.20	24.93	PCOLCE2	Procollagen C-endopeptidase enhancer 2	NM_001030181.3
A18G0528	11.20	14.27	AMPD3	AMP deaminase 3	XM_021467594.2
A6G0070	11.20	11.38	PRKAG1	5'-AMP-activated protein kinase subunit gamma-1	NM_213161.1
B1G0404	11.19	6.07	GATB	Glutamyl-tRNA(Gln) amidotransferase subunit B, mitochondrial	NM_001013446.3
B15G0745	11.19	19.78	G5714_015330	A0A7J6CCI7_9TELE Uncharacterized protein	
A17G0276	11.19	4.27	D5F01_LYC23630	A0A6G0HI59_LARCR Uncharacterized protein	
A1G0273	11.19	16.68	RNF11	RING finger protein 11	NM_201021.1
A8G0346	11.19	2.10	COLGALT2	Procollagen galactosyltransferase 2	NM_001130076.1
A23G0291	11.19	15.11	NUCKS1	Nuclear ubiquitous casein and cyclin-dependent kinase substrate 1	NM_001099239.1
A4G0953	11.18	3.92	PLEKHA5	Pleckstrin homology domain-containing family A member 5	XM_009300010.4
A14G0406	11.18	9.40	NAA15	N-alpha-acetyltransferase 15, NatA auxiliary subunit	NM_200646.2
B4G0002	11.18	7.02	PTPN12	Tyrosine-protein phosphatase non-receptor type 12	NM_200669.1
B2G0329	11.18	31.61	GBP1	Guanylate-binding protein 1	XM_694125.8
A18G0340	11.17	10.68	NAT10	RNA cytidine acetyltransferase	NM_200644.1
B23G0016	11.17	0.88	RTASE	Probable RNA-directed DNA polymerase from transposon BS	
B24G0601	11.17	5.42	CYRIB	CYFIP-related Rac1 interactor B	NM_207078.1
B23G0196	11.17	13.13	UCKL1	Uridine-cytidine kinase-like 1	XM_005162007.5
B4G0338	11.17	17.82	MSRB3	Methionine-R-sulfoxide reductase B3	NM_001320380.1
B16G0269	11.17	9.33	ASTE1	Protein asteroid homolog 1	
A21G0009	11.17	6.59	NUP54	Nucleoporin p54	XM_021468768.2

B16G0419	11.17	39.07	PNISR	serine-rich protein PNISR	NM_001083837.1
B22G0792	11.16	5.18	GNA11	Guanine nucleotide-binding protein subunit alpha-11	NM_001045036.2
B24G0169	11.16	15.66	PDPK1	3-phosphoinositide-dependent protein kinase 1	NM_205699.1
B22G1038	11.16	22.76	ACY1A	Aminoacylase-1A	NM_001272080.1
A10G0838	11.16	8.87	SPTLC1	Serine palmitoyltransferase 1	NM_001020471.1
A15G0864	11.16	5.83	GFM1	Elongation factor G, mitochondrial	NM_001079994.1
A18G0636	11.16	10.98	PRPF18	Pre-mRNA-splicing factor 18	XM_021467629.2
A12G0186	11.16	18.21	TBCC	Tubulin-specific chaperone C	NM_001256248.1
A19G0542	11.15	0.62	HOXA13A	Homeobox protein Hox-A13a	NM_001085494.1
B14G0684	11.15	13.13	G5714_014541	A0A7J6CDC5_9TELE Uncharacterized protein	
B8G0925	11.15	12.02	SSUH2	Protein SSUH2 homolog	XM_005166701.5
B3G0650	11.15	8.23	PSMC3IP	Homologous-pairing protein 2 homolog	NM_001002124.2
A22G0539	11.15	8.85	POLR3F	DNA-directed RNA polymerase III subunit RPC6	NM_201187.1
B10G0121	11.14	3.10	SREK1IP1	Protein SREK1IP1	
A5G1024	11.14	13.00	TMEM248	Transmembrane protein 248	NM_001013530.1
B2G0629	11.14	10.70	STARD3NL	STARD3 N-terminal-like protein	NM_001002320.1
A7G0988	11.14	11.44	LOC113052197	A0A6P6KJC8_CARAU uncharacterized protein LOC113052197	XR_011009630.1
B4G0235	11.14	6.32	SLC41A2	Solute carrier family 41 member 2	XM_005168525.5
A9G0841	11.13	3.20	PKP4	Plakophilin-4	
B15G0516	11.13	8.58	CPD	Carboxypeptidase D	XM_021474349.2
A5G0221	11.13	60.12	PEBP1	Phosphatidylethanolamine-binding protein 1	NM_213323.2
A19G0469	11.12	22.53	LYPLA2	Acyl-protein thioesterase 2	NM_200749.2
A14G0028	11.12	33.64	RTASE	Probable RNA-directed DNA polymerase from transposon BS	
A21G0092	11.12	19.64	PHYHD1	Phytanoyl-CoA dioxygenase domain-containing protein 1	NM_001007445.1
A11G0241	11.12	9.44	TADA3	Transcriptional adapter 3	NM_001308617.1
A5G0458	11.12	9.58	RABL6	Rab-like protein 6	XM_021476478.2
B16G0619	11.12	54.55	DTNBP1A	Dysbindin-A	XM_005158097.4
B3G0284	11.11	5.02	MRPS7	28S ribosomal protein S7, mitochondrial	NM_001033596.1
B11G0229	11.11	13.46	TMEM259	Membralin	NM_001083573.1
B3G1277	11.11	23.55	IL12RB2	Interleukin-12 receptor subunit beta-2	NM_001365786.1
B8G1003	11.10	10.31	IKBKB	Inhibitor of nuclear factor kappa-B kinase subunit beta	NM_001123265.1
A6G0009	11.10	3.09	AP1M2	AP-1 complex subunit mu-2	NM_205714.1
A10G0676	11.10	2.33	LOC113054137	A0A6P6KV52_CARAU uncharacterized protein LOC113054137 isoform X2	
B20G0771	11.09	32.62	NAPB	Beta-soluble NSF attachment protein	NM_001080702.2
A21G0392	11.09	8.02	DIABLO	Diablo IAP-binding mitochondrial protein	
B6G0657	11.09	12.02	OS9	Protein OS-9	NM_001075108.1
B5G0692	11.09	6.16	EMC6	ER membrane protein complex subunit 6	
A8G0218	11.09	13.29	MED27	Mediator of RNA polymerase II transcription subunit 27	NM_200660.1

B22G0415	11.08	3.66	LOC113078163	A0A6P6NAM5_CARAU uncharacterized protein LOC113078163	XM_021469541.2
A2G0065	11.07	14.50	PRKACB	cAMP-dependent protein kinase catalytic subunit beta	NM_001034976.1
A14G0783	11.07	12.63	HDAC3	Histone deacetylase 3	NM_200990.1
B22G0595	11.07	2.49	ARL4C	ADP-ribosylation factor-like protein 4C	NM_001017749.1
B5G1246	11.07	45.50	UBIQP	Polyubiquitin	
B15G0371	11.07	9.17	ATF5	Cyclic AMP-dependent transcription factor ATF-5	XM_690545.8
B18G0375	11.07	3.98	NAT10	RNA cytidine acetyltransferase	NM_200644.1
B12G0298	11.07	4.17	OSBPL6	Oxysterol-binding protein-related protein 6	XM_068224543.1
B6G0506	11.07	9.94	TOMM70	Mitochondrial import receptor subunit TOM70	NM_200002.1
A25G0323	11.07	7.72	PYURF	Protein preY, mitochondrial	NM_001089372.1
B22G0081	11.07	14.75	RBBP5	Retinoblastoma-binding protein 5	XM_005168268.5
B2G0154	11.06	1.98	A0A671QEX1	A0A671QEX1_9TELE G_PROTEIN_RECEP_F1_2 domain-containing protein	
B13G0366	11.06	11.07	GRIN2	GRIN2-like protein	NM_212659.1
A10G0558	11.06	5.45	NLE1	Notchless protein homolog 1	NM_001328516.1
A2G0824	11.06	7.08	PRKCI	Protein kinase C iota type	NM_131855.2
A1G0173	11.06	21.52	GNG5	G(O) subunit gamma-5	NM_001200040.1
B18G0027	11.06	8.89	YIF1B	Protein YIF1B	XM_005169969.5
A17G0411	11.06	5.72			XM_005158552.5
B11G0225	11.06	15.89	SID1	E9QH29_DANRE Secreted immunoglobulin domain 1	NM_212764.1
B8G0510	11.06	16.41	ARIH2	E3 ubiquitin-protein ligase ARIH2	XM_009303988.4
B6G1012	11.06	14.58	DCTN2	Dynactin subunit 2	XM_005166169.4
B12G0577	11.06	25.56	OPLAH	5-oxoprolinase	XM_005156046.5
B7G0664	11.06	5.53	SMAD3	Mothers against decapentaplegic homolog 3	NM_131571.2
A11G0057	11.05	5.14	CLDN22	Claudin-22	XM_021479680.2
B18G0530	11.05	6.40	NCOA5	Nuclear receptor coactivator 5	NM_001045069.1
A15G0051	11.04	17.52	IWS1-A	Protein IWS1 homolog A	XM_005157821.5
B4G0397	11.04	12.69	NRF1	Nuclear respiratory factor 1	NM_001328540.1
A2G0828	11.04	10.92	DAPK3	Death-associated protein kinase 3	XR_011006884.1
B20G0347	11.04	10.68	TMEM165	Transmembrane protein 165	NM_212683.2
B12G0390	11.04	17.49	ABLIM2	Actin-binding LIM protein 2	XM_068224765.1
A7G0620	11.04	25.52	CEBPA	enhancer-binding protein alpha	NM_131885.2
A3G1111	11.03	34.50	HAGH	Hydroxyacylglutathione hydrolase, mitochondrial	NM_200043.3
A7G0369	11.03	4.38	SMIM20	Small integral membrane protein 20	NM_001302624.1
B10G0173	11.03	12.77	GOLPH3	Golgi phosphoprotein 3	XM_021472219.2
B1G0093	11.03	18.98	TUBA	Tubulin alpha chain	NM_200691.1
B19G0342	11.03	8.09	PLEC	Plectin	NM_001430891.1
B10G0749	11.02	2.63	MRPS24	28S ribosomal protein S24, mitochondrial	NM_001020682.2
A25G0464	11.02	12.66	CNOT2	CCR4-NOT transcription complex subunit 2	NM_001089524.1
B14G0172	11.02	12.89	PFDN6	Prefoldin subunit 6	NM_200513.1
A10G0198	11.01	4.23	DUSP11	RNP complex-1-interacting phosphatase	NM_001328370.1
A20G0029	11.01	14.12	ACTR10	Actin-related protein 10	NM_200170.1
A6G0864	11.01	4.77	STX16	Syntaxin-16	XM_005172117.5

A8G0181	11.01	21.48	SLC6A8	Sodium- and chloride-dependent creatine transporter 1	NM_001327868.1
A11G0032	11.00	6.56	LDB1	LIM domain-binding protein 1	
B8G0299	11.00	6.73	ARPC5L	3 complex subunit 5-like protein	NM_200578.2
B22G0809	11.00	8.95	NEK7	threonine-protein kinase Nek7	NM_001003617.1
B10G0271	11.00	18.04	LETM2	LETM1 domain-containing protein LETM2, mitochondrial	XM_001339351.9
B7G0930	11.00	5.60			
A1G0322	11.00	10.02	AIMP1	Aminoacyl tRNA synthase complex-interacting multifunctional protein 1	NM_001045851.1
B2G1144	11.00	18.27	PDE4B	cAMP-specific 3',5'-cyclic phosphodiesterase 4B	XM_009298245.4
B4G0863	10.99	41.67	MS4A4A	Membrane-spanning 4-domains subfamily A member 4A	XM_003199992.6
B25G0302	10.99	10.60	CTDSPL2A	CTD small phosphatase-like protein 2-A	NM_001077544.1
A12G0682	10.99	4.19	CABCOCO1	Ciliary-associated calcium-binding coiled-coil protein 1	NM_001177463.1
B5G1125	10.99	3.39	SLC25A15	Mitochondrial ornithine transporter 1	XM_005165177.5
A16G0026	10.99	5.49	TMEM222	Transmembrane protein 222	NM_001013316.3
B11G0206	10.98	13.95	ZFYVE9	Zinc finger FYVE domain-containing protein 9	XM_680236.6
B13G0930	10.98	12.30			NM_001251832.1
B14G0060	10.98	2.39	SMYD5	Histone-lysine N-trimethyltransferase SMYD5	NM_001004614.2
B7G0235	10.98	4.09	SULT2B1	Sulfotransferase 2B1	NM_001076656.1
B5G1263	10.98	8.62	KCTD10	POZ domain-containing adapter for CUL3-mediated RhoA degradation protein 3	NM_213478.2
A24G0054	10.97	4.29	STXB	Stonustoxin subunit beta	XM_068217267.1
B9G0602	10.97	14.89	LOC107555238	A0A672RP63_SINGR Uncharacterized LOC107555238	
B4G0621	10.97	5.22	TPRKB	KEOPS complex subunit TPRKB	NM_001007373.1
B7G0310	10.97	7.56	LARP7	La-related protein 7	NM_199930.2
B24G0212	10.97	7.41	MCM4-A	DNA replication licensing factor mcm4-A	NM_198913.2
B18G0437	10.96	9.50	NUDT21	Cleavage and polyadenylation specificity factor subunit 5	NM_201117.1
A20G0725	10.96	5.97			
A3G0784	10.96	23.18	AKT1S1	Proline-rich AKT1 substrate 1	XM_687419.10
B23G0907	10.96	13.11	ZGC:65873	UPF0690 protein C1orf52 homolog	NM_200542.2
A7G0536	10.95	5.08	PRKDC	DNA-dependent protein kinase catalytic subunit	XM_009303401.3
B24G0375	10.95	10.69	SLC51A	Organic solute transporter subunit alpha	NM_001004546.1
B2G1089	10.95	2.55	DNTS_033861	A0A553Q641_9TELE Uncharacterized protein	
A5G0770	10.95	10.09	MTMR12	Myotubularin-related protein 12	NM_001113613.1
A23G0774	10.95	9.29	TCB1	Transposable element Tcb1 transposase	XR_011017426.1
A12G0555	10.95	10.86	ACBD4	Acyl-CoA-binding domain-containing protein 4	NM_213095.1
B6G0289	10.94	24.48	NDUFS1	NADH-ubiquinone oxidoreductase 75 kDa subunit, mitochondrial	NM_001007765.1
A2G0514	10.94	10.39	ANGPTL4	Angiopoietin-related protein 4	NM_001256203.1
B1G0091	10.94	8.11	SPRY2	Protein sprouty homolog 2	XM_005160285.5

B22G0925	10.94	6.14	JAGN1B	Protein jagunal homolog 1-B	NM_001003584.2
A5G0636	10.94	5.29	ANKRA2	Ankyrin repeat family A protein 2	NM_213129.3
A3G0919	10.94	6.51	TMEM11	Transmembrane protein 11, mitochondrial	NM_001017400.1
B10G0796	10.94	4.84	SLC25A1B	Tricarboxylate transport protein B, mitochondrial	NM_001326528.1
B15G0264	10.94	17.44	PRKD1	threonine-protein kinase D1	
B9G0317	10.93	50.53	FUNDC1	FUN14 domain-containing protein 1	NM_001002711.2
A19G0032	10.93	13.60	VPS52	Vacuolar protein sorting-associated protein 52 homolog	XM_068215267.1
B12G0074	10.93	11.84	ADK	Adenosine kinase	NM_198802.1
B24G0509	10.93	14.33	PDIA4	Protein disulfide-isomerase A4	NM_199779.2
B7G0629	10.93	3.63	DPEP2	Dipeptidase 2	XM_003198973.6
B7G0241	10.92	8.82	SLC22A23	Solute carrier family 22 member 23	XM_694062.10
A10G0508	10.92	13.00	HTATSF1	HIV Tat-specific factor 1 homolog	NM_201203.1
A11G0180	10.92	6.43	MRPL45	39S ribosomal protein L45, mitochondrial	NM_001020571.1
A3G0556	10.92	26.08	GPATCH8	G patch domain-containing protein 8	NM_001328037.1
A25G0652	10.92	8.88	IGKV3-15	Immunoglobulin kappa variable 3-15	NM_001045408.1
A21G0144	10.92	4.19	REXO4	RNA exonuclease 4	XM_002665867.6
B2G1215	10.92	9.53	THOC1	THO complex subunit 1	NM_201324.1
B22G0986	10.92	12.01	TC1A	Transposable element Tc1 transposase	
A23G0364	10.92	24.22	UBA1	Ubiquitin-like modifier-activating enzyme 1	XM_005162052.5
B16G0442	10.91	18.03	GSTK1	Glutathione S-transferase kappa 1	XM_005158344.5
B15G0054	10.91	6.33	RSRC1	Arginine-related protein 53	NM_212947.2
A2G0708	10.91	15.05	G5714_001953	A0A7J6DDU2_9TELE Uncharacterized protein	
A7G0352	10.91	24.99	PPP1R14B	Protein phosphatase 1 regulatory subunit 14B	XM_068222095.1
B22G0821	10.91	0.28	RGS21	Regulator of G-protein signaling 21	XM_001923553.8
B20G0744	10.90	19.05	SNX9	Sorting nexin-9	XM_021468448.2
B19G0032	10.90	2.78	G5714_019160	A0A7J6C1W1_9TELE Uncharacterized protein	
B21G0589	10.90	15.73	NNT	NAD(P) transhydrogenase, mitochondrial	NM_214756.1
A2G0164	10.90	8.98	UCK2B	Uridine-cytidine kinase 2-B	NM_199764.2
A17G0320	10.90	12.48	CCDC32	Coiled-coil domain-containing protein 32	NM_001198753.1
B11G0026	10.89	29.99	HRH1	Histamine H1 receptor	
A12G0116	10.89	6.82	PPP2R2D	threonine-protein phosphatase 2A 55 kDa regulatory subunit B delta isoform	NM_212880.2
A1G0603	10.89	5.12	DPCD	Protein DPCD	NM_001045377.1
B24G0131	10.89	9.59	IGKV3-11	Immunoglobulin kappa variable 3-11	XM_017354192.3
B14G0798	10.88	10.54	PHYHD1	Phytanoyl-CoA dioxygenase domain-containing protein 1	
A3G1195	10.88	10.75	PRPSAP1	Phosphoribosyl pyrophosphate synthase-associated protein 1	NM_001002199.1
B17G0212	10.88	8.50	SCCPDH	Saccharopine dehydrogenase-like oxidoreductase	NM_001002359.1
B16G0164	10.88	14.49	BOLA1	BolA-like protein 1	NM_001003557.2

B4G0776	10.88	17.94	TCB1	Transposable element Tcb1 transposase	XM_021470094.2
B16G1008	10.88	10.46	PRPF3	U6 small nuclear ribonucleoprotein Prp3	NM_205748.2
A22G0258	10.88	5.20	PCYT2	Ethanolamine-phosphate cytidylyltransferase	NM_001006037.1
B21G0174	10.88	16.29	PAFAH1B1B	Lissencephaly-1 homolog B	NM_201346.1
B21G0031	10.88	5.57	CDC23	Cell division cycle protein 23 homolog	NM_200933.1
B20G0203	10.87	9.32	PSMC1	26S proteasome regulatory subunit 4	NM_001002091.1
B1G1019	10.87	5.93	DENND1B	DENN domain-containing protein 1B	XM_005168402.5
B5G0899	10.87	12.33	PTPA	threonine-protein phosphatase 2A activator	NM_001003425.2
A13G0236	10.87	7.28	MAP3K4	Mitogen-activated protein kinase kinase kinase 4	NM_001017851.2
A4G0449	10.86	9.46	K0930	Uncharacterized protein KIAA0930 homolog	NM_001327783.1
A1G0138	10.86	8.07	SLC25A10	Mitochondrial dicarboxylate carrier	NM_201172.1
B17G0036	10.86	15.18	GCHFR	GTP cyclohydrolase 1 feedback regulatory protein	
A25G0447	10.86	10.93	UBE2Q2	Ubiquitin-conjugating enzyme E2 Q2	NM_001328201.1
B3G1645	10.86	4.18	SOX9-B	Transcription factor Sox-9-B	NM_131644.1
B22G1136	10.86	9.32	RTEL1	Regulator of telomere elongation helicase 1	NM_001013310.2
B24G0182	10.86	1.56	CCR7	C-C chemokine receptor type 7	NM_001002675.1
B19G0228	10.86	11.27	UBAP2L	Ubiquitin-associated protein 2-like	XM_021467965.2
A22G0310	10.86	7.92	MFNG	Beta-1,3-N-acetylglucosaminyltransferase manic fringe	NM_001007788.1
A20G0017	10.86	12.80	YY1	Transcriptional repressor protein YY1	XM_005170229.5
A7G0455	10.86	5.45	TSG101	Tumor susceptibility gene 101 protein	XM_002662878.6
B24G0605	10.86	4.00	CHMP4B	Charged multivesicular body protein 4b	NM_001017844.1
B10G0424	10.85	5.02	CXADR	Coxsackievirus and adenovirus receptor homolog	NM_152948.2
A10G0730	10.85	15.03	RASGEF1BB	Ras-GEF domain-containing family member 1B-B	NM_001003479.1
B7G0669	10.85	7.97	UACA	Uveal autoantigen with coiled-coil domains and ankyrin repeats	XM_021477874.2
B20G0197	10.84	15.43	PSMA6	Proteasome subunit alpha type-6	NM_001002589.1
A7G0909	10.84	18.28	GMPR2	GMP reductase 2	NM_001040304.2
B16G0752	10.84	9.67	VARS1	Valine--tRNA ligase	NM_001311346.1
A11G0713	10.84	17.47	AGTRAP	Type-1 angiotensin II receptor-associated protein-like	NM_001001823.2
A16G0181	10.84	9.14	RBM42	RNA-binding protein 42	NM_001003850.1
A8G0821	10.83	1.65	LMO4	LIM domain transcription factor LMO4	XM_002663251.7
A6G0410	10.83	25.71	LOC107710016	A0A673N461_9TELE Uncharacterized LOC107710016	NM_001083876.1
A8G0198	10.83	3.54	BCAS2	Pre-mRNA-splicing factor SPF27	NM_001007774.1

A15G0723	10.83	2.49	POL	Retrovirus-related Pol polyprotein from transposon 412	
A7G0818	10.83	12.37	NUCB2	Nucleobindin-2	NM_201493.1
A16G0462	10.83	7.01	HORMAD1	HORMA domain-containing protein 1	NM_001002357.1
A16G0465	10.82	10.77	CTSS	Cathepsin S	NM_001024409.2
A4G0744	10.82	2.02	MGST1	Microsomal glutathione S-transferase 1	NM_001005957.1
B12G0383	10.82	13.28	WACA	WW domain-containing adapter protein with coiled-coil	XM_009306588.3
A3G1082	10.82	7.11	MCM5	DNA replication licensing factor mcm5	NM_178436.3
B14G0299	10.82	11.44	CLINT1	Clathrin interactor 1	NM_001003412.1
A6G0069	10.82	5.15	PXMP4	Peroxisomal membrane protein 4	NM_001326556.1
B4G0094	10.81	15.92	BCAP29	B-cell receptor-associated protein 29	NM_001024220.1
B22G0077	10.81	35.59	DRAM2	DNA damage-regulated autophagy modulator protein 2	NM_001002135.2
B9G0573	10.81	8.31	PHF11	PHD finger protein 11	
B5G0613	10.81	6.14	NOL6	Nucleolar protein 6	NM_001161511.1
B23G0336	10.81	12.76	SRGAP3	SLIT-ROBO Rho GTPase-activating protein 3	XM_684637.8
B23G0124	10.81	0.82	CDH26	Cadherin-like protein 26	XM_017353725.3
B25G0157	10.81	0.44	IGKV1-27	Immunoglobulin kappa variable 1-27	NM_001045408.1
A25G0305	10.81	2.19	TIGARA	Probable fructose-2,6-bisphosphatase TIGAR A	NM_001039836.1
B2G0731	10.81	6.01	IMPA1	Inositol monophosphatase 1	NM_001002745.1
B20G0286	10.80	25.00	TNFAIP2	Tumor necrosis factor alpha-induced protein 2	NM_001030216.1
A11G0761	10.80	6.66	LRP1	Low-density lipoprotein receptor-related protein 1	NM_199832.2
A16G0380	10.80	38.77	PNISR	serine-rich protein PNISR	NM_001083837.1
B23G0472	10.80	12.31	SDF4	45 kDa calcium-binding protein	NM_213087.2
A2G0634	10.80	4.11	YES1	Tyrosine-protein kinase yes	NM_001013270.3
B1G0732	10.79	0.21	LOC113089746	A0A6P6NTV1_CARAU uncharacterized protein LOC113089746	XM_021476859.2
A25G0185	10.79	6.25	PSMD13	26S proteasome non-ATPase regulatory subunit 13	NM_200948.1
B11G0678	10.79	10.74	SLC45A1	Proton-associated sugar transporter A	XM_009305774.3
A17G0495	10.79	4.34	DHRS4	reductase SDR family member 4 (Fragment)	NM_200553.1
B8G1062	10.78	6.25	PGGT1B	Geranylgeranyl transferase type-1 subunit beta	NM_001030255.2
B1G0680	10.78	4.91	MED19B	Mediator of RNA polymerase II transcription subunit 19-B	NM_001003416.2
A6G0053	10.78	18.01	TXNRD3	Thioredoxin reductase 3	NM_183072.2
B8G0761	10.78	15.97	MCCC2	Methylcrotonoyl-CoA carboxylase beta chain, mitochondrial	NM_001113652.1
A3G0807	10.78	10.54	CYTH3	Cytohesin-3	XM_690136.8
B1G0103	10.78	10.91	CNPPD1	Protein CNPPD1	NM_199928.1
A15G0209	10.78	37.72	APLP2	Amyloid beta precursor like protein 2	XM_001342885.9
A17G0560	10.77	11.39	SMNDC1	Survival of motor neuron-related-splicing factor 30	NM_212601.2
B22G0787	10.77	6.00	PLPP2	Phospholipid phosphatase 2	NM_199953.2
A3G0002	10.77	8.93	WBP11	WW domain-binding protein 11	XM_068218398.1

A13G0310	10.77	23.78	HIF1A	Hypoxia-inducible factor 1-alpha	XM_009307206.4
A7G0299	10.77	8.19	TRAPPC2L	Trafficking protein particle complex subunit 2-like protein	NM_001003506.2
A5G0809	10.77	6.91	MOB1B	MOB kinase activator 1B	NM_200200.1
B10G0238	10.77	5.84	PPP2R2A	threonine-protein phosphatase 2A 55 kDa regulatory subunit B alpha isoform	NM_001309438.1
B21G0297	10.76	10.54	P33MONOX	Putative monooxygenase p33MONOX	NM_001039994.3
B13G0194	10.76	6.06	GBF1	Golgi-specific brefeldin A-resistance guanine nucleotide exchange factor 1	NM_001115133.1
B2G1155	10.76	5.78	TSEN15	tRNA-splicing endonuclease subunit Sen15	NM_001100090.1
A24G0357	10.76	7.55	NDUFB4	NADH dehydrogenase [ubiquinone] 1 beta subcomplex subunit 4	
B3G0492	10.76	34.14	HMCN1	Hemicentin-1	NM_001115104.1
A2G0416	10.76	6.16	PRG4	Proteoglycan 4	NM_212753.2
B20G0334	10.76	15.06	BRF1	Transcription factor IIIB 90 kDa subunit	NM_199889.1
B25G0396	10.76	5.93	PRR5L	Proline-rich protein 5-like	XM_680728.9
A23G0893	10.75	3.92	EIF4E2	Eukaryotic translation initiation factor 4E type 2	NM_200759.2
A11G0754	10.75	26.55	HOXC6B	Homeobox protein Hox-C6b	NM_201078.1
A19G0699	10.75	9.28	EFNA1	Ephrin-A1	NM_200597.2
B5G1229	10.75	7.63	CMKLR1	Chemerin-like receptor 1	
A7G1328	10.75	9.72	NEK1	threonine-protein kinase Nek1	XM_017356939.3
B24G0408	10.75	11.97	COPS5	COP9 signalosome complex subunit 5	NM_200725.1
A17G0597	10.75	5.35	SPTLC2	Serine palmitoyltransferase 2	NM_001020619.2
B5G1183	10.75	3.76	LOC107672636	A0A671MQN9_9TELE Uncharacterized LOC107672636	NM_001017892.1
B23G0570	10.75	5.70	SRM	Spermidine synthase	NM_201034.2
B21G0239	10.74	16.93	MED31	Mediator of RNA polymerase II transcription subunit 31	NM_001002417.2
A13G0200	10.74	2.19	ATL1	Atlastin-1	NM_001327856.1
B8G0085	10.74	6.64	NDUFA7	NADH dehydrogenase [ubiquinone] 1 alpha subcomplex subunit 7	
B19G0715	10.74	7.99	SRFBP1	Serum response factor-binding protein 1	
A3G0840	10.74	8.18	CIITA	MHC class II transactivator	XM_001343036.7
B15G0770	10.73	3.78	G5714_015348	A0A7J6CBB3_9TELE Uncharacterized protein	
B20G0134	10.73	5.19	TTC13	Tetratricopeptide repeat protein 13	NM_001130627.1
A12G0466	10.73	18.81	MAFF	Transcription factor MafF	
B16G0075	10.73	24.01	ATP6V1C1A	V-type proton ATPase subunit C 1-A	NM_201322.1
A5G1064	10.72	8.13	ALG9	Alpha-1,2-mannosyltransferase ALG9	XM_009301841.4
A18G0583	10.72	7.15	UTP4	U3 small nucleolar RNA-associated protein 4 homolog	NM_213282.2
B3G0838	10.72	14.02	SRRM2	arginine repetitive matrix protein 2	XM_021470715.2
B15G0350	10.72	12.88	CNRB	CLPTM1-like membrane protein cnrB	NM_001003647.2
A19G0613	10.72	11.19	TPM4	Tropomyosin alpha-4 chain	XM_005159376.3

B8G0443	10.72	25.52	MPEG1	Macrophage-expressed gene 1 protein	NM_212737.1
A25G0787	10.71	11.65	PDHX	Pyruvate dehydrogenase protein X component, mitochondrial	XM_005170734.5
B2G1074	10.71	8.04	POL	Retrovirus-related Pol polyprotein from transposon 412	
B12G0227	10.71	14.17	RAB40B	Ras-related protein Rab-40B	NM_001002524.1
A23G0722	10.71	4.62	PLEKHG5	Pleckstrin homology domain-containing family G member 5	XM_068216936.1
B15G0359	10.71	15.73	CLTC	Clathrin heavy chain 1	NM_001194978.1
A21G0688	10.71	16.86	NUP98	Nuclear pore complex protein Nup98-Nup96	NM_200685.2
A23G0562	10.71	13.40	SLC25A33	Solute carrier family 25 member 33	NM_213157.1
A19G0446	10.70	22.88	YRK	Proto-oncogene tyrosine-protein kinase Yrk	NM_001365553.1
B24G0241	10.70	5.19	RBIS	Ribosomal biogenesis factor	
A2G0800	10.70	9.48	PDCD10B	Programmed cell death protein 10-B	NM_212933.1
B1G0686	10.70	8.45	UNC93B1	Protein unc-93 homolog B1	XM_009293277.4
B19G0623	10.70	5.00	GALNT4	Polypeptide N-acetylgalactosaminyltransferase 4	NM_001044778.2
B8G0285	10.69	4.80	GPKOW	G-patch domain and KOW motifs-containing protein	NM_001007207.1
B17G0457	10.69	6.63	GALE	UDP-glucose 4-epimerase	XM_005158523.4
A2G0865	10.69	6.50	DHRS1	reductase SDR family member 1	NM_001002205.1
B13G0615	10.69	406.62	LGALSL-B	Galectin-related protein B	NM_001001946.4
B11G0299	10.69	6.85	LRIG1	Leucine-rich repeats and immunoglobulin-like domains protein 1	NM_201303.1
B8G0692	10.69	6.88	CA021	Uncharacterized protein C1orf21 homolog	NM_001004593.1
B14G0047	10.68	6.84	SLC43A1	Large neutral amino acids transporter small subunit 3	NM_001327986.1
B4G0566	10.68	1.05	LOC113081361	A0A6P6NL79_CARAU uncharacterized protein LOC113081361	
B8G0487	10.68	50.68	G5714_009162	A0A7J6CRP2_9TELE Uncharacterized protein	NM_001126417.1
A15G0521	10.68	5.87	TIMM50	Mitochondrial import inner membrane translocase subunit TIM50	NM_200665.2
B14G0250	10.68	8.71	NKAPL	NKAP-like protein	NM_001003414.1
A17G0364	10.67	11.55	STRN3	Striatin-3	XM_005158784.5
A14G0438	10.67	2.26	LOC107668579	A0A671MT00_9TELE SUMO-interacting motif-containing protein 1-like	
A7G1323	10.67	32.97	G5714_008691	A0A7J6CWM0_9TELE ULP_PROTEASE domain-containing protein	
A24G0128	10.67	6.56	RHBDL1	Rhomboid-related protein 1	XM_017353796.3
B14G0242	10.67	12.11	PDZD11	PDZ domain-containing protein 11	NM_212957.2
B11G0722	10.66	2.07	TMEM63B	CSC1-like protein 2	XM_021472385.2
B23G0920	10.66	3.37	SH3D19	SH3 domain-containing protein 19	XR_002456606.2
A11G0332	10.66	7.30	SOX11-A	Transcription factor Sox-11-A	NM_001114429.1
A1G0337	10.66	2.19	MSXB	Homeobox protein MSH-B	NM_131260.1
A14G0780	10.66	8.25	RHOG	Rho-related GTP-binding protein RhoG	

A7G0026	10.66	17.15	CAAP1	Caspase activity and apoptosis inhibitor 1	NM_001326703.1
B3G0219	10.65	33.61	MYH9	Myosin-9	XM_001920063.8
A5G0344	10.65	20.37	CHMP1B	Charged multivesicular body protein 1b	XM_009301289.4
A5G0404	10.65	6.29	TRMT2A	tRNA (uracil-5-)-methyltransferase homolog A	NM_199929.1
B5G1126	10.64	6.16	MRPS31	28S ribosomal protein S31, mitochondrial	NM_001128328.1
A12G0016	10.64	22.11	FAM53B	Protein FAM53B	NM_001007187.1
A22G0714	10.64	28.18	TM4SF1	Transmembrane 4 L6 family member 1	NM_001045022.2
B3G1703	10.64	2.67	G5714_002578	A0A7J6D731_9TELE Uncharacterized protein	
B6G0698	10.64	7.29	CCDC174	Coiled-coil domain-containing protein 174	NM_001013488.2
B20G0884	10.64	6.68	MGAT2	Alpha-1,6-mannosyl-glycoprotein 2-beta-N-acetylglucosaminyltransferase	XM_003200772.6
B8G0130	10.64	9.81	PLP2	Proteolipid protein 2	NM_001161445.1
A9G0408	10.64	18.75	DDB_G0268948	Putative methyltransferase DDB_G0268948	NM_001044782.2
A3G0712	10.64	11.66	RRAS	Ras-related protein R-Ras	NM_001005931.2
A16G0422	10.64	8.06	CD40	Tumor necrosis factor receptor superfamily member 5	XM_684398.10
A17G0306	10.64	38.09	LIN52	Protein lin-52 homolog	NM_001077796.1
A8G0866	10.63	25.64	RAB7	Ras-related protein rab7	NM_001002178.1
A8G0375	10.63	14.85			
B2G0092	10.63	10.55	CTSZ	Cathepsin Z	XM_002660961.7
B22G0970	10.63	37.50	SLC6A6	Sodium- and chloride-dependent taurine transporter	NM_001037661.1
A9G0801	10.63	5.85	MAP3K20	Mitogen-activated protein kinase kinase kinase 20	NM_001327940.1
A21G0847	10.62	12.16	AFF4	FMR2 family member 4	XM_068216156.1
A24G0053	10.62	6.50	A0A673G3H5	A0A673G3H5_9TELE AAA domain-containing protein	XM_068217267.1
B22G0711	10.62	6.60	TMEM161A	Transmembrane protein 161A	XM_068216285.1
A14G0585	10.62	8.05	IRF2	Interferon regulatory factor 2	NM_001320188.1
A16G0212	10.62	4.35	MTX1	Metaxin-1	NM_001128238.1
A1G0761	10.61	16.68	IPO5	Importin-5	XM_009305394.3
A8G0117	10.61	10.46	CIAO1	Probable cytosolic iron-sulfur protein assembly protein ciao1	NM_200147.2
B15G0052	10.61	6.05	PANX1	Pannexin-1	NM_200916.1
A12G0856	10.61	6.14	WDR45B	WD repeat domain phosphoinositide-interacting protein 3	NM_200240.2
A14G0411	10.61	3.57	MGST2	Microsomal glutathione S-transferase 2	
A22G0606	10.61	5.06	JAGN1B	Protein jagunal homolog 1-B	NM_001003584.2
A9G0596	10.61	3.32	NUP35	Nucleoporin NUP35	NM_200066.1
B10G0720	10.61	7.87	MCTP1	Multiple C2 and transmembrane domain-containing protein 1	XM_002663670.6
A11G0436	10.60	15.75	ARHGEF10L	Rho guanine nucleotide exchange factor 10-like protein	XM_002663787.7
A22G0155	10.60	6.29	CD84	SLAM family member 5	XM_068216403.1
B13G0787	10.60	19.54	MDGA1	MAM domain-containing glycosylphosphatidylinositol anchor protein 1	NM_001017542.2
A10G0706	10.60	12.08	PSTPIP2	Proline-serine-threonine phosphatase-interacting protein 2	XM_009305134.4
B6G0194	10.59	4.97	RGN	Regucalcin	NM_205746.1

A4G0682	10.59	8.80	NRF1	Nuclear respiratory factor 1	NM_131680.2
B3G1260	10.59	12.33	GCAT	2-amino-3-ketobutyrate coenzyme A ligase, mitochondrial	NM_001172554.1
A3G0408	10.59	2,402.96	HBA	Hemoglobin subunit alpha	NM_001033093.2
B1G0925	10.59	14.87	XPO1	Exportin-1	XM_009294702.4
A22G0181	10.59	11.34	SMARCD1	SNF-related matrix-associated actin-dependent regulator of chromatin subfamily D member 1	NM_198358.1
B4G0481	10.59	5.79	DEF6	Differentially expressed in FDCP 6 homolog	XM_009300326.4
A3G0752	10.59	4.50	CCDC43	Coiled-coil domain-containing protein 43	NM_200658.2
A16G0416	10.58	10.95	MLF2	Myeloid leukemia factor 2	NM_001025516.1
A21G0274	10.58	4.57	NOC4L	Nucleolar complex protein 4 homolog	NM_001024397.1
B18G0010	10.58	7.89	RAP2A	Ras-related protein Rap-2a	NM_001001729.2
A7G0519	10.58	17.57	PHKB	Phosphorylase b kinase regulatory subunit beta	XM_005166403.5
A18G0021	10.58	13.62	UBL7	Ubiquitin-like protein 7	XM_068214735.1
A25G0050	10.58	10.09	HBP1	HMG box-containing protein 1	NM_001024431.2
B5G0568	10.58	10.02	HINT2	Adenosine 5'-monophosphoramidase HINT2	
A6G0830	10.58	16.78	SUN2	SUN domain-containing protein 2	XM_001919656.7
A11G0632	10.58	20.97	MRPS16	28S ribosomal protein S16, mitochondrial	XM_005169409.5
B14G0495	10.57	8.26	REST	RE1-silencing transcription factor	NM_001353866.1
A12G0431	10.57	9.72	STX4	Syntaxin-4	NM_200221.1
B3G1678	10.56	6.34	A0A672N0M7	A0A672N0M7_SINGR Uncharacterized protein	XM_068220513.1
B7G0532	10.56	10.20	DNAJB6	DnaJ homolog subfamily B member 6	XM_009303365.4
A21G0737	10.56	4.36	MRPS17	28S ribosomal protein S17, mitochondrial	
B7G1247	10.56	13.46	CD84	SLAM family member 5	
B5G0039	10.56	4.89	RABEPK	Rab9 effector protein with kelch motifs	
A22G0132	10.56	11.00	MYO9B	Unconventional myosin-IXb	NM_001423631.1
B15G0724	10.56	7.18	MRPL44	39S ribosomal protein L44, mitochondrial	NM_001030260.2
A16G0961	10.56	12.08	EAF1	ELL-associated factor 1	NM_001002115.1
A11G0765	10.56	10.96	IFT52	Intraflagellar transport protein 52 homolog	NM_001002705.1
A15G0391	10.56	8.84	DHRS13	reductase SDR family member 13	NM_001007424.2
A7G0968	10.56	2.33	METTL3	N6-adenosine-methyltransferase subunit METTL3	NM_212780.1
A3G0423	10.56	22.54	ICAM2	Intercellular adhesion molecule 2	NM_001098252.2
A8G0836	10.55	10.03	VPS13A	Intermembrane lipid transfer protein VPS13A	XM_009304309.4
B3G0628	10.55	11.95	NMT1	Glycylpeptide N-tetradecanoyltransferase 1	NM_001020480.1
B5G0199	10.55	11.26	MAPK1	Mitogen-activated protein kinase 1	NM_182888.2
B6G1033	10.55	0.70	TGM2	Protein-glutamine gamma-glutamyltransferase 2	
B15G0442	10.55	15.53	USF1	Upstream stimulatory factor 1	XM_005157510.5
A16G0334	10.55	7.29	SCMH1	Polycomb protein SCMH1	NM_001089484.1

B25G0391	10.54	3.55	IL17RE	Interleukin-17 receptor E	XM_002666890.6
A5G0621	10.54	4.13	MS4A4A	Membrane-spanning 4-domains subfamily A member 4A	NM_001327935.1
B20G0563	10.54	3.15	MYCN	N-myc proto-oncogene protein	NM_212614.2
B1G0916	10.54	4.28	ZNF330	Zinc finger protein 330	NM_213373.1
B22G1189	10.54	9.64	RTASE	Probable RNA-directed DNA polymerase from transposon BS	
B14G0740	10.54	7.69	PTCD3	Pentatricopeptide repeat domain-containing protein 3, mitochondrial	NM_001037372.2
B7G0770	10.53	5.43	EXOSC6	Exosome complex component MTR3	
A4G0632	10.53	8.08	WASHC3	WASH complex subunit 3	NM_200173.1
A1G0794	10.53	10.55	CNPPD1	Protein CNPPD1	NM_199928.1
A8G0105	10.53	21.25	PPTC7	Protein phosphatase PTC7 homolog	NM_001007378.1
A3G0035	10.53	2.92	LOC113088892	A0A6P6NT19_CARAU uncharacterized protein LOC113088892	XM_068218714.1
A11G0544	10.53	4.12	RRP9	U3 small nucleolar RNA-interacting protein 2	XM_680794.9
B16G0725	10.52	3.22	G5714_016170	A0A7J6C7M3_9TELE Uncharacterized protein	
B19G0016	10.52	8.73	GLMP	Glycosylated lysosomal membrane protein	
A23G0667	10.52	58.72	TUBA1A	Tubulin alpha-1A chain	NM_001105126.2
B12G0078	10.52	3.14	TRIM16	Tripartite motif-containing protein 16	
B21G0352	10.52	20.20	HBEGF	Proheparin-binding EGF-like growth factor	XM_005161297.5
A5G0644	10.52	9.57	PTGER4	Prostaglandin E2 receptor EP4 subtype	NM_001128367.1
B1G1177	10.52	10.02	BFAR	Bifunctional apoptosis regulator	
A12G0642	10.52	8.21	ROHU_005260	A0A498NSF3_LABRO Transcription factor Adf-1-like isoform X2	XM_678327.9
A13G0688	10.52	8.43	WASHC2	WASH complex subunit 2	NM_199917.1
B2G1169	10.52	9.22	FYTTD1	UAP56-interacting factor	
A7G0876	10.52	9.56	ARL2	ADP-ribosylation factor-like protein 2	NM_001005947.1
B24G0273	10.51	5.22	HCCS	Holocytochrome c-type synthase	NM_201451.1
A16G0716	10.51	5.81	CXCR3	C-X-C chemokine receptor type 3	
A5G1080	10.51	4.96	CDC26	Anaphase-promoting complex subunit CDC26	XM_068221152.1
B7G1172	10.51	3.91	POP7	Ribonuclease P protein subunit p20	NM_001006004.1
B21G0321	10.51	5.33	SLC6A7	Sodium-dependent proline transporter	NM_001080580.1
A8G0483	10.51	14.38	AHCYL1	S-adenosylhomocysteine hydrolase-like protein 1	NM_201293.2
B23G0313	10.51	4.98	PRIM1	DNA primase small subunit	NM_201448.1
A7G0353	10.50	8.08	BRMS1LA	Breast cancer metastasis-suppressor 1-like protein-A	NM_200097.1
B1G0062	10.50	10.10	SMIM11	Small integral membrane protein 11	
A7G0226	10.50	4.70			XM_021477371.2
A4G0928	10.50	3.71	SSBP1	Single-stranded DNA-binding protein, mitochondrial	NM_001017806.1
B4G0927	10.49	4.94	CKAP4	Cytoskeleton-associated protein 4	
A13G0289	10.49	8.66	ARG2-A	Arginase, non-hepatic 1	NM_001145584.1

A7G0986	10.49	5.14	TNFSF12	Tumor necrosis factor ligand superfamily member 12	NM_001076607.1
A21G0687	10.49	17.41	NUP98	Nuclear pore complex protein Nup98-Nup96	XM_005161439.5
B1G0649	10.49	17.80	LRPAP1	Alpha-2-macroglobulin receptor-associated protein	NM_201306.2
B7G0634	10.49	32.73	HSD11B2	11-beta-hydroxysteroid dehydrogenase type 2	NM_212720.2
B12G0376	10.49	7.17	CHAC2	Putative glutathione-specific gamma-glutamylcyclotransferase 2	NM_001029957.1
A5G0189	10.49	11.01	MAPK1	Mitogen-activated protein kinase 1	NM_182888.2
B7G0688	10.49	6.67	TSSC4	Protein TSSC4	XM_005168890.5
B21G0976	10.48	0.06	G5714_021098	A0A7J6BVR6_9TELE Uncharacterized protein	
A6G0583	10.48	1.85	AP1M1	AP-1 complex subunit mu-1	XM_021476834.2
B17G0297	10.48	5.61	ALKBH1	Nucleic acid dioxygenase ALKBH1	NM_001020691.1
B22G1090	10.48	11.70	HEBP1	Heme-binding protein 1	NM_001245995.1
A14G0002	10.48	7.14	MCM7-B	DNA replication licensing factor mcm7-B	NM_212569.1
A6G0056	10.48	19.14	TPRA1	Transmembrane protein adipocyte-associated 1 homolog	NM_001030251.1
B8G0029	10.48	8.36	SUSD3	Sushi domain-containing protein 3	
B5G0634	10.48	6.89	CCNH	Cyclin-H	NM_001013453.1
A24G0139	10.48	15.09	PDPK1	3-phosphoinositide-dependent protein kinase 1	NM_205699.1
A23G0798	10.47	20.75	MYL9	Myosin regulatory light polypeptide 9	NM_001006027.1
A14G0552	10.47	12.95	RD3	Protein RD3	NM_001082979.1
B10G0370	10.47	7.05	CHORDC1	Cysteine and histidine-rich domain-containing protein 1	NM_200339.1
A18G0805	10.47	8.70	USP8	Ubiquitin carboxyl-terminal hydrolase 8	XM_009293354.4
A13G0788	10.47	4.16	SPRED2	Sprouty-related, EVH1 domain-containing protein 2	NM_001030175.1
A9G0723	10.47	16.05	RGCC	Regulator of cell cycle RGCC	NM_001002190.2
B21G0429	10.47	5.89	CLDN3	Claudin-3	NM_131767.1
A19G0732	10.47	10.63	EXOSC7	Exosome complex exonuclease RRP42	NM_001017585.1
B4G0394	10.47	9.20	KLHDC10	Kelch domain-containing protein 10	XM_005164695.5
B5G1222	10.46	23.33			NM_198374.2
B7G1020	10.46	3.99	MCTP2	Multiple C2 and transmembrane domain-containing protein 2	XM_021477724.2
A11G0109	10.46	9.28	SLMAP	Sarcolemmal membrane-associated protein	
B3G1753	10.46	10.43	TCB2	Transposable element Tcb2 transposase	
A14G0206	10.46	9.49	MOSPD1	Motile sperm domain-containing protein 1	NM_213369.1
B3G0538	10.46	15.35	UBE2IB	SUMO-conjugating enzyme UBC9-B	NM_131833.2
B18G0769	10.46	3.19	P2RY2	P2Y purinoceptor 2	XM_695238.10
A2G0552	10.46	11.80	HECTD3	E3 ubiquitin-protein ligase HECTD3	NM_001077159.2
A19G0104	10.46	4.03	CCN2	CCN family member 2	XM_005173770.5
A24G0138	10.45	7.79	TSR3	18S rRNA aminocarboxypropyltransferase	NM_001002370.1
B5G1112	10.45	3.60	ZNF703	Zinc finger protein 703	NM_131822.1

B4G0102	10.45	8.46	FGD4	FYVE, RhoGEF and PH domain-containing protein 4	XM_021475195.2
A7G0755	10.45	7.67	FBXO22	F-box only protein 22	NM_001030106.1
B12G0490	10.45	9.70	MLST8	Target of rapamycin complex subunit LST8	NM_001320388.1
A5G0743	10.44	6.88	MRPL1	39S ribosomal protein L1, mitochondrial	NM_001204133.1
A1G0302	10.44	5.16	SPAST	Spastin	NM_212915.2
B4G0265	10.44	7.26	PARP12	Protein mono-ADP-ribosyltransferase PARP12	NM_001115086.1
B2G1311	10.44	2.83	CLDN1	Claudin-1	
A11G0291	10.44	9.71	SLC6A14	Sodium- and chloride-dependent neutral and basic amino acid transporter B(0+)	XM_005172823.5
B7G0519	10.43	9.53	SCRIB	Protein scribble homolog	XM_021477418.2
A3G0934	10.43	16.09	SPNS1	Protein spinster homolog 1	NM_153663.2
A13G0715	10.43	7.87	EIF5	Eukaryotic translation initiation factor 5	NM_194412.2
B7G0942	10.43	11.60	SF1	Splicing factor 1	XM_021477589.2
B24G0554	10.43	10.66	TRAM1L1	Translocating chain-associated membrane protein 1-like 1	NM_153669.2
A1G0553	10.43	5.02	LOC106526644	A0A2I4C9R7_9TELE uncharacterized protein LOC106526644	
A7G1212	10.43	6.13	NIT1	Deaminated glutathione amidase	
A21G0679	10.43	13.16	CCDC82	Coiled-coil domain-containing protein 82	XM_009295663.4
B11G0184	10.43	10.01	VMAC	Vimentin-type intermediate filament-associated coiled-coil protein	NM_205558.1
B18G0117	10.42	2.47	NECTIN1	Nectin-1	NM_001167569.1
A10G0215	10.42	8.48	RAB11FIP1	Rab11 family-interacting protein 1	XM_005164192.5
B7G0353	10.42	8.13	LRCH4	Leucine-rich repeat and calponin homology domain-containing protein 4	XM_009303554.4
A11G0400	10.42	18.92	UTP3	Something about silencing protein 10	NM_213441.1
B7G0945	10.42	23.59	RBM4.1	RNA-binding protein 4.1	NM_213317.3
B7G0848	10.41	9.57	PELP1	Proline-, glutamic acid- and leucine-rich protein 1	XM_001338982.8
A20G0090	10.41	9.87	RBM25	RNA-binding protein 25	XM_009295006.4
B21G0339	10.41	6.16	ZMAT2	Zinc finger matrin-type protein 2	NM_212681.1
B24G0371	10.41	5.02	LOC113073264	A0A6P6MZ67_CARAU Phosphatidylinositol-3-phosphate phosphatase	NM_199791.1
B6G0505	10.41	7.71	TBC1D23	TBC1 domain family member 23	XM_005165941.5
B22G0360	10.41	5.51	LOC113040773	A0A6P6JAG2_CARAU uncharacterized protein LOC113040773	XM_068216428.1
B13G0455	10.41	14.70	CHUK	Inhibitor of nuclear factor kappa-B kinase subunit alpha	NM_001002621.1
A25G0609	10.40	7.10	HRAS	GTPase HRas	NM_001017623.1
B7G0474	10.40	9.21	CIAO2B	Cytosolic iron-sulfur assembly component 2B	NM_001002449.1
B3G0822	10.40	15.87	RUSF1	RUS family member 1	NM_001110453.2
B15G0773	10.40	7.70	SIGLEC1	Sialoadhesin	XM_017351230.3
B21G0793	10.40	10.99	FNBP1	Formin-binding protein 1	XM_021468990.2
B9G0863	10.40	12.73	DYNC1I2A	Dynein, cytoplasmic 1, intermediate chain 2a	XM_017357723.3
B8G0246	10.40	5.72	CRAT	Carnitine O-acetyltransferase	NM_001079997.1

A18G0804	10.40	4.29	TRPM7	Transient receptor potential cation channel subfamily M member 7	NM_001030061.1
A19G0014	10.39	27.28	PSMD3	26S proteasome non-ATPase regulatory subunit 3	NM_200572.1
B14G0082	10.39	3.38	STC2	Stanniocalcin-2	NM_001014827.1
B25G0365	10.39	6.40	AASS	Alpha-aminoadipic semialdehyde synthase, mitochondrial	NM_001173985.1
A5G0440	10.39	5.63	DHFR	Dihydrofolate reductase	NM_131775.1
A20G0817	10.39	4.22	BCL11B	leukemia 11B	XM_002667860.7
B23G0888	10.39	4.89	G5714_022248	A0A7J6BML3_9TELE MADF domain-containing protein	
B17G0504	10.39	10.33	NIPAL3	NIPA-like protein 3	NM_001002634.2
B7G0171	10.39	7.77	BTBD10	POZ domain-containing protein 10	XM_021472027.2
B5G0683	10.39	14.34	CXCL11.6	C-X-C motif chemokine 11-6	
B1G0913	10.39	7.43	SMARCA5	SNF-related matrix-associated actin-dependent regulator of chromatin subfamily A member 5	NM_001081629.2
A3G0084	10.39	5.13	KDELR3	ER lumen protein-retaining receptor 3	NM_200640.2
B12G0297	10.39	8.82	MRPL10	39S ribosomal protein L10, mitochondrial	NM_001013458.2
A5G1105	10.38	5.78	WDR36	WD repeat-containing protein 36	NM_001308831.1
B9G0191	10.38	9.14	CWC22	Pre-mRNA-splicing factor CWC22 homolog	NM_001077569.2
A10G0735	10.38	5.07	MRRF	Ribosome-recycling factor, mitochondrial	NM_001002174.1
A22G0040	10.38	6.95	MED20	Mediator of RNA polymerase II transcription subunit 20	
B24G0253	10.38	16.43	VIM	Vimentin	NM_131872.2
B7G0515	10.37	7.11	DPH2	2-(3-amino-3-carboxypropyl)histidine synthase subunit 2	NM_001089397.1
B24G0127	10.37	5.07	IGKV1D-8	Immunoglobulin kappa variable 1D-8	NM_001045408.1
B5G0748	10.37	6.27	MRPL1	39S ribosomal protein L1, mitochondrial	NM_001204133.1
A13G0301	10.37	6.64	WDR35	WD repeat-containing protein 35	
B22G0636	10.37	3.52	MALT1	Mucosa-associated lymphoid tissue lymphoma translocation protein 1 homolog	NM_152976.1
B10G0445	10.37	4.73	SYTL2	Synaptotagmin-like protein 2	XM_009305483.4
B18G0495	10.37	11.34	MORF4L1	Mortality factor 4-like protein 1	NM_001002604.2
A3G0579	10.36	5.47	PDXDC1	Pyridoxal-dependent decarboxylase domain-containing protein 1	XM_068216689.1
B22G1099	10.36	31.18	FETUB	Fetuin-B	XM_005161856.5
B11G0181	10.36	15.21	RANBP3	Ran-binding protein 3	NM_001164027.1
B1G0471	10.36	5.30	RAB9A	Ras-related protein Rab-9A	NM_001098741.1
A22G0005	10.36	9.63	TMEM43	Transmembrane protein 43	XM_005173929.5
B8G0783	10.36	16.13	FNBP1L	Formin-binding protein 1-like	XM_021478241.2
B13G0244	10.36	6.47	CD180	CD180 antigen	NM_199794.1
B21G0498	10.36	4.89	MYO5B	Unconventional myosin-Vb	XM_009295325.4
B20G0162	10.36	4.10	SAYSD1	SAYSvFN domain-containing protein 1	NM_001082920.1
B12G0141	10.35	12.35	UNC13D	Protein unc-13 homolog D	XM_009306839.3
B6G0577	10.35	7.28	PRPF38A	Pre-mRNA-splicing factor 38A	NM_001003483.2

B23G0487	10.35	23.21	ESYT1	Extended synaptotagmin-1	XM_690519.7
B5G0663	10.34	25.95	G5714_005554	A0A7J6D1C2_9TELE Uncharacterized protein	XR_002457848.2
A18G0758	10.34	1.10			
A10G0419	10.34	10.35	RABGEF1	GTP exchange factor	XM_009305364.3
A9G0839	10.34	11.21	IL10RB	Interleukin-10 receptor subunit beta	
A11G0430	10.34	11.29	LRRC38	Leucine-rich repeat-containing protein 38	NM_131569.2
A13G0489	10.33	0.34	ACT2	Actin, alpha skeletal muscle 2	
B6G1015	10.33	5.80	MARS1	Methionine--tRNA ligase, cytoplasmic	NM_200076.2
B24G0728	10.33	10.26	NAPG	Gamma-soluble NSF attachment protein	NM_001013480.2
A5G0309	10.33	8.76	ARHGAP25	Rho GTPase-activating protein 25	XM_005165169.5
B7G0827	10.33	1.86	SOX19B	Transcription factor Sox-19b	NM_131702.1
B10G0087	10.33	5.37	HDHD2	Haloacid dehalogenase-like hydrolase domain-containing protein 2	NM_001013473.1
B11G0716	10.33	11.20	SPTBN1	Spectrin beta chain, non-erythrocytic 1	NM_001243179.1
A9G0413	10.32	8.22	CCNYL1	Cyclin-Y-like protein 1	NM_001076720.1
A8G0124	10.32	33.11	BNIP3L	adenovirus E1B 19 kDa protein-interacting protein 3-like	NM_001012242.1
B4G0072	10.32	9.43	ANO6	Anoctamin-6	XM_005164462.5
A17G0526	10.32	10.39	PLEKHA1	Pleckstrin homology domain-containing family A member 1	XM_005158692.4
B11G0720	10.31	4.65	VIP	VIP peptides	
A1G0059	10.31	9.50	LOC113120470	A0A6P6RKB7_CARAU uncharacterized protein LOC113120470	
A17G0052	10.31	6.94	NOL10	Nucleolar protein 10	NM_200237.1
A23G0425	10.31	3.47	ARHGEF7	Rho guanine nucleotide exchange factor 7	XM_005162207.4
A4G0832	10.31	4.89	NOPCHAP1	NOP protein chaperone 1	NM_001201412.1
B5G0237	10.30	3.87	RILPL2	RILP-like protein 2	NM_001083850.1
B18G0847	10.30	15.06	USP8	Ubiquitin carboxyl-terminal hydrolase 8	XM_009293354.4
A6G0861	10.30	8.14	RAB22A	Ras-related protein Rab-22A	NM_205719.2
B12G0397	10.30	13.60	POLR2A	DNA-directed RNA polymerase II subunit RPB1	XM_005156225.5
A5G0252	10.30	13.46	PGAM5	threonine-protein phosphatase PGAM5, mitochondrial	NM_001007323.2
A18G0656	10.30	4.97	LOC107600992	A0A672PHB2_SINGR Uncharacterized LOC107600992	NM_001044882.2
B15G0247	10.30	11.53	AP2S1	AP-2 complex subunit sigma	NM_213155.2
B10G0050	10.30	27.60	PAM	Peptidyl-glycine alpha-amidating monooxygenase	XM_694344.6
A15G0746	10.29	10.73	HNRNPL	Heterogeneous nuclear ribonucleoprotein L	NM_001423736.1
B6G0710	10.29	4.05	UQCC2	Ubiquinol-cytochrome-c reductase complex assembly factor 2	NM_200758.2
B9G0653	10.29	5.14	FAM117B	Protein FAM117B	NM_001128362.1
A15G0370	10.28	7.46	BLMH	Bleomycin hydrolase	NM_200018.1
B24G0517	10.28	11.51	MLLT10	Protein AF-10	XM_009297359.4
B14G0616	10.28	11.42	LRRN4CL	LRRN4 C-terminal-like protein	
B18G0353	10.28	19.95	CLASRP	arginine rich protein	NM_001044329.1
A7G0695	10.28	1.87	ANO9	Anoctamin-9	XM_001922625.8

B11G0640	10.28	6.24	EFCC1	EF-hand and coiled-coil domain-containing protein 1	NM_001017657.1
A2G0826	10.28	8.59	PWWP3B	PWWP domain-containing DNA repair factor 3B	XM_001345991.9
B13G0596	10.27	8.02	LORF2	LINE-1 retrotransposable element ORF2 protein	NM_001013484.2
A18G0415	10.27	10.03	EDC4	Enhancer of mRNA-decapping protein 4	NM_001045085.1
B5G0931	10.27	11.48	DPM2	Dolichol phosphate-mannose biosynthesis regulatory protein	NM_001122846.2
B19G0683	10.27	11.47	ARL4A	ADP-ribosylation factor-like protein 4A	
B1G0817	10.27	8.66	PDCD11	Protein RRP5 homolog	NM_001089368.2
B13G0305	10.27	4.49	GABRB1	Gamma-aminobutyric acid receptor subunit beta-1	NM_001006079.1
B3G1261	10.27	5.83	MCM5	DNA replication licensing factor mcm5	NM_178436.3
B3G0550	10.27	14.88	LOC113071524	A0A6P6MWE8_CARAU uncharacterized protein LOC113071524	
A4G0986	10.26	9.00	FGFR1OP2	FGFR1 oncogene partner 2 homolog	NM_199955.1
B14G0327	10.26	19.34	RNF130	E3 ubiquitin-protein ligase RNF130	XM_009291118.4
B16G0781	10.26	13.97	USP5	Ubiquitin carboxyl-terminal hydrolase 5	XM_005173477.5
A11G0469	10.26	44.68	TMEM169	Transmembrane protein 169	NM_214739.1
A22G0378	10.26	8.15	POLR2E	DNA-directed RNA polymerases I, II, and III subunit RPABC1	NM_001003564.2
B12G0294	10.26	12.59	CDK5RAP3	CDK5 regulatory subunit-associated protein 3	NM_001002105.1
A4G0989	10.26	9.04	SCAF11	Protein SCAF11	XM_689753.9
B7G0274	10.26	12.34	GGH	Gamma-glutamyl hydrolase	NM_213322.1
B12G0806	10.25	0.69	FAM20A	Pseudokinase FAM20A	XM_005155987.5
B15G0528	10.25	9.82	METTL16	RNA N6-adenosine-methyltransferase mettl16	NM_001003611.1
A2G0533	10.25	8.83	PCCB	Propionyl-CoA carboxylase beta chain, mitochondrial	XM_005163372.5
B16G0395	10.25	5.27	GPN2	GPN-loop GTPase 2	NM_214801.2
A20G0177	10.25	3.97	TRMT6	tRNA (adenine(58)-N(1))-methyltransferase non-catalytic subunit TRM6	NM_001037392.2
A7G0569	10.25	25.84	KIAA0232	Uncharacterized protein KIAA0232	NM_001328036.1
A11G0051	10.24	2.44	GIMAP8	GTPase IMAP family member 8	XM_009305754.4
A16G1059	10.24	4.61	SOX4	Transcription factor SOX-4	NM_200901.1
B17G0409	10.24	11.00	MINPP1	Multiple inositol polyphosphate phosphatase 1	XM_005158515.5
B17G0247	10.23	4.94	RTRAF	RNA transcription, translation and transport factor protein	NM_200270.2
B12G0582	10.23	7.32	LSM5	U6 snRNA-associated Sm-like protein LSm5	NM_001100438.2
B13G0169	10.23	1.67	ZFP36L2-B	mRNA decay activator protein ZFP36L2-B	NM_001111187.1
B7G0559	10.23	13.10	SLC8B1	calcium exchanger protein	XM_021477930.2
B5G1271	10.23	8.07	ATP8B2	Phospholipid-transporting ATPase ID	XM_021476431.2
B6G0305	10.23	14.44	DNPEP	Aspartyl aminopeptidase	XM_005165862.4
A24G0621	10.23	344.03	CPB1	Carboxypeptidase B	NM_001328425.1
A20G0569	10.23	17.05	GINM1	Glycoprotein integral membrane protein 1	NM_001079836.1
A8G0052	10.23	6.92	DGCR6	Protein DGCR6	XM_017357308.3

B2G0335	10.23	5.43	CRLF1	Cytokine receptor-like factor 1	NM_001002650.1
B14G0358	10.22	6.36	CSTF2	Cleavage stimulation factor subunit 2	NM_200114.2
B8G0980	10.22	10.03	FUT9	4-galactosyl-N-acetylglucosaminide 3-alpha-L-fucosyltransferase 9	NM_001077246.2
B4G0035	10.22	6.58	ATP23	Mitochondrial inner membrane protease ATP23 homolog	NM_001083580.1
B19G0693	10.22	5.80	MYCL1B	Protein L-Myc-1b	NM_001045142.1
B6G0080	10.22	19.29	MED8	Mediator of RNA polymerase II transcription subunit 8	NM_207083.1
B7G1106	10.22	7.75	OXA1L	Mitochondrial inner membrane protein OXA1L	NM_001105130.2
A10G0649	10.21	17.46	TMEM230	Transmembrane protein 230	NM_001004006.1
A1G0551	10.21	8.38	TMEM184C	Transmembrane protein 184C	NM_001145596.1
A19G0812	10.21	7.30	ZNF384	Zinc finger protein 384	XM_005159417.5
B13G0816	10.21	4.26	TMEM63B	CSC1-like protein 2	
B13G0851	10.21	17.11	IRF2BP2A	Interferon regulatory factor 2-binding protein 2-A	
A14G0301	10.21	5.51	PDGFC	Platelet-derived growth factor C	XM_683962.9
A19G0702	10.21	6.49	LORF2	LINE-1 retrotransposable element ORF2 protein	XM_017352166.3
A11G0546	10.21	1.70	CAV2	Caveolin-2	XM_003199503.6
B1G0981	10.21	7.56	LGALSL	Galectin-related protein	NM_001045300.1
A23G0249	10.21	17.37	TM9SF4	Transmembrane 9 superfamily member 4	NM_200510.2
A4G0425	10.20	3.84	LOC113092456	A0A6P6NYL5_CARAU uncharacterized protein LOC113092456	
A22G0415	10.20	2.41	CFD	Complement factor D	XM_021474472.2
A5G1094	10.20	6.09	AATF	Protein AATF	XM_005165555.5
B15G0620	10.20	4.86	MRPS23	28S ribosomal protein S23, mitochondrial	XM_005157688.5
B24G0630	10.20	4.98	TMEM14C	Transmembrane protein 14C	XM_005162575.5
A20G0200	10.20	11.02	PARP1	Poly [ADP-ribose] polymerase 1	NM_001044942.1
B17G0605	10.20	6.60	KLF11	Krueppel-like factor 11	NM_001077604.2
A24G0233	10.20	16.90	ABCD3	ATP-binding cassette sub-family D member 3	NM_213482.1
B7G0539	10.20	7.06			
A7G0843	10.19	17.24	AP1S1	AP-1 complex subunit sigma-1A	NM_001328014.1
B12G0311	10.19	10.23	DHX8	ATP-dependent RNA helicase DHX8	XM_002663947.6
B8G0835	10.19	5.93	CEPT1	ethanolaminephosphotransferase 1	NM_001144792.3
A21G0743	10.19	21.99	PAFAH1B1B	Lissencephaly-1 homolog B	NM_201346.1
B5G1097	10.19	5.81	MRPL41	39S ribosomal protein L41, mitochondrial	
A7G0981	10.18	6.74	FIS1	Mitochondrial fission 1 protein	NM_001310843.2
B21G0228	10.18	12.94	RNF121	RING finger protein 121	NM_001308556.1
B24G0195	10.18	8.23	LOC113084854	A0A6P6NQN8_CARAU uncharacterized protein LOC113084854 isoform X1	XM_005159700.5
A4G0738	10.18	13.19	MSRB3	Methionine-R-sulfoxide reductase B3	NM_001320378.1
A22G0553	10.18	4.09	ROHU_005067	A0A498M6S4_LABRO Transposase domain-containing protein	
B2G1270	10.18	6.19	G5714_001543	A0A7J6DCM3_9TELE Uncharacterized protein	XM_002660687.7
A2G0575	10.18	18.78	LYN	Tyrosine-protein kinase Lyn	NM_001004543.1
A11G0579	10.18	3.75	SH2D5	SH2 domain-containing protein 5	NM_001003538.2

A22G0242	10.17	6.62	TIMM13	Mitochondrial import inner membrane translocase subunit Tim13	NM_001002465.2
B24G0437	10.17	10.49	PAN3	PAN2-PAN3 deadenylation complex subunit PAN3	NM_001105692.2
B1G0024	10.17	9.31	TFDP1	Transcription factor Dp-1	XM_068214205.1
B5G0431	10.17	6.59	SLC37A2	Glucose-6-phosphate exchanger SLC37A2	NM_213014.2
A23G0280	10.17	11.03	GID8B	Glucose-induced degradation protein 8-B homolog	NM_001386504.1
A2G0447	10.17	16.49	SEPTIN2	Septin-2	NM_001301360.1
B6G0440	10.17	13.00	PIK3R5	Phosphoinositide 3-kinase regulatory subunit 5	XM_009302367.4
A6G0616	10.16	18.54	ZRANB2	Zinc finger Ran-binding domain-containing protein 2	NM_001328210.1
A6G0682	10.16	23.45	IP6K2	Inositol hexakisphosphate kinase 2	NM_001002389.1
B14G0816	10.16	15.85	VAMP7	Vesicle-associated membrane protein 7	NM_200266.1
A25G0721	10.16	2.65	SNX20	Sorting nexin-20	XM_009298207.4
B20G0127	10.16	11.32	CNK3/IPCEF1	IPCEF1 PE=1 SV=1	NM_001076758.1
B18G0092	10.16	4.16	PEX16	Peroxisomal membrane protein PEX16	NM_001025169.2
B24G0457	10.15	5.13	GIMAP7	GTPase IMAP family member 7	XM_068218488.1
A13G0891	10.15	7.44	A33	Zinc-binding protein A33	NM_213270.2
B7G1090	10.15	12.89	NDUFS2	NADH dehydrogenase [ubiquinone] iron-sulfur protein 2, mitochondrial	NM_001020645.2
A1G0270	10.15	4.79	RAB3D	GTP-binding protein Rab-3D	NM_001145597.1
B24G0567	10.15	17.61	DCAF11	DDB1- and CUL4-associated factor 11	NM_001031675.2
B10G0318	10.15	22.34	PER1	Period circadian protein homolog 1	XM_005172625.5
A14G0275	10.15	5.41	MED7	Mediator of RNA polymerase II transcription subunit 7	NM_199741.1
B3G1411	10.15	3.42	K02A2.6	Uncharacterized protein K02A2.6	
B8G0940	10.14	3.39	CXADR	Coxsackievirus and adenovirus receptor homolog	NM_001130781.2
A6G0093	10.14	10.79	GORASP2	Golgi reassembly-stacking protein 2	NM_200703.2
B14G0630	10.14	5.05	PMT-2	Phosphoethanolamine N-methyltransferase 2	NM_001076637.1
A7G1386	10.13	2.11	LOC113093141	A0A6P6P1T5_CARAU mucin-3A-like	
A15G0605	10.13	7.24	NXN	Nucleoredoxin	NM_001020595.1
B19G0675	10.13	19.35	C6ORF62	Uncharacterized protein C6orf62	NM_001020637.1
A20G0818	10.13	16.78	PAPOLA	Poly(A) polymerase alpha	NM_205758.2
B25G0429	10.13	1.54	KCTD15L	POZ domain-containing protein kctd15-like	NM_001006012.2
B14G0005	10.13	39.52	CYTL1	Cytokine-like protein 1	XM_021481075.2
B2G1113	10.12	8.96	SMC6	Structural maintenance of chromosomes protein 6	NM_001128334.1
A14G0627	10.12	8.90	DIAPH1	Protein diaphanous homolog 1	XM_021481083.2
A2G1095	10.12	1.70	THEM6	Protein THEM6	NM_001122704.1
B14G0526	10.12	18.14	HNRNPH2	Heterogeneous nuclear ribonucleoprotein H2	NM_205684.1
A5G0452	10.12	7.35	TANGO2	Transport and Golgi organization protein 2 homolog	XM_068221148.1
B6G0591	10.12	5.64	HSD17B7	17-beta-hydroxysteroid dehydrogenase 7	NM_001077328.1

A14G0511	10.12	3.51	THG1L	Probable tRNA(His) guanylyltransferase	NM_001007455.2
A2G0426	10.12	13.67	RNF2	E3 ubiquitin-protein ligase RING2	NM_131213.2
B22G0791	10.11	8.17	TLE5	TLE family member 5	NM_200423.1
A8G0450	10.11	4.68	FOXP1B	Forkhead box protein P1-B	XM_068223144.1
B14G0793	10.11	0.40	GLOD5	Glyoxalase domain-containing protein 5	
B14G0792	10.11	0.40	GLOD5	Glyoxalase domain-containing protein 5	
A7G0912	10.11	11.52	MMP14	Matrix metalloproteinase-14	NM_194416.1
B7G0217	10.11	1.85	SCAMP2	Secretory carrier-associated membrane protein 2	NM_201194.2
B9G0147	10.11	4.49	METTL5	rRNA N6-adenosine-methyltransferase METTL5	NM_001005949.1
A5G0267	10.10	11.08	KCTD10	POZ domain-containing adapter for CUL3-mediated RhoA degradation protein 3	NM_213478.2
A1G0656	10.10	8.63	NNMT	Nicotinamide N-methyltransferase	NM_001111246.2
A23G0317	10.10	8.44	CCDC115	Coiled-coil domain-containing protein 115	NM_001013295.2
B9G0868	10.10	10.02	TLK1B	threonine-protein kinase tousled-like 1-B	XM_068223392.1
A3G1241	10.10	7.34	RAC1	Ras-related C3 botulinum toxin substrate 1	NM_001039818.1
B1G0472	10.09	36.64	GPM6B	Neuronal membrane glycoprotein M6-b	NM_203427.2
B19G0515	10.09	6.91	FKBP9	Peptidyl-prolyl cis-trans isomerase FKBP9	NM_001003520.1
B22G0818	10.09	10.27	UCHL5	Ubiquitin carboxyl-terminal hydrolase isozyme L5	NM_213084.1
A16G0242	10.09	6.27	MTDH	Protein LYRIC	XM_021466990.2
A20G0149	10.09	13.45	ZC3H14	Zinc finger CCCH domain-containing protein 14	NM_001025524.2
B15G0022	10.09	4.58	NLRP1	NACHT, LRR and PYD domains-containing protein 1	XM_001345547.8
B3G0445	10.09	1.27			XM_021474742.2
A7G0729	10.08	3.41	MPHOSPH10	U3 small nucleolar ribonucleoprotein protein MPP10	NM_001305543.1
B3G0987	10.08	10.10	CARHSP1	Calcium-regulated heat-stable protein 1	XM_005163934.5
A21G0304	10.08	8.61	GPN3	GPN-loop GTPase 3	NM_001007370.1
B15G0517	10.08	15.11	GOSR1	Golgi SNAP receptor complex member 1	XM_005157565.5
B13G0002	10.08	15.88	HARBI1	Putative nuclease HARBI1	XM_021480925.2
B5G0443	10.07	10.88	NUP88	Nucleoporin 88	NM_001020466.1
B10G0045	10.07	12.68	MAP3K1	Mitogen-activated protein kinase kinase kinase 1	XM_068223888.1
B10G0839	10.07	22.68	DYRK1A	Dual specificity tyrosine-phosphorylation-regulated kinase 1A	NM_001347739.1
A22G0209	10.07	6.36	BLOC1S1	Biogenesis of lysosome-related organelles complex 1 subunit 1	NM_001044350.2
B12G0539	10.07	3.16	RPP30	Ribonuclease P protein subunit p30	NM_001013479.1
A19G0617	10.07	6.33	PIP5K1A	Phosphatidylinositol 4-phosphate 5-kinase type-1 alpha	NM_001020602.1
A3G0618	10.07	9.38	RBBP6	E3 ubiquitin-protein ligase RBBP6	NM_001193674.1

B5G0395	10.07	4.08	CDC26	Anaphase-promoting complex subunit CDC26	
A1G0837	10.06	29.99	HAPSTR1	HUWE1-associated protein modifying stress responses	NM_199602.2
B19G0115	10.06	3.99	NECTIN2	Nectin-2	NM_001430892.1
B25G0567	10.06	5.57	PPFIBP2	Liprin-beta-2	NM_001326598.1
B22G0175	10.06	8.43	CHERP	Calcium homeostasis endoplasmic reticulum protein	XM_068216258.1
B1G0261	10.06	7.99	CHIC2	Cysteine-rich hydrophobic domain-containing protein 2	NM_201120.2
A20G0314	10.06	7.57	ENAH	Protein enabled homolog	XM_068215435.1
A16G0849	10.05	12.00	LAG3	Lymphocyte activation gene 3 protein	
B4G0592	10.05	27.81	ZC3HAV1	Zinc finger CCCH-type antiviral protein 1	
B6G0294	10.05	4.72	ARL6IP6	ADP-ribosylation factor-like protein 6-interacting protein 6	XM_017356417.3
B5G0586	10.05	12.29	ERAP2	Endoplasmic reticulum aminopeptidase 2	XM_005165468.4
B15G0025	10.05	10.55	MFSD1	Major facilitator superfamily domain-containing protein 1	NM_001037426.2
A5G0877	10.05	3.34	CERT1	Ceramide transfer protein	NM_001017914.4
A6G0922	10.05	22.41	GNAI2	Guanine nucleotide-binding protein G(i) subunit alpha-2	NM_199842.1
B13G0604	10.05	7.66	BEND3	BEN domain-containing protein 3	NM_001077718.1
B15G0607	10.05	27.85	SAMSN1	SAM domain-containing protein SAMSN-1	NM_001128669.1
B15G0476	10.05	7.05	C1QTNF5	Complement C1q tumor necrosis factor-related protein 5	NM_001029953.1
A12G0209	10.05	5.74	GALK1	Galactokinase	NM_001006002.1
B22G1048	10.05	16.63	FXR1	RNA-binding protein FXR1	NM_001312881.1
A1G1073	10.05	10.55	LIAS	Lipoyl synthase, mitochondrial	NM_001110401.1
A16G0262	10.05	4.83	GBP	GSK-3-binding protein	NM_131442.1
A7G0795	10.04	7.95	ADGRG1	Adhesion G-protein coupled receptor G1	NM_001020481.1
A23G0189	10.04	5.68	ARHGAP39	Rho GTPase-activating protein 39	XM_068219383.1
A15G0686	10.04	4.20	ARHGAP35	Rho GTPase-activating protein 35	XM_001343600.6
A4G0972	10.04	17.96	CPSF6	Cleavage and polyadenylation specificity factor subunit 6	XM_005164453.5
B17G0626	10.04	16.63	SNAP23	Synaptosomal-associated protein 23	XM_005158727.4
B17G0094	10.04	2.93	FAM167A	Protein FAM167A	
B24G0329	10.04	8.61	TNIK	TRAF2 and NCK-interacting protein kinase	XM_068217078.1
B22G0192	10.04	2.47	CD274	Programmed cell death 1 ligand 1	NM_001128805.1
B2G0879	10.03	10.29	PRRX1	Paired mesoderm homeobox protein 1	NM_214734.1
B25G0074	10.03	11.49	SPI1	Transcription factor PU.1	NM_001430957.1
A10G0403	10.03	25.53	IL2RG	Cytokine receptor common subunit gamma	NM_001128271.1
B23G0061	10.03	9.05	SLC9A8	hydrogen exchanger 8	NM_001008586.1
B20G0167	10.03	4.49	KCNK5	Potassium channel subfamily K member 5	NM_200633.1
A12G0010	10.03	17.95	ACADSB	branched chain specific acyl-CoA dehydrogenase, mitochondrial	NM_001351649.2
B3G0790	10.03	16.81	MUCM	Ig mu chain C region membrane-bound form	

A5G1342	10.02	3.81	ZCCHC8	Zinc finger CCHC domain-containing protein 8	NM_001083818.1
A4G0794	10.02	12.86	SBF1	Myotubularin-related protein 5	XM_005164591.5
B21G0071	10.02	9.07	AFF4	FMR2 family member 4	XM_068216154.1
B14G0633	10.02	11.71	DOCK2	Dedicator of cytokinesis protein 2	XM_021481082.2
B20G0346	10.02	18.46	CLOCK	Circadian locomoter output cycles protein kaput	NM_130957.2
A15G0843	10.02	10.73	CUX1	Protein CASP	NM_001003514.1
B6G0131	10.02	10.33	G5714_006664	A0A7J6CWZ4_9TELE Uncharacterized protein	
A9G0162	10.02	23.21	COL5A2	Collagen alpha-2(V) chain	NM_001145782.1
B18G0564	10.02	5.70	ZNF143	Zinc finger protein 143	XM_009293545.4
A8G0055	10.01	4.83			
A5G1231	10.01	5.92	RAB14	Ras-related protein Rab-14	NM_200683.2
A3G0446	10.01	18.50	KCTD5	POZ domain-containing protein KCTD5	NM_207049.1
B20G0408	10.01	3.69	MIDEAS	Mitotic deacetylase-associated SANT domain protein	XM_005160589.5
B14G0197	10.01	9.08	CAB39	Calcium-binding protein 39	XM_068213185.1
A13G0096	10.01	7.88	RSRP1	serine-rich protein 1	NM_001013445.1
A8G0291	10.01	5.69	RPE65B	All-trans-retinyl ester 13-cis isomerohydrolase	NM_001113653.1
B11G0405	10.00	6.40	C20ORF96	Uncharacterized protein C20orf96	XR_011017120.1
B17G0534	10.00	12.44	STRN3	Striatin-3	NM_001013266.1
A3G0323	10.00	18.07	PRKACA	cAMP-dependent protein kinase catalytic subunit alpha	XM_021470955.2
B13G0131	10.00	14.89	PHF10	PHD finger protein 10	NM_001089525.2
B2G0117	10.00	1.16			
A21G0672	10.00	9.79	RBM22	Pre-mRNA-splicing factor RBM22	NM_213214.1
B17G0469	10.00	6.63	FAM167A	Protein FAM167A	XM_021473175.2
A19G0117	10.00	9.37	SI:CH211-105D11.2	Uncharacterized protein C18orf19 homolog A	NM_001044994.2
A8G0243	9.99	11.52	LONRF1	LON peptidase N-terminal domain and RING finger protein 1	NM_001277234.1
B1G0935	9.99	9.36	IFNGR1	Interferon gamma receptor 1	
A8G0810	9.99	1.55	SAL	Rhamnose-binding lectin	
B9G0184	9.99	8.71	ITPRID2	Protein ITPRID2	XM_021479016.2
A14G0123	9.99	0.22	EMX1	Homeobox protein EMX1	NM_131279.2
A15G0461	9.99	5.40	ERCC2	General transcription and DNA repair factor IIH helicase subunit XPD	NM_200926.2
B23G0060	9.99	7.94	B4GALT5	Beta-1,4-galactosyltransferase 5	NM_001045332.2
A20G0110	9.99	4.63	CRNKL1	Crooked neck-like protein 1	NM_200946.1
A18G0329	9.99	38.78	TBCB	Tubulin-folding cofactor B	NM_212775.1
A2G1126	9.98	15.09	SPINW	Spindlin-W	XM_001339007.9
B2G1192	9.98	11.73	CDC73	Parafibromin	NM_001291396.1
B15G0879	9.98	7.47	PARL	Presenilins-associated rhomboid-like protein, mitochondrial	XM_021466506.2
A7G0438	9.98	4.31	HDDC3	Guanosine-3',5'-bis(diphosphate) 3'-pyrophosphohydrolase MESH1	NM_001017835.1
B20G0216	9.98	6.08	FAM20B	Glycosaminoglycan xylosylkinase	NM_001044818.1
A3G0154	9.98	8.35	TRIR	Telomerase RNA component interacting RNase	NR_170156.1
B25G0131	9.98	0.00	IGKV4-1	Immunoglobulin kappa variable 4-1	XM_068217596.1

B11G0474	9.98	0.45	IQSEC1	IQ motif and SEC7 domain-containing protein 1	
B21G0310	9.98	4.78	ASL	Argininosuccinate lyase	NM_200451.1
B11G0151	9.97	8.35	PIF1	ATP-dependent DNA helicase PIF1	XM_005155724.5
A6G0665	9.97	7.25	LARP4	La-related protein 4	XM_017356657.3
B2G0834	9.97	13.37	NFI1	CTD small phosphatase-like protein	XM_005163361.5
A8G0290	9.97	8.05	OSBPL9	Oxysterol-binding protein-related protein 9	NM_001077805.1
B3G0402	9.97	6.27	CRB3	Protein crumbs homolog 3	NM_001045322.1
A19G0134	9.97	6.76	CPSF1	Cleavage and polyadenylation specificity factor subunit 1	NM_001114681.2
B7G0343	9.97	6.55	PLCB3	1-phosphatidylinositol 4,5-bisphosphate phosphodiesterase beta-3	NM_001122773.1
A10G0049	9.97	2.16	EPB41L4A	Band 4.1-like protein 4	NM_131223.2
B19G0426	9.97	10.01	PREP	Prolyl endopeptidase	XM_002667552.6
B19G0176	9.96	8.50	MEAF6	Chromatin modification-related protein MEAF6	NM_001003756.1
B9G0906	9.96	8.18	IFNAR1A	beta receptor 1a	
B14G0769	9.96	5.05	MAGT1	Magnesium transporter protein 1	NM_199700.1
A6G0943	9.96	9.12	ILRUN	Protein ILRUN	NM_001013542.2
A25G0092	9.96	6.91	HDAC10	Polyamine deacetylase HDAC10	NM_199775.1
B18G0603	9.96	4.12	TMEM263	Transmembrane protein 263	NM_213141.1
A1G0036	9.95	9.33	VAV1	Proto-oncogene vav	
A1G0050	9.95	23.01	NLRC3	Protein NLRC3	XM_068220504.1
B1G0861	9.95	5.25	NDUFAF5	Arginine-hydroxylase NDUFAF5, mitochondrial	NM_001082894.2
A3G0169	9.95	6.08	NUP85	Nuclear pore complex protein Nup85	NM_001003625.1
B8G0350	9.95	1.73			
B7G0432	9.95	3.66	USP6NL	USP6 N-terminal-like protein	XM_681604.7
B5G0803	9.95	12.67	IL13RA2	Interleukin-13 receptor subunit alpha-2	NM_001113731.3
A2G0965	9.95	2.51	ARHGAP12	Rho GTPase-activating protein 12	XM_005171388.5
A17G0667	9.95	13.41	FAM177A1	Protein FAM177A1	
B12G0524	9.94	13.42	PTEN	Phosphatidylinositol 3,4,5-trisphosphate 3-phosphatase and dual-specificity protein phosphatase PTEN	NM_001423688.1
B12G0771	9.94	5.37	DAGLB	Diacylglycerol lipase-beta	
B22G0284	9.94	14.38	NLRP3	NACHT, LRR and PYD domains-containing protein 3	XM_068216562.1
A18G0075	9.94	11.38	BLVRB	Flavin reductase (NADPH)	NM_001002686.2
A21G0427	9.94	6.85	CUL5	Cullin-5	XM_021474455.2
A6G0927	9.94	36.40	RBM5	RNA-binding protein 5	NM_001100138.3
A19G0332	9.94	4.32	ELP2	Elongator complex protein 2	XM_005159658.5
B7G0804	9.93	6.97			
B1G1126	9.93	36.98	NLRC3	NLR family CARD domain-containing protein 3	XM_009295700.4
B24G0584	9.92	5.46	TGIF1	Homeobox protein TGIF1	NM_199567.1
A19G0368	9.92	3.24	COL16A1	Collagen alpha-1(XVI) chain	XM_021468177.2
A21G0872	9.92	16.51	STK10	threonine-protein kinase 10	NM_201133.2
B7G0920	9.92	1.44	BTN1A1	Butyrophilin subfamily 1 member A1	NM_001130628.1
A7G0413	9.92	12.62	KATNB1	Katanin p80 WD40 repeat-containing subunit B1	NM_213018.1
A15G0437	9.92	9.82	POLDIP2	Polymerase delta-interacting protein 2	NM_212714.1
B6G0264	9.91	8.39	CYTIP	Cytohesin-interacting protein	

A7G1043	9.91	18.08	FCER1G	High affinity immunoglobulin epsilon receptor subunit gamma	NM_001100106.2
B3G1148	9.91	4.45	TOM1	Target of Myb1 membrane trafficking protein	NM_001351651.1
A22G0211	9.91	7.72	RBCK1	RanBP-type and C3HC4-type zinc finger-containing protein 1	NM_001002168.1
B3G1114	9.91	16.38	TMUB2	Transmembrane and ubiquitin-like domain-containing protein 2	NM_001005573.1
A1G0790	9.91	5.24			NM_212965.1
B18G0483	9.91	6.80	AGK	Acylglycerol kinase, mitochondrial	NM_199880.1
A6G0253	9.91	9.56	GPD2	Glycerol-3-phosphate dehydrogenase, mitochondrial	XM_068221793.1
A18G0520	9.90	8.55	BCL2L14	Apoptosis facilitator Bcl-2-like protein 14	
A11G0384	9.90	1.07	CCDC120	Coiled-coil domain-containing protein 120	NM_001114480.1
A19G0078	9.90	9.79	CTPS1	CTP synthase 1	NM_199211.2
B16G0742	9.90	4.25	ZNF384	Zinc finger protein 384	XM_009292250.4
B12G0571	9.90	9.15	FKBP10	Peptidyl-prolyl cis-trans isomerase FKBP10	NM_001128666.1
B1G1088	9.90	43.58	NLRC3	Protein NLRC3	XM_009295700.4
A22G0037	9.90	5.85	DSTYK	threonine and tyrosine protein kinase	XM_005168270.5
B1G1125	9.89	9.21	LOC113056809	A0A6P6L4R2_CARAU piggyBac transposable element-derived protein 3-like	
A15G0165	9.89	11.45	STIM1	Stromal interaction molecule 1	XM_005173393.5
B13G0210	9.89	8.21	BAG5	BAG family molecular chaperone regulator 5	NM_001386796.1
B3G0600	9.89	9.59	CPSF4	Cleavage and polyadenylation specificity factor subunit 4	NM_131009.1
B6G0371	9.89	14.04	ZXDC	Zinc finger protein ZXDC	XM_021477089.2
B7G0793	9.88	25.05	TMEM9B	Transmembrane protein 9B	NM_199732.2
B17G0511	9.88	3.73	ASAP2	Arf-GAP with SH3 domain, ANK repeat and PH domain-containing protein 2	NM_001365098.1
B15G0910	9.88	6.19	INPPL1A	Phosphatidylinositol 3,4,5-trisphosphate 5-phosphatase 2A	NM_001039804.2
B16G0036	9.88	21.93	NCOA7	Nuclear receptor coactivator 7	
A20G0428	9.88	16.73	RNASEH1	Ribonuclease H1	
B4G0511	9.88	12.30	PPP6R2	threonine-protein phosphatase 6 regulatory subunit 2	NM_001025509.1
B25G0696	9.88	3.89	MUC5AC	Mucin-5AC	XM_021470622.2
B14G0596	9.88	12.20	FNTA	geranylgeranyltransferase type-1 subunit alpha	NM_001080560.2
B3G0031	9.87	9.75	GVIN1	Interferon-induced very large GTPase 1	XM_005164343.5
B14G0620	9.87	6.19	ARL9	ADP-ribosylation factor-like protein 9	
A23G0167	9.87	10.85	PRPF6	Pre-mRNA-processing factor 6	NM_212655.2
A6G0214	9.87	8.78	SP3	Transcription factor Sp3	XM_005165800.5
A13G0481	9.86	14.31	RAB1A	Ras-related protein Rab-1A	NM_001001841.3
A5G0740	9.86	6.70	GIMAP4	GTPase IMAP family member 4	
A3G0563	9.86	11.89	MLX	Max-like protein X	NM_001024223.2
B8G0222	9.86	3.35	LSM1	U6 snRNA-associated Sm-like protein LSm1	NM_001003551.1
B7G0648	9.86	9.20	FHOD3	FH2 domain-containing protein 3	XM_068222691.1
B10G0068	9.86	4.59	LYRM7	Complex III assembly factor LYRM7	

A1G0340	9.85	66.17	LOC107583238	A0A672L931_SINGR C-X-C motif chemokine 11-like	
B6G0548	9.85	16.26	AK4	Adenylate kinase 4, mitochondrial	NM_213299.1
B22G0228	9.85	24.01	A0A673MMJ4	A0A673MMJ4_9TELE Uncharacterized protein	
A17G0544	9.85	7.10	UBE2D4	Ubiquitin-conjugating enzyme E2 D4	NM_201110.1
A22G0323	9.85	4.55	MALT1	Mucosa-associated lymphoid tissue lymphoma translocation protein 1 homolog	NM_152976.1
B22G0958	9.85	13.54	CD276	CD276 antigen homolog	
B2G0036	9.85	10.48	ARHGAP30	Rho GTPase-activating protein 30	XM_068215009.1
B13G0059	9.85	5.21	ABCC2	ATP-binding cassette sub-family C member 2	
A2G1149	9.85	33.63	KLF2	Krueppel-like factor 2	NM_131857.2
A7G0285	9.85	9.30	B3GALT2	Beta-1,3-galactosyltransferase 2	
A3G0110	9.85	7.90			
B19G0249	9.85	15.28	CIART	Circadian-associated transcriptional repressor	NM_001327898.1
A15G0587	9.84	11.98	ITGAE	Integrin alpha-E	XM_021472928.2
A8G0850	9.84	14.47	CHMP7	Charged multivesicular body protein 7	NM_200781.1
B20G0208	9.84	16.58	GTF2A1	Transcription initiation factor IIA subunit 1	NM_212657.1
B22G1115	9.84	3.01	SIRT7	NAD-dependent protein deacetylase sirtuin-7	XM_001336402.7
B19G0246	9.84	8.38	CERS2	Ceramide synthase 2	NM_153671.2
A1G0488	9.84	2.68	RGS12	Regulator of G-protein signaling 12	XM_005160017.5
A3G0825	9.84	9.41	NAA60	N-alpha-acetyltransferase 60	NM_001082872.1
B8G0184	9.83	6.84	ARID5A	AT-rich interactive domain-containing protein 5A	NM_001327774.1
B6G0549	9.83	16.86	JAK1	Tyrosine-protein kinase JAK1	NM_131073.1
B16G0548	9.83	5.73	LOC113116628	A0A6P6R641_CARAU probable assembly chaperone of rpl4	XM_017351506.3
B1G0726	9.83	13.16	SMOX	Spermine oxidase	NM_001328186.1
A12G0379	9.83	13.53	FBXO11	F-box only protein 11	XM_691042.10
B5G0269	9.83	8.97	GANAB	Neutral alpha-glucosidase AB	NM_001327941.1
B16G0327	9.83	8.02	SNRNP48	U12 small nuclear ribonucleoprotein 48 kDa protein	NM_001115142.1
B23G0285	9.82	1.00	RBBP8NL	RBBP8 N-terminal-like protein	
A19G0945	9.82	8.53	SRSF10	arginine-rich splicing factor 10	NM_200533.2
B6G0274	9.82	3.98	CXCR4	C-X-C chemokine receptor type 4	NM_131882.3
A11G0582	9.82	7.25	YOD1	Ubiquitin thioesterase OTU1	NM_001079988.1
A18G0398	9.82	11.12	CBFB	Core-binding factor subunit beta	XM_021467588.2
A16G0132	9.82	1.74	RAB25	Ras-related protein Rab-25	NM_001029971.1
A17G0446	9.82	5.87	GALE	UDP-glucose 4-epimerase	XM_005158523.4
B5G1124	9.82	83.20	TM4SF4	Transmembrane 4 L6 family member 4	NM_001105117.1
B16G0753	9.82	9.98	ABHD16A	Phosphatidylserine lipase ABHD16A	NM_001111178.1
B5G0699	9.82	1.93	FCGBP	IgGFc-binding protein	XM_017356285.3
B25G0493	9.82	10.83	TES	Testin	NM_205720.2
B21G0697	9.81	47.51	SPTAN1	Spectrin alpha chain, non-erythrocytic 1	XM_021468826.2
A18G0672	9.81	3.72	UTP15	U3 small nucleolar RNA-associated protein 15 homolog	XM_005158982.5

B19G0951	9.81	8.83	VPS52	Vacuolar protein sorting-associated protein 52 homolog	XM_068215267.1
A2G0909	9.80	11.19	ACVR1	Activin receptor type-1	XM_021478751.2
A6G0881	9.80	11.52	MTSS1	Protein MTSS 1	XM_068221739.1
B20G0097	9.80	6.20	MFSD4B	Sodium-dependent glucose transporter 1	
B3G0021	9.80	10.92	A33	Zinc-binding protein A33	NM_001089399.2
A14G0177	9.80	5.05	MRPL11	39S ribosomal protein L11, mitochondrial	NM_001002556.1
B6G0795	9.80	4.11	FRMD4B	FERM domain-containing protein 4B	XM_021471836.2
A14G0376	9.80	8.24	GRHPR	hydroxypyruvate reductase	NM_001020525.1
A18G0027	9.79	16.75	MOB2	MOB kinase activator 2	XM_005173693.5
A5G0319	9.79	6.20	MTMR8	Myotubularin-related protein 8	NM_200394.1
B13G0925	9.79	20.12			XM_068212834.1
B19G0643	9.79	7.28	RRM2	Ribonucleoside-diphosphate reductase subunit M2	NM_001007163.2
A9G0458	9.79	19.97	LRRFIP2	Leucine-rich repeat flightless-interacting protein 2	XM_009304700.4
A18G0313	9.79	8.71	BCKDHA	2-oxoisovalerate dehydrogenase subunit alpha, mitochondrial	NM_001024419.1
B8G0857	9.79	9.86	TASOR	Protein TASOR	XM_005166734.5
B7G1206	9.79	50.48	CXCL8	Interleukin-8	
A16G1039	9.78	1.79	NDUFA3	NADH dehydrogenase [ubiquinone] 1 alpha subcomplex subunit 3	
B14G0211	9.78	2.37	CD40LG	CD40 ligand	NM_001144809.1
B3G0684	9.78	3.52	DUS1L	17) synthase [NAD(P)(+)]-like	NM_199617.2
A17G0814	9.78	15.09	CDC5L	Cell division cycle 5-like protein	NM_201084.2
A5G0896	9.78	9.19	SERINC5	Serine incorporator 5	NM_213514.1
A2G0071	9.78	90.74	TUBB2B	Tubulin beta-2B chain	NM_001024422.1
B11G0116	9.77	14.63	ATXN1	Ataxin-1	XM_005155696.5
B5G0463	9.77	4.74	RCC1L	RCC1-like G exchanging factor-like protein	NM_001082803.1
B5G1029	9.77	10.21	PPP3CC	threonine-protein phosphatase 2B catalytic subunit gamma isoform	NM_001166628.1
B24G0093	9.77	4.44	LYRM1	LYR motif containing protein 1	NM_001002506.1
A25G0104	9.77	5.16	CMASB	N-acylneuraminate cytidylyltransferase B	NM_001258409.1
B21G0209	9.77	7.32	GRK6	G protein-coupled receptor kinase 6	XM_684691.9
B21G0851	9.77	4.71	DNLZ	DNL-type zinc finger protein	
A8G0750	9.77	7.16	GPKOW	G-patch domain and KOW motifs-containing protein	NM_001007207.1
A19G0307	9.77	16.43	MTDH	Protein LYRIC	XM_005159603.4
B6G0327	9.77	34.23	ACKR3	Atypical chemokine receptor 3	NM_001083832.1
A20G0915	9.77	7.86	RARS2	Probable arginine--tRNA ligase, mitochondrial	XM_009294528.4
A23G0232	9.77	13.00	ACSS2	Acetyl-coenzyme A synthetase, cytoplasmic	NM_001328353.1
A8G0723	9.77	42.22	RNF10	RING finger protein 10	NM_001123255.1
A19G0726	9.77	6.96	SF3B4	Splicing factor 3B subunit 4	NM_153661.4
B22G0126	9.77	19.41	PAK2	threonine-protein kinase PAK 2	XM_005170360.5
A17G0865	9.76	31.75	PLD4	5'-3' exonuclease PLD4	
B18G0831	9.76	8.06	TM2D3	TM2 domain-containing protein 3	
B21G0437	9.76	7.81	TMEM223	Transmembrane protein 223	
A7G0778	9.76	6.33	TRADD	Tumor necrosis factor receptor type 1-associated DEATH domain protein	NM_131607.2

A23G0675	9.76	13.89	CALCOCO1	Calcium-binding and coiled-coil domain-containing protein 1	XM_009297039.4
A9G0799	9.76	11.05	SP3	Transcription factor Sp3	NM_001089498.1
A2G0004	9.76	9.30	SUCO	SUN domain-containing ossification factor	XM_021468609.2
B4G0605	9.75	6.12	PARP11	Protein mono-ADP-ribosyltransferase PARP11	
A23G0698	9.75	12.33	PIP4K2C	Phosphatidylinositol 5-phosphate 4-kinase type-2 gamma	NM_200101.3
B6G0675	9.75	13.56	ITGB7	Integrin beta-7	XM_021474125.2
A6G0244	9.75	9.60	MARCHF7	E3 ubiquitin-protein ligase MARCHF7	XM_005165760.5
A24G0511	9.75	4.94	TM9SF1	Transmembrane 9 superfamily member 1	NM_001003550.2
B4G0862	9.75	14.51	MS4A15	Membrane-spanning 4-domains subfamily A member 15	
B5G0462	9.75	46.28	CASTOR2	Cytosolic arginine sensor for mTORC1 subunit 2	NM_199780.2
B3G0524	9.74	6.64	POLR3K	DNA-directed RNA polymerase III subunit RPC10	NM_001002553.1
B16G0714	9.74	0.56	S100A1	Protein S100-A1	NM_001013495.3
B19G0557	9.74	9.67	BET1	BET1 homolog	NM_001002046.1
A11G0690	9.74	7.25	A0A672M2L7	A0A672M2L7_SINGR Uncharacterized protein	XM_009306298.4
A16G0853	9.74	4.45	CD27	CD27 antigen	
B7G0813	9.74	11.97	EXT2	Exostosin-2	NM_001008400.2
B25G0835	9.74	6.93	MRPS35	28S ribosomal protein S35, mitochondrial	NM_001031669.1
B19G0366	9.74	6.37	TBC1D5	TBC1 domain family member 5	NM_001328258.1
B10G0539	9.74	10.53	EURL	Protein EURL homolog	NM_001020552.1
A12G0608	9.73	11.23	EMC9	ER membrane protein complex subunit 9	NM_200126.1
A2G0833	9.73	6.05	MYO9B	Unconventional myosin-IXb	XM_005171335.4
B7G0498	9.73	9.92	ZGC:92818	UPF0547 protein C16orf87 homolog	NM_001002512.2
A24G0006	9.73	3.34	PIGM	GPI mannosyltransferase 1	
B1G0625	9.73	20.90	GIMAP9	GTPase IMAP family member 9	
B5G1207	9.73	22.87	SMARCA1	Probable global transcription activator SNF2L1	XM_005165157.5
B6G1035	9.73	9.99	TGDS	dTDP-D-glucose 4,6-dehydratase	NM_199817.1
A1G1097	9.72	5.00	ARFIP1	Arfaptin-1	NM_213368.1
B12G0164	9.72	11.28	SEC24C	Protein transport protein Sec24C	NM_001245968.3
B11G0306	9.72	2.95	EOGT	EGF domain-specific O-linked N-acetylglucosamine transferase	XM_003199484.6
A25G0040	9.72	2.84	ADPGK	ADP-dependent glucokinase	NM_001079965.2
B12G0067	9.72	7.61	COMTD1	Catechol O-methyltransferase domain-containing protein 1	
B2G0384	9.72	15.46	ACIN1	Apoptotic chromatin condensation inducer in the nucleus	XM_021471260.2
B3G0834	9.72	14.77	KAT8	Histone acetyltransferase KAT8	NM_212742.2
B21G0456	9.72	9.15	RFC2	Replication factor C subunit 2	NM_001013326.2
A5G1034	9.71	5.90	PRKRIP1	PRKR-interacting protein 1 homolog	NM_001017896.2
B3G0560	9.71	7.42	ZFAND2B	AN1-type zinc finger protein 2B	XM_005164201.5
A22G0702	9.71	20.02	IFI44L	Interferon-induced protein 44-like	
B5G1044	9.71	7.71	LMAN2L	VIP36-like protein	NM_001076733.1
B7G0696	9.71	5.27	ARL14EP	ARL14 effector protein	

A6G0768	9.71	11.57	RBM5	RNA-binding protein 5	XM_068222041.1
B14G0071	9.71	10.59	NR3C1	Glucocorticoid receptor	NM_001020711.3
B18G0501	9.71	7.84	AAGAB	Alpha- and gamma-adaptin-binding protein p34	NM_001002423.1
B8G0981	9.71	1.48	FUT9	4-galactosyl-N-acetylglucosaminide 3-alpha-L-fucosyltransferase 9	NM_001017772.2
A5G0606	9.71	7.05	PRKAA1	5'-AMP-activated protein kinase catalytic subunit alpha-1	NM_001110286.1
B19G0958	9.70	11.10	ATF6B	Cyclic AMP-dependent transcription factor ATF-6 beta	NM_001077720.1
B21G0381	9.70	12.78	RBM4.1	RNA-binding protein 4.1	NM_001115144.1
B13G0005	9.70	8.74	PRORSD1	Prolyl-tRNA synthetase associated domain-containing protein 1	NM_213270.2
A24G0137	9.70	7.76	GNPTG	N-acetylglucosamine-1-phosphotransferase subunit gamma	NM_001002057.2
A10G0886	9.70	10.06	SLC12A2	Solute carrier family 12 member 2	NM_001002080.1
B2G0413	9.70	2.88	LTB4R	Leukotriene B4 receptor 1	NM_001308970.2
A16G0463	9.69	8.89	ENSA	Alpha-endosulfine	NM_001256731.1
A5G0035	9.69	22.52	HSPA5	Endoplasmic reticulum chaperone BiP	NM_213058.1
B23G0485	9.69	4.95	ERBB3	Receptor tyrosine-protein kinase erbB-3	XM_021469802.2
A9G0486	9.69	2.93	ARL4C	ADP-ribosylation factor-like protein 4C	NM_213248.1
B6G0263	9.69	26.14	CG057	Uncharacterized protein C7orf57 homolog	
A25G0140	9.69	41.90	CAT	Catalase	NM_130912.2
B9G0661	9.69	18.54	ABI2	Abl interactor 2	XM_009304512.4
A2G0153	9.68	14.20	JAK1	Tyrosine-protein kinase JAK1	
B5G0025	9.68	4.24	BBLN	Bublin coiled-coil protein	NM_001303023.1
A13G0077	9.68	11.60	VIP	VIP peptides	NM_001351674.1
B24G0299	9.68	7.09	PRKAG2	5'-AMP-activated protein kinase subunit gamma-2	XM_009297511.4
A25G0762	9.68	11.04	SNTB2	Beta-2-syntrophin	XM_002666912.7
B1G0044	9.68	23.86	APP	Amyloid-beta A4 protein	NM_131564.3
B24G0460	9.68	5.82	GRAMD1C	Protein Aster-C	XM_005162729.5
B11G0487	9.68	1.78	PPB	Alkaline phosphatase	
B16G0093	9.67	8.29			
A3G1224	9.67	20.58	TIMP2	Metalloproteinase inhibitor 2	NM_213296.1
A11G0360	9.67	10.42	SLA2	Src-like-adapter 2	XM_001920828.8
B14G0613	9.67	3.90	LOC113113614	A0A6P6QRG3_CARAU lisH domain-containing protein C1711.05-like isoform X2	
B3G0259	9.67	4.00	1C03	Class I histocompatibility antigen, Gogo-C*0202 alpha chain	NM_001017904.2
A10G0185	9.66	2.89	LAGE3	KEOPS complex subunit LAGE3	
B4G0354	9.66	15.23	CALUA	Calumenin-A	NM_001317392.1
A16G0378	9.66	8.74	TSTD3	rhodanese-like domain-containing protein 3	
A25G0561	9.66	11.43	FBXO31	F-box only protein 31	XM_021473864.2
B5G0880	9.66	8.81	MED22	Mediator of RNA polymerase II transcription subunit 22	XM_005171831.3
B1G0067	9.66	3.12	G5714_001175	A0A7J6DK09_9TELE Ig-like domain-containing protein	
A20G0510	9.66	3.95	GMDS	GDP-mannose 4,6 dehydratase	NM_200489.3

B18G0632	9.65	18.26	PLCG2	1-phosphatidylinositol 4,5-bisphosphate phosphodiesterase gamma-2	NM_001044968.1
B25G0263	9.65	2.62	PDCD2L	Programmed cell death protein 2-like	NM_200223.1
B7G1216	9.65	9.56	PFDN2	Prefoldin subunit 2	NM_001045289.1
A3G0845	9.65	7.66	RAB40C	Ras-related protein Rab-40C	NM_001040348.1
A17G0333	9.65	1.68	SH3YL1	SH3 domain-containing YSC84-like protein 1	NM_001423240.1
A9G0420	9.65	8.25	RAB5B	Ras-related protein Rab-5B	NM_212885.1
B8G1035	9.65	5.90	MRPS27	28S ribosomal protein S27, mitochondrial	NM_001003778.2
B13G0075	9.65	8.92	BFSP1	Filensin	NM_001135791.1
B11G0402	9.65	34.17			NM_199699.2
B5G1214	9.64	8.35	ARHGAP25	Rho GTPase-activating protein 25	XM_005165169.5
A23G0456	9.64	7.43	SRGAP3	SLIT-ROBO Rho GTPase-activating protein 3	NM_200444.1
B4G0550	9.64	12.73	KMT2E	Inactive histone-lysine N-methyltransferase 2E	XM_005164910.5
A8G0707	9.64	9.30	RNF34	E3 ubiquitin-protein ligase RNF34	XM_005172464.3
A1G0567	9.64	3.57	BTLA	B- and T-lymphocyte attenuator	XM_068220914.1
A7G0339	9.64	7.80	TP53	Cellular tumor antigen p53	
B18G0695	9.64	13.50	LOC107710375	A0A673LEW5_9TELE Uncharacterized LOC107710375	NM_001044882.2
A24G0397	9.63	7.67	USP12A	Ubiquitin carboxyl-terminal hydrolase 12A	NM_001083556.1
A25G0690	9.63	13.38	GLG1	Golgi apparatus protein 1	XM_068217594.1
A5G0530	9.63	1.21	POU2AF2	POU class 2 homeobox associating-factor 2	NM_001080025.2
B18G0031	9.62	12.39	LTBP4	Latent-transforming growth factor beta-binding protein 4	XM_021467926.2
A12G0191	9.61	7.07	SLC38A10	Putative sodium-coupled neutral amino acid transporter 10	XM_017358552.3
A25G0282	9.61	7.01	FAR1	Fatty acyl-CoA reductase 1	XM_005174315.5
B11G0258	9.61	6.16	CDC6	Cell division control protein 6 homolog	NM_001003495.1
A13G0428	9.61	5.34			NM_200096.1
A5G0294	9.61	11.93	CMKLR1	Chemerin-like receptor 1	
A1G0672	9.61	11.27	MSL3	Male-specific lethal 3 homolog	
A1G0655	9.61	7.92	PWP2	Periodic tryptophan protein 2 homolog	NM_213047.1
B9G0674	9.60	8.58	FN1	Fibronectin	NM_131520.2
B8G0836	9.60	13.00	KIF2A	Kinesin-like protein KIF2A	XM_005166830.5
A11G0073	9.60	5.72	VIP	VIP peptides	NM_213273.1
A2G0829	9.60	10.57	SPPL2B	Signal peptide peptidase-like 2B	NM_001045244.2
A24G0389	9.60	11.44	PAN3	PAN2-PAN3 deadenylation complex subunit PAN3	NM_001105692.2
A13G0296	9.60	9.01	SOBPA	Sine oculis-binding protein homolog A	XM_017358739.3
B11G0302	9.60	6.88	SUCLG2	Succinate--CoA ligase [GDP-forming] subunit beta, mitochondrial (Fragment)	XM_005172872.5
B21G0753	9.60	5.58	PTPN13	Tyrosine-protein phosphatase non-receptor type 13	XM_005161466.5
B5G0497	9.60	12.65	USP2	Ubiquitin carboxyl-terminal hydrolase 2	XM_009301922.4
A7G1173	9.60	11.61	ARNT2	Aryl hydrocarbon receptor nuclear translocator 2	XM_009302940.4
A14G0478	9.60	6.29	ABHD18	Protein ABHD18	NM_001013347.2

B7G0706	9.59	16.35	NAP1L4	Nucleosome assembly protein 1-like 4	NM_001007453.1
B12G0449	9.59	9.13	ZC3H7B	Zinc finger CCCH domain-containing protein 7B	XM_680874.10
B13G0113	9.59	7.62	SFT2D1	Vesicle transport protein SFT2A	XM_068212967.1
B3G0807	9.59	9.76	ABI1	Abl interactor 1	NM_001076774.2
B2G0040	9.58	15.93	PLEKHJ1	Pleckstrin homology domain-containing family J member 1	NM_213288.1
A13G0893	9.58	27.68	G5714_004667	A0A7J6D644_9TELE Uncharacterized protein	NM_001326453.1
B16G0015	9.58	0.60	CREB3L4	Cyclic AMP-responsive element-binding protein 3-like protein 4	XM_021466785.2
B12G0378	9.58	13.99	SVIL	Supervillin	XM_068224487.1
B19G0767	9.57	3.01	SDHAF3	Succinate dehydrogenase assembly factor 3, mitochondrial	NM_001005926.2
A6G0096	9.57	8.36	SLC25A12	glutamate antiporter SLC25A12, mitochondrial	NM_212782.1
A1G0264	9.57	6.74	GLB1	Beta-galactosidase	XM_005160132.5
A2G0140	9.57	8.14	CA021	Uncharacterized protein C1orf21 homolog	NM_001291356.1
A8G0554	9.57	9.77	RHOAD	Rho-related GTP-binding protein RhoA-D	NM_001002445.1
A4G0858	9.57	6.31	ELK3	ETS domain-containing protein Elk-3	NM_001030108.1
A17G0013	9.57	4.59	FAM98B	Protein FAM98B	NM_001079662.1
B14G0122	9.57	8.13	SMAD5	Mothers against decapentaplegic homolog 5	NM_001346309.1
B23G0701	9.57	8.64	CCNT1	Cyclin-T1	XM_005162370.5
B12G0708	9.56	22.34	CD79B	B-cell antigen receptor complex-associated protein beta chain	
B5G0253	9.56	11.62	ISCA1	Iron-sulfur cluster assembly 1 homolog, mitochondrial	NM_001025178.2
A11G0157	9.56	4.27	PTP4A1	K7DYB3_DANRE Protein tyrosine phosphatase 4A1	XM_009305794.4
A13G0765	9.56	9.31	NCOA4	Nuclear receptor coactivator 4	XM_005169532.5
B5G1077	9.56	6.99	ABHD17B	beta hydrolase domain-containing protein 17B	NM_001002744.1
B1G0346	9.56	9.04	RAPGEF2	Rap guanine nucleotide exchange factor 2	XM_005170931.5
A5G0880	9.55	12.23	GCNT4	Beta-1,3-galactosyl-O-glycosyl-glycoprotein beta-1,6-N-acetylglucosaminyltransferase 4	NM_201583.2
A15G0026	9.55	5.59	INPPL1A	Phosphatidylinositol 3,4,5-trisphosphate 5-phosphatase 2A	NM_001039804.2
A15G0433	9.55	12.28	CPD	Carboxypeptidase D	XM_021474349.2
A1G0524	9.55	16.54	TMEM134	Transmembrane protein 134	NM_200369.1
A25G0034	9.55	5.30	GTF2H1	General transcription factor IIH subunit 1	NM_001004596.1
A22G0282	9.55	8.03	CNOT9	CCR4-NOT transcription complex subunit 9	NM_214760.2
B16G0613	9.55	12.01	DERL1	Derlin-1	NM_213444.2
B19G0916	9.55	19.14	NR1D2	Nuclear receptor subfamily 1 group D member 2	NM_131065.1
B20G0080	9.54	15.86	UFL1	E3 UFM1-protein ligase 1	NM_200606.1
B3G0589	9.54	18.51	WIPI2	WD repeat domain phosphoinositide-interacting protein 2	XM_005164125.5
B22G0478	9.54	14.18	LOC113074476	A0A6P6N408_CARAU signaling lymphocytic activation molecule-like	NM_001423249.2

B21G0111	9.54	11.90	ARHGAP26	Rho GTPase-activating protein 26	
B16G0974	9.54	0.07	ZG16	Zymogen granule membrane protein 16	
B20G0311	9.54	2.64	PTK2B	Protein-tyrosine kinase 2-beta	NM_212570.1
B16G0321	9.54	23.45	EMC2	ER membrane protein complex subunit 2	NM_213544.1
A8G0909	9.54	8.29	PLOD1	Procollagen-lysine,2-oxoglutarate 5-dioxygenase 1	NM_001077742.2
A13G0622	9.54	6.09	SI:CH211-233A24.2	Transmembrane protein KIAA1109 homolog	NM_001013308.2
B18G0403	9.54	6.83	MALT1	Mucosa-associated lymphoid tissue lymphoma translocation protein 1	XM_694813.10
A4G0326	9.53	6.39	LOC113040936	A0A6P6J3Z4_CARAU uncharacterized protein LOC113040936 isoform X2	
B3G0458	9.53	7.90	GMIP	GEM-interacting protein	XM_689070.9
A21G0380	9.53	10.36	LIFR	Leukemia inhibitory factor receptor	XM_009295324.4
A5G0798	9.53	1.00	ERAL1	GTPase Era, mitochondrial	NM_001128747.1
A19G0052	9.53	7.39	FEL	Fish-egg lectin	NM_001282005.1
A17G0045	9.53	9.33	PSEN1	Presenilin-1	XM_005169883.5
A25G0191	9.53	4.95	CK024	Uncharacterized protein C11orf24 homolog	XM_685812.10
B19G0775	9.53	8.21	PTP4A3	Protein tyrosine phosphatase type IVA 3	NM_213181.1
B13G0087	9.53	22.10	IFNGR1L	Interferon gamma receptor 1-like	NM_001004108.2
A16G0589	9.53	13.11	LRATD2	Protein LRATD2	NM_001017837.1
A19G0869	9.52	2.62	POLR1F	DNA-directed RNA polymerase I subunit RPA43	NM_001007153.1
B19G0806	9.52	2.41	POLR3GL	DNA-directed RNA polymerase III subunit RPC7-like	NM_001004639.1
B2G1057	9.52	14.83	GIPC1	PDZ domain-containing protein GIPC1	NM_200930.1
A1G0785	9.52	4.75	STK16	threonine-protein kinase 16	NM_001017630.1
A3G0460	9.52	7.87	RMI2	RecQ-mediated genome instability protein 2	
B15G0070	9.52	0.19	CLDN4	Claudin-4	NM_131765.1
A20G0608	9.51	1.52	SYNE2	Nesprin-2	NM_001365331.1
A10G0371	9.51	0.27	ADGRG2	Adhesion G-protein coupled receptor G2	XM_009305521.4
B4G0383	9.51	2.76	CMASA	N-acylneuraminate cytidylyltransferase A	NM_001040252.2
B9G0802	9.51	24.66	SCARA5	Scavenger receptor class A member 5	
A9G0468	9.51	8.66	COPS8	COP9 signalosome complex subunit 8	NM_200229.2
A23G0616	9.51	78.20	NR4A1	Nuclear receptor subfamily 4 group A member 1	NM_001002173.1
B9G0614	9.51	1.70	TTF2	Transcription termination factor 2	XM_005168619.5
B9G0484	9.51	2.12	ESYT3	Extended synaptotagmin-3	NM_001123233.1
B22G0992	9.50	4.63	TERF2IP	Telomeric repeat-binding factor 2-interacting protein 1	
A20G0329	9.50	6.57	PLA2G7	Platelet-activating factor acetylhydrolase	NM_213189.2
B24G0012	9.50	7.91	ZBTB14	Zinc finger and BTB domain-containing protein 14	XM_009297676.4
B11G0338	9.50	14.11	SLC2A9	Solute carrier family 2, facilitated glucose transporter member 9	NM_001044993.1

A24G0002	9.50	19.12	RMDN1	Regulator of microtubule dynamics protein 1	
B3G1103	9.50	10.82	HIRIP3	HIRA-interacting protein 3	NM_199823.1
B11G0750	9.50	8.91	OGA	Protein O-GlcNAcase	
B11G0287	9.50	20.98	LOC107696829	A0A671T4F9_9TELE Uncharacterized LOC107696829	XM_068219301.1
B16G0583	9.49	7.56	PTPN3	Tyrosine-protein phosphatase non-receptor type 3	XM_682838.10
B24G0381	9.49	12.87	PDE7A	High affinity cAMP-specific 3',5'-cyclic phosphodiesterase 7A	XM_005162668.5
B4G0084	9.49	22.75	CYP2J4	Cytochrome P450 2J4	NM_200620.1
B6G0190	9.49	27.33	CAVIN2	Caveolae-associated protein 2	NM_001004584.1
A3G0455	9.49	18.54	LOC107568934	A0A672K614_SINGR Interleukin-4 receptor subunit alpha-like	
B5G0464	9.49	21.02	NCF1	Neutrophil cytosolic factor 1	NM_001030071.2
A15G0624	9.49	8.61	MED29	Mediator of RNA polymerase II transcription subunit 29	NM_173271.2
B21G0671	9.49	3.53	ARRDC1	Arrestin domain-containing protein 1	NM_001365234.1
B16G0648	9.49	8.61	LOC107699518	A0A671Q603_9TELE Zinc finger protein 43-like	NM_001326353.1
A11G0196	9.49	2.10	ATP5F1B	ATP synthase subunit beta, mitochondrial	XM_001920527.7
B18G0243	9.49	17.69	TCB2	Transposable element Tcb2 transposase	XM_068220572.1
A3G1160	9.48	4.26	SLC25A17	Peroxisomal membrane protein PMP34	XM_001922874.8
A15G0805	9.48	7.24	PPP5C	threonine-protein phosphatase 5	XM_005157376.5
B6G0243	9.48	0.64	PTTG1IP	Pituitary tumor-transforming gene 1 protein-interacting protein	NM_001326362.1
A2G0873	9.48	8.34	PRMT5	Protein arginine N-methyltransferase 5	NM_001007183.2
B13G0935	9.48	9.62			NM_001007299.1
A8G0377	9.48	10.48	FZR1	Fizzy-related protein homolog	NM_200253.1
B4G0199	9.48	4.25	MDFIC	MyoD family inhibitor domain-containing protein	NM_001328013.1
A6G0532	9.48	17.62	DLG1	Disks large homolog 1	XM_068221618.1
B2G0786	9.48	6.95	ANGPTL4	Angiopoietin-related protein 4	NM_001256203.1
A24G0575	9.48	7.59	EEF1E1	Eukaryotic translation elongation factor 1 epsilon-1	NM_001386550.1
A15G0668	9.48	5.11	LORF2	LINE-1 retrotransposable element ORF2 protein	
A5G0280	9.48	6.52	GATC	Glutamyl-tRNA(Gln) amidotransferase subunit C, mitochondrial	NM_001301329.1
B11G0799	9.48	10.70			NM_213275.2
B5G0746	9.48	1.94	ANXA3	Annexin A3	NM_001004632.2
B9G0353	9.48	7.10	RABL3	Rab-like protein 3	XM_068223354.1
B10G0534	9.47	7.36	RFXAP	Regulatory factor X-associated protein	NM_001326367.1
A18G0002	9.47	16.38	DDB1	DNA damage-binding protein 1	NM_200626.1
A3G0724	9.47	6.24	PIH1D1	PIH1 domain-containing protein 1	NM_001159928.1
B22G0420	9.47	11.11	LOC113099137	A0A6P6PGY6_CARAU uncharacterized protein LOC113099137	
A17G0377	9.47	22.06	QKIA	Protein quaking-A	XM_009293120.4

A23G0558	9.47	6.26	CTNNBIP1	Beta-catenin-interacting protein 1	NM_131594.1
A14G0724	9.47	11.75	IMMT	MICOS complex subunit MIC60	NM_001001401.1
B21G0921	9.47	22.91	SCARB2	Lysosome membrane protein 2	NM_001365142.1
B12G0116	9.46	15.49	BNIP3	adenovirus E1B 19 kDa protein-interacting protein 3	XM_005172968.5
A11G0565	9.46	66.84	TKT	Transketolase	XM_691072.9
A4G0849	9.46	29.53	OPTN	Optineurin	XM_021474935.2
B3G0373	9.46	7.14	TUFM	Elongation factor Tu, mitochondrial	NM_001013505.2
B19G0320	9.46	16.89	ARHGEF2	Rho guanine nucleotide exchange factor 2	NM_001423384.1
B15G0535	9.46	7.41	SUSD6	Sushi domain-containing protein 6	NM_001080000.2
A5G0230	9.46	6.01	COQ5	2-methoxy-6-polyprenyl-1,4-benzoquinol methylase, mitochondrial	NM_001004541.1
B3G0198	9.45	9.11	PLBD1	Phospholipase B-like 1	NM_001130791.1
B21G0809	9.45	17.78	CCNG2	Cyclin-G2	NM_213172.1
A1G0709	9.45	7.23	DCUN1D2	DCN1-like protein 2	NM_001002156.2
B13G0340	9.45	6.03	ABHD12	Lysophosphatidylserine lipase ABHD12	XM_068213074.1
B9G0047	9.45	49.07	NLRC3	Protein NLRC3	XM_068222163.1
B8G0622	9.45	18.14	TNFRSF14	Tumor necrosis factor receptor superfamily member 14	
B9G0342	9.45	6.88	FHIP1B	FHF complex subunit HOOK interacting protein 1B	NM_001130857.1
A20G0372	9.44	4.26	SI:DKEY-97O5.1	Uncharacterized protein C1orf112 homolog	NM_001020659.2
A13G0342	9.44	12.57			NM_213389.1
B24G0428	9.44	3.21	ACOT9	Acyl-coenzyme A thioesterase 9, mitochondrial	NM_001037399.2
B3G0970	9.44	7.41	CYTH3	Cytohesin-3	XM_690136.8
B19G0219	9.44	9.65	PBXIP1	Pre-B-cell leukemia transcription factor-interacting protein 1	XM_005159476.5
B3G0900	9.44	5.92	RNF113A	E3 ubiquitin-protein ligase RNF113A	NM_001004536.1
B11G0790	9.44	16.19			NM_199774.3
A15G0247	9.44	11.28	BAG6	Large proline-rich protein BAG6	XM_005157765.3
B20G0211	9.43	7.15	ECHDC2	Enoyl-CoA hydratase domain-containing protein 2, mitochondrial	NM_212788.2
A15G0727	9.43	7.52	MRPS22	28S ribosomal protein S22, mitochondrial	
A17G0003	9.43	8.79	UBR7	Putative E3 ubiquitin-protein ligase UBR7	
B18G0516	9.43	3.96	C15ORF39	Uncharacterized protein C15orf39	XM_005159151.5
B3G0835	9.43	9.88	RNF25	E3 ubiquitin-protein ligase RNF25	NM_201183.1
A8G0865	9.43	13.09	UBL4A	Ubiquitin-like protein 4A	NM_200300.1
A20G0600	9.43	8.97	TMEM165	Transmembrane protein 165	NM_212683.2
B21G0945	9.43	7.81	TNFRSF14	Tumor necrosis factor receptor superfamily member 14	NM_001430990.1
A9G0477	9.42	11.54	PARP14	Protein mono-ADP-ribosyltransferase PARP14	NM_001114447.2
B10G0457	9.42	11.41	ZPR1	Zinc finger protein ZPR1	NM_213108.1
A18G0459	9.42	9.30	PIAS1	E3 SUMO-protein ligase PIAS1	XM_021474413.2
B19G0552	9.42	18.93	SGCE	Epsilon-sarcoglycan	XM_021468002.2

A19G0361	9.42	8.22	ILF2	Interleukin enhancer-binding factor 2 homolog	NM_213236.2
A7G1101	9.42	3.33	PRMT3	Protein arginine N-methyltransferase 3	NM_001017655.1
A23G0136	9.42	5.00	TEAD3	Transcriptional enhancer factor TEF-5	XM_068216846.1
A17G0867	9.42	8.05	AKT1	threonine-protein kinase	NM_001281801.1
B6G0229	9.41	9.69	SP3	Transcription factor Sp3	XM_068221897.1
A10G0699	9.41	20.75	EWSR1	RNA-binding protein EWS	NM_001115138.2
A5G1318	9.41	4.50	GTF3A	Transcription factor IIIA	NM_001003866.1
A7G0734	9.41	16.30	SLTM	SAFB-like transcription modulator	NM_213333.2
B2G0423	9.41	6.60	PWWP3B	PWWP domain-containing DNA repair factor 3B	
B20G0937	9.41	12.24	ACTR10	Actin-related protein 10	NM_200170.1
B19G0341	9.41	10.99	EIF3EB	Eukaryotic translation initiation factor 3 subunit E-B	NM_001362621.1
B4G0638	9.41	7.97	CRELD2	Cysteine-rich with EGF-like domain protein 2	NM_200523.1
B19G0757	9.41	5.49			NM_001044932.1
B22G0097	9.41	3.22	G5714_004116	A0A7J6DBD1_9TELE Uncharacterized protein	XM_021469384.2
B5G1004	9.41	9.44	DPP7	Dipeptidyl peptidase 2	XM_005171846.3
B17G0767	9.41	11.48	GSKIP	GSK3-beta interaction protein	NM_213435.2
B5G1215	9.40	13.37	CDS2	Phosphatidate cytidylyltransferase 2	NM_201186.1
B24G0426	9.40	10.58	SAT1	Diamine acetyltransferase 1	NM_001093748.2
B14G0517	9.40	3.79	THG1L	Probable tRNA(His) guanylyltransferase	NM_001007455.2
A10G0652	9.39	59.54	BNIP3L	adenovirus E1B 19 kDa protein-interacting protein 3-like	NM_205571.2
A3G0131	9.39	23.21	RANGAP1	Ran GTPase-activating protein 1	NM_001076616.1
B3G0361	9.39	5.98	FARSA	Phenylalanine--tRNA ligase alpha subunit	NM_001045295.2
B8G0794	9.39	7.89	ACBD6	Acyl-CoA-binding domain-containing protein 6	NM_001025455.1
B12G0288	9.38	19.42	RNF40	E3 ubiquitin-protein ligase BRE1B	XM_068224659.1
B12G0435	9.38	11.03			NM_001076595.2
B18G0013	9.38	7.48	UBE2Q2	Ubiquitin-conjugating enzyme E2 Q2	NM_001308605.1
A20G0707	9.38	2.79	TMED5	Transmembrane emp24 domain-containing protein 5	NM_200403.1
A19G0250	9.38	9.81	ABCF1	ATP-binding cassette sub-family F member 1	XM_021468041.2
B10G0323	9.37	10.84	MED11	Mediator of RNA polymerase II transcription subunit 11	NM_001160035.1
B12G0253	9.37	7.00	CASP7	Caspase-7	NM_001020607.2
B3G1087	9.37	6.24	LOC107754555	A0A673GX55_9TELE Zinc finger protein 236-like	
A5G0730	9.37	4.13	GNB2	G(T) subunit beta-2	NM_001013497.1
A24G0607	9.37	6.68	ABI1	Abl interactor 1	XM_009297227.4
B9G0012	9.37	4.99	CNOT11	CCR4-NOT transcription complex subunit 11	NM_001089341.1
B6G0060	9.37	33.95	TXNRD3	Thioredoxin reductase 3	NM_183072.2
B5G1290	9.37	8.40	AUTS2	Autism susceptibility gene 2 protein homolog	XM_021476418.2
A15G0826	9.37	14.05			
B16G0246	9.37	8.44	SMARCC1	SNF complex subunit SMARCC1	XM_003200246.6
A16G0278	9.36	17.85	TGFBR2	TGF-beta receptor type-2	NM_182855.3

A11G0267	9.36	5.76	FGD5	FYVE, RhoGEF and PH domain-containing protein 5	NM_205647.1
A18G0489	9.36	11.02	LONP2	Lon protease homolog 2, peroxisomal	NM_001008573.2
B4G0015	9.36	13.01	TMEM214	Transmembrane protein 214	NM_001256176.1
A10G0028	9.36	10.97	SMAD4	Mothers against decapentaplegic homolog 4	NM_001122700.2
B19G0844	9.35	2.57	TRIM65	Tripartite motif-containing protein 65	XM_017352169.3
A1G0375	9.35	5.54	TUBGCP3	Gamma-tubulin complex component 3 homolog	XM_005170995.5
B9G0892	9.35	10.57	PCBP2	Poly(rC)-binding protein 2	NM_201192.1
A3G0873	9.35	10.33	NKIRAS2	NF-kappa-B inhibitor-interacting Ras-like protein 2	NM_001003433.1
B4G0164	9.35	3.29	MRPS18A	39S ribosomal protein S18a, mitochondrial	NM_001030114.1
B16G0918	9.34	5.21	MYH10	Myosin-10	XM_005157931.5
A5G0397	9.34	5.87	PHF23A	PHD finger protein 23A	NM_001013499.1
A22G0038	9.34	4.99	NUAK2	NUAK family SNF1-like kinase 2	XM_683672.10
B23G0157	9.34	8.82	BCL2L14	Apoptosis facilitator Bcl-2-like protein 14	
B24G0236	9.34	19.09	A0A671PDH5	A0A671PDH5_9TELE Chemerin	NM_001128775.1
A3G0386	9.34	9.51	SHFL	Shiftless antiviral inhibitor of ribosomal frameshifting protein homolog	NM_001105527.1
A2G1148	9.34	9.87	AP1M1	AP-1 complex subunit mu-1	NM_001331127.1
A1G0658	9.34	7.31	TIMMDC1	Complex I assembly factor TIMMDC1, mitochondrial	
B19G0465	9.34	3.65	MTERF3	Transcription termination factor 3, mitochondrial	NM_001002615.1
B16G0038	9.33	10.25	WDTC1	WD and tetratricopeptide repeats protein 1	XM_005169770.5
A15G0676	9.33	10.92	GBE1	1,4-alpha-glucan-branching enzyme	XM_068213679.1
B21G0181	9.33	6.25	FAM114A1	Protein FAM114A2	XM_021469226.2
B5G0106	9.33	0.57	KIAA1671	Uncharacterized protein KIAA1671	NM_001267589.1
B23G0497	9.33	7.41	FITM2	Acyl-coenzyme A diphosphatase FITM2	NM_001020498.1
A4G0888	9.32	3.91	MRPS18A	28S ribosomal protein S18a, mitochondrial	NM_001030114.1
A5G0553	9.32	9.20	CYB5D2	Neuferricin	NM_001102674.1
B5G0006	9.32	3.64	THAP1	THAP domain-containing protein 1	NM_001423860.1
A13G0582	9.32	20.32	ADSS2	Adenylosuccinate synthetase isozyme 2	NM_131429.1
B4G0928	9.32	14.75	TERF2	Telomeric repeat-binding factor 2	XM_021474925.2
B3G0549	9.31	3.90	NSMCE1	Non-structural maintenance of chromosomes element 1 homolog	
B2G0105	9.31	13.86	SPINW	Spindlin-W	XM_001339007.9
A20G0495	9.31	10.14	TMEM251	Transmembrane protein 251	NM_001033746.2
B8G0537	9.31	15.00	TRAF3IP3	TRAF3-interacting JNK-activating modulator	XM_003199090.6
B7G0911	9.31	2.73	MRPL52	39S ribosomal protein L52, mitochondrial	
A5G1216	9.31	8.23	PSD3	PH and SEC7 domain-containing protein 3	XM_009299757.4
A4G0811	9.30	8.59	HSPA14	Heat shock 70 kDa protein 14	NM_001045076.1

B25G0282	9.30	6.96	PARP16	Protein mono-ADP-ribosyltransferase PARP16	NM_212906.1
B23G0869	9.30	0.44			
B14G0192	9.30	9.15	RAP2C	Ras-related protein Rap-2c	NM_001007055.1
B22G0620	9.30	10.18	MFNG	Beta-1,3-N-acetylglucosaminyltransferase manic fringe	NM_001007788.1
B1G0659	9.30	5.04	CD5	T-cell surface glycoprotein CD5	
A14G0129	9.30	9.56	SYVN1	E3 ubiquitin-protein ligase synoviolin	NM_212735.2
B18G0132	9.30	2.96	TSEN34	tRNA-splicing endonuclease subunit Sen34	NM_001386748.1
B21G0156	9.29	6.20	DDX52	Probable ATP-dependent RNA helicase DDX52	NM_001044315.2
B24G0636	9.29	4.76	EEF1E1	Eukaryotic translation elongation factor 1 epsilon-1	NM_001386550.1
B20G0714	9.29	10.04	HSF2	Heat shock factor protein 2	NM_131867.1
B14G0780	9.29	4.65	A33	Zinc-binding protein A33	NM_001044961.1
B14G0815	9.29	18.36	RPS6KA6	Ribosomal protein S6 kinase alpha-6	NM_200073.1
B10G0090	9.29	4.30	SMAD2	Mothers against decapentaplegic homolog 2	NM_131366.3
B3G0720	9.29	14.85	ZNF207	BUB3-interacting and GLEBS motif-containing protein ZNF207	XM_009299526.4
B25G0605	9.29	3.53	ANO10	Anoctamin-10	NM_001077382.1
B12G0242	9.29	4.90	ZDHHC6	Palmitoyltransferase ZDHHC6	NM_001204157.1
B2G1096	9.29	5.73	KLF9	Krueppel-like factor 9	NM_001077329.2
B16G0569	9.28	6.92	SEC61B	Protein transport protein Sec61 subunit beta	
A19G0310	9.28	8.44	ATP6V1C1A	V-type proton ATPase subunit C 1-A	NM_001005772.2
A14G0695	9.28	5.31	RNF4	E3 ubiquitin-protein ligase RNF4	NM_001386248.1
A3G1010	9.28	11.66	UBE2Z	Ubiquitin-conjugating enzyme E2 Z	NM_001002330.5
B22G0589	9.28	12.19	AHR	Aryl hydrocarbon receptor	NM_131264.1
A1G0744	9.28	14.33	SON	Protein SON	XM_068217693.1
A6G0006	9.28	5.54	LOC115823849	A0A6J2WJ84_CHACN LOW QUALITY PROTEIN: uncharacterized protein LOC115823849	XM_021476964.2
B3G0420	9.28	14.39	PLPP3	Phospholipid phosphatase 3	NM_001079978.1
A22G0355	9.28	4.14	TM2D1	TM2 domain-containing protein 1	XM_068216222.1
B7G1170	9.28	20.52	ROHU_024072	A0A498MNW3_LABRO Transmembrane 4 L6 family member 5-like protein	NR_125779.1
B8G0107	9.27	1.29	TP73	Tumor protein p73	XM_068222888.1
B19G0694	9.27	6.25	TRIT1	tRNA dimethylallyltransferase	NM_001044774.1
A12G0420	9.27	5.09	MRPL27	39S ribosomal protein L27, mitochondrial	NM_001020680.2
A17G0562	9.27	13.67	MGMT	Methylated-DNA--protein-cysteine methyltransferase	NM_001256246.1
A6G0501	9.27	19.01	INPP5D	Phosphatidylinositol 3,4,5-trisphosphate 5-phosphatase 1	XM_021471767.2
A2G0527	9.27	18.20	GYG1	Glycogenin-1	NM_213510.1
A3G0993	9.27	4.51	ARHGAP27	Rho GTPase-activating protein 27	XM_689796.8
B12G0463	9.27	10.31	GSPT1	Eukaryotic peptide chain release factor GTP-binding subunit ERF3A	NM_001003992.1

A3G0819	9.26	6.62	SEPTIN9	Septin-9	XM_009299472.4
B10G0053	9.26	8.76	AUH	Methylglutaconyl-CoA hydratase, mitochondrial	NM_001003576.2
B14G0679	9.26	465.48	ALDOB	Fructose-bisphosphate aldolase B	NM_194367.3
A15G0870	9.26	5.23	NLRP1	NACHT, LRR and PYD domains-containing protein 1	XM_005173355.5
A8G0242	9.26	4.25	LDLR	Low-density lipoprotein receptor	XM_021478380.2
A8G0094	9.26	1.23	FUT9	4-galactosyl-N-acetylglucosaminide 3-alpha-L-fucosyltransferase 9	NM_001007454.2
B6G0043	9.26	3.14	ADAP1	Arf-GAP with dual PH domain-containing protein 1	NM_001002715.1
B8G0888	9.26	16.52	PIM2	threonine-protein kinase pim-2	
B15G0469	9.25	8.14	ARHGEF12	Rho guanine nucleotide exchange factor 12	XM_009291733.4
B14G0229	9.25	20.58	UBE2A	Ubiquitin-conjugating enzyme E2 A	NM_201273.1
B5G0154	9.25	7.29	GAK	Cyclin-G-associated kinase	XM_009301214.4
B1G0881	9.25	23.64	RAD23A	UV excision repair protein RAD23 homolog A	NM_001003739.1
B11G0279	9.25	7.75	SLC4A10	Sodium-driven chloride bicarbonate exchanger	NM_200291.1
A23G0814	9.25	3.30	SLC10A7	bile acid cotransporter 7	NM_001003420.1
B17G0471	9.25	6.32	MTMR9	Myotubularin-related protein 9	NM_001077142.1
A19G0615	9.25	3.14	A0A672PMM6	A0A672PMM6_SINGR Si:ch73-347e22.8	
B21G0518	9.25	4.63	POLD3	DNA polymerase delta subunit 3	NM_001042769.2
A18G0513	9.25	10.39	ZC3H18	Zinc finger CCCH domain-containing protein 18	XM_068214774.1
B22G0802	9.24	4.37	SEMA4E	Semaphorin-4E	XM_005170470.5
A11G0197	9.24	6.91	ABCB11	Bile salt export pump	NM_212610.1
B2G0748	9.24	10.47	RABGGTB	Geranylgeranyl transferase type-2 subunit beta	NM_213112.2
A4G0514	9.24	10.93	GATA3	Transcription factor GATA-3	NM_131211.1
A7G0470	9.24	6.68	CRABP1	Cellular retinoic acid-binding protein 1	NM_001001842.1
A22G0338	9.24	7.75	RTCA	RNA 3'-terminal phosphate cyclase	NM_199536.2
B4G0581	9.23	10.18	ACOT4	Peroxisomal succinyl-coenzyme A thioesterase	NM_001386815.1
A13G0728	9.23	3.33	LOC113112981	A0A6P6QN79_CARAU collagen alpha-1(XIII) chain-like isoform X21	NM_001111187.1
A6G0810	9.23	5.29	FAM107A	Actin-associated protein FAM107A	XR_002459113.2
A19G0438	9.23	6.09	SNRNP40	U5 small nuclear ribonucleoprotein 40 kDa protein	NM_200322.1
A23G0210	9.23	5.63	PPP4R2A	threonine-protein phosphatase 4 regulatory subunit 2-A	NM_001115140.1
B2G0449	9.23	11.76	PDCD10	Programmed cell death protein 10	NM_212933.1
B18G0415	9.23	7.03	ELL2	RNA polymerase II elongation factor ELL2	XM_689088.8
A17G0056	9.23	6.95	NUMB	Protein numb homolog	NM_001040406.1
A9G0497	9.23	13.35	MMADHC	Cobalamin trafficking protein CblD	NM_205594.2
A14G0010	9.23	8.26	STX18	Syntaxin-18	NM_001024425.1
A18G0098	9.22	14.15	SRPRA	Signal recognition particle receptor subunit alpha	XM_692426.10

A13G0249	9.22	16.19	G5714_013627	A0A7J6CHA8_9TELE SAM domain-containing protein	NM_001080664.1
B17G0546	9.22	5.21	RCOR1	REST corepressor 1	NM_001080041.1
B2G0738	9.22	5.46	YIPF1	Protein YIPF1	NM_001003991.1
A7G1329	9.22	1.83	NEK1	threonine-protein kinase Nek1	XM_017356939.3
A16G0073	9.22	1.56	SERPINB1	Leukocyte elastase inhibitor	NM_001109730.1
B20G0431	9.22	5.59	GMDS	GDP-mannose 4,6 dehydratase	NM_200489.3
A17G0373	9.21	1.61	TMEM30B	Cell cycle control protein 50B	XM_005158776.5
B16G1015	9.21	6.38	TAF2	Transcription initiation factor TFIID subunit 2	XM_021466622.2
A22G0313	9.21	6.44	RPP21	Ribonuclease P protein subunit p21	NM_001003530.2
B4G0254	9.21	5.42	HSPA14	Heat shock 70 kDa protein 14	NM_001045076.1
B22G0664	9.20	5.55	VPS4B	Vacuolar protein sorting-associated protein 4B	XM_009298174.4
B15G0042	9.20	9.76	KPNA4	Importin subunit alpha-3	NM_201305.1
B4G0464	9.20	4.50			
B5G0590	9.20	6.82	SERINC5	Serine incorporator 5	NM_213514.1
B15G0512	9.20	10.49	POLDIP2	Polymerase delta-interacting protein 2	XM_005157562.5
A1G0016	9.20	4.91	THUMPD1	THUMP domain-containing protein 1	NM_205657.2
B20G0046	9.20	3.17	MMACHC	alkylcobalamin dealkylase	
B6G0268	9.19	8.87	STK24	threonine-protein kinase 24	XM_005165833.5
B2G0051	9.19	17.80	BTBD2	POZ domain-containing protein 2	XM_005168444.5
A19G0734	9.19	3.99	BOP1	Ribosome biogenesis protein bop1	NM_001077735.2
A12G0085	9.19	3.05	TECTA	Alpha-tectorin	XM_017358590.3
B12G0392	9.19	5.62	ZCCHC4	rRNA N6-adenosine-methyltransferase ZCCHC4	NM_001113599.1
B23G0592	9.18	12.36	SLC25A33	Solute carrier family 25 member 33	NM_213157.1
A23G0688	9.18	15.00	CBX5	Chromobox protein homolog 5	NM_001080184.1
B21G0391	9.18	9.62	MARK2	threonine-protein kinase MARK2	XM_009295511.4
A25G0543	9.18	6.58	PARP16	Protein mono-ADP-ribosyltransferase PARP16	NM_212906.1
A11G0741	9.18	15.29	NABP2	SOSS complex subunit B1	NM_214752.1
B7G0516	9.18	6.04	MCUR1	Mitochondrial calcium uniporter regulator 1	NM_001351801.1
A3G0286	9.18	9.60	AMDHD2	N-acetylglucosamine-6-phosphate deacetylase	NM_205681.1
A21G0777	9.18	6.40	TCERG1	Transcription elongation regulator 1	XM_009295682.4
B21G0378	9.17	2.53	SLC29A2	Equilibrative nucleoside transporter 2	NM_001012501.2
A7G0780	9.17	5.41	OGFOD1	Prolyl 3-hydroxylase OGFOD1	NM_199689.3
A11G0124	9.17	5.90	NR2F6	Nuclear receptor subfamily 2 group F member 6	XM_005155658.5
B22G0251	9.17	17.30	A0A672LB75	A0A672LB75_SINGR Uncharacterized protein	XM_068216417.1
B10G0588	9.17	4.98	MRPL48	39S ribosomal protein L48, mitochondrial	NM_001013281.1
B22G0319	9.17	15.85	LOC113078541	A0A6P6NE76_CARAU uncharacterized protein LOC113078541	XM_021470864.2
A8G0527	9.17	15.19	ARIH2	E3 ubiquitin-protein ligase ARIH2	XM_009303988.4
B16G0210	9.17	4.02	LOC113115846	A0A6P6R1U7_CARAU uncharacterized protein LOC113115846	XM_692272.10

B12G0687	9.17	1.06	DLX4B	Homeobox protein Dlx4b	NM_131318.1
B13G0646	9.17	1.49	RTL1	Retrotransposon-like protein 1	XM_021480767.2
A24G0527	9.16	9.23	VPS41	Vacuolar protein sorting-associated protein 41 homolog	NM_001423787.1
B12G0238	9.16	8.92	GPAM	Glycerol-3-phosphate acyltransferase 1, mitochondrial	XM_009306794.4
A6G0190	9.16	9.69	WDR12	Ribosome biogenesis protein wdr12	NM_199557.1
A10G0673	9.16	12.59	DEPDC5	GATOR complex protein DEPDC5	XM_068223938.1
A5G1293	9.16	12.16	ISCA1	Iron-sulfur cluster assembly 1 homolog, mitochondrial	
A19G0213	9.16	17.34	DDX39B	Spliceosome RNA helicase DDX39B	NM_200943.3
A13G0455	9.16	11.34	GSTO1	Glutathione S-transferase omega-1	NM_200317.1
A18G0653	9.16	46.97	ARF4	ADP-ribosylation factor 4	NM_199275.1
B2G0761	9.16	7.93	PLD1	Phospholipase D1	NM_001160095.1
B25G0606	9.16	7.61	SNRK	threonine-protein kinase	NM_200833.1
B20G0506	9.16	5.42	ASAP2	Arf-GAP with SH3 domain, ANK repeat and PH domain-containing protein 2	NM_001044933.2
B7G0088	9.15	15.46	TOLLIP	Toll-interacting protein	NM_207061.2
A18G0307	9.15	6.95	RASGRP1	RAS guanyl-releasing protein 1	XM_005173658.5
A20G0772	9.15	7.54	RDH14	Retinol dehydrogenase 14	NM_001047190.1
B18G0103	9.15	21.88	EHD2	EH domain-containing protein 2	NM_200063.1
B15G0918	9.15	14.91	MAP4K5	Mitogen-activated protein kinase kinase kinase kinase 5	XM_005173433.3
A15G0549	9.14	5.84	ACAD8	Isobutyryl-CoA dehydrogenase, mitochondrial	NM_201155.1
A2G1119	9.14	16.16	RAB31	Ras-related protein Rab-31	XM_692156.7
B22G0875	9.14	10.41	MCRIP2	MAPK regulated corepressor interacting protein 2	NM_001045127.3
B16G0441	9.14	5.35	MBOAT7	Lysophospholipid acyltransferase 7	NM_200537.1
A2G1081	9.14	15.23	CUL1	Cullin-1	NM_199659.2
A1G0918	9.14	4.21	NANS	Sialic acid synthase	NM_206829.1
B14G0050	9.13	10.18	CCNG1	Cyclin-G1	NM_199481.1
B22G0650	9.13	3.57	DPH5	Diphthine methyl ester synthase	NM_001007386.2
A21G0287	9.13	8.16	GSTT1	Glutathione S-transferase theta-1	NM_200584.1
A16G0897	9.13	7.08	MRS2	Magnesium transporter MRS2 homolog, mitochondrial	XM_688529.9
B6G0025	9.13	8.82	IL2RB	Interleukin-2 receptor subunit beta	
A9G0321	9.13	6.19	GZMB	Granzyme B	NM_001128550.1
A24G0367	9.13	9.20	COPS5	COP9 signalosome complex subunit 5	NM_200725.1
B14G0340	9.13	7.21	NIPA2	Magnesium transporter NIPA2	XM_021481008.2
B21G0288	9.13	6.16	RBM22	Pre-mRNA-splicing factor RBM22	NM_213214.1
B7G0570	9.13	18.56	ATG13	Autophagy-related protein 13	NM_200433.2
A2G0106	9.13	11.35	CDC73	Parafibromin	NM_001291396.1
A9G0591	9.13	8.94	UMPS	Uridine 5'-monophosphate synthase	NM_200174.2
B25G0662	9.12	4.24	MR1	Major histocompatibility complex class I-related gene protein	XM_021470779.2
A9G0692	9.12	3.16	NR1I2	Nuclear receptor subfamily 1 group I member 2	NM_001098617.2
B15G0579	9.12	5.14	GIT1	ARF GTPase-activating protein GIT1	XM_005157670.4

B7G0762	9.12	14.92	RAB8B	Ras-related protein Rab-8B	NM_001099259.1
B3G0597	9.12	13.36	TNRC18	Trinucleotide repeat-containing gene 18 protein	XM_005164179.4
A25G0504	9.11	18.21	DNAJA4	DnaJ homolog subfamily A member 4	XM_068217564.1
B16G0400	9.11	8.38	FHL3	Four and a half LIM domains protein 3	XM_068219340.1
A10G0766	9.11	2.34	SREK1IP1	Protein SREK1IP1	
A3G0288	9.11	1.47	SLC25A19	Mitochondrial thiamine pyrophosphate carrier	NM_205715.1
A18G0500	9.11	6.91	PSME3IP1	PSME3-interacting protein	XM_068214729.1
A8G0079	9.11	33.09	ACAA2	3-ketoacyl-CoA thiolase, mitochondrial	NM_213052.1
B13G0460	9.11	5.43	CALHM2	Calcium homeostasis modulator protein 2	NM_201292.1
B21G0633	9.11	9.70	ATP2A2	endoplasmic reticulum calcium ATPase 2	XM_005161196.5
B4G0633	9.11	8.15	TBC1D22A	TBC1 domain family member 22A	NM_214799.1
B19G0201	9.11	16.42	EXOSC7	Exosome complex component RRP42	NM_001017585.1
A5G0037	9.11	17.10	RAB35	Ras-related protein Rab-35	NM_001386745.1
A1G0612	9.10	7.73	PPP1R9A	Neurabin-1	XM_068221367.1
A17G0350	9.10	6.12	RCOR1	REST corepressor 1	NM_001080041.1
B20G0696	9.10	1.90	SCARA3	Scavenger receptor class A member 3	XM_005160848.5
A18G0504	9.10	13.40	DYE	Nuclear pore complex protein Nup93	NM_130981.2
B20G0304	9.10	2.11	ATRAID	All-trans retinoic acid-induced differentiation factor	
B2G0977	9.10	6.94	RPAP2	RNA polymerase II subunit B1 CTD phosphatase Rpap2	NM_001277930.1
B5G1181	9.10	10.93	VMA21	Vacuolar ATPase assembly integral membrane protein vma21	NM_001202424.1
A20G0455	9.10	21.55	EMC7	ER membrane protein complex subunit 7	NM_001025515.2
B13G0079	9.10	22.62	GSTA3	Glutathione S-transferase 3	NM_001030177.1
A6G0295	9.10	8.22	INPP4A	Inositol polyphosphate-4-phosphatase type I A	XM_068221803.1
B11G0380	9.10	7.91			XM_005169307.5
B4G0067	9.10	6.50	DNAJC2	DnaJ homolog subfamily C member 2	NM_212811.1
A25G0599	9.10	7.49	USP6NL	USP6 N-terminal-like protein	XM_021473875.2
B15G0746	9.09	4.63	LOC113114946	A0A6P6QXD9_CARAU FXYD domain-containing ion transport regulator	
A8G0207	9.09	5.16	FXN	Frataxin, mitochondrial	
B14G0510	9.09	4.80	ELP1	Elongator complex protein 1	XM_684442.9
A7G0364	9.09	4.02	TC1A	Transposable element Tc1 transposase	
B22G0255	9.09	29.36	LOC113040258	A0A6P6J2R5_CARAU uncharacterized protein LOC113040258 isoform X3	XM_005161637.5
A3G0843	9.09	9.11	SOCS1	Suppressor of cytokine signaling 1	NM_001003467.1
A10G0359	9.08	3.24	SMYD4	SET and MYND domain-containing protein 4	
A21G0716	9.08	9.70	TAF9	Transcription initiation factor TFIID subunit 9	NM_001044395.2
B23G0648	9.08	48.57	NR4A1	Nuclear receptor subfamily 4 group A member 1	NM_001002173.1

B6G0403	9.08	10.80	DGKE	Diacylglycerol kinase epsilon	NM_001172228.1
B1G0241	9.08	6.17	NANS	Sialic acid synthase	NM_206829.1
B8G0950	9.08	9.60	TMEM230	Transmembrane protein 230	XM_017357267.3
B20G0144	9.08	5.16	INTS7	Integrator complex subunit 7	NM_173267.1
B9G0590	9.07	21.08	ABI3BP	Target of Nesh-SH3	XM_009304780.4
B22G0107	9.07	4.90	IRF6	Interferon regulatory factor 6	NM_200598.2
A14G0719	9.06	7.58	A33	Zinc-binding protein A33	NM_001130662.1
A4G0707	9.06	3.92	PPHLN1	Periphilin-1	NM_001080080.1
B8G0528	9.06	2.60	SEMA3F	Semaphorin-3F	XM_005167031.5
B5G0396	9.06	8.70	PRPF4	U6 small nuclear ribonucleoprotein Prp4	NM_199755.1
B9G0252	9.06	10.37	CCDC93	Coiled-coil domain-containing protein 93	XM_009304844.4
B23G0651	9.06	9.57	RNF41	E3 ubiquitin-protein ligase NRDP1	NM_213516.1
A11G0737	9.06	3.39	LOC107572062	A0A672P6P2_SINGR Uncharacterized LOC107572062	NM_001386812.1
A23G0884	9.05	12.36	SERP1	Stress-associated endoplasmic reticulum protein 1	NM_212943.2
B1G0516	9.05	2.98	KCNQ5	Potassium voltage-gated channel subfamily KQT member 5	XM_691989.10
A19G0139	9.05	1.20	LOC113068217	A0A6P6MJU1_CARAU heat shock factor-binding protein 1-like	
B12G0769	9.05	14.38	SPAG9	C-Jun-amino-terminal kinase-interacting protein 4	XM_068224849.1
B1G0793	9.05	10.64	ITSN1	Intersectin-1	XM_005170972.5
A3G0505	9.05	6.38	PDAP1	28 kDa heat- and acid-stable phosphoprotein	NM_200210.1
A7G0639	9.05	29.78	MMP2	72 kDa type IV collagenase	NM_198067.1
B24G0272	9.05	5.82	TLCD4-B	TLC domain-containing protein 4-B	XM_005162852.5
B1G1116	9.04	5.06	GIMAP2	GTPase IMAP family member 2	XM_068220973.1
B14G0221	9.04	7.85			XM_021472837.2
B7G1266	9.04	10.91	HNRNPC	C2	NM_200993.1
B16G0459	9.04	8.35	RBP1	Retinol-binding protein 1	NM_199528.3
A4G1045	9.04	6.12	PRR5	Proline-rich protein 5	XM_003200642.6
B17G0571	9.04	18.27	YY1	Transcriptional repressor protein YY1	NM_212617.2
A12G0159	9.04	10.45	MAP2K6	Dual specificity mitogen-activated protein kinase kinase 6	NM_001312870.1
A1G0301	9.04	4.27	DPY30	Protein dpy-30 homolog	NM_001002438.2
B5G0343	9.04	7.12	ZMYND19	Zinc finger MYND domain-containing protein 19	NM_183069.1
B2G1234	9.04	14.35	PRKACB	cAMP-dependent protein kinase catalytic subunit beta	NM_001034976.1
B10G0208	9.04	4.33	FAM136A	Protein FAM136A	NM_213400.1
A1G0327	9.04	10.13	PPM1K	Protein phosphatase 1K, mitochondrial	NM_001327744.1
B5G0766	9.04	11.00	A0A672PKP4	A0A672PKP4_SINGR RBR-type E3 ubiquitin transferase	
A21G0284	9.04	14.86	SMARCB1A	SNF-related matrix-associated actin-dependent regulator of chromatin subfamily B member 1-A	XM_005161146.4
B21G0514	9.04	6.29	NEU3	Sialidase-3	NM_001003644.2
A13G0504	9.03	8.17	KHDRBS2	KH domain-containing, RNA-binding, signal transduction-associated protein 2	XM_021480668.2

B11G0515	9.03	5.21	LOC107737257	A0A673LFI3_9TELE Uncharacterized LOC107737257	NM_214705.1
A2G0686	9.03	5.22	KLF4	Krueppel-like factor 4	NM_131723.1
A7G0907	9.03	9.04	SLC7A8	Large neutral amino acids transporter small subunit 2	NM_001271897.1
A9G0137	9.03	9.50	NCKAP1	Nck-associated protein 1	XM_005167921.5
A16G0187	9.03	4.12	CH076	Uncharacterized protein C8orf76 homolog	NM_001007425.2
B20G0730	9.03	7.81	GOPC	Golgi-associated PDZ and coiled-coil motif-containing protein	NM_200557.1
A3G0944	9.02	11.05	HIRIP3	HIRA-interacting protein 3	NM_199823.1
A12G0271	9.02	7.73	NHLRC2	NHL repeat-containing protein 2	XM_009306763.4
A3G0557	9.02	8.28	GOSR2	Golgi SNAP receptor complex member 2	NM_199688.1
B8G0251	9.02	9.02	RFK	Riboflavin kinase	NM_001328083.1
B7G1314	9.02	16.31	DRAP1	Dr1-associated corepressor	
B4G0082	9.02	3.46	KRR1	KRR1 small subunit processome component homolog	NM_001045325.2
A6G0780	9.02	8.11	TRNT1	CCA tRNA nucleotidyltransferase 1, mitochondrial	NM_001002159.2
B5G0289	9.02	8.57	MATR3	Matrin-3	NM_001077760.2
B9G0232	9.02	6.80	IKZF2	Zinc finger protein Helios	XM_017357617.3
A19G0292	9.02	4.02			XM_068215142.1
A8G0563	9.01	21.59	RAP1B	Ras-related protein Rap-1b	NM_001002152.1
A6G0969	9.01	10.44	CTSA	Lysosomal protective protein	NM_200550.1
A18G0170	9.01	6.67	LYSMD2	LysM and putative peptidoglycan-binding domain-containing protein 2	NM_001003507.1
A6G0837	9.01	7.12	MOV10B.1	Putative helicase mov-10-B.1	XM_002662530.5
B6G0920	9.01	11.30	OSER1	Oxidative stress-responsive serine-rich protein 1	NM_200357.1
A23G0064	9.01	9.09	MAFB	Transcription factor MafB	NM_131015.3
B7G0966	9.01	6.58	MESD	LRP chaperone MESD	XM_005166364.5
B14G0306	9.01	3.83	MRPL18	39S ribosomal protein L18, mitochondrial	XM_005157321.4
B2G0032	9.01	15.12	NCSTN	Nicastrin	NM_001009556.1
B7G1081	9.01	3.00	NECTIN4	Nectin-4	XM_683919.9
A3G0115	9.01	9.72	PICK1	PRKCA-binding protein	NM_001077336.1
A19G0247	9.01	5.83	PPP1R10	threonine-protein phosphatase 1 regulatory subunit 10	NM_001277109.1
A19G0867	9.00	3.29	TWIST2	Twist-related protein 2	NM_130984.2
A20G0240	9.00	9.74	HSF2	Heat shock factor protein 2	NM_131867.1
B15G0874	9.00	8.53	PDGFD	Platelet-derived growth factor D	XM_003200098.6
B23G0702	9.00	7.07	TMEM18	Transmembrane protein 18	NM_001005601.2
B3G0319	9.00	9.49	TRAP1	Heat shock protein 75 kDa, mitochondrial	NM_001113625.1
B7G1179	9.00	7.34	TMEM256	Transmembrane protein 256	NM_001017620.1
A9G0252	8.99	13.41	GABPA	GA-binding protein alpha chain	NM_001328412.1
B16G1147	8.99	7.38	SOX4	Transcription factor SOX-4	NM_200901.1
B9G0786	8.99	3.71	CD101	Immunoglobulin superfamily member 2	NM_001002687.1
A1G1023	8.99	4.91	METTL14	N6-adenosine-methyltransferase non-catalytic subunit	NM_207071.1
A23G0505	8.99	9.22	DIP2BA	Disco-interacting protein 2 homolog B-A	XM_009296841.4
B17G0039	8.99	16.80	NLRC3	Protein NLRC3	XM_068222163.1
B3G1379	8.98	8.56	PRPSAP1	Phosphoribosyl pyrophosphate synthase-associated protein 1	NM_001002199.1

A8G0652	8.98	10.71	GAPVD1	GTPase-activating protein and VPS9 domain-containing protein 1	XM_009304094.4
B10G0017	8.98	7.50	PHAX	Phosphorylated adapter RNA export protein	NM_001003995.1
A21G0903	8.98	6.05	ACSL6	Long-chain-fatty-acid--CoA ligase 6	XM_068215943.1
A1G0597	8.98	2.79	PNPLA4	Patatin-like phospholipase domain-containing protein 4	NM_001089482.1
A17G0157	8.98	8.21	AHSA1	Activator of 90 kDa heat shock protein ATPase homolog 1	NM_212602.1
B19G0232	8.98	5.03	CKS1B	Cyclin-dependent kinases regulatory subunit 1	NM_001003593.3
B3G0677	8.98	7.25	GNA13	Guanine nucleotide-binding protein subunit alpha-13	NM_001013263.2
A20G0904	8.97	17.82	EPB41L2	Band 4.1-like protein 2	XM_021468582.2
B21G0079	8.97	4.52	ATG4A	Cysteine protease ATG4A	NM_001024434.1
A7G0402	8.97	5.94	HDAC8	Histone deacetylase 8	NM_213431.1
A21G0845	8.97	2.38	SOWAHA	Ankyrin repeat domain-containing protein SOWAHA	
B9G0518	8.97	5.02	RUBCNL	Protein associated with UVRAG as autophagy enhancer	XM_009304706.3
B15G0934	8.96	9.16	MPZL3	Myelin protein zero-like protein 3	
B12G0484	8.96	10.79	LCMT1	Leucine carboxyl methyltransferase 1	NM_001004645.2
A12G0489	8.96	7.39	LCMT1	Leucine carboxyl methyltransferase 1	NM_001004645.2
A19G0606	8.95	4.86	SH2D7	SH2 domain-containing protein 7	XM_693824.9
A19G0025	8.95	15.57	ATF6B	Cyclic AMP-dependent transcription factor ATF-6 beta	NM_001077720.1
A1G0363	8.95	23.31	AGPAT3	1-acyl-sn-glycerol-3-phosphate acyltransferase gamma	NM_213425.1
A16G1016	8.95	4.09	PPP1R8	Nuclear inhibitor of protein phosphatase 1	NM_201200.1
A3G0160	8.95	6.41	ATG4DA	Cysteine protease atg4da	XM_017354495.3
B23G0409	8.95	16.95	UBR4	E3 ubiquitin-protein ligase UBR4	XM_021473658.2
A10G0855	8.95	38.09	LOC113053987	A0A6P6KU60_CARAU tropomyosin beta chain-like isoform X4	XM_005155492.4
A21G0538	8.94	9.10	ANKHD1	Ankyrin repeat and KH domain-containing protein 1	XM_009295482.4
A22G0475	8.94	4.14	PLPP2	Phospholipid phosphatase 2	NM_199953.2
A21G0905	8.94	15.54	SAP30L	Histone deacetylase complex subunit SAP30L	NM_214703.1
A2G0427	8.94	7.97	NIBAN1	Protein Niban 1	XM_684820.8
B18G0684	8.94	20.32	ARSA	Arylsulfatase A	NM_001013543.2
B10G0843	8.94	9.47	TTC3	E3 ubiquitin-protein ligase TTC3	XM_005172576.5
B14G0543	8.94	14.43	CISD1	CDGSH iron-sulfur domain-containing protein 1	NM_001014305.1
B8G0082	8.93	3.83	ARHGEF16	Rho guanine nucleotide exchange factor 16	XM_005167281.5
B10G0879	8.93	7.67	NUP155	Nuclear pore complex protein Nup155	NM_200156.1
A24G0304	8.93	9.07	COPS9	COP9 signalosome complex subunit 9	
B20G0480	8.93	10.02	FNTB	Protein farnesyltransferase subunit beta	NM_001002128.1
B21G0621	8.93	5.75	WDR5	WD repeat-containing protein 5	NM_213099.1

B14G0160	8.93	3.57	ASAH1	Acid ceramidase	NM_001006088.1
B6G0164	8.92	7.27	PALM3	Paralemmin-3	NM_001080085.1
B9G0198	8.92	4.54	FKBP7	Peptidyl-prolyl cis-trans isomerase FKBP7	NM_001004506.1
B8G0732	8.92	8.42	SSBP3	Single-stranded DNA-binding protein 3	XM_005166809.5
B6G0776	8.92	7.05	MST1R	Macrophage-stimulating protein receptor	XM_068222037.1
B14G0617	8.92	10.24	SEC24B	Protein transport protein Sec24B	XM_001921599.8
A11G0545	8.92	10.47	GPRX	Probable G-protein coupled receptor (Fragment)	XM_005169363.5
B6G0587	8.92	7.60	SLC35D2	GDP-mannose transporter	NM_001003877.2
A15G0526	8.91	13.97	SUPT5H	Transcription elongation factor SPT5	NM_131673.2
A8G0983	8.91	5.57	IL17RE	Interleukin-17 receptor E	
B1G1115	8.91	7.62	GIMAP4	GTPase IMAP family member 4	
B11G0008	8.91	4.51	NCKAP1L	Nck-associated protein 1-like	
A8G0407	8.91	8.50	LRRC47	Leucine-rich repeat-containing protein 47	NM_001110372.1
B13G0130	8.91	10.59	ACTR2B	Actin-related protein 2-B	NM_001082827.2
B9G0864	8.91	13.22	HAT1	Histone acetyltransferase type B catalytic subunit	NM_001004572.2
A16G0795	8.91	4.97	NBEAL2	Neurobeachin-like protein 2	XM_003200162.6
B13G0441	8.90	7.46	G5714_013429	A0A7J6CEL5_9TELE Uncharacterized protein	XM_068224921.1
B3G1382	8.90	4.82	KV3AI	Ig kappa chain V-III region PC 6684	
A6G0237	8.90	13.32	SENP2	Sentrin-specific protease 2	XM_679191.10
B7G0175	8.90	10.54	PARVA	Alpha-parvin	NM_001110471.1
A6G0403	8.89	5.80	RAB11FIP4B	Rab11 family-interacting protein 4B	NM_001128825.1
B13G0799	8.89	9.88	RSRP1	serine-rich protein 1	NM_001077325.1
B18G0538	8.89	7.92	DYE	Nuclear pore complex protein Nup93	XM_009293522.3
B22G1031	8.89	12.98	NLRC3	NLR family CARD domain-containing protein 3	XM_021478497.2
A5G1143	8.89	7.94	NAA38	N-alpha-acetyltransferase 38, NatC auxiliary subunit	NM_001160291.1
B7G0418	8.89	2.26	APIP	Methylthioribulose-1-phosphate dehydratase	NM_001004679.1
A6G0262	8.89	4.06	MCM6	DNA replication licensing factor MCM6	NM_001082849.1
B8G1006	8.89	1.99	BLNK	B-cell linker protein	XM_068223080.1
B21G0802	8.88	2.66	REXO4	RNA exonuclease 4	XM_002665867.6
A10G0373	8.88	0.87	POR	NADPH--cytochrome P450 reductase	XM_005172678.5
A6G0393	8.88	4.67	ANAPC11	Anaphase-promoting complex subunit 11	NM_001098480.1
A8G0708	8.88	11.72	KDM2B	Lysine-specific demethylase 2B	XM_021478234.2
B8G0866	8.88	8.85	SLC66A1	Lysosomal amino acid transporter 1 homolog	NM_001013303.2
A2G0386	8.88	36.57	CRJ1A	Crystallin J1A	NM_001193469.1
A7G0881	8.88	10.58	MARK2	threonine-protein kinase MARK2	XM_068222598.1
B2G0275	8.88	9.20	SDHC	Succinate dehydrogenase cytochrome b560 subunit, mitochondrial	NM_001003523.2
A17G0862	8.88	3.93	PLEKHG3	Pleckstrin homology domain-containing family G member 3	XM_021467265.2

B4G0551	8.88	4.21	NDUFB2	NADH dehydrogenase [ubiquinone] 1 beta subcomplex subunit 2, mitochondrial	
B25G0557	8.88	2.82	RASSF10	Ras association domain-containing protein 10	XM_005174361.5
A2G1047	8.88	6.19	PER2	Period circadian protein homolog 2	XM_021478636.2
A13G0265	8.87	9.47	JAG2	Protein jagged-2	NM_001080606.2
B25G0902	8.87	6.25	PCLAF	PCNA-associated factor	
A11G0735	8.87	8.94	DNAJC14	DnaJ homolog subfamily C member 14	XM_017358379.3
A8G0506	8.86	9.28	WAS	Actin nucleation-promoting factor WAS	XM_694827.9
B6G0771	8.86	16.06	USP4	Ubiquitin carboxyl-terminal hydrolase 4	XM_002662510.7
B14G0404	8.86	8.07	CCDC88C	Protein Daple	NM_001386506.1
B10G0478	8.86	9.09	THAP12	52 kDa repressor of the inhibitor of the protein kinase	XM_002663575.7
B17G0088	8.86	7.20	CDC5L	Cell division cycle 5-like protein	NM_201084.2
B2G0746	8.86	5.04	HECTD3	E3 ubiquitin-protein ligase HECTD3	NM_001077159.2
A3G0793	8.86	5.19	HSD17B14	17-beta-hydroxysteroid dehydrogenase 14	NM_001003521.2
A20G0739	8.86	7.44	PSMC1	26S proteasome regulatory subunit 4	NM_001002091.1
B16G0403	8.86	7.12	RRAGC	Ras-related GTP-binding protein C	NM_001020635.1
B10G0188	8.85	1.55	DHX29	ATP-dependent RNA helicase DHX29	XM_692841.10
A1G0675	8.85	7.55	PSIP1	PC4 and SFRS1-interacting protein	XM_009291548.4
B7G0540	8.85	13.09	MAF1	Repressor of RNA polymerase III transcription MAF1 homolog	NM_001030239.1
B3G0934	8.84	3.40	PRF1	Perforin-1	NM_001317762.1
B6G0415	8.84	15.65	ZNF207	BUB3-interacting and GLEBS motif-containing protein ZNF207	XM_009302342.4
B10G0537	8.84	3.33	EXOSC8	Exosome complex component RRP43	NM_001002865.1
B11G0052	8.83	18.58	FAIM2	Protein lifeguard 2	XM_017358379.3
B7G0350	8.83	5.83	RELL1	RELT-like protein 1	NM_001201360.1
B1G1152	8.83	11.64	G5714_000077	A0A7J6DFB4_9TELE TNF_2 domain-containing protein	
B6G0538	8.83	6.39	TTC4	Tetratricopeptide repeat protein 4	NM_001002122.2
A2G0249	8.83	8.32	WACA	WW domain-containing adapter protein with coiled-coil	NM_001005971.1
A7G0410	8.83	8.13	CTSA	Lysosomal protective protein	NM_001386652.1
B24G0604	8.83	17.75	GIMAP4	GTPase IMAP family member 4	NM_001366026.1
A16G0041	8.83	4.67	NOD1	Nucleotide-binding oligomerization domain-containing protein 1	XM_002665060.7
B18G0568	8.83	20.03	AMPD3	AMP deaminase 3	XM_021467594.2
A15G0786	8.82	3.87	TFDP2	Transcription factor Dp-2	NM_198208.1
A8G0747	8.82	12.51	SF3A1	Splicing factor 3A subunit 1	NM_200094.1
A8G0082	8.82	6.76	GLE1	mRNA export factor GLE1	NM_001003885.2
B7G0229	8.82	11.22	CSK22	Casein kinase II subunit alpha'	NM_131240.1
B19G0363	8.82	22.90	RAB5A	Ras-related protein Rab-5A	NM_201485.1
A12G0216	8.82	17.67	NARF	Nuclear prelamin A recognition factor	NM_001002342.1
B4G0008	8.82	8.70	TMEM181	Transmembrane protein 181	XM_005168514.5
B25G0029	8.81	8.10	SNTB2	Beta-2-syntrophin	XM_002666912.7

B15G0421	8.81	45.46	COX7A2	Cytochrome c oxidase subunit 7A2, mitochondrial	
B8G0842	8.81	5.39	DAB2IP	Disabled homolog 2-interacting protein	XM_017357419.3
B3G0437	8.81	29.32	PMP22	Peripheral myelin protein 22	
B15G0426	8.81	4.01	TNFAIP1	POZ domain-containing adapter for CUL3-mediated RhoA degradation protein 2	NM_199799.1
B7G1082	8.81	5.62	TRMT10A	tRNA methyltransferase 10 homolog A	NM_001386359.1
A25G0769	8.81	5.51	FRS2	Fibroblast growth factor receptor substrate 2	NM_001002658.2
B8G0603	8.81	5.37	YTHDF1	YTH domain-containing family protein 1	NM_212713.1
A15G0499	8.81	3.51	NKAPD1	Uncharacterized protein NKAPD1	NM_001160409.1
A24G0343	8.81	6.83	ARMC1	Armadillo repeat-containing protein 1	NM_001008609.1
A19G0028	8.80	17.59	SLC39A7	Zinc transporter Slc39a7 (Fragment)	NM_130931.3
B8G0413	8.80	9.82	PTGES2	Prostaglandin E synthase 2	NM_200280.2
A10G0841	8.80	4.51	NFIL3	Nuclear factor interleukin-3-regulated protein	NM_001004120.3
A12G0312	8.80	6.01	OGFOD3	2-oxoglutarate and iron-dependent oxygenase domain-containing protein 3	NM_001130772.2
B5G0245	8.80	8.96	EIF2B1	Translation initiation factor eIF-2B subunit alpha	XM_009302036.4
A19G0823	8.80	10.34	BRD2	Bromodomain-containing protein 2	NM_131200.2
B21G0205	8.80	9.43	UIMC1	BRCA1-A complex subunit RAP80	XM_003200891.6
B6G0077	8.79	12.97	PRKAG1	5'-AMP-activated protein kinase subunit gamma-1	NM_213161.1
B17G0315	8.79	7.51	REEP3	Receptor expression-enhancing protein 3	NM_001037575.2
A3G0054	8.79	3.96	G5714_004046	A0A7J6DB61_9TELE Uncharacterized protein	
A13G0587	8.79	2.10	DCTN1	Dynactin subunit 1	NM_001145583.1
A4G0549	8.79	2.50	NAV3	Neuron navigator 3	XM_068219722.1
B24G0590	8.79	5.24	UBA5	Ubiquitin-like modifier-activating enzyme 5	NM_001305617.1
A4G0619	8.78	15.44	PLXNC1	Plexin-C1	XM_068220763.1
A12G0445	8.78	14.27	CBX7	Chromobox protein homolog 7	XM_009306692.4
A20G0730	8.78	2.10	COA7	Cytochrome c oxidase assembly factor 7	
A13G0542	8.78	10.26	RAP1GDS1-B	Rap1 GTPase-GDP dissociation stimulator 1-B	NM_212659.1
A18G0564	8.78	6.63	TMEM263	Transmembrane protein 263	
B17G0597	8.78	2.79	EEF1AKMT2	EEF1A lysine methyltransferase 2	NM_001013327.1
A17G0386	8.78	3.55	ASAP2	Arf-GAP with SH3 domain, ANK repeat and PH domain-containing protein 2	NM_001365098.1
B20G0362	8.77	4.67	OCIAD1	OCIA domain-containing protein 1	NM_200302.2
B5G1266	8.77	16.59	ACACA	Acetyl-CoA carboxylase 1	XM_021476434.2
B9G0827	8.77	9.02	PRKRA	Interferon-inducible double-stranded RNA-dependent protein kinase activator A homolog	XM_005172508.5
B15G0573	8.77	12.43	RILPL1	RILP-like protein 1	XM_001344147.8

A23G0455	8.77	7.37	CASP9	Caspase-9	NM_001007404.2
A22G0144	8.77	5.77	CERS5	Ceramide synthase 5	XM_021469303.2
A7G0291	8.77	23.48	IST1	IST1 homolog	NM_212585.2
A18G0745	8.77	19.12	CCPG1	Cell cycle progression protein 1	NM_001351662.1
A23G0182	8.76	4.62			
B8G0482	8.76	16.32	SLC16A1	Monocarboxylate transporter 1	NM_200085.1
B3G0916	8.76	5.38	CCDC43	Coiled-coil domain-containing protein 43	NM_200658.2
A16G0834	8.76	12.00	SKAP2	Src kinase-associated phosphoprotein 2	XM_005157963.5
B9G0790	8.76	6.56	PTGFRN	Prostaglandin F2 receptor negative regulator	XM_009304396.4
B16G0211	8.76	13.78	OXR1	Oxidation resistance protein 1	XM_021466737.2
A6G0067	8.75	0.03	TGM5	Protein-glutamine gamma-glutamyltransferase 5	NM_001326491.1
B20G0064	8.75	16.61	SNX14	Sorting nexin-14	XM_005160315.3
B18G0169	8.75	10.96	LYSMD2	LysM and putative peptidoglycan-binding domain-containing protein 2	NM_001003507.1
A6G0168	8.75	4.51	CEP20	Centrosomal protein 20	NM_001326495.1
B12G0441	8.75	7.46	RANGAP1	Ran GTPase-activating protein 1	NM_212989.2
A20G0780	8.75	13.28	BPNT1	3'(2'),5'-bisphosphate nucleotidase 1	NM_001002354.1
B22G1172	8.75	6.90	LOC104949296	A0A6I9NHJ8_9TELE uncharacterized protein LOC104949296 isoform X6	
B15G0886	8.74	28.20	LOC113058865	A0A6P6LHE9_CARAU protein IWS1 homolog	XM_005157821.5
A13G0239	8.74	10.64	ERLEC1	Endoplasmic reticulum lectin 1	XM_005156652.4
B23G0395	8.74	25.03	UBA1	Ubiquitin-like modifier-activating enzyme 1	XM_005162052.5
A19G0303	8.74	6.59	RIDA	2-iminopropanoate deaminase	NM_001012315.2
A14G0805	8.74	10.13	SH3BGRL	Adapter SH3BGRL	
B7G1208	8.74	9.81	CXCL8	Interleukin-8	
B20G0432	8.74	5.34	FOXC1B	Forkhead box C1-B	NM_131729.2
B4G0244	8.74	7.75			
A15G0593	8.74	11.20	CNRB	CLPTM1-like membrane protein cnrB	NM_001003647.2
A8G0985	8.73	0.26	ROHU_023758	A0A498MRC3_LABRO Interleukin-17 receptor C-like protein	
B18G0148	8.73	10.82	AKT2	threonine-protein kinase	NM_198146.2
B8G0928	8.73	40.27	ABTB1	POZ domain-containing protein 1	XM_003199051.6
A17G0876	8.73	0.54	ADGRG2	Adhesion G-protein coupled receptor G2	XM_068217752.1
B13G0099	8.73	15.77	FBXO9	F-box only protein 9	NM_199715.1
A12G0765	8.73	23.71	ITGAX	Integrin alpha-X	XM_005156007.5
B2G0866	8.73	12.05	RAB6B	Ras-related protein Rab-6B	NM_001013467.1
A19G0112	8.73	17.50	SLC25A32	carrier	NM_200256.1
A7G1002	8.72	15.92	ACADVL	Very long-chain specific acyl-CoA dehydrogenase, mitochondrial	XM_068222373.1
B3G0212	8.72	8.56	RANGAP1	Ran GTPase-activating protein 1	NM_001076616.1
A2G0931	8.72	8.54	SLC39A6	Zinc transporter ZIP6	NM_001001591.1
A1G0644	8.72	4.35	UBE2A	Ubiquitin-conjugating enzyme E2 A	
B4G0449	8.72	5.34	ITFG2	KICSTOR complex protein ITFG2	NM_001017908.2
A6G0639	8.72	13.16	BIN2	Bridging integrator 2	NM_199796.1
A14G0669	8.72	7.40	SMIM19	Small integral membrane protein 19	NM_001025535.2

B5G1100	8.72	5.04	EMID1	EMI domain-containing protein 1	XM_001337672.9
A17G0911	8.72	13.67	KLHDC2	Kelch domain-containing protein 2	
A9G0490	8.72	9.84	CCNT2	Cyclin-T2	NM_199828.2
A24G0653	8.72	1.72	CUZD1	CUB and zona pellucida-like domain-containing protein 1	
B10G0078	8.72	1.40	RASSF6	Ras association domain-containing protein 6	NM_001045339.1
B21G0032	8.72	4.51	LOC113052776	A0A6P6KL22_CARAU uncharacterized protein LOC113052776 isoform X1	
B22G0973	8.71	5.16	A0A673HQC6	A0A673HQC6_9TELE IG domain-containing protein	XR_011010395.1
B19G0410	8.71	12.61	CMTM7	CKLF-like MARVEL transmembrane domain-containing protein 7	
A3G1164	8.71	6.63	UBALD2	UBA-like domain-containing protein 2	NM_001020768.1
B2G0174	8.71	5.06	SEMA4E	Semaphorin-4E	NM_001083865.1
A14G0476	8.71	15.57	DDX41	Probable ATP-dependent RNA helicase DDX41	XM_017354423.1
B3G0555	8.71	9.07	SNN	Stannin	
A18G0761	8.71	20.39	PEPD	Xaa-Pro dipeptidase	NM_198912.1
B16G0045	8.70	12.78	ETHE1	Persulfide dioxygenase ETHE1, mitochondrial	XM_068213925.1
B1G1170	8.70	8.72	LPAR1-A	Lysophosphatidic acid receptor 1-A	XM_021475649.2
A3G0581	8.70	3.53	TMEM238	Transmembrane protein 238	NM_001386432.1
B6G0642	8.70	4.61	STK35	threonine-protein kinase 35	NM_001008627.1
B10G0332	8.69	3.04	NLE1	Notchless protein homolog 1	NM_001328516.1
B23G0217	8.69	7.33	SULF2	Extracellular sulfatase Sulf-2	XM_017353511.3
A7G1098	8.69	2.97	KV6AB	Ig kappa chain V-VI region NQ2-6.1	NM_001423818.1
A24G0166	8.69	3.33	TMEM14C	Transmembrane protein 14C	NM_001004610.2
A20G0440	8.69	10.40	CPSF3	Cleavage and polyadenylation specificity factor subunit 3	NM_001003836.1
A22G0126	8.69	6.19	MYDGF	Myeloid-derived growth factor	NM_001002480.2
A9G0833	8.69	6.99	CYTIP	Cytohesin-interacting protein	XM_003199175.6
B23G0213	8.69	12.17	SLA2	Src-like-adapter 2	XM_017353591.3
A9G0508	8.69	5.88	RIF1	Telomere-associated protein RIF1	NM_001080806.1
A4G0026	8.69	2.81	G5714_004516	A0A7J6D4W8_9TELE Uncharacterized protein	XM_009300923.3
A24G0331	8.68	4.00	MTMR6	Myotubularin-related protein 6	
B16G0457	8.68	20.98	CHD4	Chromodomain-helicase-DNA-binding protein 4	XM_068218819.1
B15G0711	8.68	10.92	TNF	Tumor necrosis factor	
A4G0591	8.68	5.13	IMPDH1B	Inosine-5'-monophosphate dehydrogenase 1b	XM_005164717.5
A5G0843	8.68	14.21	IQGAP1	Ras GTPase-activating-like protein IQGAP1	XM_005165243.5
A12G0611	8.68	8.99	PELO	Protein pelota homolog	NM_201136.1
B14G0058	8.68	5.29	NUDT22	Uridine diphosphate glucose pyrophosphatase NUDT22	NM_001006069.1
B22G0772	8.68	23.16	FKBP8	Peptidyl-prolyl cis-trans isomerase FKBP8	NM_200884.1
A23G0250	8.68	18.70	HCK	Tyrosine-protein kinase HCK	XM_068216859.1
A22G0734	8.68	5.90	GMPPB	Mannose-1-phosphate guanyltransferase beta	NM_001003491.2
B8G0749	8.68	7.39	MRPS36	Alpha-ketoglutarate dehydrogenase component 4	NM_001113608.1

A1G1090	8.68	5.04	DCTPP1	dCTP pyrophosphatase 1	NM_200771.1
B15G0875	8.68	22.76	BTNL8	Butyrophilin-like protein 8	
A23G0881	8.68	6.43	TSC22D4	TSC22 domain family protein 4	NM_001077734.1
A4G0705	8.68	2.67	PUS7L	Pseudouridylate synthase PUS7L	NM_001044957.2
B12G0421	8.67	14.32	CALCOCO2	Calcium-binding and coiled-coil domain-containing protein 2	NM_001020741.2
B16G0218	8.67	6.43	ATAD2	ATPase family AAA domain-containing protein 2	
A1G0614	8.67	5.83	DLX2B	Homeobox protein Dlx2b	NM_131297.2
A3G0377	8.67	13.27	BRD4	Bromodomain-containing protein 4	NM_001111281.2
B7G0249	8.67	9.66	GALNS	N-acetylgalactosamine-6-sulfatase	NM_001080641.2
B22G0552	8.67	4.16	SLC26A11	Sodium-independent sulfate anion transporter	NM_199767.1
B5G0968	8.67	1.28	POU2AF2	POU class 2 homeobox associating-factor 2	NM_001080025.2
A8G0870	8.67	9.93	KANSL3	KAT8 regulatory NSL complex subunit 3	XM_005167344.5
B9G0259	8.67	10.87	STAM2	Signal transducing adapter molecule 2	NM_001007369.2
B15G0102	8.66	5.69	TFDP2	Transcription factor Dp-2	NM_198208.1
B5G1258	8.66	2.05	TRPV4	Transient receptor potential cation channel subfamily V member 4	XM_005165151.5
B8G0404	8.65	8.17	GADD45G	Growth arrest and DNA damage-inducible protein GADD45 gamma	NM_213226.1
B3G0990	8.65	16.68	ECI1	Enoyl-CoA delta isomerase 1, mitochondrial	NM_001305584.1
A2G0431	8.65	12.08	CSNK1G2	Casein kinase I isoform gamma-2	NM_001024379.1
B6G0396	8.65	4.76	ANAPC11	Anaphase-promoting complex subunit 11	NM_001098480.1
B14G0669	8.64	8.66	STX3	Syntaxin-3	XR_011006086.1
A23G0755	8.64	20.26	MICOS10	MICOS complex subunit MIC10	NM_001142955.1
B21G0003	8.64	6.49	SEC24A	Protein transport protein Sec24A	XM_005170340.5
B13G0239	8.64	4.78	CCAR1	Cell division cycle and apoptosis regulator protein 1	NM_199432.1
B18G0334	8.64	4.88	TBRG1	Transforming growth factor beta regulator 1	XR_011007164.1
B6G0521	8.64	3.87	MRPL47	39S ribosomal protein L47, mitochondrial	
A16G0269	8.64	5.03	PLEKHF2	Pleckstrin homology domain-containing family F member 2	NM_200244.1
A22G0083	8.63	15.21	LAML2	Lamin-L(II)	NM_131002.2
B3G0648	8.63	13.62	FMNL1	Formin-like protein 1	XM_680041.8
A8G0001	8.63	10.33	SPATS2	Spermatogenesis-associated serine-rich protein 2	NM_001077726.2
A15G0119	8.63	57.33	ROHU_010637	A0A498LUS7_LABRO Uncharacterized protein	
B5G0008	8.63	9.96	RNF170	E3 ubiquitin-protein ligase RNF170	NM_214750.1
B8G0006	8.63	16.14	PPHLN1	Periphilin-1	XM_017357563.2
A23G0849	8.63	4.71	SH3D19	SH3 domain-containing protein 19	XR_002456606.2
A7G0917	8.63	21.82	DHRS4	reductase SDR family member 4 (Fragment)	NM_200567.2
A10G0004	8.62	9.77	HLCS	Biotin--protein ligase	XM_068224053.1
B2G0355	8.62	9.44	SLC25A36A	Solute carrier family 25 member 36-A	NM_001002667.1

A7G0122	8.62	12.58	TOLLIP	Toll-interacting protein	NM_207061.2
A9G0038	8.62	1.25	TRAPPC2	Trafficking protein particle complex subunit 2	NM_001320115.1
A11G0444	8.61	8.13	PLEKHA3	Pleckstrin homology domain-containing family A member 3	XM_005169307.5
B23G0031	8.61	25.45	SMC1A	Structural maintenance of chromosomes protein 1A	NM_212810.2
B13G0926	8.61	6.49			NM_205651.1
A22G0704	8.61	1.60	STXA	Stonustoxin subunit alpha	XM_681071.8
B14G0269	8.60	2.72	ZDHHC24	Probable palmitoyltransferase ZDHHC24	NM_001002324.1
A9G0690	8.60	4.06	CD200R1A	Cell surface glycoprotein CD200 receptor 1-A	XM_003199187.6
B16G0300	8.60	4.72	METTL6	tRNA N(3)-methylcytidine methyltransferase METTL6	XM_068214022.1
B11G0804	8.60	1.85			NM_001386664.1
A19G0865	8.60	9.01	SNX13	Sorting nexin-13	XM_021468239.2
A3G0954	8.60	16.10	TMUB2	Transmembrane and ubiquitin-like domain-containing protein 2	NM_001005573.1
A11G0311	8.59	8.17	LOC107590143	A0A672MTY1_SINGR Zinc finger and SCAN domain-containing protein 21-like	XM_009305927.4
B23G0589	8.59	11.24	PIK3CD	Phosphatidylinositol 4,5-bisphosphate 3-kinase catalytic subunit delta isoform	NM_201199.1
B7G0617	8.59	10.26			XM_001922501.8
B8G0531	8.59	9.75	STK38	threonine-protein kinase 38	NM_213456.1
A2G0072	8.59	3.89	SLC22A23	Solute carrier family 22 member 23	NM_001351708.1
A21G0565	8.59	8.62	MARK2	threonine-protein kinase MARK2	XM_009295511.4
B25G0472	8.59	20.25	EDIL3	EGF-like repeat and discoidin I-like domain-containing protein 3	XM_005163016.5
B7G0514	8.58	8.08	NCF2	Neutrophil cytosol factor 2	NM_001110462.1
B5G0627	8.58	8.69	LYSMD3	LysM and putative peptidoglycan-binding domain-containing protein 3	NM_001002104.1
A3G1135	8.58	6.14	TBL3	Transducin beta-like protein 3	NM_001007402.1
B9G0022	8.58	20.49	NLRC3	Protein NLRC3	NM_001423274.1
B21G0177	8.58	8.69	SEPTIN4	Septin-4	NM_001082815.1
A5G0905	8.58	5.12	MAMDC2	MAM domain-containing protein 2	NM_001130397.1
A23G0014	8.58	4.41	SAMHD1	Deoxynucleoside triphosphate triphosphohydrolase SAMHD1	NM_001159933.1
A6G0825	8.58	24.59	CD55	Complement decay-accelerating factor, GPI-anchored	
A24G0643	8.58	0.01	F13A1	Coagulation factor XIII A chain	XM_681557.9
B1G0682	8.58	6.97	ZDHHC5A	Palmitoyltransferase ZDHHC5-A	XM_005160244.5
B15G0723	8.58	12.87	AGFG1	Arf-GAP domain and FG repeat-containing protein 1	XM_021481344.2
B6G0966	8.58	4.47	TEAD3	Transcriptional enhancer factor TEF-5	XM_005166142.5
B5G1028	8.57	7.94	VPS37B	Vacuolar protein sorting-associated protein 37B	
A4G0840	8.57	7.80	LOC113048278	A0A6P6K0Y0_CARAU uncharacterized protein LOC113048278	
A8G0273	8.57	5.20	SMOX	Spermine oxidase	NM_001113603.1
A13G0149	8.57	5.40	HPSE2	Inactive heparanase-2	XM_068212993.1
A8G0457	8.57	83.39	CFP	Properdin	
A11G0401	8.57	18.07	RBM12	RNA-binding protein 12	NM_212869.1

B2G0626	8.56	5.88	OTULIN	Ubiquitin thioesterase otulin	
B13G0918	8.56	14.37			XM_021480699.2
B8G0797	8.56	6.19	DEF6	Differentially expressed in FDCP 6 homolog	XM_017357301.3
B17G0742	8.56	5.89	PELI2	E3 ubiquitin-protein ligase pellino homolog 2	NM_001002616.1
A23G0597	8.56	32.15	SGK1	threonine-protein kinase Sgk1	XM_005162299.5
B7G0328	8.56	10.26	NFIB	Nuclear factor 1 B-type	
B3G1165	8.55	9.48	MPST	3-mercaptopyruvate sulfurtransferase	NM_001089346.1
A16G0177	8.55	7.33	TMEM147	Transmembrane protein 147	NM_001002484.1
B3G1356	8.55	4.76	RAB37	Ras-related protein Rab-37	NM_001017715.1
A15G0375	8.55	4.03	GIT1	ARF GTPase-activating protein GIT1	XM_692294.9
A10G0541	8.55	2.42	TIMM10B	Mitochondrial import inner membrane translocase subunit Tim10 B	NM_001017829.1
B1G0921	8.55	5.26	B3GNT2	N-acetyllactosaminide beta-1,3-N-acetylglucosaminyltransferase 2	NM_212840.1
B9G0825	8.55	5.73	CASP8	Caspase-8	
A17G0916	8.54	3.88	POLE2	DNA polymerase epsilon subunit 2	
A8G0928	8.54	5.07	ARHGEF16	Rho guanine nucleotide exchange factor 16	XM_005167281.5
A20G0395	8.54	6.71	OSTM1	Osteopetrosis-associated transmembrane protein 1	NM_001386310.1
A11G0629	8.54	3.54	LOC113110887	A0A6P6QCH4_CARAU prothymosin alpha-like isoform X2	
B21G0866	8.54	4.34	TOR1A	Torsin-1A	
B17G0243	8.54	11.90	PTP4A2	Protein tyrosine phosphatase type IVA 2	NM_001005583.2
A19G0120	8.54	3.52	G5714_018334	A0A7J6BYR2_9TELE Uncharacterized protein	
A16G0369	8.54	59.52	PNRC2	Proline-rich nuclear receptor coactivator 2	NM_001130192.1
B9G0227	8.54	6.21	HIBCH	3-hydroxyisobutyryl-CoA hydrolase, mitochondrial	NM_001014316.2
B8G0902	8.53	12.84			
B5G0744	8.53	4.30	PAQR3	Progestin and adipoQ receptor family member 3	NM_200710.2
B10G0443	8.53	27.19	PICALM	Phosphatidylinositol-binding clathrin assembly protein	XM_009305372.4
A14G0212	8.52	3.29	VGLL1	Transcription cofactor vestigial-like protein 1	NM_001386249.1
A20G0581	8.52	9.04	FRYL	Protein furry homolog-like	NM_001159667.1
A9G0403	8.52	15.04			
A9G0299	8.52	17.58	USP9X	Probable ubiquitin carboxyl-terminal hydrolase FAF-X	XM_005167736.5
A19G0532	8.52	9.29	KANSL1	KAT8 regulatory NSL complex subunit 1	XM_009294186.4
B5G0459	8.52	17.89			XM_005168624.5
B19G0478	8.52	1.75	CGAS	Cyclic GMP-AMP synthase	XM_003200639.6
B18G0655	8.52	2.92	TSPAN9	Tetraspanin-9	XM_009293473.4
A2G0668	8.52	11.03	TMUB1	Transmembrane and ubiquitin-like domain-containing protein 1	NM_213298.2
A12G0258	8.51	5.63	ENTPD1	Ectonucleoside triphosphate diphosphohydrolase 1	XM_005156326.5
B8G0453	8.51	6.43	LOC113107551	A0A6P6PYS2_CARAU uncharacterized protein LOC113107551	

B5G0755	8.51	3.66	DGAT2	Diacylglycerol O-acyltransferase 2	NM_001008626.4
A10G0856	8.51	6.34	APH1B	Gamma-secretase subunit Aph-1b	NM_001030129.2
B7G0238	8.51	4.86	DHODH	Dihydroorotate dehydrogenase (quinone), mitochondrial	NM_001008608.1
A17G0361	8.51	12.47	SCFD1	Sec1 family domain-containing protein 1	NM_182861.2
B17G0453	8.51	6.55	GJB4	Gap junction beta-4 protein	NM_001130636.1
B16G0950	8.50	3.26	THEM4	Acyl-coenzyme A thioesterase THEM4	NM_001080634.2
B8G0212	8.50	16.10	ADAM9	Disintegrin and metalloproteinase domain-containing protein 9	NM_001004678.1
B14G0493	8.50	11.84	POLR2B	DNA-directed RNA polymerase II subunit RPB2	NM_001024461.3
A21G0275	8.50	3.26	PUS1	Pseudouridylate synthase 1 homolog	NM_001008603.2
B3G0685	8.50	15.37	CSNK1D	Casein kinase I isoform delta	NM_199583.1
B18G0536	8.50	10.33	HERPUD1	Homocysteine-responsive endoplasmic reticulum-resident ubiquitin-like domain member 1 protein	XM_005159120.5
B13G0201	8.50	17.92	LBH	Protein LBH	XM_009307462.4
B16G1055	8.50	8.72	ATG5	Autophagy protein 5	NM_001009914.2
A8G0637	8.50	6.03	IPO11	Importin-11	XM_005167156.5
A16G0827	8.50	3.94	JAZF1	Juxtaposed with another zinc finger protein 1	NM_001034992.2
B2G0696	8.49	4.03	CNPY1	Protein canopy-1	NM_001039497.2
A5G0381	8.49	10.26	RPS6KA3	Ribosomal protein S6 kinase alpha-3	XM_005165118.5
A16G0399	8.49	10.00	CNOT3	CCR4-NOT transcription complex subunit 3	XM_005158349.5
A22G0276	8.49	7.60	MMADHC	Cobalamin trafficking protein CblD	NM_001002453.1
B11G0420	8.49	6.32	KDM5B	Lysine-specific demethylase 5B	NM_001328559.1
A3G0284	8.49	8.14	LOC113041938	A0A6P6J7K4_CARAU uncharacterized protein LOC113041938	
B1G1162	8.49	4.67	UBL5	Ubiquitin-like protein 5	NM_201141.1
A19G0430	8.49	21.11	CLASP2	CLIP-associating protein 2	XM_068215070.1
A7G0645	8.49	8.20	TPPP3	Tubulin polymerization-promoting protein family member 3	NM_201335.2
A2G0477	8.49	11.67	DNAJC1	DnaJ homolog subfamily C member 1	NM_001077535.1
A25G0410	8.49	6.53	KXD1	KxDL motif-containing protein 1	NM_001003469.2
B23G0985	8.48	6.11	TEC	Tyrosine-protein kinase Tec	NM_001114743.2
B23G0258	8.48	6.61	MAPRE1	EB family member 1	NM_213640.1
B10G0328	8.48	7.36	IQCJ-SCHIP1	IQCJ-SCHIP1 readthrough transcript protein	XM_009305278.4
A4G0732	8.48	9.73	CAND1	Cullin-associated NEDD8-dissociated protein 1	NM_213485.1
A25G0513	8.48	17.23	BNIP2	adenovirus E1B 19 kDa protein-interacting protein 2	XM_009298160.4
B12G0316	8.48	8.92	ARL4D	ADP-ribosylation factor-like protein 4D	NM_199644.2
B21G0883	8.48	12.04	HMGCR	3-hydroxy-3-methylglutaryl-coenzyme A reductase	NM_001014292.3
B25G0064	8.48	1.33	MPHOSPH6	M-phase phosphoprotein 6	

B8G0111	8.47	5.52	DDX51	ATP-dependent RNA helicase DDX51	NM_001003864.1
B5G1166	8.47	34.03	PRPS1	Ribose-phosphate pyrophosphokinase 1	NM_213327.2
A14G0081	8.47	34.66	NDFIP1	NEDD4 family-interacting protein 1	NM_199666.1
B9G0311	8.47	6.01	DCAF6	DDB1- and CUL4-associated factor 6	XM_005167840.5
B21G0138	8.47	5.95	MAP4K2	Mitogen-activated protein kinase kinase kinase kinase 2	XM_005161421.5
B15G0860	8.47	8.43	LAMTOR1	Ragulator complex protein LAMTOR1	NM_001282140.1
B9G0531	8.47	5.36	ARHGAP15	Rho GTPase-activating protein 15	XM_005167522.5
A17G0504	8.46	5.96	ZNF318	Zinc finger protein 318	XM_021473150.1
B7G0852	8.46	7.71	MTMR1	Myotubularin-related protein 1	NM_001020721.1
A5G0824	8.46	46.75	DPX16_22481	A0A3N0YYL7_ANAGA Uncharacterized protein	XR_002457848.2
B21G0326	8.46	5.43	CLCN5	Cl(-) exchange transporter 5	XM_001920748.7
B15G0829	8.46	7.00	FARSB	Phenylalanine--tRNA ligase beta subunit	NM_001007768.1
A3G0379	8.46	38.39	STXBP2	Syntaxin-binding protein 2	NM_213289.1
B5G1250	8.46	6.30	GATC	Glutamyl-tRNA(Gln) amidotransferase subunit C, mitochondrial	NM_001301329.1
B23G0963	8.46	5.64	MOGAT2-A	2-acylglycerol O-acyltransferase 2-A	XM_021470057.2
B13G0041	8.46	17.24	VIT	Vitrin	
A3G0681	8.46	6.39	TMEM265	Transmembrane protein 265	XR_002456944.2
A21G0306	8.46	7.47	UBE2G1	Ubiquitin-conjugating enzyme E2 G1	NM_213530.1
B24G0304	8.46	8.04	LOC113042756	A0A6P6JFB8_CARAU eotaxin-like	
A5G0720	8.45	8.50	E1301_TTI017355	A0A5A9P5K0_9TELE Uncharacterized protein	NM_001423207.1
A5G0483	8.45	6.89	VAMP8	Vesicle-associated membrane protein 8	
B7G0231	8.45	5.17	NQO1	NAD(P)H dehydrogenase [quinone] 1	
B15G0038	8.45	5.75	PCSK7	kexin type 7	NM_001320428.1
B7G0138	8.45	9.48	USP10	Ubiquitin carboxyl-terminal hydrolase 10	XM_005169052.5
A16G0175	8.45	1.26	LOC113059251	A0A6P6LFK1_CARAU synapse differentiation-inducing gene protein 1-like	NM_001386350.1
A13G0594	8.44	4.68	SUFU	Suppressor of fused homolog	NM_173273.1
B3G0018	8.44	7.38	A33	Zinc-binding protein A33	NM_001045009.3
B4G0577	8.44	6.38	PRKCQ	Protein kinase C theta type	XM_068219633.1
B13G0687	8.44	13.18			NM_001303262.1
A3G0370	8.44	6.61	GMIP	GEM-interacting protein	XM_689070.9
B7G1127	8.44	15.77	ACAP2	Arf-GAP with coiled-coil, ANK repeat and PH domain-containing protein 2	NM_001080579.1
A25G0252	8.44	19.73	CRY2	Cryptochrome-2	NM_131786.2
B17G0436	8.44	4.12	COMMD9	COMM domain-containing protein 9	
B13G0599	8.44	4.74	NT5C1B	Cytosolic 5'-nucleotidase 1B	XM_001338200.8
A13G0645	8.44	5.99	SFXN5	Sideroflexin-5	NM_001003562.3
A12G0112	8.44	10.80	GLRX3	Glutaredoxin 3	NM_001005950.1
A10G0493	8.44	13.29	FRMD8	FERM domain-containing protein 8	NM_001423780.1

B7G0461	8.44	14.79	TP53INP2	Tumor protein p53-inducible nuclear protein 2	NM_001386724.1
B25G0722	8.44	9.60	PTDSS2	Phosphatidylserine synthase 2	NM_001423812.1
A19G0798	8.43	9.56	WIPF2	WASL-interacting protein family member 2	XM_005173756.5
A10G0802	8.43	4.74	SIGMAR1	Sigma non-opioid intracellular receptor 1	NM_200977.1
A18G0543	8.43	5.30	ITPR2	Inositol 1,4,5-trisphosphate receptor type 2	XM_021467780.2
B6G0358	8.43	8.53	MTMR14	Myotubularin-related protein 14	XM_002662387.7
B19G0200	8.43	2.40	BOP1	Ribosome biogenesis protein bop1	NM_001077735.2
B23G0691	8.43	3.39	CHST11	Carbohydrate sulfotransferase 11	XM_688938.10
B7G1207	8.43	71.17	CXCL8	Interleukin-8	
A15G0237	8.43	1.71	LOC113058621	A0A6P6LCU2_CARAU myosin-2 heavy chain-like	XM_688904.10
A20G0058	8.43	5.37	BPHL	Valacyclovir hydrolase	NM_001281461.1
A17G0680	8.42	15.02	KHK	Ketohexokinase	XM_021467144.2
B13G0724	8.42	5.68	ADGRB3	Adhesion G protein-coupled receptor B3	NM_001006081.1
B3G0175	8.42	8.00	H2-K1	H-2 class I histocompatibility antigen, K-D alpha chain	NM_001110118.1
B18G0390	8.42	10.86	PSTPIP1	Proline-serine-threonine phosphatase-interacting protein 1	NM_001045216.1
B10G0168	8.42	3.36	ITGA2	Integrin alpha-2	XM_021472220.2
A1G0942	8.42	6.44	CHIC2	Cysteine-rich hydrophobic domain-containing protein 2	NM_201120.2
B23G0504	8.42	1.97	P2RY1	P2Y purinoceptor 1	
B3G0975	8.41	3.03	EEF2KMT	Protein-lysine N-methyltransferase EEF2KMT	NM_001006058.2
B16G0297	8.41	7.89	PLEKHF2	Pleckstrin homology domain-containing family F member 2	NM_200244.1
B19G0760	8.41	11.91	MYLIPA	E3 ubiquitin-protein ligase MYLIP-A	NM_199983.2
B23G0119	8.41	7.19	RABGGTA	Geranylgeranyl transferase type-2 subunit alpha	NM_001076663.2
A1G1132	8.41	1.14	FOXE1	Forkhead box protein E1	XM_005159910.5
A12G0709	8.41	5.89	KAT7	Histone acetyltransferase KAT7	XM_021480026.2
B24G0348	8.41	1.28	CCL20	C-C motif chemokine 20	
A19G0503	8.41	2.69	MED30	Mediator of RNA polymerase II transcription subunit 30	NM_001033754.1
A15G0175	8.40	9.27	MAG	Myelin-associated glycoprotein	
B3G0674	8.40	7.25	SMIM22	A0A673IES3_9TELE Small integral membrane protein 22	NM_001271773.2
A10G0216	8.40	4.64	BRF2	Transcription factor IIIB 50 kDa subunit	
A1G0184	8.40	13.32	AFTPH	Aftiphilin	NM_001145561.1
B8G0583	8.40	34.86	NT5DC2	5'-nucleotidase domain-containing protein 2	NM_001077143.2
B11G0448	8.40	14.05	CCDC120	Coiled-coil domain-containing protein 120	NM_200414.1
B2G0398	8.40	2.07	APOL6	Apolipoprotein L6	XM_021480586.2
B6G0976	8.40	3.87	MANBAL	Protein MANBAL	NM_001303006.1
B7G1139	8.40	4.65	CNPY4	Protein canopy 4	NM_001039513.1
B3G1158	8.40	9.78	KPNA2	Importin subunit alpha-1	NM_001002335.1
B3G1276	8.40	5.97	SMARCA4	Transcription activator BRG1	NM_181603.1
A6G0578	8.39	8.25	PRPF38A	Pre-mRNA-splicing factor 38A	NM_001003483.2
B17G0831	8.39	10.83	MTHFD1	C-1-tetrahydrofolate synthase, cytoplasmic	NM_199529.1

B20G0756	8.39	42.14	LAPTM4A	Lysosomal-associated transmembrane protein 4A	NM_214685.2
A18G0056	8.39	6.98	RBM7	RNA-binding protein 7	NM_199925.1
A16G0969	8.39	7.43	ATG5	Autophagy protein 5	NM_001009914.2
A25G0308	8.39	10.26	JHDM1DB	Lysine-specific demethylase 7B	XM_005174322.4
B13G0869	8.39	11.61	BLNK	B-cell linker protein	
B12G0181	8.38	5.83	SLC38A10	Putative sodium-coupled neutral amino acid transporter 10	XM_017358553.3
B16G0609	8.38	23.86	DDX6	Probable ATP-dependent RNA helicase ddx6	XM_001340824.8
B3G0668	8.38	6.85	RRN3	RNA polymerase I-specific transcription initiation factor RRN3	XM_701870.7
B3G0177	8.38	6.54	KDELR3	ER lumen protein-retaining receptor 3	NM_200640.2
B11G0183	8.38	0.01	NDUFA11	NADH dehydrogenase [ubiquinone] 1 alpha subcomplex subunit 11	NM_001002392.1
A14G0439	8.38	5.52	SIMC1	SUMO-interacting motif-containing protein 1	XM_001922548.6
A24G0178	8.38	6.63	MCM4-A	DNA replication licensing factor mcm4-A	NM_198913.2
B6G0052	8.38	4.49	GZMK	Granzyme K	XM_001335040.7
A14G0121	8.38	9.41	FAT2	Protocadherin Fat 2	XM_001920023.8
B22G1146	8.38	28.48	TCB2	Transposable element Tcb2 transposase	XM_068220572.1
A23G0448	8.37	16.26	SDF4	45 kDa calcium-binding protein	NM_213087.2
B3G1096	8.37	9.18	ATXN2L	Ataxin-2-like protein	NM_212684.2
A18G0594	8.37	11.47	CMIP	C-Maf-inducing protein	XM_685295.10
A18G0119	8.37	12.31	DDX6	Probable ATP-dependent RNA helicase ddx6	XM_679831.10
A7G1028	8.37	6.10	CASPA	Caspase a	NM_001109712.1
B20G0305	8.36	12.89	EIF2B4	Translation initiation factor eIF-2B subunit delta	NM_001014361.2
B24G0703	8.36	19.43	ECI2	Enoyl-CoA delta isomerase 2	NM_001002645.3
A14G0330	8.36	8.01	GSR	Glutathione reductase, mitochondrial	XM_021481023.2
A7G0666	8.35	9.73	NR1H3	Oxysterols receptor LXR-alpha	NM_001017545.3
A10G0409	8.35	8.56	SKA2	Spindle and kinetochore-associated protein 2	NM_001199675.1
A22G0482	8.35	2.27	GNAQ	Guanine nucleotide-binding protein G(q) subunit alpha	NM_001003626.2
B6G0733	8.35	13.57	MUSTN1	Musculoskeletal embryonic nuclear protein 1	
B5G0530	8.35	7.03	PPIL3	Peptidyl-prolyl cis-trans isomerase-like 3	NM_001005597.1
A1G1045	8.35	4.40	ZCCHC7	Zinc finger CCHC domain-containing protein 7	NM_001301358.3
B10G0547	8.35	9.10	TMEM120A	Ion channel TACAN	NM_001082983.1
A17G0718	8.34	13.17	SEC23A	Protein transport protein Sec23A	NM_213465.1
B4G0559	8.34	16.49	MDM2	E3 ubiquitin-protein ligase Mdm2	NM_131364.3
A3G0704	8.34	8.49	RCN1	Reticulocalbin-1	NM_001002158.1
B18G0753	8.34	7.86	GOLIM4	Golgi integral membrane protein 4	NM_001098192.1
A5G0390	8.34	4.06	MRPS31	28S ribosomal protein S31, mitochondrial	NM_001128328.1
B14G0179	8.34	10.51	YIF1A	Protein YIF1A	NM_199931.1
A22G0254	8.34	1.67	SLC26A11	Sodium-independent sulfate anion transporter	
B9G0768	8.34	15.84	RGCC	Regulator of cell cycle RGCC	

A19G0483	8.33	10.10	NUP153	Nuclear pore complex protein Nup153	NM_001326384.1
B11G0321	8.33	20.59	FGD5	FYVE, RhoGEF and PH domain-containing protein 5	XM_017358331.3
A18G0384	8.33	5.60	SLCO3A1	Solute carrier organic anion transporter family member 3A1	NM_001045188.2
B23G0881	8.33	7.28	OTUD4	OTU domain-containing protein 4	XM_005174151.5
A21G0525	8.33	7.77	NF2	Merlin (Fragment)	NM_212951.1
B2G1319	8.32	9.63	EFCAB14	EF-hand calcium-binding domain-containing protein 14	NM_207075.1
B24G0158	8.32	4.44	LOC113042132	A0A6P6J827_CARAU uncharacterized protein LOC113042132	NM_001013465.1
A22G0489	8.32	6.63	SCAMP4	Secretory carrier-associated membrane protein 4	NM_001025525.1
A4G0241	8.32	1.11	DPX16_4609	A0A3N0YC08_ANAGA Uncharacterized protein	
A11G0014	8.32	9.10	ANKRD13C	Ankyrin repeat domain-containing protein 13C	NM_001040370.1
B18G0451	8.32	4.20	TSNAXIP1	Translin-associated factor X-interacting protein 1	NM_001100100.1
A10G0690	8.32	4.22	H2A	Histone H2A	
A15G0586	8.32	10.36	ITGAE	Integrin alpha-E	XM_021474339.2
A12G0053	8.32	12.53	PALD1	Paladin	NM_198811.1
B7G0251	8.32	2.20	CDT1	DNA replication factor Cdt1	XM_690072.9
A12G0296	8.31	10.90	RNF40	E3 ubiquitin-protein ligase BRE1B	NM_001423835.1
B25G0612	8.31	4.33	OLFM4	Olfactomedin-4	XM_017354130.3
A12G0517	8.31	7.53	CCZ1	Vacuolar fusion protein CCZ1 homolog	XM_017358438.3
A2G0059	8.31	11.43	SS18	Protein SSXT	NM_199744.2
B9G0707	8.31	6.85	GPR89	Golgi pH regulator	XM_002663314.6
B11G0261	8.30	7.85	TRAK1	Trafficking kinesin-binding protein 1	XM_009300579.3
B7G0905	8.30	5.84	GMPR2	GMP reductase 2	NM_001040304.2
B6G0009	8.30	5.80	AP1M2	AP-1 complex subunit mu-2	NM_205714.1
B6G0914	8.30	15.99	E1301_TTI004914	A0A5A9NWN3_9TELE Uncharacterized protein	
A8G0146	8.30	6.37	LOC113081342	A0A6P6NJQ8_CARAU uncharacterized protein LOC113081342	
A11G0010	8.30	8.23	SRSF7	arginine-rich splicing factor 7	XM_068220821.1
A21G0841	8.29	4.11	ZGC:63572	Transmembrane protein 185-like	NM_200437.2
B17G0572	8.29	2.89	DEGS2	C4-monooxygenase DES2	NM_001163366.1
B19G0434	8.29	3.00	MED30	Mediator of RNA polymerase II transcription subunit 30	NM_001033754.1
B1G0005	8.29	6.73	EED	Polycomb protein eed	NM_001017766.2
B21G0474	8.29	10.62	ZW10	kinetochore protein zw10 homolog	NM_200141.2
B3G0620	8.29	6.91	PRPSAP2	Phosphoribosyl pyrophosphate synthase-associated protein 2	NM_213496.1
A1G0961	8.29	12.25	VPS37A	Vacuolar protein sorting-associated protein 37A	NM_199990.1
B18G0660	8.29	2.16	RBM28	RNA-binding protein 28	NM_200321.1
A6G0909	8.29	12.77	OSER1	Oxidative stress-responsive serine-rich protein 1	NM_200357.1
B15G0292	8.29	4.41	TCAF	TRPM8 channel-associated factor homolog	XM_021481341.2
A10G0644	8.29	23.73	ATP6V1B	V-type proton ATPase subunit B	NM_182879.2

A2G0863	8.28	6.32	C14ORF119	Uncharacterized protein C14orf119	NM_001244794.1
A15G0260	8.28	13.14	TMEM106B	Transmembrane protein 106B	NM_001045052.1
B17G0153	8.28	5.40	KRTCAP3	Keratinocyte-associated protein 3	XM_068214526.1
A7G0771	8.28	16.58	HERC1	Probable E3 ubiquitin-protein ligase HERC1	XM_009303242.3
B13G0582	8.28	10.94	PPM1A	Protein phosphatase 1A	NM_001017804.1
B11G0113	8.27	8.84	TMEM38A	Trimeric intracellular cation channel type A	XM_694917.8
A14G0528	8.27	7.73	TRAPPC11	Trafficking protein particle complex subunit 11	NM_199626.2
B14G0142	8.27	10.13	PSMD10	26S proteasome non-ATPase regulatory subunit 10	NM_205754.1
B16G0266	8.27	5.72	MTDH	Protein LYRIC	XM_021466990.2
A12G0136	8.27	7.57	RTASE	Probable RNA-directed DNA polymerase from transposon BS	
A21G0493	8.27	1.43	G5714_020566	A0A7J6BU60_9TELE IF rod domain-containing protein	
B4G0317	8.27	3.63	MRPS33	28S ribosomal protein S33, mitochondrial	NM_001002306.2
A8G0505	8.27	5.32	SLC38A3	Sodium-coupled neutral amino acid transporter 3	XM_001344713.6
B18G0677	8.27	6.58	FRMD4A	FERM domain-containing protein 4A	XM_009293444.4
B20G0388	8.26	29.46	CCN2	CCN family member 2	NM_001015041.2
A24G0232	8.26	9.28	FAM168B	Myelin-associated neurite-outgrowth inhibitor	NM_001076569.1
B5G1210	8.26	2.62	BTNL2	Butyrophilin-like protein 2	
A5G1247	8.26	3.28	FAM78A	Protein FAM78A	NM_001114475.2
A18G0503	8.26	11.56	HERPUD1	Homocysteine-responsive endoplasmic reticulum-resident ubiquitin-like domain member 1 protein	XM_005159120.5
A21G0378	8.26	3.61	FTSJ1	guanosine(34)-2'-O)-methyltransferase	NM_001007384.2
A23G0732	8.26	7.33	MDP1	Magnesium-dependent phosphatase 1	NM_001386482.1
A9G0728	8.26	0.35	SCEL	Sciellin	XM_021478625.2
A24G0550	8.26	8.45	PRPF4B	threonine-protein kinase PRP4 homolog	XM_068217049.1
B12G0033	8.26	11.04	UROS	Uroporphyrinogen-III synthase	NM_212828.2
B16G0304	8.26	4.04	TRMT11	tRNA (guanine(10)-N2)-methyltransferase homolog	NM_200216.3
B14G0664	8.26	2.70	RAD9A	Cell cycle checkpoint control protein RAD9A	NM_200207.2
B3G0642	8.26	21.42	GPATCH8	G patch domain-containing protein 8	XM_021471027.2
A25G0804	8.26	3.78	GIMAP8	GTPase IMAP family member 8	
A10G0100	8.26	7.28	PITPNB	Phosphatidylinositol transfer protein beta isoform	NM_001328435.1
B7G0718	8.26	20.73	SORD	Sorbitol dehydrogenase	NM_001172419.2
B20G0488	8.25	38.30	THBS1	Thrombospondin-1	XM_005160762.5
B3G0609	8.25	2.88	GFER	FAD-linked sulfhydryl oxidase ALR	NM_001089386.1
A2G1128	8.25	5.79	RNF126	E3 ubiquitin-protein ligase RNF126	NM_001083017.2
B5G0215	8.24	6.63			
A12G0414	8.24	7.53	NSF	Vesicle-fusing ATPase	NM_001354163.1
B5G0188	8.24	2.28	VSIG10	V-set and immunoglobulin domain-containing protein 10	

B6G0219	8.24	18.03	SUCLA2	Succinate--CoA ligase [ADP-forming] subunit beta, mitochondrial	NM_213026.1
B9G0519	8.24	8.85	LRCH1	Leucine-rich repeat and calponin homology domain-containing protein 1	XM_021478951.2
A21G0772	8.24	12.26	RNF145	RING finger protein 145	NM_199763.1
B11G0737	8.24	6.71	A33	Zinc-binding protein A33	NM_001020716.1
B13G0670	8.24	8.26	MFSD2AA	Sodium-dependent lysophosphatidylcholine symporter 1-A	NM_199871.1
A21G0350	8.23	6.03	ERI1	3'-5' exoribonuclease 1	NM_001020614.1
B12G0372	8.23	11.65	FBXO11	F-box only protein 11	XM_691042.10
A12G0524	8.23	9.31	ATAD1B	Outer mitochondrial transmembrane helix translocase	NM_001024421.2
B22G0241	8.23	5.89	LOC113086834	A0A6P6NQN5_CARAU CD48 antigen-like	XM_068216396.1
B14G0637	8.23	10.44	DIAPH1	Protein diaphanous homolog 1	XM_021481083.2
A1G1126	8.23	9.79	USO1	General vesicular transport factor p115	NM_200155.1
A3G1089	8.23	4.64	SPC24	Kinetochore protein Spc24	NM_001024440.1
B22G0086	8.22	15.25	ARL8A	ADP-ribosylation factor-like protein 8A	NM_001200020.1
B8G0906	8.22	6.67	CXXC1	CXXC-type zinc finger protein 1	NM_200599.1
B18G0639	8.22	4.68	UQCRFS1	Cytochrome b-c1 complex subunit Rieske, mitochondrial	
B3G0403	8.22	3.62	ASF1BA	Histone chaperone asf1b-A	NM_207063.1
A3G0273	8.22	22.14	GCDH	Glutaryl-CoA dehydrogenase, mitochondrial	NM_001006024.1
B2G0419	8.22	7.86	INSR	Insulin receptor	NM_001142672.1
A13G0581	8.22	10.39	ZBTB18	Zinc finger and BTB domain-containing protein 18	NM_001045346.1
A9G0007	8.22	11.52	NMI	N-myc-interactor	
B5G0761	8.22	10.06	MINK1	Misshapen-like kinase 1	XM_017356005.3
B9G0745	8.22	3.50	WDR4	tRNA (guanine-N(7)-)-methyltransferase non-catalytic subunit wdr4	NM_001126435.2
A14G0145	8.22	30.04	ACSL4	Long-chain-fatty-acid--CoA ligase 4	NM_200649.1
A6G0800	8.21	5.83	RBM19	Probable RNA-binding protein 19	NM_198915.2
B14G0530	8.21	10.37	TRPT1	tRNA 2'-phosphotransferase 1	NM_001013291.3
B23G0035	8.21	5.74	TAFAZZIN	Tafazzin	NM_001001814.1
A10G0116	8.21	10.93	SBNO1	Protein strawberry notch homolog 1	NM_001199916.2
A23G0389	8.21	8.37	ICMT	Protein-S-isoprenylcysteine O-methyltransferase	NM_001017618.1
B18G0443	8.21	5.51	TMEM170A	Transmembrane protein 170A	NM_200689.2
A12G0094	8.21	13.77	LOC113055856	A0A6P6L0F2_CARAU uncharacterized protein LOC113055856 isoform X2	
B2G0654	8.20	4.42	YES1	Tyrosine-protein kinase yes	NM_001013270.3
A21G0021	8.20	8.54	ARSB	Arylsulfatase B	XM_021468934.2
A25G0247	8.20	2.47	USB1	U6 snRNA phosphodiesterase 1	XM_009297898.4
B21G0270	8.20	20.64	NUP98	Nuclear pore complex protein Nup98-Nup96	NM_200685.2
B7G0947	8.19	14.07	PCSK6	kexin type 6	XM_021478115.2
B21G0061	8.19	9.04	TCF7L2	Transcription factor 7-like 2	XM_068215853.1
A9G0797	8.19	2.77	CIR1	Corepressor interacting with RBPJ 1	XM_021478817.2
B24G0466	8.19	9.62	KPNA1	Importin subunit alpha-5	XM_696267.9
B20G0118	8.19	6.18	TMEM242	Transmembrane protein 242	NM_001160014.1

A19G0235	8.19	5.75	SRFBP1	Serum response factor-binding protein 1	NM_213048.3
A20G0086	8.19	13.13	NFKBIE	NF-kappa-B inhibitor epsilon	NM_001080089.1
B24G0164	8.19	7.05	NLRC3	NLR family CARD domain-containing protein 3	XM_009297629.4
B6G0701	8.19	7.61	VHL	von Hippel-Lindau disease tumor suppressor	NM_001080684.1
B16G0848	8.19	10.62	DEPTOR	DEP domain-containing mTOR-interacting protein	XM_005173523.5
B4G0624	8.19	2.13	CELSR1	Cadherin EGF LAG seven-pass G-type receptor 1	XM_009300460.4
A23G0170	8.19	13.03	UCKL1	Uridine-cytidine kinase-like 1	XM_681683.8
A16G1006	8.19	8.65	WDR26	WD repeat-containing protein 26	XM_001921621.8
B6G0745	8.19	4.88	TRUB2	Pseudouridylate synthase TRUB2, mitochondrial	
B8G0287	8.19	4.90	POLD2	DNA polymerase delta subunit 2	NM_213303.1
B2G0014	8.19	15.17	STK11	threonine-protein kinase STK11	NM_001017839.1
B3G1268	8.18	6.32	S1PR1	Sphingosine 1-phosphate receptor 1	NM_001007316.1
B14G0333	8.18	5.05	SIL1	Nucleotide exchange factor SIL1	XM_021481244.2
A17G0302	8.18	8.33	ARHGAP5	Rho GTPase-activating protein 5	XM_001335980.6
B8G0665	8.18	11.75	CSNK1G2	Casein kinase I isoform gamma-2	NM_001045850.2
A13G0452	8.18	7.43	CALHM3	Calcium homeostasis modulator protein 3	NM_201292.1
B13G0012	8.18	9.22	RTASE	Probable RNA-directed DNA polymerase from transposon BS	
A3G0465	8.18	5.48	AXIN1	Axin-1	XM_068217520.1
B19G0651	8.18	25.19	STK3	threonine-protein kinase 3	NM_199672.1
A23G0899	8.18	4.99	TEC	Tyrosine-protein kinase Tec	NM_001114743.2
B2G1213	8.17	9.31	ROCK1	Rho-associated protein kinase 1	XM_021478592.2
B4G0203	8.17	6.74	ELK3	ETS domain-containing protein Elk-3	XM_005164542.5
B15G0113	8.17	36.90	SPCS2	Signal peptidase complex subunit 2	XM_068213543.1
A7G0845	8.17	5.33	VGF	Neurosecretory protein VGF	XM_003198950.6
A10G0618	8.17	7.99	SLK	threonine-protein kinase	XM_021472247.2
A7G1067	8.17	6.14	EHBP1	EH domain-binding protein 1	XM_021477738.2
B6G0073	8.17	3.54	TGM5	Protein-glutamine gamma-glutamyltransferase 5	NM_001004647.2
B11G0423	8.17	17.94	LMOD1	Leiomodin-1	NM_201007.2
B3G1032	8.16	26.95	DNAJC7	DnaJ homolog subfamily C member 7	XM_009299335.4
B3G1230	8.16	7.49	UBE2Z	Ubiquitin-conjugating enzyme E2 Z	NM_001002330.5
B6G0954	8.16	5.13	ILRUN	Protein ILRUN	NM_001013542.2
B14G0432	8.16	12.44	QDPR	Dihydropteridine reductase	NM_001110469.1
B23G0352	8.16	9.39	OARD1	ADP-ribose glycohydrolase OARD1	NM_001020755.1
A11G0315	8.16	8.24	EPYC	Epiphycan	NM_001080008.1
A13G0551	8.16	5.52	CCDC172	Coiled-coil domain-containing protein 172	NM_001004626.1
A10G0143	8.15	17.71	IL1B	Interleukin-1 beta	
B14G0580	8.15	5.17	AP1AR	AP-1 complex-associated regulatory protein	NM_001301369.1
B10G0690	8.15	8.44	DUSP11	RNP complex-1-interacting phosphatase	NM_001328370.1
B20G0102	8.15	11.07	PUM2	Pumilio homolog 2	NM_001102570.2
A17G0492	8.15	17.57	PTEN	Phosphatidylinositol 3,4,5-trisphosphate 3-phosphatase and dual-specificity protein phosphatase PTEN	NM_200708.2

A18G0354	8.15	5.90	RCN2	Reticulocalbin-2	NM_001030263.2
B11G0121	8.15	11.51	DPX16_5304	A0A3N0XU06_ANAGA Uncharacterized protein	NM_001030244.3
B3G0137	8.15	3.42	LOC113090245	A0A6P6NSY2_CARAU gastrula zinc finger protein XlCGF57.1-like	XM_021476699.2
A2G0791	8.15	3.76	ING5	Inhibitor of growth protein 5	XM_005171273.5
A7G1107	8.15	9.64	ZDHHC13	Palmitoyltransferase ZDHHC13	NM_001008650.3
A7G0528	8.15	3.36	HRH3	Histamine H3 receptor	NM_001025518.1
B6G0074	8.15	0.31	RTASE	Probable RNA-directed DNA polymerase from transposon BS	XM_017351203.3
B8G0653	8.15	8.56	TMCO1	Calcium load-activated calcium channel	NM_001002575.1
A5G1019	8.15	15.26	NCF1	Neutrophil cytosolic factor 1	NM_001030071.2
A16G0437	8.14	9.78	MROH1	Maestro heat-like repeat-containing protein family member 1	XM_068214158.1
A20G0049	8.14	9.50	CDC40	Pre-mRNA-processing factor 17	NM_001009990.1
A6G0072	8.14	11.69	MED8	Mediator of RNA polymerase II transcription subunit 8	NM_207083.1
B20G0710	8.14	6.57	SMPDL3A	Acid sphingomyelinase-like phosphodiesterase 3a	XM_005170176.5
B18G0366	8.14	2.25	TMEM123	Porimin	
A23G0190	8.14	3.65	SULF2	Extracellular sulfatase Sulf-2	XM_068216677.1
B13G0780	8.14	20.78	CDH23	Cadherin-23	NM_212608.1
B16G0401	8.14	5.04	UTP11	Probable U3 small nucleolar RNA-associated protein 11	NM_199998.1
B7G0867	8.14	10.42	ARL2	ADP-ribosylation factor-like protein 2	NM_001005947.1
B22G1122	8.14	9.27	GMPPB	Mannose-1-phosphate guanyltransferase beta	NM_001003491.2
B10G0099	8.14	19.05	LOC107558676	A0A672M7P4_SINGR Dentin sialophosphoprotein-like	NM_001002308.1
B12G0138	8.14	4.01	COX10	Protoheme IX farnesyltransferase, mitochondrial	NM_001166259.1
A2G0092	8.14	4.63	MMTAG2	Multiple myeloma tumor-associated protein 2 homolog	XM_005171131.5
A15G0569	8.14	1.76	A2ML1	Alpha-2-macroglobulin-like protein 1	XM_068213702.1
A2G0660	8.14	14.01	MYCB	Transcriptional regulator Myc-B	NM_200172.1
B18G0214	8.13	10.89	IRF8	Interferon regulatory factor 8	NM_001002622.1
B4G0591	8.13	15.33	PARP11	Protein mono-ADP-ribosyltransferase PARP11	NM_001082810.1
B20G0564	8.13	8.63	SMEK1	threonine-protein phosphatase 4 regulatory subunit 3	NM_001044809.1
A7G0246	8.13	4.55	CCND1	S-specific cyclin-D1	NM_131025.4
A3G0939	8.13	14.30	PITPNB	Phosphatidylinositol transfer protein beta isoform	NM_213443.1
A17G0655	8.13	7.25	DAAM1	Disheveled-associated activator of morphogenesis 1	XM_017351772.3
B10G0114	8.13	12.28	PIP5K1B	Phosphatidylinositol 4-phosphate 5-kinase type-1 beta	XM_068223777.1
B20G0146	8.13	7.10	PPP2R5A	threonine-protein phosphatase 2A 56 kDa regulatory subunit alpha isoform	XM_005160442.5
A11G0094	8.13	7.80	A0A671R5R5	A0A671R5R5_9TELE Cystatin domain-containing protein	XM_068224170.1
B13G0789	8.12	15.77	ZFAND3	AN1-type zinc finger protein 3 homolog	XM_068224895.1
A25G0183	8.12	3.71	EHF	ETS homologous factor	NM_001039822.1
A20G0212	8.12	0.78	CENPJ	Centromere protein J	

A22G0058	8.12	6.01	DEF6	Differentially expressed in FDCP 6 homolog	XM_003200920.6
B22G0481	8.12	0.65	CD244	Natural killer cell receptor 2B4	XM_001346243.8
A17G0798	8.12	7.27	TRERF1	Transcriptional-regulating factor 1	XM_009292797.4
B17G0910	8.12	11.56	NEK9	threonine-protein kinase Nek9	XM_021467511.2
A11G0019	8.12	11.76	MYADML2	Myeloid-associated differentiation marker-like protein 2	
B13G0495	8.12	12.12	EEF1A1	Elongation factor 1-alpha 1	XM_068213046.1
A11G0223	8.12	16.93	SLC1A7	Excitatory amino acid transporter 5	NM_199939.1
B10G0113	8.12	4.03	TJP2	Tight junction protein ZO-2	NM_001201571.1
A18G0222	8.12	8.54	TCF25	Transcription factor 25	XM_005159180.5
B13G0607	8.11	6.32	ROCK2	Rho-associated protein kinase 2	NM_001020574.2
A4G0558	8.11	6.26	INTS13	Integrator complex subunit 13	NM_199883.1
A19G0599	8.11	4.28	IGKC	Immunoglobulin kappa constant	
A2G0318	8.11	1.26	RTEL1	Regulator of telomere elongation helicase 1	NM_001013310.2
B2G0734	8.11	30.09	U2SURP	U2 snRNP-associated SURP motif-containing protein	NM_001197074.1
A7G0328	8.11	3.09	LYPLA1	Acyl-protein thioesterase 1	NM_001017616.1
A7G0607	8.11	9.53	CELF1	CUGBP Elav-like family member 1	XM_068222380.1
B15G0558	8.11	7.90	CCDC9	Coiled-coil domain-containing protein 9	NM_001075106.2
B3G0227	8.11	12.16	PKN1	threonine-protein kinase N1	NM_001089460.1
B3G0996	8.11	8.86	HAPSTR1	HUWE1-associated protein modifying stress responses	NM_001328166.1
B7G0479	8.10	22.79	LOC107681511	A0A671LP50_9TELE Serine-aspartate repeat-containing protein I-like	XM_002662871.5
B24G0503	8.10	4.17	TPK1	Thiamin pyrophosphokinase 1	XM_005162615.5
A3G0935	8.10	3.82	LOC113044053	A0A6P6JIE9_CARAU uncharacterized protein LOC113044053	
A8G0589	8.10	33.56	MPEG1	Macrophage-expressed gene 1 protein	NM_212737.1
A15G0168	8.09	9.48	LOC113077812	A0A6P6NCB1_CARAU myeloid cell surface antigen CD33-like	
A19G0786	8.09	11.24	ZFP646	Zinc finger protein 646	XM_009293995.4
A11G0229	8.09	7.31	PSMD14	26S proteasome non-ATPase regulatory subunit 14	XM_005155960.4
B1G0851	8.09	10.14	SPAST	Spastin	NM_212915.2
B3G1094	8.09	8.38	SPNS1	Protein spinster homolog 1	NM_153663.2
A17G0283	8.09	6.40	SNX9	Sorting nexin-9	XM_005158722.4
B5G0398	8.09	2.28	A0A672K763	A0A672K763_SINGR Si:ch211-202f5.3	
A3G1215	8.09	8.24	PYCR1	Pyrroline-5-carboxylate reductase 1, mitochondrial	NM_200826.2
A16G0466	8.08	13.88			
A3G0032	8.08	2.28	G5714_002534	A0A7J6D7N2_9TELE Uncharacterized protein	
A15G0013	8.08	6.77	PSMA6	Proteasome subunit alpha type-6	NM_131795.2
A19G0760	8.08	11.03	MAP7D1	MAP7 domain-containing protein 1	XM_005173746.2
B19G0955	8.08	6.09	SLC39A7	Zinc transporter Slc39a7 (Fragment)	NM_130931.3
B20G0810	8.08	9.12	RMDN3	Regulator of microtubule dynamics protein 3	

B21G0948	8.08	10.42	UHRF2	E3 ubiquitin-protein ligase UHRF2	
B13G0565	8.08	8.50	WDFY4	WD repeat- and FYVE domain-containing protein 4	NM_001076662.1
B17G0817	8.08	10.61	DPX16_18808	A0A3N0Y1U6_ANAGA Cadherin-related family member 5	
A21G0346	8.08	10.00	NNT	NAD(P) transhydrogenase, mitochondrial	NM_214756.1
B10G0784	8.08	8.62	PITPNB	Phosphatidylinositol transfer protein beta isoform	NM_200920.2
B8G0366	8.08	3.30	FZD10-A	Frizzled-10-A	NM_130917.2
B5G1286	8.07	5.04	ASCC2	Activating signal cointegrator 1 complex subunit 2	NM_200442.1
A19G0574	8.07	6.78	TXNL4A	Thioredoxin-like protein 4A	NM_001005953.1
A25G0480	8.07	7.24	WASL	Actin nucleation-promoting factor WASL	NM_001083006.1
A5G1196	8.07	7.89	ZFPL1	Zinc finger protein-like 1	XM_005168620.5
A21G0539	8.07	3.33	DCK2	Deoxycytidine kinase 2	NM_001014352.2
A8G0784	8.07	9.05	UBAP2	Ubiquitin-associated protein 2	XM_005167271.5
B23G0641	8.07	8.00	TRAPPC6B	Trafficking protein particle complex subunit 6b	NM_001006029.1
B22G0877	8.07	5.84	GDE1	Glycerophosphodiester phosphodiesterase 1	NM_200243.1
A17G0329	8.07	16.35	TPP1	Tripeptidyl-peptidase 1	NM_001128798.1
B15G0082	8.06	4.08	MAML2	Mastermind-like protein 2	XM_002667380.7
B25G0138	8.06	1.60	IGKV1D-8	Immunoglobulin kappa variable 1D-8	NM_001045408.1
A3G0268	8.06	12.71	NFIL3	Nuclear factor interleukin-3-regulated protein	NM_001002218.2
B6G1000	8.06	5.68	WDR55	A0A671K7J9_9TELE WD repeat-containing protein 55	
A22G0583	8.06	7.77	XPNPEP1	Xaa-Pro aminopeptidase 1	NM_212980.1
B7G0565	8.06	8.61	DGKZ	Diacylglycerol kinase zeta	XM_021477928.2
B19G0940	8.06	6.90	CRAT	Carnitine O-acetyltransferase	NM_001004594.2
B2G0771	8.06	10.80	NCK1	Cytoplasmic protein NCK1	XM_687914.7
B25G0371	8.06	12.92	CNOT2	CCR4-NOT transcription complex subunit 2	XM_005163109.5
B13G0127	8.05	8.37	ETAA1	Ewing's tumor-associated antigen 1	XM_005169532.5
B16G0391	8.05	7.32	ZDHHC18A	Palmitoyltransferase ZDHHC18-A	NM_001077563.1
B18G0095	8.05	11.08	SRPRA	Signal recognition particle receptor subunit alpha	XM_692426.10
A15G0229	8.05	9.79	ZGC:66022	Mitochondrial fission factor homolog B	XM_005157755.5
B14G0458	8.05	15.52	PPP2CA	threonine-protein phosphatase 2A catalytic subunit alpha isoform	NM_001017886.1
A10G0713	8.05	10.91	PPTC7	Protein phosphatase PTC7 homolog	XM_009305138.4
A6G0621	8.05	2.23	PLCXD1	PI-PLC X domain-containing protein 1	XM_686850.7
B12G0382	8.05	9.64	BAMBI	BMP and activin membrane-bound inhibitor homolog	NM_131784.2
B15G0067	8.05	1.78	CLDN4	Claudin-4	NM_131765.1
A21G0336	8.05	5.81	ABRAXAS1	BRCA1-A complex subunit Abraxas 1	NM_001005993.1
A2G0524	8.05	2.45	PDE4D	cAMP-specific 3',5'-cyclic phosphodiesterase 4D	XM_068218026.1

A14G0586	8.05	9.38	ENPP6	Glycerophosphocholine cholinephosphodiesterase ENPP6	NM_001013527.2
B14G0457	8.05	7.15	CE024	UPF0461 protein C5orf24 homolog	NM_001386379.1
B21G0861	8.05	4.04	SPOUT1	Putative methyltransferase C9orf114 homolog	NM_001004627.1
B3G0903	8.05	10.36	DDX42	ATP-dependent RNA helicase DDX42	NM_001037805.2
B12G0803	8.04	4.47	UBTD1	Ubiquitin domain-containing protein 1	NM_001030265.1
A16G0571	8.04	6.94	ZHX2	Zinc fingers and homeoboxes protein 2	NM_198814.2
A21G0449	8.04	5.81	BUD13	BUD13 homolog	NM_001386240.1
A3G0638	8.04	16.00	ZNF207	BUB3-interacting and GLEBS motif-containing protein ZNF207	NM_213197.1
B4G0528	8.04	6.05	INTS13	Integrator complex subunit 13	NM_199883.1
B2G0399	8.04	7.41	APOLD1	Apolipoprotein L domain-containing protein 1	XM_005171301.4
A6G0048	8.04	3.42	A33	Zinc-binding protein A33	NM_001386812.1
B1G0618	8.04	7.82	AIP	AH receptor-interacting protein	NM_214712.1
B16G0805	8.04	4.42	FOXJ2	Forkhead box protein J2	XM_681709.9
A9G0878	8.04	21.36	MAP4K4	Mitogen-activated protein kinase kinase kinase kinase 4	XM_068223518.1
B3G0824	8.04	9.73	TMEM265	Transmembrane protein 265	XR_002456944.2
B10G0672	8.04	9.23	GPAT4	Glycerol-3-phosphate acyltransferase 4	NM_001040249.2
B16G0429	8.04	3.41	CALHM5.1	Calcium homeostasis modulator protein 5	XM_021466947.2
A24G0630	8.03	20.21	PFKP	ATP-dependent 6-phosphofructokinase, platelet type	XM_068217141.1
B25G0198	8.03	0.50	IGLC3	Ig lambda-3 chain C region	XM_068217596.1
A22G0401	8.03	16.11	MIDN	Midnolin	XM_005170436.5
B22G0773	8.03	4.55	SSBP3	Single-stranded DNA-binding protein 3	NM_001202512.2
A11G0547	8.03	3.96	NFYA	Nuclear transcription factor Y subunit alpha	XM_068224163.1
B16G0361	8.03	14.93	EVA1B	Protein eva-1 homolog B	NM_001089345.1
B16G0566	8.03	10.06	ERP44	Endoplasmic reticulum resident protein 44	NM_200892.1
A16G0001	8.03	5.95	FZD6	Frizzled-6	NM_200561.2
A16G0479	8.03	16.22	MEF2D	Myocyte-specific enhancer factor 2D homolog	NM_131317.1
B9G0485	8.03	9.30	DHRS12	reductase SDR family member 12	NM_001076557.1
A2G0050	8.03	8.67	LAMA3	Laminin subunit alpha-3	XM_690939.9
A12G0804	8.02	6.63	MMS19	MMS19 nucleotide excision repair protein homolog	NM_001347662.1
A6G0206	8.02	3.74	LIMA1	LIM domain and actin-binding protein 1	XM_009302196.4
B17G0202	8.02	4.52	TIMM9	Mitochondrial import inner membrane translocase subunit Tim9	NM_001159911.2
B7G0297	8.02	5.52	FASTKD3	FAST kinase domain-containing protein 3, mitochondrial	XM_001338215.8
B23G0222	8.02	13.30	MRGBP	MORF4L-binding protein	NM_200736.1
A4G0968	8.02	8.16	LOC113048532	A0A6P6K207_CARAU uncharacterized protein LOC113048532 isoform X1	XM_002661331.6

A14G0650	8.02	8.89	SLC35A4	SLC35A4 upstream open reading frame protein	
A12G0747	8.02	10.34	KANSL1	KAT8 regulatory NSL complex subunit 1	XM_005156013.5
A3G0854	8.01	9.46	RHOT1A	Mitochondrial Rho GTPase 1-A	XM_009299427.4
B6G0389	8.01	0.21	X-ELEMENT\ORF2	Probable RNA-directed DNA polymerase from transposon X-element	
A15G0097	8.01	12.59	WDFY1	WD repeat and FYVE domain-containing protein 1	NM_001007057.2
A7G0453	8.01	2.82	RASSF7	Ras association domain-containing protein 7	NM_197942.1
A2G1042	8.01	9.12	GIGYF2	GRB10-interacting GYF protein 2	NM_001030161.2
B1G1153	8.01	0.98	ROHU_028183	A0A498M5U9_LABRO Tumor necrosis factor ligand superfamily member 14-like protein	
B19G0713	8.01	8.88	LDLRAP1-B	Low density lipoprotein receptor adapter protein 1-B	NM_198980.1
B3G0618	8.01	7.19	GRAP	GRB2-related adapter protein	NM_001020573.1
B25G0643	8.01	4.91	KLHDC4	Kelch domain-containing protein 4	NM_001126430.1
A22G0157	8.01	6.65	LOC113077354	A0A6P6NBF4_CARAU uncharacterized protein LOC113077354 isoform X1	XM_068216611.1
A20G0533	8.01	6.08	SLC35F6	Solute carrier family 35 member F6	NM_001002559.1
B15G0675	8.01	2.23	FHDC1	FH2 domain-containing protein 1	XM_689466.6
B7G1068	8.00	16.90	EHBP1L1	EH domain-binding protein 1-like protein 1	XM_021477738.2
A7G0412	8.00	3.46	TMEM208	Transmembrane protein 208	NM_207100.3
B6G0656	8.00	9.46	C1GALT1B	Glycoprotein-N-acetylgalactosamine 3-beta-galactosyltransferase 1-B	NM_200051.1
B7G0914	8.00	2.70	EFS	Embryonal Fyn-associated substrate	NM_001324408.1
B16G0373	8.00	4.07	RAB39A	Ras-related protein Rab-39A	NM_001017683.2
A2G0346	8.00	5.55	GNG12	G(O) subunit gamma-12	NM_213312.2
A11G0638	8.00	3.88	MEGF6	Multiple epidermal growth factor-like domains protein 6	NM_001365489.1
B13G0705	8.00	8.37	PYGL	Glycogen phosphorylase, liver form	XM_005156618.5
A25G0811	8.00	2.94			
B3G0271	8.00	5.52	FOXK2	Forkhead box protein K2	XM_001922821.8
A12G0017	8.00	10.36	ZRANB1	Ubiquitin thioesterase ZRANB1	XM_009306985.4
B8G0440	8.00	13.34	TSPAN36	Tetraspanin-36	NM_001002748.2
A17G0380	7.99	11.15	CATL	Cathepsin L	NM_001002368.1
A24G0017	7.99	6.52	ZBTB14	Zinc finger and BTB domain-containing protein 14	XM_009297676.4
A6G0781	7.99	4.37	ARL6IP5	PRA1 family protein 3	NM_001006096.1
B19G0168	7.99	0.39	COL9A1	Collagen alpha-1(IX) chain	XM_003201680.5
B3G0988	7.99	9.35	NAA60	N-alpha-acetyltransferase 60	NM_001082872.1
A16G0402	7.99	3.70	LENG1	Leukocyte receptor cluster member 1 homolog	NM_001004591.1
A8G0516	7.99	9.80	EFHD2	EF-hand domain-containing protein D2	NM_001326493.1
A1G0081	7.99	12.77	CASPB	Caspase b	
A18G0524	7.99	5.63	ZNF143	Zinc finger protein 143	XM_009293545.4
A1G0286	7.99	1.74			NM_131897.2
B5G0762	7.99	9.28	GNB2	G(T) subunit beta-2	NM_001013497.1

A10G0192	7.99	9.44	MARCHF5	E3 ubiquitin-protein ligase MARCHF5	NM_199739.3
A13G0231	7.98	11.24	MYCL1A	Protein L-Myc-1a	XM_068213021.1
B16G0340	7.98	3.63	MYO1E	Unconventional myosin-Ie	NM_200636.1
B18G0616	7.98	7.56	URI1	Unconventional prefoldin RPB5 interactor	NM_001030145.2
A22G0194	7.98	22.36	STRIP1	Striatin-interacting protein 1 homolog	NM_213521.2
A3G0124	7.98	1.81	NF7O	Nuclear factor 7, ovary	XM_021471355.2
B23G0471	7.98	14.83	CTDSP2	Carboxy-terminal domain RNA polymerase II polypeptide A small phosphatase 2	NM_199544.1
A9G0681	7.98	18.05	SIK2	threonine-protein kinase SIK2	XM_005167449.5
B11G0368	7.98	17.03	PPFIA4	Liprin-alpha-4	XM_002663787.7
A7G0532	7.97	5.34	PLXDC2	Plexin domain-containing protein 2	NM_001146305.2
B7G0723	7.97	4.36	MYO1E	Unconventional myosin-Ie	XM_677757.10
A6G0398	7.97	4.70	CBX8	Chromobox protein homolog 8	NM_001024415.2
A12G0069	7.97	18.26	VDAC2	Voltage-dependent anion-selective channel protein 2	NM_001001404.1
B21G0023	7.97	7.82	TRIM16	Tripartite motif-containing protein 16	XM_690458.9
B13G0211	7.97	6.69	COA8	Cytochrome c oxidase assembly factor 8	NM_199917.1
A19G0763	7.97	3.49	FHL3	Four and a half LIM domains protein 3	NM_001099978.1
A9G0175	7.96	5.27	MFSD6B	Major facilitator superfamily domain-containing protein 6-B	NM_001328216.1
B13G0424	7.96	4.17	CCSAP	Centriole, cilia and spindle-associated protein	NM_201170.2
B12G0507	7.96	3.73	BHLHA15	Class A basic helix-loop-helix protein 15	NM_001077652.1
B14G0717	7.96	19.58	SLC43A3	Equilibrative nucleobase transporter 1	XM_009291002.4
A4G0735	7.96	8.18	VHL	von Hippel-Lindau disease tumor suppressor	
A8G0550	7.96	9.87	RIMKLB	Beta-citrylglutamate synthase B	NM_001004554.2
A7G0421	7.96	2.90	ZNF280D	Zinc finger protein 280D	XM_005166525.5
B24G0575	7.96	3.95	TM9SF1	Transmembrane 9 superfamily member 1	XM_021470138.2
B24G0333	7.96	4.03	CLDN11	Claudin-11	NM_131772.3
A7G1078	7.95	5.95	MAP4K2	Mitogen-activated protein kinase kinase kinase kinase 2	NM_001040358.1
A16G0718	7.95	5.70	CD4	T-cell surface glycoprotein CD4	
A12G0279	7.95	3.05	TRUB1	Pseudouridylate synthase TRUB1	NM_001128687.1
B24G0266	7.95	9.40	FAM168B	Myelin-associated neurite-outgrowth inhibitor	NM_001076569.1
B23G0591	7.95	4.73	TMEM201	Transmembrane protein 201	NM_001110466.1
A20G0568	7.95	6.11	KATNA1	Katanin p60 ATPase-containing subunit A1	NM_001020604.1
B8G0084	7.95	5.18	PPIH	Peptidyl-prolyl cis-trans isomerase H	
A10G0772	7.95	7.87	PPWD1	Peptidylprolyl isomerase domain and WD repeat-containing protein 1	NM_001098758.1
A12G0433	7.94	4.01	SNX11	Sorting nexin-11	XM_021480062.2
B3G0872	7.94	1.50	FRIM	Ferritin, middle subunit	NM_001109854.1
A3G0003	7.94	33.56	BGLAP	Osteocalcin	
B2G0987	7.94	7.01	TYW3	tRNA wybutosine-synthesizing protein 3 homolog	XM_005171159.5

B3G0577	7.94	10.39	LFNG	Beta-1,3-N-acetylglucosaminyltransferase lunatic fringe	NM_130971.2
A10G0461	7.94	10.89	DLG2	Disks large homolog 2	XM_009305411.4
A18G0296	7.94	6.83	LOC113061695	A0A6P6LQL5_CARAU uncharacterized protein LOC113061695 isoform X1	
B13G0199	7.94	4.88	AIFM2	Ferroptosis suppressor protein 1	NM_001004560.1
A24G0535	7.93	6.42	CYRIB	CYFIP-related Rac1 interactor B	NM_207078.1
B5G1095	7.93	1.59	PAXX	Protein PAXX	NM_001130597.1
A6G0965	7.93	6.19	UBE2C	Ubiquitin-conjugating enzyme E2 C	NM_001037691.2
A1G0212	7.93	13.28	TNPO2	Transportin-2	XM_021477402.2
B15G0599	7.93	7.97	XAF1	XIAP-associated factor 1	NM_001076647.2
B23G0967	7.93	10.14	SERP1	Stress-associated endoplasmic reticulum protein 1	NM_212943.2
B7G0720	7.93	4.08	MCEE	Methylmalonyl-CoA epimerase, mitochondrial	NM_001020766.1
A13G0238	7.93	6.76	AGPAT4	1-acyl-sn-glycerol-3-phosphate acyltransferase delta	NM_001018083.2
B1G1110	7.93	15.63	LOC113099797	A0A6P6PII8_CARAU zinc finger protein 709-like	XM_017357165.3
B12G0416	7.93	3.37	ST6GALNAC1	Alpha-N-acetylgalactosaminide alpha-2,6-sialyltransferase 1	NM_001328041.1
A1G0078	7.93	14.88	NLRP3	NACHT, LRR and PYD domains-containing protein 3	XM_009295700.4
B10G0710	7.93	6.41	XRCC4	DNA repair protein XRCC4	NM_200786.1
B15G0315	7.93	11.85	DHRS11	reductase SDR family member 11	NM_001002143.2
B9G0458	7.92	9.85	RIF1	Telomere-associated protein RIF1	XM_009304633.4
B1G0270	7.92	7.99	HGSNAT	Heparan-alpha-glucosaminide N-acetyltransferase	XM_002660409.6
B7G0768	7.92	5.13	APH1B	Gamma-secretase subunit Aph-1b	NM_200115.1
B9G0640	7.92	3.61	TMEM41AA	Transmembrane protein 41A-A	NM_001020765.1
A3G0576	7.92	6.52	NDE1-B	Nuclear distribution protein nudE homolog 1-B	NM_001030203.1
A25G0645	7.92	11.80	KV3A9	Ig kappa chain V-III region MOPC 63	XM_068217596.1
A14G0675	7.92	6.48	MAEA	E3 ubiquitin-protein transferase MAEA	NM_199549.2
B6G0806	7.92	12.64	PPP4R2B	threonine-protein phosphatase 4 regulatory subunit 2-B	NM_001110371.2
B1G0444	7.92	173.42	G5714_000822	A0A7J6DI45_9TELE Uncharacterized protein	XM_700557.8
B3G0172	7.92	17.72	HA1B	BOLA class I histocompatibility antigen, alpha chain BL3-7	NM_001287097.1
A21G0386	7.92	1.62	SDHAF1	Succinate dehydrogenase assembly factor 1, mitochondrial	
A23G0648	7.91	6.25	CHCHD6A	MICOS complex subunit mic25a	NM_001005584.1
B17G0475	7.91	9.19	CRYBG1	gamma crystallin domain-containing protein 1	XM_005158549.5
B3G1288	7.91	5.21	CBX8	Chromobox protein homolog 8	NM_205616.2
A21G0749	7.91	20.30	UNC119	Protein unc-119 homolog A	NM_001040394.2
B5G1063	7.91	1.08	AK6	Adenylate kinase isoenzyme 6	NM_001002118.2
A16G0692	7.91	0.93	LOC107671188	A0A671P3D7_9TELE Uncharacterized LOC107671188	NM_001328123.1
B17G0390	7.91	8.30	LTBP1	Latent-transforming growth factor beta-binding protein 1	XM_017351812.3

A6G0935	7.91	8.88	HYAL1	Hyaluronidase-1	NM_001039997.1
A8G0229	7.91	11.39	KIF2A	Kinesin-like protein KIF2A	XM_005166830.5
B4G0066	7.91	8.65	PMPCB	Mitochondrial-processing peptidase subunit beta	NM_001012496.2
A5G0660	7.91	8.83	IGBP1	Immunoglobulin-binding protein 1	XM_005165297.5
B15G0351	7.91	12.17	RELB	Transcription factor RelB	XM_001335557.6
A18G0410	7.91	4.74	HSD17B2	Estradiol 17-beta-dehydrogenase 2	NM_001080629.2
A16G0036	7.90	30.33	PTTG1IP	Pituitary tumor-transforming gene 1 protein-interacting protein	XM_021467055.2
B10G0155	7.90	4.84	RBM18	Probable RNA-binding protein 18	NM_200793.1
A2G1150	7.90	6.24	EPS15L1	Epidermal growth factor receptor substrate 15-like 1	XM_021468308.2
A6G0381	7.90	9.98	NOL11	Nucleolar protein 11-like	
B10G0703	7.90	15.09	WDR45	WD repeat domain phosphoinositide-interacting protein 4	NM_200231.1
A5G0548	7.90	5.82	RUVBL2	RuvB-like 2	NM_174860.2
B17G0007	7.90	6.55	PPP2R5C	threonine-protein phosphatase 2A 56 kDa regulatory subunit gamma isoform	XM_001344835.9
A25G0785	7.90	8.11	SLC10A3	P3 protein	NM_214740.1
A24G0412	7.90	3.33	QTRT2	Queuine tRNA-ribosyltransferase accessory subunit 2	NM_001118896.2
B20G0333	7.89	5.60	BTBD6B	POZ domain-containing protein 6-B	XM_005160494.5
A1G0058	7.89	4.64	TNFSF14	Tumor necrosis factor ligand superfamily member 14	
A3G0239	7.89	8.01	TFAP4	Transcription factor AP-4	XM_005168469.5
B7G0811	7.89	14.52	CAPRIN1	Caprin-1	NM_001003484.1
A12G0189	7.89	5.48	LOC113111687	A0A6P6QGS1_CARAU protein FAM104A-like	NM_001328019.1
A22G0290	7.89	15.78	AHR	Aryl hydrocarbon receptor	NM_131264.1
B4G0074	7.89	16.06	CPSF6	Cleavage and polyadenylation specificity factor subunit 6	XM_005164453.5
A1G0066	7.89	5.73	SLC27A1	Long-chain fatty acid transport protein 1	NM_001077248.1
A20G0464	7.89	7.34	FNTB	Protein farnesyltransferase subunit beta	NM_001002128.1
A11G0069	7.89	5.50	TMEM63B	CSC1-like protein 2	XM_068224169.1
B11G0259	7.89	6.76	JUP	Junction plakoglobin	XM_009305927.4
A20G0697	7.89	4.03	BLOC1S2	Biogenesis of lysosome-related organelles complex 1 subunit 2	NM_001033101.2
A20G0876	7.89	7.71	UBE2J1	Ubiquitin-conjugating enzyme E2 J1	NM_214767.1
A13G0769	7.89	4.68	DLK2	Protein delta homolog 2	XM_688722.9
A13G0462	7.89	7.83	KAT6B	Histone acetyltransferase KAT6B	XM_001344334.8
B25G0735	7.89	9.23	SNUPN	Snurportin-1	NM_199694.1
A16G0060	7.88	4.74	SRSF10	arginine-rich splicing factor 10	NM_200533.2
B17G0231	7.88	14.89	FBXO33	F-box only protein 33	XM_691051.8
A19G0501	7.88	6.53	TPMT	Probable thiopurine S-methyltransferase	NM_001002569.2
B15G0262	7.88	9.43	STRN4	Striatin-4	XM_021466324.2
A7G0575	7.88	6.99	CALD1	Caldesmon	NM_001423832.1
B22G1019	7.88	4.04	NLRC3	NLR family CARD domain-containing protein 3	XM_021478497.2
A2G0385	7.88	9.79	SH3GLB1	Endophilin-B1	XM_068223128.1

B10G0557	7.88	7.96	ZDHHC20A	Palmitoyltransferase ZDHHC20-A	NM_001083549.1
B21G0495	7.88	3.99	NECTIN1	Nectin-1	XM_005161129.5
B17G0485	7.88	9.79	ACSS1	Acetyl-coenzyme A synthetase 2-like, mitochondrial	NM_001080656.3
A17G0138	7.87	18.04	ISCA2	Iron-sulfur cluster assembly 2 homolog, mitochondrial	
B11G0370	7.87	9.58	LOC113055225	A0A6P6KXU3_CARAU uncharacterized protein LOC113055225 isoform X5	NM_131842.2
B3G0964	7.87	27.97	SLC25A39	Probable mitochondrial glutathione transporter SLC25A39	XM_068216183.1
A8G0623	7.87	3.75	GADD45G	Growth arrest and DNA damage-inducible protein GADD45 gamma	NM_205691.1
B5G0941	7.87	4.50			XM_021471612.2
A10G0189	7.87	1.92	FGFR1A	Fibroblast growth factor receptor 1-A	XM_021479189.2
A7G0302	7.87	7.31	PIEZO1	Piezo-type mechanosensitive ion channel component 1	XM_691263.9
B22G0575	7.87	4.71	MMADHC	Cobalamin trafficking protein CblD	NM_001002453.1
B10G0411	7.87	7.38	EHBP1L1	EH domain-binding protein 1-like protein 1	XM_017358054.3
A3G1206	7.86	4.87	TCF20	Transcription factor 20	XM_684281.8
A2G0253	7.86	29.42	TFRC	Transferrin receptor protein 1	NM_001009917.1
A23G0059	7.86	10.84	NUDT3	Diphosphoinositol polyphosphate phosphohydrolase 1	NM_201145.2
A19G0911	7.86	3.72	DUS3L	tRNA-dihydrouridine(47) synthase [NAD(P)(+)]-like	NM_200674.2
B3G0530	7.86	13.18	UNK	RING finger protein unkempt homolog	XM_005164231.5
B12G0015	7.86	7.58	ZRANB1	Ubiquitin thioesterase ZRANB1	XM_009306985.4
A1G0195	7.86	10.78	LOC113056681	A0A6P6L747_CARAU Arginine vasopressin-induced protein 1	
A4G0644	7.86	7.84	BCAT1	Branched-chain-amino-acid aminotransferase, cytosolic	NM_200064.1
B21G0411	7.86	18.25	ADSS2	Adenylosuccinate synthetase isozyme 2	NM_173237.2
B1G0480	7.86	5.14	ANKRD10	Ankyrin repeat domain-containing protein 10	NM_200980.1
A3G0782	7.85	67.74	EMC10	ER membrane protein complex subunit 10	NM_001102389.2
A13G0869	7.85	9.86	TMEM14A	Transmembrane protein 14A	XM_017358727.2
A20G0628	7.85	8.74	PPP2R5E	threonine-protein phosphatase 2A 56 kDa regulatory subunit epsilon isoform	NM_194415.2
A7G0648	7.85	13.65	ATP6V0D1	V-type proton ATPase subunit d 1	NM_199620.1
B10G0149	7.85	10.16	RABGAP1	Rab GTPase-activating protein 1	XM_017358005.3
A19G0725	7.85	5.33	GABPB2	GA-binding protein subunit beta-2	NM_001099977.2
A16G0898	7.85	9.31	SDC2	Syndecan-2	
B24G0418	7.84	4.55	ZC2HC1A	Zinc finger C2HC domain-containing protein 1A	NM_199680.1
B3G1269	7.84	4.98	SPC24	Kinetochore protein Spc24	NM_001024440.1
A6G0989	7.84	12.09	STMN3	Stathmin-3	XM_001339340.9
B1G0718	7.84	5.11	CISD2	CDGSH iron-sulfur domain-containing protein 2	NM_200383.1

B9G0791	7.84	6.56	IGSF3	Immunoglobulin superfamily member 3	XM_003199164.6
A1G0208	7.84	5.70	TSC2	Tuberin	NM_001328401.1
B2G0949	7.84	5.83	CC2D1B	Coiled-coil and C2 domain-containing protein 1B	XM_068213801.1
A21G0571	7.83	5.73	DPP3	Dipeptidyl peptidase 3	XR_011007955.1
B1G0061	7.83	10.54	RCAN1	Calcipressin-1	XM_021470802.2
B22G0678	7.83	1.50	NRDC	Nardilysin	XR_011008163.1
B13G0430	7.83	11.22	CEP68	Centrosomal protein of 68 kDa	NM_200621.1
B22G0858	7.83	5.41	SDE2	Replication stress response regulator SDE2	NM_001017752.2
A13G0689	7.82	5.49	ZFAND4	AN1-type zinc finger protein 4	NM_001386797.1
A4G0725	7.82	0.50	A0A673L1G6	A0A673L1G6_9TELE Interleukin 26	
A16G0742	7.82	10.18	ISOC2	Isochorismatase domain-containing protein 2	NM_001079953.2
B16G0309	7.82	7.11	STT3B	Dolichyl-diphosphooligosaccharide--protein glycosyltransferase subunit STT3B	NM_001328161.1
B3G0466	7.82	3.14	LPAR1	Lysophosphatidic acid receptor 1	XM_009299773.4
B5G1042	7.82	7.43	TM2D2	TM2 domain-containing protein 2	NM_001002640.1
A12G0374	7.82	4.57	CRIPT	Cysteine-rich PDZ-binding protein	NM_001017893.1
A9G0208	7.82	10.65	SNX4	Sorting nexin-4	NM_001014346.1
B10G0854	7.82	10.19	AKT2	threonine-protein kinase	NM_212815.2
B20G0839	7.82	12.83	HADHA	Trifunctional enzyme subunit alpha, mitochondrial	NM_001105276.1
B17G0564	7.82	0.35	G5714_017112	A0A7J6C959_9TELE SH3 domain-containing protein	
B24G0551	7.81	3.31	NGDN	Neuroguidin	NM_198373.2
B15G0184	7.81	8.51	GET1	Guided entry of tail-anchored proteins factor 1	NM_001003413.1
A6G0892	7.80	22.96	PSMF1	Proteasome inhibitor PI31 subunit	NM_001002547.2
B16G0595	7.80	18.99	ARHGEF1	Rho guanine nucleotide exchange factor 1	XM_021466702.2
A2G0961	7.80	12.09	EPC1	Enhancer of polycomb homolog 1	XM_068224657.1
A11G0154	7.80	10.45	LY75	Lymphocyte antigen 75	NM_201450.1
B3G0103	7.80	43.72	GIMAP8	GTPase IMAP family member 8	XM_068218152.1
B14G0841	7.80	12.42	GPR174	Probable G-protein coupled receptor 174	NM_001089527.1
B5G0502	7.80	3.19	GSN	Gelsolin	NM_001089344.1
A13G0578	7.80	13.48	CEP170	Centrosomal protein of 170 kDa	NM_198804.1
B9G0594	7.79	6.26	CRLA	Cyclic AMP receptor-like protein A	NM_001423490.1
B11G0087	7.79	0.84	BPI	Bactericidal permeability-increasing protein	
B4G0016	7.79	15.45	MAPRE3	EB family member 3	NM_001002170.1
B22G1076	7.79	7.38	SRF	Serum response factor	NM_001110526.1
B3G0607	7.79	5.34	ZNF598	E3 ubiquitin-protein ligase ZNF598	NM_001033718.1
A25G0439	7.79	4.68	HSD17B12A	Very-long-chain 3-oxoacyl-CoA reductase-A	NM_200881.1
A3G0375	7.79	4.53	LPAR1	Lysophosphatidic acid receptor 1	XM_009299773.4
A16G0575	7.79	2.14	MYLIPB	E3 ubiquitin-protein ligase MYLIP-B	NM_001080022.2
B14G0138	7.79	1.05	NEK1	threonine-protein kinase Nek1	NM_001017548.2

B23G0490	7.79	7.68	LRP1	Low-density lipoprotein receptor-related protein 1	XM_005162219.5
A15G0747	7.79	7.63	CHCHD2	Coiled-coil-helix-coiled-coil-helix domain-containing protein 2	NM_200767.1
A14G0451	7.79	11.91	RMND5B	E3 ubiquitin-protein transferase RMND5B	NM_200774.1
B20G0052	7.78	16.66	PCMT	Protein-L-isoaspartate(D-aspartate) O-methyltransferase	NM_131465.3
A11G0688	7.78	9.74	HARBI1	Putative nuclease HARBI1	NM_001002369.1
B17G0579	7.78	8.75	CCDC32	Coiled-coil domain-containing protein 32	NM_001198753.1
A21G0159	7.78	9.51	EGFL7	Epidermal growth factor-like protein 7	NM_001040385.1
B14G0275	7.78	4.34	ITK	TSK	NM_131104.2
B6G0573	7.77	9.18	GPBP1L1	Vasculin-like protein 1	XM_021477183.2
B16G0341	7.77	4.98	PRUNE1	Exopolyphosphatase PRUNE1	NM_001160294.1
B5G1110	7.77	17.36	TIA1	Cytotoxic granule associated RNA binding protein TIA1	NM_200182.2
B4G0272	7.77	4.04	NAF1	ACA ribonucleoprotein complex non-core subunit NAF1	XM_691652.7
B15G0584	7.77	6.36	BLMH	Bleomycin hydrolase	NM_200018.1
A5G0202	7.77	3.07	G5714_005261	A0A7J6D0H4_9TELE Uncharacterized protein	
A19G0618	7.77	10.43	PSMD4	26S proteasome non-ATPase regulatory subunit 4	NM_001002112.2
B3G0473	7.77	7.14	XAB2	Pre-mRNA-splicing factor SYF1	NM_001044783.1
A13G0321	7.77	5.30	PCNX4	Pecanex-like protein 4	NM_001003532.1
A8G0168	7.76	8.21	CDK16	Cyclin-dependent kinase 16	XM_001335539.8
B21G0213	7.76	17.61	CNOT8	CCR4-NOT transcription complex subunit 8	XM_005173877.4
A25G0348	7.76	4.32	ARMC10	Armadillo repeat-containing protein 10	XM_009297973.4
B17G0612	7.76	3.03	CGREF1	Cell growth regulator with EF hand domain protein 1	XM_017351827.3
B4G0564	7.76	17.38	CELF2	CUGBP Elav-like family member 2	XM_017354958.3
B23G0478	7.76	5.61	NOL9	Polynucleotide 5'-hydroxyl-kinase NOL9	XM_691014.7
A1G0870	7.76	4.19	LOC113109534	A0A6P6Q608_CARAU uncharacterized protein LOC113109534	XM_009306315.4
B5G1142	7.75	2.50	GBGT1	Globoside alpha-1,3-N-acetylgalactosaminyltransferase 1	NM_200125.1
A1G0394	7.75	12.04	F8A1	40-kDa huntingtin-associated protein	XM_005160037.5
A5G0775	7.75	8.12	PIAS2	E3 SUMO-protein ligase PIAS2	NM_001326481.1
A6G0975	7.75	27.95	RTF2	Replication termination factor 2	XM_005168778.3
A9G0454	7.75	4.31	MAIP1	m-AAA protease-interacting protein 1, mitochondrial	NM_001037226.2
B6G0318	7.75	4.86	NCK2	Cytoplasmic protein NCK2	NM_001003492.2
A12G0504	7.75	17.18	TOM1L2	TOM1-like protein 2	NM_001351676.1
B1G0901	7.74	7.73	OSBP	Oxysterol-binding protein 1	NM_001145585.1
A1G0821	7.74	1.12	GRAP	GRB2-related adapter protein	NM_001030080.2
B24G0723	7.74	9.50	ITGB1	Integrin beta-1	NM_001034971.1
B22G0232	7.74	5.07	LOC113081386	A0A6P6NJW2_CARAU uncharacterized protein LOC113081386	NM_001431258.1
B10G0525	7.74	7.02	RFC3	Replication factor C subunit 3	NM_201457.1
B16G0433	7.74	11.16	KPNA5	Importin subunit alpha-6	NM_001018153.1

B8G0493	7.74	5.85	IFRD2	Interferon-related developmental regulator 2	XM_005167029.5
A16G1056	7.74	48.84	STXA	Stonustoxin subunit alpha	
A6G0241	7.74	14.38	CALCRL	Calcitonin gene-related peptide type 1 receptor	XM_001920000.7
B9G0051	7.74	16.16	NLRC3	Protein NLRC3	XM_009295700.4
A23G0862	7.73	1.96	SGMS2	Phosphatidylcholine:ceramide cholinephosphotransferase 2	NM_001003641.2
B11G0733	7.73	7.18	SHROOM2	Protein Shroom2	NM_213273.1
B2G0200	7.73	17.94	PER2	Period circadian protein homolog 2	XM_021478636.2
B12G0191	7.73	4.35	SFXN2	Sideroflexin-2	NM_212730.1
A6G0382	7.73	13.53	SEC14L1	SEC14-like protein 1	XM_021477068.2
A11G0058	7.73	2.95	CLCN4	Cl(-) exchange transporter 4	
B19G0208	7.72	14.53	NAXE	NAD(P)H-hydrate epimerase	
A20G0917	7.72	9.23	SYNCRIP	Heterogeneous nuclear ribonucleoprotein Q	NM_214696.1
B5G0406	7.72	1.87	BICDL2	BICD family-like cargo adapter 2	NM_001193591.1
B11G0255	7.72	8.39	ABCC10	ATP-binding cassette sub-family C member 10	NM_213467.1
B9G0190	7.72	10.86	UBE2E3	Ubiquitin-conjugating enzyme E2 E3	NM_200921.3
A1G1004	7.72	4.67	LAP3	Cytosol aminopeptidase	NM_001114847.1
B22G0265	7.72	4.42	DPX16_0806	A0A3N0Z9S2_ANAGA Ig-like domain-containing protein	XM_005161645.5
A25G0814	7.72	4.22	PCLAF	PCNA-associated factor	XM_002666755.6
A3G1310	7.72	5.13	G5714_004013	A0A7J6DCD2_9TELE Uncharacterized protein	
B2G1014	7.72	10.89	WDR33	pre-mRNA 3' end processing protein WDR33	XM_005163232.5
B20G0095	7.72	4.89	CEP170B	Centrosomal protein of 170 kDa protein B	XM_005160328.5
A6G0839	7.72	6.98	PPM1H	Protein phosphatase 1H	XM_679889.9
B22G0675	7.71	3.02	ATPAF1	ATP synthase mitochondrial F1 complex assembly factor 1	NM_001017907.2
B10G0765	7.71	12.85	SBNO1	Protein strawberry notch homolog 1	NM_001199916.2
A11G0320	7.71	9.98	PLEKHA6	Pleckstrin homology domain-containing family A member 6	NM_001365237.1
B6G0466	7.71	7.26	LMO4	LIM domain transcription factor LMO4	NM_212689.2
A14G0763	7.71	9.34	RNF12-A	E3 ubiquitin-protein ligase RNF12-A	XM_009307767.4
A2G0123	7.71	7.57	PHR	Deoxyribodipyrimidine photo-lyase	NM_201064.2
B2G0922	7.70	7.64	ARHGEF4	Rho guanine nucleotide exchange factor 4	XM_009298441.4
A5G1287	7.70	3.96	ALDH2	Aldehyde dehydrogenase, mitochondrial	NM_200490.1
A24G0004	7.70	18.01	WWP1	NEDD4-like E3 ubiquitin-protein ligase WWP1	XM_068217284.1
B13G0425	7.70	1.71	RAB4A	Ras-related protein Rab-4A	NM_212982.1
B13G0909	7.70	6.25			NM_001327835.1
A16G0171	7.70	8.19	SYNDIG1L	Synapse differentiation-inducing gene protein 1-like	
B7G0433	7.70	10.26	IGF2	Insulin-like growth factor II	NM_131433.1
B19G0132	7.70	7.37	WIPF2	WASL-interacting protein family member 2	XM_005173756.5
A10G0295	7.70	12.94	SERPINH1	Serpin H1	NM_001110374.2
A5G0684	7.70	8.98	NFKBIB	NF-kappa-B inhibitor beta	NM_001128795.1

A19G0096	7.70	9.24	TCB1	Transposable element Tcb1 transposase	XM_021471168.2
A13G0204	7.70	14.22	L2HGDH	L-2-hydroxyglutarate dehydrogenase, mitochondrial	XM_692747.9
B13G0064	7.70	6.72	LOC113057258	A0A6P6L9C4_CARAU gastrula zinc finger protein XlCGF26.1-like	NM_182860.1
A5G0692	7.69	10.98	U2SURP	U2 snRNP-associated SURP motif-containing protein	NM_001089532.2
B16G0823	7.69	10.88	FLCN	Folliculin	NM_001040373.1
B24G0291	7.69	3.84	RNPC3	RNA-binding region-containing protein 3	NM_001039930.1
B17G0387	7.69	14.25	YIPF4	Protein YIPF4	NM_212891.1
A25G0428	7.69	3.45	ZNF277	Zinc finger protein 277	NM_200430.1
A1G0634	7.69	2.35	LOC107719745	A0A673GCE5_9TELE Uncharacterized LOC107719745	XM_001923447.7
B18G0508	7.69	6.02	MAP2K1	Dual specificity mitogen-activated protein kinase kinase 1	NM_213419.2
B25G0879	7.69	3.34	TUF11	Ubiquitin-60S ribosomal protein L40	
B22G0516	7.69	3.85	OGN	Mimecan	NM_001013570.2
A9G0500	7.69	7.36	EAF2	ELL-associated factor 2	NM_001002162.1
A16G0308	7.69	3.59	PI4KB	Phosphatidylinositol 4-kinase beta	XM_005158219.5
B16G0743	7.69	11.93	GNL1	Guanine nucleotide-binding protein-like 1	NM_001077253.1
A20G0347	7.69	4.34	MCM3	DNA replication licensing factor MCM3	NM_212567.2
A21G0755	7.69	4.09	DDX52	Probable ATP-dependent RNA helicase DDX52	NM_001044315.2
A5G0471	7.69	6.99	LOXL2B	Lysyl oxidase homolog 2B	NM_001080626.1
B20G0344	7.68	3.57	DAAM1	Disheveled-associated activator of morphogenesis 1	NM_001030136.2
A5G0795	7.68	11.21	TRPV1	Transient receptor potential cation channel subfamily V member 1	XM_005165327.5
B23G0777	7.68	8.99	MDP1	Magnesium-dependent phosphatase 1	
A16G0349	7.68	6.13	RCC1	Regulator of chromosome condensation	NM_213178.1
A19G0345	7.68	15.11	RNF19B	E3 ubiquitin-protein ligase RNF19B	NM_001202440.1
B20G0554	7.68	7.68	LRATD1	Protein LRATD1	NM_200618.1
A10G0798	7.68	6.30	SMAD2	Mothers against decapentaplegic homolog 2	NM_131366.3
A23G0602	7.68	5.07	ARMC1	Armadillo repeat-containing protein 1	NM_199539.2
A7G0128	7.68	23.89	YWHAE	14-3-3 protein epsilon	NM_001013341.2
A3G0562	7.68	14.57	FMNL1	Formin-like protein 1	XM_680041.8
B15G0885	7.67	10.73	ACAP2	Arf-GAP with coiled-coil, ANK repeat and PH domain-containing protein 2	XM_009291987.4
A12G0204	7.67	6.17	TRIM8	E3 ubiquitin-protein ligase TRIM8	NM_001044914.1
A17G0663	7.67	4.95	GEMIN2	Gem-associated protein 2	NM_001017608.2
B8G0525	7.67	7.59	PPP1R3A	Protein phosphatase 1 regulatory subunit 3A	
B2G0023	7.67	4.25	NOP9	Nucleolar protein 9	XM_002662078.6
B20G0190	7.67	10.69	GLRX5	Glutaredoxin-related protein 5, mitochondrial	NM_213021.1

A6G1022	7.67	5.28	MTRF1	Peptide chain release factor 1, mitochondrial	
B20G0448	7.67	9.80	E(BX)	Nucleosome-remodeling factor subunit NURF301	NM_212629.1
A23G0207	7.67	7.98	CHL1	Neural cell adhesion molecule L1-like protein	NM_001163106.2
A15G0840	7.67	6.52	PANX1	Pannexin-1	NM_200916.1
A3G0200	7.67	10.70	JAML	Junctional adhesion molecule-like	XM_068218714.1
A12G0751	7.66	4.68	IRF3	Interferon regulatory factor 3	
A4G0737	7.66	5.59	LEMD3	Inner nuclear membrane protein Man1	NM_001044864.1
A20G0789	7.66	22.60	SLC30A1	Zinc transporter 1	NM_200879.1
A8G0272	7.66	6.19	BCAR3	Breast cancer anti-estrogen resistance protein 3 homolog	XM_021478296.2
A19G0061	7.66	18.99	TOP2B	DNA topoisomerase 2-beta	NM_001045191.2
A14G0183	7.66	14.00	YIF1A	Protein YIF1A	NM_199931.1
A7G0431	7.66	10.27	IQGAP1	Ras GTPase-activating-like protein IQGAP1	NM_001308966.1
B7G1153	7.65	6.76	DNAJC3	DnaJ homolog subfamily C member 3	NM_131630.2
B5G1118	7.65	11.90	PHF23A	PHD finger protein 23A	NM_001013499.1
B6G0128	7.65	4.80	ZSCAN29	Zinc finger and SCAN domain-containing protein 29	
A3G0467	7.65	7.93	ZFAND2B	AN1-type zinc finger protein 2B	XM_005164201.5
B3G1000	7.65	5.34	NUBP1	Cytosolic Fe-S cluster assembly factor nubp1	NM_001017538.2
B8G0892	7.65	5.08	EMC3	ER membrane protein complex subunit 3	NM_001009987.2
B2G0687	7.65	5.12	TMEM70	Transmembrane protein 70, mitochondrial	XM_002660832.7
B4G0895	7.65	9.40	KDM5A	Lysine-specific demethylase 5A	XM_017354458.3
B16G0396	7.65	4.47	SNIP1	Smad nuclear-interacting protein 1	NM_001025470.1
B16G0574	7.65	6.60	INO80C	INO80 complex subunit C	NM_001002108.2
B23G0441	7.65	7.42	NOC2L	Nucleolar complex protein 2 homolog	XM_017353600.3
B19G0750	7.65	0.98	NEU1	Sialidase-1	
B13G0900	7.64	5.34			NM_201206.2
A2G0090	7.64	11.20	PIK3R1	Phosphatidylinositol 3-kinase regulatory subunit alpha	NM_001291329.1
B7G1250	7.64	11.85	MYADM	Myeloid-associated differentiation marker homolog	XM_001344498.8
B21G0291	7.64	9.83	RGS14	Regulator of G-protein signaling 14	XM_021473436.2
B13G0510	7.64	4.56	FBXW4	WD repeat-containing protein 4	NM_200907.1
B1G0230	7.64	2.70	PLA2G12A	Group XIIA secretory phospholipase A2	
B11G0314	7.64	10.66			NM_001077467.1
B22G0722	7.64	16.43	MIDN	Midnolin	XM_005170436.5
A16G0684	7.64	9.93	DDR1	Epithelial discoidin domain-containing receptor 1	XM_009292275.4
B20G0083	7.64	7.81	FYNB	Tyrosine-protein kinase fynb	XM_068215552.1
A7G0310	7.63	7.03	MAD2L1	Mitotic spindle assembly checkpoint protein MAD2A	NM_001017739.1
B18G0799	7.63	4.15	PIGB	GPI mannosyltransferase 3	
A13G0416	7.63	6.30	DDX43	Probable ATP-dependent RNA helicase DDX43	NM_213354.1
A8G0349	7.63	9.34	NPL	N-acetylneuraminate lyase	NM_207051.2
B18G0373	7.63	21.68	ACO	1-aminocyclopropane-1-carboxylate oxidase	XM_021467563.2

B5G0888	7.63	3.43	SMARCA2	Probable global transcription activator SNF2L2	XM_021475955.2
B12G0731	7.63	3.51	VKORC1L1	Vitamin K epoxide reductase complex subunit 1-like protein 1	NM_001327932.1
B21G0551	7.63	5.48	ITPKC	Inositol-trisphosphate 3-kinase C	XM_681366.10
B11G0020	7.63	7.66	RPL29	60S ribosomal protein L29	NM_001017575.1
B20G0529	7.63	3.10	FNDC1	Fibronectin type III domain-containing protein 1	XM_003200712.6
B6G0030	7.63	5.75	TAB1	TGF-beta-activated kinase 1 and MAP3K7-binding protein 1	XM_002662240.7
A9G0817	7.63	9.92	TLK1B	threonine-protein kinase tousled-like 1-B	NM_001199724.1
B12G0293	7.62	9.04	BEND6	BEN domain-containing protein 6	XM_683728.9
A22G0626	7.62	14.85	MXI1	Max-interacting protein 1	NM_131237.2
B23G0206	7.62	9.17	DDX23	Probable ATP-dependent RNA helicase DDX23	NM_199882.1
B13G0017	7.62	4.97	LOC107571481	A0A672MIG4_SINGR Uncharacterized LOC107571481	XM_001920829.9
A8G0173	7.62	7.10	EMC3	ER membrane protein complex subunit 3	NM_001009987.2
A9G0532	7.62	2.73	MRPL30	39S ribosomal protein L30, mitochondrial	
B20G0901	7.62	4.02	PYCR3	Pyrroline-5-carboxylate reductase 3	NM_001044938.1
B16G0041	7.62	1.67	KDF1	Keratinocyte differentiation factor 1	XM_001338342.9
A3G0744	7.62	0.49	DPX16_16969	A0A3N0XCQ7_ANAGA Uncharacterized protein	
B20G0774	7.62	4.47	TRMT6	tRNA (adenine(58)-N(1))-methyltransferase non-catalytic subunit TRM6	NM_001037392.2
B8G0970	7.61	13.48	PPTC7	Protein phosphatase PTC7 homolog	NM_001007378.1
A2G0359	7.61	60.78	GLUL	Glutamine synthetase	NM_181559.2
A14G0343	7.61	8.17	TAF7	Transcription initiation factor TFIID subunit 7	NM_173260.1
B17G0520	7.61	26.69	QKIA	Protein quaking-A	NM_131224.1
A20G0072	7.61	7.08	HLX	H2.0-like homeobox protein	NM_212701.2
B5G0754	7.61	14.74	ZNHIT1	Zinc finger HIT domain-containing protein 1	NM_001017401.1
A3G0425	7.61	5.84	POLR3K	DNA-directed RNA polymerase III subunit RPC10	NM_001002553.1
B14G0195	7.61	6.59	GPC4	Glypican-4	NM_131860.2
B5G0679	7.61	9.66	RUFY3	Protein RUFY3	XM_021476146.2
B12G0618	7.61	8.88	DNAJC9	DnaJ homolog subfamily C member 9	NM_001002433.1
A18G0488	7.61	8.71	IL34	Interleukin-34	NM_001128701.1
A10G0565	7.61	4.49	IQCJ-SCHIP1	IQCJ-SCHIP1 readthrough transcript protein	XM_009305278.4
A23G0544	7.61	5.08	DFFA	DNA fragmentation factor subunit alpha	NM_001002631.1
A5G0276	7.60	4.86	ANKRD13A	Ankyrin repeat domain-containing protein 13A	XM_021476185.2
B22G1051	7.60	14.01	ACAP2	Arf-GAP with coiled-coil, ANK repeat and PH domain-containing protein 2	NM_001102639.1
B20G0485	7.60	43.70	SUSD6	Sushi domain-containing protein 6	XR_011007671.1

B1G0906	7.60	21.71	LOC113097612	A0A6P6PC80_CARAU uncharacterized protein LOC113097612	
A8G0513	7.60	5.10	TMEM9	Proton-transporting V-type ATPase complex assembly regulator TMEM9	NM_001328536.1
B16G0338	7.60	13.22	SELENBP1	Methanethiol oxidase	XM_005158207.3
A15G0749	7.60	6.87	WDR62	WD repeat-containing protein 62	XM_694487.8
B22G0139	7.60	10.39	GRAMD2B	GRAM domain-containing protein 2B	
A21G0633	7.60	4.40	RRM1	Ribonucleoside-diphosphate reductase large subunit	NM_131455.2
B9G0314	7.60	6.04	SLC35A5	Probable UDP-sugar transporter protein SLC35A5	NM_200654.2
A2G1067	7.60	2.97	SH3GL1	Endophilin-A2	NM_001105606.1
A10G0615	7.59	7.52	RASSF2	Ras association domain-containing protein 2	NM_001002195.1
B18G0323	7.59	12.19	TIPARP	Protein mono-ADP-ribosyltransferase TIPARP	NM_001045019.3
B13G0625	7.59	7.82	TMEM39B	Transmembrane protein 39B	XM_009307182.3
B14G0164	7.59	3.13	SLC7A2	Cationic amino acid transporter 2	XM_068213200.1
A19G0854	7.59	5.94	CLPTM1L	Lipid scramblase CLPTM1L	NM_001002380.1
B6G0984	7.59	7.29	PLTP	Phospholipid transfer protein	XM_068221697.1
A9G0341	7.59	1.48	ITGBL1	Integrin beta-like protein 1	NM_001024072.1
B16G0057	7.59	8.60	GPATCH3	G patch domain-containing protein 3	NM_001080630.1
A8G0604	7.59	7.03	SPECC1LA	Cytospin-A	XM_005167177.5
B8G1061	7.58	3.80			
B24G0351	7.58	8.07	KLHL24	Kelch-like protein 24	NM_200440.1
B8G0288	7.58	17.37	SF3A1	Splicing factor 3A subunit 1	NM_200094.1
B3G0901	7.58	4.82	STRADA	STE20-related kinase adapter protein alpha	NM_199911.2
B20G0115	7.58	4.75	CCNK	Cyclin-K	NM_001163779.1
B24G0373	7.58	5.64	NUP58	p45	XM_005162684.4
B14G0235	7.58	6.63	CHIC1	Cysteine-rich hydrophobic domain-containing protein 1	NM_001201463.1
A12G0456	7.58	13.50	ZC3H7B	Zinc finger CCCH domain-containing protein 7B	XM_680874.10
A20G0505	7.58	13.60	TRIM25	ISG15 ligase TRIM25	XM_068215643.1
A10G0286	7.58	12.69	PCP4	Calmodulin regulator protein PCP4	NM_001166122.3
A7G0238	7.58	3.72	SINHCAF	SIN3-HDAC complex-associated factor	NM_001353917.1
A15G0122	7.58	9.06	NLRP3	NACHT, LRR and PYD domains-containing protein 3	XM_005157811.5
B8G0982	7.58	0.43	FUT9	4-galactosyl-N-acetylglucosaminide 3-alpha-L-fucosyltransferase 9	NM_001007454.2
B8G0621	7.58	6.93	LRIF1	Ligand-dependent nuclear receptor-interacting factor 1	
A23G0619	7.57	11.44	DGKB	Diacylglycerol kinase beta	NM_001045315.1
A6G0652	7.57	10.22	OS9	Protein OS-9	NM_001075108.1
B12G0226	7.57	3.77	COX11	Cytochrome c oxidase assembly protein COX11, mitochondrial	NM_001082958.1
A3G0691	7.57	12.40	KAT8	Histone acetyltransferase KAT8	NM_212742.2
B8G0119	7.57	8.73	NAA35	N-alpha-acetyltransferase 35, NatC auxiliary subunit	NM_199550.1
B5G1190	7.57	8.14	LOC113048846	A0A6P6K3W2_CARAU uncharacterized protein LOC113048846 isoform X1	XM_068218224.1

A22G0238	7.57	5.60	MFSD14A	Hippocampus abundant transcript 1 protein	NM_213527.2
B1G0094	7.57	6.94	STK16	threonine-protein kinase 16	NM_001017630.1
A3G0608	7.57	5.17	CRLF3	Cytokine receptor-like factor 3	NM_001017817.2
A3G1296	7.57	47.83	ENDOD1	Endonuclease domain-containing 1 protein	XM_017352926.2
A18G0210	7.57	13.43	IRF8	Interferon regulatory factor 8	NM_001002622.1
B18G0514	7.57	12.49	HACD3	Very-long-chain (3R)-3-hydroxyacyl-CoA dehydratase	NM_001044984.1
B23G0987	7.57	10.12	G3BP2	Ras GTPase-activating protein-binding protein 2	NM_213374.2
B4G0277	7.56	8.95	ING3	Inhibitor of growth protein 3	NM_200937.2
A13G0757	7.56	2.32	PKDCC	Extracellular tyrosine-protein kinase PKDCC	NM_001020757.2
A22G0618	7.56	5.69	SHOC2	Leucine-rich repeat protein SHOC-2	NM_001044786.1
A22G0212	7.56	4.16	RPP14	Ribonuclease P protein subunit p14	NM_001020692.2
B8G0548	7.55	12.50	SORT1	Sortilin	NM_001126465.1
B11G0352	7.55	8.97	SLC6A14	Sodium- and chloride-dependent neutral and basic amino acid transporter B(0+)	NM_213539.1
A17G0047	7.55	8.08	EIPR1	EARP and GARP complex-interacting protein 1	NM_001430912.1
A13G0205	7.55	2.36	SOS2	Son of sevenless homolog 2	
A7G0140	7.55	14.17	ACER2	Alkaline ceramidase 2	XM_002663031.7
A1G0191	7.55	13.16	R3HCC1L	Coiled-coil domain-containing protein R3HCC1L	XM_685202.9
B22G0327	7.55	7.35			
B8G0804	7.54	7.66	DEDD	Death effector domain-containing protein	NM_001002639.1
A12G0393	7.54	8.14	WACA	WW domain-containing adapter protein with coiled-coil	XM_009306588.3
B7G0687	7.54	4.18	ANO9	Anoctamin-9	XM_001922625.8
A23G0132	7.54	4.06	UBE2T	Ubiquitin-conjugating enzyme E2 T	NM_001077295.2
B20G0157	7.54	4.81	G5714_019375	A0A7J6BW86_9TELE Uncharacterized protein	NM_001020779.2
B9G0388	7.54	9.21	MORC3	MORC family CW-type zinc finger protein 3	XM_068223384.1
A8G0342	7.54	8.66	ABHD17A	beta hydrolase domain-containing protein 17A	XM_021478400.2
B11G0119	7.54	8.33	ZBTB41	Zinc finger and BTB domain-containing protein 41	XM_005155700.5
B10G0844	7.54	4.26	PIGP	Phosphatidylinositol N-acetylglucosaminyltransferase subunit P	
B3G1078	7.54	6.40	NATD1	Protein NATD1	
A6G0609	7.54	11.86	HES1	Transcription factor HES-1	NM_131079.2
A24G0581	7.54	13.23	FRI3	Ferritin, lower subunit	XM_021470123.2
B15G0618	7.54	2.83	LYRM9	LYR motif-containing protein 9	NM_001159983.2
A3G0311	7.53	4.49	METTL26B	Methyltransferase-like 26 B	NM_001004669.1
B5G1331	7.53	9.52	MTFP1	Mitochondrial fission process protein 1	
A11G0128	7.53	6.27	POLE4	DNA polymerase epsilon subunit 4	XM_068224260.1
A23G0205	7.53	8.15	EPB41L1	Band 4.1-like protein 1	XM_068216870.1
B3G1372	7.53	8.27	SEC14L1	SEC14-like protein 1	NM_201098.2
B19G0624	7.53	9.50	SI:DKEY-184P18.2	UPF0711 protein C18orf21 homolog	NM_001077280.1
B2G0690	7.53	9.63	RDH10B	Retinol dehydrogenase 10-B	NM_201331.1

A20G0809	7.53	7.15	TTC13	Tetratricopeptide repeat protein 13	NM_001130627.1
B9G0888	7.53	8.51	IL10RB	Interleukin-10 receptor subunit beta	NM_001083868.1
A4G0708	7.53	4.87	ZCRB1	Zinc finger CCHC-type and RNA-binding motif-containing protein 1	NM_001017589.2
A14G0796	7.52	7.98	RPS6KA6	Ribosomal protein S6 kinase alpha-6	NM_200073.1
B13G0731	7.52	3.68	MARVELD1	MARVEL domain-containing protein 1	NM_001037101.1
B20G0825	7.52	9.63	GPATCH2	G patch domain-containing protein 2	NM_001326693.2
B14G0019	7.52	6.90	AURKB	Aurora kinase B	NM_212566.2
B22G0531	7.52	14.97	RASSF1	Ras association domain-containing protein 1	NM_001004550.1
B20G0934	7.51	5.56			NM_199993.1
A19G0116	7.51	9.95	RNMT	mRNA cap guanine-N7 methyltransferase	NM_001045000.1
B22G0439	7.51	49.06	NLRC3	NLR family CARD domain-containing protein 3	XM_068216562.1
A8G0283	7.51	6.48	FAF1	FAS-associated factor 1	NM_212973.2
B1G0584	7.51	19.25	EDNRA	Endothelin-1 receptor	NM_001099445.2
B12G0248	7.51	6.54	ALDH18A1	Delta-1-pyrroline-5-carboxylate synthase	NM_001083546.3
B1G0738	7.51	8.50	DNAJA1	DnaJ homolog subfamily A member 1	NM_199662.1
B10G0028	7.51	6.17	NRG1	Pro-neuregulin-1, membrane-bound isoform	XM_009300713.4
A10G0862	7.51	9.97	PPIP5K2	Inositol hexakisphosphate and diphosphoinositol-pentakisphosphate kinase 2	XM_009305113.4
A18G0734	7.51	8.61	DCUN1D5	DCN1-like protein 5	XM_005173607.4
B9G0109	7.51	6.92	TANK	TRAF family member-associated NF-kappa-B activator	NM_001076600.1
A16G0516	7.51	6.69	LOC107723459	A0A673JWK6_9TELE Uncharacterized LOC107723459	
A11G0345	7.50	9.56	GPR25	Probable G-protein coupled receptor 25	XM_005155851.5
B23G0334	7.50	12.68	IKBKG	NF-kappa-B essential modulator	XM_005162070.5
B5G0685	7.50	6.73	CXCL11.6	C-X-C motif chemokine 11-6	
A12G0674	7.50	11.47	EXOC6	Exocyst complex component 6	XM_005156061.5
A21G0328	7.49	3.82	MED15	Mediator of RNA polymerase II transcription subunit 15	NM_213073.2
B19G0698	7.49	7.22	CF136	Uncharacterized protein C6orf136 homolog	NM_001082846.1
A1G0815	7.49	6.12	IFI44L	Interferon-induced protein 44-like	XM_005170873.5
A6G0224	7.49	4.11	MTX2	Metaxin-2	NM_212575.2
B11G0131	7.49	5.12	ACAP3	Arf-GAP with coiled-coil, ANK repeat and PH domain-containing protein 3	NM_001020571.1
A7G1195	7.49	4.76	TEX261	Protein TEX261	NM_001024426.1
A3G1355	7.49	7.47	SOX9-B	Transcription factor Sox-9-B	NM_131644.1
A25G0375	7.48	4.44	ACTR6	Actin-related protein 6	NM_199696.1
A16G0382	7.48	5.08	COQ3	Ubiquinone biosynthesis O-methyltransferase, mitochondrial	NM_001002620.2
B7G1108	7.48	3.42	A33	Zinc-binding protein A33	
B16G0841	7.48	4.33	POLR3C	DNA-directed RNA polymerase III subunit RPC3	NM_200232.1

A7G0624	7.48	7.41	BRD7	Bromodomain-containing protein 7	NM_213366.2
B15G0425	7.48	3.13	TIMM50	Mitochondrial import inner membrane translocase subunit TIM50	NM_200665.2
A13G0333	7.48	9.33	PRDM8	PR domain zinc finger protein 8	NM_131398.2
A4G1046	7.48	4.50	PHTF2	Protein PHTF2	NM_001002305.1
B9G0719	7.47	16.86	NAXD	ATP-dependent (S)-NAD(P)H-hydrate dehydratase	XM_005167460.5
B19G0080	7.47	11.90	CFAP20	Cilia- and flagella-associated protein 20	XM_005173723.5
B8G0620	7.47	5.23	APEX2	DNA-(apurinic or apyrimidinic site) endonuclease 2	NM_200146.1
B20G0100	7.47	3.88	RTASE	Probable RNA-directed DNA polymerase from transposon BS	XM_009304264.4
B4G0409	7.47	7.39	MEST	Mesoderm-specific transcript homolog protein	NM_001328532.1
A17G0472	7.47	14.44	UGP2	UTP--glucose-1-phosphate uridylyltransferase	XM_005158496.5
A7G1021	7.47	4.07	CPRAS1	Circularly permutated Ras protein 1	XM_001922896.7
B25G0485	7.47	5.11	ARMC10	Armadillo repeat-containing protein 10	XM_009297973.4
B8G0849	7.47	12.81	ATP2A2	endoplasmic reticulum calcium ATPase 2	NM_200965.1
B14G0479	7.46	19.70	PDLIM7	PDZ and LIM domain protein 7	NM_200840.2
A22G0640	7.46	9.74	CD276	CD276 antigen homolog	
B18G0841	7.46	7.51	SLC30A4	Zinc transporter 4	XR_011007148.1
B1G0054	7.46	10.09	SON	Protein SON	XM_689264.9
A17G0178	7.46	17.57	GSTZ1	Maleylacetoacetate isomerase	NM_001002481.2
A5G0929	7.46	1.20	NRARPB	Notch-regulated ankyrin repeat-containing protein B	NM_181496.5
A1G0618	7.46	6.09	MZT1	Mitotic-spindle organizing protein 1	NM_001302806.1
B3G0465	7.46	11.43			NM_131447.1
B15G0586	7.46	2.32	POLR1G	DNA-directed RNA polymerase I subunit RPA34	
A4G0937	7.45	5.51	WASL	Actin nucleation-promoting factor WASL	NM_001083006.1
A16G0520	7.45	8.66	ERP44	Endoplasmic reticulum resident protein 44	NM_200892.1
B7G0114	7.45	10.18	DHX15	ATP-dependent RNA helicase DHX15	NM_001115141.1
B20G0082	7.45	3.37	NDUFAF4	NADH dehydrogenase [ubiquinone] 1 alpha subcomplex assembly factor 4	
A1G0501	7.45	42.43	CANX	Calnexin	XM_005160003.5
B13G0650	7.45	10.33	JAG1B	Protein jagged-1b	XM_009307148.4
A8G0199	7.45	1.94	DENND2C	DENN domain-containing protein 2C	XM_001919051.8
A13G0595	7.45	4.01	TRIM8	E3 ubiquitin-protein ligase TRIM8	NM_001006079.1
A17G0428	7.45	11.65	CLIC4	Chloride intracellular channel protein 4	NM_201486.1
B16G0998	7.45	11.42	CCR2	C-C chemokine receptor type 2	XM_001344184.8
A5G0312	7.45	5.58	BTNL1	Butyrophilin-like protein 1	XM_005165234.5
A15G0731	7.45	6.45	SIRT2	NAD-dependent protein deacetylase sirtuin-2	
A5G1250	7.44	5.60			
B23G0344	7.44	22.68	BCL2L1	Bcl-2-like protein 1	NM_131807.1
B16G0337	7.44	9.45	POGZ	Pogo transposable element with ZNF domain	NM_001346224.1

B3G1388	7.44	6.15			
A14G0735	7.44	1.15	FHDC1	FH2 domain-containing protein 1	XM_009307807.4
A21G0853	7.44	11.82	RAPGEF6	Rap guanine nucleotide exchange factor 6	XM_017353005.2
A18G0388	7.44	17.97	CHD2	Chromodomain-helicase-DNA-binding protein 2	XM_009293623.4
A13G0635	7.44	3.39	SLC4A11	Solute carrier family 4 member 11	XM_689725.9
A7G0236	7.43	8.08	TMEM41B	Transmembrane protein 41B	NM_001079987.2
B17G0290	7.43	3.93	STON2	Stonin-2	NM_001033743.1
A20G0645	7.43	3.60	BLK	Tyrosine-protein kinase Blk	NM_001030220.1
B20G0303	7.43	14.76	SNX17	Sorting nexin-17	NM_001045157.3
A1G0729	7.43	22.13	APP	Amyloid-beta A4 protein	NM_131564.3
B14G0681	7.43	10.75	MAEA	E3 ubiquitin-protein transferase MAEA	NM_199549.2
A7G0388	7.43	5.73	ANKRD46	Ankyrin repeat domain-containing protein 46	XM_005166503.4
B21G0988	7.43	37.61	RAD23B	UV excision repair protein RAD23 homolog B	NM_200564.2
B7G0320	7.43	23.61	EIF5A	Eukaryotic translation initiation factor 5A-1	
A3G0522	7.43	5.86	GRAP	GRB2-related adapter protein	NM_001020573.1
B3G1508	7.43	7.99	LOC113040666	A0A6P6J5N2_CARAU uncharacterized protein LOC113040666	XM_009291379.3
B11G0273	7.43	8.03	MYOM2	Myomesin-2	NM_001114429.1
A2G0482	7.43	4.56	RREB1	Ras-responsive element-binding protein 1	XM_005163366.5
A16G1005	7.43	8.09	CNIH4	Protein cornichon homolog 4	XM_682723.10
B14G0779	7.42	3.74	A33	Zinc-binding protein A33	NM_001044961.1
B21G0517	7.42	26.17	CHRDL2	Chordin-like protein 2	NM_001130959.2
A22G0440	7.42	7.50	BTBD2	POZ domain-containing protein 2	NM_001045092.1
A13G0631	7.42	1.33	DNAAF9	Dynein axonemal assembly factor 9	NM_001128276.1
A1G0259	7.42	2.83	SLC44A1	Choline transporter-like protein 1	XM_002660581.7
A6G0626	7.42	16.82	CYFIP1	Cytoplasmic FMR1-interacting protein 1 homolog	NM_212759.1
A20G0193	7.42	5.91	MARK3	microtubule affinity-regulating kinase 3	XM_009294923.4
B13G0179	7.41	9.91	STPG4	Protein STPG4	
A9G0432	7.41	4.64	ARHGAP15	Rho GTPase-activating protein 15	NM_001128555.1
B9G0482	7.41	13.89	PARP9	Protein mono-ADP-ribosyltransferase PARP9	XM_001340131.9
B22G0747	7.41	7.82	BTBD2	POZ domain-containing protein 2	NM_001045092.1
A6G0812	7.41	11.18	PBRM1	Protein polybromo-1	XM_068221698.1
A24G0472	7.41	5.58	TIMM21	Mitochondrial import inner membrane translocase subunit Tim21	NM_001386503.1
B6G1016	7.41	7.73	ARHGAP9	Rho GTPase-activating protein 9	XM_005172127.5
B3G0449	7.41	8.04	SLC27A1	Long-chain fatty acid transport protein 1	NM_001013537.1
A23G0185	7.41	4.28	EXOSC10	Exosome component 10	NM_201089.2
A9G0099	7.41	11.74	SPC25	Kinetochore protein Spc25	NM_001123057.1
A8G0578	7.40	3.31	LOC113072104	A0A6P6MWS9_CARAU uncharacterized protein LOC113072104 isoform X2	

A13G0207	7.40	10.70	CDKN3	Cyclin-dependent kinase inhibitor 3	XM_005156625.2
B22G0134	7.40	4.97	LONP1	Lon protease homolog, mitochondrial	XM_686620.10
B22G0532	7.40	5.41	TUSC2	Tumor suppressor candidate 2	NM_001002402.2
B5G0096	7.40	12.08	MAPKAP1	Target of rapamycin complex 2 subunit MAPKAP1	XM_003198574.6
A5G0165	7.40	5.59	SPRING	SREBP regulating gene protein	NM_001020737.1
B12G0457	7.40	9.28	CSNK1E	Casein kinase I isoform epsilon	NM_212747.1
A1G0905	7.40	17.61	SARAF	Store-operated calcium entry-associated regulatory factor	NM_001076589.1
A11G0481	7.40	11.79	CACNA1A	Q-type calcium channel subunit alpha-1A	XM_005169291.5
A5G0663	7.40	4.60	RHOG	Rho-related GTP-binding protein RhoG	NM_199692.2
B18G0726	7.39	7.11	C2CD5	C2 domain-containing protein 5	XM_068214739.1
B14G0722	7.39	13.56	LIMCH1	LIM and calponin homology domains-containing protein 1	XM_068213319.1
B1G1021	7.39	1.96	GIMAP8	GTPase IMAP family member 8	XM_068222067.1
A7G0231	7.39	7.06	BOLA3	BolA-like protein 3	
A8G0348	7.39	6.48	EDEM3	ER degradation-enhancing alpha-mannosidase-like protein 3	XM_005166932.5
A19G0131	7.39	10.43	PARP10	Protein mono-ADP-ribosyltransferase PARP10	XM_021468293.2
A13G0882	7.39	4.31			XM_001920829.9
B2G1252	7.39	5.32	WRNIP1	ATPase WRNIP1	NM_212920.1
A11G0029	7.39	4.81	CANT1	Soluble calcium-activated nucleotidase 1	NM_001037119.2
A14G0492	7.39	4.54	EXOSC3	Exosome complex component RRP40	
B6G0463	7.39	4.75	PKN2	threonine-protein kinase N2	NM_001290179.1
A12G0172	7.39	8.14	SEC24C	Protein transport protein Sec24C	XM_005156360.5
A13G0155	7.38	17.57	PYROXD2	Pyridine nucleotide-disulfide oxidoreductase domain-containing protein 2	XM_001921555.8
B12G0384	7.38	3.35	MPP7	MAGUK p55 subfamily member 7	XM_009306585.4
A1G0816	7.38	5.45	IFI44L	Interferon-induced protein 44-like	
B14G0476	7.38	6.29	FAF2	FAS-associated factor 2	NM_001128680.1
B10G0550	7.38	1.60	ADGRG2	Adhesion G-protein coupled receptor G2	
B25G0006	7.37	5.23	CHMP1A	Charged multivesicular body protein 1a	NM_200563.3
B21G0069	7.37	19.13	HSPA4	Heat shock 70 kDa protein 4	NM_214716.1
A19G0178	7.37	6.24	UMAD1	UBAP1-MVB12-associated (UMA)-domain containing protein 1	NM_001128373.1
A15G0457	7.37	5.36	SAE1	SUMO-activating enzyme subunit 1	NM_001002058.1
A25G0419	7.37	9.20	WNK1	threonine-protein kinase WNK1	XM_002666846.7
A22G0672	7.37	5.47	TSC22D2	TSC22 domain family protein 2	XM_009296501.4
B7G0776	7.37	6.32	OGFOD1	Prolyl 3-hydroxylase OGFOD1	NM_199689.3
B4G0333	7.37	2.64	HELB	DNA helicase B	XM_068220753.1
B19G0060	7.37	10.78	ENDOD1	Endonuclease domain-containing 1 protein	
B1G0287	7.37	2.37	ACSL1	Long-chain-fatty-acid--CoA ligase 1	NM_001031837.1
A3G0463	7.37	10.49	ZC3H7A	Zinc finger CCCH domain-containing protein 7A	NM_213434.1

B14G0163	7.36	7.27	PDGFRL	Platelet-derived growth factor receptor-like protein	NM_200314.2
A17G0195	7.36	2.63	CAPN1	Calpain-1 catalytic subunit	NM_001089383.1
A21G0593	7.36	4.97			
B7G0894	7.36	23.17	CD048	Neuropeptide-like protein C4orf48 homolog	NM_001386636.1
B9G0320	7.36	22.45	MAO	Amine oxidase [flavin-containing]	NM_212827.3
A7G0714	7.36	11.50	NAP1L4	Nucleosome assembly protein 1-like 4	NM_001007453.1
B3G0697	7.36	4.77	DCTN5	Dynactin subunit 5	NM_001002497.1
B20G0864	7.36	2.44	KIF13B	Kinesin-like protein KIF13B	XM_002667519.7
B9G0637	7.36	4.74	NUP35	Nucleoporin NUP35	XM_009304508.4
A7G1053	7.36	1.74	NECTIN4	Nectin-4	XM_683919.9
B11G0470	7.35	5.33	EFCC1	EF-hand and coiled-coil domain-containing protein 1	NM_001017727.1
A19G0934	7.35	4.71	LOC113062470	A0A6P6LXB7_CARAU uncharacterized protein LOC113062470 isoform X2	
B19G0295	7.35	20.51	USF2	Upstream stimulatory factor 2	NM_001122785.1
B8G0308	7.35	7.62	FAHD2	Fumarylacetoacetate hydrolase domain-containing protein 2	NM_212918.1
A5G0395	7.35	13.69	ATP6V1A	V-type proton ATPase catalytic subunit A	NM_201135.2
B22G0321	7.35	11.63	LOC113073303	A0A6P6N0Y7_CARAU uncharacterized protein LOC113073303 isoform X2	
A11G0274	7.35	15.68	MAGI1	Membrane-associated guanylate kinase, WW and PDZ domain-containing protein 1	XM_009305864.4
A7G0635	7.35	4.05	IRX3	Iroquois-class homeodomain protein irx-3	NM_001320125.1
A11G0367	7.34	8.21	TOP1	DNA topoisomerase 1	NM_001014295.2
A5G1055	7.34	0.52	HEPACAM	Hepatocyte cell adhesion molecule	NM_001386850.1
B2G0173	7.34	8.97	CACTIN	Cactin	XM_005168431.5
A21G0139	7.34	11.80	CCNG2	Cyclin-G2	NM_213172.1
A15G0416	7.34	6.10	SUSD4	Sushi domain-containing protein 4	NM_001080000.2
A1G0088	7.34	15.14	NLRC3	NLR family CARD domain-containing protein 3	NM_001328438.1
B8G0342	7.34	4.12	LZTS1	Leucine zipper putative tumor suppressor 1	XM_692658.10
B7G1313	7.33	5.07	RELA	Transcription factor p65	XM_068222214.1
A13G0841	7.33	8.84	BMP5	Bone morphogenetic protein 5	XM_005169554.5
A16G0439	7.33	7.42	DGAT1	Diacylglycerol O-acyltransferase 1	NM_001002458.1
B6G0193	7.33	12.35	VPS16	Vacuolar protein sorting-associated protein 16 homolog	NM_001098189.1
B13G0338	7.33	10.04	SIX2	Homeobox protein SIX2	XM_021480607.2
B2G0819	7.33	2.39	RPP40	Ribonuclease P protein subunit p40	NM_001002148.1
A24G0456	7.33	9.12	CUL1	Cullin-1	XM_021470126.2
B8G0751	7.33	27.52	PLK2	threonine-protein kinase PLK2	NM_001099245.1
A13G0808	7.33	23.23	RNASET2	Ribonuclease T2	NM_001004108.2
A13G0612	7.33	5.56	ADH1	Alcohol dehydrogenase 1	NM_201036.1
B8G0724	7.33	1.74	ELOVL4	Elongation of very long chain fatty acids protein 4	NM_001024438.2
B8G0696	7.33	15.22	PLIN2	Perilipin-2	NM_001386314.1
B9G0433	7.33	18.49	LMO7	LIM domain only protein 7	XM_068223383.1
B8G0087	7.32	7.95	DPP9	Dipeptidyl peptidase 9	XM_005172485.4
A8G0584	7.32	0.47	ATOH1	Transcription factor Atoh1	NM_131091.2

B18G0513	7.32	2.96	INTS14	Integrator complex subunit 14	XM_005159052.5
A16G0429	7.32	8.47	PHF20L1	PHD finger protein 20-like protein 1	XM_021466935.2
A21G0090	7.32	6.70	UCK1	Uridine-cytidine kinase 1	NM_001004666.1
B23G0974	7.32	15.44	TRAPPC1	Trafficking protein particle complex subunit 1	NM_001430947.1
A25G0438	7.32	8.11	ALKBH3	Alpha-ketoglutarate-dependent dioxygenase alkB homolog 3	NM_001003511.2
A14G0624	7.32	10.56	DOCK2	Dedicator of cytokinesis protein 2	XM_002664252.6
A8G0636	7.32	0.59	HTR1AA	5-hydroxytryptamine receptor 1A-alpha	NM_001123321.1
B9G0213	7.32	6.79	NAB1	NGFI-A-binding protein 1	NM_001123273.1
A21G0516	7.32	1.45	BICDL1	BICD family-like cargo adapter 1	NM_001365668.1
B7G0658	7.31	1.64	LOC107663954	A0A671T3J4_9TELE Mucin-15-like	XM_021471992.2
B17G0715	7.31	2.56	NENF	Neudesin	NM_001037704.2
B8G0570	7.31	6.56	SRPK1	SRSF protein kinase 1	XM_017357280.3
B23G0904	7.31	3.73	ZNHIT6	D snoRNA protein 1	
B7G0992	7.31	10.68	CLPX	ATP-dependent Clp protease ATP-binding subunit clpX-like, mitochondrial	XM_021477471.2
A3G0464	7.31	1.26	PA2V	Phospholipase A2	
B21G0620	7.31	7.10	BRD3	Bromodomain-containing protein 3	XM_005161151.5
B12G0466	7.31	0.89	LOC107584106	A0A672JUL1_SINGR Tumor necrosis factor receptor superfamily member 17-like	XM_068219305.1
A20G0134	7.31	5.47			
A22G0501	7.31	2.21	C1ORF53	Uncharacterized protein C1orf53	
B3G0603	7.31	8.80	SHMT1	Serine hydroxymethyltransferase, cytosolic	NM_201046.1
A1G0214	7.31	8.05	RFX1	MHC class II regulatory factor RFX1	XM_021477396.2
A22G0499	7.30	6.91	NEK7	threonine-protein kinase Nek7	NM_001003617.1
A2G0655	7.30	8.67	STARD3NL	STARD3 N-terminal-like protein	NM_001002320.1
A22G0119	7.30	7.71	C19ORF44	Uncharacterized protein C19orf44	XM_001337197.9
A20G0101	7.30	0.51	RIOX1	Ribosomal oxygenase 1	NM_001089388.1
B10G0422	7.30	7.01	WDR74	WD repeat-containing protein 74	NM_001076563.1
B19G0556	7.30	18.76	ACDH-6	Probable acyl-CoA dehydrogenase 6	NM_212935.1
B18G0485	7.30	2.26	IGHMBP2	DNA-binding protein SMUBP-2	NM_001044900.1
B19G0216	7.30	9.25	PRCC	Proline-rich protein PRCC	NM_001126440.1
B15G0207	7.30	5.38	ANKRD49	Ankyrin repeat domain-containing protein 49	NM_001017584.2
A1G0990	7.29	6.80	KLF3	Krueppel-like factor 3	NM_131859.3
A23G0187	7.29	33.80	NDRG3	Protein NDRG3	NM_199797.1
B24G0691	7.29	26.22	PFKP	ATP-dependent 6-phosphofructokinase, platelet type	XM_068217141.1
B20G0314	7.29	6.61	PPP2R5E	threonine-protein phosphatase 2A 56 kDa regulatory subunit epsilon isoform	NM_194415.2
B19G0809	7.29	10.56	SEMA4A	Semaphorin-4A	XM_694864.8
B11G0207	7.29	5.71	ELOVL1	Elongation of very long chain fatty acids protein 1	NM_001005206.1

A22G0220	7.29	8.32	NISCH	Nischarin	XR_659529.4
A21G0027	7.28	7.69	TMEM38B	Trimeric intracellular cation channel type B	NM_200176.1
B21G0942	7.28	6.14	TNFRSF14	Tumor necrosis factor receptor superfamily member 14	NM_001430990.1
A1G1036	7.28	2.10	MED28	Mediator of RNA polymerase II transcription subunit 28	NM_001007770.1
B6G0606	7.28	8.60	OPA1	Dynamin-like 120 kDa protein, mitochondrial	XM_009302514.4
B25G0601	7.28	9.04	CCDC113	Coiled-coil domain-containing protein 113	NM_001006061.1
B7G0937	7.28	4.48	CCNB3	mitotic-specific cyclin-B3	NM_001076719.1
A9G0323	7.28	4.71	DNAJB11	DnaJ homolog subfamily B member 11	NM_198821.1
B15G0757	7.28	15.22	IGFLR1	IGF-like family receptor 1	NM_001386215.1
A15G0673	7.28	3.54	FKRP	Ribitol 5-phosphate transferase FKRP	NM_001042689.1
B8G0367	7.28	0.79	HA2J	H-2 class II histocompatibility antigen, I-E alpha chain (Fragment)	XM_068223045.1
A6G0376	7.28	11.60	POLG2	DNA polymerase subunit gamma-2, mitochondrial	XM_001922324.8
A16G0235	7.28	14.61	CASPA	Caspase a	NM_131505.2
A3G0695	7.27	3.94	THOC6	THO complex subunit 6 homolog	NM_001006095.1
A11G0244	7.27	15.69	SLC25A26	S-adenosylmethionine mitochondrial carrier protein	NM_212764.1
B1G0512	7.27	2.25	DLX2B	Homeobox protein Dlx2b	NM_131297.2
A5G0346	7.27	14.51	ATRX	Transcriptional regulator ATRX	NM_001423350.1
B5G0465	7.27	5.92	POLR2J	DNA-directed RNA polymerase II subunit RPB11	NM_001024400.1
B19G0476	7.27	17.29	ELOA	Elongin-A	NM_213455.1
B2G0395	7.27	16.58			
A12G0198	7.27	19.03	SOCS3	Suppressor of cytokine signaling 3	NM_213304.1
A7G0821	7.27	9.26	EXT2	Exostosin-2	NM_001008400.2
B15G0274	7.27	1.04	TMPRSS4	Transmembrane protease serine 4	XM_005157490.5
A8G0002	7.27	4.81	PYM1B	Partner of Y14 and mago B	NM_200594.2
A13G0147	7.27	7.24	ST3GAL5	Lactosylceramide alpha-2,3-sialyltransferase	NM_199907.1
A25G0060	7.26	4.72	THOC5	THO complex subunit 5 homolog	NM_212692.1
A3G0293	7.26	6.28	DHPS	Deoxyhypusine synthase	NM_213222.2
B6G0399	7.26	4.80	CBX4	E3 SUMO-protein ligase CBX4	
B17G0143	7.26	11.54	SYNE1	Nesprin-1	XM_068214520.1
A15G0351	7.26	4.80	TSKU	Tsukushi	NM_001319185.1
B5G0649	7.26	11.35	LOC113049216	A0A6P6K5P1_CARAU uncharacterized protein LOC113049216	
B16G0039	7.26	3.72	TMEM222	Transmembrane protein 222	NM_001013316.3
B3G0844	7.26	5.22	BCL2L12	Bcl-2-like protein 12	XM_068218111.1
A22G0397	7.25	1.82	GZME	Granzyme E	
B3G0404	7.25	4.29	PRKACA	cAMP-dependent protein kinase catalytic subunit alpha	NM_001082840.1
B19G0922	7.25	13.16	TOP2B	DNA topoisomerase 2-beta	NM_001045191.2
B3G0045	7.25	3.45	LOC113046763	A0A6P6JUE8_CARAU gastrula zinc finger protein XlCGF57.1-like isoform X1	XM_017354882.3
A12G0596	7.25	10.71	FKBP10	Peptidyl-prolyl cis-trans isomerase FKBP10	NM_001128666.1

A10G0661	7.25	8.51	STARD7	StAR-related lipid transfer protein 7, mitochondrial	XM_687902.9
A22G0410	7.25	4.02	BDMD1	Duodenase-1	XM_021474472.2
B22G1030	7.24	5.84	NLRC3	NLR family CARD domain-containing protein 3	XM_021478497.2
B20G0283	7.24	3.40	VIPAS39	Spermatogenesis-defective protein 39 homolog	NM_001001836.1
B20G0919	7.24	1.67	PAK6	threonine-protein kinase PAK 6	NM_001162487.1
A5G1322	7.24	6.37	MATR3	Matrin-3	NM_001077760.2
A23G0296	7.24	12.08	HSD17B10	3-hydroxyacyl-CoA dehydrogenase type-2	NM_001006098.1
B5G0100	7.24	1.84	TNC	Tenascin	XM_068221397.1
A3G0199	7.24	11.38	MIF4GDB	MIF4G domain-containing protein B	NM_001013284.1
A5G1069	7.24	0.53	IFNLR1	Interferon lambda receptor 1	
B18G0499	7.24	1.18	C15ORF61	Uncharacterized protein C15orf61	NM_001082953.1
A21G0207	7.24	0.19	AGXT2	Alanine--glyoxylate aminotransferase 2, mitochondrial	NM_001033750.1
B14G0504	7.24	2.76	TSTD2	rhodanese-like domain-containing protein 2	
B23G0787	7.24	2.37	GPSM2	G-protein-signaling modulator 2	XM_068216739.1
A17G0018	7.24	5.80	NEK9	threonine-protein kinase Nek9	XM_021467511.2
B11G0773	7.24	9.95	PLAAT1	Phospholipase A and acyltransferase 1	NM_001114412.1
A12G0214	7.24	5.74	MBTD1	MBT domain-containing protein 1	XM_001919980.7
B23G0505	7.24	5.17	F26	fructose-2,6-bisphosphatase	XM_005162149.5
A19G0038	7.24	9.52	PPT2-A	Lysosomal thioesterase PPT2-A	NM_001109859.2
A5G0728	7.23	6.83	SLC12A9	Solute carrier family 12 member 9	NM_001128548.1
A5G0921	7.23	11.77	SECISBP2	Selenocysteine insertion sequence-binding protein 2	XM_068221567.1
A25G0022	7.23	1.03	ADAMTS9	A disintegrin and metalloproteinase with thrombospondin motifs 9	
B16G0135	7.23	14.98	LOC107756895	A0A673N1J6_9TELE Uncharacterized LOC107756895	NM_001386340.1
B17G0426	7.23	13.01	UGP2	UTP--glucose-1-phosphate uridylyltransferase	XM_005158496.5
A18G0452	7.23	8.91	DDB2	DNA damage-binding protein 2	XM_005159034.5
B17G0532	7.23	17.07	HECTD1	E3 ubiquitin-protein ligase HECTD1	XM_009293117.4
B23G0588	7.23	4.84	CTNNBIP1	Beta-catenin-interacting protein 1	NM_131594.1
A3G0428	7.23	7.81	MRTFB	Myocardin-related transcription factor B	XM_009299741.4
B17G0103	7.22	13.15	PLAAT2	Phospholipase A and acyltransferase 2	NM_001128782.1
A2G0187	7.22	5.68	KLF13	Krueppel-like factor 13	NM_001077329.2
A11G0308	7.22	7.42	TMEM183A	Transmembrane protein 183A	XM_005155802.5
A3G0857	7.22	6.72	PPP4CA	threonine-protein phosphatase 4 catalytic subunit A	NM_001110414.1
B7G1198	7.22	1.53	SHTN1	Shootin-1	XM_021477700.2
B13G0233	7.22	18.73	ALOX5	Polyunsaturated fatty acid 5-lipoxygenase	NM_212581.1
B2G0574	7.22	13.57	G5714_001953	A0A7J6DDU2_9TELE Uncharacterized protein	
B11G0161	7.22	5.05	ARID3A	AT-rich interactive domain-containing protein 3A	

A12G0392	7.22	6.19	BAMBI	BMP and activin membrane-bound inhibitor homolog	NM_131784.2
A17G0263	7.21	7.92	FUT8	Alpha-(1,6)-fucosyltransferase	NM_001361235.1
A3G0699	7.21	3.27	LOC113045617	A0A6P6JQ98_CARAU mucin-21-like	
A20G0444	7.21	2.02	KLF11	Krueppel-like factor 11	NM_001044941.3
B14G0199	7.21	10.89	PHF6	PHD finger protein 6	XM_068213168.1
B20G0526	7.21	4.24	ACAT2	Acetyl-CoA acetyltransferase, cytosolic	NM_131370.2
A6G0198	7.21	2.36	TMEM237B	Transmembrane protein 237B	NM_001004636.1
B22G1073	7.21	5.23	TSC22D2	TSC22 domain family protein 2	XM_009296501.4
B24G0507	7.21	10.99	CUL1	Cullin-1	NM_213495.1
A7G0621	7.20	5.44	HEATR3	HEAT repeat-containing protein 3	NM_200889.1
A1G0652	7.20	40.78	GPM6B	Neuronal membrane glycoprotein M6-b	NM_203427.2
B6G0481	7.20	4.67	SCLY	Selenocysteine lyase	NM_001110383.1
B24G0368	7.20	6.89	SH2D1B	SH2 domain-containing protein 1B	
B9G0178	7.20	17.11	FRZB	Secreted frizzled-related protein 3	NM_130943.2
A12G0326	7.20	7.68	NBR1	Next to BRCA1 gene 1 protein	NM_001305595.1
A16G0576	7.20	8.52	DTNBP1A	Dysbindin-A	XM_005158101.4
A13G0051	7.20	6.22	ARID4B	AT-rich interactive domain-containing protein 4B	NM_001327835.1
A5G1014	7.20	7.20	ORAI2	Protein orai-2	NM_001166447.1
A22G0433	7.20	6.46	LOC113105120	A0A6P6PMR5_CARAU uncharacterized protein LOC113094532 isoform X1	
B17G0308	7.20	11.92	GALC	Galactocerebrosidase	NM_213111.2
B23G0483	7.19	6.91	FAM3A	Protein FAM3A	NM_200800.1
B3G0383	7.19	7.96	SUN1	SUN domain-containing protein 1	NM_001018135.2
B8G0369	7.19	2.48	DPX16_16742	A0A3N0YSE0_ANAGA THAP-type domain-containing protein	
A2G0471	7.19	12.17	NFI1	CTD small phosphatase-like protein	XM_005163361.5
A3G0321	7.19	3.30	CRB3	Protein crumbs homolog 3	NM_001045322.1
B16G1054	7.19	5.68	MED18	Mediator of RNA polymerase II transcription subunit 18	NM_200335.1
B6G0873	7.19	9.42	RAB22A	Ras-related protein Rab-22A	NM_205719.2
B25G0794	7.19	7.80	THOC5	THO complex subunit 5 homolog	NM_212692.1
B2G1212	7.19	8.12	EIF2B5	Translation initiation factor eIF-2B subunit epsilon	NM_001113597.1
A13G0128	7.19	9.14	BTAF1	TATA-binding protein-associated factor 172	XM_068224895.1
B11G0301	7.19	5.24	THAP4	Peroxynitrite isomerase THAP4	XM_001920828.8
B12G0639	7.19	6.70	EEF2K	Eukaryotic elongation factor 2 kinase	XM_005156067.5
B10G0492	7.18	7.79	RABGEF1	GTP exchange factor	XM_009305364.3
B7G1028	7.18	5.27	ZDHHC13	Palmitoyltransferase ZDHHC13	NM_001008650.3
B4G0287	7.18	4.35	TMTC2	Protein O-mannosyl-transferase TMTC2	NM_001130603.1
B25G0223	7.18	53.45	LDHA	L-lactate dehydrogenase A chain	NM_131246.1
B1G0928	7.18	5.85	PUS10	tRNA pseudouridine synthase Pus10	NM_001111189.1
A12G0692	7.18	2.90	POLR3D	DNA-directed RNA polymerase III subunit RPC4	NM_001109828.1
A15G0112	7.18	1.30	SGPP2	Sphingosine-1-phosphate phosphatase 2	NM_001386286.1
A8G0204	7.18	7.89	TASOR2	Protein TASOR 2	XM_005166734.5

B15G0602	7.18	3.37	TSKU	Tsukushi	NM_199733.2
B10G0861	7.18	3.85	ANKLE2	Ankyrin repeat and LEM domain-containing protein 2	XM_068223878.1
B15G0101	7.17	17.70	ATP1B3	potassium-transporting ATPase subunit beta-3	NM_131670.1
A11G0484	7.17	2.90			NM_001328559.1
A10G0718	7.17	15.34	GOLPH3	Golgi phosphoprotein 3	XM_021472219.2
B20G0656	7.17	14.46	HIVEP2	Human immunodeficiency virus type I enhancer-binding protein 2 homolog	XM_005160735.5
A7G1126	7.17	2.36	MRPL46	39S ribosomal protein L46, mitochondrial	NM_001002518.1
B22G1052	7.17	8.55	PPP1R2	Protein phosphatase inhibitor 2	NM_205668.1
B9G0099	7.17	6.21	XRCC5	X-ray repair cross-complementing protein 5	NM_001017360.2
A22G0014	7.17	5.08	FBXO42	F-box only protein 42	NM_212739.1
A8G0966	7.17	8.42	APEH	Acylamino-acid-releasing enzyme	NM_198869.2
A5G0506	7.17	2.17	P2RY4	P2Y purinoceptor 4	XM_005171951.5
A9G0181	7.17	2.50	WDR75	WD repeat-containing protein 75	NM_200090.1
B9G0857	7.17	6.36	CDCA7	Cell division cycle-associated protein 7	NM_001017574.2
B10G0473	7.16	13.50	VPS26BL	Vacuolar protein sorting-associated protein 26B-like	NM_001002415.2
A16G0451	7.16	2.62	G5714_015973	A0A7J6C771_9TELE Uncharacterized protein	
B11G0364	7.16	7.42	FOXP4	Forkhead box protein P4	NM_001313699.1
B16G0284	7.16	5.60	PTPN23	Tyrosine-protein phosphatase non-receptor type 23	NM_001202507.1
A4G0570	7.16	70.20	FGL2	Fibroleukin	NM_001025539.2
B5G0859	7.16	2.87	ARHGEF28	Rho guanine nucleotide exchange factor 28	XM_021476529.2
B23G0172	7.16	4.57	PHLDA3	Pleckstrin homology-like domain family A member 3	NM_001002455.1
A2G0173	7.16	3.90	SMC6	Structural maintenance of chromosomes protein 6	NM_001128334.1
B4G0520	7.16	5.47	GSAP	Gamma-secretase-activating protein	XM_021475259.2
A12G0630	7.16	11.14	GET4	Golgi to ER traffic protein 4 homolog	NM_001199907.1
B7G0853	7.16	7.39	MTM1	Myotubularin	NM_001037684.2
B22G0202	7.15	2.21	DNASE1L3	Deoxyribonuclease gamma	
B19G0152	7.15	8.00	CHD4	Chromodomain-helicase-DNA-binding protein 4	XM_021468085.2
B19G0622	7.15	4.81	GALNT12	Polypeptide N-acetylgalactosaminyltransferase 12	XM_683102.6
B2G0764	7.15	4.14	PPP2R3A	threonine-protein phosphatase 2A regulatory subunit B'' subunit alpha	XM_009298556.4
A8G0754	7.15	16.18	KDM5C	Lysine-specific demethylase 5C	NM_001123234.1
A2G1039	7.15	5.58	PITRM1	Presequence protease, mitochondrial	NM_213487.1
B6G0877	7.15	11.75	NPEPL1	Probable aminopeptidase NPEPL1	NM_001017688.1
A5G0519	7.15	14.69	USF1	Upstream stimulatory factor 1	NM_213194.1
A23G0577	7.15	3.85	CABLES2	CDK5 and ABL1 enzyme substrate 2	XM_021473725.2
B25G0549	7.15	5.05	ATP6V1E1	V-type proton ATPase subunit E 1	NM_001365440.1

A2G0496	7.15	6.22	SLC12A7	Solute carrier family 12 member 7	XM_005171249.5
A2G0821	7.15	8.08	NADK	NAD kinase	NM_001080002.1
B2G0207	7.15	5.62	PITRM1	Presequence protease, mitochondrial	NM_213487.1
B25G0883	7.15	2.22	DDB_G0292642	Uncharacterized protein DDB_G0292642	
A19G0144	7.15	23.76	SNAPIN	SNARE-associated protein Snapin	NM_001045148.1
A22G0334	7.15	5.37	SLC30A7	Zinc transporter 7	NM_001100086.1
B20G0611	7.14	4.61	ZMCM3	Zygotic DNA replication licensing factor mcm3	NM_212567.2
A6G0169	7.14	9.39	UBE2G2	Ubiquitin-conjugating enzyme E2 G2	NM_001017767.1
B8G0286	7.14	3.46	PQBP1	Polyglutamine-binding protein 1	NM_001002435.1
A20G0188	7.14	16.29	MMUT	Methylmalonyl-CoA mutase, mitochondrial	NM_001099226.1
A19G0121	7.14	11.74	PTPN2	Tyrosine-protein phosphatase non-receptor type 2	NM_212654.1
A8G0158	7.14	8.19	CXXC1	CXXC-type zinc finger protein 1	NM_200599.1
A19G0441	7.14	8.45	SDC3	Syndecan-3	XM_001919721.8
B16G0711	7.14	7.48	CCPG1	Cell cycle progression protein 1	XM_017351348.3
B2G0821	7.14	6.49	LYRM4	LYR motif-containing protein 4	NM_001164241.1
B3G0894	7.14	2.65	BAX	Apoptosis regulator BAX	
B25G0701	7.13	5.08	SAAL1	Protein saal1	NM_200135.2
B15G0755	7.13	3.45	ZBTB16A	Zinc finger and BTB domain-containing protein 16-A	XM_068213779.1
A20G0638	7.13	4.65	ATRAID	All-trans retinoic acid-induced differentiation factor	
A9G0187	7.13	2.39	OSGEPL1	tRNA N6-adenosine threonylcarbamoyltransferase, mitochondrial	NM_001005301.1
A16G0396	7.13	8.63	KPNA5	Importin subunit alpha-6	NM_001018153.1
A19G0325	7.13	4.74	RP9	Retinitis pigmentosa 9 protein homolog	XM_009294316.4
A19G0781	7.13	10.83	CHD4	Chromodomain-helicase-DNA-binding protein 4	XM_021468085.2
B17G0529	7.12	3.45	G5714_017084	A0A7J6C5K4_9TELE F-box domain-containing protein	
A15G0015	7.12	10.70	MAP4K5	Mitogen-activated protein kinase kinase kinase kinase 5	XM_005173433.3
A14G0662	7.12	7.76	KDM2A	Lysine-specific demethylase 2A	XM_001339761.8
B19G0460	7.12	3.32	UTP23	rRNA-processing protein UTP23 homolog	XM_068215127.1
B18G0069	7.12	75.07	BLVRB	Flavin reductase (NADPH)	NM_001002686.2
A4G0658	7.12	9.59	TCP11L2	T-complex protein 11-like protein 2	XM_005164731.5
B3G1305	7.12	9.94	METTL26	Methyltransferase-like 26	
A7G0633	7.12	6.20	AKTIP	AKT-interacting protein	NR_135206.1
B23G0325	7.12	7.20	UBA3	NEDD8-activating enzyme E1 catalytic subunit	
A16G1046	7.12	7.86	TARS1	Threonine--tRNA ligase 1, cytoplasmic	XM_021466791.2
A3G0936	7.12	7.53	ATXN2L	Ataxin-2-like protein	NM_212684.2
B5G1152	7.12	6.31	FAM76B	Protein FAM76B	NM_199932.1
A13G0574	7.12	5.34	PPM1B	Protein phosphatase 1B	NM_001030276.2
B2G1017	7.11	3.81	ERFE	Erythroferrone	
B1G0229	7.11	9.83	SARAF	Store-operated calcium entry-associated regulatory factor	NM_001076589.1
A4G0499	7.11	7.09	TMCC3	Transmembrane and coiled-coil domain protein 3	NM_001044813.2
B22G0864	7.11	1.58	CAPN2	Calpain-2 catalytic subunit	NM_001244080.1

B6G0989	7.11	2.22	EYA2	Eyes absent homolog 2	NM_001004558.1
B6G0223	7.11	8.93	LIMA1	LIM domain and actin-binding protein 1	XM_009302196.4
B22G0413	7.11	7.14	LOC113078163	A0A6P6NAM5_CARAU uncharacterized protein LOC113078163	XM_021469541.2
B12G0420	7.11	3.79	SNF8	Vacuolar-sorting protein SNF8	NM_001007412.1
B10G0183	7.11	4.46	TCTN1	Tectonic-1	XM_009305137.4
A16G0445	7.11	3.69	ZGC:113036	Uncharacterized protein C18orf19 homolog B	NM_001013340.1
B3G0470	7.11	15.88	STXBP2	Syntaxin-binding protein 2	NM_213289.1
A15G0012	7.11	10.25	SAMD4B	Protein Smaug homolog 2	XM_021466521.2
A4G0656	7.11	2.97	GOLT1B	Vesicle transport protein GOT1B	
B8G0317	7.10	8.95	TBC1D10A	TBC1 domain family member 10A	XM_009304172.3
B15G0283	7.10	4.22	TRAPPC6B	Trafficking protein particle complex subunit 6b	NM_001305574.1
B6G0528	7.10	25.94	DLG1	Disks large homolog 1	XM_005165899.5
A20G0042	7.10	46.43	HSP90AA1	Heat shock protein HSP 90-alpha	NM_001045073.1
A20G0438	7.10	7.39	E2F6	Transcription factor E2F6	XM_005160725.5
A23G0188	7.10	14.92	PHF20	PHD finger protein 20	XM_005161993.5
A2G0446	7.10	10.51	STK25	threonine-protein kinase 25	NM_213308.1
B21G0895	7.10	24.64	ADD1	Alpha-adducin	XM_021468841.2
A7G0249	7.10	6.61	FGF4-B	Fibroblast growth factor 4B	NM_131635.3
A5G1351	7.10	3.54	KAT5	Histone acetyltransferase KAT5	NM_001013309.3
A12G0783	7.09	6.39	WNK4	threonine-protein kinase WNK4	XM_021480207.2
A13G0122	7.09	3.71	MLH1	DNA mismatch repair protein Mlh1	NM_001045456.1
B12G0153	7.09	7.19	NPLOC4	Nuclear protein localization protein 4 homolog	NM_199807.1
B23G0193	7.09	9.51	PRPF6	Pre-mRNA-processing factor 6	NM_212655.2
A9G0078	7.09	9.09	TANK	TRAF family member-associated NF-kappa-B activator	NM_001076600.1
A6G0468	7.09	3.64	PKN2	threonine-protein kinase N2	NM_001290179.1
A25G0370	7.09	0.63	GLS	Glutaminase kidney isoform, mitochondrial	XM_009297984.4
B17G0834	7.09	3.54	TFB1M	Dimethyladenosine transferase 1, mitochondrial	XM_005169880.5
B16G0573	7.09	8.21	GALNT1	Polypeptide N-acetylgalactosaminyltransferase 1	XM_682380.9
B25G0154	7.08	1.69	IGKV1-27	Immunoglobulin kappa variable 1-27	XM_017354192.3
A2G1069	7.08	1.95	SEMA4E	Semaphorin-4E	NM_001083865.1
B4G0874	7.08	28.14	TCB2	Transposable element Tcb2 transposase	XM_068218059.1
A6G0349	7.08	12.03	ZXDC	Zinc finger protein ZXDC	XM_021477089.2
B3G1409	7.08	7.86	LGALS3BPA	Galectin-3-binding protein A	NM_001040041.1
B16G1126	7.08	3.43	SLC52A3B	Solute carrier family 52, riboflavin transporter, member 3-B	NM_001002182.1
B8G0328	7.08	5.11	MLEC	Malectin	NM_001113618.1
B21G0718	7.08	2.74	RHOBTB3	Rho-related BTB domain-containing protein 3	XM_686178.10
B21G0736	7.08	5.35	DNAJC21	DnaJ homolog subfamily C member 21	NM_200044.1
B14G0624	7.08	1.59	IL21	A0A142FH16_CYPCA Interleukin	
A20G0103	7.08	3.21	KIF13B	Kinesin-like protein KIF13B	XM_002667519.7

B5G0345	7.08	9.41	COQ4	Ubiquinone biosynthesis protein COQ4 homolog, mitochondrial	
A22G0559	7.08	2.02	MRPS28	28S ribosomal protein S28, mitochondrial	
B6G0804	7.07	3.35	SHQ1	Protein SHQ1 homolog	NM_001080600.2
A12G0822	7.07	1.76	LOC113056563	A0A6P6L6K2_CARAU tumor necrosis factor receptor superfamily member 13B-like	
B16G0287	7.07	10.12	DEK	Protein DEK	XM_009292539.4
B24G0449	7.07	7.91	RNF6	E3 ubiquitin-protein ligase RNF6	NM_001386592.1
A7G0286	7.07	0.85	LOC113093989	A0A6P6P321_CARAU uncharacterized protein LOC113093989	
A5G0719	7.07	5.28	G5714_005663	A0A7J6D1M3_9TELE Uncharacterized protein	XM_021471641.2
B9G0856	7.07	6.80	SP3	Transcription factor Sp3	NM_001089498.1
B12G0121	7.07	7.30	EPC1	Enhancer of polycomb homolog 1	XM_689970.9
A4G0679	7.07	7.69	EXOC4	Exocyst complex component 4	NM_001030134.2
B18G0611	7.07	6.96	TRABD	TraB domain-containing protein	NM_212623.1
A24G0574	7.07	3.80	SLC35B3	Adenosine 3'-phospho 5'-phosphosulfate transporter 2	XM_005162556.5
A19G0511	7.07	5.12	CDCA8	Borealin	XM_005173787.5
B5G1156	7.07	10.77	STAG2	Cohesin subunit SA-2	NM_001100028.1
B23G0583	7.07	9.47	UBE4B	Ubiquitin conjugation factor E4 B	XM_021469719.2
A25G0584	7.06	15.79			NM_200880.1
A3G0722	7.06	1.78	CPT1A	Carnitine O-palmitoyltransferase 1, liver isoform	XM_005164059.5
B24G0250	7.06	2.98	ITPRID1	Protein ITPRID1	XM_005162860.5
B17G0129	7.06	4.52	GRM1	Metabotropic glutamate receptor 1	NM_001302252.2
A19G0284	7.06	17.53			
B2G0048	7.06	4.70	MEX3D	RNA-binding protein MEX3D	XM_017354516.3
B12G0768	7.06	26.74	TOB1	Protein Tob1	NM_212974.1
A21G0537	7.06	18.70	EIF4EBP3L	Eukaryotic translation initiation factor 4E-binding protein 3-like	
B13G0733	7.06	7.37	ZMAT4	Zinc finger matrin-type protein 4	NM_001110022.2
B1G0635	7.05	5.88	SCOCA	Short coiled-coil protein A	
B10G0120	7.05	11.36	CWC27	Spliceosome-associated protein CWC27 homolog	XM_009305161.4
A3G0206	7.05	4.93	RNPS1	RNA-binding protein with serine-rich domain 1	NM_199761.2
B14G0322	7.05	6.43	AGA	N(4)-(beta-N-acetylglucosaminyl)-L-asparaginase	NM_001110281.1
A14G0246	7.05	45.54	SEPTIN6	Septin-6	XM_005173240.5
B19G0457	7.05	7.89	KRIT1	Krev interaction trapped protein 1	NM_001317001.1
B5G0244	7.05	5.91	GTF2H3	General transcription factor IIH subunit 3	NM_001128422.1
B12G0766	7.05	14.82	ABCC3	ATP-binding cassette sub-family C member 3	XM_009306371.4
A9G0655	7.05	5.71	IM:6907446	Ashwin	NM_001143933.1
A19G0316	7.05	6.93	CIBAR1	CBY1-interacting BAR domain-containing protein 1	NM_001003604.2
B22G0429	7.05	0.97	DPX16_4609	A0A3N0YC08_ANAGA Uncharacterized protein	
A15G0432	7.05	6.06	GOSR1	Golgi SNAP receptor complex member 1	XM_005157565.5

A16G0527	7.04	6.94	GALNT1	Polypeptide N-acetylgalactosaminyltransferase 1	XM_682380.9
A6G0705	7.04	3.93	PPIL1	Peptidyl-prolyl cis-trans isomerase-like 1	NM_001423703.1
A16G0400	7.04	7.69	PRPF31	U6 small nuclear ribonucleoprotein Prp31	NM_200504.2
A16G0534	7.04	1.17	RNF41	E3 ubiquitin-protein ligase NRDP1	XM_068214144.1
B25G0526	7.04	3.92	E2F4	Transcription factor E2F4	NM_213432.2
B10G0262	7.04	8.11	SFRP1	Secreted frizzled-related protein 1	NM_001083571.1
B10G0458	7.04	12.59	CRTAM	Cytotoxic and regulatory T-cell molecule	XM_021472313.2
B22G1027	7.04	6.08	NLRP3	NACHT, LRR and PYD domains-containing protein 3	XM_021478497.2
B17G0645	7.04	5.26	ITGB1BP1	Integrin beta-1-binding protein 1	NM_001037409.2
B23G0585	7.04	6.63	NMNAT1	nicotinic acid mononucleotide adenylyltransferase 1	NM_001328328.1
B19G0033	7.04	5.86	NRM	Nurim	NM_001045195.1
A23G0302	7.04	9.47	IKBKG	NF-kappa-B essential modulator	NM_001014344.2
A22G0463	7.03	5.81	SSBP3	Single-stranded DNA-binding protein 3	NM_001202512.2
A17G0615	7.03	3.54	SYNE3	Nesprin-3	NM_001083014.2
B8G0190	7.03	9.13	FKBP15	FK506-binding protein 15	XM_021478508.2
A19G0753	7.03	2.03			
B22G1097	7.03	3.79	NLRC3	NLR family CARD domain-containing protein 3	XM_021478497.2
B5G0260	7.03	4.40	SI:CH211-283H6.4	UPF0561 protein C2orf68 homolog	NM_001114693.1
A18G0360	7.03	4.70	KTI12	Protein KTI12 homolog	NM_001126418.1
A2G0975	7.03	68.01	MPZ	Myelin protein P0	NM_194361.2
A15G0188	7.03	6.72	LIN37	Protein lin-37 homolog	XM_068213553.1
A18G0178	7.03	9.09	FAM214A	Protein FAM214A	XM_005173655.5
B16G0064	7.03	17.94	G5714_015591	A0A7J6C606_9TELE Uncharacterized protein	NM_001386446.1
A25G0738	7.03	6.74	SVIP	p97-interacting protein	
B22G0496	7.03	6.23	CD244	Natural killer cell receptor 2B4	XM_068216632.1
B20G0519	7.03	9.11	ACBD3	Golgi resident protein GCP60	NM_001328207.1
B25G0394	7.03	8.78	BRD1	Bromodomain-containing protein 1	XM_005163142.5
B12G0446	7.02	31.49	ACO2	Aconitate hydratase, mitochondrial	NM_198908.1
A6G0320	7.02	7.82	NOMO2	Nodal modulator 2	XM_002662315.4
A5G1121	7.02	10.32	SLC30A5	Zinc transporter 5	NM_001002322.1
B22G0515	7.02	5.83	NOL8	Nucleolar protein 8	NM_001080202.1
B15G0616	7.02	5.68	NUFIP2	FMR1-interacting protein NUFIP2	XM_002664642.6
A21G0879	7.02	6.20	RNF14	E3 ubiquitin-protein ligase RNF14	NM_001002087.1
A5G0133	7.02	8.09	TNKS	Poly [ADP-ribose] polymerase tankyrase-1	XM_005171745.5
A22G0140	7.02	3.68	CCDC51	Mitochondrial potassium channel	NM_001045462.2
B19G0962	7.01	5.87			
B23G0684	7.01	12.21	PLPBP	Pyridoxal phosphate homeostasis protein	NM_001126409.2
B18G0318	7.01	5.16	GMPS	GMP synthase [glutamine-hydrolyzing]	NM_200587.2
B5G0890	7.01	6.18	GLIPR2	Golgi-associated plant pathogenesis-related protein 1	NM_001423664.1
B13G0177	7.01	19.56	MUC4	Mucin-4	NM_001256645.1

A5G1047	7.01	3.10	CENATAC	Centrosomal AT-AC splicing factor	XM_009301864.4
A2G0939	7.01	9.65	LACTB2	Endoribonuclease LACTB2	NM_212884.1
B5G0690	7.01	7.46	ERAL1	GTPase Era, mitochondrial	NM_001128747.1
A16G0328	7.00	18.08	STK40	threonine-protein kinase 40	XM_688183.10
B10G0842	7.00	11.20	VPS26C	Vacuolar protein sorting-associated protein 26C	NM_001003421.1
A20G0355	7.00	5.77	PRRX1	Paired mesoderm homeobox protein 1	XM_017352441.3
A21G0318	7.00	6.03	BRD3	Bromodomain-containing protein 3	NM_001123389.3
A12G0285	7.00	5.98	BMS1	Ribosome biogenesis protein BMS1 homolog	NM_001111150.2
B15G0810	7.00	5.70	SPSB4	SPRY domain-containing SOCS box protein 4	NM_001003474.1
B19G0877	7.00	6.43	PHACTR4A	Phosphatase and actin regulator 4A	NM_001423796.1
B5G1150	7.00	5.78	MTMR2	Myotubularin-related protein 2	NM_131371.1
B16G0635	7.00	14.13	G5714_016089	A0A7J6C7Q6_9TELE DNAX-activation protein 10	NM_001100102.2
B12G0752	7.00	4.22	PTS	6-pyruvoyl tetrahydrobiopterin synthase	
B10G0679	7.00	5.35	KDM2B	Lysine-specific demethylase 2B	NM_001423548.1
B16G0620	7.00	211.21	JARID2	Protein Jumonji	XM_001345849.8
A25G0796	7.00	7.38	PNPLA8	Calcium-independent phospholipase A2-gamma	XM_005174209.4
A16G0804	7.00	18.77	RXRBB	Retinoic acid receptor RXR-beta-B	XM_021466617.2
A14G0004	7.00	0.49	LOC113058086	A0A6P6LAE3_CARAU leucine-rich repeat extensin-like protein 3	
B24G0548	6.99	6.34	ZFHX4	Zinc finger homeobox protein 4	XM_001335046.9
B25G0465	6.99	5.16	GLS2	Glutaminase liver isoform, mitochondrial	XM_009297984.4
B18G0102	6.99	17.37	NAPA	Alpha-soluble NSF attachment protein	NM_199766.1
A10G0836	6.99	5.69	SEC31A	Protein transport protein Sec31A	XM_021479100.2
A23G0380	6.99	6.76	EMC1	ER membrane protein complex subunit 1	XM_021473642.2
B3G0468	6.99	11.65	BRD4	Bromodomain-containing protein 4	NM_001111281.2
B7G0988	6.99	7.08	ARIH1	E3 ubiquitin-protein ligase arih1	NM_212923.1
B8G0511	6.98	4.74	XPC	DNA repair protein complementing XP-C cells	NM_001045210.1
B17G0538	6.98	13.12	SCFD1	Sec1 family domain-containing protein 1	NM_182861.2
B7G0153	6.98	17.20	KARS1	Lysine--tRNA ligase	NM_001328506.1
A9G0583	6.98	5.83	GMPPAA	Mannose-1-phosphate guanyltransferase alpha-A	XM_005167476.5
B9G0694	6.98	0.92	SLC9A2	hydrogen exchanger 2	NM_001114095.1
A5G0364	6.98	11.07	STAG2	Cohesin subunit SA-2	NM_001100028.1
B16G0910	6.98	6.41	CBX3	Chromobox protein homolog 3	NM_001386347.1
A19G0339	6.98	2.99	NTAQ1	Protein N-terminal glutamine amidohydrolase	NM_001044947.1
A13G0308	6.98	7.23	SYT14	Synaptotagmin-14	NM_001346275.1
A24G0565	6.98	9.53	MAK	threonine-protein kinase MAK	NM_199946.1
B5G0239	6.98	3.73	MTRFR	Mitochondrial translation release factor in rescue	
B10G0885	6.98	2.53	EPB41L4A	Band 4.1-like protein 4	NM_131223.2
B11G0595	6.98	10.51	GRM2	Metabotropic glutamate receptor 2	NM_001004601.2

A8G0358	6.98	4.43	LPXN	Leupaxin	XM_684147.10
B3G0727	6.97	2.60	ALDH3B1	Aldehyde dehydrogenase family 3 member B1	NM_173221.2
A17G0787	6.97	2.98	TP53I3	Quinone oxidoreductase PIG3	
B18G0022	6.97	10.20	MOB2	MOB kinase activator 2	XM_005173694.5
A24G0239	6.97	3.69	HCCS	Holocytochrome c-type synthase	NM_201451.1
B5G1173	6.97	14.01	BRWD3	Bromodomain and WD repeat-containing protein 3	XM_021471594.2
A18G0149	6.97	7.51	POLR2I	DNA-directed RNA polymerase II subunit RPB9	NM_001006013.3
A14G0534	6.97	0.92	STING1	Stimulator of interferon genes protein	XM_005157121.5
B5G0722	6.97	4.43	PDZD2	PDZ domain-containing protein 2	XM_021471658.2
A22G0049	6.97	12.53	ARL8A	ADP-ribosylation factor-like protein 8A	NM_001200020.1
B8G0319	6.97	5.51	ORAI1	Calcium release-activated calcium channel protein 1	XM_689954.9
A3G0973	6.96	3.87	LOC107678872	threonine-protein kinase TAO2-like	XM_678910.7
B14G0445	6.96	1.87	SIMC1	SUMO-interacting motif-containing protein 1	
B5G1137	6.96	7.69	RPS6KA3	Ribosomal protein S6 kinase alpha-3	XM_005165118.5
A4G0692	6.96	7.07	AKR1B1	Aldo-keto reductase family 1 member B1	NM_001077247.1
A23G0052	6.96	6.33	AAR2	Protein AAR2 homolog	NM_001002221.1
B15G0182	6.96	8.70	BRWD1	Bromodomain and WD repeat-containing protein 1	XM_001920406.8
B3G0932	6.96	31.90	COX6A	Cytochrome c oxidase subunit 6A, mitochondrial	
B9G0504	6.96	3.01	TYW5	tRNA wybutosine-synthesizing protein 5	NM_001077543.1
A8G0115	6.96	20.56	UFD1	Ubiquitin recognition factor in ER-associated degradation protein 1	NM_001002451.1
B14G0234	6.95	4.40	LNX2	Ligand of Numb protein X 2	NM_212940.2
A2G0222	6.95	8.11	ZZZ3	ZZ-type zinc finger-containing protein 3	NM_001100027.1
B13G0774	6.95	6.52	CAPN2	Calpain-2 catalytic subunit	XR_002459827.2
A2G0001	6.95	4.57	ZNF830	Zinc finger protein 830	XM_003197803.4
B2G1044	6.95	12.26	NPC1	NPC intracellular cholesterol transporter 1	XM_005163659.5
B6G0076	6.95	7.14	ACVR1B	Activin receptor type-1B	XM_682541.9
A16G0885	6.95	17.90	TAPBPL	Tapasin-related protein	XM_001919950.6
A14G0447	6.95	5.21	SFXN1	Sideroflexin-1	NM_001003537.2
B22G0190	6.95	0.96	LOC107702635	A0A671PYY6_9TELE Uncharacterized LOC107702635	XM_068216394.1
A16G0792	6.94	7.35	RBM12B	RNA-binding protein 12B	NM_001290140.1
A12G0686	6.94	7.83	CCDC6	Coiled-coil domain-containing protein 6	NM_200241.2
A9G0825	6.94	10.42	FMNL2	Formin-like protein 2	XM_009304461.4
A21G0736	6.94	10.38	FAM114A2	Protein FAM114A2	XM_017352976.3
B23G0913	6.94	13.84	FBXL5	LRR-repeat protein 5	NM_001301328.1
B20G0005	6.94	7.81	TAB2	TGF-beta-activated kinase 1 and MAP3K7-binding protein 2	NM_001316693.1
B23G0034	6.94	2.72	SLC25A20	acylcarnitine carrier protein	XM_001920183.7
B22G0418	6.93	4.81	LOC113099127	A0A6P6PG04_CARAU uncharacterized protein LOC113099127	XM_068216397.1
B21G0585	6.93	6.02	ERI1	3'-5' exoribonuclease 1	NM_001020614.1

A23G0858	6.93	16.74	DDX58	Antiviral innate immune response receptor RIG-I	
A3G1086	6.93	6.17	GIMAP4	GTPase IMAP family member 4	NM_001127520.1
B15G0306	6.93	5.92	NECTIN3	Nectin-3	XM_001337576.6
A3G1257	6.93	25.32	RNF213A	E3 ubiquitin-protein ligase rnf213-alpha	NM_001353835.1
B23G0339	6.93	7.70	TRPC4AP	Short transient receptor potential channel 4-associated protein	NM_001310093.1
B11G0241	6.93	6.31	ELAVL1-B	ELAV-like protein 1-B	NM_199926.1
A16G0895	6.93	11.45	LOC107751895	PLAUR domain-containing protein 2-like	
B25G0123	6.93	18.94	GLG1	Golgi apparatus protein 1	XM_068217594.1
B1G0532	6.93	5.50	STS	Steryl-sulfatase	XM_005168397.5
B14G0781	6.93	1.51	A33	Zinc-binding protein A33	NM_001044961.1
A21G0815	6.92	2.75	CMC4	Cx9C motif-containing protein 4	NM_001303002.1
B11G0335	6.92	2.89	ADAMTS9	A disintegrin and metalloproteinase with thrombospondin motifs 9	NM_201174.1
B8G0223	6.92	9.39	GRK5	G protein-coupled receptor kinase 5	XM_005167252.5
B6G0502	6.92	7.18	LPP	Lipoma-preferred partner homolog	XM_068221782.1
B5G0165	6.92	8.32	GSTT1	Glutathione S-transferase theta-1	NM_200521.2
A9G0636	6.92	5.73	TBA	Tubulin alpha chain	XM_005167445.5
A10G0835	6.92	2.13	RTASE	Probable RNA-directed DNA polymerase from transposon BS	XM_068220297.1
A2G0143	6.92	1.14	SLC35A3	UDP-N-acetylglucosamine transporter	XM_005171166.5
A14G0185	6.92	15.51	ATL3	Atlastin-3	XM_005157232.3
B3G0832	6.91	38.53	FUS	RNA-binding protein FUS	NM_201083.2
B17G0250	6.91	2.68	PTGER2	Prostaglandin E2 receptor EP2 subtype	NM_200635.1
B7G0802	6.91	14.20	COPB1	Coatomer subunit beta	NM_001002013.1
A11G0694	6.91	5.36	BMP7	Bone morphogenetic protein 7	XM_001339444.6
B23G0087	6.91	10.75	RAB5IF	Respirasome Complex Assembly Factor 1	NM_199850.2
B15G0213	6.91	7.33	COG6	Conserved oligomeric Golgi complex subunit 6	NM_001320347.1
A3G0319	6.91	1.02	A33	Zinc-binding protein A33	XM_003198167.6
A17G0272	6.91	2.35	ACBD3	Golgi resident protein GCP60	NM_001089387.2
B8G0167	6.91	6.58	FGFR1A	Fibroblast growth factor receptor 1-A	XM_009304304.4
B16G0557	6.91	6.82	LOC113059858	A0A6P6LI57_CARAU uncharacterized protein LOC113059858	
B21G0359	6.90	9.35	PURA	Transcriptional activator protein Pur-alpha	NM_001001846.2
B9G0525	6.90	6.73	EPC2	Enhancer of polycomb homolog 2	NM_201075.1
A9G0764	6.90	4.05	RAP2A	Ras-related protein Rap-2a	XM_003199163.5
A10G0784	6.90	8.20	ERBIN	Erbin	XM_005155577.4
B24G0334	6.90	5.89	PHC3	Polyhomeotic-like protein 3	XM_009297490.4
A22G0303	6.90	11.31	SI:CH73-338D8.5	A0A0R4IZ26_DANRE Si:ch73-338d8.5	
B3G0337	6.90	3.82	GADD45GIP1	Growth arrest and DNA damage-inducible proteins-interacting protein 1	
B10G0273	6.89	6.17	STK10	threonine-protein kinase 10	XM_021472247.2
B25G0602	6.89	6.19	CSK22	Casein kinase II subunit alpha'	XM_679168.9
A5G0488	6.89	7.90	B3GALT1	Beta-1,3-galactosyltransferase 1	

A13G0680	6.89	4.65	STOX1	Storkhead-box protein 1	
A4G0434	6.89	3.19	LOC113057258	A0A6P6L9C4_CARAU gastrula zinc finger protein XlCGF26.1-like	XM_068213808.1
A9G0335	6.89	4.43	ANKRD52	threonine-protein phosphatase 6 regulatory ankyrin repeat subunit C	XM_005167783.5
A8G0598	6.89	14.20	DOCK8	Dedicator of cytokinesis protein 8	XM_021478460.2
B20G0757	6.89	9.54	MARK3	microtubule affinity-regulating kinase 3	XM_009294921.4
B2G0410	6.89	4.75	PARP2	Poly [ADP-ribose] polymerase 2	XM_021478330.2
B13G0124	6.89	1.95	ABCG2	Broad substrate specificity ATP-binding cassette transporter ABCG2	
A3G0401	6.89	3.03	UBALD1	UBA-like domain-containing protein 1	NM_001020671.2
B16G0312	6.89	5.91	K1143	Uncharacterized protein KIAA1143 homolog	
B15G0308	6.88	4.29	MED29	Mediator of RNA polymerase II transcription subunit 29	NM_173271.2
A9G0344	6.88	3.82	TMTC4	Protein O-mannosyl-transferase TMTC4	NM_001173502.1
A25G0676	6.88	6.47	IGKV3-15	Immunoglobulin kappa variable 3-15	XM_068217596.1
B1G1143	6.88	171.94	HSPG2	Basement membrane-specific heparan sulfate proteoglycan core protein	NM_001034182.2
B5G1104	6.88	13.67	AP1B1	AP-1 complex subunit beta-1	XM_005165166.4
B19G0846	6.88	6.24	SEH1L	Nucleoporin SEH1	NM_199923.2
B20G0392	6.88	6.19	PFAS	Phosphoribosylformylglycinamidine synthase	NM_001045202.1
A15G0418	6.88	5.12	TLCD2	TLC domain-containing protein 2	NM_001110382.1
A5G0223	6.88	9.10	SUDS3	Sin3 histone deacetylase corepressor complex component SDS3	NM_001003493.2
B13G0097	6.88	13.68	KLHL31	Kelch-like protein 31	NM_001003437.2
A6G0577	6.88	4.48	AKR1A1B	Aldo-keto reductase family 1 member A1-B	XM_005166285.4
B3G1250	6.88	6.25	WNK4	threonine-protein kinase WNK4	XM_680072.10
B11G0021	6.88	1.44	IARS1	Isoleucine--tRNA ligase, cytoplasmic	
B2G0567	6.87	10.08	DHX9	ATP-dependent RNA helicase A	NM_001201444.1
B25G0607	6.87	2.95	LDHD	Probable D-lactate dehydrogenase, mitochondrial	NM_199873.1
A7G0362	6.87	9.59	LRCH4	Leucine-rich repeat and calponin homology domain-containing protein 4	XM_009303554.4
B8G0854	6.87	3.28	FXN	Frataxin, mitochondrial	NM_001083016.2
B7G0604	6.87	4.52	HEATR3	HEAT repeat-containing protein 3	NM_200889.1
B17G0104	6.87	10.53	EPHX2	Bifunctional epoxide hydrolase 2	NM_001008642.1
B22G0797	6.87	16.21	AP3D1	AP-3 complex subunit delta-1	XM_009296265.4
B13G0678	6.87	4.46	MAP3K9	Mitogen-activated protein kinase kinase kinase 9	XM_009307228.4
B4G0455	6.87	6.36	GNPTAB	beta	XM_005164744.5
A18G0107	6.87	20.67	EHD2	EH domain-containing protein 2	NM_200063.1
B15G0053	6.87	10.51	HEPHL1	Ferroxidase HEPHL1	NM_001328065.1
B16G0495	6.87	3.27	G5714_015973	A0A7J6C771_9TELE Uncharacterized protein	

A3G0402	6.87	8.60	MGRN1	E3 ubiquitin-protein ligase MGRN1	NM_001144782.1
A13G0467	6.87	16.23	COL17A1	Collagen alpha-1(XVII) chain	XM_005156798.5
A8G0216	6.86	9.16	RAPGEF1	Rap guanine nucleotide exchange factor 1	XM_005166904.5
B11G0346	6.86	2.23	FEZF2	Fez family zinc finger protein 2	NM_001080005.1
B2G0838	6.86	5.54	MPLKIP	M-phase-specific PLK1-interacting protein	NM_001202486.1
A3G1299	6.86	63.50	ENDOD1	Endonuclease domain-containing 1 protein	
A12G0388	6.86	12.40	SVIL	Supervillin	XM_068224487.1
B3G0694	6.86	13.26	TRAF7	E3 ubiquitin-protein ligase TRAF7	NM_001080185.1
B22G0926	6.86	14.33	COL8A1	Collagen alpha-1(VIII) chain	XM_005161783.5
A10G0754	6.86	5.84	NRARPA	Notch-regulated ankyrin repeat-containing protein A	NM_181495.3
B19G0108	6.86	6.09	DAXX	Death domain-associated protein 6	XM_005159438.5
A1G1113	6.86	6.18	GPANK1	G patch domain and ankyrin repeat-containing protein 1	NM_001327761.1
B7G0703	6.86	6.76	LIN7C	Protein lin-7 homolog C	NM_001362514.1
B7G0398	6.86	3.99	MTFMT	Methionyl-tRNA formyltransferase, mitochondrial	NM_001077542.2
B15G0709	6.86	6.70	BAG6	Large proline-rich protein BAG6	NM_201288.1
B19G0522	6.86	7.57	VPS45	Vacuolar protein sorting-associated protein 45	NM_001256656.1
B15G0704	6.85	3.08	CRPPA	D-ribitol-5-phosphate cytidylyltransferase	XM_005157764.5
B9G0549	6.85	7.99	IDH1	Isocitrate dehydrogenase [NADP] cytoplasmic	XM_005167655.5
B10G0701	6.85	2.95	LAGE3	KEOPS complex subunit LAGE3	
A20G0660	6.85	6.51	VIPAS39	Spermatogenesis-defective protein 39 homolog	NM_001001836.1
A22G0275	6.85	11.05	RND3	Rho-related GTP-binding protein RhoE	NM_199522.1
A17G0218	6.85	5.46	MBIP	MAP3K12-binding inhibitory protein 1	NM_001256179.1
B4G0572	6.85	6.38	KIN	RNA-binding protein KIN17	NM_001030086.3
A24G0605	6.85	9.43	APBB1IP	Amyloid beta A4 precursor protein-binding family B member 1-interacting protein	NM_200634.1
A10G0829	6.85	3.07	VAMP8	Vesicle-associated membrane protein 8	NM_001293183.1
A10G0712	6.85	8.43	HVCN1	Voltage-gated hydrogen channel 1	NM_001002346.1
A5G1009	6.85	4.57	FAM98A	Protein FAM98A	NM_001326608.1
A19G0907	6.85	6.05	AKAP9	A-kinase anchor protein 9	XM_021468220.2
A22G0063	6.85	2.14	LAMB3	Laminin subunit beta-3	XM_695716.11
B12G0650	6.85	8.81	EXOC6	Exocyst complex component 6	XM_005156061.5
A18G0479	6.85	5.20	HACD3	Very-long-chain (3R)-3-hydroxyacyl-CoA dehydratase	NM_001044984.1
A12G0444	6.84	9.88	JOSD1	Josephin-1	NM_001076595.2
A7G0219	6.84	0.37	RASSF10	Ras association domain-containing protein 10	XM_001921630.8
B15G0917	6.84	4.68	WDR20	WD repeat-containing protein 20	XM_009292005.4
B10G0594	6.84	5.39	DHRS13	reductase SDR family member 13	NM_001007363.2
B9G0263	6.84	4.41	PDIA5	Protein disulfide-isomerase A5	NM_001113576.1
B8G0655	6.84	4.11	TOR1AIP2	Torsin-1A-interacting protein 2	

A1G1029	6.84	24.59	SPOCK3	Testican-3	XM_017355108.2
B12G0805	6.83	3.68	ZDHHC16B	Palmitoyltransferase ZDHHC16B	NM_001327799.1
B11G0031	6.83	5.00	PPARG	Peroxisome proliferator-activated receptor gamma	XM_021479927.2
A5G0997	6.83	9.67	ZGC:85789	Ester hydrolase C11orf54 homolog	NM_212616.1
B20G0420	6.83	7.12	ARMT1	Damage-control phosphatase ARMT1	NM_001014331.2
A8G0918	6.83	3.55	MTHFR	Methylenetetrahydrofolate reductase	NM_001281840.1
A7G0861	6.83	16.38	CD99L2	CD99 antigen-like protein 2	XM_009303030.4
B7G0841	6.83	4.82	SNAPC2	snRNA-activating protein complex subunit 2	XM_068222605.1
B18G0445	6.83	32.56	CTCF	Transcriptional repressor CTCF	XM_068214753.1
A3G0367	6.83	2.50	G5714_021002	A0A7J6BZA4_9TELE Uncharacterized protein	XM_021474798.2
B8G0938	6.82	5.10	DOCK5	Dedicator of cytokinesis protein 5	NM_001110012.1
B3G1264	6.82	9.02	ANKRD54	Ankyrin repeat domain-containing protein 54	NM_001002578.1
A24G0449	6.82	9.44	RELCH	RAB11-binding protein RELCH homolog	XM_017353808.3
A20G0379	6.82	7.34	ATF6	Cyclic AMP-dependent transcription factor ATF-6 alpha	NM_001110519.1
B17G0616	6.82	3.70	PROX1	Prospero homeobox protein 1	XM_068214325.1
A5G0135	6.82	4.30	RCHY1	RING finger and CHY zinc finger domain-containing protein 1	NM_212600.2
A16G0654	6.82	1.29	EFNA4	Ephrin-A4	XM_001342982.8
B6G0259	6.82	1.33			XM_021477036.2
A8G0174	6.81	10.33	UXT	Protein UXT	NM_001004671.1
B10G0852	6.81	8.39	EMSY	BRCA2-interacting transcriptional repressor EMSY	NM_199984.2
B20G0683	6.81	4.46	MPV17	Protein Mpv17	NM_201165.2
B18G0490	6.81	10.85	DDB2	DNA damage-binding protein 2	XM_005159034.5
B10G0131	6.81	12.88	UGCG	Ceramide glucosyltransferase	NM_001024207.2
B6G0401	6.81	3.47	CBX8	Chromobox protein homolog 8	NM_001024415.2
A10G0092	6.81	6.49	NAA25	N-alpha-acetyltransferase 25, NatB auxiliary subunit	XM_005172598.5
B9G0297	6.81	6.35	STAG2	Cohesin subunit SA-2	XM_017357835.3
B15G0631	6.81	7.28	KSR1	Kinase suppressor of Ras 1	XM_068213759.1
A11G0210	6.80	6.24	MED16	Mediator of RNA polymerase II transcription subunit 16	NM_001114739.2
A16G0373	6.80	7.19	PRSS35	Inactive serine protease 35	NM_001002212.1
B10G0104	6.80	5.75	TRAPPC13	Trafficking protein particle complex subunit 13	XM_005155559.5
B12G0671	6.80	4.23	TFAM	Transcription factor A, mitochondrial	NM_001077389.1
A7G0627	6.80	7.55	NOD2	Nucleotide-binding oligomerization domain-containing protein 2	NM_001328044.1
A13G0198	6.80	7.99	NIN	Ninein	XR_011017212.1
B7G0497	6.80	6.75	GPT2	Alanine aminotransferase 2	XM_068222422.1
B14G0809	6.80	11.19	RTASE	Probable RNA-directed DNA polymerase from transposon BS	XR_011011165.1
A7G0563	6.80	5.25	GRPEL1	GrpE protein homolog 1, mitochondrial	NM_001037384.2
B4G0454	6.80	9.21	DRAM1	DNA damage-regulated autophagy modulator protein 1	XM_068219590.1
A3G0833	6.80	19.49	USP7	Ubiquitin carboxyl-terminal hydrolase 7	XM_005163957.4

A5G0021	6.80	10.84	SURF4	Surfeit locus protein 4	NM_201127.1
B8G1052	6.80	5.92	SNCAIP	Synphilin-1	XM_009303688.4
B9G0396	6.80	4.74	WDR3	WD repeat-containing protein 3	NM_198873.1
B21G0313	6.79	7.19	ALOX15B	Polyunsaturated fatty acid lipoxygenase ALOX15B	
B17G0337	6.79	2.70	SAMD8	Sphingomyelin synthase-related protein 1	NM_001089470.1
B11G0394	6.79	1.92	CNTN2	Contactin-2	
B20G0336	6.79	4.17	JAG2	Protein jagged-2	NM_131862.1
B3G1018	6.79	22.74	MAPK1	Mitogen-activated protein kinase 1	NM_201507.1
A21G0012	6.79	27.77	JAK2	Tyrosine-protein kinase JAK2	NM_131093.1
B7G0996	6.79	17.90	ABHD2A	Monoacylglycerol lipase ABHD2	NM_200914.1
A23G0179	6.79	12.47	DDX23	Probable ATP-dependent RNA helicase DDX23	NM_199882.1
A14G0214	6.79	1.74	CD40LG	CD40 ligand	NM_001144809.1
A25G0265	6.78	2.95	NAV2	Neuron navigator 2	XM_068219404.1
A1G0574	6.78	4.52	KLF12	Krueppel-like factor 12	NM_207062.1
A18G0451	6.78	1.60	FAM180B	Protein FAM180B	XM_021473282.2
A1G0814	6.78	2.89	MMD2	Monocyte to macrophage differentiation factor 2	XM_068220992.1
A1G1003	6.78	8.04	RBM47	RNA-binding protein 47	XM_009307327.4
B9G0215	6.78	27.77	STAT1	Signal transducer and activator of transcription 1	NM_200091.2
B1G1182	6.78	2.61	REXO5	RNA exonuclease 5	
A5G1000	6.78	6.14	AKAP10	A-kinase anchor protein 10, mitochondrial	XM_009301915.4
B25G0166	6.78	16.93	IGKV3-15	Immunoglobulin kappa variable 3-15	XM_068217596.1
B13G0798	6.78	6.12	SYF2	Pre-mRNA-splicing factor syf2	NM_001083833.1
A4G0978	6.78	3.11	PWP1	Periodic tryptophan protein 1 homolog	NM_200292.1
B1G0362	6.78	6.29	KDM4C	Lysine-specific demethylase 4C	NM_001083851.1
B8G0757	6.78	19.47	NDUFAF2	NADH dehydrogenase [ubiquinone] 1 alpha subcomplex assembly factor 2	
A6G0123	6.77	6.73	DTD1	D-aminoacyl-tRNA deacylase 1	NM_001003440.1
A18G0478	6.77	3.00	INTS14	Integrator complex subunit 14	XM_005159052.5
B4G0187	6.77	70.11	CALD1	Caldesmon	NM_001114883.1
B22G0801	6.77	7.57	SCAMP4	Secretory carrier-associated membrane protein 4	NM_001025525.1
A12G0398	6.77	3.82			XM_021472535.2
B8G0762	6.77	11.18	OSBPL9	Oxysterol-binding protein-related protein 9	XM_009303857.4
A14G0335	6.77	10.83	BTK	Tyrosine-protein kinase BTK	XM_068218779.1
B22G0375	6.77	17.20	G3PMI2	G3PMI2_GASAC HECT domain-containing protein	
B19G0778	6.77	1.75	CLEC4F	C-type lectin domain family 4 member F	XM_068224046.1
B12G0231	6.77	9.38	NAT9	beta-tubulin-N-acetyltransferase 9	NM_001328002.1
B12G0077	6.77	8.09	TVP23B	Golgi apparatus membrane protein TVP23 homolog B	NM_001024428.1
B3G0795	6.77	5.64	GTF2F1	General transcription factor IIF subunit 1	NM_001423629.1
A4G0897	6.77	7.12	TMEM209	Transmembrane protein 209	NM_001270973.1
B5G1202	6.77	4.22	MTMR8	Myotubularin-related protein 8	NM_200394.1
A16G0912	6.77	4.33	ANO10	Anoctamin-10	NM_001030206.1
B23G0316	6.77	6.99	G5714_022797	A0A7J6BP71_9TELE PNPLA domain-containing protein	XR_002456574.2

B12G0631	6.77	11.40	SMARCD2	SNF-related matrix-associated actin-dependent regulator of chromatin subfamily D member 2	XM_687657.7
A2G0246	6.76	10.26	YME1L1	ATP-dependent zinc metalloprotease YME1L1	NM_001331068.1
A19G0470	6.76	4.47	ANKIB1	Ankyrin repeat and IBR domain-containing protein 1	XM_005159781.5
A13G0774	6.76	18.89	ZNF318	Zinc finger protein 318	NM_001013501.2
B2G0282	6.76	9.64	KIF5B	Kinesin-1 heavy chain	XM_005171408.5
A9G0169	6.76	3.38	COL18A1	Collagen alpha-1(XVIII) chain	XM_021478720.2
B5G0326	6.76	5.04	FERMT3	Fermitin family homolog 3	XM_021476681.2
A10G0323	6.76	7.69	MEDAG	Mesenteric estrogen-dependent adipogenesis protein	XM_021472361.2
B13G0139	6.76	9.43	TBA1B	Tubulin alpha-1B chain	NM_200605.1
A22G0322	6.76	17.23	SAFB2	Scaffold attachment factor B2	NM_214683.1
A12G0077	6.76	12.77	TVP23B	Golgi apparatus membrane protein TVP23 homolog B	NM_001024428.1
A19G0825	6.76	2.29	HSD17B8	(3R)-3-hydroxyacyl-CoA dehydrogenase	NM_001005292.2
A7G1239	6.76	16.02	RTASE	Probable RNA-directed DNA polymerase from transposon BS	XM_068214189.1
A23G0781	6.76	3.10	CYP2B15	Cytochrome P450 2B15	NM_001109701.2
B7G0753	6.76	14.51	VPS13C	Intermembrane lipid transfer protein VPS13C	XM_021471938.2
A7G1120	6.76	3.93	ANPEP	Aminopeptidase N	XM_021477719.2
A5G0169	6.76	7.39	CASTOR1	Cytosolic arginine sensor for mTORC1 subunit 1	NM_001020815.2
B14G0400	6.76	7.20	SCYL1	N-terminal kinase-like protein	XM_001332472.7
B12G0275	6.76	5.39	BMS1	Ribosome biogenesis protein BMS1 homolog	NM_001111150.2
B17G0582	6.76	10.20	IVD	Isovaleryl-CoA dehydrogenase, mitochondrial	NM_201491.1
A3G0863	6.76	5.74	RABEP2	Rab GTPase-binding effector protein 2	NM_001080625.2
B21G0046	6.75	9.32	STK10	threonine-protein kinase 10	NM_201133.2
A5G1035	6.75	3.48	VPS37D	Vacuolar protein sorting-associated protein 37D	XM_005172016.5
B21G0647	6.75	4.99	MZT2	Mitotic-spindle organizing protein 2	NM_001316932.1
A23G0599	6.75	8.44	HBS1L	HBS1-like protein	NM_001308548.1
B13G0138	6.75	3.99	CUL9	Cullin-9	NM_200518.2
B13G0815	6.75	7.95	TMEM63B	CSC1-like protein 2	NM_001076745.2
B11G0738	6.75	3.75	GPR137BA	G protein-coupled receptor 137Ba	NM_001039826.2
A13G0669	6.75	2.84	PRXL2A	Peroxiredoxin-like 2A	NM_212937.1
A1G0880	6.75	7.99	CNPY3	Protein canopy homolog 3	NM_001039805.2
A9G0063	6.75	7.31	IL1R1	Interleukin-1 receptor type 1	
A3G0834	6.75	11.13	HAPSTR1	HUWE1-associated protein modifying stress responses	NM_001328166.1
B8G0276	6.75	4.13	LOC107747213	A0A673HEJ1_9TELE Uncharacterized LOC107747213	NM_001080076.2
B21G0317	6.75	8.89	NCBP3	Nuclear cap-binding protein subunit 3	NM_200143.1
A13G0209	6.74	3.57	GMFB	Glia maturation factor beta	NM_001002321.1
B9G0623	6.74	7.00	GMPPAA	Mannose-1-phosphate guanyltransferase alpha-A	XM_005167476.5
A21G0510	6.74	4.05	TMEM223	Transmembrane protein 223	NM_001003594.2
B10G0553	6.74	2.85	CD276	CD276 antigen homolog	XM_005172670.5
B12G0793	6.74	1.37	G5714_012965	A0A7J6CK77_9TELE Uncharacterized protein	

B5G1022	6.74	22.48	VAMP8	Vesicle-associated membrane protein 8	NM_199759.1
B12G0043	6.74	4.54	DDIT4	DNA damage-inducible transcript 4 protein	
A7G0704	6.74	4.65	ARL14EP	ARL14 effector protein	
B3G0890	6.74	0.12	FRIM	Ferritin, middle subunit	NM_001109854.1
B4G0462	6.74	7.66	APAF1	Apoptotic protease-activating factor 1	XM_068219732.1
B24G0518	6.74	7.80	PIP4K2A	Phosphatidylinositol 5-phosphate 4-kinase type-2 alpha	NM_001128702.1
A2G0517	6.74	5.07	CRTC1	CREB-regulated transcription coactivator 1	XM_068212850.1
A24G0382	6.73	6.86	ACOT9	Acyl-coenzyme A thioesterase 9, mitochondrial	XM_005162711.5
A25G0665	6.73	2.69	IGKV3-20	Immunoglobulin kappa variable 3-20	XM_068217596.1
A3G0143	6.73	16.26	GIMAP8	GTPase IMAP family member 8	XM_068222067.1
B22G0651	6.73	5.17	SLC30A7	Zinc transporter 7	NM_001100086.1
B21G0504	6.73	6.53	NADK2	NAD kinase 2, mitochondrial	XM_005161165.5
A22G0043	6.73	7.73	CDKN1A	Cyclin-dependent kinase inhibitor 1	
A21G0732	6.73	3.69	AMOT	Angiomotin	XM_686779.10
A19G0251	6.73	5.45	CF136	Uncharacterized protein C6orf136 homolog	NM_001082846.1
B10G0748	6.73	7.22	GNS	N-acetylglucosamine-6-sulfatase	NM_199841.2
A5G0641	6.72	7.54	TNPO1	Transportin-1	XR_011009508.1
B9G0211	6.72	3.68	MFSD6B	Major facilitator superfamily domain-containing protein 6-B	NM_001328216.1
B9G0344	6.72	5.25	ANKRD50	Ankyrin repeat domain-containing protein 50	XM_017357830.3
A20G0371	6.72	2.72	SCYL3	Protein-associating with the carboxyl-terminal domain of ezrin	NM_199657.1
B7G0444	6.72	6.15	CRABP1	Cellular retinoic acid-binding protein 1	NM_001001842.1
A9G0767	6.72	5.41	DOCK9	Dedicator of cytokinesis protein 9	XM_068223516.1
A22G0673	6.72	3.76	RUBCN	Run domain Beclin-1-interacting and cysteine-rich domain-containing protein	XM_002666369.6
A7G1029	6.72	3.52	OXA1L	Mitochondrial inner membrane protein OXA1L	NM_001105130.2
B7G1285	6.72	5.13	TOX4-B	TOX high mobility group box family member 4-B	NM_001128286.1
A18G0803	6.72	13.31	CDC42SE2	CDC42 small effector protein 2	XR_658791.4
B23G0594	6.72	5.87	MXRA8	Matrix remodeling-associated protein 8	NM_001079960.3
A5G0892	6.72	4.50	TENT2	Poly(A) RNA polymerase GLD2	XM_021476157.2
A24G0057	6.72	5.83	LOC113076591	A0A6P6NA55_CARAU uncharacterized protein LOC113076591	XM_021470240.2
A16G0141	6.72	9.76	RPZ	Protein rapunzel	NM_001110124.1
B16G0736	6.72	2.10	A33	Zinc-binding protein A33	NM_001045168.1
A1G0734	6.72	3.51	N6AMT1	Methyltransferase N6AMT1	
B6G0468	6.72	5.00	HS2ST1	Heparan sulfate 2-O-sulfotransferase 1	XM_009302420.4
B13G0461	6.72	6.33	CALHM2	Calcium homeostasis modulator protein 2	NM_212791.2
B5G0570	6.72	16.13	BLVRB	Flavin reductase (NADPH)	
B16G0126	6.72	11.93	PBX2	Pre-B-cell leukemia transcription factor 2	XM_017351360.3

B17G0521	6.71	8.95	PTAFR	Platelet-activating factor receptor	XM_001342336.6
B12G0050	6.71	3.66	LRRC20	Leucine-rich repeat-containing protein 20	NM_001430866.1
A22G0449	6.71	9.11	MAP2K2	Dual specificity mitogen-activated protein kinase kinase 2	NM_001037391.2
A7G0682	6.71	8.17	TLE3B	Transducin-like enhancer protein 3-B	XM_009303191.4
B20G0296	6.71	5.14	SOX7	Transcription factor Sox-7	NM_001080750.2
B8G0534	6.71	1.18	KCNQ2	Potassium voltage-gated channel subfamily KQT member 2	NM_001114907.1
A25G0713	6.71	12.71	AMFR	E3 ubiquitin-protein ligase AMFR	NM_213163.2
A18G0800	6.71	5.35	SPATA5L1	Ribosome biogenesis protein SPATA5L1	NM_001076588.2
B5G0926	6.71	2.29	PKN2	threonine-protein kinase N2	XM_003198639.6
B8G0401	6.71	7.84	ZNF131	Zinc finger protein 131	NM_200505.2
B5G0963	6.71	14.36	HMBS	Porphobilinogen deaminase	NM_201154.1
B6G0034	6.71	8.70	CYTH4	Cytohesin-4	NM_001114903.2
B5G0796	6.71	19.64	LRRN4	Leucine-rich repeat neuronal protein 4	XM_068221462.1
A13G0526	6.71	6.35	GLUD1	Glutamate dehydrogenase 1, mitochondrial	XM_682373.10
B6G0370	6.70	1.53	THUMPD3	tRNA (guanine(6)-N2)-methyltransferase THUMP3	XM_068221753.1
B5G0433	6.70	1.11	POU2F3	POU domain, class 2, transcription factor 3	NM_001045255.2
A3G0489	6.70	9.61	FOXK1	Forkhead box protein K1	NM_199902.1
B20G0915	6.70	11.32	CDC40	Pre-mRNA-processing factor 17	NM_001009990.1
A9G0875	6.70	8.43	RANBP2	E3 SUMO-protein ligase RanBP2	XM_002663280.7
A4G0404	6.70	1.40	A0A672SDX4	A0A672SDX4_SINGR Uncharacterized protein	XM_068220706.1
A12G0241	6.70	17.86	NAT9	beta-tubulin-N-acetyltransferase 9	NM_001328002.1
B19G0691	6.70	4.33	YARS1	Tyrosine--tRNA ligase, cytoplasmic	NM_201316.1
A5G0642	6.69	6.11	PTCD2	Pentatricopeptide repeat-containing protein 2, mitochondrial	XM_005171851.5
B16G0366	6.69	7.98	SCMH1	Polycomb protein SCMH1	NM_001089484.1
B18G0832	6.69	3.59	LARP6	La-related protein 6	NM_199903.1
B7G0595	6.69	9.43	SLC7A10	Asc-type amino acid transporter 1	NM_001386372.1
A20G0682	6.69	2.41	MARVELD3	MARVEL domain-containing protein 3	NM_001171033.2
A3G0572	6.69	14.86	RAMP2	Receptor activity-modifying protein 2	XM_017354532.3
A1G0926	6.69	5.85	ELF2	ETS-related transcription factor Elf-2	XM_005159830.5
A16G0082	6.69	7.20	AGO3	Protein argonaute-3	XM_068213994.1
B7G0579	6.69	8.40	PSMC3-A	26S proteasome regulatory subunit 6A-A	XM_005168963.5
A21G0563	6.69	3.57	BAD	Bcl2-associated agonist of cell death	
B6G0341	6.69	7.97	SLC19A1	Reduced folate transporter	XM_017356601.3
A3G0779	6.69	5.06	MED25	Mediator of RNA polymerase II transcription subunit 25	XM_005163940.5
B23G0511	6.69	6.35	PTPN12	Tyrosine-protein phosphatase non-receptor type 12	NM_001099420.1
B11G0414	6.69	4.12	GPR25	Probable G-protein coupled receptor 25	NM_001025540.1

B2G0724	6.68	5.25	TESK2	Dual specificity testis-specific protein kinase 2	NM_001110476.1
A18G0156	6.68	9.51	CIPC	CLOCK-interacting pacemaker	NM_001045055.1
A11G0705	6.68	18.70	ADGRL4	Adhesion G protein-coupled receptor L4	XM_021479657.2
A5G1297	6.68	14.38	MAT2A	S-adenosylmethionine synthase isoform type-2	XM_017356170.3
B25G0464	6.68	14.48	SH3GL3	Endophilin-A3	NM_200181.2
A22G0569	6.68	8.57	GLIS2	Zinc finger protein GLIS2	XM_009296416.4
B12G0393	6.68	3.41	NF7O	Nuclear factor 7, ovary	XM_009306726.4
A2G0549	6.68	6.24	RABGGTB	Geranylgeranyl transferase type-2 subunit beta	NM_213112.2
A5G0529	6.68	5.93	LOC113074480	A0A6P6N254_CARAU uncharacterized protein LOC113074480	
A17G0548	6.67	6.92	CDK1	Cyclin-dependent kinase 1	NM_212564.2
B5G1308	6.67	8.74	CDCA2	Cell division cycle-associated protein 2	XM_068223540.1
A6G0792	6.67	3.44	SHQ1	Protein SHQ1 homolog	NM_001080600.2
B3G0455	6.67	3.25	PGLS	6-phosphogluconolactonase	NM_001004117.3
B16G0925	6.67	2.73	CD27	CD27 antigen	
A1G0828	6.67	5.97	ALKBH5	RNA demethylase ALKBH5	NM_001077387.1
B22G0948	6.67	19.84	ADD3	Gamma-adducin	XM_017353149.3
B20G0391	6.67	3.26	MRPL19	39S ribosomal protein L19, mitochondrial	NM_001003544.2
A11G0251	6.67	3.86	TAFA1	Chemokine-like protein TAFA-1	NM_001328585.1
A8G0851	6.67	5.91	ENTPD4	Ectonucleoside triphosphate diphosphohydrolase 4	XM_005167218.5
A2G1073	6.66	9.17	WDR48	WD repeat-containing protein 48	NM_214709.1
B25G0586	6.66	8.96	POLR2C	DNA-directed RNA polymerase II subunit RPB3	NM_001167956.2
B24G0326	6.66	4.39	FNDC3B	Fibronectin type III domain-containing protein 3B	XM_002666672.6
A23G0454	6.66	5.25	NOL9	Polynucleotide 5'-hydroxyl-kinase NOL9	XM_691014.7
A2G0132	6.66	0.84			
A2G0396	6.66	19.97	F3	Tissue factor	NM_001017728.2
A2G0941	6.66	7.90	ESYT2-A	Extended synaptotagmin-2-A	XM_693440.10
B3G1737	6.66	1.07	LOC113077176	A0A6P6NA69_CARAU uncharacterized protein LOC113077176	
B5G0409	6.66	4.66	UFC1	Ubiquitin-fold modifier-conjugating enzyme 1	NM_001003650.1
B14G0678	6.66	14.06	RNF20	E3 ubiquitin-protein ligase BRE1A	NM_001256175.1
B20G0230	6.66	2.15	ITPA	Inosine triphosphate pyrophosphatase	NM_001099986.2
B6G0581	6.66	6.62	AP1M1	AP-1 complex subunit mu-1	XM_021476834.2
A5G0446	6.66	1.29	MARVELD2	MARVEL domain-containing protein 2	NM_001126406.1
A4G0502	6.66	5.23	LOC113048243	A0A6P6K3E4_CARAU uncharacterized protein LOC113048243 isoform X4	
A25G0488	6.66	7.73	SEMA7A	Semaphorin-7A	NM_001328508.1
A12G0430	6.66	18.12	CALCOCO2	Calcium-binding and coiled-coil domain-containing protein 2	NM_001020741.2
B13G0419	6.66	4.49	URB2	Unhealthy ribosome biogenesis protein 2 homolog	NM_001076727.2
B18G0794	6.66	11.13	NEDD4	E3 ubiquitin-protein ligase NEDD4	XM_068214864.1

B23G0349	6.66	8.54	CCDC115	Coiled-coil domain-containing protein 115	NM_001013295.2
A16G0694	6.66	8.52	ABHD16A	Phosphatidylserine lipase ABHD16A	NM_001111178.1
A17G0416	6.65	15.49	ACSS1	Acetyl-coenzyme A synthetase 2-like, mitochondrial	NM_001080656.3
B4G0358	6.65	3.55	IRF5	Interferon regulatory factor 5	NM_001327817.1
A14G0377	6.65	7.91	SLC30A9	Zinc transporter 9	NM_001008575.1
B4G0396	6.65	12.49	UBE2H	Ubiquitin-conjugating enzyme E2 H	NM_201489.1
A3G0334	6.65	1.58	TMED1	Transmembrane emp24 domain-containing protein 1	NM_001003487.1
B4G0342	6.65	4.04	VHL	von Hippel-Lindau disease tumor suppressor	
B2G0162	6.65	6.69	EXOC3	Exocyst complex component 3	NM_212715.1
B5G0391	6.65	10.70	PJA2	E3 ubiquitin-protein ligase Praja-2	XM_009301875.3
A6G0748	6.65	17.31	CISH	Cytokine-inducible SH2-containing protein	NM_001076617.2
B2G0390	6.65	4.14	TOX4-B	TOX high mobility group box family member 4-B	XM_021471257.2
B25G0176	6.65	3.24	IGKV1-27	Immunoglobulin kappa variable 1-27	
A16G1008	6.65	10.19	PREB	Prolactin regulatory element-binding protein	NM_001002638.1
B19G0941	6.65	6.32	PPT2-A	Lysosomal thioesterase PPT2-A	NM_001109859.2
B1G1093	6.65	12.46	NLRC3	NLR family CARD domain-containing protein 3	XM_068222171.1
A19G0605	6.64	5.33	IRGC	Interferon-inducible GTPase 5	NM_001320423.1
B17G0864	6.64	4.61	NUMB	Protein numb homolog	XM_005169895.5
B10G0055	6.64	5.56	NFIL3	Nuclear factor interleukin-3-regulated protein	NM_001004120.3
B6G0821	6.64	3.96	G5714_007275	A0A7J6CZU9_9TELE Uncharacterized protein	XM_005172902.5
A25G0162	6.64	3.87	SERGEF	Secretion-regulating guanine nucleotide exchange factor	XM_068219113.1
A12G0631	6.64	6.55	VPS35L	VPS35 endosomal protein-sorting factor-like	NM_001098181.1
A12G0503	6.64	3.91	ATPAF2	ATP synthase mitochondrial F1 complex assembly factor 2	NM_001082821.1
B20G0278	6.64	1.51	KCTD1	POZ domain-containing protein KCTD1	XM_009294626.3
B9G0577	6.64	5.00	RNASEH2B	Ribonuclease H2 subunit B	NM_200271.1
A6G0627	6.64	5.59	NIPA2	Magnesium transporter NIPA2	XM_005165975.5
B1G0494	6.64	33.76	TM9SF2	Transmembrane 9 superfamily member 2	NM_212728.1
A24G0356	6.64	2.00	G5714_023412	A0A7J6BL67_9TELE Uncharacterized protein	XM_021473784.2
A8G0741	6.63	1.39	RTASE	Probable RNA-directed DNA polymerase from transposon BS	XM_068223275.1
B6G0668	6.63	8.35	CREB1	Cyclic AMP-responsive element-binding protein 1	NM_199723.1
B12G0208	6.63	6.17	NARF	Nuclear prelamin A recognition factor	NM_001002342.1
B2G0266	6.63	6.95	TMEM55BA	Type I phosphatidylinositol 4,5-bisphosphate 4-phosphatase-A	NM_001037376.2
B25G0310	6.63	128.67	TPM3	Tropomyosin alpha-3 chain	XM_009298156.4
B25G0366	6.62	2.98	PTPRZ1	Receptor-type tyrosine-protein phosphatase zeta	XM_009298099.4
A5G0558	6.62	3.11	P2RX1	P2X purinoceptor 1	NM_198982.1
B23G0983	6.62	10.43	MEPCE	7SK snRNA methylphosphate capping enzyme	XM_001921694.6

A14G0721	6.62	8.26	PRELID1	PRELI domain-containing protein 1, mitochondrial	NM_200366.2
B4G0378	6.62	5.51	PLXNB2	Plexin-B2	XM_009300284.4
A7G1047	6.62	2.46	HACD4	Very-long-chain (3R)-3-hydroxyacyl-CoA dehydratase 4	NM_001200011.2
A17G0590	6.62	8.74	VRK1	threonine-protein kinase VRK1	NM_213385.1
B1G0770	6.62	11.10	EIF5B	Eukaryotic translation initiation factor 5B	XM_009293741.4
A23G0122	6.62	5.24	MGAT4C	Alpha-1,3-mannosyl-glycoprotein 4-beta-N-acetylglucosaminyltransferase C	
A12G0207	6.62	1.98	MRPL43	39S ribosomal protein L43, mitochondrial	NM_001002428.2
B20G0639	6.62	11.50	LBR	Delta(14)-sterol reductase LBR	XM_021468371.2
B1G0937	6.62	1.41	CAPN9	Calpain-9	NM_001003501.1
A15G0387	6.62	1.46	SLC46A1	Proton-coupled folate transporter	NM_200285.1
B10G0638	6.62	4.58	DCPS	m7GpppX diphosphatase	NM_205588.3
B25G0269	6.62	6.29	HEXA	Beta-hexosaminidase subunit alpha	XM_021470488.2
B6G0185	6.61	12.81	UBE2G2	Ubiquitin-conjugating enzyme E2 G2	NM_001017767.1
B23G0755	6.61	4.13	CDA	Cytidine deaminase	NM_001351715.1
B14G0395	6.61	5.33	PRPF39	Pre-mRNA-processing factor 39	XM_002664461.6
A16G0421	6.61	12.83	RBP1	Retinol-binding protein 1	NM_199528.3
B3G0802	6.61	5.88	GTPBP1	GTP-binding protein 1	XM_005163999.5
A22G0484	6.61	6.95	CDC34	Ubiquitin-conjugating enzyme E2 R1	NM_200958.1
A16G0635	6.61	3.97	GTPBP10	GTP-binding protein 10	
A7G0744	6.60	2.20	WDR76	WD repeat-containing protein 76	
A10G0837	6.60	1.85	CDC42SE2	CDC42 small effector protein 2	NM_001159835.2
A8G0325	6.60	3.89	DMAP1	DNA methyltransferase 1-associated protein 1	NM_200255.1
A2G0423	6.60	16.10	IVNS1ABPB	Influenza virus NS1A-binding protein homolog B	NM_201483.1
A15G0591	6.60	9.21	RELB	Transcription factor RelB	XM_001335557.6
B7G0725	6.60	5.87	RNF111-C	E3 ubiquitin-protein ligase arkadia-C	XM_005168924.5
B5G1155	6.60	47.96	ACSL4	Long-chain-fatty-acid--CoA ligase 4	NM_001099739.1
B17G0373	6.60	12.58	HTRA1A	Serine protease HTRA1A	NM_001002219.1
B24G0425	6.60	9.17	APOO	MICOS complex subunit MIC26	
B3G1122	6.60	6.17	SPOP	Speckle-type POZ protein	NM_201130.2
A4G0777	6.60	0.04			
A4G0441	6.60	6.10	TRIM24	Transcription intermediary factor 1-alpha	NM_001002870.2
A11G0072	6.60	4.34	SLC29A1	Equilibrative nucleoside transporter 1	XM_005155659.5
A1G0272	6.60	12.47	GET3	ATPase GET3	NM_001002298.2
B22G0189	6.60	3.27	A0A671Q7M2	A0A671Q7M2_9TELE Zgc:194627	NM_001128805.1
A21G0259	6.59	5.14	EDEM3	ER degradation-enhancing alpha-mannosidase-like protein 3	XM_003200833.6
B3G1184	6.59	6.34	MRTFA	Myocardin-related transcription factor A	XM_021473680.2
A7G0784	6.59	12.16	LOC107741393	A0A673IWD1_9TELE Reticulocyte-binding protein 2 homolog a-like	NM_001386784.1
A1G1005	6.59	4.49	CLOCK	Circadian locomoter output cycles protein kaput	NM_178295.2

B9G0061	6.59	4.18	RNH1	Ribonuclease inhibitor	XM_068213791.1
A1G0037	6.59	8.17	VAV1	Proto-oncogene vav	
B8G0766	6.59	8.09	EPS15	Epidermal growth factor receptor substrate 15	XM_002663099.7
B19G0068	6.59	7.12	CLPTM1L	Lipid scramblase CLPTM1L	NM_001002380.1
A2G0247	6.59	7.78	RAB18B	Ras-related protein Rab-18-B	NM_001003449.1
A3G0952	6.59	6.48	DTX3	Probable E3 ubiquitin-protein ligase DTX3	XM_021471387.2
A12G0529	6.59	74.23	ACTA2	Actin, aortic smooth muscle	NM_212620.2
B7G0705	6.59	4.94	CARS1	Cysteine--tRNA ligase, cytoplasmic	NM_001118900.1
B1G0313	6.59	7.25	PTPN9	Tyrosine-protein phosphatase non-receptor type 9	XM_685567.10
A2G0638	6.59	7.85	STAM	Signal transducing adapter molecule 1	NM_200120.1
A23G0637	6.59	5.42	SRD5A1	3-oxo-5-alpha-steroid 4-dehydrogenase 1	XM_021470018.2
B15G0802	6.59	5.69			XM_021481446.2
B7G0193	6.59	7.11	DHCR7	7-dehydrocholesterol reductase	NM_201330.2
B16G1134	6.59	7.46	CERS2	Ceramide synthase 2	XM_688576.8
B21G0687	6.58	1.59	CLIC4	Chloride intracellular channel protein 4	NM_199524.1
B20G0893	6.58	3.06	TAF1A	TATA box-binding protein-associated factor RNA polymerase I subunit A	NM_001045274.1
A14G0610	6.58	6.76	ARL9	ADP-ribosylation factor-like protein 9	
B6G0375	6.58	7.13	GGA1	ADP-ribosylation factor-binding protein GGA1	NM_001004000.2
A5G0417	6.58	8.94	ABCA2	ATP-binding cassette sub-family A member 2	XM_068221438.1
B15G0197	6.58	15.13	SIRT2	NAD-dependent protein deacetylase sirtuin-2	NM_199596.1
A14G0333	6.58	2.83	NIPA2	Magnesium transporter NIPA2	XM_021481008.2
B14G0574	6.58	10.96	NPM1	Nucleophosmin	NM_001166155.1
A16G0528	6.58	4.39	INO80C	INO80 complex subunit C	NM_001002108.2
B20G0889	6.58	1.02	FAM177A1	Protein FAM177A1	XM_009295012.4
A23G0407	6.58	4.11	SPSB1	SPRY domain-containing SOCS box protein 1	NM_001025460.1
A17G0788	6.58	0.05	DNAJC5	DnaJ homolog subfamily C member 5	
A7G0217	6.58	4.56	BTBD10	POZ domain-containing protein 10	XM_021472027.2
B21G0770	6.58	5.07	F2RL1	Proteinase-activated receptor 2	
A21G0654	6.58	13.11	MAT2B	Methionine adenosyltransferase 2 subunit beta	NM_001013474.2
A9G0481	6.57	7.65	NDUFA10	NADH dehydrogenase [ubiquinone] 1 alpha subcomplex subunit 10, mitochondrial	NM_199578.1
A11G0758	6.57	3.63	NR1D1	Nuclear receptor subfamily 1 group D member 1	NM_001309792.1
B14G0176	6.57	7.43	FIBPB	Acidic fibroblast growth factor intracellular-binding protein B	NM_200409.1
B15G0377	6.57	10.32	ARCN1	Coatomer subunit delta	NM_201993.1
A18G0763	6.57	3.08	UGT2A2	UDP-glucuronosyltransferase 2A2	NM_001045386.1
B1G0312	6.57	3.59	EXOSC9	Exosome complex component RRP45	NM_001006077.1
B18G0049	6.57	4.02	RBM7	RNA-binding protein 7	NM_199925.1
A23G0228	6.57	4.25	ADRM1B	Proteasomal ubiquitin receptor ADRM1	XM_005161962.5

B6G0519	6.57	11.39	TMEM131	Transmembrane protein 131	XM_001923305.7
B1G0210	6.57	16.75	ATNB233	potassium-transporting ATPase subunit beta-233	NM_131671.1
B14G0044	6.57	10.05	RTASE	Probable RNA-directed DNA polymerase from transposon BS	XM_068214189.1
B5G0349	6.57	11.52	ALAD	Delta-aminolevulinic acid dehydratase	NM_001017645.1
B21G0151	6.57	39.87	SRSF1B	arginine-rich splicing factor 1B	XR_659427.4
A23G0058	6.57	8.99	SMIM29	Small integral membrane protein 29	
B20G0892	6.57	9.76	MIA3	Transport and Golgi organization protein 1 homolog	XM_005160894.5
B19G0401	6.57	2.42	COLQ	Acetylcholinesterase collagenic tail peptide	XM_001343109.9
A19G0856	6.56	5.94	GGCT	Gamma-glutamylcyclotransferase	NM_001144818.1
A18G0413	6.56	2.15	LOC107694935	A0A671M120_9TELE Translin-associated factor X-interacting protein 1-like	
A18G0811	6.56	8.39	WHAMM	WASP homolog-associated protein with actin, membranes and microtubules	XM_021467715.2
A11G0003	6.56	2.60	FILIP1L	Filamin A-interacting protein 1-like	
B15G0291	6.56	16.91	PNKP	kinase	XM_005173382.5
A6G0444	6.56	11.32	BRDT	Bromodomain testis-specific protein	XM_068221653.1
B21G0419	6.56	1.23	NIPAL4	Magnesium transporter NIPA4	NM_001002664.1
B14G0799	6.56	10.89	LOC113113565	A0A6P6QQC1_CARAU uncharacterized protein LOC113113565	XM_001344090.8
B13G0403	6.56	4.87	ASRGL1	L-asparaginase	NM_131521.2
A25G0635	6.56	4.90	IGKV4-1	Immunoglobulin kappa variable 4-1	XM_068217596.1
B20G0354	6.55	6.87	LNX1	E3 ubiquitin-protein ligase LNX	XM_005160506.5
A3G0964	6.55	6.03	SLC35B1	Solute carrier family 35 member B1	NM_001004583.1
A14G0244	6.55	5.77	STARD10	START domain-containing protein 10	NM_200220.1
A25G0053	6.55	7.71	HDHD5	Haloacid dehalogenase-like hydrolase domain-containing 5	NM_001328522.1
B19G0754	6.55	4.41	GON4L	GON-4-like protein	
B2G1099	6.55	8.99	RC3H1	Roquin-1	XM_009297322.4
B2G0203	6.55	1.20	GPR55	G-protein coupled receptor 55	XM_068214727.1
A13G0492	6.55	2.49	PGBD5	PiggyBac transposable element-derived protein 5	XM_009307400.4
A22G0202	6.55	4.02	NLRP3	NACHT, LRR and PYD domains-containing protein 3	XM_021479924.2
B8G0658	6.55	8.48	FZR1	Fizzy-related protein homolog	NM_200253.1
A18G0406	6.55	3.72	CARMIL3	3 and myosin-I linker protein 3	NM_001317766.1
B9G0756	6.55	9.51	TSC22D1	TSC22 domain family protein 1	NM_001328125.1
A19G0259	6.55	2.67	YARS1	Tyrosine--tRNA ligase, cytoplasmic	XM_005159598.5
B3G1237	6.55	8.45	CASC3	Protein CASC3	XM_068216300.1
A3G0641	6.55	5.52	TIMM29	Mitochondrial import inner membrane translocase subunit Tim29	NM_001020561.1
A19G0459	6.55	8.11	PPT1	Palmitoyl-protein thioesterase 1	NM_213339.1
A3G1104	6.55	9.29	CBX8	Chromobox protein homolog 8	NM_205616.2
B14G0188	6.55	8.56	FOXO4	Forkhead box protein O4	XM_009291168.4
A19G0900	6.55	5.17	GLMP	Glycosylated lysosomal membrane protein	NM_001004580.1

A10G0858	6.55	12.81	DCP2	m7GpppN-mRNA hydrolase	NM_200152.1
A6G0624	6.54	8.71	SLC9A7	hydrogen exchanger 7	NM_001030077.2
B9G0520	6.54	5.28	ESD	S-formylglutathione hydrolase	NM_001017796.2
A6G0436	6.54	15.34	GADD45A	Growth arrest and DNA damage-inducible protein GADD45 alpha	NM_001002216.1
B1G1134	6.54	4.73	LOC113099796	A0A6P6PHG1_CARAU zinc finger protein 271-like	XM_017357165.3
A1G0458	6.54	11.73	UNC93B1	Protein unc-93 homolog B1	NM_001288816.1
A8G0454	6.54	5.80	CCDC22	Coiled-coil domain-containing protein 22	NM_001025485.1
A7G1217	6.54	0.66	RIN2	Ras and Rab interactor 2	XM_005166310.5
A2G0923	6.54	4.12			
A7G0978	6.54	2.26	WRAP53	Telomerase Cajal body protein 1	NM_001256028.1
B22G0672	6.54	3.63	TM2D1	TM2 domain-containing protein 1	XM_068216222.1
B24G0429	6.54	4.23	ABHD10	Palmitoyl-protein thioesterase ABHD10, mitochondrial	NM_001386473.1
B5G0735	6.53	6.69	CDS1	Phosphatidate cytidylyltransferase 1	XM_001341863.8
A25G0232	6.53	26.68	CCL8	C-C motif chemokine 8	NM_001129894.1
B11G0238	6.53	11.67	FAM174C	Protein FAM174C	NM_001020583.1
A12G0168	6.53	10.39	ST6GALNAC2	Alpha-N-acetylgalactosaminide alpha-2,6-sialyltransferase 2	XM_009306854.4
B13G0083	6.53	9.11	PERP	p53 apoptosis effector related to PMP-22	XM_021480901.2
B5G0186	6.53	13.21	RFC5	Replication factor C subunit 5	NM_001003862.1
B22G0331	6.53	6.30	LOC113082914	A0A6P6NNE1_CARAU carcinoembryonic antigen-related cell adhesion molecule 3-like	XM_021470864.2
B20G0617	6.53	1.57	SCCPDH	Saccharopine dehydrogenase-like oxidoreductase	NM_001005574.1
A19G0254	6.53	6.08	CYRIA	CYFIP-related Rac1 interactor A	NM_001003606.3
A10G0115	6.53	5.66	CDK2AP1	Cyclin-dependent kinase 2-associated protein 1	
B3G0976	6.53	2.41	ALG1	Chitobiosyldiphosphodolichol beta-mannosyltransferase	NM_199867.1
B22G1045	6.53	10.69	ATP11B	Phospholipid-transporting ATPase IF	XM_021473554.2
A5G1037	6.53	9.32	RABEP1	Rab GTPase-binding effector protein 1	NM_001123287.2
B17G0758	6.53	5.38	MRPL35	39S ribosomal protein L35, mitochondrial	
B9G0321	6.53	10.59	CASK	Peripheral plasma membrane protein CASK	XM_017357627.3
B18G0856	6.53	6.83	HDGFL3	Hepatoma-derived growth factor-related protein 3	NM_001012372.1
A12G0026	6.52	9.17	FH	Fumarate hydratase, mitochondrial	NM_200963.1
B22G0543	6.52	4.85	USE1	Vesicle transport protein USE1	NM_200324.2
B3G0617	6.52	8.01	EPN2	Epsin-2	XM_681373.9
A6G0378	6.52	3.81	CEP95	Centrosomal protein of 95 kDa	
A21G0231	6.52	8.17	UBAP2	Ubiquitin-associated protein 2	NM_001082838.2
A4G0011	6.52	8.71	USP15	Ubiquitin carboxyl-terminal hydrolase 15	XM_068217770.1
A7G0870	6.52	6.55	EXOC6B	Exocyst complex component 6B	XM_021477610.2
A5G0948	6.52	3.16	PMPCA	Mitochondrial-processing peptidase subunit alpha	NM_001007442.1
A18G0454	6.52	5.67	TLE3A	Transducin-like enhancer protein 3-A	NM_001313694.1

A1G0738	6.52	5.39	BTN3A2	Butyrophilin subfamily 3 member A2	
A12G0195	6.52	6.11	BIRC5.2	Baculoviral IAP repeat-containing protein 5.2	NM_194397.3
B18G0394	6.52	6.95	LOC113118496	A0A6P6RE82_CARAU uncharacterized protein LOC113118496	NM_001083062.2
B24G0650	6.51	7.38	GALNT11	Polypeptide N-acetylgalactosaminyltransferase 11	NM_001076562.2
A14G0590	6.51	5.28	FNTA	geranylgeranyltransferase type-1 subunit alpha	NM_001080560.2
A24G0133	6.51	3.47	NLRC3	NLR family CARD domain-containing protein 3	XM_009297629.4
B5G0075	6.51	9.00	GGNBP2	Gametogenetin-binding protein 2	NM_001079961.2
B4G0341	6.51	12.71	TMBIM4	Protein lifeguard 4	NM_213138.2
A11G0781	6.51	7.96	G5714_011554	A0A7J6CIW1_9TELE Uncharacterized protein	NM_001110486.1
B14G0416	6.51	4.94	SETD7	Histone-lysine N-methyltransferase SETD7	XR_658247.4
A10G0080	6.51	3.85	MARVELD2	MARVEL domain-containing protein 2	XM_009305076.4
B16G0979	6.51	21.96	ZG16	Zymogen granule membrane protein 16	
B16G0345	6.51	4.12	ELP6	Elongator complex protein 6	NM_001103142.1
A10G0779	6.51	6.40	PIP5K1B	Phosphatidylinositol 4-phosphate 5-kinase type-1 beta	XM_068223777.1
B17G0385	6.51	1.63	AHNAK	Neuroblast differentiation-associated protein AHNAK	XM_068214583.1
B17G0862	6.51	6.70	VPS39	Vps39-like protein	NM_001113540.2
A8G0439	6.51	2.09	SLC17A9	Solute carrier family 17 member 9	XM_001336538.7
A16G0509	6.51	4.98	TBRG4	FAST kinase domain-containing protein 4	XM_685771.9
B16G0405	6.51	5.09	RHBDL2	Rhomboid-related protein 2	NM_201204.1
B20G0669	6.50	7.05	ANGEL2	Protein angel homolog 2	NM_001029960.1
A15G0541	6.50	12.52	PICALM	Phosphatidylinositol-binding clathrin assembly protein	XM_009291635.4
A12G0001	6.50	19.19	GHITM	Growth hormone-inducible transmembrane protein	
A19G0603	6.50	2.59	KV12	Ig kappa chain V region 3368	
B25G0418	6.50	3.97	FBXL14	LRR-repeat protein 14	NM_201482.1
A23G0271	6.50	8.70	COMMD7	COMM domain-containing protein 7	NM_001003577.1
B20G0238	6.50	5.17	MYSM1	Histone H2A deubiquitinase MYSM1	NM_001164029.1
B16G0364	6.50	9.17	MAP7D1	MAP7 domain-containing protein 1	XM_068213986.1
A13G0814	6.50	3.71	MCPH1	Microcephalin	XM_021480901.2
B5G0419	6.50	18.46	G5714_006100	A0A7J6D2X1_9TELE Uncharacterized protein	
B16G0439	6.49	3.22	LENG1	Leukocyte receptor cluster member 1 homolog	NM_001004591.1
B7G0885	6.49	5.16	RGP1	RAB6A-GEF complex partner protein 2	NM_001002608.1
A20G0726	6.49	7.63	FAM20B	Glycosaminoglycan xylosylkinase	NM_001044818.1
A24G0434	6.49	24.99	MYD88	Myeloid differentiation primary response protein MyD88	NM_212814.2
A14G0268	6.49	64.61	CLIC2	Chloride intracellular channel protein 2	NM_001002561.2

B13G0872	6.49	14.94	VENT1	Homeobox protein vent1	XM_068218698.1
A15G0216	6.49	7.81	NLRP3	NACHT, LRR and PYD domains-containing protein 3	XM_021466485.2
A1G0891	6.48	5.35	KNSTRN	Small kinetochore-associated protein	NM_001128774.1
A16G0225	6.48	8.36	SMARCC1	SNF complex subunit SMARCC1	XM_003200246.6
A3G0405	6.48	9.18	NPRL3	GATOR complex protein NPRL3	NM_001025534.2
A16G0309	6.48	2.02	MYO1E	Unconventional myosin-Ie	NM_200636.1
A24G0171	6.48	3.69	ABCD4	Lysosomal cobalamin transporter ABCD4	NM_001076717.2
A5G0068	6.48	2.27	ZNHIT3	Zinc finger HIT domain-containing protein 3 (Fragment)	NM_200273.1
B1G0969	6.48	5.79	PI4K2A	Phosphatidylinositol 4-kinase type 2-alpha	NM_213358.2
A3G0444	6.48	13.75	PDPK1	3-phosphoinositide-dependent protein kinase 1	XM_068217664.1
A24G0253	6.48	2.65	PRMT6	Protein arginine N-methyltransferase 6	NM_001163988.3
B15G0499	6.48	4.70	TMEM97	Sigma intracellular receptor 2	
B12G0567	6.48	2.00	MED1	Mediator of RNA polymerase II transcription subunit 1	XM_021480457.2
B21G0670	6.48	0.93	GLUL	Glutamine synthetase	XM_009295250.4
A7G0809	6.48	3.89	RRAS2	Ras-related protein R-Ras2	NM_001017815.1
B1G0386	6.48	6.70	SMIM14	Small integral membrane protein 14	
A5G0700	6.48	8.83	SPTBN1	Spectrin beta chain, non-erythrocytic 1	NM_001423118.1
B1G0034	6.48	4.43	TPP2	Tripeptidyl-peptidase 2	XM_005168011.5
B22G0556	6.47	9.05	PCYT2	Ethanolamine-phosphate cytidylyltransferase	NM_001006037.1
A19G0896	6.47	9.58	G5714_024648	A0A7J6BHK8_9TELE IG domain-containing protein	
B13G0511	6.47	2.62	WBP1L	WW domain binding protein 1-like	NM_001083564.1
A18G0177	6.47	20.73	ARPP19	cAMP-regulated phosphoprotein 19	NM_200630.1
A7G0439	6.47	5.46	VPS33B	Vacuolar protein sorting-associated protein 33B	NM_001014348.1
A2G0485	6.47	2.61	RPP40	Ribonuclease P protein subunit p40	NM_001002148.1
B22G0949	6.47	13.37	MXI1	Max-interacting protein 1	NM_001308638.1
B1G0612	6.47	2.41	IRF2	Interferon regulatory factor 2	NM_001326712.1
A5G0607	6.47	6.68	KYAT1	Kynurenine--oxoglutarate transaminase 1	NM_213359.2
A23G0021	6.47	10.85	SMC1A	Structural maintenance of chromosomes protein 1A	NM_212810.2
A14G0194	6.47	3.67	IL2RG	Cytokine receptor common subunit gamma	XM_009291083.4
A3G1221	6.47	4.38	CANT1	Soluble calcium-activated nucleotidase 1	XM_005171642.4
A5G0970	6.47	2.66	AK5	Adenylate kinase isoenzyme 5	NM_001017540.2
B7G0135	6.47	14.12	AP1G1	AP-1 complex subunit gamma-1	XM_068222168.1
B14G0618	6.47	10.61	HOPX	Homeodomain-only protein	NM_198209.2
A8G0823	6.47	8.55	FKBP15	FK506-binding protein 15	XM_021478508.2
A22G0651	6.46	7.30	DAG1	Dystroglycan 1	XM_009296487.4
B17G0802	6.46	4.98	MOCS1	Molybdenum cofactor biosynthesis protein 1	XM_009293238.4
B16G0094	6.46	6.75	LOC113115661	A0A6P6R269_CARAU uncharacterized protein LOC113115661	

B5G0167	6.46	4.98	HSCB	Iron-sulfur cluster co-chaperone protein HscB	NM_001128398.1
A13G0569	6.46	3.40	SRBD1	S1 RNA-binding domain-containing protein 1	XM_021480607.2
B18G0601	6.46	11.81	RIC8B	Synembryn-B	NM_001005293.1
A16G1012	6.46	4.77	VEGFAA	Vascular endothelial growth factor A-A	NM_131408.3
B10G0812	6.46	9.08	ASH2L	Ash2 histone methyltransferase complex subunit ASH2	NM_001110105.2
B21G0013	6.46	4.01	GALNT10	Polypeptide N-acetylgalactosaminyltransferase 10	XM_009298402.3
A5G0853	6.46	6.54	RASA1	Ras GTPase-activating protein 1	XM_068221515.1
B7G1252	6.46	2.47	LTB4R	Leukotriene B4 receptor 1	NM_001308973.1
A4G0018	6.46	12.49	ARFGAP3	ADP-ribosylation factor GTPase-activating protein 3	NM_205597.1
B16G0191	6.46	19.95	SYNDIG1L	Synapse differentiation-inducing gene protein 1-like	NM_001386409.1
A7G1205	6.45	18.81	CXCL8	Interleukin-8	
A8G0826	6.45	2.13	TCF7L1B	Transcription factor 7-like 1-B	NM_131296.2
B12G0820	6.45	3.96	NXPE3	NXPE family member 3	XM_068213769.1
B6G0234	6.45	9.50	LNPKB	Endoplasmic reticulum junction formation protein lunapark-B	NM_001159912.1
A16G0821	6.45	4.02	FKBP14	Peptidyl-prolyl cis-trans isomerase FKBP14	NM_001003471.1
A5G1201	6.45	3.37	RWDD2B	RWD domain-containing protein 2B	NM_001075115.2
A5G0054	6.45	5.57	VKORC1L1	Vitamin K epoxide reductase complex subunit 1-like protein 1	NM_001020688.1
A14G0567	6.45	14.09	NPM1	Nucleophosmin	NM_001166155.1
A1G0308	6.45	15.01	HERC3	Probable E3 ubiquitin-protein ligase HERC3	NM_001145624.1
A12G0823	6.45	8.39	USP22	Ubiquitin carboxyl-terminal hydrolase 22	XM_021480159.2
B17G0394	6.45	3.88	ZNF318	Zinc finger protein 318	XM_021474368.2
A14G0616	6.45	9.52	CETN1	Centrin-1	XM_002667181.7
B2G0131	6.44	8.73	ABHD4	(Lyso)-N-acylphosphatidylethanolamine lipase	NM_001017613.2
A16G0925	6.44	8.02	PRPF3	U6 small nuclear ribonucleoprotein Prp3	NM_205748.2
A16G0008	6.44	8.90	KHDC4	KH homology domain-containing protein 4	XM_005169775.5
B4G0627	6.44	5.43	ALG12	Dol-P-Man:Man(7)GlcNAc(2)-PP-Dol alpha-1,6-mannosyltransferase	NM_001098749.1
B12G0270	6.44	3.92	TRUB1	Pseudouridylate synthase TRUB1	NM_001128687.1
A12G0092	6.44	1.08	LOC113076502	A0A6P6N9P7_CARAU uncharacterized protein LOC113076502 isoform X1	
B22G0504	6.44	4.04	RPP14	Ribonuclease P protein subunit p14	NM_001020692.2
B5G0469	6.44	17.80	BCKDK	[3-methyl-2-oxobutanoate dehydrogenase [lipoamide]] kinase, mitochondrial	NM_213060.2
A24G0428	6.44	2.21	NFI1	CTD small phosphatase-like protein	XM_005162710.5
B19G0726	6.44	7.59	NSUN2	RNA cytosine C(5)-methyltransferase NSUN2	NM_199711.1
B14G0724	6.44	8.70	SLBP	Histone RNA hairpin-binding protein	

B24G0565	6.44	4.32	UBE2WB	Probable ubiquitin-conjugating enzyme E2 W-B	NM_001045293.2
A1G0296	6.44	2.75	JAG1A	Protein jagged-1a	NM_131861.1
B6G1023	6.44	18.81	SHMT2	Serine hydroxymethyltransferase, mitochondrial	NM_001123374.1
A17G0404	6.44	6.30	TTC7B	Tetratricopeptide repeat protein 7B	XM_009293100.4
A18G0684	6.44	14.88	FKBP4	Peptidyl-prolyl cis-trans isomerase FKBP4	NM_201469.1
B19G0739	6.44	5.30	GTF2H4	General transcription factor IIH subunit 4	NM_199927.1
A2G1114	6.43	14.44	PLPP2	Phospholipid phosphatase 2	XM_687169.10
A5G0725	6.43	7.27	A0A671S8K4	A0A671S8K4_9TELE RBR-type E3 ubiquitin transferase	NM_001386836.1
B18G0534	6.43	12.91	NLRC5	Protein NLRC5	NM_001386270.1
A13G0789	6.43	17.00	MEIS1	Homeobox protein Meis1	NM_001030175.1
B9G0328	6.43	14.37	USP9X	Probable ubiquitin carboxyl-terminal hydrolase FAF-X	XM_005167736.5
B3G0332	6.43	7.22	XPO6	Exportin-6	NM_194374.1
A25G0470	6.43	11.98	AASS	Alpha-aminoadipic semialdehyde synthase, mitochondrial	NM_001173985.1
A25G0343	6.43	30.19	CAV1	Caveolin-1	NM_212651.1
A17G0288	6.43	10.06	CGREF1	Cell growth regulator with EF hand domain protein 1	XM_017351827.3
B18G0855	6.43	3.30	HOMER2	Homer protein homolog 2	NM_001020634.1
B19G0089	6.42	6.14	SLC44A4	Choline transporter-like protein 4	NM_200413.1
B6G0805	6.42	9.64	GXYLT2	Glucoside xylosyltransferase 2	NM_001110200.1
A23G0587	6.42	3.24			XM_021473733.2
B14G0589	6.42	8.32	IRF2	Interferon regulatory factor 2	NM_001320188.1
B17G0544	6.42	10.39	EHD4	EH domain-containing protein 4	NM_001386837.1
A14G0405	6.42	4.24	NDUFC1	NADH dehydrogenase [ubiquinone] 1 subunit C1, mitochondrial	
A19G0182	6.42	3.84	SDHAF3	Succinate dehydrogenase assembly factor 3, mitochondrial	NM_001005926.2
B1G0369	6.42	27.98	UCHL1	Ubiquitin carboxyl-terminal hydrolase isozyme L1	NM_201477.1
B14G0512	6.42	2.64	TBC1D2	TBC1 domain family member 2A	XM_684512.8
B10G0743	6.42	10.34	PURB	Transcriptional activator protein Pur-beta	NM_200547.1
A12G0802	6.42	12.33	PRKAR1A	cAMP-dependent protein kinase type I-alpha regulatory subunit	NM_001017732.1
A8G0829	6.42	4.70	ARID5A	AT-rich interactive domain-containing protein 5A	NM_001327774.1
A24G0518	6.42	3.06	TGIF1	Homeobox protein TGIF1	NM_199567.1
A9G0028	6.42	6.74	A33	Zinc-binding protein A33	NM_001089399.2
B14G0591	6.41	7.02	SNX25	Sorting nexin-25	NM_001163293.1
A24G0200	6.41	6.78	RMC1	Regulator of MON1-CCZ1 complex	NM_001362546.1
A16G0704	6.41	9.77	GSK3A	Glycogen synthase kinase-3 alpha	NM_131390.2
B10G0023	6.41	10.08	UBXN8	UBX domain-containing protein 8	
A5G0055	6.41	11.28	GUSB	Beta-glucuronidase	XM_009301129.4
A11G0034	6.41	5.53	CFAP410	Cilia- and flagella-associated protein 410	XM_017358231.3
A13G0334	6.41	1.77	GDF10	differentiation factor 10	NM_001145616.1

A15G0230	6.41	3.81	MRPL44	39S ribosomal protein L44, mitochondrial	NM_001030260.2
B17G0354	6.41	5.74	ABHD12	Lysophosphatidylserine lipase ABHD12	NM_001076597.1
A6G0676	6.41	10.70	PFKFB4	fructose-2,6-bisphosphatase 4	NM_198816.2
B18G0384	6.41	7.16	CD81	CD81 antigen	NM_001003735.1
A9G0604	6.41	5.09	PTTG1IP	Pituitary tumor-transforming gene 1 protein-interacting protein	XM_005167479.5
A14G0066	6.40	10.80	A0A673MJJ4	A0A673MJJ4_9TELE Uncharacterized protein	
A25G0342	6.40	12.53	CAV2	Caveolin-2	NM_001002150.1
B2G0115	6.40	7.28	TLE1	Transducin-like enhancer protein 1	NM_001045238.2
A12G0137	6.40	8.42	ABCA5	Cholesterol transporter ABCA5	NM_001099246.3
B15G0258	6.40	6.30	SLC7A1	High affinity cationic amino acid transporter 1	NM_001328209.1
A23G0708	6.40	6.04	CPSF3L	Integrator complex subunit 11	NM_001020621.1
A24G0122	6.40	3.00	CCSMST1	Protein ccsmst1	
B7G1209	6.40	0.78	CXCL8	Interleukin-8	
A12G0203	6.40	12.29	ARL3	ADP-ribosylation factor-like protein 3	NM_001044908.2
A14G0540	6.40	2.01	SH2D4A	SH2 domain-containing protein 4A	NM_001002626.1
B3G0665	6.40	3.96	PDXDC1	Pyridoxal-dependent decarboxylase domain-containing protein 1	XM_068216686.1
A19G0884	6.40	3.81	AZGP1	Zinc-alpha-2-glycoprotein	
A25G0283	6.40	9.17	DKK3	Dickkopf-related protein 3	NM_001089545.1
A6G0937	6.40	7.11	TUSC2	Tumor suppressor candidate 2	NM_001077299.2
A5G1234	6.40	3.75	SPR	Sepiapterin reductase	
A20G0439	6.40	4.88	ASAP2	Arf-GAP with SH3 domain, ANK repeat and PH domain-containing protein 2	NM_001044933.2
B8G0807	6.40	6.62	G5714_009459	A0A7J6CSC7_9TELE Uncharacterized protein	NM_001123261.1
A15G0641	6.40	16.89	TCAF	TRPM8 channel-associated factor homolog	XM_021481341.2
B2G1129	6.40	21.92	SCP2	Sterol carrier protein 2	NM_200865.2
A3G0313	6.40	9.66	MXE	Interferon-induced GTP-binding protein MxE	
A5G0639	6.40	9.62			
A5G0567	6.39	6.98	UAP1L1	UDP-N-acetylhexosamine pyrophosphorylase-like protein 1	NM_200294.1
B19G0836	6.39	9.69	RNF19A	E3 ubiquitin-protein ligase RNF19A	NM_001326695.1
A23G0566	6.39	4.25	CCDC50	Coiled-coil domain-containing protein 50	NM_001089533.2
A18G0517	6.39	12.03	TBC1D15	TBC1 domain family member 15	NM_001172625.1
B20G0895	6.39	7.78	HLX	H2.0-like homeobox protein	NM_212701.2
B5G1108	6.39	2.40	TRMT2A	tRNA (uracil-5-)-methyltransferase homolog A	NM_199929.1
B24G0201	6.39	0.57	TMEM14C	Transmembrane protein 14C	NM_001004610.2
B1G1183	6.39	3.72	GTF3C1	General transcription factor 3C polypeptide 1	XM_017357147.3
A14G0019	6.39	9.71	HS3ST1	Heparan sulfate glucosamine 3-O-sulfotransferase 1	XM_005173051.5
A5G0925	6.39	12.90	GLDC	Glycine dehydrogenase (decarboxylating), mitochondrial	NM_199554.1
A11G0359	6.39	7.52	NDRG3	Protein NDRG3	NM_001114928.2

B8G0868	6.39	3.84	BRPF3	Bromodomain and PHD finger-containing protein 3	XM_005166736.5
A2G1130	6.39	12.56	ANKRD12	Ankyrin repeat domain-containing protein 12	NM_200150.2
A5G1003	6.39	10.58	NAPA	Alpha-soluble NSF attachment protein	NM_001083561.2
A18G0008	6.38	6.07	ST7	Suppressor of tumorigenicity 7 protein homolog	NM_001040336.1
A18G0430	6.38	7.51	KMT5B	Histone-lysine N-methyltransferase KMT5B	NM_001007337.2
A21G0623	6.38	5.40	SLC7A3	Cationic amino acid transporter 3	NM_001007329.2
A9G0563	6.38	3.83	GDAP2	Ganglioside-induced differentiation-associated protein 2	NM_001004563.1
B5G0401	6.38	0.68	A0A671Q8V4	A0A671Q8V4_9TELE Zgc:193711	NM_001128753.1
B9G0538	6.38	1.29	TEX30	Testis-expressed protein 30	
B9G0882	6.38	10.90	CYTIP	Cytohesin-interacting protein	XM_003199175.6
B18G0084	6.38	10.73	KIFC3	Kinesin-like protein KIFC3	XM_009293853.4
A7G1221	6.38	15.54	CCS	Copper chaperone for superoxide dismutase	NM_001204222.2
B21G0636	6.38	4.98	GPN3	GPN-loop GTPase 3	NM_001007370.1
B22G0758	6.38	3.08	PIAS4A	E3 SUMO-protein ligase PIAS4-A	NM_213403.2
A4G0430	6.37	4.62	DPX16_3461	A0A3N0XKN8_ANAGA Zinc finger protein 568	XM_692772.10
B4G0258	6.37	5.21	DMTF1	Cyclin-D-binding Myb-like transcription factor 1	NM_001002665.1
A25G0760	6.37	6.20	CDK10	Cyclin-dependent kinase 10	NM_001017622.2
B23G0714	6.37	0.35	HOXC11A	Homeobox protein Hox-C11a	NM_131165.2
A7G0501	6.37	5.52	NAE1	NEDD8-activating enzyme E1 regulatory subunit	NM_200499.1
A24G0471	6.37	13.69	G5714_023534	A0A7J6BMJ9_9TELE Uncharacterized protein	XM_002666623.6
B1G0236	6.37	1.30	STRA6L	Stimulated by retinoic acid gene 6 protein-like	XM_005159799.5
A12G0217	6.36	7.17	CYBC1	Cytochrome b-245 chaperone 1 homolog	NM_001002669.1
B19G0862	6.36	2.45	TRNAU1AP	tRNA selenocysteine 1-associated protein 1	NM_001020609.1
A17G0489	6.36	5.55	SGMS1	Phosphatidylcholine:ceramide cholinephosphotransferase 1	NM_001007325.2
A10G0183	6.36	11.58	WDR45	WD repeat domain phosphoinositide-interacting protein 4	NM_200231.1
A5G0919	6.36	4.08	MALT1	Mucosa-associated lymphoid tissue lymphoma translocation protein 1	XM_685317.8
A11G0769	6.36	6.15	FAM217B	Protein FAM217B	XM_068224453.1
A17G0351	6.36	5.10	TRAF3	TNF receptor-associated factor 3	NM_001003513.1
A18G0144	6.36	11.85	TRIP12	E3 ubiquitin-protein ligase TRIP12	XM_068214800.1
B18G0172	6.36	11.01	MAPK6	Mitogen-activated protein kinase 6	NM_001045552.1
A12G0064	6.36	5.00	DLG5	Disks large homolog 5	XM_021480451.2
B2G1024	6.36	11.14	DLG1L	Discs large homolog 1-like protein	XM_009296932.4
B8G0853	6.36	6.80	TJP2	Tight junction protein ZO-2	XM_068222919.1
B7G0550	6.36	10.09	PTPRJ	Receptor-type tyrosine-protein phosphatase eta	NM_001136242.1
B6G0088	6.36	3.17	FAAH	Fatty-acid amide hydrolase 1	NM_001109825.1

A12G0297	6.36	4.60	PHKG2	testis isoform	NM_200962.1
A3G0654	6.36	5.20	GTF2F1	General transcription factor IIF subunit 1	NM_001423629.1
B13G0253	6.36	5.77	SLF2	SMC5-SMC6 complex localization factor protein 2	XM_005156968.5
A5G0409	6.36	8.62	AP1B1	AP-1 complex subunit beta-1	XM_005165166.4
A14G0217	6.35	8.25	TM9SF2	Transmembrane 9 superfamily member 2	NM_001423226.1
B11G0308	6.35	5.02	UBA3	NEDD8-activating enzyme E1 catalytic subunit	NM_001014295.2
B16G0387	6.35	2.21	RCC1	Regulator of chromosome condensation	NM_213178.1
B10G0352	6.35	7.32	PAXBP1	PAX3- and PAX7-binding protein 1	NM_001080183.2
B20G0584	6.35	2.86	SCYL3	Protein-associating with the carboxyl-terminal domain of ezrin	NM_199657.1
B21G0383	6.35	9.71	DPP3	Dipeptidyl peptidase 3	NM_001002683.1
A4G0985	6.35	3.92	MED21	Mediator of RNA polymerase II transcription subunit 21	NM_213423.1
A13G0860	6.35	8.70	HSP30	Heat shock protein 30	
A13G0589	6.35	5.80	CML1	Probable N-acetyltransferase CML1	NM_001123056.1
B3G0978	6.34	3.44	TTC30A	Tetratricopeptide repeat protein 30A	XM_021471716.2
B15G0717	6.34	4.69	PPT2	Lysosomal thioesterase PPT2	NM_200166.2
A7G0079	6.34	7.90	TCB2	Transposable element Tcb2 transposase	XM_068218059.1
B5G0456	6.34	6.27	LIG3	DNA ligase 3	NM_001030174.2
A21G0809	6.34	3.60	DPX16_18808	A0A3N0Y1U6_ANAGA Cadherin-related family member 5	
A5G0321	6.34	9.38	ZC4H2	Zinc finger C4H2 domain-containing protein	NM_199642.1
B24G0490	6.34	41.75	CSRNP1	serine-rich nuclear protein 1	XM_005162674.5
A21G0326	6.34	6.22	SMPD4	Sphingomyelin phosphodiesterase 4	NM_213355.1
A10G0214	6.34	11.12	GPAT4	Glycerol-3-phosphate acyltransferase 4	NM_001040249.2
A15G0333	6.34	3.38	MRPS23	28S ribosomal protein S23, mitochondrial	XM_005157688.5
A23G0072	6.34	1.64	GGT7	Glutathione hydrolase 7	XM_068216828.1
B17G0091	6.34	4.05	RUNX2	Runt-related transcription factor 2	NM_212858.2
A11G0005	6.34	5.47	NLRC3	Protein NLRC3	NM_001386526.1
A5G1329	6.34	9.07	ZBTB20	Zinc finger and BTB domain-containing protein 20	XM_068221291.1
A4G0853	6.34	4.67	CDC123	Cell division cycle protein 123 homolog	NM_200721.1
B24G0498	6.34	7.79	RELCH	RAB11-binding protein RELCH homolog	XM_017353809.3
A3G0372	6.34	6.08	ATP13A1	Endoplasmic reticulum transmembrane helix translocase	NM_001001403.2
B18G0336	6.34	6.68	SNRPA	U1 small nuclear ribonucleoprotein A	NM_199671.1
A8G0367	6.34	6.82	ZAP70	Tyrosine-protein kinase ZAP-70	NM_001020589.1
A11G0201	6.34	5.13	HOMER3	Homer protein homolog 3	NM_200485.2
A2G0551	6.34	32.31	ACADM	Medium-chain specific acyl-CoA dehydrogenase, mitochondrial	NM_213010.2
B20G0905	6.33	6.65	DSP	Desmoplakin	XM_021468722.2

A18G0392	6.33	3.10	YTX2	Transposon TX1 uncharacterized 149 kDa protein	
A1G0419	6.33	7.73	RNF24	RING finger protein 24	XM_005160065.5
A15G0820	6.33	4.87	CLDN4	Claudin-4	
B10G0579	6.33	21.32	ALOX5AP	Arachidonate 5-lipoxygenase-activating protein	NM_200061.2
A1G0189	6.33	3.22	SLC25A23	Calcium-binding mitochondrial carrier protein SCaMC-3	XM_005160183.5
A25G0203	6.33	3.10	YTX2	Transposon TX1 uncharacterized 149 kDa protein	
A4G0689	6.33	4.34	TSPAN33	Tetraspanin-33	
B11G0765	6.33	9.47	TMC6	Transmembrane channel-like protein 6	NM_001001834.2
A6G0672	6.33	24.89	MYL6	Myosin light polypeptide 6	XM_005166077.5
A6G0470	6.33	3.87	LMO4	LIM domain transcription factor LMO4	NM_212689.2
B8G0617	6.32	98.88			NM_001199436.2
B20G0500	6.32	2.28	KLF11	Krueppel-like factor 11	NM_001044941.3
B8G0245	6.32	5.11	TIMM17B	Mitochondrial import inner membrane translocase subunit Tim17-B	
B7G0253	6.32	4.43	CTU2	Cytoplasmic tRNA 2-thiolation protein 2	NM_001003560.2
B5G0559	6.32	3.26	COQ2	4-hydroxybenzoate polyprenyltransferase, mitochondrial	NM_001089486.1
B3G0837	6.32	1.73	THOC6	THO complex subunit 6 homolog	NM_001006095.1
B23G0180	6.32	4.41	RBM38	RNA-binding protein 38	NM_001110127.1
B2G0262	6.32	3.33	MTERF4	Transcription termination factor 4, mitochondrial	NM_001126468.1
A11G0607	6.32	7.40	SVBP	Small vasohibin-binding protein	NM_001424089.1
B18G0059	6.32	6.07	DAW1	Dynein assembly factor with WDR repeat domains 1	NM_001044352.1
B2G0097	6.32	5.31	RAB12	Ras-related protein Rab-12	NM_001020630.1
A22G0304	6.32	2.97			
A5G0901	6.32	5.99	ERAP2	Endoplasmic reticulum aminopeptidase 2	XM_005165468.4
A12G0609	6.32	10.64	IRF8	Interferon regulatory factor 8	NM_205710.2
A9G0029	6.32	3.98	A33	Zinc-binding protein A33	XM_068219023.1
B6G0498	6.32	23.12	INPP5D	Phosphatidylinositol 3,4,5-trisphosphate 5-phosphatase 1	XM_001922972.7
B3G0254	6.32	4.61	NUP85	Nuclear pore complex protein Nup85	NM_001003625.1
B14G0771	6.31	4.16	A33	Zinc-binding protein A33	XM_009299148.4
B11G0710	6.31	6.00	GIMAP4	GTPase IMAP family member 4	NM_001168317.1
B11G0344	6.31	6.68	CADPS	Calcium-dependent secretion activator 1	NM_001077251.1
B23G0711	6.31	13.50	CALCOCO1	Calcium-binding and coiled-coil domain-containing protein 1	XM_009297039.4
A4G0817	6.31	1.93	LSM8	U6 snRNA-associated Sm-like protein LSm8	NM_001302466.1
B14G0023	6.31	4.43	TRIM16	Tripartite motif-containing protein 16	XM_681071.8
B1G0575	6.31	5.66	SMAD1	Mothers against decapentaplegic homolog 1	NM_131356.2
B3G1522	6.31	17.05	IRGC	Interferon-inducible GTPase 5	XM_068213839.1
A4G0364	6.31	0.74	CLEC4M	C-type lectin domain family 4 member M	XM_068218586.1
A21G0446	6.31	11.41	LOC107551640	A0A672LQL7_SINGR Uncharacterized LOC107551640	NM_001245988.1

A6G0785	6.31	3.34	BDH2	reductase SDR family member 6	NM_001013450.1
B10G0229	6.31	4.07	STARD7	StAR-related lipid transfer protein 7, mitochondrial	XM_687902.9
A11G0382	6.31	9.04	CXXC1	CXXC-type zinc finger protein 1	NM_001077467.1
B3G1194	6.30	6.61	SP100	Nuclear autoantigen Sp-100	
A18G0544	6.30	1.72	PLEKHG7	Pleckstrin homology domain-containing family G member 7	XM_005169952.5
A6G1005	6.30	7.35	PPP1R16B	Protein phosphatase 1 regulatory inhibitor subunit 16B	XM_001340056.8
B13G0682	6.30	12.22	DCAF5	DDB1- and CUL4-associated factor 5	
B13G0278	6.30	14.75	EIF2AK3	Eukaryotic translation initiation factor 2-alpha kinase 3	NM_001083577.1
B3G0722	6.30	4.76	TIMM29	Mitochondrial import inner membrane translocase subunit Tim29	NM_001020561.1
B5G1254	6.30	4.04	ANKRD13A	Ankyrin repeat domain-containing protein 13A	XM_021476185.2
B7G0915	6.30	11.09	DHRS4	reductase SDR family member 4 (Fragment)	NM_200567.2
A2G0850	6.30	20.72	DCAF8	DDB1- and CUL4-associated factor 8	XM_021471269.2
B11G0584	6.30	3.89	SAND	Protein SAND	NM_001365490.1
A8G0020	6.30	6.95	SNCAIP	Synphilin-1	XM_009303688.4
B22G0453	6.30	5.48	STRIP1	Striatin-interacting protein 1 homolog	NM_213521.2
B8G0865	6.30	6.85	TRIM33	E3 ubiquitin-protein ligase TRIM33	NM_001002871.2
B2G0339	6.30	3.44	D2HGDH	D-2-hydroxyglutarate dehydrogenase, mitochondrial	NM_001080597.1
A16G0227	6.30	8.18	PPOX	Protoporphyrinogen oxidase	NM_001039979.2
A8G0305	6.30	2.71	DIMT1	Probable dimethyladenosine transferase	NM_001003556.3
B13G0564	6.30	4.23	WDFY4	WD repeat- and FYVE domain-containing protein 4	NM_001167661.1
B5G0192	6.29	6.46	LZTR1	Leucine-zipper-like transcriptional regulator 1	NM_001080605.2
A19G0656	6.29	9.96	LOC113062173	A0A6P6LSX5_CARAU LOW QUALITY PROTEIN: uncharacterized protein LOC113062173	XM_005159403.5
A1G0710	6.29	3.72	TMCO3	Transmembrane and coiled-coil domain-containing protein 3	XM_068220978.1
B22G0512	6.29	9.88	NISCH	Nischarin	XR_659529.4
A16G0668	6.29	5.72	IL16	Pro-interleukin-16	XM_005169750.5
A13G0284	6.29	69.71	LGALSL-B	Galectin-related protein B	
B6G0429	6.29	6.46	GGA3	ADP-ribosylation factor-binding protein GGA3	NM_001089354.2
A21G0644	6.29	1.58	CRCP	DNA-directed RNA polymerase III subunit RPC9	NM_001045373.2
A2G0263	6.29	8.06	WDR33	pre-mRNA 3' end processing protein WDR33	NM_001194928.1
B1G0951	6.28	11.50	TNPO2	Transportin-2	XM_021477402.2
B14G0073	6.28	3.64	SH3RF2	E3 ubiquitin-protein ligase SH3RF2	XM_068219324.1
A14G0532	6.28	10.75	BRD8	Bromodomain-containing protein 8	NM_001431083.1
B22G0966	6.28	8.28	SSUH2	Protein SSUH2 homolog	NM_001040377.1
B16G0138	6.28	4.71	GLCCI1	Glucocorticoid-induced transcript 1 protein	XM_021467028.2
B9G0647	6.28	10.33	GLB1	Beta-galactosidase	NM_001122856.1

A24G0190	6.28	3.71	ABCB8	Mitochondrial potassium channel ATP-binding subunit	NM_001017544.1
A5G0594	6.28	5.70	PTPA	threonine-protein phosphatase 2A activator	NM_001003425.2
A12G0532	6.27	23.11	IFIT5	Interferon-induced protein with tetratricopeptide repeats 5	NM_001300872.1
A25G0739	6.27	1.19	GAS2	Growth arrest-specific protein 2	XM_680348.8
B22G0347	6.27	8.45	A0A673M587	A0A673M587_9TELE Uncharacterized protein	XM_005161645.5
B12G0512	6.27	7.53	CCZ1	Vacuolar fusion protein CCZ1 homolog	XM_017358438.3
A18G0322	6.27	5.78	CS047	Uncharacterized protein C19orf47 homolog	NM_001045241.1
B15G0623	6.27	9.60	AKAP1	A-kinase anchor protein 1, mitochondrial	NM_001098179.2
B8G0976	6.27	5.23	RAB35	Ras-related protein Rab-35	NM_001003548.2
A12G0450	6.27	4.94	RANGAP1	Ran GTPase-activating protein 1	NM_212989.2
B8G0717	6.27	5.98	DMAP1	DNA methyltransferase 1-associated protein 1	NM_200255.1
B3G0972	6.27	7.52	GRAP2	GRB2-related adaptor protein 2	NM_001017756.2
B14G0650	6.27	9.79	FBXW11A	F-box and WD repeat domain-containing 11-A	XM_005173069.5
A7G1317	6.27	16.47	FUCL7	Fucolectin-7	
B11G0351	6.27	16.40	TAF4	Transcription initiation factor TFIID subunit 4	NM_001347607.1
A13G0435	6.27	5.98	PCBD1	Pterin-4-alpha-carbinolamine dehydratase	XM_068219861.1
B10G0634	6.27	9.20	EI24	Etoposide-induced protein 2.4 homolog	NM_001017898.1
B10G0428	6.26	4.29	RNFT1	E3 ubiquitin-protein ligase RNFT1	NM_213228.2
A18G0401	6.26	5.84	NUDT21	Cleavage and polyadenylation specificity factor subunit 5	NM_201117.1
A9G0689	6.26	3.64	G5714_010433	A0A7J6CQ51_9TELE Uncharacterized protein	
A9G0815	6.26	2.52	DCAF17	DDB1- and CUL4-associated factor 17	XM_068223398.1
A3G0404	6.26	4.11	RHBDF1	Inactive rhomboid protein 1	XM_005164335.5
B6G0680	6.26	4.81	TSFM	Elongation factor Ts, mitochondrial	NM_001080035.1
A19G0526	6.26	8.95	DYNC1LI1	Cytoplasmic dynein 1 light intermediate chain 1	NM_001102428.1
B21G0121	6.26	9.38	RAD50	DNA repair protein RAD50	XM_005167938.5
A13G0803	6.26	5.36	TBXT	T-box transcription factor T	NM_001080635.1
A13G0261	6.26	4.86	GM4952	Glycine N-acyltransferase-like protein	NM_001161599.1
B3G1697	6.26	13.34	LOC113070348	A0A6P6MSS2_CARAU uncharacterized protein LOC113070348	
B10G0433	6.26	6.62	BTG3	Protein BTG3	
B7G1288	6.26	6.20	N4BP1	NEDD4-binding protein 1	XM_005166301.5
B2G0328	6.26	21.52	GBP1	Guanylate-binding protein 1	NM_001002343.1
B25G0319	6.26	2.79	ICE2	Little elongation complex subunit 2	XM_001344415.7
B14G0560	6.26	4.91	CENPF	Centromere protein F	XM_021472747.2
B16G0493	6.26	8.11	PDIA3	Protein disulfide-isomerase A3	NM_212905.1
A17G0859	6.25	6.90	ZFYVE19	NoCut checkpoint regulator	
B15G0203	6.25	5.82	PIK3CB	Phosphatidylinositol 4,5-bisphosphate 3-kinase catalytic subunit beta isoform	XM_068213678.1
A7G0760	6.25	9.65	RORAB	Nuclear receptor ROR-alpha B	XM_009303216.4
B15G0762	6.25	6.77	LIN37	Protein lin-37 homolog	XM_068213553.1

B8G1055	6.25	16.17	HSD17B4	Peroxisomal multifunctional enzyme type 2	NM_200136.1
B8G0050	6.25	5.68	G5714_004116	A0A7J6DBD1_9TELE Uncharacterized protein	XM_009303021.4
A11G0327	6.25	5.09	CHIA	Acidic mammalian chitinase	XM_687846.10
B7G0653	6.25	4.90	NR1H3	Oxysterols receptor LXR-alpha	NM_001017545.3
A22G0070	6.25	6.80	IRF6	Interferon regulatory factor 6	NM_200598.2
A2G0930	6.25	2.05	CYP7B1	Cytochrome P450 7B1	XM_693936.9
A6G0158	6.25	16.25	SLC44A2	Choline transporter-like protein 2	XM_005165750.5
B5G0892	6.25	6.05	LMO4.1	LIM domain transcription factor LMO4.1	NM_177984.1
B23G0257	6.25	9.02	ZMYND8	Protein kinase C-binding protein 1	NM_199641.2
B15G0228	6.25	5.19	PRCP	Lysosomal Pro-X carboxypeptidase	NM_001002694.1
A3G0933	6.24	9.87	NFATC2IP	NFATC2-interacting protein	NM_001327756.1
A7G0463	6.24	5.38	TRAF6	TNF receptor-associated factor 6	NM_001044752.1
A7G1142	6.24	5.00	PDCD7	Programmed cell death protein 7	NM_001003415.1
B22G0849	6.24	1.63	AARS2	Alanine--tRNA ligase, mitochondrial	XM_001332352.9
B5G1096	6.24	5.14	DPH7	Diphthine methyltransferase	NM_001082839.1
B12G0740	6.24	4.51	TMEM238	Transmembrane protein 238	
B22G1072	6.24	5.32	RUBCN	Run domain Beclin-1-interacting and cysteine-rich domain-containing protein	XM_005174059.4
B3G1098	6.24	11.39	PITPNB	Phosphatidylinositol transfer protein beta isoform	NM_213443.1
A15G0714	6.24	6.24	RCBTB2	RCC1 and BTB domain-containing protein 2	XM_005173365.5
A14G0433	6.23	9.21	ETFDH	Electron transfer flavoprotein-ubiquinone oxidoreductase, mitochondrial	NM_001004598.1
A9G0393	6.23	5.93	FDX1	Adrenodoxin, mitochondrial (Fragment)	XM_001922687.5
B9G0365	6.23	10.56	ANKRD52	threonine-protein phosphatase 6 regulatory ankyrin repeat subunit C	XM_005167783.5
A20G0543	6.23	1.81	PPAT	Amidophosphoribosyltransferase	NM_001082877.2
B7G0168	6.23	13.01	EIF4G2	Eukaryotic translation initiation factor 4 gamma 2	NM_001014289.2
B10G0696	6.23	8.04	MARCHF5	E3 ubiquitin-protein ligase MARCHF5	NM_199739.3
B7G1156	6.23	12.09	GIGYF1	GRB10-interacting GYF protein 1	XM_021477778.2
B22G0943	6.23	20.44	SMC3	Structural maintenance of chromosomes protein 3	NM_214689.1
A9G0793	6.23	8.63	WIPF1	WASL-interacting protein family member 1	
A6G0401	6.23	7.97	TRIM25	ISG15 ligase TRIM25	XM_017356449.3
B3G1014	6.22	3.92	ADAP2	Arf-GAP with dual PH domain-containing protein 2	XM_021473800.2
B1G0753	6.22	5.81	G5714_000513	A0A7J6DGJ9_9TELE Uncharacterized protein	
A25G0310	6.22	17.38	CNOT1	CCR4-NOT transcription complex subunit 1	NM_001079951.2
B14G0121	6.22	8.06	SYVN1	E3 ubiquitin-protein ligase synoviolin	NM_212735.2
B20G0505	6.22	3.30	CPSF3	Cleavage and polyadenylation specificity factor subunit 3	NM_001003836.1

A2G0616	6.22	12.89	MARCHF6	E3 ubiquitin-protein ligase MARCHF6	NM_001291707.1
A6G0664	6.21	15.36	ATF7	Cyclic AMP-dependent transcription factor ATF-7	XM_005166050.5
B10G0129	6.21	9.61	HSDL2	Hydroxysteroid dehydrogenase-like protein 2	NM_199599.2
A21G0068	6.21	2.82	CKS2	Cyclin-dependent kinases regulatory subunit 2	NM_001002110.1
B17G0393	6.21	3.19	FAM98A	Protein FAM98A	XM_021473146.2
B24G0444	6.21	5.19	USP12A	Ubiquitin carboxyl-terminal hydrolase 12A	NM_001083556.1
A4G0580	6.21	22.03	ATP6V1F	V-type proton ATPase subunit F	NM_001002526.2
A2G0340	6.21	0.98	DPX16_4609	A0A3N0YC08_ANAGA Uncharacterized protein	
B17G0384	6.21	3.44	EDARADD	Ectodysplasin-A receptor-associated adapter protein	XM_690046.9
B13G0472	6.21	3.81	SEC23IP	SEC23-interacting protein	NM_001004531.1
B7G0067	6.21	1.11	DOK7	Protein Dok-7	XM_009300204.4
A13G0636	6.21	5.28	RPIA	Ribose-5-phosphate isomerase	XM_005156963.5
A12G0223	6.21	3.62	SLC16A3	Monocarboxylate transporter 4	XM_005169469.5
B18G0618	6.20	6.36	SPATA2L	Spermatogenesis-associated protein 2-like protein	NM_001386847.1
A15G0524	6.20	51.80	COX7A2	Cytochrome c oxidase subunit 7A2, mitochondrial	
B17G0044	6.20	13.70	AKT1	threonine-protein kinase	NM_001281801.1
A16G0907	6.20	6.14	FRRS1	Putative ferric-chelate reductase 1	
A2G0294	6.20	13.96	BPNT2	Inositol monophosphatase 3	NM_001037568.2
B14G0228	6.20	6.17	NKRF	NF-kappa-B-repressing factor	NM_001004499.3
A19G0468	6.20	6.62	PITHD1	PITH domain-containing protein 1	NM_207074.1
A15G0372	6.19	2.64	SI:CH211-225B7.5	X1WGJ9_DANRE Si:ch211-225b7.5	
B7G0226	6.19	5.59	SIRT3	NAD-dependent protein deacetylase sirtuin-3, mitochondrial	NM_001080174.1
B15G0606	6.19	4.98	HSPA13	Heat shock 70 kDa protein 13	NM_001089479.1
A25G0401	6.19	9.35	DNM1L	Dynamin-1-like protein	XM_005163049.3
B21G0681	6.19	4.49	NIBAN2	Protein Niban 2	NM_001080586.1
B9G0422	6.19	9.93	MPHOSPH8	M-phase phosphoprotein 8	NM_001126433.2
A6G0974	6.19	2.47	EYA2	Eyes absent homolog 2	NM_001004558.1
B3G0183	6.19	5.82	MICALL1	MICAL-like protein 1	XM_005157990.5
B18G0423	6.19	5.32	SLCO3A1	Solute carrier organic anion transporter family member 3A1	NM_001045188.2
A17G0301	6.19	3.53	ABRAXAS2	BRISC complex subunit Abraxas 2	XM_068214379.1
A3G1171	6.19	3.38	CASKIN2	Caskin-2	XM_021470899.2
A16G0285	6.19	4.81	EBAG9	Receptor-binding cancer antigen expressed on SiSo cells	NM_201094.1
B5G0815	6.19	5.42	HNRNPL	Heterogeneous nuclear ribonucleoprotein L	NM_201099.1
B9G0362	6.19	11.63	COQ10B	Coenzyme Q-binding protein COQ10 homolog B, mitochondrial	NM_001017747.1
B18G0428	6.18	2.56	LYSMD4	LysM and putative peptidoglycan-binding domain-containing protein 4	NM_200850.2
B4G0423	6.18	19.33	TCP11L2	T-complex protein 11-like protein 2	XM_005164731.5
A1G0674	6.18	5.26	CHST10	Carbohydrate sulfotransferase 10	XM_005159890.5

A23G0358	6.18	3.07	SLC35C2	Solute carrier family 35 member C2	NM_212643.1
A3G0061	6.18	16.55	GIMAP8	GTPase IMAP family member 8	XM_068218152.1
B20G0262	6.18	2.97	MEP1B	Meprin A subunit beta	NM_001076621.2
A3G0172	6.18	3.88	A33	Zinc-binding protein A33	XM_009299148.4
A23G0184	6.18	7.25	GSS	Glutathione synthetase	NM_001006104.1
B23G0239	6.18	3.67	CHL1	Neural cell adhesion molecule L1-like protein	XM_009296667.4
A19G0482	6.18	5.50	KRIT1	Krev interaction trapped protein 1	NM_001317001.1
B4G0044	6.17	14.76	FBXO7	F-box only protein 7	NM_001025499.1
B4G0463	6.17	6.26	ANKS1B	Ankyrin repeat and sterile alpha motif domain-containing protein 1B	XM_017355052.3
B12G0283	6.17	34.45	CD82	CD82 antigen	NM_001102622.1
A13G0381	6.17	3.13	ZGC:153142	UPF0728 protein C10orf53 homolog	NM_131621.3
A17G0890	6.17	6.02	ACP7	Acid phosphatase type 7	NM_001099250.1
A23G0794	6.17	26.71	PI16	Peptidase inhibitor 16	
A18G0725	6.17	7.99	INTS4	Integrator complex subunit 4	XM_017351982.3
A3G0512	6.17	5.08	GFER	FAD-linked sulfhydryl oxidase ALR	
B14G0201	6.17	4.69	PABIR1	P2R1A-PPP2R2A-interacting phosphatase regulator 1	XM_005173207.4
B9G0114	6.17	9.44	IFIH1	Interferon-induced helicase C domain-containing protein 1	NM_001308563.1
A2G1066	6.17	5.58	RORB	Nuclear receptor ROR-beta	NM_001277094.2
B6G0253	6.17	2.95	COL5A2	Collagen alpha-2(V) chain	XM_001919970.8
A13G0717	6.17	4.76	TNFAIP2	Tumor necrosis factor alpha-induced protein 2	XM_001343183.8
A18G0419	6.17	7.27	MBTPS1	Membrane-bound transcription factor site-1 protease	NM_199213.2
B7G0907	6.17	2.68	TRIM65	Tripartite motif-containing protein 65	XM_003200506.6
B23G0521	6.17	6.79	GALNT6	Polypeptide N-acetylgalactosaminyltransferase 6	NM_213196.1
B9G0509	6.17	6.25	ZGC:153521	Mitochondrial protein C2orf69 homolog	NM_001077728.1
B7G0879	6.16	11.93	OTUB1	Ubiquitin thioesterase OTUB1	NM_001002500.2
A16G0314	6.16	3.90	ELP6	Elongator complex protein 6	NM_001103142.1
B12G0685	6.16	9.24	KAT7	Histone acetyltransferase KAT7	XM_021480026.2
A19G0566	6.16	5.01	TBC1D5	TBC1 domain family member 5	NM_001328258.1
A8G0956	6.16	4.49	DCP1A	mRNA-decapping enzyme 1A	NM_182893.2
A17G0668	6.16	5.30	PPP2R3C	threonine-protein phosphatase 2A regulatory subunit B" subunit gamma	NM_200131.1
B7G0962	6.16	10.95	ARNT2	Aryl hydrocarbon receptor nuclear translocator 2	XM_009302940.4
B21G0220	6.16	6.01	RARS1	Arginine--tRNA ligase, cytoplasmic	NM_200048.1
B23G0054	6.16	2.48	G5714_023032	A0A7J6BRH4_9TELE C2H2-type domain-containing protein	NM_001386474.1
B10G0118	6.16	5.48	PPWD1	Peptidylprolyl isomerase domain and WD repeat-containing protein 1	NM_001098758.1
B21G0065	6.16	7.83	RAPGEF6	Rap guanine nucleotide exchange factor 6	XM_017353005.2
B2G0107	6.16	5.04	SIRT6	NAD-dependent protein deacylase sirtuin-6	NM_001002071.1
B1G0465	6.16	7.81	TIMMDC1	Complex I assembly factor TIMMDC1, mitochondrial	NM_001017828.1

B7G0344	6.16	7.58	PPP1R14B	Protein phosphatase 1 regulatory subunit 14B	XM_068222095.1
A19G0280	6.16	1.98	HEY1	enhancer-of-split related with YRPW motif protein 1	NM_212561.1
B25G0096	6.15	8.32	NLRC3	NLR family CARD domain-containing protein 3	NM_001423274.1
A5G0247	6.15	5.51	HIRA	Protein HIRA	XM_691386.8
A5G0448	6.15	6.95	GTF2H2	General transcription factor IIH subunit 2	NM_201581.1
B25G0450	6.15	1.94	FAM3C	Protein FAM3C	XM_005163005.5
B2G0828	6.15	6.83	BMI1B	Polycomb complex protein BMI-1-B	NM_001080751.2
A14G0097	6.15	12.52	FAM13B	Protein FAM13B	NM_001328042.1
A5G0503	6.15	8.73	EHMT2	Histone-lysine N-methyltransferase EHMT2	XM_068221046.1
A17G0593	6.15	6.95	SETD3	Actin-histidine N-methyltransferase	NM_200054.1
A24G0450	6.15	6.30	TNFRSF11A	Tumor necrosis factor receptor superfamily member 11A	XM_005162629.5
A2G0898	6.15	13.18	A0A673KQH2	A0A673KQH2_9TELE SCY domain-containing protein	NM_001130655.1
A17G0082	6.15	2.31	YTX2	Transposon TX1 uncharacterized 149 kDa protein	
B6G0967	6.15	7.14	SMPD2	Sphingomyelin phosphodiesterase 2	XR_011016104.1
B8G0438	6.15	11.43	SLC20A2	Sodium-dependent phosphate transporter 2	XM_005167168.5
B11G0110	6.15	6.12	REEP6	Receptor expression-enhancing protein 6	XM_017358373.3
B16G0115	6.15	1.44	MAIP1	m-AAA protease-interacting protein 1, mitochondrial	XM_001338222.6
B24G0205	6.15	1.86	ABCD4	Lysosomal cobalamin transporter ABCD4	NM_001076717.2
B9G0816	6.15	4.35	RAP2A	Ras-related protein Rap-2a	XM_003199163.5
B9G0250	6.15	2.02	BCS1L	Mitochondrial chaperone BCS1	NM_201182.2
A15G0575	6.14	2.09	ADAMTS8	A disintegrin and metalloproteinase with thrombospondin motifs 8	XM_021472930.2
A3G0022	6.14	8.02	GIMAP4	GTPase IMAP family member 4	
B19G0336	6.14	5.23	IGKC	Immunoglobulin kappa constant	
A7G0853	6.14	6.05	BAP18	Chromatin complexes subunit BAP18	NM_001002422.1
A17G0394	6.14	5.15	NIPAL3	NIPA-like protein 3	NM_001002634.2
B8G0358	6.14	8.11	NCOR2	Nuclear receptor corepressor 2	XM_009304185.4
B7G0858	6.14	14.21	CYP26B1	Cytochrome P450 26B1	NM_212666.1
A7G0990	6.14	3.40	TRERF1	Transcriptional-regulating factor 1	XM_068218382.1
B8G0503	6.14	3.39	WDR6	WD repeat-containing protein 6	NM_001113654.1
B17G0669	6.14	3.91			
B3G1093	6.14	7.19	NFATC2IP	NFATC2-interacting protein	NM_001327756.1
A18G0808	6.14	7.51	PDE8A	High affinity cAMP-specific and IBMX-insensitive 3',5'-cyclic phosphodiesterase 8A	NM_001080627.1
A9G0040	6.14	11.02	NF7O	Nuclear factor 7, ovary	XM_068219023.1
A20G0787	6.14	4.95	LOC107549498	A0A672MJW0_SINGR Pinin-like	NM_001020779.2
B23G0733	6.13	9.66	SPRYD3	SPRY domain-containing protein 3	NM_001105694.1
A11G0559	6.13	2.04	DPX16_23249	A0A3N0XHL6_ANAGA Uncharacterized protein	NM_001079969.1
B9G0663	6.13	7.30	G5714_010506	A0A7J6CSC9_9TELE Uncharacterized protein	

B13G0134	6.13	6.94	PPM1G	Protein phosphatase 1G	NM_001004568.2
B7G0623	6.13	2.96	IRX5	Iroquois-class homeodomain protein irx-5	NM_001045227.1
A1G0513	6.13	5.56	ZFP91	E3 ubiquitin-protein ligase ZFP91	XM_021471225.2
B11G0079	6.13	35.09	MMP19	Matrix metalloproteinase-19	NM_001365192.1
B3G0794	6.13	23.44	MUCM	Ig mu chain C region membrane-bound form	XM_068218290.1
B7G0123	6.13	2.39	USP53	Inactive ubiquitin carboxyl-terminal hydrolase 53	XM_017357133.3
B3G0131	6.13	2.83	NOD1	Nucleotide-binding oligomerization domain-containing protein 1	XM_009299257.4
B17G0543	6.12	0.02	PIF1	ATP-dependent DNA helicase PIF1	
B15G0894	6.12	8.76	EIF4G1	Eukaryotic translation initiation factor 4 gamma 1	XM_068217716.1
A20G0057	6.12	1.31	IRF4	Interferon regulatory factor 4	XM_021468723.2
B5G0287	6.12	5.07	MTIF3	Translation initiation factor IF-3, mitochondrial	
B19G0243	6.12	11.91	SNX27	Sorting nexin-27	NM_001045100.1
B25G0156	6.12	0.36	IGKV4-1	Immunoglobulin kappa variable 4-1	XM_068217596.1
B23G0343	6.12	14.07	COX42	Cytochrome c oxidase subunit 4 isoform 2, mitochondrial	NM_200803.1
A5G0579	6.12	5.09	ZMAT5	Zinc finger matrin-type protein 5	NM_001003771.1
A5G0866	6.12	8.13	ARRDC3	Arrestin domain-containing protein 3	NM_001080029.1
B6G0177	6.12	5.27	ILF3	Interleukin enhancer-binding factor 3 homolog	XM_009302153.4
A1G1060	6.12	8.45	FGFBP1	Fibroblast growth factor-binding protein 1	
A8G0478	6.12	11.89	RBM15	RNA-binding protein 15	XM_001335784.8
A17G0099	6.12	8.59	BABAM2	BRISC and BRCA1-A complex member 2	NM_001017777.1
B11G0115	6.11	50.32	BMP7	Bone morphogenetic protein 7	NM_001105595.1
B3G0588	6.11	8.04	MMD2	Monocyte to macrophage differentiation factor 2	NM_001174058.1
B12G0344	6.11	19.05	ITGB3	Integrin beta-3	NM_001082948.1
B14G0308	6.11	3.14	PDGFC	Platelet-derived growth factor C	XM_068213389.1
B12G0165	6.11	14.35	CAMK2G	calmodulin-dependent protein kinase type II subunit gamma	XM_005156363.5
B13G0482	6.11	3.28	PAPOLG	Poly(A) polymerase gamma	NM_001076766.2
A13G0086	6.11	2.03	MARCKSL1A	MARCKS-related protein 1-A	
B16G0281	6.11	3.87	CD22	B-cell receptor CD22 (Fragment)	
A2G0716	6.11	6.54	LAMC1	Laminin subunit gamma-1	NM_173277.1
B23G0345	6.11	7.62	ARFGEF2	Brefeldin A-inhibited guanine nucleotide-exchange protein 2	XM_002666467.6
B24G0267	6.11	2.20	ABCD3	ATP-binding cassette sub-family D member 3	NM_213482.1
A16G0693	6.11	6.98	VARS1	Valine--tRNA ligase	NM_001311346.1
B17G0526	6.11	4.64	TMEM30B	Cell cycle control protein 50B	XM_005158776.5
A13G0187	6.11	7.04	GPHB5	Glycoprotein hormone beta-5	NM_001110022.2
B15G0907	6.11	5.46	STARD10	START domain-containing protein 10	NM_205646.2
B5G0130	6.11	6.99	PAIP1	Polyadenylate-binding protein-interacting protein 1	NM_001003504.1
B17G0568	6.11	3.88	LDAH	Lipid droplet-associated hydrolase	NM_001130785.1
A7G0858	6.11	9.01	PELP1	Proline-, glutamic acid- and leucine-rich protein 1	XM_001338982.8

B11G0003	6.11	5.25	MYG1	MYG1 exonuclease	NM_001110486.1
A10G0436	6.11	12.89	LHFPL6	LHFPL tetraspan subfamily member 6 protein	NM_001008653.1
A17G0826	6.10	7.96	TMX1	Thioredoxin-related transmembrane protein 1	NM_001030159.2
B7G0376	6.10	6.64	USP12	Ubiquitin carboxyl-terminal hydrolase 12	XM_683267.10
A9G0538	6.10	9.46	MPHOSPH8	M-phase phosphoprotein 8	NM_001126433.2
B24G0579	6.10	6.44	IPO4	Importin-4	XM_679071.10
A17G0800	6.10	26.42	EPHX2	Bifunctional epoxide hydrolase 2	NM_001008642.1
B25G0423	6.10	6.47	KXD1	KxDL motif-containing protein 1	NM_001003469.2
A13G0295	6.10	8.12			
B13G0635	6.10	8.90	JAG2	Protein jagged-2	NM_001159828.1
B8G0843	6.10	3.08	ENDOG	Endonuclease G, mitochondrial	NM_001024214.1
B12G0553	6.10	5.16	LOC113096529	A0A6P6PAI8_CARAU uncharacterized protein LOC113096529	XR_002459555.2
A9G0474	6.10	1.52	DHRS12	reductase SDR family member 12	NM_001076557.1
B7G1102	6.10	5.46	TTC5	Tetratricopeptide repeat protein 5	NM_001017654.2
B1G0681	6.10	6.99	TMX2B	Thioredoxin-related transmembrane protein 2-B	NM_001002113.1
B3G0375	6.09	8.67	AMDHD2	N-acetylglucosamine-6-phosphate deacetylase	NM_205681.1
B6G0081	6.09	6.43	HYI	Putative hydroxypyruvate isomerase	
B25G0750	6.09	23.88	SNX24	Sorting nexin-24	NM_001045374.2
A6G0269	6.09	3.95	CASP8	Caspase-8	NM_001136252.2
B5G0799	6.09	10.51	WDR44	WD repeat-containing protein 44	NM_001100038.2
A7G1336	6.09	4.25	NEK1	threonine-protein kinase Nek1	NM_001327832.1
B10G0382	6.09	6.10	SCAF4	SR-related and CTD-associated factor 4	XM_690795.9
B5G0767	6.09	10.98	A0A671S8K4	A0A671S8K4_9TELE RBR-type E3 ubiquitin transferase	
A11G0309	6.09	0.43	PPFIA4	Liprin-alpha-4	NM_001114554.2
B3G1296	6.09	5.16	FAHD1	Acylpyruvase FAHD1, mitochondrial	NM_001020728.1
B4G0589	6.09	4.82	USP44	Ubiquitin carboxyl-terminal hydrolase 44	NM_200257.1
A21G0735	6.09	14.92	PGRMC1	Membrane-associated progesterone receptor component 1	NM_001007392.1
B14G0831	6.09	10.90	NLRC3	Protein NLRC3	NM_001423275.1
B4G0206	6.09	11.31	PHYH	Phytanoyl-CoA dioxygenase, peroxisomal	NM_001017823.2
B21G0656	6.09	26.82	GSTT3	Glutathione S-transferase theta-3	NM_200584.1
A9G0562	6.09	5.02	WDR3	WD repeat-containing protein 3	NM_198873.1
B8G0691	6.09	8.03	EDEM3	ER degradation-enhancing alpha-mannosidase-like protein 3	XM_005166932.5
A19G0892	6.09	4.31	LOC113062510	A0A6P6LUL0_CARAU uncharacterized protein LOC113062510 isoform X2	
B16G0496	6.09	4.92	TMEM79	Transmembrane protein 79	XM_009292402.4
A8G0143	6.08	9.71	SSUH2	Protein SSUH2 homolog	XM_005166701.5
A17G0382	6.08	5.76	YTHDF2	YTH domain-containing family protein 2	XM_005158763.5

A12G0770	6.08	5.90	LOC113056409	A0A6P6L335_CARAU COP1-interactive protein 1-like isoform X1	XM_001340752.9
B18G0482	6.08	8.85	DENND11	DENN domain-containing protein 11	NM_001044347.1
B8G0901	6.08	4.04	PLXNB3	Plexin-B3	XM_068223098.1
B18G0171	6.08	9.66	LEO1	RNA polymerase-associated protein LEO1	NM_207067.1
A2G0802	6.08	8.77	NRDC	Nardilysin	XM_005171326.5
B10G0110	6.08	11.33	SMC5	Structural maintenance of chromosomes protein 5	NM_001193541.1
B23G0627	6.08	20.18	SGK1	threonine-protein kinase Sgk1	NM_199212.1
B9G0486	6.08	5.86	WDFY2	WD repeat and FYVE domain-containing protein 2	NM_205745.1
A25G0349	6.08	10.81	LOC107574797	H(+) antiporter-like	XM_681933.8
B15G0688	6.08	9.79	PDS5B	Sister chromatid cohesion protein PDS5 homolog B	XM_688861.8
B22G0129	6.08	13.12	ING5	Inhibitor of growth protein 5	NM_001100049.2
A4G0680	6.08	3.91	CHCHD3	MICOS complex subunit MIC19	XM_009300263.4
B2G1000	6.08	2.64	POL	RNA-directed DNA polymerase from mobile element jockey	
B7G0240	6.08	20.02	IST1	IST1 homolog	NM_212585.2
B17G0884	6.08	7.25	AQR	RNA helicase aquarius	NM_200464.2
A16G0888	6.08	10.44	NCALDA	Neurocalcin-delta A	NM_001114410.1
B3G1322	6.08	9.73	SREBF1	Sterol regulatory element-binding protein 1	NM_001105129.1
A2G0009	6.08	3.73	TADA1	Transcriptional adapter 1	NM_205606.2
B23G0500	6.07	3.46	TTPAL	Alpha-tocopherol transfer protein-like	NM_212590.2
A14G0041	6.07	8.90	USP43	Ubiquitin carboxyl-terminal hydrolase 43	
A3G0047	6.07	1.51	ST14	Suppressor of tumorigenicity 14 protein homolog	NM_001037562.2
A7G0947	6.07	12.90	FXR2	RNA-binding protein FXR2	NM_200211.2
B19G0125	6.07	10.21	CEACAM5	Carcinoembryonic antigen-related cell adhesion molecule 5	
A10G0596	6.07	4.01	KCNIP1	Kv channel-interacting protein 1	XM_021479166.2
B1G1044	6.07	1.64	NXPE3	NXPE family member 3	XM_068217145.1
A24G0130	6.07	4.02	RHOT2	Mitochondrial Rho GTPase 2	NM_001037679.2
A25G0670	6.07	6.97	IGKV3-15	Immunoglobulin kappa variable 3-15	XM_068217596.1
A9G0712	6.07	8.76	TSC22D1	TSC22 domain family protein 1	NM_001328125.1
B6G0404	6.06	25.86	TRIM25	ISG15 ligase TRIM25	NM_200175.1
B18G0642	6.06	6.89	PARP12	Protein mono-ADP-ribosyltransferase PARP12	NM_001082852.1
A6G0130	6.06	3.90	NHEJ1	Non-homologous end-joining factor 1	
A11G0532	6.06	30.69	MST1R	Macrophage-stimulating protein receptor	NM_001037102.1
A14G0147	6.06	3.56	PSMD10	26S proteasome non-ATPase regulatory subunit 10	NM_205754.1
A2G1020	6.06	15.90	GPC1	Glypican-1	NM_201020.2
B2G0706	6.06	197.61	CAHZ	Carbonic anhydrase	NM_131110.1
B8G0059	6.06	10.05	RBSN	Rabenosyn-5	XM_068223241.1
B5G1326	6.06	6.81	RAB14	Ras-related protein Rab-14	NM_200683.2
B1G0984	6.06	1,007.63	MB	Myoglobin	
A12G0670	6.06	5.55	MYOF	Myoferlin	XM_068224535.1
A15G0389	6.06	6.98	UNC119	Protein unc-119 homolog A	XM_690112.7
B12G0249	6.06	4.78	ENTPD1	Ectonucleoside triphosphate diphosphohydrolase 1	XM_005156326.5
A2G0624	6.06	21.95	ATP6V1H	V-type proton ATPase subunit H	NM_001291708.1

A6G0068	6.06	0.03	TGM5	Protein-glutamine gamma-glutamyltransferase 5	
B3G0001	6.06	6.88	WBP11	WW domain-binding protein 11	XM_068218398.1
B6G0982	6.06	7.23	PCIF1	mRNA (2'-O-methyladenosine-N(6)-)-methyltransferase	NM_001423772.1
A12G0441	6.06	5.32	DESI1	Desumoylating isopeptidase 1	NM_200208.1
A13G0731	6.05	3.62	HELLS	Lymphoid-specific helicase	NM_001202417.1
B10G0167	6.05	5.28	MOCS2	Molybdopterin synthase catalytic subunit	XM_017358006.3
B6G1003	6.05	2.83	TOX2	TOX high mobility group box family member 2	NM_212908.1
B3G1125	6.05	5.01	FAM117A	Protein FAM117A	XM_001922225.6
B20G0227	6.05	7.87	PRRC2C	Protein PRRC2C	XM_021473359.2
A1G1093	6.05	15.26	MND1	Meiotic nuclear division protein 1 homolog	XM_005170917.5
B11G0659	6.05	3.31	PHF13	PHD finger protein 13	NM_001020570.1
A12G0122	6.05	10.27	EPC1	Enhancer of polycomb homolog 1	XM_689970.9
B6G0702	6.05	11.14	CRBN	Protein cereblon	NM_001003996.1
B22G0471	6.05	1.70	LOC113080865	A0A6P6NKR3_CARAU CD48 antigen-like	XM_001346243.8
B18G0023	6.05	6.46	DUSP8	Dual specificity protein phosphatase 8	XM_002665301.7
A15G0823	6.05	1.64	CLDN3	Claudin-3	
B19G0615	6.05	4.06	SLA	Src-like-adapter	NM_001034989.2
B23G0110	6.05	4.79	SLC34A2	Sodium-dependent phosphate transport protein 2B	XM_068216694.1
B7G0843	6.04	6.52	BAP18	Chromatin complexes subunit BAP18	NM_001002422.1
B15G0408	6.04	6.10			NM_001347831.1
B19G0542	6.04	5.10	LOC113119356	A0A6P6RHC1_CARAU uncharacterized protein LOC113119356 isoform X1	XM_005173781.5
B11G0410	6.04	13.03	MXRA8	Matrix remodeling-associated protein 8	NM_001045307.1
A12G0165	6.04	5.54	NPLOC4	Nuclear protein localization protein 4 homolog	NM_199807.1
B5G0536	6.04	4.14	PMPCA	Mitochondrial-processing peptidase subunit alpha	NM_001007442.1
A20G0621	6.04	7.76	PPM1A	Protein phosphatase 1A	NM_001161332.1
A1G0440	6.04	3.58	LOC113070356	A0A6P6MU08_CARAU uncharacterized protein LOC113070356	
A5G0668	6.04	3.62	NAIF1	Nuclear apoptosis-inducing factor 1	NM_001077271.1
B16G0908	6.04	11.12	SKAP2	Src kinase-associated phosphoprotein 2	NM_200628.1
A11G0482	6.04	9.40	CACNA1A	Q-type calcium channel subunit alpha-1A	
B11G0034	6.04	6.27	RAF1	threonine-protein kinase	NM_001386666.1
B5G0027	6.04	10.82	GOLGA2	Golgin subfamily A member 2	XM_009301121.4
B2G1038	6.03	13.07	YME1L1	ATP-dependent zinc metalloprotease YME1L1	NM_001331068.1
B19G0833	6.03	9.58	PARP10	Protein mono-ADP-ribosyltransferase PARP10	XM_021468293.2
B20G0246	6.03	7.08			
B17G0497	6.03	2.49	NRDE2	Nuclear exosome regulator NRDE2	
A7G0696	6.03	7.92	TSSC4	Protein TSSC4	
A2G0438	6.03	6.11	AMOTL2A	Angiomotin-like 2a	NM_001077606.2
B19G0294	6.03	0.90	FPR2	Formyl peptide receptor 2	NM_001082891.1

B7G1035	6.03	1.56	PRMT3	Protein arginine N-methyltransferase 3	XM_009302968.4
A19G0698	6.03	4.45	DPM3	Dolichol-phosphate mannosyltransferase subunit 3	NM_200809.2
A6G0610	6.03	8.30	OPA1	Dynamin-like 120 kDa protein, mitochondrial	XM_009302514.4
A17G0113	6.03	4.58	SLC39A9	Zinc transporter ZIP9	NM_001013540.1
B25G0501	6.03	11.10	KITLG	Kit ligand	NM_001018123.1
B25G0671	6.03	7.73	RDH5	Retinol dehydrogenase 5	
B1G0254	6.03	16.20	CXCL8	Interleukin-8	XM_001342570.8
B13G0885	6.03	40.98	SRF	Serum response factor	NM_001327772.1
A5G1298	6.03	4.05	SI:CH211-283H6.4	UPF0561 protein C2orf68 homolog	NM_001114693.1
B9G0490	6.03	5.49	COPS8	COP9 signalosome complex subunit 8	NM_200229.2
B5G0179	6.03	7.28	SPRING	SREBP regulating gene protein	NM_001020737.1
A5G1148	6.03	7.14	TAF6	Transcription initiation factor TFIID subunit 6	NM_001004557.1
A21G0222	6.03	8.41	ELL2	RNA polymerase II elongation factor ELL2	NM_001083812.2
B10G0136	6.02	9.78	NRARPA	Notch-regulated ankyrin repeat-containing protein A	NM_181495.3
A5G0662	6.02	9.12	TAF1	Transcription initiation factor TFIID subunit 1	XM_021476170.2
A16G0441	6.02	2.41	SLC45A4	Solute carrier family 45 member 4	XM_017351511.3
B1G0015	6.02	24.13	CD247	T-cell surface glycoprotein CD3 zeta chain	
A10G0774	6.02	75.23	GVIN1	Interferon-induced very large GTPase 1	
A7G0300	6.02	9.60	GALNS	N-acetylgalactosamine-6-sulfatase	NM_001080641.2
B7G1034	6.02	3.10	HTATIP2	Oxidoreductase HTATIP2	NM_131681.2
B3G0575	6.02	9.92	SNX8	Sorting nexin-8	NM_001326490.1
A14G0087	6.02	0.98	A0A6G9W2S3	A0A6G9W2S3_CYPCA Interleukin-5 family A	
B4G0037	6.02	8.27	TBK1	threonine-protein kinase TBK1	NM_001044748.2
B20G0691	6.02	9.91	MSRA	Mitochondrial peptide methionine sulfoxide reductase	NM_001082893.2
B24G0023	6.02	9.50	LOC113079928	A0A6P6NIJ5_CARAU uncharacterized protein LOC113079928	XM_021470420.2
B21G0126	6.02	3.83	BOD1	Biorientation of chromosomes in cell division protein 1	
A6G0347	6.02	4.51	PLXNB1	Plexin-B1	XM_005166242.5
A15G0780	6.02	1.50	LIPT2	Putative lipoyltransferase 2, mitochondrial	NM_001039993.1
B20G0763	6.02	24.60	FKBP1B	Peptidyl-prolyl cis-trans isomerase FKBP1B	NM_200812.2
B6G1014	6.01	8.75	DDIT3	DNA damage-inducible transcript 3 protein	XM_068221752.1
B6G0872	6.01	6.53	PPP4R1	threonine-protein phosphatase 4 regulatory subunit 1	XM_021471846.2
B5G0743	6.01	9.59	ANTXR2	Anthrax toxin receptor 2	NM_001044709.2
B10G0389	6.01	5.51	A33	Zinc-binding protein A33	NM_200284.1
B8G0911	6.01	5.97	MECP2	Methyl-CpG-binding protein 2	NM_212736.2
A15G0646	6.01	12.26	CLCA4A	Calcium-activated chloride channel regulator 4A	XM_009291544.4
B20G0412	6.01	1.04	ZNF106	Zinc finger protein 106	
A7G0543	6.01	8.12	SCRIB	Protein scribble homolog	XM_021477417.2
B25G0705	6.01	7.97	CAMK1D	calmodulin-dependent protein kinase type 1D	NM_001080658.2

B24G0321	6.01	8.57	STAG1	Cohesin subunit SA-1	XM_687028.9
A2G0709	6.01	11.41	TNN	Tenascin-N	XM_005171264.5
A16G0100	6.01	1.26	ZNF385D	Zinc finger protein 385D	XM_021467037.2
A4G0759	6.01	28.66	CRY1	Cryptochrome-1	NM_001077297.2
B15G0482	6.01	4.85	NLRX1	NLR family member X1	XM_021466414.2
A1G0561	6.01	7.47	LOC113114234	A0A6P6QTQ9_CARAU uncharacterized protein LOC113114234	
B16G0421	6.01	4.25	COQ3	Ubiquinone biosynthesis O-methyltransferase, mitochondrial	NM_001002620.2
B9G0271	6.01	5.41	LZTR1	Leucine-zipper-like transcriptional regulator 1 homolog	XM_678615.7
B20G0031	6.00	2.17	RANBP2	E3 SUMO-protein ligase RanBP2	
B14G0547	6.00	5.50	PSD2	PH and SEC7 domain-containing protein 2	XM_068213300.1
B17G0799	6.00	7.57	RBKS	Ribokinase	NM_001002117.1
B10G0452	6.00	8.23	HYOU1	Hypoxia up-regulated protein 1	NM_212703.1
A3G0831	6.00	1.32	LITAF	Cell death-inducing p53-target protein 1 homolog	
A18G0628	6.00	4.29	SEMA3AB	Semaphorin-3ab	NM_131061.1
A11G0767	6.00	7.16	PHACTR3	Phosphatase and actin regulator 3	NM_001320425.1
B15G0699	6.00	2.93	ARL4A	ADP-ribosylation factor-like protein 4A	NM_200860.1
A9G0621	6.00	2.38	G5714_010506	A0A7J6CSC9_9TELE Uncharacterized protein	
B11G0357	6.00	12.59	IL10	Interleukin-10	NM_001007295.1
A2G0956	6.00	32.02	CREM	cAMP-responsive element modulator	XM_068214537.1
A18G0040	6.00	8.38	PPP1R37	Protein phosphatase 1 regulatory subunit 37	NM_001080167.2
B17G0706	6.00	1.61	CAPN1	Calpain-1 catalytic subunit	XM_017351848.3
A10G0175	6.00	2.95	XRCC4	DNA repair protein XRCC4	NM_200786.1
B15G0842	6.00	8.33	DHRS12	reductase SDR family member 12	NM_203511.1
A11G0512	6.00	28.62	ATP13A3	Polyamine-transporting ATPase 13A3	NM_200414.1
B14G0778	6.00	5.37	A33	Zinc-binding protein A33	NM_001044961.1
B15G0316	6.00	27.04	DHRS11	reductase SDR family member 11	NM_001002143.2
A2G1005	6.00	5.42	CAPN10	Calpain-10	XM_009298795.4
B7G0069	5.99	5.99	ADGRL3	Adhesion G protein-coupled receptor L3	XM_068222452.1
B4G0292	5.99	0.45	POL	LINE-1 retrotransposable element ORF2 protein	
B4G0065	5.99	7.15	MED21	Mediator of RNA polymerase II transcription subunit 21	NM_213423.1
B18G0096	5.99	5.16	FAM118B	Protein FAM118B	XM_005159255.5
A2G0547	5.99	16.93	PTBP1	Polypyrimidine tract-binding protein 1	NM_001020477.1
A20G0846	5.99	7.81	MARCKS	Myristoylated alanine-rich C-kinase substrate	XM_005170139.5
B8G0226	5.99	20.23	AEBP1	Adipocyte enhancer-binding protein 1	XM_005167251.5
B22G0385	5.99	9.05	LOC113076568	A0A6P6N983_CARAU uncharacterized protein LOC113076568	XM_021469439.2
B20G0474	5.99	3.29	DCAF4	DDB1- and CUL4-associated factor 4	XM_021468379.2
A6G0495	5.99	6.49	CEP63	Centrosomal protein of 63 kDa	XM_068221680.1

A14G0442	5.99	12.07	SCOCB	Short coiled-coil protein B	NM_001005966.2
A1G0600	5.99	5.28	NT5C2	Cytosolic purine 5'-nucleotidase	NM_001079966.2
B22G0655	5.99	4.59	DBT	Lipoamide acyltransferase component of branched-chain alpha-keto acid dehydrogenase complex, mitochondrial	NM_001013515.1
B20G0818	5.99	7.14	BATF	Basic leucine zipper transcriptional factor ATF-like	NM_001045059.1
A20G0689	5.99	1.17	TMEM205	Transmembrane protein 205	XM_693051.7
A24G0089	5.99	4.62	CAPN15	Calpain-15	XM_021470406.2
A10G0212	5.99	10.05	GGT5	Glutathione hydrolase 5 proenzyme	XM_002667837.7
B16G0788	5.99	4.57	LPCAT3	Lysophospholipid acyltransferase 5	NM_001316738.1
B3G0405	5.98	6.17	SAMD1	Sterile alpha motif domain-containing protein 1	NM_001316989.1
B16G0926	5.98	7.18	FAS	Tumor necrosis factor receptor superfamily member 6	XM_002664841.7
B23G0244	5.98	14.19	EIF4E3	Eukaryotic translation initiation factor 4E type 3	NM_001004589.1
B23G0390	5.98	5.19	SLC35C2	Solute carrier family 35 member C2	NM_212643.1
A6G0860	5.98	4.21	PPP4R1	threonine-protein phosphatase 4 regulatory subunit 1	XM_021471846.2
B3G0493	5.98	3.88	UBALD1	UBA-like domain-containing protein 1	NM_001020671.2
B13G0574	5.98	10.31	RRP12	RRP12-like protein	XM_068224919.1
A13G0406	5.98	1.29	LBX1	Transcription factor LBX1	
A16G0564	5.98	6.20	TGTP1	T-cell-specific guanine nucleotide triphosphate-binding protein 1	XM_688591.9
A3G0730	5.98	8.97	BAX	Apoptosis regulator BAX	
A19G0257	5.98	2.67	MYCL1B	Protein L-Myc-1b	NM_001045142.1
A9G0546	5.98	4.61	EEF1AKMT1	EEF1A lysine methyltransferase 1	XM_005167551.5
A5G1107	5.98	7.83	KCMF1	E3 ubiquitin-protein ligase KCMF1	NM_200952.2
A9G0161	5.98	3.82	FKBP7	Peptidyl-prolyl cis-trans isomerase FKBP7	NM_001004506.1
B19G0307	5.98	4.27	LIPE	Hormone-sensitive lipase	NM_001316725.1
B24G0308	5.98	13.81	LOC113042371	A0A6P6JF00_CARAU eotaxin-like	NM_001361396.1
A18G0554	5.97	6.26	PPFIBP1	Liprin-beta-1	XM_021467668.2
B22G0447	5.97	0.94	HEPACAM	Hepatocyte cell adhesion molecule	XM_068216396.1
B1G0207	5.97	1.75	A0A673GNM5	A0A673GNM5_9TELE Zgc:174945	
A5G1197	5.97	2.09	LOC113112134	A0A6P6QJK3_CARAU uncharacterized protein LOC113112134	
A4G0763	5.97	6.39	MKLN1	Muskelin	NM_212694.1
A6G0159	5.97	3.90	GCDH	Glutaryl-CoA dehydrogenase, mitochondrial	NM_200886.1
B18G0808	5.97	1.70	DCUN1D5	DCN1-like protein 5	
A7G0385	5.97	1.73	PCOLCE2	Procollagen C-endopeptidase enhancer 2	NM_001128787.1
A7G0523	5.97	7.20	GPT2	Alanine aminotransferase 2	XM_068222422.1
A22G0052	5.97	6.09	RBBP5	Retinoblastoma-binding protein 5	XM_005168268.5
B9G0723	5.97	25.90	SIK2	threonine-protein kinase SIK2	XM_005167449.5
A23G0357	5.97	3.46	TAF13	Transcription initiation factor TFIID subunit 13	NM_001004665.1

B15G0492	5.97	6.46	ERCC2	General transcription and DNA repair factor IIH helicase subunit XPD	NM_200926.2
A15G0373	5.97	6.65	SSH2	Protein phosphatase Slingshot homolog 2	XM_005157671.5
B7G0426	5.97	5.18	HRAS	GTPase HRas	NM_001020629.1
A14G0491	5.97	15.30	B4GALT1	Beta-1,4-galactosyltransferase 1	
A11G0613	5.97	1.62	VAMP3	Vesicle-associated membrane protein 3	
A7G0520	5.97	9.09	ITFG1	T-cell immunomodulatory protein	XM_021472011.2
A17G0303	5.97	4.53	NUBPL	Iron-sulfur protein NUBPL	NM_001002442.1
B16G1142	5.96	4.57	LPCAT1	Lysophosphatidylcholine acyltransferase 1	XM_068214056.1
B14G0531	5.96	32.12	CTSF	Cathepsin F	NM_001077568.1
B12G0180	5.96	1.81	NDUFAF8	NADH dehydrogenase [ubiquinone] 1 alpha subcomplex assembly factor 8	NM_001303010.1
B5G0606	5.96	2.33	POMT1	Protein O-mannosyl-transferase 1	NM_001048067.2
A8G0711	5.96	8.73	TBC1D10A	TBC1 domain family member 10A	XM_009304172.3
B8G0526	5.96	7.07	FOXP3	Forkhead box protein P3	NM_001329567.1
B8G0192	5.96	4.53	CCN1	CCN family member 1	
B19G0503	5.96	2.98	ZCCHC17	Zinc finger CCHC domain-containing protein 17	NM_200544.2
A20G0521	5.96	3.78	ZBTB2	Zinc finger and BTB domain-containing protein 2	NM_213415.2
A17G0254	5.96	4.33	ITGB1BP1	Integrin beta-1-binding protein 1	NM_001037409.2
A2G0999	5.96	5.65	ALG3	Dol-P-Man:Man(5)GlcNAc(2)-PP-Dol alpha-1,3-mannosyltransferase	XM_068224138.1
B9G0894	5.96	4.12	AAAS	Aladin	NM_213225.2
B2G0018	5.96	11.10	TRIM16	Tripartite motif-containing protein 16	XM_681071.8
B8G0051	5.96	5.83	CHDH	Choline dehydrogenase, mitochondrial	XM_002663255.6
A11G0318	5.96	7.74	A0A671LNI1	A0A671LNI1_9TELE Uncharacterized protein	NM_200467.1
A24G0409	5.95	13.03	ATP6V1A	V-type proton ATPase catalytic subunit A	XM_021470316.2
A1G0668	5.95	6.39	MAP4K4	Mitogen-activated protein kinase kinase kinase kinase 4	XM_021478794.2
B14G0405	5.95	7.21	MACROD1	ADP-ribose glycohydrolase MACROD1	NM_001004573.2
B16G0900	5.95	3.73	JAZF1	Juxtaposed with another zinc finger protein 1	NM_001034992.2
B24G0314	5.95	4.59	LOC113042371	A0A6P6JF00_CARAU eotaxin-like	
A11G0576	5.95	8.47	ERC2	ERC protein 2	NM_001423692.1
A24G0502	5.95	12.19	DCAF11	DDB1- and CUL4-associated factor 11	NM_001031675.2
A19G0328	5.95	3.71	GALNT4	Polypeptide N-acetylgalactosaminyltransferase 4	NM_001044778.2
B23G0215	5.95	9.65	PHF20	PHD finger protein 20	XM_005161993.5
A14G0687	5.95	6.74	FMR1-A	Fragile X messenger ribonucleoprotein 1 homolog A	NM_152963.1
B16G0486	5.95	5.42	PTK2	Focal adhesion kinase 1	XM_068213873.1
B15G0539	5.94	3.59	TP53I13	Tumor protein p53-inducible protein 13	XM_001338767.8
B12G0075	5.94	5.48	VCL	Vinculin	NM_001317752.1

A16G0757	5.94	5.26	JOSD2	Josephin-2	NM_200151.2
B1G0092	5.94	19.01	NDFIP2	NEDD4 family-interacting protein 2	NM_213347.2
B8G1066	5.94	10.81	SPATS2	Spermatogenesis-associated serine-rich protein 2	NM_001077726.2
B19G0361	5.94	5.38	NGLY1	Peptide-N(4)-(N-acetyl-beta-glucosaminyl)asparagine amidase	NM_001020601.1
B4G0060	5.94	6.89	SCAF11	Protein SCAF11	XM_689753.9
A17G0725	5.94	10.38	SPTSSA	Serine palmitoyltransferase small subunit A	NM_001093727.1
A23G0655	5.94	7.43	KLF15	Krueppel-like factor 15	NM_212746.3
A25G0497	5.94	6.73	TBC1D2B	TBC1 domain family member 2B	XM_002666866.7
B21G0881	5.94	15.44	CERT1	Ceramide transfer protein	XM_021468957.2
A6G0187	5.94	8.63	MAP3K2	Mitogen-activated protein kinase kinase kinase 2	XM_681521.6
B25G0513	5.94	4.80	VAC14	Protein VAC14 homolog	XM_009297900.4
A9G0247	5.94	5.66	SON	Protein SON	NM_001130400.1
B23G0136	5.94	5.24	TMEM106C	Transmembrane protein 106C	NM_001077296.1
B14G0200	5.94	23.24	HPRT1	Hypoxanthine-guanine phosphoribosyltransferase	NM_212986.2
A4G0524	5.94	2.92	SLC35E3	Solute carrier family 35 member E3	NM_001030179.1
B5G1226	5.93	5.67	CMKLR1	Chemerin-like receptor 1	XM_005165199.5
B17G0056	5.93	8.80	INAFM2	Putative transmembrane protein INAFM2	
B23G0540	5.93	10.75	PHF8	Histone lysine demethylase PHF8	NM_001202447.1
A18G0003	5.93	6.87	PDPR	Pyruvate dehydrogenase phosphatase regulatory subunit, mitochondrial	XM_002665429.7
A1G1010	5.93	5.66	ECPAS	Proteasome adapter and scaffold protein ECM29	NM_001365155.1
B20G0121	5.93	4.02	GALC	Galactocerebrosidase	NM_001005921.1
B20G0909	5.93	8.12	NCOA7	Nuclear receptor coactivator 7	XM_005173833.5
A22G0137	5.93	4.11	GGH	Gamma-glutamyl hydrolase	NM_001111175.1
A16G0542	5.93	3.12	PTPN3	Tyrosine-protein phosphatase non-receptor type 3	XM_682838.10
A8G0276	5.93	8.37	GCLM	Glutamate--cysteine ligase regulatory subunit	NM_199845.1
B5G0189	5.93	6.39	EHD2	EH domain-containing protein 2	NM_001128748.1
A5G0808	5.93	1.70	GRSF1	G-rich sequence factor 1	
A8G0080	5.93	3.29	MRPL40	39S ribosomal protein L40, mitochondrial	
A5G0651	5.93	7.89	OPHN1	Oligophrenin-1	XM_068218245.1
A23G0797	5.93	5.49	PFKFB2	fructose-2,6-bisphosphatase 2	XM_068216712.1
A24G0499	5.92	11.74	STAU2	Double-stranded RNA-binding protein Staufen homolog 2	XM_021470116.2
B7G0262	5.92	1.64	LOC113047499	A0A6P6JXW1_CARAU uncharacterized protein LOC113047499	
A3G0876	5.92	4.05	KAT2A	Histone acetyltransferase KAT2A	NM_001423774.1
B25G0466	5.92	5.92	CO040	UPF0235 protein C15orf40 homolog	
B13G0491	5.92	3.42	G5714_013473	A0A7J6CFA7_9TELE DUF4806 domain-containing protein	XM_009307348.4
B3G1202	5.92	75.94	PRVM	Parvalbumin, muscle	NM_205573.3
B12G0373	5.92	7.57	FOXN2	Forkhead box protein N2	NM_001017555.2

B5G0374	5.92	2.41	WDR36	WD repeat-containing protein 36	NM_001308831.1
A8G0038	5.92	5.94	ZNF366	Zinc finger protein 366	XM_693094.9
A18G0786	5.92	10.78	TARS3	Threonine--tRNA ligase 2, cytoplasmic	XR_011007191.1
B16G1084	5.92	6.83	INPP5B	Type II inositol 1,4,5-trisphosphate 5-phosphatase	XM_009292060.4
A16G0772	5.92	10.97	ASH1L	Histone-lysine N-methyltransferase ASH1L	NR_147500.1
B2G0978	5.92	4.57	GLMN	Glomulin	XM_021480037.2
A3G0566	5.92	4.57	TUBG1	Tubulin gamma-1 chain	NM_200908.2
A20G0499	5.92	7.87	SEL1L	Protein sel-1 homolog 1	NM_001045164.1
B1G1128	5.91	5.10	LOC113099769	A0A6P6PG31_CARAU zinc finger protein 883-like	XM_017357165.3
A5G0457	5.91	27.60	TCN2	Transcobalamin-2	NM_001123231.2
A24G0143	5.91	8.91	COG7	Conserved oligomeric Golgi complex subunit 7	NM_001077393.1
B8G0956	5.91	19.68	UFD1	Ubiquitin recognition factor in ER-associated degradation protein 1	NM_001002451.1
A3G1026	5.91	13.17	COPZ1	Coatomer subunit zeta-1	XM_009299363.4
A2G0131	5.91	3.00	DVL3	Segment polarity protein dishevelled homolog DVL-3	NM_131757.2
A16G0055	5.91	8.49	ATP6V1C1A	V-type proton ATPase subunit C 1-A	
A6G0771	5.91	37.61	EDEM1	ER degradation-enhancing alpha-mannosidase-like protein 1	NM_201189.2
A6G0623	5.91	7.02	RP2	Protein XRP2	NM_213446.1
B5G0015	5.91	0.97	G5714_006520	A0A7J6D4D5_9TELE Uncharacterized protein	XM_002661923.7
B13G0393	5.91	13.29	TSPAN15	Tetraspanin-15	XM_068224908.1
A8G0615	5.91	4.74			XM_009304058.4
B25G0822	5.91	3.07	RAB3IL1	Guanine nucleotide exchange factor for Rab-3A	
B2G0854	5.91	11.23	ZNF644	Zinc finger protein 644	NM_001278254.1
A11G0584	5.91	4.83	SOX13	Transcription factor SOX-13	NM_001320431.1
A7G0787	5.91	6.24	COG4	Conserved oligomeric Golgi complex subunit 4	NM_001039823.1
A2G0397	5.90	4.22	CNN3	Calponin-3	NM_001024073.2
B2G0720	5.90	11.11	ZSWIM5	Zinc finger SWIM domain-containing protein 5	NM_001144819.1
A20G0530	5.90	3.32	ZNF106	Zinc finger protein 106	
A11G0007	5.90	5.19	ATL2	Atlastin-2	NM_001077777.2
B16G0512	5.90	20.06	CTSS	Cathepsin S	XM_009292324.4
B6G0625	5.90	9.60	SLC9A7	hydrogen exchanger 7	NM_001030077.2
A13G0391	5.90	5.69	HPS6	BLOC-2 complex member HPS6	NM_001327924.1
B9G0293	5.90	4.80	ASMTL	methyltransferase protein	NM_213511.2
B21G0448	5.90	10.70	PPME1	Protein phosphatase methylesterase 1	NM_199937.1
B8G0025	5.90	3.58	IL17RE	Interleukin-17 receptor E	
B3G1417	5.90	3.48	NRBP2	U3JEJ8_FICAL Nuclear receptor binding protein 2	
B3G1735	5.90	13.63	IFI44	Interferon-induced protein 44	
A6G0844	5.90	4.55	LOC107708059	A0A673LY08_9TELE Uncharacterized LOC107708059	NM_001386780.1
B14G0666	5.90	4.35	TBC1D10B	TBC1 domain family member 10B	XM_005173146.5
B7G1202	5.90	3.77	GIMAP8	GTPase IMAP family member 8	XM_005166357.5
B4G0340	5.90	4.29	WIF1	Wnt inhibitory factor 1	NM_131229.2

B21G0117	5.89	3.12	LARS1	Leucine--tRNA ligase, cytoplasmic	XM_693187.7
A22G0632	5.89	27.65	SSUH2	Protein SSUH2 homolog	NM_001040377.1
B13G0690	5.89	5.82	GMFB	Glia maturation factor beta	XM_009307235.4
B18G0555	5.89	7.91	TBC1D15	TBC1 domain family member 15	NM_001172625.1
A8G0338	5.89	6.21	MPND	MPN domain-containing protein	NM_001076565.1
A21G0158	5.89	3.92	AGPAT1	1-acyl-sn-glycerol-3-phosphate acyltransferase alpha	NM_001077732.1
B16G0912	5.89	3.57	OSBPL3	Oxysterol-binding protein-related protein 3	XM_068214116.1
B5G0157	5.89	4.47	DDR2	Discoidin domain-containing receptor 2	XM_021474018.2
B3G0241	5.89	9.39	GVINP1	Interferon-induced very large GTPase 1	XM_021474816.2
B3G0171	5.89	4.15	CCDC134	Coiled-coil domain-containing protein 134	XM_003198004.6
B11G0086	5.89	0.68	RTASE	Probable RNA-directed DNA polymerase from transposon BS	XM_005169417.5
B19G0223	5.89	4.04	PYGO2	Pygopus homolog 2	NM_001033111.3
A14G0734	5.88	7.74	TCIRG1	V-type proton ATPase 116 kDa subunit a 3	XM_068213184.1
A12G0479	5.88	8.16	MGRN1	Probable E3 ubiquitin-protein ligase MGRN1	NM_001328544.1
B12G0010	5.88	3.90	BUB3	Mitotic checkpoint protein BUB3	NM_205709.1
A9G0749	5.88	6.33	USP37	Ubiquitin carboxyl-terminal hydrolase 37	XM_009304386.4
A4G0791	5.88	8.00	FLNC	Filamin-C	XM_009300205.4
A13G0344	5.88	4.30	VSTM4	V-set and transmembrane domain-containing protein 4	NM_001167661.1
B2G1013	5.88	16.58	WDR33	pre-mRNA 3' end processing protein WDR33	NM_001194928.1
B12G0763	5.88	19.35	SULT2B1	Sulfotransferase 2B1	NM_001078169.1
B25G0720	5.88	1.47	MYO5A	Unconventional myosin-Va	XM_009297774.4
B1G0107	5.87	4.15	FASTKD2	FAST kinase domain-containing protein 2, mitochondrial	XM_021475935.2
B2G0271	5.87	6.70	LIG1	DNA ligase 1	NM_001126388.1
B18G0614	5.87	5.90	MTSS2	Protein MTSS 2	XM_009293500.4
B22G1180	5.87	17.81	G2E3	M phase-specific E3 ubiquitin-protein ligase	
B15G0393	5.87	5.87	VPS26B	Vacuolar protein sorting-associated protein 26B	NM_213375.1
A11G0125	5.87	17.05	TAX1BP3	Tax1-binding protein 3	NM_001313759.1
A6G0906	5.87	3.86	UQCC1	Ubiquinol-cytochrome-c reductase complex assembly factor 1	NM_001076755.1
B5G0662	5.87	9.18	GAS1	Growth arrest-specific protein 1	NM_001111220.1
A11G0238	5.87	7.19	TBL1XR1	WD repeat-containing protein TBL1XR1	NM_001308551.1
B10G0558	5.87	8.03	SMG6	Telomerase-binding protein EST1A	NM_001423852.1
A7G0252	5.87	5.37	ANO1	Anoctamin-1	XM_009303473.4
B6G0215	5.87	6.45	CYP20A1	Cytochrome P450 20A1	NM_213332.2
A21G0817	5.87	8.48	TMEM164	Transmembrane protein 164	XM_021469256.2
B2G0053	5.87	6.09	ARMC6	Armadillo repeat-containing protein 6	XM_009298943.4
A8G0743	5.87	2.90	A0A673FFY0	A0A673FFY0_9TELE Uncharacterized protein	
B18G0457	5.86	6.79	MBTPS1	Membrane-bound transcription factor site-1 protease	NM_199213.2
B3G0850	5.86	7.83	SCAF1	serine-rich 19	XM_005164068.5
A13G0119	5.86	4.67	CANX	Calpain-1 catalytic subunit	NM_001077325.1

A11G0654	5.86	3.20	MED26	Mediator of RNA polymerase II transcription subunit 26	NM_001020669.1
A22G0339	5.86	5.15	DBT	Lipoamide acyltransferase component of branched-chain alpha-keto acid dehydrogenase complex, mitochondrial	NM_001013515.1
A11G0147	5.86	9.19	CRTC1	CREB-regulated transcription coactivator 1	XM_001333889.6
A3G0340	5.86	9.64	FN3KRP	Ketosamine-3-kinase	NM_001110108.1
A13G0161	5.86	0.98	ZMAT4	Zinc finger matrin-type protein 4	XM_068216355.1
A13G0450	5.86	9.66	CALHM2	Calcium homeostasis modulator protein 2	XM_009307394.4
B23G0436	5.86	2.83	GPR157	G-protein coupled receptor 157	NM_001034174.1
B17G0106	5.86	10.35	PTK2B	Protein-tyrosine kinase 2-beta	XM_005158577.5
B16G1115	5.86	4.79	TPBG	Trophoblast glycoprotein	NM_001252462.1
B21G0300	5.86	8.60	MAPK9	Mitogen-activated protein kinase 9	XM_001919653.7
B25G0416	5.86	0.67	RAD52	DNA repair protein RAD52 homolog	XM_005163057.5
B1G0481	5.86	6.20	UBE2A	Ubiquitin-conjugating enzyme E2 A	
B20G0169	5.86	6.09	NT5C1A	Cytosolic 5'-nucleotidase 1A	XM_685092.10
B12G0263	5.86	7.67	CCDC186	Coiled-coil domain-containing protein 186	XM_009306762.4
A14G0673	5.86	5.34	CTBP1	C-terminal-binding protein 1	NM_001040390.1
B22G0944	5.86	14.85	DUSP5	Dual specificity protein phosphatase 5	NM_212565.1
A5G0081	5.86	16.91	LIPO	Lipocalin	
B21G0358	5.86	2.77	SH2D4A	SH2 domain-containing protein 4A	NM_001003543.2
A7G1037	5.86	9.10	DOCK11	Dedicator of cytokinesis protein 11	XM_009303084.4
B5G0998	5.86	4.77	EHMT2	Histone-lysine N-methyltransferase EHMT2	XM_021475966.2
B23G0675	5.86	6.56	A0A673I1P2	A0A673I1P2_9TELE Si:ch211-210c8.7	
A3G1005	5.85	9.71	CASC3	Protein CASC3	NM_205716.1
B8G0144	5.85	7.08	KANSL3	KAT8 regulatory NSL complex subunit 3	XM_005167343.5
A1G0552	5.85	11.94	EDNRA	Endothelin-1 receptor	NM_001099445.2
A18G0474	5.85	28.84	RAB11A	Ras-related protein Rab-11A	NM_001007359.1
B9G0704	5.85	9.64	UXS1	UDP-glucuronic acid decarboxylase 1	NM_173242.2
B11G0471	5.85	10.71	ACAD9	Complex I assembly factor ACAD9, mitochondrial	XM_068224367.1
A23G0758	5.85	6.15	OTUD3	OTU domain-containing protein 3	NM_212922.1
B17G0279	5.85	10.12	CLMN	Calmin	XM_002665160.6
A20G0457	5.85	28.47	THBS1	Thrombospondin-1	XM_685303.10
A9G0172	5.85	8.21	CTDSP1	Carboxy-terminal domain RNA polymerase II polypeptide A small phosphatase 1	XM_009304902.4
B25G0819	5.85	5.83	GTF2H1	General transcription factor IIH subunit 1	NM_001004596.1
A17G0175	5.85	8.37	COQ6	Ubiquinone biosynthesis monooxygenase COQ6, mitochondrial	NM_001045404.1
A19G0276	5.85	2.80	GMNN	Geminin	
A1G0928	5.84	5.09	HPF1	Histone PARylation factor 1	NM_001005769.1
A9G0410	5.84	1.56	LOC113053220	Leu-3	XM_021472194.2
A25G0681	5.84	5.70	IGKV4-1	Immunoglobulin kappa variable 4-1	XM_068217596.1

A5G0898	5.84	5.61	SETBP1	SET-binding protein	XM_005165675.5
B24G0077	5.84	15.14	IGKV1-13	Immunoglobulin kappa variable 1-13	NM_001045408.1
A6G0721	5.84	5.47	MUSTN1	Musculoskeletal embryonic nuclear protein 1	
A11G0028	5.84	9.16	AFMID	Kynurenine formamidase	NM_213275.2
A16G0997	5.84	5.06	YRDC	Threonylcarbamoyl-AMP synthase	NM_001083867.2
A6G0427	5.84	7.11	PIK3R5	Phosphoinositide 3-kinase regulatory subunit 5	XM_009302367.4
B22G0699	5.84	23.24	ARHGAP45	Rho GTPase-activating protein 45	XM_068216452.1
B9G0853	5.84	19.91	SCRN3	Secernin-3	XM_009304428.4
B2G1040	5.84	7.71	MIB1	E3 ubiquitin-protein ligase mib1	XM_009296844.4
B1G1196	5.84	12.01	TMC7	Transmembrane channel-like protein 7	
B13G0513	5.84	22.76	BORCS7	BLOC-1-related complex subunit 7	NM_213252.1
B21G0871	5.84	5.88	SMAD7	Mothers against decapentaplegic homolog 7	NM_175082.3
A20G0849	5.84	7.70	FYNB	Tyrosine-protein kinase fynb	XM_068215552.1
B20G0378	5.84	3.44	KATNA1	Katanin p60 ATPase-containing subunit A1	NM_001020604.1
A3G1202	5.83	2.55	NATD1	Protein NATD1	XM_003198184.6
B8G0472	5.83	3.82	DDX20	Probable ATP-dependent RNA helicase DDX20	XM_068222967.1
B8G0580	5.83	8.14	PPARD	Peroxisome proliferator-activated receptor delta	NM_131468.2
B1G0704	5.83	3.65	F1QCI3	F1QCI3_DANRE IG domain-containing protein	
B15G0683	5.83	9.25	SPART	Spartin	
A7G0937	5.83	7.74	TNFSF10	Tumor necrosis factor ligand superfamily member 10	XM_005172247.5
B22G0850	5.83	7.53	B3GALT2	Beta-1,3-galactosyltransferase 2	NM_001144810.1
A1G1086	5.83	5.28	LRBA	Lipopolysaccharide-responsive and beige-like anchor protein	XM_009291242.4
B13G0170	5.83	7.43	THADA	Thyroid adenoma-associated protein homolog	NM_001145594.1
A25G0047	5.83	5.75	GOLT1B	Vesicle transport protein GOT1B	NM_001031671.2
B14G0230	5.83	1.82	LOC113113952	A0A6P6QSE5_CARAU uncharacterized protein LOC113113952 isoform X1	XM_068213367.1
B1G0526	5.83	6.07	WBP1L	WW domain binding protein 1-like	NM_001020631.1
B22G0708	5.83	9.50	BORCS8	BLOC-1-related complex subunit 8	NM_001098247.2
B9G0652	5.82	10.59	BMPR2	Bone morphogenetic protein receptor type-2	NM_001039807.1
B8G0731	5.82	22.70	DIO1	Type I iodothyronine deiodinase	
A1G1076	5.82	11.11	UBE2K	Ubiquitin-conjugating enzyme E2 K	NM_001008611.2
A17G0366	5.82	19.23	HECTD1	E3 ubiquitin-protein ligase HECTD1	XM_009293112.4
A3G1070	5.82	6.69	BPTF	Nucleosome-remodeling factor subunit BPTF	XM_017354770.3
B5G0589	5.82	5.03	CDK7	Cyclin-dependent kinase 7	NM_212961.2
A3G0509	5.82	18.06	FLII	Protein flightless-1 homolog	NM_001257146.1
B2G0273	5.82	46.53			NM_194361.2
B4G0648	5.82	5.67	K0930	Uncharacterized protein KIAA0930 homolog	NM_001327783.1

A1G0421	5.81	1.06	LOC113065508	A0A6P6M7M2_CARAU complement receptor type 1-like isoform X2	
A18G0499	5.81	5.75	RSPRY1	RING finger and SPRY domain-containing protein 1	NM_001045072.2
B22G0475	5.81	5.34	LOC113079613	A0A6P6NHM2_CARAU uncharacterized protein LOC113079613	XM_001336832.9
A20G0604	5.81	2.73	DAAM1	Disheveled-associated activator of morphogenesis 1	NM_001030136.2
A5G0287	5.81	4.99	SSH1	Protein phosphatase Slingshot homolog 1	XM_005165198.5
A11G0254	5.81	7.48	TMF1	TATA element modulatory factor	NM_001099982.2
A19G0838	5.81	4.28	RNF5	E3 ubiquitin-protein ligase RNF5	XM_003200504.6
B10G0637	5.81	5.01	CRY2	Cryptochrome-2	XM_005155405.4
B24G0382	5.81	2.89	MTFR1	Mitochondrial fission regulator 1	NM_001008578.1
B22G0535	5.81	6.88	MFSD14A	Hippocampus abundant transcript 1 protein	NM_213527.2
A18G0464	5.81	4.91	AAGAB	Alpha- and gamma-adaptin-binding protein p34	NM_001002423.1
B13G0352	5.81	3.80	ATRNL1	Attractin-like protein 1	NM_001009985.2
A10G0184	5.81	13.41	RAB7	Ras-related protein rab7	NM_001005591.1
A20G0349	5.81	0.27	LOC113062944	A0A6P6LWM0_CARAU uncharacterized protein LOC113062944	
B20G0161	5.81	5.50	SI:DKEY-29F10.1	Protein C10	NM_212646.2
A19G0681	5.81	13.85	ASH1L	Histone-lysine N-methyltransferase ASH1L	NM_001346407.1
B21G0018	5.81	9.25	PCBD2	Pterin-4-alpha-carbinolamine dehydratase 2	
A17G0370	5.81	0.81	G5714_017084	A0A7J6C5K4_9TELE F-box domain-containing protein	
B12G0733	5.80	4.68	ITGAX	Integrin alpha-X	XM_005156007.5
B16G0890	5.80	6.86	ANKRD28	threonine-protein phosphatase 6 regulatory ankyrin repeat subunit A	XM_009292265.4
B15G0500	5.80	4.58	HTL	Fibroblast growth factor receptor homolog 1	XM_688510.8
B9G0560	5.80	4.97	TMEM177	Transmembrane protein 177	NM_199594.1
A3G0862	5.80	7.96	EHD4	EH domain-containing protein 4	XM_702892.9
A25G0056	5.80	5.63	INCENP-A	Inner centromere protein A	XM_009297716.4
A5G0327	5.80	1.80	LOC113048846	A0A6P6K3W2_CARAU uncharacterized protein LOC113048846 isoform X1	XM_021471581.2
B14G0114	5.80	14.47	CCDC69	Coiled-coil domain-containing protein 69	NM_001423999.1
B24G0098	5.80	12.23	MPRIP	Myosin phosphatase Rho-interacting protein	XM_068217286.1
B17G0004	5.80	3.95	LRR1	Leucine-rich repeat protein 1	
B25G0721	5.80	5.49	FCSK	L-fucose kinase	XM_009297770.4
A13G0370	5.80	10.90	HIF1AN	Hypoxia-inducible factor 1-alpha inhibitor	XM_009307277.4
A10G0015	5.80	13.40	LRRC32	Transforming growth factor beta activator LRRC32	
A9G0503	5.80	5.21	ARL5A	ADP-ribosylation factor-like protein 5A	NM_001002339.1
B8G1043	5.80	4.73	LRRC2	Leucine-rich repeat-containing protein 2	

A25G0601	5.79	5.68	SPTY2D1	Protein SPT2 homolog	XM_005170676.5
A14G0671	5.79	4.81	LOC113069581	A0A6P6MPC7_CARAU uncharacterized protein LOC113069581 isoform X1	XM_021472768.2
B9G0606	5.79	6.20	ZBTB11	Zinc finger and BTB domain-containing protein 11	XM_682097.8
B1G0755	5.79	13.25	ATF7IP	Activating transcription factor 7-interacting protein 1	NM_001077603.2
B2G1020	5.79	9.24	GNB2	G(T) subunit beta-2	XM_021466697.2
A14G0130	5.79	4.29	SMAD5	Mothers against decapentaplegic homolog 5	NM_001346309.1
A15G0348	5.79	12.65	GDPD5	Glycerophosphodiester phosphodiesterase domain-containing protein 5	XR_222297.5
B2G1142	5.79	19.85	JAK1	Tyrosine-protein kinase JAK1	
B25G0724	5.79	3.19	IFITM5	Interferon-induced transmembrane protein 5	NM_001177312.2
A7G0243	5.79	6.79	DHCR7	7-dehydrocholesterol reductase	NM_201330.2
A6G0765	5.79	2.30	MST1R	Macrophage-stimulating protein receptor	XM_068222037.1
A12G0154	5.79	7.76	UNC13D	Protein unc-13 homolog D	XM_009306839.3
B14G0787	5.79	2.94	ABCB7	Iron-sulfur clusters transporter ABCB7, mitochondrial	NM_001423974.1
A25G0369	5.79	0.56	LOC113043289	A0A6P6JDR4_CARAU Glutaminase	XM_009297984.4
B3G0015	5.78	3.87	A0A673LUR3	A0A673LUR3_9TELE Uncharacterized protein	XM_021475149.2
A19G0398	5.78	5.18	DLX5A	Homeobox protein Dlx5a	NM_131306.2
B7G0738	5.78	6.18	RDH11	Retinol dehydrogenase 11	XM_005168869.5
B20G0284	5.78	7.52	CDC42BPB	threonine-protein kinase MRCK beta	XM_021468493.2
B23G0479	5.78	8.92	CASP9	Caspase-9	NM_001007404.2
B5G0383	5.78	6.90	ACAC	Acetyl-CoA carboxylase	NM_001271308.1
B13G0688	5.78	9.43	SYNJ2BP	Synaptojanin-2-binding protein	XM_021472697.2
A21G0727	5.78	5.01	RBM41	RNA-binding protein 41	NM_001007405.2
B12G0331	5.78	6.63	MSL1	Male-specific lethal 1 homolog	XM_686625.10
A5G0298	5.78	5.07	INE	Sodium- and chloride-dependent GABA transporter ine	NM_001110461.2
B24G0702	5.77	4.55	INHBA	Inhibin beta A chain	NM_130916.1
B5G1109	5.77	8.27	DGCR8	Microprocessor complex subunit DGCR8	NM_001122749.1
A1G0077	5.77	4.34	NLRC3	Protein NLRC3	XM_009295700.4
A6G0559	5.77	5.21	ATG4C	Cysteine protease ATG4C	NM_001002103.1
B5G1335	5.77	6.25	PSD3	PH and SEC7 domain-containing protein 3	XM_009299757.4
A6G0172	5.77	9.70	CAVIN2	Caveolae-associated protein 2	NM_001004584.1
A11G0570	5.77	3.92	ITIH3	Inter-alpha-trypsin inhibitor heavy chain H3	NM_212642.1
B9G0068	5.77	11.85	MOSPD2	Motile sperm domain-containing protein 2	NM_001007293.2
B1G0142	5.77	6.84	ALKBH5	RNA demethylase ALKBH5	NM_001077387.1
B21G0312	5.77	3.14	TPST1	Protein-tyrosine sulfotransferase 1	NM_200202.2
B19G0390	5.77	0.90	HOXA13A	Homeobox protein Hox-A13a	NM_001085494.1
A6G0591	5.77	7.91	IL12RB2	Interleukin-12 receptor subunit beta-2	XM_021476742.2
A13G0375	5.76	9.37	CDHR1	Cadherin-related family member 1	XM_005156904.5
B24G0323	5.76	2.74			
B9G0747	5.76	5.96	SLC37A1	Glucose-6-phosphate exchanger SLC37A1	XM_021478645.2

A8G0486	5.76	4.74	SORT1	Sortilin	NM_001126465.1
B23G0803	5.76	10.86	OTUD3	OTU domain-containing protein 3	NM_212922.1
B2G0744	5.76	2.22	BEST4	Bestrophin-4	XM_687068.7
A20G0091	5.76	3.10	EIF2S1	Eukaryotic translation initiation factor 2 subunit 1	NM_131800.2
A5G1347	5.76	4.39	RPS6KA4	Ribosomal protein S6 kinase alpha-4	XM_005165615.5
B16G1046	5.76	8.95	LOC113111807	A0A6P6QGI0_CARAU Nuclear apoptosis-inducing factor 1	
B11G0268	5.76	8.21	MRPL4	39S ribosomal protein L4, mitochondrial	NM_001079955.1
B8G0457	5.76	3.56	SRXN1	Sulfiredoxin-1	
A24G0025	5.76	2.91			
B23G0482	5.76	3.51	IDH3G	Isocitrate dehydrogenase [NAD] subunit gamma 1, mitochondrial	NM_001243172.1
B24G0586	5.75	5.01	KIAA1217	Sickle tail protein homolog	XM_021470282.2
B1G0568	5.75	4.21	GAB1	GRB2-associated-binding protein 1	XM_021470020.2
B7G1057	5.75	5.59	MEN1	Menin	NM_130953.1
A2G0347	5.75	5.72	GADD45A	Growth arrest and DNA damage-inducible protein GADD45 alpha	NM_200576.2
A7G0361	5.75	7.33	ADAM19	Disintegrin and metalloproteinase domain-containing protein 19	XM_005172361.5
B20G0371	5.75	4.99	CBR4	Carbonyl reductase family member 4	NM_205656.2
A23G0686	5.75	1.57	HOXC3A	Homeobox protein Hox-C3a	NM_001134685.2
B16G1007	5.75	4.44	ICE1	Little elongation complex subunit 1	NM_001034965.3
A12G0838	5.75	7.28	ABCC3	ATP-binding cassette sub-family C member 3	XM_009306370.4
A1G1112	5.75	23.53	ART1	GPI-linked NAD(P)(+)--arginine ADP-ribosyltransferase 1	
A12G0573	5.75	3.68	LOC113065508	A0A6P6M7M2_CARAU complement receptor type 1-like isoform X2	
A20G0807	5.75	4.33	FAM89A	Protein FAM89A	
A15G0086	5.75	9.22	NLRC3	NLR family CARD domain-containing protein 3	
A16G0002	5.75	2.57	POP1	MRP protein subunit POP1	XM_068214292.1
B19G0570	5.75	9.02	ZMYM4	Zinc finger MYM-type protein 4	XM_005159706.5
A6G0622	5.75	5.21	JADE3	Protein Jade-3	NM_201652.2
A2G0633	5.75	25.03			XM_702758.9
B23G0766	5.75	10.75	CD40	Tumor necrosis factor receptor superfamily member 5	
B10G0805	5.75	7.08	MARVELD2	MARVEL domain-containing protein 2	XM_009305076.4
A23G0740	5.75	6.59	TENT5A	Terminal nucleotidyltransferase 5A	NM_001103125.1
B18G0502	5.75	6.94	SMAD3	Mothers against decapentaplegic homolog 3	NM_175083.2
B23G0779	5.74	11.21	RNF146	E3 ubiquitin-protein ligase rnf146	XM_068216685.1
A1G0657	5.74	12.10	GK	Glycerol kinase	NM_001431102.1
A16G0891	5.74	3.60	PTPN2	Tyrosine-protein phosphatase non-receptor type 2	NM_200466.2
B7G0566	5.74	2.31	CREB3L1	Cyclic AMP-responsive element-binding protein 3-like protein 1	XM_005168966.4

A2G0118	5.74	6.56	PRKAG2	5'-AMP-activated protein kinase subunit gamma-2	XM_005163300.5
B25G0171	5.74	0.38	IGKV4-1	Immunoglobulin kappa variable 4-1	XM_068217596.1
B21G0468	5.74	6.94	CADM1	Cell adhesion molecule 1	XM_005161197.5
A3G1225	5.74	6.10	USP36	Ubiquitin carboxyl-terminal hydrolase 36	NM_001305580.1
A24G0341	5.74	9.40	PDE7A	High affinity cAMP-specific 3',5'-cyclic phosphodiesterase 7A	XM_068217018.1
B16G0625	5.74	4.98	ZFAT	Zinc finger protein ZFAT	NM_001423169.1
B11G0209	5.74	7.77	MOB3C	MOB kinase activator 3C	XM_005155960.4
B14G0004	5.74	3.04	EVC2	Limbin	
A22G0717	5.74	2.06	COMMD2	COMM domain-containing protein 2	
A2G0585	5.74	3.88	ZGC:56556	UPF0489 protein C5orf22 homolog	NM_200331.1
A3G1167	5.74	5.71	JMJD6	Bifunctional arginine demethylase and lysyl-hydroxylase JMJD6	NM_170761.3
A5G0274	5.74	4.16	TCHP	Trichoplein keratin filament-binding protein	NM_001040343.1
A11G0055	5.74	4.36	SHROOM2	Protein Shroom2	NM_001083310.2
A4G0983	5.74	4.08	PMPCB	Mitochondrial-processing peptidase subunit beta	NM_001012496.2
A5G0847	5.74	2.95	ARRDC3	Arrestin domain-containing protein 3	NM_001017889.1
B17G0360	5.74	1.35	PLA2G4F	Cytosolic phospholipase A2 zeta	XM_009293030.4
A13G0662	5.74	4.00	DCAF10	DDB1- and CUL4-associated factor 10	XM_068213082.1
B11G0490	5.74	4.38	NMUR2	Neuromedin-U receptor 2	XM_009306155.4
B3G0724	5.74	7.77	CARM1	Histone-arginine methyltransferase CARM1	NM_001003645.2
B20G0605	5.73	3.91	ELP3	Elongator complex protein 3	NM_001014322.2
B4G0242	5.73	3.72	IPO8	Importin-8	NM_001044334.2
A1G0719	5.73	6.03	TPP2	Tripeptidyl-peptidase 2	XM_002660321.7
A19G0128	5.73	8.00	RNF19A	E3 ubiquitin-protein ligase RNF19A	NM_001326695.1
B6G0738	5.73	3.62	SFI1	Protein SFI1 homolog	XM_683537.9
B9G0266	5.73	11.56	SLC49A4	Solute carrier family 49 member 4	NM_001004597.1
B9G0468	5.73	5.94	MMADHC	Cobalamin trafficking protein CblD	NM_205594.2
B24G0178	5.73	3.21	DCAKD	Dephospho-CoA kinase domain-containing protein	NM_001320180.1
B24G0174	5.73	10.94	KCNC1	Potassium voltage-gated channel subfamily C member 1	XM_021470205.2
B18G0253	5.73	3.88	PDCD5	Programmed cell death protein 5	NM_201177.1
A19G0539	5.72	4.33	TAX1BP1A	Tax1-binding protein 1 homolog A	NM_001346178.1
A3G0050	5.72	7.09	TRIM16	Tripartite motif-containing protein 16	NM_001423725.1
B8G1046	5.72	4.06	NUDT12	NAD-capped RNA hydrolase NUDT12	XM_021478254.2
B19G0393	5.72	8.78	TAX1BP1A	Tax1-binding protein 1 homolog A	NM_001346178.1
A22G0701	5.72	5.25	MAP1S	Microtubule-associated protein 1S	XM_021473552.2
B12G0454	5.72	3.82	CDC42EP1	Cdc42 effector protein 1	XM_021480298.2
A16G0304	5.72	5.59	PLEKHO1	Pleckstrin homology domain-containing family O member 1	NM_001326535.1

B14G0585	5.72	4.25	SLC25A51	Mitochondrial nicotinamide adenine dinucleotide transporter SLC25A51	NM_001386244.1
B9G0208	5.72	5.95	CTDSP1	Carboxy-terminal domain RNA polymerase II polypeptide A small phosphatase 1	XM_021479012.2
A25G0356	5.72	4.54	TDG	T mismatch-specific thymine DNA glycosylase	NM_001020751.1
B2G0930	5.72	3.36	SI:DKEYP-13A3.3	Probable flap endonuclease 1 homolog	NM_001017611.2
B7G1134	5.72	3.05	OR131-2	Odorant receptor 131-2	XM_005172217.5
B12G0115	5.71	6.33	PPP2R2D	threonine-protein phosphatase 2A 55 kDa regulatory subunit B delta isoform	NM_212880.2
A12G0493	5.71	4.47	ZDHHC4	Palmitoyltransferase ZDHHC4	NM_200049.2
A16G0198	5.71	4.37	CCDC127	Coiled-coil domain-containing protein 127	XM_021466750.2
B12G0319	5.71	6.23	NBR1	Next to BRCA1 gene 1 protein	NM_001305595.1
B5G0946	5.71	10.33	UBE2G1	Ubiquitin-conjugating enzyme E2 G1	NM_199863.1
A7G1331	5.71	0.54	NEK1	threonine-protein kinase Nek1	XM_068222504.1
B20G0827	5.71	10.76	GNPAT	Dihydroxyacetone phosphate acyltransferase	XM_002665777.7
B6G0685	5.71	8.18	PFKFB1	fructose-2,6-bisphosphatase 1	NM_001430986.1
A6G0557	5.71	2.64	ALG6	Dolichyl pyrophosphate Man9GlcNAc2 alpha-1,3-glucosyltransferase	NM_001003784.2
B13G0707	5.71	4.07	TRIM9	E3 ubiquitin-protein ligase TRIM9	XM_005156693.5
B19G0038	5.71	11.11	TGFBR2	TGF-beta receptor type-2	
B5G0714	5.71	6.09	PIAS2	E3 SUMO-protein ligase PIAS2	XM_005165396.4
A2G0823	5.70	5.34	SKIL	Ski-like protein	XM_017351932.3
B9G0528	5.70	9.82	ACVR2A	Activin receptor type-2A	XM_005167561.5
A4G0060	5.70	6.82	A0A673LUR3	A0A673LUR3_9TELE Uncharacterized protein	XM_068213652.1
A7G0581	5.70	8.18	ACP2	Lysosomal acid phosphatase	NM_001013337.2
B10G0698	5.70	6.07	KCTD9	POZ domain-containing protein KCTD9	NM_001002738.1
B14G0341	5.70	13.17	BTK	Tyrosine-protein kinase BTK	XM_068218779.1
B5G0032	5.70	4.31	ROHU_009172	A0A498M0V7_LABRO AIG1-type G domain-containing protein	NM_001386526.1
A11G0016	5.69	10.38	TRAPPC10	Trafficking protein particle complex subunit 10	
B16G0230	5.69	4.82	NUDT17	Nucleoside diphosphate-linked moiety X motif 17	NM_001030103.1
B3G0156	5.69	1.65	ROHU_033666	A0A498LC53_LABRO Nuclease HARBI1	
B12G0617	5.69	9.47	ROHU_005260	A0A498NSF3_LABRO Transcription factor Adf-1-like isoform X2	XM_678327.9
B20G0761	5.69	11.17	MMUT	Methylmalonyl-CoA mutase, mitochondrial	NM_001099226.1
B17G0305	5.69	12.71	VRK1	threonine-protein kinase VRK1	NM_213385.1
A20G0402	5.69	30.60	SESN1	Sestrin-1	XM_005160671.5
B2G0853	5.69	5.16	LRRC8D	Volume-regulated anion channel subunit LRRC8D	XM_002660814.7
B14G0202	5.69	6.39	MOSPD1	Motile sperm domain-containing protein 1	NM_213369.1
A11G0387	5.69	8.93	OTUD5A	OTU domain-containing protein 5-A	XM_021479833.2

B5G0311	5.69	8.25	CLIP1	CAP-Gly domain-containing linker protein 1	XM_009301992.4
A16G0769	5.69	13.00	FDPS	Farnesyl pyrophosphate synthase	NM_001025471.1
B9G0789	5.69	4.20	G5714_010378	A0A7J6CRS3_9TELE Ig-like domain-containing protein	
A8G0152	5.68	8.87	HCFC1	Host cell factor 1	NM_001128537.1
A24G0119	5.68	6.78	TELO2	Telomere length regulation protein TEL2 homolog	NM_001077741.1
B9G0374	5.68	6.35	TMTC4	Protein O-mannosyl-transferase TMTC4	NM_001173502.1
B10G0499	5.68	4.30	TBL2	Transducin beta-like protein 2	NM_212767.1
B18G0705	5.68	1.75	CERS2	Ceramide synthase 2	XM_005158997.5
A20G0519	5.68	1.44	MTHFD1L	Monofunctional C1-tetrahydrofolate synthase, mitochondrial	NM_001242996.1
B20G0307	5.68	3.47	NME6	Nucleoside diphosphate kinase 6	
B20G0318	5.68	3.52	PRKCH	Protein kinase C eta type	XM_005160500.5
A6G0900	5.68	7.17	ZC3H10	Zinc finger CCCH domain-containing protein 10	NM_207098.1
A6G0878	5.68	4.04	ERGIC3	Endoplasmic reticulum-Golgi intermediate compartment protein 3	NM_001033105.1
A6G0636	5.68	9.03	UBE3A	Ubiquitin-protein ligase E3A	NM_001007318.1
A5G0767	5.68	7.54	PARP8	Protein mono-ADP-ribosyltransferase PARP8	NM_001077175.1
A2G0719	5.68	13.15	SMG7	Nonsense-mediated mRNA decay factor SMG7	XM_005171308.5
B19G0494	5.67	14.84	SESN2	Sestrin-2	NM_001079975.2
A4G0889	5.67	9.01	VEGFAA	Vascular endothelial growth factor A-A	XM_005164518.5
A23G0581	5.67	3.66	SELENOI	Ethanolaminephosphotransferase 1	NM_001329437.1
B4G0210	5.67	2.93	NUDT5	ADP-sugar pyrophosphatase	NM_001002086.1
B1G1086	5.67	2.39	TRIM35	E3 ubiquitin-protein ligase TRIM35	NM_001018146.1
B6G0284	5.67	3.00	CASP8	Caspase-8	
A16G0363	5.67	4.90	RRAGC	Ras-related GTP-binding protein C	NM_001020635.1
B8G0263	5.67	3.43	RAB11FIP1	Rab11 family-interacting protein 1	XM_003199114.6
B3G1446	5.67	9.60	SAMD9L	Sterile alpha motif domain-containing protein 9-like	XM_021474496.2
B25G0689	5.67	4.11	UNC45A	Protein unc-45 homolog A	
A14G0399	5.67	5.72	FERMT3	Fermitin family homolog 3	NM_200904.2
A1G0105	5.67	2.83	LOC113067422	A0A6P6MHX5_CARAU uncharacterized protein LOC113067422 isoform X2	
A21G0784	5.67	3.85	BOD1	Biorientation of chromosomes in cell division protein 1	
B8G0048	5.67	11.09	SELENOK	Selenoprotein K	
B5G0139	5.67	8.50	TNKS	Poly [ADP-ribose] polymerase tankyrase-1	XM_005171745.5
A20G0437	5.67	4.46	MBOAT2	Lysophospholipid acyltransferase 2	NM_001278602.1
B12G0371	5.66	3.14	MSH2	DNA mismatch repair protein Msh2	NM_213524.1
B21G1009	5.66	12.43	KLHL8	Kelch-like protein 8	NM_001110480.2
A2G0658	5.66	3.77	OTULIN	Ubiquitin thioesterase otulin	NM_001166015.1
A8G0380	5.66	6.64	TOR1AIP2	Torsin-1A-interacting protein 2	XM_001339566.9
A2G0820	5.66	10.43	SEC62	Translocation protein SEC62	NM_001025530.1
B25G0373	5.66	12.18	WASHC4	WASH complex subunit 4	XM_689025.9

B3G0238	5.66	5.96	HOOK2	Protein Hook homolog 2	NM_001423849.1
A1G0129	5.66	0.41	GIMAP7	GTPase IMAP family member 7	XM_068220946.1
A20G0732	5.66	4.76	ECHDC2	Enoyl-CoA hydratase domain-containing protein 2, mitochondrial	NM_212788.2
B2G0718	5.66	21.05	LYN	Tyrosine-protein kinase Lyn	NM_001004543.1
B10G0081	5.66	7.64	IL11RA	Interleukin-11 receptor subunit alpha	NM_001113499.3
A2G0684	5.66	4.24	TMEM53	Transmembrane protein 53	NM_001002637.1
A15G0615	5.66	3.19	RAB34	Ras-related protein Rab-34	XM_005157422.5
B1G0271	5.66	5.55	PI4K2B	Phosphatidylinositol 4-kinase type 2-beta	NM_001045485.2
B14G0178	5.65	26.86	FOSL1	Fos-related antigen 1	XM_009291125.4
A20G0703	5.65	3.19	OMA1	Metalloendopeptidase OMA1, mitochondrial	NM_001423841.1
B23G0270	5.65	4.65	TCEA2	Transcription elongation factor A protein 2	NM_200986.2
B12G0580	5.65	4.88	DROSHA	Ribonuclease 3	NM_001110472.1
B7G0387	5.65	4.33	SLC38A7	Putative sodium-coupled neutral amino acid transporter 7	XM_068222234.1
B22G0185	5.65	6.74	NCL1	Nicalin-1	NM_207057.1
B6G1031	5.65	3.82			
B11G0422	5.65	9.80	SHISA4	Protein shisa-4	NM_001077792.2
A17G0485	5.65	5.72	PEX13	Peroxisomal membrane protein PEX13	NM_200645.1
B17G0808	5.65	7.88	LOC113046332	A0A6P6JV48_CARAU protein ALP1-like	
A21G0208	5.65	0.31	DNAJC21	DnaJ homolog subfamily C member 21	NM_200044.1
A6G0817	5.65	4.14	TESPA1	Protein TESPA1	XM_002662524.7
A11G0143	5.65	2.92	RAB3A	Ras-related protein Rab-3A	NM_001002297.1
B24G0349	5.64	4.89	MAP3K15	Mitogen-activated protein kinase kinase kinase 15	XM_021473796.2
A7G0469	5.64	7.54	SIN3A	Paired amphipathic helix protein Sin3a	XM_002662877.7
A5G0176	5.64	1.60	VSIG10	V-set and immunoglobulin domain-containing protein 10	
B21G0226	5.64	3.90	UBLCP1	Ubiquitin-like domain-containing CTD phosphatase 1	NM_001199375.1
A15G0438	5.64	5.70	TMEM199	Transmembrane protein 199	NM_001105597.1
A3G0844	5.64	7.75	PIGQ	Phosphatidylinositol N-acetylglucosaminyltransferase subunit Q	NM_199429.1
B20G0728	5.64	6.74	NUS1	Dehydrodolichyl diphosphate synthase complex subunit nus1	NM_001002356.1
A10G0507	5.64	1.21	G5714_010894	A0A7J6CNI8_9TELE Uncharacterized protein	
B7G0859	5.64	4.02	EXOC6B	Exocyst complex component 6B	XM_021477610.2
A5G0476	5.64	11.27	PPP3CC	threonine-protein phosphatase 2B catalytic subunit gamma isoform	NM_001166628.1
B11G0749	5.64	4.19	PPRC1	Peroxisome proliferator-activated receptor gamma coactivator-related protein 1	
B21G0070	5.63	4.75	ZCCHC10	Zinc finger CCHC domain-containing protein 10	NM_001089339.2
A12G0711	5.63	0.46	DLX4B	Homeobox protein Dlx4b	NM_131318.1
A1G0726	5.63	2.55	C3AR1	C3a anaphylatoxin chemotactic receptor	
A15G0353	5.63	5.00	RAP1GAP2	Rap1 GTPase-activating protein 2	XM_017350967.3
B12G0014	5.63	8.42	FAM53B	Protein FAM53B	NM_001007187.1

B5G1147	5.63	4.65	TBC1D8B	TBC1 domain family member 8B	XM_009301353.4
A18G0316	5.63	11.13	CLASRP	arginine rich protein	NM_001044329.1
B6G0628	5.63	12.48	CYFIP1	Cytoplasmic FMR1-interacting protein 1 homolog	NM_212759.1
B16G0033	5.63	2.94	EOMES	Eomesodermin	
A23G0174	5.63	12.54	G6PD	Glucose-6-phosphate 1-dehydrogenase	XM_021474507.2
B25G0293	5.63	2.81	G5714_024356	A0A7J6BJS2_9TELE Uncharacterized protein	XM_009298146.4
A7G0220	5.63	6.23	TEAD1	Transcriptional enhancer factor TEF-1	XM_009303583.4
B8G0771	5.62	5.07	FAF1	FAS-associated factor 1	NM_212973.2
A1G0333	5.62	1.77	PDCD11	Protein RRP5 homolog	XM_005160117.5
B20G0438	5.62	10.13	SERTAD2	SERTA domain-containing protein 2	NM_001075116.2
A18G0022	5.62	3.17	CD276	CD276 antigen	
B19G0935	5.62	4.41	P3H4	Endoplasmic reticulum protein SC65	NM_001126438.1
A1G0629	5.62	20.62	TM9SF2	Transmembrane 9 superfamily member 2	NM_212728.1
A4G0720	5.62	11.46	TNPO3	Transportin-3	NM_201087.1
B2G0381	5.62	9.29	A33	Zinc-binding protein A33	
A18G0318	5.62	3.47	BCL11A	leukemia 11A	NM_001044319.1
B3G1501	5.62	9.20	RTASE	Probable RNA-directed DNA polymerase from transposon BS	XM_068214189.1
B17G0463	5.62	3.50	ARHGEF10	Rho guanine nucleotide exchange factor 10	XM_009293079.4
A12G0805	5.62	9.21	UBTD1	Ubiquitin domain-containing protein 1	NM_001030265.1
A17G0507	5.62	3.12	LTBP1	Latent-transforming growth factor beta-binding protein 1	XM_017351812.3
A11G0764	5.62	7.63	LOC113068143	A0A6P6MJJ5_CARAU uncharacterized protein LOC113068143	XM_009306333.3
A16G0161	5.61	10.30	SMG5	Nonsense-mediated mRNA decay factor SMG5	NM_001024923.1
B12G0646	5.61	2.76	MYOF	Myoferlin	XM_068224535.1
A1G0235	5.61	12.00	XPO1	Exportin-1	XM_009294702.4
A25G0535	5.61	3.58	SLC27A2	Long-chain fatty acid transport protein 2	NM_001386644.1
B20G0348	5.61	2.33	SRD5A3	Polyprenol reductase	NM_001044939.1
B3G0985	5.61	5.15	METTL22	Methyltransferase-like protein 22	NM_001177460.1
B19G0406	5.61	8.63	PTPN23	Tyrosine-protein phosphatase non-receptor type 23	XM_005159517.5
A6G0268	5.61	3.58	CASP8	Caspase-8	NM_001098619.1
A19G0425	5.61	1.44	SYTL1	Synaptotagmin-like protein 1	NM_001128533.1
A10G0453	5.61	6.54	CRTAM	Cytotoxic and regulatory T-cell molecule	XM_021472313.2
B23G0161	5.61	6.44	LOC113041019	A0A6P6JB77_CARAU uncharacterized protein LOC113041019	NM_001128815.1
A7G0274	5.61	3.38	ADAMTS7	A disintegrin and metalloproteinase with thrombospondin motifs 7	XM_021478001.2
A22G0693	5.61	13.87	FXR1	RNA-binding protein FXR1	NM_201301.2
A10G0524	5.61	2.18	TIAM1	Rho guanine nucleotide exchange factor TIAM1	XM_001924009.8
B23G0314	5.61	8.90	TFIP11	Tuftelin-interacting protein 11	NM_001002721.1
A14G0570	5.61	3.31	PCGF1	Polycomb group RING finger protein 1	NM_001007158.2

A6G0505	5.61	5.17	LPP	Lipoma-preferred partner homolog	XM_068221782.1
B25G0118	5.61	2.81	AFG3L1	AFG3-like protein 1	XM_005163207.5
A22G0369	5.61	11.54	IFI44L	Interferon-induced protein 44-like	XM_021469588.2
B24G0695	5.61	12.83	WDR37	WD repeat-containing protein 37	XM_009297244.4
A24G0244	5.61	5.82	SNX7	Sorting nexin-7	XM_005162844.5
B7G0801	5.60	5.02	RRAS2	Ras-related protein R-Ras2	NM_001017815.1
B7G0300	5.60	4.90	HAUS6	HAUS augmin-like complex subunit 6	NM_001079974.1
B17G0737	5.60	6.14	EXOC5	Exocyst complex component 5	NM_001113796.1
A5G0603	5.60	6.54	GLIPR2	Golgi-associated plant pathogenesis-related protein 1	NM_001423664.1
A22G0135	5.60	2.11	CD84	SLAM family member 5	XM_009295970.4
A18G0148	5.60	8.16	RTASE	Probable RNA-directed DNA polymerase from transposon BS	XM_068220707.1
A15G0398	5.60	6.75	CCDC9	Coiled-coil domain-containing protein 9	NM_001075106.2
A9G0485	5.60	2.58	SH3BP4	SH3 domain-binding protein 4	NM_001123295.1
B16G0793	5.60	0.77	TRPV6	Transient receptor potential cation channel subfamily V member 6	XM_005173468.3
A21G0205	5.60	5.53	PRLR	Prolactin receptor	
B6G1025	5.60	5.26	PPP1R16B	Protein phosphatase 1 regulatory inhibitor subunit 16B	XM_001340056.8
A20G0850	5.60	3.42	NDUFAF4	NADH dehydrogenase [ubiquinone] 1 alpha subcomplex assembly factor 4	NM_001386274.1
A1G0030	5.60	9.65	X-ELEMENT\ORF2	Probable RNA-directed DNA polymerase from transposon X-element	
B21G0501	5.60	4.76	LMBRD2B	G-protein coupled receptor-associated protein LMBRD2B	NM_199944.1
B4G0925	5.60	8.18	ARFGAP3	ADP-ribosylation factor GTPase-activating protein 3	NM_205597.1
A8G0029	5.60	5.29	SHLD3	Shieldin complex subunit 3	
B4G0837	5.60	1.61	DPX16_4609	A0A3N0YC08_ANAGA Uncharacterized protein	
B24G0320	5.60	5.67	LARP4B	La-related protein 4B	XM_021470200.2
B21G0320	5.60	9.20	STIM1	Stromal interaction molecule 1	XM_005161288.5
B19G0020	5.60	1.24	LOC113064817	A0A6P6M6L1_CARAU uncharacterized protein LOC113064817	
B15G0214	5.60	8.22	FOXO1A	Forkhead box protein O1-A	XM_009291427.4
B3G0457	5.60	5.13	COLGALT1	Procollagen galactosyltransferase 1	XM_689125.6
A15G0267	5.59	10.80	PDS5B	Sister chromatid cohesion protein PDS5 homolog B	XM_688861.8
A24G0520	5.59	11.24	OTUD1	OTU domain-containing protein 1	XM_005162584.5
A3G0251	5.59	8.79	XPO6	Exportin-6	NM_194374.1
A18G0223	5.59	2.83	CYBA	Cytochrome b-245 light chain	NM_200579.1
A20G0591	5.59	2.64	RASL11B	Ras-like protein family member 11B	NM_200140.1
B10G0397	5.59	4.67	SLC9A6	hydrogen exchanger 6	NM_001113476.1
B25G0415	5.59	9.57	WNK1	threonine-protein kinase WNK1	XM_009298027.4
A16G0367	5.59	14.63	CNR1	Cannabinoid receptor 1	XM_068214025.1
A23G0056	5.59	4.17	SL9A8	hydrogen exchanger 8	NM_001008586.1
B11G0554	5.59	6.01	PCDH17	Protocadherin-17	NM_001424089.1
B1G0213	5.59	1.84	IL10RB	Interleukin-10 receptor subunit beta	XM_021476362.2

B17G0727	5.59	9.62	COQ6	Ubiquinone biosynthesis monooxygenase COQ6, mitochondrial	NM_001045404.1
A5G1144	5.59	2.14	SAT2	Thialysine N-epsilon-acetyltransferase	
B12G0628	5.59	1.86	NGFR	Tumor necrosis factor receptor superfamily member 16	NM_001198660.1
B19G0213	5.59	9.62	MINDY1	Ubiquitin carboxyl-terminal hydrolase MINDY-1	NM_001100011.1
A15G0345	5.58	6.09	SAMSN1	SAM domain-containing protein SAMSN-1	XM_021481348.2
B6G0041	5.58	15.02	LECG	Galactose-specific lectin nattectin	
B12G0717	5.58	8.02	PRR12	Proline-rich protein 12	XM_005156015.5
B20G0149	5.58	2.82	SYTL3	Synaptotagmin-like protein 3	XM_021468533.2
B10G0807	5.58	10.57	ERAP1	Endoplasmic reticulum aminopeptidase 1	XM_687424.9
A21G0639	5.58	11.32	NCBP3	Nuclear cap-binding protein subunit 3	NM_200143.1
B22G0941	5.58	16.85	PDCD4	Programmed cell death protein 4	NM_198978.1
B17G0302	5.58	4.41	SETD3	Actin-histidine N-methyltransferase	XR_011006883.1
A12G0655	5.58	3.15	ATXN7L3	Ataxin-7-like protein 3	XM_005156094.5
A16G0110	5.58	7.84	PBX2	Pre-B-cell leukemia transcription factor 2	XM_017351360.3
B18G0432	5.58	12.23	MEF2A	Myocyte-specific enhancer factor 2A	XM_005159044.5
A7G1146	5.58	11.12	PLEKHO2	Pleckstrin homology domain-containing family O member 2	XM_002662752.7
B17G0783	5.58	10.42	SMOC1	SPARC-related modular calcium-binding protein 1	XM_068214308.1
A5G0814	5.58	3.76	DBN1	Drebrin	XM_005165437.5
A23G0043	5.58	9.74	AIG1	Androgen-induced gene 1 protein	NM_001017719.2
A2G0041	5.58	8.56	DUSP22B	Dual specificity protein phosphatase 22-B	NM_001017742.2
B18G0729	5.58	17.79	FKBP4	Peptidyl-prolyl cis-trans isomerase FKBP4	XM_005173616.5
B22G0817	5.58	4.75	GLRX2	Glutaredoxin-2, mitochondrial	NM_001002404.2
B5G1135	5.58	3.48	CTDNEP1A	CTD nuclear envelope phosphatase 1A	NM_001007309.1
B2G0447	5.58	6.85	NRDC	Nardilysin	XM_005171326.5
B10G0310	5.58	6.87	GIGYF1	GRB10-interacting GYF protein 1	XM_068223955.1
B25G0443	5.58	6.51	CNOT4	CCR4-NOT transcription complex subunit 4	XM_692417.8
A8G0071	5.57	32.20	PLAT	Tissue-type plasminogen activator	XM_002663051.6
B15G0869	5.57	6.07	CWF19L2	CWF19-like protein 2	NM_001033752.2
B24G0196	5.57	11.56	RIOK3	threonine-protein kinase RIO3	NM_001003614.2
A12G0540	5.57	6.47	PCGF5A	Polycomb group RING finger protein 5-A	NM_001045235.1
B6G0958	5.57	7.95	SYS1	Protein SYS1 homolog	NM_200010.2
A3G0155	5.57	6.80	HOOK2	Protein Hook homolog 2	NM_001423849.1
A15G0006	5.57	10.34	ZGC:66101	threonine-protein kinase SIK3 homolog	NM_200541.1
B5G0410	5.57	2.90	LOC107743817	A0A673M274_9TELE Uncharacterized LOC107743817	NM_001100055.1
B7G1097	5.57	4.48	RADX	RPA-related protein RADX	XM_003198933.6
A12G0460	5.56	6.85	HSD3B	Delta 5-->4-isomerase	XM_021474252.2
B23G0712	5.56	3.64	HOXC13A	Homeobox protein Hox-C13a	NM_131543.1

A1G0535	5.56	3.18	HPGD	15-hydroxyprostaglandin dehydrogenase [NAD(+)]	NM_001326541.1
B16G0728	5.56	13.75	G5714_016171	A0A7J6C7M9_9TELE Uncharacterized protein	
A13G0104	5.56	6.99	ZNF1118	A0A0G2KFJ8_DANRE Zinc finger protein 1118	NM_001076745.2
A5G1020	5.56	2.79	RCC1L	RCC1-like G exchanging factor-like protein	NM_001082803.1
A8G0458	5.56	3.29	PPARD	Peroxisome proliferator-activated receptor delta	NM_131468.2
A21G0228	5.56	14.16	UBE2R2	Ubiquitin-conjugating enzyme E2 R2	NM_001002600.1
B2G0101	5.56	14.07	ANKRD12	Ankyrin repeat domain-containing protein 12	NM_200150.2
B7G0173	5.56	0.77	RASSF10	Ras association domain-containing protein 10	XM_001921630.8
B20G0067	5.56	3.73	SENP6	Sentrin-specific protease 6	XM_021468558.2
B2G0710	5.56	4.33	CDH6	Cadherin-6	XM_005163632.5
A21G0149	5.56	5.21	FPGS	Folylpolyglutamate synthase, mitochondrial	NM_213437.2
A4G0685	5.56	4.56	KLHDC10	Kelch domain-containing protein 10	XM_005164695.5
A18G0288	5.56	8.54	GMPS	GMP synthase [glutamine-hydrolyzing]	NM_200587.2
A24G0433	5.56	3.09	CRY-DASH	Cryptochrome DASH	NM_205686.1
B19G0265	5.56	6.47	ING4	Inhibitor of growth protein 4	NM_001020468.2
A19G0431	5.56	8.68	UBP1	Upstream-binding protein 1	XM_005159726.5
A12G0278	5.56	17.58	ABLIM1	Actin-binding LIM protein 1	XM_068224564.1
B20G0055	5.56	9.77	TC1A	Transposable element Tc1 transposase	
A8G0834	5.56	2.85	POLR3D	DNA-directed RNA polymerase III subunit RPC4	NM_200287.1
A11G0488	5.55	6.21			
B8G0803	5.55	0.27	PLAAT2	Phospholipase A and acyltransferase 2	
B24G0385	5.55	5.67	YTHDF3	YTH domain-containing family protein 3	NM_199870.1
A21G0286	5.55	8.86	DDT-A	D-dopachrome decarboxylase-A	NM_200784.1
B21G0627	5.55	8.61	STOM	Stomatin	XM_017352833.3
A13G0211	5.55	2.77	SYNJ2BP	Synaptojanin-2-binding protein	NM_001145619.1
A20G0223	5.55	6.40	NUS1	Dehydrodolichyl diphosphate synthase complex subunit nus1	NM_001002356.1
A17G0694	5.55	10.09	ARID4A	AT-rich interactive domain-containing protein 4A	XM_005158629.5
B4G0523	5.55	4.40	DCP1B	mRNA-decapping enzyme 1B	XM_021475124.2
A15G0742	5.55	12.99	GET1	Guided entry of tail-anchored proteins factor 1	NM_001003413.1
B18G0846	5.55	10.54	TRPM7	Transient receptor potential cation channel subfamily M member 7	NM_001030061.1
B20G0327	5.54	5.86	PPP1R13B	Apoptosis-stimulating of p53 protein 1	XM_009294640.4
B21G0583	5.54	8.06	TNKS	Poly [ADP-ribose] polymerase tankyrase-1	XM_068219363.1
A7G1007	5.54	6.00	AGFG2	Arf-GAP domain and FG repeat-containing protein 2	NM_001030238.1
B15G0617	5.54	11.71	NLK	threonine-protein kinase NLK	XM_017350980.3
B1G0324	5.54	6.30	ECPAS	Proteasome adapter and scaffold protein ECM29	NM_001365155.1
B18G0180	5.54	8.29	FAM214A	Protein FAM214A	XM_005173655.5
B7G0053	5.54	9.91	TC1A	Transposable element Tc1 transposase	XM_021470094.2

A13G0643	5.54	3.76	NOT2	Homeobox protein not2	XM_005156968.5
A10G0132	5.54	5.96	ZMIZ2	Zinc finger MIZ domain-containing protein 2	XM_068224040.1
A7G0560	5.54	9.91	MAF1	Repressor of RNA polymerase III transcription MAF1 homolog	XM_009303371.4
B19G0237	5.54	4.29	G5714_018888	A0A7J6C4C7_9TELE DUF4806 domain-containing protein	NM_001100099.1
B9G0692	5.54	2.47	LOC107688734	A0A671K497_9TELE Inosine-uridine preferring nucleoside hydrolase-like	
A15G0281	5.54	0.87	FHDC1	FH2 domain-containing protein 1	XM_689466.6
A4G0663	5.54	9.56	SINHCAF	SIN3-HDAC complex-associated factor	NM_001025531.1
A14G0236	5.53	3.34	CHIC1	Cysteine-rich hydrophobic domain-containing protein 1	NM_001201463.1
B5G1177	5.53	3.34	APOOL	MICOS complex subunit MIC27	NM_001014351.1
A15G0509	5.53	4.63	USF1	Upstream stimulatory factor 1	XM_005157509.5
A9G0693	5.53	6.77	GSK3B	Glycogen synthase kinase-3 beta	NM_131381.1
A22G0431	5.53	6.67	KLK7	Kallikrein-7	
A11G0296	5.53	9.36	PRELP	Prolargin	NM_199926.1
B13G0422	5.53	2.19	ABCB10	ATP-binding cassette sub-family B member 10, mitochondrial	
B17G0525	5.53	11.77	KDM1A	Lysine-specific histone demethylase 1A	NM_001242995.1
A19G0387	5.53	6.96	BET1	BET1 homolog	NM_001002046.1
B5G0951	5.53	14.67	TRIM16	Tripartite motif-containing protein 16	NM_001075103.1
A8G0203	5.53	9.20	PLEKHA2	Pleckstrin homology domain-containing family A member 2	NM_001128363.1
B12G0160	5.53	9.37	ST6GALNAC2	Alpha-N-acetylgalactosaminide alpha-2,6-sialyltransferase 2	XM_009306854.4
B3G1001	5.53	6.12	CIITA	MHC class II transactivator	XM_001343036.7
B13G0238	5.53	3.89	NFU1	NFU1 iron-sulfur cluster scaffold homolog, mitochondrial	NM_212992.2
B25G0184	5.52	6.35	KV5A2	Ig kappa chain V-V region MOPC 21	NM_001045408.1
A14G0711	5.52	3.53	CCDC142	Coiled-coil domain-containing protein 142	XM_005157205.5
B3G0651	5.52	13.04	RETREG3	Reticulophagy regulator 3	XM_689279.9
B1G1130	5.52	3.99	LOC113099796	A0A6P6PHG1_CARAU zinc finger protein 271-like	XM_017357165.3
B5G1236	5.52	7.81	SELPLG	P-selectin glycoprotein ligand 1	
A22G0231	5.52	5.09	BAP1	Ubiquitin carboxyl-terminal hydrolase BAP1	XM_005173970.5
A1G0711	5.52	5.67	TFDP1	Transcription factor Dp-1	XM_068214205.1
B20G0699	5.52	5.17	PBK	Lymphokine-activated killer T-cell-originated protein kinase homolog	NM_001002387.1
A19G0289	5.52	4.66	FAM8A1	Protein FAM8A1	XM_003200618.6
B11G0699	5.52	4.07	ARF4	ADP-ribosylation factor 4	NM_001013551.1
A22G0566	5.52	2.03	B3GALT2	Beta-1,3-galactosyltransferase 2	NM_001128796.1
A9G0577	5.52	5.67	SPOPLA	Speckle-type POZ protein-like A	NM_001013447.1
A23G0865	5.52	8.16	TTC39B	Tetratricopeptide repeat protein 39B	XM_068216969.1
A23G0459	5.52	2.90	FAM3A	Protein FAM3A	NM_200800.1
B22G0308	5.52	2.61	G5714_021230	A0A7J6BU98_9TELE Ig-like domain-containing protein	XM_017353433.3
B10G0806	5.52	4.90	OCLN	Occludin	NM_212832.2

B15G0546	5.52	2.64	DPH1	2-(3-amino-3-carboxypropyl)histidine synthase subunit 1	XM_005157581.5
A20G0356	5.51	2.27	GORAB	RAB6-interacting golgin	NM_200385.2
B16G0555	5.51	6.78	GLIPR2	Golgi-associated plant pathogenesis-related protein 1	NM_001005978.1
A25G0816	5.51	8.87	X-ELEMENT\ORF2	Probable RNA-directed DNA polymerase from transposon X-element	
A18G0681	5.51	10.52	C2CD5	C2 domain-containing protein 5	XM_068214739.1
A15G0856	5.51	6.66	PCSK7	kexin type 7	NM_001320428.1
A25G0240	5.51	5.52	LDHD	Probable D-lactate dehydrogenase, mitochondrial	NM_199873.1
B7G1199	5.51	4.23	TEX261	Protein TEX261	NM_001024426.1
B2G0031	5.51	13.32	GIMAP4	GTPase IMAP family member 4	XM_068215016.1
A10G0137	5.51	1.40	MRPS24	28S ribosomal protein S24, mitochondrial	
A24G0065	5.51	3.28			
A23G0225	5.51	8.99	ZMYND8	Protein kinase C-binding protein 1	NM_199641.2
A9G0874	5.51	7.95	GCC2	GRIP and coiled-coil domain-containing protein 2	XM_068223519.1
B20G0231	5.51	2.65	METTL13	eEF1A lysine and N-terminal methyltransferase	NM_001044769.4
A4G0597	5.50	3.76	CBLL1	E3 ubiquitin-protein ligase Hakai	NM_001309369.1
A6G0988	5.50	14.48	ARFRP1	ADP-ribosylation factor-related protein 1	
A24G0161	5.50	5.91	LOC113092779	A0A6P6P063_CARAU uncharacterized protein LOC113092779	
A10G0138	5.50	7.21	GNS	N-acetylglucosamine-6-sulfatase	NM_199841.2
A2G0550	5.50	7.55	RABGGTB	Geranylgeranyl transferase type-2 subunit beta	NM_213112.2
A7G0281	5.50	8.07	CSK22	Casein kinase II subunit alpha'	NM_131240.1
B2G0007	5.50	34.07	MAU2	MAU2 chromatid cohesion factor homolog	XM_068215039.1
B13G0449	5.50	2.13	DUSP13	Dual specificity protein phosphatase 13 isoform B	NM_001003458.1
B11G0444	5.50	6.80	MITF	Microphthalmia-associated transcription factor	NM_201207.1
B17G0451	5.50	2.44	TRMT61B	tRNA (adenine(58)-N(1))-methyltransferase, mitochondrial	NM_001327933.1
A11G0677	5.50	39.37	UBE2J2	Ubiquitin-conjugating enzyme E2 J2	NM_213313.1
B1G0751	5.50	8.79	F8A1	40-kDa huntingtin-associated protein	XM_021473414.2
B20G0631	5.50	1.49	ADGRF5	Adhesion G protein-coupled receptor F5	XM_017352626.3
B14G0290	5.50	4.73	ADRB2	Beta-2 adrenergic receptor	NM_001102652.1
B12G0127	5.50	11.39	ABCA5	Cholesterol transporter ABCA5	NM_001099246.3
A2G1102	5.50	8.94	ABHD4	(Lyso)-N-acylphosphatidylethanolamine lipase	NM_001017613.2
A7G0855	5.50	7.62	IQCJ-SCHIP1	IQCJ-SCHIP1 readthrough transcript protein	XM_001338781.7
B3G1310	5.50	9.12	IGKV6D-41	Probable non-functional immunoglobulin kappa variable 6D-41	
B25G0388	5.50	2.88	UGT2A3	UDP-glucuronosyltransferase 2A3	NM_001177498.2
B25G0252	5.49	4.26	LSP1	Lymphocyte-specific protein 1	XM_003201460.6

B12G0603	5.49	11.57	GET4	Golgi to ER traffic protein 4 homolog	NM_001199907.1
B17G0223	5.49	3.67	BRMS1LA	Breast cancer metastasis-suppressor 1-like protein-A	XM_021467237.2
B2G0796	5.49	2.57	ANO8	Anoctamin-8	XM_002660812.6
A5G0698	5.49	13.18	LTBP3	Latent-transforming growth factor beta-binding protein 3	NM_001257141.1
B21G0808	5.49	5.01	TAF1C	TATA box-binding protein-associated factor RNA polymerase I subunit C	XM_002665873.7
B16G0612	5.49	4.16	NSMCE2	E3 SUMO-protein ligase NSE2	NM_001128718.1
A12G0842	5.49	6.17	MPRIP	Myosin phosphatase Rho-interacting protein	XM_021480197.2
B6G0586	5.49	6.15	MIER1	Mesoderm induction early response protein 1	NM_199559.1
B5G0298	5.49	7.26	ZBTB20	Zinc finger and BTB domain-containing protein 20	NM_001080604.1
B15G0079	5.49	2.61	TGFB1	Transforming growth factor beta-1 proprotein	NM_182873.1
A22G0388	5.49	2.12	RFXANK	DNA-binding protein RFXANK	NM_001130641.1
A11G0307	5.49	9.74	TFEB	Transcription factor EB	NM_001003522.2
B2G1149	5.49	7.88	BCL6	B-cell lymphoma 6 protein homolog	NM_001100074.1
B19G0409	5.49	8.80	DYNC1LI1	Cytoplasmic dynein 1 light intermediate chain 1	NM_001102428.1
B5G1304	5.48	10.46	TAOK3	threonine-protein kinase TAO3	XM_001922630.8
A12G0009	5.48	1.90	PSTK	L-seryl-tRNA(Sec) kinase	
B22G0188	5.48	9.00	MYO9B	Unconventional myosin-IXb	NM_001423631.1
A3G1251	5.48	4.83	ERN1	endoribonuclease IRE1	NM_001423672.1
A22G0730	5.48	2.00	ARHGEF3	Rho guanine nucleotide exchange factor 3	XM_005161867.5
A19G0862	5.48	2.10	ANKRD28	threonine-protein phosphatase 6 regulatory ankyrin repeat subunit A	XM_009301169.4
B8G0024	5.48	1.31	IL17RC	Interleukin-17 receptor C	
A17G0261	5.48	5.24	PALS1A	Protein PALS1	NM_194363.1
A15G0839	5.48	18.20	HEPHL1	Ferroxidase HEPHL1	NM_001328065.1
B23G0847	5.48	2.04	SLAMF8	SLAM family member 8	
A22G0393	5.48	7.42	GATAD2A	Transcriptional repressor p66-alpha	XM_005170444.5
B20G0360	5.47	10.16	SGCB	Beta-sarcoglycan	NM_001034973.3
B9G0742	5.47	4.78	PKNOX1	Homeobox protein PKNOX1	NM_131891.3
A10G0852	5.47	10.42	HMGCS1	Hydroxymethylglutaryl-CoA synthase, cytoplasmic	NM_201085.2
A18G0814	5.47	0.86	BNC1	Zinc finger protein basonuclin-1	XM_009300994.3
B25G0755	5.47	6.14	APPL2	DCC-interacting protein 13-beta	NM_001128075.1
A17G0542	5.47	5.79	ENTPD6	Ectonucleoside triphosphate diphosphohydrolase 6	NM_001017862.1
A9G0547	5.47	6.01	XPO4	Exportin-4	NM_212674.2
B22G1008	5.47	5.28	NLRC3	NLR family CARD domain-containing protein 3	XM_021478497.2
B13G0507	5.47	9.03	LBX1	Transcription factor LBX1	XM_009307382.4
A1G0659	5.47	4.16	POGLUT1	Protein O-glucosyltransferase 1	NM_001037434.2
A13G0395	5.47	20.89	POLR1C	DNA-directed RNA polymerases I and III subunit RPAC1	NM_213252.1
A8G0304	5.47	3.26	POLA2	DNA polymerase alpha subunit B	NM_199581.2
A16G0343	5.47	78.11	STMN1	Stathmin	NM_001017850.1
B4G0234	5.47	6.67	ALDH1L2	Mitochondrial 10-formyltetrahydrofolate dehydrogenase	XM_002661372.7

A23G0554	5.47	8.06	UBE4B	Ubiquitin conjugation factor E4 B	NM_194362.2
A15G0059	5.46	3.80	PARL	Presenilins-associated rhomboid-like protein, mitochondrial	XM_021466506.2
B20G0552	5.46	6.13	SEC63	Translocation protein SEC63 homolog	NM_001002588.1
B18G0004	5.46	15.45	DDB1	DNA damage-binding protein 1	NM_200626.1
B5G1227	5.46	8.43	CMKLR1	Chemerin-like receptor 1	NM_001082812.2
A2G0299	5.46	13.69	BPNT2	Inositol monophosphatase 3	NM_001037568.2
B9G0465	5.46	5.63	EAF2	ELL-associated factor 2	NM_001002162.1
A7G0212	5.46	7.88	CTR9	RNA polymerase-associated protein CTR9 homolog	NM_001083583.1
A24G0420	5.46	4.79	LOC113066283	arginine repetitive matrix protein 2-like isoform X4	XM_005162726.5
A7G0144	5.46	5.76	SEPSECS	O-phosphoseryl-tRNA(Sec) selenium transferase	NM_200154.1
B15G0297	5.46	6.12	FBXO46	F-box only protein 46	NM_001082831.1
B1G0022	5.45	25.11	LAMP1	Lysosome-associated membrane glycoprotein 1	NM_199702.1
A1G0160	5.45	3.81	TECR	Very-long-chain enoyl-CoA reductase	XM_021479665.2
A12G0683	5.45	16.93	TMEM26	Transmembrane protein 26	NM_001114592.1
A11G0653	5.45	4.67	ODAD1	Outer dynein arm-docking complex subunit 1	XM_002663819.6
A2G0133	5.45	7.15	PCYT1A	Choline-phosphate cytidylyltransferase A	XM_021480218.2
B14G0300	5.45	2.94	LSM11	U7 snRNA-associated Sm-like protein LSm11	XM_017359151.3
B2G0892	5.45	5.93	DUSP12	Dual specificity protein phosphatase 12	XM_005163623.5
B8G0478	5.45	4.57	RHOAD	Rho-related GTP-binding protein RhoA-D	NM_001002445.1
B21G0025	5.45	2.18	TRIM16	Tripartite motif-containing protein 16	XM_681071.8
A18G0573	5.45	4.81	TRABD	TraB domain-containing protein	NM_212623.1
B1G0192	5.45	5.54	RNF175	RING finger protein 175	NM_001328343.1
B1G0205	5.45	5.59	CNPY3	Protein canopy homolog 3	NM_001039805.2
B3G1056	5.45	1.48	EPS8L1	Epidermal growth factor receptor kinase substrate 8-like protein 1	NM_001013313.1
B6G0884	5.45	16.26	CHMP4B	Charged multivesicular body protein 4b	NM_200195.1
A12G0478	5.45	5.74	UBALD1	UBA-like domain-containing protein 1	XM_009306668.4
B16G0268	5.44	5.19	ATP2C1	Calcium-transporting ATPase type 2C member 1	XM_003200239.6
B6G0623	5.44	4.73	JADE3	Protein Jade-3	NM_201652.2
A8G0944	5.44	1.26	WDR46	WD repeat-containing protein 46	NM_200623.1
B10G0423	5.44	22.94	EHD1	EH domain-containing protein 1	NM_001004578.1
B12G0236	5.44	9.92	CPN1	Carboxypeptidase N catalytic chain	NM_212770.1
A8G0943	5.44	4.70	NADK	NAD kinase	NM_001017580.1
B22G0392	5.44	4.65	G5714_021433	A0A7J6BSY8_9TELE Uncharacterized protein	
B10G0548	5.44	7.79	POR	NADPH--cytochrome P450 reductase	NM_001034982.1
A18G0349	5.44	3.27	CD81	CD81 antigen	NM_001003735.1
B8G0073	5.44	33.94	SULT1ST3	Cytosolic sulfotransferase 3	NM_183348.2
A3G0696	5.44	10.61	SRRM2	arginine repetitive matrix protein 2	XM_021470715.2

B15G0714	5.44	5.64	MGAT1	Alpha-1,3-mannosyl-glycoprotein 2-beta-N-acetylglucosaminyltransferase	NM_200676.1
A20G0506	5.44	5.59	TRIM25	ISG15 ligase TRIM25	XM_692207.6
A22G0655	5.44	2.85	TERF2IP	Telomeric repeat-binding factor 2-interacting protein 1	
B2G0983	5.44	5.16	SSBP3	Single-stranded DNA-binding protein 3	NM_001130643.1
B15G0330	5.44	7.67	KIAA0100	Protein KIAA0100	
A25G0498	5.44	2.01	SH2D7	SH2 domain-containing protein 7	
B7G1009	5.44	3.41	MRPL46	39S ribosomal protein L46, mitochondrial	NM_001002518.1
B16G0480	5.43	2.07	DGAT1	Diacylglycerol O-acyltransferase 1	NM_001002458.1
A21G0665	5.43	4.21	CNOT6	CCR4-NOT transcription complex subunit 6	NM_212660.2
B12G0206	5.43	5.31	MBTD1	MBT domain-containing protein 1	XM_005169482.5
B5G0706	5.43	4.90	ATXN2	Ataxin-2	XM_009301668.4
B11G0495	5.43	10.24	DHRS3	reductase 3	NM_001002595.2
B19G0580	5.43	6.48	SMAP2	Stromal membrane-associated protein 2	NM_001044795.1
A14G0285	5.43	6.93	PTTG1	Securin	NM_001271474.1
B21G0195	5.43	6.06	SLC25A14	Brain mitochondrial carrier protein 1	NM_200164.2
B9G0552	5.43	4.13	CCNYL1	Cyclin-Y-like protein 1	NM_001076720.1
B13G0023	5.43	10.52	TRIM35	E3 ubiquitin-protein ligase TRIM35	NM_001423274.1
A13G0838	5.43	5.76	HCRTR2	Orexin receptor type 2	XM_005157071.5
B24G0494	5.43	3.51	PREX2	Phosphatidylinositol 3,4,5-trisphosphate-dependent Rac exchanger 2 protein	XM_001923322.6
B3G1142	5.43	10.78	KANSL1	KAT8 regulatory NSL complex subunit 1	XM_068218892.1
B3G1547	5.43	1.01	IFI44	Interferon-induced protein 44	XM_021475951.2
B11G0781	5.43	14.32	FILIP1L	Filamin A-interacting protein 1-like	
A14G0111	5.43	4.05	THOC3	THO complex subunit 3	NM_001003758.1
A10G0364	5.42	7.54	ZDHHC20A	Palmitoyltransferase ZDHHC20-A	XM_005172672.5
A20G0578	5.42	7.83	SLAIN2	SLAIN motif-containing protein 2	XM_009294668.4
B21G0077	5.42	8.67	ZGC:63572	Transmembrane protein 185-like	NM_200437.2
A10G0512	5.42	12.42	SLC46A3	Solute carrier family 46 member 3	XM_679321.9
B16G0778	5.42	6.79	CXCR3	C-X-C chemokine receptor type 3	NM_001089430.2
A19G0720	5.42	6.54	PRCC	Proline-rich protein PRCC	NM_001126440.1
B12G0436	5.42	11.87	CBX7	Chromobox protein homolog 7	XM_009306692.4
A1G0771	5.42	7.94	RTASE	Probable RNA-directed DNA polymerase from transposon BS	XM_068218007.1
B17G0659	5.42	6.04	DNMT3A	DNA (cytosine-5)-methyltransferase 3A	NM_001328167.1
A9G0744	5.42	36.24	SACS	Sacsin	XM_677774.10
A1G0490	5.42	5.72	LRPAP1	Alpha-2-macroglobulin receptor-associated protein	NM_201306.2
A13G0885	5.42	3.05	PPP3R1	Calcineurin subunit B type 1	NM_200631.1
A10G0002	5.42	5.89	CRYBG3	gamma crystallin domain-containing protein 3	

A1G0661	5.42	11.84	A0A673LZZ1	A0A673LZZ1_9TELE SGNH_hydro domain-containing protein	XM_068219418.1
A22G0580	5.42	31.44	POL	Retrovirus-related Pol polyprotein from transposon opus	
A10G0270	5.42	7.64	GIT2	ARF GTPase-activating protein GIT2	NM_001080188.2
A20G0226	5.42	4.70	CEP85L	Centrosomal protein of 85 kDa-like	XM_005170197.5
A10G0793	5.42	13.38	PARM1	Prostate androgen-regulated mucin-like protein 1 homolog	XM_003199311.6
B19G0510	5.42	22.45	CLASP2	CLIP-associating protein 2	NM_001328259.1
B25G0614	5.41	8.65	OLFM4	Olfactomedin-4	XM_068217504.1
B1G0476	5.41	5.50	CBS	Cystathionine beta-synthase	NM_001111232.1
B24G0167	5.41	5.18	GNPTG	N-acetylglucosamine-1-phosphotransferase subunit gamma	NM_001002057.2
B2G0910	5.41	26.95	F3	Tissue factor	NM_001017728.2
A10G0780	5.41	2.73	TJP2	Tight junction protein ZO-2	XM_009305192.4
A8G0585	5.41	5.88	SMARCAD1A	H box 1A	NM_001020774.2
B19G0771	5.41	5.16	UMAD1	UBAP1-MVB12-associated (UMA)-domain containing protein 1	NM_001128373.1
A24G0442	5.41	2.67	SULF1	Extracellular sulfatase Sulf-1	XM_068217038.1
B23G0609	5.41	5.43	ADRM1B	Proteasomal ubiquitin receptor ADRM1	
A23G0749	5.41	2.39	XCR1	Chemokine XC receptor 1	NM_001386469.1
B14G0625	5.41	8.37	CETN1	Centrin-1	XM_002667181.7
B8G0932	5.41	6.99	WDR13	WD repeat-containing protein 13	NM_001023573.1
A5G0339	5.41	2.61	APOOL	MICOS complex subunit MIC27	NM_001014351.1
A16G0241	5.40	5.28	B4GALT3	Beta-1,4-galactosyltransferase 3	XM_021473033.2
A4G0490	5.40	3.71	SCYL2	SCY1-like protein 2	NM_001080799.1
B18G0133	5.40	12.53	SELENOT1B	Thioredoxin reductase-like selenoprotein T1b	NM_178292.5
A4G1002	5.40	11.51	UPF2	Regulator of nonsense transcripts 2	XM_017355122.1
B25G0077	5.40	7.65	SLC5A12	Sodium-coupled monocarboxylate transporter 2	XM_068217337.1
A13G0397	5.40	3.71	INA	Alpha-internexin	NM_001083564.1
B2G0182	5.40	14.10	CDC34	Ubiquitin-conjugating enzyme E2 R1	NM_001002688.1
A25G0675	5.40	8.68	IGLC7	Immunoglobulin lambda constant 7	XM_068217596.1
A8G0295	5.40	2.61	ERCC8	DNA excision repair protein ERCC-8	NM_001005984.1
A19G0815	5.40	7.63	DAXX	Death domain-associated protein 6	XM_005159438.5
A14G0521	5.40	6.74	RUFY1	RUN and FYVE domain-containing protein 1	XM_068213299.1
A3G0495	5.40	6.52	SMURF1	E3 ubiquitin-protein ligase SMURF1	NM_001001943.1
B16G0822	5.40	7.97	LENG9	Leukocyte receptor cluster member 9	XM_005157900.5
A25G0360	5.40	2.74	AEN	Apoptosis-enhancing nuclease	XM_021470676.2
A5G0718	5.40	6.01	RNF214	RING finger protein 214	XM_021476555.2
A16G0238	5.40	5.99	GRAMD1A	Protein Aster-A	XM_068214218.1
A9G0359	5.40	18.24	MORC3	MORC family CW-type zinc finger protein 3	
A10G0860	5.40	6.32	CHD1	Chromodomain-helicase-DNA-binding protein 1	XM_009305101.4

B6G0740	5.40	6.86	EIF4ENIF1	Eukaryotic translation initiation factor 4E transporter	XM_068221718.1
B2G1306	5.40	10.46	A0A673NF18	A0A673NF18_9TELE Uncharacterized protein	
B15G0057	5.39	23.26	A0A673FYH8	A0A673FYH8_9TELE Uncharacterized protein	
A23G0486	5.39	14.31	PTPN12	Tyrosine-protein phosphatase non-receptor type 12	NM_001099420.1
A1G0902	5.39	9.82	GRK4	G protein-coupled receptor kinase 4	NM_001362619.1
B10G0084	5.39	3.84	GALT	Galactose-1-phosphate uridylyltransferase	NM_001328380.1
A25G0149	5.39	17.15	BET1L	BET1-like protein	NM_001003998.2
B3G0927	5.39	3.45	THAP1	THAP domain-containing protein 1	NM_001076669.1
B7G0548	5.39	25.87	KIAA0232	Uncharacterized protein KIAA0232	NM_001328036.1
B22G0654	5.39	5.77	RTCA	RNA 3'-terminal phosphate cyclase	NM_199536.2
B9G0733	5.39	9.14	GSK3B	Glycogen synthase kinase-3 beta	XM_009304494.4
B17G0661	5.39	8.75	ASXL1	Polycomb group protein ASXL1	XM_005158855.5
A20G0350	5.39	9.65	ZNF395	Zinc finger protein 395	NM_001045115.1
B8G0034	5.39	3.56	UBIAD1	UbiA prenyltransferase domain-containing protein 1	NM_001199726.3
A17G0183	5.38	7.93	ZNF106	Zinc finger protein 106	XM_003200378.5
B3G1293	5.38	6.42	NARFL	Cytosolic Fe-S cluster assembly factor narfl	NM_001080991.2
A3G1165	5.38	4.91	MFSD11	UNC93-like protein MFSD11	
A3G0400	5.38	8.76	ICAM2	Intercellular adhesion molecule 2	XM_001921980.8
A11G0038	5.38	7.47	EIF4A3	Eukaryotic initiation factor 4A-III	NM_001045347.1
B14G0533	5.38	6.63	TRAPPC11	Trafficking protein particle complex subunit 11	NM_199626.2
B24G0114	5.38	7.15	SLC6A15	Sodium-dependent neutral amino acid transporter B(0)AT2	XR_011008554.1
B10G0367	5.38	5.75	NECTIN3	Nectin-3	
B25G0779	5.38	7.63			
A8G0253	5.38	114.96	BGN	Biglycan	NM_001002227.2
A2G0421	5.38	7.32	PLA2G4A	Cytosolic phospholipase A2	NM_131295.2
A7G1168	5.38	9.72	IL16	Pro-interleukin-16	XM_003198898.6
A16G0118	5.38	3.02	SLC25A32	carrier	NM_001013336.2
A16G0173	5.38	5.87	SYNDIG1L	Synapse differentiation-inducing gene protein 1-like	
B20G0030	5.37	14.23	EPB41L2	Band 4.1-like protein 2	XM_021468582.2
B1G0985	5.37	12.58	RAB1	Ras-related protein ORAB-1	NM_001326521.1
B23G0084	5.37	5.16	RNF114	E3 ubiquitin-protein ligase RNF114	NM_001001828.4
B5G0248	5.37	5.63	MAPKAPK5	MAP kinase-activated protein kinase 5	NM_001002336.1
B15G0237	5.37	7.91	SGCG	Gamma-sarcoglycan	NM_001003748.2
A23G0900	5.37	7.15	TEC	Tyrosine-protein kinase Tec	XM_003201244.5
B1G0247	5.37	5.83	NOCT	Nocturnin	XM_068217951.1
A8G0065	5.37	4.30	SNAP29	Synaptosomal-associated protein 29	NM_001256256.1
B11G0290	5.37	4.40	LOC113111007	A0A6P6QCC0_CARAU uncharacterized protein LOC113111007	XM_005172888.5
B1G0445	5.37	6.78	TRMO	tRNA (adenine(37)-N6)-methyltransferase	NM_001327757.1
A7G1015	5.37	16.12	TMEM179B	Transmembrane protein 179B	NM_001020489.2
B1G0070	5.37	3.49	CD48	CD48 antigen	

B15G0846	5.36	35.40	ACTN4	Alpha-actinin-4	XM_021481339.2
B9G0511	5.36	4.12	NABP1	SOSS complex subunit B2	XM_005167556.5
B5G0581	5.36	11.33	MAMDC2	MAM domain-containing protein 2	NM_001130397.1
A12G0210	5.36	6.65	LLGL2	LLGL scribble cell polarity complex component 2	NM_212582.1
A20G0833	5.36	7.24	PUM2	Pumilio homolog 2	NM_001102570.2
A4G0367	5.36	0.16	CD276	CD276 antigen homolog	
B19G0178	5.36	12.36	MAP7D1	MAP7 domain-containing protein 1	XM_005173746.2
A19G0484	5.36	5.38	PTDSS1	Phosphatidylserine synthase 1	XM_682780.10
A9G0409	5.36	2.22	LOC113053220	Leu-3	
A19G0049	5.36	9.43	NKIRAS1	NF-kappa-B inhibitor-interacting Ras-like protein 1	
A4G0909	5.36	10.45	LOC107656420	A0A671TAE7_9TELE Uncharacterized LOC107656420	NM_001025503.1
A8G0404	5.36	7.26	SI:CH211-147A11.3	UPF0688 protein C1orf174 homolog	NM_001044837.1
A18G0086	5.36	6.09	CSTF3	Cleavage stimulation factor subunit 3	NM_213053.2
A19G0544	5.36	0.56	HOXA9A	Homeobox protein Hox-A9a	NM_131532.2
B17G0501	5.36	5.10	JADE1	Protein Jade-1	XM_005158491.5
A12G0337	5.36	7.74	MSL1	Male-specific lethal 1 homolog	XM_686625.10
B2G1087	5.36	3.25	DCUN1D1	DCN1-like protein 1	NM_199772.1
B16G0023	5.36	2.62	TRIM16	Tripartite motif-containing protein 16	XM_690458.9
B17G0590	5.36	7.42	ZNF839	Zinc finger protein 839	NM_001423822.1
B1G1043	5.36	2.87	NXPE4	NXPE family member 4	XM_068217146.1
B13G0050	5.36	5.81	NF7O	Nuclear factor 7, ovary	XM_017358708.3
B20G0405	5.35	4.95	CHST14	Carbohydrate sulfotransferase 14	NM_212829.1
B23G0695	5.35	8.55	CMTR1	Cap-specific mRNA (nucleoside-2'-O-)-methyltransferase 1	NM_200133.1
A10G0707	5.35	5.37	DHX29	ATP-dependent RNA helicase DHX29	XM_692841.10
B2G0868	5.35	6.80	AMOTL2A	Angiomotin-like 2a	NM_001077606.2
B4G0088	5.35	8.96	OSBPL8	Oxysterol-binding protein-related protein 8	XM_021475194.2
A24G0276	5.35	1.33	A0A673JX81	A0A673JX81_9TELE SCY domain-containing protein	
A16G0741	5.35	5.06	FOXJ2	Forkhead box protein J2	XM_681709.9
B15G0527	5.35	2.71	ROHU_009800	A0A498LYC5_LABRO Solute carrier family 35 member F2-like protein	
B20G0536	5.35	4.58	SLC22A16	Solute carrier family 22 member 16	NM_001025488.1
B18G0396	5.35	2.94	KTI12	Protein KTI12 homolog	NM_001126418.1
B22G0753	5.35	7.39	UQCR11	Cytochrome b-c1 complex subunit 10	
A14G0555	5.35	4.70	TGFB2	Transforming growth factor beta-2 proprotein	XM_683088.6
A21G0518	5.35	8.09	CLDN3	Claudin-3	
A2G0539	5.35	10.11	PLD1	Phospholipase D1	NM_001160095.1
B3G1002	5.35	13.97	CLEC16A	Protein CLEC16A	XM_005163916.5
B23G0614	5.35	0.98	TBX18	T-box transcription factor TBX18	NM_153665.2
A16G0563	5.35	3.09	IRGC	Interferon-inducible GTPase 5	
A5G0289	5.35	8.02	SELPLG	P-selectin glycoprotein ligand 1	
A17G0294	5.35	5.52	KLF11	Krueppel-like factor 11	NM_001077604.2
A9G0230	5.34	3.33	PDIA5	Protein disulfide-isomerase A5	NM_001113576.1

A14G0770	5.34	6.42	LOC113113565	A0A6P6QQC1_CARAU uncharacterized protein LOC113113565	XM_001344090.8
A4G0904	5.34	20.94	ATP6V1E1	V-type proton ATPase subunit E 1	NM_173254.1
A10G0104	5.34	9.76	PLK2	threonine-protein kinase PLK2	XM_003199277.6
B24G0560	5.34	6.54	TERF1	Telomeric repeat-binding factor 1	XM_690237.7
A7G0137	5.34	5.69	METTL4	N(6)-adenine-specific methyltransferase METTL4	XM_684086.9
B4G0299	5.34	4.77	SEMA3AA	Semaphorin-3aa	NM_131060.1
A20G0824	5.34	2.95	CCNK	Cyclin-K	NM_001163779.1
B5G0670	5.34	4.04	SMN1	Survival motor neuron protein 1	NM_131191.1
B23G0439	5.34	7.04	RERE	Arginine-glutamic acid dipeptide repeats protein	XM_005162204.5
A19G0890	5.34	5.93	PPP1R18	Phostensin	XM_693482.9
A5G0254	5.33	6.62	GOLGA3	Golgin subfamily A member 3	XM_005165081.5
A1G0989	5.33	3.49	FAM114A1	Protein Noxp20	NM_001089478.1
B20G0229	5.33	5.26	VAMP4	Vesicle-associated membrane protein 4	NM_200735.3
B7G0089	5.33	3.68	SLC35C1	GDP-fucose transporter 1	XM_005169053.5
B20G0844	5.33	2.74	TRAM2	Translocating chain-associated membrane protein 2	XM_005173812.4
B8G1064	5.33	3.41	PYM1B	Partner of Y14 and mago B	NM_200594.2
B7G0427	5.33	13.61	TSG101	Tumor susceptibility gene 101 protein	XM_002662878.6
A5G1134	5.33	7.47	ZMYND19	Zinc finger MYND domain-containing protein 19	NM_183069.1
B25G0346	5.33	2.27	COMMD4	COMM domain-containing protein 4	NM_001320175.1
A21G0396	5.33	13.76	TGFB1	Transforming growth factor beta-1 proprotein	XM_687246.9
A18G0051	5.33	16.70	ETS1	Protein C-ets-1	NM_001017558.1
A1G0706	5.33	22.78	LAMP1	Lysosome-associated membrane glycoprotein 1	NM_199702.1
B7G0958	5.32	2.69	MTHFS	5-formyltetrahydrofolate cyclo-ligase	NM_001386414.1
B6G0652	5.32	12.91	GPR84	G-protein coupled receptor 84	NM_001105697.2
B16G0225	5.32	4.19	SLC25A40	Probable mitochondrial glutathione transporter SLC25A40	NM_001002360.1
A7G0667	5.32	16.28	MADD	MAP kinase-activating death domain protein	XM_068218439.1
A5G0562	5.32	8.00	SH3GLB2	Endophilin-B2	XM_021476044.2
B10G0810	5.32	15.03	AEBP1	Adipocyte enhancer-binding protein 1	NM_001324409.1
B15G0816	5.32	4.27	NLRP3	NACHT, LRR and PYD domains-containing protein 3	XM_021470830.2
B10G0769	5.32	7.33	PITPNM2	Membrane-associated phosphatidylinositol transfer protein 2	XM_017354413.3
B8G0292	5.32	6.47	GLT1D1	Glycosyltransferase 1 domain-containing protein 1	XR_011016673.1
A7G0380	5.32	10.02	RTASE	Probable RNA-directed DNA polymerase from transposon BS	XM_068220136.1
A2G0384	5.32	3.78	ARHGEF4	Rho guanine nucleotide exchange factor 4	XM_009298440.4
B3G0662	5.32	3.58	NDE1	Nuclear distribution protein nudE homolog 1	NM_001030203.1
A5G0679	5.32	3.52	KPTN	KICSTOR complex protein kaptin	NM_001003642.2
A16G0703	5.32	3.29	ZNF574	Zinc finger protein 574	XM_689335.10

A15G0663	5.32	9.63	LOC107699179	A0A671KZ96_9TELE Uncharacterized LOC107699179	NM_001077625.1
B3G1017	5.32	3.12	NUDT9	ADP-ribose pyrophosphatase, mitochondrial	NM_213352.2
B22G0766	5.31	2.61	PEAK1	Inactive tyrosine-protein kinase PEAK1	
B24G0202	5.31	11.99	GNAL	Guanine nucleotide-binding protein G(olf) subunit alpha	NM_001366715.1
A12G0254	5.31	4.47	TCF7L2	Transcription factor 7-like 2	XM_068224506.1
B2G1147	5.31	9.37	NLRC3	NLR family CARD domain-containing protein 3	XM_009295700.4
B4G0040	5.31	6.47	DUSP16	Dual specificity protein phosphatase 16	NM_213240.2
B9G0861	5.31	2.42	PPP1R9A	Neurabin-1	XM_009304433.4
A1G0731	5.31	3.53	ADAMTS1	A disintegrin and metalloproteinase with thrombospondin motifs 1	XM_021475923.2
B2G0586	5.31	4.86	EIF2B3	Translation initiation factor eIF-2B subunit gamma	NM_201074.2
A24G0457	5.31	3.27	EZH2	Histone-lysine N-methyltransferase EZH2	NM_001077279.1
B14G0364	5.31	6.58	ITGA6	Integrin alpha-6	XM_021481280.2
A6G0035	5.31	9.58	TAB1	TGF-beta-activated kinase 1 and MAP3K7-binding protein 1	XM_002662240.7
B7G1041	5.31	0.96	G5714_008509	A0A7J6CVV9_9TELE Ig-like domain-containing protein	
B9G0463	5.31	5.41	ARL5A	ADP-ribosylation factor-like protein 5A	NM_001002339.1
A6G0247	5.31	7.63	WDSUB1	WD repeat, SAM and U-box domain-containing protein 1	NM_001077769.1
B21G0460	5.31	6.37	DDX10	Probable ATP-dependent RNA helicase DDX10	XM_001922185.7
A19G0186	5.31	4.37	BLOC1S4	Biogenesis of lysosome-related organelles complex 1 subunit 4	NM_001003475.1
B7G0590	5.31	4.97	NUDT19	Acyl-coenzyme A diphosphatase NUDT19	NM_001128786.1
B4G0324	5.31	2.81	IL17RA	Interleukin-17 receptor A	NM_001100003.1
A8G0730	5.31	6.72	LOC113070747	A0A6P6MUD5_CARAU uncharacterized protein LOC113070747	
A18G0196	5.30	2.15	CMC2	COX assembly mitochondrial protein 2 homolog	
B14G0677	5.30	1.21	TMEM109	A0A673K3H8_9TELE Transmembrane protein 109	
B18G0725	5.30	2.71	LMF2	Lipase maturation factor 2	NM_001128764.1
A16G0913	5.30	4.09	SNRK	threonine-protein kinase	NM_001193327.2
A11G0256	5.30	0.74	CISH	Cytokine-inducible SH2-containing protein	XM_021474227.2
A23G0445	5.30	9.45	KANSL2	KAT8 regulatory NSL complex subunit 2	XM_068216740.1
B15G0369	5.30	14.02	POU2AF1	POU domain class 2-associating factor 1	XM_009291580.4
A3G0244	5.30	9.39	CREBBP	Histone lysine acetyltransferase CREBBP	NM_001365186.1
B24G0004	5.30	5.19	HUS1	Checkpoint protein HUS1	NM_001089496.1
A2G0468	5.30	8.87	AFG3L2	AFG3-like protein 2	NM_001111197.1
A17G0514	5.30	1.93	EDARADD	Ectodysplasin-A receptor-associated adapter protein	XM_690046.9
B21G0743	5.30	0.82	GRP	Gastrin-releasing peptide	

A17G0904	5.30	9.08	PPP2R5C	threonine-protein phosphatase 2A 56 kDa regulatory subunit gamma isoform	XM_001344835.9
A18G0498	5.30	4.31	NCOA5	Nuclear receptor coactivator 5	NM_001045069.1
B15G0529	5.30	13.50	PAFAH1B1A	Lissencephaly-1 homolog A	XM_005157575.5
B24G0219	5.30	5.71	AGAP3	Arf-GAP with GTPase, ANK repeat and PH domain-containing protein 3	XM_009297585.4
B24G0243	5.30	2.31	RDH12	Retinol dehydrogenase 12	NM_001009912.2
B15G0664	5.30	20.90	HMGB1	High mobility group protein B1	NM_001099251.2
B4G0123	5.30	11.27	CREB3L2	Cyclic AMP-responsive element-binding protein 3-like protein 2	NM_001080651.2
A7G1197	5.29	3.17	AEP1	Aerolysin-like protein	NM_001013304.3
A16G0562	5.29	2.57	IRGC	Interferon-inducible GTPase 5	XM_068213839.1
A15G0279	5.29	78.90	C4	Complement C4	XM_005157372.5
B25G0511	5.29	4.38	PIGY	Phosphatidylinositol N-acetylglucosaminyltransferase subunit Y	
A18G0569	5.29	7.31	CRY1	Cryptochrome-1	NM_131790.5
B25G0508	5.29	6.73	BTBD10	POZ domain-containing protein 10	XR_223121.5
A13G0136	5.29	5.60	CYP1B1	Cytochrome P450 1B1	XM_002664096.6
B24G0216	5.29	9.51	PCMTD1	Protein-L-isoaspartate O-methyltransferase domain-containing protein 1	NM_001030067.3
B6G0218	5.29	4.11	NUDT15	Nucleotide triphosphate diphosphatase NUDT15	
B7G1229	5.29	3.06	DUSP19	Dual specificity protein phosphatase 19	
A9G0716	5.29	6.04	DNAJC15	DnaJ homolog subfamily C member 15	NM_001003476.2
A5G1343	5.29	4.36	CLIP1	CAP-Gly domain-containing linker protein 1	XM_009301992.4
A2G0466	5.29	3.37	MPLKIP	M-phase-specific PLK1-interacting protein	NM_001202486.1
A5G1299	5.29	5.95	USP39	U6.U5 tri-snRNP-associated protein 2	NM_001080070.1
B18G0771	5.29	0.57	NWD2	NACHT and WD repeat domain-containing protein 2	XM_017351983.3
A19G0496	5.28	8.87	MED30	Mediator of RNA polymerase II transcription subunit 30	NM_200717.1
A20G0808	5.28	3.52	ARV1	Protein ARV1	NM_001113611.1
B6G0708	5.28	4.35	DDI2	Protein DDI1 homolog 2	XM_021476799.2
B3G0092	5.28	11.08	TCB1	Transposable element Tcb1 transposase	XM_021471168.2
B22G0632	5.28	23.90	LPL	Lipoprotein lipase	NM_131127.1
A8G0383	5.28	3.88	UCK2A	Uridine-cytidine kinase 2-A	NM_001171067.1
B7G0383	5.28	12.89	PHKA1	Phosphorylase b kinase regulatory subunit alpha, skeletal muscle isoform	XM_017357095.3
A21G0211	5.28	1.30	RTKN	Rhotekin	NM_001328566.1
A17G0031	5.28	1.91	JDP2	Jun dimerization protein 2	XM_005158934.5
B1G0029	5.28	4.56	CENPE	Centromere-associated protein E	NM_001327759.1
B5G1233	5.28	10.19	SART3	Squamous cell carcinoma antigen recognized by T-cells 3	XM_068221218.1
B24G0064	5.28	2.45	IGKV1D-8	Immunoglobulin kappa variable 1D-8	NM_001045408.1
B3G1318	5.28	2.86	TBL3	Transducin beta-like protein 3	NM_001007402.1
A24G0051	5.28	16.53	IFI44	Interferon-induced protein 44	XM_009306148.4
A20G0281	5.28	5.59	G5714_019870	A0A7J6BY51_9TELE Uncharacterized protein	NM_001326469.1

A23G0251	5.28	2.37	RBBP8NL	RBBP8 N-terminal-like protein	XM_005174119.5
A7G0733	5.27	6.09	RNF111-C	E3 ubiquitin-protein ligase arkadia-C	XM_005168924.5
B7G0508	5.27	9.42	NFX1	Transcriptional repressor NF-X1	XM_685467.10
B15G0494	5.27	3.35	SAE1	SUMO-activating enzyme subunit 1	NM_001002058.1
B25G0265	5.27	12.54	FBXO31	F-box only protein 31	XM_021473864.2
A5G0936	5.27	5.43	NOTCH1	Neurogenic locus notch homolog protein 1	XM_005168649.5
B24G0648	5.27	22.84	CAVIN1	Caveolae-associated protein 1	NM_001114549.2
A3G0167	5.27	17.16	SAMD9	Sterile alpha motif domain-containing protein 9	XM_068218626.1
A24G0219	5.27	29.78	VIM	Vimentin	NM_131872.2
B21G0821	5.27	8.59	SEC16A	Protein transport protein Sec16A	XM_009292712.4
B2G0595	5.27	7.97	KLF4	Krueppel-like factor 4	NM_131723.1
A9G0756	5.27	10.44	A0A673HWZ2	A0A673HWZ2_9TELE Si:ch73-250a16.5	
A6G0305	5.27	4.99	NCK2	Cytoplasmic protein NCK2	NM_001003492.2
B3G1189	5.26	2.21	SP140	Nuclear body protein SP140	
A24G0213	5.26	4.78	YME1L1	ATP-dependent zinc metalloprotease YME1L1	XM_005162861.5
A23G0694	5.26	4.89	LIMA1	LIM domain and actin-binding protein 1	NM_131664.1
A24G0504	5.26	2.72	PCK1	Phosphoenolpyruvate carboxykinase, cytosolic [GTP]	NM_213192.1
A19G0803	5.26	10.99	GSK3B	Glycogen synthase kinase-3 beta	NM_001044921.3
B3G0944	5.26	6.88	MED25	Mediator of RNA polymerase II transcription subunit 25	XM_005163938.5
B19G0239	5.26	5.11	EFNA3	Ephrin-A3	XM_001340876.7
B7G0289	5.26	8.63	MRC1	Macrophage mannose receptor 1	
B11G0416	5.26	0.61	RNPEP	Aminopeptidase B	NM_001024949.1
B25G0063	5.26	3.69	SNX20	Sorting nexin-20	XM_009298207.4
A1G0465	5.26	4.47	SLC43A1	Large neutral amino acids transporter small subunit 3	NM_001083000.1
B25G0798	5.26	7.37	I6LAK0	I6LAK0_CYPCA Prion-like protein 1	NM_001013298.1
B15G0097	5.25	5.24	RNF168	E3 ubiquitin-protein ligase rnf168	NM_199855.1
B23G0744	5.25	6.54	CPSF3L	Integrator complex subunit 11	NM_001020621.1
B10G0179	5.25	4.10	TBC1D10A	TBC1 domain family member 10A	XM_686009.6
B24G0441	5.25	8.13	POLR1D	Protein POLR1D, isoform 2	NM_001386556.1
B13G0392	5.25	2.41	RUFY2	RUN and FYVE domain-containing protein 2	XM_009307409.4
B24G0637	5.25	4.77	BLOC1S5	Biogenesis of lysosome-related organelles complex 1 subunit 5	NM_001030066.2
A20G0469	5.25	2.83	DCAF4	DDB1- and CUL4-associated factor 4	XM_021468379.2
B10G0156	5.25	1.56	MRRF	Ribosome-recycling factor, mitochondrial	NM_001002174.1
A14G0188	5.25	12.26	UBL3	Ubiquitin-like protein 3	NM_212856.1
B18G0636	5.25	12.91	TAT	Tyrosine aminotransferase	NM_001077554.2
A18G0576	5.25	5.04	MTSS2	Protein MTSS 2	XM_009293500.4
B17G0494	5.25	6.80	TTC7B	Tetratricopeptide repeat protein 7B	XM_009293100.4
A1G1127	5.25	1.31	CORO2A	Coronin-2A	XM_005159876.5
A6G0546	5.25	2.25	LEPR	Leptin receptor	NM_001309403.1
B19G0357	5.25	18.90	CNDP2	Cytosolic non-specific dipeptidase	NM_214704.2
B13G0575	5.25	5.91	UBTD1	Ubiquitin domain-containing protein 1	NM_001142592.1

A6G0304	5.25	2.11	DPH3	Diphthamide biosynthesis protein 3	NM_001159824.2
A15G0626	5.25	8.61	NECTIN1	Nectin-1	XM_001337576.6
A2G0911	5.25	7.51	SSBP3	Single-stranded DNA-binding protein 3	NM_001362582.1
B21G0642	5.24	7.13	TNFRSF22	Tumor necrosis factor receptor superfamily member 22	
A2G0098	5.24	3.62	JMJD4	2-oxoglutarate and iron-dependent oxygenase JMJD4	NM_001076628.1
A1G0360	5.24	8.40	PKNOX1	Homeobox protein PKNOX1	XM_068221700.1
A25G0318	5.24	11.18			
A8G0668	5.24	2.80			NM_001007032.1
A8G0968	5.24	6.28	DCAF1	DDB1- and CUL4-associated factor 1	XM_002663268.7
A10G0200	5.24	6.78	ESS2	Splicing factor ESS-2 homolog	NM_001089330.2
A17G0290	5.24	1.75	EVA1A	Protein eva-1 homolog A	NM_001076587.3
B3G0625	5.24	6.57	PRSS36	Polyserase-2	NM_001003526.2
A9G0809	5.24	13.33	DYNC1I2A	Dynein, cytoplasmic 1, intermediate chain 2a	XM_017357723.3
A1G0978	5.24	7.27	UFSP2	Ufm1-specific protease 2	XM_005168337.5
B21G0878	5.24	2.71	CKS2	Cyclin-dependent kinases regulatory subunit 2	NM_001002110.1
B12G0530	5.24	149.66	ACTA2	Actin, aortic smooth muscle	NM_212620.2
A9G0565	5.24	3.38			
A23G0308	5.24	1.68	EMP3	Epithelial membrane protein 3	NM_001025508.1
A2G0528	5.24	2.46	HLTF	Helicase-like transcription factor	XM_005163376.5
B11G0641	5.24	3.98	SLC41A1	Solute carrier family 41 member 1	XM_021479909.2
A7G0340	5.24	12.15	SLC2A4	Solute carrier family 2, facilitated glucose transporter member 4	
A25G0327	5.24	7.79	ARNTL	Aryl hydrocarbon receptor nuclear translocator-like protein 1	XM_005163022.5
B18G0566	5.24	7.88	SWAP70	Switch-associated protein 70	NM_001044986.1
B4G0081	5.24	11.25	GLIPR1L1	GLIPR1-like protein 1	
A15G0449	5.24	8.19	APPBP2	Amyloid protein-binding protein 2	NM_212633.1
B10G0253	5.24	8.24	DOK3	Docking protein 3	XM_021472246.2
A9G0730	5.24	14.43	MYCBP2	E3 ubiquitin-protein ligase MYCBP2	XM_009304563.4
A8G0178	5.23	29.96	PIM2	threonine-protein kinase pim-2	NM_131539.2
A18G0750	5.23	3.28	NEDD4	E3 ubiquitin-protein ligase NEDD4	XM_068214864.1
B10G0799	5.23	1.96	HIC2	Hypermethylated in cancer 2 protein	NM_182869.2
B20G0922	5.23	30.66	HSP90A.1	Heat shock protein HSP 90-alpha 1	NM_131328.2
B5G0764	5.23	11.83	SLC12A9	Solute carrier family 12 member 9	NM_001128548.1
A5G0900	5.23	4.72	LNPEP	Leucyl-cystinyl aminopeptidase	NM_199621.1
A21G0253	5.23	4.94	PNPLA7	Patatin-like phospholipase domain-containing protein 7	XM_687184.9
A22G0255	5.23	1.64	SGSH	N-sulphoglucosamine sulphohydrolase	
B8G0359	5.23	6.87	EP400	E1A-binding protein p400	XM_068223193.1
B24G0599	5.23	3.48	ANKHB	Progressive ankylosis protein homolog B	NM_001030259.2
B3G0150	5.23	2.56	DPX16_1011	A0A3N0XR67_ANAGA Zinc finger protein 845	XM_068220167.1
B17G0042	5.23	8.45	SIVA1	Apoptosis regulatory protein Siva	

A14G0449	5.23	7.74	CE024	UPF0461 protein C5orf24 homolog	NM_001386379.1
A10G0865	5.22	5.47	SH2D4A	SH2 domain-containing protein 4A	XM_005156459.5
A9G0539	5.22	5.67	ZMYM2	Zinc finger MYM-type protein 2	XM_068223591.1
A3G0243	5.22	23.41	CREBBP	CREB-binding protein	XM_021472888.2
B7G0167	5.22	8.95	CTR9	RNA polymerase-associated protein CTR9 homolog	NM_001083583.1
B23G0566	5.22	14.44	HUWE1	E3 ubiquitin-protein ligase HUWE1	NM_001365654.1
B21G0094	5.22	2.61	SRA1	Steroid receptor RNA activator 1	NM_001002047.1
B24G0173	5.22	5.84	COG7	Conserved oligomeric Golgi complex subunit 7	NM_001077393.1
A17G0721	5.22	6.42	BAZ1A	Bromodomain adjacent to zinc finger domain protein 1A	NM_001365144.1
B23G0480	5.22	10.17	SRGAP3	SLIT-ROBO Rho GTPase-activating protein 3	XM_005162155.5
A22G0235	5.22	5.24	TUSC2	Tumor suppressor candidate 2	NM_001002402.2
A15G0430	5.22	1.11	AIPL1	Aryl-hydrocarbon-interacting protein-like 1	XM_021466431.2
A13G0102	5.22	3.27	ZBTB8OS	Protein archease	XM_017358922.3
B3G0576	5.22	8.62	TTYH3	Protein tweety homolog 3	XM_679082.9
B3G1544	5.21	3.61	NUGGC	Nuclear GTPase SLIP-GC	XM_068215007.1
B6G0339	5.21	16.32	NOMO2	Nodal modulator 2	XM_002662315.4
A22G0713	5.21	7.60	HPS3	BLOC-2 complex member HPS3	NM_001193542.1
B9G0574	5.21	3.45	SPRYD7	SPRY domain-containing protein 7	NM_200526.1
A4G0957	5.21	12.90	BCAP29	B-cell receptor-associated protein 29	NM_001024220.1
B19G0056	5.21	2.74	TWIST2	Twist-related protein 2	NM_130984.2
B19G0749	5.21	3.13	NEU1	Sialidase-1	NM_001044909.2
B14G0129	5.21	27.00	AHNAK	Neuroblast differentiation-associated protein AHNAK	XM_005173182.5
B6G0493	5.21	13.47	AMOTL2A	Angiomotin-like 2a	NM_001080177.2
A7G0965	5.21	3.15	UFSP1	Ufm1-specific protease 1	NM_001089530.1
A3G1069	5.21	9.53	KPNA2	Importin subunit alpha-1	NM_001002335.1
B10G0670	5.21	8.13	RAB11FIP1	Rab11 family-interacting protein 1	XM_005164192.5
A2G0242	5.20	5.29	EGFR	Epidermal growth factor receptor	NM_194424.1
A7G0508	5.20	8.53	CKLF	Chemokine-like factor	XM_002662874.6
A25G0278	5.20	3.10	PPFIBP2	Liprin-beta-2	NM_001326598.1
B14G0014	5.20	9.05	HS3ST1	Heparan sulfate glucosamine 3-O-sulfotransferase 1	XM_005173051.5
B12G0578	5.20	9.06	STAT5B	Signal transducer and activator of transcription 5B	NM_001003984.2
B21G0782	5.20	7.72	EGFL7	Epidermal growth factor-like protein 7	NM_001040385.1
A6G0759	5.20	9.27	TEX264	Testis-expressed protein 264	NM_205661.1
B7G0111	5.20	3.61	IRE-1	endoribonuclease ire-1	XM_021478061.2
A24G0336	5.20	8.85	PDK3	[Pyruvate dehydrogenase (acetyl-transferring)] kinase isozyme 3, mitochondrial	XM_683459.9
B20G0489	5.20	3.29	KATNBL1	KATNB1-like protein 1	NM_001006019.2
A22G0727	5.20	2.38	NCBP2	Nuclear cap-binding protein subunit 2	NM_173249.1
B11G0022	5.20	6.56	IARS1	Isoleucine--tRNA ligase, cytoplasmic	NM_001002705.1
B24G0087	5.20	48.28	COX6B1	Cytochrome c oxidase subunit 6B1	NM_001002695.3
A17G0858	5.20	6.43	ZFYVE19	NoCut checkpoint regulator	

B1G0488	5.20	3.30	CD28	T-cell-specific surface glycoprotein CD28 homolog	XM_001923447.7
A22G0377	5.20	74.16	GPX4	Phospholipid hydroperoxide glutathione peroxidase	
B22G0007	5.20	12.44	SPEN	Msx2-interacting protein	XM_005170347.5
A18G0787	5.19	4.99	TM2D3	TM2 domain-containing protein 3	NM_001099264.2
B14G0243	5.19	4.79	STARD10	START domain-containing protein 10	NM_200220.1
A8G0022	5.19	9.93	PPIC	Peptidyl-prolyl cis-trans isomerase C	XM_002667830.7
A10G0557	5.19	5.16	LTN1	E3 ubiquitin-protein ligase listerin	XM_001340364.8
A3G0396	5.19	3.85	P2RY11	P2Y purinoceptor 11	NM_001204454.1
A21G0064	5.19	7.91	HMGCR	3-hydroxy-3-methylglutaryl-coenzyme A reductase	NM_001014292.3
A10G0539	5.19	9.41	BCAM	Basal cell adhesion molecule	XM_068223964.1
A21G0861	5.19	18.35	UBE2B	Ubiquitin-conjugating enzyme E2 B	NM_001002747.1
A8G0577	5.19	8.04	LOC113107522	A0A6P6PXP8_CARAU uncharacterized protein LOC113107522	
A22G0009	5.19	10.20	SLC25A20	acylcarnitine carrier protein	NM_200859.1
A5G0125	5.19	11.29	PAIP1	Polyadenylate-binding protein-interacting protein 1	NM_001003504.1
B19G0147	5.18	9.41	ZFP646	Zinc finger protein 646	XM_009293995.4
B16G0741	5.18	9.18	KIF21A	Kinesin-like protein KIF21A	NM_001045031.1
A23G0746	5.18	1.92	GPSM2	G-protein-signaling modulator 2	XM_021469788.2
A23G0563	5.18	16.36	MXRA8	Matrix remodeling-associated protein 8	NM_001079960.3
A10G0202	5.18	0.00			NM_001002656.2
B22G0832	5.18	7.34	LPAR3	Lysophosphatidic acid receptor 3	NM_001285534.1
A15G0243	5.18	8.54	SH3BP5L	SH3 domain-binding protein 5-like	NM_213160.1
B6G0008	5.18	7.13	LDLR	Low-density lipoprotein receptor	XM_017356726.2
A5G1304	5.18	1.36	GANAB	Neutral alpha-glucosidase AB	NM_001327941.1
B16G0278	5.18	22.68	CMTM6	CKLF-like MARVEL transmembrane domain-containing protein 6	
A18G0605	5.18	14.08	PARP12	Protein mono-ADP-ribosyltransferase PARP12	NM_001082852.1
A3G0811	5.18	14.68	LEG	Beta-galactoside-binding lectin	
B21G0396	5.18	4.71	PPP1R14B	Protein phosphatase 1 regulatory subunit 14B	NM_001045853.1
B3G0354	5.18	16.04	SLC44A2	Choline transporter-like protein 2	NM_198871.1
B17G0845	5.18	4.36	PRKD3	threonine-protein kinase D3	XM_680391.9
A14G0033	5.18	3.61	SPRY1	Protein sprouty homolog 1	NM_001122600.1
A1G0698	5.17	108.04	CREG1	Protein CREG1	
B23G0550	5.17	5.71	ACVR1B	Activin receptor type-1B	NM_130990.1
B3G0960	5.17	6.30	BCAT1	Branched-chain-amino-acid aminotransferase, cytosolic	NM_001309527.1
B23G0871	5.17	5.13	RPRD1B	Regulation of nuclear pre-mRNA domain-containing protein 1B	XM_005162423.2
B25G0678	5.17	3.01	EHF	ETS homologous factor	NM_001039822.1
A14G0040	5.17	5.92	RNF19B	E3 ubiquitin-protein ligase RNF19B	
B15G0808	5.17	2.50	USP16	Ubiquitin carboxyl-terminal hydrolase 16	XM_009291915.4

B22G0216	5.17	3.06	LOC113040394	A0A6P6J2X5_CARAU uncharacterized protein LOC113040394	NM_001113586.2
B25G0209	5.17	0.19	IGLC7	Immunoglobulin lambda constant 7	XM_068217596.1
A20G0712	5.17	5.77	VAMP4	Vesicle-associated membrane protein 4	NM_001433724.1
B5G0898	5.17	10.09	CRAT	Carnitine O-acetyltransferase	NM_001005587.1
A5G0300	5.17	11.62	PIK3IP1	Phosphoinositide-3-kinase-interacting protein 1	NM_198374.2
B14G0380	5.17	4.36	GRHPR	hydroxypyruvate reductase	NM_001020525.1
A17G0304	5.17	2.04	DTD2	D-aminoacyl-tRNA deacylase 2	NM_001004011.3
A11G0439	5.17	6.02	RPL22	60S ribosomal protein L22	NM_001080015.2
A8G0844	5.17	5.23	KCTD9	POZ domain-containing protein KCTD9	NM_001013567.3
B25G0617	5.17	10.48	CCL14	C-C motif chemokine 14	NM_001129894.1
B19G0832	5.17	5.48	CPSF1	Cleavage and polyadenylation specificity factor subunit 1	XM_005159584.5
B10G0109	5.17	13.33	ZFAND5	AN1-type zinc finger protein 5	NM_200949.1
A15G0825	5.17	12.69	CUL3	Cullin-3	NM_001190485.1
B2G0081	5.17	17.53	RAB8A	Ras-related protein Rab-8A	NM_001089562.2
B15G0814	5.17	3.29	SLC25A36A	Solute carrier family 25 member 36-A	XR_011006362.1
A8G0793	5.17	3.36	STAR	Steroidogenic acute regulatory protein, mitochondrial	NM_131663.1
B15G0581	5.16	6.65	SSH2	Protein phosphatase Slingshot homolog 2	XM_005157671.5
A2G0811	5.16	6.33	REEP6	Receptor expression-enhancing protein 6	NM_001013536.2
A8G0759	5.16	0.57	LOC113070729	A0A6P6MTL6_CARAU uncharacterized protein LOC113070729 isoform X1	NM_001080076.2
A19G0835	5.16	2.84	ZDHHC3	Palmitoyltransferase ZDHHC3	NM_001077757.1
A24G0068	5.16	0.55	PTP10D	Tyrosine-protein phosphatase 10D	
B6G0161	5.16	12.76	MYH9	Myosin-9	NM_001098177.2
A3G0267	5.16	0.02	YTX2	Transposon TX1 uncharacterized 149 kDa protein	XM_068222685.1
A10G0733	5.16	3.18	PAQR3	Progestin and adipoQ receptor family member 3	XR_011016939.1
B15G0830	5.16	6.16	SCAF4	SR-related and CTD-associated factor 4	NM_001386228.1
B5G0635	5.16	8.17	RASA1	Ras GTPase-activating protein 1	XM_009301766.4
B18G0770	5.16	9.94	FCHSD2	F-BAR and double SH3 domains protein 2	XM_005169913.5
B24G0486	5.16	4.62	MYD88	Myeloid differentiation primary response protein MyD88	
A14G0548	5.16	6.72	TMCO6	Transmembrane and coiled-coil domain-containing protein 6	
A15G0008	5.16	0.93	CEP164	Centrosomal protein of 164 kDa	
B9G0303	5.16	4.21	CD247	T-cell surface glycoprotein CD3 zeta chain	NM_001100107.1
A1G1135	5.15	9.83	PSPC1	Paraspeckle component 1	NM_001045258.1
A1G0737	5.15	12.01	ROHU_027471	A0A498M800_LABRO Uncharacterized protein	XM_068220980.1
B8G1037	5.15	3.56	RNF165	E3 ubiquitin-protein ligase RNF165	XM_005166679.4
B25G0741	5.15	11.92	IREB2	Iron-responsive element-binding protein 2	XM_005162972.4
B20G0191	5.15	1.90	CLMN	Calmin	NM_001003605.2

B1G0128	5.15	11.47	LOC113073303	A0A6P6N0Y7_CARAU uncharacterized protein LOC113073303 isoform X2	
B2G0215	5.15	59.03	MBNL1	Muscleblind-like protein 1	NM_001076634.1
B2G0253	5.15	0.85	EEF1AKMT4	EEF1A lysine methyltransferase 4	XM_009298793.4
B14G0790	5.15	8.22	RNF12-A	E3 ubiquitin-protein ligase RNF12-A	XM_009307767.4
A4G0008	5.15	5.30	KDM5A	Lysine-specific demethylase 5A	XM_017354458.3
A1G0760	5.15	2.32	RAP2A	Ras-related protein Rap-2a	NM_001145705.1
B18G0139	5.15	3.98	ZBTB38	Zinc finger and BTB domain-containing protein 38	XM_009293816.4
A10G0846	5.15	3.35	ST8SIA4	CMP-N-acetylneuraminate-poly-alpha-2,8-sialyltransferase	NM_001099416.1
A3G0666	5.15	6.49	ABI1	Abl interactor 1	NM_001076774.2
A4G0575	5.15	3.76	PPP6R2	threonine-protein phosphatase 6 regulatory subunit 2	NM_001025509.1
A12G0397	5.15	9.07	RAB18B	Ras-related protein Rab-18-B	NM_206830.1
A23G0186	5.15	12.33	SLA2	Src-like-adapter 2	NM_001114435.1
B3G1429	5.15	5.77	LOC107568598	A0A672NK94_SINGR Protein FAM104A-like	NM_001386581.1
B18G0007	5.15	8.12	ST7	Suppressor of tumorigenicity 7 protein homolog	NM_001040336.1
B11G0154	5.15	20.80	CSE1L	Exportin-2	XM_009305864.4
B18G0493	5.15	5.55	TLE3A	Transducin-like enhancer protein 3-A	NM_131012.2
A7G0490	5.14	1.00	PLEKHF2	Pleckstrin homology domain-containing family F member 2	
B22G0055	5.14	39.40	NLRC3	Protein NLRC3	XM_068216562.1
B3G1180	5.14	25.24	CBX7	Chromobox protein homolog 7	NM_001017853.2
A7G0527	5.14	19.66	VPS35	Vacuolar protein sorting-associated protein 35	NM_001025517.2
A7G0452	5.14	4.34	MFHAS1	Malignant fibrous histiocytoma-amplified sequence 1	
A16G0672	5.14	7.73	IL6RA	Interleukin-6 receptor subunit alpha	NM_001330258.2
B4G0019	5.14	6.03	SI:CH211-152C2.3	F1RAX9_DANRE Si:ch211-152c2.3 (Fragment)	
B24G0635	5.14	3.44	SLC35B3	Adenosine 3'-phospho 5'-phosphosulfate transporter 2	NM_001039995.1
B15G0600	5.14	4.43	RAP1GAP2	Rap1 GTPase-activating protein 2	XM_017350967.3
B8G0845	5.14	3.90	MED27	Mediator of RNA polymerase II transcription subunit 27	NM_200660.1
A9G0451	5.14	10.36	STK17B	threonine-protein kinase 17B	NM_200535.1
A16G0721	5.14	3.88	LOC113059964	A0A6P6LLW7_CARAU cell division cycle-associated protein 3-like	NM_001045049.1
A3G0673	5.13	5.31	CASP6	Caspase-6	NM_001020497.1
B8G0120	5.13	5.65	AGTPBP1	Cytosolic carboxypeptidase 1	NM_001024445.2
A20G0271	5.13	5.61			
A3G0058	5.13	4.29	GIMAP9	GTPase IMAP family member 9	NM_001045009.3
A2G0834	5.13	11.75	MYADM	Myeloid-associated differentiation marker homolog	XM_685301.9
A14G0007	5.13	4.21	CYTL1	Cytokine-like protein 1	XM_021481075.2
B17G0213	5.13	9.94	AHCTF1	Protein ELYS	XM_009292870.4
B1G0822	5.13	29.65	HERC3	Probable E3 ubiquitin-protein ligase HERC3	XM_021475845.2
A5G0632	5.13	2.28	GFM2	Ribosome-releasing factor 2, mitochondrial	NM_001077545.1
A21G0447	5.13	0.19	ROHU_020477	A0A498N5R9_LABRO Gig2 b	NM_001245989.1

A15G0007	5.13	9.79	SIDT2	SID1 transmembrane family member 2	XM_068213804.1
B5G0811	5.13	43.49	HIS3.3A	Histone H3.3A	NM_200003.1
A24G0500	5.13	4.65	UBE2WB	Probable ubiquitin-conjugating enzyme E2 W-B	NM_001045293.2
B20G0562	5.13	0.98	DDX1	ATP-dependent RNA helicase DDX1	NM_001197324.1
B1G0438	5.13	5.40	INTS12	Integrator complex subunit 12	NM_001002161.3
B8G0947	5.13	7.10	DPYSL2	Dihydropyrimidinase-related protein 2	XM_003199046.6
B11G0138	5.13	2.53	CDA	Cytidine deaminase	NM_001423552.1
B12G0439	5.13	3.94	L3MBTL2	Lethal(3)malignant brain tumor-like protein 2	NM_200032.1
B5G0878	5.12	8.28	USP20	Ubiquitin carboxyl-terminal hydrolase 20	XM_009301458.4
B2G0725	5.12	3.54	TOE1	Target of EGR1 protein 1	NM_001256682.1
A10G0155	5.12	6.94	TPST2	Protein-tyrosine sulfotransferase 2	NM_200419.1
B16G0072	5.12	2.35	CEP72	Centrosomal protein of 72 kDa	NM_001319132.1
A12G0523	5.12	5.61	PAPSS2	Bifunctional 3'-phosphoadenosine 5'-phosphosulfate synthase 2	NM_212562.1
B23G0672	5.12	9.06	TFCP2	Transcription factor CP2	XM_017353731.3
B1G0819	5.12	9.13			
A21G0673	5.12	5.29	NDST1	N-sulfotransferase 1	NM_001324455.1
B21G0822	5.12	4.52	LOC113038974	A0A6P6J251_CARAU uncharacterized protein LOC113038974 isoform X1	XM_694356.10
B25G0734	5.12	4.44	SNX33	Sorting nexin-33	NM_001122855.2
B19G0701	5.12	8.96	PPP1R10	threonine-protein phosphatase 1 regulatory subunit 10	NM_001277109.1
B6G0778	5.12	11.22	RBM6	RNA-binding protein 6	XM_068222041.1
B23G0481	5.12	0.99	AVPR2	Vasopressin V2 receptor	XM_001345969.8
B12G0831	5.12	1.46	ITGAX	Integrin alpha-X	XM_068224852.1
A11G0364	5.12	3.36	SNAI1	Zinc finger protein SNAI1	NM_001199491.1
A8G0072	5.11	4.39	IKBKB	Inhibitor of nuclear factor kappa-B kinase subunit beta	NM_001123265.1
A7G0176	5.11	3.27	CTDNEP1A	CTD nuclear envelope phosphatase 1A	NM_001007440.2
B19G0590	5.11	7.87	SMIM12	Small integral membrane protein 12	
A3G0663	5.11	6.86	GTPBP1	GTP-binding protein 1	XM_005163999.5
B14G0772	5.11	1.92	A33	Zinc-binding protein A33	NM_001044961.1
A14G0113	5.11	3.04	CPLX2	Complexin-2	NM_001199318.1
B16G0303	5.11	2.77	CENPW	Centromere protein W	NM_001326437.1
B24G0698	5.11	5.75	NCK1	Cytoplasmic protein NCK1	XM_068217135.1
A20G0931	5.11	10.60	TAB2	TGF-beta-activated kinase 1 and MAP3K7-binding protein 2	XM_017352472.3
B5G0960	5.11	2.81	DPAGT1	UDP-N-acetylglucosamine--dolichyl-phosphate N-acetylglucosaminephosphotransferase	NM_001089411.1
A18G0743	5.11	1.07	PIGBOS1	A0A6P6RBK2_CARAU protein PIGBOS1	NM_001308439.1
B8G0255	5.11	14.69	INPP5A	Inositol polyphosphate-5-phosphatase A	NM_001037569.2
B25G0590	5.11	14.15	TMEM263-B	Transmembrane protein 263-B	NM_001005996.2
B4G0762	5.11	3.26	G5714_004447	A0A7J6D5J8_9TELE Uncharacterized protein	XM_068215354.1
B13G0683	5.11	0.51	RTASE	Probable RNA-directed DNA polymerase from transposon BS	
B19G0187	5.10	9.57	HIVEP1	Zinc finger protein 40	XM_021468250.2

A3G0861	5.10	7.47	YPEL3	Protein yippee-like 3	NM_212790.1
A1G0616	5.10	3.97	DIS3	Exosome complex exonuclease RRP44	XM_001336814.9
B25G0442	5.10	1.39	LOC107753568	A0A673G310_9TELE Pseudouridine-metabolizing bifunctional protein C1861.05-like	NM_001040302.1
A7G0626	5.10	6.69	NOD2	Nucleotide-binding oligomerization domain-containing protein 2	NM_001328044.1
B10G0392	5.10	4.17	ARFIP2	Arfaptin-2	XM_005155373.5
A5G0994	5.10	18.44	ABR	Active breakpoint cluster region-related protein	NM_001100016.1
B2G1175	5.10	5.36	PRKAG2	5'-AMP-activated protein kinase subunit gamma-2	XM_691638.9
B1G1103	5.10	17.41	SAMHD1	Deoxynucleoside triphosphate triphosphohydrolase SAMHD1	XM_068220882.1
B1G0406	5.10	6.11	FBXW7	WD repeat-containing protein 7	XM_021473906.2
A11G0766	5.10	4.97	SRSF6	arginine-rich splicing factor 6	NM_001017575.1
A10G0843	5.10	16.44	SYK	Tyrosine-protein kinase SYK	XM_005155538.5
A22G0485	5.10	13.05	AP3D1	AP-3 complex subunit delta-1	XM_009296268.4
B25G0417	5.10	7.12	PRR5	Proline-rich protein 5	XM_005163058.5
B5G1143	5.10	3.88	GBGT1	Globoside alpha-1,3-N-acetylgalactosaminyltransferase 1	NM_001123269.1
A2G1031	5.10	8.24	CUL3B	Cullin-3-B	NM_199691.2
B2G1066	5.10	2.42	PIGK	GPI-anchor transamidase	NM_001002149.1
A19G0440	5.10	10.25	PUM1	Pumilio homolog 1	NM_001277119.2
B12G0151	5.09	1.50	OXLD1	Oxidoreductase-like domain-containing protein 1	
B9G0280	5.09	8.25	SON	Protein SON	NM_001130400.1
A14G0817	5.09	5.01	GPR174	Probable G-protein coupled receptor 174	NM_001089527.1
A6G0693	5.09	2.80	VHL	von Hippel-Lindau disease tumor suppressor	NM_001080684.1
B25G0128	5.09	0.00	IGKV1-6	Immunoglobulin kappa variable 1-6	NM_001045408.1
B10G0793	5.09	6.22	BRAP	BRCA1-associated protein	XM_021479154.2
A15G0003	5.09	1.54	MPZL3	Myelin protein zero-like protein 3	
A15G0331	5.09	3.75	AKAP1	A-kinase anchor protein 1, mitochondrial	NM_001098179.2
A20G0549	5.08	4.60	HOOK1	Protein Hook homolog 1	NM_001045198.1
A5G0514	5.08	2.54	DDX31	Probable ATP-dependent RNA helicase DDX31	XM_021476489.2
B8G0364	5.08	4.45	STX2	Syntaxin-2	XM_005167257.5
B3G1118	5.08	4.10	MIEN1	Migration and invasion enhancer 1	NM_194418.3
A4G0373	5.08	2.94	LOC107573893	A0A672MGK6_SINGR Gastrula zinc finger protein XlCGF57.1-like	XM_021475327.2
A5G0551	5.08	17.06	UBE2G1	Ubiquitin-conjugating enzyme E2 G1	NM_199863.1
A6G0051	5.08	2.50	ORC4	Origin recognition complex subunit 4	XM_005168697.3
A10G0125	5.08	5.59	CLIP1	CAP-Gly domain-containing linker protein 1	XM_021479478.2
B23G0602	5.08	3.58	ASXL1	Polycomb group protein ASXL1	NM_001386454.1
B21G0762	5.08	13.32	NEK6	threonine-protein kinase Nek6	XM_005161495.5
A15G0613	5.08	4.92	TADA2A	Transcriptional adapter 2-alpha	XM_005157436.5
B7G0178	5.08	10.14	USP47	Ubiquitin carboxyl-terminal hydrolase 47	XM_021477371.2

B25G0736	5.08	5.91	PTPN9	Tyrosine-protein phosphatase non-receptor type 9	XM_005162973.5
B17G0006	5.07	16.31	DYNC1H1	Cytoplasmic dynein 1 heavy chain 1	NM_001042745.1
B19G0738	5.07	3.12	DDX39B	Spliceosome RNA helicase DDX39B	NM_200943.3
A15G0633	5.07	3.54	SARS2	Serine--tRNA ligase, mitochondrial	NM_201179.2
A21G0273	5.07	10.00	PHPT1	14 kDa phosphohistidine phosphatase	NM_001003566.2
B10G0324	5.07	21.42	RNASEKB	Ribonuclease kappa-B	NM_001045375.1
B7G0709	5.07	4.09	CILP	Cartilage intermediate layer protein 1	NM_001199362.1
A7G0263	5.07	5.53	TRIP4	Activating signal cointegrator 1	NM_001077575.2
A23G0414	5.07	6.06	SAMD11	Sterile alpha motif domain-containing protein 11	XM_009296907.4
A16G0656	5.07	8.36	SHC1	SHC-transforming protein 1	XM_005169755.5
A17G0322	5.07	2.51	RPUSD2	Pseudouridylate synthase RPUSD2	NM_001327917.1
B2G0573	5.07	0.36	TNN	Tenascin-N	XM_005171264.5
A5G0431	5.07	3.63	CI085	Uncharacterized protein C9orf85 homolog	NM_001004001.2
B13G0039	5.07	3.50	IRF2	Interferon regulatory factor 2	XM_003199759.6
A24G0260	5.07	12.63	NRROS	Transforming growth factor beta activator LRRC33	NM_001003775.2
A25G0770	5.07	4.95	NUP160	Nuclear pore complex protein Nup160	NM_001423844.1
A22G0488	5.07	4.54	CHAF1A	Chromatin assembly factor 1 subunit A	NM_001045013.2
A8G0782	5.06	9.79	RFK	Riboflavin kinase	NM_001328083.1
A12G0660	5.06	4.70	TUT1	Speckle targeted PIP5K1A-regulated poly(A) polymerase	NM_001030188.1
B22G0701	5.06	13.84	SBNO2	Protein strawberry notch homolog 2	NM_001423267.1
A16G0504	5.06	3.73	LOC107689883	A0A671LSX4_9TELE Probable assembly chaperone of rpl4	XM_017351506.3
B7G0445	5.06	1.97	SLC25A44	Solute carrier family 25 member 44	NM_001013519.1
A8G0727	5.06	4.78	GOLGA1	Golgin subfamily A member 1	NM_200838.2
A5G0119	5.06	10.83	LMBRD2B	G-protein coupled receptor-associated protein LMBRD2B	NM_001327914.1
A8G0923	5.06	12.86	DPP9	Dipeptidyl peptidase 9	NM_001077313.2
B19G0819	5.06	3.97	PEX11B	Peroxisomal membrane protein 11B	NM_001045854.3
A5G0535	5.06	7.09	H2AX	Histone H2AX	NM_201073.1
A4G0466	5.06	3.96	TPRKB	KEOPS complex subunit TPRKB	XM_068219608.1
A6G0407	5.06	2.87	SUZ12B	Polycomb protein suz12-B	NM_001082824.2
A13G0140	5.06	7.41	IPMK	Inositol polyphosphate multikinase	XM_005156511.5
B3G0006	5.06	9.17	TCF20	Transcription factor 20	XM_005171536.5
A25G0025	5.06	1.90	WNT7B	Protein Wnt-7b	XM_001920184.8
B21G0995	5.06	8.06	TRIM25	ISG15 ligase TRIM25	XM_681071.8
A18G0471	5.06	3.24	MAP2K1	Dual specificity mitogen-activated protein kinase kinase 1	NM_213419.2
A24G0084	5.05	28.18	ATP6V0C	V-type proton ATPase 16 kDa proteolipid subunit c	NM_205554.1
B6G0565	5.05	9.24	CYLD	Ubiquitin carboxyl-terminal hydrolase CYLD	XM_003198803.5
B21G0273	5.05	4.95	SSH2	Protein phosphatase Slingshot homolog 2	NM_001135103.1

B12G0025	5.05	6.97	HHEX	Hematopoietically-expressed homeobox protein hhex	NM_130934.1
B21G0129	5.05	13.60	CREBRF	CREB3 regulatory factor	XM_005170286.5
B1G0699	5.05	9.39	G5714_000560	A0A7J6DGW9_9TELE Uncharacterized protein	XM_021476923.2
A5G0924	5.05	4.69	COQ2	4-hydroxybenzoate polyprenyltransferase, mitochondrial	NM_001089486.1
B13G0042	5.05	21.77	RTASE	Probable RNA-directed DNA polymerase from transposon BS	NM_001040376.2
A4G0540	5.05	7.06	ACSS3	Acyl-CoA synthetase short-chain family member 3, mitochondrial	XM_689284.10
A2G0695	5.05	6.78	EIF2B3	Translation initiation factor eIF-2B subunit gamma	NM_201074.2
B1G1172	5.05	7.68	MVB12A	Multivesicular body subunit 12A	NM_200484.1
A25G0810	5.05	2.36	LIPC	Hepatic triacylglycerol lipase	XM_021470555.2
A8G0372	5.05	8.46	CSNK1G2	Casein kinase I isoform gamma-2	NM_001045850.2
A7G0317	5.05	5.04	ASPH	asparaginyl beta-hydroxylase	XM_021478003.2
A4G0755	5.05	4.17	BRAF	threonine-protein kinase B-raf	NM_001324516.1
A13G0588	5.04	3.53	NAT8L	N-acetylaspartate synthetase	NM_001020472.1
A15G0537	5.04	6.19	DYRK1A	Dual specificity tyrosine-phosphorylation-regulated kinase 1A	NM_001347831.1
B1G0302	5.04	3.14	FAM114A1	Protein Noxp20	XM_009307313.4
B7G0788	5.04	6.22	ADGRG1	Adhesion G-protein coupled receptor G1	NM_001020481.1
A20G0919	5.04	7.09	ZNF292	Zinc finger protein 292	NM_001030212.1
B22G0510	5.04	7.62	STAB2	Stabilin-2	NM_001195614.2
B22G0048	5.04	66.46	NLRC3	NLR family CARD domain-containing protein 3	XM_068216562.1
B4G0812	5.04	1.79	LOC113066133	A0A6P6MAN3_CARAU gastrula zinc finger protein XlCGF57.1-like	NM_001424595.1
A8G0180	5.04	33.96	BCAP31	B-cell receptor-associated protein 31	NM_200092.1
B15G0559	5.04	4.03	PPM1D	Protein phosphatase 1D	NM_201090.2
B12G0532	5.04	9.84	CH25H	Cholesterol 25-hydroxylase-like protein	NM_001008652.1
A14G0122	5.04	3.78	CCDC69	Coiled-coil domain-containing protein 69	NM_001423999.1
A23G0268	5.04	4.24	CDC42	Cell division control protein 42 homolog	NM_001039923.1
B11G0098	5.04	7.23	SAMD13	Sterile alpha motif domain-containing protein 13	XM_021479657.2
B3G1029	5.04	9.21	ACLY	ATP-citrate synthase	NM_001002649.2
A12G0535	5.04	5.64	PANK1	Pantothenate kinase 1	NM_001083841.1
B9G0785	5.04	1.42	CD58	Lymphocyte function-associated antigen 3	
A20G0088	5.04	2.17			
B14G0276	5.04	3.51	MED7	Mediator of RNA polymerase II transcription subunit 7	NM_199741.1
B5G0948	5.04	7.82	RUVBL2	RuvB-like 2	NM_174860.2
B13G0453	5.04	1.40	VCL	Vinculin	NM_001017872.1
B3G0199	5.03	15.35	G5714_003947	A0A7J6DAV0_9TELE Uncharacterized protein	
B16G1100	5.03	5.38	PREB	Prolactin regulatory element-binding protein	NM_001002638.1
A17G0757	5.03	4.52	RMND1	Required for meiotic nuclear division protein 1 homolog	NM_001020772.1

B19G0954	5.03	10.43	RTASE	Probable RNA-directed DNA polymerase from transposon BS	XM_068222002.1
A20G0082	5.03	19.82	THBD	Thrombomodulin	
B13G0266	5.03	7.47	MAVS	Mitochondrial antiviral-signaling protein	NM_001346164.1
B3G0394	5.03	9.35	HGS	Hepatocyte growth factor-regulated tyrosine kinase substrate	XM_068215940.1
B20G0541	5.03	11.17	RAB32	Ras-related protein Rab-32	NM_201332.1
B5G1303	5.03	5.70	SUDS3	Sin3 histone deacetylase corepressor complex component SDS3	NM_001003493.2
A3G0889	5.03	14.19	GYS1	Glycogen [starch] synthase, muscle	NM_201180.2
B9G0632	5.03	4.60	AAD-A	Alpha-aspartyl dipeptidase	NM_001144055.1
B1G0176	5.03	3.33	MICALL2	MICAL-like protein 2	NM_001312897.1
B8G0416	5.03	6.49	DERL2	Derlin-2	NM_200903.2
B7G0821	5.03	11.27	PLSCR1	Phospholipid scramblase 1	NM_212866.1
B9G0559	5.03	7.72	PTPN4	Tyrosine-protein phosphatase non-receptor type 4	XM_068223323.1
A5G0249	5.03	5.92	AACS	Acetoacetyl-CoA synthetase	XM_005165076.5
A2G0478	5.02	5.25	BMI1B	Polycomb complex protein BMI-1-B	XM_021479461.2
A4G0760	5.02	3.03	ABTB3A	POZ domain-containing protein 3-A	NM_001327842.1
B3G0003	5.02	29.96	MGP	Matrix Gla protein	
A22G0558	5.02	4.61	WDR83	WD repeat domain-containing protein 83	NM_001002429.1
A4G0631	5.02	10.37	DRAM1	DNA damage-regulated autophagy modulator protein 1	NM_001006049.1
B21G0390	5.02	14.18	MARK2	threonine-protein kinase MARK2	XM_009295510.4
A6G0736	5.02	7.22	SRSF3	arginine-rich splicing factor 3	NM_001002053.2
A5G0886	5.02	3.60	UBAC1	Ubiquitin-associated domain-containing protein 1	NM_001110283.2
A8G0287	5.02	8.13	EPS15	Epidermal growth factor receptor substrate 15	XM_002663099.7
A3G0885	5.02	8.16	ATP6V0A1	V-type proton ATPase 116 kDa subunit a 1	XM_005163798.4
A17G0164	5.02	4.99	AP5M1	AP-5 complex subunit mu-1	XM_005158894.4
A2G0892	5.02	5.51	SLC25A36A	Solute carrier family 25 member 36-A	NM_001002667.1
A18G0353	5.02	9.66	PIK3C2A	Phosphatidylinositol 4-phosphate 3-kinase C2 domain-containing subunit alpha	XM_068214908.1
A9G0355	5.02	3.13	TBC1D4	TBC1 domain family member 4	XM_068223650.1
B20G0112	5.02	10.26	CYP46A1	Cholesterol 24-hydroxylase	NM_200479.2
A15G0426	5.02	6.98	RFLNB	Refilin-B	NM_001386285.1
B19G0841	5.02	10.46	NCALDB	Neurocalcin-delta B	NM_001003776.1
A1G0215	5.02	3.73	DCAF15	DDB1- and CUL4-associated factor 15	XM_677708.10
A4G0489	5.01	6.09	PARP11	Protein mono-ADP-ribosyltransferase PARP11	NM_001082810.1
B20G0165	5.01	8.55	YLPM1	YLP motif-containing protein 1	XM_003200679.6
A20G0688	5.01	1.38	TRIM16	Tripartite motif-containing protein 16	XM_693013.9
A1G0320	5.01	6.73	NPNT	Nephronectin	XM_005160098.5
A2G0582	5.01	1.28	RNF152	E3 ubiquitin-protein ligase rnf152	NM_001014358.2
A4G1048	5.01	8.19	PTPN12	Tyrosine-protein phosphatase non-receptor type 12	NM_200669.1

A5G1118	5.01	4.79	LIFR	Leukemia inhibitory factor receptor	XM_068221529.1
B5G0829	5.01	3.68	RHOG	Rho-related GTP-binding protein RhoG	NM_199692.2
B12G0009	5.01	10.71	PARG	Poly(ADP-ribose) glycohydrolase	
A6G0869	5.01	6.48	GNAS	Guanine nucleotide-binding protein G(s) subunit alpha	XM_001335696.7
A12G0038	5.01	7.95	CYP4F3	Cytochrome P450 4F3	NM_001089541.2
A8G0824	5.01	3.67	STAMBPA	STAM-binding protein-like A	XM_068223022.1
A6G0002	5.01	5.00	MBD5	Methyl-CpG-binding domain protein 5	XM_068221854.1
B10G0377	5.01	9.91	BACH1	Transcription regulator protein BACH1	NM_001384147.1
B21G0427	5.01	4.99	METTL27	Methyltransferase-like protein 27	
A9G0315	5.01	3.15	MX	Interferon-induced GTP-binding protein Mx	XM_005167664.5
A22G0217	5.01	6.35	CCDC71	Coiled-coil domain-containing protein 71	NM_001044710.1
A21G0016	5.01	2.87	TBC1D13	TBC1 domain family member 13	NM_001114422.1
B24G0007	5.01	3.11	PPP4R1	threonine-protein phosphatase 4 regulatory subunit 1	NM_200260.1
A25G0321	5.01	7.81	VAC14	Protein VAC14 homolog	XM_009297900.4
A12G0739	5.01	16.32	CD79B	B-cell antigen receptor complex-associated protein beta chain	
B11G0082	5.00	9.84	GDF11	differentiation factor 11	NM_001001823.2
A24G0390	5.00	13.66	FLT3	Receptor-type tyrosine-protein kinase FLT3	XM_001921725.6
B13G0400	5.00	2.77	KIFBP	KIF-binding protein	NM_001039988.1
B5G0863	5.00	4.10	GFM2	Ribosome-releasing factor 2, mitochondrial	NM_001077545.1
A14G0182	5.00	16.83	FOSL1	Fos-related antigen 1	XM_009291125.4
A4G0310	5.00	2.04	G5714_004461	A0A7J6D4R8_9TELE Uncharacterized protein	XM_068213652.1
A10G0067	5.00	5.04	MYO3B	Myosin-IIIb	XM_005172588.5
B7G1024	5.00	2.57	IMMP1L	Mitochondrial inner membrane protease subunit 1	NM_001386390.1
B9G0886	5.00	3.93	IL22RA2	Interleukin-22 receptor subunit alpha-2	XM_005167960.5
A10G0297	5.00	3.28	SAMSN1	SAM domain-containing protein SAMSN-1	XM_005169164.5
A5G0870	5.00	5.04	FAM172A	Cotranscriptional regulator FAM172A homolog	NM_001431106.1
A10G0136	5.00	4.78	NUDCD3	NudC domain-containing protein 3	
A7G0774	5.00	4.99	EXOSC6	Exosome complex component MTR3	NM_001020486.1
B14G0783	5.00	11.22	ATRX	Transcriptional regulator ATRX	NM_200653.2
A13G0720	5.00	36.49	LBH	Protein LBH	
A17G0656	5.00	5.78	JKAMP	MAPK8-associated membrane protein	NM_200482.2
A21G0165	5.00	4.05	AGGF1	Angiogenic factor with G patch and FHA domains 1	NM_001079982.1
B9G0456	5.00	7.81	ITGB5	Integrin beta-5	NM_001082836.1
B8G0221	5.00	2.35	BAG4	BAG family molecular chaperone regulator 4	NM_001256729.2
B1G0898	5.00	15.28	ABCA1	Phospholipid-transporting ATPase ABCA1	NM_001309465.1

B9G0880	5.00	5.17	GALNT5	Polypeptide N-acetylgalactosaminyltransferase 5	XM_068223539.1
B3G1006	5.00	6.41	RAB40C	Ras-related protein Rab-40C	NM_001040348.1
B22G0733	5.00	2.21	GZMG	Granzyme G	XM_021473474.2
A21G0254	5.00	2.05	CLIC4	Chloride intracellular channel protein 4	NM_199524.1
B9G0798	5.00	2.62	YBEY	Endoribonuclease YbeY	NM_212594.2
B8G0433	5.00	1.57	AOPEP	Aminopeptidase O	NM_001123258.1
A7G1208	4.99	13.64	INO80B	INO80 complex subunit B	NM_001077258.1
A6G1019	4.99	5.16	TSC22D1	TSC22 domain family protein 1	NM_200574.4
A5G0537	4.99	4.16	DPAGT1	UDP-N-acetylglucosamine--dolichyl-phosphate N-acetylglucosaminephosphotransferase	NM_001089411.1
B4G0213	4.99	23.00	OPTN	Optineurin	XM_021474935.2
A13G0439	4.99	3.32	CSGALNACT2	Chondroitin sulfate N-acetylgalactosaminyltransferase 2	NM_131272.2
A14G0547	4.99	14.14	TMCO6	Transmembrane and coiled-coil domain-containing protein 6	
A23G0137	4.99	5.42	SMPD2	Sphingomyelin phosphodiesterase 2	NM_001030219.2
A25G0135	4.99	5.30	HMG20A	High mobility group protein 20A	NM_001089334.1
A25G0242	4.99	3.61	ANO10	Anoctamin-10	NM_001077382.1
A11G0774	4.99	5.70	CDK4	Cyclin-dependent kinase 4	NM_001082993.1
B23G0649	4.99	16.40	TAMALIN	General receptor for phosphoinositides 1-associated scaffold protein	NM_001039924.1
A2G0564	4.99	3.14	IMPA1	Inositol monophosphatase 1	NM_001002745.1
B21G0296	4.99	5.17	G5714_020420	A0A7J6BTR3_9TELE Uncharacterized protein	XM_017352953.3
B19G0714	4.99	4.87	MACO1A	Macoilin-1	NM_200319.2
A13G0703	4.99	4.14	VWC2	Brorin	NM_001004560.1
B6G0056	4.99	3.87	ACVR2A	Activin receptor type-2A	XM_009302086.4
B2G0951	4.99	4.28	ELOVL1	Elongation of very long chain fatty acids protein 1	NM_001005989.3
A13G0858	4.99	7.07	HSP30	Heat shock protein 30	XM_686064.9
A6G0904	4.99	4.86	SI:CH211-239J9.1	A0A0R4IBW7_DANRE Si:ch211-239j9.1	NR_170221.1
B3G1353	4.99	4.51	CASKIN2	Caskin-2	XM_002661289.7
A20G0632	4.99	3.45	PTK2B	Protein-tyrosine kinase 2-beta	NM_212570.1
B1G0601	4.98	2.67	HPGD	15-hydroxyprostaglandin dehydrogenase [NAD(+)]	NM_001326541.1
B17G0676	4.98	1.02	SLC25A21	Mitochondrial 2-oxodicarboxylate carrier	NM_001076632.1
A1G0260	4.98	21.77	ABCA1	Phospholipid-transporting ATPase ABCA1	NM_001309465.1
B15G0420	4.98	6.41	SUPT5H	Transcription elongation factor SPT5	NM_131673.2
A9G0585	4.98	4.01	CHPF	Chondroitin sulfate synthase 2	XM_002663329.7
A9G0736	4.98	4.78	C3ORF38	Uncharacterized protein C3orf38	XM_002663346.7
B16G0963	4.98	5.65	SEH1L	Nucleoporin SEH1	NM_199923.2
B7G0232	4.98	1.12	B3GALT2	Beta-1,3-galactosyltransferase 2	NM_001128726.1
A23G0818	4.98	1.80	ANAPC10	Anaphase-promoting complex subunit 10	NM_001267697.1
A25G0277	4.98	1.75	OLFML1	Olfactomedin-like protein 1	XM_001342657.8
B16G0967	4.98	22.48	SDC2	Syndecan-2	
A7G0437	4.98	13.14	MFAP1	Microfibrillar-associated protein 1	NM_201017.1

B9G0119	4.98	2.28	COBLL1	Cordon-bleu protein-like 1	NM_001365214.1
B7G0389	4.98	9.85	TMEM208	Transmembrane protein 208	NM_207100.3
A9G0430	4.98	2.24	EFNB2A	Ephrin-B2a	NM_131023.1
B17G0253	4.98	3.14	ERO1A	ERO1-like protein alpha	NM_200350.2
B18G0388	4.98	8.80	PIK3C2A	Phosphatidylinositol 4-phosphate 3-kinase C2 domain-containing subunit alpha	XM_068214908.1
A1G0862	4.97	1.90	INTS15	Integrator complex subunit 15	XM_005170851.5
A8G0796	4.97	2.82	BAG4	BAG family molecular chaperone regulator 4	
A8G0140	4.97	14.03	ABTB1	POZ domain-containing protein 1	XM_003199051.6
A6G0290	4.97	7.08	DNPEP	Aspartyl aminopeptidase	XM_005165862.4
B23G0734	4.97	3.63	IGFBP6	Insulin-like growth factor-binding protein 6	NM_001161402.2
A7G1277	4.97	9.39	ARHGEF11	Rho guanine nucleotide exchange factor 11	NM_001031840.1
B4G0104	4.97	22.56	NAMPT	Nicotinamide phosphoribosyltransferase	XM_002661340.6
B25G0874	4.97	3.70	IRAK4	Interleukin-1 receptor-associated kinase 4	
B20G0027	4.97	5.59	MED23	Mediator of RNA polymerase II transcription subunit 23	XM_005160335.5
B4G0356	4.97	12.00	TNPO3	Transportin-3	NM_201087.1
B3G0028	4.97	4.20	SREBF2	Sterol regulatory element-binding protein 2	NM_001089466.1
A4G0633	4.97	4.86	CWF19L1	CWF19-like protein 1	NM_001044758.1
B1G0519	4.97	4.04	CENPK	Centromere protein K	NM_001256665.2
B5G0601	4.97	4.81	UBAC1	Ubiquitin-associated domain-containing protein 1	NM_001110283.2
A4G0507	4.97	3.94	SFMBT2	Scm-like with four MBT domains protein 2	XM_677950.9
A25G0267	4.97	2.63	DNAJC24	DnaJ homolog subfamily C member 24	NM_200452.1
B13G0842	4.96	3.92	FOXN2	Forkhead box protein N2	XM_017358912.3
A8G0466	4.96	5.99	SRPK1	SRSF protein kinase 1	XM_017357280.3
B4G0303	4.96	4.49	ANKRD16	Ankyrin repeat domain-containing protein 16	NM_001017563.2
B13G0263	4.96	7.24	NEFL	Neurofilament light polypeptide	XM_005156963.5
B2G1214	4.96	5.54	USP14	Ubiquitin carboxyl-terminal hydrolase 14	NM_199973.1
B2G0283	4.96	9.12	EPC1	Enhancer of polycomb homolog 1	NM_001089409.1
B2G0151	4.96	5.64	FYCO1	FYVE and coiled-coil domain-containing protein 1	XM_003197950.6
A20G0800	4.96	3.12	INTS7	Integrator complex subunit 7	NM_173267.1
B7G1145	4.96	3.35	TRERF1	Transcriptional-regulating factor 1	XM_068218382.1
B22G0953	4.96	7.76	GVINP1	Interferon-induced very large GTPase 1	XM_021473534.2
A17G0236	4.96	5.17	ASXL1	Polycomb group protein ASXL1	XM_005158855.5
B12G0529	4.96	113.77	MA2	4A	NM_212620.2
B15G0450	4.96	2.20	NKAPD1	Uncharacterized protein NKAPD1	NM_001160409.1
A2G0665	4.96	15.49	FAIM2	Protein lifeguard 2	XM_005163497.5
A1G0186	4.96	3.22	PELI1	E3 ubiquitin-protein ligase pellino homolog 1	XM_009294959.4
A4G0880	4.95	4.46	PGM3	Phosphoacetylglucosamine mutase	NM_001007053.1
A16G0259	4.95	5.40	SCAP	Sterol regulatory element-binding protein cleavage-activating protein	XM_681493.8

A1G0316	4.95	8.31	HADH	Hydroxyacyl-coenzyme A dehydrogenase, mitochondrial	NM_001003515.1
B5G0672	4.95	5.57	DBN1	Drebrin	XM_005165436.5
B5G0451	4.95	3.57	CSTEINMANNI_LOCUS4741958	A0A6F9CUR8_9TELE Uncharacterized protein	
B15G0296	4.95	6.41	COQ8B	Atypical kinase COQ8B, mitochondrial	NM_001424002.1
B15G0217	4.95	5.19	RCBTB2	RCC1 and BTB domain-containing protein 2	XM_005173365.5
B5G0070	4.95	3.68	RPAIN-A	RPA-interacting protein A	XM_005171732.5
B21G0863	4.95	7.39	TOR1B	Torsin-1B	
B2G1007	4.95	8.34	DIS3L2	DIS3-like exonuclease 2	XM_005163248.4
B3G1239	4.95	9.88	PPP1R9B	Neurabin-2	XM_681422.9
A17G0439	4.95	4.69	ARHGEF10	Rho guanine nucleotide exchange factor 10	XM_009293079.4
A25G0607	4.95	10.73	TSG101	Tumor susceptibility gene 101 protein	NM_001002089.1
B5G1003	4.95	6.44	MAN1B1	Endoplasmic reticulum mannosyl-oligosaccharide 1,2-alpha-mannosidase	NM_001099979.1
A10G0312	4.95	5.00	DHRS13	reductase SDR family member 13	NM_001004641.1
B14G0251	4.95	2.84	ZBTB33	Transcriptional regulator Kaiso	XM_005173251.5
B9G0332	4.95	10.55	DOP1B	Protein dopey-2	XM_017357831.3
A22G0486	4.95	3.47	HDGFL2	Hepatoma-derived growth factor-related protein 2	NM_001002037.2
A3G0587	4.95	24.92	GLYR1	Cytokine-like nuclear factor N-PAC	XM_005164104.5
A23G0595	4.95	4.27	TBPL1	TATA box-binding protein-like 1	XM_005162305.4
A21G0229	4.95	7.10	UBAP1	Ubiquitin-associated protein 1	NM_001328344.1
A8G0196	4.95	6.31	SLC66A1	Lysosomal amino acid transporter 1 homolog	NM_001013303.2
A2G0036	4.95	10.07	TRIM25	ISG15 ligase TRIM25	XM_690458.9
B7G0252	4.95	3.91	PIEZO1	Piezo-type mechanosensitive ion channel component 1	XM_691263.9
B16G0112	4.94	8.26	UBE2E2	Ubiquitin-conjugating enzyme E2 E2	NM_001003494.1
B6G0867	4.94	3.39	BCL2L14	Apoptosis facilitator Bcl-2-like protein 14	XM_005172116.5
B7G0655	4.94	7.10			XM_021471991.2
A23G0835	4.94	2.13	POL	Retrovirus-related Pol polyprotein from transposon 17.6	
B5G0577	4.94	4.30	ENTREP1	Endosomal transmembrane epsin interactor 1	XM_009301814.4
A10G0571	4.94	5.08	ARR3	Arrestin red cell isoform 3	XM_009305269.4
B3G0568	4.94	8.83	TMEM184A	Transmembrane protein 184A	NM_213520.1
A8G0569	4.94	7.64	KLHL12	Kelch-like protein 12	NM_001007328.1
A18G0112	4.94	2.00	ST14	Suppressor of tumorigenicity 14 protein homolog	XM_005159249.4
A16G1009	4.94	1.13	LYPLAL1	Lysophospholipase-like protein 1	XM_017351345.3
B17G0714	4.94	6.75	TAGAP	T-cell activation Rho GTPase-activating protein	NM_200868.2
A8G0227	4.94	4.67	G5714_009481	A0A7J6CSC8_9TELE Uncharacterized protein	XR_223934.5
B3G0203	4.94	2.81	POLR3H	DNA-directed RNA polymerase III subunit RPC8	NM_001002476.1
A15G0155	4.94	2.93	LPAR6	Lysophosphatidic acid receptor 6	NM_001039991.2

B6G0132	4.94	6.49	DTD1	D-aminoacyl-tRNA deacylase 1	NM_001003440.1
B9G0287	4.94	12.65	TFDP1	Transcription factor Dp-1	NM_213492.2
A25G0772	4.94	6.60	TRIM44	Tripartite motif-containing protein 44	
B6G0720	4.94	1.75	C1ORF159	Uncharacterized protein C1orf159	NM_001326426.1
B6G0825	4.94	15.30	PBRM1	Protein polybromo-1	XM_068221698.1
A21G0049	4.94	13.96	SCARB2	Lysosome membrane protein 2	NM_001365142.1
B12G0229	4.93	3.95	LRRC59	Leucine-rich repeat-containing protein 59	NM_212932.1
A17G0426	4.93	1.38	RTN4IP1	Reticulon-4-interacting protein 1 homolog, mitochondrial	NM_200352.2
A17G0724	4.93	5.21	EAPP	E2F-associated phosphoprotein	NM_199784.1
B20G0509	4.93	20.89	KIDINS220B	Kinase D-interacting substrate of 220 kDa B	XM_068215388.1
A19G0777	4.93	6.63	CDK12	Cyclin-dependent kinase 12	XM_003200531.6
A23G0047	4.93	16.30	ARL6IP5	PRA1 family protein 3	NM_001002517.2
A13G0287	4.93	10.32	RDH11	Retinol dehydrogenase 11	XM_068212916.1
B17G0037	4.93	4.95	DNAJC17	DnaJ homolog subfamily C member 17	NM_200246.2
B11G0035	4.93	7.36	DPX16_8117	A0A3N0Y8F3_ANAGA Cellular nucleic acid-binding protein	NM_001328389.1
A14G0086	4.93	3.86	IL4/13B	13B PE=2 SV=1	
B25G0173	4.93	1.43	IGKV1-8	Immunoglobulin kappa variable 1-8	XM_068217596.1
B17G0141	4.93	5.27	RMND1	Required for meiotic nuclear division protein 1 homolog	NM_001020772.1
B3G0314	4.93	5.09	GLIS2	Zinc finger protein GLIS2	XM_021469271.2
A2G0007	4.92	0.92	PRDX6	Peroxiredoxin-6	
A7G0824	4.92	3.11	TP53I11	Tumor protein p53-inducible protein 11	NM_001013352.2
B3G0334	4.92	18.61	NFIX	Nuclear factor 1 X-type	XM_068217605.1
A4G0748	4.92	3.31	IL17RA	Interleukin-17 receptor A	NM_001100003.1
A11G0059	4.92	2.18	TRAF3IP2	E3 ubiquitin ligase TRAF3IP2	
B4G0346	4.92	6.31	DYRK2	Dual specificity tyrosine-phosphorylation-regulated kinase 2	XM_005164640.5
B15G0871	4.92	2.69	AASDHPPT	L-aminoadipate-semialdehyde dehydrogenase-phosphopantetheinyl transferase	NM_001033729.1
B5G0865	4.92	2.52	LAMC3	Laminin subunit gamma-3	XM_682251.10
B18G0035	4.92	8.75	PPP1R37	Protein phosphatase 1 regulatory subunit 37	NM_001080167.2
B18G0648	4.92	7.60	NUP205	Nuclear pore complex protein Nup205	NM_001313687.1
B8G0778	4.92	8.36	GCLM	Glutamate--cysteine ligase regulatory subunit	NM_199845.1
A7G0819	4.92	6.57	CAPRIN1	Caprin-1	XM_005172274.5
A17G0540	4.92	7.31	ABHD12	Lysophosphatidylserine lipase ABHD12	NM_001076597.1
A2G1151	4.92	12.22	CALR	Calreticulin	NM_201465.3
B3G1416	4.92	34.37	NLRC3	NLR family CARD domain-containing protein 3	XM_021470978.2
A7G1124	4.92	7.97	AKAP13	A-kinase anchor protein 13	XM_021471854.2
A5G1335	4.92	2.82	UNG	Uracil-DNA glycosylase	
A2G0491	4.92	17.87	CSRNP1	serine-rich nuclear protein 1	XM_683666.9
A2G0219	4.91	4.93	MIGA1	Mitoguardin 1	XM_005163281.5
A24G0463	4.91	7.45	PIP4K2A	Phosphatidylinositol 5-phosphate 4-kinase type-2 alpha	NM_001128702.1
A15G0417	4.91	2.25	PAK4	threonine-protein kinase PAK 4	NM_001002222.2

A17G0641	4.91	3.73	GPR137C	Integral membrane protein GPR137C	NM_001007433.1
B2G1172	4.91	14.57	VPS4B	Vacuolar protein sorting-associated protein 4B	XM_009298075.4
B2G0942	4.91	3.85	UBXN7	UBX domain-containing protein 7	NM_001001951.1
B20G0436	4.91	6.99	TRIM25	ISG15 ligase TRIM25	XM_068215643.1
B2G1033	4.91	7.15	WACA	WW domain-containing adapter protein with coiled-coil	NM_001005971.1
A17G0308	4.91	8.71	ZNF839	Zinc finger protein 839	NM_001423822.1
B8G1025	4.91	0.98	ZNF367	Zinc finger protein 367	NM_001017726.2
A11G0447	4.91	6.92	DYNLT3	Dynein light chain Tctex-type 3	NM_001044753.1
B21G0760	4.91	4.15	DENND1A	DENN domain-containing protein 1A	XM_005161496.4
B21G0073	4.91	13.22	SEPT8A	Septin-8-A	NM_001115117.1
A1G0048	4.91	5.16	DNM2	Dynamin-2	NM_213242.1
B12G0184	4.91	2.40	BIRC5.2	Baculoviral IAP repeat-containing protein 5.2	NM_194397.3
A3G1040	4.91	7.07	SP140L	Nuclear body protein SP140-like protein	XM_001337203.8
A17G0321	4.91	7.87	PCMT	Protein-L-isoaspartate(D-aspartate) O-methyltransferase	NM_200768.2
A19G0514	4.90	9.53	PRDM1	PR domain zinc finger protein 1	
A19G0057	4.90	46.17	ROHU_029625	A0A498LUZ7_LABRO Thyroid hormone receptor beta isoform X2	NM_131340.1
B3G0709	4.90	13.69	PSMD11A	26S proteasome non-ATPase regulatory subunit 11A	NM_001166238.2
A2G0381	4.90	2.85	SI:DKEYP-13A3.3	Probable flap endonuclease 1 homolog	NM_001017611.2
A9G0850	4.90	4.50	POL	RNA-directed DNA polymerase from mobile element jockey	
B1G0403	4.90	1.19	FHIP1A	FHF complex subunit HOOK interacting protein 1A	XM_009291274.4
A8G0979	4.90	5.03	SUSD3	Sushi domain-containing protein 3	
A20G0493	4.90	5.28	UBR7	Putative E3 ubiquitin-protein ligase UBR7	NM_212629.1
B18G0033	4.90	5.56	SHKBP1	SH3KBP1-binding protein 1	XM_001920900.8
B21G0965	4.90	5.05	TRIM16	Tripartite motif-containing protein 16	XM_681071.8
A18G0507	4.90	8.74	PHAF1	Phagosome assembly factor 1	NM_001082813.1
A7G0256	4.90	7.90	CSNK1G1	Casein kinase I isoform gamma-1	XM_009303474.4
B5G1062	4.90	0.94	MARVELD2	MARVEL domain-containing protein 2	NM_001126406.1
B18G0427	4.90	3.17	RGMA	Repulsive guidance molecule A	NM_001045011.1
B24G0719	4.90	13.50	CUL2	Cullin-2	XM_021470256.2
B24G0075	4.90	3.87	IGLC3	Ig lambda-3 chain C region	XM_068217596.1
B14G0505	4.90	5.36	NCBP1	Nuclear cap-binding protein subunit 1	XM_005157337.5
B5G0073	4.89	1.93	MYO19	Unconventional myosin-XIX	XM_021476376.2
B22G0730	4.89	5.31	CFD	Complement factor D	XM_021473474.2
B13G0252	4.89	2.73	SEMA4G	Semaphorin-4G	NM_001012376.1
B12G0621	4.89	3.66	ERBB2	Receptor tyrosine-protein kinase erbB-2	NM_200119.2
A20G0040	4.89	4.85	WDR20	WD repeat-containing protein 20	XM_003200785.6
B15G0081	4.89	1.90	NLRC3	Protein NLRC3	XM_068222163.1
B1G0940	4.89	3.49	NUP133	Nuclear pore complex protein Nup133	NM_213531.1
B12G0599	4.89	8.27	PAAT	ATPase PAAT	XM_694146.9

B23G0625	4.89	3.60	TBPL1	TATA box-binding protein-like 1	XM_005162305.4
B18G0470	4.89	7.69	ARPIN	Arpin	XR_002456007.2
B19G0764	4.89	6.10	BLOC1S4	Biogenesis of lysosome-related organelles complex 1 subunit 4	NM_001003475.1
B12G0282	4.89	6.88	GAL	Galanin peptides	
B4G0607	4.89	2.87	PYROXD1	Pyridine nucleotide-disulfide oxidoreductase domain-containing protein 1	NM_200763.1
A11G0131	4.88	5.22	CTNS	Cystinosin	XM_068224075.1
B12G0653	4.88	6.86	JMJD1C	Probable JmjC domain-containing histone demethylation protein 2C	XM_009306514.3
B19G0848	4.88	3.34	SI:CH211-105D11.2	Uncharacterized protein C18orf19 homolog A	NM_001044994.2
A7G0863	4.88	7.22	MTM1	Myotubularin	NM_001365554.1
A5G0210	4.88	2.01	HK2	Hexokinase-2	NM_213066.1
A8G0520	4.88	4.18	TMEM115	Transmembrane protein 115	NM_001033745.1
A1G1022	4.88	7.50	APBB2	Amyloid beta precursor protein binding family B member 2	XM_021471178.2
B1G0456	4.88	7.26	LONRF2	LON peptidase N-terminal domain and RING finger protein 2	XM_001340407.9
B19G0639	4.88	5.96	SPIRE1	Protein spire homolog 1	XM_009294320.4
B13G0717	4.88	7.37	SGPP1	Sphingosine-1-phosphate phosphatase 1	XM_692747.9
A25G0334	4.88	9.13	KITLG	Kit ligand	XM_005163011.5
A22G0139	4.88	4.89	MCM2	DNA replication licensing factor mcm2	NM_173257.2
B19G0368	4.88	1.28	RFTN1	Raftlin	XM_009294248.4
A6G0012	4.88	7.52	KEAP1B	Kelch-like ECH-associated protein 1B	NM_001113477.1
B22G0661	4.88	8.04	LAMA3	Laminin subunit alpha-3	XM_021473453.1
B25G0818	4.87	8.11	RCCD1	RCC1 domain-containing protein 1	XM_009297709.4
A6G0005	4.87	6.41	MARF1	Meiosis regulator and mRNA stability factor 1	XM_021476961.2
B7G0435	4.87	19.71	EDIL3	EGF-like repeat and discoidin I-like domain-containing protein 3	NM_001014299.2
A10G0392	4.87	3.96	RFC3	Replication factor C subunit 3	NM_201457.1
A16G0548	4.87	2.79	A33	Zinc-binding protein A33	NM_001109704.1
A14G0488	4.87	7.64	POLR2B	DNA-directed RNA polymerase II subunit RPB2	NM_001024461.3
A19G0667	4.87	6.37	ING4	Inhibitor of growth protein 4	NM_001020468.2
B22G0533	4.87	5.47	HYAL2	Hyaluronidase-2	XM_005174007.5
B21G0724	4.87	4.00	CD8B1	A8HDP4_CYPCA CD8 beta 1	XM_001345212.8
B4G0480	4.87	2.63	ATXN10	Ataxin-10	NM_001130612.1
B7G1149	4.87	5.69	SAT2	Thialysine N-epsilon-acetyltransferase	XM_692551.6
A18G0344	4.87	4.40	TSPAN18	Tetraspanin-18	NM_001002439.2
A14G0726	4.87	3.98	POLR1A	DNA-directed RNA polymerase I subunit RPA1	XM_001922804.7
B13G0448	4.87	9.85	DUSP13	Dual specificity protein phosphatase 13 isoform A	XM_001344334.8
B11G0300	4.87	6.65	KBTBD8	Kelch repeat and BTB domain-containing protein 8	NM_001114928.2
A9G0255	4.87	6.50			NM_213492.2
B24G0099	4.87	8.14	MFSD1	Major facilitator superfamily domain-containing protein 1	XM_002666748.7
B15G0503	4.87	9.87	APPBP2	Amyloid protein-binding protein 2	NM_212633.1

B22G0024	4.87	2.43	NUAK2	NUAK family SNF1-like kinase 2	XM_683672.10
A12G0382	4.87	1.97	IRLA	threonine-protein kinase irlA	XM_017353768.3
B20G0586	4.87	11.36	TPR	Nucleoprotein TPR	NM_001030123.1
B18G0019	4.86	4.93	LOXL1	Lysyl oxidase homolog 1	NM_001043325.1
A20G0140	4.86	4.72	FEZ2	Fasciculation and elongation protein zeta-2	XM_005160865.5
B20G0821	4.86	9.29	WDR26	WD repeat-containing protein 26	NM_001202442.2
A10G0546	4.86	5.32	VPS36	Vacuolar protein-sorting-associated protein 36	NM_199977.1
A8G0352	4.86	2.76	SLC35A3	UDP-N-acetylglucosamine transporter	NM_001123249.1
B8G0710	4.86	5.70	TAB3	TGF-beta-activated kinase 1 and MAP3K7-binding protein 3	NM_001045105.1
B14G0393	4.86	12.61	EHD1	EH domain-containing protein 1	NM_212874.2
B9G0761	4.86	29.22	DNAJC15	DnaJ homolog subfamily C member 15	NM_001003476.2
B7G0460	4.86	2.32	F13E9.13	Uncharacterized protein F13E9.13, mitochondrial	NM_001082801.1
B2G0389	4.86	7.14	CHD8	Chromodomain-helicase-DNA-binding protein 8	NM_001347671.1
B17G0030	4.86	5.82	EIF2AK4	eIF-2-alpha kinase GCN2	XM_017354479.3
B16G1045	4.86	4.03	EAF1	ELL-associated factor 1	NM_001002115.1
B2G0130	4.86	7.04	TGM1	Protein-glutamine gamma-glutamyltransferase K	XM_689858.8
A16G1019	4.86	3.63	TPBG	Trophoblast glycoprotein	NM_001252462.1
B6G0898	4.86	2.71	RBL1	Retinoblastoma-like protein 1	XM_021476946.2
A18G0355	4.86	5.02	PSTPIP1	Proline-serine-threonine phosphatase-interacting protein 1	NM_001045216.1
B15G0200	4.86	3.04	MRPS22	28S ribosomal protein S22, mitochondrial	NM_001098735.1
B20G0086	4.86	3.55	TUBE1	Tubulin epsilon chain	NM_200985.2
B16G0819	4.86	9.60	UBE2S	Ubiquitin-conjugating enzyme E2 S	NM_001025536.1
A24G0041	4.86	7.96	WDR48	WD repeat-containing protein 48	NM_001423795.1
A9G0388	4.85	4.24	PHF11	PHD finger protein 11	
A21G0196	4.85	4.83	LMAN1	Protein ERGIC-53	NM_001386234.1
A25G0517	4.85	2.97	ICE2	Little elongation complex subunit 2	XM_001344415.7
B5G0770	4.85	4.27	G5714_005658	A0A7J6D1M7_9TELE Uncharacterized protein	
B13G0038	4.85	5.13	CHS-2	Chitin synthase chs-2	XM_001334404.7
B19G0867	4.85	4.07	TPBG	Trophoblast glycoprotein	NM_194392.2
B22G0309	4.85	24.80	LOC113039542	A0A6P6IZP0_CARAU uncharacterized protein LOC113039542	XM_005161645.5
A17G0425	4.85	3.87	QRSL1	Glutamyl-tRNA(Gln) amidotransferase subunit A, mitochondrial	NM_001118898.1
B4G0353	4.85	3.91	MDM1	Nuclear protein MDM1	XM_005164639.5
B18G0177	4.85	7.40	TRIM16	E3 ubiquitin-protein ligase TRIM16	XM_068224942.1
A18G0055	4.85	1.52	BARX2	Homeobox protein BarH-like 2	XM_001342008.8
A9G0804	4.85	5.18	PPP1R9A	Neurabin-1	XM_009304433.4
A16G1030	4.85	3.26	ECHDC1	Ethylmalonyl-CoA decarboxylase	NM_001328136.1
A1G0953	4.85	8.68	PI4K2B	Phosphatidylinositol 4-kinase type 2-beta	NM_001045485.2
A18G0092	4.85	5.94	KIFC3	Kinesin-like protein KIFC3	XM_068214962.1

A14G0402	4.85	11.85	NOCT	Nocturnin	XM_695702.9
B14G0691	4.85	8.88	KDM3B	Lysine-specific demethylase 3B	XM_021472769.1
A6G0039	4.85	11.98	CYTH2	Cytohesin-2	NM_001114903.2
B9G0712	4.85	4.23	TNFSF13B	Tumor necrosis factor ligand superfamily member 13B	NM_001113590.1
B7G0256	4.85	8.73	K-CAM	B-cadherin (Fragment)	XM_017357101.3
B2G0208	4.84	3.68	KLF6	Krueppel-like factor 6	XM_001920617.6
A16G0249	4.84	2.79	RPP25L	Ribonuclease P protein subunit p25-like protein	NM_001002540.1
A6G0726	4.84	1.55	SFI1	Protein SFI1 homolog	XM_683537.9
A9G0741	4.84	0.47	G5714_010378	A0A7J6CRS3_9TELE Ig-like domain-containing protein	
A18G0490	4.84	6.25	SIAH1	E3 ubiquitin-protein ligase Siah1	XM_005159143.5
B6G0822	4.84	0.99	FAM3D	Protein FAM3D	NM_001366242.1
B3G1270	4.84	6.55	KRI1	Protein KRI1 homolog	NM_001002041.1
B12G0488	4.84	11.64	PGP	Glycerol-3-phosphate phosphatase	NM_212726.2
B7G0890	4.84	2.49	TESK1	Dual specificity testis-specific protein kinase 1	XM_009303137.4
B5G0524	4.84	4.90	BRD3	Bromodomain-containing protein 3	XM_005165593.5
A4G0629	4.84	8.20	CHPT1	Cholinephosphotransferase 1	NM_212624.1
A6G0784	4.84	2.04	LOC113050480	A0A6P6KBI8_CARAU uncharacterized protein LOC113050480	XM_001343386.8
A15G0095	4.84	9.42	PRSS16	Thymus-specific serine protease	NM_001080562.2
B3G1677	4.84	1.66	DPX16_1504	A0A3N0YZ26_ANAGA Zinc finger protein 568	XM_068220515.1
B17G0722	4.84	4.27	TMED8	Protein TMED8	NM_001018142.3
A8G0846	4.83	4.95	FGFR1A	Fibroblast growth factor receptor 1-A	NM_001309399.1
A3G0837	4.83	6.68	EMP2	Epithelial membrane protein 2	XM_068216679.1
B17G0628	4.83	1.55	SNAP23	Synaptosomal-associated protein 23	XM_005158727.4
A2G0181	4.83	6.14	XPR1	Xenotropic and polytropic retrovirus receptor 1 homolog	NM_001126390.1
A16G0017	4.83	2.89	TRIM16	Tripartite motif-containing protein 16	XM_690458.9
B16G0789	4.83	31.88	TPI1B	Triosephosphate isomerase B	NM_153668.4
B2G0028	4.83	12.24	TRIM16	Tripartite motif-containing protein 16	XM_681071.8
A10G0475	4.83	20.17	CLTC	Clathrin heavy chain 1	NM_001005391.2
B10G0432	4.83	20.72	CLTC	Clathrin heavy chain 1	NM_001005391.2
A19G0217	4.83	3.95	D17H6S53E	Uncharacterized protein C6orf47 homolog	NM_001423403.1
B1G1193	4.83	10.75	ITGAM	Integrin alpha-M	NM_001386624.1
B16G0390	4.83	5.44	TENT5A	Terminal nucleotidyltransferase 5A	NM_001077806.1
B11G0698	4.83	4.60	DENND6A	Protein DENND6A	NM_001002355.1
B12G0204	4.83	3.05	TDRKH	Tudor and KH domain-containing protein	NM_001014355.2
A7G0916	4.83	1.98	EFS	Embryonal Fyn-associated substrate	NM_001324408.1
A7G1102	4.83	4.97	HTATIP2	Oxidoreductase HTATIP2	NM_131681.2
A9G0473	4.83	7.10	WDFY2	WD repeat and FYVE domain-containing protein 2	NM_205745.1
A10G0469	4.83	12.56	ALCAMA	CD166 antigen homolog A	NM_131000.1
B25G0844	4.83	8.09	MON2	Protein MON2 homolog	XM_021470577.2
B20G0332	4.83	9.94	BRF1	Transcription factor IIIB 90 kDa subunit	
A23G0571	4.83	6.46	ASXL1	Polycomb group protein ASXL1	NM_001386454.1

B23G0600	4.83	6.36	DNAJC11	DnaJ homolog subfamily C member 11	NM_212631.1
B10G0181	4.83	5.17	PPTC7	Protein phosphatase PTC7 homolog	XM_009305138.4
A1G0376	4.83	7.06	ATP11A	Phospholipid-transporting ATPase IH	XM_005171001.5
A20G0084	4.83	8.09	RNF8	E3 ubiquitin-protein ligase rnf8	NM_205553.1
B23G0188	4.83	0.74	A33	Zinc-binding protein A33	NM_001113583.1
B13G0722	4.83	4.53	PHF3	PHD finger protein 3	NM_001200010.1
B21G0034	4.82	2.01	LIPH	Lipase member H	
A19G0335	4.82	7.59	SLA	Src-like-adapter	NM_001034989.2
B8G0385	4.82	2.55	RFESD	Rieske domain-containing protein	XM_683247.6
A3G0176	4.82	2.87	A33	Zinc-binding protein A33	XM_009299148.4
B13G0786	4.82	5.03	ZFYVE21	Zinc finger FYVE domain-containing protein 21	NM_200655.3
B21G0812	4.82	1.63			
A7G1194	4.82	3.06	SHTN1	Shootin-1	XM_021477700.2
A10G0854	4.82	27.44	TLN1	Talin-1	XM_005155500.5
B13G0540	4.82	0.90	E1301_TTI015661	A0A5A9PPN1_9TELE Uncharacterized protein	XM_685557.7
B3G1412	4.82	6.79	TIMP2	Metalloproteinase inhibitor 2	NM_213296.1
B24G0640	4.81	2.27	ECHS1	Probable enoyl-CoA hydratase, mitochondrial	NM_001244902.1
A10G0728	4.81	6.18	ARL15	ADP-ribosylation factor-like protein 15	XM_021472227.2
B13G0093	4.81	7.39	COL9A1	Collagen alpha-1(IX) chain	XM_021480543.2
B7G0158	4.81	5.06	ZNF143	Zinc finger protein 143	NM_001007441.1
A20G0767	4.81	3.68	PTGER2	Prostaglandin E2 receptor EP2 subtype	
A10G0070	4.81	6.50	MIER3	Mesoderm induction early response protein 3	NM_001030149.2
B5G0760	4.81	4.08	PLD2	Phospholipase D2	XM_021471642.2
A18G0618	4.81	3.47	TSPAN9	Tetraspanin-9	XM_009293473.4
B22G0522	4.81	2.26	ATRIP	ATR-interacting protein	NM_001044867.2
A15G0779	4.81	24.12	SPCS2	Signal peptidase complex subunit 2	XM_068213543.1
A18G0502	4.81	11.00	NLRC5	Protein NLRC5	NM_001386270.1
B12G0747	4.81	5.03	MAP2K4	Dual specificity mitogen-activated protein kinase kinase 4	NM_001089421.1
A13G0039	4.81	1.80	CACUL1	CDK2-associated and cullin domain-containing protein 1	
B20G0678	4.81	6.64	G5714_019870	A0A7J6BY51_9TELE Uncharacterized protein	
A5G0152	4.81	2.25	CHEK2	threonine-protein kinase Chk2	NM_200045.1
B7G0944	4.81	17.22	RBM4.1	RNA-binding protein 4.1	NM_199677.1
A1G0122	4.81	16.06	GIMAP9	GTPase IMAP family member 9	
B4G0668	4.81	1.50	G5714_004666	A0A7J6D5A0_9TELE Uncharacterized protein	XM_005157281.5
A3G0246	4.81	8.42	BTN1A1	Butyrophilin subfamily 1 member A1	XM_068218666.1
A1G0222	4.81	5.18	NUP133	Nuclear pore complex protein Nup133	NM_213531.1
A24G0263	4.80	55.33	COX42	Cytochrome c oxidase subunit 4 isoform 2, mitochondrial	NM_001386468.1
A8G0689	4.80	3.38	HPS4	BLOC-3 complex member HPS4	XM_068222895.1
B13G0055	4.80	5.70	LCORL	Ligand-dependent nuclear receptor corepressor-like protein	NM_001030104.1
B7G0093	4.80	31.25	YWHAE	14-3-3 protein epsilon	NM_001013341.2
A9G0199	4.80	4.46	IKZF2	Zinc finger protein Helios	XM_017357619.3
B24G0161	4.80	5.24	RHOT2	Mitochondrial Rho GTPase 2	NM_001037679.2
B18G0764	4.80	12.51	FAM168A	Protein FAM168A	XM_005173612.5

B6G0206	4.80	3.87	WDR12	Ribosome biogenesis protein wdr12	NM_199557.1
A17G0722	4.80	14.46	CFL2	Cofilin-2	NM_205700.1
A15G0473	4.80	9.27	KMT2A	Histone-lysine N-methyltransferase 2A	XM_005157583.5
A6G0962	4.80	6.47	NFATC2	Nuclear factor of activated T-cells, cytoplasmic 2	XM_695499.10
A24G0286	4.80	4.91	TNFSF10	Tumor necrosis factor ligand superfamily member 10	NM_001002593.2
B25G0795	4.80	12.35	TMEM248	Transmembrane protein 248	NM_001103121.1
B13G0679	4.80	21.63	SLC8A3	calcium exchanger 3	NM_001145579.1
B23G0825	4.80	12.55	G5714_022302	A0A7J6BPJ6_9TELE Uncharacterized protein	
B22G0936	4.80	6.89	PDGFB	Platelet-derived growth factor subunit B	NM_001423262.1
A5G0511	4.80	9.15	TSC1	Hamartin	XM_005171796.5
A1G0064	4.80	8.82	ADGRE5	Adhesion G protein-coupled receptor E5	XM_005171037.5
A3G1088	4.80	4.45	S1PR1	Sphingosine 1-phosphate receptor 1	NM_001007316.1
A6G0099	4.80	8.81	TRAK2	Trafficking kinesin-binding protein 2	XM_684210.10
A20G0776	4.80	3.83	KCNK5	Potassium channel subfamily K member 5	NM_200633.1
A11G0299	4.80	2.62	MAPKAPK2	MAP kinase-activated protein kinase 2	XM_683725.10
A23G0671	4.79	3.70	MFSD5	Molybdate-anion transporter	NM_001002713.1
A10G0291	4.79	5.28	MMP13	Collagenase 3 (Fragment)	NM_001040291.1
B17G0154	4.79	13.62	CTBP2	C-terminal-binding protein 2	XM_068214444.1
A5G0480	4.79	2.00	TSPAN15	Tetraspanin-15	NM_001017802.1
B2G0078	4.79	17.33	AP1M1	AP-1 complex subunit mu-1	NM_001331127.1
B1G0280	4.79	8.52	PCM1	Pericentriolar material 1 protein	XM_068223107.1
B25G0447	4.79	6.04	GRAMD4	GRAM domain-containing protein 4	XM_009297989.4
A4G0532	4.79	12.88	KMT2E	Inactive histone-lysine N-methyltransferase 2E	XM_009300439.4
A1G0124	4.79	0.77	RAC1	Ras-related C3 botulinum toxin substrate 1	NM_199771.1
A19G0654	4.79	4.35	CNOT3	CCR4-NOT transcription complex subunit 3	XM_017352187.3
A17G0188	4.79	5.08	KCNK3	Potassium channel subfamily K member 3	XM_694909.9
B11G0395	4.79	2.72	CHIA	Acidic mammalian chitinase	XM_009306042.4
A17G0561	4.79	7.78	BNIP3	adenovirus E1B 19 kDa protein-interacting protein 3	NM_212693.2
B24G0623	4.79	2.87	MPPE1	Metallophosphoesterase 1	NM_001017701.1
A17G0781	4.79	3.94	AFG1L	AFG1-like ATPase	NM_001199767.1
B7G0888	4.79	5.30	PIK3C3	Phosphatidylinositol 3-kinase catalytic subunit type 3	NM_001328533.1
A5G0394	4.79	4.86	SENP3	Sentrin-specific protease 3	NM_001105114.1
A15G0868	4.79	5.55	SCHIP1	Schwannomin-interacting protein 1	NM_001017906.1
A25G0333	4.79	4.19	TMTC3	Protein O-mannosyl-transferase TMTC3	XM_001922451.8
B13G0910	4.79	3.65			XM_068212982.1
B16G0306	4.79	4.13	HINT3	Adenosine 5'-monophosphoramidase HINT3	NM_001326468.1
B14G0112	4.79	7.95	FAT2	Protocadherin Fat 2	XM_001920023.8
A17G0186	4.78	12.09	TAGAP	T-cell activation Rho GTPase-activating protein	NM_200868.2
A20G0180	4.78	18.03	NAPB	Beta-soluble NSF attachment protein	NM_001080702.2

A1G0896	4.78	6.11	EIF3B	Eukaryotic translation initiation factor 3 subunit B	XM_068221005.1
B5G0566	4.78	15.33	EMB	Embigin	NM_001115111.1
A24G0347	4.78	7.82	RPS6KA3	Ribosomal protein S6 kinase alpha-3	XM_021470093.2
B23G0261	4.78	13.39	OSBPL2	Oxysterol-binding protein-related protein 2	NM_199872.2
A5G0789	4.78	9.59	NCOR1	Nuclear receptor corepressor 1	XM_009301627.4
A19G0513	4.78	8.00	PREP	Prolyl endopeptidase	XM_002667552.6
B13G0274	4.78	4.09	AP5S1	AP-5 complex subunit sigma-1	NM_199860.1
B1G0823	4.78	13.16	PPM1K	Protein phosphatase 1K, mitochondrial	NM_001327744.1
B8G0879	4.78	3.79	PTPN18	Tyrosine-protein phosphatase non-receptor type 18	NM_001013452.2
A18G0405	4.78	3.62	TMEM170A	Transmembrane protein 170A	NM_200689.2
B19G0424	4.78	1.98	CRYBG2	gamma crystallin domain-containing protein 2	XM_009294480.4
B15G0923	4.78	16.55	PAF1	RNA polymerase II-associated factor 1 homolog	NM_001024453.1
B5G0284	4.78	13.83	RNF167	E3 ubiquitin-protein ligase RNF167	XM_021476685.2
A19G0891	4.78	6.75	LOC113062540	A0A6P6LWL8_CARAU uncharacterized protein LOC113062540 isoform X1	
B6G0473	4.78	5.61	FARP2	FERM, ARHGEF and pleckstrin domain-containing protein 2	XM_068221972.1
B16G0893	4.78	6.68	PLEKHA8	Pleckstrin homology domain-containing family A member 8	NM_001007374.1
A13G0894	4.78	6.95	CLEC4M	C-type lectin domain family 4 member M	XM_021480925.2
A18G0220	4.78	9.83	GAS8	Dynein regulatory complex subunit 4	NM_199634.1
B16G0252	4.77	6.11	DYRK1A	Dual specificity tyrosine-phosphorylation-regulated kinase 1A	XM_005158214.5
A16G0558	4.77	3.88	MRPL17	39S ribosomal protein L17, mitochondrial	NM_001004683.1
B13G0474	4.77	5.02	RET	Proto-oncogene tyrosine-protein kinase receptor Ret	NM_001004530.1
A5G0655	4.77	4.42	STEEP1	STING ER exit protein	NM_001004659.1
A12G0199	4.77	7.40	PGS1	CDP-diacylglycerol--glycerol-3-phosphate 3-phosphatidyltransferase, mitochondrial	XM_003199632.6
A2G1043	4.77	1.71	GPR55	G-protein coupled receptor 55	XM_068214727.1
A7G0397	4.77	5.25	ARHGAP6	Rho GTPase-activating protein 6	XM_009303441.4
B9G0539	4.77	5.45	POGLUT2	Protein O-glucosyltransferase 2	NM_200931.1
B17G0915	4.77	4.93	ATXN3	Ataxin-3	NM_001320532.1
A16G0473	4.77	2.36	METTL25B	Methyltransferase-like protein 25B	XM_009292345.4
B5G0937	4.77	2.76	MEGF9	Multiple epidermal growth factor-like domains protein 9	XM_001336167.8
B5G0602	4.77	3.27	ENTR1	Endosome-associated-trafficking regulator 1	NM_001110284.2
A11G0673	4.77	7.65	TRYP	Trypsin	NM_001089510.2
B2G1285	4.77	2.90	STXA	Stonustoxin subunit alpha	XM_681071.8
B5G0174	4.77	6.27	FBXO21	F-box only protein 21	XM_691038.8
A16G0930	4.76	12.40	ENPP2	phosphodiesterase family member 2	NM_200603.1
B17G0313	4.76	8.11	SLC22A15B	Solute carrier family 22 member 15-like	NM_001109699.1
B8G0993	4.76	3.46	CRBN	Protein cereblon	

A23G0557	4.76	23.71	CLSTN1	Calsyntenin-1	XM_005162269.5
B19G0359	4.76	4.81	ADNP2	Activity-dependent neuroprotector homeobox protein 2	XM_005170054.5
A15G0083	4.76	9.98	LAMTOR1	Ragulator complex protein LAMTOR1	NM_001282140.1
B8G0802	4.76	188.34	BGN	Biglycan	NM_001002227.2
B9G0738	4.76	3.63	LOC113109383	A0A6P6Q5E1_CARAU uncharacterized protein LOC113109383	XR_001800369.3
B2G1280	4.76	9.50	TRIM25	ISG15 ligase TRIM25	XM_681071.8
A25G0444	4.76	2.60	PIM3	threonine-protein kinase pim-3	NM_001034978.2
B25G0047	4.76	2.23	BMB	Protein brambleberry	NM_001270554.1
A9G0438	4.76	5.15	MED4	Mediator of RNA polymerase II transcription subunit 4	NM_001017883.1
B7G0367	4.75	35.14	PER1	Period circadian protein homolog 1	NM_212439.3
B25G0112	4.75	5.92	ANKRD27	Ankyrin repeat domain-containing protein 27	XM_021470747.2
A19G0805	4.75	1.46	ERF	ETS domain-containing transcription factor ERF	XM_005159364.5
B11G0578	4.75	16.31	NTN4	Netrin-4	XM_005169409.5
A12G0564	4.75	2.32	KLHDC1	Kelch domain-containing protein 1	
B5G0554	4.75	1.54	NRARPB	Notch-regulated ankyrin repeat-containing protein B	NM_181496.5
B25G0291	4.75	7.65	SLC27A2	Long-chain fatty acid transport protein 2	NM_001008639.2
B11G0210	4.75	5.53	MKNK1	threonine-protein kinase 1	XM_005155957.4
A5G0071	4.75	8.71	GGNBP2	Gametogenetin-binding protein 2	XM_005165016.5
A8G0910	4.75	6.57	MFN2	Mitofusin-2	NM_001128254.2
A20G0715	4.75	7.35	PRRC2C	Protein PRRC2C	XM_009294615.4
A7G0368	4.75	5.35	PLAA	Phospholipase A-2-activating protein	NM_212690.1
A2G0669	4.75	4.69	FASTK	threonine kinase	NM_001170819.1
A24G0523	4.75	3.03	UBA5	Ubiquitin-like modifier-activating enzyme 5	NM_001305617.1
B2G1299	4.75	10.95	STXA	Neoverrucotoxin subunit alpha	XM_002666602.6
A5G1150	4.74	9.94	ITGAX	Integrin alpha-X	
B4G0045	4.74	1.90	ECHDC3	Enoyl-CoA hydratase domain-containing protein 3, mitochondrial	NM_001079663.1
A21G0616	4.74	6.45	LCP2	Lymphocyte cytosolic protein 2	
B15G0047	4.74	4.01	RABGEF1	GTP exchange factor	NM_200941.1
B1G1111	4.74	5.82	LOC113073303	A0A6P6N0Y7_CARAU uncharacterized protein LOC113073303 isoform X2	
B17G0331	4.74	10.89	BNIP3	adenovirus E1B 19 kDa protein-interacting protein 3	
B10G0615	4.74	9.97	USP25	Ubiquitin carboxyl-terminal hydrolase 25	XM_005169156.5
B17G0352	4.74	5.57	ENTPD6	Ectonucleoside triphosphate diphosphohydrolase 6	NM_001017862.1
A23G0104	4.74	6.34	RBM10	RNA-binding protein 10	XM_068216821.1
A10G0205	4.74	2.80	TMEM120B	Transmembrane protein 120B	XM_005169222.4
B9G0084	4.74	3.56	MID1	E3 ubiquitin-protein ligase Midline-1	XM_068223694.1
A2G0529	4.74	3.40	HLTF	Helicase-like transcription factor	XM_021467328.2
B22G0026	4.74	2.33	USP49	Ubiquitin carboxyl-terminal hydrolase 49	NM_001044896.1
B16G0552	4.74	12.97	STEAP4	Metalloreductase STEAP4	NM_001017734.2

B15G0713	4.74	7.10	SH3BP5L	SH3 domain-binding protein 5-like	NM_213160.1
B14G0378	4.74	4.16	G5714_014240	A0A7J6CC74_9TELE Uncharacterized protein	NM_001044886.1
B5G1278	4.74	2.10	ANKLE2	Ankyrin repeat and LEM domain-containing protein 2	NM_001126395.2
A12G0790	4.74	3.97	TMEM101	Transmembrane protein 101	NM_001327885.1
A24G0410	4.74	4.07	GRAMD1C	Protein Aster-C	XM_005162729.5
B24G0570	4.73	10.41	PCK1	Phosphoenolpyruvate carboxykinase, cytosolic [GTP]	NM_213192.1
A1G0803	4.73	7.31	UBE2F	NEDD8-conjugating enzyme UBE2F	NM_001423799.1
B12G0552	4.73	9.29	LOC113048371	A0A6P6K279_CARAU uncharacterized protein LOC113048371 isoform X2	
B24G0083	4.73	11.03	STUB1	E3 ubiquitin-protein ligase CHIP	NM_199674.2
A4G0752	4.73	5.46	CNOT4	CCR4-NOT transcription complex subunit 4	NM_201169.1
A8G0459	4.73	3.10	MKRN1	Probable E3 ubiquitin-protein ligase makorin-1	XM_005167043.5
B12G0729	4.73	11.29	PRSS8	Prostasin	NM_001002596.1
B1G0243	4.73	5.87	SCLT1	Sodium channel and clathrin linker 1	XM_005159813.5
A14G0505	4.73	3.72	ELP1	Elongator complex protein 1	XM_684442.9
A9G0258	4.73	7.10	CDC16	Cell division cycle protein 16 homolog	NM_001037389.2
B9G0177	4.73	4.29	DNAJC10	DnaJ homolog subfamily C member 10	NM_001083547.1
B10G0260	4.73	45.60	TCIM	Transcriptional and immune response regulator	NM_001386670.1
B25G0862	4.73	5.81	LOC107710862	A0A673KC33_9TELE Uncharacterized LOC107710862	XM_005174215.5
A5G0928	4.73	6.49	JAK2	Tyrosine-protein kinase JAK2	NM_131087.1
B21G0672	4.73	5.99	EHMT1	Histone-lysine N-methyltransferase EHMT1	XM_005161105.5
B2G0374	4.73	7.26	PRMT5	Protein arginine N-methyltransferase 5	NM_001007183.2
B3G1079	4.73	2.67	PHOSPHO1	Probable phosphatase phospho1	
B12G0257	4.73	0.23	PLEKHS1	Pleckstrin homology domain-containing family S member 1	NM_001080565.1
B8G0187	4.73	6.96	UBE2D4	Ubiquitin-conjugating enzyme E2 D4	NM_001089453.1
A24G0554	4.72	3.91	GNAL	Guanine nucleotide-binding protein G(olf) subunit alpha	NM_001007339.2
B8G0507	4.72	8.27	DALRD3	DALR anticodon-binding domain-containing protein 3	NM_001123326.1
A10G0691	4.72	22.14	MPO	Myeloperoxidase	NM_001351837.1
A5G0890	4.72	4.97	LHFPL2	LHFPL tetraspan subfamily member 2 protein	XM_021476148.2
B5G0752	4.72	7.08	G5714_005643	A0A7J6D1K2_9TELE Uncharacterized protein	NM_001128250.1
B8G0698	4.72	6.56	ABHD17A	beta hydrolase domain-containing protein 17A	XM_001342960.8
A22G0224	4.72	3.47	ATRIP	ATR-interacting protein	XM_068216311.1
B2G0604	4.72	34.41	BFSP2	Phakinin	XM_009298615.4
B1G0920	4.72	2.21	ELMOD2	ELMO domain-containing protein 2	NM_001204132.1
A3G0708	4.72	7.75	SCAF1	serine-rich 19	XM_005164068.5
B10G0223	4.72	51.78	NT5C2	Cytosolic purine 5'-nucleotidase	XM_068223773.1
A9G0847	4.72	3.33	ACTR5	Actin-related protein 5	NM_213488.1

B6G0492	4.72	5.30	CEP63	Centrosomal protein of 63 kDa	NM_200602.1
B4G0845	4.72	3.79	G5714_002766	A0A7J6D7N0_9TELE Uncharacterized protein	XM_068213652.1
A12G0733	4.72	5.07	ARHGAP27	Rho GTPase-activating protein 27	XM_005156029.5
B6G0156	4.72	16.95	MYH10	Myosin-10	XM_009302149.4
A3G0473	4.72	5.89	TMEM184A	Transmembrane protein 184A	NM_213520.1
B18G0345	4.72	5.05	OPA3	Optic atrophy 3 protein homolog	NM_001080166.1
A17G0024	4.72	7.43	RPS6KL1	Ribosomal protein S6 kinase-like 1	XM_021473217.2
B4G0190	4.72	4.52			XM_005164999.5
A9G0310	4.72	5.30	FHIP1B	FHF complex subunit HOOK interacting protein 1B	NM_001130857.1
B3G0355	4.72	5.98	IRE-1	endoribonuclease ire-1	
B7G0978	4.72	4.50	SH3GL3	Endophilin-A3	NM_001081691.2
B5G1225	4.72	4.40	WSCD2	WSC domain-containing protein 2	XM_005165158.5
B4G0047	4.72	10.83	UPF2	Regulator of nonsense transcripts 2	XM_017355122.1
A4G0458	4.72	6.92	ZBED4	Zinc finger BED domain-containing protein 4	NM_001082941.1
B9G0392	4.72	6.31	MAN1A2	Mannosyl-oligosaccharide 1,2-alpha-mannosidase IB	XM_005167537.5
B22G0502	4.72	5.64	RBCK1	RanBP-type and C3HC4-type zinc finger-containing protein 1	NM_001002168.1
B25G0115	4.72	115.78	HPRT1	Hypoxanthine-guanine phosphoribosyltransferase	
A21G0466	4.71	1.50	ALG8	Probable dolichyl pyrophosphate Glc1Man9GlcNAc2 alpha-1,3-glucosyltransferase	NM_001017647.1
A3G0644	4.71	10.83	CARM1	Histone-arginine methyltransferase CARM1	NM_001003645.2
B2G1200	4.71	8.41	IBA57	Putative transferase CAF17 homolog, mitochondrial	NM_001076635.2
A4G0513	4.71	5.07	TAF3	Transcription initiation factor TFIID subunit 3	NM_001042744.2
B24G0741	4.71	5.02	RECK	Reversion-inducing cysteine-rich protein with Kazal motifs	NM_001423853.1
A14G0606	4.71	8.10	SEC24B	Protein transport protein Sec24B	XM_001921599.8
B17G0757	4.71	5.83	G5714_017296	A0A7J6C5L0_9TELE TNFR-Cys domain-containing protein	XM_695132.8
B21G0797	4.71	6.35	FPGS	Folylpolyglutamate synthase, mitochondrial	NM_213437.2
B5G0030	4.71	4.86	TC1A	Transposable element Tc1 transposase	
B17G0608	4.71	4.47	GCFC2	Intron Large complex component GCFC2	NM_001110107.2
A7G1385	4.71	22.78	DCTN1	Dynactin subunit 1	XM_021477458.2
A14G0179	4.71	14.69	EFEMP2	EGF-containing fibulin-like extracellular matrix protein 2	NM_001008587.1
A2G1134	4.71	5.59	RAB12	Ras-related protein Rab-12	NM_001020630.1
A22G0023	4.71	23.88	SSU72	RNA polymerase II subunit A C-terminal domain phosphatase SSU72	NM_200728.1
A21G0089	4.71	5.69	RAPGEF1	Rap guanine nucleotide exchange factor 1	NM_001328346.1
A24G0355	4.71	507.92	LYZ	Lysozyme C	NM_139180.1
B3G1236	4.71	7.20	MSL1L1	Male-specific lethal 1-like 1	NM_001114468.1

B3G0041	4.70	2.24	LOC113047427	A0A6P6JXB4_CARAU zinc finger protein 271-like	XM_021470951.2
B21G0160	4.70	0.71	TRAF4	TNF receptor-associated factor 4	NM_212817.2
B2G1105	4.70	6.02	XPR1	Xenotropic and polytropic retrovirus receptor 1 homolog	NM_001126390.1
B14G0845	4.70	0.72	MS4A4A	Membrane-spanning 4-domains subfamily A member 4A	XM_003199992.6
B15G0312	4.70	0.93	RIMBP2	RIMS-binding protein 2	NM_001386253.1
A3G0917	4.70	4.79	CRAMP1	Protein cramped-like	XM_005163839.4
A1G0447	4.70	3.44	LOC113095129	A0A6P6P6C0_CARAU PR domain zinc finger protein 1-like isoform X2	XM_001340227.8
A3G0260	4.70	13.99	TMEM205	Transmembrane protein 205	NM_001080686.1
B22G0474	4.70	2.42	LOC113074463	A0A6P6N241_CARAU SLAM family member 9-like	NM_001423249.2
A18G0137	4.70	6.50	BAZ1B	Tyrosine-protein kinase BAZ1B	NM_001347672.1
A22G0669	4.70	6.39	SRF	Serum response factor	NM_001110526.1
B16G0248	4.70	4.73	PPOX	Protoporphyrinogen oxidase	NM_001039979.2
A17G0797	4.69	8.42	PTK2B	Protein-tyrosine kinase 2-beta	XM_005158577.5
A13G0681	4.69	16.47	CCAR1	Cell division cycle and apoptosis regulator protein 1	XM_068212865.1
B9G0053	4.69	18.95	RNH1	Ribonuclease inhibitor	XM_068221903.1
B5G0632	4.69	3.29	TMEM161B	Transmembrane protein 161B	NM_213371.1
A19G0500	4.69	7.88	SDC2	Syndecan-2	NM_173223.2
A5G1232	4.69	6.93	ABL1	Tyrosine-protein kinase ABL1	XM_005172047.5
B1G0975	4.69	8.21	R3HCC1L	Coiled-coil domain-containing protein R3HCC1L	
B3G1657	4.69	4.41	G5714_004447	A0A7J6D5J8_9TELE Uncharacterized protein	XM_017355605.3
B16G0415	4.69	5.76	USP45	Ubiquitin carboxyl-terminal hydrolase 45	NM_001423966.1
A8G0709	4.69	2.39	ORAI1	Calcium release-activated calcium channel protein 1	XM_689954.9
A17G0257	4.69	3.67	MBOAT2	Lysophospholipid acyltransferase 2	XM_694203.7
B10G0222	4.69	5.33	SPPL3	Signal peptide peptidase-like 3	NM_001015068.1
B17G0081	4.69	2.66	CRLS1	Cardiolipin synthase (CMP-forming)	NM_212931.1
A14G0774	4.69	8.79	ZNF711	Zinc finger protein 711	NM_001105523.2
A3G1309	4.69	3.29	G5714_004013	A0A7J6DCD2_9TELE Uncharacterized protein	
A8G0542	4.69	7.10	IFRD2	Interferon-related developmental regulator 2	XM_005167029.5
A23G0105	4.69	2.03	FRMD4B	FERM domain-containing protein 4B	XM_009296561.4
A24G0298	4.69	6.81	SKIL	Ski-like protein	NM_001130669.1
A5G1066	4.69	6.25	LOC107743817	A0A673M274_9TELE Uncharacterized LOC107743817	NM_001100055.1
A13G0826	4.68	1.61	COX20	Cytochrome c oxidase assembly protein COX20, mitochondrial	
A2G0068	4.68	6.75	BCL10	leukemia 10	XM_002660692.6
B15G0634	4.68	3.10	VEZF1	Vascular endothelial zinc finger 1	NM_001079963.1
A10G0773	4.68	9.75	TRIM23	E3 ubiquitin-protein ligase TRIM23	XM_005155602.5
B20G0806	4.68	1.33			XM_005160866.5
A11G0729	4.68	0.97	PLST	Plasticin	XM_021479917.2
A9G0009	4.68	9.90	UBXN4	UBX domain-containing protein 4	
A2G0440	4.68	6.10	RAB6B	Ras-related protein Rab-6B	NM_001013467.1
B11G0254	4.68	6.98	SP2	Transcription factor Sp2	NM_001003522.2

B9G0523	4.68	3.16	MED4	Mediator of RNA polymerase II transcription subunit 4	NM_001017883.1
B16G0251	4.68	3.04	ALKBH8	Alkylated DNA repair protein alkB homolog 8	XM_009292590.4
A3G0836	4.68	2.44	ATF7IP	Activating transcription factor 7-interacting protein 1	XM_009299462.4
A8G0847	4.68	5.38	FGFR1A	Fibroblast growth factor receptor 1-A	NM_001309399.1
A23G0570	4.68	10.73	DNAJC11	DnaJ homolog subfamily C member 11	NM_212631.1
A24G0126	4.68	1.48			
B16G0761	4.68	8.88	ERF	ETS domain-containing transcription factor ERF	XM_689171.10
B12G0536	4.68	7.81	PANK1	Pantothenate kinase 1	NM_001083841.1
A7G0998	4.68	6.18	ABR	Active breakpoint cluster region-related protein	XM_021471891.2
B21G0099	4.68	7.59	AMMECR1	Nuclear protein AMMECR1	NM_200581.1
B20G0413	4.68	3.12	CAPN3	Calpain-3	NM_001243321.1
B17G0344	4.68	10.57	CCDC6	Coiled-coil domain-containing protein 6	NM_199985.2
A21G0395	4.68	3.28	B9D2	B9 domain-containing protein 2	NM_001002394.1
B8G0918	4.68	5.76	HDAC6	Histone deacetylase 6	XM_009303752.3
A25G0761	4.68	14.29	VPS4A	Vacuolar protein sorting-associated protein 4A	XM_009298174.4
A7G1363	4.68	6.22	RIPK3	threonine-protein kinase 3	XM_001343791.6
A7G1026	4.68	6.83	USP8	Ubiquitin carboxyl-terminal hydrolase 8	
B13G0483	4.68	2.76	BCL11A	leukemia 11A	NM_200096.1
B1G0765	4.68	8.09	PKN1	threonine-protein kinase N1	XM_684239.8
A20G0822	4.67	3.40	TMEM242	Transmembrane protein 242	
A3G0980	4.67	4.21	CDC27	Cell division cycle protein 27 homolog	XM_005171566.5
B6G0544	4.67	5.51	LEPR	Leptin receptor	NM_001309403.1
A2G0609	4.67	1.10	TMEM70	Transmembrane protein 70, mitochondrial	XM_002660832.7
A2G0371	4.67	3.69	UBXN7	UBX domain-containing protein 7	NM_001001951.1
A7G0420	4.67	6.22	TCF12	Transcription factor 12	XM_005166510.4
A20G0435	4.67	12.38	ID2	DNA-binding protein inhibitor ID-2	XM_005160630.3
B23G0698	4.67	1.31	FAM110C	Protein FAM110C	XM_021470021.2
A14G0090	4.67	4.11	CPEB4	Cytoplasmic polyadenylation element-binding protein 4	NM_001351720.1
A6G0339	4.67	14.50	PPP1R12C	Protein phosphatase 1 regulatory subunit 12C	XM_005166225.5
A12G0245	4.67	5.78	DNMBP	Dynamin-binding protein	NM_001424046.1
B18G0567	4.67	3.71	ADM	Pro-adrenomedullin	
B7G0938	4.67	6.06	AKAP11	A-kinase anchor protein 11	XM_001339501.8
A10G0379	4.67	5.27	POM121	Nuclear envelope pore membrane protein POM 121	NM_001366408.1
B4G0194	4.67	3.19	TMEM168A	Transmembrane protein 168-A	NM_001083086.1
B5G0564	4.67	2.21	MALT1	Mucosa-associated lymphoid tissue lymphoma translocation protein 1	XM_005165612.5
A8G0197	4.67	5.72	TRIM33	E3 ubiquitin-protein ligase TRIM33	NM_001002871.2
B18G0080	4.67	5.06	QSER1	Glutamine and serine-rich protein 1	XM_005169967.5
B22G0853	4.67	4.52	DNAJA3	DnaJ homolog subfamily A member 3, mitochondrial	NM_001123236.1
B5G0837	4.67	7.10	EFNB1	Ephrin-B1	NM_131805.1

A15G0536	4.67	2.58	THAP12	52 kDa repressor of the inhibitor of the protein kinase	NM_001002680.1
B19G0632	4.67	12.18	TRIQK	R motif-containing protein	NM_001127256.3
A7G0509	4.66	4.07	TK2	Thymidine kinase 2, mitochondrial	XM_068222226.1
B18G0442	4.66	4.03	CFDP1	Craniofacial development protein 1	NM_001100035.1
A7G0489	4.66	4.37	CS012	Protein C19orf12 homolog	
B22G0240	4.66	6.63	G5714_021278	A0A7J6BQK4_9TELE Uncharacterized protein	XM_005161637.5
B4G0513	4.66	3.63	SORT1	Sortilin	XM_683991.10
B2G1246	4.66	6.21	NLRC3	Protein NLRC3	XM_068222163.1
B16G0626	4.66	4.83	ZFAT	Zinc finger protein ZFAT	NM_001423169.1
A2G0002	4.66	12.95	EFCAB14	EF-hand calcium-binding domain-containing protein 14	NM_207075.1
A13G0677	4.66	2.63	ZMYM1	Zinc finger MYM-type protein 1	XM_684103.9
B6G0514	4.66	6.75	NME7	Nucleoside diphosphate kinase 7	NM_130929.2
B3G1028	4.66	8.87	ZGC:73324	Coenzyme Q-binding protein COQ10 homolog, mitochondrial	NM_200789.2
B1G0402	4.66	1.69	SH3D19	SH3 domain-containing protein 19	NM_001020773.2
A12G0084	4.66	1.62	HID1	Protein HID1	NM_001002739.1
A15G0134	4.66	5.49	USP16	Ubiquitin carboxyl-terminal hydrolase 16	NM_001146097.1
B10G0219	4.66	13.66	DEPDC5	GATOR complex protein DEPDC5	XM_686358.9
B14G0675	4.66	3.88	SPON2	Spondin-2	NM_131007.1
A4G0454	4.66	3.34	ZGC:171422	A0A0R4IU53_DANRE Zgc:171422	NM_001114472.1
A6G0246	4.66	10.25	BAZ2B	Bromodomain adjacent to zinc finger domain protein 2B	XM_021477030.2
A20G0352	4.66	4.46	SLC25A24	Calcium-binding mitochondrial carrier protein SCaMC-1	NM_001004606.1
A12G0299	4.66	8.39	CBX1	Chromobox protein homolog 1	NM_001002090.1
A5G1353	4.65	8.55	RTASE	Probable RNA-directed DNA polymerase from transposon BS	XM_068218007.1
A14G0271	4.65	4.23	ZDHHC24	Probable palmitoyltransferase ZDHHC24	NM_001002324.1
A23G0370	4.65	2.16	LOC113094736	A0A6P6P5A9_CARAU coiled-coil domain-containing protein 30-like isoform X1	XM_068216893.1
B19G0435	4.65	5.60	NCDN	Neurochondrin	
A9G0645	4.65	5.64	CREG2	Protein CREG2	NM_001007305.2
B2G0812	4.65	12.61	CSRNP1	serine-rich nuclear protein 1	XM_683666.9
A10G0424	4.65	4.57	ATG101	Autophagy-related protein 101	NM_001423721.1
B19G0669	4.65	2.28	HEY1	enhancer-of-split related with YRPW motif protein 1	NM_212561.1
B1G0462	4.65	4.34	VANGL1	Vang-like protein 1	XM_005167686.5
B7G0425	4.65	3.02	RASSF7	Ras association domain-containing protein 7	NM_197942.1
A10G0656	4.65	5.73	PRKAB1	5'-AMP-activated protein kinase subunit beta-1	NM_001002632.1
B19G0045	4.65	5.09	IL6	Interleukin-6	
B5G0476	4.65	4.24	SYMPK	Symplekin	XM_694383.9
A7G0123	4.65	3.85	SLC35C1	GDP-fucose transporter 1	XM_005169053.5
B11G0780	4.65	0.16	G5714_012190	A0A7J6CKX7_9TELE Uncharacterized protein	NM_001040365.1
B2G0598	4.65	9.51	PRPF4B	threonine-protein kinase PRP4 homolog	XM_017351885.3
B15G0407	4.65	16.98	DYRK1A	Dual specificity tyrosine-phosphorylation-regulated kinase 1A	NM_001347831.1

A4G0459	4.65	4.56	BRD1	Bromodomain-containing protein 1	XM_021471544.2
A25G0329	4.65	6.32	BORCS5	BLOC-1-related complex subunit 5	XM_005163008.5
A10G0636	4.65	11.15			NM_001128230.2
B21G0657	4.64	11.78	DDT-A	D-dopachrome decarboxylase-A	NM_001002147.1
B11G0727	4.64	4.00	CLASP1B	CLIP-associating protein 1-B	NM_213406.1
A8G0940	4.64	6.48	MTOR	threonine-protein kinase mTOR	NM_001077211.3
B2G0618	4.64	4.53	TTC19	Tetratricopeptide repeat protein 19, mitochondrial	NM_001346244.1
A19G0123	4.64	5.91	NCALDB	Neurocalcin-delta B	NM_001003776.1
A8G0234	4.64	10.69	ITGB4	Integrin beta-4	XM_005166790.5
B3G0951	4.64	12.99	TBC1D17	TBC1 domain family member 17	XM_005163947.5
B7G0959	4.64	9.02	ZFAND6	AN1-type zinc finger protein 6	NM_001328569.1
B17G0179	4.64	10.15	BAZ1A	Bromodomain adjacent to zinc finger domain protein 1A	NM_001365143.1
A12G0513	4.64	5.94	BHLHA15	Class A basic helix-loop-helix protein 15	NM_001077652.1
A19G0608	4.64	9.67	DAPK2	Death-associated protein kinase 2	XM_021468288.2
A18G0482	4.64	3.87	ARL2BP	ADP-ribosylation factor-like protein 2-binding protein	NM_201001.2
A9G0467	4.64	14.57	COL6A3	Collagen alpha-3(VI) chain	XM_017357806.3
B5G0197	4.64	5.07	PPIL2	RING-type E3 ubiquitin-protein ligase PPIL2	NM_200991.2
A14G0446	4.64	4.23	ZDHHC5B	Palmitoyltransferase ZDHHC5-B	XM_009291295.4
A2G0504	4.64	4.65	MRPL34	39S ribosomal protein L34, mitochondrial	NM_001199680.2
B6G0446	4.64	12.74	GADD45A	Growth arrest and DNA damage-inducible protein GADD45 alpha	NM_001002216.1
A12G0639	4.64	4.07	HEATR1	HEAT repeat-containing protein 1	NM_199900.1
B19G0202	4.64	476.45	CLEC3B	Tetranectin	
A11G0276	4.64	10.14	ADAMTS9	A disintegrin and metalloproteinase with thrombospondin motifs 9	XM_005155722.5
B23G0891	4.64	6.16	CDKL5	Cyclin-dependent kinase-like 5	
B22G0210	4.64	3.52	CERS5	Ceramide synthase 5	XM_021469303.2
B7G0912	4.64	2.33	AJUBA	LIM domain-containing protein ajuba	XM_696274.10
A9G0605	4.64	5.54	MYO10	Unconventional myosin-X	XM_009304521.4
A3G0701	4.64	18.13	TSPAN4	Tetraspanin-4	NM_001082807.1
B11G0141	4.63	2.43	RPN1	Dolichyl-diphosphooligosaccharide--protein glycosyltransferase subunit 1	XM_021479706.2
A23G0610	4.63	6.05	TPX2-B	Targeting protein for Xklp2-B	XM_017353499.3
A24G0665	4.63	11.81	ITGB1	Integrin beta-1	NM_001034971.1
B21G0200	4.63	2.98	RAB24	Ras-related protein Rab-24	NM_001029970.2
B11G0670	4.63	5.82	ZNF362	Zinc finger protein 362	XM_021479694.2
A8G0657	4.63	1.47	HA2J	H-2 class II histocompatibility antigen, I-E alpha chain (Fragment)	NM_001424011.1
B11G0398	4.63	6.18	CHIA	Acidic mammalian chitinase	XM_001922238.7
B8G0352	4.63	11.76	SLC23A2	Solute carrier family 23 member 2	XM_002663198.6
B12G0343	4.63	10.89	NDEL1A	Nuclear distribution protein nudE-like 1-A	NM_201344.1
B9G0461	4.63	3.70	TSN	Translin	NM_001025452.1

A16G0701	4.63	9.37	ERF	ETS domain-containing transcription factor ERF	XM_689171.10
B22G0054	4.63	6.72	POL	RNA-directed DNA polymerase from mobile element jockey	
A25G0391	4.63	4.52	CNOT4	CCR4-NOT transcription complex subunit 4	XM_692417.8
B1G0912	4.63	6.47	PATL1	Protein PAT1 homolog 1	NM_001083028.1
B2G0027	4.63	5.96	PNP	Purine nucleoside phosphorylase	NM_001004628.1
B2G0689	4.63	4.66	UBE2W	Ubiquitin-conjugating enzyme E2 W	NM_001122972.1
B5G1129	4.63	8.22	CAPG	Macrophage-capping protein	NM_001289065.1
B3G1304	4.63	4.47	ANKRD40	Ankyrin repeat domain-containing protein 40	XM_001341341.9
B3G0672	4.63	23.89	GLYR1	Cytokine-like nuclear factor N-PAC	XM_005164104.5
B19G0154	4.63	6.18	STARD3	StAR-related lipid transfer protein 3	XM_005159353.5
A17G0191	4.63	8.96	THBD	Thrombomodulin	
A11G0452	4.63	3.80	PIR	Pirin	XM_001922150.6
B23G0242	4.63	4.75	PPP4R2A	threonine-protein phosphatase 4 regulatory subunit 2-A	NM_001115140.1
A18G0100	4.63	3.28	ILVBL	2-hydroxyacyl-CoA lyase 2	XM_021467622.2
A4G0268	4.63	2.68	TRIM14	Tripartite motif-containing protein 14	XM_691353.8
B11G0600	4.63	3.38	SORT1	Sortilin	XM_002663819.6
B5G0562	4.62	5.98	SECISBP2	Selenocysteine insertion sequence-binding protein 2	XM_068221567.1
A13G0071	4.62	12.84	MERTK	Tyrosine-protein kinase Mer	NM_200841.1
A14G0679	4.62	1.76	RNF20	E3 ubiquitin-protein ligase BRE1A	NM_001256175.1
B19G0566	4.62	7.58	GRN	Progranulin	
A6G0978	4.62	14.88	ZNFX1	NFX1-type zinc finger-containing protein 1	XM_021477327.2
B19G0018	4.62	2.52	LOC113119666	A0A6P6RHZ7_CARAU uncharacterized protein LOC113119666	
B8G0933	4.62	3.73	ASB6	Ankyrin repeat and SOCS box protein 6	NM_001020576.2
A15G0528	4.62	4.64	PLEKHG2	Pleckstrin homology domain-containing family G member 2	XM_068213708.1
B11G0030	4.62	8.80	SYN2	Synapsin-2	XM_009306329.4
B8G0466	4.62	9.11	KDM5BB	Lysine-specific demethylase 5B-B	XM_009303989.4
A1G0696	4.62	10.97	CD247	T-cell surface glycoprotein CD3 zeta chain	
A10G0411	4.62	6.43	C2CD2	C2 domain-containing protein 2	XM_009305499.4
B23G0088	4.62	5.36	ZHX3	Zinc fingers and homeoboxes protein 3	NM_198813.1
A3G0890	4.62	3.90	POLD1	DNA polymerase delta catalytic subunit	NM_001039810.2
A21G0643	4.62	7.33	TPST1	Protein-tyrosine sulfotransferase 1	NM_200202.2
B13G0429	4.62	3.05	RAB1A	Ras-related protein Rab-1A	NM_001040391.1
A5G0854	4.62	4.84	CCNH	Cyclin-H	XM_005165512.5
B11G0505	4.62	3.85	RPL22	60S ribosomal protein L22	NM_001079969.1
B7G0502	4.62	2.35	HRH3	Histamine H3 receptor	NM_001025518.1
A8G0084	4.62	5.93	ZDHHC12A	Palmitoyltransferase ZDHHC12-A	NM_001423845.1
A13G0382	4.61	5.69	OGDHL	2-oxoglutarate dehydrogenase-like, mitochondrial	NM_001145706.1
A19G0877	4.61	5.39	FAM126A	Hyccin	NM_205630.1

A24G0230	4.61	15.96	CPNE3	Copine-3	XM_005162839.5
A6G0980	4.61	13.44	ITCH	E3 ubiquitin-protein ligase Itchy	NM_001287095.1
B21G0731	4.61	2.31	GTF3C5	General transcription factor 3C polypeptide 5	XM_688483.9
A5G0402	4.61	5.19	ZNF703	Zinc finger protein 703	NM_131822.1
A7G0462	4.61	8.87	CPT1A	Carnitine O-palmitoyltransferase 1, liver isoform	XM_068222315.1
A5G0032	4.61	1.88	FASTKD5	FAST kinase domain-containing protein 5, mitochondrial	XM_693356.10
B5G0351	4.61	4.58	SSNA1	Microtubule nucleation factor SSNA1	NM_001144793.1
A19G0374	4.61	6.09	ZMYM4	Zinc finger MYM-type protein 4	XM_005159706.5
A18G0526	4.61	10.91	SWAP70	Switch-associated protein 70	NM_001044986.1
A3G0510	4.61	7.31	ZNF598	E3 ubiquitin-protein ligase ZNF598	NM_001033718.1
B23G0693	4.60	3.82	KLF15	Krueppel-like factor 15	NM_212746.3
B10G0840	4.60	4.17			
A9G0649	4.60	13.24	INPP4A	Inositol polyphosphate-4-phosphatase type I A	NM_001039973.2
A3G0948	4.60	5.85	TLK2	threonine-protein kinase tousled-like 2	NM_001110417.1
A7G0271	4.60	5.43	MYO9AA	Unconventional myosin-IXAa	NM_001423815.1
B6G0832	4.60	5.85	TESPA1	Protein TESPA1	XM_002662524.7
B20G0805	4.60	7.14	FLVCR2	Feline leukemia virus subgroup C receptor-related protein 2	XM_005160868.5
B17G0664	4.60	8.87	HADHA	Trifunctional enzyme subunit alpha, mitochondrial	NM_001089437.1
B12G0068	4.60	9.21	VDAC2	Voltage-dependent anion-selective channel protein 2	NM_199585.3
A17G0055	4.60	2.59	EXD2	Exonuclease 3'-5' domain-containing protein 2	NM_001114406.1
B9G0279	4.60	16.68	RCAN1	Calcipressin-1	NM_001003616.1
B17G0813	4.60	2.40	LOC113052278	A0A6P6KJQ4_CARAU uncharacterized protein LOC113048991 isoform X1	
A8G0061	4.60	1.70			
A3G0813	4.60	3.75	ALG1	Chitobiosyldiphosphodolichol beta-mannosyltransferase	NM_199867.1
A23G0507	4.60	6.11	ASB8	Ankyrin repeat and SOCS box protein 8	NM_199770.1
B5G0056	4.60	2.82	VKORC1L1	Vitamin K epoxide reductase complex subunit 1-like protein 1	NM_001020688.1
B16G1087	4.60	4.72	YRDC	Threonylcarbamoyl-AMP synthase	NM_001083867.2
A20G0765	4.60	2.49	CGRRF1	Cell growth regulator with RING finger domain protein 1	NM_001030246.1
B15G0815	4.60	3.65	PXYLP1	2-phosphoxylose phosphatase 1	XM_005157794.5
B21G0118	4.60	2.72	RBM27	RNA-binding protein 27	XM_005167934.5
A16G0877	4.60	2.11	GIMAP4	GTPase IMAP family member 4	XM_021478109.1
B1G0982	4.60	5.85	AFTPH	Aftiphilin	XM_005160172.5
B4G0628	4.60	4.33	ZBED4	Zinc finger BED domain-containing protein 4	NM_001082941.1
B15G0043	4.60	2.54	PPM1L	Protein phosphatase 1L	NM_001320183.1
B1G0953	4.60	6.61	TSC2	Tuberin	NM_001328401.1
B22G0272	4.60	5.68	G5714_021256	A0A7J6BQF0_9TELE Uncharacterized protein	XM_005161645.5
A23G0345	4.59	22.29	BGN	Biglycan	NM_001001825.2
B6G0970	4.59	76.70	TNNI1	Troponin I, slow skeletal muscle	NM_001008613.1
B13G0593	4.59	7.03	SYT14	Synaptotagmin-14	XM_001342476.9
B21G0850	4.59	5.19	LRRC8A	Volume-regulated anion channel subunit LRRC8A	NM_001029949.1
B2G0319	4.59	3.62	SLC39A6	Zinc transporter ZIP6	XM_005171365.5

B23G0168	4.59	9.70	PKP1	Plakophilin-1	XM_021469887.2
A12G0677	4.59	7.84	JMJD1C	Probable JmjC domain-containing histone demethylation protein 2C	XM_009306514.3
A20G0507	4.59	5.70	SERPINB1	Leukocyte elastase inhibitor	NM_001201454.1
B10G0549	4.59	1.42	RHBDD2	Rhomboid domain-containing protein 2	XM_001344064.8
B18G0444	4.59	4.68	CARMIL3	3 and myosin-I linker protein 3	NM_001317766.1
B25G0610	4.59	2.57	GINS3	DNA replication complex GINS protein PSF3	NM_001002874.1
A5G1270	4.59	2.65	RTASE	Probable RNA-directed DNA polymerase from transposon BS	
B9G0716	4.59	9.07	COL4A1	Collagen alpha-1(IV) chain	XM_688948.10
A3G1210	4.59	4.49	CP52B	Uncharacterized protein C16orf52 homolog B	XM_003198185.6
A24G0066	4.59	0.44	PTP10D	Tyrosine-protein phosphatase 10D	
B6G0768	4.59	8.67	TEX264	Testis-expressed protein 264	NM_205661.1
B20G0642	4.59	5.80	EPHX1	Epoxide hydrolase 1	NM_201068.1
A2G0365	4.59	6.02	ZFYVE9	Zinc finger FYVE domain-containing protein 9	XM_005171197.5
A25G0146	4.58	1.46	MYO5A	Unconventional myosin-Va	XM_009297774.4
A5G1136	4.58	13.67	RANGRF	Ran guanine nucleotide release factor	NM_001099995.1
B19G0820	4.58	9.63	RIT1	GTP-binding protein Rit1	NM_001128781.1
B5G0905	4.58	6.15	FBXW2	WD repeat-containing protein 2	NM_001018165.1
A25G0339	4.58	2.99	CEP41	Centrosomal protein of 41 kDa	NM_001002194.1
A18G0291	4.58	7.49	TIPARP	Protein mono-ADP-ribosyltransferase TIPARP	NM_001045019.3
B12G0638	4.58	7.17	MKI67	Proliferation marker protein Ki-67	NM_001277446.2
B15G0484	4.58	3.63	HMBS	Porphobilinogen deaminase	NM_001024388.1
A16G1049	4.58	2.39	IRX1-A	Iroquois-class homeodomain protein irx-1-A	NM_207185.2
B7G0889	4.58	1.49	LOC113105826	A0A6P6PMY4_CARAU uncharacterized protein LOC113105826	XM_005172330.5
A24G0414	4.58	1.70	TSTD1	Thiosulfate:glutathione sulfurtransferase	XM_001340371.8
B2G0114	4.58	12.34	TLE5	TLE family member 5	XM_068222908.1
A8G0096	4.58	3.48	CTU1	Cytoplasmic tRNA 2-thiolation protein 1	NM_200130.1
B11G0185	4.58	1.11	LARP6	La-related protein 6	XM_001920527.7
A3G0016	4.58	11.32	WBP2	WW domain-binding protein 2	NM_199710.1
B23G0400	4.58	2.67	TNFRSF1A	Tumor necrosis factor receptor superfamily member 1A	XM_009296781.4
A10G0433	4.58	4.69	THAP12	52 kDa repressor of the inhibitor of the protein kinase	XM_002663575.7
A2G0796	4.58	5.07	KLHL6	Kelch-like protein 6	NM_001005316.2
A8G0763	4.58	3.97	EIF2D	Eukaryotic translation initiation factor 2D	NM_201162.1
A3G1054	4.57	4.25	XPNPEP3	Xaa-Pro aminopeptidase 3	NM_207079.1
A7G1318	4.57	8.02	POL	Retrovirus-related Pol polyprotein from transposon 297	XR_011017212.1
A10G0401	4.57	4.89	CFAP298	Cilia- and flagella-associated protein 298	NM_200088.1
A20G0075	4.57	5.33	AIDA	Axin interactor, dorsalization-associated protein	NM_200790.2
B3G1004	4.57	6.29	SOCS1	Suppressor of cytokine signaling 1	NM_001003467.1
A3G0528	4.57	0.43	PRSS36	Polyserase-2	NM_001100150.1

B3G1088	4.57	2.69	ZBED4	Zinc finger BED domain-containing protein 4	XM_021471375.2
B7G0906	4.57	1.82	A33	Zinc-binding protein A33	NM_001386813.1
B11G0103	4.57	4.47	ADGRL4	Adhesion G protein-coupled receptor L4	
B1G0264	4.56	3.78	RBPJ	Recombining binding protein suppressor of hairless	NM_198878.2
A10G0302	4.56	2.33	MSI2	RNA-binding protein Musashi homolog 2	XM_009305537.4
B25G0737	4.56	4.91	SIN3A	Paired amphipathic helix protein Sin3a	NM_001098180.1
A11G0165	4.56	5.86	K1C1	Keratin, type I cytoskeletal 50 kDa	XM_005155697.5
B23G0356	4.56	7.66	TASOR	Protein TASOR	XM_001922115.7
A15G0354	4.56	5.20	RAP1GAP2	Rap1 GTPase-activating protein 2	XM_021481435.2
B14G0467	4.56	8.96	SYNPO	Synaptopodin	
B20G0775	4.56	4.16	FERMT1	Fermitin family homolog 1	XM_009294974.4
B13G0102	4.56	4.52	XPO5	Exportin-5	NM_001017687.1
A9G0773	4.56	4.00	CASP8	Caspase-8	
B12G0780	4.56	6.27	PEMT	Phosphatidylethanolamine N-methyltransferase	NM_200158.1
A3G0287	4.56	2.45	JMJD8	JmjC domain-containing protein 8	XM_003197992.6
A3G0159	4.56	0.94	GIMAP7	GTPase IMAP family member 7	XM_068218488.1
A18G0379	4.56	2.98	ELL2	RNA polymerase II elongation factor ELL2	XM_689088.8
A14G0202	4.56	6.34	PHF6	PHD finger protein 6	XM_068213168.1
B2G0403	4.56	6.27	TEP1	Telomerase protein component 1	XM_009298675.4
B7G0329	4.56	4.35	ZDHHC21	Palmitoyltransferase ZDHHC21	NM_001123055.1
A12G0764	4.56	0.31	PRSS21	Testisin	XM_021480208.2
B7G0360	4.56	4.49	RBPJ	Suppressor of hairless protein homolog	NM_001083551.1
A5G0590	4.56	6.30	GPR107	Protein GPR107	NM_001080046.1
B1G0587	4.56	2.56	ARHGAP10	Rho GTPase-activating protein 10	NM_001045250.1
A3G0151	4.55	16.01	POL	Retrovirus-related Pol polyprotein from transposon opus	
A8G0410	4.55	4.29	TNFRSF14	Tumor necrosis factor receptor superfamily member 14	NM_001430990.1
A1G1134	4.55	0.93	BNC2	Zinc finger protein basonuclin-2	NM_001327751.1
B14G0181	4.55	8.65	ATL3	Atlastin-3	XM_005157232.3
A1G0841	4.55	12.44	SOCS1	Suppressor of cytokine signaling 1	
A1G0620	4.55	4.52	GPR183B	G-protein coupled receptor 183-B	NM_001130812.1
A6G0698	4.55	6.01	DDI2	Protein DDI1 homolog 2	XM_068221662.1
B20G0472	4.55	5.11	PIGH	Phosphatidylinositol N-acetylglucosaminyltransferase subunit H	NM_001002565.2
A14G0199	4.55	3.25	GPC4	Glypican-4	NM_131860.2
B4G0322	4.55	7.36	MKRN1	Probable E3 ubiquitin-protein ligase makorin-1	NM_152978.1
A9G0129	4.55	4.78	ADARB1	Double-stranded RNA-specific editase 1	XM_021479018.2
A19G0036	4.55	28.85	CLIC1	Chloride intracellular channel protein 1	
A19G0437	4.55	3.02	ZCCHC17	Zinc finger CCHC domain-containing protein 17	NM_200544.2
B7G1129	4.55	2.52	IRS1-B	Insulin receptor substrate 1-B	XM_021477765.2

A6G0154	4.55	6.81	NFIL3	Nuclear factor interleukin-3-regulated protein	NM_001197058.1
A8G0258	4.55	3.43	DEF6	Differentially expressed in FDCP 6 homolog	NM_201040.1
B14G0475	4.55	4.88	RNF44	RING finger protein 44	NM_001076624.1
B6G0006	4.55	3.70	LOC115823849	A0A6J2WJ84_CHACN LOW QUALITY PROTEIN: uncharacterized protein LOC115823849	XM_021476964.2
B3G0799	4.55	5.04	NACC1	Nucleus accumbens-associated protein 1	NM_001423471.1
A10G0111	4.54	6.13	ZSWIM6	Zinc finger SWIM domain-containing protein 6	NM_001136487.1
A3G0680	4.54	5.45	RUSF1	RUS family member 1	NM_001110453.2
A21G0020	4.54	8.43	DMGDH	Dimethylglycine dehydrogenase, mitochondrial	XM_068215944.1
A7G0247	4.54	1.26	LTO1	Protein LTO1 homolog	
B4G0834	4.54	0.61	CD209C	CD209 antigen-like protein C	NM_001199373.2
B6G0501	4.54	5.68	BCL6	B-cell lymphoma 6 protein homolog	XM_021476815.2
B19G0720	4.54	2.67	MRPL36	39S ribosomal protein L36, mitochondrial	NM_001099257.1
B4G0155	4.54	3.39	TMEM209	Transmembrane protein 209	NM_001270973.1
A7G0782	4.54	3.29	LOC113052314	A0A6P6KJV9_CARAU uncharacterized protein LOC113052314 isoform X1	NM_001431059.1
A23G0807	4.54	5.67	CTNNBL1	Beta-catenin-like protein 1	NM_200866.1
B9G0654	4.54	3.66	CARF	Calcium-responsive transcription factor	XM_068223558.1
A16G0405	4.54	7.89	GSTK1	Glutathione S-transferase kappa 1	NM_001002560.1
A6G0729	4.54	6.78	PATZ1	POZ (BTB) and AT hook-containing zinc finger 1	XM_068222027.1
B3G0417	4.54	0.82	TMED1	Transmembrane emp24 domain-containing protein 1	NM_001003487.1
B14G0520	4.54	2.97	COL25A1	Collagen alpha-1(XXV) chain	XM_021481100.2
A18G0620	4.54	13.29	MICAL3A	Protein-methionine sulfoxide oxidase mical3a	XM_068218895.1
B5G0945	4.54	2.63	SPNS3	Protein spinster homolog 3	NM_001100036.1
B25G0024	4.54	3.82	FRS2	Fibroblast growth factor receptor substrate 2	NM_001002658.2
A1G0442	4.53	2.59	F1QCI3	F1QCI3_DANRE IG domain-containing protein	
A15G0150	4.53	4.38	STXA	Neoverrucotoxin subunit alpha	XM_017351236.3
B23G0507	4.53	15.64	GDI1	Rab GDP dissociation inhibitor alpha	NM_001020608.2
B5G0801	4.53	8.84	G5714_005685	A0A7J6D1Q4_9TELE Uncharacterized protein	
A7G0856	4.53	7.71	RNASEKA	Ribonuclease kappa-A	NM_001045405.2
A25G0647	4.53	2.91	TCB1	Transposable element Tcb1 transposase	
A9G0656	4.53	15.37	FHL2	Four and a half LIM domains protein 2	XM_068223359.1
A14G0514	4.53	1.07	LOC110073446	A0A6J0STU2_9SAUR collagen alpha-1(XXV) chain-like	XM_021481100.2
B7G0187	4.53	2.74	SINHCAF	SIN3-HDAC complex-associated factor	NM_001353917.1
A16G0947	4.53	1.72			
A14G0607	4.53	4.22	HOPX	Homeodomain-only protein	NM_198209.2
B19G0122	4.53	4.39	GSK3B	Glycogen synthase kinase-3 beta	NM_001044921.3
A15G0882	4.53	9.63	LIMK1	LIM domain kinase 1	XM_005169646.5

B22G0379	4.53	4.14	ROHU_001830	A0A498P2A2_LABRO Guanylate-binding 1-like protein	
A5G0897	4.53	4.72	CDK7	Cyclin-dependent kinase 7	NM_212961.2
B24G0606	4.53	3.40	ZFAND1	AN1-type zinc finger protein 1	NM_001003651.1
B21G0137	4.53	5.59	RNF145	RING finger protein 145	NM_199763.1
A7G0404	4.53	3.25	PHKA1	Phosphorylase b kinase regulatory subunit alpha, skeletal muscle isoform	XM_009303454.4
A17G0017	4.52	2.67	DPH6	Diphthine--ammonia ligase	
A23G0412	4.52	10.98	RERE	Arginine-glutamic acid dipeptide repeats protein	XM_001922746.7
A11G0420	4.52	11.32	CSTF1	Cleavage stimulation factor subunit 1	XM_005169264.4
B4G0823	4.52	13.17	TCB2	Transposable element Tcb2 transposase	XM_068220572.1
B24G0071	4.52	4.32	IGKV1D-8	Immunoglobulin kappa variable 1D-8	NM_001045408.1
B20G0608	4.52	5.12	ZNF395	Zinc finger protein 395	NM_001045115.1
B17G0623	4.52	1.11	ACBD3	Golgi resident protein GCP60	NM_001089387.2
B18G0352	4.52	4.56	SIAE	Sialate O-acetylesterase	NM_001044331.1
B1G0551	4.52	4.81	ARL6	ADP-ribosylation factor-like protein 6	NM_001008733.1
B24G0714	4.52	0.01	F13A1	Coagulation factor XIII A chain	XM_681557.9
B3G1349	4.52	10.37	MXRA7	Matrix-remodeling-associated protein 7	
A3G1207	4.52	2.35	CDR2	Cerebellar degeneration-related protein 2	XM_001921397.7
B16G0437	4.52	5.94	PRPF31	U6 small nuclear ribonucleoprotein Prp31	NM_200504.2
A10G0113	4.52	7.29	PITPNM2	Membrane-associated phosphatidylinositol transfer protein 2	XM_017354413.3
A2G0124	4.52	5.16	FYTTD1	UAP56-interacting factor	
A3G0250	4.52	4.83	ANTKMT	Adenine nucleotide translocase lysine N-methyltransferase	NM_001002083.1
A9G0052	4.52	4.76	COG3	Conserved oligomeric Golgi complex subunit 3	NM_001144040.2
B7G0352	4.52	7.20	ADAM19	Disintegrin and metalloproteinase domain-containing protein 19	XM_005172361.5
B2G1283	4.52	3.24	TRIM16	Tripartite motif-containing protein 16	XM_681071.8
B1G0922	4.52	6.68	COMMD1	COMM domain-containing protein 1	NM_001045265.1
B22G0076	4.52	5.55	CEPT1	ethanolaminephosphotransferase 1	NM_001109717.1
B25G0855	4.52	7.79	SPG11	Spatacsin	NM_001194994.1
B23G0553	4.52	4.06	TRIO	Triple functional domain protein	XM_009296957.4
B22G0771	4.52	5.83	ELL	RNA polymerase II elongation factor ELL	NM_199707.1
A15G0241	4.52	4.69	LOC113058619	A0A6P6LCT7_CARAU uncharacterized protein LOC113058619 isoform X1	
B2G0116	4.52	10.92	PLPP2	Phospholipid phosphatase 2	XM_687169.10
B21G1004	4.52	6.61	NUP54	Nucleoporin p54	XM_021468768.2
A12G0413	4.52	2.25	ARHGEF15	Rho guanine nucleotide exchange factor 15	XM_021472530.2
B13G0751	4.51	5.11	LOC107747254	A0A673HZ75_9TELE cGMP-dependent protein kinase	XM_068218707.1
B12G0269	4.51	16.68	ABLIM1	Actin-binding LIM protein 1	XM_068224571.1
B14G0557	4.51	10.64	STX5	Syntaxin-5	NM_001002333.1

A12G0346	4.51	7.72	ABCB9	ABC-type oligopeptide transporter ABCB9	XM_005156299.5
A12G0015	4.51	9.02	OAT	Ornithine aminotransferase, mitochondrial	NM_001317170.1
A23G0719	4.51	11.49	PINK1	threonine-protein kinase PINK1, mitochondrial	NM_001008628.1
A12G0695	4.51	5.19	IPMK	Inositol polyphosphate multikinase	NM_001080064.1
B2G0237	4.51	3.19	NLRP1	NACHT, LRR and PYD domains-containing protein 1 homolog	NM_001423861.1
B4G0860	4.51	2.74	X-ELEMENT\ORF2	Probable RNA-directed DNA polymerase from transposon X-element	XM_021472984.2
B13G0613	4.51	0.00	RDH11	Retinol dehydrogenase 11	
B7G0396	4.51	5.35	TCF12	Transcription factor 12	XM_005166508.4
A7G0896	4.51	3.66	NELFA	Negative elongation factor A	NM_001173501.1
A8G0757	4.51	5.74	BICD2	Protein bicaudal D homolog 2	NM_001083548.1
A25G0379	4.51	3.01	LOC107716650	A0A673J8H1_9TELE Uncharacterized LOC107716650	NM_001025519.2
B22G0222	4.51	18.42	A0A673MMJ4	A0A673MMJ4_9TELE Uncharacterized protein	XM_017353481.3
B12G0193	4.51	5.56	TRIM8	E3 ubiquitin-protein ligase TRIM8	NM_001044914.1
A20G0571	4.51	6.65	TNF	Tumor necrosis factor	
B23G0224	4.51	5.15	HAUS8	HAUS augmin-like complex subunit 8	XM_017353767.3
A14G0499	4.51	3.65	NCBP1	Nuclear cap-binding protein subunit 1	XM_005157337.5
B11G0072	4.51	15.14	INPP1	Inositol polyphosphate 1-phosphatase	XM_021479916.2
B10G0362	4.51	8.58	SMPD1	Sphingomyelin phosphodiesterase	NM_001326536.1
A13G0770	4.51	3.22	DNPH1	2'-deoxynucleoside 5'-phosphate N-hydrolase 1	NM_001431064.1
B4G0579	4.51	2.98	IL15RA	A0A6P6LNW9_CARAU interleukin-15 receptor subunit alpha isoform X7	
A23G0699	4.51	4.06	GPR182	G-protein coupled receptor 182	NM_001020478.1
B9G0787	4.50	5.60	CD58	Lymphocyte function-associated antigen 3	
A6G0058	4.50	3.59	LPIN2	Phosphatidate phosphatase LPIN2	NM_001423775.1
B19G0254	4.50	11.20	ASH1L	Histone-lysine N-methyltransferase ASH1L	NM_001346407.1
A1G0193	4.50	6.68	ZFYVE27	Protrudin	XM_005160177.5
A18G0522	4.50	2.92	LOC107740345	A0A673JTX9_9TELE A-kinase-interacting protein 1-like	NM_001044983.2
A11G0049	4.50	23.55	RTASE	Probable RNA-directed DNA polymerase from transposon BS	XM_009305746.4
A5G1167	4.50	6.63	LOC113040130	A0A6P6J2B3_CARAU sterile alpha motif domain-containing protein 3-like isoform X1	
A12G0679	4.50	4.03	EGR2B	Early growth response protein 2b	NM_130997.2
B16G0614	4.50	7.93	ZHX2	Zinc fingers and homeoboxes protein 2	NM_198814.2
B13G0044	4.50	4.07	LGI1	Leucine-rich glioma-inactivated protein 1	NM_001424033.1
A2G0919	4.50	5.78	GBP1	Guanylate-binding protein 1	NM_001002343.1

B1G0491	4.50	5.76	ALG11	GDP-Man:Man(3)GlcNAc(2)-PP-Dol alpha-1,2-mannosyltransferase	NM_001161351.1
A17G0640	4.50	6.49	ERO1A	ERO1-like protein alpha	XM_068214339.1
B12G0785	4.50	7.21	USP22	Ubiquitin carboxyl-terminal hydrolase 22	XM_021480159.2
A21G0892	4.50	0.30	PITX1	Pituitary homeobox 1	XM_021468902.2
B19G0568	4.50	3.87	RBM48	RNA-binding protein 48	
B3G0320	4.50	2.15	BTN1A1	Butyrophilin subfamily 1 member A1	XM_068218666.1
B13G0777	4.50	10.12	CDH23	Cadherin-23	XM_068217855.1
B7G0589	4.50	17.97	CELF1	CUGBP Elav-like family member 1	XM_005168991.4
B19G0350	4.50	2.02			
A21G0065	4.50	10.85	CERT1	Ceramide transfer protein	XM_021468956.2
B12G0614	4.50	8.06	P4HA1	Prolyl 4-hydroxylase subunit alpha-1	XM_009306501.4
B5G0235	4.50	3.56	FKTN	Ribitol-5-phosphate transferase FKTN	NM_001042694.2
B3G1681	4.50	6.12	GBP6	Guanylate-binding protein 6	XM_001920619.8
B15G0605	4.49	7.03	GDPD5	Glycerophosphodiester phosphodiesterase domain-containing protein 5	XM_684040.9
B19G0448	4.49	27.20	GRINA	Protein lifeguard 1	NM_001045310.1
A8G0340	4.49	11.40	RORB	Nuclear receptor ROR-beta	NM_001082819.1
A13G0213	4.49	3.37	SRSF5	arginine-rich splicing factor 5	XM_005156693.5
A25G0241	4.49	5.99	SNRK	threonine-protein kinase	NM_200833.1
B18G0631	4.49	12.02	CMIP	C-Maf-inducing protein	XM_685295.10
B2G0905	4.49	3.32	RWDD3	RWD domain-containing protein 3	NM_200756.1
A20G0456	4.49	3.80	KATNBL1	KATNB1-like protein 1	NM_001006019.2
A1G0797	4.49	2.42	FASTKD2	FAST kinase domain-containing protein 2, mitochondrial	XM_021475935.2
A11G0018	4.49	9.17	PYCR2	Pyrroline-5-carboxylate reductase 2	NM_199774.3
A14G0798	4.49	3.30	VAMP7	Vesicle-associated membrane protein 7	XM_005157135.5
A11G0518	4.49	2.77	PIK3CA	Phosphatidylinositol 4,5-bisphosphate 3-kinase catalytic subunit alpha isoform	
B15G0844	4.49	10.28	SERPINE2	Glia-derived nexin	NM_200184.1
A5G0415	4.49	3.51	DPH7	Diphthine methyltransferase	NM_001082839.1
A21G0685	4.49	3.58	SSH2	Protein phosphatase Slingshot homolog 2	NM_001135103.1
A16G0050	4.49	2.96	NKD2L	Protein naked cuticle homolog 2-like	NM_001098197.1
A6G0504	4.49	6.77	BCL6	B-cell lymphoma 6 protein homolog	NM_200734.2
B5G0924	4.49	4.12	TMEM119	Transmembrane protein 119	
A12G0474	4.49	4.24	CPPED1	threonine-protein phosphatase CPPED1	NM_001007413.1
B21G0869	4.49	1.13	ATP8B1	Phospholipid-transporting ATPase IC	XM_001920475.8
B9G0488	4.49	7.12	SERPINE3	Probable serpin E3	NM_001123237.1
B8G0677	4.49	4.45	RFX2	DNA-binding protein RFX2	NM_001013278.1
A17G0734	4.49	1.05	LOC107734221	A0A673MIB3_9TELE Antimicrobial peptide NK-lysin-like	
B6G0173	4.48	18.75	SLC44A2	Choline transporter-like protein 2	XM_005165750.5
B4G0359	4.48	3.23	IRF6	Interferon regulatory factor 6	NM_001327817.1

A7G0653	4.48	2.99	DDX28	Probable ATP-dependent RNA helicase DDX28	NM_001077332.1
B8G0674	4.48	0.50	ACER1	Alkaline ceramidase 1	NM_001017603.2
A3G0912	4.48	4.75	NME3	Nucleoside diphosphate kinase 3	NM_130928.2
A5G0559	4.48	3.08	MEGF9	Multiple epidermal growth factor-like domains protein 9	XM_001336167.8
A19G0829	4.48	2.32	CCDC127	Coiled-coil domain-containing protein 127	NM_001082883.1
A22G0071	4.48	3.73	IRF6	Interferon regulatory factor 6	NM_200598.2
B1G0524	4.48	0.71	DPCD	Protein DPCD	NM_001045377.1
A13G0625	4.48	6.02	LETM1	calcium exchanger protein	NM_199860.1
B10G0161	4.48	8.40	RASGEF1BB	Ras-GEF domain-containing family member 1B-B	NM_001003479.1
A6G0565	4.48	5.56	CYLD	Ubiquitin carboxyl-terminal hydrolase CYLD	XM_003198803.5
B3G0114	4.48	9.42	POL	Retrovirus-related Pol polyprotein from transposon 412	
B3G1566	4.48	8.92	X-ELEMENT\ORF2	Probable RNA-directed DNA polymerase from transposon X-element	
B8G0043	4.48	1.36	WNT5A	Protein Wnt-5a	XM_068223002.1
A13G0393	4.48	3.03	LDB1A	LIM domain-binding protein 1-A	NM_001017773.1
B24G0487	4.47	7.22	OXSR1	threonine-protein kinase OSR1	NM_201175.3
B19G0706	4.47	2.92	KPNA6	Importin subunit alpha-7	XM_001923886.7
B19G0394	4.47	2.37	JAZF1	Juxtaposed with another zinc finger protein 1	XM_005159518.5
B3G0275	4.47	2.67	ELAC2	Zinc phosphodiesterase ELAC protein 2	NM_001256204.1
B16G0837	4.47	7.42	DAP3	28S ribosomal protein S29, mitochondrial	
A25G0402	4.47	7.23	DNAJB9	DnaJ homolog subfamily B member 9	XM_005163063.4
B19G0034	4.47	4.05	PPP1R18	Phostensin	XM_693482.9
B2G0320	4.47	4.56	CYP7B1	Cytochrome P450 7B1	XM_693936.9
A8G0696	4.47	3.02	TRIM25	ISG15 ligase TRIM25	NM_001328104.1
A4G0984	4.47	2.66	TM7SF3	Transmembrane 7 superfamily member 3	XR_011011406.1
B8G0153	4.47	9.10	BIN3	Bridging integrator 3	NM_001123044.2
A5G0881	4.47	2.74	POMT1	Protein O-mannosyl-transferase 1	NM_001048067.2
B22G0879	4.47	12.03	HMOX2	Heme oxygenase 2	NM_001103139.1
B23G0639	4.47	8.64	PAN2	PAN2-PAN3 deadenylation complex catalytic subunit PAN2	XM_001919965.7
A23G0071	4.47	5.15	NCOA6	Nuclear receptor coactivator 6	XM_005170566.5
B11G0316	4.47	2.59	MUSTN1	Musculoskeletal embryonic nuclear protein 1	NM_001114480.1
B22G0653	4.47	8.63	CDC14AB	Dual specificity protein phosphatase CDC14AB	XM_068216345.1
B16G1098	4.47	5.99	WDR26	WD repeat-containing protein 26	XM_001921621.8
A4G1014	4.47	7.25	XPOT	Exportin-T	NM_001044852.1
A7G1200	4.46	5.95	PARP14	Protein mono-ADP-ribosyltransferase PARP14	XM_005166355.5
B1G0525	4.46	3.54	POLL	DNA polymerase lambda	NM_213243.1
A7G1362	4.46	7.10	N4BP1	NEDD4-binding protein 1	XM_005166301.5
B22G1049	4.46	3.61	G5714_021976	A0A7J6BSL6_9TELE Uncharacterized protein	
A25G0657	4.46	1.99	IGKV1-27	Immunoglobulin kappa variable 1-27	XM_068217596.1

B12G0516	4.46	5.97	USP42	Ubiquitin carboxyl-terminal hydrolase 42	XM_021472511.2
B1G0668	4.46	11.01	KDM2A	Lysine-specific demethylase 2A	XM_009293362.4
B3G1742	4.46	5.11	LOC107721246	A0A673MDT1_9TELE Gastrula zinc finger protein XlCGF57.1-like	XM_021469564.2
A14G0379	4.46	6.31	ATP8A1	Phospholipid-transporting ATPase IA	XM_068213406.1
B25G0324	4.46	1.63	GCNT3	Beta-1,3-galactosyl-O-glycosyl-glycoprotein beta-1,6-N-acetylglucosaminyltransferase 3	XM_002666917.7
A21G0682	4.46	4.85	TAOK1-B	threonine-protein kinase TAO1-B	XM_009295669.4
B20G0648	4.46	4.10	HECA	Headcase protein homolog	NM_001045126.2
A19G0215	4.46	6.48	PPP1R11	E3 ubiquitin-protein ligase PPP1R11	NM_001017627.1
A7G1151	4.46	2.50	SDHAF2	Succinate dehydrogenase assembly factor 2, mitochondrial	NM_001082864.1
A20G0652	4.46	7.12	CCM2	Cerebral cavernous malformations protein 2 homolog	NM_001002315.3
A7G0552	4.46	11.44	LMBR1	Limb region 1 protein homolog	XM_005166430.5
B8G0181	4.46	14.73	PPP1R3C	Protein phosphatase 1 regulatory subunit 3C	NM_213180.1
B22G1104	4.46	11.12	COMMD2	COMM domain-containing protein 2	
A11G0351	4.46	5.10	KDM5B	Lysine-specific demethylase 5B	XM_001920909.7
B11G0682	4.46	11.26	MUC4	Mucin-4	XM_017358195.3
A8G0210	4.46	6.89	PIERCE1	Piercer of microtubule wall 2 protein	NM_001123243.1
A17G0541	4.46	8.83	PYGB	Glycogen phosphorylase, brain form	NM_212809.2
B12G0224	4.46	4.59	HLF	Hepatic leukemia factor	NM_001197059.1
A8G0947	4.46	4.13	MRPS25	28S ribosomal protein S25, mitochondrial	NM_001017590.1
B16G1155	4.46	24.68	CTXA	Cytolytic toxin-alpha	XM_001335818.8
B22G0856	4.46	4.90	YJU2B	Probable splicing factor YJU2B	NM_205595.2
A18G0465	4.45	10.84	SMAD3	Mothers against decapentaplegic homolog 3	NM_175083.2
A1G0916	4.45	4.95	GNE	N-acetylmannosamine kinase	XM_068214395.1
A5G0597	4.45	5.36	LRSAM1	E3 ubiquitin-protein ligase LRSAM1	NM_001100004.1
A22G0441	4.45	6.54	HMG20B	SNF-related matrix-associated actin-dependent regulator of chromatin subfamily E member 1-related	NM_001020551.2
B24G0363	4.45	0.37	ADGRG2	Adhesion G-protein coupled receptor G2	XM_005162761.5
A13G0623	4.45	4.79	SI:CH211-233A24.2	Transmembrane protein KIAA1109 homolog	NM_001145694.1
A25G0195	4.45	1.92	MICA	MHC class I polypeptide-related sequence A	XM_021470779.2
A9G0663	4.45	4.47	GPR89	Golgi pH regulator	XM_002663314.6
A8G0362	4.45	8.48	ACSBG2	Long-chain-fatty-acid--CoA ligase ACSBG2	NM_001126379.1
B1G0434	4.45	3.90	TET2	Methylcytosine dioxygenase TET2	XM_005159903.5
A16G0995	4.45	9.35	INPP5B	Type II inositol 1,4,5-trisphosphate 5-phosphatase	XM_009292060.4
A6G0898	4.45	4.82	TOMM34	Mitochondrial import receptor subunit TOM34	NM_199638.2

B19G0240	4.45	2.68	DPM3	Dolichol-phosphate mannosyltransferase subunit 3	NM_200809.2
A20G0790	4.45	1.99	NEK2	threonine-protein kinase Nek2	NM_201050.1
A5G0855	4.45	3.98	TMEM161B	Transmembrane protein 161B	NM_213371.1
A14G0161	4.45	3.60	ASAH1	Acid ceramidase	NM_001006088.1
A9G0851	4.45	7.39	NDUFA4L2	NADH dehydrogenase [ubiquinone] 1 alpha subcomplex subunit 4-like 2	XM_001342673.8
A15G0249	4.45	0.89	TSPAN13	Tetraspanin-13	NM_001002334.1
A4G0465	4.44	2.28	TRMU	Mitochondrial tRNA-specific 2-thiouridylase 1	NM_001020599.1
B11G0587	4.44	11.64	SEMA3F	Semaphorin-3F	NM_001423793.1
B19G0015	4.44	4.66	MSTO1	Protein misato homolog 1	NM_199887.1
B5G0739	4.44	7.75	BMP3	Bone morphogenetic protein 3	NM_001077765.1
B3G0145	4.44	2.07	LOC113046763	A0A6P6JUE8_CARAU gastrula zinc finger protein XlCGF57.1-like isoform X1	XM_068220671.1
B5G1203	4.44	3.56	ASB12	Ankyrin repeat and SOCS box protein 12	NM_001126376.1
A19G0683	4.44	3.09	RNF115	E3 ubiquitin-protein ligase RNF115	XM_003200546.6
B3G0867	4.44	4.05	G5714_003578	A0A7J6DA54_9TELE Uncharacterized protein	
B11G0173	4.44	1.69	NR2F6	Nuclear receptor subfamily 2 group F member 6	NM_001127333.1
A17G0011	4.44	4.99	ATXN3	Ataxin-3	NM_001320532.1
A24G0029	4.44	5.61	XYLB	Xylulose kinase	NM_200379.1
A16G0038	4.44	3.39	FZD1	Frizzled-1	NM_001130614.1
A23G0696	4.44	9.90	SPRYD3	SPRY domain-containing protein 3	NM_001105694.1
A19G0412	4.44	12.97	NFYC	Nuclear transcription factor Y subunit gamma	XM_009294381.4
B9G0206	4.44	3.06	COL18A1	Collagen alpha-1(XVIII) chain	NM_001349195.1
A9G0798	4.44	1.57	SP9	Transcription factor Sp9	NM_212960.2
B20G0579	4.44	6.00	ATF6	Cyclic AMP-dependent transcription factor ATF-6 alpha	NM_001110519.1
B17G0796	4.44	3.77	SLC4A1AP	Kanadaptin	XM_685743.10
A1G0155	4.44	3.28	ASF1BB	Histone chaperone asf1b-B	NM_214757.2
A8G0309	4.44	7.86	LOC107743857	A0A673MK43_9TELE Emerin-like	NM_001386329.1
B23G0791	4.44	3.70	XCR1	Chemokine XC receptor 1	NM_001386469.1
A25G0131	4.44	1.71	SNX33	Sorting nexin-33	NM_001122855.2
B14G0483	4.43	2.43	DDX41	Probable ATP-dependent RNA helicase DDX41	XM_017354423.1
B13G0852	4.43	2.42	TOMM20	Mitochondrial import receptor subunit TOM20 homolog	
A6G0311	4.43	8.72	ACKR3	Atypical chemokine receptor 3	NM_001083832.1
A10G0482	4.43	5.96	MED13	Mediator of RNA polymerase II transcription subunit 13	NM_001281468.1
A6G0225	4.43	0.38	NFE2L2	Nuclear factor erythroid 2-related factor 2	NM_001257183.1
A10G0218	4.43	5.80	AAK1	AP2-associated protein kinase 1	XM_009305653.4
A22G0085	4.43	8.06	LONP1	Lon protease homolog, mitochondrial	XM_686620.10
B12G0823	4.43	0.79	TBX6	T-box transcription factor TBX6	
B17G0420	4.43	6.86	B3GNT2	N-acetyllactosaminide beta-1,3-N-acetylglucosaminyltransferase 2	NM_205752.2
B2G0925	4.43	1.54	ATR	threonine-protein kinase ATR	XM_691071.9
A6G0489	4.43	0.44	ROHU_033980	A0A498LCH7_LABRO Rootletin-like protein	XM_021477135.2

B8G0790	4.43	5.76	CEP350	Centrosome-associated protein 350	XM_068223108.1
A16G0920	4.43	7.40	CCR2	C-C chemokine receptor type 2	XM_001344115.5
B17G0507	4.43	3.66	CNKSR1	Connector enhancer of kinase suppressor of ras 1	NM_205659.1
B23G0144	4.43	4.52	ANKS1A	Ankyrin repeat and SAM domain-containing protein 1A	XM_005174081.4
B5G0539	4.43	3.71	PTRH1	Probable peptidyl-tRNA hydrolase	NM_001013330.3
A19G0297	4.43	1.90	HEYL	enhancer-of-split related with YRPW motif-like protein	NM_181736.1
B12G0366	4.43	5.46	PIGF	Phosphatidylinositol-glycan biosynthesis class F protein	NM_205645.1
A3G0830	4.43	7.78	CLCN7	Cl(-) exchange transporter 7	NM_001077537.1
A2G0146	4.42	8.98	BCL6	B-cell lymphoma 6 protein	NM_001100074.1
B7G0605	4.42	6.72	TENT4B	Terminal nucleotidyltransferase 4B	NM_001326696.1
A14G0078	4.42	2.57	MARS2	Methionine--tRNA ligase, mitochondrial	XM_021474305.2
B10G0033	4.42	4.52	PPIP5K2	Inositol hexakisphosphate and diphosphoinositol-pentakisphosphate kinase 2	XM_009305118.4
B16G0702	4.42	6.09	SLC50A1	Sugar transporter SWEET1	NM_001012497.2
A12G0496	4.42	5.24	MLST8	Target of rapamycin complex subunit LST8	NM_001320388.1
B3G1076	4.42	4.23	DHRS7B	reductase SDR family member 7B	NM_001017758.2
B24G0508	4.42	5.02	EZH2	Histone-lysine N-methyltransferase EZH2	NM_001077279.1
B10G0145	4.42	2.65	URM1	Ubiquitin-related modifier 1	NM_001328092.1
A2G0086	4.42	4.54	EIF2B5	Translation initiation factor eIF-2B subunit epsilon	NM_001113597.1
A4G0534	4.42	4.81	ORC5	Origin recognition complex subunit 5	NM_213198.1
A10G0472	4.42	3.04	LOC113077119	A0A6P6NAR6_CARAU uncharacterized protein LOC113077119	
A25G0443	4.42	7.99	BRD1	Bromodomain-containing protein 1	XM_005163142.5
A23G0789	4.42	3.69	GMEB2	Glucocorticoid modulatory element-binding protein 2	XM_009297110.4
A1G0627	4.42	8.78	PCCA	Propionyl-CoA carboxylase alpha chain, mitochondrial	NM_001002746.2
B13G0855	4.42	8.31	GNG4	G(O) subunit gamma-4	XM_687830.8
B22G0042	4.42	19.70	NLRC3	Protein NLRC3	XM_068216562.1
B24G0297	4.41	16.98	NRROS	Transforming growth factor beta activator LRRC33	NM_001003775.2
B5G0246	4.41	7.00	VPS33A	Vacuolar protein sorting-associated protein 33A	NM_001099973.1
B16G1131	4.41	3.39	TFPT	TCF3 fusion partner homolog	NM_001160707.1
A22G0223	4.41	7.61	IPPK	Inositol-pentakisphosphate 2-kinase	NM_001014324.1
A5G0023	4.41	0.00	SURF2	A0A6P6MSE6_CARAU surfeit locus protein 2	
B16G0326	4.41	1.17	COL22A1	A0A6P5IL90_PHACI LOW QUALITY PROTEIN: collagen alpha-1(XXII) chain	NM_001014359.1
B14G0028	4.41	8.02	TRIM16	Tripartite motif-containing protein 16	XM_681071.8
B10G0171	4.41	10.29	ITGA1	Integrin alpha-1	XM_694393.8
A12G0855	4.41	2.60	FOXK2	Forkhead box protein K2	NM_001197257.1

A5G1103	4.41	4.60	SLC25A46	Mitochondrial outer membrane protein SLC25A46	NM_001002558.1
B24G0594	4.41	9.23	VPS41	Vacuolar protein sorting-associated protein 41 homolog	NM_001423787.1
B2G0878	4.41	7.81	MFSD12	Major facilitator superfamily domain-containing protein 12	XM_005171209.5
A24G0519	4.41	6.58	KIAA1217	Sickle tail protein homolog	XM_021470275.2
B13G0299	4.41	6.45	LAMTOR3	Ragulator complex protein LAMTOR3	NM_001145590.1
A22G0101	4.41	1.16	TICAM1	TIR domain-containing adapter molecule 1	
A14G0596	4.41	6.77	RGS14	Regulator of G-protein signaling 14	XM_009307644.4
A8G0416	4.41	5.91	LRIF1	Ligand-dependent nuclear receptor-interacting factor 1	
B8G0417	4.41	1.36	MMP11	Stromelysin-3	XM_687113.10
B22G0244	4.41	2.38	A0A671LWW2	A0A671LWW2_9TELE Uncharacterized protein	XM_068216417.1
A3G0341	4.41	8.65	TBCD	Tubulin-specific chaperone D	XM_001922549.6
B13G0844	4.41	10.70	SPTAN1	Spectrin alpha chain, non-erythrocytic 1	XM_068213134.1
B9G0553	4.41	4.45	CREB1	Cyclic AMP-responsive element-binding protein 1	XM_005167701.5
B22G0684	4.41	4.72	IFI44L	Interferon-induced protein 44-like	XM_021469588.2
B20G0079	4.41	4.52	DSE	Dermatan-sulfate epimerase	NM_001030225.1
B2G1116	4.41	5.58	PPM1L	Protein phosphatase 1L	NM_001077600.1
B23G0723	4.41	11.05	CBX5	Chromobox protein homolog 5	NM_001080184.1
A15G0412	4.40	2.15	TP53I13	Tumor protein p53-inducible protein 13	XM_001338767.8
B17G0878	4.40	4.99	TRAPPC12	Trafficking protein particle complex subunit 12	XM_068214463.1
B12G0515	4.40	7.59	EIF2AK1	Eukaryotic translation initiation factor 2-alpha kinase 1	XM_021480133.2
A4G0743	4.40	3.90	DERA	Deoxyribose-phosphate aldolase	NM_001006097.2
B20G0212	4.40	6.32	ZYG11	Protein zyg-11 homolog	NM_001040332.2
B21G0826	4.40	0.22	LOXHD1	Lipoxygenase homology domain-containing protein 1	XM_021468985.2
B5G0567	4.40	2.98	MRPS30	39S ribosomal protein S30, mitochondrial	NM_001089371.1
A2G0991	4.40	4.34	NCAPD2	Condensin complex subunit 1	NM_001162502.2
B6G0462	4.40	13.79	KYAT3	Kynurenine--oxoglutarate transaminase 3	NM_200344.2
A4G1049	4.40	5.43	RSBN1L	Lysine-specific demethylase RSBN1L	
A3G0133	4.40	6.06	TRIM16	Tripartite motif-containing protein 16	NM_001423724.1
A3G0975	4.40	7.14	PRSS27	Serine protease 27	NM_001037562.2
B20G0370	4.40	11.39	PALLD	Palladin	XM_021468620.2
B6G0916	4.40	2.73	FAM83C	Protein FAM83C	NM_001326537.1
A16G0360	4.40	5.08	FHL3	Four and a half LIM domains protein 3	XM_068219340.1
B18G0854	4.40	15.55	WHAMM	WASP homolog-associated protein with actin, membranes and microtubules	XM_021467715.2
B11G0010	4.40	4.56	NLRP1	NACHT, LRR and PYD domains-containing protein 1 homolog	NM_001030267.1
A22G0611	4.40	3.89	CBX6	Chromobox protein homolog 6	XM_021469381.2
A5G1036	4.40	5.63	NUP88	Nucleoporin 88	NM_001020466.1
B3G0490	4.40	4.88	DNMT1	DNA (cytosine-5)-methyltransferase 1	NM_131189.2

A3G0024	4.40	4.80	GIMAP8	GTPase IMAP family member 8	XM_068218491.1
B1G0844	4.40	7.24	HERC3	Probable E3 ubiquitin-protein ligase HERC3	NM_001145624.1
B22G0845	4.40	4.14	KAT14	Cysteine-rich protein 2-binding protein	NM_001002699.3
A4G0961	4.39	5.78	CSRP2	Cysteine and glycine-rich protein 2	
A22G0105	4.39	3.20	CERS2	Ceramide synthase 2	NM_153670.1
B2G1104	4.39	4.08	DBT	Lipoamide acyltransferase component of branched-chain alpha-keto acid dehydrogenase complex, mitochondrial	
B22G0731	4.39	7.21	CFD	Complement factor D	XM_068219368.1
A20G0141	4.39	4.97	WDR26	WD repeat-containing protein 26	NM_001202442.2
A22G0450	4.39	2.85	PIAS4A	E3 SUMO-protein ligase PIAS4-A	NM_213403.2
B16G0747	4.39	5.16	MDC1	Mediator of DNA damage checkpoint protein 1	NM_001365561.1
A23G0657	4.39	7.41	CMTR1	Cap-specific mRNA (nucleoside-2'-O-)-methyltransferase 1	NM_200133.1
B7G1299	4.39	0.97	PLA2R1	Secretory phospholipase A2 receptor	
B9G0219	4.39	16.75	ASDURF	ASNSD1 upstream open reading frame protein	
A19G0170	4.39	5.59	EYA3	Eyes absent homolog 3	XM_005170066.5
A9G0322	4.39	4.27	RABL3	Rab-like protein 3	NM_205733.1
A7G0897	4.39	12.73	CD048	Neuropeptide-like protein C4orf48 homolog	NM_001386636.1
A21G0508	4.38	21.54	FIBPB	Acidic fibroblast growth factor intracellular-binding protein B	NM_212861.2
B2G0749	4.38	9.37	RABGGTB	Geranylgeranyl transferase type-2 subunit beta	NM_213112.2
B7G0090	4.38	6.41	RASGRP2	RAS guanyl-releasing protein 2	XM_009303654.4
A25G0437	4.38	18.02	C11ORF96	Uncharacterized protein C11orf96	
B23G0756	4.38	15.20	PINK1	threonine-protein kinase PINK1, mitochondrial	NM_001008628.1
B16G0959	4.38	13.21	ID4	DNA-binding protein inhibitor ID-4	NM_001039990.1
A4G0451	4.38	0.66	SAPCD1	Suppressor APC domain-containing protein 1	
A14G0587	4.38	7.12	SNX25	Sorting nexin-25	NM_001163293.1
A17G0697	4.38	12.42	IRF2BPL	Probable E3 ubiquitin-protein ligase IRF2BPL	NM_212687.1
A16G0643	4.38	10.35	ADAR	Double-stranded RNA-specific adenosine deaminase	NM_131596.2
A8G0508	4.38	7.02	TSPYL1	Testis-specific Y-encoded-like protein 1	XM_005167018.4
B17G0578	4.38	11.69	PCMT	Protein-L-isoaspartate(D-aspartate) O-methyltransferase	XM_005158767.5
A22G0269	4.38	6.85	MGAT5	Alpha-1,6-mannosylglycoprotein 6-beta-N-acetylglucosaminyltransferase A	NM_001045311.1
B3G0869	4.38	3.57	TNNT1	Troponin T, slow skeletal muscle	NM_001128695.1
B22G0619	4.38	7.85	TCB1	Transposable element Tcb1 transposase	
A1G0632	4.38	6.32	SPP2	Secreted phosphoprotein 24	NM_001327983.1
A21G0867	4.38	10.43	P4HA2	Prolyl 4-hydroxylase subunit alpha-2	XM_005173892.5

A9G0502	4.37	3.93	TSN	Translin	NM_001025452.1
B18G0680	4.37	3.85	SEPHS1	Selenide, water dikinase 1	NM_199701.2
B23G0817	4.37	4.78	ZNF217	Zinc finger protein 217	NM_001351640.1
B21G0747	4.37	1.81	CCBE1	Collagen and calcium-binding EGF domain-containing protein 1	NM_001163923.1
A5G0057	4.37	6.30	MTMR4	Myotubularin-related protein 4	XM_002666986.7
B22G0036	4.37	33.68	NLRC3	Protein NLRC3	XM_068216562.1
A24G0199	4.37	1.51	EGFR	Epidermal growth factor receptor	XM_695018.10
A11G0365	4.37	2.71	G5714_011842	A0A7J6CJH8_9TELE Uncharacterized protein	XM_003199484.6
A23G0431	4.37	5.32	ZBTB17	Zinc finger and BTB domain-containing protein 17	XM_003201203.6
B20G0602	4.37	2.69	PRRX1	Paired mesoderm homeobox protein 1	NM_200050.1
A24G0305	4.37	0.06	LOC107590237	A0A672Q5Z1_SINGR Uncharacterized LOC107590237	NM_001197254.1
B9G0547	4.37	4.46	ARMC8	Armadillo repeat-containing protein 8	NM_001079683.2
B3G0197	4.37	3.63	PICK1	PRKCA-binding protein	NM_001077336.1
A10G0459	4.36	7.27	VPS11	Vacuolar protein sorting-associated protein 11 homolog	NM_001037708.2
B6G0520	4.36	6.56	ACTL6A	Actin-like protein 6A	NM_173240.1
B5G1221	4.36	17.38	PIK3IP1	Phosphoinositide-3-kinase-interacting protein 1	NM_198374.2
B22G0503	4.36	7.53	RBCK1	RanBP-type and C3HC4-type zinc finger-containing protein 1	NM_001002168.1
B7G0878	4.36	1.52	CSKMT	Citrate synthase-lysine N-methyltransferase CSKMT, mitochondrial	NM_001020777.2
A12G0311	4.36	5.84	PSMD11B	26S proteasome non-ATPase regulatory subunit 11B	NM_199592.1
A6G0998	4.36	7.37	DDIT3	DNA damage-inducible transcript 3 protein	NM_001082825.1
A13G0802	4.36	9.49	PDE10A	cAMP and cAMP-inhibited cGMP 3',5'-cyclic phosphodiesterase 10A	XM_021480543.2
B21G0185	4.36	3.73	AMOT	Angiomotin	XM_686779.10
B11G0761	4.36	10.07	TK1	Thymidine kinase, cytosolic	XM_021479679.2
B5G0686	4.36	14.39	CXCL11.6	C-X-C motif chemokine 11-6	
B3G0583	4.36	9.44	CARD11	Caspase recruitment domain-containing protein 11	XM_068219349.1
B15G0721	4.36	4.51	BLOC1S3	Biogenesis of lysosome-related organelles complex 1 subunit 3	NM_001025476.1
A9G0207	4.36	4.25	ZNF148	Zinc finger protein 148	NM_001080180.2
B15G0176	4.36	3.07	URB1	Nucleolar pre-ribosomal-associated protein 1	NM_001326445.1
A19G0020	4.36	2.01			
A23G0763	4.36	5.49	MICAL1	[F-actin]-monooxygenase mical1	NM_001316730.1
B16G0541	4.36	10.46	CROT	Peroxisomal carnitine O-octanoyltransferase	XM_068213959.1
A22G0234	4.36	7.09	RASSF1	Ras association domain-containing protein 1	NM_001004550.1
A4G0422	4.36	1.85	G5714_004666	A0A7J6D5A0_9TELE Uncharacterized protein	XM_068219851.1
A10G0495	4.36	5.45	RAB1A	Ras-related protein Rab-1A	NM_001002129.2
A16G0025	4.36	12.66	WDTC1	WD and tetratricopeptide repeats protein 1	XM_005169770.5
B4G0243	4.36	6.24	TMTC1	Protein O-mannosyl-transferase TMTC1	XM_001921990.8

B10G0354	4.36	4.24	SLC7A1	High affinity cationic amino acid transporter 1	XM_021472276.2
B4G0311	4.36	2.62	ABTB3A	POZ domain-containing protein 3-A	NM_001327842.1
A13G0131	4.36	4.16	IDE	Insulin-degrading enzyme	NM_200655.3
B23G0191	4.35	2.92	EDEM2	ER degradation-enhancing alpha-mannosidase-like protein 2	NM_001305614.1
A16G0048	4.35	23.10	G5714_015592	A0A7J6C5Y9_9TELE Uncharacterized protein	
B16G0914	4.35	5.53	PALS2	Protein PALS2	NM_001256189.1
A23G0024	4.35	4.63	NSMCE3	Non-structural maintenance of chromosomes element 3 homolog	NM_001282090.1
A19G0622	4.35	6.99	TOMM40	Mitochondrial import receptor subunit TOM40 homolog	NM_212959.1
B2G0676	4.35	2.00	ATPSCKMT	ATP synthase subunit C lysine N-methyltransferase	NM_001291706.1
B14G0356	4.35	4.74	XK	Endoplasmic reticulum membrane adapter protein XK	NM_001007406.1
A12G0320	4.35	2.80	MEOX1	Homeobox protein MOX-1	NM_001002450.2
B17G0790	4.35	3.54	CLIP4	CAP-Gly domain-containing linker protein 4	NM_001362516.1
A23G0403	4.35	2.64	PHC2	Polyhomeotic-like protein 2	NM_152958.2
B12G0719	4.35	5.29	IRF3	Interferon regulatory factor 3	
B22G0559	4.35	3.54	METTL21A	Protein N-lysine methyltransferase METTL21A	
A20G0077	4.35	5.63	ADF1	Transcription factor Adf-1	NM_001386313.1
A14G0490	4.35	5.16	REST	RE1-silencing transcription factor	NM_001353866.1
B22G0485	4.35	3.08	NLRC3	Protein NLRC3	XM_021479924.2
B6G0829	4.35	2.28	PTGIS	Prostacyclin synthase	NM_001111160.1
B25G0661	4.35	2.73	HA1F	Class I histocompatibility antigen, F10 alpha chain	NM_001327918.1
B19G0796	4.34	4.68	EYA3	Eyes absent homolog 3	XM_005170064.5
B17G0596	4.34	3.48	ABRAXAS2	BRISC complex subunit Abraxas 2	XM_068214379.1
A20G0516	4.34	3.37	HINT3	Adenosine 5'-monophosphoramidase HINT3	NM_001025555.2
B2G0411	4.34	2.93	METTL17	Methyltransferase-like protein 17, mitochondrial	XM_021471253.2
A5G0981	4.34	2.96	CNTRL	Centriolin	XM_068221551.1
A8G0365	4.34	12.08	MYO1F	Unconventional myosin-If	NM_001256671.2
B21G0825	4.34	7.26	PSMD5	26S proteasome non-ATPase regulatory subunit 5	NM_001017573.2
B18G0071	4.34	1.53	PCYT1A	Choline-phosphate cytidylyltransferase A	NM_001020735.1
A20G0052	4.34	8.15	FIG4	Polyphosphoinositide phosphatase	XM_021468724.2
B8G0036	4.34	8.60	MIB2	E3 ubiquitin-protein ligase MIB2	NM_001079678.1
B3G1414	4.34	4.57	USP36	Ubiquitin carboxyl-terminal hydrolase 36	NM_001305580.1
B1G0496	4.34	4.36	PCCA	Propionyl-CoA carboxylase alpha chain, mitochondrial	NM_001002746.2
B25G0225	4.34	3.07	ABTB2	POZ domain-containing protein 2	XM_021473879.2
B22G0008	4.34	5.04	FBXO42	F-box only protein 42	NM_212739.1
A3G0480	4.34	8.99	LFNG	Beta-1,3-N-acetylglucosaminyltransferase lunatic fringe	NM_130971.2
A13G0470	4.34	3.35	ERLIN1	Erlin-1	XM_068224922.1

A22G0206	4.34	3.58	CD84	SLAM family member 5	XM_068216397.1
B8G0627	4.34	5.97	TNFRSF14	Tumor necrosis factor receptor superfamily member 14	
A3G0692	4.34	4.95	RNF25	E3 ubiquitin-protein ligase RNF25	NM_201183.1
B10G0581	4.34	6.01	NIPBLB	Nipped-B-like protein B	NM_001161447.2
B3G0381	4.34	5.10			
B7G0960	4.34	5.32	FAH	Fumarylacetoacetase	XM_021477397.2
B4G0673	4.34	4.77	A0A671K3I7	A0A671K3I7_9TELE Uncharacterized protein	XM_017355732.2
A5G0894	4.33	4.50	MTX3	Metaxin-3	NM_205621.2
A6G0886	4.33	2.76	RBL1	Retinoblastoma-like protein 1	XM_021476946.2
B17G0687	4.33	21.12	DGLUCY	D-glutamate cyclase, mitochondrial	XM_001923855.7
A4G0469	4.33	6.04	KIF21A	Kinesin-like protein KIF21A	XM_003198579.6
B8G0708	4.33	7.54	PRRG1	Transmembrane gamma-carboxyglutamic acid protein 1	NM_001005948.1
A17G0813	4.33	8.97	SUPT3H	Transcription initiation protein SPT3 homolog	XM_021467143.2
B2G0136	4.33	15.64	PIGR	Polymeric immunoglobulin receptor	NM_001306096.1
B13G0231	4.33	1.76	WASHC2	WASH complex subunit 2	NM_212937.1
B2G1272	4.33	2.78	LOC113046181	A0A6P6JST0_CARAU gastrula zinc finger protein XlCGF8.2DB-like	XM_021475395.2
A5G1350	4.33	6.19	FERMT3	Fermitin family homolog 3	XM_021476681.2
A8G0974	4.33	3.04	SLC35E2A	Solute carrier family 35 member E2A	NM_001436596.1
B25G0773	4.33	11.96	PPP1R12A	Protein phosphatase 1 regulatory subunit 12A	XM_002666774.7
B25G0560	4.33	3.45	MICAL2B	[F-actin]-monooxygenase mical2b	NM_001395867.1
B10G0357	4.33	3.93	PROSER1	Proline and serine-rich protein 1	XM_679230.10
A12G0645	4.33	3.86	PGAP3	Post-GPI attachment to proteins factor 3	XM_005156045.5
B5G0041	4.33	13.74	RAB35	Ras-related protein Rab-35	NM_001386745.1
B2G0076	4.33	4.92	EPS15L1	Epidermal growth factor receptor substrate 15-like 1	XM_021468308.2
A9G0542	4.33	5.70	CRYL1	Lambda-crystallin homolog	NM_001045341.1
A1G0068	4.33	10.67	NLRC3	Protein NLRC3	XM_068222163.1
A5G1125	4.33	3.14	ANAPC2	Anaphase-promoting complex subunit 2	NM_001256746.2
A16G0220	4.33	5.52	DPY19L1	Probable C-mannosyltransferase DPY19L1	NM_001002868.1
B25G0685	4.33	3.72	ACCS	1-aminocyclopropane-1-carboxylate synthase-like protein 1	NM_001014323.4
A13G0620	4.33	4.39	RTASE	Probable RNA-directed DNA polymerase from transposon BS	NM_213411.2
A4G0993	4.33	8.17	A33	Zinc-binding protein A33	XM_001344987.7
A17G0375	4.32	3.97	LUZP1	Leucine zipper protein 1	XM_009293139.4
A6G0013	4.32	0.82	S1PR3	Sphingosine 1-phosphate receptor 3	
A17G0213	4.32	4.42	CCDC88C	Protein Daple	NM_001423870.1
B9G0645	4.32	28.65	PTTG1IP	Pituitary tumor-transforming gene 1 protein-interacting protein	XM_005167479.5
B23G0227	4.32	4.54	PIGT	GPI transamidase component PIG-T	XM_679516.9
B6G1050	4.32	6.08	KDM6A	Lysine-specific demethylase 6A	XM_005168796.5
A16G0104	4.32	7.50	PLCL2	Inactive phospholipase C-like protein 2	XM_002667679.6

B3G1141	4.32	3.88	CDC27	Cell division cycle protein 27 homolog	XM_005171566.5
B10G0395	4.32	10.72	SYNJ1	Synaptojanin-1	NM_001007030.2
A19G0230	4.32	3.18	LPCAT1	Lysophosphatidylcholine acyltransferase 1	NM_001044341.2
B16G0580	4.32	6.91	VIRMA	Protein virilizer homolog	XM_068214141.1
B13G0929	4.32	10.17			NM_001040322.1
B16G0643	4.32	3.59	LOC107699513	A0A671QBN0_9TELE Uncharacterized LOC107699513	NM_199800.1
A1G0430	4.32	10.60	CISD2	CDGSH iron-sulfur domain-containing protein 2	NM_200383.1
A11G0548	4.32	8.68	SORT1	Sortilin	NM_001002595.2
B6G0854	4.31	4.25	PPM1H	Protein phosphatase 1H	XM_679889.9
A3G0320	4.31	15.24	TUBB4B	Tubulin beta-4B chain	NM_001076715.2
A25G0216	4.31	11.74	UBA2	SUMO-activating enzyme subunit 2	XM_009297857.4
B18G0668	4.31	2.54	SEMA3AB	Semaphorin-3ab	NM_131061.1
A7G1355	4.31	7.85	SLC12A6	Solute carrier family 12 member 6	XM_068217266.1
A7G0701	4.31	3.44	SLC39A13	Zinc transporter ZIP13	NM_001005306.3
A13G0840	4.31	2.80	GFRAL	GDNF family receptor alpha-like	NM_182860.1
B24G0372	4.31	5.62	MTMR6	Myotubularin-related protein 6	NM_199791.1
B22G0404	4.31	1.11	LOC107667496	A0A671P9C7_9TELE Uncharacterized LOC107667496	XM_017353440.3
B16G0355	4.31	4.40	CAPN7	Calpain-7	NM_001135108.2
B13G0711	4.31	1.92	SYNE2	Nesprin-2	NM_001002321.1
B7G0525	4.31	11.84	RBM33	RNA-binding protein 33	XM_021472007.2
A19G0728	4.31	5.29	NAXE	NAD(P)H-hydrate epimerase	
B5G0243	4.31	3.22	DGUOK	Deoxyguanosine kinase, mitochondrial	NM_001100091.1
B1G0615	4.31	4.87	ING2	Inhibitor of growth protein 2	NM_001002448.1
A10G0208	4.31	4.14	KDM2B	Lysine-specific demethylase 2B	NM_001423548.1
A1G0851	4.31	3.60	CHTF18	Chromosome transmission fidelity protein 18 homolog	NM_001110102.3
A2G0166	4.30	9.77	FZR1	Fizzy-related protein homolog	XM_005163252.5
A17G0103	4.30	7.59	SLC4A1AP	Kanadaptin	XM_685743.10
A10G0680	4.30	0.36			
B6G0138	4.30	4.16	BIN1	Myc box-dependent-interacting protein 1	NM_001004602.3
B6G0517	4.30	9.53	BIVM	Basic immunoglobulin-like variable motif-containing protein	
A6G0813	4.30	11.02	STAU1	Double-stranded RNA-binding protein Staufen homolog 1	XM_005168759.5
B5G0571	4.30	13.70	NAAA	N-acylethanolamine-hydrolyzing acid amidase	
A18G0198	4.30	5.58	CENPN	Centromere protein N	NM_001002341.1
B18G0425	4.30	11.26	FAM174B	Membrane protein FAM174B	NM_001103135.1
A9G0679	4.30	5.61	ING1	Inhibitor of growth protein 1	NM_001430945.1
B1G1050	4.30	3.61	CD22	B-cell receptor CD22	XM_068220962.1
A5G0523	4.30	5.50	UBASH3B	Ubiquitin-associated and SH3 domain-containing protein B	XM_001923850.7
A15G0257	4.30	1.23	ARL4A	ADP-ribosylation factor-like protein 4A	NM_200860.1
B19G0596	4.30	0.80	ANGPT2	Angiopoietin-2	NM_131814.1
A23G0517	4.30	3.53	LMBR1L	Limb region 1 homolog-like protein	NM_198874.1
A22G0711	4.30	5.61	TRAF5	TNF receptor-associated factor 5	XM_021469653.2
A20G0459	4.30	4.70	ACOT1	Acyl-coenzyme A thioesterase 1	XM_685444.5

B1G0914	4.30	4.70	INPP4B	Type II inositol 3,4-bisphosphate 4-phosphatase	XM_005160151.5
A15G0138	4.30	2.54	SLC36A4	Proton-coupled amino acid transporter 4	XM_005157799.5
B20G0687	4.30	3.38	CAD	CAD protein	XM_009294886.4
B19G0617	4.29	3.93	ABITRAM	Protein Abitram	NM_001044929.3
B21G0424	4.29	1.68	SDF2	Stromal cell-derived factor 2	NM_200039.1
B21G0480	4.29	2.44	ALG8	Probable dolichyl pyrophosphate Glc1Man9GlcNAc2 alpha-1,3-glucosyltransferase	NM_001017647.1
A3G0538	4.29	1.02	CLEC4M	C-type lectin domain family 4 member M	
A16G0170	4.29	8.05	ROHU_024850	A0A498MUW1_LABRO Citrate hydro-lyase	
B1G0343	4.29	36.80	CPE	Carboxypeptidase E	NM_214810.2
B10G0757	4.29	0.86	BCL7A	lymphoma 7 protein family member A	
A21G0802	4.29	2.94	ARHGAP26	Rho GTPase-activating protein 26	
B22G0674	4.29	1.78	FREM1	FRAS1-related extracellular matrix protein 1	XM_009296245.4
A19G0813	4.29	5.14	CTK2	Carboxy-terminal kinesin 2	NM_001044954.2
B6G0605	4.29	6.76	HES1	Transcription factor HES-1	NM_131079.2
A15G0774	4.29	1.48	DPX16_14616	A0A3N0YXQ6_ANAGA Uncharacterized protein	
B8G0215	4.29	4.32	FHIP2B	FHF complex subunit HOOK interacting protein 2B	NM_001077766.1
B19G0120	4.29	4.03	ERF	ETS domain-containing transcription factor ERF	XM_005159364.5
B7G0185	4.29	4.55	TMEM41B	Transmembrane protein 41B	NM_001079987.2
B12G0339	4.29	9.30	TAP2	Antigen peptide transporter 2	XM_005156299.5
B8G0725	4.29	5.64	JOCKEY\POL	RNA-directed DNA polymerase from mobile element jockey	XM_068213228.1
B5G0552	4.29	2.06	SLC2A8	Solute carrier family 2, facilitated glucose transporter member 8	NM_212798.1
B8G1038	4.29	11.03	PLPP1	Phospholipid phosphatase 1	XM_001334553.9
A10G0319	4.29	5.39	RSF1	Remodeling and spacing factor 1	XM_068223991.1
A11G0426	4.29	10.30	NMUR2	Neuromedin-U receptor 2	NM_001007295.1
A16G0138	4.28	6.12	LOC107726691	A0A673GT54_9TELE Uncharacterized LOC107726651	XM_009292662.4
B9G0164	4.28	14.59			NM_001005956.2
B3G0606	4.28	7.49	FLII	Protein flightless-1 homolog	NM_001257146.1
B4G0774	4.28	2.58	G5714_004287	A0A7J6D4T3_9TELE EF_TS domain-containing protein	
A10G0598	4.28	11.74	PARP14	Protein mono-ADP-ribosyltransferase PARP14	XM_021472253.2
B12G0748	4.28	5.72	ADPRM	CDP-alcohol diphosphatase	
A13G0046	4.28	2.32	LYST	Lysosomal-trafficking regulator	XM_068212982.1
B20G0066	4.28	6.18	MYO6	Unconventional myosin-VI	NM_001004111.2
A25G0326	4.28	5.21	BTBD10	POZ domain-containing protein 10	XR_223121.5
B16G0988	4.28	3.12	TCAIM	T-cell activation inhibitor, mitochondrial	XM_682578.7
A5G0036	4.28	2.65	GNG10	G(O) subunit gamma-10	
B5G0588	4.28	3.94	SETBP1	SET-binding protein	XM_005165675.5
A17G0872	4.28	14.96	INF2	Inverted formin-2	XM_021467252.2
A24G0181	4.28	4.32	PCMTD1	Protein-L-isoaspartate O-methyltransferase domain-containing protein 1	NM_001030067.3

B4G0334	4.28	8.40	IRAK3	Interleukin-1 receptor-associated kinase 3	XM_017355295.3
B2G0202	4.28	2.46	LPAR6	Lysophosphatidic acid receptor 6	
B20G0692	4.28	7.44	RCAN2	Calcipressin-2	NM_001320228.1
A8G0667	4.28	6.33	NCOR2	Nuclear receptor corepressor 2	XM_021478272.2
A7G0405	4.28	5.36	PHKA1	Phosphorylase b kinase regulatory subunit alpha, skeletal muscle isoform	XM_009303454.4
B11G0751	4.28	3.53	STRN	Striatin	XM_009305758.4
A6G0791	4.27	4.78	RYBPB	RING1 and YY1-binding protein B	NM_001012373.2
A18G0409	4.27	4.23	TCB1	Transposable element Tcb1 transposase	XM_021470211.2
B11G0542	4.27	3.02			XM_021472498.2
B4G0090	4.27	3.03	CSRP2	Cysteine and glycine-rich protein 2	NM_200897.2
A9G0184	4.27	4.39	ASNSD1	Asparagine synthetase domain-containing protein 1	NM_200095.2
B19G0445	4.27	7.95	MED30	Mediator of RNA polymerase II transcription subunit 30	
A8G0930	4.27	5.11	ACOT7	Cytosolic acyl coenzyme A thioester hydrolase	NM_001430975.1
A8G0269	4.27	9.73	SOAT1	Sterol O-acyltransferase 1	NM_001123272.1
B21G0714	4.27	7.25	UBAP1	Ubiquitin-associated protein 1	NM_001328344.1
B4G0420	4.27	11.66	SINHCAF	SIN3-HDAC complex-associated factor	NM_001025531.1
A5G1126	4.27	3.43	SSNA1	Microtubule nucleation factor SSNA1	NM_001144793.1
A12G0439	4.27	6.45	MRTFA	Myocardin-related transcription factor A	NM_001201558.1
A20G0680	4.27	2.80	RNF166	E3 ubiquitin-protein ligase RNF166	
A10G0597	4.27	15.57	PARP14	Protein mono-ADP-ribosyltransferase PARP14	XM_021472253.2
B24G0159	4.27	2.07	RHBDL1	Rhomboid-related protein 1	XM_017353796.3
A9G0313	4.27	2.01	ANKRD50	Ankyrin repeat domain-containing protein 50	XM_017357830.3
A11G0451	4.27	4.93	VEGFD	Vascular endothelial growth factor D	NM_001327765.1
A18G0412	4.27	4.36	RANBP10	Ran-binding protein 10	XM_005159142.5
A17G0801	4.26	6.84	PLAAT2	Phospholipase A and acyltransferase 2	
A24G0632	4.26	4.26	WDR37	WD repeat-containing protein 37	NM_001168264.1
A16G0223	4.26	1.78	LOC107737834	A0A673MQR7_9TELE Uncharacterized LOC107737834	XM_001335250.8
A3G0130	4.26	5.72	ZC3H7B	Zinc finger CCCH domain-containing protein 7B	NM_001089332.2
A6G0199	4.26	8.02	CYP20A1	Cytochrome P450 20A1	NM_213332.2
A23G0221	4.26	1.14	MAPK14B	Mitogen-activated protein kinase 14B	NM_001111167.1
B5G0993	4.26	0.89	ENTPD2	Ectonucleoside triphosphate diphosphohydrolase 2	XM_682630.7
A5G0251	4.26	2.82	POLI	DNA polymerase iota	NM_001017834.1
A4G0611	4.26	5.50	RINT1	RAD50-interacting protein 1	NM_001045003.2
B10G0498	4.26	9.49	BCL7BA	lymphoma 7 protein family member B-A	NM_213165.1
B10G0795	4.26	9.79	VDAC2	Voltage-dependent anion-selective channel protein 2	NM_200217.2
B18G0113	4.26	5.01	BCL9L	lymphoma 9-like protein	XM_005159238.5

B8G0748	4.26	2.05	CENPH	Centromere protein H	NM_001113609.1
B5G0418	4.26	7.13	G5714_006100	A0A7J6D2X1_9TELE Uncharacterized protein	
B4G0694	4.26	0.00	NLRC3	Protein NLRC3	XM_068222163.1
B13G0736	4.26	2.01	CRTAC1	Cartilage acidic protein 1	XM_021472628.2
A15G0527	4.26	3.53	LOC107657543	A0A671L8T6_9TELE Interleukin	
B4G0009	4.26	9.59	DYNLT1	Dynein light chain Tctex-type 1	NM_001135783.1
A4G0820	4.25	4.50	LMF2	Lipase maturation factor 2	NM_001142563.1
B4G0073	4.25	4.14	FAR1	Fatty acyl-CoA reductase 1	XM_005164445.5
B25G0276	4.25	4.61	LAMB1	Laminin subunit beta-1	NM_173275.1
A5G0100	4.25	0.95	KIAA1671	Uncharacterized protein KIAA1671	NM_001267589.1
A22G0341	4.25	3.35	TRMT13	tRNA:m(4)X modification enzyme TRM13 homolog	NM_001014309.2
B18G0062	4.25	5.06	FOXA1	Forkhead box protein A1	NM_131299.1
A10G0720	4.25	6.42	ITGA1	Integrin alpha-1	XM_694393.8
B5G1066	4.25	3.13	FAM151B	Protein FAM151B	NM_001431116.1
B3G0463	4.25	1.97	DSN1	Kinetochore-associated protein DSN1 homolog	
A5G0238	4.25	11.72	RTASE	Probable RNA-directed DNA polymerase from transposon BS	XR_011011165.1
B3G0592	4.25	4.96	SMURF1	E3 ubiquitin-protein ligase SMURF1	XM_005164115.5
B12G0215	4.25	14.59	C1QTNF1	Complement C1q tumor necrosis factor-related protein 1	NM_001017875.2
B11G0802	4.25	2.07			NM_131467.1
A14G0314	4.25	5.93	CTSO	Cathepsin O	XM_690625.9
B6G0682	4.25	7.36	SUMF1	Formylglycine-generating enzyme	XM_005166041.5
B6G0232	4.25	5.64	CHRNA1	Acetylcholine receptor subunit alpha	NM_131445.1
A8G0776	4.24	1.33	NEURL3	E3 ubiquitin-protein ligase NEURL3	
B11G0662	4.24	7.68	VAMP3	Vesicle-associated membrane protein 3	NM_201012.1
A6G0066	4.24	5.56	ACVR1B	Activin receptor type-1B	XM_682541.9
B25G0023	4.24	4.84	NUP160	Nuclear pore complex protein Nup160	NM_001423844.1
B5G0902	4.24	4.21	NDOR1	NADPH-dependent diflavin oxidoreductase 1	NM_200648.1
B7G0693	4.24	4.91	SLC39A13	Zinc transporter ZIP13	NM_001005306.3
B6G0627	4.24	4.89	TUBGCP5	Gamma-tubulin complex component 5	XM_021476871.2
A11G0067	4.24	2.24	LOC113110726	A0A6P6QB89_CARAU synapse differentiation-inducing gene protein 1-like	XM_005172805.5
B19G0156	4.24	4.95	CDK12	Cyclin-dependent kinase 12	XM_003200531.6
A15G0478	4.24	5.84	CBL	E3 ubiquitin-protein ligase CBL	XM_005157579.5
B8G0889	4.24	8.54	OTUD5A	OTU domain-containing protein 5-A	NM_001075110.1
B25G0885	4.24	5.88	DDB_G0292642	Uncharacterized protein DDB_G0292642	
A12G0644	4.23	3.92	NFU_G_1_000828	A0A1A8E8L1_9TELE Uncharacterized protein	NM_001327904.1
A22G0318	4.23	220.44	TPM3	Tropomyosin alpha-3 chain	NM_001024467.1
B10G0766	4.23	3.92	CDK2AP1	Cyclin-dependent kinase 2-associated protein 1	
A3G1320	4.23	1.16	TRIM16	Tripartite motif-containing protein 16	XM_690458.9
B5G0034	4.23	3.54	HAUS4	HAUS augmin-like complex subunit 4	NM_001002425.1

B6G0689	4.23	0.56	CHCHD4-B	Mitochondrial intermembrane space import and assembly protein 40-B	
B2G1012	4.23	3.68	DUSP28	Dual specificity phosphatase 28	
A15G0253	4.23	1.84	MEOX2	Homeobox protein MOX-2	XM_017351207.3
B2G0080	4.23	23.34	TPM1	Tropomyosin alpha-1 chain	XM_017357672.3
B5G0848	4.23	3.84	TTC33	Tetratricopeptide repeat protein 33	NM_001002722.1
A20G0640	4.23	2.81	ZNF513	Zinc finger protein 513	NM_001309467.1
A17G0483	4.23	3.59			
A6G0711	4.23	2.61	C1ORF159	Uncharacterized protein C1orf159	NM_001326426.1
A7G0826	4.23	12.41	PHF23B	PHD finger protein 23B	NM_199844.1
B25G0516	4.23	6.31	MMP15	Matrix metalloproteinase-15	XM_001924007.8
A8G0095	4.23	2.82	FUT9	4-galactosyl-N-acetylglucosaminide 3-alpha-L-fucosyltransferase 9	NM_001007454.2
A15G0156	4.23	4.94	LPAR5	Lysophosphatidic acid receptor 5	
B8G0322	4.23	4.43	RNF34	E3 ubiquitin-protein ligase RNF34	XM_005172464.3
A16G0107	4.23	0.65	HLA-DOA	HLA class II histocompatibility antigen, DO alpha chain	
A8G0778	4.23	12.98	INPP5A	Inositol polyphosphate-5-phosphatase A	NM_001037569.2
B6G0938	4.22	0.27			
A4G0618	4.22	14.29	PLXNC1	Plexin-C1	XM_068220763.1
B17G0005	4.22	4.03	WDR20	WD repeat-containing protein 20	XM_021467251.2
A8G0102	4.22	14.83	MTMR3	Myotubularin-related protein 3	NM_200864.3
B25G0424	4.22	4.16	EDC3	Enhancer of mRNA-decapping protein 3	NM_001018126.2
A19G0547	4.22	1.31	HOXA3A	Homeobox protein Hox-A3a	NM_131534.2
A1G0061	4.22	3.44	NLRC3	Protein NLRC3	XM_068220885.1
B21G0739	4.22	8.26	NEDD4L	E3 ubiquitin-protein ligase NEDD4-like	XM_068216002.1
B23G0783	4.22	10.29	TENT5A	Terminal nucleotidyltransferase 5A	NM_001103125.1
B12G0235	4.22	5.04	DNMBP	Dynamin-binding protein	NM_001424046.1
A15G0130	4.22	2.59	A0A673GBN9	A0A673GBN9_9TELE Uncharacterized protein	
B25G0809	4.22	4.99	GOLT1B	Vesicle transport protein GOT1B	NM_001031671.2
A18G0638	4.22	4.55	SEPHS1	Selenide, water dikinase 1	NM_199701.2
A16G0199	4.22	2.71	MRPL32	39S ribosomal protein L32, mitochondrial	
A5G0753	4.22	16.75	RASGEF1BA	Ras-GEF domain-containing family member 1B-A	NM_199829.2
A11G0418	4.22	17.55	FAM120A	Constitutive coactivator of PPAR-gamma-like protein 1	NM_001327754.1
A6G0411	4.22	11.40	ZNF207	BUB3-interacting and GLEBS motif-containing protein ZNF207	XM_009302342.4
A17G0638	4.22	5.00	GCH1	GTP cyclohydrolase 1	NM_001136255.2
B17G0417	4.22	7.94	XPO1	Exportin-1	XM_009293053.4
B5G0198	4.22	10.47	YPEL1	Protein yippee-like 1	XM_005165045.5
A18G0686	4.22	4.65	WASHC1	WASH complex subunit 1	NM_001110450.1
A15G0571	4.22	0.72	NFKBIZ	NF-kappa-B inhibitor zeta	NM_001020720.1
A15G0186	4.22	2.62	C3AR1	C3a anaphylatoxin chemotactic receptor	XM_005173403.5

B14G0732	4.22	2.58	ERGIC1	Endoplasmic reticulum-Golgi intermediate compartment protein 1	NM_001029962.2
B24G0068	4.22	2.65	IGLC3	Ig lambda-3 chain C region	XM_068217596.1
A6G0357	4.21	0.87	MS4A4A	Membrane-spanning 4-domains subfamily A member 4A	
B13G0831	4.21	18.97	TMEM87A	Transmembrane protein 87A	NM_199277.2
B6G0588	4.21	3.08	IL12RB2	Interleukin-12 receptor subunit beta-2	XM_021476745.2
A16G0594	4.21	8.35	ZNF576	Zinc finger protein 576	NM_001114425.2
B12G0549	4.21	4.47	HEXIM1	Protein HEXIM1	
B11G0786	4.21	7.73			NM_001040370.1
B14G0396	4.21	12.26	GANAB	Neutral alpha-glucosidase AB	XM_009291260.4
A5G0365	4.21	11.23	ACSL4	Long-chain-fatty-acid--CoA ligase 4	NM_001099739.1
A22G0622	4.21	20.04	SMC3	Structural maintenance of chromosomes protein 3	NM_214689.1
A2G0530	4.21	4.37	NCK1	Cytoplasmic protein NCK1	XM_687914.7
B4G0570	4.21	4.24	TAF3	Transcription initiation factor TFIID subunit 3	NM_001042744.2
A19G0214	4.21	4.27	MGAT1	Alpha-1,3-mannosyl-glycoprotein 2-beta-N-acetylglucosaminyltransferase	NM_001079971.2
B17G0518	4.21	18.42	CATL	Cathepsin L	NM_001002368.1
B22G0461	4.21	1.94	LOC113080859	A0A6P6NKR0_CARAU zinc finger protein 729-like	XM_021469564.2
A22G0623	4.20	12.93	DUSP5	Dual specificity protein phosphatase 5	NM_212565.1
B1G0323	4.20	4.31	SMC2	Structural maintenance of chromosomes protein 2	NM_199542.2
B15G0758	4.20	7.64	KMT2B	Histone-lysine N-methyltransferase 2B	XM_009291876.4
A19G0384	4.20	13.25	TFPI2	Tissue factor pathway inhibitor 2	NM_001040096.2
A14G0180	4.20	7.53	FIBPB	Acidic fibroblast growth factor intracellular-binding protein B	NM_200409.1
B1G1046	4.20	4.15	LOC113095506	A0A6P6P7S8_CARAU Nuclear apoptosis-inducing factor 1	
A6G0882	4.20	13.21	OSGIN2	Oxidative stress-induced growth inhibitor 2	NM_200186.1
B13G0351	4.20	11.57	HABP2	Hyaluronan-binding protein 2	
B18G0167	4.20	23.51	ACADL	Long-chain specific acyl-CoA dehydrogenase, mitochondrial	NM_201181.2
A3G0139	4.20	48.95	MYH9	Myosin-9	XM_001920063.8
A16G0873	4.20	5.74	SPAG9	C-Jun-amino-terminal kinase-interacting protein 4	XM_001336948.8
A21G0315	4.20	17.78	GSN	Gelsolin	NM_001012312.2
B10G0865	4.20	7.26	SMAD4	Mothers against decapentaplegic homolog 4	NM_001122700.2
B14G0558	4.20	2.32	RD3	Protein RD3	NM_001082979.1
A2G0374	4.20	5.64	XRN1	5'-3' exoribonuclease 1	XM_005163317.5
B15G0215	4.20	1.68	SLC25A15	Mitochondrial ornithine transporter 1	NM_001080638.1
A20G0083	4.20	5.28	MGAT2	Alpha-1,6-mannosyl-glycoprotein 2-beta-N-acetylglucosaminyltransferase	XM_003200772.6
A2G0458	4.20	0.80	CYP7A1	Cytochrome P450 7A1	NM_201173.2
A23G0864	4.20	22.77	CFI	Complement factor I	
B10G0516	4.20	6.65	CFAP298	Cilia- and flagella-associated protein 298	NM_200088.1
A7G0871	4.20	2.09	BAD	Bcl2-associated agonist of cell death	

A24G0633	4.20	6.10	IDI1	Isopentenyl-diphosphate Delta-isomerase 1	NM_001025475.2
A24G0288	4.20	3.03	FNDC3B	Fibronectin type III domain-containing protein 3B	XM_002666672.6
B14G0194	4.20	2.37	HS6ST2	Heparan-sulfate 6-O-sulfotransferase 2	NM_194421.2
B11G0011	4.20	3.43	AVIL	Advillin	
A3G1110	4.20	2.88	HEXD	Hexosaminidase D	XM_009299336.4
A18G0612	4.20	10.64	FGD4	FYVE, RhoGEF and PH domain-containing protein 4	NM_001423694.1
B5G0644	4.20	8.90	IQGAP1	Ras GTPase-activating-like protein IQGAP1	XM_005165243.5
B22G0950	4.19	2.93	G5714_004123	A0A7J6DCN4_9TELE Uncharacterized protein	XM_068218685.1
B7G0401	4.19	6.00	TP53BP1	TP53-binding protein 1	XM_017356868.2
B11G0573	4.19	3.13			XM_009306242.4
B12G0126	4.19	0.07	TECTA	Alpha-tectorin	XM_068224822.1
B17G0640	4.19	4.89	KIDINS220B	Kinase D-interacting substrate of 220 kDa B	XM_005158747.5
B19G0501	4.19	4.07	NKAIN1	potassium-transporting ATPase subunit beta-1-interacting protein 1	NM_200545.1
A11G0721	4.19	24.20	EPB41L1	Band 4.1-like protein 1	XM_021479916.2
B9G0212	4.19	2.05	NEMP2	Nuclear envelope integral membrane protein 2	NM_001423789.1
A4G0303	4.19	1.47	LOC113072931	A0A6P6MY53_CARAU uncharacterized protein LOC113072931	
B11G0551	4.19	3.41	DIAPH3	Protein diaphanous homolog 3	XM_005172906.4
A16G0759	4.19	5.83	LENG9	Leukocyte receptor cluster member 9	XM_005157900.5
B16G0318	4.19	1.77	ENY2	Transcription and mRNA export factor ENY2	NM_001002427.2
A20G0315	4.19	6.51	LBR	Delta(14)-sterol reductase LBR	XM_021468371.2
A17G0744	4.19	1.77	FZD3	Frizzled-3	NM_001080601.1
A3G0686	4.19	7.37	TBC1D10B	TBC1 domain family member 10B	NM_001365256.1
A23G0680	4.19	0.96	HOXC10A	Homeobox protein Hox-C10a	XM_017353498.3
B16G0867	4.19	1.47	TMEM276	Transmembrane protein 276	
A8G0209	4.19	5.16	TJP2	Tight junction protein ZO-2	XM_068222921.1
A20G0381	4.19	6.52	FAM102B	Protein FAM102B	NM_001044844.1
B1G0146	4.19	2.65	PTCD1	Pentatricopeptide repeat-containing protein 1, mitochondrial	XM_021471105.2
B8G0110	4.19	3.45	AAK1	AP2-associated protein kinase 1	XM_068223234.1
A20G0639	4.19	12.04	SNX17	Sorting nexin-17	XM_009294624.3
B3G1052	4.19	2.99	POLD1	DNA polymerase delta catalytic subunit	NM_001039810.2
A11G0718	4.19	4.08	RTASE	Probable RNA-directed DNA polymerase from transposon BS	NM_212914.1
B25G0502	4.19	3.21	TMTC3	Protein O-mannosyl-transferase TMTC3	XM_001922451.8
B20G0831	4.19	8.99	DTNB	Dystrobrevin beta	XM_068215384.1
B18G0765	4.18	1.26	P2RY3	P2Y purinoceptor 3	XM_689367.9
A7G1276	4.18	1.15	VANGL2	Vang-like protein 2	NM_153674.2
A9G0877	4.18	7.88	IFNAR1A	beta receptor 1a	
B9G0776	4.18	8.58	CLN5	Ceroid-lipofuscinosis neuronal protein 5	XM_009304580.4
A17G0508	4.18	2.16	TTC27	Tetratricopeptide repeat protein 27	NM_173285.2
B4G0775	4.18	2.57	MS4A4A	Membrane-spanning 4-domains subfamily A member 4A	

B19G0444	4.18	6.70	EXT1B	Exostosin-1b	NM_001012369.2
B10G0044	4.18	6.22	HMGCS1	Hydroxymethylglutaryl-CoA synthase, cytoplasmic	XM_005155491.4
A11G0748	4.18	6.43	IGFBP5	Insulin-like growth factor-binding protein 5	NM_001362321.1
A6G0124	4.18	4.98	RIN2	Ras and Rab interactor 2	XM_017356561.3
A2G0696	4.18	4.89	KLHL20	Kelch-like protein 20	XM_005163492.5
B2G0609	4.18	14.32	NRBP2	Nuclear receptor-binding protein 2	XM_001920637.7
A12G0803	4.18	4.18	ZDHHC16B	Palmitoyltransferase ZDHHC16B	XM_009306385.4
B16G0764	4.18	10.33	GSK3A	Glycogen synthase kinase-3 alpha	NM_131390.2
B12G0387	4.18	7.98	RAB18	Ras-related protein Rab-18	NM_206830.1
A20G0781	4.18	4.15	SI:DKEY-29F10.1	Protein C10	NM_212646.2
A24G0159	4.18	1.44	PUS3	39) synthase	NM_200067.1
A6G1025	4.18	6.91			
B2G1168	4.18	6.42	TSC22D2	TSC22 domain family protein 2	
A14G0809	4.18	8.92	TMEM129	E3 ubiquitin-protein ligase TM129	NM_213447.1
A9G0263	4.17	30.58	SLC25A5	ATP translocase 2	NM_001128732.1
A9G0881	4.17	6.43	RTASE	Probable RNA-directed DNA polymerase from transposon BS	
A10G0517	4.17	5.14	A33	Zinc-binding protein A33	NM_200284.1
B7G0817	4.17	4.88	TP53I11	Tumor protein p53-inducible protein 11	NM_001013352.2
A10G0787	4.17	4.51	TRAPPC13	Trafficking protein particle complex subunit 13	XM_005155559.5
A25G0249	4.17	14.68	CKAP5	Cytoskeleton-associated protein 5	NM_001037667.3
A4G0903	4.17	0.61	GALNT8	Probable polypeptide N-acetylgalactosaminyltransferase 8	XM_003198223.6
B25G0534	4.17	13.46	CCND2	S-specific cyclin-D2	NM_001089445.1
A12G0661	4.17	5.57	NDC80	Kinetochore protein NDC80 homolog	NM_001003863.1
A7G1024	4.17	6.24	DDB_G0292642	Uncharacterized protein DDB_G0292642	NM_001386555.1
A6G0708	4.17	7.05	RERE	Arginine-glutamic acid dipeptide repeats protein	XM_009302602.3
A6G0580	4.17	6.28	MPL	Thrombopoietin receptor	NM_001003858.1
A17G0250	4.17	64.55	RSAD2	Radical S-adenosyl methionine domain-containing protein 2	NM_001025556.1
B16G0833	4.17	5.48	FDPS	Farnesyl pyrophosphate synthase	NM_001025471.1
A18G0308	4.17	10.20	OPA3	Optic atrophy 3 protein homolog	NM_001080166.1
B1G0947	4.17	3.41	DCAF15	DDB1- and CUL4-associated factor 15	XM_677708.10
B1G0090	4.17	5.00	LOC113052278	A0A6P6KJQ4_CARAU uncharacterized protein LOC113048991 isoform X1	
A16G0336	4.17	9.94	CTPS1	CTP synthase 1	NM_001111247.1
B18G0467	4.17	5.64	KMT5B	Histone-lysine N-methyltransferase KMT5B	NM_001007337.2
A24G0686	4.17	6.61	COL15A1	Collagen alpha-1(XV) chain	
B21G0547	4.17	2.65	SDHAF1	Succinate dehydrogenase assembly factor 1, mitochondrial	
A18G0173	4.17	7.31	MAPK6	Mitogen-activated protein kinase 6	NM_001045552.1
B8G0321	4.17	8.11	KDM2B	Lysine-specific demethylase 2B	NM_213167.2

A10G0170	4.16	7.35	MEF2C	Myocyte-specific enhancer factor 2C	XR_011016938.1
A17G0291	4.16	2.99	GCFC2	Intron Large complex component GCFC2	NM_001110107.2
B21G0682	4.16	11.94	SLC31A2	Probable low affinity copper uptake protein 2	
A5G0790	4.16	2.83	PIGL	N-acetylglucosaminyl-phosphatidylinositol de-N-acetylase	NM_001256655.1
B11G0042	4.16	5.68	FAM217B	Protein FAM217B	NM_214752.1
B9G0378	4.16	4.32	CLYBL	Citramalyl-CoA lyase, mitochondrial	NM_001037416.2
B20G0547	4.16	20.09	FOXO3	Forkhead box protein O3	NM_131085.1
B8G0436	4.16	4.67	ERCC6L2	DNA excision repair protein ERCC-6-like 2	NM_001327769.1
A17G0077	4.16	1.87	MOCS3	Adenylyltransferase and sulfurtransferase MOCS3	XM_068214335.1
B7G0531	4.16	8.04	UBE3C	Ubiquitin-protein ligase E3C	NM_200473.2
B8G0995	4.16	3.52	CV039	UPF0545 protein C22orf39 homolog	NM_001123264.1
A21G0840	4.16	7.03	ANKRD46	Ankyrin repeat domain-containing protein 46	XM_002666073.7
B19G0229	4.16	10.13	HAX1	HCLS1-associated protein X-1	NM_001002337.1
A10G0791	4.16	5.06	SH3BP2	SH3 domain-binding protein 2	XM_005155583.5
B3G0886	4.16	0.32	FRIM	Ferritin, middle subunit	NM_001109854.1
B19G0872	4.16	4.24	ARID1A	AT-rich interactive domain-containing protein 1A	XM_021468300.2
B7G0321	4.16	3.39	GPS2	G protein pathway suppressor 2	
A25G0818	4.16	10.49	SRPK2	SRSF protein kinase 2	XM_021470561.2
A24G0014	4.16	7.31	PPP4R1	threonine-protein phosphatase 4 regulatory subunit 1	NM_200260.1
B16G0078	4.16	8.30	UBR5	E3 ubiquitin-protein ligase UBR5	XM_005158440.5
A5G0611	4.16	6.59	MED22	Mediator of RNA polymerase II transcription subunit 22	XM_005171831.3
B20G0797	4.16	4.92	STX7	Syntaxin-7	XM_021474444.2
B9G0387	4.16	14.59	MORC3	MORC family CW-type zinc finger protein 3	NM_001089366.1
B15G0510	4.16	8.71	SMTNL2	Smoothelin-like protein 2	NM_212805.2
B17G0516	4.16	4.67	YTHDF2	YTH domain-containing family protein 2	XM_005158763.5
B3G0879	4.16	0.32	FRIM	Ferritin, middle subunit	NM_001109854.1
A5G0256	4.16	8.23	FAM214B	Protein FAM214B	NM_001433130.1
A5G1307	4.16	6.45	MEPCE	7SK snRNA methylphosphate capping enzyme	NM_001128529.1
B8G1024	4.16	4.65	CDC14A	Dual specificity protein phosphatase CDC14A	XM_005166671.5
B11G0292	4.16	3.77	KCNMB2	Calcium-activated potassium channel subunit beta-2	NM_001030072.1
A22G0244	4.16	4.88	ARHGEF18	Rho guanine nucleotide exchange factor 18	XM_068216437.1
B11G0167	4.16	10.46	ABHD8	Protein ABHD8	
B21G0461	4.16	4.58	DIXDC1	Dixin	XM_005161265.5
B21G0158	4.15	1.21	POL	Retrovirus-related Pol polyprotein from transposon 297	
A4G0844	4.15	4.81	FBXL14	LRR-repeat protein 14	NM_001015043.2
B14G0686	4.15	6.34	LOC113113594	A0A6P6QQL5_CARAU uncharacterized protein LOC113113594 isoform X2	NM_001198864.1
B23G0101	4.15	5.80	PLEKHG1	Pleckstrin homology domain-containing family G member 1	XM_021469870.2

B22G0442	4.15	1.19	LOC113116518	A0A6P6R433_CARAU zinc finger protein 271-like	NM_001012485.1
A13G0274	4.15	4.73	TMEM39B	Transmembrane protein 39B	NM_001305568.1
B20G0944	4.15	13.95	CIPC	CLOCK-interacting pacemaker	
A16G0315	4.15	7.33	ALDH5A1	Succinate-semialdehyde dehydrogenase, mitochondrial	XM_009292538.4
A16G0586	4.15	5.26	LOC113059097	A0A6P6LEX5_CARAU uncharacterized protein LOC113059097 isoform X1	XM_001340637.8
A6G0589	4.15	7.09	SLC35D2	GDP-mannose transporter (Fragment)	NM_001003877.2
A11G0542	4.15	2.47	TEX264	Testis-expressed protein 264	
A3G1081	4.15	0.93	GCAT	2-amino-3-ketobutyrate coenzyme A ligase, mitochondrial	NM_001172554.1
A9G0855	4.15	1.05	POL	Retrovirus-related Pol polyprotein from transposon opus	
B5G1034	4.15	5.15	LOXL2B	Lysyl oxidase homolog 2B	NM_001080626.1
A24G0039	4.15	4.03	EXOG	Nuclease EXOG, mitochondrial	
B12G0797	4.15	6.57	MAPK8B	Mitogen-activated protein kinase 8B	XM_017358485.3
B12G0474	4.15	7.64	UBALD1	UBA-like domain-containing protein 1	XM_009306668.4
B20G0201	4.15	5.18	BRMS1LA	Breast cancer metastasis-suppressor 1-like protein-A	XM_003200688.6
A16G0655	4.15	3.23	FLAD1	FAD synthase	XM_005169746.5
A3G0921	4.15	2.32	PHOSPHO1	Probable phosphatase phospho1	
B24G0305	4.15	5.65	LOC107757706	A0A673IHB8_9TELE Eotaxin-like	
B24G0128	4.15	5.00	IGKV1-39	Immunoglobulin kappa variable 1-39	NM_001045408.1
B19G0183	4.15	7.42	ZC3H12A	Endoribonuclease ZC3H12A	NM_001171029.1
B11G0122	4.15	3.29	KIF21B	Kinesin-like protein KIF21B	NM_001003752.2
A6G0049	4.15	5.63	EIF4G1	Eukaryotic translation initiation factor 4 gamma 1	NM_001386812.1
B15G0085	4.15	2.67	GPR171	G-protein coupled receptor 171	XM_068213672.1
A8G0161	4.15	2.02	CCDC120	Coiled-coil domain-containing protein 120	XM_009303768.4
B24G0125	4.15	5.00	IGKV1-39	Immunoglobulin kappa variable 1-39	NM_001045408.1
A20G0176	4.14	0.35	FERMT1	Fermitin family homolog 1	XM_002665785.7
B6G0476	4.14	7.79	ATG4B	Cysteine protease ATG4B	NM_001089352.1
A14G0393	4.14	7.58	SCYL1	N-terminal kinase-like protein	XM_001332472.7
B16G0763	4.14	3.25	ZNF574	Zinc finger protein 574	XM_689335.10
A5G0547	4.14	1.02	TRIM16	Tripartite motif-containing protein 16	NM_001326487.1
A1G0217	4.14	2.02	TRIOBP	TRIO and F-actin-binding protein	XM_005160161.5
A22G0232	4.14	4.97	RAD54L2	Helicase ARIP4	XM_005174006.5
A18G0363	4.14	5.50	ZNF592	Zinc finger protein 592	XM_005159296.5
B15G0922	4.14	7.22	SAMD4B	Protein Smaug homolog 2	XM_021466521.2
B5G0790	4.14	8.21	REQ	Zinc finger protein ubi-d4	XM_005165302.5
B3G0415	4.14	2.24	QTRT1	Queuine tRNA-ribosyltransferase catalytic subunit 1	NM_201010.1
B1G0990	4.14	8.72	CC2D1A	Coiled-coil and C2 domain-containing protein 1A	XM_017356645.3
A5G1252	4.14	1.85	TMEM250	Transmembrane protein 250	XR_011015641.1
B5G1211	4.14	2.37	BTN1A1	Butyrophilin subfamily 1 member A1	XM_068221425.1

A1G0559	4.14	4.16	SMAD1	Mothers against decapentaplegic homolog 1	NM_131356.2
B8G0049	4.14	3.47	ACTR8	Actin-related protein 8	NM_001001400.1
A9G0619	4.14	3.42	RAPH1	Ras-associated and pleckstrin homology domains-containing protein 1	NM_001366727.1
B1G0577	4.14	4.31	MMAA	Methylmalonic aciduria type A protein, mitochondrial	XM_017355297.2
B21G0001	4.14	1.91	PHYPO_G00066290	A0A5N5M4I3_PANHP Uncharacterized protein	
B21G0206	4.14	3.73	ARID5B	AT-rich interactive domain-containing protein 5B	NM_205565.2
A20G0813	4.14	2.64	CNK3/IPCEF1	IPCEF1 PE=1 SV=1	NM_001076758.1
B12G0670	4.13	3.90	POLR3D	DNA-directed RNA polymerase III subunit RPC4	NM_001109828.1
A6G0008	4.13	8.14	LDLR	Low-density lipoprotein receptor	XM_017356726.2
B10G0440	4.13	6.76	CBLB	E3 ubiquitin-protein ligase CBL-B	XM_001921926.8
B13G0085	4.13	12.21	OLIG3	Oligodendrocyte transcription factor 3	NM_178303.3
A7G0266	4.13	3.31	SENP8	Sentrin-specific protease 8	NM_001077165.1
B4G0460	4.13	6.84	DNAJB9	DnaJ homolog subfamily B member 9	NM_001024393.1
A8G0679	4.13	8.41	DOK2	Docking protein 2	XM_001341478.9
A18G0228	4.13	6.23	MVD	Diphosphomevalonate decarboxylase	NM_001007422.1
B22G1024	4.13	4.83	NLRC3	NLR family CARD domain-containing protein 3	XM_068213794.1
B8G0403	4.13	9.47	CREB3L3	Cyclic AMP-responsive element-binding protein 3-like protein 3	XM_005167154.4
B7G0120	4.13	2.72	SEC24D	Protein transport protein Sec24D	NM_001177932.2
B16G0719	4.13	4.14	CA043	Protein C1orf43 homolog	NM_001037112.2
B9G0007	4.13	11.87	RAB3GAP1	Rab3 GTPase-activating protein catalytic subunit	NM_001077788.2
B20G0907	4.13	1.79	PSMG4	Proteasome assembly chaperone 4	NM_001386356.1
B10G0348	4.13	47.27	SLC43A2	Large neutral amino acids transporter small subunit 4	XM_009305270.4
B8G0977	4.13	7.17	GCN1	eIF-2-alpha kinase activator GCN1	XM_009303726.3
A19G0657	4.13	9.90	HLF	Hepatic leukemia factor	NM_001197060.1
A1G0040	4.13	2.22	TRIP10	Cdc42-interacting protein 4 homolog	
A18G0319	4.13	23.80	CAPN1	Calpain-1 catalytic subunit	XM_005173670.5
A23G0033	4.13	9.05	RAF1	threonine-protein kinase	XM_017353505.2
A16G0307	4.12	5.22	SELENBP1	Methanethiol oxidase	XM_005158207.3
B10G0716	4.12	5.23	CETN3	Centrin-3	NM_001020499.1
A12G0303	4.12	5.52	MRPL10	39S ribosomal protein L10, mitochondrial	NM_001013458.2
B21G0555	4.12	3.13	FTSJ1	guanosine(34)-2'-O)-methyltransferase	NM_001007384.2
B8G0455	4.12	12.29	LOC113107522	A0A6P6PXP8_CARAU uncharacterized protein LOC113107522	
B8G0334	4.12	3.08	HPS4	BLOC-3 complex member HPS4	XM_068222895.1
B11G0493	4.12	5.03			XM_068224163.1
A7G0172	4.12	38.86	TSPAN5	Tetraspanin-5	NM_001006047.2
A15G0128	4.12	4.62	TRIM16	Tripartite motif-containing protein 16	XM_068213621.1

B12G0568	4.12	6.19	KBTBD2	Kelch repeat and BTB domain-containing protein 2	NM_001077160.2
B18G0399	4.12	6.15	ZNF592	Zinc finger protein 592	XM_005159296.5
B8G0188	4.12	1.67	TCF7L1B	Transcription factor 7-like 1-B	NM_131296.2
B5G0903	4.12	13.68	GPR107	Protein GPR107	XM_009301549.4
A2G0034	4.12	1.62	DHX30	ATP-dependent RNA helicase DHX30	XM_005171083.4
B10G0035	4.12	4.08			
A12G0696	4.12	0.96	POL	Retrovirus-related Pol polyprotein from transposon 17.6	
A7G0025	4.12	3.96	TRIM25	ISG15 ligase TRIM25	NM_001002723.2
A10G0005	4.12	9.44			
A17G0090	4.12	4.19	AREL1	Apoptosis-resistant E3 ubiquitin protein ligase 1	XM_002663035.7
A10G0758	4.12	10.26	UGCG	Ceramide glucosyltransferase	NM_001024207.2
B9G0592	4.12	11.41	SENP7	Sentrin-specific protease 7	XM_068223648.1
A20G0071	4.12	5.97	NAPRT	Nicotinate phosphoribosyltransferase	NM_200890.2
B13G0605	4.12	9.31	SOBPA	Sine oculis-binding protein homolog A	XM_005156574.5
A7G1169	4.12	8.11	TLNRD1	Talin rod domain-containing protein 1	XM_005166366.5
B17G0238	4.12	2.13	GPR135	G-protein coupled receptor 135	XM_681149.8
A24G0598	4.11	6.16	THAP1	THAP domain-containing protein 1	
B18G0586	4.11	1.99	PLEKHG7	Pleckstrin homology domain-containing family G member 7	XM_005169952.5
A9G0419	4.11	4.63	DBR1	Lariat debranching enzyme	NM_199653.1
A1G0782	4.11	2.81	SPRY2	Protein sprouty homolog 2	XM_005160285.5
B22G0519	4.11	6.28	ECM2	Extracellular matrix protein 2	XM_681415.7
B24G0290	4.11	1.86	PRMT6	Protein arginine N-methyltransferase 6	NM_001163988.3
A11G0471	4.11	2.80	MAP4K3	Mitogen-activated protein kinase kinase kinase kinase 3	NM_001045307.1
B4G0236	4.11	2.74	CHST11	Carbohydrate sulfotransferase 11	NM_212824.2
A15G0798	4.11	5.02	GPR171	G-protein coupled receptor 171	XM_068213672.1
B3G0616	4.11	2.92	B9D1	B9 domain-containing protein 1	NM_001024373.1
B12G0705	4.11	4.98	ARHGAP27	Rho GTPase-activating protein 27	XM_005156029.5
B15G0248	4.11	4.66	ARHGAP35	Rho GTPase-activating protein 35	XM_001343600.6
A17G0897	4.11	20.02	HEBP2	Heme-binding protein 2	NM_001045401.2
A5G0582	4.11	3.59	TESC	Calcineurin B homologous protein 3	NM_205693.1
B21G0813	4.11	4.14	SHROOM3	Protein Shroom3	NM_001278851.1
A23G0695	4.11	7.55	LOC107697924	A0A671RLP4_9TELE Uncharacterized LOC107697924	
B2G1316	4.11	3.07	ZNF1013	X1WE00_DANRE Zinc finger protein 1013 (Fragment)	XM_068220444.1
B10G0030	4.11	5.46	SH2D4A	SH2 domain-containing protein 4A	XM_005156459.5
A7G1243	4.11	5.07	A0A671XQM7	A0A671XQM7_SPAAU Uncharacterized protein	
A22G0205	4.11	2.30	LOC113064527	A0A6P6M3I6_CARAU zinc finger protein 585A-like	XM_021469564.2
B13G0597	4.11	16.23	G2E3	M phase-specific E3 ubiquitin-protein ligase	NM_001305556.1
B9G0641	4.11	5.54	IGF2BP2	Insulin-like growth factor 2 mRNA-binding protein 2	NM_001114558.2

B7G0695	4.11	6.72	RFESD	Rieske domain-containing protein	XM_005168863.5
B17G0735	4.11	5.62	NAA30	N-alpha-acetyltransferase 30	NM_001136249.2
B21G0845	4.11	10.93	CDK5RAP2	CDK5 regulatory subunit-associated protein 2	NM_001430821.1
A10G0755	4.11	4.63	EXD3	Exonuclease mut-7 homolog	XM_003199307.6
B1G1006	4.11	7.32	PRKACA	cAMP-dependent protein kinase catalytic subunit alpha	NM_001003470.1
B2G0590	4.11	15.71	ATP6V0B	V-type proton ATPase 21 kDa proteolipid subunit c"	NM_199561.2
B7G0722	4.11	4.09	MTMR10	Myotubularin-related protein 10	XM_005168881.5
B2G0002	4.10	2.69	HARBI1	Putative nuclease HARBI1	XM_021468401.2
B14G0784	4.10	0.54	FGF16	Fibroblast growth factor 16	NM_001040407.1
A23G0559	4.10	12.49	PIK3CD	Phosphatidylinositol 4,5-bisphosphate 3-kinase catalytic subunit delta isoform	NM_201199.1
A20G0420	4.10	3.66	ACAT2	Acetyl-CoA acetyltransferase, cytosolic	NM_131370.2
B7G0044	4.10	15.79	TRIM21	E3 ubiquitin-protein ligase TRIM21	
B3G0556	4.10	7.77	ZC3H7A	Zinc finger CCCH domain-containing protein 7A	NM_213434.1
B6G0027	4.10	8.29	C1QTNF1	Complement C1q tumor necrosis factor-related protein 1	
A3G0077	4.10	2.30	NF7O	Nuclear factor 7, ovary	NM_001089399.2
A4G0687	4.10	6.54	SLC26A5	Prestin	NM_201473.1
B15G0051	4.10	3.00	WDR53	WD repeat-containing protein 53	
B7G0824	4.10	4.82	SENP3	Sentrin-specific protease 3	NM_001083842.2
B3G0687	4.10	8.05	ENGASE	Cytosolic endo-beta-N-acetylglucosaminidase	XM_021470912.2
A6G0136	4.10	7.54	ABCC4	ATP-binding cassette sub-family C member 4	NM_001007038.2
B15G0926	4.10	13.19	SIDT2	SID1 transmembrane family member 2	XM_021466515.2
A3G0728	4.10	4.51	ALDH16A1	Aldehyde dehydrogenase family 16 member A1	NM_001079954.2
B5G0140	4.10	4.29	IDUA	Alpha-L-iduronidase	XM_021474008.2
A1G0038	4.10	8.15	VAV1	Proto-oncogene vav	XR_011014280.1
A19G0718	4.10	14.82	PBXIP1	Pre-B-cell leukemia transcription factor-interacting protein 1	XM_005159476.5
A23G0292	4.10	4.23	UBA3	NEDD8-activating enzyme E1 catalytic subunit	
A16G0246	4.10	8.66	ASTE1	Protein asteroid homolog 1	NM_001099430.2
B13G0804	4.10	3.31	S100PBP	S100P-binding protein	XM_005156512.5
A24G0670	4.10	4.97	CDK13	Cyclin-dependent kinase 13	NM_001366829.1
A8G0493	4.10	3.92	GNAI3	Guanine nucleotide-binding protein G(i) subunit alpha-3	NM_001111250.1
B1G0784	4.10	8.18	SPRYD7	SPRY domain-containing protein 7	NM_214776.1
B3G0621	4.10	2.88	CLN3	Battenin	NM_001007306.1
A12G0637	4.10	5.10	PRDM9	Histone-lysine N-methyltransferase PRDM9	NM_200902.1
B7G0782	4.10	5.19	COG4	Conserved oligomeric Golgi complex subunit 4	NM_001039823.1
B4G0721	4.10	0.47	DPX16_3617	A0A3N0XR47_ANAGA Zinc finger protein 850	XM_068219487.1
A3G0345	4.09	14.76	NDEL1B	Nuclear distribution protein nudE-like 1-B	NM_201307.2
A16G0609	4.09	4.72	SMG9	Nonsense-mediated mRNA decay factor SMG9	XM_001923780.8

A13G0825	4.09	4.35	CGBB	Gonadotropin subunit beta-2	NM_001135791.1
B14G0092	4.09	3.53	PPARGC1B	Peroxisome proliferator-activated receptor gamma coactivator 1-beta	XM_009291061.4
A3G0369	4.09	5.53	COLGALT1	Procollagen galactosyltransferase 1	XM_689125.6
A5G0305	4.09	16.65	CAMK2B	calmodulin-dependent protein kinase type II subunit beta	XM_021476247.2
A5G1141	4.09	15.11	FIS1	Mitochondrial fission 1 protein	
B22G0430	4.09	0.62	DPX16_4609	A0A3N0YC08_ANAGA Uncharacterized protein	
B1G0316	4.09	6.17	RBM47	RNA-binding protein 47	XM_009307327.4
A20G0877	4.09	5.32	ANKRD6	Ankyrin repeat domain-containing protein 6	NM_194423.2
B10G0063	4.09	9.37	CARM1	Histone-arginine methyltransferase CARM1	XM_068219281.1
A25G0669	4.09	3.79	IGKV3-15	Immunoglobulin kappa variable 3-15	XM_068217596.1
B24G0026	4.09	8.10	NUB1	NEDD8 ultimate buster 1	
B7G1157	4.09	2.92	WRAP53	Telomerase Cajal body protein 1	NM_001256028.1
A17G0262	4.09	3.49	GPHN	Gephyrin	NM_001177444.1
B16G0896	4.09	9.53	CHN2	Beta-chimaerin	XM_694550.10
B9G0075	4.09	4.53	RAB9A	Ras-related protein Rab-9A	NM_200683.2
B24G0270	4.09	4.70	ALG14	UDP-N-acetylglucosamine transferase subunit ALG14 homolog	
B2G0585	4.09	5.19	KLHL20	Kelch-like protein 20	XM_005163492.5
A15G0035	4.09	1.01	G5714_015484	A0A7J6CB07_9TELE Uncharacterized protein	
B7G0161	4.08	4.48	ADM	Pro-adrenomedullin	NM_001326709.1
B10G0242	4.08	4.35	TMEM230	Transmembrane protein 230	NM_001004006.1
A9G0106	4.08	2.18	FASTKD1	FAST kinase domain-containing protein 1, mitochondrial	
B9G0731	4.08	3.04	LOC113108969	A0A6P6Q3Z1_CARAU cell surface glycoprotein CD200 receptor 1-A-like isoform X1	
B25G0259	4.08	17.58	KRAS	GTPase KRas	NM_001362494.1
B1G0389	4.08	6.84	RHOH	Rho-related GTP-binding protein RhoH	NM_001327973.1
A19G0091	4.08	5.60	ARID1A	AT-rich interactive domain-containing protein 1A	XM_021468300.2
B25G0459	4.08	2.87	ACTR6	Actin-related protein 6	NM_199696.1
A25G0677	4.08	14.74	IGKV3D-7	Immunoglobulin kappa variable 3D-7	NM_001045408.1
A3G1063	4.08	5.36	IL2RB	Interleukin-2 receptor subunit beta	XM_005171592.5
B3G1406	4.08	8.17	TMC6	Transmembrane channel-like protein 6	XM_017354837.3
A23G0309	4.08	6.14	NSFL1C	NSFL1 cofactor p47	XM_009296732.2
B25G0796	4.08	6.36	MORC2A	ATPase MORC2A	NM_001003994.1
B15G0431	4.08	3.94	ULK2	threonine-protein kinase ULK2	XM_002664615.6
A3G0291	4.08	6.75	FBXW9	WD repeat-containing protein 9	
A20G0474	4.08	4.41	INO80	Chromatin-remodeling ATPase INO80	XM_068215525.1
A11G0012	4.08	7.76	SOS1	Son of sevenless homolog 1	
B16G0885	4.08	2.14	MACC1	Metastasis-associated in colon cancer protein 1	NM_001199766.1
B20G0897	4.08	2.09	NAPRT	Nicotinate phosphoribosyltransferase	NM_200890.2
A19G0322	4.07	1.65	OTUD6B	Deubiquitinase OTUD6B	NM_200225.1
A23G0140	4.07	2.42	LAD1	Ladinin-1	NM_001386486.1
B13G0713	4.07	12.52	GPHB5	Glycoprotein hormone beta-5	XM_005156625.2

B20G0394	4.07	5.84	HOOK1	Protein Hook homolog 1	NM_001045198.1
A3G0193	4.07	2.96	ELAC2	Zinc phosphodiesterase ELAC protein 2	NM_001256204.1
B19G0919	4.07	8.77	THRB	Thyroid hormone receptor beta	XM_005159502.5
B11G0719	4.07	3.49	SULT6B1	Sulfotransferase 6B1	NM_001110379.2
B4G0093	4.07	1.94	DUS4L	20b) synthase [NAD(P)+]-like	NM_001003490.2
A4G0671	4.07	3.09	LOC107565638	A0A672KMX3_SINGR Uncharacterized LOC107565638	NM_001123290.1
A19G0404	4.07	4.75	GATAD2B	Transcriptional repressor p66-beta	NM_001044975.1
A8G0876	4.07	6.22	NAA35	N-alpha-acetyltransferase 35, NatC auxiliary subunit	NM_199550.1
A3G0567	4.07	7.28			
B1G0778	4.07	12.62	CAB39L	Calcium-binding protein 39-like	XM_005170986.5
B20G0809	4.07	3.12	RAD51-A	DNA repair protein RAD51 homolog A	NM_213206.2
A23G0506	4.07	4.97	ATF7	Cyclic AMP-dependent transcription factor ATF-7	NM_001030205.1
B16G1077	4.07	2.60			
A18G0312	4.07	3.68	EXOSC5	Exosome complex component RRP46	NM_001045352.2
A5G0004	4.07	5.35	THAP1	THAP domain-containing protein 1	NM_001423860.1
A8G0874	4.07	3.65	ANTXR1	Anthrax toxin receptor 1	XM_001332805.9
A7G0939	4.07	10.47			
A7G0124	4.07	2.95	RASGRP2	RAS guanyl-releasing protein 2	XM_009303654.4
B22G0524	4.07	4.76	SHISA5	Protein shisa-5	
A1G0355	4.07	4.06	CRYZL1	Quinone oxidoreductase-like protein 1	
A9G0291	4.07	23.42	FUNDC1	FUN14 domain-containing protein 1	NM_001002711.2
A16G0604	4.07	3.53	LOC107723548	A0A673JI71_9TELE Zinc finger protein 429-like	NM_001326353.1
A21G0598	4.06	6.60	DNAJC18	DnaJ homolog subfamily C member 18	NM_001113588.1
A5G0006	4.06	12.45	HOOK3	Protein Hook homolog 3	XM_017356208.3
A3G0535	4.06	3.43	CLEC17A	C-type lectin domain family 17, member A	
A19G0068	4.06	6.12	CENPA	Histone H3-like centromeric protein A	NM_001164240.2
B17G0282	4.06	7.51	ZNF395	Zinc finger protein 395	NM_001080054.2
B17G0763	4.06	6.61	ITPK1	Inositol-tetrakisphosphate 1-kinase	XM_001922333.5
B7G1114	4.06	0.86	OVOL2	Transcription factor Ovo-like 2	NM_001114744.2
B3G1652	4.06	4.50	RTASE	Probable RNA-directed DNA polymerase from transposon BS	XM_068214189.1
A8G0948	4.06	8.52	RBSN	Rabenosyn-5	XM_068223241.1
B17G0466	4.06	1.13	TMEM54	Transmembrane protein 54	XM_005158499.5
A1G0630	4.06	6.36	ZC3H13	Zinc finger CCCH domain-containing protein 13	NM_200522.2
A22G0287	4.06	3.49	MFSD6A	Major facilitator superfamily domain-containing protein 6-A	NM_001083056.1
B19G0446	4.06	5.89	RPRD1A	Regulation of nuclear pre-mRNA domain-containing protein 1A	XM_068215337.1
B22G0649	4.06	9.55	S1PR1	Sphingosine 1-phosphate receptor 1	NM_131691.3
B24G0036	4.06	6.88	WDR48	WD repeat-containing protein 48	NM_001423795.1
B5G0255	4.06	11.21	SMTN	Smoothelin	XM_068221575.1

B17G0825	4.06	6.45	AREL1	Apoptosis-resistant E3 ubiquitin protein ligase 1	XM_002663035.7
A1G0335	4.06	5.69	TAF5	Transcription initiation factor TFIID subunit 5	NM_001037431.2
A13G0407	4.06	3.97	LOC113097471	A0A6P6PC16_CARAU uncharacterized protein LOC113076207 isoform X1	XM_005156926.5
B15G0738	4.06	0.75	OSTN	Osteocrin	NM_001326409.1
A6G0469	4.06	4.30	G5714_006964	A0A7J6CXW5_9TELE RING-CH-type domain-containing protein	
B1G0099	4.06	4.92	NRP2	Neuropilin-2	NM_212965.1
B20G0891	4.06	3.84	AIDA	Axin interactor, dorsalization-associated protein	NM_200790.2
B12G0289	4.06	6.54	CFAP119	Cilia- and flagella-associated protein 119	NM_001328026.1
B24G0427	4.06	7.83	ACOT9	Acyl-coenzyme A thioesterase 9, mitochondrial	XM_005162711.5
B21G0587	4.06	7.79	FYB1	FYN-binding protein 1	XM_009295371.4
B3G1420	4.06	18.87	CYTH1	Cytohesin-1	XM_017353253.3
A21G0833	4.06	0.83	HTR2C	5-hydroxytryptamine receptor 2C	XM_001339004.8
A12G0451	4.06	9.61	KCTD17	POZ domain-containing protein KCTD17	XM_021474246.2
B14G0602	4.06	1.39	ADRA2	Alpha-2 adrenergic receptor	NM_194364.2
A7G1131	4.05	3.37	FES	Fps	NM_001199869.1
A6G0574	4.05	7.44	GPBP1L1	Vasculin-like protein 1	XM_021477183.2
B7G0309	4.05	1.74	A0A669BJX9	A0A669BJX9_ORENI Uncharacterized protein	
B6G0813	4.05	7.87	EPHA2	Ephrin type-A receptor 2	NM_131415.1
B7G1136	4.05	4.30	BCR	Breakpoint cluster region protein	
A8G0330	4.05	4.12	TAB3	TGF-beta-activated kinase 1 and MAP3K7-binding protein 3	NM_001045105.1
A18G0480	4.05	2.75	CO039	Uncharacterized protein C15orf39 homolog	XM_005159151.5
A6G0189	4.05	7.85	ERCC3	General transcription and DNA repair factor IIH helicase subunit XPB	NM_201582.1
B8G0877	4.05	9.51	GRIPAP1	GRIP1-associated protein 1	NM_001123320.1
B1G0510	4.05	1.85	METAP1D	Methionine aminopeptidase 1D, mitochondrial	NM_001024392.1
A3G0672	4.05	9.19	DMBT1	Deleted in malignant brain tumors 1 protein	XM_683847.9
B23G0391	4.05	1.26	COL14A1	Collagen alpha-1(XIV) chain	XM_017353714.3
B14G0239	4.05	3.22			XM_021472854.2
B16G1044	4.05	2.15	PMVK	Phosphomevalonate kinase	XM_068213834.1
A11G0060	4.05	7.34	CLASP1B	CLIP-associating protein 1-B	
B2G0809	4.05	2.14			
B14G0115	4.04	0.21	EMX1	Homeobox protein EMX1	NM_131279.2
B15G0219	4.04	8.02	FNDC3A	Fibronectin type-III domain-containing protein 3a	XM_021466300.2
B11G0283	4.04	12.02	TCB1	Transposable element Tcb1 transposase	NM_212668.2
A21G0707	4.04	10.94	DBN1	Drebrin	XM_009295615.4
B20G0869	4.04	3.01	CPSF2	Cleavage and polyadenylation specificity factor subunit 2	NM_001002384.1
B5G0209	4.04	7.15	PRRC2B	Protein PRRC2B	XM_068221580.1
B16G1073	4.04	0.00	ROHU_025891	A0A498MDC1_LABRO Uncharacterized protein	
B4G0063	4.04	5.01	FGFR1OP2	FGFR1 oncogene partner 2 homolog	NM_199955.1

A3G0585	4.04	3.56	PPL	Periplakin	NM_001137666.1
B3G1483	4.04	2.45	ROHU_008943	A0A498M1H8_LABRO Coiled-coil domain-containing protein 106-like	
B10G0514	4.04	12.15	HSF1	Heat shock factor protein 1	XM_001332159.7
B3G1444	4.04	3.60	AXIN2	Axin-2	NM_131561.1
A16G0541	4.04	7.03	EPB41L4B	Band 4.1-like protein 4B	XM_009292361.4
A5G0343	4.04	4.00	BRWD3	Bromodomain and WD repeat-containing protein 3	XM_021471594.2
B17G0050	4.04	4.01	ZFYVE19	NoCut checkpoint regulator	
B1G1194	4.03	21.95	ITGAL	Integrin alpha-L	NM_001386624.1
B11G0199	4.03	2.67	RTEL1	Regulator of telomere elongation helicase 1	XM_009305916.4
B17G0077	4.03	5.83	TMX4	Thioredoxin-related transmembrane protein 4	NM_001030159.2
B9G0607	4.03	1.71	CHAF1B	Chromatin assembly factor 1 subunit B	NM_001328129.1
B9G0818	4.03	5.12	DOCK9	Dedicator of cytokinesis protein 9	XM_068223516.1
A17G0086	4.03	2.45	ORC3	Origin recognition complex subunit 3	NM_212727.2
A10G0543	4.03	9.59	SMPD1	Sphingomyelin phosphodiesterase	NM_001326536.1
B6G0862	4.03	8.73	CD81	CD81 protein	NM_001018150.1
B3G1574	4.03	7.64	G5714_002846	A0A7J6D7V4_9TELE Uncharacterized protein	
A4G0517	4.03	14.42	CELF2	CUGBP Elav-like family member 2	XM_068219532.1
B9G0743	4.03	4.29	NDUFV3	NADH dehydrogenase [ubiquinone] flavoprotein 3, mitochondrial	XM_009304656.4
B14G0465	4.03	15.93	GPX3	Glutathione peroxidase 3	NM_001137555.3
B18G0525	4.03	4.07	SIAH1	E3 ubiquitin-protein ligase Siah1	XM_005159143.5
A3G1006	4.03	5.20	MSL1L1	Male-specific lethal 1-like 1	NM_001114468.1
A21G0030	4.03	16.86	UHRF2	E3 ubiquitin-protein ligase UHRF2	
A19G0337	4.03	40.90	NDRG1	Protein NDRG1	NM_213348.3
A13G0513	4.03	2.20	HK1	Hexokinase-1	NM_001039988.1
A17G0078	4.03	3.39	QPCT	Glutaminyl-peptide cyclotransferase	XM_009293242.4
B2G0663	4.03	13.94	ATP6V1H	V-type proton ATPase subunit H	NM_001291708.1
A15G0425	4.03	5.13	VPS53	Vacuolar protein sorting-associated protein 53 homolog	XM_005157580.5
A16G0182	4.03	13.48	ARHGAP32	Rho GTPase-activating protein 32	XM_005158402.5
B18G0166	4.03	8.52	TNFAIP8L3	Tumor necrosis factor alpha-induced protein 8-like protein 3	NM_200351.1
B12G0758	4.02	8.34	MPRIP	Myosin phosphatase Rho-interacting protein	XM_021480197.2
A19G0113	4.02	12.15	CTHRC1	Collagen triple helix repeat-containing protein 1	NM_001003765.3
A9G0643	4.02	2.85	MITD1	MIT domain-containing protein 1	NM_200201.2
B19G0456	4.02	3.46	NUP153	Nuclear pore complex protein Nup153	NM_001326384.1
A10G0326	4.02	4.25	LOC113067422	A0A6P6MHX5_CARAU uncharacterized protein LOC113067422 isoform X2	
B2G1049	4.02	15.01	GPT2L	Alanine aminotransferase 2-like	XM_017357999.3
A15G0123	4.02	2.56	PXYLP1	2-phosphoxylose phosphatase 1	XM_005157794.5

A7G1179	4.02	2.46	ADAMTS17	A disintegrin and metalloproteinase with thrombospondin motifs 17	XM_021477710.2
A15G0464	4.02	30.42	MARK4	microtubule affinity-regulating kinase 4	XM_694854.10
A4G0882	4.02	4.23	CCNC	Cyclin-C	NM_199951.2
A19G0571	4.02	3.55	NGLY1	Peptide-N(4)-(N-acetyl-beta-glucosaminyl)asparagine amidase	NM_001020601.1
A22G0576	4.02	3.47	GDE1	Glycerophosphodiester phosphodiesterase 1	NM_200243.1
A21G0698	4.02	2.63	UBTD2	Ubiquitin domain-containing protein 2	NM_001002718.2
B19G0058	4.02	6.58	SNX13	Sorting nexin-13	XM_021468239.2
A14G0436	4.01	5.35	CNOT6	CCR4-NOT transcription complex subunit 6	NM_001110028.1
B22G1091	4.01	4.61	PTK7	Inactive tyrosine-protein kinase 7	
A17G0790	4.01	6.49	TATDN3	Putative deoxyribonuclease TATDN3	XM_005158571.4
B11G0524	4.01	2.04	XLRS1	Retinoschisin	NM_001003538.2
B6G0290	4.01	8.00	INO80DB	INO80 complex subunit D-B	NM_001080588.1
A3G0256	4.01	8.92	TSPAN1	Tetraspanin-1	NM_200408.2
B13G0315	4.01	4.14	RNF145	RING finger protein 145	NM_001006100.1
A12G0471	4.01	0.34	TNFRSF17	Tumor necrosis factor receptor superfamily member 17	XM_068219305.1
B9G0427	4.01	6.85	TXNDC9	Thioredoxin domain-containing protein 9	NM_001328576.1
A5G1029	4.01	5.49	DTX2	Probable E3 ubiquitin-protein ligase DTX2	XM_068221281.1
A20G0685	4.01	8.56	IGFBP1	Insulin-like growth factor-binding protein 1	NM_173283.4
B6G0640	4.01	14.76	UBE3A	Ubiquitin-protein ligase E3A	NM_001007318.1
B11G0753	4.01	2.40	G5714_012156	A0A7J6CPQ7_9TELE G_PROTEIN_RECEP_F1_2 domain-containing protein	NM_001386344.1
A1G0370	4.01	3.54	RCBTB1	RCC1 and BTB domain-containing protein 1	XM_005170970.5
B3G1718	4.01	7.48	A33	Zinc-binding protein A33	NM_001044711.1
A12G0400	4.01	8.78	ABLIM2	Actin-binding LIM protein 2	XM_068218673.1
A7G0862	4.01	9.51	MTMR1	Myotubularin-related protein 1	XM_005172286.5
B23G0111	4.01	1.52	SEL1L3	Protein sel-1 homolog 3	XM_021469868.2
A22G0131	4.01	23.77	S1PR3	Sphingosine 1-phosphate receptor 3	NM_001118891.1
B5G0543	4.01	1.15	COL27A1B	Collagen alpha-1(XXVII) chain B	NM_001080575.1
A16G0599	4.01	3.73	A0A672SAS1	A0A672SAS1_SINGR Zgc:66448	NM_199800.1
B4G0453	4.01	6.42	WASHC3	WASH complex subunit 3	NM_200173.1
A17G0066	4.01	5.09	COL12A1	Collagen alpha-1(XII) chain	XM_009293296.4
B7G0466	4.01	0.82	PLEKHF2	Pleckstrin homology domain-containing family F member 2	
A2G0841	4.01	2.25	PARP2	Poly [ADP-ribose] polymerase 2	NM_001204270.1
A20G0679	4.00	7.60	TRAPPC8	Trafficking protein particle complex subunit 8	XM_017352637.3
A5G1213	4.00	2.87	HIP1R	Huntingtin-interacting protein 1-related protein	NM_001083565.2
B5G0372	4.00	6.93	KCMF1	E3 ubiquitin-protein ligase KCMF1	NM_200952.2
B5G0305	4.00	3.79	UNG	Uracil-DNA glycosylase	NM_200974.1
A10G0522	4.00	6.80	SCAF4	SR-related and CTD-associated factor 4	XM_690795.9

A10G0169	4.00	4.66	CETN3	Centrin-3	NM_001020499.1
A1G0993	4.00	7.07	PGM2	Phosphopentomutase	NM_212886.1
B13G0328	4.00	2.47	CEP170	Centrosomal protein of 170 kDa	XR_011005843.1
A21G0818	4.00	11.32	AMMECR1	Nuclear protein AMMECR1	NM_200581.1
B4G0412	4.00	4.22	LOC107565638	A0A672KMX3_SINGR Uncharacterized LOC107565638	NM_001123290.1
A15G0872	4.00	3.61	SMC4	Structural maintenance of chromosomes protein 4	NM_173253.2
B9G0324	4.00	2.81	GPR34	Probable G-protein coupled receptor 34	NM_001128546.1
B14G0258	4.00	5.16	ZDHHC9	Palmitoyltransferase ZDHHC9	NM_001110026.1
B19G0788	4.00	3.02	CLEC4F	C-type lectin domain family 4 member F	XM_068224046.1
B2G0777	4.00	4.87	MPV17L2	Mpv17-like protein 2	NM_001002567.1
A21G0771	4.00	4.88	MAP4K2	Mitogen-activated protein kinase kinase kinase kinase 2	XM_005161421.5
A8G0113	4.00	17.20	CLDN5	Claudin-5	NM_213274.1
A7G0426	4.00	4.28	TP53BP1	TP53-binding protein 1	XM_017356868.2
B22G0530	4.00	6.61	RAD54L2	Helicase ARIP4	XM_005174006.5
B13G0904	4.00	5.07			XM_068212832.1
B4G0858	4.00	2.36	A0A673G440	A0A673G440_9TELE Uncharacterized protein	XM_068213652.1
A14G0725	4.00	5.23	PTCD3	Pentatricopeptide repeat domain-containing protein 3, mitochondrial	NM_001037372.2
B2G0984	4.00	2.48	ACOT11	Acyl-coenzyme A thioesterase 11	XM_068224164.1
A24G0608	4.00	11.97	ACBD5A	Acyl-CoA-binding domain-containing protein 5A	XM_068217073.1
B10G0320	4.00	3.13	AP4M1	AP-4 complex subunit mu-1	NM_001002672.1
A17G0796	4.00	2.17	DUSP23	Dual specificity protein phosphatase 23	
B13G0277	4.00	4.51	ATRN	Attractin	NM_001013308.2
B6G0297	4.00	3.36	GMPPAB	Mannose-1-phosphate guanyltransferase alpha-B	NM_200497.1
B1G0485	4.00	3.68	LIG4	DNA ligase 4	XM_005159958.5
B19G0562	4.00	1.32	HEPACAM2	HEPACAM family member 2	NM_001245085.1
A5G0560	4.00	2.90	LOC113074943	A0A6P6N4S4_CARAU uncharacterized protein LOC113074943	
B5G0680	4.00	8.94	G5714_005574	A0A7J6D1E4_9TELE SCY domain-containing protein	
B6G0157	3.99	6.72	UBN2	Ubinuclein-2	NM_001080169.1
A10G0010	3.99	3.48	ERG	Transcriptional regulator Erg	NM_001008616.1
B9G0193	3.99	9.22	SESTD1	SEC14 domain and spectrin repeat-containing protein 1	NM_182881.2
B11G0303	3.99	5.56	POL	Retrovirus-related Pol polyprotein from transposon 17.6	NM_001048235.1
A17G0160	3.99	5.96	PELI2	E3 ubiquitin-protein ligase pellino homolog 2	NM_001002616.1
A15G0834	3.99	3.41	SBDS	Ribosome maturation protein SBDS	NM_201121.2
A4G0886	3.99	7.25	TMEM63A	CSC1-like protein 1	XM_068219650.1
B10G0501	3.99	5.77	C2CD2	C2 domain-containing protein 2	XM_009305499.4
B4G0467	3.99	8.13	PLXNC1	Plexin-C1	XM_068220763.1
B10G0494	3.99	2.85	DNAJC30	DnaJ homolog subfamily C member 30, mitochondrial	
A3G0392	3.99	0.87	TRIP10	Cdc42-interacting protein 4 homolog	

B19G0707	3.99	2.29	THAP6	THAP domain-containing protein 6	
B18G0379	3.99	6.51	TSPAN18	Tetraspanin-18	XM_017351914.2
A5G1112	3.99	9.19	PRUNE2	Protein prune homolog 2	NM_001111154.2
A20G0891	3.99	2.99	PTPRK	Receptor-type tyrosine-protein phosphatase kappa	XM_021468580.2
B4G0202	3.99	8.83	LTA4H	Leukotriene A-4 hydrolase	NM_213286.1
B2G0859	3.99	4.42	STK25	threonine-protein kinase 25	NM_213308.1
B3G1092	3.98	4.01	SGF29	SAGA-associated factor 29	XM_005163811.5
B4G0553	3.98	1.92	SEMA3C	Semaphorin-3C	XM_017355318.3
A25G0373	3.98	2.14	RNF44	RING finger protein 44	
B10G0595	3.98	6.95	PHF12	PHD finger protein 12	XM_001919920.8
B16G0561	3.98	4.18	TEX10	Testis-expressed protein 10 homolog	NM_212705.1
B5G0541	3.98	3.40	SH2D3C	SH2 domain-containing protein 3C	XM_021476041.2
A24G0094	3.98	1.31	EPS8L1	Epidermal growth factor receptor kinase substrate 8-like protein 1	XM_005170798.5
A20G0099	3.98	4.42	CPSF2	Cleavage and polyadenylation specificity factor subunit 2	XM_017352464.3
A3G0916	3.98	1.12	LIN1	LINE-1 reverse transcriptase homolog	
A5G0883	3.98	5.49	CARD9	Caspase recruitment domain-containing protein 9	NM_001110285.2
B5G0484	3.98	1.65	LOC107712161	A0A673JGA4_9TELE Titin-like	
B7G0903	3.98	11.38	SLC7A8	Large neutral amino acids transporter small subunit 2	NM_001271897.1
A23G0461	3.98	6.93	ERBB3	Receptor tyrosine-protein kinase erbB-3	NM_001014826.2
B8G0090	3.98	3.90	PEX10	Peroxisome biogenesis factor 10	NM_001005994.1
B17G0711	3.98	14.06	MTARC1	Mitochondrial amidoxime-reducing component 1	
B22G0565	3.98	7.27	PTPN4	Tyrosine-protein phosphatase non-receptor type 4	XM_005173946.5
B4G0623	3.98	2.56	TRMU	Mitochondrial tRNA-specific 2-thiouridylase 1	NM_001020599.1
A1G0024	3.98	3.87	CAMSAP3	Calmodulin-regulated spectrin-associated protein 3	XM_003197797.6
A2G0964	3.98	4.90	TRIM16	Tripartite motif-containing protein 16	NM_001430853.1
A24G0458	3.98	5.60	PDIA4	Protein disulfide-isomerase A4	NM_199779.2
A18G0581	3.98	4.40	PECR	Peroxisomal trans-2-enoyl-CoA reductase	NM_001099999.1
A2G0394	3.98	4.44	ARHGAP29	Rho GTPase-activating protein 29	NM_200111.2
B21G0885	3.98	4.77	CD274	Programmed cell death 1 ligand 1	
A24G0345	3.98	3.48	YTHDF3	YTH domain-containing family protein 3	NM_199870.1
A4G0898	3.97	9.85	CCND2	S-specific cyclin-D2	NM_001045068.1
A6G0139	3.97	2.11	FKBP11	Peptidyl-prolyl cis-trans isomerase FKBP11	XM_005165698.5
A16G0216	3.97	2.33	POLR3GL	DNA-directed RNA polymerase III subunit RPC7-like	NM_001005959.2
A12G0260	3.97	1.45	KCNK1	Potassium channel subfamily K member 1	NM_001098753.2
A3G0477	3.97	2.73	SNX8	Sorting nexin-8	NM_001326490.1
A13G0522	3.97	5.52			NM_001020523.1
A8G0837	3.97	0.73	GNA14	Guanine nucleotide-binding protein subunit alpha-14	XM_005170475.5

A20G0611	3.97	3.94	NUDT14	Uridine diphosphate glucose pyrophosphatase NUDT14	NM_001044888.1
B7G0104	3.97	3.61	TYMS	Thymidylate synthase	NM_131760.2
B23G0089	3.97	6.99	PLCG1	1-phosphatidylinositol 4,5-bisphosphate phosphodiesterase gamma-1	NM_194407.1
A14G0619	3.97	5.38	MORC4	MORC family CW-type zinc finger protein 4	NM_001320211.1
A22G0011	3.97	8.68	SPEN	Msx2-interacting protein	XM_005170347.5
A3G0978	3.97	5.40	FAM171A2	Protein FAM171A2	XM_679189.8
A19G0124	3.97	1.15	GRHL2	Grainyhead-like protein 2 homolog	XM_005159580.5
A19G0377	3.97	513.51	GRN3	Granulin-3	
B2G0577	3.97	7.75	COP1	E3 ubiquitin-protein ligase COP1	NM_001089542.1
B22G0195	3.97	1.34	LOC107702631	A0A671Q216_9TELE Uncharacterized LOC107702631	XM_003200968.6
A19G0559	3.97	4.73	CCDC126	Coiled-coil domain-containing protein 126	NM_001045403.1
B10G0190	3.97	8.32	PSTPIP2	Proline-serine-threonine phosphatase-interacting protein 2	XM_009305134.4
B21G0150	3.97	5.51	PIGS	GPI transamidase component PIG-S	NM_001076614.1
B22G0706	3.97	0.65	NR2C2AP	Nuclear receptor 2C2-associated protein	
A6G0200	3.97	5.41	ABI2	Abl interactor 2	XM_005165776.5
A22G0296	3.97	15.77	AOX1	Aldehyde oxidase 1	NM_001278817.1
B9G0292	3.97	4.33	P2RY8	P2Y purinoceptor 8	XM_021472205.2
B3G0823	3.97	24.34	RUSF1	RUS family member 1	NM_001110453.2
A23G0497	3.97	8.00	DTX3	Probable E3 ubiquitin-protein ligase DTX3	XM_005162244.4
A14G0543	3.97	3.45	PURA	Transcriptional activator protein Pur-alpha	XM_005157161.5
B3G1717	3.97	1.28	LOC107741889	A0A673H388_9TELE Uncharacterized LOC107741889	
B19G0144	3.97	4.13	LOC113119871	A0A6P6RI26_CARAU uncharacterized protein LOC113119871	XM_009293998.4
B20G0504	3.97	3.03	ADAM17	Disintegrin and metalloproteinase domain-containing protein 17	XM_021468637.2
B1G0296	3.97	3.77	TLR3	Toll-like receptor 3	NM_001013269.3
B8G0177	3.97	2.73	RNF122	RING finger protein 122	NM_001327816.1
B12G0699	3.97	6.42	FRA10AC1	Protein FRA10AC1	NM_001006006.2
B3G1108	3.97	4.09	MRC2	C-type mannose receptor 2	XM_021471386.2
A25G0244	3.97	5.82	CSK22	Casein kinase II subunit alpha'	XM_679168.9
B5G0695	3.97	6.83	NCOR1	Nuclear receptor corepressor 1	XM_009301627.4
A21G0081	3.97	0.64	SPOUT1	Putative methyltransferase C9orf114	NM_001004627.1
B6G0532	3.97	2.39	SHROOM2	Protein Shroom2	XM_017356634.3
B1G0490	3.97	11.42	SPP2	Secreted phosphoprotein 24	NM_001327983.1
A19G0940	3.97	8.64	COL14A1	Collagen alpha-1(XIV) chain	XM_068215347.1
A24G0220	3.97	2.22	TRDMT1	tRNA (cytosine(38)-C(5))-methyltransferase	NM_001018143.1
B16G0845	3.97	1.98	DPX16_11321	A0A3N0XID6_ANAGA Uncharacterized protein	
A16G0140	3.97	5.42	RPZ	Protein rapunzel	NM_001017910.1
B25G0838	3.97	1.19	YARS2	Tyrosine--tRNA ligase, mitochondrial	NM_001143920.1

A2G0262	3.96	4.74	TCB1	Transposable element Tcb1 transposase	XR_011017426.1
B16G0624	3.96	2.90	RBFA	Putative ribosome-binding factor A, mitochondrial	NM_001089485.2
B8G0244	3.96	4.25	PRAF2	PRA1 family protein 2	NM_001017698.1
A13G0373	3.96	4.13			XM_068218731.1
B3G0630	3.96	1.88	SVIL	Supervillin	XM_021474702.2
A24G0439	3.96	24.73	CSRNP1	serine-rich nuclear protein 1	XM_005162674.5
A5G0090	3.96	4.82	MAPKAP1	Target of rapamycin complex 2 subunit MAPKAP1	XM_003198574.6
B3G1607	3.96	2.72	A0A673G4G7	A0A673G4G7_9TELE Uncharacterized protein	XM_068217817.1
B1G0482	3.96	3.63	CARS2	Probable cysteine--tRNA ligase, mitochondrial	XM_677640.10
B4G0231	3.96	5.36	SCUBE1	Signal peptide, CUB and EGF-like domain-containing protein 1	NM_001328599.1
A2G0464	3.96	3.99	ZGC:55781	UPF0415 protein C7orf25 homolog	NM_214694.2
B2G1286	3.96	5.59	TRIM16	Tripartite motif-containing protein 16	XM_681071.8
A13G0141	3.96	6.89	PCDH15	Protocadherin-15	XR_002459827.2
A9G0789	3.96	6.67	LNPKA	Endoplasmic reticulum junction formation protein lunapark-A	NM_199480.1
A4G0948	3.96	22.71	NAMPT	Nicotinamide phosphoribosyltransferase	XM_002661340.6
A23G0402	3.96	3.39	EIF4G3	Eukaryotic translation initiation factor 4 gamma 3	XM_068219396.1
A3G0537	3.96	1.78	CD209D	CD209 antigen-like protein D	
A4G0042	3.96	1.30	DPX16_1179	A0A3N0XUS6_ANAGA Zinc finger protein 569	XM_021468205.2
B1G1154	3.96	7.99	TNFSF14	Tumor necrosis factor ligand superfamily member 14	
B7G0031	3.96	3.79	A0A673MQU1	A0A673MQU1_9TELE Uncharacterized protein	
B16G0693	3.96	11.46	ADAR	Double-stranded RNA-specific adenosine deaminase	NM_131596.2
A6G0647	3.96	1.62	GPR84	G-protein coupled receptor 84	NM_001105697.2
B14G0257	3.96	2.99	C1GALT1C1	C1GALT1-specific chaperone 1	NM_199667.3
A25G0306	3.96	17.31	CCND2	S-specific cyclin-D2	NM_001089445.1
B19G0516	3.95	1.52	SYTL1	Synaptotagmin-like protein 1	NM_001128533.1
B4G0371	3.95	5.84	PRICKLE1	Prickle-like protein 1	NM_001030098.2
B25G0529	3.95	12.79	CNOT1	CCR4-NOT transcription complex subunit 1	NM_001079951.2
B16G0732	3.95	4.49	SETDB1B	Histone-lysine N-methyltransferase SETDB1-B	NM_001077277.3
B18G0048	3.95	2.89	BARX2	Homeobox protein BarH-like 2	XM_001342008.8
B3G0552	3.95	5.37	MFSD11	UNC93-like protein MFSD11	XM_001339605.8
B6G0369	3.95	3.78	THUMPD3	tRNA (guanine(6)-N2)-methyltransferase THUMP3	NM_001089394.2
B14G0102	3.95	6.94	THOC3	THO complex subunit 3	NM_001003758.1
B23G0216	3.95	4.16	ARHGAP39	Rho GTPase-activating protein 39	XM_068219384.1
A8G0845	3.95	1.89	ANKRD39	Ankyrin repeat domain-containing protein 39	NM_001113640.1
A17G0327	3.95	4.24	DEGS2	C4-monooxygenase DES2	NM_001163366.1
A7G0389	3.95	1.47	COL4A6	Collagen alpha-6(IV) chain	XM_068222724.1
A9G0475	3.95	1.18	ESYT3	Extended synaptotagmin-3	NM_001123233.1
B16G0655	3.95	5.80	SMG9	Nonsense-mediated mRNA decay factor SMG9	XM_001923780.8
B1G0845	3.95	3.18	FAM13A	Protein FAM13A	XM_017356507.3
A5G0787	3.95	2.67	ALKBH2	DNA oxidative demethylase ALKBH2	NM_001256684.1

B16G0986	3.95	4.18	CNOT10	CCR4-NOT transcription complex subunit 10	XM_005157863.5
A9G0697	3.95	2.23	LOC113053765	A0A6P6KR97_CARAU uncharacterized protein LOC113053765	XR_001800369.3
A18G0632	3.94	3.93	TMEM53	Transmembrane protein 53	NM_001201568.1
A2G0281	3.94	1.32	DPX16_7361	A0A3N0Z270_ANAGA Uncharacterized protein	
B1G0970	3.94	7.20			
A7G0136	3.94	10.41	SMCHD1	Structural maintenance of chromosomes flexible hinge domain-containing protein 1	XM_009303635.4
B1G1129	3.94	3.30	LOC113099796	A0A6P6PHG1_CARAU zinc finger protein 271-like	XM_017357165.3
B10G0046	3.94	7.17	SYK	Tyrosine-protein kinase SYK	XM_005155538.5
B22G1137	3.94	8.64	RTASE	Probable RNA-directed DNA polymerase from transposon BS	
B24G0481	3.94	5.23	PLCD1	1-phosphatidylinositol 4,5-bisphosphate phosphodiesterase delta-1	NM_001109700.1
A16G0431	3.94	3.81	CCN4	CCN family member 4	NM_001113628.1
A17G0431	3.94	4.43	MTMR9L	Myotubularin-related protein 9-like	XM_009293091.4
B8G0782	3.94	4.81	BCAR3	Breast cancer anti-estrogen resistance protein 3 homolog	XM_021478295.2
A8G0081	3.94	3.72	CV039	UPF0545 protein C22orf39 homolog	NM_001123264.1
B12G0451	3.94	1.89	SLC16A8	Monocarboxylate transporter 3	NM_001324527.1
B17G0107	3.94	3.12	TRERF1	Transcriptional-regulating factor 1	XM_009292797.4
B17G0152	3.94	2.71	FZD3	Frizzled-3	NM_001080601.1
B7G1194	3.94	6.12	FXR2	RNA-binding protein FXR2	NM_200211.2
B11G0046	3.94	4.70	IFT52	Intraflagellar transport protein 52 homolog	NM_001039989.2
B3G0825	3.94	11.66	PRR14L	Protein PRR14L	XM_021473976.2
A23G0641	3.94	3.47	COQ10B-A	Coenzyme Q-binding protein COQ10 homolog A, mitochondrial	XM_009297030.3
A22G0436	3.94	4.14	RGMB	Repulsive guidance molecule B	XM_021469302.2
B14G0657	3.94	14.34	SLC35A4	SLC35A4 upstream open reading frame protein	NR_045688.1
B10G0166	3.94	5.06	MOCS2	Molybdopterin synthase sulfur carrier subunit	XM_068218567.1
B16G0256	3.94	5.54	ETS1	c-ets-1	NM_001037375.1
B22G0553	3.93	4.13	SGSH	N-sulphoglucosamine sulphohydrolase	NM_001123268.1
A3G0067	3.93	2.88	BTNL2	Butyrophilin-like protein 2	XM_021472967.2
A8G0523	3.93	4.83	BORCS6	BLOC-1-related complex subunit 6	NM_001044988.1
B2G0499	3.93	4.88	TRAV18	T cell receptor alpha variable 18	
A14G0018	3.93	5.33	CLNK	Cytokine-dependent hematopoietic cell linker	
B22G0923	3.93	8.28	ADGRG7	Adhesion G-protein coupled receptor G7	XR_011008357.1
B20G0236	3.93	8.86	SUCO	SUN domain-containing ossification factor	XM_068215626.1
B23G0251	3.93	1.33	MAPK12	Mitogen-activated protein kinase 12	NM_001111167.1
B15G0567	3.93	4.97	UNC119	Protein unc-119 homolog A	XM_690112.7
A1G0176	3.93	7.31	CC2D1A	Coiled-coil and C2 domain-containing protein 1A	XM_017356645.3

B7G0041	3.93	27.54	TRIM21	E3 ubiquitin-protein ligase TRIM21	
B18G0348	3.93	2.58	EXOSC5	Exosome complex component RRP46	NM_001045352.2
B1G0531	3.93	3.01	ANOS1	Anosmin-1	NM_131358.2
B15G0928	3.93	18.17	ZGC:66101	threonine-protein kinase SIK3 homolog	NM_200541.1
B9G0885	3.93	7.35	IFNAR2	beta receptor 2	
B8G0038	3.93	6.53	DCAF1	DDB1- and CUL4-associated factor 1	XM_002663268.7
B18G0531	3.93	3.43	RSPRY1	RING finger and SPRY domain-containing protein 1	NM_001045072.2
B20G0590	3.92	3.51	OJAV_G00032190	A0A3S2PEG9_ORYJA Uncharacterized protein	XM_068215400.1
A11G0526	3.92	8.89	GNAI2	Guanine nucleotide-binding protein G(i) subunit alpha-2	NM_001177346.1
A9G0574	3.92	6.31	UGT1A1	UDP-glucuronosyltransferase 1A1	NM_213422.2
B8G0755	3.92	1.47	ELOVL7	Elongation of very long chain fatty acids protein 7	NM_199875.2
B22G0005	3.92	4.58	NCKIPSD	NCK-interacting protein with SH3 domain	NM_001423277.1
A5G1326	3.92	2.30	B4GALT4	Beta-1,4-galactosyltransferase 4	NM_001366733.1
B15G0498	3.92	10.33	IFT20	Intraflagellar transport protein 20 homolog	XM_005157577.5
A11G0672	3.92	1.90	PRSS2	Anionic trypsin-2	XM_021479666.2
B5G1310	3.92	7.17	AP3M2	AP-3 complex subunit mu-2	NM_001002154.1
B25G0230	3.92	4.42	UEVLD	Ubiquitin-conjugating enzyme E2 variant 3	NM_001003619.2
A12G0602	3.92	4.40	STAT5B	Signal transducer and activator of transcription 5B	NM_001003984.2
B23G0382	3.92	8.21	USP19	Ubiquitin carboxyl-terminal hydrolase 19	XM_005162120.5
A4G0684	3.92	4.64	ZC3HC1	Zinc finger C3HC-type protein 1	NM_001077378.2
A8G0567	3.92	12.12	KDM5BB	Lysine-specific demethylase 5B-B	XM_009303989.4
A5G0188	3.92	6.75	YPEL1	Protein yippee-like 1	NM_001003780.1
B8G0527	3.92	6.28	TSPYL1	Testis-specific Y-encoded-like protein 1	NM_200055.1
A23G0775	3.92	3.36	G5714_022319	A0A7J6BMV9_9TELE Uncharacterized protein	XM_068216764.1
B25G0880	3.91	0.91	RL402	Ubiquitin-60S ribosomal protein L40	
A5G0154	3.91	4.57	HSCB	Iron-sulfur cluster co-chaperone protein HscB	NM_001128398.1
B9G0352	3.91	4.68	GZMB	Granzyme B	NM_001114694.1
A21G0094	3.91	6.92	NUP188	Nucleoporin NUP188	XM_001922983.6
B24G0264	3.91	18.48	CPNE3	Copine-3	XM_005162839.5
A13G0567	3.91	2.54	TM9SF3	Transmembrane 9 superfamily member 3	
B14G0514	3.91	3.15	ABCA1	Phospholipid-transporting ATPase ABCA1	XM_005173080.5
A15G0532	3.91	4.59	ZGC:153454	Mediator of RNA polymerase II transcription subunit 13-like	NM_001083838.2
A3G0265	3.91	0.61	DPX16_7980	A0A3N0Z4Z7_ANAGA Nucleotide-binding oligomerization domain-containing protein 2	XM_068218778.1
A15G0269	3.91	1.27	HDAC11	Histone deacetylase 11	XM_001920632.8
B23G0192	3.91	3.35	G5714_022907	A0A7J6BPI5_9TELE Uncharacterized protein	

B12G0604	3.91	6.84	VPS35L	VPS35 endosomal protein-sorting factor-like	NM_001098181.1
A4G0959	3.91	5.37	COG5	Conserved oligomeric Golgi complex subunit 5	XM_005164473.4
B20G0578	3.91	4.58	OLFML2B	Olfactomedin-like protein 2B	XM_005160712.5
B1G0883	3.91	20.83	GET3	ATPase GET3	NM_001002298.2
B14G0382	3.91	4.65	SLC30A9	Zinc transporter 9	NM_001008575.1
A2G0569	3.91	5.89	TESK2	Dual specificity testis-specific protein kinase 2	NM_001110476.1
B7G0370	3.91	7.64	ANKRD46	Ankyrin repeat domain-containing protein 46	XM_005166503.4
B16G0756	3.91	25.18	ATP1A3	potassium-transporting ATPase subunit alpha-3	NM_131685.2
B2G0940	3.91	9.31	GK5	Putative glycerol kinase 5	NM_001077803.3
B22G0526	3.91	3.34	LOC113079557	A0A6P6NGP1_CARAU protein shisa-5-like	
B16G0582	3.91	4.02	EPB41L4B	Band 4.1-like protein 4B	XM_005158033.5
B3G0500	3.91	90.32	HBA	Hemoglobin subunit alpha	NM_001013461.2
B2G1180	3.91	5.65	ARHGAP21	Rho GTPase-activating protein 21	XM_001918933.7
A11G0237	3.91	8.65	LOC113111007	A0A6P6QCC0_CARAU uncharacterized protein LOC113111007	XM_005155938.5
A8G0215	3.91	14.32	ANAPC7	Anaphase-promoting complex subunit 7	NM_001113585.1
B16G0590	3.91	15.88	A33	Zinc-binding protein A33	NM_001109704.1
A15G0368	3.91	0.89	POLR1G	DNA-directed RNA polymerase I subunit RPA34	
B17G0902	3.90	9.30	IFT43A	Intraflagellar transport protein 43 homolog A	XM_005173583.5
A24G0020	3.90	12.54	L3MBTL4	Lethal(3)malignant brain tumor-like protein 4	XR_660045.4
B6G0273	3.90	6.65	ICA1L	Islet cell autoantigen 1-like protein	XM_009302228.4
A7G1006	3.90	2.35	IRS1-B	Insulin receptor substrate 1-B	XM_021477766.2
B22G0043	3.90	17.96	NLRC3	Protein NLRC3	XM_068216562.1
B23G0408	3.90	17.93	UBR4	E3 ubiquitin-protein ligase UBR4	XM_021473658.2
B7G0614	3.90	3.31	SALL1	Sal-like protein 1	NM_001146014.1
B1G0143	3.90	9.29	MIEF1	Mitochondrial dynamics protein MID51	NM_001076648.2
A23G0843	3.90	5.34	FBXL5	LRR-repeat protein 5	NM_001301328.1
A17G0122	3.90	4.37	TRBV7-9	T cell receptor beta variable 7-9	
B8G0984	3.90	14.41	FUT9	4-galactosyl-N-acetylglucosaminide 3-alpha-L-fucosyltransferase 9	NM_001007454.2
A23G0877	3.90	4.84	PLOD3	Multifunctional procollagen lysine hydroxylase and glycosyltransferase LH3	NM_001044343.2
B24G0339	3.90	4.84	MYNN	Myoneurin	XM_005162774.5
B21G0035	3.90	2.76	POGLUT3	Protein O-glucosyltransferase 3	
B22G0051	3.90	16.20	NLRC3	NLR family CARD domain-containing protein 3	XM_068216562.1
A25G0394	3.90	0.76	IL17RA	Interleukin-17 receptor A	XM_017354312.2
A5G1005	3.90	6.84	SYMPK	Symplekin	XM_694383.9
A13G0092	3.90	8.30	LDLRAP1-B	Low density lipoprotein receptor adapter protein 1-B	XM_068213134.1
B12G0423	3.90	6.28	SNX11	Sorting nexin-11	XM_021480062.2
A7G0534	3.90	4.65	CHPF2	Chondroitin sulfate glucuronyltransferase	XM_001337169.7
A5G0145	3.90	3.59	DDR2	Discoidin domain-containing receptor 2	XM_021474018.2

B23G0939	3.90	6.28	TTC39B	Tetratricopeptide repeat protein 39B	XM_068216969.1
A14G0520	3.90	6.67	HNRNPH2	Heterogeneous nuclear ribonucleoprotein H2	XM_005169579.5
B24G0256	3.90	8.93	RSU1	Ras suppressor protein 1	XM_021470373.2
A8G0954	3.90	0.46	G5714_008782	A0A7J6CQL0_9TELE THAP-type domain-containing protein	
B17G0595	3.89	6.52	ARHGAP5	Rho GTPase-activating protein 5	XM_001335980.6
A14G0191	3.89	5.90	FOXO4	Forkhead box protein O4	XM_009291168.4
B19G0735	3.89	7.50	PPP1R11	E3 ubiquitin-protein ligase PPP1R11	NM_001017627.1
B11G0610	3.89	7.60	G5714_011669	A0A7J6CL58_9TELE Uncharacterized protein	XM_003201652.6
A2G0255	3.89	5.68	DLG1L	Discs large homolog 1-like protein	XM_009296951.4
B12G0380	3.89	2.00	MTPAP	Poly(A) RNA polymerase, mitochondrial	XM_687164.9
B7G0598	3.89	3.97	CNEP1R1	Nuclear envelope phosphatase-regulatory subunit 1	NM_001017874.1
A14G0045	3.89	9.58	UBE2K	Ubiquitin-conjugating enzyme E2 K	NM_001013482.1
B17G0346	3.89	5.53	CDK1	Cyclin-dependent kinase 1	NM_212564.2
B15G0071	3.89	0.61	CLDN4	Claudin-4	
A23G0690	3.89	4.64	NFE2L2	Nuclear factor erythroid 2-related factor 2	XM_068216670.1
B1G0986	3.89	3.12	CENPM	Centromere protein M	
B7G0390	3.89	12.54	KATNB1	Katanin p80 WD40 repeat-containing subunit B1	NM_213018.1
B10G0611	3.89	3.91	MSI2	RNA-binding protein Musashi homolog 2	NM_001320102.1
B14G0485	3.88	2.90	ABHD18	Protein ABHD18	NM_001013347.2
B5G1012	3.88	8.43	TRAFD1	TRAF-type zinc finger domain-containing protein 1	NM_001089515.1
A25G0331	3.88	6.25	CEP290	Centrosomal protein of 290 kDa	NM_001168267.1
B23G0269	3.88	7.54	SOX18	Transcription factor Sox-18	XM_017353772.3
B19G0783	3.88	8.30	POL	Retrovirus-related Pol polyprotein from transposon 297	XR_011017212.1
B6G0014	3.88	1.36	FDX2	Ferredoxin-2, mitochondrial	
B16G0352	3.88	5.68	PIK3R4	Phosphoinositide 3-kinase regulatory subunit 4	XM_005158299.5
A1G0378	3.88	3.18	ARHGEF7	Rho guanine nucleotide exchange factor 7	XM_021474150.2
A3G0858	3.88	3.99	NUDT9	ADP-ribose pyrophosphatase, mitochondrial	NM_213352.2
A5G0011	3.88	12.23	MBD2	Methyl-CpG-binding domain protein 2	NM_212768.1
A10G0880	3.88	9.71	APBB1	Amyloid beta precursor protein binding family B member 1	XM_003199317.6
A2G1070	3.88	5.50	CACTIN	Cactin	XM_005168431.5
B3G0893	3.88	3.59	BAX	Apoptosis regulator BAX	
B17G0114	3.88	2.17	TATDN3	Putative deoxyribonuclease TATDN3	NM_001200022.1
B16G0817	3.88	8.37	ZNF865	Zinc finger protein 865	XM_021472965.2
B24G0369	3.88	5.05	PHLDB2	Pleckstrin homology-like domain family B member 2	XM_021470325.2
B5G0633	3.88	2.98	LOC113049053	A0A6P6K715_CARAU uncharacterized protein LOC113049053	
A6G0281	3.88	2.83	GMPPAB	Mannose-1-phosphate guanyltransferase alpha-B	NM_200497.1
B13G0272	3.88	1.68	CDC25A	M-phase inducer phosphatase 1	XM_005156901.5

B23G0021	3.88	3.27	MTG2	Mitochondrial ribosome-associated GTPase 2	XM_001336598.8
B13G0456	3.88	11.65	GSTO1	Glutathione S-transferase omega-1	NM_200317.1
B4G0920	3.88	9.02	NLRC3	Protein NLRC3	XM_068213794.1
A17G0063	3.88	16.76	TMEM30A	Cell cycle control protein 50A	NM_212776.2
A7G0294	3.88	6.10	SPG7	Paraplegin	XM_001923083.7
B20G0682	3.88	4.11	UCKL1	Uridine-cytidine kinase-like 1	XM_002665748.4
B3G1458	3.87	3.10	LOC113097005	A0A6P6PBS8_CARAU zinc finger protein 845-like	NM_001013496.1
B16G0117	3.87	3.92	SATB1	DNA-binding protein SATB1	XM_017351562.3
B18G0497	3.87	4.62	PIAS1	E3 SUMO-protein ligase PIAS1	XM_021474413.2
A6G0344	3.87	6.56	SETD5	Histone-lysine N-methyltransferase SETD5	XM_693742.9
B20G0047	3.87	6.13	MAP3K5	Mitogen-activated protein kinase kinase kinase 5	NM_001161750.1
B24G0668	3.87	3.46	APBB1IP	Amyloid beta A4 precursor protein-binding family B member 1-interacting protein	NM_200634.1
B23G0956	3.87	11.97	PLOD3	Multifunctional procollagen lysine hydroxylase and glycosyltransferase LH3	NM_001044343.2
A3G0195	3.87	3.04	TMEM220	Transmembrane protein 220	NM_001328099.1
A24G0160	3.87	4.24	COASY	Bifunctional coenzyme A synthase	NM_001004577.1
A4G1011	3.87	1.14			
A7G1069	3.87	2.57	TAF6L	CBP-associated factor-associated factor 65 kDa subunit 6L	NM_001199722.1
A24G0077	3.87	4.00	DCUN1D3	DCN1-like protein 3	NM_001103112.2
B7G1267	3.87	15.87	NDRG2	Protein NDRG2	XM_005160380.5
A18G0367	3.87	3.97	MALT1	Mucosa-associated lymphoid tissue lymphoma translocation protein 1	XM_694813.10
A13G0710	3.87	3.27	BAG5	BAG family molecular chaperone regulator 5	NM_174862.3
A4G0193	3.87	10.46	NLRP3	NACHT, LRR and PYD domains-containing protein 3	NM_001423275.1
A7G0694	3.87	12.88	PKP3	Plakophilin-3	XM_002662838.6
B1G0596	3.87	5.60	GALNT7	N-acetylgalactosaminyltransferase 7	NM_001020641.1
B3G1196	3.87	5.91	SP140	Nuclear body protein SP140	XM_678217.8
A20G0852	3.87	3.76	UFL1	E3 UFM1-protein ligase 1	NM_200606.1
B24G0395	3.87	2.19	CHODL	Chondrolectin	NM_001017578.2
A7G0311	3.87	3.60	CAMK2D1	calmodulin-dependent protein kinase type II delta 1 chain	XM_021477468.2
B11G0193	3.87	3.28	CRTC1	CREB-regulated transcription coactivator 1	NM_001002191.1
A5G1129	3.87	2.10	TMEM203	Transmembrane protein 203	NM_001002519.1
B18G0730	3.86	3.99	WASHC1	WASH complex subunit 1	NM_001110450.1
B25G0337	3.86	3.91	TBC1D2B	TBC1 domain family member 2B	XM_002666866.7
B12G0312	3.86	3.07	ETV4	ETS translocation variant 4	NM_131425.2
A3G0882	3.86	9.99	STAT5B	Signal transducer and activator of transcription 5B	NM_194387.2
A21G0782	3.86	4.16	BNIP1	Vesicle transport protein SEC20	XM_001333689.9
A20G0260	3.86	3.25	REPS1	RalBP1-associated Eps domain-containing protein 1	NM_001045056.2
B11G0246	3.86	4.59	BCL2L15	Bcl-2-like protein 15	NM_001110030.1
B21G0414	3.86	4.63	DCK.2	Deoxycytidine kinase 2	NM_001014352.2
A6G0694	3.86	9.57	CRBN	Protein cereblon	NM_001003996.1

B22G0631	3.86	110.21	TPM4	Tropomyosin alpha-4 chain	XM_017353110.2
A13G0278	3.86	3.31	RTF1	RNA polymerase-associated protein RTF1 homolog	XM_021480766.2
B11G0721	3.86	6.30	SLC29A1	Equilibrative nucleoside transporter 1	
B21G0790	3.86	10.29	OLFM1	Noelin	NM_001328415.1
A7G0783	3.86	4.78	VRK3	threonine-protein kinase VRK3	NM_001013568.3
A2G0200	3.86	2.97	TBL1XR1	WD repeat-containing protein TBL1XR1	NM_001326540.1
A25G0214	3.86	5.19	SLC7A5	Large neutral amino acids transporter small subunit 1	NM_001128358.1
A3G0758	3.86	0.86	CLEC19A	C-type lectin domain family 19 member A	NM_001328076.1
A5G0664	3.86	5.60	MICU2	Calcium uptake protein 2, mitochondrial	NM_001017539.2
B22G0118	3.86	4.50	LOC113075279	A0A6P6N435_CARAU zinc finger protein 271-like isoform X3	NM_001003622.2
A7G0590	3.86	10.23	ATG13	Autophagy-related protein 13	XM_005168970.5
A24G0662	3.86	5.71	CUL2	Cullin-2	XM_021470256.2
A5G1280	3.86	2.48	RILPL2	RILP-like protein 2	XM_005168657.5
B23G0148	3.86	10.14	MGAT4C	Alpha-1,3-mannosyl-glycoprotein 4-beta-N-acetylglucosaminyltransferase C	
A3G0875	3.86	12.94	DHX58	ATP-dependent RNA helicase DHX58	NM_001257157.1
B24G0092	3.86	4.13	DCUN1D3	DCN1-like protein 3	NM_001103112.2
B3G0525	3.85	1.57	SNRNP25	U12 small nuclear ribonucleoprotein 25 kDa protein	NM_001017690.1
B25G0323	3.85	1.05	GCNT3	Beta-1,3-galactosyl-O-glycosyl-glycoprotein beta-1,6-N-acetylglucosaminyltransferase 3	
A15G0833	3.85	7.44	POR	NADPH--cytochrome P450 reductase	NM_001320439.1
B6G0345	3.85	1.69	IGFBP2A	Insulin-like growth factor-binding protein 2-A	NM_131458.2
B1G0948	3.85	5.00	RFX1	MHC class II regulatory factor RFX1	XM_021477396.2
B23G0844	3.85	7.71	MYL9	Myosin regulatory light polypeptide 9	NM_001006027.1
B7G0812	3.85	2.22	ALX4	Homeobox protein aristaless-like 4	XM_001340930.7
B2G0103	3.85	4.66	RNF126	E3 ubiquitin-protein ligase RNF126	NM_001083017.2
B18G0773	3.85	1.58	KCTD14	POZ domain-containing protein KCTD14	XM_687949.8
A14G0273	3.85	10.02	CYFIP2	Cytoplasmic FMR1-interacting protein 2	NM_001097587.1
B1G0282	3.85	1.04	FRG1	Protein FRG1	NM_001017793.2
A16G0166	3.85	2.19	SIGLEC1	Sialoadhesin	
B3G1397	3.85	7.03	KLHL24	Kelch-like protein 24	XM_003198186.6
A16G0283	3.85	2.27	TMEM42	Transmembrane protein 42	NM_001386405.1
A10G0438	3.85	7.75	VPS26BL	Vacuolar protein sorting-associated protein 26B-like	NM_001002415.2
A23G0607	3.85	4.83	PAN2	PAN2-PAN3 deadenylation complex catalytic subunit PAN2	XM_005162345.4
A25G0469	3.85	3.25	PTPRZ1	Receptor-type tyrosine-protein phosphatase zeta	XM_002666879.7
B3G1542	3.85	6.27	G5714_002838	A0A7J6D7S9_9TELE Uncharacterized protein	
B5G0594	3.85	6.18	TENT2	Poly(A) RNA polymerase GLD2	NM_001020600.1

A20G0274	3.85	1.11	MPV17	Protein Mpv17	NM_201165.2
A21G0770	3.85	2.29	EOLA1	Protein EOLA1	NM_001017643.1
B8G0131	3.85	1.38	PRICKLE3	Prickle planar cell polarity protein 3	XM_693557.9
A3G1142	3.85	13.51	SREBF1	Sterol regulatory element-binding protein 1	NM_001105129.1
A4G0573	3.85	5.60	CEP83	Centrosomal protein of 83 kDa	NM_001353931.1
B16G0905	3.85	0.26	HOXA10B	Homeobox protein Hox-A10b	NM_131155.2
B16G0712	3.85	2.16	TLR12	Toll-like receptor 12	XM_002664846.7
B25G0524	3.85	8.00	ELMO3	Engulfment and cell motility protein 3	XM_009297958.4
A2G0101	3.85	2.68	CYP8B1	5-beta-cholestane-3-alpha,7-alpha-diol 12-alpha-hydroxylase	NM_001003736.1
B5G1049	3.84	4.55	RABL6	Rab-like protein 6	XM_021476477.2
B16G0839	3.84	6.16	GBA1	Lysosomal acid glucosylceramidase	XM_068214099.1
B19G0734	3.84	6.50	G4	Uncharacterized protein C6orf47 homolog	NM_001423403.1
B19G0483	3.84	12.83	KIAA0319L	Dyslexia-associated protein KIAA0319-like protein	XM_003200638.6
B20G0622	3.84	4.77	CDC42BPA	threonine-protein kinase MRCK alpha	XM_068215521.1
B6G0633	3.84	8.87	HERC2	E3 ubiquitin-protein ligase HERC2	XM_021471809.2
A16G0577	3.84	85.71	JARID2	Protein Jumonji	XM_001345849.8
B17G0170	3.84	9.68	ZRANB1B	Ubiquitin thioesterase zranb1-B	NM_001077768.1
B24G0448	3.84	4.90	CDK8	Cyclin-dependent kinase 8	NM_194408.2
A14G0240	3.84	5.60	DLG3	Disks large homolog 3	XM_068218767.1
A3G0326	3.84	5.97	DNAJB1	DnaJ homolog subfamily B member 1	NM_001003571.1
A9G0271	3.84	5.50	MED14	Mediator of RNA polymerase II transcription subunit 14	XM_005167822.4
B18G0523	3.84	7.52	IL34	Interleukin-34	NM_001128701.1
B20G0517	3.84	9.25	CEP43	Centrosomal protein 43	NM_001002125.1
B25G0222	3.84	5.16	TSG101	Tumor susceptibility gene 101 protein	NM_001002089.1
B15G0689	3.84	2.39	N4BP2L2	NEDD4-binding protein 2-like 2	XM_688657.9
A19G0079	3.84	7.89	CITED3	p300-interacting transactivator 3	
A25G0028	3.84	0.80	CDKN1B	Cyclin-dependent kinase inhibitor 1B	NM_212792.2
B7G0169	3.84	1.16	GALNT18	Polypeptide N-acetylgalactosaminyltransferase 18	XM_003199013.6
B4G0669	3.84	1.62	A0A671K3I7	A0A671K3I7_9TELE Uncharacterized protein	XR_011011350.1
B8G0760	3.84	7.48	RPE65B	All-trans-retinyl ester 13-cis isomerohydrolase	NM_001113653.1
A21G0204	3.84	6.97	NEDD4L	E3 ubiquitin-protein ligase NEDD4-like	XM_068216002.1
A4G0637	3.83	2.42	RECQL	ATP-dependent DNA helicase Q1	XM_005164761.5
B18G0543	3.83	5.12	PHAF1	Phagosome assembly factor 1	NM_001082813.1
B21G0475	3.83	10.12	DLAT	Dihydrolipoyllysine-residue acetyltransferase component of pyruvate dehydrogenase complex, mitochondrial	NM_212667.2
B18G0450	3.83	3.89	TSNAXIP1	Translin-associated factor X-interacting protein 1	NM_001100100.1
B16G0503	3.83	3.81	LYSMD1	LysM and putative peptidoglycan-binding domain-containing protein 1	NM_001076750.1

A7G0622	3.83	11.12	TENT4B	Terminal nucleotidyltransferase 4B	NM_001326696.1
A21G0594	3.83	8.55	SH2D4A	SH2 domain-containing protein 4A	NM_001003543.2
A18G0439	3.83	5.33	CTTNBP2	Cortactin-binding protein 2	XM_021467802.2
B1G0183	3.83	2.80	INTS15	Integrator complex subunit 15	XM_005170851.5
A10G0301	3.83	0.08	BHLHA9	Class A basic helix-loop-helix protein 9	
A14G0297	3.83	4.96	SYTL4	Synaptotagmin-like protein 4	XM_009291117.4
A20G0529	3.83	7.09	NRBP1	Nuclear receptor-binding protein	NM_001045189.2
B16G1110	3.83	3.51	PPP1R8	Nuclear inhibitor of protein phosphatase 1	NM_201200.1
A25G0272	3.83	9.54	TCP11L1	T-complex protein 11-like protein 1	NM_001014301.2
A19G0751	3.83	7.03	NEDD9	Enhancer of filamentation 1	NM_001077282.1
A3G0955	3.83	4.23	ATXN7L3	Ataxin-7-like protein 3	NM_001040341.2
A10G0124	3.83	1.38	BCL7A	lymphoma 7 protein family member A	
A11G0033	3.83	3.96	ORC2	Origin recognition complex subunit 2	NM_152979.2
B21G0123	3.83	1.73	MSXD	Homeobox protein MSH-D	NM_131276.3
B22G0389	3.82	1.74	LOC113076556	A0A6P6N9V2_CARAU uncharacterized protein LOC113076556	XM_068216570.1
A7G0946	3.82	5.63	CNFN	Cornifelin	NM_001327905.1
A9G0275	3.82	3.78	CD247	T-cell surface glycoprotein CD3 zeta chain	NM_001100107.1
A23G0734	3.82	4.66	RNF146	E3 ubiquitin-protein ligase rnf146	NM_199854.2
B19G0028	3.82	5.27	NLRC3	NLR family CARD domain-containing protein 3	XM_021468229.2
B19G0480	3.82	5.19	SLC17A5	Sialin	XM_005159769.5
B11G0033	3.82	3.81	MKRN2	E3 ubiquitin-protein ligase makorin-2	NM_001077304.1
A18G0317	3.82	6.93	CLASRP	arginine rich protein	NM_001044329.1
A18G0446	3.82	10.79	DENND11	DENN domain-containing protein 11	NM_001044347.1
A5G0960	3.82	4.91	BRD3	Bromodomain-containing protein 3	XM_005165593.5
A10G0688	3.82	5.17	SNX24	Sorting nexin-24	NM_001033731.1
A25G0355	3.82	2.47	TDG	T mismatch-specific thymine DNA glycosylase	NM_001020751.1
B8G0154	3.82	13.76	EGR3	Early growth response protein 3	NM_001002647.1
A19G0252	3.82	4.73	BAG6	Large proline-rich protein BAG6	XM_017352270.3
B25G0358	3.82	5.52	WASL	Actin nucleation-promoting factor WASL	NM_001083006.1
B4G0226	3.81	2.33	LOC113058903	A0A6P6LE90_CARAU uncharacterized protein LOC113058903	
A21G0731	3.81	4.68	MFF	Mitochondrial fission factor	XM_068215745.1
B24G0340	3.81	6.81	APOD	Apolipoprotein D	NM_001037695.1
B2G0972	3.81	2.34	MTF2	Metal-response element-binding transcription factor 2	NM_001045264.1
A4G0493	3.81	3.17	USP44	Ubiquitin carboxyl-terminal hydrolase 44	NM_200257.1
A25G0781	3.81	3.97	ZNF276	Zinc finger protein 276	XM_021470427.2
B3G1530	3.81	1.79	A0A673M8N8	A0A673M8N8_9TELE Uncharacterized protein	XM_068217670.1
A18G0117	3.81	4.04	BCL9L	lymphoma 9-like protein	XM_005159238.5

A6G0773	3.81	14.60	ARL8BA	ADP-ribosylation factor-like protein 8B-A	NM_205566.1
B15G0416	3.81	3.75	CPI17	Protein phosphatase 1 regulatory subunit 14A	NM_200810.1
B19G0668	3.81	5.85	ZBTB10	Zinc finger and BTB domain-containing protein 10	NM_001128539.1
A10G0311	3.81	4.40	PHF12	PHD finger protein 12	XM_001919920.8
A14G0281	3.81	4.31	LCP2	Lymphocyte cytosolic protein 2	XM_021474329.2
A8G0438	3.81	3.74	GID8A	Glucose-induced degradation protein 8-A homolog	XM_017357294.3
A3G0927	3.81	5.26	A0A673M2R6	A0A673M2R6_9TELE Uncharacterized protein	NM_199815.1
A18G0356	3.81	36.52	TSPAN3	Tetraspanin-3	NM_001025538.2
A8G0016	3.81	6.74	DMXL1	DmX-like protein 1	XM_021471068.1
B13G0907	3.80	4.49			XM_678958.10
B10G0133	3.80	5.10			
A17G0226	3.80	3.86	MTA1	Metastasis-associated protein MTA1	XM_001333237.8
B18G0011	3.80	1.35	P2RY1	P2Y purinoceptor 1	XM_001343319.8
B9G0323	3.80	3.92	F2RL1	Proteinase-activated receptor 2	XM_017357833.3
B5G0166	3.80	3.13	CHEK2	threonine-protein kinase Chk2	NM_200045.1
A10G0499	3.80	3.19	FAM89B	Leucine repeat adapter protein 25	XM_001921384.7
B24G0492	3.80	2.86	SULF1	Extracellular sulfatase Sulf-1	NM_001003846.2
A16G0481	3.80	9.81	ASGR2	Asialoglycoprotein receptor 2	XM_009292397.4
A22G0186	3.80	14.97	LOC113092160	A0A6P6NY37_CARAU uncharacterized protein LOC113092160 isoform X2	XM_068216590.1
A17G0202	3.80	6.20	UBR1	E3 ubiquitin-protein ligase UBR1	XM_021467517.2
B22G1039	3.80	3.38	IFI44L	Interferon-induced protein 44-like	
A19G0844	3.80	8.16	CFAP20	Cilia- and flagella-associated protein 20	XM_005173723.5
B19G0748	3.80	4.39	TMEM268	Transmembrane protein 268	
A11G0411	3.80	10.79	IQSEC1	IQ motif and SEC7 domain-containing protein 1	XM_068224343.1
A10G0686	3.80	7.10	GMCL1	Germ cell-less protein-like 1	NM_207050.1
B8G0636	3.80	2.77	DFFB	DNA fragmentation factor subunit beta	NM_194404.1
B24G0030	3.80	8.71	TRIM16	Tripartite motif-containing protein 16	XM_681071.8
B2G0979	3.80	2.62	CDC7	Cell division cycle 7-related protein kinase	XM_005171160.5
A19G0791	3.80	2.52	LOC115829920	A0A6J2X091_CHACN zinc finger protein 436-like	XM_005159346.5
A7G0951	3.80	1.19	CNFN-A	Cornifelin homolog A	
B6G0035	3.80	2.45	FAM83F	Protein FAM83F	NM_001007320.3
B9G0366	3.80	4.49	PIKFYVE	1-phosphatidylinositol 3-phosphate 5-kinase	XM_005167670.5
B1G0250	3.80	5.64	HPF1	Histone PARylation factor 1	NM_001005769.1
B10G0374	3.79	7.46	TMEM135	Transmembrane protein 135	NM_001089418.1
A18G0044	3.79	4.08	ST3GAL4	CMP-N-acetylneuraminate-beta-galactosamide-alpha-2,3-sialyltransferase 4	NM_001083029.1
B12G0434	3.79	9.05	JOSD1	Josephin-1	NM_001076595.2
A11G0743	3.79	6.99	TP53RK	KEOPS complex subunit TP53RK	NM_001080642.1
B13G0561	3.79	1.08	CEFIP	Cardiac-enriched FHL2-interacting protein	NM_131280.2

B13G0642	3.79	2.52	LOC107654912	A0A671QSA1_9TELE Uncharacterized LOC107654912	NM_001115095.2
B1G0585	3.79	3.94	TMEM184C	Transmembrane protein 184C	NM_001145596.1
B17G0800	3.79	6.41	BABAM2	BRISC and BRCA1-A complex member 2	NM_001017777.1
B7G0443	3.79	5.72	SIN3A	Paired amphipathic helix protein Sin3a	XM_002662877.7
B7G0932	3.79	5.35	DGKD	Diacylglycerol kinase delta	XM_005172317.5
A10G0381	3.79	2.95	RIPK4	threonine-protein kinase 4	XM_068223759.1
B18G0649	3.79	7.56	FGD4	FYVE, RhoGEF and PH domain-containing protein 4	NM_001423694.1
A18G0593	3.79	3.17	GAN	Gigaxonin	XM_003200434.6
A24G0034	3.79	2.78	TRIM16	Tripartite motif-containing protein 16	XM_681071.8
B21G0974	3.79	0.94	NUP54	Nucleoporin p54	XM_021468762.2
A16G0056	3.79	8.77	ATP6V1C1A	V-type proton ATPase subunit C 1-A	NM_201322.1
A14G0576	3.79	2.66	GUF1	Translation factor GUF1, mitochondrial	XM_009307691.4
B20G0912	3.79	6.29	RTASE	Probable RNA-directed DNA polymerase from transposon BS	
A6G0681	3.79	10.42	LOC107735491	A0A673G4K5_9TELE Beta-1,4-N-acetylgalactosaminyltransferase	
B11G0382	3.79	4.71	ATP2B4	Plasma membrane calcium-transporting ATPase 4	NM_200333.1
B6G0069	3.79	3.94	PLEKHG4B	Pleckstrin homology domain-containing family G member 4B	XM_068221648.1
B18G0168	3.79	22.87	SCG3	Secretogranin-3	NM_200757.1
B13G0356	3.79	1.33	ZSCAN29	Zinc finger and SCAN domain-containing protein 29	NM_001004626.1
A3G1103	3.79	5.54	CBX4	E3 SUMO-protein ligase CBX4	NM_205749.1
B12G0475	3.79	9.41	MGRN1	Probable E3 ubiquitin-protein ligase MGRN1	NM_001328544.1
A11G0562	3.79	5.16	CACNA2D3	delta-3	XM_009306167.4
A14G0702	3.79	2.10	JOCKEY\POL	RNA-directed DNA polymerase from mobile element jockey	XM_068217263.1
A8G0631	3.79	13.63	OXCT1	Succinyl-CoA:3-ketoacid coenzyme A transferase 1, mitochondrial	NM_001007291.1
B15G0763	3.79	4.95	C3AR1	C3a anaphylatoxin chemotactic receptor	XM_068213639.1
B8G0840	3.79	3.71	TRAF2	TNF receptor-associated factor 2	NM_001128381.1
B17G0224	3.78	12.68	RALGAPA1	Ral GTPase-activating protein subunit alpha-1	XM_021467316.2
A6G0477	3.78	2.68	STK25	threonine-protein kinase 25	XM_005165925.5
B3G1010	3.78	6.86	C17ORF75	Protein Njmu-R1	NM_001045389.1
B19G0334	3.78	13.00	IGKV1-6	Immunoglobulin kappa variable 1-6	XM_068217596.1
A12G0369	3.78	7.23	MTA3	Metastasis-associated protein MTA3	XM_068224467.1
B1G0771	3.78	5.98	ARHGEF7	Rho guanine nucleotide exchange factor 7	NM_001008624.1
B7G0397	3.78	3.23	ZNF280D	Zinc finger protein 280D	XM_005166525.5
B13G0045	3.78	19.51	RTASE	Probable RNA-directed DNA polymerase from transposon BS	XM_686064.9
B4G0642	3.78	3.80	TMEM19	Transmembrane protein 19	NM_001114472.1
B25G0579	3.78	2.16	MPPED2	Metallophosphoesterase MPPED2	NM_200042.2

A6G0679	3.78	0.54	CHCHD4-A	Mitochondrial intermembrane space import and assembly protein 40-A	
B22G0215	3.78	7.27	LOC113040278	A0A6P6J976_CARAU uncharacterized protein LOC113040278	XM_068216570.1
B16G0966	3.78	3.68	MRS2	Magnesium transporter MRS2 homolog, mitochondrial	XM_688529.9
A5G0142	3.78	5.97	GAK	Cyclin-G-associated kinase	XM_009301214.4
A10G0485	3.78	7.88	CXADR	Coxsackievirus and adenovirus receptor homolog	NM_152948.2
B23G0965	3.78	2.72	PPP1R35	Protein phosphatase 1 regulatory subunit 35	NM_001045391.1
A24G0285	3.78	3.43	MSL2	E3 ubiquitin-protein ligase MSL2	NM_001163313.1
A2G0264	3.78	8.95	WDR33	pre-mRNA 3' end processing protein WDR33	NM_001194928.1
B13G0118	3.78	19.52	PLA2G12B	Group XIIB secretory phospholipase A2-like protein	NM_001013501.2
A9G0448	3.78	2.85	NABP1	SOSS complex subunit B2	XM_005167556.5
A21G0686	3.78	5.83	NSRP1	Nuclear speckle splicing regulatory protein 1	NM_001077430.2
B5G0979	3.78	5.27	UBASH3B	Ubiquitin-associated and SH3 domain-containing protein B	XM_001923850.7
B8G0546	3.78	2.25	ATXN7L2	Ataxin-7-like protein 2	NM_001002655.2
A11G0328	3.78	6.45	CHIA	Acidic mammalian chitinase	XM_021472481.2
A11G0368	3.78	4.96	ZHX3	Zinc fingers and homeoboxes protein 3	XM_021479533.2
A16G0433	3.78	9.55	PTP4A3	Protein tyrosine phosphatase type IVA 3	
B20G0725	3.78	5.00	CEP85L	Centrosomal protein of 85 kDa-like	XM_005170197.5
B12G0595	3.78	7.04	WAPL	Wings apart-like protein homolog	XM_021480269.2
A7G0869	3.77	15.43	CYP26B1	Cytochrome P450 26B1	NM_212666.1
B2G0204	3.77	12.05	GIGYF2	GRB10-interacting GYF protein 2	NM_001030161.2
B3G0356	3.77	0.92	MAG	Myelin-associated glycoprotein	
B10G0144	3.77	1.76	CERCAM	Probable inactive glycosyltransferase 25 family member 3	XM_009305170.4
B16G0545	3.77	4.70	FAM171A1	Protein FAM171A1	XM_001335895.6
A18G0481	3.77	8.25	ARIH1	E3 ubiquitin-protein ligase arih1	NM_199758.1
B21G0431	3.77	2.47	BICDL1	BICD family-like cargo adapter 1	NM_001365668.1
A11G0516	3.77	3.06	XXYLT1	Xyloside xylosyltransferase 1	NM_001040178.1
A22G0218	3.77	7.16	STAB1	Stabilin-1	NM_001195614.2
B22G0058	3.77	0.94	GIMAP9	GTPase IMAP family member 9	XM_021471487.2
A22G0692	3.77	22.01	FXR1	RNA-binding protein FXR1	NM_201301.2
A11G0181	3.77	5.03	NPEPPS	Puromycin-sensitive aminopeptidase	XM_021479570.2
B6G0741	3.77	7.16	PATZ1	POZ (BTB) and AT hook-containing zinc finger 1	XM_068222027.1
A23G0605	3.77	6.89	ORMDL2	ORM1-like protein 2	NM_001040048.1
B6G0803	3.77	4.38	RYBPB	RING1 and YY1-binding protein B	NM_001012373.2
B16G0346	3.77	6.74	ALDH5A1	Succinate-semialdehyde dehydrogenase, mitochondrial	XM_009292538.4
B20G0302	3.77	3.57	ZNF513	Zinc finger protein 513	NM_001309467.1
A25G0564	3.77	4.15	BANP	Protein BANP	XM_005160361.5
A24G0163	3.77	12.54	RIOK3	threonine-protein kinase RIO3	NM_001003614.2
B25G0386	3.77	6.87	NPTN	Neuroplastin	NM_001160156.2

A13G0327	3.77	1.52	METTL18	Histidine protein methyltransferase 1 homolog	NM_001346162.1
B5G0638	3.77	2.67	EDIL3	EGF-like repeat and discoidin I-like domain-containing protein 3	XM_005165250.5
B10G0334	3.77	5.02	CADM2	Cell adhesion molecule 2	XM_005155264.5
A7G0151	3.77	3.48	CCDC149A	Coiled-coil domain-containing protein 149-A	NM_001430977.1
A6G0078	3.77	4.05	ST3GAL3	CMP-N-acetylneuraminate-beta-1,4-galactoside alpha-2,3-sialyltransferase	
A12G0663	3.77	6.57	EEF2K	Eukaryotic elongation factor 2 kinase	XM_005156067.5
A13G0471	3.77	4.55	ADAM8	Disintegrin and metalloproteinase domain-containing protein 8	NM_001077379.1
B16G0553	3.76	3.72	TBRG4	FAST kinase domain-containing protein 4	XM_685771.9
B14G0453	3.76	2.78	CLP1	Polyribonucleotide 5'-hydroxyl-kinase Clp1	NM_001346238.1
A23G0284	3.76	1.85	PRIM1	DNA primase small subunit	NM_201448.1
B8G0601	3.76	7.55	GID8A	Glucose-induced degradation protein 8-A homolog	XM_017357294.3
A21G0764	3.76	6.08	CHML	Rab proteins geranylgeranyltransferase component A 2	NM_203461.1
B18G0857	3.76	0.82	BNC1	Zinc finger protein basonuclin-1	XM_009300994.3
A12G0494	3.76	4.55	PGP	Glycerol-3-phosphate phosphatase	NM_212726.2
A21G0004	3.76	5.56	LOC107574301	FMR2 family member 4-like	XM_003200795.6
B12G0528	3.76	10.96	STAMBPL1	AMSH-like protease	XM_694037.7
B10G0585	3.76	0.94	UCP2	Mitochondrial uncoupling protein 2	NM_200353.2
A2G0451	3.76	7.75	LRRC8D	Volume-regulated anion channel subunit LRRC8D	XM_002660814.7
A10G0466	3.76	21.37	PICALM	Phosphatidylinositol-binding clathrin assembly protein	XM_009305392.4
A10G0046	3.76	6.45	TRIAP1	TP53-regulated inhibitor of apoptosis 1	NM_001159820.3
A24G0301	3.76	4.12	MYNN	Myoneurin	XM_005162774.5
A2G0070	3.76	8.81	SSX2IP	Afadin- and alpha-actinin-binding protein	NM_200835.2
A1G1092	3.76	2.65	LOC107757586	A0A673HPT7_9TELE Transmembrane protein 154-like	
A21G0257	3.76	7.44	NPDC1	Neural proliferation differentiation and control protein 1	NM_001128783.1
B16G0241	3.76	10.65	SLC4A7	Sodium bicarbonate cotransporter 3	XM_068214223.1
A5G0783	3.76	4.87	ATXN2	Ataxin-2	XM_005165355.5
A24G0333	3.76	6.70	NUP58	p45	XM_005162681.4
A3G1035	3.76	3.19	BAIAP2L2	Brain-specific angiogenesis inhibitor 1-associated protein 2-like protein 2	XM_021473971.2
B22G0541	3.76	3.21	ARHGEF18	Rho guanine nucleotide exchange factor 18	XM_068216437.1
B4G0370	3.76	3.38	ZCRB1	Zinc finger CCHC-type and RNA-binding motif-containing protein 1	NM_001017589.2
B22G0388	3.76	11.43	LOC113079612	A0A6P6NEN4_CARAU uncharacterized protein LOC113079612	XM_068216617.1

B12G0418	3.76	6.99	MXRA7	Matrix-remodeling-associated protein 7	
B5G0477	3.76	9.71	NAPA	Alpha-soluble NSF attachment protein	NM_001083561.2
B21G0683	3.76	4.11	EDEM3	ER degradation-enhancing alpha-mannosidase-like protein 3	XM_003200833.6
B5G1163	3.76	7.11	TPCN1	Two pore channel protein 1	XM_005165220.4
B23G0556	3.76	5.67	B4GALNT1	Beta-1,4 N-acetylgalactosaminyltransferase 1	XM_021469816.2
A15G0703	3.75	2.89	PRCP	Lysosomal Pro-X carboxypeptidase	NM_001002694.1
A10G0689	3.75	3.68	LOX	Protein-lysine 6-oxidase	NM_207089.1
B6G0598	3.75	2.48	RGS16	Regulator of G-protein signaling 16	NM_001017846.1
A4G0977	3.75	8.38	ANO6	Anoctamin-6	XM_005164462.5
B10G0682	3.75	5.59	TMEM120B	Transmembrane protein 120B	XM_005169222.4
B17G0389	3.75	2.11	TTC27	Tetratricopeptide repeat protein 27	NM_173285.2
B11G0244	3.75	3.57	SGTA	Small glutamine-rich tetratricopeptide repeat-containing protein alpha	NM_001030143.1
A6G0260	3.75	3.57	ICA1	Islet cell autoantigen 1	XM_009302224.4
A5G0214	3.75	6.49	KAT6A	Histone acetyltransferase KAT6A	NM_001123312.4
A24G0476	3.75	5.51	SOCS6	Suppressor of cytokine signaling 6	XM_681949.9
B16G0106	3.75	9.05	TCB1	Transposable element Tcb1 transposase	
B12G0318	3.75	4.19	TMEM106B	Transmembrane protein 106B	NM_001128676.1
B25G0271	3.75	7.12	STOML1	Stomatin-like protein 1	NM_001110032.1
B20G0841	3.75	3.94	PREP	Prolyl endopeptidase	NM_001020753.1
B16G0894	3.75	2.07	FKBP14	Peptidyl-prolyl cis-trans isomerase FKBP14	NM_001003471.1
B3G0515	3.75	3.15	DOCK6	Dedicator of cytokinesis protein 6	XM_068219443.1
B6G0839	3.75	5.65	G5714_007294	A0A7J6CZX2_9TELE Uncharacterized protein	XM_002662526.7
B16G0686	3.75	3.45	NBN	Nibrin	NM_001014819.1
A9G0406	3.75	0.60	DDB_G0268948	Putative methyltransferase DDB_G0268948	NM_001044784.1
B20G0751	3.75	4.65	LIN9	Protein lin-9 homolog	NM_001044946.1
B5G0466	3.75	4.14	ALKBH4	Alpha-ketoglutarate-dependent dioxygenase alkB homolog 4	NM_001326370.1
A16G0013	3.75	5.26	CREB3L4	Cyclic AMP-responsive element-binding protein 3-like protein 4	XM_021466785.2
B10G0486	3.75	8.51	ATG101	Autophagy-related protein 101	NM_001423721.1
B2G0836	3.75	8.97	AFG3L2	AFG3-like protein 2	NM_001111197.1
B21G0592	3.75	7.78	RAI14	Ankycorbin	NM_001111217.2
B9G0651	3.74	4.29	CEP250	Centrosome-associated protein CEP250	XM_021478844.2
A17G0579	3.74	7.08	REEP3	Receptor expression-enhancing protein 3	NM_001037575.2
B6G0589	3.74	2.18	IL12RB2	Interleukin-12 receptor subunit beta-2	XM_021476742.2
B16G0122	3.74	1.14	H2-AA	H-2 class II histocompatibility antigen, A-K alpha chain	
B7G1171	3.74	3.57	METTL3	N6-adenosine-methyltransferase subunit METTL3	NM_212780.1
A10G0110	3.74	0.54	AHR	Aryl hydrocarbon receptor	

A20G0358	3.74	8.99	KIFAP3	Kinesin-associated protein 3	NM_001004644.1
B22G0742	3.74	5.24	RGMB	Repulsive guidance molecule B	XM_021469302.2
B7G0946	3.74	5.22	TIFA	TRAF-interacting protein with FHA domain-containing protein A	NM_001386546.1
B9G0715	3.74	5.55	IRS2	Insulin receptor substrate 2	XM_695654.10
A24G0534	3.74	2.59	MYCA	Transcriptional regulator Myc-1	NM_131412.1
A7G0608	3.74	4.87	NUDT19	Acyl-coenzyme A diphosphatase NUDT19	NM_001128786.1
A14G0080	3.74	6.09	GNPDA1	Glucosamine-6-phosphate isomerase 1	NM_001017867.1
B3G0818	3.74	7.93	NBR1	Next to BRCA1 gene 1 protein	NM_001386643.1
A15G0480	3.74	2.56	POL	Retrovirus-related Pol polyprotein from transposon opus	
A20G0179	3.74	14.11	GPCPD1	Glycerophosphocholine phosphodiesterase GPCPD1	NM_001110523.1
A24G0075	3.74	6.93	COX6B1	Cytochrome c oxidase subunit 6B1	NM_001002695.3
B8G0281	3.74	10.77	KDM5C	Lysine-specific demethylase 5C	NM_001123234.1
A14G0351	3.74	3.05	CSTF2	Cleavage stimulation factor subunit 2	XM_009291098.4
B14G0282	3.74	4.57	LCP2	Lymphocyte cytosolic protein 2	XM_021474328.2
B24G0539	3.74	5.95	SOCS6	Suppressor of cytokine signaling 6	XM_681949.9
B25G0803	3.74	1.94	RAB19	Ras-related protein Rab-19	XM_678213.9
A16G0551	3.74	9.04	DPX16_20361	A0A3N0XI57_ANAGA Kinesin-like protein KIF26A	
A2G0936	3.73	2.85	A0A674PP12	A0A674PP12_TAKRU Uncharacterized protein	XM_693558.9
B12G0453	3.73	5.62	HSD3B	Delta 5-->4-isomerase	XM_001345321.7
A4G0860	3.73	2.82	MDFIC	MyoD family inhibitor domain-containing protein	NM_001328013.1
A3G0594	3.73	4.90	WIPI1	WD repeat domain phosphoinositide-interacting protein 1	NM_200391.1
B13G0106	3.73	5.66	JOCKEY\POL	RNA-directed DNA polymerase from mobile element jockey	NM_001002198.1
A8G0264	3.73	4.81	CEP350	Centrosome-associated protein 350	XM_068223113.1
B13G0237	3.73	6.82	TMEM150A	Transmembrane protein 150A	XM_021480882.2
B13G0279	3.73	5.11	RPIA	Ribose-5-phosphate isomerase	NM_213411.2
B2G1293	3.73	4.72	ZGC:174263	X1WG44_DANRE Zgc:174263	NM_001114314.2
A19G0742	3.73	1.36	CLEC4F	C-type lectin domain family 4 member F	
B14G0409	3.73	7.76	NOCT	Nocturnin	XM_695702.9
A12G0202	3.73	1.86	SFXN2	Sideroflexin-2	NM_212730.1
A20G0716	3.73	7.49	FMO5	Flavin-containing monooxygenase 5	NM_198910.1
B21G0026	3.73	6.22	TRIM16	Tripartite motif-containing protein 16	XM_681071.8
A16G0983	3.73	7.00	CSF3R	Granulocyte colony-stimulating factor receptor	XM_005173451.5
B13G0903	3.73	0.59			XM_021472600.2
B6G0070	3.73	8.90	TGM2	Protein-glutamine gamma-glutamyltransferase 2	NM_212656.2
B2G0765	3.72	2.18	NCEH1	Neutral cholesterol ester hydrolase 1	NM_001002303.2
A9G0050	3.72	1.70	ARHGAP6	Rho GTPase-activating protein 6	XM_017354543.3
B14G0827	3.72	4.30	TC1A	Transposable element Tc1 transposase	
A21G0715	3.72	3.90	RAB24	Ras-related protein Rab-24	NM_001029970.2

B1G0201	3.72	4.34	TMEM55BB	Type I phosphatidylinositol 4,5-bisphosphate 4-phosphatase-B	NM_001159921.1
B8G0664	3.72	3.81	MFSD12	Major facilitator superfamily domain-containing protein 12	NM_001423440.1
B13G0739	3.72	5.27	LOC107702739	A0A671QKQ0_9TELE Tetratricopeptide repeat protein 31-like	NM_001005939.2
A7G0237	3.72	8.56	DENND5A	DENN domain-containing protein 5A	XM_009303572.4
A25G0759	3.72	7.69	G5714_024498	A0A7J6BJX9_9TELE Uncharacterized protein	
B1G0514	3.72	2.98	PPP1R9A	Neurabin-1	XM_009292002.4
B10G0102	3.72	1.50	VLDLR	Very low-density lipoprotein receptor	XM_005155561.5
B1G1073	3.72	1.25	PIGR	Polymeric immunoglobulin receptor	XM_021475896.2
B17G0460	3.72	2.82	LOC107721138	A0A673L1J0_9TELE Glutamate-rich protein 1-like	XM_068214593.1
B25G0599	3.72	5.03	PLA2G15	Phospholipase A2 group XV	NM_001386706.1
A21G0028	3.72	4.43	PRAG1	Inactive tyrosine-protein kinase PRAG1	XM_068216167.1
B5G1086	3.72	3.74	MFSD3	Major facilitator superfamily domain-containing protein 3	XM_002661995.7
B5G1117	3.72	21.39	ATP6V1A	V-type proton ATPase catalytic subunit A	NM_201135.2
B24G0677	3.72	4.33			
B7G0394	3.72	2.99	MYZAP	Myocardial zonula adherens protein	XM_678415.9
A23G0801	3.72	0.42	LOC113065813	A0A6P6MB54_CARAU uncharacterized protein LOC113065813	
B20G0034	3.72	2.59	L3MBTL3	Lethal(3)malignant brain tumor-like protein 3	NM_001245964.1
A23G0773	3.72	3.20	ZNF217	Zinc finger protein 217	NM_001351640.1
A23G0757	3.72	6.04	VWA1	von Willebrand factor A domain-containing protein 1	
B6G0559	3.72	4.12	ATG4C	Cysteine protease ATG4C	NM_001002103.1
B12G0456	3.71	9.43	TNRC6B	Trinucleotide repeat-containing gene 6B protein	XM_068224758.1
B8G0257	3.71	0.32	NEURL3	E3 ubiquitin-protein ligase NEURL3	
A6G0990	3.71	17.40	HELZ2	Helicase with zinc finger domain 2	XM_003198847.5
A18G0036	3.71	8.62	LTBP4	Latent-transforming growth factor beta-binding protein 4	XM_021467926.2
A15G0233	3.71	4.01	BLOC1S3	Biogenesis of lysosome-related organelles complex 1 subunit 3	NM_001025476.1
B7G0162	3.71	4.98	DPX16_10341	A0A3N0Y4V5_ANAGA Adrenomedullin	NM_001326709.1
B10G0070	3.71	3.79	SLC46A2	Thymic stromal cotransporter homolog	XM_017357996.3
B6G0943	3.71	1.60	ABHD14B	Putative protein-lysine deacylase ABHD14B	NM_200367.1
A24G0005	3.71	6.92	SNX16	Sorting nexin-16	
B5G0187	3.71	7.80	WSB2	WD repeat and SOCS box-containing protein 2	XM_017356013.3
B11G0383	3.71	5.09	EPYC	Epiphycan	NM_001044753.1
A22G0639	3.71	3.08	LOC107567310	A0A672JY86_SINGR Uncharacterized LOC107567310	

B19G0166	3.71	1.07	KHDRBS3	KH domain-containing, RNA-binding, signal transduction-associated protein 3	XM_068217882.1
B2G0613	3.71	3.55	FASTK	threonine kinase	NM_001170819.1
A5G0742	3.71	8.81	CNOT6L	CCR4-NOT transcription complex subunit 6-like	XM_005165315.5
A18G0779	3.71	7.92	CYP1A1	Cytochrome P450 1A1	NM_131879.2
B2G0123	3.71	2.08			
A24G0052	3.71	8.18	LOC113076586	A0A6P6N795_CARAU uncharacterized protein LOC113076586	XM_068217268.1
B20G0291	3.71	4.63	CCM2	Cerebral cavernous malformations protein 2 homolog	NM_001002315.3
B11G0397	3.71	3.70	CHIA	Acidic mammalian chitinase	XM_002663788.7
A24G0627	3.71	3.76	CCNY	Cyclin-Y	XM_678927.9
A19G0135	3.71	8.31	TCB1	Transposable element Tcb1 transposase	XM_021471168.2
A15G0145	3.71	27.52	LOC113080887	A0A6P6NJS2_CARAU uncharacterized protein LOC113080887 isoform X1	
A16G0756	3.71	6.07	UBE2S	Ubiquitin-conjugating enzyme E2 S	NM_001025536.1
A1G0372	3.71	13.84	CAB39L	Calcium-binding protein 39-like	XM_005170986.5
B5G1088	3.70	4.24	TRPM6	Transient receptor potential cation channel subfamily M member 6	NM_001251831.1
B5G0057	3.70	12.73	GUSB	Beta-glucuronidase	XM_009301129.4
A16G0390	3.70	1.11	CALHM6	Calcium homeostasis modulator protein 6	NM_001079661.1
A5G0444	3.70	3.57	RAD17	Cell cycle checkpoint protein RAD17	NM_001431112.1
A12G0372	3.70	2.85	RHOQ	Rho-related GTP-binding protein RhoQ	NM_199818.1
B16G0119	3.70	4.94	PLCL2	Inactive phospholipase C-like protein 2	XM_002667679.6
A25G0239	3.70	2.78	ZNRF1	E3 ubiquitin-protein ligase znrf1	NM_200870.1
B2G0438	3.70	8.25	REEP6	Receptor expression-enhancing protein 6	NM_001013536.2
A3G1184	3.70	5.76	PECAM1	Platelet endothelial cell adhesion molecule	XM_021471101.2
B3G0243	3.70	3.04	ATG4DA	Cysteine protease atg4da	XM_009299158.4
B15G0208	3.70	5.85	MRE11	Double-strand break repair protein MRE11	NM_001320259.1
B11G0454	3.70	2.74	KBP	Probable glutamate receptor	
A23G0165	3.70	3.99	EDEM2	ER degradation-enhancing alpha-mannosidase-like protein 2	NM_001305614.1
A1G1116	3.70	3.07	TOPORS	E3 ubiquitin-protein ligase Topors	
B15G0196	3.70	3.29	DMAC2	Distal membrane-arm assembly complex protein 2	NM_001386288.1
A9G0232	3.70	2.02	SLC49A4	Solute carrier family 49 member 4	NM_001004597.1
B5G0938	3.70	1.06	P2RX1	P2X purinoceptor 1	NM_198982.1
B14G0527	3.70	4.39	RUFY1	RUN and FYVE domain-containing protein 1	XM_002667315.7
A3G0962	3.70	4.77	SPOP	Speckle-type POZ protein	NM_201130.2
B20G0847	3.70	11.27	LOC107745905	A0A673FLS6_9TELE Cell wall protein RBR3-like	XM_001342341.6
A1G1049	3.70	5.26	KDM4C	Lysine-specific demethylase 4C	NM_001083851.1

A14G0508	3.70	3.04	ABCA1	Phospholipid-transporting ATPase ABCA1	XM_005173080.5
A1G0522	3.70	4.25	ING2	Inhibitor of growth protein 2	NM_001002448.1
B2G0302	3.70	5.59	TRIM16	Tripartite motif-containing protein 16	NM_001045270.2
A2G0380	3.70	5.51	AGFG1	Arf-GAP domain and FG repeat-containing protein 1	NM_200588.2
B16G1079	3.70	4.94	CSF3R	Granulocyte colony-stimulating factor receptor	NM_001113377.1
B1G0780	3.70	5.85	RCBTB1	RCC1 and BTB domain-containing protein 1	XM_005170970.5
B17G0287	3.69	3.60	TDP1	Tyrosyl-DNA phosphodiesterase 1	XM_695082.8
B7G1065	3.69	2.73	TAF6L	CBP-associated factor-associated factor 65 kDa subunit 6L	NM_001199722.1
B13G0122	3.69	3.25	MRPS6	28S ribosomal protein S6, mitochondrial	NM_001431064.1
A16G0339	3.69	3.90	DCBLD1	Discoidin, CUB and LCCL domain-containing protein 1	XM_021473022.2
B22G0847	3.69	6.03	RRBP1	Ribosome-binding protein 1	NM_199431.2
B8G0437	3.69	2.80	HSD17B3	17-beta-hydroxysteroid dehydrogenase type 3	XM_005167148.5
A15G0838	3.69	2.36	RSRC1	Arginine-related protein 53	NM_212947.2
B1G1139	3.69	2.15	LOC113099775	A0A6P6PJG2_CARAU zinc finger protein 883-like isoform X2	NM_001122845.1
B7G0884	3.69	4.27	GBA2	Non-lysosomal glucosylceramidase	XM_005172332.5
B21G0193	3.69	9.20	SPRY2	Protein sprouty homolog 2	XM_021473441.2
B23G0346	3.69	2.97	PARD6B	Partitioning defective 6 homolog beta	NM_001102675.1
A13G0607	3.69	6.80	LAMTOR3	Ragulator complex protein LAMTOR3	XM_068224920.1
A13G0184	3.69	1.96	WDR89	WD repeat-containing protein 89	XM_021472628.2
A7G1087	3.69	1.09	KVM5	Ig kappa chain V region Mem5 (Fragment)	NM_131649.2
A8G0946	3.69	10.24	NR2C2	Nuclear receptor subfamily 2 group C member 2	XM_005167355.5
A15G0680	3.69	3.52	MIPEP	Mitochondrial intermediate peptidase	XM_068213680.1
B22G0356	3.69	8.31	SMARCD1	SNF-related matrix-associated actin-dependent regulator of chromatin subfamily D member 1	NM_198358.1
B9G0913	3.69	0.60	MS4A15	Membrane-spanning 4-domains subfamily A member 15	
B13G0049	3.69	3.40	A33	Zinc-binding protein A33	NM_001020687.1
B18G0210	3.69	6.61	G5714_018025	A0A7J6C4C3_9TELE Uncharacterized protein	XM_068214915.1
B1G0864	3.69	3.16	ATG4DA	Cysteine protease atg4da	NM_001423806.1
B22G0336	3.69	1.90	SLAMF9	SLAM family member 9	XM_068216413.1
B8G0100	3.69	5.06	K2013	Uncharacterized protein KIAA2013 homolog	NM_001113612.1
A21G0761	3.69	55.19	SRSF1B	arginine-rich splicing factor 1B	XR_659427.4
B20G0469	3.68	4.20	INO80	Chromatin-remodeling ATPase INO80	NM_001045119.1
B14G0576	3.68	2.48	PCGF1	Polycomb group RING finger protein 1	NM_001007158.2
A21G0465	3.68	4.25	CCDC90B	Coiled-coil domain-containing protein 90B, mitochondrial	NM_001166258.2

B23G0811	3.68	3.20	NFATC2	Nuclear factor of activated T-cells, cytoplasmic 2	XM_003201225.6
B4G0456	3.68	12.27	CHPT1	Cholinephosphotransferase 1	NM_212624.1
A4G0992	3.68	3.96	TULP3	Tubby-related protein 3	XR_011011405.1
B3G0854	3.68	4.00	PRRG2	Transmembrane gamma-carboxyglutamic acid protein 2	NM_001202480.1
B20G0379	3.68	4.93	MDN1	Midasin	XM_003200703.6
A17G0249	3.68	16.89	CMPK2	UMP-CMP kinase 2, mitochondrial	XM_693963.9
B15G0927	3.68	4.17	PAFAH1B2	Platelet-activating factor acetylhydrolase IB subunit alpha2	
B5G0621	3.68	12.41	FAM172A	Cotranscriptional regulator FAM172A homolog	NM_001431106.1
A20G0834	3.68	12.27	RHOB	Rho-related GTP-binding protein RhoB	
A20G0473	3.68	7.00	VPS18	Vacuolar protein sorting-associated protein 18 homolog	NM_173245.2
B7G1015	3.68	1.42	ANPEP	Aminopeptidase N	XM_001922952.8
A7G0742	3.68	4.69	ZNF710	Zinc finger protein 710	XM_005168921.5
A10G0362	3.68	1.81	OVCA2	Esterase OVCA2	NM_001020555.1
B23G0731	3.68	9.75	LOC107714991	A0A673HQG5_9TELE Uncharacterized LOC107714991	
B3G0707	3.68	4.48	ITGA3	Integrin alpha-3	XM_021474550.2
B5G0574	3.68	13.94	SCARB2	Lysosome membrane protein 2	NM_173259.1
B15G0257	3.68	5.40	GBE1	1,4-alpha-glucan-branching enzyme	XM_068213679.1
B7G0243	3.68	5.37	SPG7	Paraplegin	XM_001923083.7
A17G0001	3.68	2.33	LOC107745417	A0A673HNR1_9TELE Gastrula zinc finger protein XlCGF26.1-like	XM_068220220.1
B5G0994	3.68	4.52	NELFB	Negative elongation factor B	NM_200168.1
A9G0702	3.68	4.47	NDUFV3	NADH dehydrogenase [ubiquinone] flavoprotein 3, mitochondrial	
B16G0990	3.67	5.99	ANO10	Anoctamin-10	NM_001030206.1
B12G0403	3.67	3.93	NSF	Vesicle-fusing ATPase	NM_001354163.1
B18G0468	3.67	3.32	LOC113118398	A0A6P6RCW4_CARAU uncharacterized protein LOC113118398	NM_001044713.2
B11G0507	3.67	7.55	NR0B1	Nuclear receptor subfamily 0 group B member 1	
A3G0853	3.67	1.00	LOC113047144	A0A6P6JYU1_CARAU uncharacterized protein LOC113047144	XM_002661170.6
B23G0526	3.67	5.44	DTX3	Probable E3 ubiquitin-protein ligase DTX3	NM_200528.3
A9G0375	3.67	4.63	ABI3BP	Target of Nesh-SH3	XM_068223647.1
A15G0238	3.67	4.08	DHX16	Pre-mRNA-splicing factor ATP-dependent RNA helicase DHX16	NM_200024.1
B7G0051	3.67	2.82	GPANK1	A0A673HQ08_9TELE G-patch domain and ankyrin repeats 1	
A25G0136	3.67	3.98	PEAK1	Inactive tyrosine-protein kinase PEAK1	XM_001921567.7
A7G0958	3.67	2.33	MB21D2	Nucleotidyltransferase MB21D2	NM_001327862.1
A19G0808	3.67	4.00	NECTIN2	Nectin-2	NM_001430892.1
B5G1107	3.67	4.83	ATP6V0A2	V-type proton ATPase 116 kDa subunit a 2	NM_001122747.2
B5G0704	3.67	3.45	ALKBH2	DNA oxidative demethylase ALKBH2	NM_001256684.1

A12G0239	3.67	5.86	RAB40B	Ras-related protein Rab-40B	NM_001002524.1
A24G0685	3.67	5.23	RECK	Reversion-inducing cysteine-rich protein with Kazal motifs	NM_001423853.1
B21G0294	3.67	2.68	CNOT6	CCR4-NOT transcription complex subunit 6	XM_009295585.4
B5G1061	3.67	1.47	OCLN	Occludin	NM_001008618.1
A1G0371	3.67	4.61	CDADC1	Cytidine and dCMP deaminase domain-containing protein 1	XM_005170988.5
B1G0749	3.67	2.71	SENS	Zinc finger protein sens	XM_021477008.2
B12G0802	3.67	6.99	ANTXR1	Anthrax toxin receptor 1	XM_687270.8
B13G0712	3.67	60.46	SYNE2	Nesprin-2	XM_017358772.3
B25G0189	3.67	0.36	IGKV1-27	Immunoglobulin kappa variable 1-27	XM_017354192.3
A18G0448	3.67	4.54	IGHMBP2	DNA-binding protein SMUBP-2	NM_001044900.1
A23G0609	3.67	4.89	TRAPPC6B	Trafficking protein particle complex subunit 6b	NM_001006029.1
A22G0022	3.66	5.82	TMEM240	Transmembrane protein 240	XM_003200912.6
B1G1091	3.66	9.11	GGACT.2	Gamma-glutamylaminecyclotransferase B	
B1G1133	3.66	3.38	LOC113099796	A0A6P6PHG1_CARAU zinc finger protein 271-like	XM_017357165.3
B22G0673	3.66	8.42	NFIA	Nuclear factor 1 A-type	XM_068216299.1
B17G0561	3.66	10.72	ITSN2	Intersectin-2	XM_005158778.5
B6G0111	3.66	4.41	RBM26	RNA-binding protein 26	XM_002662263.7
B8G0432	3.66	12.00	DOCK8	Dedicator of cytokinesis protein 8	XM_001920072.8
B13G0545	3.66	2.99	SAR1B	GTP-binding protein SAR1b	XM_009307279.4
B22G0093	3.66	2.30	LOC113075279	A0A6P6N435_CARAU zinc finger protein 271-like isoform X3	NM_001003622.2
A23G0369	3.66	0.79	CCDC30	Coiled-coil domain-containing protein 30	XM_068216893.1
B14G0604	3.66	3.57	RGS14	Regulator of G-protein signaling 14	XM_009307644.4
B6G0441	3.66	5.61	PIK3R6	Phosphoinositide 3-kinase regulatory subunit 6	XM_009302373.4
B6G0170	3.66	6.24	NFIL3	Nuclear factor interleukin-3-regulated protein	NM_001197058.1
B15G0321	3.66	4.66	TRAF4	TNF receptor-associated factor 4	NM_205762.1
A3G0679	3.66	6.12	LOC113045461	A0A6P6JP74_CARAU zinc finger protein 524-like	NM_001020712.1
A7G0936	3.66	3.65	AKAP11	A-kinase anchor protein 11	XM_001339501.8
B7G0795	3.66	4.48	TRIM66	Tripartite motif-containing protein 66	XM_017357045.3
A12G0205	3.66	3.89	MFSD13A	Transmembrane protein 180	XM_017358451.3
B7G0610	3.66	2.02	NOD2	Nucleotide-binding oligomerization domain-containing protein 2	NM_001328044.1
A13G0414	3.66	4.79	RIMS1	Regulating synaptic membrane exocytosis protein 1	XM_068213046.1
B14G0331	3.66	5.38	FBXO38	F-box only protein 38	XM_005169601.5
A1G0258	3.66	1.49	APTX	Aprataxin	NM_214729.1
A20G0120	3.66	2.56	TRAM2	Translocating chain-associated membrane protein 2	NM_213071.1
A3G1242	3.66	2.82	SMIM10L2A	Small integral membrane protein 10-like protein 2A	NM_001163443.1
B7G0998	3.66	7.80	SLC39A1	Zinc transporter ZIP1	NM_212583.2
B3G1494	3.66	2.37	G5714_004666	A0A7J6D5A0_9TELE Uncharacterized protein	XM_068220086.1
A24G0532	3.66	6.60	OTULIN	Ubiquitin thioesterase otulin	XM_068217164.1

A16G0498	3.66	3.01	RPP38	Ribonuclease P protein subunit p38	
B18G0720	3.66	16.84	DNAJA2	DnaJ homolog subfamily A member 2	NM_212665.1
A13G0091	3.66	4.98	S100PBP	S100P-binding protein	
A1G1080	3.66	6.33	RHOH	Rho-related GTP-binding protein RhoH	
A19G0171	3.66	4.64	XKR8	XK-related protein 8	XM_001922777.7
A6G0541	3.66	2.66	PARS2	Probable proline--tRNA ligase, mitochondrial	
B9G0646	3.66	2.87	MYO10	Unconventional myosin-X	XM_009304520.4
B21G0924	3.65	2.49	RIC1	Guanine nucleotide exchange factor subunit RIC1	XM_068217721.1
A8G0175	3.65	4.97	ELK1	ETS domain-containing protein Elk-1	XM_691202.8
A2G1012	3.65	4.29	GPSM2	G-protein-signaling modulator 2	XM_005171366.5
B1G0836	3.65	1.20	LEF1	Lymphoid enhancer-binding factor 1	XM_068222889.1
B16G0716	3.65	10.10	SHE	SH2 domain-containing adapter protein E	XM_684895.9
A12G0629	3.65	1.98	ADAM9	Disintegrin and metalloproteinase domain-containing protein 9	NM_001114439.1
A17G0457	3.65	5.08	OXR1	Oxidation resistance protein 1	XM_021473161.2
A16G0864	3.65	9.25	ZYX	Zyxin	NM_001109824.2
B3G0054	3.65	2.62	LOC113075489	A0A6P6N4M8_CARAU zinc finger protein 271-like	XM_021469419.2
A1G0679	3.65	8.92	DNAJB14	DnaJ homolog subfamily B member 14	NM_001077787.1
A2G0864	3.65	1.54	LOC113119913	A0A6P6RIA1_CARAU myosin-11-like isoform X1	
B9G0575	3.65	5.78	TRIM13	Tripartite motif-containing 13	NM_001020603.2
A3G0525	3.65	3.77	CLN3	Battenin	NM_001007306.1
B5G1051	3.65	4.11	CCDC117	Coiled-coil domain-containing protein 117	NM_001123279.2
B8G0834	3.65	0.93	DRAM2	DNA damage-regulated autophagy modulator protein 2	
A11G0602	3.65	24.40	AP5B1	AP-5 complex subunit beta-1	NM_200667.2
A15G0325	3.65	5.06	CUEDC1	CUE domain-containing protein 1	XM_068213558.1
B23G0681	3.65	4.52	COQ10B-A	Coenzyme Q-binding protein COQ10 homolog A, mitochondrial	XM_009297030.3
B7G0599	3.65	6.56	ARY1	Arylamine N-acetyltransferase, pineal gland isozyme NAT-10	XM_021477926.2
B16G0755	3.65	9.64	NECTIN2	Nectin-2	XM_021466855.2
B20G0120	3.65	4.31	LGALS8	Galectin-8	XM_003200667.6
A17G0227	3.65	5.20	CRIP2	Cysteine-rich protein 2	NM_213497.2
A7G1211	3.65	7.13	MFAP3L	Microfibrillar-associated protein 3-like	NM_001328051.1
B8G0494	3.65	3.97	AMT	Aminomethyltransferase, mitochondrial	NM_001006021.1
A5G0323	3.65	3.46	LAS1L	Ribosomal biogenesis protein LAS1L	NM_001126400.1
B18G0528	3.65	5.31	PEDS1	Plasmanylethanolamine desaturase	XM_005159087.5
B18G0367	3.65	8.94	YAP1	Transcriptional coactivator YAP1	NM_001139480.1
A23G0522	3.65	6.15	ACVR1	Activin receptor type-1	NM_153643.1
B19G0474	3.65	7.14	PITHD1	PITH domain-containing protein 1	NM_207074.1

B6G0100	3.65	4.91	TLK1B	threonine-protein kinase tousled-like 1-B	NM_001024951.2
A7G0460	3.64	16.80	IGF2	Insulin-like growth factor II	NM_131433.1
B1G0273	3.64	3.59	SGCZ	Zeta-sarcoglycan	XM_017355993.3
B20G0219	3.64	1.68	FASLG	Tumor necrosis factor ligand superfamily member 6	NM_001042701.2
A4G0857	3.64	6.69	CDK17	Cyclin-dependent kinase 17	NM_001201357.1
B13G0503	3.64	2.69	B3GAT2	Galactosylgalactosylxylosylprotein 3-beta-glucuronosyltransferase 2	NM_214782.2
A23G0633	3.64	6.75	POU6F1	POU domain, class 6, transcription factor 1	XM_068216663.1
B13G0480	3.64	5.29	PDLIM1	PDZ and LIM domain protein 1	XM_009307316.4
B9G0487	3.64	1.94	INTS6	Integrator complex subunit 6	NM_001126428.1
A13G0577	3.64	1.63	ABCG5	ATP-binding cassette sub-family G member 5	NM_001145617.1
A2G1093	3.64	4.49	TRIM25	ISG15 ligase TRIM25	NM_001039719.2
B18G0612	3.64	6.59	SELENOO1	Protein adenylyltransferase SelO-1, mitochondrial	NM_001044871.3
B16G0559	3.64	2.81	RBM12B	RNA-binding protein 12B	
A2G0805	3.64	5.30	IFI44	Interferon-induced protein 44	NM_001077169.2
A3G1158	3.64	9.64	HELZ	Probable helicase with zinc finger domain	NM_213266.1
B13G0176	3.64	6.48	EPCAM	Epithelial cell adhesion molecule	XM_017358845.3
A8G0494	3.64	15.27	GNAT2	Guanine nucleotide-binding protein G(t) subunit alpha-2	NM_131869.2
B22G0446	3.64	9.41	LOC113073075	A0A6P6MZJ3_CARAU uncharacterized protein LOC113073075 isoform X1	XM_009295967.4
A5G1288	3.64	4.12	MAPKAPK5	MAP kinase-activated protein kinase 5	NM_001002336.1
A21G0361	3.64	3.41	BCL11B	leukemia 11B	XM_683166.10
B3G0942	3.64	14.22	PACSIN1B	Protein kinase C and casein kinase substrate in neurons protein 1	XM_692166.9
A7G0524	3.64	2.82	ZGC:92818	UPF0547 protein C16orf87 homolog	NM_001002512.2
A1G0546	3.64	6.62	FAM193A	Protein FAM193A	NM_001328395.1
A6G0282	3.64	4.21	RTASE	Probable RNA-directed DNA polymerase from transposon BS	XM_068220297.1
B11G0006	3.64	1.99	ELMO2	Engulfment and cell motility protein 2	XM_021479923.2
A21G0471	3.64	11.06	DLAT	Dihydrolipoyllysine-residue acetyltransferase component of pyruvate dehydrogenase complex, mitochondrial	NM_212667.2
A4G0614	3.63	2.81	SCO2	Protein SCO2 homolog, mitochondrial	
B24G0553	3.63	6.92	NCOA2	Nuclear receptor coactivator 2	XM_009297336.4
A18G0094	3.63	1.62	LARGE2	Xylosyl- and glucuronyltransferase LARGE2s	XM_021467636.2
B10G0623	3.63	1.13	LOC107661940	A0A671N9N2_9TELE Uncharacterized LOC107661940	
B3G1437	3.63	4.82	STK19	threonine-protein kinase 19	
B5G0035	3.63	3.71	LOC107687229	A0A671M681_9TELE Transmembrane protein 127-like	
B6G1004	3.63	5.52	RALGAPB	Ral GTPase-activating protein subunit beta	XM_009302762.4
B15G0059	3.63	6.46	DOCK10	Dedicator of cytokinesis protein 10	XM_001341012.9

A6G0594	3.63	1.98	HSD17B7	17-beta-hydroxysteroid dehydrogenase 7	NM_001077328.1
A5G0694	3.63	5.99	KLHL13	Kelch-like protein 13	XM_005165272.5
A13G0722	3.63	5.43	AIFM2	Ferroptosis suppressor protein 1	XM_017358845.3
A8G0653	3.63	3.29	HPD	4-hydroxyphenylpyruvate dioxygenase	NM_001003742.1
B5G0713	3.63	162.78	EEF2	Elongation factor 2	XM_021476571.2
A5G1050	3.63	0.92	POU2F3	POU domain, class 2, transcription factor 3	NM_001045255.2
A9G0361	3.63	3.88	ZBTB11	Zinc finger and BTB domain-containing protein 11	XM_682097.8
B8G0444	3.63	2.18	PRF1	Perforin-1	XM_005167173.5
B11G0642	3.63	10.76	KLHDC8A	Kelch domain-containing protein 8A	NM_201158.1
A23G0764	3.63	5.18	SRC	Proto-oncogene tyrosine-protein kinase Src	XM_017353519.3
A7G0623	3.63	9.20	ADCY2	Adenylate cyclase type 2	NM_001166272.2
A13G0291	3.62	8.43	KCNF1	Potassium voltage-gated channel subfamily F member 1	NM_205603.1
A4G1034	3.62	3.53	TMEM214-A	Transmembrane protein 214-A	NM_001256176.1
A7G0833	3.62	2.51	MPDU1	Mannose-P-dolichol utilization defect 1 protein	NM_001002130.1
A11G0509	3.62	4.51	COL6A2	Collagen alpha-2(VI) chain	NM_201207.1
A9G0791	3.62	5.53	ATF2	Cyclic AMP-dependent transcription factor ATF-2	XM_009304424.4
A20G0566	3.62	2.78	MDN1	Midasin	
B8G0584	3.62	6.02	CCDC22	Coiled-coil domain-containing protein 22	NM_001025485.1
B2G0287	3.62	42.32	ITGB1-A	Integrin beta-1-A	NM_001034987.1
A20G0258	3.62	5.19	SCARA3	Scavenger receptor class A member 3	XM_005160848.5
A25G0314	3.62	2.49	E2F4	Transcription factor E2F4	NM_213432.2
A2G1045	3.62	10.18	ITM2C	Integral membrane protein 2C	XM_021479096.1
A18G0561	3.62	6.01	RIC8B	Synembryn-B	NM_001005293.1
B11G0491	3.62	5.95	DDB_G0282555	Beta-lactamase-like protein 3	XM_005169363.5
A4G0741	3.62	4.82	IRAK3	Interleukin-1 receptor-associated kinase 3	XM_017355295.3
A10G0458	3.62	5.07	HYOU1	Hypoxia up-regulated protein 1	NM_212703.1
B2G0145	3.62	2.07	THEM6	Protein THEM6	
B19G0373	3.62	4.96	CCDC126	Coiled-coil domain-containing protein 126	NM_001045403.1
B11G0330	3.62	19.66	ID1	DNA-binding protein inhibitor ID-1	NM_213441.1
B6G0366	3.62	14.65	C24G6.8	Probable peptidyl-tRNA hydrolase 2	NM_001326569.1
A8G0702	3.62	2.42	GGT5	Glutathione hydrolase 5 proenzyme	NM_001123298.1
A16G0284	3.62	5.91	ZDHHC3A	Palmitoyltransferase ZDHHC3-A	NM_001002725.1
B15G0333	3.61	2.18	MRM3A	rRNA methyltransferase 3A, mitochondrial	NM_001017695.2
A17G0432	3.61	10.13	FAM167A	Protein FAM167A	XM_021473175.2
A24G0299	3.61	1.95	LRRC31	Leucine-rich repeat-containing protein 31	XM_009297491.4
A1G0307	3.61	7.14	FAM13A	Protein FAM13A	XM_068221730.1
B25G0752	3.61	10.03	NUAK1	NUAK family SNF1-like kinase 1	XM_684898.6
B19G0137	3.61	86.76	K1C1	Keratin, type I cytoskeletal 50 kDa	NM_001017824.2
A21G0086	3.61	8.90	SETX	Probable helicase senataxin	XM_685853.9

A14G0205	3.61	3.31	PABIR1	P2R1A-PPP2R2A-interacting phosphatase regulator 1	XM_005173206.4
B14G0209	3.61	1.17	VGLL1	Transcription cofactor vestigial-like protein 1	NM_001386249.1
B3G1347	3.61	3.75	METTL23	Histone-arginine methyltransferase METTL23	NM_001328039.1
B6G0266	3.61	4.00	GPD2	Glycerol-3-phosphate dehydrogenase, mitochondrial	XM_005165843.5
A13G0528	3.61	10.93	LOC113112767	A0A6P6QPZ4_CARAU Gamma-synuclein	XM_682449.7
B15G0282	3.61	2.77	ZFP36L1	mRNA decay activator protein ZFP36L1	NM_001030247.2
B5G0985	3.61	3.10	DDX31	Probable ATP-dependent RNA helicase DDX31	XM_678740.9
B8G0894	3.61	5.51	USP11	Ubiquitin carboxyl-terminal hydrolase 11	XM_002663073.7
B7G0501	3.61	16.15	VPS35	Vacuolar protein sorting-associated protein 35	NM_001025517.2
B10G0125	3.61	3.83	LIN54	Protein lin-54 homolog	XM_009305164.4
B5G0737	3.61	15.52	RASGEF1BA	Ras-GEF domain-containing family member 1B-A	NM_001431104.1
A15G0520	3.61	2.34	TNFAIP1	POZ domain-containing adapter for CUL3-mediated RhoA degradation protein 2	NM_199799.1
A14G0579	3.61	5.64	SLC25A51	Mitochondrial nicotinamide adenine dinucleotide transporter SLC25A51	NM_001386244.1
B3G1610	3.61	1.23			
B9G0148	3.60	1.93	CXCR1	C-X-C chemokine receptor type 1	XM_068223678.1
B8G0808	3.60	0.87	IGF2	Insulin-like growth factor II	NM_001115050.2
A4G0078	3.60	12.18	LOC113052278	A0A6P6KJQ4_CARAU uncharacterized protein LOC113048991 isoform X1	
B20G0886	3.60	8.14	THBD	Thrombomodulin	
B2G0780	3.60	5.14	MAST3	threonine-protein kinase 3	XM_021471249.2
B13G0214	3.60	5.14	FIGNL1	Fidgetin-like protein 1	XM_009307483.4
A20G0288	3.60	2.85	ANGEL2	Protein angel homolog 2	NM_001029960.1
B22G0184	3.60	6.97	CERS1	Ceramide synthase 1	XM_009295953.4
B10G0239	3.60	60.72	BNIP3L	adenovirus E1B 19 kDa protein-interacting protein 3-like	NM_205571.2
A24G0613	3.60	2.28	DIPK2A	Divergent protein kinase domain 2A	NM_001114926.1
B20G0258	3.60	4.33	TNS3	Tensin-3	NM_001423502.1
B2G0717	3.60	4.11	TGS1	Trimethylguanosine synthase	NM_001423263.1
B3G0279	3.60	4.56	BTNL2	Butyrophilin-like protein 2	XM_068218714.1
B19G0429	3.60	2.57	ZGC:109790	Ribosome biogenesis protein C1orf109 homolog	
B17G0594	3.60	4.15	NUBPL	Iron-sulfur protein NUBPL	NM_001002442.1
B9G0699	3.60	2.91	IM:6907446	Ashwin	NM_001143933.1
A6G0425	3.60	5.73	STX8	Syntaxin-8	NM_200375.1
B9G0775	3.60	11.68	MYCBP2	E3 ubiquitin-protein ligase MYCBP2	XM_009304563.4
B25G0888	3.60	0.00	F2R	Proteinase-activated receptor 1	XM_001334272.9
B1G0387	3.60	9.92	PDS5A	Sister chromatid cohesion protein PDS5 homolog A	XM_068214606.1
A16G0047	3.60	11.52	LOC107659080	A0A671LFZ5_9TELE Uncharacterized LOC107659080	NM_001386446.1
B20G0136	3.60	10.66	FAM89A	Protein FAM89A	
B5G0442	3.60	8.45	RABEP1	Rab GTPase-binding effector protein 1	NM_001365498.1

B18G0597	3.60	6.64	PPFIBP1	Liprin-beta-1	NM_001044970.1
B13G0312	3.60	5.01	ARL3	ADP-ribosylation factor-like protein 3	NM_001123056.1
A25G0352	3.59	2.00			
B24G0359	3.59	5.06	MBTPS2	Membrane-bound transcription factor site-2 protease	NM_001025461.1
B5G0843	3.59	3.38	AR	Androgen receptor	NM_001083123.1
A14G0408	3.59	2.76	RAB33B	Ras-related protein Rab-33B	NM_001033592.1
A9G0365	3.59	3.17	PIGA	Phosphatidylinositol N-acetylglucosaminyltransferase subunit A	NM_001126446.1
A12G0605	3.59	3.90	DROSHA	Ribonuclease 3	NM_001110472.1
A13G0600	3.59	1.56	GNG2	G(O) subunit gamma-2	NM_001006031.1
A16G0072	3.59	0.39	SERPINB1	Leukocyte elastase inhibitor	NM_001109730.1
B17G0888	3.59	5.58	STXBP6	Syntaxin-binding protein 6	NM_001002063.1
B24G0458	3.59	9.57	A0A672KPP2	A0A672KPP2_SINGR AIG1-type G domain-containing protein	
A23G0464	3.59	9.08	ESYT1	Extended synaptotagmin-1	XM_690519.7
A20G0268	3.59	5.08	CAD	CAD protein	NM_001308825.1
B2G1032	3.59	3.72	BAMBI	BMP and activin membrane-bound inhibitor homolog	NM_001002189.1
B7G0980	3.59	47.58	SAA1	Serum amyloid A-1 protein	NM_001005599.2
B4G0390	3.59	9.86	SLC26A5	Prestin	NM_201473.1
B12G0759	3.59	5.13	TCF20	Transcription factor 20	XM_009306365.4
B21G0969	3.59	40.92	BHMT	Betaine--homocysteine S-methyltransferase 1	NM_001012480.2
A7G1326	3.58	4.10	LOC113079659	A0A6P6NHS8_CARAU uncharacterized protein LOC113079659	
B3G1157	3.58	6.54	BPTF	Nucleosome-remodeling factor subunit BPTF	XM_017354770.3
A11G0435	3.58	2.78	IGSF21	Immunoglobulin superfamily member 21	NM_001044324.2
B18G0337	3.58	2.69	SI:DKEY-233H2.2	UPF0692 protein C19orf54 homolog	NM_001077275.2
B2G0113	3.58	6.58	GNA11	Guanine nucleotide-binding protein subunit alpha-11	NM_001007773.1
B8G0652	3.58	8.82	UCK2A	Uridine-cytidine kinase 2-A	NM_001171067.1
A8G0149	3.58	4.63	HDAC6	Histone deacetylase 6	XM_009303752.3
B8G0973	3.58	17.83	MTMR3	Myotubularin-related protein 3	NM_200864.3
A10G0019	3.58	5.39	VASP	Vasodilator-stimulated phosphoprotein	NM_001110452.1
A17G0529	3.58	0.36	HMX3	Homeobox protein HMX3	XM_005158695.5
A2G0459	3.58	3.63	TOX	Thymocyte selection-associated high mobility group box protein TOX	NM_001256694.1
B16G0860	3.58	7.09	RBM12B	RNA-binding protein 12B	NM_001290140.1
A16G0057	3.58	10.07	AZIN1	Antizyme inhibitor 1	NM_001017568.3
A12G0799	3.58	8.31	LRRC45	Leucine-rich repeat-containing protein 45	XM_068224690.1
B8G0701	3.58	12.62	SH3GL1	Endophilin-A2	NM_201497.1
A17G0081	3.58	1.61	YTX2	Transposon TX1 uncharacterized 149 kDa protein	
A10G0383	3.58	2.96	SUPT20H	Transcription factor SPT20 homolog	XM_005172734.4
B11G0188	3.58	3.22	PDE4D	cAMP-specific 3',5'-cyclic phosphodiesterase 4D	NM_001013310.2
B15G0273	3.58	6.62	LOC107752292	A0A673MZD6_9TELE Uncharacterized LOC107752292	NM_001077625.1

B25G0411	3.58	3.56	IMMP2L	Mitochondrial inner membrane protease subunit 2	NM_001003755.2
A14G0380	3.58	1.23	SHISA3	Protein shisa-3 homolog	NM_001080662.2
A3G0443	3.58	2.95	IL21R	Interleukin-21 receptor	
A16G0813	3.57	0.95	MACC1	Metastasis-associated in colon cancer protein 1	NM_001199766.1
A25G0196	3.57	3.04	MR1	Major histocompatibility complex class I-related gene protein	NM_001327918.1
A22G0728	3.57	2.79	SIRT7	NAD-dependent protein deacetylase sirtuin-7	XM_001336402.7
A17G0482	3.57	6.91	XPO1	Exportin-1	XM_009293053.4
B17G0432	3.57	0.73	DYNLT1	Dynein light chain Tctex-type 1	NM_001423210.1
B6G1036	3.57	6.65	TSC22D1	TSC22 domain family protein 1	NM_200574.4
A12G0304	3.57	5.69	OSBPL6	Oxysterol-binding protein-related protein 6	XM_009306609.4
B19G0890	3.57	6.81	GMEB1	Glucocorticoid modulatory element-binding protein 1	XM_005159542.5
B19G0664	3.57	1.03	MRPL53	39S ribosomal protein L53, mitochondrial	
A24G0528	3.57	9.54	VPS41	Vacuolar protein sorting-associated protein 41 homolog	NM_001423787.1
A12G0534	3.57	1.94	SLC16A12B	Monocarboxylate transporter 12-B	NM_001145814.1
A15G0242	3.57	5.52	MGAT1	Alpha-1,3-mannosyl-glycoprotein 2-beta-N-acetylglucosaminyltransferase	NM_200676.1
A22G0057	3.57	3.41	STK38	threonine-protein kinase 38	XM_068216377.1
A10G0696	3.57	3.26	SMTNL1	Smoothelin-like protein 1	XM_003199295.6
A11G0171	3.57	6.95	TRAK1	Trafficking kinesin-binding protein 1	NM_001003752.2
A16G0893	3.57	3.82	SEH1L	Nucleoporin SEH1	NM_199923.2
A25G0604	3.57	2.26	ABTB2	POZ domain-containing protein 2	XM_021473879.2
B9G0558	3.57	6.04	EPB41L5	Band 4.1-like protein 5	NM_200089.2
B25G0469	3.57	9.43	PPIP5K1	Inositol hexakisphosphate and diphosphoinositol-pentakisphosphate kinase 1	XM_021473848.2
B6G0236	3.57	0.84	NFE2L2	Nuclear factor erythroid 2-related factor 2	NM_001257183.1
A17G0684	3.57	7.92	AHCTF1	Protein ELYS	XM_009292870.4
B4G0395	3.57	5.06	ZC3HC1	Zinc finger C3HC-type protein 1	NM_001077378.2
B21G0002	3.57	6.20	CAMLG	Guided entry of tail-anchored proteins factor CAMLG	NM_207090.2
B10G0218	3.56	26.04	YWHAG	14-3-3 protein gamma	NM_213164.2
A16G1018	3.56	2.46	RPA2	Replication protein A 32 kDa subunit	NM_131711.2
A1G0390	3.56	9.27	ATF7IP	Activating transcription factor 7-interacting protein 1	XM_005160041.4
A18G0390	3.56	2.71	LYSMD4	LysM and putative peptidoglycan-binding domain-containing protein 4	NM_200850.2
B3G1408	3.56	3.39	CANT1	Soluble calcium-activated nucleotidase 1	XM_005171642.4
B5G0493	3.56	3.47	TIMM22	Mitochondrial import inner membrane translocase subunit Tim22	NM_001386807.1
B22G0247	3.56	1.41	A0A673LD17	A0A673LD17_9TELE Uncharacterized protein	XM_068216394.1
A9G0482	3.56	10.70	GYPC	Glycophorin-C	NM_001005394.1
B2G0921	3.56	7.75	SH3GLB1	Endophilin-B1	XM_005163307.5

B22G0689	3.56	6.76	IFI44L	Interferon-induced protein 44-like	NM_001128684.1
B6G0202	3.56	8.69	CYP27A1	Sterol 26-hydroxylase, mitochondrial	XM_681430.8
A3G1042	3.56	6.94	GVIN1	Interferon-induced very large GTPase 1	XM_021471422.2
B24G0463	3.56	1.68	QTRT2	Queuine tRNA-ribosyltransferase accessory subunit 2	NM_001118896.2
B24G0479	3.56	5.44	NFI1	CTD small phosphatase-like protein	XM_005162710.5
B23G0535	3.56	5.93	ATF7	Cyclic AMP-dependent transcription factor ATF-7	NM_001030205.1
B7G1061	3.56	2.43	ECSIT	Evolutionarily conserved signaling intermediate in Toll pathway, mitochondrial	NM_001242938.1
A20G0583	3.56	6.73	OCIAD1	OCIA domain-containing protein 1	
A3G0922	3.56	5.99	ZNF652	Zinc finger protein 652	XM_680657.9
A15G0029	3.56	2.57	STARD10	START domain-containing protein 10	NM_205646.2
A4G0047	3.56	6.27	MS4A4A	Membrane-spanning 4-domains subfamily A member 4A	
B3G1386	3.56	10.61	NLRC3	NLR family CARD domain-containing protein 3	XM_068219453.1
A21G0271	3.56	6.13	ARRDC1	Arrestin domain-containing protein 1	NM_001365234.1
A2G0607	3.56	3.49	UBE2W	Ubiquitin-conjugating enzyme E2 W	NM_001122972.1
A16G0835	3.56	0.00	SNX10B	Sorting nexin-10B	NM_001110396.1
A21G0372	3.56	4.14	RNF180	E3 ubiquitin-protein ligase RNF180	NM_001110404.1
A15G0508	3.56	4.99	LOC107717281	A0A673KB88_9TELE Glucuronokinase 1-like	NM_001113616.2
B20G0701	3.56	4.01	PEX7	Peroxisomal targeting signal 2 receptor	XR_011007674.1
B23G0495	3.55	3.38	SENP1	Sentrin-specific protease 1	XM_021473699.2
B16G0723	3.55	9.44	IL6R	Interleukin-6 receptor subunit alpha	NM_001330258.2
B17G0828	3.55	5.02	ZFP292	Zinc finger protein 292	NM_001105141.2
B10G0487	3.55	6.44	RAD51C	DNA repair protein RAD51 homolog 3	XM_021479164.2
B8G0375	3.55	7.43	GAPVD1	GTPase-activating protein and VPS9 domain-containing protein 1	XM_009304093.4
A24G0342	3.55	2.38	MTFR1	Mitochondrial fission regulator 1	
A21G0276	3.55	9.57	ULK1	threonine-protein kinase ULK1	XM_021469039.2
A22G0264	3.55	1.01	KRT222	Keratin-like protein KRT222	XM_001343189.7
B25G0859	3.55	3.86			
B1G0453	3.55	4.73	PSIP1	PC4 and SFRS1-interacting protein	XM_009291548.4
A2G0848	3.55	2.97	APOL3	Apolipoprotein L3	XM_021480586.2
A7G0537	3.55	2.52	GAREM1	GRB2-associated and regulator of MAPK protein 1	NM_200169.2
A24G0313	3.55	6.14	KLHL24	Kelch-like protein 24	NM_200440.1
B4G0487	3.55	0.35			
A24G0469	3.55	3.63	MTRR	Methionine synthase reductase	XM_005162620.4
B17G0422	3.55	3.75	EHBP1	EH domain-binding protein 1	XM_068214587.1
A6G0271	3.55	8.60			

B13G0032	3.55	5.23	LOC113071101	A0A6P6MUK5_CARAU uncharacterized protein LOC113071101 isoform X1	NM_212838.1
B24G0175	3.55	5.75	CPT1A	Carnitine O-palmitoyltransferase 1, liver isoform	XM_017353900.3
A20G0046	3.55	0.98	PAK6	threonine-protein kinase PAK 6	NM_001162487.1
B14G0548	3.55	4.14	PURA	Transcriptional activator protein Pur-alpha	XM_005157161.5
B10G0412	3.55	0.82	RAB1A	Ras-related protein Rab-1A	NM_001002129.2
A15G0716	3.55	1.30	STOM	Stomatin	NM_001098750.1
A7G0345	3.55	5.95	DOK1	Docking protein 1	NM_213088.1
A6G0733	3.55	1.72	ENTPD8	Ectonucleoside triphosphate diphosphohydrolase 8	NM_001002379.2
A8G0153	3.55	3.99	IRAK1	Interleukin-1 receptor-associated kinase 1	XM_005166703.5
B16G0744	3.55	8.73	DDR1	Epithelial discoidin domain-containing receptor 1	XM_009292274.4
B18G0057	3.55	8.50	PID1	PTB-containing, cubilin and LRP1-interacting protein	NM_001013504.2
A23G0266	3.55	13.03	FBXO6	F-box only protein 6	NM_001008577.2
B6G0572	3.55	3.19	TMEM69	Transmembrane protein 69	NM_001287082.1
A14G0775	3.55	4.36	ZGC:66447	SLAIN motif-containing protein-like	NM_199735.1
B16G1094	3.55	1.52	CDCP1	CUB domain-containing protein 1	NM_001014333.1
A7G0979	3.55	4.91			
B22G0794	3.55	2.17	GNAQ	Guanine nucleotide-binding protein G(q) subunit alpha	NM_001003626.2
A1G0578	3.54	7.51	ARL6	ADP-ribosylation factor-like protein 6	NM_001008733.1
A5G0315	3.54	6.34	GAB3	GRB2-associated-binding protein 3	XM_001337679.9
B14G0411	3.54	4.49	G5714_014270	A0A7J6CCA3_9TELE AIF-MLS domain-containing protein	XM_005169620.4
A7G0006	3.54	0.03	TC1A	Transposable element Tc1 transposase	XM_021470094.2
A17G0463	3.54	2.46	COMMD9	COMM domain-containing protein 9	
B8G0533	3.54	6.83	PREX1	Phosphatidylinositol 3,4,5-trisphosphate-dependent Rac exchanger 1 protein	XM_694535.10
B5G0997	3.54	2.33	ARRDC1	Arrestin domain-containing protein 1	NM_001004561.1
A24G0415	3.54	14.77	KPNA1	Importin subunit alpha-5	XM_696267.9
B3G0453	3.54	4.17	LOC113095450	A0A6P6P853_CARAU uncharacterized protein LOC113095450	NM_001013514.3
A2G0213	3.54	7.23	PLSCR1	Phospholipid scramblase 1	NM_001128395.1
B13G0881	3.54	9.71	INPP5A	Inositol polyphosphate-5-phosphatase A	XM_068219469.1
B6G0781	3.54	5.12	SEMA3F	Semaphorin-3F	XM_005166039.4
B6G0410	3.54	3.95	SUZ12B	Polycomb protein suz12-B	XM_068221748.1
B22G0462	3.54	1.84	LOC113100689	A0A6P6PK86_CARAU CD48 antigen-like	XM_001346234.7
B13G0319	3.54	14.01	DCTN1	Dynactin subunit 1	XM_005156774.5
B11G0027	3.54	2.40	ATG7	Ubiquitin-like modifier-activating enzyme ATG7	NM_199832.2
B17G0345	3.54	10.64	ANK3	Ankyrin-3	XM_009293011.4
B13G0353	3.54	6.26	LOC107676263	A0A671QF11_9TELE Vasohibin-2-like	NM_001328052.1
A19G0504	3.54	3.88	NCDN	Neurochondrin	

B3G1327	3.54	5.98	BAIAP2L1	Brain-specific angiogenesis inhibitor 1-associated protein 2-like protein 1	NM_001017830.1
A18G0754	3.54	5.78	GARRE1	Granule associated Rac and RHOG effector protein 1	NM_001423511.1
B12G0774	3.54	1.45	CDR2	Cerebellar degeneration-related protein 2	
A23G0659	3.54	4.77	FBXO25	F-box only protein 25	XM_021469771.2
B2G0343	3.54	5.68	UGGT1	UDP-glucose:glycoprotein glucosyltransferase 1	XM_021480334.2
B2G0943	3.54	12.05	EIF4G1	Eukaryotic translation initiation factor 4 gamma 1	NM_001080200.1
B3G0002	3.54	11.20	BGLAP	Osteocalcin	
B24G0438	3.54	10.45	FLT3	Receptor-type tyrosine-protein kinase FLT3	XM_021470319.2
A7G0992	3.53	24.06	SLC3A2	4F2 cell-surface antigen heavy chain	NM_131601.2
B8G0173	3.53	4.79	CABIN1	Calcineurin-binding protein cabin-1	XM_009304314.4
B12G0203	3.53	2.83	TMEM94	Transmembrane protein 94	XM_021480382.2
B25G0043	3.53	4.74	KIF15-A	Kinesin-like protein KIF15-A	NM_001423817.1
A3G0786	3.53	13.16	TBC1D17	TBC1 domain family member 17	XM_005163947.5
B1G0279	3.53	5.98	MTUS1A	Microtubule-associated tumor suppressor 1 homolog A	XM_021471156.2
B19G0203	3.53	1.75	CDCP1	CUB domain-containing protein 1	
B10G0010	3.53	6.82	SLC12A2	Solute carrier family 12 member 2	NM_001002080.1
A16G0737	3.53	0.31			
B5G0797	3.53	7.96	KLHL13	Kelch-like protein 13	XM_005165272.5
A15G0240	3.53	0.58	RNF225	RING finger protein 225	NM_001076668.1
B3G0069	3.53	0.54	LOC113075477	A0A6P6N4L9_CARAU zinc finger protein OZF-like isoform X2	XM_068214695.1
A14G0423	3.53	3.19	TAPT1	Transmembrane anterior posterior transformation protein 1 homolog	XM_009291279.4
B8G0848	3.53	4.83	ANAPC7	Anaphase-promoting complex subunit 7	NM_001113585.1
B22G0798	3.53	4.05	HDGFL2	Hepatoma-derived growth factor-related protein 2	NM_001002037.2
B2G1294	3.53	1.74			
A8G0012	3.53	4.41	AP3S1	AP-3 complex subunit sigma-1	NM_001005964.1
A4G0704	3.53	1.91	PUS7L	Pseudouridylate synthase PUS7L	NM_001044957.2
B19G0665	3.53	15.19	PAG1	Phosphoprotein associated with glycosphingolipid-enriched microdomains 1	XM_021473350.2
B24G0199	3.53	4.90	ESCO1	N-acetyltransferase ESCO1	XM_005174190.5
A12G0678	3.53	4.01	NRBF2	Nuclear receptor-binding factor 2	NM_001002613.1
A2G0418	3.53	4.94	ODR4	Protein odr-4 homolog	NM_200616.3
A13G0667	3.53	9.36			NM_212581.1
B15G0903	3.52	2.98	CEP57	Centrosomal protein of 57 kDa	NM_001080683.1
B5G0592	3.52	3.91	MTX3	Metaxin-3	NM_205621.2
A6G0853	3.52	1.29	BCL2L14	Apoptosis facilitator Bcl-2-like protein 14	XM_005172116.5
B23G0320	3.52	8.06	PM20D1.2	hydrolase PM20D1.2	NM_001077301.1
A22G0527	3.52	3.49	LPAR3	Lysophosphatidic acid receptor 3	NM_001285534.1
B12G0178	3.52	3.62	LOC113111687	A0A6P6QGS1_CARAU protein FAM104A-like	NM_001328019.1

B18G0789	3.52	14.13	LSM14A	Protein LSM14 homolog A	XM_005158973.4
A13G0859	3.52	2.19	HSP30	Heat shock protein 30	NM_001424033.1
B8G0022	3.52	3.87	CRELD1	Protein disulfide isomerase Creld1	XM_009304364.4
A8G0032	3.52	4.68	LRRC2	Leucine-rich repeat-containing protein 2	
B3G1570	3.52	3.60	LOC113047095	A0A6P6JYI2_CARAU uncharacterized protein LOC113047095	XM_068222460.1
A7G0261	3.52	3.18			NM_001030081.2
B25G0451	3.52	0.24	LADD	Ladderlectin	
B20G0015	3.52	8.03	SLC35A1	CMP-sialic acid transporter	NM_001128811.1
B2G0235	3.52	2.12	NLRP1	NACHT, LRR and PYD domains-containing protein 1 homolog	
B13G0772	3.52	7.98	MRPL14	39S ribosomal protein L14, mitochondrial	NM_213318.1
A3G0382	3.52	4.92	XAB2	Pre-mRNA-splicing factor SYF1	NM_001044783.1
B19G0399	3.52	8.78	WIPF3	WASL-interacting protein family member 3	XM_002665520.7
B5G0804	3.52	2.31	POL	Retrovirus-related Pol polyprotein from transposon 412	
A23G0593	3.52	2.55	EYA4	Eyes absent homolog 4	XM_005162316.5
A7G0945	3.52	20.92	TIFA	TRAF-interacting protein with FHA domain-containing protein A	
B1G0105	3.52	7.67	RETREG2	Reticulophagy regulator 2	NM_001080014.1
A14G0397	3.52	9.34	CCDC88C	Protein Daple	NM_001386506.1
B6G0784	3.52	11.88	EDEM1	ER degradation-enhancing alpha-mannosidase-like protein 1	NM_201189.2
A11G0665	3.52	4.55	GNL3	Guanine nucleotide-binding protein-like 3	XM_005155908.5
A25G0387	3.51	6.54	GRAMD4	GRAM domain-containing protein 4	XM_005163043.5
B3G1405	3.51	2.00	HNRNPA0	Heterogeneous nuclear ribonucleoprotein A0	
B9G0693	3.51	7.80	INPP4A	Inositol polyphosphate-4-phosphatase type I A	XM_017357717.3
B23G0986	3.51	3.66	TXK	Tyrosine-protein kinase TXK	XM_003201244.5
B7G1214	3.51	8.12	MFAP3L	Microfibrillar-associated protein 3-like	NM_001328051.1
B22G0840	3.51	5.92	SIGLEC1	Sialoadhesin	XM_021474479.2
B25G0432	3.51	8.88	DNM1L	Dynamin-1-like protein	XM_005163050.3
A9G0549	3.51	6.29	LATS2	threonine-protein kinase LATS2	NM_001423734.1
A11G0461	3.51	5.53	CDKL5	Cyclin-dependent kinase-like 5	XM_001922238.7
A25G0503	3.51	0.24			
A11G0446	3.51	6.35	RPGR	X-linked retinitis pigmentosa GTPase regulator	NM_200333.1
B15G0598	3.51	3.49	NAIF1	Nuclear apoptosis-inducing factor 1	NM_001013490.2
B7G1128	3.51	2.82	AGFG2	Arf-GAP domain and FG repeat-containing protein 2	NM_001030238.1
B13G0936	3.51	3.47			
B3G1084	3.51	3.16	LOC107567227	A0A672PC62_SINGR Zinc finger protein 16-like	NM_213535.1
B1G1065	3.51	4.99	CD300A	CMRF35-like molecule 8	XR_011015214.1
B17G0786	3.51	7.36	SLC39A9	Zinc transporter ZIP9	NM_001013540.1
A23G0628	3.51	9.21	CHI3L1	Chitinase-3-like protein 1	NM_001204373.1
B3G1725	3.51	0.53	DPX16_22263	A0A3N0Y039_ANAGA Uncharacterized protein	

A4G0808	3.51	3.34	DMTF1	Cyclin-D-binding Myb-like transcription factor 1	NM_001002665.1
A16G0565	3.51	1.40	CBL	E3 ubiquitin-protein ligase CBL	XM_021466724.2
B16G0784	3.51	3.61	LOC113116389	A0A6P6R517_CARAU cell division cycle-associated protein 3-like	NM_001045049.1
A6G0802	3.51	5.42	H6PD	6PGL endoplasmic bifunctional protein	XM_692728.9
A19G0349	3.51	4.22	COL8A2	Collagen alpha-2(VIII) chain	XM_068215201.1
A10G0460	3.51	16.47	IFT46	Intraflagellar transport protein 46 homolog	NM_001346250.1
A15G0106	3.51	0.41	CLDN8	Claudin-8	NM_001003733.1
B15G0730	3.51	5.72	RHBDD1	Rhomboid-related protein 4	NM_001017614.1
A7G0173	3.51	6.38	RAP1GDS1-A	Rap1 GTPase-GDP dissociation stimulator 1-A	XM_021478054.2
B23G0853	3.50	5.05	POL	Retrovirus-related Pol polyprotein from transposon 412	
B14G0286	3.50	8.22	PTTG1	Securin	NM_001271474.1
B15G0804	3.50	2.35	SLC36A4	Proton-coupled amino acid transporter 4	XM_005157799.5
B15G0936	3.50	6.95	LOC113114657	A0A6P6QV34_CARAU uncharacterized protein LOC113114657 isoform X1	XM_068216615.1
A3G0634	3.50	8.06	SEPTIN9	Septin-9	NM_001423665.1
A7G0592	3.50	13.28	ARHGAP1	Rho GTPase-activating protein 1	NM_001017781.1
A24G0231	3.50	15.47	CREM	cAMP-responsive element modulator	NM_001017664.3
B11G0393	3.50	3.98	RTL1	Retrotransposon-like protein 1	NM_130938.1
B13G0155	3.50	10.25	UBR2	E3 ubiquitin-protein ligase UBR2	NM_001256216.1
A1G1091	3.50	3.18	FBXW7	WD repeat-containing protein 7	XM_021473906.2
B17G0588	3.50	3.52	ADPRS	ADP-ribosylhydrolase ARH3	NM_001004565.2
B15G0674	3.50	4.69	ARFIP2	Arfaptin-2	XM_005157358.5
B20G0020	3.50	4.84	ZFP292	Zinc finger protein 292	NM_001030212.1
A3G0555	3.50	29.94	GPATCH8	G patch domain-containing protein 8	NM_001328037.1
B1G0546	3.50	4.99	ZNF654	Zinc finger protein 654	XM_003197672.6
A5G0198	3.50	10.70	SEC14L2	SEC14-like protein 2	NM_001099993.2
A16G0258	3.50	3.99	PTPN23	Tyrosine-protein phosphatase non-receptor type 23	XM_005158195.5
B17G0246	3.50	5.18	ZMYM4	Zinc finger MYM-type protein 4	XM_684300.10
B10G0255	3.50	18.07	XPO7	Exportin-7	NM_001128230.2
B23G0292	3.50	56.16	FKBP1A	Peptidyl-prolyl cis-trans isomerase FKBP1A	NM_001005594.1
B13G0754	3.50	46.94	PCDH15	Protocadherin-15	NM_001308554.1
A8G0028	3.50	3.77	IL6ST	Interleukin-6 receptor subunit beta	
A7G1133	3.50	8.44	FURIN	Furin-1	NM_001045106.2
B20G0661	3.49	2.31	PEX3	Peroxisomal biogenesis factor 3	NM_200228.1
B8G0847	3.49	5.26	RAPGEF1	Rap guanine nucleotide exchange factor 1	XM_005166904.5
B8G0917	3.49	4.09	HDAC6	Histone deacetylase 6	XM_009303752.3
B2G1070	3.49	13.54	USP33	Ubiquitin carboxyl-terminal hydrolase 33	NM_213227.1
A7G0396	3.49	9.36			
B12G0704	3.49	2.58	KIF22	Kinesin-like protein KIF22	NM_001113591.1
B18G0414	3.49	3.34	FKBP8	Peptidyl-prolyl cis-trans isomerase FKBP8	NM_001386852.1
B8G0776	3.49	3.56	SPATA6	Spermatogenesis-associated protein 6	NM_001430981.1

A21G0825	3.49	1.47	SRA1	Steroid receptor RNA activator 1	NM_001002047.1
A17G0397	3.49	6.73	JADE1	Protein Jade-1	XM_005158491.5
B21G0144	3.49	4.23	CHML	Rab proteins geranylgeranyltransferase component A 2	NM_203461.1
A2G0232	3.49	2.43	TXNDC12	Thioredoxin domain-containing protein 12	
A2G0035	3.49	1.58	TRIM16	Tripartite motif-containing protein 16	XM_690458.9
A7G0146	3.49	3.68	LOC113067422	A0A6P6MHX5_CARAU uncharacterized protein LOC113067422 isoform X2	
A4G0526	3.49	5.47	NUP107	Nuclear pore complex protein Nup107	NM_001030167.1
A12G0007	3.49	0.96	PARG	Poly(ADP-ribose) glycohydrolase	
A7G0647	3.49	23.21	HSD11B2	11-beta-hydroxysteroid dehydrogenase type 2	NM_212720.2
B2G0779	3.49	5.77	PIK3R1	Phosphatidylinositol 3-kinase regulatory subunit alpha	NM_212822.2
A15G0158	3.49	8.55	CAB39	Calcium-binding protein 39	NM_001002145.1
B22G0488	3.49	2.02	DPX16_1947	A0A3N0Y742_ANAGA Zinc finger protein 595	NM_001386816.1
B4G0643	3.49	6.84	SLC37A3	Sugar phosphate exchanger 3	
A18G0219	3.49	3.93	EMC8	ER membrane protein complex subunit 8	NM_213370.1
B18G0526	3.49	7.61	N4BP1	NEDD4-binding protein 1	NM_199787.2
B8G0733	3.49	5.02	ACOT11	Acyl-coenzyme A thioesterase 11	NM_001430895.1
B11G0650	3.49	3.59	NUCKS1	Nuclear ubiquitous casein and cyclin-dependent kinase substrate 1	XM_003199417.5
B17G0336	3.49	4.56	SH3PXD2A	SH3 and PX domain-containing protein 2A	XM_068218851.1
B7G0415	3.49	6.15	TTC17	Tetratricopeptide repeat protein 17	NM_001423800.1
A14G0392	3.49	8.65	PLAAT3	Phospholipase A and acyltransferase 3	
B8G0898	3.49	8.63	CDK16	Cyclin-dependent kinase 16	XM_021478368.2
A20G0143	3.49	2.89	BATF	Basic leucine zipper transcriptional factor ATF-like	XM_005160860.5
B23G0464	3.49	2.44	SULT1C4	Sulfotransferase 1C4	NM_001199903.1
A16G0705	3.49	4.36	ZFY1	Zinc finger Y-chromosomal protein 1	XM_009292104.4
B22G0886	3.49	0.00	VTCN1	V-set domain-containing T-cell activation inhibitor 1	
B5G0142	3.48	4.83	RCHY1	RING finger and CHY zinc finger domain-containing protein 1	NM_212600.2
A8G0333	3.48	7.09	CPOX	Oxygen-dependent coproporphyrinogen-III oxidase, mitochondrial	NM_001040094.2
A25G0266	3.48	2.28	MPPED2	Metallophosphoesterase MPPED2	NM_200042.2
A21G0340	3.48	2.17	BTC	Probetacellulin	
B4G0604	3.48	4.89	PARP11	Protein mono-ADP-ribosyltransferase PARP11	
A16G0172	3.48	6.30	SYNDIG1L	Synapse differentiation-inducing gene protein 1-like	NM_001386350.1
A6G0818	3.48	4.99	TARBP2	RISC-loading complex subunit tarbp2	NM_199997.1

B9G0516	3.48	3.66	SLC39A10	Zinc transporter ZIP10	XM_068223350.1
A24G0602	3.48	3.51	ZMYM3	Zinc finger MYM-type protein 3	NM_001430955.1
A3G0497	3.48	5.35	RNF216	E3 ubiquitin-protein ligase RNF216	XM_068219338.1
A11G0126	3.48	4.05	SLC1A3	Excitatory amino acid transporter 1	
B10G0691	3.48	4.68	CASP7	Caspase-7	
A7G0510	3.48	1.96	SPATA2	Spermatogenesis-associated protein 2	XM_017357077.3
B1G0945	3.48	3.26	TRIOBP	TRIO and F-actin-binding protein	XM_005160161.5
A10G0497	3.48	8.44	EHBP1L1	EH domain-binding protein 1-like protein 1	XM_021479366.2
B21G0598	3.48	2.04	HPSE	Heparanase	NM_001045005.1
B22G0808	3.48	10.14	PTPRC	Receptor-type tyrosine-protein phosphatase C	
B3G0098	3.48	0.05	GIMAP9	GTPase IMAP family member 9	XM_068218152.1
B18G0114	3.48	2.17	CXCR5	C-X-C chemokine receptor type 5	
A7G1383	3.48	2.82	ALPK1	Alpha-protein kinase 1	XM_009302790.3
B21G0708	3.48	6.35	AQP7	Aquaporin-7	XM_068215802.1
A5G0804	3.48	4.16	CXCL11.1	C-X-C motif chemokine 11-1	
B22G1058	3.48	3.95	ARMC9	LisH domain-containing protein ARMC9	XM_068216489.1
A18G0233	3.48	5.36	DHX36	RNA helicase DHX36	XM_005159207.5
A9G0016	3.48	3.20	TBC1D8	TBC1 domain family member 8	XM_002663446.7
B21G0199	3.48	3.05	TAF9	Transcription initiation factor TFIID subunit 9	NM_001044395.2
B22G0333	3.48	6.97	LOC107694292	A0A671R105_9TELE Uncharacterized LOC107694292	XM_021470864.2
B15G0246	3.47	10.13	RAB4B	Ras-related protein Rab-4B	NM_001004002.1
A14G0107	3.47	10.38	MATR3	Matrin-3	NM_001122784.2
B14G0754	3.47	0.93	A33	Zinc-binding protein A33	NM_001044961.1
A13G0579	3.47	34.09	SDCCAG8	Serologically defined colon cancer antigen 8 homolog	NM_001029967.3
A2G0025	3.47	6.66	PHLPP1	PH domain leucine-rich repeat protein phosphatase 1	XM_009296464.4
A13G0060	3.47	3.71	SPTBN5	Spectrin beta chain, non-erythrocytic 5	NM_201206.2
A5G0711	3.47	0.46	TMPRSS13	Transmembrane protease serine 13	
A8G0512	3.47	1.57	SUV39H1	Histone-lysine N-methyltransferase SUV39H1	NM_001126482.1
B13G0775	3.47	6.80	CDH23	Cadherin-23	XM_009307078.4
B11G0372	3.47	4.26	X-ELEMENT\ORF2	Probable RNA-directed DNA polymerase from transposon X-element	NM_001080015.2
B16G0775	3.47	3.87	CXCR3.2	C-X-C chemokine receptor type 3-2	NM_001007314.1
B7G0744	3.47	0.56	LY75	Lymphocyte antigen 75	
B3G1115	3.47	5.00	ATXN7L3	Ataxin-7-like protein 3	NM_001040341.2
B25G0427	3.47	2.28	ADAL	Adenosine deaminase-like protein	NM_001033744.1
A19G0881	3.47	2.98	RAPGEF5	Rap guanine nucleotide exchange factor 5	XM_068215224.1
A2G0245	3.47	5.87	MIB1	E3 ubiquitin-protein ligase mib1	NM_173286.3
B5G0472	3.47	1.98	PEX12	Peroxisome assembly protein 12	NM_200205.2
A3G0571	3.47	24.04	EZH1	Histone-lysine N-methyltransferase EZH1	NM_001039983.2
B20G0324	3.47	12.19	RTN1	Reticulon-1	NM_001328271.1

B25G0159	3.47	7.58	IGKV1-13	Immunoglobulin kappa variable 1-13	NM_001045408.1
A2G1076	3.47	5.47	CCR4	C-C chemokine receptor type 4	
B6G0250	3.47	8.91	SENP2	Sentrin-specific protease 2	XM_679191.10
B15G0519	3.47	10.95	AIPL1	Aryl-hydrocarbon-interacting protein-like 1	XM_021466431.2
B7G0967	3.46	7.66	TLNRD1	Talin rod domain-containing protein 1	XM_005166366.5
A20G0617	3.46	4.85	PPP1R13B	Apoptosis-stimulating of p53 protein 1	NM_001044824.2
B22G1025	3.46	2.33	NLRC3	NLR family CARD domain-containing protein 3	XM_021470830.2
B22G0250	3.46	3.21	G5714_021277	A0A7J6BQI0_9TELE Ig-like domain-containing protein	XM_068216396.1
A14G0549	3.46	7.83	DNAJC4	DnaJ homolog subfamily C member 4	NM_212677.1
B20G0470	3.46	5.61	VPS18	Vacuolar protein sorting-associated protein 18 homolog	NM_173245.2
A14G0355	3.46	13.27	TSPAN7	Tetraspanin-7	NM_214774.2
A14G0808	3.46	4.80	ATOH8	Transcription factor atoh8	NM_001079991.2
B5G0261	3.46	3.35	USP39	U6.U5 tri-snRNP-associated protein 2	NM_001080070.1
B21G0274	3.46	3.33	CORO6	Coronin-6	XM_017352760.3
B5G1267	3.46	8.14	ACACB	Acetyl-CoA carboxylase 2	XM_021476434.2
A21G0160	3.46	5.74	AP3B1	AP-3 complex subunit beta-1	XM_021469001.2
B3G0097	3.46	0.92	LOC113074991	A0A6P6N523_CARAU gastrula zinc finger protein XlCGF57.1-like isoform X1	XM_005161560.5
A20G0869	3.46	12.02	NT5E	5'-nucleotidase	NM_200932.2
B8G0349	3.46	2.39	KIAA1671	Uncharacterized protein KIAA1671	XM_002663199.7
A15G0819	3.46	1.91	CLDN4	Claudin-4	NM_131765.1
B18G0584	3.46	4.87	ITPR2	Inositol 1,4,5-trisphosphate receptor type 2	XM_021467780.2
B15G0015	3.46	0.39	VSTM5	V-set and transmembrane domain-containing protein 5	
A7G0293	3.46	5.05	ANKRD11	Ankyrin repeat domain-containing protein 11	XM_009303497.4
B17G0256	3.46	2.71	WDHD1	WD repeat and HMG-box DNA-binding protein 1	NM_001002726.2
A25G0078	3.46	6.86	PPP1R12A	Protein phosphatase 1 regulatory subunit 12A	XM_002666774.7
A7G0606	3.46	3.69	PTPMT1	Phosphatidylglycerophosphatase and protein-tyrosine phosphatase 1	NM_001080187.1
B2G0568	3.46	6.68	RASAL2	Ras GTPase-activating protein nGAP	XM_005171321.5
A4G0771	3.46	3.49	SEMA3AA	Semaphorin-3aa	NM_131060.1
A11G0508	3.46	2.25	PHKA2	Phosphorylase b kinase regulatory subunit alpha, liver isoform	
B3G0480	3.46	5.13	RDH8	Retinol dehydrogenase 8	NM_200707.1
B4G0822	3.46	2.16	A0A671MNT3	A0A671MNT3_9TELE Uncharacterized protein	XM_068220086.1
B23G0642	3.46	4.56	TPX2-A	Targeting protein for Xklp2-A	XM_017353499.3
A8G0908	3.46	3.92	K2013	Uncharacterized protein KIAA2013 homolog	NM_001113612.1
B3G1342	3.46	3.56	TSEN54	tRNA-splicing endonuclease subunit Sen54	XM_021470735.2
B5G0959	3.45	3.99	HINFP	Histone H4 transcription factor	NM_213424.3
A4G0622	3.45	5.08	APAF1	Apoptotic protease-activating factor 1	XM_005164770.5

B9G0289	3.45	3.95	CDC16	Cell division cycle protein 16 homolog	NM_001037389.2
A23G0081	3.45	1.51	NDNF	Protein NDNF	NM_001423783.1
A6G0933	3.45	4.40	ABHD14B	Putative protein-lysine deacylase ABHD14B	NM_200367.1
A5G1017	3.45	1.46	LRWD1	Leucine-rich repeat and WD repeat-containing protein 1	XM_678221.9
B19G0049	3.45	3.25	CDCA7L	Cell division cycle-associated 7-like protein	XM_002665462.6
B4G0908	3.45	1.08	G5714_004666	A0A7J6D5A0_9TELE Uncharacterized protein	XM_068219623.1
A22G0510	3.45	10.46	B3GALT2	Beta-1,3-galactosyltransferase 2	NM_207101.1
B10G0815	3.45	3.90	PDXP	Chronophin	NM_001128705.1
A5G0088	3.45	4.52	MVB12B	Multivesicular body subunit 12B	NM_001431133.1
B4G0840	3.45	0.55	MRC2	C-type mannose receptor 2	NM_001082802.1
A5G1292	3.45	8.46	TUT7	Terminal uridylyltransferase 7	XM_068221378.1
B16G0048	3.45	6.19	XRCC1	DNA repair protein XRCC1	NM_001003988.2
B11G0369	3.45	1.79	MYOG	Myogenin	XM_068224160.1
A5G0045	3.45	1.82	CYTB	Cystatin-B	
A14G0486	3.45	1.96	COX18	Cytochrome c oxidase assembly protein COX18, mitochondrial	
A8G0683	3.45	1.50	TMIGD2	Transmembrane and immunoglobulin domain-containing protein 2	XM_017357515.3
A2G0224	3.45	2.64	PIGK	GPI-anchor transamidase	NM_001002149.1
B19G0682	3.45	20.24	SCIN	Scinderin	NM_001045118.2
B5G0338	3.45	2.64	ARHGEF15	Rho guanine nucleotide exchange factor 15	XM_017356341.3
B8G0723	3.45	1.59	MUTYH	Adenine DNA glycosylase	NM_001436603.1
B13G0606	3.45	2.90	SLC66A3	Solute carrier family 66 member 3	NM_001017691.1
B14G0330	3.45	6.72	PCDH1	Protocadherin-1	XM_009291219.4
A17G0711	3.45	2.82	TYRO3	Tyrosine-protein kinase receptor TYRO3	NM_131432.2
A10G0742	3.44	10.07	STRBP	Spermatid perinuclear RNA-binding protein	XM_005155606.5
B6G0516	3.44	3.10	MTR	Methionine synthase	NM_001114914.1
A15G0636	3.44	5.56	COQ8B	Atypical kinase COQ8B, mitochondrial	NM_001424002.1
B20G0269	3.44	8.31			
B24G0399	3.44	29.04	HGD	Homogentisate 1,2-dioxygenase	NM_152966.2
B16G0521	3.44	12.09	RHBG	Ammonium transporter Rh type B	NM_200071.3
A12G0319	3.44	3.24	ETV4	ETS translocation variant 4	NM_131425.2
B3G1235	3.44	7.02	MSL1L1	Male-specific lethal 1-like 1	NM_001114468.1
A23G0552	3.44	20.33	PGD	6-phosphogluconate dehydrogenase, decarboxylating	NM_213453.2
A3G1249	3.44	2.90	AXIN2	Axin-2	NM_131561.1
B7G0209	3.44	6.61	GRAMD2A	GRAM domain-containing protein 2A	XM_005166601.5
A25G0061	3.44	5.44	CHID1	Chitinase domain-containing protein 1	
B19G0929	3.44	10.04	CENPA	Histone H3-like centromeric protein A	NM_001164240.2
B1G1007	3.44	4.25	ASF1BB	Histone chaperone asf1b-B	NM_214757.2
B20G0520	3.44	4.59	NHSL1	NHS-like protein 1	XM_005160764.5
B19G0687	3.44	17.21	BZW2	eIF5-mimic protein 1	XM_005159605.4
A6G0868	3.44	3.81	NELFCD	D	NM_201190.1
A7G0422	3.44	2.63	MTFMT	Methionyl-tRNA formyltransferase, mitochondrial	NM_001077542.2

B4G0293	3.44	2.43	CCDC59	Thyroid transcription factor 1-associated protein 26 homolog	NM_001045054.1
A20G0866	3.44	4.51	MYO6	Unconventional myosin-VI	XM_005160309.5
A7G1198	3.44	3.57	CXCL8	Interleukin-8	
B23G0374	3.44	5.83	CCNQ	Cyclin-Q	NM_001020623.1
B20G0676	3.44	3.78	ITPKB	Inositol-trisphosphate 3-kinase B	XM_005160841.5
A12G0376	3.44	3.05	SOCS5	Suppressor of cytokine signaling 5	NM_001113758.2
B22G0335	3.44	1.37	CD244	Natural killer cell receptor 2B4	XM_068216413.1
B16G1114	3.43	26.22	IBTK	Inhibitor of Bruton tyrosine kinase	XM_005157859.5
A5G0505	3.43	2.58	ARRDC1	Arrestin domain-containing protein 1	NM_001004561.1
B25G0083	3.43	5.60	ANO5	Anoctamin-5	XM_021470757.2
B20G0660	3.43	2.17	ADAT2	tRNA-specific adenosine deaminase 2	XM_005160618.5
A25G0673	3.43	3.28	IGKV4-1	Immunoglobulin kappa variable 4-1	XM_068217596.1
A7G0530	3.43	5.11	ARL5B	ADP-ribosylation factor-like protein 5B	NM_199824.1
A24G0429	3.43	2.05	AVIL	Advillin	XM_009297407.4
A5G0163	3.43	5.82	FBXW8	WD repeat-containing protein 8	NM_001114578.1
A12G0401	3.43	0.00			XM_021472538.2
B23G0122	3.43	6.22	BEND2	BEN domain-containing protein 2	NM_001030233.1
A7G0471	3.43	2.50	SLC25A44	Solute carrier family 25 member 44	NM_001013519.1
A24G0537	3.43	3.96	ASAP1	Arf-GAP with SH3 domain, ANK repeat and PH domain-containing protein 1	XM_003201277.6
A2G0031	3.43	18.31	IRGC	Interferon-inducible GTPase 5	XM_001338161.8
A18G0574	3.43	5.60	SELENOO1	Protein adenylyltransferase SelO-1, mitochondrial	NM_001044871.3
A25G0654	3.43	3.28	IGKV4-1	Immunoglobulin kappa variable 4-1	XM_068217596.1
A11G0615	3.43	2.45	UTS2B	Prepro-urotensin II-beta	
A22G0041	3.43	2.64	USP49	Ubiquitin carboxyl-terminal hydrolase 49	NM_001044896.1
A18G0705	3.43	5.32	GOLIM4	Golgi integral membrane protein 4	NM_001098192.1
A15G0584	3.43	2.67	DHX40	Probable ATP-dependent RNA helicase DHX40	XM_021466351.2
B16G0014	3.43	4.73	CLK2	Dual specificity protein kinase CLK2	NM_001076751.1
B20G0603	3.42	7.00	PDE4DIP	Myomegalin	XM_005160795.5
B10G0814	3.42	4.77	MIER3	Mesoderm induction early response protein 3	NM_001030149.2
B13G0207	3.42	4.66	MARK3	microtubule affinity-regulating kinase 3	NM_001145681.1
A13G0138	3.42	8.28	POLR1B	DNA-directed RNA polymerase I subunit RPA2	XM_068217855.1
A16G0818	3.42	7.46	ANKRD28	threonine-protein phosphatase 6 regulatory ankyrin repeat subunit A	XM_009292265.4
A19G0023	3.42	2.49	TNXB	Tenascin-X	XM_068215342.1
A4G0608	3.42	5.90	CDPF1	Cysteine-rich DPF motif domain-containing protein 1	
A7G0181	3.42	13.64			XM_068222169.1
A11G0472	3.42	4.33	TMEM178A	Transmembrane protein 178A	
B6G0530	3.42	3.01	LRRC40	Leucine-rich repeat-containing protein 40	NM_199862.2

A23G0285	3.42	4.64	TFIP11	Tuftelin-interacting protein 11	NM_001002721.1
B2G1202	3.42	8.33	SNAP47	Synaptosomal-associated protein 47	NM_001045437.1
B13G0636	3.42	6.70	RRBP1	Ribosome-binding protein 1	NM_001007289.1
A2G0516	3.42	3.52	MED26	Mediator of RNA polymerase II transcription subunit 26	NM_213059.1
B20G0138	3.42	6.79	EXOC8	Exocyst complex component 8	NM_001110475.1
A18G0115	3.42	3.51	CDON	down-regulated by oncogenes	NM_001081628.1
B16G0063	3.42	3.86	PTDSS1	Phosphatidylserine synthase 1	NM_200956.1
A17G0014	3.42	5.27	SPRED1	Sprouty-related, EVH1 domain-containing protein 1	NM_213232.1
B3G1292	3.42	4.46	E4F1	Transcription factor E4F1	NM_001025513.1
A4G0892	3.42	7.15	MAPK11	Mitogen-activated protein kinase 11	XM_068219582.1
B15G0365	3.42	4.16	ADAMTS8	A disintegrin and metalloproteinase with thrombospondin motifs 8	XM_021472930.2
B3G0100	3.42	3.14	GIMAP7	GTPase IMAP family member 7	
A24G0435	3.42	5.07	OXSR1	threonine-protein kinase OSR1	NM_201175.3
A22G0462	3.42	3.38	FKBP8	Peptidyl-prolyl cis-trans isomerase FKBP8	NM_200884.1
B16G1103	3.42	0.28	G5714_016531	A0A7J6C8L4_9TELE Uncharacterized protein	
A14G0390	3.42	11.36	GANAB	Neutral alpha-glucosidase AB	XM_009291260.4
A8G0241	3.42	9.78	JAK2	Tyrosine-protein kinase JAK2	XM_002663087.7
A8G0676	3.42	4.51	KIAA1671	Uncharacterized protein KIAA1671	XM_002663199.7
B18G0638	3.42	1.95	SI:CH211-238A12.2	Protein C19orf12 homolog	
B3G0614	3.42	5.83	CCDC97	Coiled-coil domain-containing protein 97	XM_001333080.7
B25G0309	3.41	4.33	LACTB	Serine beta-lactamase-like protein LACTB, mitochondrial	XM_009298158.4
B5G0214	3.41	8.19	NUP214	Nuclear pore complex protein Nup214	XM_017356400.3
B17G0897	3.41	3.32	JDP2	Jun dimerization protein 2	XM_005158934.5
B10G0268	3.41	4.89	DDHD2	Phospholipase DDHD2	XM_021479324.2
A12G0448	3.41	1.62	L3MBTL2	Lethal(3)malignant brain tumor-like protein 2	NM_200032.1
A23G0470	3.41	3.63	SENP1	Sentrin-specific protease 1	XM_021473699.2
A6G0588	3.41	2.96	MIER1	Mesoderm induction early response protein 1	XM_017356436.3
B2G0904	3.41	7.51	PTBP2	Polypyrimidine tract-binding protein 2	NM_001100007.1
B3G1357	3.41	3.10	XYLT2	Xylosyltransferase 2	NM_001130778.2
A14G0234	3.41	5.64	SLC25A43	Solute carrier family 25 member 43	NM_001004497.2
A10G0487	3.41	2.66	WDR74	WD repeat-containing protein 74	NM_001076563.1
A19G0063	3.41	6.26	INTS8	Integrator complex subunit 8	NM_001020756.2
A12G0587	3.41	4.93	KBTBD2	Kelch repeat and BTB domain-containing protein 2	NM_001077160.2
A19G0118	3.41	4.90	LDLRAD4	Low-density lipoprotein receptor class A domain-containing protein 4	XM_021468294.2
A11G0529	3.41	6.14	TRAIP	E3 ubiquitin-protein ligase TRAIP	XM_068224367.1
A22G0253	3.41	29.97	TSPAN7	Tetraspanin-7	NM_001130818.1
B3G1371	3.41	6.04	TCB1	Transposable element Tcb1 transposase	XM_021471168.2

A7G0680	3.41	4.41	UACA	Uveal autoantigen with coiled-coil domains and ankyrin repeats protein	XM_005168952.5
A14G0389	3.41	4.13	UBXN1	UBX domain-containing protein 1	NM_212607.1
B18G0599	3.41	3.88	POLR3B	DNA-directed RNA polymerase III subunit RPC2	XM_685719.8
A4G0578	3.41	2.79	GCC1	GRIP and coiled-coil domain-containing protein 1	NM_001003599.2
B14G0813	3.41	2.13	GJB1	Gap junction beta-1 protein	
B19G0143	3.41	2.35	LOC108891019	A0A4W6F6U8_LATCA Zinc finger protein 260-like	XM_005159346.5
B7G0368	3.41	20.03	PCOLCE2	Procollagen C-endopeptidase enhancer 2	XM_009303443.4
A7G0930	3.41	6.09			
B14G0120	3.41	6.20	GPR137	Integral membrane protein GPR137	NM_001002691.1
A5G1317	3.41	13.94	RNF167	E3 ubiquitin-protein ligase RNF167	XM_021476685.2
A13G0785	3.41	4.52	DLD	Delta-like protein D	NM_001328189.1
B18G0137	3.41	18.58	TRIP12	E3 ubiquitin-protein ligase TRIP12	XM_068214800.1
B15G0855	3.40	8.12	NLRC3	NLR family CARD domain-containing protein 3	XM_009291963.4
B25G0882	3.40	2.97	A0A672M7T8	A0A672M7T8_SINGR RING-type domain-containing protein	
A8G0185	3.40	6.91	GRIPAP1	GRIP1-associated protein 1	NM_001123320.1
A12G0166	3.40	13.54	RNF213B	E3 ubiquitin-protein ligase rnf213-beta	NM_001353867.2
B2G0958	3.40	7.38	KDM4A	Lysine-specific demethylase 4A	XM_001339628.9
A12G0261	3.40	5.94	THBS2	Thrombospondin-2	XM_009306791.4
A6G0497	3.40	2.70	SAP130	Histone deacetylase complex subunit SAP130	NM_001098777.2
B15G0392	3.40	5.52	JAM3B	Junctional adhesion molecule 3B	XM_001338087.8
A3G0429	3.40	6.55	MRTFB	Myocardin-related transcription factor B	XM_009299740.4
A21G0528	3.40	14.22	SUPT6H	Transcription elongation factor SPT6	NM_145118.1
A25G0411	3.40	6.89	ERGIC2	Endoplasmic reticulum-Golgi intermediate compartment protein 2	NM_200407.1
A20G0647	3.40	4.66	SOX7	Transcription factor Sox-7	NM_001080750.2
B11G0216	3.40	7.27	ROHU_016520	A0A498NJ77_LABRO Uncharacterized protein	NM_001122654.1
A13G0255	3.40	3.78	THAP2	THAP domain-containing protein 2	NM_212946.1
A24G0411	3.40	1.99	ZDHHC23B	Palmitoyltransferase ZDHHC23-B	NM_001003757.2
B16G0936	3.40	13.56	ZYX	Zyxin	NM_001109824.2
B3G0655	3.40	22.58	EZH1	Histone-lysine N-methyltransferase EZH1	XM_009299640.4
A19G0714	3.40	3.36	PYGO2	Pygopus homolog 2	NM_001033111.3
A6G0463	3.40	3.48	ZNF644	Zinc finger protein 644	XM_021474596.2
B23G0868	3.40	1.66	LOC113052750	A0A6P6KP89_CARAU uncharacterized protein LOC113052750	
B4G0223	3.40	3.77	WNT5B	Protein Wnt-5b	NM_001362273.1
B10G0293	3.40	0.37	PARP14	Protein mono-ADP-ribosyltransferase PARP14	XM_017358033.3
A25G0416	3.39	3.77	FBXL14	LRR-repeat protein 14	NM_201482.1
B15G0771	3.39	2.37	MAG	Myelin-associated glycoprotein	XM_009291880.4

B23G0163	3.39	1.28	SPIC	Transcription factor Spi-C	XM_009296622.3
A8G0050	3.39	5.60	FBXL17	LRR-repeat protein 17	XM_001335061.8
B1G0216	3.39	3.71	LOC107723170	A0A673G7Y8_9TELE Small kinetochore-associated protein-like	NM_001128774.1
B3G1284	3.39	11.38	TNPO2	Transportin-2	NM_001004003.2
B25G0292	3.39	4.35	SLC27A2	Long-chain fatty acid transport protein 2	NM_001386644.1
B7G0197	3.39	3.21	LTO1	Protein LTO1 homolog	NM_001083552.1
A16G0323	3.39	4.23	CAPN7	Calpain-7	NM_001135108.2
A20G0722	3.39	5.77	LOC107739858	A0A673JP40_9TELE Uncharacterized LOC107739858	NM_001025491.1
B24G0088	3.39	6.25	KATNIP	Katanin-interacting protein	XM_009297662.4
B25G0430	3.39	3.10	TAF4	Transcription initiation factor TFIID subunit 4	XM_005163068.5
B23G0879	3.39	2.55	SLC10A7	bile acid cotransporter 7	NM_001003420.1
B16G0392	3.39	7.65	ZDHHC18A	Palmitoyltransferase ZDHHC18-A	NM_001077563.1
A15G0592	3.39	1.05	TCB1	Transposable element Tcb1 transposase	XM_005166950.5
B1G0399	3.39	3.07	LRBA	Lipopolysaccharide-responsive and beige-like anchor protein	XM_009291242.4
A9G0472	3.39	2.01	INTS6	Integrator complex subunit 6	NM_001126428.1
B19G0758	3.39	5.47	INTS3	Integrator complex subunit 3	NM_001044932.1
A5G0064	3.39	72.41	HSPB1	Heat shock protein beta-1	NM_001008615.2
A25G0685	3.39	6.31	IGKV1-27	Immunoglobulin kappa variable 1-27	NM_001045408.1
B12G0111	3.39	3.37	G5714_012318	A0A7J6CG80_9TELE Uncharacterized protein	
B1G0219	3.39	2.43	BRAT1	BRCA1-associated ATM activator 1	NM_001040305.1
B3G1613	3.39	2.38	USP15	Ubiquitin carboxyl-terminal hydrolase 15	XM_068224942.1
B16G0636	3.39	2.70	LOC107699509	A0A671PWR5_9TELE Zinc finger and BTB domain-containing protein 41-like	XM_068214132.1
B25G0507	3.39	5.49	ARNTL	Aryl hydrocarbon receptor nuclear translocator-like protein 1	XM_005163022.5
B19G0008	3.39	6.47	AKAP9	A-kinase anchor protein 9	XM_021468220.2
A23G0520	3.39	5.92	KMT2D	Histone-lysine N-methyltransferase 2D	XM_017353673.3
B13G0486	3.39	11.63	VRK2	threonine-protein kinase VRK2	NM_001126381.1
B15G0349	3.39	35.07	LOC107715485	A0A673KKD3_9TELE Uncharacterized LOC107715485	
B23G0362	3.38	4.90	KCTD6	POZ domain-containing protein KCTD6	NM_001002329.1
B22G0200	3.38	2.55	CCDC51	Mitochondrial potassium channel	
B23G0123	3.38	3.47	BEND2	BEN domain-containing protein 2	
B5G1191	3.38	28.26	STXB	Stonustoxin subunit beta	XM_068218224.1
B18G0112	3.38	4.31	CDON	down-regulated by oncogenes	NM_001081628.1
A7G1322	3.38	1.47	ROHU_001101	A0A498M017_LABRO Septin-like protein	
B5G0001	3.38	4.62	LRRC19	Leucine-rich repeat-containing protein 19	
A14G0710	3.38	3.14	SLBP	Histone RNA hairpin-binding protein	NM_001256193.1

A12G0394	3.38	3.21	MPP7	MAGUK p55 subfamily member 7	XM_009306585.4
A8G0046	3.38	7.73	CDC14A	Dual specificity protein phosphatase CDC14A	XM_021478208.2
B21G0859	3.38	2.21	MIGA2	Mitoguardin 2	NM_001013557.2
B15G0718	3.38	3.11	LOC107752121	A0A673NB01_9TELE Uncharacterized LOC107752121	XM_688904.10
B17G0755	3.38	3.54	ZFYVE28	Lateral signaling target protein 2 homolog	NM_001077723.1
A8G0392	3.38	4.04	ITPR3	Inositol 1,4,5-trisphosphate receptor type 3	XM_017357380.3
B7G0042	3.38	1.01	ENDOD1	Endonuclease domain-containing 1 protein	
B18G0658	3.38	2.65	TTC38	Tetratricopeptide repeat protein 38	XM_005159026.5
A12G0257	3.38	1.79	ALDH18A1	Delta-1-pyrroline-5-carboxylate synthase	XM_009306786.4
A1G0857	3.38	5.25	GAA	Lysosomal alpha-glucosidase	XM_005170879.5
A16G0306	3.38	6.52	POGZ	Pogo transposable element with ZNF domain	NM_001346224.1
A4G1022	3.38	11.98	TIMP3	Metalloproteinase inhibitor 3	XM_005174662.5
A17G0311	3.38	3.64	ADPRS	ADP-ribosylhydrolase ARH3	NM_001004565.2
B20G0585	3.38	7.10	PRG4	Proteoglycan 4	NM_001328330.1
B20G0950	3.38	6.06	BAHD1	Bromo adjacent homology domain-containing 1 protein	XM_005173837.4
B6G0610	3.38	3.65	CHD1L	Chromodomain-helicase-DNA-binding protein 1-like	XM_068221667.1
A22G0233	3.38	2.67	CYB561D2	Transmembrane reductase CYB561D2	
A19G0723	3.38	4.58	MINDY1	Ubiquitin carboxyl-terminal hydrolase MINDY-1	NM_001100011.1
A3G1308	3.38	4.82	G5714_004013	A0A7J6DCD2_9TELE Uncharacterized protein	
A3G0029	3.38	8.60	A0A671PU89	A0A671PU89_9TELE Palmitoyl-protein thioesterase 2	
B9G0780	3.38	1.13			
A7G0550	3.37	10.04	RBM33	RNA-binding protein 33	XM_021472007.2
B13G0402	3.37	6.99	SORBS1	Sorbin and SH3 domain-containing protein 1	NM_001044333.2
A9G0008	3.37	3.94	PARP14	Protein mono-ADP-ribosyltransferase PARP14	XM_001920524.8
A11G0698	3.37	1.51	THOP1	Thimet oligopeptidase	NM_001430830.1
A20G0526	3.37	4.30	SERAC1	Protein SERAC1	XM_021468495.2
A19G0295	3.37	5.78	ZBTB8B	Zinc finger and BTB domain-containing protein 8B	NM_001328347.1
B2G0763	3.37	2.34	FNDC3B	Fibronectin type III domain-containing protein 3B	NM_001159832.1
A7G0913	3.37	0.91	MRPL52	39S ribosomal protein L52, mitochondrial	NM_001327910.1
B9G0664	3.37	1.89	CD28	T-cell-specific surface glycoprotein CD28	
A16G0675	3.37	4.25	ZNF687A	Zinc finger protein 687a	NM_001353864.1
B5G0769	3.37	6.45	G5714_005657	A0A7J6D1Q9_9TELE C2H2-type domain-containing protein	XM_005165376.5
A9G0077	3.37	13.78	RBMS1	RNA-binding motif, single-stranded-interacting protein 1	XM_068223685.1
B18G0851	3.37	9.17	PDE8A	High affinity cAMP-specific and IBMX-insensitive 3',5'-cyclic phosphodiesterase 8A	NM_001080627.1
B6G0715	3.37	1.07	PPIL1	Peptidyl-prolyl cis-trans isomerase-like 1	NM_001423703.1

A20G0791	3.37	5.82	LPGAT1	Acyl-CoA:lysophosphatidylglycerol acyltransferase 1	NM_001029958.1
B13G0591	3.37	3.97	DPX16_0105	A0A3N0Z3T1_ANAGA Uncharacterized protein	NM_200159.1
A19G0889	3.37	10.07	EOMES	Eomesodermin	NM_131679.3
A15G0135	3.37	0.33	CCDC150	Coiled-coil domain-containing protein 150	
B20G0153	3.37	7.45	LPGAT1	Acyl-CoA:lysophosphatidylglycerol acyltransferase 1	NM_001029958.1
B15G0790	3.37	2.76			
A12G0732	3.37	3.36	KIF22	Kinesin-like protein KIF22	NM_001113591.1
A21G0573	3.37	10.89	RBM4.1	RNA-binding protein 4.1	NM_001115144.1
A7G1143	3.37	16.29	CLPX	ATP-dependent Clp protease ATP-binding subunit clpX-like, mitochondrial	XM_021477471.2
B4G0688	3.37	2.05	G5714_004652	A0A7J6D586_9TELE Uncharacterized protein	NM_001109840.1
B14G0110	3.37	3.29	SLC36A1	Proton-coupled amino acid transporter 1	XM_021481161.2
A16G0321	3.37	5.56	PIK3R4	Phosphoinositide 3-kinase regulatory subunit 4	XM_068214199.1
A11G0432	3.37	8.05	DHRS3	reductase 3	NM_001313699.1
B4G0163	3.37	8.08	VEGFAA	Vascular endothelial growth factor A-A	NM_001328597.1
B8G0412	3.37	5.93	RALGDS	Ral guanine nucleotide dissociation stimulator	XM_005167182.5
B7G1101	3.37	2.99	MAN2B2	Epididymis-specific alpha-mannosidase	XM_001337483.7
B2G0176	3.36	2.18	SH3GL1	Endophilin-A2	NM_001105606.1
A4G0991	3.36	20.13	SLC38A4	Sodium-coupled neutral amino acid transporter 4	XM_017355003.3
A14G0761	3.36	4.58	ABCB7	Iron-sulfur clusters transporter ABCB7, mitochondrial	NM_001423974.1
A24G0376	3.36	3.74	G5714_023432	A0A7J6BL83_9TELE Uncharacterized protein	
B2G0428	3.36	5.78	NADK	NAD kinase	NM_001080002.1
B3G0924	3.36	7.93	SMG1	threonine-protein kinase SMG1	XM_009299453.4
A17G0791	3.36	8.42	FUCA2	Plasma alpha-L-fucosidase	NM_001004115.1
B19G0708	3.36	6.84	TXLNA	Alpha-taxilin	NM_001099230.1
B16G1082	3.36	2.84	ZC3H12A	Endoribonuclease ZC3H12A	XM_021466808.2
B5G0093	3.36	2.10	MVB12B	Multivesicular body subunit 12B	NM_001076606.2
B8G0515	3.36	3.49	BORCS6	BLOC-1-related complex subunit 6	XR_011016487.1
A22G0163	3.36	8.03	LOC107707595	A0A673KDQ7_9TELE Uncharacterized LOC107707595	XM_005161637.5
A3G1150	3.36	5.52	LMTK2	threonine-protein kinase LMTK2	NM_001326472.1
B2G0742	3.36	5.70	GLIS1	Zinc finger protein GLIS1	XM_068213999.1
A5G0477	3.36	5.45	VPS37B	Vacuolar protein sorting-associated protein 37B	NM_001099996.1
B25G0604	3.36	13.33	FA2H	Fatty acid 2-hydroxylase	NM_001430959.1
B1G0108	3.36	2.66	GTF3C3	General transcription factor 3C polypeptide 3	NM_001256242.1
A22G0537	3.36	8.28	DNAL4	Dynein axonemal light chain 4	NM_001020502.1
A19G0852	3.36	6.96	PTK2	Focal adhesion kinase 1	NM_198819.1
B2G0679	3.36	3.81	SOCS6	Suppressor of cytokine signaling 6	NM_199625.2
A20G0714	3.36	42.39	MYOC	Myocilin	NM_001015062.2

B2G0427	3.36	1.52	GPR160	Probable G-protein coupled receptor 160	
A17G0785	3.36	1.98	SLC5A6	Sodium-dependent multivitamin transporter	XM_005158575.5
A7G1224	3.36	10.30	PACS1	Phosphofurin acidic cluster sorting protein 1	XM_005172169.5
A21G0244	3.36	7.03	DYNC2I2	Cytoplasmic dynein 2 intermediate chain 2	NM_001105690.1
B2G1109	3.35	1.09	BRINP3	retinoic acid-inducible neural-specific protein 3	XM_021466751.2
B14G0656	3.35	2.92	SLC35A4	Probable UDP-sugar transporter protein SLC35A4	
A6G1031	3.35	7.11	KDM6A	Lysine-specific demethylase 6A	XM_005168796.5
A18G0347	3.35	2.47	TP53I11	Tumor protein p53-inducible protein 11	XM_021467658.2
B19G0436	3.35	2.88	EXT1C	Exostosin-1c	NM_001040330.2
B23G0743	3.35	6.53	ACAP3	Arf-GAP with coiled-coil, ANK repeat and PH domain-containing protein 3	XM_068216935.1
A24G0184	3.35	4.89	AGAP3	Arf-GAP with GTPase, ANK repeat and PH domain-containing protein 3	XM_009297585.4
A17G0326	3.35	4.90	EVL	VASP-like protein	XM_005158793.5
B5G0508	3.35	2.60	CIZ1	Cip1-interacting zinc finger protein	XM_068221134.1
B23G0612	3.35	5.67	SELENOI	Ethanolaminephosphotransferase 1	NM_001329437.1
B9G0028	3.35	4.93	A0A673LZZ1	A0A673LZZ1_9TELE SGNH_hydro domain-containing protein	XM_068219418.1
B1G0874	3.35	1.97	RNASEH2A	Ribonuclease H2 subunit A	NM_200226.2
B3G1246	3.35	3.23	LSM12B	Protein LSM12 homolog B	NM_214817.1
A6G0480	3.35	2.43	DTYMK	Thymidylate kinase	
B2G0316	3.35	7.27	IFT57	Intraflagellar transport protein 57 homolog	NM_001001832.1
A1G0309	3.35	4.83	HERC5	E3 ISG15--protein ligase HERC5	XM_068221727.1
B16G0895	3.35	0.23	A0A671L0Q4	A0A671L0Q4_9TELE Si:dkeyp-86h10.3	
A17G0471	3.35	4.47	PELI1	E3 ubiquitin-protein ligase pellino homolog 1	NM_001077601.2
A2G0178	3.35	12.93	RGS16	Regulator of G-protein signaling 16	NM_001017821.2
B15G0428	3.35	5.12	TPST1	Protein-tyrosine sulfotransferase 1	XM_005157502.5
B19G0963	3.35	4.12	FBXL19	LRR-repeat protein 19	NM_001100147.1
A16G0124	3.35	3.87	GLCCI1	Glucocorticoid-induced transcript 1 protein	XM_021467028.2
A16G0355	3.35	8.07	ARID1A	AT-rich interactive domain-containing protein 1A	XM_021466949.2
A7G0227	3.34	9.84	USP47	Ubiquitin carboxyl-terminal hydrolase 47	NM_001331129.1
A20G0705	3.34	6.77	SCRN2	Secernin-2	NM_001030116.1
A19G0461	3.34	2.71	SLC17A5	Sialin	XM_005159769.5
B15G0621	3.34	4.77	MSI2	RNA-binding protein Musashi homolog 2	XM_009291629.4
B12G0485	3.34	6.60	ARHGAP17	Rho GTPase-activating protein 17	NM_001430836.1
B5G1094	3.34	7.06	ABCA2	ATP-binding cassette sub-family A member 2	XM_005165230.5
A18G0600	3.34	23.05	TAT	Tyrosine aminotransferase	NM_001077554.2

B7G0267	3.34	5.47	MAD2L1	Mitotic spindle assembly checkpoint protein MAD2A	NM_001017739.1
A13G0709	3.34	3.42	COA8	Cytochrome c oxidase assembly factor 8	NM_001020816.1
B16G1113	3.34	2.96	UBE3D	E3 ubiquitin-protein ligase E3D	NM_001326410.1
B20G0385	3.34	1.88	EPHA7	Ephrin type-A receptor 7	NM_001044979.2
B12G0501	3.34	4.00	FAM20C	threonine protein kinase FAM20C	XM_021480291.2
B15G0319	3.34	5.62	RAB34	Ras-related protein Rab-34	XM_005157422.5
A14G0323	3.34	4.57	FBXO38	F-box only protein 38	XM_005169601.5
B20G0601	3.34	1.55	GORAB	RAB6-interacting golgin	NM_200385.2
B15G0474	3.34	10.65	MCAM	Cell surface glycoprotein MUC18	XM_005157570.3
A17G0754	3.34	9.84	SYNE1	Nesprin-1	XM_068214520.1
B23G0347	3.34	1.55	FKBPL	FK506-binding protein-like	XM_005162114.5
B7G0487	3.34	11.19	CDH11	Cadherin-11	NM_131214.1
A21G0115	3.34	7.32	PSMD5	26S proteasome non-ATPase regulatory subunit 5	NM_001017573.2
A3G0729	3.34	2.15	BAX	Apoptosis regulator BAX	
A25G0130	3.34	3.06	SNUPN	Snurportin-1	NM_199694.1
B22G0920	3.34	6.63	SENP7	Sentrin-specific protease 7	NM_001122946.1
B24G0744	3.34	8.17	TRIM16	Tripartite motif-containing protein 16	XM_003201267.6
B6G0880	3.34	3.36	NELFCD	D	NM_201190.1
A15G0474	3.34	2.42	C1QTNF5	Complement C1q tumor necrosis factor-related protein 5	NM_001029953.1
B18G0811	3.34	3.70	CAPN5	Calpain-5	NM_001080007.1
A7G0338	3.34	5.23	TRMT44	Probable tRNA (uracil-O(2)-)-methyltransferase	NM_001006086.2
A17G0420	3.34	71.72	PPP1CB	threonine-protein phosphatase PP1-beta catalytic subunit	NM_001004527.4
A21G0584	3.33	4.69	RASGRP2	RAS guanyl-releasing protein 2	XM_680043.8
B7G0541	3.33	4.85	RBCK1	RanBP-type and C3HC4-type zinc finger-containing protein 1	NM_001111200.1
B6G0759	3.33	16.16	CISH	Cytokine-inducible SH2-containing protein	NM_001076617.2
B2G0314	3.33	11.79	LOC107659730	A0A671K430_9TELE Apolipoprotein M	
B24G0231	3.33	3.74	G5714_023267	A0A7J6BL65_9TELE Uncharacterized protein	XM_002660922.7
B22G0104	3.33	2.73	LOC113075279	A0A6P6N435_CARAU zinc finger protein 271-like isoform X3	XM_017356396.3
A21G0314	3.33	14.11	STOM	Stomatin	NM_131758.2
B4G0730	3.33	5.26	ZFY1	Zinc finger Y-chromosomal protein 1	XM_009301034.4
B4G0515	3.33	2.97	CEP83	Centrosomal protein of 83 kDa	NM_001353931.1
B8G0796	3.33	6.37	SIN3B	Paired amphipathic helix protein Sin3b	NM_001044945.1
A22G0561	3.33	0.38	CANX	Calpain-1 catalytic subunit	XM_068216342.1
B23G0706	3.33	6.23	JPH2	Junctophilin-2	NM_001089364.1
B15G0334	3.33	6.72	NXN	Nucleoredoxin	NM_001020595.1
B1G0539	3.33	9.25	CREB1	Cyclic AMP-responsive element-binding protein 1	NM_200909.1
B9G0483	3.33	5.95	PARP14	Protein mono-ADP-ribosyltransferase PARP14	NM_001114447.2
A17G0445	3.33	2.63	SPECC1L	Cytospin-A	XM_009293070.4
A3G1034	3.33	33.93	PVALB7	Parvalbumin-7	NM_001430919.1
B5G0731	3.33	9.36	SNX18	Sorting nexin-18	NM_001100006.1
B16G0294	3.33	3.14	NAGPA	N-acetylglucosamine-1-phosphodiester alpha-N-acetylglucosaminidase	NM_001109715.2

B11G0307	3.33	2.79	TMF1	TATA element modulatory factor	XM_005172885.5
A6G0167	3.33	17.96	MYH11	Myosin-11	NM_001024448.1
B5G0077	3.33	3.50	ASGR1	Asialoglycoprotein receptor 1	
A5G0405	3.33	12.90	DGCR8	Microprocessor complex subunit DGCR8	NM_001122749.1
A9G0121	3.33	1.78	GJC1	Gap junction gamma-1 protein	NM_131069.2
A3G0763	3.33	1.85	HARBI1	Putative nuclease HARBI1	XM_021471442.2
A11G0466	3.33	7.32	SVEP1	Sushi, von Willebrand factor type A, EGF and pentraxin domain-containing protein 1	XM_685280.9
B21G0934	3.33	2.08	GIMAP8	GTPase IMAP family member 8	XM_017354601.3
A5G0704	3.33	5.52	MYO1C	Unconventional myosin-Ic	NM_001431130.1
B7G0737	3.33	3.62	WDR76	WD repeat-containing protein 76	NM_001198567.1
A7G0507	3.33	6.60	CMTM3	CKLF-like MARVEL transmembrane domain-containing protein 3	NM_001003468.2
B11G0446	3.32	3.93	CXXC1	CXXC-type zinc finger protein 1	NM_001312677.1
A23G0152	3.32	2.86	RBM38	RNA-binding protein 38	NM_001110127.1
B19G0353	3.32	365.87	G5714_018785	A0A7J6BZX5_9TELE Uncharacterized protein	XM_001340280.5
B2G0784	3.32	4.27	MED26	Mediator of RNA polymerase II transcription subunit 26	NM_213059.1
A9G0418	3.32	4.91	ARMC8	Armadillo repeat-containing protein 8	NM_001079683.2
A7G0572	3.32	6.09	SLC22A18	Solute carrier family 22 member 18	NM_001037385.2
B9G0657	3.32	2.89	FZD7-A	Frizzled-7-A	NM_131139.2
A3G1133	3.32	3.84	IGLV3-21	Immunoglobulin lambda variable 3-21	XM_021474765.2
A18G0595	3.32	3.55	MEAK7	MTOR-associated protein MEAK7	NM_001044965.2
B20G0306	3.32	10.70	EIF2B4	Translation initiation factor eIF-2B subunit delta	NM_001014361.2
B7G0424	3.32	7.06	MFHAS1	Malignant fibrous histiocytoma-amplified sequence 1	XM_683840.8
B17G0865	3.32	2.25	EXD2	Exonuclease 3'-5' domain-containing protein 2	NM_001114406.1
A8G0831	3.32	5.91	GINS4	DNA replication complex GINS protein SLD5	NM_001003546.2
A5G1243	3.32	6.47	PRRC2B	Protein PRRC2B	XM_068221580.1
B12G0375	3.32	3.76	ASB3	Ankyrin repeat and SOCS box protein 3	NM_199874.1
B14G0088	3.32	11.82	FAM13B	Protein FAM13B	XM_005173130.5
A25G0653	3.32	6.88	IGKV1-5	Immunoglobulin kappa variable 1-5	NM_001045408.1
A14G0104	3.32	4.17	TMEM216	Transmembrane protein 216	NM_001328140.1
B3G1627	3.32	2.63	G5714_002766	A0A7J6D7N0_9TELE Uncharacterized protein	XM_017354466.3
A24G0296	3.32	4.22	PHC3	Polyhomeotic-like protein 3	XM_009297490.4
A2G0846	3.32	4.78	TEP1	Telomerase protein component 1	XM_009298675.4
B9G0799	3.32	7.83	USP37	Ubiquitin carboxyl-terminal hydrolase 37	XM_009304386.4
B3G1495	3.32	2.25	NLRP1	NACHT, LRR and PYD domains-containing protein 1 homolog	XM_068220504.1
B12G0365	3.32	2.56	RHOQ	Rho-related GTP-binding protein RhoQ	NM_199818.1

B13G0293	3.32	6.77	LINGO2	Leucine-rich repeat and immunoglobulin-like domain-containing nogo receptor-interacting protein 2	XM_068224920.1
A8G0138	3.31	3.18	NLRP12	NACHT, LRR and PYD domains-containing protein 12	XM_002663065.7
B4G0879	3.31	2.57	G5714_004381	A0A7J6D4J8_9TELE Uncharacterized protein	
B14G0712	3.31	6.93	SPON2	Spondin-2	NM_131008.4
A25G0044	3.31	5.33	TMPO	gamma	
B4G0667	3.31	3.56	LOC107752214	A0A673N233_9TELE Gastrula zinc finger protein XlCGF57.1-like	XM_068220328.1
B16G0299	3.31	2.90	CFAP418	Cilia- and flagella-associated protein 418	NM_001424183.1
A25G0055	3.31	4.00	INCENP-A	Inner centromere protein A	XM_009297716.4
B2G0835	3.31	2.70	PLCD1	1-phosphatidylinositol 4,5-bisphosphate phosphodiesterase delta-1	NM_200068.1
B17G0533	3.31	4.31	AP4S1	AP-4 complex subunit sigma-1	XM_005158761.5
A20G0704	3.31	3.61	MYSM1	Histone H2A deubiquitinase MYSM1	NM_001164029.1
A19G0705	3.31	1.83	CKS1B	Cyclin-dependent kinases regulatory subunit 1	NM_001003593.3
A25G0446	3.31	1.00	IL17RE	Interleukin-17 receptor E	XM_002666890.6
A16G0243	3.31	2.76	CEP85	Centrosomal protein of 85 kDa	XM_685394.10
A3G0820	3.31	5.05	RBFOX1	RNA binding protein fox-1 homolog 1	NM_001005596.1
A15G0725	3.31	3.21	PIK3CB	Phosphatidylinositol 4,5-bisphosphate 3-kinase catalytic subunit beta isoform	XM_068213678.1
B25G0030	3.31	2.35	VPS4A	Vacuolar protein sorting-associated protein 4A	XM_009298174.4
B25G0578	3.31	3.16	DNAJC24	DnaJ homolog subfamily C member 24	
A8G0456	3.31	4.73	TBC1D25	TBC1 domain family member 25	NM_001128236.1
A4G0370	3.31	2.32	G5714_004123	A0A7J6DCN4_9TELE Uncharacterized protein	NM_001003622.2
B9G0889	3.31	2.65	PKP4	Plakophilin-4	
B25G0528	3.31	1.99	SETD6	N-lysine methyltransferase setd6	NM_199600.1
A8G0045	3.31	1.65	ZNF367	Zinc finger protein 367	NM_001017726.2
A25G0260	3.31	9.33	COQ9	Ubiquinone biosynthesis protein COQ9, mitochondrial	NM_001098746.1
A5G0858	3.31	4.44	LYSMD3	LysM and putative peptidoglycan-binding domain-containing protein 3	NM_001002104.1
A8G0098	3.31	8.28	GCN1	eIF-2-alpha kinase activator GCN1	XM_009303726.3
A22G0128	3.31	3.34	TNFAIP8L1	Tumor necrosis factor alpha-induced protein 8-like protein 1	NM_200332.2
B7G0275	3.31	5.51	ASPH	asparaginyl beta-hydroxylase	XM_021478003.2
B6G0317	3.30	3.71	DPH3	Diphthamide biosynthesis protein 3	NM_001159824.2
B24G0726	3.30	5.72	RALA	Ras-related protein Ral-A	NM_201018.1
B3G0374	3.30	6.03	G5714_003208	A0A7J6D8U9_9TELE Uncharacterized protein	
B3G0343	3.30	6.36	RAB3D	Ras-related protein Rab-3D	XM_005168476.5
B3G1124	3.30	3.93	SLC35B1	Solute carrier family 35 member B1	NM_001004583.1
B22G0061	3.30	5.95	GIMAP7	GTPase IMAP family member 7	XM_017354638.3
B3G0831	3.30	7.71	SETD1A	Histone-lysine N-methyltransferase SETD1A	XM_005164080.5

A1G0963	3.30	4.68	MTUS1A	Microtubule-associated tumor suppressor 1 homolog A	XM_021471156.2
A20G0798	3.30	4.00	PPP2R5A	threonine-protein phosphatase 2A 56 kDa regulatory subunit alpha isoform	XM_005160442.5
A9G0185	3.30	3.58	ROHU_017062	A0A498NHW5_LABRO Mannose-1-phosphate guanyltransferase-like protein	NM_001040372.1
B21G0423	3.30	15.49	SUPT6H	Transcription elongation factor SPT6	NM_145118.1
A20G0406	3.30	6.10	RAB32	Ras-related protein Rab-32	NM_201332.1
A3G0140	3.30	1.67	TXN2	Thioredoxin, mitochondrial	NM_205641.1
A23G0493	3.30	1.84	GALNT6	Polypeptide N-acetylgalactosaminyltransferase 6	NM_213196.1
B5G0555	3.30	4.97	JAK2	Tyrosine-protein kinase JAK2	NM_131087.1
B4G0832	3.30	4.00	A0A673IFZ5	A0A673IFZ5_9TELE Uncharacterized protein	NM_001089338.1
A3G1319	3.30	4.97	LOC107675477	A0A671KBP6_9TELE Gastrula zinc finger protein XlCGF57.1-like	XM_068220573.1
B5G0827	3.30	2.45	ALKBH6	Alpha-ketoglutarate-dependent dioxygenase alkB homolog 6	NM_001005390.1
B6G0931	3.30	11.72	UBA1	Ubiquitin-like modifier-activating enzyme 1	XM_005155876.5
A1G0910	3.30	281.10	ISK1	Probable pancreatic secretory proteinase inhibitor	
B18G0074	3.30	4.17	DVL3	Segment polarity protein dishevelled homolog DVL-3	XM_001920207.8
B2G0912	3.30	6.83	ARHGAP29	Rho GTPase-activating protein 29	NM_200111.2
A8G0839	3.30	4.42	CABIN1	Calcineurin-binding protein cabin-1	XM_009304316.4
B20G0492	3.30	6.53	AVEN	Cell death regulator Aven	NM_001045292.2
B16G0861	3.30	5.30	SETD2	Histone-lysine N-methyltransferase SETD2	XM_009292184.4
B20G0716	3.30	9.58	CX32.2	Gap junction Cx32.2 protein	NM_001030210.2
B5G0461	3.30	1.58	LOC113049753	A0A6P6KAJ9_CARAU uncharacterized protein LOC113049753 isoform X2	NM_001327796.1
B2G1003	3.30	0.36	G2E3	M phase-specific E3 ubiquitin-protein ligase	
B21G0384	3.30	6.36	DPP3	Dipeptidyl peptidase 3	NM_001002683.1
A21G0724	3.29	0.34	BRN3	POU domain protein 3	NM_001080058.1
B20G0477	3.29	5.70			
B12G0039	3.29	7.31	SEC31A	Protein transport protein Sec31A	XM_001921546.8
B4G0780	3.29	1.93	LOC113066133	A0A6P6MAN3_CARAU gastrula zinc finger protein XlCGF57.1-like	XM_009300923.3
A21G0839	3.29	4.08	ATG4A	Cysteine protease ATG4A	NM_001024434.1
B7G0677	3.29	4.94	ITLN	Intelectin	NM_001076581.1
A1G0545	3.29	3.08	TNIP2	TNFAIP3-interacting protein 2	XM_687625.9
B1G1138	3.29	2.53	LOC113099775	A0A6P6PJG2_CARAU zinc finger protein 883-like isoform X2	NM_001122845.1
B5G1180	3.29	6.56	CLOCK	Circadian locomoter output cycles protein kaput	NM_178299.1
B4G0656	3.29	3.73	TRIM24	Transcription intermediary factor 1-alpha	NM_001002870.2
B13G0420	3.29	4.71	URB2	Unhealthy ribosome biogenesis protein 2 homolog	XM_009307400.4

B6G0592	3.29	5.18	DDR2	Discoidin domain-containing receptor 2	XM_068222006.1
A11G0011	3.29	0.76	GEMIN6	Gem-associated protein 6	
B16G0633	3.29	6.10	LRATD2	Protein LRATD2	NM_001017837.1
B10G0410	3.29	3.09	FAM89A	Protein FAM89A	XM_001921384.7
A21G0189	3.29	4.14	PTPN13	Tyrosine-protein phosphatase non-receptor type 13	XM_068215903.1
B7G1107	3.29	5.66	CASPA	Caspase a	NM_001013502.1
B20G0252	3.29	2.19	TRIM16	Tripartite motif-containing protein 16	XM_693013.9
B25G0277	3.29	0.30	LAMB4	Laminin subunit beta-4	NM_173276.2
A17G0705	3.29	6.31	ATG14	Beclin 1-associated autophagy-related key regulator	NM_001024812.2
A1G0177	3.29	5.61	MRI1	Methylthioribose-1-phosphate isomerase	NM_001111238.2
B16G0174	3.29	2.72	PAQR6	Membrane progestin receptor delta	XM_017351556.3
A19G0011	3.29	2.67	DPX16_1179	A0A3N0XUS6_ANAGA Zinc finger protein 569	XM_068213808.1
A22G0448	3.29	5.11	ZBTB7A	Zinc finger and BTB domain-containing protein 7A	XM_005170531.5
B6G0127	3.29	1.82	HARBI1	Putative nuclease HARBI1	NM_001014319.2
B10G0325	3.28	4.56	BAP18	Chromatin complexes subunit BAP18	NM_001008605.1
B24G0268	3.28	1.76	ACP5	Tartrate-resistant acid phosphatase type 5	NM_001002452.1
A17G0626	3.28	15.91	MARCKS	Myristoylated alanine-rich C-kinase substrate	NM_001015060.2
A16G0613	3.28	10.79	PON2	arylesterase 2	NM_212734.1
A17G0331	3.28	3.86	LOC107674049	A0A671QY30_9TELE Uncharacterized protein	
A16G0714	3.28	4.27	CXCR3	C-X-C chemokine receptor type 3	NM_001144813.1
A5G0954	3.28	3.46	DHS-21	L-xylulose reductase	NM_001005597.1
B3G0067	3.28	2.56	LOC113093308	A0A6P6P2F6_CARAU gastrula zinc finger protein XlCGF26.1-like	XM_068217631.1
A9G0776	3.28	6.36	RBM45	RNA-binding protein 45	NM_001127402.1
B16G0649	3.28	5.83	CMKLR1	Chemerin-like receptor 1	XM_003200188.6
B17G0901	3.28	7.46	TGFB3	Transforming growth factor beta-3 proprotein	NM_194386.2
A15G0245	3.28	2.75	TNF	Tumor necrosis factor	
A5G0456	3.28	5.72	CCDC117	Coiled-coil domain-containing protein 117	NM_001123279.2
A6G0342	3.28	4.47	MTMR14	Myotubularin-related protein 14	XM_002662387.7
B6G0470	3.28	0.61	SOX14	Transcription factor Sox-14	NM_001037680.2
B19G0511	3.28	18.35	CLASP2	CLIP-associating protein 2	XM_068215074.1
B6G0057	3.28	2.35	ORC4	Origin recognition complex subunit 4	XM_005168697.3
B12G0313	3.28	2.53	MEOX1	Homeobox protein MOX-1	NM_001002450.2
A23G0134	3.28	0.35	SPIC	Transcription factor Spi-C	XM_009296622.3
B21G0087	3.28	2.78	TMLHE	Trimethyllysine dioxygenase, mitochondrial	XM_677739.7
A2G0984	3.28	1.49	MTERF4	Transcription termination factor 4, mitochondrial	
B23G0127	3.28	0.40	DPX16_8122	A0A3N0Y885_ANAGA Pro-FMRFamide-related neuropeptide FF	
A10G0294	3.28	3.87	ACER3	Alkaline ceramidase 3	NM_001293692.1
A18G0562	3.27	2.22	G5714_017690	A0A7J6C1W2_9TELE Deacetylase sirtuin-type domain-containing protein	

A5G0420	3.27	3.26	G5714_005951	A0A7J6D2G8_9TELE WRNPLPNID domain-containing protein	NM_001281985.1
A14G0745	3.27	4.14	RAB9B	Ras-related protein Rab-9B	XM_009307804.4
A19G0277	3.27	5.54	RIPOR2	Rho family-interacting cell polarization regulator 2	NM_001045026.1
B3G0148	3.27	1.38	LOC113096977	A0A6P6PAM1_CARAU gastrula zinc finger protein XlCGF26.1-like	XM_068220671.1
B17G0239	3.27	9.00	DAAM1	Disheveled-associated activator of morphogenesis 1	XM_017351772.3
B13G0737	3.27	7.58	LOXL4	Lysyl oxidase homolog 4	XM_005156601.5
B9G0270	3.27	2.10	INHBB	Inhibin beta B chain	NM_131068.2
B9G0357	3.27	4.58	PLCL1	Inactive phospholipase C-like protein 1	XM_001921763.8
B21G0978	3.27	1.50	AFF4	FMR2 family member 4	
A13G0701	3.27	9.07	MOCOS	Molybdenum cofactor sulfurase	XM_009307462.4
A10G0687	3.27	7.03	GGCX	Vitamin K-dependent gamma-carboxylase	XM_003199291.6
B16G0527	3.27	5.17	GON4L	GON-4-like protein	NM_001201535.1
A17G0677	3.27	0.31	IL17F	Interleukin-17F	
B15G0020	3.27	6.18	IFT80	Intraflagellar transport protein 80 homolog	NM_001008625.1
B17G0026	3.27	1.69	FMN1	Formin-1	XM_021467248.2
B23G0809	3.27	3.01	SRC	Proto-oncogene tyrosine-protein kinase Src	XM_017353519.3
B7G0345	3.27	10.41	BRMS1LA	Breast cancer metastasis-suppressor 1-like protein-A	NM_200097.1
B21G0575	3.27	3.44	BCL11B	leukemia 11B	XM_683166.10
A19G0133	3.27	5.81	CPSF1	Cleavage and polyadenylation specificity factor subunit 1	XM_005159584.5
B9G0078	3.27	1.18	EGFL6	Epidermal growth factor-like protein 6	NM_001002457.2
B9G0626	3.27	3.90	CHPF	Chondroitin sulfate synthase 2	XM_002663329.7
A3G0702	3.27	2.33	BCL2L12	Bcl-2-like protein 12	
B10G0485	3.27	5.43	ZBTB21	Zinc finger and BTB domain-containing protein 21	XM_690101.9
A19G0389	3.27	7.50	CASD1	N-acetylneuraminate 9-O-acetyltransferase	NM_001278180.1
A5G0829	3.26	4.61	FBP1	Fructose-1,6-bisphosphatase 1	NM_199942.2
A10G0382	3.26	5.04	EURL	Protein EURL homolog	NM_001020552.1
B4G0048	3.26	9.45	UPF2	Regulator of nonsense transcripts 2	
B11G0806	3.26	6.19			NM_200310.1
A21G0230	3.26	4.49	DCAF12	DDB1- and CUL4-associated factor 12	NM_001077580.1
A12G0041	3.26	1.54	SEC31A	Protein transport protein Sec31A	XM_001921546.8
B9G0426	3.26	4.27	REV1	DNA repair protein REV1	XM_009304637.4
A1G0809	3.26	17.16	MXA	Interferon-induced GTP-binding protein MxA	NM_182942.4
B22G1101	3.26	12.24	TM4SF1	Transmembrane 4 L6 family member 1	
A9G0295	3.26	4.66	CASK	Peripheral plasma membrane protein CASK	XM_017357643.3
B25G0548	3.26	5.37	SLC25A22	Mitochondrial glutamate carrier 1	XM_005174363.5
B1G1059	3.26	3.76	CD300LD3	CMRF35-like molecule 3	
A1G0996	3.26	1.67	RNF38	E3 ubiquitin-protein ligase RNF38	XM_690108.9
B14G0263	3.26	10.20			
A7G0722	3.26	10.12	GOLM2	Protein GOLM2	XM_005168941.5

B3G0659	3.26	5.59	ABCC1	Multidrug resistance-associated protein 1	XM_068219215.1
B19G0543	3.26	3.53	AGO2	Protein argonaute-2	NM_001302222.1
A21G0838	3.26	2.47	E1301_TTI019815	A0A5A9PEL3_9TELE Insulin receptor substrate 2-B	XM_002666075.7
B7G0216	3.26	5.50	MYO9AA	Unconventional myosin-IXAa	NM_001423815.1
A17G0758	3.26	4.79	ZBTB2	Zinc finger and BTB domain-containing protein 2	NM_200160.1
A4G0455	3.26	9.29	TMEM19	Transmembrane protein 19	NM_001002098.1
B15G0569	3.26	1.73	SLC46A1	Proton-coupled folate transporter	NM_200285.1
B2G1015	3.26	4.69	SFT2D3	Vesicle transport protein SFT2C	NM_001163995.1
A5G1133	3.26	0.93	BSPRY	B box and SPRY domain-containing protein	NM_001326435.1
B12G0381	3.26	7.80	MAP3K8	Mitogen-activated protein kinase kinase kinase 8	XM_687085.8
B2G0290	3.26	16.59	CREM	cAMP-responsive element modulator	XM_068214537.1
A13G0633	3.26	5.13	KY	Kyphoscoliosis peptidase	NM_001346164.1
B7G0004	3.26	0.28	HIS4	Histone H4	XM_068222533.1
B11G0593	3.26	4.86	BRK1	Probable protein BRICK1	XM_678953.6
A16G0515	3.26	1.94	RBM12B	RNA-binding protein 12B	XM_068214018.1
A10G0190	3.26	2.36	KCTD9	POZ domain-containing protein KCTD9	NM_001002738.1
B7G0451	3.25	7.10			
A1G0481	3.25	6.32	VPS37C	Vacuolar protein sorting-associated protein 37C	NM_001077785.1
A12G0100	3.25	2.60	FANK1	Fibronectin type 3 and ankyrin repeat domains 1 protein	
A10G0851	3.25	5.76	MAP3K1	Mitogen-activated protein kinase kinase kinase 1	XM_005155507.5
B22G0842	3.25	6.02	DNAL4	Dynein axonemal light chain 4	NM_001020502.1
A19G0203	3.25	4.64	SKIV2L	Helicase SKI2W	XM_005170047.4
B2G0840	3.25	3.06	FBXL7	LRR-repeat protein 7	NM_001080042.1
A19G0141	3.25	4.70	EEPD1	phosphatase family domain-containing protein 1	NM_205759.1
B4G0261	3.25	10.22	TSPAN9	Tetraspanin-9	XM_068219583.1
B15G0908	3.25	3.03	CLPB	Caseinolytic peptidase B protein homolog	XM_009292000.4
B23G0501	3.25	12.12	PKIG	cAMP-dependent protein kinase inhibitor gamma	NM_001309188.1
B14G0081	3.25	3.23	CPEB4	Cytoplasmic polyadenylation element-binding protein 4	NM_001351720.1
A4G0518	3.25	3.23	USP6NL	USP6 N-terminal-like protein	XM_005164877.5
A21G0873	3.25	3.33	SH3PXD2B	SH3 and PX domain-containing protein 2B	XM_003200907.5
B3G1234	3.25	4.10	MR1	Major histocompatibility complex class I-related gene protein	XM_001340377.8
B12G0525	3.25	5.32	RNLS	Renalase	NM_001002607.1
B3G0815	3.25	8.68	DMBT1	Deleted in malignant brain tumors 1 protein	
B11G0088	3.25	0.00	RPN2	Dolichyl-diphosphooligosaccharide--protein glycosyltransferase subunit 2	
A3G1109	3.25	4.39	E4F1	Transcription factor E4F1	NM_001025513.1
A5G0826	3.25	4.89	DAPK1	Death-associated protein kinase 1	NM_001099990.2
A3G1051	3.25	15.56	CBX7	Chromobox protein homolog 7	NM_001017853.2
A25G0087	3.25	2.78	MYO5C	Unconventional myosin-Vc	XM_686051.8
B1G0358	3.25	3.00	HARBI1	Putative nuclease HARBI1	

A16G0087	3.25	3.86	FFAR3	Free fatty acid receptor 3	XM_017351433.3
A5G0805	3.25	20.50	LOC107718359	A0A673LV56_9TELE C-X-C motif chemokine 10-like	
A3G0118	3.25	4.01	PMM1	Phosphomannomutase 1	XR_011009816.1
B21G0972	3.25	5.96	JAK2	Tyrosine-protein kinase JAK2	NM_131093.1
A20G0186	3.25	2.01	WDCP	WD repeat and coiled-coil-containing protein	NM_001013534.2
B13G0354	3.25	1.97	PXN1	Jeltraxin	
A23G0307	3.25	6.42	TRPC4AP	Short transient receptor potential channel 4-associated protein	NM_001310093.1
A9G0214	3.25	0.80	BCS1L	Mitochondrial chaperone BCS1	NM_201182.2
B18G0327	3.25	5.39	LOC113073439	A0A6P6N0L9_CARAU HMG domain-containing protein 3-like isoform X2	
B10G0358	3.25	0.59	VPS36	Vacuolar protein-sorting-associated protein 36	NM_199977.1
A15G0679	3.24	6.71	BACH1	Transcription regulator protein BACH1	NM_001040313.2
B22G0487	3.24	0.46	A0A671LAR5	A0A671LAR5_9TELE Ig-like domain-containing protein	XM_001346243.8
B1G0295	3.24	17.69	SORBS2	Sorbin and SH3 domain-containing protein 2	XM_068217979.1
A4G0662	3.24	1.53			
B4G0451	3.24	4.41	CWF19L1	CWF19-like protein 1	NM_001044758.1
B18G0503	3.24	5.16	SMAD6	Mothers against decapentaplegic homolog 6	NM_001045051.2
B1G0240	3.24	7.55	GNE	N-acetylmannosamine kinase	XM_068214418.1
A5G1309	3.24	3.56	EPHB4A	Ephrin type-B receptor 4a	XM_021476001.2
B22G0324	3.24	5.65	LOC113078540	A0A6P6NEM3_CARAU uncharacterized protein LOC113078540 isoform X1	XM_005161637.5
A7G1073	3.24	1.67	ECSIT	Evolutionarily conserved signaling intermediate in Toll pathway, mitochondrial	NM_001242938.1
A8G0370	3.24	2.70	REXO1	RNA exonuclease 1 homolog	NM_001126416.1
A5G0538	3.24	2.13	HINFP	Histone H4 transcription factor	NM_213424.3
A1G0297	3.24	5.38	A0A673FZL3	A0A673FZL3_9TELE Uncharacterized protein	
A6G0554	3.24	56.14	PGM1	Phosphoglucomutase-1	NM_201025.1
B23G0778	3.24	3.08	CDH24	Cadherin-24	XM_021470042.2
A19G0323	3.24	3.17	PEX1	Peroxisome biogenesis factor 1	NM_001170835.1
B3G0132	3.24	0.48	TRIM35	E3 ubiquitin-protein ligase TRIM35	NM_001045009.3
B22G0911	3.24	0.00	VTCN1	V-set domain-containing T-cell activation inhibitor 1	
B1G0547	3.24	4.62	ARL13B	ADP-ribosylation factor-like protein 13B	XM_068221797.1
B3G1682	3.24	2.09	LOC107700625	A0A671SS62_9TELE Gastrula zinc finger protein XlCGF7.1-like	XM_017355700.3
B4G0620	3.24	2.14	ALG10	Dol-P-Glc:Glc(2)Man(9)GlcNAc(2)-PP-Dol alpha-1,2-glucosyltransferase	NM_001327833.1
B4G0645	3.24	0.92	SAPCD1	Suppressor APC domain-containing protein 1	
B20G0268	3.24	5.04	B4GALT6	Beta-1,4-galactosyltransferase 6	NM_200938.1
B13G0619	3.24	5.52	TRIP11	Thyroid receptor-interacting protein 11	NM_205603.1
B13G0744	3.24	11.13	GOT1	Aspartate aminotransferase, cytoplasmic	NM_001017593.2

B6G0003	3.23	4.33	BMERB1	bMERB domain-containing protein 1	
A6G0421	3.23	5.19	GRB2	Growth factor receptor-bound protein 2	NM_001007769.1
B1G0042	3.23	6.95	JAM2A	Junctional adhesion molecule 2A	
B7G0950	3.23	4.54	CHSY1	Chondroitin sulfate synthase 1	NM_212678.1
B3G0520	3.23	10.42	TYK2	Non-receptor tyrosine-protein kinase TYK2	XM_005164236.5
B9G0678	3.23	6.61	TBA	Tubulin alpha chain	NM_001003558.2
B5G1313	3.23	13.81	HK2	Hexokinase-2	NM_213066.1
B3G0229	3.23	10.00	MCOLN1	Mucolipin-1	NM_001386626.1
A16G0230	3.23	6.42	DYRK1A	Dual specificity tyrosine-phosphorylation-regulated kinase 1A	XM_005158215.5
A13G0473	3.23	4.62	MSXC	Homeobox protein MSH-C	NM_001126397.1
A1G0549	3.23	3.43	ARHGAP10	Rho GTPase-activating protein 10	NM_001045250.1
A7G1088	3.23	2.35	KV5AK	Ig kappa chain V-V region J606	NM_131649.2
B1G0293	3.23	3.04	UFSP2	Ufm1-specific protease 2	XM_005168337.5
A20G0137	3.23	3.87	GPATCH2	G patch domain-containing protein 2	NM_001326693.2
A7G0739	3.23	2.47	POLR2M	DNA-directed RNA polymerase II subunit GRINL1A	NM_001346180.1
B5G1120	3.23	3.82	SENP3	Sentrin-specific protease 3	NM_001105114.1
B18G0620	3.23	3.35	VPS9D1	VPS9 domain-containing protein 1	XM_068214762.1
B10G0336	3.23	0.45	CLEC4M	C-type lectin domain family 4 member M	XM_068218586.1
A3G0655	3.23	2.48	ALKBH7	Alpha-ketoglutarate-dependent dioxygenase alkB homolog 7, mitochondrial	NM_001017609.1
B3G0488	3.23	4.23	P2RY11	P2Y purinoceptor 11	
A1G1085	3.23	2.85	SLIT2	Slit homolog 2 protein	XM_068220867.1
A13G0654	3.23	7.55	BTN2A2	Butyrophilin subfamily 2 member A2	NM_199794.1
A3G0350	3.23	3.42	CDC42EP4	Cdc42 effector protein 4	NM_001002183.1
A14G0299	3.23	10.12	SSH3	Protein phosphatase Slingshot homolog 3	XM_021474332.2
B7G1007	3.22	2.33	KIF7	Kinesin-like protein kif7	NM_001014816.1
B20G0130	3.22	5.97	WU:FA18F11	Uncharacterized protein C1orf198 homolog	NM_001201453.2
A21G0425	3.22	3.40	SLC35F2	Solute carrier family 35 member F2	NM_001076556.1
B14G0189	3.22	6.38	SESN3	Sestrin-3	XM_009291172.4
A12G0298	3.22	6.90	NFE2L1	Endoplasmic reticulum membrane sensor NFE2L1	NM_001278842.1
B5G0702	3.22	1.52	DPX16_22523	A0A3N0YYP8_ANAGA IGv domain-containing protein	
B22G0529	3.22	6.03	BAP1	Ubiquitin carboxyl-terminal hydrolase BAP1	NM_001163837.1
B5G0049	3.22	16.91	PLA2G4C	Cytosolic phospholipase A2 gamma	NM_001007430.2
B17G0585	3.22	2.30	RPAP1	RNA polymerase II-associated protein 1	XM_021473203.2
B19G0455	3.22	2.74	PTDSS1	Phosphatidylserine synthase 1	XM_682780.10
B22G0145	3.22	5.47	KDM4B	Lysine-specific demethylase 4B	NM_001082805.1
B15G0190	3.22	3.60	BACE2	Beta-secretase 2	XM_009291473.4
A6G0564	3.22	3.94	ZC3H3	Zinc finger CCCH domain-containing protein 3	XM_684588.6
B18G0372	3.22	4.05	NUCB2	Nucleobindin-2	NM_201479.1
B8G1020	3.22	2.72	DGCR6	Protein DGCR6	XM_017357308.3

B2G0345	3.22	2.54	CCL4	C-C motif chemokine 4	
A8G0603	3.22	5.38	BCR	Breakpoint cluster region protein	XM_005167174.5
B19G0188	3.22	1.21	EDN1	Endothelin-1	NM_131519.1
A5G1320	3.22	5.92	ABHD10	Palmitoyl-protein thioesterase ABHD10, mitochondrial	NM_001326523.1
A17G0125	3.22	1.59	TRB	M1-specific T cell receptor beta chain	XM_068214488.1
B3G0376	3.22	1.31	MYADM	Myeloid-associated differentiation marker	XM_003197991.6
B9G0717	3.22	6.70	COL4A2	Collagen alpha-2(IV) chain	XR_011016782.1
A15G0874	3.22	5.10	CEP295	Centrosomal protein of 295 kDa	XM_021466549.2
B15G0231	3.22	5.08	TCB2	Transposable element Tcb2 transposase	XR_002459242.2
B6G0646	3.22	8.05	VDRB	Vitamin D3 receptor B	NM_001159985.1
B19G0423	3.22	4.89	ANXA2	Annexin A2	
B7G0358	3.21	4.93	PLAA	Phospholipase A-2-activating protein	NM_212690.1
B1G1199	3.21	4.12	EARS2	Probable glutamate--tRNA ligase, mitochondrial	NM_001045442.2
A16G0491	3.21	4.39	TRIM33	E3 ubiquitin-protein ligase TRIM33	XM_005158077.5
A16G0823	3.21	4.00	CHN2	Beta-chimaerin	XM_694550.10
A15G0100	3.21	8.66	KCNE4	Potassium voltage-gated channel subfamily E member 4	NM_001082897.1
A3G1101	3.21	11.72	TNPO2	Transportin-2	NM_001004003.2
A6G0491	3.21	5.09	RYK	Tyrosine-protein kinase RYK	XM_005165931.5
A15G0635	3.21	5.66	FBXO46	F-box only protein 46	NM_001082831.1
A6G0302	3.21	3.70	TGFBRAP1	Transforming growth factor-beta receptor-associated protein 1 homolog	NM_001083554.1
B4G0001	3.21	3.58	RSBN1L	Lysine-specific demethylase RSBN1L	
A20G0411	3.21	2.43	UST	Uronyl 2-sulfotransferase	NM_001032729.1
B2G0817	3.21	5.87	OXSR1	threonine-protein kinase OSR1	NM_001098747.1
A8G0129	3.21	4.85	CXADR	Coxsackievirus and adenovirus receptor homolog	NM_001130781.2
A10G0029	3.21	4.10	RFX3	Transcription factor RFX3	NM_001080048.1
A17G0096	3.21	10.49	MOCS1	Molybdenum cofactor biosynthesis protein 1	NM_001105660.2
B2G0980	3.21	2.00	TCEANC2	Transcription elongation factor A N-terminal and central domain-containing protein 2	
A6G0412	3.21	11.43	ZNF207	BUB3-interacting and GLEBS motif-containing protein ZNF207	XM_009302342.4
B21G0343	3.21	14.95	TIMD4	T-cell immunoglobulin and mucin domain-containing protein 4	XM_021468804.2
B14G0554	3.21	8.81	DNAJC4	DnaJ homolog subfamily C member 4	NM_212677.1
A4G0106	3.21	2.06	G5714_004461	A0A7J6D4R8_9TELE Uncharacterized protein	XM_068220569.1
A18G0453	3.21	5.94	KIF23	Kinesin-like protein KIF23	XM_021467586.2
A22G0087	3.21	1.78	GPR55	G-protein coupled receptor 55	XM_002667652.7
A25G0163	3.21	1.37	TPH1	Tryptophan 5-hydroxylase	NM_178306.3
A6G0728	3.21	2.87	EIF4ENIF1	Eukaryotic translation initiation factor 4E transporter	XM_068221718.1
B6G0893	3.21	2.31	MTSS1	Protein MTSS 1	XM_068221739.1
B6G0176	3.21	4.57	ACP5	Tartrate-resistant acid phosphatase type 5	NM_214773.1
A14G0105	3.21	4.11	MATR3	Matrin-3	XM_068213212.1

B13G0068	3.21	6.84	DNAAF10	Dynein axonemal assembly factor 10	XM_682645.10
B10G0040	3.21	7.94	APH1B	Gamma-secretase subunit Aph-1b	NM_001030129.2
A7G1018	3.21	2.16	MUS81	Crossover junction endonuclease MUS81	XM_005172211.5
A18G0334	3.21	3.04	A0A671Q0E0	A0A671Q0E0_9TELE Uncharacterized protein	
A20G0625	3.21	2.85	PRKCH	Protein kinase C eta type	XM_005160500.5
B5G1239	3.21	4.56	SSH1	Protein phosphatase Slingshot homolog 1	XM_005165198.5
B9G0301	3.20	1.32	GRTP1A	Growth hormone-regulated TBC protein 1-A	XR_011016725.1
B6G0750	3.20	6.26	SLC6A13	Sodium- and chloride-dependent GABA transporter 2	XM_005166019.5
A19G0234	3.20	1.57	E2F3	Transcription factor E2F3	NM_001080628.2
A21G0832	3.20	4.67	TCB1	Transposable element Tcb1 transposase	XM_021471168.2
B21G0198	3.20	3.75	SIAH2L	E3 ubiquitin-protein ligase Siah2	NM_200427.2
B10G0519	3.20	1.06	HUNK-A	Hormonally up-regulated neu tumor-associated kinase homolog A	XM_005172726.5
B22G1118	3.20	6.67	TASOR	Protein TASOR	XM_009296539.4
A12G0648	3.20	2.62	TACO1	Translational activator of cytochrome c oxidase 1	NM_001316971.1
B4G0331	3.20	8.66	DERA	Deoxyribose-phosphate aldolase	NM_001006097.2
A5G0012	3.20	3.66	RNF185	E3 ubiquitin-protein ligase RNF185	NM_213037.1
A14G0219	3.20	2.71	ZIC3	Zinc finger protein ZIC 3	NM_001001950.2
A9G0569	3.20	4.75	PARP4	Protein mono-ADP-ribosyltransferase PARP4	XM_017357877.2
B22G1064	3.20	2.28	HYKK	Hydroxylysine kinase	NM_001113587.2
A5G0830	3.20	3.97	KANK1	KN motif and ankyrin repeat domain-containing protein 1	XM_005165446.5
B24G0499	3.20	4.04	TNFRSF11A	Tumor necrosis factor receptor superfamily member 11A	XM_005162629.5
A4G0296	3.20	4.62	NLRC3	NLR family CARD domain-containing protein 3	XM_017352412.3
B3G1537	3.20	1.43	A0A672LA92	A0A672LA92_SINGR Uncharacterized protein	XM_068213808.1
A20G0577	3.20	3.48	ANAPC4	Anaphase-promoting complex subunit 4	XM_017352471.3
B20G0111	3.20	8.88	CYP46A1	Cholesterol 24-hydroxylase	NM_001045298.1
B6G0452	3.20	9.97	BTBD8	POZ domain-containing protein 8	XM_068221969.1
B16G0295	3.20	2.27	NDUFAF6	NADH dehydrogenase (ubiquinone) complex I, assembly factor 6	XM_021466778.2
A8G0387	3.20	6.15	SPRYD4	SPRY domain-containing protein 4	NM_001002477.1
B25G0126	3.20	4.20	NUDT7	Peroxisomal coenzyme A diphosphatase NUDT7	
A7G0207	3.20	3.45	SBF2	Myotubularin-related protein 13	XM_009303601.4
A25G0684	3.20	2.55	IGKV4-1	Immunoglobulin kappa variable 4-1	XM_068217596.1
A11G0459	3.20	3.30	RAI2	Retinoic acid-induced protein 2	XM_017358249.3
B15G0019	3.20	4.47	SMC4	Structural maintenance of chromosomes protein 4	NM_173253.2
B19G0544	3.20	4.59	DLX5A	Homeobox protein Dlx5a	NM_131306.2
A17G0731	3.19	9.18	ZRANB1B	Ubiquitin thioesterase zranb1-B	NM_001077768.1

B4G0103	3.19	7.23	PIK3CG	Phosphatidylinositol 4,5-bisphosphate 3-kinase catalytic subunit gamma isoform	NM_213306.1
A8G0135	3.19	3.85	WDR13	WD repeat-containing protein 13	NM_001023573.1
B16G0847	3.19	1.94	DSCC1	Sister chromatid cohesion protein DCC1	NM_001004611.1
B22G0428	3.19	0.00	HEPACAM	Hepatocyte cell adhesion molecule	XM_017353283.3
B22G0659	3.19	3.63	OSBPL1A	Oxysterol-binding protein-related protein 1	XM_021473458.2
A12G0693	3.19	2.76	TFAM	Transcription factor A, mitochondrial	NM_001077389.1
B19G0946	3.19	1.14	G5714_018247	Ly6 domain-containing protein	
A7G1222	3.19	2.84	PRKD3	threonine-protein kinase D3	NM_001177454.1
B1G0896	3.19	1.20	ACY3.1	N-acyl-aromatic-L-amino acid amidohydrolase (carboxylate-forming) A	NM_001002347.1
A12G0722	3.19	5.52	TBC1D12	TBC1 domain family member 12	XM_005169448.5
B4G0807	3.19	1.49	A0A672PUH7	A0A672PUH7_SINGR Uncharacterized protein	XM_068220363.1
A1G0653	3.19	5.82	RAB9A	Ras-related protein Rab-9A	NM_001098741.1
B11G0201	3.19	4.55	ZGPAT	Zinc finger CCCH-type with G patch domain-containing protein	NM_201113.1
A17G0582	3.19	4.55	SLC22A15B	Solute carrier family 22 member 15-like	NM_001109699.1
A11G0325	3.19	3.37	CHIA	Acidic mammalian chitinase	
A25G0655	3.19	2.35	IGKV3-15	Immunoglobulin kappa variable 3-15	XM_068217596.1
B1G0470	3.19	1.35	TCEANC	Transcription elongation factor A N-terminal and central domain-containing protein	
A11G0250	3.19	15.05	TAFA4	Chemokine-like protein TAFA-4	NM_001164027.1
B4G0831	3.19	2.52	LOC113066842	A0A6P6MDE2_CARAU gastrula zinc finger protein XlCGF8.2DB-like	XM_068220183.1
A22G0643	3.19	1.21	KV2A5	Ig kappa chain V-II region 17S29.1	XM_068219015.1
B22G0279	3.19	3.75	A0A671LX12	A0A671LX12_9TELE Uncharacterized protein	XM_068216405.1
A18G0611	3.19	3.59	NUP205	Nuclear pore complex protein Nup205	NM_001313687.1
B18G0042	3.19	6.48	FLI1	Friend leukemia integration 1 transcription factor	XM_021467582.2
B12G0188	3.19	1.60	PGS1	CDP-diacylglycerol--glycerol-3-phosphate 3-phosphatidyltransferase, mitochondrial	XM_003199632.6
A22G0667	3.19	4.82	ARMH3	Armadillo-like helical domain-containing protein 3	NM_200619.2
A9G0627	3.19	2.26	TTLL4	Tubulin monoglutamylase TTLL4	NM_001430833.1
B16G0469	3.19	13.06	SLA	Src-like-adapter	
A12G0380	3.19	3.09	FOXN2	Forkhead box protein N2	NM_001017555.2
B23G0174	3.18	5.97	NAV1	Neuron navigator 1	XM_068216849.1
B5G0478	3.18	0.75	ZNF541	Zinc finger protein 541	
A20G0152	3.18	0.90	ADGRG2	Adhesion G-protein coupled receptor G2	XM_017352711.3
B5G0309	3.18	2.55	G5714_006223	A0A7J6D397_9TELE Uncharacterized protein	NM_001326457.1
A13G0170	3.18	3.91	FAM135A	Protein FAM135A	

A19G0531	3.18	4.76	SMARCC1	SNF complex subunit SMARCC1	XM_068215128.1
A10G0413	3.18	3.47	TBL2	Transducin beta-like protein 2	NM_212767.1
A17G0010	3.18	9.01	SERPINA10	Protein Z-dependent protease inhibitor	XM_009293350.4
B15G0720	3.18	6.64	SESN3	Sestrin-3	NM_213519.2
A19G0206	3.18	7.29	SACM1LA	Phosphatidylinositol-3-phosphatase SAC1-A	NM_001044878.2
A23G0804	3.18	3.69	RPRD1B	Regulation of nuclear pre-mRNA domain-containing protein 1B	XM_005162423.2
A5G0666	3.18	1.21	ALKBH6	Alpha-ketoglutarate-dependent dioxygenase alkB homolog 6	NM_001005390.1
B1G0815	3.18	3.67	TAF5	Transcription initiation factor TFIID subunit 5	NM_001037431.2
A21G0085	3.18	4.26	DOLPP1	Dolichyldiphosphatase 1	NM_001007061.1
B23G0430	3.18	5.78	EIF4G3	Eukaryotic translation initiation factor 4 gamma 3	XM_068219396.1
B22G0884	3.18	2.66	SODC	Superoxide dismutase [Cu-Zn]	XM_001343614.8
B13G0303	3.18	2.48	ATP10D	Phospholipid-transporting ATPase VD	NM_001006031.1
A14G0631	3.18	12.05	PCDHA3	Protocadherin alpha-3	NM_001008484.1
B3G0438	3.18	4.17	CDC42EP4	Cdc42 effector protein 4	NM_001002183.1
A25G0002	3.18	5.81	MON2	Protein MON2 homolog	XM_021470577.2
A13G0493	3.18	4.90	COG2	Conserved oligomeric Golgi complex subunit 2	NM_001076727.2
B6G0932	3.18	1.59	FAM212AB	PAK4-inhibitor inka2	NM_199788.1
A17G0528	3.18	3.61	IKZF5	Zinc finger protein Pegasus	XM_009293021.4
B2G0453	3.18	3.49	KLHL6	Kelch-like protein 6	NM_001005316.2
A3G0605	3.18	2.25	COIL	Coilin	NM_131512.2
B2G1288	3.18	6.13	TRIM16	Tripartite motif-containing protein 16	XM_681071.8
A1G1121	3.18	6.02	TET2	Methylcytosine dioxygenase TET2	XM_005159903.5
B13G0468	3.17	2.96	BAG3	BAG family molecular chaperone regulator 3	NM_001082860.1
B21G0287	3.17	7.70	NDST1	N-sulfotransferase 1	NM_001324455.1
A5G0460	3.17	15.98	ARAF	threonine-protein kinase A-Raf	XM_005171818.5
A20G0321	3.17	4.41	TNFRSF21	Tumor necrosis factor receptor superfamily member 21	NM_001042688.3
B22G0407	3.17	2.19	DPX16_1947	A0A3N0Y742_ANAGA Zinc finger protein 595	XM_068219202.1
A15G0204	3.17	15.95	IGFLR1	IGF-like family receptor 1	
A9G0742	3.17	5.38	PTGFRN	Prostaglandin F2 receptor negative regulator	
A5G0281	3.17	30.78	UBIQP	Polyubiquitin	
A6G0334	3.17	3.65	PRKCA	Protein kinase C alpha type	XM_021476862.2
A4G0839	3.17	4.59	TTLL12	Tubulin--tyrosine ligase-like protein 12	NM_200252.1
B19G0629	3.17	3.02	OTUD6B	Deubiquitinase OTUD6B	NM_200225.1
B16G0466	3.17	6.14	PHF20L1	PHD finger protein 20-like protein 1	XM_021466935.2
B10G0004	3.17	1.45	DPX16_2875	A0A3N0ZA51_ANAGA Zinc finger protein 271	XM_068218685.1
B7G0670	3.17	8.19	TLE3B	Transducin-like enhancer protein 3-B	XM_009303191.4
B3G1040	3.17	8.98	STAT5B	Signal transducer and activator of transcription 5B	NM_194387.2
A3G0043	3.17	2.18	NEK1	threonine-protein kinase Nek1	XM_017356939.3
B19G0325	3.17	7.13	IRGC	Interferon-inducible GTPase 5	NM_001320423.1
A20G0468	3.17	8.53	DPF3	Zinc finger protein DPF3	XM_005160743.5

A14G0401	3.17	1.31	C1QTNF2	Complement C1q tumor necrosis factor-related protein 2	XM_009291263.4
A1G0095	3.17	1.78	CD300LF	CMRF35-like molecule 1	
B23G0337	3.17	2.51	NUDT16	U8 snoRNA-decapping enzyme	XM_009296728.4
B8G0132	3.17	2.27	FAM110A	Protein FAM110A	XR_011016699.1
A19G0381	3.17	1.77	HEPACAM2	HEPACAM family member 2	NM_001245085.1
B3G1287	3.17	1.44	CBX4	E3 SUMO-protein ligase CBX4	NM_205749.1
A6G0141	3.17	1.94	ERBB3	Receptor tyrosine-protein kinase erbB-3	XM_005165701.5
B21G0709	3.16	6.59	RDH12	Retinol dehydrogenase 12	NM_200377.2
A22G0625	3.16	17.10	ADD3	Gamma-adducin	XM_068216305.1
A21G0535	3.16	0.93	TMSB	Thymosin beta	
B11G0455	3.16	3.46	PLCH2	1-phosphatidylinositol 4,5-bisphosphate phosphodiesterase eta-2	NM_001282078.1
B3G0675	3.16	4.88	COG1	Conserved oligomeric Golgi complex subunit 1	XM_002661160.7
A8G0230	3.16	5.48	CEPT1	ethanolaminephosphotransferase 1	NM_001144792.3
B10G0276	3.16	3.61	RASSF2	Ras association domain-containing protein 2	NM_001002195.1
B4G0658	3.16	3.46	PTPRR	Receptor-type tyrosine-protein phosphatase R	XM_021475305.2
A2G0401	3.16	5.35	PTBP2	Polypyrimidine tract-binding protein 2	NM_001100007.1
B14G0694	3.16	20.26	LYG	Lysozyme g	NM_001002706.1
A4G0218	3.16	2.44	LOC113047446	A0A6P6JXS5_CARAU gastrula zinc finger protein XlCGF57.1-like isoform X1	XM_068220573.1
A16G0135	3.16	2.52	LOC107726691	A0A673GT57_9TELE Uncharacterized LOC107726651	XM_009292662.4
B8G0673	3.16	5.99	MYO1F	Unconventional myosin-If	NM_001256671.2
B22G0963	3.16	5.36	CD276	CD276 antigen homolog	
B1G0624	3.16	5.28	ZFP91	E3 ubiquitin-protein ligase ZFP91	XM_021471225.2
A6G0265	3.16	5.08	PNKD	Probable hydrolase PNKD	NM_001256210.1
B14G0734	3.16	2.69	A33	Zinc-binding protein A33	NM_001130662.1
B1G0333	3.16	5.79	UBA6	Ubiquitin-like modifier-activating enzyme 6	XM_690663.9
A6G0157	3.16	4.22	ODAD3	Outer dynein arm-docking complex subunit 3	NM_001077369.2
A5G1054	3.16	4.04	CCDC15	Coiled-coil domain-containing protein 15	XM_009301883.3
B16G0694	3.16	4.91	SNX27	Sorting nexin-27	XM_005158092.5
B2G0251	3.16	1.63	MUL1A	Mitochondrial ubiquitin ligase activator of nfkb 1-A	NM_001018124.1
B23G0299	3.16	2.88	DNMT3B	DNA (cytosine-5)-methyltransferase 3B	NM_001025450.1
A19G0497	3.16	4.56	EXT1B	Exostosin-1b	NM_001012369.2
B11G0668	3.16	5.61	EMP3	Epithelial membrane protein 3	NM_131233.1
A20G0249	3.16	3.19	HDDC2	5'-deoxynucleotidase HDDC2	NM_001045231.1
A10G0526	3.16	8.38	BACH1	Transcription regulator protein BACH1	NM_001384147.1
A12G0826	3.16	1.35	MED9	Mediator of RNA polymerase II transcription subunit 9	XM_002667433.7
B21G0846	3.16	4.74	NUP188	Nucleoporin NUP188	XM_001922983.6
B20G0640	3.16	6.81	ENAH	Protein enabled homolog	NM_001045028.3
B17G0326	3.15	4.36	ECD	Protein ecdysoneless homolog	NM_199891.2
B2G0991	3.15	1.08	LHX8	homeobox protein Lhx8	XM_005171154.5
B19G0279	3.15	5.38	CNOT3	CCR4-NOT transcription complex subunit 3	XM_017352187.3

A1G0373	3.15	4.40	MCF2L	Guanine nucleotide exchange factor DBS	XM_021477145.2
B19G0329	3.15	6.20	KV12	Ig kappa chain V region 3368	
A5G1336	3.15	4.68	MLXIP	MLX-interacting protein	NM_001317759.2
B3G0138	3.15	2.35	G5714_023023	A0A7J6BQ16_9TELE Uncharacterized protein	XM_068218685.1
A13G0332	3.15	4.48	ANTXR1	Anthrax toxin receptor 1	NM_001142592.1
A16G0676	3.15	3.73	SETDB1B	Histone-lysine N-methyltransferase SETDB1-B	NM_001077277.3
A11G0646	3.15	1.24	R3HDM4	R3H domain-containing protein 4	NM_200531.2
A4G0253	3.15	8.18	NLRP3	NACHT, LRR and PYD domains-containing protein 3	XM_017352412.3
A21G0603	3.15	0.18			
B9G0060	3.15	0.00			
A21G0885	3.15	2.92	KIF20A	Kinesin-like protein KIF20A	NM_001161470.1
A18G0028	3.15	6.96	DUSP8	Dual specificity protein phosphatase 8	XM_002665301.7
B2G0325	3.15	2.83	CSPP1	Centrosome and spindle pole-associated protein 1	XM_694098.9
B2G0245	3.15	3.40	EIF4E2	Eukaryotic translation initiation factor 4E type 2	NM_001014815.2
B14G0538	3.15	6.38	BRD8	Bromodomain-containing protein 8	XM_068213171.1
B4G0414	3.15	5.91	KERA	Keratocan	NM_001025548.2
A1G1071	3.14	3.23	RFC1	Replication factor C subunit 1	NM_001089491.2
A24G0601	3.14	20.15	ANXA13	Annexin A13	NM_131774.2
A6G0178	3.14	5.08	PLCXD1	PI-PLC X domain-containing protein 1	XM_009302157.4
B25G0400	3.14	28.56	C11ORF96	Uncharacterized protein C11orf96	
A13G0790	3.14	4.51	ETAA1	Ewing's tumor-associated antigen 1	NM_001002198.1
B14G0191	3.14	3.46	ALG13	UDP-N-acetylglucosamine transferase subunit ALG13 homolog	
B8G0891	3.14	3.15	ELK1	ETS domain-containing protein Elk-1	XM_691202.8
A16G0407	3.14	0.49	STYK1	Tyrosine-protein kinase STYK1	NM_001305565.1
A14G0461	3.14	3.41	TSPAN17	Tetraspanin-17	NM_001123045.2
A7G0586	3.14	2.40	CREB3L1	Cyclic AMP-responsive element-binding protein 3-like protein 1	XM_005168966.4
B7G1105	3.14	6.82	SLC7A7	Y+L amino acid transporter 1	NM_001037559.2
A20G0199	3.14	3.01	LIN9	Protein lin-9 homolog	NM_001044946.1
B25G0700	3.14	0.10	MUC5B	Mucin-5B	XM_021470610.2
A3G0643	3.14	0.97			XM_005164016.5
B8G0722	3.14	3.88	NLRC3	NLR family CARD domain-containing protein 3	XM_068222163.1
A4G0162	3.14	0.14	LOC113046018	A0A6P6JTT2_CARAU uncharacterized protein LOC113046018	
B16G0353	3.14	30.85	COL6A6	Collagen alpha-6(VI) chain	XM_068214283.1
B2G1230	3.14	3.67	EXOSC4	Exosome complex component RRP41	XM_021466653.2
B18G0016	3.14	1.39	CD276	CD276 antigen	
B23G0826	3.14	10.20	CYP2B12	Cytochrome P450 2B12	NM_001109701.2
A1G0894	3.14	4.92	GNA12	Guanine nucleotide-binding protein subunit alpha-12	XM_068221006.1
A1G0336	3.14	2.12	GSK3B	Glycogen synthase kinase-3 beta	NM_001145688.1
A7G0731	3.14	3.51	MYO1E	Unconventional myosin-Ie	XM_005168926.5

A15G0324	3.14	1.77	VEZF1	Vascular endothelial zinc finger 1	NM_001079963.1
B1G0626	3.14	2.35	GIMAP7	GTPase IMAP family member 7	
A6G0738	3.14	2.99	SLC6A13	Sodium- and chloride-dependent GABA transporter 2	XM_005166019.5
B23G0964	3.14	5.63	TSC22D4	TSC22 domain family protein 4	NM_001077734.1
B8G0765	3.14	9.19	CALR	Calreticulin	NM_199713.2
A12G0594	3.14	6.94	ACLY	ATP-citrate synthase	XM_009306539.2
A13G0644	3.14	2.09	EMX1	Homeobox protein EMX1	NM_001012376.1
A18G0078	3.14	4.75	PCYT1A	Choline-phosphate cytidylyltransferase A	NM_001020735.1
A1G0033	3.14	1.73	MFAP4	Microfibril-associated glycoprotein 4	NM_001328078.1
B21G0125	3.14	18.32	CPEB4	Cytoplasmic polyadenylation element-binding protein 4	NM_001351713.1
B1G0051	3.14	4.74	LOC113044667	A0A6P6JLF9_CARAU uncharacterized protein LOC113044667 isoform X1	
A5G1078	3.13	3.41	PRPF4	U6 small nuclear ribonucleoprotein Prp4	NM_199755.1
B18G0006	3.13	6.51	PPFIA1	Liprin-alpha-1	NM_001111244.1
B1G0599	3.13	7.91	FBXO8	F-box only protein 8	NM_001040307.1
A19G0076	3.13	14.71	GMEB1	Glucocorticoid modulatory element-binding protein 1	XM_005159542.5
A10G0425	3.13	6.51	ZBTB21	Zinc finger and BTB domain-containing protein 21	XM_690101.9
B13G0546	3.13	1.40	PPA1	Inorganic pyrophosphatase	NM_201500.1
B1G0832	3.13	6.18	NPNT	Nephronectin	XM_009294216.4
B20G0057	3.13	3.66	UBE2J1	Ubiquitin-conjugating enzyme E2 J1	NM_214767.1
A14G0346	3.13	3.05	CENPI	Centromere protein I	NM_001386250.1
A3G0164	3.13	5.73	BTN3A3	Butyrophilin subfamily 3 member A3	XM_021472975.2
B12G0706	3.13	3.85	PLEKHM1	Pleckstrin homology domain-containing family M member 1	NM_001327851.1
B8G0886	3.13	4.45			
A16G0519	3.13	2.14	INVS	Inversin	XM_005158039.5
A22G0381	3.13	13.92	SBNO2	Protein strawberry notch homolog 2	NM_001423267.1
B6G0282	3.13	11.28	CASP8	Caspase-8	NM_131510.2
A2G0269	3.13	0.65	IL20RB	Interleukin-20 receptor subunit beta	
B4G0335	3.13	7.88	IRAK3	Interleukin-1 receptor-associated kinase 3	XM_017355295.3
B22G0509	3.13	5.69	CCDC71	Coiled-coil domain-containing protein 71	NM_001044710.1
B11G0757	3.13	1.64	LDB1	LIM domain-binding protein 1	NM_001083310.2
B21G0128	3.13	2.26	BNIP1	Vesicle transport protein SEC20	XM_001333689.9
B5G1306	3.13	0.88	TBX1	T-box transcription factor TBX1	XM_005168611.5
B21G0371	3.13	5.18	PCNX3	Pecanex-like protein 3	NM_001328187.1
A24G0074	3.13	3.50	FAM234A	Protein FAM234A	XM_021470412.2
A15G0397	3.13	3.33	PPM1D	Protein phosphatase 1D	NM_201090.2
A7G0942	3.12	5.34	RBM4.1	RNA-binding protein 4.1	NM_199705.1
B1G0148	3.12	2.15	ENDOD1	Endonuclease domain-containing 1 protein	XM_021475944.2
A9G0274	3.12	11.16	LAMP1	Lysosome-associated membrane glycoprotein 1	NM_001326532.1
B6G0280	3.12	6.32	PNKD	Probable hydrolase PNKD	NM_001256210.1
B18G0682	3.12	3.44	CHKA	Choline kinase alpha	NM_001100012.2
B2G0213	3.12	2.65	TRIM16	E3 ubiquitin-protein ligase TRIM16	NM_001365971.1

B23G0310	3.12	2.50	SLC17A9	Solute carrier family 17 member 9	NM_001002635.1
B16G0707	3.12	3.70	FLAD1	FAD synthase	XM_005169746.5
A10G0480	3.12	2.19	RPS6KB1	Ribosomal protein S6 kinase beta-1	XM_685634.9
A23G0040	3.12	3.84	FBXW12	WD repeat-containing protein 12	NM_001122628.2
A4G0793	3.12	2.59	NAF1	ACA ribonucleoprotein complex non-core subunit NAF1	XM_691652.7
A4G0672	3.12	22.26	ATP2B1	Plasma membrane calcium-transporting ATPase 1	NM_001044757.1
B7G0337	3.12	6.80	DOK1	Docking protein 1	NM_213088.1
B7G1075	3.12	3.17	ZNF16L	Zinc finger protein 16-like	NM_001423803.1
A24G0072	3.12	6.15	STUB1	E3 ubiquitin-protein ligase CHIP	NM_199674.2
B9G0385	3.12	3.26	TBC1D4	TBC1 domain family member 4	XM_002663395.6
A5G1193	3.12	5.72			
B13G0501	3.12	3.56			XM_005156926.5
A17G0784	3.12	0.60	ADGRF5	Adhesion G protein-coupled receptor F5	XM_021473051.2
B25G0412	3.12	6.03	TMEM60	Transmembrane protein 60	
B16G0435	3.12	6.21	SPHK2	Sphingosine kinase 2	XM_068214165.1
A3G0499	3.12	6.15	TNRC18	Trinucleotide repeat-containing gene 18 protein	XM_005164179.4
A5G0727	3.12	11.85	HSD17B12	Very-long-chain 3-oxoacyl-CoA reductase	NM_001386628.1
A12G0008	3.12	4.75	PARG	Poly(ADP-ribose) glycohydrolase	
A8G0350	3.12	5.57	SEC22BB	Vesicle-trafficking protein SEC22b-B	NM_001328526.1
A2G0239	3.12	2.97	PTER	Phosphotriesterase-related protein	NM_001045381.2
A22G0479	3.12	1.72	GNA11	Guanine nucleotide-binding protein subunit alpha-11	NM_001045036.2
B5G0583	3.12	3.65	RGMB	Repulsive guidance molecule B	NM_001001727.2
B8G0298	3.11	4.28	GOLGA1	Golgin subfamily A member 1	XM_005169080.5
A3G0742	3.11	14.32	LIMD2	LIM domain-containing protein 2	NM_001328061.1
A5G0504	3.11	7.69	EHMT1	Histone-lysine N-methyltransferase EHMT1	NM_001030131.2
B14G0116	3.11	4.20	B4GALT7	Beta-1,4-galactosyltransferase 7	XM_021481002.2
A2G0976	3.11	3.14	LIG1	DNA ligase 1	NM_001126388.1
B1G0284	3.11	7.23	FAT1	Protocadherin Fat 1	XM_021469232.2
B14G0097	3.11	2.28	THAP4	Peroxynitrite isomerase THAP4	
A17G0423	3.11	7.40	IARS2	Isoleucine--tRNA ligase, mitochondrial	NM_001163798.1
B3G0594	3.11	5.42	RNF216	E3 ubiquitin-protein ligase RNF216	XM_068219338.1
A20G0151	3.11	3.09	RAD51-A	DNA repair protein RAD51 homolog A	NM_213206.2
B4G0154	3.11	11.39	CCND2	S-specific cyclin-D2	XM_068219693.1
A17G0309	3.11	1.31	CINP	Cyclin-dependent kinase 2-interacting protein	
A24G0680	3.11	5.76	LANCL2	LanC-like protein 2	NM_001083003.2
A4G0546	3.11	6.02	PPP1R12A	Protein phosphatase 1 regulatory subunit 12A	NM_001003870.2
A4G0723	3.11	3.00	MDM1	Nuclear protein MDM1	XM_009300141.4
A4G0659	3.11	0.93	PKP2	Plakophilin-2	NM_001113433.1
A16G0112	3.11	3.37	ROHU_006075	A0A498N7I7_LABRO Uncharacterized protein	NM_001012368.2

B6G0324	3.11	5.81	LRRFIP2	Leucine-rich repeat flightless-interacting protein 2	XM_005168724.5
B24G0147	3.11	9.36	TMEM204	Transmembrane protein 204	NM_001163897.1
A12G0538	3.11	1.70	RPP30	Ribonuclease P protein subunit p30	NM_001013479.1
A7G0612	3.11	4.28	RHPN2	Rhophilin-2	NM_201178.1
B22G0648	3.11	6.36	ZBTB37	Zinc finger and BTB domain-containing protein 37	NM_001045160.1
B12G0663	3.11	17.73	ANK3	Ankyrin-3	XM_068224721.1
A1G0581	3.11	3.78	ARL13B	ADP-ribosylation factor-like protein 13B	XM_068221797.1
B13G0344	3.11	5.11	DENND10	DENN domain-containing protein 10	NM_001083819.2
B18G0097	3.11	4.48	ILVBL	2-hydroxyacyl-CoA lyase 2	XM_021467622.2
B5G0368	3.11	0.29	GNA14	Guanine nucleotide-binding protein subunit alpha-14	NM_001003753.1
B20G0148	3.11	4.25	ENPP1	phosphodiesterase family member 1	XM_005160441.5
A13G0212	3.11	16.94	DESI2	Deubiquitinase DESI2	XM_005156635.5
A16G0059	3.11	9.67	UBR5	E3 ubiquitin-protein ligase UBR5	XM_005158437.5
B24G0086	3.10	9.80	FAM234A	Protein FAM234A	XM_021470412.2
A11G0620	3.10	5.04	G5714_011616	A0A7J6CNA0_9TELE Uncharacterized protein	XM_005155890.5
B4G0177	3.10	3.98	PEX26	Peroxisome assembly protein 26	NM_199920.1
B17G0545	3.10	4.39	TRAF3	TNF receptor-associated factor 3	NM_001003513.1
B2G0849	3.10	3.17	HS2ST1	Heparan sulfate 2-O-sulfotransferase 1	NM_001080670.1
B15G0575	3.10	20.46	MYO1C	Unconventional myosin-Ic	NM_001317189.1
B8G0648	3.10	3.14	SPRYD4	SPRY domain-containing protein 4	
A8G0285	3.10	8.75	RNF11	RING finger protein 11	NM_001128408.1
A16G0939	3.10	0.83	MBOAT1	Lysophospholipid acyltransferase 1	NM_001007396.2
B13G0465	3.10	10.67	PKD2L1	Polycystic kidney disease 2-like 1 protein	NM_001145601.1
A12G0286	3.10	5.26	BMS1	Ribosome biogenesis protein BMS1 homolog	NM_001111150.2
A14G0493	3.10	2.94	TCERG1	Transcription elongation regulator 1	NM_198357.3
B11G0180	3.10	3.69	CTNS	Cystinosin	NM_001328585.1
A11G0670	3.10	1.21	DNAJC16	DnaJ homolog subfamily C member 16	NM_001077733.1
A3G0590	3.10	4.25	COG1	Conserved oligomeric Golgi complex subunit 1	XM_002661160.7
B5G0297	3.10	1.52	TEX55	Testis-specific expressed protein 55	
B3G1479	3.10	2.64	NLRC3	Protein NLRC3	XM_068220504.1
A10G0082	3.10	2.95	ANKRD34B	Ankyrin repeat domain-containing protein 34B	XM_684338.7
B19G0349	3.10	5.56	ATP9B	Probable phospholipid-transporting ATPase IIB	XM_005170057.5
B22G1077	3.10	14.14	EFEMP1	EGF-containing fibulin-like extracellular matrix protein 1	XM_009296503.4
A25G0255	3.10	8.14	TMEM263-B	Transmembrane protein 263-B	NM_001005996.2
B1G0503	3.10	5.33	IGF2BP2	Insulin-like growth factor 2 mRNA-binding protein 2	XM_005159949.5
B17G0383	3.10	3.51	SLC8A1	calcium exchanger 1	XM_009293025.4
B4G0178	3.10	10.13	USP18	Ubl carboxyl-terminal hydrolase 18	XM_005164532.5

A19G0439	3.10	2.89	NKAIN1	potassium-transporting ATPase subunit beta-1-interacting protein 1	NM_200545.1
B12G0521	3.10	6.40	PAPSS2	Bifunctional 3'-phosphoadenosine 5'-phosphosulfate synthase 2	NM_212562.1
B25G0806	3.10	7.16	HBP1	HMG box-containing protein 1	NM_001024431.2
B2G0169	3.10	8.10	WDR48	WD repeat-containing protein 48	NM_214709.1
B14G0506	3.10	2.04	SLC49A3	Solute carrier family 49 member A3	
B5G1178	3.10	2.64	RADX	RPA-related protein RADX	XM_021476453.2
B7G0851	3.09	8.12	CD99L2	CD99 antigen-like protein 2	XM_017356813.3
B5G1141	3.09	0.78	ARAP1	Arf-GAP with Rho-GAP domain, ANK repeat and PH domain-containing protein 1	XM_695389.9
B11G0735	3.09	3.35	MFN1	Mitofusin-1	XM_009305773.4
A1G0718	3.09	5.51	RASA3	Ras GTPase-activating protein 3	XM_009305229.4
A3G1235	3.09	4.76	G5714_002933	A0A7J6D968_9TELE Uncharacterized protein	NM_001386581.1
B19G0810	3.09	3.02	LOC113119217	A0A6P6RFT5_CARAU uncharacterized protein LOC113119217	XM_017352417.3
A1G0414	3.09	3.00	SI:DKEY-56E3.3	B8A4C5_DANRE Si:dkey-56e3.3	XM_005160080.5
A13G0313	3.09	6.77	SLC38A6	Probable sodium-coupled neutral amino acid transporter 6	XM_009307145.4
B16G1075	3.09	0.16	HA19	A9 alpha chain	
A7G1387	3.09	0.10	G5714_008746	A0A7J6CYD3_9TELE Uncharacterized protein	
B13G0525	3.09	7.12	UNC5B-B	Netrin receptor UNC5B-b	NM_001001840.3
A21G0130	3.09	0.32	G5714_020931	A0A7J6BVV8_9TELE Uncharacterized protein	
B21G0746	3.09	4.49	LMAN1	Protein ERGIC-53	NM_001386234.1
B4G0431	3.09	5.41	TMPO	Lamina-associated polypeptide 2, isoform beta	NM_001328565.1
A10G0398	3.09	1.20	HUNK-A	Hormonally up-regulated neu tumor-associated kinase homolog A	XM_005172726.5
B12G0623	3.09	2.02	TACO1	Translational activator of cytochrome c oxidase 1	NM_001316971.1
A8G0982	3.09	2.27	BRPF1	Peregrin	NM_201016.1
A11G0158	3.09	4.55	PTP4A1	Protein tyrosine phosphatase type IVA 1	NM_001030172.1
B21G0944	3.09	6.37	TCB1	Transposable element Tcb1 transposase	XM_021471168.2
A12G0378	3.09	2.55	MSH2	DNA mismatch repair protein Msh2	NM_213524.1
B7G1042	3.09	1.68	LOC113105537	A0A6P6PNV6_CARAU uncharacterized protein LOC113105537	NM_001423818.1
B6G1017	3.09	3.99	ZEB2	Zinc finger E-box-binding homeobox 2	
A4G0901	3.09	4.12	RAD51AP1	RAD51-associated protein 1	NM_001044338.2
B22G0394	3.09	8.59	LOC113076563	A0A6P6N7L9_CARAU uncharacterized protein LOC113076563	XM_009296056.4
A6G0613	3.09	0.69	SFT2D2	Vesicle transport protein SFT2B	NM_001013288.2
A12G0724	3.09	1.29	PLCE1	1-phosphatidylinositol 4,5-bisphosphate phosphodiesterase epsilon-1	XM_021480149.2
A19G0313	3.09	4.39	FAM91A1	Protein FAM91A1	NM_200441.1

A21G0648	3.09	0.52	A0A3P9MQR4	A0A3P9MQR4_ORYLA YqaJ domain-containing protein	
B6G0083	3.09	10.27	KDM4A	Lysine-specific demethylase 4A	XM_005165688.5
B14G0287	3.09	4.44	AFAP1L1	Actin filament-associated protein 1-like 1	XM_068213234.1
B21G0341	3.08	2.52	FAM53C	Protein FAM53C	NM_001111239.2
B8G0700	3.08	9.19	RORB	Nuclear receptor ROR-beta	NM_001082819.1
A24G0106	3.08	32.01	IGKV1-13	Immunoglobulin kappa variable 1-13	NM_001045408.1
A8G0881	3.08	3.47	QSPP	Queuosine salvage protein	NM_001083558.1
A15G0355	3.08	11.14	TRAFD1	TRAF-type zinc finger domain-containing protein 1	NM_001076647.2
A7G0724	3.08	10.39	TJP1	Tight junction protein ZO-1	XM_005168936.5
B24G0301	3.08	5.15	BCL2	Apoptosis regulator Bcl-2	NM_001030253.2
B24G0225	3.08	8.44	CDK5	Cyclin-dependent kinase 5	NM_131719.2
B25G0463	3.08	3.18	NEDD1	Protein NEDD1	XM_005163004.5
A19G0804	3.08	7.36	CIC	Protein capicua homolog	XM_003200533.6
B10G0598	3.08	1.06	VEZF1	Vascular endothelial zinc finger 1	XM_005169186.5
A9G0544	3.08	5.69	IFT88	Intraflagellar transport protein 88 homolog	NM_001001725.1
B20G0694	3.08	4.30	REPS1	RalBP1-associated Eps domain-containing protein 1	NM_001045056.2
A15G0709	3.08	5.72	TTMP	TPA-induced transmembrane protein	
B24G0515	3.08	6.96	BMI1A	Polycomb complex protein BMI-1-A	NM_194366.1
B8G0561	3.08	6.49	PIGR	Polymeric immunoglobulin receptor	
B9G0541	3.08	2.97	GTF2E1	General transcription factor IIE subunit 1	NM_001045145.1
B9G0905	3.08	6.20	RANBP2	E3 SUMO-protein ligase RanBP2	XM_002663280.7
A19G0375	3.08	2.88	RBM48	RNA-binding protein 48	NM_205604.2
B2G0243	3.08	1.46	CCL20	C-C motif chemokine 20	
B9G0852	3.08	7.65	GPR155	Integral membrane protein GPR155	XM_009304429.4
A3G0023	3.08	0.73	G5714_004074	A0A7J6DC39_9TELE Uncharacterized protein	
A18G0762	3.08	14.32	LOC113061399	A0A6P6LR52_CARAU uncharacterized protein LOC113061399	
B11G0657	3.08	1.47	WNT4A	Protein Wnt-4a	XM_068224230.1
A16G0960	3.08	1.71	PMVK	Phosphomevalonate kinase	NM_001083579.2
A16G0671	3.08	13.04	LOC113060219	A0A6P6LJY9_CARAU uncharacterized protein LOC113060219 isoform X1	NM_001330258.2
A4G0797	3.08	3.18	FBH1	F-box DNA helicase 1	XM_017355080.3
B7G1251	3.08	0.22	GIMAP7	GTPase IMAP family member 7	
B21G0732	3.07	3.30	BDP1	Transcription factor TFIIIB component B" homolog	NM_001430814.1
A25G0119	3.07	1.53	CPEB1-A	Cytoplasmic polyadenylation element-binding protein 1-A	NM_131725.1
B19G0604	3.07	14.80	RNF19B	E3 ubiquitin-protein ligase RNF19B	NM_001202440.1
A6G0251	3.07	2.91	CYTIP	Cytohesin-interacting protein	
B6G0797	3.07	3.58	LOC113096085	A0A6P6P7W6_CARAU uncharacterized protein LOC113096085	XM_001343386.8
B9G0394	3.07	6.50	GDAP2	Ganglioside-induced differentiation-associated protein 2	NM_001004563.1

A18G0715	3.07	9.06	FAM168A	Protein FAM168A	XM_005173612.5
A18G0407	3.07	22.18	CTCF	Transcriptional repressor CTCF	NM_001001844.1
A9G0216	3.07	8.13	PLCD4	1-phosphatidylinositol 4,5-bisphosphate phosphodiesterase delta-4	XM_684872.9
A8G0306	3.07	7.93	SKI	Ski oncogene	NM_130935.3
B10G0390	3.07	5.37	TRIM3	Tripartite motif-containing protein 3	NM_001430889.1
B7G0748	3.07	5.99	FBXO22	F-box only protein 22	NM_001030106.1
B10G0790	3.07	0.80	DDX55	ATP-dependent RNA helicase DDX55	NM_173229.2
A4G1040	3.07	6.17	TMEM181	Transmembrane protein 181	XM_005168513.5
B17G0797	3.07	8.20	LOC107656750	A0A671PHC4_9TELE Uncharacterized LOC107656750	XM_002665221.6
A23G0158	3.07	9.66	P4HTM	Transmembrane prolyl 4-hydroxylase	NM_001080691.1
B5G0693	3.07	2.95	P2RX5	P2X purinoceptor 5	NM_194413.2
A1G0642	3.07	1.52	CARS2	Probable cysteine--tRNA ligase, mitochondrial	XM_677640.10
A25G0246	3.07	5.14	ZNF319	Zinc finger protein 319	XM_005174354.5
B20G0863	3.07	4.30	HMBOX1	Homeobox-containing protein 1	XM_005173819.5
B21G0756	3.07	4.00	A0A671XQM7	A0A671XQM7_SPAAU Uncharacterized protein	
A18G0540	3.07	12.70	BHLHE41	Class E basic helix-loop-helix protein 41	NM_001039107.1
B13G0488	3.07	2.43	CCDC85A	Coiled-coil domain-containing protein 85A	NM_001123301.2
A13G0827	3.07	2.22	G5714_013102	A0A7J6CDZ5_9TELE Uncharacterized protein	XM_692467.8
B21G0754	3.07	0.87	SDAD1	Protein SDA1 homolog	NM_173230.1
A25G0412	3.07	2.87	G5714_024243	A0A7J6BLQ6_9TELE Uncharacterized protein	
A5G0199	3.07	5.56	CNNM4	Metal transporter CNNM4	XM_021476410.2
B23G0942	3.07	20.01	LOC113041378	A0A6P6JCU9_CARAU NACHT, LRR and PYD domains-containing protein 12-like isoform X2	
A6G0793	3.07	11.60	GXYLT2	Glucoside xylosyltransferase 2	NM_001110200.1
A2G0013	3.07	0.30	CLDN1	Claudin-1	
A24G0541	3.07	0.13	CHMP4C	Charged multivesicular body protein 4c	NM_001017844.1
B7G0434	3.06	4.47	CPT1A	Carnitine O-palmitoyltransferase 1, liver isoform	NM_001044854.1
A16G0878	3.06	1.38	GIMAP5	GTPase IMAP family member 5	
B17G0873	3.06	4.30	PXDN	Peroxidasin homolog	XM_009293329.4
B7G0522	3.06	11.37	INSIG1	Insulin-induced gene 1 protein	NM_199869.1
B16G0897	3.06	3.81	CPVL	Probable serine carboxypeptidase CPVL	NM_199278.1
A8G0076	3.06	0.15	DLC-1	Dynein light chain 1, cytoplasmic	
A8G0920	3.06	4.51	PEX10	Peroxisome biogenesis factor 10	NM_001005994.1
B3G0211	3.06	9.90	ZC3H7B	Zinc finger CCCH domain-containing protein 7B	XM_009299075.4
B25G0231	3.06	3.43	USP6NL	USP6 N-terminal-like protein	XM_021473875.2
A3G0324	3.06	5.31	SAMD1	Sterile alpha motif domain-containing protein 1	NM_001316989.1
B12G0738	3.06	8.43	WDR45B	WD repeat domain phosphoinositide-interacting protein 3	NM_200240.2
A24G0490	3.06	10.80	TRAM1L1	Translocating chain-associated membrane protein 1-like 1	NM_153669.2

A19G0427	3.06	2.24	CRTAP	Cartilage-associated protein	NM_001001406.1
B7G0941	3.06	31.21	PYGM	Glycogen phosphorylase, muscle form	NM_001328031.1
B22G0790	3.06	5.58	TLE4	Transducin-like enhancer protein 4	XM_021473487.2
A15G0483	3.06	1.82	OAF	Out at first protein homolog	XM_695363.10
B25G0212	3.06	3.22	MLKL	Mixed lineage kinase domain-like protein	
A21G0125	3.06	0.19	DPX16_22892	A0A3N0Y080_ANAGA Lymphocyte antigen 6E	
A9G0812	3.06	0.37	DLX2A	Homeobox protein Dlx2a	NM_131311.2
B8G0959	3.06	14.00	CLDN5	Claudin-5	NM_213274.1
A18G0580	3.06	3.51	SPATA2L	Spermatogenesis-associated protein 2-like protein	NM_001386847.1
B15G0504	3.06	14.61	MYO18A	Unconventional myosin-XVIIIa	XM_068213741.1
B5G0669	3.06	2.97	HSPB11	Intraflagellar transport protein 25 homolog	NM_001080065.1
B1G0382	3.06	3.04	RFC1	Replication factor C subunit 1	NM_001089491.2
B10G0429	3.06	9.28	RPS6KB1	Ribosomal protein S6 kinase beta-1	XM_685634.9
B10G0130	3.06	8.48	PTBP3	Polypyrimidine tract-binding protein 3	XM_009305172.4
A2G0275	3.06	2.84	MIER1	Mesoderm induction early response protein 1	NM_001291343.1
A17G0433	3.06	6.87	LCK	Tyrosine-protein kinase Lck	NM_001040328.1
A2G0373	3.06	3.29	GK5	Putative glycerol kinase 5	NM_001077803.3
B13G0645	3.06	2.37	SPTLC3	Serine palmitoyltransferase 3	NM_001243309.1
B18G0090	3.06	4.33	LARGE2	Xylosyl- and glucuronyltransferase LARGE2s	XM_017351916.3
A6G0556	3.05	1.78	ITGB3BP	Centromere protein R	
A3G0486	3.05	6.88	CARD11	Caspase recruitment domain-containing protein 11	XM_068219349.1
B14G0727	3.05	1.51	UVSSA	UV-stimulated scaffold protein A	
A6G0949	3.05	4.52	NLRP3	NACHT, LRR and PYD domains-containing protein 3	XM_021470830.2
A21G0416	3.05	2.80	POLD3	DNA polymerase delta subunit 3	NM_001042769.2
B2G0490	3.05	2.05	TRAV3	T cell receptor alpha variable 3	
B15G0040	3.05	3.18	NUDT8	Mitochondrial coenzyme A diphosphatase NUDT8	NM_001077316.2
A21G0641	3.05	1.20	ALOX8	Polyunsaturated fatty acid lipoxygenase ALOX8	NM_001076710.2
A4G0954	3.05	2.27	AEBP2	Zinc finger protein AEBP2	NM_213494.2
A15G0052	3.05	2.47	ILKAP	threonine phosphatase 2C	NM_001089504.2
A17G0252	3.05	5.41	ADAM17	Disintegrin and metalloproteinase domain-containing protein 17	NM_199673.1
B19G0277	3.05	7.49	LOC113120162	A0A6P6RLA1_CARAU uncharacterized protein LOC113120162	XM_005159403.5
B14G0305	3.05	9.18	SSH3	Protein phosphatase Slingshot homolog 3	XM_021474332.2
B9G0316	3.05	3.83	KDM6A	Lysine-specific demethylase 6A	XM_005167794.5
B18G0554	3.05	1.73	CHTF8	Chromosome transmission fidelity protein 8 homolog	NM_001044336.3
A3G0457	3.05	1.57	MFSD11	UNC93-like protein MFSD11	XM_001339605.8
A17G0141	3.05	7.29	ITPK1	Inositol-tetrakisphosphate 1-kinase	XM_001922333.5
A11G0144	3.05	2.72	LSM4	U6 snRNA-associated Sm-like protein LSm4	NM_001007338.1
A23G0239	3.05	1.61	RGS19	Regulator of G-protein signaling 19	XM_021469715.2

A14G0713	3.05	4.65	UVSSA	UV-stimulated scaffold protein A	NM_001346152.1
A7G1117	3.05	3.91	LRRC28	Leucine-rich repeat-containing protein 28	XM_068218359.1
B17G0565	3.05	2.68	ALKAL2B	ALK and LTK ligand 2b	NM_001424080.1
A16G0123	3.05	4.74	MIOS	GATOR complex protein MIOS	NM_201333.1
B7G0767	3.05	7.44	HERC1	Probable E3 ubiquitin-protein ligase HERC1	XM_009303239.3
B22G0486	3.05	1.53	NLRC3	Protein NLRC3	XM_021479924.2
B6G1006	3.05	6.51	ARFRP1	ADP-ribosylation factor-related protein 1	XM_001339340.9
A5G0656	3.05	4.20	EFNB1	Ephrin-B1	NM_131805.1
A7G0983	3.05	5.69	DNAJC3	DnaJ homolog subfamily C member 3	NM_131630.2
A5G1273	3.05	3.78	DNAJC25	DnaJ homolog subfamily C member 25	NM_001205027.1
B23G0730	3.05	8.25	CSAD	Cysteine sulfinic acid decarboxylase	NM_001007348.2
B3G0149	3.04	1.78	LOC113096980	A0A6P6PBC3_CARAU zinc finger protein 271-like isoform X1	NM_001030094.3
A4G0322	3.04	5.43	STXB	Stonustoxin subunit beta	XM_021475476.2
B3G0339	3.04	3.39	TSPAN1	Tetraspanin-1	NM_200408.2
B3G0111	3.04	2.12	LOC113093308	A0A6P6P2F6_CARAU gastrula zinc finger protein XlCGF26.1-like	
B3G0447	3.04	36.98	ADGRE5	Adhesion G protein-coupled receptor E5	XM_068218136.1
A23G0116	3.04	3.81	ANKS1A	Ankyrin repeat and SAM domain-containing protein 1A	XM_021470076.2
A10G0290	3.04	22.70	ACAT1	Acetyl-CoA acetyltransferase, mitochondrial	NM_001003746.1
B7G0131	3.04	4.09	CTDNEP1A	CTD nuclear envelope phosphatase 1A	NM_001007440.2
A4G0068	3.04	1.89	LOC113047446	A0A6P6JXS5_CARAU gastrula zinc finger protein XlCGF57.1-like isoform X1	XM_068220573.1
B9G0076	3.04	0.30	NF7O	Nuclear factor 7, ovary	XM_068219023.1
B2G0045	3.04	13.51	TCF3	Transcription factor E2-alpha	NM_131094.1
B2G0146	3.04	6.53	TRIM25	ISG15 ligase TRIM25	NM_001039719.2
B24G0533	3.04	1.92	TIMM21	Mitochondrial import inner membrane translocase subunit Tim21	NM_001386503.1
A1G0379	3.04	5.33	EIF5B	Eukaryotic translation initiation factor 5B	XM_009293741.4
A17G0006	3.04	2.85	EXD1	piRNA biogenesis protein EXD1	
A17G0076	3.04	3.84	PRKD3	threonine-protein kinase D3	XM_680391.9
B8G0908	3.04	5.49	MITF	Microphthalmia-associated transcription factor	NM_131848.2
A23G0586	3.04	4.22	PHIP	PH-interacting protein	XM_009297004.4
A1G0798	3.04	0.09	GTF3C3	General transcription factor 3C polypeptide 3	NM_001256242.1
A19G0752	3.04	2.60	SMIM13	Small integral membrane protein 13	NM_001135579.2
B9G0214	3.04	16.33	GLS	Glutaminase kidney isoform, mitochondrial	XM_688079.10
A24G0165	3.04	3.87	ESCO1	N-acetyltransferase ESCO1	XM_005174190.5
A15G0160	3.04	4.19	MSH5	MutS protein homolog 5	XM_005157792.5
B21G0467	3.04	9.01	SC5D	Lathosterol oxidase	NM_001004630.1
A9G0457	3.04	5.03	UBE2E3	Ubiquitin-conjugating enzyme E2 E3	

A1G0686	3.04	2.12	ILDR1	Immunoglobulin-like domain-containing receptor 1	
B4G0855	3.04	1.42	LOC113080876	A0A6P6NHV3_CARAU gastrula zinc finger protein XlCGF26.1-like isoform X1	XM_017355333.3
A21G0018	3.04	2.05	JMY	Junction-mediating and -regulatory protein	NM_213272.1
B17G0199	3.04	3.22	DACT1	Dapper homolog 1	NM_214731.3
B22G0573	3.04	13.40	R3HDM1	R3H domain-containing protein 1	XM_021469576.2
B19G0925	3.04	3.32	INTS8	Integrator complex subunit 8	NM_001020756.2
B25G0789	3.03	3.28	TMEM138	Transmembrane protein 138	NM_001423846.1
B1G1022	3.03	7.13	SLC25A10	Mitochondrial dicarboxylate carrier	NM_201172.1
A5G0104	3.03	1.56	HBEGF	Proheparin-binding EGF-like growth factor	XM_009301165.4
A9G0611	3.03	2.72	BMPR2	Bone morphogenetic protein receptor type-2	NM_001039807.1
B8G0345	3.03	6.77	DOK2	Docking protein 2	XM_001341478.9
B17G0889	3.03	16.62	FERMT2	Fermitin family homolog 2	XM_005158915.5
B24G0459	3.03	8.19	ATP6V1A	V-type proton ATPase catalytic subunit A	XM_021470316.2
B19G0661	3.03	3.72	ATXN1	Ataxin-1	NM_001044826.1
B9G0453	3.03	2.90	DGKB	Diacylglycerol kinase beta	XM_021472166.2
A11G0275	3.03	6.63	OPSD1	Rhodopsin, deep-sea form	XM_005155724.5
A21G0174	3.03	1.65	F2R	Proteinase-activated receptor 1	XM_001339441.9
A14G0198	3.03	1.60	HS6ST2	Heparan-sulfate 6-O-sulfotransferase 2	NM_194421.2
B7G0901	3.03	4.18	SVEP1	Sushi, von Willebrand factor type A, EGF and pentraxin domain-containing protein 1	XM_009303134.4
A13G0518	3.03	5.06	PBLD	Phenazine biosynthesis-like domain-containing protein	NM_200566.1
A1G0017	3.03	3.90	TXNDC11	Thioredoxin domain-containing protein 11	NM_001326705.1
B17G0633	3.03	5.92	DNAL1	Dynein axonemal light chain 1	NM_001003442.1
B1G0613	3.03	10.63	TMEM134	Transmembrane protein 134	NM_200369.1
B22G0091	3.03	32.13	NLRC3	Protein NLRC3	XM_068216562.1
B23G0863	3.03	3.77	G5714_022276	A0A7J6BMP8_9TELE Uncharacterized protein	
B3G0331	3.03	3.96	A0A672QFP3	A0A672QFP3_SINGR Si:dkey-282h22.5	NM_001328134.1
B13G0051	3.03	8.67	FBXO28	F-box only protein 28	XM_005173033.5
B9G0510	3.03	2.61	FTCDNL1	Formiminotransferase N-terminal subdomain-containing protein	NM_001076645.1
B10G0165	3.03	5.62	FSTA	Follistatin-A	NM_001039631.1
A6G0671	3.03	4.82	SMARCC2	SNF complex subunit SMARCC2	XM_690772.8
B23G0824	3.03	4.15	CYP2B15	Cytochrome P450 2B15	NM_001025557.2
A7G1111	3.03	2.93	IMMP1L	Mitochondrial inner membrane protease subunit 1	NM_001386390.1
A10G0091	3.03	6.78	BRAP	BRCA1-associated protein	XM_021479154.2
B1G0621	3.03	4.54	G5714_000631	A0A7J6DH28_9TELE SET domain-containing protein	XM_021476794.2
A13G0563	3.03	1.44			NM_001083819.2
B19G0339	3.03	3.78	NUDCD1	NudC domain-containing protein 1	NM_001020627.2
B8G0346	3.03	6.15	KAZALD1	Kazal-type serine protease inhibitor domain-containing protein 1	NM_001123319.1
B6G0619	3.03	4.98	NIT2	Omega-amidase NIT2	NM_205611.3

A20G0889	3.03	3.13	LEG1A	Protein leg1a	
A12G0662	3.02	7.12	MKI67	Proliferation marker protein Ki-67	NM_001277446.2
B14G0388	3.02	3.48	SFXN5	Sideroflexin-5	NM_001030163.3
A18G0747	3.02	0.00	PIERCE2	Piercer of microtubule wall 2 protein	
A9G0179	3.02	31.85	STAT1	Signal transducer and activator of transcription 1	NM_200091.2
B1G0112	3.02	9.61	UBE2F	NEDD8-conjugating enzyme UBE2F	NM_001423799.1
A24G0183	3.02	8.71	RB1CC1	RB1-inducible coiled-coil protein 1	XM_009302198.4
A3G0715	3.02	23.06	AP2A1	AP-2 complex subunit alpha-1	XM_068219093.1
B14G0494	3.02	2.35	NOA1	Nitric oxide-associated protein 1	
B3G1534	3.02	0.53			
A10G0207	3.02	3.16	ORAI1	Calcium release-activated calcium channel protein 1	NM_205600.1
B8G0071	3.02	5.64	OGG1	DNA lyase	NM_001123308.2
A2G0653	3.02	12.98	RANBP9	Ran-binding protein 9	NM_001080571.2
B6G0251	3.02	2.39	LOC107584823	A0A672NMM8_SINGR Sentrin-specific protease 2-like	
B25G0368	3.02	1.51	TMEM17B	Transmembrane protein 17B	NM_001098391.1
A17G0325	3.02	14.48	EML1	Echinoderm microtubule-associated protein-like 1	NM_001193598.1
A16G0980	3.02	2.83	HACE1	E3 ubiquitin-protein ligase HACE1	NM_001423976.1
A22G0191	3.02	4.87	LOC113092167	A0A6P6NYL3_CARAU Envelope glycoprotein E	
A22G0243	3.02	3.01	ENC1	Ectoderm-neural cortex protein 1	NM_001001402.2
A5G0945	3.02	2.82	SH2D3C	SH2 domain-containing protein 3C	XM_021476041.2
B19G0918	3.02	6.72	TRIM16	Tripartite motif-containing protein 16	NM_001430853.1
A1G0444	3.02	3.93	LOC107674214	A0A671S8N8_9TELE Uncharacterized LOC107674214	XM_021476923.2
B7G1248	3.02	2.35	VANGL2	Vang-like protein 2	NM_153674.2
B2G0916	3.02	4.34	COL11A1	Collagen alpha-1(XI) chain	NM_001184954.1
B10G0272	3.02	4.00	SLK	threonine-protein kinase	XM_021472247.2
A23G0324	3.02	7.61	TASOR	Protein TASOR	XM_001922115.7
B6G0322	3.02	1.88	ST6GAL2	Beta-galactoside alpha-2,6-sialyltransferase 2	NM_001171221.1
B5G1013	3.02	7.36	TRAF2	TNF receptor-associated factor 2	XM_021471606.2
B8G0445	3.02	4.37	PRF1	Perforin-1	NM_001123246.1
B11G0741	3.02	11.67	IRF2BP2B	Interferon regulatory factor 2-binding protein 2-B	XM_005172805.5
B16G0568	3.02	7.03	NR4A3	Nuclear receptor subfamily 4 group A member 3	XM_009292332.4
B4G0170	3.02	4.77			
A7G0557	3.02	1.32	ESYT2	Extended synaptotagmin-2	XM_002662855.6
B5G0059	3.02	5.07	MTMR4	Myotubularin-related protein 4	XM_068221350.1
A8G0561	3.02	3.25	DDX20	Probable ATP-dependent RNA helicase DDX20	XM_068222967.1
A22G0458	3.02	1.38	PRAG1	Inactive tyrosine-protein kinase PRAG1	
B1G0111	3.01	7.66	DDB_G0268948	Putative methyltransferase DDB_G0268948	NM_001145627.1
B14G0672	3.01	4.37	KDM2A	Lysine-specific demethylase 2A	XM_005173143.5
A5G0464	3.01	3.52	TM2D2	TM2 domain-containing protein 2	NM_001002640.1
A14G0526	3.01	22.62	CTSF	Cathepsin F	NM_001077568.1

A2G0775	3.01	6.40 RTASE	Probable RNA-directed DNA polymerase from transposon BS	XM_068218007.1
A19G0754	3.01	1.32 TRIM39	E3 ubiquitin-protein ligase TRIM39	NM_001386812.1
B3G1393	3.01	6.36		XM_003198185.6
B23G0470	3.01	4.40 NCKAP5L	Nck-associated protein 5-like	XM_021473692.2
A13G0036	3.01	5.66 HERC4	Probable E3 ubiquitin-protein ligase HERC4	NM_205651.1
B15G0633	3.01	3.63 CUEDC1	CUE domain-containing protein 1	XM_068213558.1
B5G0547	3.01	3.72 CAMSAP1B	Calmodulin-regulated spectrin-associated protein 1-B	XM_005168648.5
B10G0753	3.01	5.91 ZMIZ2	Zinc finger MIZ domain-containing protein 2	XM_068224040.1
B10G0321	3.01	4.45 ARR3	Arrestin red cell isoform 3	XM_009305269.4
B5G0674	3.01	4.06 WDR54	WD repeat-containing protein 54	NM_001003630.2
A9G0677	3.01	3.00 ANKRD10	Ankyrin repeat domain-containing protein 10	NM_001077377.1
B20G0609	3.01	0.32 LOC113120845	A0A6P6RM12_CARAU uncharacterized protein LOC113120845	
A19G0281	3.01	3.58 ZBTB10	Zinc finger and BTB domain-containing protein 10	NM_001128539.1
A20G0668	3.01	11.81 CDH2	Cadherin-2	NM_131081.2
B13G0191	3.01	3.97 P2RX7	P2X purinoceptor 7	XM_068213089.1
A14G0666	3.01	7.22 KDM3B	Lysine-specific demethylase 3B	XM_068213330.1
A13G0835	3.01	8.34 MLIP	Muscular LMNA-interacting protein	XM_682645.10
B7G0152	3.01	3.30 GCSH	Glycine cleavage system H protein, mitochondrial	NM_001002579.1
A7G1330	3.01	0.27 LOC113114442	A0A6P6QUK4_CARAU uncharacterized protein LOC113114442	
B25G0141	3.01	3.72 IGKV1D-8	Immunoglobulin kappa variable 1D-8	NM_001045408.1
A2G0415	3.01	5.89 RXRGA	Retinoic acid receptor RXR-gamma-A	NM_131217.4
A11G0707	3.01	1.51 ADGRL2	Adhesion G protein-coupled receptor L2	NM_001328553.2
B25G0580	3.01	3.05 NAV2	Neuron navigator 2	XM_068219403.1
A16G0146	3.00	1.31 RPZ	Protein rapunzel	
B5G1219	3.00	2.21 RDH11	Retinol dehydrogenase 11	
A5G1272	3.00	8.09 RGS3	Regulator of G-protein signaling 3	NM_001430835.1
B3G1086	3.00	3.83 A0A673M2R6	A0A673M2R6_9TELE Uncharacterized protein	
B23G0735	3.00	5.40 PIP4K2C	Phosphatidylinositol 5-phosphate 4-kinase type-2 gamma	NM_200101.3
A1G0393	3.00	2.38 G5714_000513	A0A7J6DGJ9_9TELE Uncharacterized protein	
B20G0361	3.00	9.36 DCUN1D4	DCN1-like protein 4	NM_001032366.3
B7G1303	3.00	1.65 MRC2	C-type mannose receptor 2	
A14G0166	3.00	3.23 SLC7A2	Cationic amino acid transporter 2	XM_068213200.1
B11G0591	3.00	1.56 ITPR1	Inositol 1,4,5-trisphosphate receptor type 1	NM_200531.2
B10G0449	3.00	6.29 IFT46	Intraflagellar transport protein 46 homolog	NM_001346250.1
A8G0529	3.00	3.30 DALRD3	DALR anticodon-binding domain-containing protein 3	NM_001123326.1

B11G0361	3.00	11.13	IKBKE	Inhibitor of nuclear factor kappa-B kinase subunit epsilon	NM_131569.2
A7G1210	3.00	7.63	CLCN3	Cl(-) exchange transporter 3	XM_005166345.5
A1G1037	3.00	2.68	TMEM144	Transmembrane protein 144	NM_001003999.2
B2G1239	3.00	4.80	GPAA1	Glycosylphosphatidylinositol anchor attachment 1 protein	XM_678021.10
B1G0493	3.00	5.66	ZC3H13	Zinc finger CCCH domain-containing protein 13	NM_200522.2
A23G0363	3.00	20.07	UBA1	Ubiquitin-like modifier-activating enzyme 1	XM_005162052.5
B24G0526	3.00	3.80	KLHL15	Kelch-like protein 15	NM_001003580.2
B20G0061	3.00	3.49	RNGTT	mRNA-capping enzyme	NM_212867.1
B7G0032	3.00	1.69	PYCARD	Apoptosis-associated speck-like protein containing a CARD	
A7G0585	3.00	4.43	DGKZ	Diacylglycerol kinase zeta	XM_005169034.5
B19G0731	3.00	3.50	RGS1	Regulator of G-protein signaling 1	
B3G0653	3.00	5.79	MYO10	Unconventional myosin-X	XM_694923.9
A3G1007	3.00	12.10	NR1D1	Nuclear receptor subfamily 1 group D member 1	NM_205729.2
A19G0600	3.00	3.93	KV12	Ig kappa chain V region 3368	
A22G0688	3.00	5.00	PPP1R2	Protein phosphatase inhibitor 2	NM_205668.1
B9G0765	3.00	7.14	DGKH	Diacylglycerol kinase eta	XM_068223576.1
A16G1003	3.00	1.12	CDCP1	CUB domain-containing protein 1	NM_001014333.1
B25G0162	2.99	16.20	IGKV3D-7	Immunoglobulin kappa variable 3D-7	NM_001045408.1
A1G0625	2.99	6.83	VPS8	Vacuolar protein sorting-associated protein 8 homolog	NM_001080616.2
B3G0363	2.99	3.89	PTGER1	Prostaglandin E2 receptor EP1 subtype	
A23G0262	2.99	8.11	FKBP1A	Peptidyl-prolyl cis-trans isomerase FKBP1A	NM_001005594.1
B16G0492	2.99	3.61	MEGF10	Multiple epidermal growth factor-like domains protein 10	XM_695441.8
A1G0238	2.99	0.01	LYSC	Lysozyme C	XM_017354378.3
B25G0536	2.99	0.62	TIGARA	Probable fructose-2,6-bisphosphatase TIGAR A	
A20G0007	2.99	8.79	PLCB4	1-phosphatidylinositol 4,5-bisphosphate phosphodiesterase beta-4	XM_068217787.1
B17G0478	2.99	5.72	IARS2	Isoleucine--tRNA ligase, mitochondrial	NM_001163798.1
A5G0292	2.99	5.14	SART3	Squamous cell carcinoma antigen recognized by T-cells 3	XM_068221218.1
B11G0023	2.99	4.35	ATP2B2	Plasma membrane calcium-transporting ATPase 2	XM_009306333.3
B14G0777	2.99	3.33	POL	RNA-directed DNA polymerase from mobile element jockey	XR_011006276.1
A15G0452	2.99	1.57	CAD96CA	Tyrosine kinase receptor Cad96Ca	XM_688510.8
B22G0049	2.99	2.58	A0A673JZZ6	A0A673JZZ6_9TELE Uncharacterized protein	XM_068217865.1
A11G0561	2.99	4.14	LIN1	LINE-1 reverse transcriptase homolog	
A22G0502	2.99	3.63	DENND1B	DENN domain-containing protein 1B	XM_009296375.4
B1G0439	2.99	4.48	G3BP2	Ras GTPase-activating protein-binding protein 2	NM_001080563.1
B21G0734	2.99	1.48	ARID5A	AT-rich interactive domain-containing protein 5A	NM_001080201.1

A17G0285	2.99	7.49	SMYD2A	N-lysine methyltransferase SMYD2-A	NM_001013550.1
B25G0404	2.99	24.75	CKMT1A	Creatine kinase U-type, mitochondrial	NM_198801.1
B7G1298	2.99	1.24	RTASE	Probable RNA-directed DNA polymerase from transposon BS	
B17G0670	2.99	10.96	CRIP2	Cysteine-rich protein 2	NM_213497.2
B15G0534	2.99	4.83	PAK4	threonine-protein kinase PAK 4	NM_001002222.2
B18G0199	2.99	4.22	MAF	Transcription factor Maf	NM_131844.2
A10G0620	2.99	4.27	NSD3	Histone-lysine N-methyltransferase NSD3	XM_021479326.2
A18G0491	2.98	6.29	N4BP1	NEDD4-binding protein 1	NM_199787.2
B4G0064	2.98	4.11	TM7SF3	Transmembrane 7 superfamily member 3	XR_011011406.1
A2G0221	2.98	11.18	USP33	Ubiquitin carboxyl-terminal hydrolase 33	NM_213227.1
A16G0508	2.98	13.14	STEAP4	Metalloreductase STEAP4	NM_001017734.2
B15G0322	2.98	4.45	TADA2A	Transcriptional adapter 2-alpha	XM_005157436.5
A1G0861	2.98	2.14	PMM2	Phosphomannomutase 2	NM_200084.1
A16G0790	2.98	3.41	UPP1	Uridine phosphorylase 1	XM_005157923.5
A4G0342	2.98	3.85	LOC104949296	A0A6I9NHJ8_9TELE uncharacterized protein LOC104949296 isoform X6	
A3G1201	2.98	17.47	NLRC3	Protein NLRC3	XM_021470978.2
B20G0767	2.98	4.03	ATAD2B	ATPase family AAA domain-containing protein 2B	NM_001365967.1
A21G0156	2.98	2.83	MRPS2	28S ribosomal protein S2, mitochondrial	NM_001080693.1
B2G0795	2.98	12.58	PLVAP	Plasmalemma vesicle-associated protein	NM_001114577.1
A12G0236	2.98	4.74	HLF	Hepatic leukemia factor	NM_001197059.1
B10G0202	2.98	47.04	PSAP	Prosaposin	XM_001334062.8
A7G0914	2.98	2.17	AJUBA	LIM domain-containing protein ajuba	XM_696274.10
B7G0147	2.98	4.21	DHX38	Pre-mRNA-splicing factor ATP-dependent RNA helicase PRP16	NM_200876.1
A10G0831	2.98	1.87	ENOPH1	Enolase-phosphatase E1	NM_001002226.1
B5G0213	2.98	12.72	FAM78A	Protein FAM78A	NM_001114475.2
B8G0054	2.98	2.58	DCP1A	mRNA-decapping enzyme 1A	NM_182893.2
B8G0410	2.98	5.07	SNRNP200	U5 small nuclear ribonucleoprotein 200 kDa helicase	NM_001123257.1
B21G0249	2.98	1.56	X-ELEMENT\ORF2	Probable RNA-directed DNA polymerase from transposon X-element	
B4G0339	2.98	7.55	LEMD3	Inner nuclear membrane protein Man1	NM_001044864.1
A22G0656	2.98	4.81	LOC113065145	A0A6P6M8D0_CARAU Fc receptor-like protein 5 isoform X2	
B22G1046	2.98	2.11	SOX2	Transcription factor Sox-2	NM_213118.1
A16G0550	2.98	5.66			XM_005158031.5
A6G0751	2.98	2.67	RAD18	E3 ubiquitin-protein ligase RAD18	NM_200137.1
A6G0095	2.98	3.44	DYNC1I2A	Dynein, cytoplasmic 1, intermediate chain 2a	XM_009302110.4
A23G0342	2.98	3.52	CCNQ	Cyclin-Q	NM_001020623.1
B4G0563	2.98	1.92	USP6NL	USP6 N-terminal-like protein	XM_005164878.5
A10G0448	2.98	2.59	NTM	Neurotrimin	XM_021479200.2
B24G0238	2.98	7.21	IGKV4-1	Immunoglobulin kappa variable 4-1	
A3G0253	2.98	15.96	NFIX	Nuclear factor 1 X-type	XM_068217607.1

A13G0190	2.98	3.59	SYNE2	Nesprin-2	NM_001365219.1
A19G0507	2.97	2.60	CLSPN	Claspin	XM_068215340.1
A25G0347	2.97	1.42	FAM185A	Protein FAM185A	XM_002666837.6
A16G0183	2.97	5.44	ARHGAP32	Rho GTPase-activating protein 32	XM_068214245.1
B1G1120	2.97	0.00	TC1A	Transposable element Tc1 transposase	
B12G0174	2.97	3.39	BICRAL	BRD4-interacting chromatin-remodeling complex-associated protein-like	XM_005156379.5
B2G0825	2.97	0.00	TC1A	Transposable element Tc1 transposase	
B6G0746	2.97	3.05	ENTPD8	Ectonucleoside triphosphate diphosphohydrolase 8	NM_001002379.2
B24G0613	2.97	4.28	ACAD11	Acyl-CoA dehydrogenase family member 11	NM_200178.1
A4G0286	2.97	0.00	TC1A	Transposable element Tc1 transposase	
B25G0449	2.97	0.74	CBLL1	E3 ubiquitin-protein ligase Hakai	XM_005163040.5
B18G0674	2.97	2.94	TMEM53	Transmembrane protein 53	NM_001201568.1
A2G1079	2.97	3.55	EXOC3	Exocyst complex component 3	NM_212715.1
A9G0795	2.97	8.57	SCRN3	Secernin-3	XM_009304428.4
A19G0103	2.97	0.00	TC1A	Transposable element Tc1 transposase	
B5G0436	2.97	5.96	ARHGEF12	Rho guanine nucleotide exchange factor 12	XM_009301886.4
A18G0007	2.97	6.58	PPFIA1	Liprin-alpha-1	NM_001111244.1
B13G0386	2.97	6.75	NFKB2	Nuclear factor NF-kappa-B p100 subunit	XM_682373.10
A2G0595	2.97	2.82	KIAA1217	Sickle tail protein homolog	XM_068214043.1
B18G0131	2.97	6.46	BCL7BB	lymphoma 7 protein family member B-B	NM_001006018.1
B13G0738	2.97	1.75	HRP65	Hrp65 protein	NM_001177461.2
A19G0809	2.97	0.00	TC1A	Transposable element Tc1 transposase	
A14G0583	2.97	6.86	DHRS13	reductase SDR family member 13	NM_205648.1
A5G0640	2.97	7.52	FCHO2	F-BAR domain only protein 2	NM_001020781.2
A21G0557	2.97	15.98	PC	Pyruvate carboxylase, mitochondrial	NM_001328356.1
B8G0597	2.97	0.75	PTK6	Protein-tyrosine kinase 6	XM_677686.9
A8G0596	2.97	1.02	FANCC	Fanconi anemia group C protein	XM_005167179.3
B20G0096	2.97	2.38	AKT1	threonine-protein kinase	
B9G0154	2.97	0.00	TC1A	Transposable element Tc1 transposase	
A1G0631	2.97	3.53	ALG11	GDP-Man:Man(3)GlcNAc(2)-PP-Dol alpha-1,2-mannosyltransferase	NM_001161351.1
B25G0887	2.96	1.72	MX2	Interferon-induced GTP-binding protein Mx2	XM_009297890.3
B1G0145	2.96	3.42	LOC107564628	A0A672LMW4_SINGR Uncharacterized LOC107564628	NM_001013300.2
A14G0032	2.96	6.25	TRIM16	Tripartite motif-containing protein 16	XM_681071.8
A22G0008	2.96	8.54	NCKIPSD	NCK-interacting protein with SH3 domain	NM_001423277.1
A8G0437	2.96	5.38	YTHDF1	YTH domain-containing family protein 1	NM_212713.1
B8G0179	2.96	2.26	POLR3D	DNA-directed RNA polymerase III subunit RPC4	NM_200287.1

B1G0357	2.96	1.66	ZCCHC7	Zinc finger CCHC domain-containing protein 7	
B6G0891	2.96	4.18	ERGIC3	Endoplasmic reticulum-Golgi intermediate compartment protein 3	NM_001033105.1
A4G0823	2.96	2.55	IPO8	Importin-8	NM_001044334.2
B5G0232	2.96	3.00	DNAJC25	DnaJ homolog subfamily C member 25	NM_001205027.1
B14G0541	2.96	8.78	PPP3CA	Protein phosphatase 3 catalytic subunit alpha	XM_009307722.4
A15G0421	2.96	17.36	PAFAH1B1A	Lissencephaly-1 homolog A	XM_005157575.5
B25G0649	2.96	3.62	DET1	DET1 homolog	NM_199670.1
B17G0700	2.96	1.44	KIF26A	Kinesin-like protein KIF26A	XM_005158960.5
B24G0067	2.96	4.15	LOC113086373	A0A6P6NQL7_CARAU Nuclear apoptosis-inducing factor 1	
B9G0669	2.96	3.72	TTLL4	Tubulin monoglutamylase TTLL4	NM_001430833.1
A16G0982	2.96	2.45	CYB5R4	Cytochrome b5 reductase 4	XM_068213961.1
B22G0089	2.96	1.25	LOC113075279	A0A6P6N435_CARAU zinc finger protein 271-like isoform X3	XM_009304648.4
A7G0316	2.96	3.73	NSMAF	Protein FAN	XM_003199002.6
B3G0856	2.96	9.83	DUSP8	Dual specificity protein phosphatase 8	XM_681250.8
B22G0311	2.96	10.67	LOC113040792	A0A6P6J5X6_CARAU carcinoembryonic antigen-related cell adhesion molecule 1-like	XM_068217854.1
B25G0484	2.96	3.01	NT5DC3	5'-nucleotidase domain-containing protein 3	XM_681686.7
B6G0852	2.96	7.18	MOV10B.1	Putative helicase mov-10-B.1	XM_002662530.5
B11G0560	2.96	0.00	NXPH2	Neurexophilin-2	
B5G0580	2.96	4.64	PTAR1	Protein prenyltransferase alpha subunit repeat-containing protein 1	NM_001130074.1
A23G0069	2.96	5.48	TOP1	DNA topoisomerase 1	XM_002666402.6
B4G0097	2.96	6.85	AEBP2	Zinc finger protein AEBP2	NM_213494.2
A6G0519	2.96	3.07	NME7	Nucleoside diphosphate kinase 7	NM_130929.2
A20G0674	2.96	2.35	TTR	Transthyretin	NM_001005598.2
A4G0809	2.96	5.84	TMEM243	Transmembrane protein 243	NM_212919.1
B12G0287	2.96	4.37	ZNF646	Zinc finger protein 646	XM_684608.9
B21G0856	2.96	5.72	SETX	Probable helicase senataxin	XM_685853.9
A19G0056	2.96	18.50	THRB	Thyroid hormone receptor beta	XM_005159501.5
B19G0674	2.96	2.34	GMNN	Geminin	NM_200086.1
A15G0718	2.96	4.41	FOXO1A	Forkhead box protein O1-A	XM_009291427.4
A2G0395	2.96	2.24	ABCD3	ATP-binding cassette sub-family D member 3	NM_001130620.1
A1G0685	2.95	4.89	TMEM39A	Transmembrane protein 39A	NM_199632.1
B7G1246	2.95	4.08	CD2	T-cell surface antigen CD2	
B15G0507	2.95	4.74	PHF12	PHD finger protein 12	XM_021466429.2
B7G0215	2.95	7.15	MYO9AA	Unconventional myosin-IXAa	NM_001423815.1
B7G1118	2.95	4.82	TMEM179B	Transmembrane protein 179B	NM_001020489.2
A12G0343	2.95	6.53	KCTD2	POZ domain-containing protein KCTD2	NM_001045434.1
A5G0745	2.95	5.30	BMP2K	BMP-2-inducible protein kinase	XM_005165303.5
B7G0546	2.95	22.76	CCL3	C-C motif chemokine 3	
A17G0764	2.95	3.52	SHPRH	E3 ubiquitin-protein ligase SHPRH	XM_009292830.4
A2G0362	2.95	1.31	ELOVL1	Elongation of very long chain fatty acids protein 1	NM_001005989.3
A7G0830	2.95	2.59	SENP3	Sentrin-specific protease 3	NM_001083842.2

B17G0195	2.95	6.06	ATG14	Beclin 1-associated autophagy-related key regulator	NM_001024812.2
B8G0703	2.95	4.17	STAP2	Signal-transducing adaptor protein 2	NM_001076565.1
B24G0232	2.95	4.52	LOC107732090	A0A673K1U2_9TELE Uncharacterized LOC107732090	XM_002660922.7
B19G0592	2.95	0.54	GJB4	Gap junction beta-4 protein	NM_001007288.2
A4G0843	2.95	3.70	WNT5B	Protein Wnt-5b	NM_001362273.1
B25G0832	2.95	3.85	DENND2A	DENN domain-containing protein 2A	XM_021470581.2
A17G0564	2.95	2.71	ECD	Protein ecdysoneless homolog	NM_199891.2
A7G1389	2.95	2.29	LOC113093137	A0A6P6P233_CARAU uncharacterized protein LOC113093137	XM_021477648.1
A2G0704	2.95	6.19	COP1	E3 ubiquitin-protein ligase COP1	NM_001089542.1
A14G0759	2.95	0.53	FGF16	Fibroblast growth factor 16	NM_001040407.1
B22G0150	2.95	2.75			XM_021469374.2
A18G0191	2.95	3.04	MAF	Transcription factor Maf	NM_131844.2
A4G0461	2.95	2.50	TBC1D22A	TBC1 domain family member 22A	XM_005168538.5
B13G0217	2.95	9.69	SPATA48	Spermatogenesis-associated protein 48	NM_001017594.1
A3G0297	2.95	1.35	DNAAF5	Dynein axonemal assembly factor 5	XM_021471368.2
A18G0348	2.95	2.33	TRIM25	ISG15 ligase TRIM25	XM_068214910.1
A1G0863	2.95	1.66	PGAP6	Post-GPI attachment to proteins factor 6	XM_005170863.5
B11G0418	2.95	5.00	ADIPOR1	Adiponectin receptor protein 1	
A13G0288	2.95	2.75	VTI1B	Vesicle transport through interaction with t-SNAREs homolog 1B	NM_001082551.1
B7G0481	2.95	5.20	CMTM4	CKLF-like MARVEL transmembrane domain-containing protein 4	NM_001002492.1
A7G0370	2.95	3.45	RBPJ	Suppressor of hairless protein homolog	NM_001083551.1
B22G0495	2.95	0.20	G5714_021498	A0A7J6BR45_9TELE Ig-like domain-containing protein	XM_068216400.1
B23G0940	2.95	5.09	PSIP1	PC4 and SFRS1-interacting protein	XM_021470060.2
B21G0090	2.95	9.44	PDLIM4	PDZ and LIM domain protein 4	NM_001042696.2
B12G0400	2.95	5.76	NEURL4	Neuralized-like protein 4	XM_005156224.5
B2G0880	2.94	2.84	KIFAP3	Kinesin-associated protein 3	NM_001144045.1
B19G0030	2.94	5.60	TC1A	Transposable element Tc1 transposase	
A5G0550	2.94	3.44	SPNS3	Protein spinster homolog 3	NM_001100036.1
B21G0781	2.94	2.69	AP3B1	AP-3 complex subunit beta-1	XM_021469001.2
A1G0695	2.94	7.47	POU2F1	POU domain, class 2, transcription factor 1	NM_131438.1
B23G0456	2.94	5.71	ZBTB17	Zinc finger and BTB domain-containing protein 17	XM_003201203.6
A10G0750	2.94	11.89	SWI5	DNA repair protein SWI5 homolog	NM_001326527.1
B17G0682	2.94	2.25	CDAN1	Codanin-1	XM_688592.10
B18G0837	2.94	7.87	SECISBP2L	Selenocysteine insertion sequence-binding protein 2-like	XM_068214990.1
B20G0812	2.94	3.12	ZC3H14	Zinc finger CCCH domain-containing protein 14	NM_001025524.2
A7G1354	2.94	4.14	TOX4	TOX high mobility group box family member 4	NM_001128286.1

B14G0361	2.94	13.80	TSPAN7	Tetraspanin-7	NM_214774.2
A24G0553	2.94	5.21	LZTFL1	Leucine zipper transcription factor-like protein 1	NM_199587.1
B2G0359	2.94	3.62	NMNAT3	nicotinic acid mononucleotide adenylyltransferase 3	XM_695734.8
A7G0282	2.94	5.26	MATCAP	Microtubule-associated tyrosine carboxypeptidase	XM_021477988.2
A3G1317	2.94	1.53	LOC107675477	A0A671KBP6_9TELE Gastrula zinc finger protein XlCGF57.1-like	XM_068220086.1
B2G1275	2.94	10.49	G5714_001543	A0A7J6DCM3_9TELE Uncharacterized protein	
B2G0766	2.94	2.69	MSL2	E3 ubiquitin-protein ligase MSL2	NM_001163298.1
B7G1224	2.94	2.02	PRKD3	threonine-protein kinase D3	NM_001177454.1
B22G1021	2.94	0.97	NLRC3	NLR family CARD domain-containing protein 3	XM_021478497.2
B5G1220	2.94	3.64	THADA	Thyroid adenoma-associated protein homolog	XM_005165200.5
B24G0102	2.94	36.38	ATP6V0C	V-type proton ATPase 16 kDa proteolipid subunit c	NM_205554.1
B23G0017	2.94	9.89	IRF4	Interferon regulatory factor 4	XM_005161894.4
B3G0667	2.94	1.61	TMEM238	Transmembrane protein 238	NM_001386432.1
B4G0447	2.94	5.54	RECQL	ATP-dependent DNA helicase Q1	XM_068219694.1
A13G0322	2.94	6.25	LRRC9	Leucine-rich repeat-containing protein 9	NM_001017804.1
A23G0079	2.94	1.87	NLRP3	NACHT, LRR and PYD domains-containing protein 3	XM_068216979.1
A6G0219	2.94	8.59	WIPF1	WASL-interacting protein family member 1	XM_068221901.1
A3G1080	2.94	5.71	TOM1	Target of Myb1 membrane trafficking protein	NM_001351651.1
B12G0053	2.94	8.12	PALD1	Paladin	NM_198811.1
A7G0037	2.94	3.24	LURAP1L	Leucine rich adaptor protein 1-like	
A6G0561	2.94	7.95	USP1	Ubiquitin carboxyl-terminal hydrolase 1	NM_199579.1
A16G1040	2.93	2.92	TFPT	TCF3 fusion partner homolog	NM_001160707.1
A14G0030	2.93	3.73	ANKRD50	Ankyrin repeat domain-containing protein 50	XM_068213424.1
A8G0957	2.93	3.42	CHDH	Choline dehydrogenase, mitochondrial	XM_002663255.6
B13G0442	2.93	2.46	SPEF1	Sperm flagellar protein 1	NM_001126432.1
A13G0314	2.93	5.56	TRMT5	tRNA (guanine(37)-N1)-methyltransferase	XM_005156536.5
A8G0134	2.93	3.19	ASB6	Ankyrin repeat and SOCS box protein 6	NM_001020576.2
A23G0197	2.93	4.13	G5714_022879	A0A7J6BPH9_9TELE Uncharacterized protein	NM_001386475.1
B19G0677	2.93	1.81	TDP2	Tyrosyl-DNA phosphodiesterase 2	NM_001079703.1
A3G0021	2.93	6.86	SREBF2	Sterol regulatory element-binding protein 2	NM_001089466.1
B5G0884	2.93	16.21	KYAT1	Kynurenine--oxoglutarate transaminase 1	NM_213359.2
B1G0345	2.93	3.36	KLHL2	Kelch-like protein 2	XM_021473892.2
A24G0240	2.93	3.82	PTBP2	Polypyrimidine tract-binding protein 2	NM_001328429.1
A5G1090	2.93	5.70	RTASE	Probable RNA-directed DNA polymerase from transposon BS	XM_068222002.1

B19G0868	2.93	1.65	KDF1	Keratinocyte differentiation factor 1	XM_005173776.5
B5G0249	2.93	2.42	DUSP18	Dual specificity protein phosphatase 18	
A1G0353	2.93	7.10	TC3A	Transposable element Tc3 transposase	
A7G0206	2.93	8.45	SWAP70	Switch-associated protein 70	NM_001362396.1
A20G0055	2.93	12.70	CTSB	Cathepsin B	NM_001431146.1
B1G0226	2.93	8.18	GRK4	G protein-coupled receptor kinase 4	NM_001079986.2
A15G0664	2.93	1.98	TMPRSS13	Transmembrane protease serine 13	
B1G0632	2.93	4.34	FAAH2A	Fatty-acid amide hydrolase 2-A	NM_001002700.2
A4G0949	2.93	6.95	PIK3CG	Phosphatidylinositol 4,5-bisphosphate 3-kinase catalytic subunit gamma isoform	XM_021474981.2
A7G0329	2.93	7.10	TC3A	Transposable element Tc3 transposase	
A3G0812	2.93	1.55	EEF2KMT	Protein-lysine N-methyltransferase EEF2KMT	
B7G1072	2.93	1.97	RCE1	CAAX prenyl protease 2	XM_021471869.2
A19G0596	2.93	2.70	NUDCD1	NudC domain-containing protein 1	NM_001020627.2
B13G0286	2.93	3.96	FGFR3	Fibroblast growth factor receptor 3	NM_001305560.1
A18G0338	2.93	5.35	NUCB2	Nucleobindin-2	NM_201479.1
A25G0820	2.93	7.10	TC3A	Transposable element Tc3 transposase	
B5G0156	2.93	3.59	PHETA1	Sesquipedalian-1	XM_688552.8
A25G0009	2.93	7.10	TC3A	Transposable element Tc3 transposase	
B24G0028	2.93	7.48	TRIM25	ISG15 ligase TRIM25	XM_690458.9
A19G0148	2.93	6.69	RIT1	GTP-binding protein Rit1	NM_001128781.1
B20G0011	2.93	5.26	SLC16A10	Monocarboxylate transporter 10	XM_005160300.5
B13G0205	2.93	4.80	LOC113113188	A0A6P6QNZ5_CARAU uncharacterized protein LOC113113188	NM_001347683.1
A5G0231	2.92	1.62	KREMEN1	Kremen protein 1	NM_001114917.1
A19G0333	2.92	3.42	ELP2	Elongator complex protein 2	NM_001044925.1
B21G0476	2.92	1.93	RB1	Retinoblastoma-associated protein	NM_001077780.1
B14G0587	2.92	0.87	CASP3	Caspase-3	
B21G0447	2.92	13.18	SMS	Spermine synthase	NM_131756.1
B1G0014	2.92	3.26	POU2F1	POU domain, class 2, transcription factor 1	NM_131438.1
B11G0256	2.92	3.75	DPX16_16135	A0A3N0YNI3_ANAGA Multidrug resistance-associated protein 7	NM_001114554.2
B9G0228	2.92	3.10	POFUT2	GDP-fucose protein O-fucosyltransferase 2	NM_001077630.2
A23G0565	2.92	12.10	TNFRSF9	Tumor necrosis factor receptor superfamily member 9	
B5G0666	2.92	15.73	DAB2	Disabled homolog 2	XM_021471673.2
A13G0725	2.92	8.47	PITX3	Pituitary homeobox 3	NM_001008612.1
B20G0467	2.92	4.39	DLL4	Delta-like protein 4	NM_001079835.1
B14G0025	2.92	10.72	TRIM16	Tripartite motif-containing protein 16	XM_681071.8
A21G0675	2.92	2.66	SLC35A4	SLC35A4 upstream open reading frame protein	NM_001386395.1
B22G0688	2.92	3.18	IFI44L	Interferon-induced protein 44-like	NM_001386825.1
A21G0883	2.92	3.51	LIPH	Lipase member H	

A2G0125	2.92	6.21	TSC22D2	TSC22 domain family protein 2	
A20G0511	2.92	1.73	FOXC1B	Forkhead box C1-B	NM_131729.2
A9G0428	2.92	2.90	GTPBP8	GTP-binding protein 8	NM_001126402.1
A7G0178	2.92	2.75	ATXN1L	Ataxin-1-like	XM_001333745.8
A8G0303	2.92	2.27	CENPH	Centromere protein H	NM_001113609.1
A10G0470	2.92	5.17	CBLB	E3 ubiquitin-protein ligase CBL-B	XM_021474212.2
B22G0258	2.92	0.22	LOC113040559	A0A6P6J3I0_CARAU uncharacterized protein LOC113040559	XM_692962.9
A9G0622	2.92	1.38	CD28	T-cell-specific surface glycoprotein CD28	
B18G0633	2.92	2.71	MEAK7	MTOR-associated protein MEAK7	NM_001044965.2
A6G0769	2.92	3.28	SEMA3F	Semaphorin-3F	XM_005166039.4
B18G0075	2.92	3.52	RNPEPL1	Aminopeptidase RNPEPL1	NM_001348188.1
B3G1464	2.92	1.31	LOC113078295	A0A6P6NB09_CARAU gastrula zinc finger protein XlCGF57.1-like	XM_017356396.3
B16G0720	2.92	2.19	IL16	Pro-interleukin-16	XM_005169750.5
B16G0238	2.92	3.74	POLR3GL	DNA-directed RNA polymerase III subunit RPC7-like	NM_001005959.2
A13G0222	2.92	6.76			XM_068212912.1
B6G0346	2.92	4.71	STK11IP	threonine-protein kinase 11-interacting protein	XM_005166228.5
B13G0082	2.92	8.13	PSMB1-A	Proteasome subunit beta type-1-A	XM_002664229.7
A7G1128	2.92	3.63	KIF7	Kinesin-like protein kif7	NM_001014816.1
A4G0963	2.92	14.74	OSBPL8	Oxysterol-binding protein-related protein 8	XM_021475194.2
A19G0583	2.92	5.16	ATP9B	Probable phospholipid-transporting ATPase IIB	NM_001045154.1
A1G0976	2.92	3.34	CFAP97	Cilia- and flagella-associated protein 97	
A2G0856	2.91	6.69	CHD8	Chromodomain-helicase-DNA-binding protein 8	NM_001347671.1
B24G0085	2.91	12.71	FAM234A	Protein FAM234A	
B6G0018	2.91	5.66	HMGXB4	HMG box-containing protein 4	
B2G0888	2.91	2.42	ODR4	Protein odr-4 homolog	NM_200616.3
B25G0482	2.91	3.92	TDG	T mismatch-specific thymine DNA glycosylase	XM_009297976.4
A3G0107	2.91	1.35	X-ELEMENT\ORF2	Probable RNA-directed DNA polymerase from transposon X-element	
B14G0671	2.91	7.32	KDM2A	Lysine-specific demethylase 2A	XM_001339761.8
A22G0391	2.91	3.46	TMEM161A	Transmembrane protein 161A	XM_068216285.1
A1G0383	2.91	4.11	PKN1	threonine-protein kinase N1	XM_005160051.5
B13G0594	2.91	7.15	UTP25	U3 small nucleolar RNA-associated protein 25 homolog	NM_201309.1
B21G0106	2.91	3.24	GPR34	Probable G-protein coupled receptor 34	
B12G0637	2.91	3.54	TUT1	Speckle targeted PIP5K1A-regulated poly(A) polymerase	NM_001030188.1
A1G0991	2.91	5.79	TBC1D1	TBC1 domain family member 1	XM_684525.10
B14G0020	2.91	4.95	TMEM107	Transmembrane protein 107	NM_213211.1
A25G0559	2.91	38.28	GSE1	Genetic suppressor element 1	XM_003201442.6
B14G0316	2.91	4.59	ELOVL6	Elongation of very long chain fatty acids protein 6	NM_199532.1
B15G0198	2.91	4.51	RIN2	Ras and Rab interactor 2	XM_068213677.1
B20G0890	2.91	13.90	BROX	BRO1 domain-containing protein BROX	NM_001007427.2

A21G0355	2.91	1.33	LOC107591698	A0A672NVN4_SINGR Protein regulator of cytokinesis 1-like	NM_001002684.2
B12G0778	2.91	3.97	LMF1	Lipase maturation factor 1	XM_017358494.3
B2G1194	2.91	6.48	ABHD3	Phospholipase ABHD3	NM_001004569.2
B17G0402	2.90	1.19	HTR7	5-hydroxytryptamine receptor 7	XM_685507.10
B9G0700	2.90	17.05	FHL2	Four and a half LIM domains protein 2	XM_068223359.1
B16G0188	2.90	12.58	DPX16_6815	A0A3N0Y5I1_ANAGA Proline-rich transmembrane protein 1	
B11G0253	2.90	1.43	SOCS7	Suppressor of cytokine signaling 7	XM_005155820.5
B2G0646	2.90	3.33	MRC1	Macrophage mannose receptor 1	NM_001423726.1
B19G0636	2.90	3.57	LRATD2	Protein LRATD2	NM_001044880.2
A25G0780	2.90	8.63	ZFC3H1	Zinc finger C3H1 domain-containing protein	XM_068217602.1
B12G0669	2.90	2.55	POLR3D	DNA-directed RNA polymerase III subunit RPC4	NM_001109828.1
B23G0348	2.90	6.39	EBP	3-beta-hydroxysteroid-Delta(8),Delta(7)-isomerase	NM_001002328.1
A20G0294	2.90	3.67	PEX3	Peroxisomal biogenesis factor 3	NM_200228.1
B18G0449	2.90	3.76	RANBP10	Ran-binding protein 10	XM_005159142.5
A4G0347	2.90	0.59	NLRC3	Protein NLRC3	XM_068220538.1
B3G0539	2.90	5.68	IL21R	Interleukin-21 receptor	NM_001113510.2
A3G0447	2.90	4.54	USP31	Ubiquitin carboxyl-terminal hydrolase 31	XM_005164229.5
B1G0766	2.90	3.96	MAP2K7	Dual specificity mitogen-activated protein kinase kinase 7	NM_001190306.1
A17G0503	2.90	6.04	TBC1D12	TBC1 domain family member 12	XM_021473152.2
A2G0724	2.90	0.74	TRAV17	T cell receptor alpha variable 17	
B5G0558	2.90	15.78	GLDC	Glycine dehydrogenase (decarboxylating), mitochondrial	NM_199554.1
A1G0651	2.90	2.76	GEMIN8	Gem-associated protein 8	XM_005168381.5
B22G0044	2.90	6.53	TC1A	Transposable element Tc1 transposase	
B7G0860	2.90	7.55	BAD	Bcl2-associated agonist of cell death	NM_131579.2
A23G0709	2.90	6.39	CPTP	Ceramide-1-phosphate transfer protein	NM_001003497.1
A11G0198	2.90	6.31	ROHU_016520	A0A498NJ77_LABRO Uncharacterized protein	NM_001044311.2
A17G0238	2.90	3.78	DNMT3A	DNA (cytosine-5)-methyltransferase 3A	NM_001328167.1
B21G0078	2.90	6.54	ANKRD46	Ankyrin repeat domain-containing protein 46	XM_002666073.7
B8G0697	2.90	15.59	ZFAND5	AN1-type zinc finger protein 5	XM_005166804.5
B25G0114	2.90	3.04	WWOX	WW domain-containing oxidoreductase	NM_200913.1
A18G0722	2.90	6.73	FCHSD2	F-BAR and double SH3 domains protein 2	XM_017354494.1
A11G0043	2.90	3.32			NM_001020537.1
A25G0712	2.90	4.45	NFATC3	Nuclear factor of activated T-cells, cytoplasmic 3	XM_009298212.4
B22G0810	2.90	1.72	LHX9	homeobox protein Lhx9	NM_001037243.2
A25G0514	2.90	0.64	GCNT3	Beta-1,3-galactosyl-O-glycosyl-glycoprotein beta-1,6-N-acetylglucosaminyltransferase 3	XM_002666917.7
B19G0697	2.90	5.30	BAG6	Large proline-rich protein BAG6	XM_017352270.3
A3G0483	2.90	1.49	CHST12	Carbohydrate sulfotransferase 12	NM_213547.2
A5G1026	2.90	2.94	LIG3	DNA ligase 3	NM_001030174.2

B16G0283	2.90	4.07	PTPN23	Tyrosine-protein phosphatase non-receptor type 23	NM_001202507.1
B3G0986	2.90	17.80	ABAT	4-aminobutyrate aminotransferase, mitochondrial	NM_201498.2
A9G0435	2.90	5.81	ACVR2A	Activin receptor type-2A	XM_021478661.2
A9G0423	2.90	2.88	GTF2E1	General transcription factor IIE subunit 1	XM_005167734.5
A4G0061	2.90	2.13	G5714_022074	A0A7J6BSV8_9TELE Uncharacterized protein	XM_068220328.1
B6G0557	2.89	1.30	ALG6	Dolichyl pyrophosphate Man9GlcNAc2 alpha-1,3-glucosyltransferase	NM_001003784.2
B16G0831	2.89	2.93	ROHU_019498	A0A498N8V9_LABRO Uncharacterized protein	
B16G0647	2.89	6.79	ZNF711	Zinc finger protein 711	NM_001328215.1
B17G0492	2.89	3.43	FAM149B1	Protein FAM149B1	XM_005158524.5
A6G0552	2.89	1.37	CACHD1	VWFA and cache domain-containing protein 1	XM_001334793.8
A17G0153	2.89	8.59	ADO	2-aminoethanethiol dioxygenase	NM_213193.2
A9G0624	2.89	3.10	LOC113053617	A0A6P6KQJ6_CARAU uncharacterized protein LOC113053617 isoform X1	XM_005167514.5
A3G0593	2.89	12.45	SLC16A6	Monocarboxylate transporter 7	NM_001365238.1
B23G0419	2.89	3.63	ICMT	Protein-S-isoprenylcysteine O-methyltransferase	NM_001017618.1
B16G0133	2.89	2.19	SLC25A32	carrier	NM_001013336.2
A14G0758	2.89	12.74	ATRX	Transcriptional regulator ATRX	NM_200653.2
A5G0751	2.89	5.14	BMP3	Bone morphogenetic protein 3	NM_001077765.1
B23G0262	2.89	9.63	OSBPL2	Oxysterol-binding protein-related protein 2	XM_005161948.5
A16G0202	2.89	3.16	ATAD2	ATPase family AAA domain-containing protein 2	
B5G0315	2.89	6.43	RPS6KA4	Ribosomal protein S6 kinase alpha-4	XM_005165615.5
B1G1000	2.89	0.22	ADGRE3	Adhesion G protein-coupled receptor E3	XM_017356711.3
B20G0054	2.89	4.00	LATS1	threonine-protein kinase LATS1	XM_005160312.5
A13G0125	2.89	1.86	BTAF1	TATA-binding protein-associated factor 172	XM_021480713.2
B17G0222	2.89	4.81	LRRC57	Leucine-rich repeat-containing protein 57	NM_001002627.1
A13G0405	2.89	3.19	SLC2A9	Solute carrier family 2, facilitated glucose transporter member 9	NM_214782.2
A13G0256	2.89	2.42	MACROD2	ADP-ribose glycohydrolase MACROD2	NM_001007414.1
B17G0833	2.89	2.99	ORC3	Origin recognition complex subunit 3	NM_212727.2
A18G0099	2.89	3.73	FAM118B	Protein FAM118B	XM_005159255.5
A23G0155	2.89	6.09	BAZ2A	Bromodomain adjacent to zinc finger domain protein 2A	NM_001320131.1
B5G0047	2.89	5.63	MCC	Colorectal mutant cancer protein	XM_021476718.2
B17G0500	2.89	4.17	ASMT	Acetylserotonin O-methyltransferase	NM_001110477.1
A1G0586	2.89	10.61	CREB1	Cyclic AMP-responsive element-binding protein 1	NM_200909.1
B20G0315	2.89	1.31	SGPP1	Sphingosine-1-phosphate phosphatase 1	XM_679255.9
B5G0944	2.89	10.48	ANKFY1	Rabankyrin-5	XM_005171905.5
B14G0414	2.89	2.64	RAB33B	Ras-related protein Rab-33B	NM_001033592.1
A3G0238	2.88	5.65	GLIS2	Zinc finger protein GLIS2	XM_021469271.2

B17G0127	2.88	6.49	STXBP5	Syntaxin-binding protein 5	XM_068214517.1
B1G0301	2.88	3.09	LOC113044187	A0A6P6JJA0_CARAU uncharacterized protein LOC113044187	
B3G0902	2.88	8.56	DDX42	ATP-dependent RNA helicase DDX42	NM_001037805.2
B12G0477	2.88	2.39	RHBDF1	Inactive rhomboid protein 1	XM_021472526.2
B9G0268	2.88	4.76	SLC15A2	Solute carrier family 15 member 2	NM_001039828.1
B21G0868	2.88	9.19	SCAMP1	Secretory carrier-associated membrane protein 1	NM_205692.1
B24G0680	2.88	2.36	DIPK2A	Divergent protein kinase domain 2A	XM_005162483.5
B1G0831	2.88	3.53	TBCK	TBC domain-containing protein kinase-like protein	NM_001080637.2
A17G0401	2.88	4.99	NRDE2	Nuclear exosome regulator NRDE2	XM_005158556.5
A9G0373	2.88	0.57	IMPG2	Interphotoreceptor matrix proteoglycan 2	XM_021478983.2
A22G0689	2.88	11.42	ACAP2	Arf-GAP with coiled-coil, ANK repeat and PH domain-containing protein 2	NM_001102639.1
A14G0016	2.88	3.65	ZNF518B	Zinc finger protein 518B	XM_005173052.5
B10G0674	2.88	1.62	CABP1	Calcium-binding protein 1	XM_068223769.1
A15G0683	2.88	1.75			
B10G0027	2.88	4.59	WRN	ATP-dependent helicase WRN	XM_068223902.1
A7G1110	2.88	1.97	TRIM27	Zinc finger protein RFP	NM_001127479.1
B9G0866	2.88	0.64	DLX2A	Homeobox protein Dlx2a	NM_131311.2
A12G0634	2.88	2.80	MED24	Mediator of RNA polymerase II transcription subunit 24	NM_001039062.1
B20G0904	2.88	4.08	TBC1D7	TBC1 domain family member 7	XM_021468380.2
B5G0276	2.88	2.19	EPHB4A	Ephrin type-B receptor 4a	NM_131414.1
A21G0592	2.88	8.32	PURA	Transcriptional activator protein Pur-alpha	NM_001001846.2
A8G0992	2.88	3.75	PLXND1	Plexin-D1	NM_205697.2
A25G0238	2.88	0.39	GINS3	DNA replication complex GINS protein PSF3	
A14G0127	2.88	2.68	PLD3	5'-3' exonuclease PLD3	XM_005173180.5
B14G0649	2.88	0.90	FGF18	Fibroblast growth factor 18	NM_001013264.1
A7G0660	2.88	4.06	FHOD3	FH2 domain-containing protein 3	XM_021477913.2
A1G0065	2.88	2.15	SI:CH73-236C18.8	F1R3I8_DANRE Si:ch73-236c18.8	NM_001386651.1
B2G1260	2.88	2.25	RIPK1	threonine-protein kinase 1	NM_001043350.1
B15G0006	2.88	5.88	AXL	Tyrosine-protein kinase receptor UFO	XM_017351109.3
A3G0802	2.88	4.76	APOL3	Apolipoprotein L3	XM_001339206.9
A5G0330	2.88	1.19	GLRA4	Glycine receptor subunit alpha-4	NM_001202511.1
B14G0446	2.87	1.65	SIMC1	SUMO-interacting motif-containing protein 1	XM_001922548.6
A25G0689	2.87	4.85	RFWD3	E3 ubiquitin-protein ligase RFWD3	XM_002666962.5
A24G0681	2.87	2.57			
A20G0848	2.87	1.54	SLC22A6-A	Solute carrier family 22 member 6-A	NM_001044934.1
B25G0067	2.87	9.62	LSM14	Protein LSM14 homolog	
A3G0129	2.87	3.06	A33	Zinc-binding protein A33	NM_001089399.2
B21G0489	2.87	2.54	ARHGAP42A	Rho GTPase-activating protein 42	NM_001202443.2
A12G0819	2.87	2.69	SLC39A11	Zinc transporter ZIP11	XM_068224850.1
A19G0822	2.87	4.80	TAP2	Antigen peptide transporter 2	NM_001006594.1

B2G1241	2.87	3.44	G5714_001499	A0A7J6DCA4_9TELE Uncharacterized protein	XM_005171117.5
B22G0665	2.87	17.53	PLEKHB2	Pleckstrin homology domain-containing family B member 2	NM_201002.1
A15G0234	2.87	5.97	SESN3	Sestrin-3	NM_213519.2
B6G0539	2.87	1.83	PARS2	Probable proline--tRNA ligase, mitochondrial	NM_205555.1
B9G0690	2.87	11.88	MGAT4A	Alpha-1,3-mannosyl-glycoprotein 4-beta-N-acetylglucosaminyltransferase A	XM_068223540.1
B2G0534	2.87	2.43	IFI44L	Interferon-induced protein 44-like	NM_001077169.2
B3G0474	2.87	2.51	CAMSAP3	Calmodulin-regulated spectrin-associated protein 3	XM_021474746.2
B4G0663	2.87	0.57	TRIM16	Tripartite motif-containing protein 16	XM_690458.9
A21G0344	2.87	2.64	RAI14	Ankycorbin	NM_001111217.2
B24G0706	2.87	1.33	FARS2	Phenylalanine--tRNA ligase, mitochondrial	XM_068217123.1
B16G0660	2.87	3.25	ELMO1	Engulfment and cell motility protein 1	XM_001338913.8
B7G0260	2.87	4.89	ROHU_003968	A0A498NRK7_LABRO Interferon-induced 44	
A14G0070	2.87	2.16	SMYD5	Histone-lysine N-trimethyltransferase SMYD5	NM_001004614.2
A4G0805	2.87	7.76	TSPAN9	Tetraspanin-9	XM_068219583.1
A20G0441	2.87	5.51	ADAM17	Disintegrin and metalloproteinase domain-containing protein 17	XM_021468637.2
B7G0781	2.87	2.28	THAP11	THAP domain-containing protein 11	NM_213102.1
A7G0832	2.87	0.38	SOX19B	Transcription factor Sox-19b	NM_131702.1
A16G0267	2.87	2.38	NDUFAF6	NADH dehydrogenase (ubiquinone) complex I, assembly factor 6	XM_021466778.2
B11G0332	2.87	3.52	PHTF1	Protein PHTF1	NM_001030278.2
B7G0403	2.87	2.43	CDKN2AIP-A	Protein CDKN2AIP homolog A	NM_001006085.1
A3G0932	2.87	5.35	SGF29	SAGA-associated factor 29	XM_005163811.5
B6G0794	2.87	3.04	ARL6IP5	PRA1 family protein 3	NM_001006096.1
A9G0239	2.86	12.23	ZBED4	Zinc finger BED domain-containing protein 4	
B3G0652	2.86	4.21	TUBG1	Tubulin gamma-1 chain	NM_200908.2
A6G0714	2.86	10.97	P4HTM	Transmembrane prolyl 4-hydroxylase	XM_001340198.6
A19G0312	2.86	3.88	SPIRE1	Protein spire homolog 1	XM_009294322.4
A3G0881	2.86	9.13	PLCL2	Inactive phospholipase C-like protein 2	XM_068216120.1
B19G0063	2.86	7.96	SH3BP5	SH3 domain-binding protein 5	XM_677677.10
B2G0532	2.86	0.32	TRAV3	T cell receptor alpha variable 3	
A7G0895	2.86	2.31	TESK1	Dual specificity testis-specific protein kinase 1	XM_009303137.4
A8G0357	2.86	12.23	ZBED4	Zinc finger BED domain-containing protein 4	
A15G0288	2.86	3.84	B3GLCT	Beta-1,3-glucosyltransferase	NM_001302251.1
A15G0611	2.86	4.82	SYNRG	Synergin gamma	XM_005157439.5
A4G0024	2.86	2.05	DPX16_8534	A0A3N0YAL6_ANAGA Uncharacterized protein	
B17G0691	2.86	1.45	G5714_017230	A0A7J6C5D0_9TELE Uncharacterized protein	XM_700969.7
B1G0866	2.86	1.98	MEIS1	Homeobox protein Meis1	NM_131897.2
A9G0854	2.86	0.62	G5714_004447	A0A7J6D5J8_9TELE Uncharacterized protein	XM_068213809.1

A8G0317	2.86	6.20	SSBP3	Single-stranded DNA-binding protein 3	XM_005166809.5
A3G0599	2.86	1.61	DUS1L	17) synthase [NAD(P)(+)]-like	XM_005163994.4
B15G0867	2.86	0.94	LOC113081243	A0A6P6NKT8_CARAU zinc finger protein 420-like	XM_068218143.1
B22G0878	2.86	2.71	LOC113065055	A0A6P6M5E7_CARAU uncharacterized protein LOC113065055	
A1G0127	2.86	0.63	GIMAP4	GTPase IMAP family member 4	XM_021478077.2
B22G1184	2.86	2.03	GIMAP8	GTPase IMAP family member 8	XM_017354628.3
B14G0342	2.86	6.38	GLA	Alpha-galactosidase A	NM_001006103.2
A10G0018	2.86	9.56	NHLRC3	NHL repeat-containing protein 3	NR_023334.2
B16G0370	2.86	1.57	DPX16_9330	A0A3N0Y6D8_ANAGA Integrase catalytic domain-containing protein	
B16G0836	2.86	14.53	ASH1L	Histone-lysine N-methyltransferase ASH1L	NR_147500.1
B3G1266	2.86	4.77	GIMAP4	GTPase IMAP family member 4	NM_001127520.1
A5G0174	2.86	3.70	RFC5	Replication factor C subunit 5	NM_001003862.1
A5G0786	2.86	6.18	BCL7A	lymphoma 7 protein family member A	XM_005165362.5
A14G0522	2.86	8.63	RUFY1	RUN and FYVE domain-containing protein 1	XM_002667315.7
A11G0666	2.86	1.17	PBRM1	Protein polybromo-1	
B22G0295	2.86	9.35	LOC113039555	A0A6P6IZQ1_CARAU junctional adhesion molecule C-like isoform X1	XM_005161645.5
B1G0056	2.86	3.02	DONSON	Protein downstream neighbor of Son	
B8G0961	2.86	8.71	SEPTIN5	Septin-5	NM_199988.1
B3G0014	2.85	2.02	LOC113067371	A0A6P6MGL9_CARAU zinc finger protein 239-like isoform X1	NM_001114464.1
B2G0740	2.85	4.50	NDC1	Nucleoporin NDC1	NM_199943.1
A9G0441	2.85	4.16	LRCH1	Leucine-rich repeat and calponin homology domain-containing protein 1	XM_005167643.5
B24G0133	2.85	14.47	IGKV1D-8	Immunoglobulin kappa variable 1D-8	NM_001045408.1
A3G0442	2.85	3.05	IL21R	Interleukin-21 receptor	NM_001113510.2
B9G0871	2.85	20.21	UBR3	E3 ubiquitin-protein ligase ubr3	XM_021478823.2
B25G0179	2.85	2.79	IGKV3D-7	Immunoglobulin kappa variable 3D-7	
A18G0326	2.85	2.28	QPCTL	Glutaminyl-peptide cyclotransferase-like protein	NM_001044953.1
A23G0784	2.85	1.52	GPX7	Glutathione peroxidase 7	NM_001020501.1
B6G0486	2.85	3.70	RYK	Tyrosine-protein kinase RYK	XM_005165931.5
A19G0204	2.85	3.37	ZBTB12	Zinc finger and BTB domain-containing protein 12	NM_001044887.1
B13G0390	2.85	2.78	PBLD	Phenazine biosynthesis-like domain-containing protein	NM_001020523.1
A23G0514	2.85	3.06	IKZF4	Zinc finger protein Eos	XM_009296869.4
B24G0193	2.85	4.59	COASY	Bifunctional coenzyme A synthase	NM_001004577.1
B6G0333	2.85	6.35	SLC19A2	Thiamine transporter 1	XM_005168735.5
A11G0645	2.85	4.54	HES5	Transcription factor HES-5	XM_001919300.7
A3G0149	2.85	8.39	PRR36	Proline-rich protein 36	XM_021472648.2
A17G0029	2.85	6.02	TGFB3	Transforming growth factor beta-3 proprotein	NM_194386.2
B7G0017	2.85	1.78	LURAP1L	Leucine rich adaptor protein 1-like	
A6G0338	2.85	4.34	MICALL1	MICAL-like protein 1	XM_001922140.7

A9G0209	2.85	3.39	TGFBR2	TGF-beta receptor type-2	XM_683846.6
B24G0142	2.85	1.82	METRN	Meteorin	NM_001009983.2
B3G1632	2.85	1.29	G5714_002766	A0A7J6D7N0_9TELE Uncharacterized protein	XM_068213808.1
B13G0417	2.85	2.99	PGBD5	PiggyBac transposable element-derived protein 5	XM_680019.6
A22G0192	2.85	2.75	LOC113092156	A0A6P6NYJ6_CARAU NACHT, LRR and PYD domains-containing protein 3-like isoform X1	XM_068216562.1
A25G0385	2.85	1.49	CBLL1	E3 ubiquitin-protein ligase Hakai	XM_005163040.5
B23G0840	2.85	1.74	SARG	Specifically androgen-regulated gene protein	XM_005162415.5
A20G0659	2.85	5.71	CDC42BPB	threonine-protein kinase MRCK beta	XM_021468493.2
B14G0136	2.85	4.80	MID2	Probable E3 ubiquitin-protein ligase MID2	XM_009291075.4
B5G0441	2.85	5.04	SCIMP	A0A6P6K8H4_CARAU SLP adapter and CSK-interacting membrane protein	
B22G0712	2.85	5.55	TMEM161A	Transmembrane protein 161A	NM_001044317.1
A10G0114	2.85	2.87	MPHOSPH9	M-phase phosphoprotein 9	XM_005172750.5
A1G0793	2.85	7.71	ABCB6	ATP-binding cassette sub-family B member 6	XM_005160290.5
B1G0795	2.85	2.14	SETD4	SET domain-containing protein 4	NM_001045562.1
B13G0076	2.85	3.07	PCSK2	Neuroendocrine convertase 2	XM_001921133.5
A4G0247	2.84	1.47	STXB	Neoverrucotoxin subunit beta	XM_005158613.5
A18G0300	2.84	2.71	TBRG1	Transforming growth factor beta regulator 1	XR_011007164.1
A3G0886	2.84	12.45	ATP6V0A1	V-type proton ATPase 116 kDa subunit a 1	NM_212672.1
B20G0507	2.84	3.16	E2F6	Transcription factor E2F6	XM_005160725.5
A7G0346	2.84	1.51	NAGK	N-acetyl-D-glucosamine kinase	XM_068222131.1
B15G0304	2.84	20.43	DIABLO	Diablo IAP-binding mitochondrial protein	
B22G0257	2.84	1.49	G5714_021341	A0A7J6BS79_9TELE Ig-like domain-containing protein	XM_068216610.1
B7G1226	2.84	7.50	PACS1	Phosphofurin acidic cluster sorting protein 1	XM_005172169.5
B8G0678	2.84	3.78	FCHO1	F-BAR domain only protein 1	NM_001423857.1
A11G0779	2.84	1.48	TMBIM6	Probable Bax inhibitor 1	XM_021479924.2
A7G1049	2.84	3.23	FOCAD	Focadhesin	NM_001423838.1
B8G0293	2.84	3.58	SLC15A4	Solute carrier family 15 member 4	NM_001320529.1
B3G0689	2.84	1.87	COIL	Coilin	NM_131512.2
A10G0206	2.84	2.55	MORN3	MORN repeat-containing protein 3	NM_001017666.1
B7G0207	2.84	5.27	NEO1	Neogenin	NM_173218.2
A4G0101	2.84	2.33	LOC113047446	A0A6P6JXS5_CARAU gastrula zinc finger protein XlCGF57.1-like isoform X1	XM_068220573.1
B6G0121	2.84	2.74	MTIF2	Translation initiation factor IF-2, mitochondrial	NM_001089324.1
B6G0687	2.84	0.54	WAS	Actin nucleation-promoting factor WAS	XM_001919081.7
B20G0155	2.84	8.80	SLC30A1	Zinc transporter 1	NM_200879.1
B1G1114	2.84	3.16	GIMAP9	GTPase IMAP family member 9	XM_021478109.1
B7G0861	2.84	1.89	P100/11E	11E PE=1 SV=3	NM_001006056.2
B6G0258	2.84	7.37	BAZ2B	Bromodomain adjacent to zinc finger domain protein 2B	XM_017356582.3

B3G0513	2.84	14.47	KANK2	KN motif and ankyrin repeat domain-containing protein 2	NM_001305620.1
A7G1180	2.84	4.67	CERS2	Ceramide synthase 2	XM_002662744.7
B9G0849	2.84	3.72	ATF2	Cyclic AMP-dependent transcription factor ATF-2	XM_009304424.4
B24G0669	2.84	5.38	SELENOO1	Protein adenylyltransferase SelO-1, mitochondrial	
B25G0455	2.84	3.62	LOC113043302	A0A6P6JD52_CARAU rho guanine nucleotide exchange factor 18-like isoform X1	NM_001025519.2
B7G1177	2.83	14.82	GVIN1	Interferon-induced very large GTPase 1	XM_005172242.5
B3G0409	2.83	1.73	A33	Zinc-binding protein A33	XR_011010824.1
B21G0957	2.83	2.28	TMEM38B	Trimeric intracellular cation channel type B	
A13G0510	2.83	2.94	VPS26A	Vacuolar protein sorting-associated protein 26A	NM_131521.2
B15G0024	2.83	9.17	SCHIP1	Schwannomin-interacting protein 1	NM_001017906.1
B8G0471	2.83	0.55	INKA2	PAK4-inhibitor INKA2	XM_005167130.5
B8G0579	2.83	3.94	MKRN2	E3 ubiquitin-protein ligase makorin-2	NM_001128336.1
A5G0241	2.83	4.23	AUTS2	Autism susceptibility gene 2 protein homolog	XM_021476418.2
B1G0170	2.83	3.28	CHTF18	Chromosome transmission fidelity protein 18 homolog	NM_001110102.3
A5G0308	2.83	7.55	CDS2	Phosphatidate cytidylyltransferase 2	NM_201186.1
A6G0930	2.83	3.30	CC018	Uncharacterized protein C3orf18 homolog	XM_009302725.4
A21G0351	2.83	1.89	PPP1R3B	Protein phosphatase 1 regulatory subunit 3B	XM_005161147.5
A14G0573	2.83	2.39	AP1AR	AP-1 complex-associated regulatory protein	NM_001301369.1
B25G0134	2.83	4.76	IGKV3D-7	Immunoglobulin kappa variable 3D-7	
A16G0618	2.83	3.48	LOC113059722	A0A6P6LKS0_CARAU zinc finger protein 135-like	NM_001326591.1
A24G0447	2.83	4.90	ARFGEF1	Brefeldin A-inhibited guanine nucleotide-exchange protein 1	XM_068217189.1
B8G0616	2.83	1.91	PORCN	Protein-serine O-palmitoleoyltransferase porcupine	NM_199849.1
B12G0591	2.83	0.93	PATL2	Protein PAT1 homolog 2	XM_678169.8
B3G0843	2.83	26.48	TSPAN4	Tetraspanin-4	NM_001082807.1
B21G0706	2.83	4.06	KIAA1328	Protein hinderin	NM_001083836.1
B16G0386	2.83	8.02			XM_068213918.1
B5G0573	2.83	2.78	PPEF2	threonine-protein phosphatase with EF-hands 2	XM_009301816.4
B20G0594	2.83	2.97	SWT1	Transcriptional protein SWT1	XM_009294798.4
A4G0453	2.83	6.80	SLC37A3	Sugar phosphate exchanger 3	XM_005174588.4
A15G0266	2.83	8.39	N4BP2L2	NEDD4-binding protein 2-like 2	XM_688657.9
A8G0503	2.83	8.33	PREX1	Phosphatidylinositol 3,4,5-trisphosphate-dependent Rac exchanger 1 protein	XM_694535.10
A14G0020	2.83	5.92	TRIM16	Tripartite motif-containing protein 16	XM_681071.8
B17G0531	2.83	5.33	HEATR5A	HEAT repeat-containing protein 5A	XM_009293122.2
A1G0196	2.83	4.28	PI4K2A	Phosphatidylinositol 4-kinase type 2-alpha	NM_213358.2

B13G0568	2.83	2.04	ARHGAP22	Rho GTPase-activating protein 22	XM_678562.8
B5G1275	2.83	3.94	FAM214B	Protein FAM214B	NM_001433130.1
B5G1193	2.83	1.12	BTN1A1	Butyrophilin subfamily 1 member A1	XM_021471582.2
B9G0565	2.82	1.67	UGGT2	UDP-glucose:glycoprotein glucosyltransferase 2	XM_009304772.4
B25G0628	2.82	0.00	CCL13	C-C motif chemokine 13	
B15G0495	2.82	7.28	ZC3H4	Zinc finger CCCH domain-containing protein 4	XM_005157605.5
B19G0610	2.82	2.30	NTAQ1	Protein N-terminal glutamine amidohydrolase	NM_001044947.1
A15G0581	2.82	3.08	TUBD1	Tubulin delta chain	NM_001002093.1
B24G0614	2.82	4.45	PXDC1	PX domain-containing protein 1	XM_689621.5
A13G0215	2.82	10.31	DCAF5	DDB1- and CUL4-associated factor 5	XM_005156618.5
A21G0309	2.82	16.95	GFI1B	Zinc finger protein Gfi-1b	XM_005161133.5
A7G0196	2.82	11.01	CYB5A	Cytochrome b5	NM_212876.1
A8G0224	2.82	1.95	TRAF2	TNF receptor-associated factor 2	NM_001128381.1
A13G0214	2.82	1.95	PACS2	Phosphofurin acidic cluster sorting protein 2	XM_005156684.5
B5G1023	2.82	3.39	NCAPH	Condensin complex subunit 2	NM_001080196.1
A17G0873	2.82	3.75	CDC42EP3	Cdc42 effector protein 3	
B6G0593	2.82	6.24	UAP1	UDP-N-acetylhexosamine pyrophosphorylase	NM_001365262.1
A1G0405	2.82	0.75	NF7O	Nuclear factor 7, ovary	XM_686415.9
A1G0587	2.82	1.64	DHRSX	reductase SDR family member on chromosome X	NM_001256719.1
A3G1083	2.82	31.16	HMOX	Heme oxygenase	NM_001127516.1
B20G0339	2.82	2.57	SYNE2	Nesprin-2	NM_001365330.1
B9G0024	2.82	1.73	DPX16_0232	A0A3N0Z4H4_ANAGA HECT domain-containing protein	
B5G0375	2.82	7.46	SLC25A46	Mitochondrial outer membrane protein SLC25A46	NM_001002558.1
B16G0311	2.82	4.09	GPD1L	Glycerol-3-phosphate dehydrogenase 1-like protein	NM_001005934.2
A17G0310	2.82	1.72	HAUS2	HAUS augmin-like complex subunit 2	
B1G0848	2.82	0.17	OTX1A	Homeobox protein OTX1 A	
A14G0103	2.82	36.26	PDE6A	Rod cGMP-specific 3',5'-cyclic phosphodiesterase subunit alpha	NM_001007160.2
B14G0077	2.82	0.28	A0A6G9W2S3	A0A6G9W2S3_CYPCA Interleukin-5 family A	
B1G0464	2.82	3.25	POGLUT1	Protein O-glucosyltransferase 1	NM_001037434.2
B16G0137	2.82	4.85	MIOS	GATOR complex protein MIOS	NM_201333.1
A21G0549	2.82	7.77	ESRRG	Estrogen-related receptor gamma	XM_009295473.4
A14G0481	2.82	9.93	NFKB1	Nuclear factor NF-kappa-B p105 subunit	XM_021481269.2
B10G0521	2.81	1.17	RXFP2	Relaxin receptor 2	XM_068223827.1
A20G0187	2.81	9.46	FKBP1B	Peptidyl-prolyl cis-trans isomerase FKBP1B	NM_200812.2
B23G0934	2.81	1.76	DDX58	Antiviral innate immune response receptor RIG-I	
A4G0500	2.81	8.04	LAMB1	Laminin subunit beta-1	NM_001277130.1
A17G0674	2.81	3.94	BRMS1LA	Breast cancer metastasis-suppressor 1-like protein-A	NM_001030102.2
A8G0345	2.81	8.43	RGL1	Ral guanine nucleotide dissociation stimulator-like 1	NM_001320403.1
A20G0805	2.81	3.86	EXOC8	Exocyst complex component 8	NM_001110475.1
A25G0330	2.81	4.42	ZGC:103499	Uncharacterized protein C12orf29 homolog	NM_001008606.1

B8G0067	2.81	2.01	MTOR	threonine-protein kinase mTOR	NM_001077211.3
B25G0004	2.81	4.39	PTPRB	Receptor-type tyrosine-protein phosphatase beta	XM_068217725.1
B7G0500	2.81	1.67	ORC6	Origin recognition complex subunit 6	NM_212671.2
A6G0150	2.81	2.96	PALM3	Paralemmin-3	NM_001080085.1
A24G0661	2.81	3.56	G5714_023720	A0A7J6BM18_9TELE Uncharacterized protein	XM_009297216.4
B20G0409	2.81	1.29	SLC35F6	Solute carrier family 35 member F6	NM_001002559.1
B22G0016	2.81	3.12	CASPA	Caspase a	NM_001013502.1
B7G0732	2.81	1.87	POLR2M	DNA-directed RNA polymerase II subunit GRINL1A	NM_001346180.1
B22G0013	2.81	11.68	USP47	Ubiquitin carboxyl-terminal hydrolase 47	
A2G0487	2.81	2.85	OXSR1	threonine-protein kinase OSR1	NM_001098747.1
B5G0525	2.81	5.62	WDR5	WD repeat-containing protein 5	
B17G0671	2.81	5.44	MTA1	Metastasis-associated protein MTA1	XM_001333237.8
B6G0246	2.81	8.48	ATG9A	Autophagy-related protein 9A	XM_068221601.1
A7G0138	2.81	1.25	PACA	Glucagon family neuropeptides	NM_001328496.1
A13G0469	2.81	3.31	CFAP43	Cilia- and flagella-associated protein 43	NM_001126432.1
A4G0912	2.81	3.02	ZKSCAN2	Zinc finger protein with KRAB and SCAN domains 2	NM_001013331.3
A3G0518	2.81	1.60	CCDC97	Coiled-coil domain-containing protein 97	XM_001333080.7
A7G1145	2.81	1.11	ANKDD1A	Ankyrin repeat and death domain-containing protein 1A	XM_068222363.1
B6G0204	2.81	6.18	CYP27C1	Cytochrome P450 27C1	NM_001113337.3
A15G0466	2.81	1.87	LOC113115067	A0A6P6QY42_CARAU zinc finger protein 708-like isoform X1	NM_001423242.1
A5G0225	2.81	24.86	HSPB8	Heat shock protein beta-8	NM_001100957.2
A2G0948	2.81	21.51	TRIM16	Tripartite motif-containing protein 16	XM_068218040.1
A3G0456	2.81	0.75	MFSD11	UNC93-like protein MFSD11	
A7G1112	2.81	2.68	ELP4	Elongator complex protein 4	NM_001017638.1
B5G0950	2.80	0.80	TRIM16	Tripartite motif-containing protein 16	NM_001326487.1
B21G0800	2.80	5.33	MOB1B	MOB kinase activator 1B	NM_214783.2
B25G0505	2.80	3.83	BORCS5	BLOC-1-related complex subunit 5	NM_001007052.2
A24G0145	2.80	2.42	DNAAF3	Dynein axonemal assembly factor 3	XM_068217254.1
A11G0152	2.80	6.31	PLA2R1	Secretory phospholipase A2 receptor	XM_068216952.1
A16G0342	2.80	2.10	PAQR7B	Membrane progestin receptor alpha-B	XM_005158330.5
A2G1103	2.80	11.48	TGM1	Protein-glutamine gamma-glutamyltransferase K	XM_689858.8
A11G0417	2.80	20.29	PHF2	Lysine-specific demethylase phf2	NM_200189.2
A14G0658	2.80	4.75	RAD9A	Cell cycle checkpoint control protein RAD9A	NM_200207.2
B16G0863	2.80	4.98	NBEAL2	Neurobeachin-like protein 2	XM_021472976.2
B16G0232	2.80	6.32	MTX1	Metaxin-1	NM_001128238.1
A3G0759	2.80	6.12	SMG1	threonine-protein kinase SMG1	XM_009299453.4
B13G0584	2.80	3.76	SIX1A	Homeobox protein six1a	NM_001003532.1
A1G0512	2.80	6.27	GIMAP7	GTPase IMAP family member 7	NM_001076574.1
A16G0687	2.80	3.27	MDC1	Mediator of DNA damage checkpoint protein 1	NM_001365561.1

A19G0535	2.80	0.57	A0A671M6A8	A0A671M6A8_9TELE Uncharacterized protein	
B9G0262	2.80	3.02	SEC22A	Vesicle-trafficking protein SEC22a	XM_021478609.2
B14G0430	2.80	5.06	TAPT1	Transmembrane anterior posterior transformation protein 1 homolog	XM_009291277.4
B13G0383	2.80	9.73	LOC107743564	A0A673GHN3_9TELE Gamma-synuclein	XM_682449.7
A13G0346	2.80	0.72	DRGX	Dorsal root ganglia homeobox protein	NM_131280.2
A1G0011	2.80	3.40	NLRP1	NACHT, LRR and PYD domains-containing protein 1 homolog	
B8G0254	2.80	2.70	GPAT4	Glycerol-3-phosphate acyltransferase 4	NM_001040249.2
A1G0537	2.80	7.31	FBXO8	F-box only protein 8	NM_001040307.1
B24G0126	2.80	3.29	IGKV1-39	Immunoglobulin kappa variable 1-39	NM_001045408.1
B20G0045	2.80	3.29	THEMIS	Protein THEMIS	NM_001346242.1
A1G0367	2.80	7.49	SPRYD7	SPRY domain-containing protein 7	NM_214776.1
B22G0774	2.79	27.67	TMEM59L	Transmembrane protein 59-like	NM_213337.2
A2G0045	2.79	2.56	TRIM16	Tripartite motif-containing protein 16	XM_005161933.5
B9G0713	2.79	4.57	TCB1	Transposable element Tcb1 transposase	XM_021471168.2
B14G0076	2.79	0.80	A0A672MX55	A0A672MX55_SINGR Interleukin 4	
B2G0420	2.79	2.41	ARHGEF18	Rho guanine nucleotide exchange factor 18	NM_214818.1
A15G0458	2.79	10.43	ZC3H4	Zinc finger CCCH domain-containing protein 4	XM_005157605.5
A3G0426	2.79	2.57	SNRNP25	U12 small nuclear ribonucleoprotein 25 kDa protein	NM_001017690.1
B8G0693	2.79	0.35	COLGALT2	Procollagen galactosyltransferase 2	NM_001130076.1
B20G0646	2.79	2.33	PTRHD1	Putative peptidyl-tRNA hydrolase PTRHD1	
B6G1042	2.79	6.23	CASK	Peripheral plasma membrane protein CASK	NM_001142376.1
A13G0666	2.79	3.84	PDZD7	PDZ domain-containing protein 7	NM_001290348.1
A3G0700	2.79	24.87	LOC113045623	A0A6P6JRL5_CARAU mucin-5AC-like	
B1G0235	2.79	1.38	B4GALT1	Beta-1,4-galactosyltransferase 1	NM_001045332.2
B5G1078	2.79	9.31	CI085	Uncharacterized protein C9orf85 homolog	NM_001004001.2
B9G0782	2.79	1.17	C3ORF38	Uncharacterized protein C3orf38	XM_002663346.7
B13G0649	2.79	4.80	THAP4	Peroxynitrite isomerase THAP4	NM_001080606.2
B6G0054	2.79	2.39	LOC107744762	A0A673G9P7_9TELE Uncharacterized LOC107744762	NM_001098259.2
B7G1255	2.79	0.34	MRC2	C-type mannose receptor 2	XM_068222099.1
A19G0187	2.79	1.44	PARD6G	Partitioning defective 6 homolog gamma	NM_212563.1
B5G0915	2.79	3.24	ZMAT5	Zinc finger matrin-type protein 5	NM_001003771.1
A5G0475	2.79	5.40	ADAM28	Disintegrin and metalloproteinase domain-containing protein 28	

A20G0710	2.79	1.22	METTL13	eEF1A lysine and N-terminal methyltransferase	NM_001044769.4
B18G0515	2.79	3.18	PPCDC	Phosphopantothenoylcysteine decarboxylase	NM_200780.2
B21G0795	2.79	48.52	PLAUR	Urokinase plasminogen activator surface receptor	XM_068216177.1
B19G0086	2.79	3.14	HFE	Hereditary hemochromatosis protein homolog	
A1G0681	2.79	4.52	CEP97	Centrosomal protein of 97 kDa	
A24G0269	2.79	1.74	CCL11	Eotaxin	
A17G0469	2.79	8.57	AFTPH	Aftiphilin	XM_021467414.2
B3G1609	2.79	1.82	DPX16_3453	A0A3N0XKN3_ANAGA Zinc finger protein 569	XM_068220573.1
A9G0326	2.79	3.40	PLCL1	Inactive phospholipase C-like protein 1	XM_001921763.8
B18G0565	2.79	2.78	WEE1-B	Wee1-like protein kinase 1-B	NM_001309435.1
B3G0605	2.79	2.76	TOP3A	DNA topoisomerase 3-alpha	XM_683603.10
B2G0876	2.79	10.11	CSNK1G2	Casein kinase I isoform gamma-2	NM_001024379.1
A1G0852	2.79	0.55	GNG13	G(O) subunit gamma-13	
B9G0429	2.79	4.33	ACOD1	Cis-aconitate decarboxylase	NM_001126456.2
A21G0099	2.79	2.86	NOTCH1A	Neurogenic locus notch homolog protein 1	NM_131441.1
B9G0820	2.79	3.80	STK24	threonine-protein kinase 24	NM_001110451.1
B16G0159	2.79	3.60	RPZ	Protein rapunzel	
B10G0495	2.79	4.16	CUX1	Homeobox protein cut-like 1	XM_005172719.5
B11G0631	2.79	9.89	BSN	Protein bassoon	NM_001083570.1
B14G0399	2.79	14.57	PLAAT3	Phospholipase A and acyltransferase 3	NM_001002623.1
B4G0486	2.79	2.90	CBLL1	E3 ubiquitin-protein ligase Hakai	NM_001309368.1
B6G0017	2.79	2.51	GIMAP8	GTPase IMAP family member 8	
A1G0883	2.79	5.71	MPC2	Mitochondrial pyruvate carrier 2	NM_001004662.3
B4G0723	2.78	0.37	NF7O	Nuclear factor 7, ovary	NM_001089399.2
B18G0545	2.78	8.41	KIAA0513	Uncharacterized protein KIAA0513	NM_001002193.1
B10G0069	2.78	4.76	SNX30	Sorting nexin-30	NM_001017798.1
A13G0266	2.78	4.00	TRIM25	ISG15 ligase TRIM25	
A1G0563	2.78	8.93	GAB1	GRB2-associated-binding protein 1	NM_001326713.1
B19G0345	2.78	2.41	LOC107748696	A0A673M1Y3_9TELE Uncharacterized LOC107748696	
B10G0732	2.78	13.98	SCARB1	Scavenger receptor class B member 1	XM_017357940.3
B12G0606	2.78	7.82	TOP2A	DNA topoisomerase 2-alpha	NM_001310015.1
B12G0597	2.78	7.32	PLEKHA1	Pleckstrin homology domain-containing family A member 1	XM_003199578.6
A5G0931	2.78	3.79	SLC2A8	Solute carrier family 2, facilitated glucose transporter member 8	NM_212798.1
A23G0312	2.78	29.74	BCL2L1	Bcl-2-like protein 1	NM_131807.1
B1G0957	2.78	2.01	LOC113097617	A0A6P6PBN1_CARAU oocyte zinc finger protein XlCOF6-like isoform X2	NM_001327886.1
A3G1125	2.78	2.70	EPN3	Epsin-3	XM_021471467.2
B13G0223	2.78	2.35	KCNMA1	Calcium-activated potassium channel subunit alpha-1	NM_001002084.1
B17G0237	2.78	3.30	JKAMP	MAPK8-associated membrane protein	NM_200482.2
A16G0207	2.78	2.65	DBF4	Protein DBF4 homolog A	NM_001135135.1

A17G0653	2.78	3.94	E1301_TTI002949	A0A5A9NNB8_9TELE Apoptosis-stimulating of p53 protein 1	NM_001204257.1
B3G1016	2.78	1.71	PPP4CA	threonine-protein phosphatase 4 catalytic subunit A	NM_001110414.1
B5G0569	2.78	3.63	ACSBG2	Long-chain-fatty-acid--CoA ligase ACSBG2	
B22G0888	2.78	0.00	VTCN1	V-set domain-containing T-cell activation inhibitor 1	
A23G0273	2.78	5.81	SRMS	Tyrosine-protein kinase Srms	NM_001136250.2
A3G0653	2.78	3.38	MUCM	Ig mu chain C region membrane-bound form	XM_068218290.1
A12G0349	2.78	8.05	NDEL1A	Nuclear distribution protein nudE-like 1-A	NM_201344.1
B12G0491	2.78	4.86	MEIOB	Meiosis-specific with OB domain-containing protein	XM_021472512.2
B25G0298	2.78	2.16	ATP8B2	Phospholipid-transporting ATPase ID	XM_682623.8
A18G0395	2.78	11.43	MEF2A	Myocyte-specific enhancer factor 2A	XM_005159044.5
B12G0680	2.78	3.54	SGMS1	Phosphatidylcholine:ceramide cholinephosphotransferase 1	NM_001077614.2
A1G1089	2.78	0.95	FHIP1A	FHF complex subunit HOOK interacting protein 1A	XM_021473904.2
A5G0105	2.77	10.60	ME2	NAD-dependent malic enzyme, mitochondrial	NM_001003627.2
A16G1007	2.77	1.90	ADCY3	Adenylate cyclase type 3	XM_009292011.4
A10G0491	2.77	2.74	ZNHIT2	Zinc finger HIT domain-containing protein 2	NM_001077618.1
B16G0628	2.77	3.79	TBC1D31	TBC1 domain family member 31	XM_689914.7
B22G1007	2.77	0.00	LOC113039796	A0A6P6J0X4_CARAU uncharacterized protein LOC113039796 isoform X2	
A17G0692	2.77	1.94	ADPGK	ADP-dependent glucokinase	XM_689665.9
A24G0290	2.77	5.04	PLD1	Phospholipase D1	XM_021470348.2
A8G0248	2.77	3.34	G5714_009459	A0A7J6CSC7_9TELE Uncharacterized protein	
A24G0158	2.77	7.19	GRN	Progranulin	XM_021470114.2
A9G0548	2.77	8.85	XPO4	Exportin-4	NM_212674.2
A7G1283	2.77	8.37	GIMAP4	GTPase IMAP family member 4	XM_068222510.1
A16G0696	2.77	7.21	NECTIN2	Nectin-2	XM_021466855.2
A10G0672	2.77	13.00	DEPDC5	GATOR complex protein DEPDC5	XM_068223938.1
B6G1007	2.77	17.64	HELZ2	Helicase with zinc finger domain 2	XM_003198847.5
B13G0515	2.77	1.77	NT5C2	Cytosolic purine 5'-nucleotidase	NM_001017773.1
B4G0923	2.77	9.44	TTLL1	Polyglutamylase complex subunit TTLL1	NM_001320519.1
B17G0187	2.77	1.81	MIPOL1	Mirror-image polydactyly gene 1 protein	NM_001305467.1
A14G0450	2.77	3.69	NSD1	Histone-lysine N-methyltransferase, H3 lysine-36 specific	XM_678798.9
B15G0415	2.77	11.56	EXOC3L2	Exocyst complex component 3-like protein 2	XM_021472941.2
A20G0250	2.77	2.30	HDDC2	5'-deoxynucleotidase HDDC2	NM_001045231.1
A13G0559	2.77	3.03	FAM204A	Protein FAM204A	XM_017358831.3
A7G0643	2.77	2.12	DPEP2	Dipeptidase 2	XM_003198973.6
A9G0210	2.77	5.43	OSBPL11	Oxysterol-binding protein-related protein 11	XM_683921.8

A6G0883	2.77	2.57	PIGU	Phosphatidylinositol glycan anchor biosynthesis class U protein	NM_001184955.1
A6G1029	2.77	10.38	USP9X	Probable ubiquitin carboxyl-terminal hydrolase FAF-X	XM_009304756.4
A11G0017	2.77	12.75	MAFK	Transcription factor MafK	NM_201287.1
B16G0976	2.76	8.10	ZG16	Zymogen granule membrane protein 16	
B16G0077	2.76	0.55	KLF10	Krueppel-like factor 10	NM_001386438.1
A20G0610	2.76	2.57	JAG2	Protein jagged-2	NM_131862.1
A10G0017	2.76	4.81	EMSY	BRCA2-interacting transcriptional repressor EMSY	NM_199984.2
B6G0334	2.76	2.86	ZBED1	E3 SUMO-protein ligase ZBED1	XM_068221765.1
B22G0940	2.76	16.60	BBIP1	BBSome-interacting protein 1	NM_001166123.1
B13G0332	2.76	2.93	LRPPRC	Leucine-rich PPR motif-containing protein, mitochondrial	XM_686496.7
A11G0460	2.76	3.67	SCML2	Sex comb on midleg-like protein 2	XM_002663788.7
A1G0441	2.76	2.75	LOC113070356	A0A6P6MU08_CARAU uncharacterized protein LOC113070356	
B2G0236	2.76	2.05	NLRP1	NACHT, LRR and PYD domains-containing protein 1 homolog	NM_001423861.1
A3G0842	2.76	9.01	CLEC16A	Protein CLEC16A	XM_005163916.5
B1G0487	2.76	0.31	CCKAR	Cholecystokinin receptor	NM_001114714.2
B1G1058	2.76	4.35	CD300LD3	CMRF35-like molecule 3	
A1G0754	2.76	2.07	LOC113098471	A0A6P6PG22_CARAU T-cell surface antigen CD2-like	
B5G0825	2.76	6.73	NAIF1	Nuclear apoptosis-inducing factor 1	NM_001077271.1
A6G0370	2.76	4.42	RHBDF2	Inactive rhomboid protein 2	
A9G0353	2.76	0.89	KLF5	Krueppel-like factor 5	XM_683433.10
A1G0480	2.76	13.90	G5714_000595	A0A7J6DIC0_9TELE Uncharacterized protein	
B19G0376	2.76	20.49	GSDME	Gasdermin-E	
B3G0412	2.76	4.41	CBR1	Carbonyl reductase [NADPH] 1	NM_001083559.1
A21G0294	2.76	3.27	MZT2	Mitotic-spindle organizing protein 2	
A18G0114	2.76	3.52	PRDM10	PR domain zinc finger protein 10	NM_001347687.1
A16G0660	2.76	0.30	S100A1	Protein S100-A1	NM_001013495.3
A2G1071	2.76	1.54	SLAMF9	SLAM family member 9	XM_068214760.1
A8G0060	2.76	0.27			
B7G0940	2.76	4.97	CLCN5	Cl(-) exchange transporter 5	XM_680670.7
B8G0353	2.76	9.81	RASSF2	Ras association domain-containing protein 2	XM_005169091.5
A8G0024	2.76	3.66	CEP120	Centrosomal protein of 120 kDa	XM_068223073.1
B15G0011	2.76	3.47	LIMK1	LIM domain kinase 1	NM_001042691.2
B4G0344	2.76	8.95	CAND1	Cullin-associated NEDD8-dissociated protein 1	NM_213485.1
A17G0232	2.75	2.17	TMEM62	Transmembrane protein 62	XM_001923726.7
A4G0567	2.75	4.01	GSAP	Gamma-secretase-activating protein	XM_021475259.2
A16G0932	2.75	0.36	TNFRSF11B	Tumor necrosis factor receptor superfamily member 11B	
A3G0373	2.75	5.54	DSN1	Kinetochore-associated protein DSN1 homolog	NM_001386492.1
A20G0879	2.75	4.33	LATS1	threonine-protein kinase LATS1	XM_005160312.5

A9G0135	2.75	2.40	DNAJC10	DnaJ homolog subfamily C member 10	NM_001083547.1
A18G0506	2.75	3.47	BBS2	Bardet-Biedl syndrome 2 protein homolog	NM_152887.1
B3G0714	2.75	5.67	THAP1	THAP domain-containing protein 1	NM_001083569.1
A13G0586	2.75	2.55	DCTN1	Dynactin subunit 1	NM_001006100.1
A10G0086	2.75	0.94	HIC2	Hypermethylated in cancer 2 protein	NM_182869.2
A1G0564	2.75	4.74	USP38	Ubiquitin carboxyl-terminal hydrolase 38	XM_003197674.6
A8G0511	2.75	5.20	PPP1R3A	Protein phosphatase 1 regulatory subunit 3A	
A25G0366	2.75	3.54	PPIP5K1	Inositol hexakisphosphate and diphosphoinositol-pentakisphosphate kinase 1	XM_021473848.2
B4G0558	2.75	3.06	SLC35E3	Solute carrier family 35 member E3	NM_001030179.1
A9G0132	2.75	34.16	VIL1	Villin-1	XM_005167920.5
A23G0452	2.75	6.30	KLHL21	Kelch-like protein 21	NM_001320407.1
A7G0442	2.75	4.01	TTC17	Tetratricopeptide repeat protein 17	NM_001423800.1
B6G0817	2.75	2.97	CENPS	Centromere protein S	NM_001128749.1
A16G0590	2.75	3.55	TYROBP	TYRO protein tyrosine kinase-binding protein	XM_009292219.2
A12G0300	2.75	3.42	BEND6	BEN domain-containing protein 6	XM_683728.9
B14G0553	2.75	3.63	TMCO6	Transmembrane and coiled-coil domain-containing protein 6	NM_001089537.1
A12G0491	2.75	2.42	LOC107731407	A0A673JTR4_9TELE Rho GTPase-activating protein 17-like	
A19G0902	2.75	3.87	CYP51	Lanosterol 14-alpha demethylase	NM_001001730.2
B3G0527	2.75	7.05	MRTFB	Myocardin-related transcription factor B	XM_009299741.4
A14G0289	2.75	3.11	ADRB2	Beta-2 adrenergic receptor	NM_001102652.1
B25G0050	2.75	12.54	AMFR	E3 ubiquitin-protein ligase AMFR	NM_213163.2
A12G0613	2.75	1.85	G5714_004412	A0A7J6D4L6_9TELE Uncharacterized protein	
A21G0078	2.75	6.25	SCAMP1	Secretory carrier-associated membrane protein 1	NM_205692.1
A17G0376	2.74	2.49	PTAFR	Platelet-activating factor receptor	XM_001342336.6
A21G0742	2.74	3.38	MNT	Max-binding protein MNT	NM_001103111.1
A8G0251	2.74	7.28	DEDD	Death effector domain-containing protein	NM_001002639.1
A12G0334	2.74	10.67	SRCAP	Helicase SRCAP	XM_005156310.5
A3G0808	2.74	2.05	FAM83F	Protein FAM83F	XM_690066.10
B20G0383	2.74	2.51	BACH2	Transcription regulator protein BACH2	XM_005160548.5
B5G1017	2.74	3.83	TESK2	Dual specificity testis-specific protein kinase 2	NM_001089574.2
A5G1040	2.74	2.43	MIS12	Protein MIS12 homolog	NM_200378.1
B12G0369	2.74	2.72	SOCS5	Suppressor of cytokine signaling 5	NM_001113758.2
B2G0415	2.74	0.42	TRIM16	Tripartite motif-containing protein 16	XM_685228.7
A25G0550	2.74	7.37	ISLR2	Immunoglobulin superfamily containing leucine-rich repeat protein 2	NM_001013523.1

A15G0475	2.74	2.69	RNF26	E3 ubiquitin-protein ligase RNF26	XM_005157594.5
B11G0145	2.74	2.88	SPCS1	Signal peptidase complex subunit 1	XM_068224182.1
A7G0384	2.74	20.83	PER1	Period circadian protein homolog 1	NM_212439.3
B7G1184	2.74	0.95	CLDN7A	Claudin-7-A	NM_001002340.1
A15G0802	2.74	3.01	MAML2	Mastermind-like protein 2	XM_002667380.7
A23G0482	2.74	18.64	GDI1	Rab GDP dissociation inhibitor alpha	NM_001020608.2
B9G0706	2.74	2.99	TCB1	Transposable element Tcb1 transposase	XM_021471168.2
B17G0410	2.74	5.33	SGMS1	Phosphatidylcholine:ceramide cholinephosphotransferase 1	NM_001007325.2
A1G0445	2.74	2.97	LOC107674213	A0A671S8I0_9TELE Zinc finger protein 578-like	XM_005160085.5
A1G0039	2.74	1.45	BCAR3	Breast cancer anti-estrogen resistance protein 3 homolog	XM_002665063.7
B12G0194	2.74	2.48	MFSD13A	Transmembrane protein 180	XM_017358451.3
A15G0170	2.74	8.07	CD22	B-cell receptor CD22	NM_001098245.1
B1G0272	2.74	7.26	DLC1	Rho GTPase-activating protein 7	XM_017355991.3
A23G0813	2.74	2.11	GVIN1	Interferon-induced very large GTPase 1	XM_005174153.3
B14G0468	2.74	4.75	TSPAN17	Tetraspanin-17	NM_001123045.2
B16G0605	2.74	2.35	IRGC	Interferon-inducible GTPase 5	
A1G0743	2.74	0.53	LOC113094186	A0A6P6P3Y7_CARAU uncharacterized protein LOC113094186	
A12G0177	2.74	3.77	LOC113079881	A0A6P6NFW5_CARAU uncharacterized protein LOC113056002	NM_001278598.1
B21G0092	2.74	1.93	CSF1R1	Macrophage colony-stimulating factor 1 receptor 1	XM_009295703.4
B7G1151	2.74	12.18	CHD3	Chromodomain-helicase-DNA-binding protein 3	XM_005172235.5
B3G0407	2.74	7.56	DNAJB1	DnaJ homolog subfamily B member 1	NM_001003571.1
A16G0103	2.73	2.65	SATB1	DNA-binding protein SATB1	XM_017351562.3
B8G0564	2.73	19.38	MMP9	Matrix metalloproteinase-9	NM_213123.1
B24G0117	2.73	11.24	FCGR1A	High affinity immunoglobulin gamma Fc receptor I	
A14G0785	2.73	5.81	RRAGA	Ras-related GTP-binding protein A	NM_199714.1
B16G0450	2.73	1.57	G5714_015935	A0A7J6CB65_9TELE Uncharacterized protein	
B9G0847	2.73	4.15	LNPKA	Endoplasmic reticulum junction formation protein lunapark-A	NM_199480.1
B20G0109	2.73	12.00	SPTLC2	Serine palmitoyltransferase 2	NM_001114741.1
B25G0297	2.73	2.10	GALK2	N-acetylgalactosamine kinase	NM_001007432.2
B7G1049	2.73	1.40	G5714_008500	A0A7J6CVU5_9TELE Uncharacterized protein	XM_017356910.2
A2G0470	2.73	3.54	PLCD1	1-phosphatidylinositol 4,5-bisphosphate phosphodiesterase delta-1	NM_200068.1
A15G0116	2.73	6.68	LOC113058706	A0A6P6LDJ1_CARAU uncharacterized protein LOC113058706	XM_021466547.2
B23G0633	2.73	2.26	NLRC3	Protein NLRC3	XM_068216925.1
A2G0444	2.73	3.83	THAP4	Peroxynitrite isomerase THAP4	
A3G0190	2.73	2.67	FOXK2	Forkhead box protein K2	XM_001922821.8
A2G0871	2.73	3.15	AP1G1	AP-1 complex subunit gamma-1	NM_001289858.1

A16G0245	2.73	6.65	ATP2C1	Calcium-transporting ATPase type 2C member 1	XM_005158254.5
A25G0151	2.73	3.50	PRDM11	PR domain-containing protein 11	XM_688756.10
A25G0486	2.73	2.72	ZNF800	Zinc finger protein 800	NM_001324549.1
B4G0173	2.73	4.16	PGM3	Phosphoacetylglucosamine mutase	NM_001007053.1
A16G0456	2.73	0.46	LYSMD1	LysM and putative peptidoglycan-binding domain-containing protein 1	NM_001076750.1
A4G0925	2.73	5.02	RASSF5	Ras association domain-containing protein 5	XM_005164499.5
B13G0516	2.73	5.43	INA	Alpha-internexin	NM_001105681.2
A10G0041	2.73	3.33	NUP155	Nuclear pore complex protein Nup155	NM_200156.1
A17G0150	2.73	3.93	ZFYVE28	Lateral signaling target protein 2 homolog	NM_001077723.1
B18G0453	2.73	5.36	EDC4	Enhancer of mRNA-decapping protein 4	NM_001045085.1
B16G0002	2.73	4.27	OSR2	Protein odd-skipped-related 2	XM_005173555.5
A18G0494	2.73	6.74	PEDS1	Plasmanylethanolamine desaturase	XM_005159087.5
B19G0314	2.73	6.68	PIP5K1A	Phosphatidylinositol 4-phosphate 5-kinase type-1 alpha	NM_001020602.1
A24G0465	2.72	4.88	ZFY1	Zinc finger Y-chromosomal protein 1	XM_021470300.2
B1G0854	2.72	11.52	SRD5A2	3-oxo-5-alpha-steroid 4-dehydrogenase 2	NM_001017703.1
A16G0079	2.72	2.80	DHDDS	Dehydrodolichyl diphosphate synthase complex subunit DHDDS	NM_213187.1
B24G0224	2.72	5.42	ATG9A	Autophagy-related protein 9A	NM_001320078.1
B4G0372	2.72	5.27	TWF1	Twinfilin-1	NM_001002304.1
A15G0460	2.72	2.63	ERCC2	General transcription and DNA repair factor IIH helicase subunit XPD	NM_200926.2
B23G0670	2.72	7.74	ITGA5	Integrin alpha-5	NM_001004288.2
B8G0754	2.72	2.30	PDE4D	cAMP-specific 3',5'-cyclic phosphodiesterase 4D	XM_021474146.2
A14G0227	2.72	3.00	INPPL1B	Phosphatidylinositol 3,4,5-trisphosphate 5-phosphatase 2B	NM_001039135.2
B22G0489	2.72	0.66	LOC113077509	A0A6P6NAU4_CARAU gastrula zinc finger protein XlCGF57.1-like	XM_068220236.1
A16G0391	2.72	1.28	CALHM5.1	Calcium homeostasis modulator protein 5	XM_021466947.2
A10G0828	2.72	2.14	MAT2A	S-adenosylmethionine synthase isoform type-2	NM_001040379.1
B18G0780	2.72	8.80	PAK1	threonine-protein kinase PAK 1	XM_021467598.2
B12G0467	2.72	4.86	SNX29	Sorting nexin-29	XM_688793.9
B17G0017	2.72	3.50	KLHL28	Kelch-like protein 28	XM_017351727.3
A8G0917	2.72	2.41	CLCN6	Cl(-) exchange transporter 6	XM_009304277.4
B7G0381	2.72	1.14	HDAC8	Histone deacetylase 8	NM_213431.1
B18G0121	2.72	1.17			
A19G0740	2.72	1.38	CLEC4E	C-type lectin domain family 4 member E	NM_001044742.1
B4G0352	2.72	0.47	G5714_004946	A0A7J6D637_9TELE Uncharacterized protein	
A7G0999	2.72	5.25	BCR	Breakpoint cluster region protein	
B7G0943	2.72	3.47	RBM4.1	RNA-binding protein 4.1	NM_199705.1
A14G0207	2.72	4.24	INTS6	Integrator complex subunit 6	NM_200434.2

A1G1034	2.72	6.10	RAPGEF2	Rap guanine nucleotide exchange factor 2	XM_005170931.5
B25G0262	2.72	5.99	BANP	Protein BANP	NM_001020632.1
A2G0506	2.72	32.87	PLVAP	Plasmalemma vesicle-associated protein	NM_001114577.1
B17G0496	2.72	3.05	NRDE2	Nuclear exosome regulator NRDE2	XM_005158556.5
B23G0218	2.72	4.50	A0A3B1IUE0	A0A3B1IUE0_ASTMX SWIM-type domain-containing protein	XR_660182.4
A7G0180	2.72	9.61	AP1G1	AP-1 complex subunit gamma-1	XM_068222168.1
B3G0829	2.72	4.95	TBC1D10B	TBC1 domain family member 10B	NM_001365256.1
B11G0162	2.72	0.00	CTDSPL2	CTD small phosphatase-like protein 2	NM_001040359.1
B23G0440	2.72	7.45	SAMD11	Sterile alpha motif domain-containing protein 11	XM_009296907.4
B18G0039	2.72	8.63	ST3GAL4	CMP-N-acetylneuraminate-beta-galactosamide-alpha-2,3-sialyltransferase 4	NM_001083029.1
B8G0635	2.71	8.76	SI:CH211-147A11.3	UPF0688 protein C1orf174 homolog	XM_005166902.4
B5G0561	2.71	4.12	LOC107720253	A0A673M4G5_9TELE Selenocysteine insertion sequence-binding protein 2-like	XM_068221566.1
B13G0897	2.71	2.02			XM_021475709.2
B7G0095	2.71	0.99			
A19G0261	2.71	2.11	SYNC	Syncoilin	XM_003200615.6
B16G0607	2.71	1.85	CBL	E3 ubiquitin-protein ligase CBL	XM_021466724.2
A2G0434	2.71	3.25	MLLT1	Protein ENL	XM_068224918.1
B20G0128	2.71	3.12	CNKSR3	Connector enhancer of kinase suppressor of ras 3	NM_001113647.1
A9G0704	2.71	1.71	SLC37A1	Glucose-6-phosphate exchanger SLC37A1	XM_021478645.2
A5G1262	2.71	12.69	RTASE	Probable RNA-directed DNA polymerase from transposon BS	XM_068220297.1
A14G0031	2.71	1.37	YTX2	Transposon TX1 uncharacterized 149 kDa protein	
A10G0511	2.71	7.88	SYNJ1	Synaptojanin-1	NM_001007030.2
B24G0674	2.71	3.46	PCOLCE2	Procollagen C-endopeptidase enhancer 2	NM_200460.1
A13G0864	2.71	4.64	HSP30	Heat shock protein 30	XM_001334404.7
A9G0148	2.71	5.11	CWC22	Pre-mRNA-splicing factor CWC22 homolog	NM_001077569.2
A20G0572	2.71	1.49	LOC107737357	A0A673HZI6_9TELE Polymeric immunoglobulin receptor-like	
A5G0695	2.71	5.11	LRRN4	Leucine-rich repeat neuronal protein 4	XM_068221462.1
B15G0813	2.71	9.34	TRIM25	ISG15 ligase TRIM25	NM_001007387.2
A1G0749	2.71	4.76	RCAN1	Calcipressin-1	XM_021470802.2
B4G0476	2.71	4.57	CDKN1B	Cyclin-dependent kinase inhibitor 1B	NM_001013268.1
A8G0172	2.71	3.72	USP11	Ubiquitin carboxyl-terminal hydrolase 11	XM_002663073.7
A20G0252	2.71	4.09	PEX7	Peroxisomal targeting signal 2 receptor	XR_011007674.1
B22G1158	2.71	2.38	G5714_021497	A0A7J6BR43_9TELE Uncharacterized protein	XM_021469564.2
A18G0635	2.71	5.83	FRMD4A	FERM domain-containing protein 4A	XM_017351947.3
A23G0866	2.71	4.18	PSIP1	PC4 and SFRS1-interacting protein	XM_021470062.2

B3G0812	2.71	6.11	RARAB	Retinoic acid receptor alpha-B	NM_131399.1
B23G0800	2.71	5.32	TMCO4	Transmembrane and coiled-coil domain-containing protein 4	XM_003201227.6
A7G0670	2.71	0.26	LOC107664376	A0A671RCZ2_9TELE Mucin-15-like	XM_021471992.2
A20G0584	2.71	1.43	OCIAD2	OCIA domain-containing protein 2	NM_001044856.2
B14G0304	2.70	3.40	SYTL4	Synaptotagmin-like protein 4	XM_009291117.4
A14G0288	2.70	4.93	SH3TC2	SH3 domain and tetratricopeptide repeat-containing protein 2	XM_068213382.1
A8G0421	2.70	2.77	PORCN	Protein-serine O-palmitoleoyltransferase porcupine	NM_199849.1
A12G0734	2.70	3.82	PLEKHM1	Pleckstrin homology domain-containing family M member 1	NM_001327851.1
A17G0739	2.70	1.68	TONSL	Tonsoku-like protein	NM_001111148.1
A3G0360	2.70	7.06	SLC27A1	Long-chain fatty acid transport protein 1	NM_001013537.1
B18G0569	2.70	14.97	RNF141	RING finger protein 141	NM_001007290.2
B10G0216	2.70	8.57	RNF208	RING finger protein 208	XM_003199320.6
A17G0365	2.70	3.81	AP4S1	AP-4 complex subunit sigma-1	
B9G0005	2.70	12.23	PARP14	Protein mono-ADP-ribosyltransferase PARP14	XM_009305033.4
B15G0854	2.70	2.30	CHORDC1	Cysteine and histidine-rich domain-containing protein 1	NM_001025175.1
B3G1178	2.70	5.33	EP300	Histone acetyltransferase p300	XM_009299413.4
B4G0716	2.70	2.38	A0A673GQ01	A0A673GQ01_9TELE Uncharacterized protein	XM_017355716.3
A13G0767	2.70	6.12	LRRC73	Leucine-rich repeat-containing protein 73	XM_005169532.5
B11G0347	2.70	1.08	OGFR	Opioid growth factor receptor	NM_001002741.2
A14G0038	2.70	4.71	RNF217	E3 ubiquitin-protein ligase RNF217	
B15G0489	2.70	4.17	MARK4	microtubule affinity-regulating kinase 4	XM_005157600.5
B25G0729	2.70	5.19	PEAK1	Inactive tyrosine-protein kinase PEAK1	XM_001921567.7
A7G0891	2.70	3.36	RGP1	RAB6A-GEF complex partner protein 2	NM_001002608.1
A25G0597	2.70	10.24	IGF2	Insulin-like growth factor II	NM_001001815.1
B21G0684	2.70	8.64	NPDC1	Neural proliferation differentiation and control protein 1	
A25G0175	2.70	2.09	ACCS	1-aminocyclopropane-1-carboxylate synthase-like protein 1	NM_001014323.4
A16G0523	2.70	2.10	ALG2	1,6-mannosyltransferase ALG2	NM_001104936.2
A11G0609	2.70	0.61	PHF13	PHD finger protein 13	
A8G0875	2.70	6.88	GFPT1	Glutamine--fructose-6-phosphate aminotransferase [isomerizing] 1	NM_001034981.1
A23G0352	2.70	1.99	LAMB1	Laminin subunit beta-1	XM_021473631.2
A21G0904	2.70	5.16	GALNT10	Polypeptide N-acetylgalactosaminyltransferase 10	XM_009298402.3
A1G0514	2.70	2.28	G5714_000629	A0A7J6DGV8_9TELE C3H1-type domain-containing protein	XM_021471222.2
A11G0633	2.69	1.42	MUC17	Mucin-17	NM_001362557.1
B23G0028	2.69	4.88	DIDO1	Death-inducer obliterator 1	XM_003201155.6
B7G0597	2.69	5.74	GPATCH1	G patch domain-containing protein 1	NM_001204123.1

A22G0521	2.69	3.66	P3H2	Prolyl 3-hydroxylase 2	XM_021474475.2
B14G0260	2.69	10.54	XPNPEP2	Xaa-Pro aminopeptidase 2	NM_201032.2
A16G0593	2.69	1.99	LOC113076523	A0A6P6N9X2_CARAU zinc finger and BTB domain-containing protein 41-like	XM_068214132.1
B16G0478	2.69	7.05	MROH1	Maestro heat-like repeat-containing protein family member 1	XM_068214158.1
B20G0260	2.69	2.30	MARVELD3	MARVEL domain-containing protein 3	NM_001171033.2
B5G0074	2.69	1.85	PIGW	Phosphatidylinositol-glycan biosynthesis class W protein	NM_001114572.2
A19G0298	2.69	2.76	HMBOX1	Homeobox-containing protein 1	XM_009294314.4
A13G0697	2.69	7.01	KCNMA1	Calcium-activated potassium channel subunit alpha-1 (Fragment)	
B12G0654	2.69	4.01	NRBF2	Nuclear receptor-binding factor 2	NM_001002613.1
B14G0037	2.69	1.72	TLR6	Toll-like receptor 6	NM_001130593.1
B3G1186	2.69	2.54	SLC25A17	Peroxisomal membrane protein PMP34	NM_001099261.2
B12G0425	2.69	1.83	ARSG	Arylsulfatase G	NM_001365361.1
B8G0650	2.69	3.71	CPT2	Carnitine O-palmitoyltransferase 2, mitochondrial	NM_001007447.2
B16G0091	2.69	3.17	AGO1	Protein argonaute-1	NM_001317223.1
B2G0163	2.69	4.11	ZCCHC2	Zinc finger CCHC domain-containing protein 2	XM_021471307.2
B4G0410	2.69	12.82	ATP2B1	Plasma membrane calcium-transporting ATPase 1	XM_009300256.4
A1G0007	2.69	12.11	ITGAL	Integrin alpha-L	
B5G0563	2.69	2.33	PRLR	Prolactin receptor	NM_001113500.1
A16G0984	2.69	12.69	RTASE	Probable RNA-directed DNA polymerase from transposon BS	
B22G0670	2.69	12.70	LOC113040113	A0A6P6J8J3_CARAU PDZK1-interacting protein 1-like	
B14G0038	2.69	6.36	INTS10	Integrator complex subunit 10	XM_068213163.1
A15G0674	2.69	15.02	SLC1A5	Neutral amino acid transporter B(0)	NM_001190755.1
B18G0819	2.69	7.24	FAM219B	Protein FAM219B	NM_001014356.2
A4G0974	2.69	0.21			
A5G0212	2.69	4.48	TET3-A	Methylcytosine dioxygenase tet3-A	NM_001327945.1
B9G0679	2.68	16.32	DNAJB2	DnaJ homolog subfamily B member 2	NM_001079993.1
B20G0883	2.68	3.13	RNF8	E3 ubiquitin-protein ligase rnf8	NM_205553.1
A1G0502	2.68	3.54	SCOCA	Short coiled-coil protein A	NM_001077559.1
B1G1149	2.68	1.47	STXB	Stonustoxin subunit beta	XM_068222150.1
B9G0248	2.68	4.90	CYP27A1	Sterol 26-hydroxylase, mitochondrial	XM_002663399.6
B4G0098	2.68	3.80	PLEKHA5	Pleckstrin homology domain-containing family A member 5	XM_021475079.2
A5G0314	2.68	7.44	SMARCA5	SNF-related matrix-associated actin-dependent regulator of chromatin subfamily A member 5	XM_005165157.5
A8G0418	2.68	7.38	APEX2	DNA-(apurinic or apyrimidinic site) endonuclease 2	
A14G0551	2.68	13.24	STX5	Syntaxin-5	NM_001002333.1
A5G0070	2.68	1.54	PIGW	Phosphatidylinositol-glycan biosynthesis class W protein	NM_001114572.2
A7G1392	2.68	9.08	BTN1A1	Butyrophilin subfamily 1 member A1	NM_001423720.1

B17G0680	2.68	2.61	MBIP	MAP3K12-binding inhibitory protein 1	NM_001256179.1
B17G0647	2.68	4.13	RNF144AA	Probable E3 ubiquitin-protein ligase RNF144A-A	XM_005158736.5
A16G0352	2.68	6.72	TENT5A	Terminal nucleotidyltransferase 5A	NM_001077806.1
B11G0311	2.68	3.34	QRICH1	Transcriptional regulator QRICH1	
A18G0596	2.68	1.23			
B18G0728	2.68	0.80	B4GALNT3	Beta-1,4-N-acetylgalactosaminyltransferase 3	XM_001342847.9
B23G0796	2.68	5.47	PPP1R14C	Protein phosphatase 1 regulatory subunit 14C	NM_001029963.1
B8G0340	2.68	1.13	TMIGD2	Transmembrane and immunoglobulin domain-containing protein 2	
A4G0262	2.68	3.17	G5714_002600	A0A7J6D7F9_9TELE Uncharacterized protein	
A25G0295	2.68	3.13	CECR2	Chromatin remodeling regulator CECR2	XM_002666820.6
A19G0138	2.68	2.01	SLC66A2	Solute carrier family 66 member 2	XM_021468289.2
B21G0049	2.68	10.99	DPYSL3	Dihydropyrimidinase-related protein 3	XM_068215866.1
B25G0745	2.68	9.33	RBPMS2A	RNA-binding protein, mRNA-processing factor 2a	NM_001002409.1
B3G0126	2.68	0.58	LOC113096980	A0A6P6PBC3_CARAU zinc finger protein 271-like isoform X1	XM_017356396.3
A2G0565	2.68	4.17	RNF31	E3 ubiquitin-protein ligase RNF31	XM_690941.10
B3G1020	2.68	4.58	YPEL3	Protein yippee-like 3	NM_212790.1
B7G1021	2.68	2.62	RCN1	Reticulocalbin-1	XM_686046.9
A9G0382	2.68	2.05	TRIM39	E3 ubiquitin-protein ligase TRIM39	
A2G0804	2.68	1.17	RABGAP1L	Rab GTPase-activating protein 1-like, isoform 10	
A14G0085	2.68	11.86	KIF3A	Kinesin-like protein KIF3A	NM_001017604.2
B19G0781	2.68	1.08	CLEC4F	C-type lectin domain family 4 member F	XM_068218583.1
B15G0324	2.68	6.72	SYNRG	Synergin gamma	XM_021472920.2
B23G0093	2.68	9.07	GGT7	Glutathione hydrolase 7	XM_068216828.1
A5G0507	2.68	2.66	NELFB	Negative elongation factor B	NM_200168.1
B20G0596	2.68	2.29	TRMT1L	TRMT1-like protein	XM_682939.8
B23G0725	2.68	11.19	NFE2L2	Nuclear factor erythroid 2-related factor 2	XM_068216670.1
B22G0568	2.68	3.56	MGAT5	Alpha-1,6-mannosylglycoprotein 6-beta-N-acetylglucosaminyltransferase A	NM_001045311.1
B12G0133	2.68	3.14	ZBED1	E3 SUMO-protein ligase ZBED1	
B4G0644	2.68	8.27	CDKN1A	Cyclin-dependent kinase inhibitor 1	
B1G0996	2.68	0.94	CD22	B-cell receptor CD22	XM_017356663.3
B8G0860	2.68	7.90	NRAS	GTPase NRas	NM_131145.2
A9G0625	2.67	0.40	FER1L6	Fer-1-like protein 6	XM_068223565.1
B7G1166	2.67	11.35	KDM6B	Lysine-specific demethylase 6B	XM_021477780.2
A25G0302	2.67	3.60	CL004	Protein C12orf4 homolog	NM_001077789.1
A17G0314	2.67	3.91	RPAP1	RNA polymerase II-associated protein 1	XM_021473203.2

B24G0247	2.67	7.33	YME1L1	ATP-dependent zinc metalloprotease YME1L1	XM_005162861.5
B10G0723	2.67	5.01	ACADS	Short-chain specific acyl-CoA dehydrogenase, mitochondrial	NM_001003743.1
A6G0188	2.67	8.21	CYP27C1	Cytochrome P450 27C1	NM_001113337.3
B16G0017	2.67	7.67	DENND4B	DENN domain-containing protein 4B	NM_001423202.1
B11G0016	2.67	7.72			XM_002663842.6
A5G0561	2.67	12.48	LOC113074953	A0A6P6N379_CARAU uncharacterized protein LOC113074953	
B22G0035	2.67	10.92	JOCKEY\POL	RNA-directed DNA polymerase from mobile element jockey	XR_011011165.1
B5G0864	2.67	9.04	AIF1L	Allograft inflammatory factor 1-like	NM_198870.1
B19G0090	2.67	3.17	EHMT2	Histone-lysine N-methyltransferase EHMT2	NM_001113615.1
A15G0166	2.67	3.79	RHOG	Rho-related GTP-binding protein RhoG	
A20G0500	2.67	8.71	SEL1L	Protein sel-1 homolog 1	NM_001045164.1
B4G0489	2.67	2.78	MCAT	Malonyl-CoA-acyl carrier protein transacylase, mitochondrial	NM_001045043.1
B1G0088	2.67	0.58	SLITRK6	SLIT and NTRK-like protein 6	NM_001305553.1
B4G0212	2.67	1.45	CCDC3	Coiled-coil domain-containing protein 3	NM_001025510.1
B20G0331	2.67	2.49	XRCC3	DNA repair protein XRCC3	NM_001013541.1
A15G0440	2.67	1.90	TAPBPL	Tapasin-related protein	NM_001083830.1
A16G0905	2.67	2.01	TRAK1	Trafficking kinesin-binding protein 1	XM_068214077.1
A9G0794	2.67	3.10	GPR155	Integral membrane protein GPR155	XM_009304429.4
B7G0643	2.67	1.95	LTB4R	Leukotriene B4 receptor 1	NM_001328706.1
A19G0651	2.67	0.39	TMEM238	Transmembrane protein 238	NM_001083074.2
A21G0057	2.67	3.59	RIC1	Guanine nucleotide exchange factor subunit RIC1	XM_068217721.1
B1G0505	2.67	3.17	UBAC2	Ubiquitin-associated domain-containing protein 2	NM_001080561.2
A13G0854	2.67	3.85	TCB1	Transposable element Tcb1 transposase	
A1G0262	2.67	2.54	ACY3.2	N-acyl-aromatic-L-amino acid amidohydrolase (carboxylate-forming) B	NM_001002347.1
A20G0865	2.67	3.93	SENP6	Sentrin-specific protease 6	XM_021468561.2
B13G0137	2.67	5.59	SYNDIG1	Synapse differentiation-inducing gene protein 1	NM_001020757.2
A1G0210	2.67	1.98	NTHL1	Endonuclease III-like protein 1	XM_001346607.8
A5G0026	2.67	14.36	GOLGA2	Golgin subfamily A member 2	XM_009301121.4
A17G0037	2.67	0.88	BMP4	Bone morphogenetic protein 4	NM_131342.2
A16G0999	2.66	4.54	MECR	Enoyl-[acyl-carrier-protein] reductase, mitochondrial	NM_001017852.1
B5G0387	2.66	2.69	PPM1D	Protein phosphatase 1D	NM_001007340.1
A22G0584	2.66	6.60	SODA	Superoxide dismutase [Cu-Zn] 1	XM_001343614.8
B21G0112	2.66	6.36	NR3C1	Glucocorticoid receptor	NM_001020711.3
A6G0239	2.66	30.63	COL5A2	Collagen alpha-2(V) chain	XM_001919970.8
B17G0701	2.66	7.04	ASPG	L-asparaginase	XM_021467087.2
A12G0703	2.66	6.62	AARSD1	Alanyl-tRNA editing protein Aarsd1	NM_001003572.1
B12G0761	2.66	13.69	LOC107595630	A0A672LXB6_SINGR Uncharacterized LOC107595630	

A10G0701	2.66	2.13	NFKBIL1	NF-kappa-B inhibitor-like protein 1	XM_005155451.5
A3G0148	2.66	8.33	PKN1	threonine-protein kinase N1	NM_001089460.1
A1G0400	2.66	4.87	SENS	Zinc finger protein sens	XM_021477008.2
B10G0792	2.66	7.96	NAA25	N-alpha-acetyltransferase 25, NatB auxiliary subunit	XM_005172598.5
A18G0359	2.66	5.83	LOC113061635	A0A6P6LSE4_CARAU uncharacterized protein LOC113061635	NM_001083062.2
B18G0742	2.66	2.66	WDR59	GATOR complex protein WDR59	XM_009293384.4
B16G0567	2.66	6.94	STX17	Syntaxin-17	NM_001007449.2
B24G0699	2.66	7.09	BDH1	D-beta-hydroxybutyrate dehydrogenase, mitochondrial	NM_001089509.1
A4G0071	2.66	1.82	A0A672NKX7	A0A672NKX7_SINGR Uncharacterized protein	XM_017355605.3
B9G0604	2.66	1.08	GJA5	Gap junction alpha-5 protein	NM_001007213.1
A1G0860	2.66	6.70	MAD1L1	Mitotic spindle assembly checkpoint protein MAD1	NM_200912.2
A12G0680	2.66	3.22	ADO	2-aminoethanethiol dioxygenase	NM_001008634.1
B20G0865	2.66	2.73	INTS9	Integrator complex subunit 9	NM_001077270.1
B25G0793	2.66	4.29	CHID1	Chitinase domain-containing protein 1	NM_200057.1
A16G0443	2.66	4.34	PTK2	Focal adhesion kinase 1	XM_009292411.4
A15G0497	2.66	11.65	SIK2	threonine-protein kinase SIK2	XM_691233.9
A16G0205	2.66	6.03	WASHC5	WASH complex subunit 5	NM_200183.1
A17G0621	2.66	1.72	MANEA	Glycoprotein endo-alpha-1,2-mannosidase	NM_001004566.2
A16G0667	2.66	4.30	CA043	Protein C1orf43 homolog	
A25G0059	2.66	6.84	THOC5	THO complex subunit 5 homolog	NM_212692.1
B12G0730	2.66	3.29	MAPK7	Mitogen-activated protein kinase 7	XM_005156002.5
A1G0387	2.66	6.46	SERHL	Serine hydrolase-like protein	NM_199615.2
A25G0471	2.66	10.14	AASS	Alpha-aminoadipic semialdehyde synthase, mitochondrial	NM_001173985.1
A2G0673	2.66	6.88	NRBP2	Nuclear receptor-binding protein 2	XM_001920637.7
A21G0417	2.66	3.92	CHRDL2	Chordin-like protein 2	NM_001130959.2
A7G0545	2.66	1.35	A0A3B1KLL1	A0A3B1KLL1_ASTMX Uncharacterized protein	
B2G0037	2.66	4.50	A0A674ACG0	A0A674ACG0_SALTR Uncharacterized protein	XM_001920546.6
A15G0835	2.65	6.98	DOCK10	Dedicator of cytokinesis protein 10	XM_068213668.1
A1G0877	2.65	2.08	TMEM55BB	Type I phosphatidylinositol 4,5-bisphosphate 4-phosphatase-B	NM_001159921.1
A2G0094	2.65	0.44	WNT3A	Protein Wnt-3a	XM_005163660.5
B22G0690	2.65	7.02	IFI44L	Interferon-induced protein 44-like	XM_003201036.6
B5G0470	2.65	4.31	ORAI2	Protein orai-2	XM_005165570.5
A15G0444	2.65	4.38	PHF12	PHD finger protein 12	XM_021466429.2
A7G1171	2.65	3.20	CEMIP	Cell migration-inducing and hyaluronan-binding protein	XM_021477713.2
A4G1016	2.65	3.69	LRIG3	Leucine-rich repeats and immunoglobulin-like domains protein 3	NM_001110347.1
A10G0741	2.65	7.74	RABGAP1	Rab GTPase-activating protein 1	XM_017358005.3
B12G0526	2.65	10.84	LIPA	cholesteryl ester hydrolase	NM_213404.1
A14G0295	2.65	1.93	RNASEH2C	Ribonuclease H2 subunit C	NM_001161377.1

A10G0840	2.65	2.96	NFIL3	Nuclear factor interleukin-3-regulated protein	
A23G0068	2.65	7.46	PLCG1	1-phosphatidylinositol 4,5-bisphosphate phosphodiesterase gamma-1	NM_194407.1
B12G0739	2.65	10.07	FOXK2	Forkhead box protein K2	XM_001922821.8
A19G0623	2.65	7.00	DDX6	Probable ATP-dependent RNA helicase DDX6	XM_001340824.8
B8G0823	2.65	2.36	TRIM39	E3 ubiquitin-protein ligase TRIM39	NM_001130790.1
B21G0777	2.65	3.06	WDR41	WD repeat-containing protein 41	NM_001030261.2
A15G0847	2.65	4.71	RABGEF1	GTP exchange factor	NM_200941.1
A22G0564	2.65	0.78	CAPN2	Calpain-2 catalytic subunit	NM_001244080.1
A21G0708	2.65	2.27	ARID5B	AT-rich interactive domain-containing protein 5B	NM_205565.2
B22G0141	2.65	4.16	TBXA2R	Thromboxane A2 receptor	NM_001285535.1
B24G0524	2.65	5.38	ZFY1	Zinc finger Y-chromosomal protein 1	XM_021470300.2
A5G0601	2.65	4.93	LMO4-B	LIM domain transcription factor LMO4-B	XM_068221084.1
A12G0350	2.65	17.82	ITGB3	Integrin beta-3	NM_001082948.1
B25G0619	2.65	3.57	CCL2	C-C motif chemokine 2	
A12G0373	2.65	2.48	PIGF	Phosphatidylinositol-glycan biosynthesis class F protein	NM_205645.1
A8G0541	2.65	3.87	AMT	Aminomethyltransferase, mitochondrial	NM_001006021.1
A23G0535	2.65	11.86	HUWE1	E3 ubiquitin-protein ligase HUWE1	NM_001365654.1
A6G1000	2.64	2.18	ZEB1	Zinc finger E-box-binding homeobox 1	
A12G0102	2.64	3.25	DOCK1	Dedicator of cytokinesis protein 1	NM_001110011.1
B20G0163	2.64	6.39	BPNT1	3'(2'),5'-bisphosphate nucleotidase 1	NM_001002354.1
B2G0424	2.64	1.62	PHC3	Polyhomeotic-like protein 3	XM_009298654.4
B12G0811	2.64	2.25	TMEM101	Transmembrane protein 101	NM_001327885.1
A12G0384	2.64	2.91	ASB3	Ankyrin repeat and SOCS box protein 3	NM_199874.1
B19G0855	2.64	4.56	LRP12	Low-density lipoprotein receptor-related protein 12	XM_005159560.4
B2G0830	2.64	1.96	VIPR1	Vasoactive intestinal polypeptide receptor	XM_002660797.5
A10G0521	2.64	0.77	POSTN	Periostin	XM_002663548.6
B25G0422	2.64	7.48	ERGIC2	Endoplasmic reticulum-Golgi intermediate compartment protein 2	NM_200407.1
A17G0720	2.64	2.84	LOC107733912	A0A673MQN2_9TELE Uncharacterized LOC107733912	NM_001020545.1
B23G0465	2.64	5.35	ATP13A2	Polyamine-transporting ATPase 13A2	XM_005162183.5
B20G0663	2.64	3.23	BATF	Basic leucine zipper transcriptional factor ATF-like	NM_001045392.1
A24G0283	2.64	3.66	LARP4B	La-related protein 4B	XM_021470200.2
A25G0384	2.64	5.49	FAM3C	Protein FAM3C	XM_005163005.5
B6G0950	2.64	6.10	NUDT3	Diphosphoinositol polyphosphate phosphohydrolase 1	NM_001006034.2
B23G0342	2.64	6.22	FAM110B	Protein FAM110B	XM_009296762.4
B3G1751	2.64	0.79	GHR	Growth hormone receptor	

B3G1504	2.64	0.01	LOC113040506	A0A6P6J346_CARAU uncharacterized protein LOC113040506	
B7G0534	2.64	2.62	NCAPG2	Condensin-2 complex subunit G2	NM_001089492.3
A15G0522	2.64	4.32	DLC	Delta-like protein C	NM_130944.1
B2G0133	2.64	4.33	PIGR	Polymeric immunoglobulin receptor	XM_005163603.5
A8G0958	2.64	1.73	IL17RB	Interleukin-17 receptor B	
B23G0442	2.64	2.63	KLHL17	Kelch-like protein 17	XM_021473682.2
B5G0357	2.64	7.87	CCNB1	mitotic-specific cyclin-B1	NM_131513.1
A11G0681	2.64	9.88	TMEM167B	Protein kish-B	NM_001083570.1
A6G0640	2.63	5.08	SLC48A1B	Heme transporter hrg1-A	NM_200006.1
A14G0128	2.63	3.76	GPR137	Integral membrane protein GPR137	NM_001002691.1
A8G0714	2.63	2.55	ROHU_028827	A0A498LH15_LABRO Uncharacterized protein	
B25G0440	2.63	0.89	IL17RA	Interleukin-17 receptor A	
A5G0271	2.63	4.86	FAM222A	Protein FAM222A	XM_068221316.1
B1G0330	2.63	2.21	FNIP2	Folliculin-interacting protein 2	XM_068220861.1
B19G0744	2.63	3.05	ZBTB12	Zinc finger and BTB domain-containing protein 12	NM_001044884.1
B6G0311	2.63	8.68	INPP4A	Inositol polyphosphate-4-phosphatase type I A	XM_068221803.1
B8G0441	2.63	97.00	H1-5	Histone H1.5	
B9G0223	2.63	7.47	ORMDL1	ORM1-like protein 1	NM_200093.1
B2G0950	2.63	3.17	CDC20	Cell division cycle protein 20 homolog	NM_213080.2
A3G0393	2.63	5.99	TRIP10	Cdc42-interacting protein 4 homolog	XM_017354074.3
A8G0405	2.63	0.19	B3GNT7	UDP-GlcNAc:betaGal beta-1,3-N-acetylglucosaminyltransferase 7	NM_214777.1
B2G0632	2.63	9.14	RANBP9	Ran-binding protein 9	NM_001080571.2
A5G1241	2.63	1.43	EDF1	Endothelial differentiation-related factor 1 homolog	
A23G0855	2.63	3.17	PRELID1	PRELI domain-containing protein 1, mitochondrial	XM_017353568.3
A19G0423	2.63	1.90	CFAD	Counting factor associated protein D	NM_001017633.2
B16G0175	2.63	6.60	SMG5	Nonsense-mediated mRNA decay factor SMG5	NM_001024923.1
B13G0063	2.63	7.78	ZNF665	Zinc finger protein 665	XM_005169554.5
B15G0103	2.63	6.41	RFFL	E3 ubiquitin-protein ligase rififylin	NM_212896.1
A2G0832	2.63	6.04	MFSD14A	Hippocampus abundant transcript 1 protein	NM_199584.1
A3G0490	2.63	2.56	AP5Z1	AP-5 complex subunit zeta-1	XM_017353076.3
B11G0520	2.63	8.39			NM_001080079.1
B20G0415	2.63	2.90	MACO1	Macoilin	XM_021473364.2
A17G0097	2.63	11.76	MOCS1	Molybdenum cofactor biosynthesis protein 1	NM_001105660.2
A5G0732	2.63	7.12	PLD2	Phospholipase D2	XM_021471642.2
A5G0092	2.63	2.54	RABL6	Rab-like protein 6	XM_068221396.1
B21G0629	2.63	2.63	GTF3C4	General transcription factor 3C polypeptide 4	XM_021473407.2
A22G0311	2.63	0.94	SULT3A1	Amine sulfotransferase	NM_001079947.1
A3G0153	2.63	2.87	UBN2	Ubinuclein-2	NM_001104940.1
A19G0788	2.62	3.09	LOC107753946	A0A673NFI0_9TELE Myoneurin-like	XM_009293998.4
B12G0821	2.62	2.34	NXPE3	NXPE family member 3	

B6G0010	2.62	1.24	CDKN2D	Cyclin-dependent kinase 4 inhibitor D	
A14G0102	2.62	4.08	PPARGC1B	Peroxisome proliferator-activated receptor gamma coactivator 1-beta	XM_009291061.4
B11G0296	2.62	5.49	TADA3	Transcriptional adapter 3	XM_001923555.8
B7G1073	2.62	5.68	CD248	Endosialin	NM_001099228.3
B4G0153	2.62	4.37	TIGARB	Fructose-2,6-bisphosphatase TIGAR B	NM_200191.1
A15G0467	2.62	1.77	A0A672QM30	A0A672QM30_SINGR Uncharacterized protein	
A19G0573	2.62	3.45	ADNP2	Activity-dependent neuroprotector homeobox protein 2	XM_005170054.5
B19G0736	2.62	2.71	VARS2	Valine--tRNA ligase, mitochondrial	NM_001130661.1
A1G0356	2.62	0.00	CRYZL1	Quinone oxidoreductase-like protein 1	NM_001002633.1
B19G0472	2.62	3.73	ANKIB1	Ankyrin repeat and IBR domain-containing protein 1	XM_005159781.5
B3G0462	2.62	4.77	ATP13A1	Endoplasmic reticulum transmembrane helix translocase	NM_001001403.2
B7G0377	2.62	7.12	ARHGAP6	Rho GTPase-activating protein 6	NM_001351681.2
B21G0161	2.62	9.50	ALDH3A2	Aldehyde dehydrogenase family 3 member A2	NM_001328218.1
A14G0670	2.62	1.32	LOC107742755	A0A673FK77_9TELE Uncharacterized LOC107742755	NM_001198864.1
B23G0912	2.62	12.90	TRIM16	Tripartite motif-containing protein 16	XM_005174166.5
B5G0661	2.62	4.51	DAPK1	Death-associated protein kinase 1	NM_001099990.2
A21G0502	2.62	10.41	SMS	Spermine synthase	NM_131756.1
B3G1605	2.62	1.40	LOC113100676	A0A6P6PHM3_CARAU zinc finger protein 721-like	XM_068219873.1
B3G1586	2.62	1.36	CD9	CD9 antigen	
A10G0670	2.62	7.10	GNAZ	Guanine nucleotide-binding protein G(z) subunit alpha	XM_005155633.5
A8G0070	2.62	4.33	BLNK	B-cell linker protein	XM_068223080.1
A8G0259	2.62	4.87	SIN3B	Paired amphipathic helix protein Sin3b	NM_001044945.1
B7G0299	2.62	9.40	DENND4C	DENN domain-containing protein 4C	XM_005166553.5
A19G0797	2.62	2.78	LOC113058242	A0A6P6LDP6_CARAU uncharacterized protein LOC113058242	NM_001327796.1
A5G1183	2.62	4.80	PTEN	Phosphatidylinositol 3,4,5-trisphosphate 3-phosphatase and dual-specificity protein phosphatase PTEN	NM_001423688.1
B23G0741	2.62	2.73	HIPK1	Homeodomain-interacting protein kinase 1	XM_009297053.4
A16G0329	2.62	1.53	EVA1B	Protein eva-1 homolog B	NM_001089345.1
B25G0461	2.62	2.40	RNF44	RING finger protein 44	NM_001365354.1
A11G0390	2.62	3.05	KBP	Glutamate receptor U1	XM_009306014.4
B4G0534	2.62	4.01	NAV3	Neuron navigator 3	XM_021475109.2
A21G0898	2.62	6.86	SEC24A	Protein transport protein Sec24A	XM_005170340.5
B12G0162	2.62	13.18	BAIAP2	Brain-specific angiogenesis inhibitor 1-associated protein 2	XM_068219313.1
B16G0794	2.62	1.61	EPHB5	Ephrin type-B receptor 5	XM_005173518.5

A7G0213	2.61	4.37	EIF4G2	Eukaryotic translation initiation factor 4 gamma 2	NM_001014289.2
B1G0858	2.61	3.15	JAG1A	Protein jagged-1a	NM_131861.1
B25G0596	2.61	10.54	CKAP5	Cytoskeleton-associated protein 5	NM_001037667.3
B23G0669	2.61	6.90	RAPGEF3	Rap guanine nucleotide exchange factor 3	XM_005162353.5
A7G0890	2.61	3.45	GBA2	Non-lysosomal glucosylceramidase	XM_005172332.5
B24G0053	2.61	40.96	LOC113042718	A0A6P6JAT4_CARAU uncharacterized protein LOC113042718	XM_068217268.1
A13G0615	2.61	0.83	ADH1	Alcohol dehydrogenase 1	
A15G0099	2.61	8.39	SCG2	Secretogranin-2	
A14G0142	2.61	3.07	MID2	Probable E3 ubiquitin-protein ligase MID2	XM_009291077.4
A18G0048	2.61	7.01	FLI1	Friend leukemia integration 1 transcription factor	XM_021467582.2
A17G0534	2.61	0.38	PLA2G4F	Cytosolic phospholipase A2 zeta	XM_009293030.4
B11G0298	2.61	1.94	SLC25A26	S-adenosylmethionine mitochondrial carrier protein	NM_001020785.2
A8G0787	2.61	3.33	CRAT	Carnitine O-acetyltransferase	NM_001079997.1
A15G0717	2.61	2.47	SLC25A15	Mitochondrial ornithine transporter 1	NM_001080638.1
A25G0602	2.61	9.12	TMEM86A	Lysoplasmalogenase-like protein TMEM86A	NM_001013512.2
B2G0336	2.61	6.17	KEAP1A	Kelch-like ECH-associated protein 1A	NM_182864.2
B6G0556	2.61	0.83	ITGB3BP	Centromere protein R	XM_017356427.3
A20G0039	2.61	4.80	MGAT2	Alpha-1,6-mannosyl-glycoprotein 2-beta-N-acetylglucosaminyltransferase	
B23G0105	2.61	2.67	C1QTNF7	Complement C1q tumor necrosis factor-related protein 7	XM_687939.7
B4G0149	2.61	0.40	GALNT8	Probable polypeptide N-acetylgalactosaminyltransferase 8	XM_003198223.6
B23G0764	2.61	7.76	SOWAHA	Ankyrin repeat domain-containing protein SOWAHA	XM_068216940.1
A15G0647	2.61	0.44	SLC6A13	Sodium- and chloride-dependent GABA transporter 2	NM_001045058.2
B24G0746	2.61	0.00	TMX3	Protein disulfide-isomerase TMX3	NM_001045561.1
B22G1055	2.61	35.21	CNP-4	C-type natriuretic peptide 4	NM_001109940.1
B15G0826	2.61	0.52	SGPP2	Sphingosine-1-phosphate phosphatase 2	NM_001386286.1
B19G0326	2.61	6.61	GRB10	Growth factor receptor-bound protein 10	NM_001004287.2
A2G0732	2.61	0.00	TRAV25	T cell receptor alpha variable 25	
B8G1023	2.61	1.43	PRXL2C	Peroxiredoxin-like 2C	XM_009303700.4
B6G0489	2.61	0.72	RNF152	E3 ubiquitin-protein ligase rnf152	
B5G0916	2.61	3.60	ARPC5L	3 complex subunit 5-like protein	NM_001128686.2
B10G0031	2.61	1.25	SLC38A9	Sodium-coupled neutral amino acid transporter 9	NM_001079999.1
B20G0200	2.61	6.44	BRMS1LA	Breast cancer metastasis-suppressor 1-like protein-A	XM_005160423.5
A12G0357	2.61	2.56	BMPR1A	Bone morphogenetic protein receptor type-1A	NM_001004585.2
A11G0265	2.61	2.06	SFMBT1	Scm-like with four MBT domains protein 1	NM_001102641.1

A24G0152	2.61	1.49	KCNC1	Potassium voltage-gated channel subfamily C member 1	
A4G0429	2.60	1.01	G5714_019313	A0A7J6BW24_9TELE Uncharacterized protein	XM_021475732.2
A10G0201	2.60	5.61	DGCR2	IDD	NM_001002656.2
A12G0599	2.60	1.29	PPP1R16A	Protein phosphatase 1 regulatory subunit 16A	XM_068224737.1
B13G0759	2.60	9.22	RMDN2	Regulator of microtubule dynamics protein 2	XM_005156498.5
A10G0481	2.60	2.18	RNFT1	E3 ubiquitin-protein ligase RNFT1	NM_213228.2
A3G1058	2.60	7.75	MIEF1	Mitochondrial dynamics protein MID51	NM_001083840.1
B7G0483	2.60	2.76	TK2	Thymidine kinase 2, mitochondrial	XM_068222226.1
A5G0604	2.60	2.04	SMARCA2	Probable global transcription activator SNF2L2	NM_001110226.1
B23G0894	2.60	4.76	TRIM39	E3 ubiquitin-protein ligase TRIM39	NM_001040374.2
A13G0055	2.60	5.60	TSNAX	Translin-associated protein X	XM_021480700.2
B6G0183	2.60	35.77	MYH11	Myosin-11	NM_001024448.1
A22G0492	2.60	2.19	DDX59	Probable ATP-dependent RNA helicase DDX59	NM_001044978.2
A19G0267	2.60	12.69	ARL4A	ADP-ribosylation factor-like protein 4A	NM_200318.2
B1G1027	2.60	1.41	ADGRE3	Adhesion G protein-coupled receptor E3	XM_068220943.1
A22G0389	2.60	4.49	BORCS8	BLOC-1-related complex subunit 8	XM_005170457.5
B2G0068	2.60	6.27	PGPEP1	Pyroglutamyl-peptidase 1	NM_001020526.2
A14G0317	2.60	1.80	F2RL3	Proteinase-activated receptor 4	XM_009291214.4
B9G0399	2.60	3.71	HAO2	2-Hydroxyacid oxidase 2	NM_200483.3
B3G0048	2.60	1.81	LOC113075489	A0A6P6N4M8_CARAU zinc finger protein 271-like	NM_001109866.1
A10G0203	2.60	5.29	SETD1BA	Histone-lysine N-methyltransferase SETD1B-A	XM_068223817.1
B7G0245	2.60	1.55	ACSF3	Malonate--CoA ligase ACSF3, mitochondrial	XM_021477983.2
B22G0180	2.60	9.98	NMRK2	Nicotinamide riboside kinase 2	NM_131693.2
A14G0732	2.60	3.58	MGAT4BQ9UQ53	Alpha-1,3-mannosyl-glycoprotein 4-beta-N-acetylglucosaminyltransferase B	NM_001002180.1
B23G0697	2.60	7.72	FBXO25	F-box only protein 25	XM_021469771.2
B22G0945	2.60	18.11	NLRC3	NLR family CARD domain-containing protein 3	XM_068214525.1
B19G0185	2.60	3.03	NEDD9	Enhancer of filamentation 1	NM_001077282.1
B6G0681	2.60	2.70	ENDOUA	Uridylate-specific endoribonuclease A	
B6G0023	2.60	4.06	G5714_006561	A0A7J6CWR1_9TELE HIT domain-containing protein	XM_680842.9
A6G0972	2.60	3.26	NAT8F3	N-acetyltransferase family 8 member 3	
A7G0010	2.60	5.41	E1301_TTI007949	A0A5A9MYK4_9TELE Uncharacterized protein	XM_068217621.1
A5G0524	2.60	3.06	BCO2	Carotenoid-cleaving dioxygenase, mitochondrial	XM_002662014.7
A2G0566	2.59	1.99	CDH7	Cadherin-7	XM_005163402.5
B20G0597	2.59	0.48	NIBAN1	Protein Niban 1	NM_001144791.1
A21G0119	2.59	8.56	SEC16A	Protein transport protein Sec16A	XM_009292712.4
B1G0169	2.59	1.70	ERN1	endoribonuclease IRE1	XM_021471109.2

A22G0274	2.59	12.71	R3HDM1	R3H domain-containing protein 1	XM_021469576.2
A17G0083	2.59	5.79	SCAF8	SR-related and CTD-associated factor 8	NM_001436747.1
B15G0204	2.59	4.13	PER2	Period circadian protein homolog 2	
B21G0186	2.59	3.68	MFF	Mitochondrial fission factor	NM_001386279.1
B6G0826	2.59	5.20	STAU1	Double-stranded RNA-binding protein Staufen homolog 1	XM_005168759.5
B10G0441	2.59	14.38	ALCAM	CD166 antigen homolog	NM_131000.1
B24G0598	2.59	4.66	OTULIN	Ubiquitin thioesterase otulin	XM_001341582.8
A3G0682	2.59	5.47	PRR14L	Protein PRR14L	XM_021473976.2
B21G0454	2.59	4.55	TMEM45B	Transmembrane protein 45B	NM_205677.1
B11G0083	2.59	1.14	G2E3	M phase-specific E3 ubiquitin-protein ligase	
B12G0519	2.59	3.08	RTASE	Probable RNA-directed DNA polymerase from transposon BS	
B7G0891	2.59	7.87	NF7B	Nuclear factor 7, brain	NM_001130628.1
B3G1075	2.59	5.22	CRAMP1	Protein cramped-like	XM_005163839.4
A5G1269	2.59	0.31	PAPPA	Pappalysin-1	NM_001278805.1
A3G0122	2.59	8.36	TEF	Thyrotroph embryonic factor	NM_001020661.2
B3G0034	2.59	0.06	G2E3	M phase-specific E3 ubiquitin-protein ligase	XM_021470292.2
B4G0482	2.59	5.62	DEF6	Differentially expressed in FDCP 6 homolog	XM_687050.7
B20G0077	2.59	1.69	FRK	Tyrosine-protein kinase FRK	XM_021468553.2
A2G0570	2.59	1.85	TOE1	Target of EGR1 protein 1	NM_001256682.1
A7G0571	2.58	2.46	PTPRJ	Receptor-type tyrosine-protein phosphatase eta	NM_001136242.1
A2G0839	2.58	2.09	METTL17	Methyltransferase-like protein 17, mitochondrial	XM_021471253.2
B1G0548	2.58	2.32	NSUN3	tRNA (cytosine(34)-C(5))-methyltransferase, mitochondrial	NM_001431082.1
A1G0156	2.58	3.74	PRKACA	cAMP-dependent protein kinase catalytic subunit alpha	NM_001003470.1
A2G1028	2.58	1.81	AP1S1	AP-1 complex subunit sigma-1A	XM_021481407.2
B23G0295	2.58	8.75	FBXO6	F-box only protein 6	NM_001008577.2
A4G0825	2.58	1.74	KICS2	KICSTOR subunit 2	NM_001030090.3
B5G0604	2.58	3.80	CARD9	Caspase recruitment domain-containing protein 9	XM_009301787.4
B5G1309	2.58	6.19	KAT6A	Histone acetyltransferase KAT6A	NM_001123312.4
B5G1064	2.58	5.49	RAD17	Cell cycle checkpoint protein RAD17	NM_001431112.1
A5G0237	2.58	4.90	ATAD1A	Outer mitochondrial transmembrane helix translocase	NM_001004640.3
B6G0579	2.58	4.52	TIE1	Tyrosine-protein kinase receptor Tie-1	XM_001334637.10
B11G0417	2.58	12.57	CYB5R1	NADH-cytochrome b5 reductase 1	XM_005169291.5
B6G0535	2.58	6.76	WWC3	Protein WWC3	NM_001110470.1
B9G0134	2.58	2.32	SPC25	Kinetochore protein Spc25	NM_001123057.1
B11G0220	2.58	2.25	LOC113055083	A0A6P6KZP4_CARAU uncharacterized protein LOC113055083	
A18G0325	2.58	4.13	SIPA1L3	Signal-induced proliferation-associated 1-like protein 3	XM_021467865.2
A18G0450	2.58	2.49	KBTBD4	Kelch repeat and BTB domain-containing protein 4	NM_200209.1
A2G0845	2.58	9.43	SFTPD	Pulmonary surfactant-associated protein D	XM_068218032.1
A24G0664	2.58	5.82	NRP1A	Neuropilin-1a	XM_068217042.1

A2G1038	2.58	4.17	KLF6	Krueppel-like factor 6	XM_001920617.6
B13G0689	2.58	3.03	LOC107736502	A0A673GEW1_9TELE Galectin	NM_001202471.1
A5G1085	2.58	14.03	PJA2	E3 ubiquitin-protein ligase Praja-2	XM_009301875.3
B10G0100	2.58	5.12	SH3BP2	SH3 domain-binding protein 2	XM_005155583.5
B16G0862	2.58	5.18	NGFR	Tumor necrosis factor receptor superfamily member 16	XM_695893.9
B7G0847	2.58	8.34	ALOX12	Polyunsaturated fatty acid lipoxygenase ALOX12	NM_199618.1
A16G0031	2.58	5.55	ETHE1	Persulfide dioxygenase ETHE1, mitochondrial	NM_212929.1
B14G0415	2.58	3.36	LOC113114055	A0A6P6QST0_CARAU uncharacterized protein LOC113114055	NM_001013552.2
B15G0925	2.58	6.36	CEP164	Centrosomal protein of 164 kDa	XR_002455698.2
A1G0392	2.58	5.62	A0A673HW03	A0A673HW03_9TELE Si:ch211-243a20.3	NM_001145709.1
B7G0606	2.58	9.13	ADCY2	Adenylate cyclase type 2	NM_001166272.2
B3G1562	2.58	2.89	A0A673M8N8	A0A673M8N8_9TELE Uncharacterized protein	XM_021475732.2
B16G0065	2.58	7.81	G5714_015592	A0A7J6C5Y9_9TELE Uncharacterized protein	
A6G0494	2.58	1.52	RNF152	E3 ubiquitin-protein ligase rnf152	NM_001326424.1
A12G0019	2.58	2.02	HPDL	4-hydroxyphenylpyruvate dioxygenase-like protein	XM_021471074.2
A1G0172	2.58	4.28	A0A672QZG6	A0A672QZG6_SINGR Ig-like domain-containing protein	XM_017356656.3
B17G0629	2.58	5.37	RIN3	Ras and Rab interactor 3	XM_001339586.9
B5G0004	2.58	5.15	TIE2	Tyrosine-protein kinase receptor Tie-2	NM_131461.1
B8G0176	2.58	6.63	VPS13A	Intermembrane lipid transfer protein VPS13A	XM_009304309.4
B16G0550	2.58	0.19			
B15G0414	2.58	4.06	ZGC:153454	Mediator of RNA polymerase II transcription subunit 13-like	NM_001083838.2
A25G0703	2.58	11.30	H4	Histone H4	XM_068222533.1
B1G1131	2.58	2.29	SI:CH73-236C18.8	F1R3I8_DANRE Si:ch73-236c18.8	NM_001386651.1
A15G0193	2.58	4.07	PROSER3	A0A6P6QWF7_CARAU proline and serine-rich protein 3 isoform X3	NM_001305583.1
A22G0267	2.58	8.14	MRAS	Ras-related protein M-Ras	XM_003200999.6
B10G0754	2.58	2.99	RNF122	RING finger protein 122	NM_001327816.1
B5G0814	2.58	3.87	KPTN	KICSTOR complex protein kaptin	NM_001003642.2
A2G0032	2.58	3.77	G5714_001543	A0A7J6DCM3_9TELE Uncharacterized protein	XM_002660687.7
A20G0796	2.57	5.02	ENPP1	phosphodiesterase family member 1	XM_005160441.5
A11G0343	2.57	7.29	SYT6	Synaptotagmin-6	NM_212668.2
B16G0887	2.57	5.24	TWIST1	Twist-related protein	NM_001017820.1
A14G0089	2.57	0.74	MSXA	Homeobox protein MSH-A	NM_001030269.1
B22G0094	2.57	2.44	LOC113040329	A0A6P6J303_CARAU zinc finger protein 239-like	NM_001109866.1
B3G0782	2.57	3.25	IGHV3-48	Immunoglobulin heavy variable 3-48	
B16G0071	2.57	9.46	TPPP	Tubulin polymerization-promoting protein	XM_002667721.7
A8G0288	2.57	4.79	CALR	Calreticulin	NM_199713.2
B16G0851	2.57	2.08	MTBP	Mdm2-binding protein	XM_003200157.6

B17G0416	2.57	6.62	SANBR	SANT and BTB domain regulator of class switch recombination	XM_001333632.8
B14G0755	2.57	0.92	FHDC1	FH2 domain-containing protein 1	XM_009307807.4
A23G0693	2.57	4.28	CSAD	Cysteine sulfinic acid decarboxylase	NM_001007348.2
B24G0410	2.57	0.31	PIGN	GPI ethanolamine phosphate transferase 1	
B17G0066	2.57	2.38	GPR65	Psychosine receptor	
B13G0066	2.57	5.00	A0A672LD09	A0A672LD09_SINGR Uncharacterized protein	XM_005157071.5
A1G0319	2.57	1.43	GSTCD	Glutathione S-transferase C-terminal domain-containing protein	NM_001024462.1
A21G0758	2.57	3.17	HEATR6	HEAT repeat-containing protein 6	NM_001114313.1
B15G0522	2.57	2.06	ABHD15	Protein ABHD15	XM_684039.10
A21G0606	2.57	6.01	PWWP2A	PWWP domain-containing protein 2A	XM_682396.10
A15G0585	2.57	8.65	ITGAE	Integrin alpha-E	XM_002664585.7
A13G0272	2.57	2.54	IQCC	IQ domain-containing protein C	NM_001115095.2
A14G0723	2.57	2.20	RNF103	E3 ubiquitin-protein ligase RNF103	NM_001004600.1
A14G0664	2.57	20.58	CTBP1	C-terminal-binding protein 1	NM_131714.3
B16G0525	2.57	5.51			
B12G0533	2.57	8.27	IFIT5	Interferon-induced protein with tetratricopeptide repeats 5	NM_001328711.1
B7G1174	2.57	1.15	UFSP1	Ufm1-specific protease 1	NM_001089530.1
A12G0750	2.57	7.50	PRR12	Proline-rich protein 12	XM_005156015.5
B16G0348	2.57	4.64	TDP2	Tyrosyl-DNA phosphodiesterase 2	XM_005158303.5
B14G0586	2.57	1.91	CENPU	Centromere protein U	XM_009307685.4
A15G0161	2.57	2.00	ROBO1	Roundabout homolog 1	NM_001365166.1
B2G0288	2.57	11.26	NRP1A	Neuropilin-1a	NM_205674.1
A11G0071	2.57	2.47	TRIM25	ISG15 ligase TRIM25	XM_009305773.4
B22G0092	2.57	3.30	LOC113075279	A0A6P6N435_CARAU zinc finger protein 271-like isoform X3	XM_017354709.3
B18G0605	2.57	0.36	ROHU_037332	A0A498LV84_LABRO Uncharacterized protein	
A23G0705	2.56	7.11	UBE2J2	Ubiquitin-conjugating enzyme E2 J2	NM_001089535.1
A1G1000	2.56	8.74	PTPN9	Tyrosine-protein phosphatase non-receptor type 9	XM_685567.10
A13G0401	2.56	5.84	CYP17A1	17,20 lyase	XM_021474293.2
B16G1089	2.56	3.00	MECR	Enoyl-[acyl-carrier-protein] reductase, mitochondrial	NM_001017852.1
A8G0861	2.56	8.16	BIN3	Bridging integrator 3	NM_001123044.2
A24G0079	2.56	4.83	MPRIP	Myosin phosphatase Rho-interacting protein	XM_005162903.5
A14G0124	2.56	1.68	B4GALT7	Beta-1,4-galactosyltransferase 7	NM_001003417.1
A7G0205	2.56	2.12	ZNF143	Zinc finger protein 143	NM_001007441.1
A24G0146	2.56	5.38	CPT1A	Carnitine O-palmitoyltransferase 1, liver isoform	XM_017353900.3
B16G0273	2.56	7.21	RPP25L	Ribonuclease P protein subunit p25-like protein	NM_001002540.1
B22G0111	2.56	1.57	A0A672MBK6	A0A672MBK6_SINGR Ig-like domain-containing protein	XM_068216396.1
B2G1162	2.56	2.38	DVL3	Segment polarity protein dishevelled homolog DVL-3	NM_131757.2

A22G0056	2.56	6.84	CEPT1	ethanolaminephosphotransferase 1	NM_001109717.1
B12G0212	2.56	2.57	CCDC57	Coiled-coil domain-containing protein 57	XM_009306774.4
B10G0071	2.56	13.58	AKAP2	A-kinase anchor protein 2	NM_001370836.1
B3G1278	2.56	23.35	LOC107758123	A0A673N6B0_9TELE Interleukin-12 receptor subunit beta-2-like	
A5G0802	2.56	2.48	CXCL11.6	C-X-C motif chemokine 11-6	
A14G0506	2.56	0.07	TBC1D2	TBC1 domain family member 2A	XM_684512.8
A20G0709	2.56	4.26	PIGC	Phosphatidylinositol N-acetylglucosaminyltransferase subunit C	NM_213261.1
B9G0085	2.56	6.07	COG3	Conserved oligomeric Golgi complex subunit 3	NM_001144040.2
A17G0443	2.56	5.30	LOC107654878	A0A671MHG8_9TELE Glutamate-rich protein 1-like	XM_068214593.1
B11G0387	2.56	3.46	PLEKHA6	Pleckstrin homology domain-containing family A member 6	NM_001327765.1
B25G0217	2.56	3.84	HIC2	Hypermethylated in cancer 2 protein	XM_021470449.2
A19G0373	2.56	4.73	ZMYM4	Zinc finger MYM-type protein 4	XM_005159697.5
A25G0493	2.56	5.31	USP3	Ubiquitin carboxyl-terminal hydrolase 3	NM_001199871.1
A2G0897	2.56	1.39	G5714_002145	A0A7J6DE78_9TELE EF-hand domain-containing protein	
A6G0723	2.56	1.46	ZGC:112163	UPF0739 protein C1orf74 homolog	
A23G0350	2.56	7.12	USP19	Ubiquitin carboxyl-terminal hydrolase 19	XM_005162120.5
B3G0236	2.56	2.19	UBN2	Ubinuclein-2	NM_001104940.1
A3G1126	2.56	2.42	IGKV6D-41	Probable non-functional immunoglobulin kappa variable 6D-41	
A8G0904	2.56	0.78	TP73	Tumor protein p73	XM_005167294.4
B18G0362	2.56	4.26	SIPA1L3	Signal-induced proliferation-associated 1-like protein 3	XM_021467865.2
A1G0462	2.55	3.44	ZDHHC5A	Palmitoyltransferase ZDHHC5-A	XR_222664.5
A10G0415	2.55	4.46	COL26A1	Collagen alpha-1(XXVI) chain	XM_005172722.5
A6G0628	2.55	2.22	NIPA1	Magnesium transporter NIPA1	NM_001006062.1
B20G0293	2.55	3.36	MTMR9	Myotubularin-related protein 9	NM_001045070.1
B3G0545	2.55	1.30	USP31	Ubiquitin carboxyl-terminal hydrolase 31	XM_005164229.5
B25G0799	2.55	2.40	INCENP	Inner centromere protein	XM_009297716.4
B6G0149	2.55	4.74	ABCC4	ATP-binding cassette sub-family C member 4	NM_001007038.2
A23G0627	2.55	7.06	RAPGEF3	Rap guanine nucleotide exchange factor 3	XM_005162353.5
A2G0355	2.55	5.80	KDM4A	Lysine-specific demethylase 4A	XM_001339628.9
A20G0858	2.55	9.03	FRK	Tyrosine-protein kinase FRK	XM_021468553.2
A10G0195	2.55	2.22	DBNL	Drebrin-like protein	NM_001002201.1
B5G0720	2.55	7.69	MTMR12	Myotubularin-related protein 12	NM_001113613.1
A20G0477	2.55	5.14	DLL4	Delta-like protein 4	NM_001079835.1
B5G0499	2.55	15.17	TBCEL	Tubulin-specific chaperone cofactor E-like protein	XM_002662169.7
B24G0230	2.55	5.75	RMC1	Regulator of MON1-CCZ1 complex	NM_001362546.1
A21G0239	2.55	4.23	MYORG	Myogenesis-regulating glycosidase	XM_068216011.1

B16G0302	2.55	2.33	LOC113111498	A0A6P6QES6_CARAU uncharacterized protein LOC113061694	
B23G0051	2.55	2.23	MYBL2	Myb-related protein B	NM_001309826.1
A9G0733	2.55	0.60	STX19	Syntaxin-19	XM_021478870.2
B3G1192	2.55	5.71	G5714_003133	A0A7J6D9Q3_9TELE Uncharacterized protein	XM_678043.8
A9G0444	2.55	4.36	SLC39A10	Zinc transporter ZIP10	NM_200671.1
B22G1043	2.55	24.44	LAMP3	Lysosome-associated membrane glycoprotein 3	
B20G0276	2.55	11.22	CDH2	Cadherin-2	NM_131081.2
B21G0445	2.55	5.20	NLRC3	NLR family CARD domain-containing protein 3	XM_009295431.3
B11G0476	2.55	6.28	FBLN2	Fibulin-2	NM_001177445.2
B16G0806	2.55	7.32	U2AF2	Splicing factor U2AF 65 kDa subunit	NM_001029956.1
A13G0041	2.55	1.53	EIF2AK2	Interferon-induced, double-stranded RNA-activated protein kinase	XM_021480699.2
A11G0281	2.55	1.27	THOC7	THO complex subunit 7 homolog	XM_005155717.5
A13G0888	2.55	12.68	HSP30	Heat shock protein 30	NM_199954.1
A5G1021	2.55	20.63	CASTOR2	Cytosolic arginine sensor for mTORC1 subunit 2	NM_199780.2
A11G0340	2.55	2.68	DVL1	Segment polarity protein dishevelled homolog DVL-1	XM_009305965.4
A4G0339	2.55	9.90	NLRC3	NLR family CARD domain-containing protein 3	XM_017352412.3
B17G0677	2.55	1.11	PAX9	Paired box protein Pax-9	NM_131298.1
B6G0571	2.55	8.27	MAST2	threonine-protein kinase 2	XM_009302494.4
B25G0655	2.55	13.02	AKAP13	A-kinase anchor protein 13	XM_068217499.1
A1G0325	2.54	8.68	PKD2	Polycystin-2	NM_001002310.1
A23G0783	2.54	4.79	TUT4	Terminal uridylyltransferase 4	XM_001335483.8
B21G0021	2.54	4.35	FAXDC2	Fatty acid hydroxylase domain-containing protein 2	NM_213507.1
A21G0382	2.54	8.66	ITPKB	Inositol-trisphosphate 3-kinase B	XM_681366.10
B1G0871	2.54	2.47	FBXO48	F-box only protein 48	
B6G0512	2.54	5.01	ZBTB11	Zinc finger and BTB domain-containing protein 11	NM_212650.2
B18G0492	2.54	2.76	KIF23	Kinesin-like protein KIF23	XM_009293529.4
B9G0299	2.54	4.32	MED14	Mediator of RNA polymerase II transcription subunit 14	XM_005167821.5
B24G0600	2.54	5.05	MYCB	Transcriptional regulator Myc-2	NM_131412.1
B7G1191	2.54	0.07	PLAC8	Placenta-specific gene 8 protein	
A3G1128	2.54	2.31	LAC	Ig lambda chain C region	XM_021478474.2
B20G0888	2.54	2.61	ADF1	Transcription factor Adf-1	NM_001386313.1
B25G0878	2.54	4.65	GXYLT1	Glucoside xylosyltransferase 1	XM_017354203.3
A19G0009	2.54	0.13	LOC106511497	A0A2I4AJN5_9TELE uncharacterized protein LOC106511497	
B25G0740	2.54	3.79	SLC25A44	Solute carrier family 25 member 44	NM_001007350.2
A21G0148	2.54	10.32	SLC4A4	Electrogenic sodium bicarbonate cotransporter 1	XM_009295135.4
A22G0519	2.54	5.70	CCDC50	Coiled-coil domain-containing protein 50	NM_001365998.1
A12G0760	2.54	2.87	MAPK7	Mitogen-activated protein kinase 7	NM_001013451.3
B10G0681	2.54	1.00	MORN3	MORN repeat-containing protein 3	NM_001017666.1
A12G0727	2.54	5.25	FRA10AC1	Protein FRA10AC1	NM_001006006.2

A2G0190	2.54	3.91	AGRP	Agouti-related protein	
B20G0251	2.54	2.39	TMEM205	Transmembrane protein 205	XM_693051.7
A25G0557	2.54	1.37	SIGIRR	Single Ig IL-1-related receptor	NM_001098250.1
B16G0530	2.54	1.80	SMAD4	Mothers against decapentaplegic homolog 4	XM_005158079.5
B17G0479	2.54	6.99	RAB3GAP2	Rab3 GTPase-activating protein non-catalytic subunit	XM_017351602.3
B13G0404	2.54	1.82	KHDRBS2	KH domain-containing, RNA-binding, signal transduction-associated protein 2	NM_131281.2
B5G0458	2.54	16.03	TMEM248	Transmembrane protein 248	XM_005168624.5
B9G0158	2.54	6.11	IGFBP2B	Insulin-like growth factor-binding protein 2-B	NM_001126464.1
B10G0886	2.54	8.02	APC	Adenomatous polyposis coli protein	XM_005155523.5
B20G0224	2.54	2.16	PLPP6	phosphate phosphohydrolase PLPP6	NM_001328338.1
B13G0771	2.54	6.04	LRRFIP2	Leucine-rich repeat flightless-interacting protein 2	NM_001431065.1
B1G0760	2.54	6.56	SERHL	Serine hydrolase-like protein	NM_199615.2
B4G0866	2.54	3.90	DPX16_3453	A0A3N0XKN3_ANAGA Zinc finger protein 569	XM_068220196.1
A16G1038	2.54	7.34	BAIAP2	Brain-specific angiogenesis inhibitor 1-associated protein 2	NM_001328388.1
B23G0070	2.54	3.57	SMIM29	Small integral membrane protein 29	
B18G0277	2.54	5.58	RTASE	Probable RNA-directed DNA polymerase from transposon BS	XM_068218007.1
A23G0816	2.54	3.72	OTUD4	OTU domain-containing protein 4	XM_005174151.5
B1G0872	2.53	4.77	APLF	Aprataxin and PNK-like factor	XM_068220986.1
A13G0768	2.53	0.91	TJAP1	Tight junction-associated protein 1	
A22G0615	2.53	2.50	NCF4	Neutrophil cytosol factor 4	NM_205711.2
B22G0468	2.53	0.96	LOC113100686	A0A6P6PL60_CARAU CD48 antigen-like	XM_001346243.8
B6G0731	2.53	3.85	EEFSEC	Selenocysteine-specific elongation factor	NM_001037436.2
A23G0735	2.53	6.86	G5714_022360	A0A7J6BNW5_9TELE DUF4482 domain-containing protein	
A2G1074	2.53	3.33	BLVRA	Biliverdin reductase A	NM_001076601.1
B4G0790	2.53	2.79	A0A673HWK9	A0A673HWK9_9TELE Uncharacterized protein	XM_068219487.1
B7G0789	2.53	2.15	RIPK2	threonine-protein kinase 2	XM_005166398.5
A22G0447	2.53	13.49	TCF3	Transcription factor E2-alpha	NM_001172683.1
A7G0561	2.53	3.66	RBCK1	RanBP-type and C3HC4-type zinc finger-containing protein 1	NM_001111200.1
B11G0566	2.53	5.58	COL6A1	Collagen alpha-1(VI) chain	XM_680437.7
B3G1308	2.53	3.73	EPN3	Epsin-3	XM_005164380.4
B21G0658	2.53	0.72	CHCHD2	Coiled-coil-helix-coiled-coil-helix domain-containing protein 2	NM_200784.1
A4G0620	2.53	5.75	UHRF1BP1L	UHRF1-binding protein 1-like	XM_009300290.4
B6G0007	2.53	4.30	SMARCA4	Transcription activator BRG1	XM_021476960.2
A1G0868	2.53	9.22	TRIM2	Tripartite motif-containing protein 2	XM_009306306.4
B3G1439	2.53	2.32	SMIM10L2A	Small integral membrane protein 10-like protein 2A	NM_001163443.1
A13G0696	2.53	4.08	LRMDA	Leucine-rich melanocyte differentiation-associated protein	NM_001347683.1

A15G0400	2.53	2.43	PITPNM3	Membrane-associated phosphatidylinositol transfer protein 3	XM_005157661.5
B5G0928	2.53	4.20	UAP1L1	UDP-N-acetylhexosamine pyrophosphorylase-like protein 1	NM_200294.1
B2G0244	2.53	34.50	LOC107578657	A0A672K282_SINGR Uncharacterized LOC107578657	
A9G0177	2.53	3.05	NAB1	NGFI-A-binding protein 1	NM_001123273.1
B6G0001	2.53	3.71	MBD5	Methyl-CpG-binding domain protein 5	XM_068221854.1
B2G0375	2.53	9.04	SLC7A8	Large neutral amino acids transporter small subunit 2	XM_068214399.1
A11G0156	2.53	3.38	LGSN	Lengsin	NM_213074.1
A25G0172	2.52	3.26	UNC45A	Protein unc-45 homolog A	NM_001017671.1
A9G0360	2.52	1.14	CHAF1B	Chromatin assembly factor 1 subunit B	NM_001328129.1
B23G0883	2.52	2.94	ANAPC10	Anaphase-promoting complex subunit 10	NM_001267697.1
B5G1025	2.52	1.43	TSPAN15	Tetraspanin-15	NM_001017802.1
B17G0414	2.52	6.06	PEX13	Peroxisomal membrane protein PEX13	NM_200645.1
A1G0385	2.52	10.13	MCOLN1	Mucolipin-1	NM_001328165.1
A19G0255	2.52	4.11	TRIT1	tRNA dimethylallyltransferase	NM_001044774.1
A7G0829	2.52	0.69	TNK2	Activated CDC42 kinase 1	NM_001080017.2
B7G0626	2.52	7.66	LPCAT2	Lysophosphatidylcholine acyltransferase 2	XM_005168979.5
B5G0653	2.52	5.20	SMARCA2	Probable global transcription activator SNF2L2	NM_001044775.2
B19G0656	2.52	4.91	HMBOX1	Homeobox-containing protein 1	NM_001002668.1
A1G0317	2.52	3.00	CYP2U1	Cytochrome P450 2U1	NM_001145564.1
B17G0183	2.52	2.84	SSTR1	Somatostatin receptor type 1	XM_068214529.1
A14G0582	2.52	9.79	DHRS13	reductase SDR family member 13	NM_205648.1
A21G0335	2.52	6.08	GPAT3	Glycerol-3-phosphate acyltransferase 3	NM_001002685.1
B19G0070	2.52	5.99	PTK2	Focal adhesion kinase 1	NM_198819.1
B19G0072	2.52	3.32	KLHL7	Kelch-like protein 7	NM_200877.2
A11G0640	2.52	1.08	MRTO4	mRNA turnover protein 4 homolog	XM_009306257.4
B3G0304	2.52	4.84	ABCA3	Phospholipid-transporting ATPase ABCA3	XM_068218749.1
A23G0824	2.52	1.58	G5714_022248	A0A7J6BML3_9TELE MADF domain-containing protein	
A16G0955	2.52	3.17	ZNF407	Zinc finger protein 407	XM_005157848.5
B19G0529	2.52	8.90	NFYC	Nuclear transcription factor Y subunit gamma	XM_009294376.4
A2G0831	2.52	6.00	INSR	Insulin receptor	NM_001142672.1
B23G0915	2.52	7.86	VDRA	Vitamin D3 receptor A	NM_130919.1
A19G0273	2.52	1.19	TDP2	Tyrosyl-DNA phosphodiesterase 2	NM_001079703.1
B18G0358	2.52	7.70	CS047	Uncharacterized protein C19orf47 homolog	NM_001045241.1
A11G0527	2.52	14.30	FAM212AA	PAK4-inhibitor inka1	NM_001313772.1
A4G0716	2.51	5.20	PNPLA8	Calcium-independent phospholipase A2-gamma	XM_001918696.6
B6G0876	2.51	5.42	STX16	Syntaxin-16	XM_005172117.5
B22G0292	2.51	6.74	F7725_023674	A0A7J5XY10_DISMA Integrase catalytic domain-containing protein	
B7G0838	2.51	4.57	HISAT	Histidine N-acetyltransferase	NM_001123054.2

A12G0345	2.51	6.20	TAP2	Antigen peptide transporter 2	XM_005156299.5
B14G0488	2.51	8.98	NFKB1	Nuclear factor NF-kappa-B p105 subunit	XM_021481269.2
B17G0841	2.51	1.96	CNK3/IPCEF1	IPCEF1 PE=1 SV=1	
A6G0832	2.51	1.74	PHC2	Polyhomeotic-like protein 2	NM_201202.3
A13G0315	2.51	2.68	MNAT1	CDK-activating kinase assembly factor MAT1	XM_001342476.9
A17G0490	2.51	4.16	MINPP1	Multiple inositol polyphosphate phosphatase 1	XM_005158515.5
A7G0972	2.51	5.59	KDM6B	Lysine-specific demethylase 6B	XM_021477780.2
B7G0833	2.51	3.64	TRAPPC14	Trafficking protein particle complex subunit 14	NM_001423811.1
B10G0058	2.51	2.06	SPTLC1	Serine palmitoyltransferase 1	NM_001020471.1
A23G0828	2.51	6.87	CDKL5	Cyclin-dependent kinase-like 5	
B2G0837	2.51	1.86	INHBA	Inhibin beta A chain	NM_001018156.1
B10G0636	2.51	2.08	FOXRED1	FAD-dependent oxidoreductase domain-containing protein 1	NM_001113578.1
A24G0320	2.51	2.55	MBTPS2	Membrane-bound transcription factor site-2 protease	NM_001025461.1
A20G0482	2.51	2.17	ZBTB1	Zinc finger and BTB domain-containing protein 1	NM_214797.1
B5G0173	2.51	4.75	NF2	Merlin	NM_001128707.1
B9G0406	2.51	1.74	SKA3	Spindle and kinetochore-associated protein 3	NM_001202437.1
B7G0228	2.51	7.88	ZNF319	Zinc finger protein 319	XM_001923303.8
B8G0592	2.51	2.31	CF226	Uncharacterized protein C6orf226 homolog	
A19G0641	2.51	3.28	C3AR1	C3a anaphylatoxin chemotactic receptor	XM_068215250.1
A13G0277	2.51	5.30	NDUFAF1	Complex I intermediate-associated protein 30, mitochondrial	NM_001353962.1
B2G0539	2.51	0.14	A0A673L8Z6	delta variable 17.0	
B6G0205	2.51	4.07	ERCC3	General transcription and DNA repair factor IIH helicase subunit XPB	NM_201582.1
A14G0571	2.51	3.79	NANOS1	Nanos homolog 1	NM_001305661.1
A9G0647	2.51	5.85	COA5	Cytochrome c oxidase assembly factor 5	NM_001161497.2
A5G0061	2.51	2.21	TSPOAP1	Peripheral-type benzodiazepine receptor-associated protein 1	XM_068221385.1
B15G0418	2.51	4.32	PLEKHG2	Pleckstrin homology domain-containing family G member 2	XM_068213708.1
B5G0123	2.51	5.78	SLC1A3	Excitatory amino acid transporter 1	XM_009301170.3
A3G1084	2.51	4.38	ANKRD54	Ankyrin repeat domain-containing protein 54	NM_001002578.1
B19G0385	2.50	1.76	HOXA3A	Homeobox protein Hox-A3a	NM_131534.2
A6G0384	2.50	2.48	ENDOV	Endonuclease V	
B22G0148	2.50	4.54	TIMM44	Mitochondrial import inner membrane translocase subunit TIM44	NM_001013507.1
B16G0807	2.50	3.28	CACNG6	Voltage-dependent calcium channel gamma-6 subunit	XM_001342797.7
A17G0274	2.50	17.45	XDH	oxidase	XM_683891.8
B20G0811	2.50	7.36	RMDN3	Regulator of microtubule dynamics protein 3	NM_001083050.1
B13G0297	2.50	1.70	ENPEP	Glutamyl aminopeptidase	NM_001002351.1
A8G0964	2.50	6.68	TCB1	Transposable element Tcb1 transposase	XM_068216952.1
A20G0888	2.50	4.57	CDK19	Cyclin-dependent kinase 19	XM_021473354.2

A11G0173	2.50	1.77	CDC6	Cell division control protein 6 homolog	NM_213239.1
A5G0527	2.50	0.62	ADAMTS8	A disintegrin and metalloproteinase with thrombospondin motifs 8	XM_693015.8
A8G0629	2.50	1.99	FBXO4	F-box only protein 4	XM_002663139.7
B5G0788	2.50	10.37	MYO1C	Unconventional myosin-Ic	NM_001431130.1
A11G0050	2.50	8.30	RTASE	Probable RNA-directed DNA polymerase from transposon BS	XM_021479679.2
B21G0775	2.50	0.33	AGGF1	Angiogenic factor with G patch and FHA domains 1	NM_001079982.1
B4G0691	2.50	1.21	G5714_004666	A0A7J6D5A0_9TELE Uncharacterized protein	XM_068220087.1
B20G0533	2.50	4.44	HPCAL1	Hippocalcin-like protein 1	NM_201164.1
B15G0331	2.50	4.86	KIAA0100	Protein KIAA0100	
A2G0363	2.50	5.50	CDC20	Cell division cycle protein 20 homolog	NM_213080.2
A12G0029	2.50	4.60	LZTS2	Leucine zipper putative tumor suppressor 2 homolog	XM_017358600.3
B13G0719	2.50	2.50	IFI27L2B	Interferon alpha-inducible protein 27-like protein 2B	NM_001144794.1
B18G0197	2.50	4.61	RTASE	Probable RNA-directed DNA polymerase from transposon BS	XR_011011165.1
A9G0572	2.50	2.09	TTF2	Transcription termination factor 2	XM_005168619.5
B22G0574	2.50	9.94	RND3	Rho-related GTP-binding protein RhoE	NM_199522.1
A13G0235	2.50	3.71			XM_021472702.2
B13G0126	2.50	7.21	C1D	Nuclear nucleic acid-binding protein C1D	XM_005169532.5
B16G0587	2.50	50.80	EPB41	Protein 4.1	NM_175084.3
A23G0681	2.50	0.92	HOXC9A	Homeobox protein Hox-C9a	NM_131528.3
B5G1106	2.50	6.92	OSBP2	Oxysterol-binding protein 2	NM_001122748.1
A9G0434	2.50	14.46	ZEB2	Zinc finger E-box-binding homeobox 2	NM_001135104.2
B3G0391	2.50	9.97	GIMAP7	GTPase IMAP family member 7	
B10G0587	2.50	1.92	DNAJB13	DnaJ homolog subfamily B member 13	NM_001017606.1
B1G0696	2.50	1.70	SI:CH73-109D9.2	F1QNC0_DANRE Si:ch73-109d9.2	
A10G0566	2.50	5.90	PABIR1	P2R1A-PPP2R2A-interacting phosphatase regulator 1	XM_009305276.4
A8G0706	2.50	3.42	ANAPC5	Anaphase-promoting complex subunit 5	NM_199622.2
B22G0171	2.49	3.23	LOC113040289	A0A6P6J2N4_CARAU carcinoembryonic antigen-related cell adhesion molecule 3-like	XM_017353228.3
B11G0304	2.49	3.42	TAFA1	Chemokine-like protein TAFA-1	NM_001013545.2
B5G0300	2.49	0.13	SLC25A5	ATP translocase 2	NM_001089554.2
A1G0202	2.49	0.38	LITAF	Lipopolysaccharide-induced tumor necrosis factor-alpha factor homolog	XM_068221884.1
B21G0751	2.49	6.21	ARHGAP24	Rho GTPase-activating protein 24	XM_001338974.7
B25G0730	2.49	2.95	HMG20A	High mobility group protein 20A	NM_001089334.1
A13G0491	2.49	2.09	GALNT2	Polypeptide N-acetylgalactosaminyltransferase 2	XM_009307399.3

A23G0622	2.49	4.65	ANKRD52	threonine-protein phosphatase 6 regulatory ankyrin repeat subunit C	NM_001018154.1
A2G1011	2.49	4.68	WDR47	WD repeat-containing protein 47	XM_021471290.2
B23G0829	2.49	2.31	GPX7	Glutathione peroxidase 7	NM_001020501.1
B4G0241	2.49	2.59	KICS2	KICSTOR subunit 2	NM_001030090.3
B18G0441	2.49	6.12	BCAR1	Breast cancer anti-estrogen resistance protein 1	XM_021467686.2
A9G0705	2.49	3.64	LOC107551988	A0A672TFN3_SINGR Uncharacterized LOC107551988	NM_001077176.1
A2G0536	2.49	3.74	PPP2R3A	threonine-protein phosphatase 2A regulatory subunit B" subunit alpha	XM_009298556.4
A3G1228	2.49	2.64	G5714_004447	A0A7J6D5J8_9TELE Uncharacterized protein	
A5G0546	2.49	20.59	TRIM16	Tripartite motif-containing protein 16	NM_001075103.1
A8G0808	2.49	2.43	TTI2	TELO2-interacting protein 2	XM_002663205.6
B3G1102	2.49	10.76	KCTD13	POZ domain-containing adapter for CUL3-mediated RhoA degradation protein 1	NM_001077151.1
A10G0093	2.49	5.31	NAA25	N-alpha-acetyltransferase 25, NatB auxiliary subunit	XM_005172598.5
A20G0906	2.49	5.12	MED23	Mediator of RNA polymerase II transcription subunit 23	NM_001431152.1
B22G0138	2.49	3.15	GPR55	G-protein coupled receptor 55	
B12G0608	2.49	3.06	MED24	Mediator of RNA polymerase II transcription subunit 24	NM_001039062.1
A15G0487	2.49	2.78	LOC113058995	A0A6P6LEF4_CARAU uncharacterized protein LOC113058995	XM_001344636.8
A5G0373	2.49	12.01	RNF128	E3 ubiquitin-protein ligase RNF128	NM_212615.1
B6G0775	2.49	6.20	MST1R	Macrophage-stimulating protein receptor	XM_068222037.1
A16G0354	2.49	1.46	PIGV	GPI mannosyltransferase 2	NM_001200060.1
A14G0600	2.49	7.66	ACSL1	Long-chain-fatty-acid--CoA ligase 1	XM_005169565.5
B3G0371	2.49	6.72	FCRLA	Fc receptor-like A	XM_068218085.1
A19G0237	2.49	5.00	LDLRAP1	Low density lipoprotein receptor adapter protein 1	XM_005159645.4
B19G0602	2.49	3.51	ANLN	Anillin	NM_001102676.2
A18G0634	2.49	3.92	IMPDH1A	Inosine-5'-monophosphate dehydrogenase 1a	XM_005159007.5
B2G0714	2.49	4.92	RNF31	E3 ubiquitin-protein ligase RNF31	XM_009298565.4
B15G0094	2.49	1.49	P2RY14	P2Y purinoceptor 14	XM_009291431.4
A15G0264	2.49	5.35	INPP5K	Inositol polyphosphate 5-phosphatase K	NM_001089493.1
A2G0573	2.49	3.83	ZSWIM5	Zinc finger SWIM domain-containing protein 5	NM_001144819.1
B20G0561	2.49	5.18	DDX1	ATP-dependent RNA helicase DDX1	NM_001197324.1
B11G0585	2.49	4.74	MST1R	Macrophage-stimulating protein receptor	NM_001034175.2
B23G0631	2.49	2.42	MTFR2	Mitochondrial fission regulator 2	
B24G0672	2.49	10.67	ACBD5A	Acyl-CoA-binding domain-containing protein 5A	XM_021470174.2
B8G0547	2.49	2.68	SYPL1	Synaptophysin-like protein 1	XM_017357386.2

A6G0863	2.49	0.95	APCDD1L	Protein APCDD1-like	XM_021471845.2
B18G0582	2.49	3.27	SSPN	Sarcospan	NM_001037580.2
A6G0057	2.49	1.59	TRIM21	E3 ubiquitin-protein ligase TRIM21	
B11G0194	2.49	4.52	THBS4	Thrombospondin-4	NM_001114739.2
A19G0136	2.49	3.32	CTDP1	RNA polymerase II subunit A C-terminal domain phosphatase	NM_001002873.1
B22G0938	2.49	5.85	SHOC2	Leucine-rich repeat protein SHOC-2	NM_001044786.1
B1G0109	2.49	3.47	HECW2	E3 ubiquitin-protein ligase HECW2	XM_003197612.6
B14G0301	2.49	1.25	ZBTB3	Zinc finger and BTB domain-containing protein 3	XM_009291211.4
A11G0622	2.48	6.07	G5714_011614	A0A7J6CJ20_9TELE Uncharacterized protein	
A2G0029	2.48	3.16	G5714_001543	A0A7J6DCM3_9TELE Uncharacterized protein	NM_001114314.2
A6G0684	2.48	1.25	COL7A1	Collagen alpha-1(VII) chain	XM_068222026.1
B5G0734	2.48	12.58	WDFY3	WD repeat and FYVE domain-containing protein 3	XM_068221471.1
A9G0072	2.48	5.51	SMARCAL1	SNF-related matrix-associated actin-dependent regulator of chromatin subfamily A-like protein 1	XM_021478691.2
B22G0070	2.48	5.46	PPARD	Peroxisome proliferator-activated receptor delta	XM_068216510.1
B16G0556	2.48	10.49	LOC113116158	A0A6P6R2C2_CARAU uncharacterized protein LOC113116158	NM_001326485.1
B13G0639	2.48	4.89	CYT	Cystatin	NM_001353962.1
A23G0812	2.48	2.90	LOC113065431	A0A6P6M780_CARAU uncharacterized protein LOC113065431 isoform X2	
A12G0385	2.48	6.88	PSME4B	Proteasome activator complex subunit 4B	NM_001191020.1
B3G1022	2.48	6.90	RABEP2	Rab GTPase-binding effector protein 2	NM_001080625.2
A17G0450	2.48	0.55	GJB4	Gap junction beta-4 protein	
B23G0393	2.48	4.55	ADNP	Activity-dependent neuroprotector homeobox protein	NM_001347570.2
B14G0049	2.48	3.68	GATAD1	GATA zinc finger domain-containing protein 1	NM_001386276.1
A5G1153	2.48	3.62	X-ELEMENT\ORF2	Probable RNA-directed DNA polymerase from transposon X-element	
A1G0248	2.48	1.90	RNF150	RING finger protein 150	NM_001145572.2
B19G0308	2.48	3.88	DDX6	Probable ATP-dependent RNA helicase DDX6	XM_001340824.8
A5G0478	2.48	1.67	OGFOD2	2-oxoglutarate and iron-dependent oxygenase domain-containing protein 2	NM_001320174.1
A5G0723	2.48	4.55	LOC113077308	A0A6P6N8K5_CARAU uncharacterized protein LOC113077308	XM_005165376.5
B21G0432	2.48	1.39	SUMF2	Inactive C-alpha-formylglycine-generating enzyme 2	NM_001005980.2
B12G0342	2.48	2.75	ARMC7	Armadillo repeat-containing protein 7	NM_001076599.1
B21G0016	2.48	2.54	ZGC:77058	UPF0461 protein C5orf24 homolog	

A7G0985	2.48	15.89	CHD3	Chromodomain-helicase-DNA-binding protein 3	XM_005172235.5
A5G0491	2.48	3.83	TRAF2	TNF receptor-associated factor 2	XM_021471606.2
A13G0619	2.48	6.22	C9ORF72	Guanine nucleotide exchange factor C9orf72	NM_001278703.1
B5G1284	2.48	3.19	HIRA	Protein HIRA	XM_691386.8
A24G0394	2.48	3.06	LOC107701649	A0A671RU23_9TELE A-kinase anchor protein 17A-like	
B18G0248	2.48	3.62	X-ELEMENT\ORF2	Probable RNA-directed DNA polymerase from transposon X-element	
B16G1148	2.48	4.26	CDKAL1	Threonylcarbamoyladenosine tRNA methylthiotransferase	
A23G0332	2.48	1.61	KCTD6	POZ domain-containing protein KCTD6	NM_001002329.1
A12G0586	2.48	7.24	AVL9	Late secretory pathway protein AVL9 homolog	NM_001327830.1
B5G1091	2.48	2.57	CARNMT1	Carnosine N-methyltransferase	XM_005171768.5
A10G0796	2.48	0.15	G5714_010694	A0A7J6CMY6_9TELE EGF-like domain-containing protein	
B5G0076	2.48	2.27	DHRS11	reductase SDR family member 11	NM_001100048.1
B16G0735	2.48	1.71	LOC113116444	A0A6P6R4E9_CARAU contactin-3-like isoform X1	XM_068213863.1
A16G0303	2.48	9.23	SI:CH211-198C19.3	F1R5R7_DANRE Si:ch211-198c19.3 (Fragment)	
A18G0118	2.48	3.12	CXCR5	C-X-C chemokine receptor type 5	
A16G0793	2.48	4.38	SETD2	Histone-lysine N-methyltransferase SETD2	XM_009292184.4
B6G0171	2.48	4.02	RGL1	Ral guanine nucleotide dissociation stimulator-like 1	XM_021471714.2
B3G0464	2.48	6.98	PBX1	Pre-B-cell leukemia transcription factor 1	NM_131447.1
A8G0972	2.48	1.44	MMP23	Matrix metalloproteinase-23	XM_009304336.3
B15G0502	2.47	2.21	PIS	CDP-diacylglycerol--inositol 3-phosphatidyltransferase	XM_005157607.5
A10G0714	2.47	4.17	TBC1D10A	TBC1 domain family member 10A	XM_686009.6
A13G0186	2.47	3.49	KCNH5	Potassium voltage-gated channel subfamily H member 5	XM_001921028.6
B11G0018	2.47	4.88	DUSP7	Dual specificity protein phosphatase 7	NM_001320425.1
B7G0931	2.47	4.80	CHORDC1	Cysteine and histidine-rich domain-containing protein 1	NM_001003457.1
B13G0439	2.47	5.41	MSXC	Homeobox protein MSH-C	NM_001126397.1
A11G0541	2.47	4.84	GPX1	Glutathione peroxidase 1	NM_001326447.1
B12G0092	2.47	0.97	G5714_012294	A0A7J6CGT9_9TELE Uncharacterized protein	
A12G0671	2.47	7.12	TUBGCP2	Gamma-tubulin complex component 2	NM_200122.1
B22G0352	2.47	1.76	LOC113082004	A0A6P6NL26_CARAU oocyte zinc finger protein XlCOF6-like	NM_001386816.1
B5G0745	2.47	7.30	BMP2K	BMP-2-inducible protein kinase	NM_001193540.1
A14G0605	2.47	2.86	LRRN4CL	LRRN4 C-terminal-like protein	
A18G0083	2.47	2.80	RNPEPL1	Aminopeptidase RNPEPL1	NM_001348188.1
A14G0229	2.47	3.89	NKRF	NF-kappa-B-repressing factor	NM_001004499.3
B14G0484	2.47	3.88	FAM193B	Protein FAM193B	XM_692607.9
A3G0471	2.47	5.59	INTS1	Integrator complex subunit 1	XM_003198152.6
B1G0069	2.47	10.97	CD2	T-cell surface antigen CD2	
B4G0288	2.47	2.28	METTL25	Probable methyltransferase-like protein 25	NM_001045057.1

A23G0545	2.47	3.73	PEX14	Peroxisomal membrane protein PEX14	XM_005162283.5
A4G0398	2.47	1.87	G5714_022074	A0A7J6BSV8_9TELE Uncharacterized protein	XM_068213649.1
B5G0854	2.47	3.58	FCHO2	F-BAR domain only protein 2	NM_001020781.2
A23G0198	2.47	1.63	PIGT	GPI transamidase component PIG-T	XM_679516.9
A15G0198	2.47	0.60	C3AR1	C3a anaphylatoxin chemotactic receptor	XM_068213637.1
B6G0094	2.47	26.35	PHOSPHO2	Probable phosphatase phospho2	XM_005172076.5
A19G0201	2.47	4.46	TMEM268	Transmembrane protein 268	
A17G0163	2.47	3.32	EXOC5	Exocyst complex component 5	NM_001113796.1
A25G0262	2.47	2.38	PRMT7	Protein arginine N-methyltransferase 7	NM_200503.2
B19G0826	2.47	3.47	EEPD1	phosphatase family domain-containing protein 1	NM_205759.1
B23G0876	2.47	16.23	ITCH	E3 ubiquitin-protein ligase Itchy	NM_001302226.1
A7G0432	2.47	6.18	CRTC3	CREB-regulated transcription coactivator 3	XM_005166492.5
B21G0720	2.47	7.18	ELL2	RNA polymerase II elongation factor ELL2	NM_001083812.2
A8G0600	2.47	3.60	ZNG1	Zinc-regulated GTPase metalloprotein activator 1	NM_213253.1
B12G0781	2.47	4.27	RASD1	Dexamethasone-induced Ras-related protein 1	NM_200532.1
A13G0684	2.47	2.68	EIF4E	Eukaryotic translation initiation factor 4E	XM_009307483.4
B5G1243	2.47	7.29	PXMP2	Peroxisomal membrane protein 2	NM_001004607.1
B25G0646	2.47	2.59	ZCCHC14	Zinc finger CCHC domain-containing protein 14	XM_001336490.8
A7G0938	2.47	4.80	CLCN5	Cl(-) exchange transporter 5	XM_680670.7
B6G0621	2.47	0.75	PLCXD1	PI-PLC X domain-containing protein 1	XM_005165995.5
A7G0910	2.46	0.16	A33	Zinc-binding protein A33	NM_001386813.1
A25G0145	2.46	2.71	GIMAP9	GTPase IMAP family member 9	XM_068217486.1
B22G1173	2.46	2.73	PFLUV_G00196910	A0A6A5DVP0_PERFL HECT domain-containing protein	
B25G0583	2.46	3.07	PRMT7	Protein arginine N-methyltransferase 7	NM_200503.2
A9G0772	2.46	11.59	CMKLR2	Chemerin-like receptor 2	
A25G0450	2.46	0.78	UGT2A2	UDP-glucuronosyltransferase 2A2	NM_001177497.2
A24G0400	2.46	3.44	CDK8	Cyclin-dependent kinase 8	NM_194408.2
A1G0288	2.46	4.95	SPRED2	Sprouty-related, EVH1 domain-containing protein 2	XM_681645.7
B21G0415	2.46	6.82	ANKHD1	Ankyrin repeat and KH domain-containing protein 1	XM_009295482.4
A9G0269	2.46	1.25	STAG2	Cohesin subunit SA-2	XM_017357835.3
B12G0142	2.46	3.68	UNK	RING finger protein unkempt homolog	NM_200236.2
B9G0611	2.46	3.25	LOC107663926	A0A671ST22_9TELE Transcription termination factor 2-like	XM_005168619.5
B25G0632	2.46	4.44	CCL2	C-C motif chemokine 2	
A5G0756	2.46	6.60	WDFY3	WD repeat and FYVE domain-containing protein 3	XM_068221467.1
B19G0672	2.46	6.78	RIPOR2	Rho family-interacting cell polarization regulator 2	NM_001045026.1
B18G0776	2.46	5.34	AAMDC	Mth938 domain-containing protein	NM_001020764.1
B14G0786	2.46	1.76			

A23G0191	2.46	2.31	EEF1A2	Elongation factor 1-alpha 2	NM_001002371.1
A8G0617	2.46	2.48	PTGES2	Prostaglandin E synthase 2	
A15G0283	2.46	2.29	ARFIP2	Arfaptin-2	XM_005157358.5
A3G0255	2.46	5.41	TTC39A	Tetratricopeptide repeat protein 39A	NM_001080084.1
B2G0134	2.46	8.45	CD300E	CMRF35-like molecule 2	XR_011007163.1
A15G0572	2.46	7.78	POU2AF1	POU domain class 2-associating factor 1	
A2G0934	2.46	4.21	TMEM71	Transmembrane protein 71	NM_001130853.3
A16G0944	2.46	1.11	X-ELEMENT\ORF2	Probable RNA-directed DNA polymerase from transposon X-element	
A20G0183	2.46	3.13	ATAD2B	ATPase family AAA domain-containing protein 2B	NM_001365966.1
B21G0178	2.46	8.05	LIMK1	LIM domain kinase 1	XM_005161446.5
B7G0994	2.46	1.06	RASL12	Ras-like protein family member 12	NM_200395.1
A23G0169	2.46	3.30	ZNF512B	Zinc finger protein 512B	XM_005162004.5
B12G0095	2.46	3.73	FDXR	NADPH:adrenodoxin oxidoreductase, mitochondrial	XM_005169494.5
B23G0277	2.45	5.57	PCMTD2	Protein-L-isoaspartate O-methyltransferase domain-containing protein 2	XR_011008410.1
A7G0885	2.45	1.15	CSKMT	Citrate synthase-lysine N-methyltransferase CSKMT, mitochondrial	
A1G0575	2.45	3.34	LMO7	LIM domain only protein 7	NM_001045444.2
A11G0001	2.45	1.49			NM_212662.2
B16G0363	2.45	13.80	THRAP3	Thyroid hormone receptor-associated protein 3	XM_068214170.1
B22G0931	2.45	3.30	PLA2G6	88 kDa calcium-independent phospholipase A2	NM_213097.2
B23G0708	2.45	1.87	MFSD5	Molybdate-anion transporter	NM_001002713.1
A11G0081	2.45	4.90	PCDH20	Protocadherin-20	NM_001278600.1
A21G0702	2.45	2.34	WWC1	Protein KIBRA	XM_684183.9
A23G0229	2.45	9.73	OSBPL2	Oxysterol-binding protein-related protein 2	NM_199872.2
B1G0995	2.45	5.23	LOC113083558	A0A6P6NQ08_CARAU B-cell receptor CD22-like isoform X1	
B25G0387	2.45	2.79	UGT2A2	UDP-glucuronosyltransferase 2A2	NM_001177497.2
A12G0269	2.45	0.30	LOC107581625	A0A672KQH3_SINGR Uncharacterized LOC107581625	
A12G0582	2.45	5.41	TGFB1I1	Transforming growth factor beta-1-induced transcript 1 protein	
B14G0030	2.45	2.12	SPRY1	Protein sprouty homolog 1	NM_001122600.1
B16G1116	2.45	5.12	TENT5A	Terminal nucleotidyltransferase 5A	XM_005157860.5
A7G0106	2.45	1.07	HGFAC	Hepatocyte growth factor activator	XM_002667671.7
A10G0434	2.45	3.40	THAP12	52 kDa repressor of the inhibitor of the protein kinase	NM_213081.1
A5G0761	2.45	9.08	SNX18	Sorting nexin-18	NM_001100006.1
B21G0100	2.45	4.85	TMEM164	Transmembrane protein 164	XM_021469256.2
B14G0027	2.45	3.34	ANKRD50	Ankyrin repeat domain-containing protein 50	XM_068213424.1
B20G0512	2.45	10.22	SOX11	Transcription factor SOX-11	NM_131337.1
B23G0715	2.45	0.47	HOXC10A	Homeobox protein Hox-C10a	XM_017353498.3
A24G0030	2.45	0.29	TRIM16	Tripartite motif-containing protein 16	XM_681071.8

B16G0394	2.45	6.76	ARID1A	AT-rich interactive domain-containing protein 1A	XM_021466949.2
B21G0735	2.45	2.30	DBNL	Drebrin-like protein	XM_005173870.5
A24G0032	2.45	2.98	TRIM16	Tripartite motif-containing protein 16	XM_681071.8
A3G1064	2.45	4.03	C1QTNF1	Complement C1q tumor necrosis factor-related protein 1	XM_688410.10
B15G0716	2.45	7.01	DHX16	Pre-mRNA-splicing factor ATP-dependent RNA helicase DHX16	NM_200024.1
A17G0908	2.45	3.40	WDR20	WD repeat-containing protein 20	XM_021467251.2
B5G1055	2.45	3.95	TANGO2	Transport and Golgi organization protein 2 homolog	XM_005171840.5
B21G0354	2.45	7.02	DNAJC18	DnaJ homolog subfamily C member 18	NM_001113588.1
A20G0392	2.45	2.93	LRATD1	Protein LRATD1	NM_200618.1
A15G0881	2.45	13.14	ELN	Elastin	XM_017351026.3
B16G0476	2.44	1.20	A0A672JY48	A0A672JY48_SINGR Zgc:194210	
A1G0004	2.44	2.66	ZBTB45	Zinc finger and BTB domain-containing protein 45	NM_205706.1
B8G0168	2.44	3.47	KCTD9	POZ domain-containing protein KCTD9	NM_001013567.3
B5G0439	2.44	1.63	MIS12	Protein MIS12 homolog	NM_200378.1
A19G0779	2.44	4.38	STARD3	StAR-related lipid transfer protein 3	XM_005159353.5
A18G0088	2.44	3.40	QSER1	Glutamine and serine-rich protein 1	XM_005169967.5
A2G0282	2.44	4.21	SLC35D1	UDP-N-acetylgalactosamine transporter	NM_200475.2
B19G0804	2.44	2.13	MTX1	Metaxin-1	NM_001007280.1
B7G0242	2.44	4.54	ANKRD11	Ankyrin repeat domain-containing protein 11	XM_068222739.1
A16G0616	2.44	3.43	NFKBID	NF-kappa-B inhibitor delta	NM_001435885.1
A24G0134	2.44	3.81	LOC107677312	A0A671LJD7_9TELE Uncharacterized LOC107677312	XM_695016.9
A4G0099	2.44	9.25	NLRC3	NLR family CARD domain-containing protein 3	XM_009291920.4
B17G0625	2.44	1.49	CCDC28A	Coiled-coil domain-containing protein 28A	XM_005158720.5
A11G0429	2.44	1.78			
B14G0844	2.44	2.21	CYSLTR1	Cysteinyl leukotriene receptor 1	NM_001423673.1
B22G0816	2.44	8.18	B3GALT2	Beta-1,3-galactosyltransferase 2	NM_207101.1
A6G0301	2.44	2.57	MRPS9	28S ribosomal protein S9, mitochondrial	NM_001045396.1
B20G0140	2.44	3.57	GNPAT	Dihydroxyacetone phosphate acyltransferase	XM_002665648.7
A9G0351	2.44	3.24	GPR18	N-arachidonyl glycine receptor	XM_005167779.4
A11G0655	2.44	3.30	CSE1L	Exportin-2	NM_001326456.1
B19G0231	2.44	5.35	ZNF687B	Zinc finger protein 687b	NM_001347673.1
A15G0732	2.44	6.53	SIRT2	NAD-dependent protein deacetylase sirtuin-2	NM_199596.1
B12G0702	2.44	2.82	CEP55	Centrosomal protein of 55 kDa	NM_001020762.1
A11G0639	2.44	4.49	AKR7A2	Aflatoxin B1 aldehyde reductase member 2	NM_001034175.2
B2G0719	2.44	6.72	UROD	Uroporphyrinogen decarboxylase	NM_131347.1
B2G0683	2.44	0.51	ROHU_033253	A0A498LC36_LABRO Transmembrane mitochondrial-like isoform X1	

B24G0727	2.44	4.30	CDK13	Cyclin-dependent kinase 13	NM_001366829.1
A5G0220	2.44	4.89	TXNRD2	Thioredoxin reductase 2, mitochondrial	NM_001353394.1
B24G0388	2.44	2.67	LOC113042430	A0A6P6J9K1_CARAU zinc finger protein 112-like	
B10G0853	2.44	2.61	NHLRC3	NHL repeat-containing protein 3	NR_023334.2
B14G0442	2.44	3.22	ETFDH	Electron transfer flavoprotein-ubiquinone oxidoreductase, mitochondrial	NM_001431298.1
A23G0094	2.44	2.92	RABGGTA	Geranylgeranyl transferase type-2 subunit alpha	NM_001076663.2
B6G0953	2.44	0.77	SPDEF	SAM pointed domain-containing Ets transcription factor	XM_001343041.6
A12G0263	2.44	12.14	CASP7	Caspase-7	NM_001020607.2
A18G0150	2.44	1.44	PGRP-SC2	Peptidoglycan-recognition protein SC2	NM_001044321.1
A3G0103	2.44	3.17			
A3G0629	2.43	1.78	THRAA	Thyroid hormone receptor alpha-A	XM_009299516.4
B9G0529	2.43	13.84	ZEB2	Zinc finger E-box-binding homeobox 2	NM_001114551.2
A9G0047	2.43	1.72	TLR8	Toll-like receptor 8	XM_001920559.7
B22G0725	2.43	8.40	FSTL3	Follistatin-related protein 3	NM_001030111.2
A6G0496	2.43	7.87	AMOTL2A	Angiomotin-like 2a	NM_001080177.2
B2G0445	2.43	12.22	IFI44L	Interferon-induced protein 44-like	NM_001077169.2
B10G0692	2.43	3.68	TCF7L1A	Transcription factor 7-like 1-A	NM_131269.2
B2G1075	2.43	9.88	PLSCR1	Phospholipid scramblase 1	NM_001128395.1
B10G0776	2.43	7.72	PDE4D	cAMP-specific 3',5'-cyclic phosphodiesterase 4D	XM_021474146.2
B14G0284	2.43	0.94	FOXI1	Forkhead box protein I1	NM_198918.1
A13G0418	2.43	3.31	EEF1A1	Elongation factor 1-alpha 1	NM_001128351.1
B3G0940	2.43	5.53	KMT5C	Histone-lysine N-methyltransferase KMT5C	NM_001098186.2
A7G0064	2.43	6.68	H4	Histone H4	XM_068222533.1
A25G0701	2.43	2.23	MAF	Transcription factor Maf	NM_001316669.2
A10G0076	2.43	2.63	CCAR2	Cell cycle and apoptosis regulator protein 2	XM_068223884.1
A25G0518	2.43	2.20	RORAA	Nuclear receptor ROR-alpha A	XM_017354088.3
A25G0090	2.43	1.49	AP4E1	AP-4 complex subunit epsilon-1	XM_693950.8
A16G0812	2.43	0.87	SP8B	Transcription factor Sp8	XM_005157958.5
A12G0121	2.43	13.81	KIF5B	Kinesin-1 heavy chain	XM_002664019.6
B19G0830	2.43	4.21	CTDP1	RNA polymerase II subunit A C-terminal domain phosphatase	NM_001002873.1
B21G0052	2.43	0.03	MX2	Interferon-induced GTP-binding protein Mx2	XM_690470.10
A20G0209	2.43	7.73	AFDN	Afadin	XM_068215697.1
B6G0271	2.43	7.45	BMPR2	Bone morphogenetic protein receptor type-2	XM_005165835.5
A15G0022	2.43	4.15	GSONMT00016217001	A0A060ZE97_ONCMY TGFb_propeptide domain-containing protein (Fragment)	
B2G0963	2.43	1.84	NSUN4	5-methylcytosine rRNA methyltransferase NSUN4	NM_001008649.1
A19G0577	2.43	5.64	TSHZ1	Teashirt homolog 1	XM_068215044.1
B22G0819	2.43	4.49	RGS2	Regulator of G-protein signaling 2	
B23G0673	2.43	7.00	POU6F1	POU domain, class 6, transcription factor 1	XM_068216663.1

B5G1069	2.42	1.36	LOC113049897	A0A6P6K7P9_CARAU uncharacterized protein LOC113049897	NM_001098191.1
A9G0821	2.42	4.38	UBR3	E3 ubiquitin-protein ligase ubr3	XM_021478823.2
B1G0046	2.42	28.08	ADAMTS1	A disintegrin and metalloproteinase with thrombospondin motifs 1	XM_021475923.2
A22G0251	2.42	3.24	BCOR	BCL-6 corepressor	XM_005173943.5
B24G0529	2.42	2.15	MTRR	Methionine synthase reductase	XM_005162620.4
A16G0702	2.42	9.12	CIC	Protein capicua homolog	XM_002664811.7
B17G0577	2.42	2.29	RPUSD2	Pseudouridylate synthase RPUSD2	NM_001327917.1
A19G0358	2.42	0.71	SMIM12	Small integral membrane protein 12	NM_001013528.3
B7G0098	2.42	1.98	NSUN6	tRNA (cytosine(72)-C(5))-methyltransferase NSUN6	
A21G0363	2.42	1.10	EFNA5B	Ephrin-A5b	XM_001337352.8
B25G0249	2.42	4.59	FRIH	Ferritin, heavy subunit	NM_001126392.1
A18G0154	2.42	10.81	AKT2	threonine-protein kinase	NM_198146.2
B2G0976	2.42	0.78	GFI1	Zinc finger protein Gfi-1	XM_068224140.1
B21G0382	2.42	2.65	RIN2	Ras and Rab interactor 2	XM_021473434.2
A4G0706	2.42	3.34	PRICKLE1	Prickle-like protein 1	NM_001030098.2
A16G0511	2.42	2.11	GLIPR2	Golgi-associated plant pathogenesis-related protein 1	NM_001005978.1
B7G0366	2.42	3.28	ARAP2	Arf-GAP with Rho-GAP domain, ANK repeat and PH domain-containing protein 2	XM_068222759.1
B16G1057	2.42	0.12	RTASE	Probable RNA-directed DNA polymerase from transposon BS	XR_011015637.1
B23G0513	2.42	1.72	MAGI3	Membrane-associated guanylate kinase, WW and PDZ domain-containing protein 3	XM_021469973.2
A13G0047	2.42	6.16	LIN1	LINE-1 reverse transcriptase homolog	XM_009307044.4
B7G0594	2.42	3.73	RHPN2	Rhophilin-2	NM_201178.1
A9G0720	2.42	6.97	AKAP11	A-kinase anchor protein 11	XM_068223573.1
A2G0450	2.42	2.49	ZNF644	Zinc finger protein 644	NM_001278254.1
B3G1354	2.42	19.79	GIMAP9	GTPase IMAP family member 9	
A2G0520	2.42	5.68	MAST3	threonine-protein kinase 3	XM_021471251.2
B10G0164	2.42	5.26	ARL15	ADP-ribosylation factor-like protein 15	XM_021472227.2
A13G0234	2.42	1.39	KCNK1	Potassium channel subfamily K member 1	XM_009307225.4
A7G1175	2.42	7.61	FAH	Fumarylacetoacetase	NM_199601.1
B2G0152	2.42	2.32	XCR1	Chemokine XC receptor 1	
A20G0778	2.42	7.97	YLPM1	YLP motif-containing protein 1	XM_003200679.6
A18G0806	2.42	0.65	SLC27A2	Long-chain fatty acid transport protein 2	NM_001083019.1
B5G0940	2.42	15.31	ATP2A2	endoplasmic reticulum calcium ATPase 2	XM_692016.10
B3G0921	2.42	1.24	ITPRIPL2	Inositol 1,4,5-trisphosphate receptor-interacting protein-like 2	XM_068219060.1
A18G0698	2.42	2.62	COG8	Conserved oligomeric Golgi complex subunit 8	NM_001013506.1
B12G0290	2.42	4.87	PHKG2	testis isoform	NM_200962.1
A22G0114	2.42	7.82	DPX16_4881	A0A3N0YIX4_ANAGA CD48 antigen	XR_002456444.2
B24G0082	2.42	1.06	IGKV1-39	Immunoglobulin kappa variable 1-39	NM_001045408.1
B2G0356	2.42	2.50	SPSB4	SPRY domain-containing SOCS box protein 4	NM_001256642.1

B20G0723	2.42	5.63	MAN1A1	Mannosyl-oligosaccharide 1,2-alpha-mannosidase IA	XM_689343.10
B12G0061	2.42	0.47	ZCCHC24	Zinc finger CCHC domain-containing protein 24	
A25G0210	2.42	1.27	ZCCHC14	Zinc finger CCHC domain-containing protein 14	XM_001336490.8
A21G0795	2.42	3.03	RBM27	RNA-binding protein 27	XM_005167934.5
A3G0398	2.41	4.86	DNMT1	DNA (cytosine-5)-methyltransferase 1	NM_131189.2
A21G0195	2.41	0.97	CCBE1	Collagen and calcium-binding EGF domain-containing protein 1	NM_001163923.1
A20G0383	2.41	6.46	ROCK2	Rho-associated protein kinase 2	XR_011007666.1
B11G0040	2.41	8.87	X-ELEMENT\ORF2	Probable RNA-directed DNA polymerase from transposon X-element	NM_001080642.1
A6G0523	2.41	7.96	TMEM131	Transmembrane protein 131	XM_001923305.7
A2G0417	2.41	5.56	TPR	Nucleoprotein TPR	XM_009298498.4
B10G0085	2.41	2.76	SIGMAR1	Sigma non-opioid intracellular receptor 1	NM_200977.1
A8G0822	2.41	1.14	CCN1	CCN family member 1	
B17G0314	2.41	0.18	CYP26B1	Cytochrome P450 26B1	NM_001029951.2
B6G0179	2.41	2.22	WIZ	Protein Wiz	XM_068221883.1
B21G0689	2.41	4.86	PNPLA7	Patatin-like phospholipase domain-containing protein 7	XM_687184.9
B9G0415	2.41	2.58	CRYL1	Lambda-crystallin	NM_001045341.1
B7G0361	2.41	2.06	CCKAR	Cholecystokinin receptor type A	XM_692401.8
A12G0295	2.41	4.35	ZNF646	Zinc finger protein 646	XM_684608.9
B14G0627	2.41	5.62	TBC1D9B	TBC1 domain family member 9B	XM_009307655.4
B22G0930	2.41	2.92	CBX6	Chromobox protein homolog 6	XM_021469381.2
B22G0937	2.41	0.78	ADRA2A	Alpha-2A adrenergic receptor	NM_207637.3
B24G0033	2.41	2.74	TRIM25	ISG15 ligase TRIM25	XM_681071.8
A4G1031	2.41	3.67	SI:CH211-152C2.3	F1RAX9_DANRE Si:ch211-152c2.3 (Fragment)	
A2G0409	2.41	6.25	AGL	Glycogen debranching enzyme	XM_068224754.1
A1G0113	2.41	0.36	ZNF750	Zinc finger protein 750	
A13G0203	2.41	13.85	DMAC2L	ATP synthase subunit s, mitochondrial	XM_692747.9
A13G0638	2.41	1.64	EIF2AK3	Eukaryotic translation initiation factor 2-alpha kinase 3	NM_001008640.1
A7G0491	2.41	4.82	POP4	Ribonuclease P protein subunit p29	NM_001020749.2
A2G0557	2.41	3.18	NDC1	Nucleoporin NDC1	NM_199943.1
A5G0912	2.41	4.21	NAAA	N-acylethanolamine-hydrolyzing acid amidase	
B6G0153	2.41	6.61	ARF3	ADP-ribosylation factor 3	NM_001012248.2
B13G0298	2.41	2.83	RRH	Visual pigment-like receptor peropsin	NM_001145591.1
B25G0336	2.41	2.32	SH2D7	SH2 domain-containing protein 7	
A25G0118	2.41	5.20	SV2B	Synaptic vesicle glycoprotein 2B	XM_009297765.3
B24G0470	2.41	1.75	LOC113041890	A0A6P6JE85_CARAU uncharacterized protein LOC113041890	XM_009297410.4
B2G0700	2.41	3.49	PAXIP1	PAX-interacting protein 1	NM_001005929.2
A10G0711	2.41	3.86	CSGALNACT1	Chondroitin sulfate N-acetylgalactosaminyltransferase 1	XM_001333443.8
A22G0343	2.41	5.66	OSBPL1A	Oxysterol-binding protein-related protein 1	

A10G0888	2.41	19.09	SLC27A6	Long-chain fatty acid transport protein 6	
B24G0191	2.41	1.56	PUS3	39) synthase	NM_200067.1
B1G0396	2.41	3.45	NCAPG	Condensin complex subunit 3	NM_001256205.1
A25G0451	2.40	31.85	NPTN	Neuroplastin	XM_068217331.1
A13G0088	2.40	14.48	OLIG3	Oligodendrocyte transcription factor 3	
A4G0865	2.40	2.14	TMEM168A	Transmembrane protein 168-A	NM_001083086.1
B19G0581	2.40	2.96	PHF14	PHD finger protein 14	NM_001423773.1
A1G0410	2.40	3.86	DTX4	E3 ubiquitin-protein ligase DTX4	XM_002660524.6
A2G0613	2.40	3.06	SOCS6	Suppressor of cytokine signaling 6	NM_199625.2
A3G0183	2.40	3.84	CBX2	Chromobox protein homolog 2	NM_194373.2
A21G0301	2.40	3.33	AQP3	Aquaporin-3	NM_001166121.1
B9G0668	2.40	1.25	PRKAG3	5'-AMP-activated protein kinase subunit gamma-3	NM_001040366.2
A9G0125	2.40	4.14	TWIST2	Twist-related protein 2	NM_001005956.2
A8G0145	2.40	1.27	SSUH2	Protein SSUH2 homolog	
B21G0601	2.40	9.64	GPAT3	Glycerol-3-phosphate acyltransferase 3	NM_001002685.1
B4G0448	2.40	4.40	ITFG2	KICSTOR complex protein ITFG2	NM_001017908.2
B12G0063	2.40	4.95	DLG5	Disks large homolog 5	XM_021480451.2
B16G0022	2.40	3.66	TRIM16	Tripartite motif-containing protein 16	XM_681071.8
A22G0476	2.40	4.07	ESG1	Transducin-like enhancer protein 1	NM_001130604.2
B4G0516	2.40	1.94	CEP83	Centrosomal protein of 83 kDa	NM_001353931.1
A19G0926	2.40	5.47	RNF139	E3 ubiquitin-protein ligase RNF139	XM_005173717.5
B2G1050	2.40	1.05	FUZ	Protein fuzzy homolog	XM_068213439.1
A5G0624	2.40	2.25	DAB2IP	Disabled homolog 2-interacting protein	XM_005171881.5
A9G0618	2.40	5.59	ABI2	Abl interactor 2	XM_009304512.4
A15G0250	2.40	3.29	ANKMY2	Ankyrin repeat and MYND domain-containing protein 2	NM_199894.1
B25G0621	2.40	3.11	CCL14	C-C motif chemokine 14	NM_001129894.1
B23G0900	2.40	2.11	TRIM27	Zinc finger protein RFP	NM_001040374.2
A20G0111	2.40	2.07	INSM1A	Insulinoma-associated protein 1a	NM_205644.1
B22G1094	2.40	2.27	TRAF5	TNF receptor-associated factor 5	XM_021469653.2
A4G0615	2.39	2.56	NCAPH2	Condensin-2 complex subunit H2	NM_001044991.1
B16G0061	2.39	2.40	KIF13A	Kinesin-like protein KIF13A	XM_021473036.1
A2G0997	2.39	10.31	CAMK2N2	calmodulin-dependent protein kinase II inhibitor 2	NM_001145089.2
B24G0366	2.39	2.00	CD274	Programmed cell death 1 ligand 1	
A12G0233	2.39	0.88	ANKFN1	Ankyrin repeat and fibronectin type-III domain-containing protein 1	XM_009306802.4
A22G0257	2.39	4.82	GNAO	Guanine nucleotide-binding protein G(o) subunit alpha	NM_001166014.1
B1G0414	2.39	1.54	SFRP2	Secreted frizzled-related protein 2	NM_001077384.2
B16G0274	2.39	0.63	LOC107717367	A0A673KTX0_9TELE Uncharacterized LOC107717367	XM_002664976.5
A17G0701	2.39	3.31	DACT1	Dapper homolog 1	NM_214731.3
B3G1050	2.39	2.74	ASPDH	Aspartate dehydrogenase domain-containing protein	XM_021470416.2

A8G0487	2.39	5.16	LOC113093903	A0A6P6P3H7_CARAU 4-(cytidine-5'-diphospho)-2-C-methyl-D-erythritol kinase	
B4G0056	2.39	5.27	A33	Zinc-binding protein A33	XM_001344987.7
B19G0256	2.39	3.35	SETDB1A	Histone-lysine N-methyltransferase SETDB1-A	NM_001044767.2
A1G0946	2.39	2.15	PGBD4	PiggyBac transposable element-derived protein 4	
B17G0444	2.39	7.02	OXR1	Oxidation resistance protein 1	XM_021473161.2
A22G0075	2.39	3.45	LOC113097005	A0A6P6PBS8_CARAU zinc finger protein 845-like	XM_068219202.1
B14G0718	2.39	3.63	SMTN	Smoothelin	NM_001013321.1
A12G0519	2.39	2.68	PMS2	Mismatch repair endonuclease PMS2	XM_688556.9
B1G0636	2.39	14.05	CANX	Calnexin	NM_001077541.1
B17G0827	2.39	0.91	CGAA	Glycoprotein hormones alpha chain 1	NM_205687.2
B7G0188	2.39	4.55	ADGRG3	Adhesion G protein-coupled receptor G3	
A5G0369	2.39	2.64	MTMR2	Myotubularin-related protein 2	NM_131371.1
A21G0075	2.39	5.86	SMAD7	Mothers against decapentaplegic homolog 7	NM_175082.3
A10G0118	2.39	1.49	SNRNP35	U12 small nuclear ribonucleoprotein 35 kDa protein	
B7G0482	2.39	4.08	CMTM3	CKLF-like MARVEL transmembrane domain-containing protein 3	NM_001003468.2
B25G0514	2.39	5.50	VAC14	Protein VAC14 homolog	XM_009297900.4
B10G0794	2.39	6.98	TOR4A	Torsin-4A	
A10G0417	2.39	4.32	CUX1	Homeobox protein cut-like 1	XM_005172719.5
A23G0382	2.39	1.80	IFFO2	Intermediate filament family orphan 2	XM_021473643.2
A14G0651	2.39	2.99	HMGXB3	HMG domain-containing protein 3	XM_005169574.5
A4G0674	2.39	3.23	MEST	Mesoderm-specific transcript homolog protein	NM_001328532.1
B8G0447	2.38	0.32	ATOH1	Transcription factor ATOH1	NM_131091.2
B13G0184	2.38	2.08	PRKCE	Protein kinase C epsilon type	NM_001167958.1
B24G0452	2.38	2.18	SPICE1	Spindle and centriole-associated protein 1	XM_005162693.5
B2G0429	2.38	10.00	SEC62	Translocation protein SEC62	NM_001025530.1
A5G0531	2.38	1.84	STK36	threonine-protein kinase 36	XM_009301536.3
A4G0926	2.38	6.92	BCL2L14	Apoptosis facilitator Bcl-2-like protein 14	
B18G0110	2.38	3.58	PRDM10	PR domain zinc finger protein 10	NM_001347687.1
A7G0480	2.38	3.38	ZNF507	Zinc finger protein 507	XM_005166460.5
A24G0651	2.38	5.24	CYB5A	Cytochrome b5	NM_213135.2
A8G0792	2.38	8.94	ELMOD3	ELMO domain-containing protein 3	XM_003199132.6
B25G0290	2.38	5.31	TJP1	Tight junction protein ZO-1	XM_009298141.4
B3G1450	2.38	23.25	RNF213A	E3 ubiquitin-protein ligase rnf213-alpha	NM_001353835.1
A15G0032	2.38	2.23	CEP57	Centrosomal protein of 57 kDa	NM_001080683.1
A4G0729	2.38	0.59	LOC113048604	A0A6P6K262_CARAU uncharacterized protein LOC113048604	NM_001123239.1
A3G0229	2.38	1.69	ABCA3	Phospholipid-transporting ATPase ABCA3	XM_068218749.1
A17G0070	2.38	4.78	KIZ	Centrosomal protein kizuna	XM_005158902.5

A21G0697	2.38	4.83	RTASE	Probable RNA-directed DNA polymerase from transposon BS	XM_068220297.1
B16G0098	2.38	1.26			XM_009292700.4
B17G0415	2.38	5.67	SANBR	SANT and BTB domain regulator of class switch recombination	XR_011007000.1
A10G0431	2.38	4.79	UVRAG	UV radiation resistance-associated gene protein	XM_005172655.3
A11G0485	2.38	4.07	IER2	Immediate early response gene 2 protein	XM_689398.9
B24G0370	2.38	2.74	AMER2	APC membrane recruitment protein 2	NM_001100152.1
B5G0974	2.38	5.86	ZBTB44	Zinc finger and BTB domain-containing protein 44	NM_001144798.1
B4G0588	2.38	4.13	METAP2	Methionine aminopeptidase 2	NM_001100054.1
A5G0388	2.38	3.13	CAPG	Macrophage-capping protein	NM_001289065.1
B21G0784	2.38	5.95	AGPAT1	1-acyl-sn-glycerol-3-phosphate acyltransferase alpha	NM_001077732.1
B20G0538	2.38	6.01	SASH1	SAM and SH3 domain-containing protein 1	XM_021468433.2
B18G0643	2.38	9.79	TBXAS1	Thromboxane-A synthase	NM_205609.2
A16G0526	2.38	3.98	TMEM245	Transmembrane protein 245	XM_001340361.9
B18G0767	2.38	1.67	RELT	Tumor necrosis factor receptor superfamily member 19L	NM_207073.2
A13G0738	2.38	3.14	PROP1	Homeobox protein prophet of Pit-1	NM_001145554.1
B22G0645	2.38	2.16	STIL	SCL-interrupting locus protein homolog	NM_173244.2
B2G0639	2.38	0.99	ACKR4	Atypical chemokine receptor 4	
B9G0666	2.38	3.20	LOC113108865	A0A6P6Q328_CARAU uncharacterized protein LOC113108865 isoform X1	XM_005167514.5
A17G0809	2.37	0.18	RAB39A	Ras-related protein Rab-39A	XM_021473050.2
B5G1060	2.37	1.56	GTF2H2	General transcription factor IIH subunit 2	NM_201581.1
A24G0432	2.37	5.55	MAP3K3	Mitogen-activated protein kinase kinase kinase 3	NM_001423668.1
A17G0478	2.37	3.42	B3GNT2	N-acetyllactosaminide beta-1,3-N-acetylglucosaminyltransferase 2	NM_205752.2
A1G1032	2.37	6.71	MSMO1	Methylsterol monooxygenase 1	NM_213353.1
B3G0195	2.37	0.00	ROHU_024522	A0A498MLB4_LABRO Uncharacterized protein	
A10G0562	2.37	13.91	RTASE	Probable RNA-directed DNA polymerase from transposon BS	XM_068218007.1
A24G0588	2.37	3.79	GALNT11	Polypeptide N-acetylgalactosaminyltransferase 11	NM_001076562.2
A22G0347	2.37	1.54	VPS4B	Vacuolar protein sorting-associated protein 4B	
B8G0747	2.37	1.66	POLA2	DNA polymerase alpha subunit B	NM_199581.2
A2G0559	2.37	4.89	YIPF1	Protein YIPF1	NM_001003991.1
A13G0292	2.37	6.98	C2ORF50	Uncharacterized protein C2orf50	NM_205603.1
A20G0518	2.37	0.40	STX11	Syntaxin-11	NM_001105602.1
A8G0755	2.37	1.84	RENBP	N-acylglucosamine 2-epimerase	NM_001126443.1
B25G0665	2.37	3.02	AP3S2	AP-3 complex subunit sigma-2	NM_001002539.1
A7G1033	2.37	4.79	TTC5	Tetratricopeptide repeat protein 5	NM_001017654.2
B14G0152	2.37	1.21	ZGC:153146	UPF0602 protein C4orf47 homolog	XM_009291135.4

B7G1093	2.37	1.84	DOCK11	Dedicator of cytokinesis protein 11	XM_009303084.4
B6G0881	2.37	8.71	GNAS	Guanine nucleotide-binding protein G(s) subunit alpha	XM_001335696.7
A22G0709	2.37	3.47	PTK7	Inactive tyrosine-protein kinase 7	NM_001020665.1
A7G0676	2.37	5.72	MAP2K5	Dual specificity mitogen-activated protein kinase kinase 5	NM_001114317.1
A21G0487	2.37	2.74	DIXDC1	Dixin	XM_005161265.5
A19G0694	2.37	13.38	SNX27	Sorting nexin-27	NM_001045100.1
A22G0097	2.37	7.28	RTASE	Probable RNA-directed DNA polymerase from transposon BS	
A14G0618	2.37	4.77	TBC1D9B	TBC1 domain family member 9B	XM_009307655.4
B1G0430	2.37	2.43	TOPORS	E3 ubiquitin-protein ligase Topors	NM_001305555.1
A21G0485	2.37	9.54	JAM3B	Junctional adhesion molecule 3B	NM_001082863.3
B4G0257	2.37	5.97	TMEM243	Transmembrane protein 243	NM_212919.1
A1G0895	2.37	2.89	BRAT1	BRCA1-associated ATM activator 1	XM_021475839.2
A16G0483	2.37	5.96	GON4L	GON-4-like protein	NM_001201535.1
B3G1281	2.37	4.50			
A12G0463	2.37	11.28	TNRC6B	Trinucleotide repeat-containing gene 6B protein	XM_068224758.1
B4G0349	2.37	1.10	IFNG1R	Interferon gamma-related	
A20G0558	2.37	1.62	EPHA7	Ephrin type-A receptor 7	XM_021468464.2
B7G0102	2.37	4.06	SMCHD1	Structural maintenance of chromosomes flexible hinge domain-containing protein 1	
A13G0879	2.37	59.24	POL	RNA-directed DNA polymerase from mobile element jockey	
A2G0151	2.37	5.86	PDE4B	cAMP-specific 3',5'-cyclic phosphodiesterase 4B	XM_009298245.4
A21G0060	2.36	3.41	CD274	Programmed cell death 1 ligand 1	
A7G0485	2.36	0.94	CCNE1	S-specific cyclin-E1	NM_130995.1
A8G0593	2.36	0.35			
B6G0594	2.36	5.72	UHMK1	threonine-protein kinase Kist	NM_001076659.2
A16G0776	2.36	2.45	POLR3C	DNA-directed RNA polymerase III subunit RPC3	NM_200232.1
A14G0612	2.36	9.28	SRP72	Signal recognition particle subunit SRP72	XM_017359078.3
B24G0374	2.36	3.86	ZDHHC20B	Palmitoyltransferase ZDHHC20-B	NM_001423810.1
A19G0149	2.36	5.94	PEX11B	Peroxisomal membrane protein 11B	NM_001045854.3
B16G0842	2.36	4.31	ITGA10	Integrin alpha-10	XM_005157916.3
A12G0779	2.36	8.14	VEZF1	Vascular endothelial zinc finger 1	XM_001918941.8
B8G0929	2.36	0.88	KRT8	Keratin, type II cytoskeletal 8	XM_003199050.6
B19G0050	2.36	6.59	SP4	Transcription factor Sp4	NM_200124.1
A4G0025	2.36	2.17	DPX16_1179	A0A3N0XUS6_ANAGA Zinc finger protein 569	XM_009300923.3
A16G0424	2.36	2.11	C1S	Complement C1s subcomponent	
A5G0566	2.36	7.08	SLC25A25	Calcium-binding mitochondrial carrier protein SCaMC-2	XM_009301543.4
A5G0080	2.36	12.78	ZFY1	Zinc finger Y-chromosomal protein 1	XM_005170809.5
B17G0155	2.36	3.84	EDRF1	Erythroid differentiation-related factor 1	XM_021473083.2
B16G0816	2.36	9.03	HLF	Hepatic leukemia factor	XM_021472965.2

A24G0546	2.36	6.53	RTASE	Probable RNA-directed DNA polymerase from transposon BS	
A5G1182	2.36	1.85	RTEL1	Regulator of telomere elongation helicase 1	NM_001013310.2
A3G1301	2.36	5.58	RTASE	Probable RNA-directed DNA polymerase from transposon BS	
A4G0950	2.36	3.14	FGD4	FYVE, RhoGEF and PH domain-containing protein 4	XM_021475195.2
B14G0753	2.36	18.36	POL	Retrovirus-related Pol polyprotein from transposon opus	
A4G0452	2.36	6.89	CDKN1B	Cyclin-dependent kinase inhibitor 1B	NM_001281477.1
B5G0448	2.36	4.77	CLIP2	CAP-Gly domain-containing linker protein 2	XM_679197.9
B24G0383	2.36	3.17	ARMC1	Armadillo repeat-containing protein 1	NM_001008609.1
A22G0461	2.36	4.40	ELL	RNA polymerase II elongation factor ELL	NM_199707.1
A24G0203	2.36	5.93	LOC107599662	A0A672RMV3_SINGR Uncharacterized LOC107599662	XM_002660922.7
A10G0090	2.36	1.04	TOR4A	Torsin-4A	NM_001017561.3
B3G1181	2.36	13.19	DESI1	Desumoylating isopeptidase 1	NM_200700.1
B15G0263	2.36	7.05	PRKD2	threonine-protein kinase D2	XM_021466325.2
B22G0783	2.36	1.57	SHD	SH2 domain-containing adapter protein D	XM_021469342.2
A20G0825	2.36	1.31	CCDC85CB	Coiled-coil domain-containing protein 85C-B	NM_001002628.1
A9G0246	2.36	12.37	RCAN1	Calcipressin-1	NM_001003616.1
A10G0591	2.36	0.56	CNFN-B	Cornifelin homolog B	
B13G0656	2.36	4.53	WDR27	WD repeat-containing protein 27	XM_685283.9
A13G0498	2.36	1.20	RGS17	Regulator of G-protein signaling 17	XM_021480847.2
B23G0200	2.36	14.12	G6PD	Glucose-6-phosphate 1-dehydrogenase	XM_021474503.2
B1G1039	2.36	2.18	B3GNTL1	UDP-GlcNAc:betaGal beta-1,3-N-acetylglucosaminyltransferase-like protein 1	NM_001423740.1
B7G0834	2.35	4.49	SNO1	Senecionine N-oxygenase	NM_001197289.1
B25G0840	2.35	2.02			
B18G0839	2.35	1.84	DTWD1	tRNA-uridine aminocarboxypropyltransferase 1	NM_001005951.2
B3G1116	2.35	14.27	UBTF	Nucleolar transcription factor 1	NM_001005395.1
B3G1249	2.35	2.69	TANC2	Protein TANC2	XM_068218910.1
B17G0750	2.35	3.70	EGR2B	Early growth response protein 2b	NM_001328404.2
A6G0663	2.35	4.25	DIP2BA	Disco-interacting protein 2 homolog B-A	XM_009302576.4
B23G0721	2.35	0.83	HOXC3A	Homeobox protein Hox-C3a	NM_001134685.2
A23G0316	2.35	1.34	EBP	3-beta-hydroxysteroid-Delta(8),Delta(7)-isomerase	NM_001002328.1
A17G0605	2.35	3.33	FOXN3	Forkhead box protein N3	NM_001045263.2
B17G0267	2.35	16.61	MARCKS	Myristoylated alanine-rich C-kinase substrate	NM_001015060.2
A8G0937	2.35	6.84	PRRT3	Proline-rich transmembrane protein 3	XM_021478487.2
A22G0542	2.35	3.53	KAT14	Cysteine-rich protein 2-binding protein	NM_001002699.3

B22G0329	2.35	1.82	LOC113080627	A0A6P6NJD1_CARAU uncharacterized protein LOC113080627	XM_005161637.5
B1G0448	2.35	13.32	SH3GL2	Endophilin-A1	NM_201116.1
B23G0468	2.35	2.00	MLH3	DNA mismatch repair protein Mlh3	XM_005162210.5
A8G0701	2.35	3.46	ASPHD2	Aspartate beta-hydroxylase domain-containing protein 2	NM_001080045.1
B18G0550	2.35	8.76	ZC3H18	Zinc finger CCCH domain-containing protein 18	XM_068214774.1
B9G0642	2.35	2.10	TRA2B	Transformer-2 protein homolog beta	XM_005167474.5
A5G0533	2.35	3.70	PHLDB1	Pleckstrin homology-like domain family B member 1	XM_009301573.4
A1G0084	2.35	4.35	PIGR	Polymeric immunoglobulin receptor	XM_003197773.6
A23G0560	2.35	0.21	FOXL2	Forkhead box protein L2	NM_001128810.1
B23G0207	2.35	3.61	BIRC7-A	Baculoviral IAP repeat-containing protein 7-A	NM_001430917.1
B12G0042	2.34	5.62	DNAJB12	DnaJ homolog subfamily B member 12	NM_001089508.1
A6G0143	2.34	11.71	MYH10	Myosin-10	XM_009302145.4
A11G0636	2.34	3.20	PARK7	Parkinson disease protein 7 homolog	NM_001017716.2
B20G0704	2.34	4.24	TPD52L1	Tumor protein D53 homolog	XM_005160819.5
B22G0424	2.34	2.20	LOC113099123	A0A6P6PH34_CARAU uncharacterized protein LOC113099123	XM_068216574.1
B14G0443	2.34	8.43	ADAMTS2	A disintegrin and metalloproteinase with thrombospondin motifs 2	XM_009303692.4
B20G0501	2.34	2.39	TAF1B	TATA box-binding protein-associated factor RNA polymerase I subunit B	NM_001025298.1
A5G1110	2.34	2.90	GNAQ	Guanine nucleotide-binding protein G(q) subunit alpha	NM_001144799.1
B10G0666	2.34	6.35	AAK1	AP2-associated protein kinase 1	XM_009305653.4
A5G0586	2.34	7.90	DAB2	Disabled homolog 2	NM_205757.3
A6G0516	2.34	3.33	ZBTB11	Zinc finger and BTB domain-containing protein 11	NM_212650.2
A13G0675	2.34	3.66	TLR4	Toll-like receptor 4	XM_009307500.4
B8G0414	2.34	2.56	CARF	Calcium-responsive transcription factor	NM_001386261.1
A14G0472	2.34	11.80	PDLIM7	PDZ and LIM domain protein 7	NM_200840.2
A5G0196	2.34	34.75	CCL19	C-C motif chemokine 19	
B3G0364	2.34	3.04	WIZ	Protein Wiz	XM_009299233.4
A14G0477	2.34	3.24	FAM193B	Protein FAM193B	XM_692607.9
A17G0580	2.34	0.58	CYP26B1	Cytochrome P450 26B1	NM_001029951.2
A17G0205	2.34	1.68	LOC113060461	A0A6P6LKR7_CARAU uncharacterized protein LOC113060461	XM_700969.7
A8G0035	2.34	6.87	PLPP1	Phospholipid phosphatase 1	XM_001334553.9
A24G0007	2.34	0.90	AHRR	Aryl hydrocarbon receptor repressor	NM_001035265.1
B23G0832	2.34	3.18	PODN	Podocan	XM_021473738.2
B2G0340	2.34	5.41	ACVR1	Activin receptor type-1	NM_131345.1
B19G0968	2.34	2.64	ANKLE1	Ankyrin repeat and LEM domain-containing protein 1	
B1G0475	2.34	5.15	CBS	Cystathionine beta-synthase	NM_001111232.1
A4G0102	2.34	0.98	CD209C	CD209 antigen-like protein C	XM_068218585.1
A4G0750	2.34	4.87	MKRN1	Probable E3 ubiquitin-protein ligase makorin-1	NM_152978.1

A22G0319	2.34	9.76	LPL	Lipoprotein lipase	NM_131127.1
A17G0817	2.34	22.19	NAPB	Beta-soluble NSF attachment protein	NM_001020655.2
A7G0548	2.34	8.13			
A20G0925	2.34	2.23	BTBD9	POZ domain-containing protein 9	NM_001002198.1
A8G0801	2.34	0.90	NUDT18	8-oxo-dGDP phosphatase NUDT18	XM_005172467.4
A7G0847	2.34	1.57	HISAT	Histidine N-acetyltransferase	NM_001123054.2
B3G0228	2.33	9.41	PRR36	Proline-rich protein 36	XM_021472648.2
A16G0816	2.33	5.41	HDAC9B	Histone deacetylase 9-B	XM_005157964.4
B5G0358	2.33	3.26	SLC30A5	Zinc transporter 5	NM_001002322.1
A15G0620	2.33	1.47	RIMBP2	RIMS-binding protein 2	NM_001386253.1
B21G0275	2.33	5.85	TAOK1-B	threonine-protein kinase TAO1-B	XM_009295669.4
A5G0259	2.33	5.30	ATP8B2	Phospholipid-transporting ATPase ID	XM_021476431.2
B13G0046	2.33	6.41	EFEMP1	EGF-containing fibulin-like extracellular matrix protein 1	XM_009307601.4
B12G0712	2.33	14.52	MAPT	Microtubule-associated protein tau	XM_068224694.1
A3G1087	2.33	2.74	FOXD3-A	Forkhead box protein D3-A	NM_001089488.1
B10G0351	2.33	10.43	PAXBP1	PAX3- and PAX7-binding protein 1	NM_001080183.2
A25G0782	2.33	2.33	FANCA	Fanconi anemia group A protein	
A2G1078	2.33	4.57	ZCCHC2	Zinc finger CCHC domain-containing protein 2	XM_005168439.5
B14G0796	2.33	8.06	YRDC	Threonylcarbamoyl-AMP synthase	NM_001305473.1
A1G0249	2.33	3.25	LOC107594297	A0A672RNM7_SINGR Interleukin	NM_001039565.2
B7G0618	2.33	9.50	CHD9	Chromodomain-helicase-DNA-binding protein 9	XM_068218460.1
A1G1021	2.33	4.20	UBA6	Ubiquitin-like modifier-activating enzyme 6	XM_690663.9
A17G0675	2.33	3.82	LRRC57	Leucine-rich repeat-containing protein 57	NM_001002627.1
A7G1095	2.33	1.18	TRAV7	T cell receptor alpha variable 7	NM_001005577.1
B16G0544	2.33	3.82	NMT2	Glycylpeptide N-tetradecanoyltransferase 2	NM_001199754.1
A19G0674	2.33	6.39	KMT2B	Histone-lysine N-methyltransferase 2B	XM_005159485.5
A13G0064	2.33	1.43	MSH6	DNA mismatch repair protein Msh6	XM_068220571.1
B2G0818	2.33	2.76	CDYL	Chromodomain Y-like protein	XM_691787.9
A23G0077	2.33	4.74	PLEKHG1	Pleckstrin homology domain-containing family G member 1	XM_021469870.2
B25G0462	2.33	4.39	LYRM5B	LYR motif-containing protein 5B	NM_001159975.2
B2G0256	2.33	0.95	ACSM3	Acyl-coenzyme A synthetase ACSM3, mitochondrial	NM_001111236.1
A22G0686	2.33	0.55	ALPI	Intestinal-type alkaline phosphatase	NM_001014353.1
B19G0716	2.33	1.23	E2F3	Transcription factor E2F3	NM_001080628.2
B1G0900	2.33	1.39	APTX	Aprataxin	NM_214729.1
B2G0564	2.33	5.26	NMNAT2	nicotinic acid mononucleotide adenylyltransferase 2	NM_200004.1
B16G0113	2.33	1.25	ZNF385D	Zinc finger protein 385D	XM_021467038.2
A18G0066	2.33	6.66	IRF2BP1	Interferon regulatory factor 2-binding protein 1	NM_001044354.2
A12G0416	2.33	5.46	ORMDL3	ORM1-like protein 3	NM_001006087.1
B14G0431	2.33	3.49	LDB2	LIM domain-binding protein 2	XM_005157342.5

B23G0086	2.33	4.41	DHX35	Probable ATP-dependent RNA helicase DHX35	XM_068216829.1
B23G0092	2.32	4.30	NCOA6	Nuclear receptor coactivator 6	XM_001918962.8
B4G0783	2.32	0.23	G5714_004447	A0A7J6D5J8_9TELE Uncharacterized protein	XM_021475732.2
B14G0829	2.32	4.29	ST3GAL5	Lactosylceramide alpha-2,3-sialyltransferase	XM_005157139.5
B10G0326	2.32	7.29	PABIR1	P2R1A-PPP2R2A-interacting phosphatase regulator 1	XM_009305276.4
B13G0391	2.32	3.80	HNRNPH1	Heterogeneous nuclear ribonucleoprotein H	XM_005156935.5
A19G0748	2.32	1.53	EDN1	Endothelin-1	NM_131519.1
B10G0475	2.32	9.22	LHFPL6	LHFPL tetraspan subfamily member 6 protein	NM_001008653.1
B18G0607	2.32	2.45	CRY1	Cryptochrome-1	NM_131790.5
B11G0123	2.32	4.47	LHFPL5	LHFPL tetraspan subfamily member 5 protein	XM_005155707.5
B3G1330	2.32	5.06	LMTK2	threonine-protein kinase LMTK2	NM_001326472.1
B3G0983	2.32	8.01	RBFOX1	RNA binding protein fox-1 homolog 1	NM_001005596.1
B2G0347	2.32	0.45	CCL11	Eotaxin	
A21G0497	2.32	2.70	NFRKB	Nuclear factor related to kappa-B-binding protein	NM_001243035.1
B1G0695	2.32	1.78	LOC113095129	A0A6P6P6C0_CARAU PR domain zinc finger protein 1-like isoform X2	XM_001340227.8
B4G0576	2.32	4.29	SFMBT2	Scm-like with four MBT domains protein 2	XM_677950.9
B6G0761	2.32	3.51	SRGAP3	SLIT-ROBO Rho GTPase-activating protein 3	XM_005166115.5
B21G0610	2.32	3.09	SMPD4	Sphingomyelin phosphodiesterase 4	NM_213355.1
B17G0190	2.32	3.91	MGA	MAX gene-associated protein	XM_005158622.5
B23G0536	2.32	6.52	ASB8	Ankyrin repeat and SOCS box protein 8	NM_199770.1
B23G0878	2.32	17.48	ROHU_003822	A0A498NT53_LABRO Uncharacterized protein	
A15G0534	2.32	2.46	HINFP	Histone H4 transcription factor	NM_001017764.1
B16G0769	2.32	1.45	CEACAM5	Carcinoembryonic antigen-related cell adhesion molecule 5	XM_068213867.1
B14G0035	2.32	10.24	UBE2K	Ubiquitin-conjugating enzyme E2 K	NM_001013482.1
B17G0836	2.32	6.68	SCAF8	SR-related and CTD-associated factor 8	NM_001436747.1
B23G0376	2.32	24.69	BGN	Biglycan	NM_001001825.2
B1G0309	2.32	3.00	RNF38	E3 ubiquitin-protein ligase RNF38	XM_690108.9
B6G0553	2.32	7.49	PGM1	Phosphoglucomutase-1	NM_201025.1
A24G0026	2.32	4.65	RHEB	GTP-binding protein Rheb	NM_200729.1
A11G0750	2.32	2.99	CD27	CD27 antigen	XM_009306329.4
A21G0037	2.32	1.11	GSONMT00056795001	A0A060X777_ONCMY Uncharacterized protein	
B20G0351	2.32	5.58	PDGFRA	Platelet-derived growth factor receptor alpha	NM_131459.3
B9G0381	2.32	2.06	GPR18	N-arachidonyl glycine receptor	XM_005167779.4
B7G1123	2.32	1.61	BCL6B	lymphoma 6 member B protein	XM_021477763.2
A24G0372	2.32	1.68	PEX2	Peroxisome biogenesis factor 2	NM_001423777.1
B9G0884	2.32	2.25	ACVR1	Activin receptor type-1	
A20G0368	2.32	4.37	TPR	Nucleoprotein TPR	NM_001030123.1
A17G0422	2.32	5.80	RAB3GAP2	Rab3 GTPase-activating protein non-catalytic subunit	XM_017351601.3

A12G0498	2.31	5.08	MSRB1	Methionine-R-sulfoxide reductase B1-A	NM_001105128.3
B15G0174	2.31	3.14	WDR62	WD repeat-containing protein 62	XM_694487.8
A25G0170	2.31	1.86	RHCGL2	Ammonium transporter Rh type C-like 2	NM_001320382.1
A14G0131	2.31	19.42	TGFBI	Transforming growth factor-beta-induced protein ig-h3	NM_182862.1
B6G0145	2.31	0.15	CLDN10	Claudin-10	XM_687354.9
A4G0464	2.31	1.82	CELSR1	Cadherin EGF LAG seven-pass G-type receptor 1	XM_005168547.5
A14G0462	2.31	0.84	MCHR2	Melanin-concentrating hormone receptor 2	NM_001423431.1
B18G0351	2.31	5.34	SIAE	Sialate O-acetylesterase	NM_001044331.1
A3G0957	2.31	3.42	G5714_003786	A0A7J6DAG2_9TELE Uncharacterized protein	NM_001005395.1
B24G0585	2.31	10.40	OTUD1	OTU domain-containing protein 1	XM_005162584.5
B21G0947	2.31	3.34	KIAA2026	Uncharacterized protein KIAA2026	XM_005173857.5
A8G0969	2.31	7.29	MIB2	E3 ubiquitin-protein ligase MIB2	NM_001079678.1
A7G0555	2.31	8.59	DNAJB6-A	DnaJ homolog subfamily B member 6-A	XM_009303365.4
A9G0283	2.31	1.44	MPC2	Mitochondrial pyruvate carrier 2	NM_212592.1
A25G0121	2.31	7.02	RBPMS2A	RNA-binding protein, mRNA-processing factor 2a	NM_001002409.1
A5G1030	2.31	4.72	GTF2IRD1	General transcription factor II-I repeat domain-containing protein 1	XM_005172019.5
B11G0322	2.31	3.95	LOC107746895	A0A673L4S2_9TELE Uncharacterized LOC107746895	XM_009306014.4
B5G0798	2.31	7.19	WDR44	WD repeat-containing protein 44	NM_001100038.2
B20G0369	2.31	1.40	ANAPC4	Anaphase-promoting complex subunit 4	XM_017352471.3
B2G0931	2.31	3.75	AGFG1	Arf-GAP domain and FG repeat-containing protein 1	NM_200588.2
B15G0210	2.31	3.21	NEK3	threonine-protein kinase Nek3	XM_068213671.1
B17G0465	2.31	62.25	PABPC4	Polyadenylate-binding protein 4	NM_201296.1
B8G0742	2.31	3.58	SSC5D	Soluble scavenger receptor cysteine-rich domain-containing protein SSC5D	XM_021478396.2
B19G0695	2.31	5.77	CYRIA	CYFIP-related Rac1 interactor A	XM_017352223.2
A7G0143	2.31	12.29	ACO1	Cytoplasmic aconitate hydratase	NM_001034983.1
A17G0838	2.31	1.79	GPR65	Psychosine receptor	
B1G0775	2.31	3.13	TUBGCP3	Gamma-tubulin complex component 3 homolog	XM_005170995.5
B22G0041	2.31	0.83	LOC113067423	A0A6P6MGK2_CARAU zinc finger protein 585A-like isoform X2	XM_017356397.3
B22G0147	2.31	1.42	TICAM1	TIR domain-containing adapter molecule 1	
B5G0540	2.31	3.97	TOR2A	Torsin-2A	NM_001034180.2
A23G0083	2.31	3.84	SLC34A2	Sodium-dependent phosphate transport protein 2B	XM_068216694.1
A21G0470	2.31	2.33	RB1	Retinoblastoma-associated protein	NM_001077780.1
B7G0160	2.31	4.27	SBF2	Myotubularin-related protein 13	XM_009303601.4

B15G0342	2.31	6.30	TYW1	S-adenosyl-L-methionine-dependent tRNA 4-demethylwyosine synthase TYW1	NM_001077156.2
B12G0401	2.31	7.49	HDLBP	Vigilin	XM_677913.5
A10G0514	2.31	4.78	ARFIP2	Arfaptin-2	XM_009305312.4
B12G0691	2.31	0.35	ABI1	Abl interactor 1	XM_068224696.1
A13G0254	2.31	10.22	KIF16B	Kinesin-like protein KIF16B	XM_688164.8
B2G0289	2.31	4.99	PARD3	Partitioning defective 3 homolog	XM_068223515.1
A16G0908	2.31	3.08	CNOT10	CCR4-NOT transcription complex subunit 10	XM_068213947.1
A11G0300	2.31	2.33	DYRK2	Dual specificity tyrosine-phosphorylation-regulated kinase 2	NM_001110030.1
A2G0578	2.31	3.11	TMEM68	Transmembrane protein 68	NM_200492.1
A18G0589	2.31	2.45	MLYCD	Malonyl-CoA decarboxylase, mitochondrial	NM_001089503.1
B21G0109	2.31	13.19	SPRY4	Protein sprouty homolog 4	NM_131826.2
A18G0147	2.31	0.75	TMEM255B	Transmembrane protein 255B	XM_021467871.2
A5G0487	2.31	1.80	TESK2	Dual specificity testis-specific protein kinase 2	NM_001431118.1
A5G0015	2.31	1.52	ADF1	Transcription factor Adf-1	XM_021476368.2
B20G0092	2.30	4.73	RPS6KC1	Ribosomal protein S6 kinase delta-1	NM_001365551.1
A20G0551	2.30	0.72	PFAS	Phosphoribosylformylglycinamidine synthase	NM_001045202.1
B6G0487	2.30	9.33	SLCO2A1	Solute carrier organic anion transporter family member 2A1	NM_001089582.1
B7G0503	2.30	2.95	ARL5B	ADP-ribosylation factor-like protein 5B	NM_199824.1
A11G0101	2.30	4.67	RPS23	40S ribosomal protein S23	
B3G1013	2.30	7.93	RHOT1A	Mitochondrial Rho GTPase 1-A	NM_212704.1
B13G0560	2.30	2.74	DRGX	Dorsal root ganglia homeobox protein	XM_009307270.4
B4G0509	2.30	2.32	GCC1	GRIP and coiled-coil domain-containing protein 1	NM_001003599.2
A7G1291	2.30	3.34	LTB4R	Leukotriene B4 receptor 1	NM_001308975.1
B11G0099	2.30	6.07	PRKACA	cAMP-dependent protein kinase catalytic subunit alpha	NM_001128074.1
B1G0556	2.30	6.04	KLF12	Krueppel-like factor 12	NM_207062.1
A10G0135	2.30	9.19	CAMK2B	calmodulin-dependent protein kinase type II subunit beta	XM_009305704.4
B17G0192	2.30	4.63	MAPKBP1	Mitogen-activated protein kinase-binding protein 1	XM_009292858.4
B7G0454	2.30	3.21	ZNF507	Zinc finger protein 507	XM_005166460.5
A17G0645	2.30	3.55	PTGER2	Prostaglandin E2 receptor EP2 subtype	NM_200635.1
B17G0719	2.30	1.69	GANC	Neutral alpha-glucosidase C	XM_003200376.6
A4G0988	2.30	4.31	ARID2	AT-rich interactive domain-containing protein 2	XM_005164457.5
B9G0542	2.30	2.76	LCMT2	tRNA wybutosine-synthesizing protein 4	NM_001128557.1
B14G0437	2.30	340.03	LECG	Galactose-specific lectin nattectin	
A11G0129	2.30	10.33	NIM1K	threonine-protein kinase NIM1	XM_009305774.3
A11G0357	2.30	1.86	ARHGAP39	Rho GTPase-activating protein 39	
A17G0509	2.30	7.48	BIRC6	Baculoviral IAP repeat-containing protein 6	XM_021473132.1
A7G0594	2.30	3.25	ZNF408	Zinc finger protein 408	NM_001328275.1
B10G0803	2.30	0.15	ANKRD34B	Ankyrin repeat domain-containing protein 34B	XM_684338.7

A17G0102	2.30	6.82	LOC107566112	A0A672TD06_SINGR Uncharacterized LOC107566112	XM_002665221.6
A13G0329	2.30	2.99			NM_200480.1
A11G0422	2.29	5.23	ECE1	Endothelin-converting enzyme 1	NM_001017869.1
A4G0922	2.29	0.16			
B18G0046	2.29	0.85			
B14G0518	2.29	5.89	ADAM19	Disintegrin and metalloproteinase domain-containing protein 19	XM_694823.10
A3G0123	2.29	0.86	RAC1	Ras-related C3 botulinum toxin substrate 1	NM_199771.1
A20G0254	2.29	2.20	PBK	Lymphokine-activated killer T-cell-originated protein kinase homolog	NM_001002387.1
A15G0420	2.29	3.83	CLUH	Clustered mitochondria protein homolog	NM_001077560.1
A13G0392	2.29	5.20	PROM1A	Prominin-1-A	NM_001244919.1
B4G0672	2.29	0.54	SI:CABZ01054394.6	A0A0G2KM98_DANRE Si:cabz01054394.6	XM_068220164.1
B8G0656	2.29	4.17	TOR1AIP2	Torsin-1A-interacting protein 2	XM_001339566.9
A2G0115	2.29	3.89	ARHGAP21	Rho GTPase-activating protein 21	XM_001918933.7
B19G0785	2.29	2.01	ING3	Inhibitor of growth protein 3	
A13G0425	2.29	2.37	FANCL	E3 ubiquitin-protein ligase FANCL	NM_001126381.1
B25G0316	2.29	0.39	RTASE	Probable RNA-directed DNA polymerase from transposon BS	XM_068218007.1
B7G1122	2.29	2.42	TRIP6	Thyroid receptor-interacting protein 6	XM_009303106.4
B24G0237	2.29	18.44	NRBP2	Nuclear receptor-binding protein 2	NM_001130617.2
A15G0057	2.29	9.84	ATG16L1	Autophagy-related protein 16-1	NM_001017854.2
B12G0157	2.29	7.79	RNF213B	E3 ubiquitin-protein ligase rnf213-beta	NM_001353867.2
B2G0521	2.29	1.16	TVA1	T-cell receptor alpha chain V region RL-5	
B11G0009	2.29	5.43	TMBIM6	Probable Bax inhibitor 1	XM_021479925.2
B22G0584	2.29	2.96	GLS	Glutaminase kidney isoform, mitochondrial	XM_068216331.1
A7G0654	2.29	2.90	NFATC3	Nuclear factor of activated T-cells, cytoplasmic 3	XM_068218457.1
B2G1305	2.29	2.47	TRIM25	ISG15 ligase TRIM25	XM_009301048.4
A5G1012	2.29	9.66	AP2B1	AP-1 complex subunit beta-1	NM_199919.2
B11G0616	2.29	3.62	OPSP	Parapinopsin	
A23G0029	2.29	8.19	VGLL4	Transcription cofactor vestigial-like protein 4	XM_005161921.5
A16G0573	2.29	6.20	FAM8A1	Protein FAM8A1	NM_001017551.2
B14G0623	2.29	0.34	A0A0H4D859	A0A0H4D859_CYPCA Interleukin-2b	
B16G1056	2.29	1.79	PRDM1	PR domain zinc finger protein 1	NM_199515.2
B18G0635	2.29	2.56	KLHL36	Kelch-like protein 36	NM_001020580.2
A5G0492	2.29	5.45	TRAFD1	TRAF-type zinc finger domain-containing protein 1	NM_001089515.1
B13G0081	2.29	4.06			XM_695036.9
B14G0774	2.29	1.41	A33	Zinc-binding protein A33	NM_001044961.1
B10G0335	2.29	4.12	CLEC4F	C-type lectin domain family 4 member F	XM_068218586.1
B25G0712	2.29	58.09	MDK-A	Midkine-A	NM_131716.1
A18G0262	2.29	4.50	LOC113114093	A0A6P6QT89_CARAU uncharacterized protein LOC113114093	

B19G0889	2.29	6.11	GMEB1	Glucocorticoid modulatory element-binding protein 1	XM_005159542.5
A24G0551	2.29	1.02	GLI3	Transcriptional activator GLI3	NM_205728.1
B22G0028	2.29	1.87	CDKN1A	Cyclin-dependent kinase inhibitor 1	
B16G0292	2.28	1.87	CCNE2	S-specific cyclin-E2	NM_001002075.1
B9G0580	2.28	0.55	LOC107663846	A0A671SW84_9TELE Uncharacterized protein	XM_001334236.7
B3G0408	2.28	6.75	GIPC1	PDZ domain-containing protein GIPC1	NM_001004018.1
B22G0301	2.28	10.56	G5714_021236	A0A7J6BQD1_9TELE Uncharacterized protein	XM_005161645.5
B6G0199	2.28	1.74	IQCB1	IQ calmodulin-binding motif-containing protein 1	NM_001008622.2
B15G0727	2.28	2.02	MECOM-A	MDS1 and EVI1 complex locus protein EVI1-A	XM_017351212.3
B19G0024	2.28	0.41	ADGRF1	Adhesion G-protein coupled receptor F1	XM_068215668.1
A25G0730	2.28	1.76	LRRK2	threonine-protein kinase 2	NM_001201456.2
B14G0294	2.28	3.90	RNF145	RING finger protein 145	XM_009291206.4
B17G0809	2.28	6.78	GNMT	Glycine N-methyltransferase	NM_212816.1
B21G0216	2.28	3.31	RTASE	Probable RNA-directed DNA polymerase from transposon BS	XM_068222002.1
A3G0185	2.28	1.78	PCTP	Phosphatidylcholine transfer protein	
B12G0032	2.28	5.06	ELOVL6	Elongation of very long chain fatty acids protein 6	NM_001002570.1
A12G0264	2.28	0.41	GAB1	GRB2-associated-binding protein 1	
A16G0028	2.28	0.61	KDF1	Keratinocyte differentiation factor 1	XM_001338342.9
B1G0904	2.28	5.27	G5714_000382	A0A7J6DGX3_9TELE Uncharacterized protein	
B12G0616	2.28	2.03	NUDT13	NAD(P)H pyrophosphatase NUDT13, mitochondrial	NM_001044816.1
B4G0675	2.28	1.77	LOC113092458	A0A6P6NZC9_CARAU gastrula zinc finger protein XlCGF57.1-like	XM_021475732.2
B1G1137	2.28	0.95	LOC113099744	A0A6P6PFZ2_CARAU zinc finger protein 501-like isoform X2	NM_001122845.1
A14G0349	2.28	3.53	XK	Endoplasmic reticulum membrane adapter protein XK	NM_001007406.1
A20G0309	2.28	6.02	NCOA1	Nuclear receptor coactivator 1	XM_021468660.2
B24G0662	2.28	23.94	ANXA13	Annexin A13	NM_131774.2
A6G1024	2.28	4.38	FBXL3	LRR-repeat protein 3	NM_001005773.3
A11G0423	2.28	4.54	PPB	Alkaline phosphatase	XM_021479838.2
B2G0594	2.28	4.97	SLC6A9	Sodium- and chloride-dependent glycine transporter 1	XM_005163494.5
B10G0123	2.28	0.97	LIN1	LINE-1 reverse transcriptase homolog	
A5G0317	2.28	2.08	AMER1	APC membrane recruitment protein 1	NM_001346277.1
A14G0678	2.28	3.25	RNF20	E3 ubiquitin-protein ligase BRE1A	NM_001256175.1
B21G0788	2.28	5.98	PPP1R26	Protein phosphatase 1 regulatory subunit 26	XM_068215925.1
A16G0164	2.28	8.83	RGL2	Ral guanine nucleotide dissociation stimulator-like 2	XM_068214008.1
A22G0013	2.28	3.65	SLC25A34	Solute carrier family 25 member 34	NM_200580.1

A19G0457	2.28	4.61	KIAA0319L	Dyslexia-associated protein KIAA0319-like protein	XM_068215332.1
A1G0591	2.28	3.15	TCB1	Transposable element Tcb1 transposase	XR_011017426.1
A15G0535	2.28	1.22	MAP6	Microtubule-associated protein 6 homolog	XM_002664611.7
A3G0995	2.27	4.77	WNK4	threonine-protein kinase WNK4	XM_680072.10
B6G0847	2.27	2.90	PHC2	Polyhomeotic-like protein 2	NM_201202.3
A9G0633	2.27	2.07	FN1	Fibronectin	NM_131520.2
A18G0138	2.27	2.48	BCL7BB	lymphoma 7 protein family member B-B	NM_001006018.1
A15G0124	2.27	4.45	SLC25A36A	Solute carrier family 25 member 36-A	XR_011006362.1
B10G0207	2.27	4.14	GMCL1	Germ cell-less protein-like 1	NM_207050.1
B8G0588	2.27	1.15	FOXP3	Forkhead box protein P3	XM_068223144.1
B22G0506	2.27	9.44	PXK	PX domain-containing protein kinase-like protein	NM_001082808.1
A7G0272	2.27	4.01	SCAMP2	Secretory carrier-associated membrane protein 2	NM_201194.2
A21G0431	2.27	3.51	NADK2	NAD kinase 2, mitochondrial	XM_005161165.5
B2G1242	2.27	15.10	DSG2	Desmoglein-2	NM_001423856.1
A16G0850	2.27	0.26	PLEKHG6	Pleckstrin homology domain-containing family G member 6	XM_009292203.4
B2G0823	2.27	1.99	RREB1	Ras-responsive element-binding protein 1	XM_678293.10
B23G0233	2.27	3.27	PPP1R3D	Protein phosphatase 1 regulatory subunit 3D	NM_001110412.2
A23G0638	2.27	1.61	RACGAP1	Rac GTPase-activating protein 1	NM_199631.1
B20G0769	2.27	4.20	TRIB2	Tribbles homolog 2	XM_002667743.6
A16G0843	2.27	3.54	SNAPC3	snRNA-activating protein complex subunit 3	NM_201115.3
A15G0657	2.27	3.78	RTASE	Probable RNA-directed DNA polymerase from transposon BS	XM_068214189.1
B6G0608	2.27	3.04	TMEM45B	Transmembrane protein 45B	NM_001020617.1
B17G0804	2.27	3.23	X-ELEMENT\ORF2	Probable RNA-directed DNA polymerase from transposon X-element	
A17G0087	2.27	3.62	ZFP292	Zinc finger protein 292	NM_001105141.2
A17G0669	2.27	1.01	PRORP	Mitochondrial ribonuclease P catalytic subunit	XM_678197.9
B19G0561	2.27	3.11	VPS50	Syndetin	NM_001365104.1
B22G0427	2.27	0.91	A0A671N4Y4	A0A671N4Y4_9TELE Uncharacterized protein	XM_021469670.2
B9G0203	2.27	3.40	G5714_009872	A0A7J6CN90_9TELE CARD domain-containing protein	
B8G0913	2.27	7.83	HCFC1	Host cell factor 1	NM_001128537.1
A16G0677	2.27	1.25	A33	Zinc-binding protein A33	NM_001045168.1
B19G0681	2.27	14.42	TMEM106B	Transmembrane protein 106B	NM_001045052.1
B1G0208	2.27	1.06	CD200	OX-2 membrane glycoprotein	
A9G0806	2.27	4.71	TRIM16	Tripartite motif-containing protein 16	NM_001045270.2
B15G0824	2.27	1.24	LOC107670100	A0A671M1W4_9TELE Uncharacterized LOC107670100	
B16G1020	2.27	5.75	RTASE	Probable RNA-directed DNA polymerase from transposon BS	
A10G0369	2.27	1.01	CD276	CD276 antigen homolog	XM_005172670.5
B7G0612	2.27	8.76			
A11G0413	2.27	3.51	FBLN2	Fibulin-2	NM_001077251.1
A25G0204	2.27	7.59	AKAP13	A-kinase anchor protein 13	XM_068217499.1
A13G0210	2.27	3.09	LGALS3	Galectin-3	NM_001034183.2
A7G0428	2.26	1.93	CDKN2AIP-A	Protein CDKN2AIP homolog A	XM_005169040.4

A24G0259	2.26	3.65	FBXO45	SPRY domain-containing protein 1	NM_200595.1
A21G0432	2.26	2.53	SKP2	S-phase kinase-associated protein 2	
B16G0572	2.26	6.54	TMEM245	Transmembrane protein 245	XM_009292372.4
B25G0349	2.26	2.83	ZNF800	Zinc finger protein 800	NM_001324549.1
A12G0827	2.26	3.55	RASD1	Dexamethasone-induced Ras-related protein 1	NM_200532.1
B5G0127	2.26	3.56	RICTOR	Rapamycin-insensitive companion of mTOR	XM_009301197.4
A7G0371	2.26	1.31	CCKAR	Cholecystokinin receptor type A	XM_692401.8
A21G0218	2.26	1.03	LOC113079674	A0A6P6NHU7_CARAU uncharacterized protein LOC113079674 isoform X1	XM_001345212.8
A1G0528	2.26	2.04	VEGFC	Vascular endothelial growth factor C	XM_005159998.5
A19G0549	2.26	0.38	HOXA2	Homeobox protein Hox-A2	
B24G0721	2.26	1.83	PARD3	Partitioning defective 3 homolog	XM_017353937.3
B13G0370	2.26	5.23	ZSWIM8	Zinc finger SWIM domain-containing protein 8	NM_201496.1
B13G0173	2.26	5.59	PLEKHH2	Pleckstrin homology domain-containing family H member 2	NM_001008612.1
A5G0263	2.26	6.79	ACACB	Acetyl-CoA carboxylase 2	XM_021476433.2
B9G0254	2.26	6.97	CLASP1	CLIP-associating protein 1	XM_068223405.1
B25G0592	2.26	7.47	CRY2	Cryptochrome-2	NM_131786.2
A2G0414	2.26	1.96	DUSP12	Dual specificity protein phosphatase 12	XM_005163623.5
B20G0053	2.26	0.58	NUP43	Nucleoporin Nup43	NM_212892.1
B24G0717	2.26	1.31	LOC107653801	A0A671M571_9TELE Zinc finger protein 271-like	
A18G0089	2.26	2.63	PRRG4	Transmembrane gamma-carboxyglutamic acid protein 4	NM_001017637.2
A7G0264	2.26	4.57	NLRC3	Protein NLRC3	XM_068220648.1
B7G0715	2.26	7.21	TJP1	Tight junction protein ZO-1	XM_068222634.1
B22G0406	2.26	2.96	DPX16_1947	A0A3N0Y742_ANAGA Zinc finger protein 595	XM_017356397.3
B15G0493	2.25	7.54	CDC42EP2	Cdc42 effector protein 2	NM_001077731.1
A20G0222	2.25	5.74	GOPC	Golgi-associated PDZ and coiled-coil motif-containing protein	NM_200557.1
A1G0969	2.25	6.82	FAT1	Protocadherin Fat 1	XM_005168343.5
B17G0003	2.25	5.16	POLE2	DNA polymerase epsilon subunit 2	NM_173246.1
B2G0889	2.25	5.03	TPR	Nucleoprotein TPR	XM_009298498.4
B23G0677	2.25	4.07	A0A673HZ42	A0A673HZ42_9TELE Si:ch211-210c8.6	XM_021470018.2
B15G0358	2.25	1.98	DHX40	Probable ATP-dependent RNA helicase DHX40	XM_021466352.2
B11G0163	2.25	15.12	NDUFS7	NADH dehydrogenase [ubiquinone] iron-sulfur protein 7, mitochondrial	NM_001077454.1
A4G1030	2.25	2.58	LOC113048450	gamma crystallin domain-containing protein 1-like	XM_068220711.1
A1G0906	2.25	6.40	PLA2G12A	Group XIIA secretory phospholipase A2	
A17G0046	2.25	4.11	TRAPPC12	Trafficking protein particle complex subunit 12	NM_001080644.2
B25G0161	2.25	7.18	IGKV1-13	Immunoglobulin kappa variable 1-13	NM_001045408.1
A8G0800	2.25	3.29	FHIP2B	FHF complex subunit HOOK interacting protein 2B	NM_001077766.1

B19G0002	2.25	4.32	VPS72	Vacuolar protein sorting-associated protein 72 homolog	
B19G0403	2.25	3.26	KANSL1	KAT8 regulatory NSL complex subunit 1	XM_009294186.4
B7G0780	2.25	5.29	GFOD2	Glucose-fructose oxidoreductase domain-containing protein 2	NM_001077746.2
B20G0679	2.25	4.22	ZNF512	Zinc finger protein 512	XM_068215681.1
B11G0265	2.25	3.92	KRT42	Keratin, type I cytoskeletal 42	XM_021479793.2
A14G0154	2.25	1.81	TSTD1	Thiosulfate:glutathione sulfurtransferase	XM_686628.10
B5G0306	2.25	3.78	MLXIP	MLX-interacting protein	NM_001317759.2
B15G0508	2.25	2.61	GEMIN4	Gem-associated protein 4	NM_001018144.2
B17G0523	2.25	4.11	LUZP1	Leucine zipper protein 1	XM_009293139.4
A14G0029	2.25	20.82	TRIM16	Tripartite motif-containing protein 16	XM_681071.8
B13G0197	2.25	4.93	POL	Retrovirus-related Pol polyprotein from transposon 297	NM_001423690.1
B24G0051	2.25	9.96	AOC1	Amiloride-sensitive amine oxidase [copper-containing]	NM_001077598.1
A15G0737	2.25	3.15	BACE2	Beta-secretase 2	XM_009291473.4
B21G0306	2.25	12.38	CYSTM1	Cysteine-rich and transmembrane domain-containing protein 1	NM_001098766.1
A7G1333	2.25	8.71	NEK1	threonine-protein kinase Nek1	XM_068222504.1
A8G0361	2.25	3.22	RFX2	DNA-binding protein RFX2	XM_005166837.5
B25G0142	2.25	0.00	IGLC7	Immunoglobulin lambda constant 7	
B1G1034	2.25	6.55	DPX16_23495	A0A3N0Z1A1_ANAGA Uncharacterized protein	
B17G0704	2.25	2.08	VCPKMT	Protein N-lysine methyltransferase METTL21D	XM_005158957.2
A3G1068	2.25	6.60	SMURF2	E3 ubiquitin-protein ligase SMURF2	NM_001114426.1
A10G0385	2.25	2.83	SMAD9	Mothers against decapentaplegic homolog 9	NM_001328499.1
A19G0391	2.25	3.96	PPP1R9A	Neurabin-1	XM_009294367.4
A9G0856	2.25	1.41	LOC113057383	A0A6P6L7R6_CARAU gastrula zinc finger protein XlCGF7.1-like isoform X2	XM_068220341.1
B10G0771	2.25	3.53			
A18G0721	2.25	0.45	P2RY2	P2Y purinoceptor 2	XM_695238.10
A19G0807	2.25	13.96	ATP1A3	potassium-transporting ATPase subunit alpha-3	NM_131684.2
B24G0367	2.25	1.71	CIP2A	Protein CIP2A	NM_001114421.1
B24G0531	2.25	4.07	SEMA5A	Semaphorin-5A	XM_005162619.5
A13G0422	2.25	5.80	HEBP2	Heme-binding protein 2	XM_001343182.9
A18G0386	2.25	9.32	FAM174B	Membrane protein FAM174B	NM_001103135.1
A7G0454	2.25	2.00	HRAS	GTPase HRas	NM_001020629.1
B1G0168	2.24	3.37	PLK1	threonine-protein kinase PLK1	NM_001003890.3
A5G1127	2.24	5.02			XM_005165497.5
A4G0572	2.24	2.87	LRRC17	Leucine-rich repeat-containing protein 17	
A8G0552	2.24	14.66	SLC16A1	Monocarboxylate transporter 1	NM_200085.1
B2G0396	2.24	13.64	DCAF8	DDB1- and CUL4-associated factor 8	XM_021471269.2
B1G0859	2.24	5.38	BTBD3	POZ domain-containing protein 3	NM_001145581.1
B16G0968	2.24	4.15	CPQ	Carboxypeptidase Q	
B23G0547	2.24	1.89	LMBR1L	Limb region 1 homolog-like protein	NM_198874.1

B1G0939	2.24	3.28	NUP133	Nuclear pore complex protein Nup133	NM_213531.1
A20G0361	2.24	5.33	SWT1	Transcriptional protein SWT1	XM_009294798.4
B5G0200	2.24	6.73	PPM1F	Protein phosphatase 1F	NM_001018155.1
B3G1746	2.24	4.41	GBP6	Guanylate-binding protein 6	XM_068214657.1
A4G0947	2.24	3.64	SYPL1	Synaptophysin-like protein 1	XM_002661341.7
A9G0599	2.24	1.90	IGF2BP2	Insulin-like growth factor 2 mRNA-binding protein 2	XM_009304504.4
A5G1089	2.24	3.49	PPM1D	Protein phosphatase 1D	NM_001007340.1
B25G0702	2.24	7.43	TPH1	Tryptophan 5-hydroxylase	NM_178306.3
B3G0762	2.24	12.72	HV02	Ig heavy chain V region XIG14 (Fragment)	XM_068217859.1
B6G0842	2.24	3.04	LRIG2	Leucine-rich repeats and immunoglobulin-like domains protein 2	XM_694658.10
A17G0468	2.24	5.04	RLF	Zinc finger protein Rlf	XM_021467415.2
B8G0625	2.24	5.45	TNFRSF14	Tumor necrosis factor receptor superfamily member 14	
A20G0125	2.24	7.95	HADHA	Trifunctional enzyme subunit alpha, mitochondrial	NM_001105276.1
A23G0624	2.24	37.48	LOC107549039	A0A672MRI5_SINGR Uncharacterized LOC107549039	
B25G0753	2.24	0.54	DEPDC7	DEP domain-containing protein 7	XM_684969.7
B21G0515	2.24	2.33	NEU3	Sialidase-3	XM_005161205.5
B14G0237	2.24	11.00	KDRL	Vascular endothelial growth factor receptor kdr-like	NM_131472.1
B7G1120	2.24	3.95	SNX15	Sorting nexin-15	NM_001111237.1
B9G0613	2.24	1.47	TTF2	Transcription termination factor 2	XM_005168619.5
A10G0283	2.24	2.93	MIPEP	Mitochondrial intermediate peptidase	XM_021479450.2
A16G0310	2.24	3.97	PRUNE1	Exopolyphosphatase PRUNE1	NM_001160294.1
B12G0213	2.24	5.92	SLC16A3	Monocarboxylate transporter 4	XM_005169469.5
B12G0409	2.24	2.82	EME1	Crossover junction endonuclease EME1	XM_702004.7
B13G0819	2.24	6.71	SLC29A1	Equilibrative nucleoside transporter 1	NM_001423963.1
A24G0258	2.24	5.89	ARHGAP29	Rho GTPase-activating protein 29	XM_009297526.4
B13G0760	2.24	3.02	CDC42EP3	Cdc42 effector protein 3	NM_001017635.1
A6G0016	2.24	10.42	LYL1	Protein lyl-1	XM_068221859.1
A23G0630	2.24	7.39	ITGA5	Integrin alpha-5	NM_001004288.2
B12G0402	2.24	1.86	ARHGEF15	Rho guanine nucleotide exchange factor 15	XM_021472530.2
A8G0718	2.23	1.67	ADRA2B	Alpha-2B adrenergic receptor	NM_207638.1
A25G0728	2.23	2.05	SLC2A13	Proton myo-inositol cotransporter	
A25G0481	2.23	3.36	CXORF38	Uncharacterized protein CXorf38	XM_021473882.2
B6G0511	2.23	1.73	DIPK1A	Divergent protein kinase domain 1A	XM_017356629.3
B1G0731	2.23	1.62	MOGS	Mannosyl-oligosaccharide glucosidase	NM_001080190.1
B18G0823	2.23	16.31	SEMA7A	Semaphorin-7A	
A22G0668	2.23	14.64	EFEMP1	EGF-containing fibulin-like extracellular matrix protein 1	XM_009296503.4
B2G0954	2.23	2.53	PBX1	Pre-B-cell leukemia transcription factor 1	NM_131614.1
B3G0819	2.23	9.14	AOCX	Primary amine oxidase, liver isozyme	NM_001013555.3

A9G0436	2.23	3.03	EPC2	Enhancer of polycomb homolog 2	NM_201075.1
A5G1281	2.23	1.44	KMT5AA	N-lysine methyltransferase KMT5A-A	
A13G0154	2.23	14.47	SLC25A28	Mitoferrin-2	XM_005156498.5
B15G0705	2.23	3.03	SOSTDC1	Sclerostin domain-containing protein 1	NM_001017598.2
A13G0552	2.23	4.90	LOC107666573	A0A671RS06_9TELE Uncharacterized LOC107666573	NM_001098388.2
B17G0644	2.23	4.33	ASAP2	Arf-GAP with SH3 domain, ANK repeat and PH domain-containing protein 2	NM_001045207.1
A16G0996	2.23	3.79	MTF1	Metal regulatory transcription factor 1	XM_021466610.2
B3G0971	2.23	2.88	FAM83F	Protein FAM83F	XM_690066.10
A6G0646	2.23	0.04	PRSS8	Prostasin	NM_001105601.1
A16G0042	2.23	2.33	SACM1LB	Phosphatidylinositol-3-phosphatase SAC1-B	NM_001080624.2
A18G0024	2.23	7.19	LOXL1	Lysyl oxidase homolog 1	NM_001043325.1
A22G0080	2.23	15.84	ING5	Inhibitor of growth protein 5	NM_001100049.2
B21G0509	2.23	2.25	SLC35F2	Solute carrier family 35 member F2	NM_001076556.1
A23G0579	2.23	5.71	OSBPL1A	Oxysterol-binding protein-related protein 1	XM_005162340.5
B7G0929	2.23	15.12	PICALM	Phosphatidylinositol-binding clathrin assembly protein	XM_005172320.5
B21G0453	2.23	3.27	NFRKB	Nuclear factor related to kappa-B-binding protein	NM_001243035.1
A7G1077	2.23	4.56	MEN1	Menin	NM_130953.1
B13G0821	2.23	4.79	FBXO5	F-box only protein 5	XM_068214189.1
B24G0496	2.23	4.42	ARFGEF1	Brefeldin A-inhibited guanine nucleotide-exchange protein 1	XM_068217189.1
A9G0676	2.23	5.96	NAXD	ATP-dependent (S)-NAD(P)H-hydrate dehydratase	NM_001328150.1
A15G0463	2.23	1.52	KLC1	Kinesin light chain 1	NM_001077178.2
B12G0040	2.22	4.46	ACOD	Acyl-CoA desaturase	NM_198815.2
B21G1001	2.22	19.88	JAK2	Tyrosine-protein kinase JAK2	NM_131093.1
A13G0478	2.22	2.34	SERTAD2	SERTA domain-containing protein 2	NM_001089469.1
A13G0554	2.22	6.20	FP	Female protein	NM_001328052.1
A8G0441	2.22	5.77	TPD52L2	Tumor protein D54	XM_005167010.5
B3G0248	2.22	1.54	FBF1	Fas-binding factor 1 homolog	XM_021470860.2
B6G0645	2.22	7.28	HDAC4	Histone deacetylase 4	XM_068218339.1
A16G0006	2.22	4.35	VPS13B	Intermembrane lipid transfer protein VPS13B	XM_068214290.1
A15G0518	2.22	4.21	TPST1	Protein-tyrosine sulfotransferase 1	XM_005157502.5
B24G0641	2.22	3.36	HARBI1	Putative nuclease HARBI1	XM_692391.10
B11G0041	2.22	1.99	PDRG1	p53 and DNA damage-regulated protein 1	NM_200298.2
B1G1156	2.22	7.96	DNM2	Dynamin-2	NM_213242.1
B7G0172	2.22	5.13	ARNTL	Aryl hydrocarbon receptor nuclear translocator-like protein 1	NM_178300.1
A16G0921	2.22	2.42	CCR2	C-C chemokine receptor type 2	XM_005173463.5
A10G0483	2.22	4.02	INTS2	Integrator complex subunit 2	NM_001020542.2
A25G0076	2.22	0.93	PIDD1	p53-induced death domain-containing protein 1	XM_021470600.2
B4G0450	2.22	10.79	NRIP2	Nuclear receptor-interacting protein 2	NM_001123515.1

B20G0423	2.22	2.01	MTHFD1L	Monofunctional C1-tetrahydrofolate synthase, mitochondrial	NM_001242996.1
A1G0610	2.22	2.65	KCNQ5	Potassium voltage-gated channel subfamily KQT member 5	XM_691989.10
B22G0734	2.22	1.34	CELA1	Chymotrypsin-like elastase family member 1	XM_021469696.2
B20G0237	2.22	12.40	SCRN2	Secernin-2	NM_001030116.1
A15G0505	2.22	4.59	SORL1	Sortilin-related receptor	XM_068213715.1
B16G0547	2.22	4.41	MINDY3	Ubiquitin carboxyl-terminal hydrolase MINDY-3	NM_001077756.1
B19G0079	2.22	5.34	A33	Zinc-binding protein A33	NM_001020475.1
B14G0709	2.22	7.88	RNF4	E3 ubiquitin-protein ligase RNF4	NM_001386248.1
B24G0322	2.22	3.52	MSL2	E3 ubiquitin-protein ligase MSL2	NM_001163313.1
A17G0427	2.22	3.24	CRYBG1	gamma crystallin domain-containing protein 1	XM_005158549.5
A3G1033	2.22	8.33	CSF2RB2	Interleukin-3 receptor class 2 subunit beta	
B16G0090	2.22	2.92	DHDDS	Dehydrodolichyl diphosphate synthase complex subunit DHDDS	NM_213187.1
B16G1111	2.22	5.31	THEMIS2	Protein THEMIS2	XM_068214070.1
B18G0130	2.22	8.77	BAZ1B	Tyrosine-protein kinase BAZ1B	NM_001347672.1
B21G0791	2.21	15.79	COL5A1	Collagen alpha-1(V) chain	XM_021468999.2
A2G1115	2.21	4.43	TLE2	Transducin-like enhancer protein 2	NM_001045238.2
B4G0387	2.21	0.90	HARBI1	Putative nuclease HARBI1	
A18G0145	2.21	3.74	ZBTB38	Zinc finger and BTB domain-containing protein 38	XM_009293816.4
A7G0615	2.21	2.54	GPATCH1	G patch domain-containing protein 1	NM_001204123.1
B19G0261	2.21	6.75	KMT2B	Histone-lysine N-methyltransferase 2B	XM_005159485.5
B6G0491	2.21	3.67	CEP63	Centrosomal protein of 63 kDa	XM_068221680.1
A15G0206	2.21	2.00	ZBTB16A	Zinc finger and BTB domain-containing protein 16-A	XM_068213779.1
A19G0168	2.21	4.36	LOC113068241	A0A6P6MJW0_CARAU uncharacterized protein LOC113068241 isoform X1	
B6G0694	2.21	2.02	COL7A1	Collagen alpha-1(VII) chain	XM_068222026.1
A1G0352	2.21	1.75	BCL9	lymphoma 9 protein	XM_005170982.5
A24G0452	2.21	2.54	TPK1	Thiamin pyrophosphokinase 1	XM_005162615.5
A6G0021	2.21	8.82	TOM1	Target of Myb1 membrane trafficking protein	
A17G0477	2.21	2.50	EHBP1	EH domain-binding protein 1	XM_068214587.1
A10G0540	2.21	2.13	G5714_010927	A0A7J6CLG9_9TELE Uncharacterized protein	XM_021479349.2
B6G0697	2.21	5.76	SNX5	Sorting nexin-5	XM_021477235.2
B12G0714	2.21	7.24	KANSL1	KAT8 regulatory NSL complex subunit 1	XM_005156013.5
A7G0920	2.21	2.76	ZHX1	Zinc fingers and homeoboxes protein 1	NM_001130784.1
A6G0011	2.21	0.54	LRRC8E	Volume-regulated anion channel subunit LRRC8E	XM_009302065.4
B2G0383	2.21	8.40	ACIN1	Apoptotic chromatin condensation inducer in the nucleus	XM_021471261.2
B17G0272	2.21	2.68	MANEA	Glycoprotein endo-alpha-1,2-mannosidase	NM_001004566.2
A17G0648	2.21	4.23	ZMYM4	Zinc finger MYM-type protein 4	XM_684300.10

B4G0156	2.21	19.17	AHCYL2	Adenosylhomocysteinase 3	NM_201340.1
B14G0144	2.21	13.88	STAG2	Cohesin subunit SA-2	XM_005173193.5
B19G0601	2.21	2.83	ANLN	Anillin	NM_001102676.2
A11G0172	2.21	2.82	JUP	Junction plakoglobin	XM_005155707.5
B23G0519	2.21	11.46	HSPG2	Basement membrane-specific heparan sulfate proteoglycan core protein	XM_021469988.2
A21G0769	2.21	3.51	MTMR1	Myotubularin-related protein 1	XM_005161411.5
A19G0929	2.20	9.31	TNFRSF11B	Tumor necrosis factor receptor superfamily member 11B	NM_001423407.1
B5G0971	2.20	5.33	SNX19	Sorting nexin-19	NM_001130642.1
B19G0404	2.20	4.65	SMARCC1	SNF complex subunit SMARCC1	XM_068215128.1
B21G0072	2.20	1.61	SHROOM1	Protein Shroom1	XM_682698.10
B23G0211	2.20	11.18	ZNF704	Zinc finger protein 704	XM_005162014.5
B22G0282	2.20	0.59	LOC113040346	A0A6P6J2T9_CARAU uncharacterized protein LOC113040346	XM_068217854.1
B6G0082	2.20	7.63	PTPRF	Receptor-type tyrosine-protein phosphatase F	XM_068221630.1
A21G0352	2.20	6.67	TNKS	Poly [ADP-ribose] polymerase tankyrase-1	XM_009295373.4
A2G0793	2.20	0.41	TRAV14DV4	delta variable 4	
B19G0777	2.20	3.80	A0A673WMR2	A0A673WMR2_SALTR Uncharacterized protein	
B21G0852	2.20	4.44	UCK1	Uridine-cytidine kinase 1	NM_001004666.1
A20G0181	2.20	3.46	TRIB2	Tribbles homolog 2	XM_002667743.6
B1G0814	2.20	1.02	GSK3B	Glycogen synthase kinase-3 beta	NM_001145688.1
A15G0634	2.20	1.86	KBTBD8	Kelch repeat and BTB domain-containing protein 8	NM_001079948.2
B15G0537	2.20	3.41	NPAT	Protein NPAT	XM_685884.8
A25G0640	2.20	4.08	KV3A9	Ig kappa chain V-III region MOPC 63	XM_068217596.1
A13G0326	2.20	1.00	PGAM1	Phosphoglycerate mutase 1	
B11G0263	2.20	2.59	KRT14	Keratin, type I cytoskeletal 14	
B1G0033	2.20	6.04	RASA3	Ras GTPase-activating protein 3	XM_009305235.4
A1G0079	2.20	1.96	COLGALT1	Procollagen galactosyltransferase 1	NM_001110522.1
B3G0830	2.20	21.50	HSD3B6	Delta 5-->4-isomerase type 6	NM_199809.1
B16G0410	2.20	6.18	DOP1A	Protein dopey-1	XM_021473020.2
B4G0224	2.20	11.53	FBXL14	LRR-repeat protein 14	NM_001015043.2
A22G0285	2.20	11.35	GLS	Glutaminase kidney isoform, mitochondrial	XM_009296204.4
A17G0461	2.20	5.62	MAP4K3	Mitogen-activated protein kinase kinase kinase kinase 3	XM_021473170.2
B15G0707	2.20	3.06	ANKMY2	Ankyrin repeat and MYND domain-containing protein 2	NM_199894.1
A12G0839	2.20	2.94	SULT2B1	Sulfotransferase 2B1	
B13G0648	2.20	6.87	LOC113056740	A0A6P6L7A4_CARAU uncharacterized protein LOC113056740	
A12G0570	2.20	1.66			
A3G0100	2.20	1.15	LOC107731602	A0A673LSC2_9TELE GTPase IMAP family member 7-like	
B1G0057	2.20	3.99	RUNX1	Runt-related transcription factor 1	XM_068220752.1
A20G0731	2.20	4.60	ZYG11	Protein zyg-11 homolog	NM_001040332.2
A6G0581	2.20	4.92	TIE1	Tyrosine-protein kinase receptor Tie-1	XM_001334637.10
A12G0145	2.20	4.92	CDC42EP4	Cdc42 effector protein 4	
A19G0207	2.20	8.47	SACM1LA	Phosphatidylinositol-3-phosphatase SAC1-A	NM_001044878.2

A16G0820	2.20	2.12	PLEKHA8	Pleckstrin homology domain-containing family A member 8	NM_001007374.1
B16G0629	2.20	2.78	G5714_016084	A0A7J6C845_9TELE CARD domain-containing protein	
B17G0455	2.20	6.74	HMGCL	Hydroxymethylglutaryl-CoA lyase, mitochondrial	NM_201215.1
A2G0596	2.19	4.54	PAXIP1	PAX-interacting protein 1	XM_021480006.2
A13G0183	2.19	5.00			XM_005156601.5
A22G0237	2.19	4.80	ABHD14A	Protein ABHD14A	NM_207066.2
B1G0436	2.19	2.30	PPA1	Inorganic pyrophosphatase	XM_005159884.4
A5G0001	2.19	7.25	IFT74	Intraflagellar transport protein 74 homolog	NM_001002385.1
B23G0645	2.19	4.79	LETMD1	LETM1 domain-containing protein 1	
B14G0406	2.19	10.23	FERMT3	Fermitin family homolog 3	NM_200904.2
B13G0892	2.19	1.44	SLC30A10	Zinc transporter 10	XM_017355732.2
B9G0584	2.19	2.41	TMEM30A	Cell cycle control protein 50A	NM_200596.2
B21G0134	2.19	5.43	CLINT1	Clathrin interactor 1	XM_009295602.4
A3G0010	2.19	6.53	TCF20	Transcription factor 20	XM_005171536.5
A22G0468	2.19	6.74	CKS1B	Cyclin-dependent kinases regulatory subunit 1	NM_001044969.1
B17G0609	2.19	2.03	EVA1A	Protein eva-1 homolog A	NM_001076587.3
A17G0104	2.19	2.07	SUPT7L	STAGA complex 65 subunit gamma	NM_001013292.2
B3G1714	2.19	0.95	G5714_004123	A0A7J6DCN4_9TELE Uncharacterized protein	XM_017354729.3
B5G1083	2.19	6.71	CYP1A1	Cytochrome P450 1A1	NM_001007310.1
A23G0125	2.19	4.02	CD81	CD81 antigen	XM_005161898.5
A7G0616	2.19	8.03	GPATCH1	G patch domain-containing protein 1	
A19G0105	2.19	16.02	EPB41	Protein 4.1	XM_068215258.1
B12G0517	2.19	7.35	CYTH3	Cytohesin-3	XM_001342001.8
B19G0061	2.19	2.53	ANKRD28	threonine-protein phosphatase 6 regulatory ankyrin repeat subunit A	XM_009301169.4
A16G0686	2.19	8.52	TUBB	Tubulin beta chain	NM_198818.1
A9G0769	2.19	6.91	STK24	threonine-protein kinase 24	NM_001110451.1
A22G0619	2.19	3.64	BBIP1	BBSome-interacting protein 1	
B8G0463	2.19	7.50	ADIPOR1	Adiponectin receptor protein 1	NM_213500.1
B6G0439	2.19	0.97	NTN1	Netrin-1	NM_131029.2
B3G0118	2.19	1.06	LOC113046763	A0A6P6JUE8_CARAU gastrula zinc finger protein XlCGF57.1-like isoform X1	
B6G0474	2.19	3.63	STK25	threonine-protein kinase 25	XM_005165925.5
B24G0296	2.19	6.80	FBXO45	SPRY domain-containing protein 1	NM_200595.1
B20G0135	2.19	2.55	ARV1	Protein ARV1	NM_001113611.1
B7G0369	2.19	6.61	PAQR9	Membrane progesterone receptor epsilon	XM_017357091.3
A7G0148	2.19	2.91	ERN1	endoribonuclease IRE1	XM_005172372.5
B14G0741	2.19	1.68	POLR1A	DNA-directed RNA polymerase I subunit RPA1	XM_001922804.7
A6G0040	2.19	0.96	FAM83F	Protein FAM83F	NM_001007320.3
A2G0117	2.19	5.31	KMT2C	Histone-lysine N-methyltransferase 2C	XM_068213615.1
B5G1256	2.19	0.55	TCHP	Trichoplein keratin filament-binding protein	NM_001040343.1
A15G0062	2.19	4.97	IL1RAP	Interleukin-1 receptor accessory protein	XM_021466505.2
B11G0160	2.19	2.46	R3HDM4	R3H domain-containing protein 4	XM_005155750.5

B8G0573	2.18	3.11	ELAPOR1	lysosome-associated apoptosis and autophagy regulator 1	XM_021478432.2
B21G0214	2.18	3.14	GEMIN5	Gem-associated protein 5	XM_009295614.4
A9G0688	2.18	0.68	LOC113053760	A0A6P6KTR3_CARAU uncharacterized protein LOC113053760	
A5G1271	2.18	2.50	ZNF618	Zinc finger protein 618	NM_001326692.1
B24G0242	2.18	26.38	CA2	Carbonic anhydrase 2	NM_199215.2
B15G0708	2.18	3.24	TSPAN13	Tetraspanin-13	NM_001002334.1
A5G1006	2.18	0.62	LOC107587718	A0A672PIF3_SINGR Symplekin	
B6G0525	2.18	5.61	PDE6D	Retinal rod rhodopsin-sensitive cGMP 3',5'-cyclic phosphodiesterase subunit delta	NM_001002708.1
A13G0520	2.18	2.20	PBLD	Phenazine biosynthesis-like domain-containing protein	NM_001302229.1
B3G0399	2.18	1.03	A33	Zinc-binding protein A33	XM_003198167.6
B17G0033	2.18	0.37	ADGRG2	Adhesion G-protein coupled receptor G2	XM_021467532.2
A21G0077	2.18	1.63	ATP8B1	Phospholipid-transporting ATPase IC	XM_001920475.8
B7G0953	2.18	1.53	ASB7	Ankyrin repeat and SOCS box protein 7	XM_068222203.1
A12G0653	2.18	2.11	NGFR	Tumor necrosis factor receptor superfamily member 16	NM_001198660.1
B9G0718	2.18	2.60	RAB20	Ras-related protein Rab-20	NM_212771.2
A7G0401	2.18	1.48	CITED1	p300-interacting transactivator 1	NM_001114891.2
B19G0845	2.18	2.68	TRIM25	ISG15 ligase TRIM25	XM_003200557.6
A11G0716	2.18	24.67	RAP1A	Ras-related protein Rap-1A	NM_001365192.1
B24G0549	2.18	3.83	THTPA	Thiamine-triphosphatase	
B22G0208	2.18	1.32	BCDIN3D	Pre-miRNA 5'-monophosphate methyltransferase	
A9G0824	2.18	5.44	MYLK	Myosin light chain kinase, smooth muscle	XM_009304446.2
A9G0136	2.18	10.05	FRZB	Secreted frizzled-related protein 3	NM_130943.2
A7G1201	2.18	9.55	CXCL8	Interleukin-8	
B22G0908	2.18	0.88	VTCN1	V-set domain-containing T-cell activation inhibitor 1	
B14G0752	2.18	4.41	TCIRG1	V-type proton ATPase 116 kDa subunit a 3	NM_213069.1
A6G0547	2.18	3.97	GAK	Cyclin-G-associated kinase	XM_009302472.4
A2G0714	2.18	6.02	RASAL2	Ras GTPase-activating protein nGAP	XM_068214281.1
A2G0390	2.18	2.40	COL11A1	Collagen alpha-1(XI) chain	NM_001184954.1
B1G0567	2.18	3.43	USP38	Ubiquitin carboxyl-terminal hydrolase 38	XM_003197674.6
A25G0372	2.18	2.79	NEDD1	Protein NEDD1	XM_005163004.5
A23G0714	2.18	2.96	MUL1	Mitochondrial ubiquitin ligase activator of NFKB 1	NM_001098760.1
B21G0870	2.18	5.03	DYM	Dymeclin	XM_017353033.2
B13G0634	2.18	5.28	POL	RNA-directed DNA polymerase from mobile element jockey	XM_068212857.1
B6G0509	2.18	7.45	EVI5	Ecotropic viral integration site 5 protein homolog	XM_068221984.1
A4G0391	2.17	0.28	CLEC17A	C-type lectin domain family 17, member A	XM_068218582.1
A21G0630	2.17	1.20	TNFSF10	Tumor necrosis factor ligand superfamily member 10	NM_001042713.1
B25G0242	2.17	4.35	PTPRJ	Receptor-type tyrosine-protein phosphatase eta	XM_068217452.1

B3G0772	2.17	4.60	IGHV3-33	Immunoglobulin heavy variable 3-33	
B10G0647	2.17	0.73			XM_021472374.2
B18G0478	2.17	0.93	WNT2	Protein Wnt-2	NM_130950.1
A15G0672	2.17	7.89	STRN4	Striatin-4	XM_021466324.2
A9G0356	2.17	2.14	COMMD6	COMM domain-containing protein 6	XM_001332052.6
B5G0017	2.17	5.45	YTHDC2	3'-5' RNA helicase YTHDC2	XM_021476371.2
A17G0556	2.17	2.56	SH3PXD2A	SH3 and PX domain-containing protein 2A	XM_068218851.1
A8G0244	2.17	5.61	CABP4	Calcium-binding protein 4	NM_001328057.1
B23G0827	2.17	4.76	TUT4	Terminal uridylyltransferase 4	XM_001335483.8
B22G0757	2.17	6.65	FOXC2	Forkhead box protein C2	NM_001104941.2
B1G0949	2.17	1.52	YTX2	Transposon TX1 uncharacterized 149 kDa protein	
B8G0372	2.17	1.18	DPX16_3461	A0A3N0XKN8_ANAGA Zinc finger protein 568	XM_017355829.3
B3G0430	2.17	0.49	TBCD	Tubulin-specific chaperone D	
A5G0796	2.17	2.98	TRPV1	Transient receptor potential cation channel subfamily V member 1	NM_001126399.1
A21G0507	2.17	22.95	EFEMP2	EGF-containing fibulin-like extracellular matrix protein 2	NM_001160358.2
A8G0271	2.17	6.58	FNBP1L	Formin-binding protein 1-like	XM_021478241.2
B12G0328	2.17	6.22	SRCAP	Helicase SRCAP	XM_005156310.5
B17G0176	2.17	4.35	EAPP	E2F-associated phosphoprotein	NM_199784.1
A25G0097	2.17	1.73	CMASB	N-acylneuraminate cytidylyltransferase B	NM_001258409.1
B19G0840	2.17	0.62	GRHL2	Grainyhead-like protein 2 homolog	XM_005159580.5
B13G0260	2.17	1.70	ALMS1	Centrosome-associated protein ALMS1	NM_001327778.1
B10G0617	2.17	2.63	SAMSN1	SAM domain-containing protein SAMSN-1	XM_005169164.5
B7G0678	2.17	9.91	ITLN	Intelectin	NM_001076581.1
B13G0581	2.17	26.24	DHRS7	reductase SDR family member 7	NM_001423910.1
B1G0276	2.17	5.67	MICU3	Calcium uptake protein 3, mitochondrial	XM_068221058.1
A11G0520	2.17	1.94	MAPKAPK3	MAP kinase-activated protein kinase 3	
A22G0102	2.17	3.05	TIMM44	Mitochondrial import inner membrane translocase subunit TIM44	NM_001013507.1
B6G0172	2.17	3.02	ODAD3	Outer dynein arm-docking complex subunit 3	NM_001077369.2
B2G0379	2.17	3.20	ZHX1	Zinc fingers and homeoboxes protein 1	NM_001007120.2
A18G0566	2.17	1.39	MTERF2	Transcription termination factor 2, mitochondrial	NM_001044828.2
B16G0166	2.17	3.27	ROHU_034445	A0A498LDF5_LABRO E3 ubiquitin-ligase TRIM39-like protein	
A21G0182	2.17	5.90	DENND1A	DENN domain-containing protein 1A	XM_005161496.4
A9G0660	2.16	3.10	UXS1	UDP-glucuronic acid decarboxylase 1	NM_173242.2
B23G0704	2.16	19.49	TUBA1A	Tubulin alpha-1A chain	NM_001105126.2
B21G0053	2.16	3.68	P4HA2	Prolyl 4-hydroxylase subunit alpha-2	XM_068215810.1
B23G0794	2.16	4.55	MAP7	Ensconsin	XM_068216944.1
B24G0198	2.16	1.34	RBBP8	DNA endonuclease RBBP8	XM_005174189.5
A20G0565	2.16	2.34	MDN1	Midasin	XM_003200703.6

B15G0888	2.16	2.46	IGSF11	Immunoglobulin superfamily member 11	NM_001278822.1
A7G0987	2.16	1.10	SAT2	Thialysine N-epsilon-acetyltransferase	XM_692551.6
A20G0159	2.16	1.12	STX7	Syntaxin-7	XM_021474444.2
B1G0998	2.16	1.01	ADGRE3	Adhesion G protein-coupled receptor E3	XM_021477530.2
B10G0699	2.16	3.27	FGFR1A	Fibroblast growth factor receptor 1-A	XM_005169218.5
B4G0150	2.16	1.91	RAD51AP1	RAD51-associated protein 1	NM_001044338.2
A25G0600	2.16	4.12	UEVLD	Ubiquitin-conjugating enzyme E2 variant 3	NM_001003619.2
A17G0741	2.16	3.88	EDRF1	Erythroid differentiation-related factor 1	XM_021473083.2
B14G0026	2.16	0.92	TRIM16	Tripartite motif-containing protein 16	XM_681071.8
B13G0357	2.16	3.81	POL	Retrovirus-related Pol polyprotein from transposon 412	XM_005156854.5
A16G0034	2.16	4.92	XRCC1	DNA repair protein XRCC1	NM_001003988.2
B25G0186	2.16	1.08	IGKV2-40	Immunoglobulin kappa variable 2-40	
B22G0494	2.16	1.30	G5714_021498	A0A7J6BR45_9TELE Ig-like domain-containing protein	XM_068216396.1
A9G0513	2.16	19.39	ETV5	ETS translocation variant 5	NM_001328592.1
B20G0665	2.16	2.51	TATDN3	Putative deoxyribonuclease tatdn3	NM_001020569.1
B25G0804	2.16	4.22	CCDC34	Coiled-coil domain-containing protein 34	
B16G0705	2.16	0.53	EFNA4	Ephrin-A4	XM_001342982.8
A14G0056	2.16	2.87	PMF1	Polyamine-modulated factor 1	NM_001386276.1
A2G0455	2.16	4.11	HS2ST1	Heparan sulfate 2-O-sulfotransferase 1	NM_001080670.1
B8G0562	2.16	3.09	RTASE	Probable RNA-directed DNA polymerase from transposon BS	XM_068222002.1
B19G0852	2.16	10.43	SLC25A32	carrier	NM_200256.1
A20G0135	2.16	3.03	GNPAT	Dihydroxyacetone phosphate acyltransferase	XM_002665777.7
B15G0048	2.16	1.47	SH2B2	SH2B adapter protein 2	XM_009291417.4
B21G0662	2.16	3.58	MRC1	Macrophage mannose receptor 1	XM_021469040.2
B21G0986	2.16	6.64	TMEM38B	Trimeric intracellular cation channel type B	NM_200176.1
A24G0462	2.16	1.50	SKIDA1	DACH domain-containing protein 1	XM_001342808.8
A7G1289	2.16	3.74	GIMAP4	GTPase IMAP family member 4	XM_068222510.1
B14G0676	2.16	2.39	LOC107661940	A0A671N9N2_9TELE Uncharacterized LOC107661940	
B4G0179	2.16	2.09	LYRM5A	LYR motif-containing protein 5A	
B21G0903	2.16	18.57	FECH	Ferrochelatase, mitochondrial	NM_131631.2
B16G0606	2.16	9.02	TGTP1	T-cell-specific guanine nucleotide triphosphate-binding protein 1	XM_688591.9
B23G0367	2.15	0.11			
B22G0828	2.15	1.20	LOC113040672	A0A6P6J3H2_CARAU uncharacterized protein LOC113040672	
B23G0446	2.15	7.77	AGRN	Agrin	XM_009296864.4
B13G0070	2.15	5.88	PPP3R1	Calcineurin subunit B type 1	XM_021480905.2
B6G0948	2.15	3.13	TUSC2	Tumor suppressor candidate 2	NM_001077299.2

B20G0667	2.15	19.20	FLVCR1	Feline leukemia virus subgroup C receptor-related protein 1	XM_005160739.4
A1G0128	2.15	0.79	GIMAP8	GTPase IMAP family member 8	
A17G0687	2.15	2.13	CNST	Consortin	XM_692809.8
B4G0233	2.15	5.25	ALDH1L2	Mitochondrial 10-formyltetrahydrofolate dehydrogenase	XM_002661372.7
A19G0925	2.15	8.59	TMEM65	Transmembrane protein 65	NM_001128812.1
A22G0514	2.15	3.43	RGS2	Regulator of G-protein signaling 2	
A10G0221	2.15	4.09	ANTXR1	Anthrax toxin receptor 1	XM_068224030.1
B20G0350	2.15	2.81	KITA	stem cell growth factor receptor kita	XM_009294635.4
A15G0818	2.15	1.43	CLDN4	Claudin-4	
B18G0435	2.15	8.39	GNA0	Guanine nucleotide-binding protein G(o) subunit alpha	XM_005159069.5
A5G0985	2.15	6.30	TBCEL	Tubulin-specific chaperone cofactor E-like protein	XM_002662169.7
B17G0551	2.15	2.29	KCTD3	POZ domain-containing protein KCTD3	XM_692476.10
A9G0402	2.15	10.65	EPB41L5	Band 4.1-like protein 5	NM_200089.2
A2G0741	2.15	1.90	TRAV18	T cell receptor alpha variable 18	
B24G0455	2.15	4.08	USF3	Basic helix-loop-helix domain-containing protein USF3	XM_005162733.5
B23G0534	2.15	9.28	DIP2BA	Disco-interacting protein 2 homolog B-A	XM_009296841.4
B15G0583	2.15	5.87	SLC6A4	Sodium-dependent serotonin transporter	XR_011006357.1
B10G0685	2.15	2.38			XM_068223820.1
B24G0031	2.15	5.14	TRIM16	Tripartite motif-containing protein 16	XM_005161933.5
B5G0723	2.14	7.47	PARP8	Protein mono-ADP-ribosyltransferase PARP8	NM_001077175.1
A4G0063	2.14	5.27	TRIM16	Tripartite motif-containing protein 16	XM_681071.8
B5G0296	2.14	4.61	ARHGAP31	Rho GTPase-activating protein 31	XM_005165631.5
B22G1009	2.14	1.49	FCRL5	Fc receptor-like protein 5	XM_068218085.1
A24G0033	2.14	0.28	RTASE	Probable RNA-directed DNA polymerase from transposon BS	
B12G0727	2.14	2.37	MMP25	Matrix metalloproteinase-25	XM_002663896.6
B12G0575	2.14	2.79	PPP1R16A	Protein phosphatase 1 regulatory subunit 16A	XM_068224737.1
B7G1296	2.14	0.54	PLA2R1	Secretory phospholipase A2 receptor	
B23G0717	2.14	1.91	HOXC8A	Homeobox protein Hox-C8a	NM_001005771.1
B3G1003	2.14	1.63	DEXI	Dexamethasone-induced protein homolog	NM_001003764.3
A9G0668	2.14	5.68	TNFSF13B	Tumor necrosis factor ligand superfamily member 13B	XM_017357686.3
A23G0131	2.14	4.31	ETV7	Transcription factor ETV7	NM_001083024.1
A8G0201	2.14	6.12	NRAS	GTPase NRas	NM_131145.2
B13G0763	2.14	5.13	MARCHF5	E3 ubiquitin-protein ligase MARCHF5	XM_068212993.1
B11G0531	2.14	4.94	TMEM178A	Transmembrane protein 178A	NM_001320431.1
A18G0342	2.14	6.63	LMO2	Rhombotin-2	NM_131111.2
B13G0110	2.14	4.19	MYOM2	Myomesin-2	NM_001328189.1
B13G0215	2.14	3.97	IKZF1	DNA-binding protein Ikaros	NM_001077552.1
A1G0516	2.14	4.20	LOC113078289	A0A6P6ND30_CARAU uncharacterized protein LOC113078289	XM_021476794.2
A25G0171	2.14	1.54	GDPGP1	GDP-D-glucose phosphorylase 1	

A5G0161	2.14	3.88	FBXO21	F-box only protein 21	XM_691038.8
A25G0424	2.14	2.11	TMEM60	Transmembrane protein 60	
B4G0176	2.14	1.27	RHNO1	RAD9, HUS1, RAD1-interacting nuclear orphan protein 1	
A17G0184	2.14	3.46	PACC1	Proton-activated chloride channel	NM_001291762.1
A3G1144	2.14	2.43	FAM20C	threonine protein kinase FAM20C	XM_009299891.4
B5G0832	2.14	8.58	LOC113049558	A0A6P6K761_CARAU uncharacterized protein LOC113049558 isoform X1	
A13G0564	2.14	9.57	DENND10	DENN domain-containing protein 10	XM_005156949.5
B21G0820	2.14	9.14	SEC16A	Protein transport protein Sec16A	XM_009292712.4
A21G0710	2.14	4.83	ZNF346	Zinc finger protein 346	NM_001081749.1
B1G0118	2.14	19.74	MXA	Interferon-induced GTP-binding protein MxA	NM_182942.4
B8G0904	2.14	2.25	CCDC120	Coiled-coil domain-containing protein 120	XM_009303768.4
A2G0606	2.14	2.60	RDH10B	Retinol dehydrogenase 10-B	NM_201331.1
A17G0335	2.14	8.33	ITSN2	Intersectin-2	NM_001003547.2
A15G0154	2.14	4.23	NGEF	Ephexin-1	XM_692454.7
B17G0795	2.14	1.83	SUPT7L	STAGA complex 65 subunit gamma	NM_001013292.2
B25G0860	2.14	7.90	GRAMD2A	GRAM domain-containing protein 2A	XM_017354145.3
A2G0042	2.14	6.05	IRF4	Interferon regulatory factor 4	NM_001122710.1
B7G0975	2.14	2.23	ADAMTSL3	ADAMTS-like protein 3	XM_068222541.1
B20G0731	2.14	1.94	DCBLD1	Discoidin, CUB and LCCL domain-containing protein 1	NM_001039977.2
B5G1059	2.14	3.34	ERAP1	Endoplasmic reticulum aminopeptidase 1	NM_200206.2
B3G0083	2.14	6.80	TCB1	Transposable element Tcb1 transposase	
B5G0390	2.14	4.10	MAN2A1	Alpha-mannosidase 2	NM_001110027.2
B6G0716	2.14	17.50	ENOA	Alpha-enolase	NM_200695.1
B10G0762	2.14	5.18	RILPL1	RILP-like protein 1	NM_001079664.1
A2G0998	2.14	5.67	MUL1A	Mitochondrial ubiquitin ligase activator of nfkb 1-A	NM_001018124.1
B21G0556	2.13	1.36	G5714_020656	A0A7J6BUG0_9TELE Uncharacterized protein	
A16G0522	2.13	12.04	NR4A3	Nuclear receptor subfamily 4 group A member 3	XM_009292332.4
A6G0324	2.13	1.71	IGFBP5	Insulin-like growth factor-binding protein 5	NM_001098754.1
A17G0866	2.13	1.69	CEP170B	Centrosomal protein of 170 kDa protein B	XM_021467255.2
B5G1065	2.13	3.25	CCDC125	Coiled-coil domain-containing protein 125	NM_001098182.1
A2G0631	2.13	5.26	EMILIN2	EMILIN-2	XM_001341633.9
A5G0433	2.13	5.03	CEMIP2	Cell surface hyaluronidase	XM_005171900.5
A4G0012	2.13	1.24	DPX16_2480	A0A3N0Z6T3_ANAGA Zinc finger protein 84	XM_068220573.1
A13G0682	2.13	3.69	NFU1	NFU1 iron-sulfur cluster scaffold homolog, mitochondrial	NM_001077552.1
B15G0422	2.13	1.39	CHP1	Calcineurin B homologous protein 1	NM_199836.2
A9G0010	2.13	5.56	RAB3GAP1	Rab3 GTPase-activating protein catalytic subunit	NM_001077788.2

B8G0628	2.13	2.13	TNFRSF14	Tumor necrosis factor receptor superfamily member 14	NM_001430990.1
A3G0091	2.13	0.24	GIMAP8	GTPase IMAP family member 8	
B14G0032	2.13	2.52	FGF2	Fibroblast growth factor 2	NM_212823.2
B16G0889	2.13	2.95	AHR	Aryl hydrocarbon receptor	NM_001430948.1
A4G0020	2.13	3.60	NLRC3	NLR family CARD domain-containing protein 3	XM_017352412.3
B5G0204	2.13	3.43	YDJC	Carbohydrate deacetylase	XM_068221292.1
A12G0762	2.13	0.71	TCB1	Transposable element Tcb1 transposase	XM_021471168.2
B20G0508	2.13	4.26	MBOAT2	Lysophospholipid acyltransferase 2	NM_001278602.1
A6G0707	2.13	15.34	ENOA	Alpha-enolase	NM_200695.1
B20G0368	2.13	7.49	SLAIN2	SLAIN motif-containing protein 2	XM_017352483.3
B23G0728	2.13	2.92	ZNF740	Zinc finger protein 740	NM_001076631.2
A4G0506	2.13	4.27	PRKCQ	Protein kinase C theta type	XM_068219633.1
B25G0180	2.13	2.81	IGKV2-29	Immunoglobulin kappa variable 2-29	
A4G0410	2.13	1.48	A0A673IFZ5	A0A673IFZ5_9TELE Uncharacterized protein	XM_009300496.4
B21G0858	2.13	3.09	DOLPP1	Dolichyldiphosphatase 1	NM_001007061.1
B10G0097	2.13	12.34			
B9G0508	2.13	12.39	STK17B	threonine-protein kinase 17B	NM_200535.1
A5G0295	2.13	4.84	CMLKR1	Chemerin-like receptor 1	NM_001082812.2
B16G0024	2.13	1.84	TRIM16	Tripartite motif-containing protein 16	XM_681071.8
A1G0830	2.13	1.65	LOC107702537	A0A671MNN8_9TELE Uncharacterized LOC107702537	NM_001013300.2
B16G0456	2.13	14.59	CHD4	Chromodomain-helicase-DNA-binding protein 4	XM_680607.10
B11G0791	2.13	14.85			
B19G0499	2.13	17.02	SDC3	Syndecan-3	XM_001919721.8
A5G1295	2.13	8.93	SMTN	Smoothelin	XM_068221574.1
B14G0162	2.13	2.18	MTUS1A	Microtubule-associated tumor suppressor 1 homolog A	XM_068213222.1
A14G0714	2.13	54.01	ISK1L	Trypsin inhibitor ClTI-1	
A14G0422	2.12	4.86	PROM1A	Prominin-1-A	XM_068213162.1
A12G0338	2.12	6.75	ZNF438	Zinc finger protein 438	NM_001077161.1
B3G1107	2.12	6.93	TLK2	threonine-protein kinase tousled-like 2	NM_001110417.1
B16G0964	2.12	3.34	CEP192	Centrosomal protein of 192 kDa	XM_009292113.4
A19G0592	2.12	0.86	EIF3EB	Eukaryotic translation initiation factor 3 subunit E-B	NM_001362621.1
A19G0686	2.12	7.43	CRABP2	Cellular retinoic acid-binding protein 2	NM_001320394.1
A3G0175	2.12	4.11	TRIM25	ISG15 ligase TRIM25	NM_001007387.2
B5G0122	2.12	8.26	NIPBLA	Nipped-B-like protein A	NM_001246284.1
B10G0476	2.12	3.24	CKAP2	Cytoskeleton-associated protein 2	XM_692182.8
A12G0253	2.12	9.95	VTI1A	Vesicle transport through interaction with t-SNAREs homolog 1A	XM_005156335.4
A3G0280	2.12	3.49	WIZ	Protein Wiz	XM_001341758.9
A24G0242	2.12	1.13			XM_017353846.3
A2G0641	2.12	9.42	MRC1	Macrophage mannose receptor 1	NM_001423726.1
B3G0695	2.12	15.12	TRAF7	E3 ubiquitin-protein ligase TRAF7	XM_009299553.3
A13G0135	2.12	4.56	RMDN2	Regulator of microtubule dynamics protein 2	NM_212608.1
B17G0707	2.12	5.62	THBD	Thrombomodulin	

A17G0506	2.12	6.48	RASGRP3	Ras guanyl-releasing protein 3	NM_001110464.1
B5G0363	2.12	4.53	LIFR	Leukemia inhibitory factor receptor	XM_068221529.1
A12G0301	2.12	2.57	PNPO	Pyridoxine-5'-phosphate oxidase	NM_001256178.1
B16G0051	2.12	4.00	FZD1	Frizzled-1	NM_001130614.1
B7G1225	2.12	6.36	KLC2	Kinesin light chain 2	NM_001083550.1
A19G0757	2.12	1.89	LSM10	U7 snRNA-associated Sm-like protein LSm10	NM_001386623.1
A13G0748	2.12	2.21	THADA	Thyroid adenoma-associated protein homolog	NM_001017559.1
A14G0730	2.12	2.86	MAML1	Mastermind-like protein 1	XM_690627.8
B12G0764	2.12	1.08	RADIL	Ras-associating and dilute domain-containing protein	
A21G0645	2.12	1.67	ASL	Argininosuccinate lyase	NM_200451.1
A1G0716	2.12	3.98	TCB1	Transposable element Tcb1 transposase	XM_021471168.2
B5G0111	2.12	19.16	ME2	NAD-dependent malic enzyme, mitochondrial	NM_001003627.2
A3G0227	2.12	0.76	CYP2K1	Cytochrome P450 2K1	XM_005163733.5
A10G0377	2.12	5.84	SMG8	Nonsense-mediated mRNA decay factor SMG8	XM_690719.10
A10G0051	2.12	2.49	C3AR1	C3a anaphylatoxin chemotactic receptor	NM_001423804.1
A21G0210	2.12	0.79	ARID5A	AT-rich interactive domain-containing protein 5A	NM_001080201.1
B14G0424	2.12	3.75	CCNA2	Cyclin-A2	NM_152949.1
A23G0782	2.12	1.80	CYP2B15	Cytochrome P450 2B15	NM_001109701.2
A7G1022	2.12	4.55	TKFC	FMN cyclase	NM_001076630.1
B22G0354	2.12	5.93	A0A671R154	A0A671R154_9TELE Ig-like domain-containing protein	NM_001135552.1
A15G0084	2.12	2.51	LRRC51	Leucine-rich repeat-containing protein 51	NM_001076768.1
B3G1564	2.11	27.71	G5714_002846	A0A7J6D7V4_9TELE Uncharacterized protein	
A13G0004	2.11	1.61	SLC30A10	Zinc transporter 10	XM_068213863.1
A3G1174	2.11	3.15	XYLT2	Xylosyltransferase 2	NM_001130778.2
A23G0222	2.11	1.03	MAPK12	Mitogen-activated protein kinase 12	NM_001111167.1
B9G0727	2.11	2.84	PVR	Poliovirus receptor	XM_005167422.5
B12G0296	2.11	5.80	PNPO	Pyridoxine-5'-phosphate oxidase	NM_001256178.1
B16G0570	2.11	2.49	ALG2	1,6-mannosyltransferase ALG2	NM_001104936.2
A16G1047	2.11	6.72	RPRD2	Regulation of nuclear pre-mRNA domain-containing protein 2	XM_679241.10
B13G0556	2.11	2.70	A0A672QUA0	A0A672QUA0_SINGR SCY domain-containing protein	XM_068220136.1
A22G0579	2.11	17.78	CXCL12	Stromal cell-derived factor 1	NM_001320414.1
B14G0334	2.11	4.08	SPDL1	Protein Spindly	NM_001104942.1
B18G0017	2.11	1.43	TC1A	Transposable element Tc1 transposase	
A20G0582	2.11	9.41	FRYL	Protein furry homolog-like	XM_009294673.4
A2G0637	2.11	7.16	COLEC12	Collectin-12	NM_001122840.1
A25G0400	2.11	1.91	TNNT2	Troponin T, cardiac muscle	NM_001025179.1
A13G0280	2.11	3.97	TRIP11	Thyroid receptor-interacting protein 11	NM_001159828.1
A25G0751	2.11	6.48	H2B	Histone H2B	NM_001013463.2
A14G0628	2.11	9.72	PCDHGA1	Protocadherin gamma-A1	NM_001024201.1
A23G0791	2.11	5.40	STK35	threonine-protein kinase 35	NM_001077616.1
A12G0766	2.11	20.06	ALDOA	Fructose-bisphosphate aldolase A	NM_213215.1
B18G0462	2.11	2.73	CALB2	Calretinin	NM_200718.1
B9G0783	2.11	8.77	MYO1B	Unconventional myosin-Ib	XM_003199193.6

A13G0794	2.11	1.53	PSAP	Prosaposin	NM_001017687.1
B15G0678	2.11	17.20	NXPE3	NXPE family member 3	XM_001345784.9
A18G0733	2.11	9.59	CAPN5	Calpain-5	NM_001080007.1
A3G0743	2.11	5.99	MAP3K3	Mitogen-activated protein kinase kinase kinase 3	XM_683602.8
B14G0389	2.11	5.57	TMEM179	Transmembrane protein 179	
B8G0626	2.11	2.86	TNFRSF14	Tumor necrosis factor receptor superfamily member 14	XM_068223141.1
A14G0054	2.11	0.49	CLCF1	Cardiotrophin-like cytokine factor 1	XM_685641.9
B23G0751	2.11	1.30	MUL1	Mitochondrial ubiquitin ligase activator of NFKB 1	NM_001098760.1
A3G0304	2.11	5.46	LILRA1	Leukocyte immunoglobulin-like receptor subfamily A member 1	XM_068218085.1
B2G1124	2.11	7.97	PRDX1	Peroxiredoxin-1	NM_001013471.2
B19G0613	2.11	1.04	CCN4	CCN family member 4	NM_001166230.1
B19G0598	2.11	3.79	COL8A2	Collagen alpha-2(VIII) chain	XM_068215201.1
A4G0516	2.11	4.34	A0A671THQ2	A0A671THQ2_SPAAU THAP-type domain-containing protein	
B22G0344	2.11	2.30	DPX16_4323	A0A3N0YSU6_ANAGA Zinc finger protein 721	XM_068219920.1
B9G0513	2.11	0.94	TMEFF2	Tomoregulin-2	NM_001128365.1
B1G0511	2.10	0.00			
A2G0292	2.10	0.62	SET-1	Histone-lysine N-methyltransferase set-1	
A23G0836	2.10	1.16	COL24A1	Collagen alpha-1(XXIV) chain	XM_068216816.1
A23G0706	2.10	4.63	ACAP3	Arf-GAP with coiled-coil, ANK repeat and PH domain-containing protein 3	XM_068216935.1
A9G0279	2.10	1.06	ILDR1	Immunoglobulin-like domain-containing receptor 1	NM_001004642.2
B11G0028	2.10	6.44	VGLL4	Transcription cofactor vestigial-like protein 4	NM_001017677.1
A19G0755	2.10	4.64	ZC3H12A	Endoribonuclease ZC3H12A	NM_001171029.1
A2G0942	2.10	9.37	ESYT2-A	Extended synaptotagmin-2-A	XM_005171399.5
A15G0151	2.10	1.06			
B6G0973	2.10	3.95	NAV1	Neuron navigator 1	XM_068222077.1
B23G0848	2.10	4.62	G5714_022278	A0A7J6BMP2_9TELE Uncharacterized protein	
A1G0133	2.10	0.11	ADGRE3	Adhesion G protein-coupled receptor E3	XM_017356711.3
A3G0363	2.10	2.88	LOC107588011	A0A672N126_SINGR Zinc finger protein 239-like	XR_011010832.1
B2G1069	2.10	7.08	ZZZ3	ZZ-type zinc finger-containing protein 3	NM_001100027.1
A10G0069	2.10	1.89	SETD9	SET domain-containing protein 9	XM_001332769.6
A3G0956	2.10	14.20	UBTF	Nucleolar transcription factor 1	NM_001005395.1
B11G0396	2.10	3.69	CHIA	Acidic mammalian chitinase	XM_017358249.3
A20G0556	2.10	1.55	MOXD1	DBH-like monooxygenase protein 1 homolog	XM_005160591.5
A3G0926	2.10	2.63	LOC107552485	A0A672RL98_SINGR Zinc finger protein 16-like	NM_213535.1
A7G0239	2.10	2.88	ADGRG3	Adhesion G protein-coupled receptor G3	XM_021478015.2
A13G0057	2.10	3.55	SIPA1L2	Signal-induced proliferation-associated 1-like protein 2	NM_214814.1
A11G0363	2.10	2.20	MYH7B	Myosin-7B	NM_001013545.2
A5G0777	2.10	2.03	FANCG	Fanconi anemia group G protein homolog	NM_205639.1
B9G0874	2.10	8.52	FMNL2	Formin-like protein 2	XM_001345159.9

A10G0360	2.10	2.14	SMYD4	SET and MYND domain-containing protein 4	NM_001076594.1
A3G1172	2.10	9.46	SLC9A3R1	H(+) exchange regulatory cofactor NHE-RF1	XM_005174539.5
A14G0680	2.10	1.88	RNF20	E3 ubiquitin-protein ligase BRE1A	NM_001256175.1
B13G0438	2.10	1.60	LOC113056991	A0A6P6L8F8_CARAU uncharacterized protein LOC113056991	NM_181662.2
A5G0268	2.10	4.75	UBE3B	Ubiquitin-protein ligase E3B	NM_001114682.1
B16G0277	2.10	7.73	CMTM7	CKLF-like MARVEL transmembrane domain-containing protein 7	NM_001130114.1
A14G0318	2.10	175.38	LECT2	Leukocyte cell-derived chemotaxin-2	NM_001048055.1
B3G1527	2.10	0.97	ROHU_000950	A0A498P350_LABRO Serine threonine-kinase pim-2-like protein	
B14G0337	2.10	8.26	GSR	Glutathione reductase, mitochondrial	XM_017359015.3
B4G0781	2.10	1.95	NLRC3	NLR family CARD domain-containing protein 3	XM_068218404.1
B23G0475	2.10	4.59	SYT6	Synaptotagmin-6	XM_017353709.3
B2G0326	2.09	3.27	CSPP1	Centrosome and spindle pole-associated protein 1	XM_068214441.1
A23G0679	2.09	0.05	HOXC11A	Homeobox protein Hox-C11a	NM_131165.2
A11G0770	2.09	6.79	PDRG1	p53 and DNA damage-regulated protein 1	XM_002663842.6
B2G0661	2.09	4.57	CHMP5	Charged multivesicular body protein 5	XM_681842.10
B5G0763	2.09	2.18	LOC107746401	A0A673MWV3_9TELE Zinc finger protein 629-like	XM_001923522.8
B21G0224	2.09	1.67	UBTD2	Ubiquitin domain-containing protein 2	NM_001002718.2
B14G0794	2.09	2.28	ACOD1	Cis-aconitate decarboxylase	NM_001077607.1
A8G0284	2.09	1.26	CDKN2A	Cyclin-dependent kinase inhibitor 2A	NM_001199994.1
A13G0766	2.09	5.61	YIPF3	Protein YIPF3	XM_005169532.5
A25G0148	2.09	1.94	KCNJ11	ATP-sensitive inward rectifier potassium channel 11	NM_001012387.3
B16G0731	2.09	3.96	ZNF687A	Zinc finger protein 687a	NM_001353864.1
B20G0356	2.09	3.53	SCFD2	Sec1 family domain-containing protein 2	NM_001013564.2
B2G1161	2.09	9.18	PCYT1A	Choline-phosphate cytidylyltransferase A	XM_021480218.2
B18G0787	2.09	1.22	KCTD15	POZ domain-containing protein kctd15	NM_001003527.1
B16G0623	2.09	2.41	ADNP2	Activity-dependent neuroprotector homeobox protein 2	NM_001098265.1
B23G0795	2.09	0.67	XCR1	Chemokine XC receptor 1	
B7G1302	2.09	1.63	PLA2R1	Secretory phospholipase A2 receptor	
B3G1434	2.09	1.84	TVP23B	Golgi apparatus membrane protein TVP23 homolog B	NM_001017729.1
A21G0590	2.09	1.75	NRG2	Pro-neuregulin-2, membrane-bound isoform	XM_009295460.4
A18G0403	2.09	4.86	BCAR1	Breast cancer anti-estrogen resistance protein 1	XM_021467686.2
B3G0874	2.09	0.30	FRIM	Ferritin, middle subunit	NM_001109854.1
B25G0279	2.09	7.80	UBL7	Ubiquitin-like protein 7	NM_001017765.2
A1G0083	2.09	6.57	NLRC3	Protein NLRC3	XM_068222147.1

B21G0175	2.09	2.31	MNT	Max-binding protein MNT	NM_001103111.1
B2G0373	2.09	6.47	PRMT5	Protein arginine N-methyltransferase 5	NM_001007183.2
B2G0135	2.09	3.71	CD300LF	CMRF35-like molecule 1	XR_011007163.1
B16G0960	2.09	3.22	CEP76	Centrosomal protein of 76 kDa	NM_001103119.2
A1G0300	2.09	6.43	PSEN2	Presenilin-2	NM_131514.2
A19G0914	2.09	3.17	VPS72	Vacuolar protein sorting-associated protein 72 homolog	NM_001082944.1
B14G0737	2.09	1.40	MXD3	Max dimerization protein 3	NM_201056.1
A7G0850	2.09	0.06	A0A671N790	A0A671N790_9TELE Small nuclear RNA activating complex, polypeptide 2	
A9G0717	2.09	15.24	EPSTI1	Epithelial-stromal interaction protein 1	
B5G1170	2.09	6.63	ATRX	Transcriptional regulator ATRX	NM_001423350.1
B13G0125	2.09	7.41	SLC29A3	Equilibrative nucleoside transporter 3	XM_005169532.5
B6G0315	2.09	2.21	TGFBRAP1	Transforming growth factor-beta receptor-associated protein 1 homolog	NM_001083554.1
A21G0753	2.09	0.92	TRAF4	TNF receptor-associated factor 4	NM_212817.2
B11G0713	2.09	9.27	TDRD3	Tudor domain-containing protein 3	
B22G0493	2.09	1.52	LOC113040442	A0A6P6J536_CARAU gastrula zinc finger protein XlCGF52.1-like	NM_001012485.1
B12G0301	2.09	3.98	PDK2	[Pyruvate dehydrogenase (acetyl-transferring)] kinase isozyme 2, mitochondrial	XM_001332558.7
B3G1559	2.09	1.90	A0A673M8N8	A0A673M8N8_9TELE Uncharacterized protein	XM_017355493.3
B2G0873	2.08	4.35	ZNF414	Zinc finger protein 414	XM_021480706.2
A1G0988	2.08	5.51	LOC113107252	A0A6P6PV59_CARAU uncharacterized protein LOC113107252	
B12G0655	2.08	1.61	EGR2B	Early growth response protein 2b	NM_130997.2
B1G0185	2.08	1.63	PGAP6	Post-GPI attachment to proteins factor 6	XM_005170863.5
A6G0307	2.08	1.71	ST6GAL2	Beta-galactoside alpha-2,6-sialyltransferase 2	NM_001171221.1
A25G0699	2.08	3.55	HPRT1	Hypoxanthine-guanine phosphoribosyltransferase	
B21G0325	2.08	1.15	TNFSF10	Tumor necrosis factor ligand superfamily member 10	NM_001042713.1
A7G0468	2.08	3.87	RTASE	Probable RNA-directed DNA polymerase from transposon BS	XM_068220297.1
B24G0218	2.08	6.90	RB1CC1	RB1-inducible coiled-coil protein 1	XM_009302198.4
A11G0648	2.08	3.51	ABCA1	Phospholipid-transporting ATPase ABCA1	XM_678953.6
B25G0351	2.08	1.39	POT1	Protection of telomeres protein 1	NM_001245960.1
B16G0824	2.08	5.87	LOC107692552	A0A671KBR4_9TELE Uncharacterized LOC107692550	NM_001113584.2
A11G0187	2.08	1.52	TXLNG	Gamma-taxilin	
B20G0908	2.08	0.43	IRF4	Interferon regulatory factor 4	XM_021468723.2
A4G0028	2.08	1.33	ALOX5	Polyunsaturated fatty acid 5-lipoxygenase	NM_001045331.1
B3G0202	2.08	7.08	CSDC2	Cold shock domain-containing protein C2	

B7G0873	2.08	11.32	MARK2	threonine-protein kinase MARK2	XM_009303151.4
B7G0176	2.08	9.26	MICAL2B	[F-actin]-monooxygenase mical2b	XM_021478018.2
B10G0105	2.08	4.73	RTASE	Probable RNA-directed DNA polymerase from transposon BS	XM_068222501.1
A2G0038	2.08	1.35	LOC107662522	A0A671NF28_9TELE Gastrula zinc finger protein XlCGF28.1-like	XM_021470910.2
B4G0890	2.08	4.78	TCB1	Transposable element Tcb1 transposase	XM_068216952.1
B15G0523	2.08	3.32	EXO5	Exonuclease V	
A4G0226	2.08	0.42	ROHU_006198	A0A498MU84_LABRO Sterile alpha motif domain-containing 3	
B5G0272	2.08	4.25	MEPCE	7SK snRNA methylphosphate capping enzyme	NM_001128529.1
B9G0530	2.08	1.46	GTDC1	Glycosyltransferase-like domain-containing protein 1	XM_005167557.5
A12G0151	2.08	2.89	HS3ST3B1	Heparan sulfate glucosamine 3-O-sulfotransferase 3B1	NM_001080568.1
A17G0550	2.08	6.15	CCDC6	Coiled-coil domain-containing protein 6	NM_199985.2
A7G0877	2.08	10.35	LOC113052094	A0A6P6KIQ1_CARAU uncharacterized protein LOC113052094	
B2G1152	2.08	1.34	SLC35A3	UDP-N-acetylglucosamine transporter	XM_005171166.5
A5G0766	2.08	1.78	DTX3	Probable E3 ubiquitin-protein ligase DTX3	NM_001123062.1
A7G0492	2.08	1.48	SHCBP1	SHC SH2 domain-binding protein 1	NM_199843.2
B4G0125	2.08	10.51	BCL2L14	Apoptosis facilitator Bcl-2-like protein 14	
A23G0585	2.08	1.42	LOC113065346	A0A6P6M6L9_CARAU uncharacterized protein LOC113065346	
B7G1003	2.08	5.32	FES	Fps	XM_005166383.5
B18G0622	2.08	2.15	WFDC1	WAP four-disulfide core domain protein 1	NM_001100000.1
B12G0437	2.08	5.65	EP300	Histone acetyltransferase p300	XM_021474237.2
B5G0211	2.08	3.50	G5714_006321	A0A7J6D3H9_9TELE Uncharacterized protein	XR_011015642.1
B15G0265	2.08	3.63	GPR4	G-protein coupled receptor 4	NM_001309853.1
A12G0316	2.08	2.95	SPATA20	Spermatogenesis-associated protein 20	XM_001920553.7
B3G1262	2.08	11.03	HMOX	Heme oxygenase	NM_001127516.1
B8G1034	2.08	2.96	ZNF366	Zinc finger protein 366	XM_693094.9
A7G0244	2.08	2.09	NADSYN1	Glutamine-dependent NAD(+) synthetase	NM_001099253.1
A14G0489	2.08	2.30	NOA1	Nitric oxide-associated protein 1	
A25G0783	2.08	3.10	FANCA	Fanconi anemia group A protein homolog	
B17G0759	2.07	3.14	REEP1	Receptor expression-enhancing protein 1	XM_021467468.2
A13G0232	2.07	9.80	TACC3	Transforming acidic coiled-coil-containing protein 3	XM_021472697.2
B3G1021	2.07	3.08	EHD4	EH domain-containing protein 4	XM_702892.9
B9G0472	2.07	1.11	MAP3K19	Mitogen-activated protein kinase kinase kinase 19	XM_068223601.1
B16G0482	2.07	8.82	SLC45A4	Solute carrier family 45 member 4	XM_017351511.3
A16G0213	2.07	2.37	THBS3A	Thrombospondin-3a	NM_173225.2

B16G0019	2.07	1.72	TRIM16	Tripartite motif-containing protein 16	XM_681071.8
B7G0126	2.07	14.45	TSPAN5	Tetraspanin-5	NM_001006047.2
A17G0129	2.07	0.19	A0A4W4E526	A0A4W4E526_ELEEL Ig-like domain-containing protein	
A18G0330	2.07	0.72	TMEM123	Porimin	
B10G0036	2.07	6.16	CHD1	Chromodomain-helicase-DNA-binding protein 1	XM_009305101.4
A17G0799	2.07	2.19	CHRNA2	Neuronal acetylcholine receptor subunit alpha-2	NM_001040327.1
A12G0163	2.07	2.96	OXLD1	Oxidoreductase-like domain-containing protein 1	
A14G0075	2.07	3.57	ENOX2	Ecto-NOX disulfide-thiol exchanger 2	XM_005173148.5
B16G0084	2.07	0.83	FAM110C	Protein FAM110C	NM_001082926.1
B15G0233	2.07	2.21	NARS2	Probable asparagine--tRNA ligase, mitochondrial	NM_001077155.1
A5G1286	2.07	5.28	VPS33A	Vacuolar protein sorting-associated protein 33A	NM_001099973.1
B16G0724	2.07	1.28	ROHU_000496	A0A498P5B8_LABRO Uncharacterized protein	
B5G0231	2.07	9.33	RGS3	Regulator of G-protein signaling 3	NM_001430835.1
B17G0347	2.07	3.08	TMEM26	Transmembrane protein 26	XM_021473112.2
B4G0204	2.07	6.18	CDK17	Cyclin-dependent kinase 17	NM_001201357.1
A18G0043	2.07	3.21	TIRAP	interleukin-1 receptor domain-containing adapter protein	
A23G0045	2.07	23.62	SGK2	threonine-protein kinase Sgk2	NM_001113622.1
B22G0795	2.07	0.53	C2CD4C	C2 calcium-dependent domain-containing protein 4C	XM_005170537.5
B3G1033	2.07	3.67	NKIRAS2	NF-kappa-B inhibitor-interacting Ras-like protein 2	NM_001003433.1
B25G0244	2.07	12.77	DUSP8	Dual specificity protein phosphatase 8	NM_200880.1
A17G0905	2.07	21.69	DYNC1H1	Cytoplasmic dynein 1 heavy chain 1	NM_001042745.1
A1G1083	2.07	2.93	NCAPG	Condensin complex subunit 3	NM_001256205.1
B19G0468	2.07	1.12	NKD2	Protein naked cuticle homolog 2	NM_001195615.2
A16G0838	2.07	1.50	OSBPL3	Oxysterol-binding protein-related protein 3	XM_009292271.3
A10G0173	2.07	5.61	HAPLN1	Hyaluronan and proteoglycan link protein 1	XM_009305685.4
B3G1531	2.07	1.45	LOC113039841	A0A6P6J106_CARAU gastrula zinc finger protein XlCGF7.1-like	NM_001114486.1
A17G0118	2.07	1.06	TRBV30	T cell receptor beta variable 30	
B14G0680	2.07	53.31	ASPG	L-asparaginase	
B7G0117	2.07	1.68	GBA3	Cytosolic beta-glucosidase	NM_001020693.1
B22G0456	2.07	1.10	LOC113040442	A0A6P6J536_CARAU gastrula zinc finger protein XlCGF52.1-like	XM_021473962.2
B18G0149	2.07	2.72	TCB1	Transposable element Tcb1 transposase	XM_021471168.2
A8G0300	2.07	4.86	PLK2	threonine-protein kinase PLK2	NM_001099245.1
B1G0788	2.07	24.47	AGPAT3	1-acyl-sn-glycerol-3-phosphate acyltransferase gamma	NM_213425.1
A10G0386	2.07	2.43	RFXAP	Regulatory factor X-associated protein	NM_001326367.1
A11G0641	2.07	3.02	IFFO2	Intermediate filament family orphan 2	NM_001423793.1
A17G0783	2.07	3.55	SMC6	Structural maintenance of chromosomes protein 6	XM_021473054.2

A17G0848	2.07	0.17	HARBI1	Putative nuclease HARBI1	XM_068220297.1
B7G0672	2.06	1.90	GLCEB	D-glucuronyl C5-epimerase B	XM_005168839.4
A25G0186	2.06	62.32	COX8B	Cytochrome c oxidase subunit 8B, mitochondrial	
A3G0307	2.06	20.41	LOC113066474	A0A6P6MC78_CARAU uncharacterized protein LOC113066474	
B2G0254	2.06	5.84	NCAPD2	Condensin complex subunit 1	NM_001162502.2
B18G0676	2.06	5.16	IMPDH1A	Inosine-5'-monophosphate dehydrogenase 1a	XM_005159007.5
B23G0576	2.06	2.82	CASZ1	Zinc finger protein castor homolog 1	XM_005162286.5
A16G0989	2.06	2.01	ZC3H12A	Endoribonuclease ZC3H12A	XM_021466808.2
A21G0613	2.06	2.42	FAM53C	Protein FAM53C	NM_001111239.2
B19G0025	2.06	8.70	LOC113075365	A0A6P6N494_CARAU uncharacterized protein LOC113075365	
B21G0010	2.06	1.15	LOC113038186	A0A6P6IV53_CARAU uncharacterized protein LOC113038186 isoform X1	
B23G0746	2.06	2.38	CPTP	Ceramide-1-phosphate transfer protein	NM_001003497.1
B10G0851	2.06	9.55	LRRC32	Transforming growth factor beta activator LRRC32	
A13G0511	2.06	3.10	SUPV3L1	ATP-dependent RNA helicase SUPV3L1, mitochondrial	NM_001044333.2
B22G0177	2.06	3.70	MATK	Megakaryocyte-associated tyrosine-protein kinase	
A10G0807	2.06	3.52	TMEM267	Transmembrane protein 267	NM_001003574.2
A22G0077	2.06	0.49	LOC113075417	A0A6P6N4F7_CARAU uncharacterized protein LOC113075417	XR_002456444.2
B20G0740	2.06	10.06	AFDN	Afadin	XM_021468691.2
A13G0784	2.06	9.50	FAM120B	Constitutive coactivator of peroxisome proliferator-activated receptor gamma	XM_068224874.1
B25G0684	2.06	1.60	LOC113098358	A0A6P6PCY5_CARAU uncharacterized protein LOC113098358	XR_011008815.1
B12G0291	2.06	6.61	CBX1	Chromobox protein homolog 1	XM_005156237.5
B3G0094	2.06	3.69	TCB1	Transposable element Tcb1 transposase	XM_021471168.2
A20G0873	2.06	4.56	RNGTT	mRNA-capping enzyme	NM_212867.1
B4G0062	2.06	2.45	GAS2L3	GAS2-like protein 3	NM_001045098.1
A24G0477	2.06	0.44	RTTN	Rotatin	XM_068217176.1
A19G0941	2.06	12.32	COL14A1	Collagen alpha-1(XIV) chain	XM_068215216.1
B10G0648	2.06	4.39	GIT2	ARF GTPase-activating protein GIT2	NM_001080188.2
B16G0667	2.06	7.36	CADM4	Cell adhesion molecule 4	XM_005158112.3
B12G0630	2.06	4.36	ATXN7L3	Ataxin-7-like protein 3	NM_001326694.1
B18G0217	2.06	0.00	ROHU_006041	A0A498N6V7_LABRO Sterile alpha motif domain-containing 3-like isoform X2	
B15G0900	2.06	1.41	CHD	Chordin	NM_130973.3
B8G0686	2.06	9.25	NOTCH2	Neurogenic locus notch homolog protein 2	NM_001115094.2
A3G1048	2.06	5.57	MRTFA	Myocardin-related transcription factor A	XM_068218103.1
B20G0250	2.06	3.83	DHCR24	Delta(24)-sterol reductase	NM_001008645.1
A19G0749	2.06	5.69	HIVEP1	Zinc finger protein 40	XM_021468250.2
B18G0552	2.06	2.79	TANGO6	Transport and Golgi organization protein 6 homolog	XM_009293595.3

B7G0357	2.06	8.37	TDRD7B	Tudor domain-containing protein 7B	NM_001353929.1
B24G0336	2.06	6.69	SKIL	Ski-like protein	XM_005162773.5
A23G0213	2.06	4.68	FOXP1B	Forkhead box protein P1-B	XM_005161959.4
B3G0154	2.06	1.68	LOC113074991	A0A6P6N523_CARAU gastrula zinc finger protein XlCGF57.1-like isoform X1	XM_017354882.3
B14G0325	2.06	3.68	CTSO	Cathepsin O	XM_690625.9
B6G0431	2.05	12.32	LOC107733913	A0A673KZF6_9TELE Uncharacterized LOC107733913	
B9G0634	2.05	0.61			
A1G0349	2.05	1.21			
A21G0339	2.05	1.02	HPSE	Heparanase	NM_001045005.1
B11G0557	2.05	0.73	LIMS1	LIM and senescent cell antigen-like-containing domain protein 1	
A8G0263	2.05	2.95	QSOX1	Sulfhydryl oxidase 1	NM_001128364.1
A4G0197	2.05	2.22	LOC113047446	A0A6P6JXS5_CARAU gastrula zinc finger protein XlCGF57.1-like isoform X1	XM_068220573.1
A19G0285	2.05	12.73	PAG1	Phosphoprotein associated with glycosphingolipid-enriched microdomains 1	XM_021473350.2
B13G0730	2.05	3.05	SLC35F4	Solute carrier family 35 member F4	NM_001365219.1
B17G0349	2.05	1.30	BICC1-B	Protein bicaudal C homolog 1-B	XM_068218870.1
A16G0681	2.05	1.91	NSMAF	Protein FAN	NM_001045031.1
A15G0009	2.05	1.34	SOCS5	Suppressor of cytokine signaling 5	NM_001113801.1
B20G0518	2.05	1.75	CCR6	C-C chemokine receptor type 6	NM_001099991.1
B7G1271	2.05	0.24	LOC113105422	A0A6P6PPL3_CARAU uncharacterized protein LOC113105422	XM_021477654.2
B17G0216	2.05	4.82	KHK	Ketohexokinase	XM_021467144.2
B15G0898	2.05	1.51			
A8G0977	2.05	0.64	YTX2	Transposon TX1 uncharacterized 149 kDa protein	
A4G0174	2.05	9.28	LOC107688608	A0A671RXI1_9TELE NACHT, LRR and PYD domains-containing protein 3-like	XM_017355548.3
B12G0278	2.05	1.55	ZNF518A	Zinc finger protein 518A	XM_001338990.9
B25G0471	2.05	4.49	HAPLN3	Hyaluronan and proteoglycan link protein 3	NM_213101.1
B21G0804	2.05	9.41	PRODH	Proline dehydrogenase 1, mitochondrial	XM_002665870.7
B16G0944	2.05	1.02	TMEM238	Transmembrane protein 238	
B5G1260	2.05	2.02	MVK	Mevalonate kinase	NM_001007349.1
B7G0882	2.05	2.80	CMTR2	Cap-specific mRNA (nucleoside-2'-O-)-methyltransferase 2	NM_213433.1
B25G0748	2.05	3.92	SV2B	Synaptic vesicle glycoprotein 2B	XM_009297765.3
A4G0876	2.05	9.32	USP18	Ubl carboxyl-terminal hydrolase 18	XM_005164532.5
A1G0098	2.05	0.38	ROHU_013787	A0A498L1N5_LABRO CMRF35-like molecule 8	
A19G0543	2.05	0.39	HOXA11A	Homeobox protein Hox-A11a	XM_005159523.5
A5G1092	2.05	9.70	ACACA	Acetyl-CoA carboxylase 1	XM_017356117.3
A12G0155	2.05	2.07	UNK	RING finger protein unkempt homolog	NM_200236.2
B6G0412	2.05	1.25	TEFM	Transcription elongation factor, mitochondrial	NM_001080026.1

B10G0477	2.05	3.29	THAP12	52 kDa repressor of the inhibitor of the protein kinase	NM_213081.1
B2G0986	2.04	2.13	CRYZ	Quinone oxidoreductase	NM_001099976.1
B15G0403	2.04	11.04	PICALM	Phosphatidylinositol-binding clathrin assembly protein	XM_009291638.4
A22G0522	2.04	1.31	LOC113064611	A0A6P6M3K7_CARAU uncharacterized protein LOC113064611	
B14G0064	2.04	3.57	ENOX2	Ecto-NOX disulfide-thiol exchanger 2	XM_068213334.1
B6G0107	2.04	7.54	TRAK2	Trafficking kinesin-binding protein 2	XM_005172089.5
B6G0455	2.04	6.50	BRDT	Bromodomain testis-specific protein	XM_068221653.1
B2G0716	2.04	1.53	TMEM68	Transmembrane protein 68	NM_200492.1
A15G0688	2.04	3.91	RAB4B	Ras-related protein Rab-4B	NM_001004002.1
B25G0492	2.04	2.84	CAV2	Caveolin-2	NM_001002150.1
A16G0724	2.04	3.23	LPCAT3	Lysophospholipid acyltransferase 5	NM_001316738.1
A5G1258	2.04	0.69	LOC113097471	A0A6P6PBP9_CARAU uncharacterized protein LOC113076207 isoform X2	
A21G0826	2.04	3.03	CSF1R	Macrophage colony-stimulating factor 1 receptor	XM_009295703.4
B7G0609	2.04	1.29	NOD2	Nucleotide-binding oligomerization domain-containing protein 2	
A19G0151	2.04	3.38	THAP7	THAP domain-containing protein 7	NM_001103198.1
A15G0684	2.04	3.65	TMEM160	Transmembrane protein 160	NM_001128700.1
A8G0891	2.04	1.87	FAM110A	Protein FAM110A	XR_011016699.1
A10G0674	2.04	15.72	YWHAG	14-3-3 protein gamma	NM_213164.2
A14G0417	2.04	3.97	CCNA2	Cyclin-A2	NM_152949.1
B11G0421	2.04	4.83	IPO9	Importin-9	XM_689398.9
A3G0507	2.04	2.32	SMCR8A	Guanine nucleotide exchange protein smcr8a	NM_001353855.1
A4G0967	2.04	40.24	CMAH	Cytidine monophosphate-N-acetylneuraminic acid hydroxylase	NM_001002192.1
A2G0962	2.04	0.76	NLRP1	NACHT, LRR and PYD domains-containing protein 1 homolog	XM_068214578.1
B20G0091	2.04	4.24	NDUFAF7	Protein arginine methyltransferase NDUFAF7, mitochondrial	NM_001076763.1
A11G0619	2.04	3.55	EMP3	Epithelial membrane protein 3	
A6G0651	2.04	5.52	C1GALT1B	Glycoprotein-N-acetylgalactosamine 3-beta-galactosyltransferase 1-B	NM_200051.1
B5G0407	2.04	0.33	IFNLR1	Interferon lambda receptor 1	NM_001040353.1
A8G0194	2.04	6.67	BRPF3	Bromodomain and PHD finger-containing protein 3	XM_005166736.5
A2G1159	2.04	3.80	PGPEP1	Pyroglutamyl-peptidase 1	
A21G0657	2.04	2.08	ARHGAP24	Rho GTPase-activating protein 24	XM_005161401.5
A17G0552	2.04	7.74	SLC16A9	Monocarboxylate transporter 9	NM_200410.1
B8G0118	2.04	4.28	GFPT1	Glutamine--fructose-6-phosphate aminotransferase [isomerizing] 1	NM_001034981.1
B2G0891	2.04	6.63	RXRGA	Retinoic acid receptor RXR-gamma-A	NM_131217.4
A8G0469	2.04	2.60	DCLRE1B	5' exonuclease Apollo	NM_001077283.1

B4G0705	2.04	1.07	LOC107675477	A0A671KBP6_9TELE Gastrula zinc finger protein XlCGF57.1-like	XM_017354466.3
A1G0381	2.04	8.57	PNPLA6	Patatin-like phospholipase domain-containing protein 6	XM_005160052.5
A20G0802	2.04	3.17	GNPAT	Dihydroxyacetone phosphate acyltransferase	XM_002665648.7
B22G1154	2.04	2.20	A0A672PZA3	A0A672PZA3_SINGR Uncharacterized protein	XM_068220164.1
A14G0276	2.04	8.35	LONRF1	LON peptidase N-terminal domain and RING finger protein 1	XM_021481225.2
A23G0381	2.04	11.70	UBR4	E3 ubiquitin-protein ligase UBR4	XM_021473658.2
B7G0713	2.04	4.19	GOLM2	Protein GOLM2	XM_005168941.5
B21G0192	2.04	0.25	BRN3	POU domain protein 3	NM_001080058.1
A16G0024	2.04	10.22	NCOA7	Nuclear receptor coactivator 7	XM_005169772.5
B3G1581	2.04	0.73	A0A673M8N8	A0A673M8N8_9TELE Uncharacterized protein	XM_017355566.3
B1G0177	2.04	2.81	GAA	Lysosomal alpha-glucosidase	XM_005170879.5
A16G1020	2.04	12.94	IBTK	Inhibitor of Bruton tyrosine kinase	XM_005157859.5
B6G0210	2.04	4.09	FZD7-A	Frizzled-7-A	NM_170763.2
A16G0580	2.04	2.06	ADNP2	Activity-dependent neuroprotector homeobox protein 2	NM_001098265.1
A7G0629	2.04	1.79	SALL1	Sal-like protein 1	NM_001146014.1
B16G0087	2.04	0.47	SERPINB1	Leukocyte elastase inhibitor	NM_001109730.1
B12G0737	2.04	1.44	MRPL58	Peptidyl-tRNA hydrolase ICT1, mitochondrial	
B24G0485	2.04	0.79	CRY-DASH	Cryptochrome DASH	NM_205686.1
A5G0185	2.04	1.52	PES	Pescadillo	NM_131030.3
B3G0914	2.04	4.94	FZD2	Frizzled-2	NM_131140.1
A8G0879	2.04	4.36	RMI1	RecQ-mediated genome instability protein 1	NM_200180.1
A6G0372	2.04	2.97	TEN1	CST complex subunit TEN1	NM_001326365.1
A5G0468	2.03	3.18	H4	Histone H4	NM_001076590.1
A15G0589	2.03	4.84	CUL5	Cullin-5	NM_001328059.1
A11G0462	2.03	3.61	CDKL5	Cyclin-dependent kinase-like 5	NM_001045240.1
B4G0196	2.03	8.39	SMIM30	Small integral membrane protein 30	XM_068220734.1
B2G0303	2.03	3.65	MYO10	Unconventional myosin-X	XM_005171401.5
B7G1025	2.03	1.70	TRIM39	E3 ubiquitin-protein ligase TRIM39	NM_001127479.1
A9G0368	2.03	2.85	SRPX	Sushi repeat-containing protein SRPX	NM_001014326.2
B17G0618	2.03	7.88	SNX9	Sorting nexin-9	XM_005158722.4
B4G0526	2.03	7.60	NAPEPLD	N-acyl-phosphatidylethanolamine-hydrolyzing phospholipase D	NM_001080613.2
A14G0404	2.03	4.04	ROHU_005577	A0A498NBY8_LABRO Calphotin-like isoform X1	XM_005169620.4
B8G0380	2.03	2.29	ZBTB34	Zinc finger and BTB domain-containing protein 34	NM_001122711.1
A5G1327	2.03	4.53	ARHGAP31	Rho GTPase-activating protein 31	XM_005165631.5
B14G0738	2.03	5.11	RNF103	E3 ubiquitin-protein ligase RNF103	NM_001004600.1
B3G1263	2.03	3.09	FOXRED2	FAD-dependent oxidoreductase domain-containing protein 2	XM_005163913.5
B16G0881	2.03	3.31	RAPGEF5	Rap guanine nucleotide exchange factor 5	NM_001098779.1

A14G0238	2.03	7.47	KDRL	Vascular endothelial growth factor receptor kdr-like	NM_131472.1
B3G0032	2.03	1.46			
B2G0348	2.03	0.96	LOC113046345	A0A6P6JTK2_CARAU C-C motif chemokine 2-like	
B24G0541	2.03	1.33	RTTN	Rotatin	XM_068217175.1
B22G0793	2.03	0.62	GNAQ	Guanine nucleotide-binding protein G(q) subunit alpha	
B11G0342	2.03	7.90	SYNPR	Synaptoporin	XM_021479837.2
A18G0286	2.03	5.46	RTASE	Probable RNA-directed DNA polymerase from transposon BS	
B3G1702	2.03	1.85	G5714_002689	A0A7J6D7H4_9TELE Uncharacterized protein	XM_068218685.1
B6G0090	2.03	1.00	COL28A1	Collagen alpha-1(XXVIII) chain	XM_021476979.2
A3G0259	2.03	1.30	RAB3D	Ras-related protein Rab-3D	
B2G0054	2.03	5.49	SLC25A42	Mitochondrial coenzyme A transporter SLC25A42	NM_001045453.2
B21G0608	2.03	4.41	MED15	Mediator of RNA polymerase II transcription subunit 15	NM_213073.2
A9G0337	2.03	6.53	PIKFYVE	1-phosphatidylinositol 3-phosphate 5-kinase	XM_005167670.5
B18G0698	2.03	1.97	ANLN	Anillin	XM_021467749.2
A23G0247	2.03	4.55	KIF3B	Kinesin-like protein KIF3B	XM_005174101.5
A11G0480	2.03	0.89	LOC113111049	Q-type calcium channel subunit alpha-1A-like isoform X9	NM_001024949.1
A22G0477	2.03	2.93	TLE4	Transducin-like enhancer protein 4	XM_021473487.2
A1G1033	2.03	2.13	KLHL2	Kelch-like protein 2	XM_021473892.2
B3G0764	2.03	18.43	IGHV3-30-3	Immunoglobulin heavy variable 3-30-3	
B16G0956	2.03	4.61	TAPBPL	Tapasin-related protein	XM_001919950.6
A21G0227	2.03	8.65			
A15G0278	2.03	6.34	NXPE3	NXPE family member 3	XM_068213769.1
B24G0696	2.03	8.27	IDI1	Isopentenyl-diphosphate Delta-isomerase 1	NM_001025475.2
A10G0444	2.03	3.50	TMEM218	Transmembrane protein 218	XM_021479119.2
B11G0242	2.03	6.54	SLC1A7	Excitatory amino acid transporter 5	NM_001099418.1
A1G1009	2.02	2.34	SMC2	Structural maintenance of chromosomes protein 2	NM_199542.2
B16G0011	2.02	1.38	ZBTB7B	Zinc finger and BTB domain-containing protein 7B	NM_200629.2
B2G0267	2.02	0.05	ROHU_028282	A0A498M3L8_LABRO Phosphatidylinositol-4,5-bisphosphate 4-phosphatase	
B23G0736	2.02	4.14	GPR182	G-protein coupled receptor 182	NM_001020478.1
B6G0678	2.02	4.31	SMARCC2	SNF complex subunit SMARCC2	XM_021474611.2
A5G0416	2.02	3.52	PAXX	Protein PAXX	NM_001130597.1
B20G0427	2.02	2.69	IRF4	Interferon regulatory factor 4	XM_009303157.4
B4G0200	2.02	3.45	TFEC	Transcription factor EC	XM_017355023.3
B24G0739	2.02	5.96	LANCL2	LanC-like protein 2	NM_001083003.2
A15G0744	2.02	5.61	BRWD1	Bromodomain and WD repeat-containing protein 1	XM_001920406.8
B5G0023	2.02	3.67	SURF4	Surfeit locus protein 4	NM_201127.1
A9G0284	2.02	3.53	DCAF6	DDB1- and CUL4-associated factor 6	XM_005167840.5
A5G0191	2.02	2.92	TOP3B	DNA topoisomerase 3-beta-1	XM_003198603.6
B9G0106	2.02	2.39	BAZ2B	Bromodomain adjacent to zinc finger domain protein 2B	XM_021479049.2

B4G0567	2.02	1.26	LOC113081360	A0A6P6NKW7_CARAU uncharacterized protein LOC113081360	
A5G0701	2.02	4.39	CDC42EP2	Cdc42 effector protein 2	NM_001013531.1
B12G0168	2.02	3.69	LOC113111677	A0A6P6QG72_CARAU uncharacterized protein LOC113111677	NM_001278598.1
B10G0094	2.02	2.13	CLDN23	Claudin-23	NM_001386714.1
A12G0411	2.02	2.81	NEURL4	Neuralized-like protein 4	XM_005156224.5
B4G0682	2.02	0.63	A0A673G440	A0A673G440_9TELE Uncharacterized protein	NM_001366639.1
B9G0421	2.02	4.39	ZMYM2	Zinc finger MYM-type protein 2	XM_068223591.1
B9G0376	2.02	30.92	ZIC2	Zinc finger protein ZIC 2	NM_131558.1
A23G0652	2.02	5.95	CHST11	Carbohydrate sulfotransferase 11	XM_688938.10
B19G0924	2.02	1.71	CCNE2	S-specific cyclin-E2	
B23G0522	2.02	2.32	ADCY6	Adenylate cyclase type 6	XM_021473700.2
B3G1631	2.02	1.65	G5714_002766	A0A7J6D7N0_9TELE Uncharacterized protein	XM_068220526.1
A1G0469	2.02	7.72	SSRP1	FACT complex subunit SSRP1	XM_068221595.1
B25G0015	2.02	6.24	PAMR1	Inactive serine protease PAMR1	XM_068217597.1
A13G0253	2.02	4.74	TASP1	Threonine aspartase 1	XM_005156608.5
A15G0332	2.02	3.92	MSI2	RNA-binding protein Musashi homolog 2	XM_009291629.4
B8G0566	2.02	3.32	ADORA1	Adenosine receptor A1	NM_001128584.1
A5G0003	2.02	2.13	MOB3B	MOB kinase activator 3B	
B5G0178	2.02	4.32	RNFT2	RING finger and transmembrane domain-containing protein 2	XM_005165042.5
A17G0898	2.02	1.55	SLC25A29	Mitochondrial basic amino acids transporter	XM_002665086.7
B9G0052	2.02	0.49	RTASE	Probable RNA-directed DNA polymerase from transposon BS	XM_068220297.1
A22G0660	2.02	2.10	LOC113065145	A0A6P6M7D5_CARAU Fc receptor-like protein 5 isoform X3	
B3G1383	2.01	12.18	NLRC3	Protein NLRC3	XM_068219453.1
B1G0455	2.01	1.74	MAP4K4	Mitogen-activated protein kinase kinase kinase kinase 4	XM_021478794.2
B11G0068	2.01	3.33	CNPY2	Protein canopy homolog 2	XM_068217874.1
A1G0965	2.01	8.56	PCM1	Pericentriolar material 1 protein	XM_068223107.1
B22G1182	2.01	1.43	MS4A4A	Membrane-spanning 4-domains subfamily A member 4A	XM_003199992.6
B2G0743	2.01	1.51	A0A673FR66	A0A673FR66_9TELE Si:ch211-106k21.5	XM_001337273.8
B12G0176	2.01	4.25	TBCC	Tubulin-specific chaperone C	NM_001256248.1
B4G0424	2.01	0.00	STMP1	Short transmembrane mitochondrial protein 1	NM_001302634.1
A14G0308	2.01	4.48	ELOVL6	Elongation of very long chain fatty acids protein 6	NM_199532.1
B5G0531	2.01	0.99	ZBTB26	Zinc finger and BTB domain-containing protein 26	NM_001122625.1
A20G0717	2.01	13.42	FMO5	Flavin-containing monooxygenase 5	NM_001044834.2
B7G0621	2.01	4.67	FTO	Alpha-ketoglutarate-dependent dioxygenase FTO	XM_009303351.4
B13G0265	2.01	3.86	PANK2	Pantothenate kinase 2, mitochondrial	XM_005156965.5
A10G0414	2.01	11.55	BCL7BA	lymphoma 7 protein family member B-A	NM_213165.1
B19G0553	2.01	6.43	CASD1	N-acetylneuraminate 9-O-acetyltransferase	NM_001278180.1
A4G0009	2.01	0.77	CASPA	Caspase a	

B1G1142	2.01	1.81	DPX16_23266	A0A3N0Z999_ANAGA Zinc finger protein 2	NM_001122845.1
B4G0459	2.01	0.65	SPIC	Transcription factor Spi-C	NM_001004621.2
B16G0738	2.01	7.23	CLEC17A	C-type lectin domain family 17, member A	NM_001045039.2
B4G0836	2.01	2.01	G5714_004447	A0A7J6D5J8_9TELE Uncharacterized protein	XM_021470798.2
A23G0892	2.01	3.64	CNTROB	Centrobin	XM_021470055.1
B8G0702	2.01	3.39	MPND	MPN domain-containing protein	NM_001076565.1
A5G0072	2.01	1.92	DHRS11	reductase SDR family member 11	NM_001100048.1
B4G0248	2.01	2.33	CD82	CD82 antigen	XM_068218168.1
B5G0548	2.01	5.87	NOTCH1	Neurogenic locus notch homolog protein 1	XM_005168649.5
A8G0626	2.01	3.64	ZNF131	Zinc finger protein 131	NM_200505.2
B20G0441	2.01	6.77	SEL1L	Protein sel-1 homolog 1	NM_001045164.1
B3G1716	2.01	2.74	RNASEL3	Ribonuclease-like 3	
B9G0914	2.01	1.42	GHR	Growth hormone receptor	
B10G0042	2.01	7.38	TLN1	Talin-1	XM_005155500.5
B6G0490	2.01	1.92	CEP63	Centrosomal protein of 63 kDa	XM_068221683.1
A2G0238	2.01	11.14	GPT2L	Alanine aminotransferase 2-like	XM_017357999.3
B21G0618	2.00	3.87	RTASE	Probable RNA-directed DNA polymerase from transposon BS	XM_068214189.1
B16G0313	2.00	1.66	TMEM42	Transmembrane protein 42	NM_001386405.1
B7G0354	2.00	6.95	ACOX3	Peroxisomal acyl-coenzyme A oxidase 3	NM_213147.2
B19G0180	2.00	3.87	EVA1B	Protein eva-1 homolog B	NM_001114461.1
A1G1013	2.00	5.05	TMEM192	Transmembrane protein 192	
B7G0510	2.00	4.43	CHPF2	Chondroitin sulfate glucuronyltransferase	XM_001337169.7
B16G0939	2.00	5.13	CASP2	Caspase-2	NM_001042695.1
A10G0740	2.00	7.68	RC3H2	Roquin-2	XM_068223907.1
B5G0775	2.00	3.04	RNF214	RING finger protein 214	XM_021476555.2
A1G1031	2.00	42.72	CPE	Carboxypeptidase E	NM_214810.2
A23G0547	2.00	4.09	CASZ1	Zinc finger protein castor homolog 1	XM_021469994.2
A10G0763	2.00	8.04	LIN54	Protein lin-54 homolog	XM_009305164.4
B1G0278	2.00	3.43	MTMR7	Myotubularin-related protein 7	XM_005168352.5
B11G0025	2.00	75.84	SLC6A1	Sodium- and chloride-dependent GABA transporter 1	
A4G0650	2.00	3.48	TMPO	Lamina-associated polypeptide 2, isoform beta	NM_001328563.1
B5G0105	2.00	3.62	MSI1	RNA-binding protein Musashi homolog 1	XM_003198578.6
A15G0853	2.00	2.50	PPM1L	Protein phosphatase 1L	NM_001320183.1
A21G0495	2.00	0.24	GNG8	G(O) subunit gamma-8	NM_001177743.2
B25G0107	2.00	1.90	PGBD4	PiggyBac transposable element-derived protein 4	
B9G0566	2.00	3.55	DNAJC3	DnaJ homolog subfamily C member 3	NM_199610.2
B17G0311	2.00	3.18	DICER1	Endoribonuclease Dicer	NM_001161453.2
A21G0806	2.00	16.35	MPP1	55 kDa erythrocyte membrane protein	NM_214692.2
B16G0785	2.00	7.03	PEX5	Peroxisomal targeting signal 1 receptor	NM_201156.1
A22G0552	2.00	1.34	VASN	Vasorin	NM_001326685.1
A6G0596	2.00	9.35	UAP1	UDP-N-acetylhexosamine pyrophosphorylase	NM_001365262.1
B13G0371	2.00	2.16			NM_001017994.2
A21G0494	2.00	2.95	RFC2	Replication factor C subunit 2	NM_001013326.2
A7G0759	2.00	10.96	VPS13C	Intermembrane lipid transfer protein VPS13C	XM_021471934.2

A12G0099	1.99	0.78	DQX1	ATP-dependent RNA helicase DQX1	NM_001202472.3
A22G0630	1.99	4.52	SEMA3D	Semaphorin-3D	XM_068216482.1
B5G1016	1.99	0.62	B3GALT1	Beta-1,3-galactosyltransferase 1	
A12G0688	1.99	0.00			
B19G0743	1.99	4.60	SACM1LA	Phosphatidylinositol-3-phosphatase SAC1-A	NM_001044878.2
A11G0604	1.99	1.19	CDC42	Cell division control protein 42 homolog	NM_001004590.1
A1G0721	1.99	2.44	ERCC5	DNA excision repair protein ERCC-5 homolog	XM_003197605.6
B5G0132	1.99	1.53	A33	Zinc-binding protein A33	NM_001020475.1
B10G0522	1.99	5.82	FRY	Protein furry homolog	XM_068223836.1
A4G0997	1.99	4.83	SMP	Schwann cell myelin protein	XM_068219776.1
A12G0344	1.99	2.38	SLC16A5	Monocarboxylate transporter 6	XM_688794.9
B2G1203	1.99	0.97	WNT9A	Protein Wnt-9a	XM_005163661.5
A1G0850	1.99	2.31	ERN1	endoribonuclease IRE1	XM_021471109.2
A2G0996	1.99	0.90	EEF1AKMT4	EEF1A lysine methyltransferase 4	XM_009298793.4
B16G0339	1.99	4.69	PI4KB	Phosphatidylinositol 4-kinase beta	XM_005158219.5
B22G0775	1.99	5.71	CRLF1	Cytokine receptor-like factor 1	XM_068216333.1
B1G0388	1.99	2.15	N4BP2	NEDD4-binding protein 2	NM_001079967.1
B3G0027	1.99	1.63	G5714_004077	A0A7J6DB72_9TELE Uncharacterized protein	XM_068214695.1
B16G0776	1.99	4.03	CXCR3	C-X-C chemokine receptor type 3	NM_001144813.1
A18G0438	1.99	5.87	TTC23	Tetratricopeptide repeat protein 23	XM_021474417.2
B9G0658	1.99	3.68	ALS2	Alsin	XM_679972.9
A6G0966	1.99	7.71	DNTTIP1	Deoxynucleotidyltransferase terminal-interacting protein 1	XM_002662552.7
B20G0923	1.99	2.35	WDR20	WD repeat-containing protein 20	XM_003200785.6
B14G0084	1.99	1.62	BNIP1	Vesicle transport protein SEC20	XM_684156.9
A20G0842	1.99	6.67	A0A671PU89	A0A671PU89_9TELE Palmitoyl-protein thioesterase 2	
B1G0162	1.99	8.74	PRKCBB	Protein kinase C beta type	XM_005170844.5
B2G0003	1.99	7.21	FEM1A	Protein fem-1 homolog A	NM_199837.1
B3G1337	1.99	7.74	HELZ	Probable helicase with zinc finger domain	NM_213266.1
A22G0698	1.99	55.47	LOC106584303	Kininogen	
B2G0189	1.99	2.44	SLAMF9	SLAM family member 9	XM_068213188.1
A5G0157	1.99	2.20	SCARF2	Scavenger receptor class F member 2	XM_017356231.3
B1G0326	1.99	0.64	TMEM192	Transmembrane protein 192	NM_001102393.1
B15G0676	1.99	3.79	TRIM3	Tripartite motif-containing protein 3	XM_068213766.1
A19G0570	1.99	7.04	KAT2B	Histone acetyltransferase KAT2B	NM_001045034.1
B17G0057	1.99	5.37	CCDC9B	Coiled-coil domain-containing protein 9B	XM_068214495.1
B1G0653	1.99	4.68	HTT	Huntingtin	XM_009292964.4
B1G0484	1.99	8.71	IRS2	Insulin receptor substrate 2	NM_200315.1
A20G0560	1.99	2.21	BACH2	Transcription regulator protein BACH2	XM_005160548.5
B5G1325	1.99	5.20	ABL1	Tyrosine-protein kinase ABL1	XM_005172048.5
B6G0833	1.99	3.86	TARBP2	RISC-loading complex subunit tarbp2	NM_199997.1
A24G0013	1.99	1.49	TWSG1A	Twisted gastrulation protein homolog 1-A	NM_153672.2

A20G0910	1.98	3.50	IL20RA	Interleukin-20 receptor subunit alpha	
A5G0918	1.98	6.79	CPLX4	Complexin-4	
A23G0541	1.98	1.63	G5714_002794	A0A7J6D8F5_9TELE Uncharacterized protein	
A10G0463	1.98	2.36	TMEM126A	Transmembrane protein 126A	NM_001328432.1
B8G0930	1.98	6.12	GPD1	Glycerol-3-phosphate dehydrogenase [NAD(+)], cytoplasmic	NM_001017709.1
B23G0582	1.98	10.72	KIF1B	Kinesin-like protein KIF1B	XM_068216698.1
B1G0374	1.98	4.26	TAPT1	Transmembrane anterior posterior transformation protein 1 homolog	NM_001123250.1
B22G0826	1.98	2.91	P3H2	Prolyl 3-hydroxylase 2	XM_021474475.2
A22G0029	1.98	8.53	A0A671M3Y4	A0A671M3Y4_9TELE Ubiquitin-like domain-containing protein	
B15G0271	1.98	0.97	TMPRSS13	Transmembrane protease serine 13	NM_001159512.1
A7G1114	1.98	1.81	RCN1	Reticulocalbin-1	XM_686046.9
A16G0005	1.98	3.61	OSR2	Protein odd-skipped-related 2	XM_005173555.5
B16G0773	1.98	1.53	TLR13	Toll-like receptor 13	NM_001199335.1
A1G0617	1.98	1.33	BORA	Protein aurora borealis	XM_005159946.5
A7G0838	1.98	3.07	GAL3ST4	Galactose-3-O-sulfotransferase 4	NM_001326376.1
B23G0971	1.98	9.27	RTASE	Probable RNA-directed DNA polymerase from transposon BS	
A13G0550	1.98	2.57	NRG3	Pro-neuregulin-3, membrane-bound isoform	XM_005156854.5
B20G0630	1.98	0.20	ADGRF3	Adhesion G-protein coupled receptor F3	XM_688747.6
A10G0821	1.98	3.39	SNX30	Sorting nexin-30	NM_001017798.1
A3G0420	1.98	2.15	RAVER1	Ribonucleoprotein PTB-binding 1	XM_021470765.2
A4G0965	1.98	1.31	PHLDA1	Pleckstrin homology-like domain family A member 1	NM_001006011.1
A1G0136	1.98	1.06	GIMAP9	GTPase IMAP family member 9	XM_068220949.1
B24G0059	1.98	2.79			
B16G0795	1.98	6.37	SLC2A3	Solute carrier family 2, facilitated glucose transporter member 3	XM_002667123.6
A11G0701	1.98	0.79	CS025	UPF0449 protein C19orf25 homolog	NM_001003590.1
A19G0382	1.98	3.22	VPS50	Syndetin	NM_001365104.1
B21G0486	1.98	14.59	ANGPTL5	Angiopoietin-related protein 5	XM_068218968.1
B9G0411	1.98	3.33	XPO4	Exportin-4	NM_212674.2
B20G0323	1.98	3.87	PPM1A	Protein phosphatase 1A	NM_001161332.1
B9G0662	1.98	1.84	RAPH1	Ras-associated and pleckstrin homology domains-containing protein 1	NM_001366727.1
A12G0347	1.98	1.58	FAAP100	Fanconi anemia core complex-associated protein 100	XM_017358533.3
A11G0324	1.98	0.37	CNTN2	Contactin-2	
A5G0906	1.98	3.36	PTAR1	Protein prenyltransferase alpha subunit repeat-containing protein 1	NM_001130074.1
A9G0349	1.97	0.52	GPR183	G-protein coupled receptor 183	XM_005167754.5
B9G0244	1.97	4.33	OSBPL11	Oxysterol-binding protein-related protein 11	XM_683921.8
A19G0109	1.97	7.09	LRP12	Low-density lipoprotein receptor-related protein 12	XM_005159560.4
A2G0900	1.97	0.85	LOC113046345	A0A6P6JTK2_CARAU C-C motif chemokine 2-like	

B2G1079	1.97	5.58	CHST2	Carbohydrate sulfotransferase 2	NM_001079995.2
B7G1192	1.97	0.94	UGT2B	UDP-glucuronosyltransferase 2B2	NM_001114442.1
A9G0461	1.97	1.19	RPE	Ribulose-phosphate 3-epimerase	NM_001243424.1
B5G1262	1.97	3.33	UBE3B	Ubiquitin-protein ligase E3B	NM_001114682.1
A7G0391	1.97	5.31	IRS2-B	Insulin receptor substrate 2-B	XM_009303448.4
B4G0347	1.97	1.60	LOC113071149	A0A6P6MV92_CARAU uncharacterized protein LOC113071149	
B25G0546	1.97	4.26	CECR2	Chromatin remodeling regulator CECR2	XM_005174362.4
B23G0587	1.97	27.39	CLSTN1	Calsyntenin-1	XM_005162269.5
B4G0438	1.97	5.74	IGF1A	Insulin-like growth factor I, adult form	NM_131825.2
A6G0579	1.97	1.50	ORC1	Origin recognition complex subunit 1	XM_005166281.5
B21G0715	1.97	14.00	UBE2R2	Ubiquitin-conjugating enzyme E2 R2	NM_001002600.1
A15G0107	1.97	0.89	CLDN8	Claudin-8	NM_001017799.2
A16G0201	1.97	5.16	ATAD2	ATPase family AAA domain-containing protein 2	
B2G0305	1.97	3.51	DIP2C	Disco-interacting protein 2 homolog C	XM_021471279.2
A5G1119	1.97	2.91	MAST4	threonine-protein kinase 4	XM_009301828.4
B16G0700	1.97	0.00	ROHU_030849	A0A498LQA4_LABRO Uncharacterized protein	
A21G0034	1.97	1.42	WDR7	WD repeat-containing protein 7	XM_017352997.3
A3G0943	1.97	9.25	KCTD13	POZ domain-containing adapter for CUL3-mediated RhoA degradation protein 1	NM_001077151.1
B25G0376	1.97	3.25	AMN1	Protein AMN1 homolog	NM_001045454.1
A21G0045	1.97	2.79	WDR91	WD repeat-containing protein 91	XM_068215824.1
B10G0451	1.97	3.80	VPS11	Vacuolar protein sorting-associated protein 11 homolog	XM_068223812.1
A20G0695	1.97	1.16			
B10G0755	1.97	2.21	DUSP26	Dual specificity protein phosphatase 26	
A5G0563	1.97	1.02	PIP5KL1	Phosphatidylinositol 4-phosphate 5-kinase-like protein 1	XM_001923957.7
B14G0289	1.97	1.96	SH3TC2	SH3 domain and tetratricopeptide repeat-containing protein 2	XM_068213382.1
B19G0330	1.97	0.17	IGKV3D-7	Immunoglobulin kappa variable 3D-7	
A22G0645	1.97	3.54	POL	Retrovirus-related Pol polyprotein from transposon 17.6	
B18G0393	1.97	5.44	LOC107725742	A0A673LX68_9TELE Uncharacterized protein	
B9G0094	1.97	5.40	IL1R1	Interleukin-1 receptor type 1	
A7G0320	1.97	1.69	CHCHD7	Coiled-coil-helix-coiled-coil-helix domain-containing protein 7	
A6G0473	1.97	0.21	SOX14	Transcription factor Sox-14	NM_001037680.2
A17G0385	1.97	0.55	E2F2	Transcription factor E2F2	XM_068214603.1
A7G0149	1.97	3.53	ERN1	endoribonuclease IRE1	XM_021478061.2
B12G0179	1.97	3.81	TEPSIN	AP-4 complex accessory subunit Tepsin	XM_001919649.6
A18G0785	1.97	7.24	TARS3	Threonine--tRNA ligase 2, cytoplasmic	XR_011007191.1

B16G0073	1.97	6.64	CEP72	Centrosomal protein of 72 kDa	NM_001319132.1
A9G0304	1.97	4.91	SLC5A3	myo-inositol cotransporter	XM_005167791.5
A24G0121	1.96	3.34	DECR2	Peroxisomal 2,4-dienoyl-CoA reductase [(3E)-enoyl-CoA-producing]	XM_005162880.5
A15G0356	1.96	1.89	NAIF1	Nuclear apoptosis-inducing factor 1	NM_001013490.2
A3G0461	1.96	7.56	LITAF	Lipopolysaccharide-induced tumor necrosis factor-alpha factor homolog	
A10G0324	1.96	17.67	ALOX5AP	Arachidonate 5-lipoxygenase-activating protein	NM_200061.2
A8G0699	1.96	7.31	MLEC	Malectin	NM_001113618.1
B9G0454	1.96	16.52	ETV5	ETS translocation variant 5	NM_001126461.2
A17G0567	1.96	4.42	P4HA1	Prolyl 4-hydroxylase subunit alpha-1	NM_214691.1
B24G0061	1.96	1.53	IGKV4-1	Immunoglobulin kappa variable 4-1	NM_001045408.1
B15G0419	1.96	4.02	A0A673H6T8	A0A673H6T8_9TELE Interleukin	
A16G0273	1.96	1.10	CENPW	Centromere protein W	
A20G0727	1.96	7.20	RALGPS1	Ras-specific guanine nucleotide-releasing factor RalGPS1	NM_200474.2
B1G0008	1.96	4.13	TMEM39A	Transmembrane protein 39A	NM_199632.1
A1G0507	1.96	3.18	VWA7	von Willebrand factor A domain-containing protein 7	XM_021476833.2
A5G0065	1.96	6.34	SRRM3	arginine repetitive matrix protein 3	XM_068221388.1
B2G0281	1.96	0.45	RANBP2	E3 SUMO-protein ligase RanBP2 (Fragment)	NM_001430853.1
A10G0006	1.96	12.12	DYRK1A	Dual specificity tyrosine-phosphorylation-regulated kinase 1A	NM_001347739.1
B25G0504	1.96	2.06	ZGC:103499	Uncharacterized protein C12orf29 homolog	NM_001008606.1
A11G0453	1.96	4.42	PIR	A0A673MDE0_9TELE L-ornithine N(5)-monooxygenase	XR_011009878.1
A16G0219	1.96	8.27	SLC4A7	Sodium bicarbonate cotransporter 3	XM_068214223.1
A1G0977	1.96	4.28	ANKRD37	Ankyrin repeat domain-containing protein 37	NM_001145614.1
A1G0849	1.96	3.43	PLK1	threonine-protein kinase PLK1	NM_001003890.3
A13G0189	1.96	1.79	SYNE2	Nesprin-2	NM_001037101.1
B25G0049	1.96	5.61	NFATC3	Nuclear factor of activated T-cells, cytoplasmic 3	XM_001921732.6
A16G0735	1.96	3.08	ZNF628	Zinc finger protein 628	XM_021466864.2
A25G0007	1.96	2.44	FEM1B	Protein fem-1 homolog B	XM_690410.9
A23G0692	1.96	1.87	ZNF740	Zinc finger protein 740	NM_001076631.2
B19G0029	1.96	4.52	RTASE	Probable RNA-directed DNA polymerase from transposon BS	XM_068214189.1
B22G0248	1.96	6.44	RTASE	Probable RNA-directed DNA polymerase from transposon BS	XR_011015637.1
B7G0777	1.96	0.61	G5714_008218	A0A7J6CV26_9TELE TPP1 domain-containing protein	
B23G0759	1.96	3.16	TOR1A	Torsin-1A	NM_001386481.1
A16G0063	1.96	6.59	MAN1C1	Mannosyl-oligosaccharide 1,2-alpha-mannosidase IC	XM_017351571.3
A5G0290	1.96	2.78	LOC107741221	A0A673GVY5_9TELE Coronin	XM_682863.7
A2G0027	1.96	1.87	LOC113046181	A0A6P6JST0_CARAU gastrula zinc finger protein XlCGF8.2DB-like	XM_005171082.5

B17G0203	1.96	11.41	ARID4A	AT-rich interactive domain-containing protein 4A	XM_005158629.5
B25G0564	1.96	0.54	TMPRSS6	Transmembrane protease serine 6	XM_021470656.2
A5G0063	1.95	12.79	YWHAG-B	14-3-3 protein gamma-B	NM_213022.2
B4G0562	1.95	4.67	CPM	Carboxypeptidase M	
B9G0379	1.95	0.93	GPR183	G-protein coupled receptor 183	XM_005167754.5
A8G0924	1.95	4.81	KANK3	KN motif and ankyrin repeat domain-containing protein 3	NM_001257149.1
B4G0779	1.95	1.18	LOC113080876	A0A6P6NHV3_CARAU gastrula zinc finger protein XlCGF26.1-like isoform X1	XM_068220705.1
B22G0687	1.95	6.80	IFI44L	Interferon-induced protein 44-like	XM_021469588.2
A23G0436	1.95	4.82	SPEN	Msx2-interacting protein	XM_003201204.6
A8G0607	1.95	2.81	GUCD1	Protein GUCD1	NM_001077162.2
A5G0812	1.95	2.71	WDR54	WD repeat-containing protein 54	NM_001003630.2
A23G0810	1.95	8.78	ITCH	E3 ubiquitin-protein ligase Itchy	NM_001302226.1
B3G0679	1.95	4.47	WIPI1	WD repeat domain phosphoinositide-interacting protein 1	NM_200391.1
A2G0905	1.95	2.85	UGGT1	UDP-glucose:glycoprotein glucosyltransferase 1	NM_001077534.1
B1G1198	1.95	5.63	ACDH-11	Acyl-CoA dehydrogenase family member 11	XR_224731.5
A13G0328	1.95	5.41	ZDHHC16A	Palmitoyltransferase ZDHHC16A	XM_068218719.1
A24G0641	1.95	2.05	INHBA	Inhibin beta A chain	NM_130916.1
B9G0673	1.95	2.86	PARD3B	Partitioning defective 3 homolog B	XM_685546.10
A19G0910	1.95	1.87	MTERF1A	Transcription termination factor 1a, mitochondrial	XM_003200502.4
B25G0375	1.95	2.43	G5714_024284	A0A7J6BJW4_9TELE Uncharacterized protein	XM_021470702.2
B3G1005	1.95	3.65	PIGQ	Phosphatidylinositol N-acetylglucosaminyltransferase subunit Q	NM_199429.1
A7G1011	1.95	1.74	TRIP6	Thyroid receptor-interacting protein 6	XM_009303106.4
A12G0443	1.95	8.74			
A12G0472	1.95	5.74	SNX29	Sorting nexin-29	XM_688793.9
A2G1010	1.95	6.74	CAMSAP2	Calmodulin-regulated spectrin-associated protein 2	XM_021471288.2
A20G0297	1.95	9.39	HIVEP2	Human immunodeficiency virus type I enhancer-binding protein 2 homolog	XM_005160735.5
A20G0723	1.95	1.43	FASLG	Tumor necrosis factor ligand superfamily member 6	
A8G0720	1.95	1.42	FAHD2	Fumarylacetoacetate hydrolase domain-containing protein 2	XM_068222909.1
A23G0359	1.95	1.13	COL20A1	Collagen alpha-1(XX) chain	XM_021473628.2
B3G0479	1.95	5.96	NOTCH3	Neurogenic locus notch homolog protein 3	NM_131549.2
B13G0284	1.95	3.60	CFD	Complement factor D	NM_001145765.1
A4G0598	1.95	14.40	SLC26A3	Chloride anion exchanger	XM_005164755.5
B4G0421	1.95	10.17	DENND5B	DENN domain-containing protein 5B	XM_005164711.5
B10G0629	1.95	0.40	IGSF5	Immunoglobulin superfamily member 5	
A3G1253	1.95	5.88	SAMD9L	Sterile alpha motif domain-containing protein 9-like	XM_021474496.2

A7G0427	1.95	4.20	TUBGCP4	Gamma-tubulin complex component 4	NM_001135130.1
B1G0310	1.95	8.65	LIMCH1	LIM and calponin homology domains-containing protein 1	XM_021475267.2
A16G0016	1.95	7.00	DENND4B	DENN domain-containing protein 4B	NM_001423202.1
B25G0876	1.94	1.46	PRICKLE1	Prickle-like protein 1	XM_005174210.5
B20G0272	1.94	0.18	DSG2	Desmoglein-2	XM_021468600.2
B18G0506	1.94	2.33	ZWILCH	Protein zwilch homolog	NM_001199735.1
A1G0101	1.94	3.15	CD22	B-cell receptor CD22	XM_068220962.1
B17G0464	1.94	3.86	KBTBD11	Kelch repeat and BTB domain-containing protein 11	XM_021473178.2
B8G0026	1.94	3.40	BRPF1	Peregrin	NM_201016.1
B1G1097	1.94	6.27	NLRC3	Protein NLRC3	XM_068222171.1
A3G0713	1.94	10.06	DUSP8	Dual specificity protein phosphatase 8	XM_681250.8
B14G0761	1.94	1.88	FUT11	Alpha-(1,3)-fucosyltransferase 11	NM_001077174.1
A11G0603	1.94	2.31	AP5B1	AP-5 complex subunit beta-1	XM_005172905.5
B22G0318	1.94	1.53	CD244	Natural killer cell receptor 2B4	XM_068216406.1
A10G0749	1.94	1.78	HARBI1	Putative nuclease HARBI1	
A20G0597	1.94	4.26	PDGFRA	Platelet-derived growth factor receptor alpha	NM_131459.3
A25G0224	1.94	1.47	CCL13	C-C motif chemokine 13	
B16G0044	1.94	1.88	PABPC1A	Polyadenylate-binding protein 1A	
B4G0758	1.94	0.83	A0A671LA65	A0A671LA65_9TELE Uncharacterized protein	XM_068219943.1
B2G0311	1.94	3.01	LOC107721745	A0A673LUT6_9TELE Zinc finger protein 2-like	XM_693558.9
B21G0703	1.94	2.72	MYORG	Myogenesis-regulating glycosidase	XM_068216011.1
B18G0377	1.94	16.40	LMO2	Rhombotin-2	NM_131111.2
B21G0884	1.94	1.90	GIMAP9	GTPase IMAP family member 9	
B19G0657	1.94	1.76	HEYL	enhancer-of-split related with YRPW motif-like protein	NM_181736.1
B9G0296	1.94	2.94	PPP2R3B	threonine-protein phosphatase 2A regulatory subunit B" subunit beta	XM_068223661.1
A15G0185	1.94	1.49	C3AR1	C3a anaphylatoxin chemotactic receptor	
B5G1235	1.94	2.89	A0A672P2K5	A0A672P2K5_SINGR Transmembrane protein 119a	XM_682863.7
B13G0846	1.94	2.59	SIPA1L2	Signal-induced proliferation-associated 1-like protein 2	XM_017358909.3
B1G0828	1.94	4.53	PAPSS1	Bifunctional 3'-phosphoadenosine 5'-phosphosulfate synthase 1	NM_001128699.2
B17G0087	1.94	0.95			
A19G0918	1.94	1.79	GOLPH3L	Golgi phosphoprotein 3-like	NM_001037699.2
A2G0945	1.94	2.46	MYO10	Unconventional myosin-X	XM_005171401.5
B1G0200	1.94	2.60	ZDHHC3	Palmitoyltransferase ZDHHC3	NM_200891.1
B2G0675	1.94	11.39	MARCHF6	E3 ubiquitin-protein ligase MARCHF6	NM_001291707.1
A24G0319	1.94	16.19	SMPX	Small muscular protein	XM_005162767.5
A1G0446	1.93	2.41	LOC107746722	A0A673N8G9_9TELE Zinc finger and SCAN domain-containing protein 5A-like	
B17G0120	1.93	1.73	SMC6	Structural maintenance of chromosomes protein 6	XM_021473054.2
A18G0020	1.93	2.93	SHF	SH2 domain-containing adapter protein F	NM_001080621.1

A5G1075	1.93	2.58	PRR14L	Protein PRR14L	XM_009301882.4
A23G0772	1.93	5.73	TSHZ2	Teashirt homolog 2	XM_021470030.2
A25G0776	1.93	2.66	RIC3	Protein RIC-3	
B10G0680	1.93	2.48	ORAI1	Calcium release-activated calcium channel protein 1	NM_205600.1
B3G0268	1.93	8.11	MMD	Monocyte to macrophage differentiation factor	NM_001423757.1
A23G0361	1.93	3.32	ADNP	Activity-dependent neuroprotector homeobox protein	NM_001347570.2
A5G0631	1.93	9.95	HEXB	Beta-hexosaminidase subunit beta	NM_001114845.2
A25G0618	1.93	1.42	MLKL	Mixed lineage kinase domain-like protein	
A22G0418	1.93	2.36	KLK7	Kallikrein-7	
B19G0174	1.93	2.00	A33	Zinc-binding protein A33	NM_001100041.2
B15G0479	1.93	3.95	UBE4A	Ubiquitin conjugation factor E4 A	XM_003200047.6
A7G0640	1.93	3.01	LPCAT2	Lysophosphatidylcholine acyltransferase 2	XM_005168979.5
B12G0815	1.93	1.51	NXPE3	NXPE family member 3	XM_001345784.9
A21G0655	1.93	5.02	COL25A1	Collagen alpha-1(XXV) chain	XM_005161399.5
B19G0428	1.93	2.39	CDCA8	Borealin	XM_005173787.5
A21G0660	1.93	15.74	GFPT2	Glutamine--fructose-6-phosphate aminotransferase [isomerizing] 2	NM_001033921.1
B8G0464	1.93	7.28	KLHL12	Kelch-like protein 12	NM_001007328.1
A7G0409	1.93	2.38	SLC38A7	Putative sodium-coupled neutral amino acid transporter 7	NM_001003648.1
A7G0943	1.93	5.91	RBM4.1	RNA-binding protein 4.1	XM_005172289.2
A2G0685	1.93	5.39	RNF220	E3 ubiquitin-protein ligase RNF220	XM_021480627.2
A23G0067	1.93	4.98	ZHX3	Zinc fingers and homeoboxes protein 3	NM_198813.1
A25G0633	1.93	3.48	IGKV4-1	Immunoglobulin kappa variable 4-1	XM_068217596.1
A12G0058	1.93	5.59	POLR3A	DNA-directed RNA polymerase III subunit RPC1	NM_001276496.1
A12G0111	1.93	1.43	G5714_012318	A0A7J6CG80_9TELE Uncharacterized protein	XR_011017469.1
A13G0338	1.93	0.81	MAPK8B	Mitogen-activated protein kinase 8B	NM_131783.2
A3G1076	1.92	2.34	PIK3R6	Phosphoinositide 3-kinase regulatory subunit 6	XM_009299422.4
B25G0194	1.92	2.37	IGKV1-13	Immunoglobulin kappa variable 1-13	
B1G0135	1.92	1.72	GRAP	GRB2-related adapter protein	NM_001030080.2
A24G0127	1.92	2.84	WDR24	GATOR complex protein WDR24	XM_005162879.5
B4G0159	1.92	5.19	MAPK11	Mitogen-activated protein kinase 11	NM_001002095.1
B16G0978	1.92	8.09	ZG16	Zymogen granule membrane protein 16	
B4G0894	1.92	2.01	LOC114428908	A0A6P7HY98_9TELE uncharacterized protein LOC114428737	
B12G0271	1.92	7.35	ATRNL1	Attractin-like protein 1	XM_692563.10
B5G1232	1.92	3.54	FICD	Protein adenylyltransferase FICD	NM_001017794.2
A23G0771	1.92	1.99	ZFP64	Zinc finger protein 64	NM_001386517.1
B20G0078	1.92	4.54	NT5DC1	5'-nucleotidase domain-containing protein 1	NM_001104943.1

A5G0336	1.92	5.17	CLOCK	Circadian locomoter output cycles protein kaput	NM_178299.1
B2G0153	1.92	1.26	XCR1	Chemokine XC receptor 1	
A12G0095	1.92	4.72	LOC113076502	A0A6P6N9P7_CARAU uncharacterized protein LOC113076502 isoform X1	
B3G0153	1.92	0.94	LOC113096977	A0A6P6PAM1_CARAU gastrula zinc finger protein XlCGF26.1-like	XM_021475458.2
B5G0952	1.92	2.20	IFT22	Intraflagellar transport protein 22 homolog	NM_001017884.1
A17G0348	1.92	5.86	TECPR2	Tectonin beta-propeller repeat-containing protein 2	NM_001045179.1
A16G0348	1.92	3.85	PHACTR4B	Phosphatase and actin regulator 4B	XM_005158332.5
A5G0552	1.92	6.96	ANKFY1	Rabankyrin-5	XM_005171905.5
B17G0219	1.92	2.72	STMN4	Stathmin-4	XM_005158637.5
B6G0167	1.92	1.93	G5714_006695	A0A7J6CXA6_9TELE Ig-like domain-containing protein	XM_068221881.1
B4G0256	1.92	18.41	MEIG1	Meiosis expressed gene 1 protein homolog	
A3G0604	1.92	8.33	SCPEP1	Retinoid-inducible serine carboxypeptidase	NM_200192.1
A21G0785	1.92	12.56	CPEB4	Cytoplasmic polyadenylation element-binding protein 4	NM_001351713.1
A9G0701	1.92	9.87	PKNOX1	Homeobox protein PKNOX1	NM_131891.3
A24G0422	1.92	2.23	SEC22C	Vesicle-trafficking protein SEC22c	XM_702251.8
B3G0341	1.92	3.32	SWSAP1	ATPase SWSAP1	NM_001328088.1
A25G0001	1.92	3.12	SLC6A13	Sodium- and chloride-dependent GABA transporter 2	XM_002667362.7
A7G0195	1.92	12.25	DHX38	Pre-mRNA-splicing factor ATP-dependent RNA helicase PRP16	NM_200876.1
B3G1424	1.92	8.82	NLRC3	Protein NLRC3	XM_068219453.1
B10G0646	1.92	6.21	GRAMD1B	Protein Aster-B	XM_021472374.2
B20G0440	1.92	8.31	SEL1L	Protein sel-1 homolog 1	NM_001045164.1
B19G0658	1.91	2.91	ZBTB8B	Zinc finger and BTB domain-containing protein 8B	NM_001328347.1
A10G0739	1.91	0.97	KRT18	Keratin, type I cytoskeletal 18	NM_001044910.1
B4G0166	1.91	3.65	TMEM63A	CSC1-like protein 1	NM_001089378.2
A13G0237	1.91	0.67	MAP3K4	Mitogen-activated protein kinase kinase kinase 4	
B11G0411	1.91	4.76	PLEKHG5	Pleckstrin homology domain-containing family G member 5	XM_005169333.5
A2G0838	1.91	0.09	SDR39U1	Epimerase family protein SDR39U1	NM_001076578.1
A3G0969	1.91	4.35	TLCD3B	Ceramide synthase	NM_001430857.1
B3G0634	1.91	2.55	ZNF281	Zinc finger protein 281	XM_001922353.8
B7G0735	1.91	2.30	ZNF710	Zinc finger protein 710	XM_021477854.2
A21G0155	1.91	3.51	PPP1R26	Protein phosphatase 1 regulatory subunit 26	XM_068215925.1
A7G1182	1.91	1.92	ASB7	Ankyrin repeat and SOCS box protein 7	XM_068222203.1
B17G0437	1.91	2.08	THUMPD2	THUMP domain-containing protein 2	XM_021473168.2
B4G0083	1.91	2.87	KRR1	KRR1 small subunit processome component homolog	NM_001045325.2
B9G0629	1.91	0.52	FEV	Protein FEV	NM_001080657.1
B25G0626	1.91	1.74	CCL13	C-C motif chemokine 13	
B8G0360	1.91	3.03	ULK1	threonine-protein kinase ULK1	NM_001130631.1
A2G0465	1.91	4.36	RALA	Ras-related protein Ral-A	NM_001007321.1
B11G0627	1.91	0.86	SSTR2	Somatostatin receptor type 2	

B9G0502	1.91	3.79	LET-70	Ubiquitin-conjugating enzyme E2 2	
B22G1018	1.91	1.42	NLRC3	NLR family CARD domain-containing protein 3	XM_021478497.2
A19G0195	1.91	2.85	GON4L	GON-4-like protein	XM_068215286.1
A19G0314	1.91	1.60	LRATD2	Protein LRATD2	NM_001044880.2
B22G0346	1.91	0.86	LOC113061572	A0A6P6LS07_CARAU Nuclear apoptosis-inducing factor 1	
A5G0270	1.91	0.79	MVK	Mevalonate kinase	NM_001007349.1
A22G0710	1.91	3.62	MARK1	threonine-protein kinase MARK1	NM_001114476.1
B8G0099	1.91	5.82	PLOD1	Procollagen-lysine,2-oxoglutarate 5-dioxygenase 1	NM_001077742.2
A1G0550	1.91	1.57	PRMT9	Protein arginine N-methyltransferase 9	NM_001130767.1
B5G0352	1.91	3.65	ANAPC2	Anaphase-promoting complex subunit 2	NM_001256746.2
A14G0167	1.91	3.58	MTMR7	Myotubularin-related protein 7	NM_001040316.2
B10G0427	1.91	5.83	MED13	Mediator of RNA polymerase II transcription subunit 13	NM_001281468.1
B8G0282	1.91	6.21	IQSEC2	IQ motif and SEC7 domain-containing protein 2	XM_005167237.5
B4G0507	1.91	7.77	ATP6V1F	V-type proton ATPase subunit F	NM_001002526.2
B16G0197	1.91	7.51	CD22	B-cell receptor CD22	NM_001083823.2
A5G0685	1.91	0.10	TRIM16	Tripartite motif-containing protein 16	XM_005165370.5
B1G1132	1.91	3.08	LOC113099796	A0A6P6PHG1_CARAU zinc finger protein 271-like	XM_017357165.3
A7G1019	1.91	0.92	OVOL2	Transcription factor Ovo-like 2	NM_001114744.2
A18G0525	1.91	2.75	WEE1-B	Wee1-like protein kinase 1-B	NM_001309435.1
A7G1273	1.91	0.38	LOC113096654	A0A6P6P9F8_CARAU uncharacterized protein LOC113096654	
A6G0073	1.91	5.31	SZT2	KICSTOR complex protein SZT2	XM_068221837.1
B20G0428	1.91	6.94	EXOC2	Exocyst complex component 2	NM_200604.2
B8G0615	1.91	1.56	PORCN	Protein-serine O-palmitoleoyltransferase porcupine	NM_199849.1
A11G0098	1.91	3.40	APPL1	DCC-interacting protein 13-alpha	NM_001168317.1
B6G0247	1.91	4.13	PCNT	Pericentrin	XM_002662304.7
A16G0454	1.91	2.50	SEMA6D	Semaphorin-6D	NM_001199365.1
A9G0774	1.91	7.50	PRKRA	Interferon-inducible double-stranded RNA-dependent protein kinase activator A homolog	XM_005172508.5
B20G0381	1.91	3.35	CASP8AP2	CASP8-associated protein 2	NM_001014334.2
B19G0637	1.91	4.96	FAM91A1	Protein FAM91A1	NM_200441.1
B19G0425	1.91	2.01	PRDM1	PR domain zinc finger protein 1	
A10G0659	1.90	1.70	PIGO	GPI ethanolamine phosphate transferase 3	XM_687353.9
A19G0132	1.90	2.92	CPSF1	Cleavage and polyadenylation specificity factor subunit 1	NM_001114681.2
B18G0583	1.90	11.86	BHLHB3	Class E basic helix-loop-helix protein 41	NM_001039107.1
B3G0096	1.90	0.00	CTXA	Cytolytic toxin-alpha	XM_068218440.1
B25G0763	1.90	16.36	CYP19A1	Brain aromatase	NM_131642.2
A25G0129	1.90	3.67	PTPN9	Tyrosine-protein phosphatase non-receptor type 9	XM_005162973.5
A20G0217	1.90	4.65	IGF2R	Cation-independent mannose-6-phosphate receptor	NM_001039627.2

B17G0278	1.90	4.58	SYNE3	Nesprin-3	NM_001083014.2
A16G0600	1.90	2.44	MYPOP	Myb-related transcription factor, partner of profilin	XM_003200185.6
B4G0828	1.90	2.42	LOC107563856	A0A672SLD2_SINGR Gastrula zinc finger protein XlCGF57.1-like	XM_003201487.6
A18G0529	1.90	5.86	RNF141	RING finger protein 141	NM_001007290.2
B16G0913	1.90	4.92	GSDME	Gasdermin-E	NM_001001947.1
A7G0671	1.90	2.54	TIPIN	TIMELESS-interacting protein	XM_005168874.5
B8G0578	1.90	1.28	FANCE	Fanconi anemia group E protein	NM_001040634.1
A7G0099	1.90	6.81	RTASE	Probable RNA-directed DNA polymerase from transposon BS	XM_068220487.1
A19G0679	1.90	5.08	SETDB1A	Histone-lysine N-methyltransferase SETDB1-A	NM_001044767.2
A1G1015	1.90	2.36	MARCHF1	E3 ubiquitin-protein ligase MARCHF1	XM_005168374.5
B19G0650	1.90	5.25	RIDA	2-iminopropanoate deaminase	NM_001012315.2
B14G0801	1.90	7.58	ZNF711	Zinc finger protein 711	NM_001105523.2
A15G0110	1.90	4.58	SCAF4	SR-related and CTD-associated factor 4	NM_001386228.1
B25G0294	1.90	1.75	CEP152	Centrosomal protein of 152 kDa	XM_068217560.1
A7G1270	1.90	0.59	LOC113096654	A0A6P6P9F8_CARAU uncharacterized protein LOC113096654	
B25G0343	1.90	2.89	MAN2C1	Alpha-mannosidase 2C1	XM_692992.8
A4G0419	1.90	0.88	POL	RNA-directed DNA polymerase from mobile element jockey	XM_068218007.1
A4G0031	1.90	2.15	G5714_004461	A0A7J6D4R8_9TELE Uncharacterized protein	XM_009298241.4
A1G0637	1.90	2.46	LIG4	DNA ligase 4	NM_001103123.1
A3G0434	1.90	15.96	UNK	RING finger protein unkempt homolog	XM_005164232.5
B7G0814	1.90	2.15	G5714_008251	A0A7J6CVC7_9TELE Nuclear apoptosis-inducing factor 1	NM_001386565.1
A2G0994	1.90	3.94	NCAPD2	Condensin complex subunit 1	NM_001162502.2
A23G0878	1.90	0.09	ST8SIA5	Alpha-2,8-sialyltransferase 8E	XM_021473740.2
A7G0589	1.90	1.90	HARBI1	Putative nuclease HARBI1	NM_001003734.1
A5G0569	1.90	1.51	PKN2	threonine-protein kinase N2	XM_003198639.6
A15G0121	1.90	2.48	EPHA4	Ephrin type-A receptor 4	XM_682606.7
B15G0473	1.90	5.61	CBL	E3 ubiquitin-protein ligase CBL	XM_005157579.5
A4G0845	1.89	3.08	ERC1	CAST family member 1	XM_021475210.2
A16G0674	1.89	2.13	G5714_016173	A0A7J6C7Q9_9TELE Uncharacterized protein	
B12G0472	1.89	1.42	LITAF	Lipopolysaccharide-induced tumor necrosis factor-alpha factor homolog	
B25G0473	1.89	5.15	ABHD2B	Monoacylglycerol lipase ABHD2	XM_009297903.4
B21G0789	1.89	1.51	LOC113038934	A0A6P6IXT4_CARAU uncharacterized protein LOC113038934	XM_003200821.6
B18G0517	1.89	4.48	ARIH1	E3 ubiquitin-protein ligase arih1	NM_199758.1
A22G0545	1.89	1.66	AARS2	Alanine--tRNA ligase, mitochondrial	XM_001332352.9
A5G0878	1.89	4.89	HMGCR	3-hydroxy-3-methylglutaryl-coenzyme A reductase	NM_001079977.2
A8G0559	1.89	3.52	CTTNBP2NL	CTTNBP2 N-terminal-like protein	NM_001430982.1
A18G0603	1.89	0.51	UQCRFS1	Cytochrome b-c1 complex subunit Rieske, mitochondrial	
A17G0229	1.89	8.33	MIDEAS	Mitotic deacetylase-associated SANT domain protein	XM_009293218.4

B25G0654	1.89	3.14	KLHL25	Kelch-like protein 25	XM_009297852.4
B23G0841	1.89	4.88	SCUBE3	Signal peptide, CUB and EGF-like domain-containing protein 3	XM_021469709.2
A1G0499	1.89	2.65	RAB33B	Ras-related protein Rab-33B	XM_017356390.3
A7G1059	1.89	2.52	ZNF16L	Zinc finger protein 16-like	NM_001423803.1
B13G0530	1.89	3.82	CHAT	Choline O-acetyltransferase	NM_131621.3
A21G0552	1.89	3.57	BSCL2	Seipin	
A3G0034	1.89	2.75	BTNL2	Butyrophilin-like protein 2	XM_068218140.1
B9G0830	1.89	2.72	RBM45	RNA-binding protein 45	NM_001127402.1
A17G0710	1.89	4.17	MGA	MAX gene-associated protein	XM_005158622.5
B18G0746	1.89	2.53	COG8	Conserved oligomeric Golgi complex subunit 8	NM_001013506.1
A12G0585	1.89	10.64	SULT1D1	Sulfotransferase 1 family member D1	NM_001002599.1
B24G0190	1.89	4.87	GRN	Progranulin	NM_212738.1
A23G0374	1.89	5.06	LOC107712677	A0A673KMS6_9TELE Uncharacterized LOC107712677	NM_001079964.1
B5G0545	1.89	3.27	KCNT1	Potassium channel subfamily T member 1	XM_068221564.1
A9G0192	1.89	5.64	MSTNB	differentiation factor 8	NM_131019.5
A23G0613	1.89	3.80	LETMD1	LETM1 domain-containing protein 1	XM_017353588.3
A4G0634	1.89	6.92	NRIP2	Nuclear receptor-interacting protein 2	NM_001123515.1
A12G0691	1.89	3.19	BICC1	Protein bicaudal C homolog 1	XM_694995.8
A8G0177	1.89	6.23	OTUD5A	OTU domain-containing protein 5-A	NM_001075110.1
B17G0885	1.89	18.40	G5714_017394	A0A7J6C6E8_9TELE Uncharacterized protein	
B8G0641	1.89	144.36	ALAS2	5-aminolevulinate synthase, erythroid-specific, mitochondrial	NM_131682.2
A18G0812	1.89	2.54	HOMER2	Homer protein homolog 2	NM_001020634.1
B16G1105	1.89	6.73	VEGFAA	Vascular endothelial growth factor A-A	XR_002455712.2
A16G0501	1.89	3.41	A33	Zinc-binding protein A33	
B23G0506	1.89	1.37	ITIH6	Inter-alpha-trypsin inhibitor heavy chain H6	XM_009296935.4
B12G0697	1.89	2.92	SLC35G1	Solute carrier family 35 member G1	XM_009306432.4
B12G0264	1.89	0.28	ERGIC2	Endoplasmic reticulum-Golgi intermediate compartment protein 2	XM_003199620.6
B15G0039	1.88	1.07	TRIM16	Tripartite motif-containing protein 16	
A7G0888	1.88	3.77	CMTR2	Cap-specific mRNA (nucleoside-2'-O-)-methyltransferase 2	NM_213433.1
A5G0691	1.88	2.88	G5714_005685	A0A7J6D1Q4_9TELE Uncharacterized protein	XM_021474592.2
B16G0543	1.88	0.94	RPP38	Ribonuclease P protein subunit p38	
B7G1142	1.88	2.37	CDA	Cytidine deaminase	NM_001007389.2
B19G0627	1.88	5.02	LOC113105921	A0A6P6PQM9_CARAU uncharacterized protein LOC113105921	
A20G0248	1.88	5.90	TPD52L1	Tumor protein D53 homolog	XM_005160819.5
B19G0936	1.88	3.78	FKBP10	Peptidyl-prolyl cis-trans isomerase FKBP10	XR_658990.4
B21G0503	1.88	2.44	SKP2	S-phase kinase-associated protein 2	NM_001082837.2

A21G0095	1.88	8.89	CDK5RAP2	CDK5 regulatory subunit-associated protein 2	XM_068215797.1
B7G0407	1.88	5.85	CRTC3	CREB-regulated transcription coactivator 3	XM_005166492.5
B5G0236	1.88	5.88	FSD1L	FSD1-like protein	NM_001365569.1
A5G0835	1.88	2.50	ADAMTS3	A disintegrin and metalloproteinase with thrombospondin motifs 3	NM_001287096.1
B6G1047	1.88	4.19	USP9X	Probable ubiquitin carboxyl-terminal hydrolase FAF-X	
B19G0371	1.88	3.67	IGF2BP3	Insulin-like growth factor 2 mRNA-binding protein 3	XM_009294198.4
A4G0470	1.88	2.33	ABCD2	ATP-binding cassette sub-family D member 2	XM_003200118.6
A15G0403	1.88	3.94	WDR81	WD repeat-containing protein 81	NM_001424091.1
B17G0566	1.88	3.56	CA115	Uncharacterized protein C1orf115 homolog	
B4G0290	1.88	1.68	CCDC59	Thyroid transcription factor 1-associated protein 26 homolog	
A11G0184	1.88	1.17	AP1S2	AP-1 complex subunit sigma-2	XM_021479704.2
A16G0253	1.88	5.08	CMTM7	CKLF-like MARVEL transmembrane domain-containing protein 7	NM_001130114.1
B13G0462	1.88	11.17	RNF122	RING finger protein 122	XM_009307394.4
A20G0292	1.88	1.87	NSL1	Kinetochore-associated protein NSL1 homolog	NM_001025521.2
B3G1253	1.88	0.14	G5714_003820	A0A7J6DB12_9TELE Uncharacterized protein	
B25G0220	1.88	2.26	HRAS	GTPase HRas	NM_001017623.1
A25G0528	1.88	6.41	SCAPER	S phase cyclin A-associated protein in the endoplasmic reticulum	XM_005163154.5
B16G0444	1.88	0.84	STYK1	Tyrosine-protein kinase STYK1	NM_001305565.1
B24G0229	1.88	3.91	EGFR	Epidermal growth factor receptor	XM_695018.10
A3G0756	1.88	1.98	ITPRIPL2	Inositol 1,4,5-trisphosphate receptor-interacting protein-like 2	XM_068219060.1
B11G0652	1.88	4.57	CLDN19	Claudin-19	NM_213074.1
B4G0578	1.88	11.62	PFKFB3	fructose-2,6-bisphosphatase 3	NM_213397.1
B5G1101	1.88	2.71	RHBDD3	Rhomboid domain-containing protein 3	
B17G0438	1.88	4.40	MAP4K3	Mitogen-activated protein kinase kinase kinase kinase 3	XM_021473170.2
A12G0213	1.88	3.21	TDRKH	Tudor and KH domain-containing protein	NM_001014355.2
A13G0719	1.88	15.80	EXOC3L4	Exocyst complex component 3-like protein 4	
A25G0099	1.88	2.19	CMASB	N-acylneuraminate cytidylyltransferase B	NM_001258409.1
B4G0695	1.88	1.03	LOC113057383	A0A6P6L7R6_CARAU gastrula zinc finger protein XlCGF7.1-like isoform X2	NM_001366639.1
B7G0492	1.88	2.51	PHKB	Phosphorylase b kinase regulatory subunit beta	XM_693105.9
B5G0170	1.88	2.40	SCARF2	Scavenger receptor class F member 2	XM_017356231.3
B11G0614	1.88	11.54	CACNA2D3	delta-3	XM_068224215.1
A25G0459	1.87	1.35	AMN1	Protein AMN1 homolog	NM_001045454.1
A9G0612	1.87	2.24	FAM117B	Protein FAM117B	NM_001128362.1
B2G0547	1.87	1.31	TRAV17	T cell receptor alpha variable 17	

B6G0447	1.87	3.30	GNG12	G(O) subunit gamma-12	XM_005168746.5
B3G1111	1.87	2.98	DTX3	Probable E3 ubiquitin-protein ligase DTX3	XM_021471387.2
B15G0191	1.87	3.19	ATM	Serine-protein kinase ATM	XM_002664557.4
B12G0243	1.87	6.06	VTI1A	Vesicle transport through interaction with t-SNAREs homolog 1A	NM_001313750.1
B8G0015	1.87	6.36	PLXND1	Plexin-D1	NM_205697.2
A17G0742	1.87	4.66	CTBP2	C-terminal-binding protein 2	NM_131715.2
A9G0703	1.87	0.83	PDE9A	High affinity cGMP-specific 3',5'-cyclic phosphodiesterase 9A	XM_009304653.4
A16G0512	1.87	2.63	LOC113059858	A0A6P6LI57_CARAU uncharacterized protein LOC113059858	XM_003200197.6
A1G0415	1.87	2.42	MOGS	Mannosyl-oligosaccharide glucosidase	NM_001080190.1
B12G0406	1.87	2.27	THRA1	Thyroid hormone receptor alpha	XM_001920978.8
B22G0127	1.87	1.34	CEP19	Centrosomal protein of 19 kDa	NM_001033734.1
A7G0806	1.87	1.08	LMO1	Rhombotin-1	NM_173219.3
B21G1007	1.87	3.70	AFF1	FMR2 family member 1	
B16G0999	1.87	1.78	CCR5	C-C chemokine receptor type 5	XM_005173463.5
A15G0606	1.87	3.24	KIAA0100	Protein KIAA0100	NR_023325.1
B2G0402	1.87	0.46	G5714_002095	A0A7J6DE23_9TELE C1q domain-containing protein	XM_009298697.4
B23G0147	1.87	2.73	AP4B1	AP-4 complex subunit beta-1	NM_200338.1
A6G0298	1.87	8.97	LOC113046332	A0A6P6JV48_CARAU protein ALP1-like	
A19G0604	1.87	8.61	GRB10	Growth factor receptor-bound protein 10	NM_001004287.2
B6G0016	1.87	4.53	LYL1	Protein lyl-1	XM_068221859.1
B14G0714	1.87	5.44	WFS1	Wolframin	XM_679418.8
A8G0619	1.87	44.52	SLC20A1A	Sodium-dependent phosphate transporter 1-A	NM_213179.1
A12G0614	1.87	4.66	HARBI1	Putative nuclease HARBI1	
B11G0415	1.87	2.25	INAVA	Innate immunity activator protein	NM_001080596.2
A16G0384	1.87	2.70	FBXL4	LRR-repeat protein 4	NM_001007315.2
B24G0667	1.87	0.39	G5714_023657	A0A7J6BM62_9TELE TNFR-Cys domain-containing protein	XM_005162533.5
A7G1325	1.87	0.79	LOC113080388	A0A6P6NHA1_CARAU uncharacterized protein LOC113080388	
A13G0335	1.87	1.66	PTPN20	Tyrosine-protein phosphatase non-receptor type 20	XM_021472682.2
A20G0264	1.87	5.00			
B6G0464	1.87	1.36	G5714_006964	A0A7J6CXW5_9TELE RING-CH-type domain-containing protein	
B11G0553	1.87	2.26			
B5G1238	1.87	6.35	CORO1CA	Coronin-1C-A	NM_201295.1
B1G0002	1.87	2.26	CEP97	Centrosomal protein of 97 kDa	
A5G0528	1.87	5.68	ADAMTS15	A disintegrin and metalloproteinase with thrombospondin motifs 15	NM_001126429.1
A9G0401	1.87	6.83	PTPN4	Tyrosine-protein phosphatase non-receptor type 4	XM_068223323.1
B25G0582	1.87	3.24	EXOC3L1	Exocyst complex component 3-like protein	NM_001099422.1
A11G0119	1.87	1.63	DDA1	DET1- and DDB1-associated protein 1	NM_001201560.1
B2G0728	1.86	2.88	CDH7	Cadherin-7	XM_005163402.5

B18G0791	1.86	48.06	GPI	Glucose-6-phosphate isomerase	NM_144764.2
A16G0780	1.86	0.99	DSCC1	Sister chromatid cohesion protein DCC1	NM_001004611.1
B23G0406	1.86	7.61	EMC1	ER membrane protein complex subunit 1	XM_021473642.2
A18G0169	1.86	16.89	SCG3	Secretogranin-3	NM_200757.1
A11G0319	1.86	2.13	GOLT1A	Vesicle transport protein GOT1A	XM_021479792.2
B25G0214	1.86	5.59	PHRF1	PHD and RING finger domain-containing protein 1	XM_002666868.6
B4G0537	1.86	5.88	PPP1R12A	Protein phosphatase 1 regulatory subunit 12A	XM_017354997.3
B3G0157	1.86	1.15	LOC113078295	A0A6P6NB09_CARAU gastrula zinc finger protein XlCGF57.1-like	XM_068219920.1
A10G0675	1.86	9.23	RNF208	RING finger protein 208	XM_003199320.6
B3G1360	1.86	2.41	RECQL5	ATP-dependent DNA helicase Q5	XM_005164274.5
B24G0328	1.86	4.89	PLD1	Phospholipase D1	XM_021470348.2
B19G0905	1.86	1.45	A0A671XQM7	A0A671XQM7_SPAAU Uncharacterized protein	
B19G0834	1.86	0.93	POLR2K	DNA-directed RNA polymerases I, II, and III subunit RPABC4	NM_001199106.1
A12G0358	1.86	1.23	MMRN2	Multimerin-2	XM_068224779.1
A25G0096	1.86	4.55	CMASB	N-acylneuraminate cytidylyltransferase B	NM_001258409.1
A13G0127	1.86	0.13	BTAF1	TATA-binding protein-associated factor 172	
A5G0253	1.86	4.93	ANKLE2	Ankyrin repeat and LEM domain-containing protein 2	NM_001126395.2
B16G1144	1.86	0.77	DCDC2	Doublecortin domain-containing protein 2	
B11G0291	1.86	1.64	TBL1XR1	WD repeat-containing protein TBL1XR1	NM_001326450.1
A20G0687	1.86	4.61	SIRT5	NAD-dependent protein deacylase sirtuin-5, mitochondrial	NM_001002605.1
A7G0202	1.86	4.50	NFAT5	Nuclear factor of activated T-cells 5	XM_068222776.1
B19G0585	1.86	37.59	G5714_018566	A0A7J6BZK3_9TELE Uncharacterized protein	XM_680892.10
A6G0946	1.86	6.22	SYS1	Protein SYS1 homolog	NM_200010.2
B4G0804	1.86	1.64	G5714_004461	A0A7J6D4R8_9TELE Uncharacterized protein	XM_068220170.1
A25G0638	1.86	5.54	IGKV3D-7	Immunoglobulin kappa variable 3D-7	NM_001045408.1
A6G0889	1.86	19.49	RIPOR3	RIPOR family member 3	NM_001012499.3
B1G0336	1.86	1.35	METTL14	N6-adenosine-methyltransferase non-catalytic subunit	NM_207071.1
B14G0764	1.86	0.74	PCDH20	Protocadherin-20	NM_001351716.1
B12G0508	1.86	1.02	PRVT	Parvalbumin, thymic	
B17G0051	1.86	2.05	BUB1B	threonine-protein kinase BUB1 beta	XM_690292.9
A4G0369	1.86	1.29	ROHU_010619	A0A498M1A4_LABRO Uncharacterized protein	
B23G0183	1.86	7.13	BAZ2A	Bromodomain adjacent to zinc finger domain protein 2A	NM_001320131.1
B24G0288	1.86	7.43	VAV3	Guanine nucleotide exchange factor VAV3	XM_021470353.2
A1G0359	1.86	2.25	ATP5PF	ATP synthase-coupling factor 6, mitochondrial	XM_003197714.6

B1G1113	1.86	2.25	PIGR	Polymeric immunoglobulin receptor	XM_068220971.1
A21G0599	1.86	2.60	BRD8	Bromodomain-containing protein 8	XM_017352983.3
B8G0712	1.86	3.59	MINDY4	Probable ubiquitin carboxyl-terminal hydrolase MINDY-4	XM_002663403.7
A14G0747	1.86	0.80	PCDH20	Protocadherin-20	NM_001351716.1
B5G0360	1.86	3.18	CD180	CD180 antigen	NM_001310490.1
A5G1086	1.86	20.44	MAN2A1	Alpha-mannosidase 2	NM_001110027.2
B21G0496	1.86	5.27	CHEK1	threonine-protein kinase Chk1	XM_021468776.2
A4G0902	1.86	0.23	GALNT8	Probable polypeptide N-acetylgalactosaminyltransferase 8	XM_003198223.6
B20G0424	1.86	0.31	STX11	Syntaxin-11	NM_001105602.1
B24G0592	1.86	9.04	TMEM108	Transmembrane protein 108	XM_005162594.4
A14G0644	1.85	9.27	FBXW11A	F-box and WD repeat domain-containing 11-A	NM_201310.1
B7G0972	1.85	2.29	MEX3B	RNA-binding protein MEX3B	NM_001080993.1
A3G0611	1.85	5.37	TRAF7	E3 ubiquitin-protein ligase TRAF7	NM_001080185.1
A23G0142	1.85	2.07	PHLDA3	Pleckstrin homology-like domain family A member 3	NM_001002455.1
B22G0799	1.85	3.13	UBXN6	UBX domain-containing protein 6	NM_001044780.1
B23G0304	1.85	26.97	G5714_022808	A0A7J6BP87_9TELE Uncharacterized protein	NR_170145.1
B22G0264	1.85	0.77	LOC113040705	A0A6P6J3P3_CARAU SLAM family member 9-like	XM_068216406.1
B19G0091	1.85	2.83	ZDHHC3	Palmitoyltransferase ZDHHC3	NM_001077757.1
A16G0915	1.85	1.33	GASK1A	Golgi-associated kinase 1A	XM_003200134.6
A17G0637	1.85	1.16	WDHD1	WD repeat and HMG-box DNA-binding protein 1	XM_021467145.2
B11G0496	1.85	4.61	AADACL4	Arylacetamide deacetylase-like 4	NM_001430897.1
A5G1315	1.85	2.09	SLC25A11	malate carrier protein	NM_001002099.1
B25G0200	1.85	7.24	IGKV4-1	Immunoglobulin kappa variable 4-1	XM_017354192.3
B10G0747	1.85	1.16	POLM	RNA polymerase mu	XM_068223735.1
B19G0550	1.85	11.12	PDK2	[Pyruvate dehydrogenase (acetyl-transferring)] kinase isozyme 2, mitochondrial	NM_001128704.1
A23G0073	1.85	19.01	TP53INP2	Tumor protein p53-inducible nuclear protein 2	XM_021469873.2
A15G0456	1.85	4.10	A0A671LFB7	A0A671LFB7_9TELE BCL2 binding component 3	NM_001045472.2
B4G0587	1.85	3.09	VEZT	Vezatin	XM_068219480.1
A9G0290	1.85	4.28	KDM6A	Lysine-specific demethylase 6A	XM_005167794.5
B4G0392	1.85	2.23	SMO	Smoothened homolog (Fragment)	NM_131027.2
B1G1072	1.85	0.00	SP6	Transcription factor Sp6	XM_021468395.2
B23G0835	1.85	3.96	GMEB2	Glucocorticoid modulatory element-binding protein 2	XM_009297110.4
A22G0487	1.85	6.62	UBXN6	UBX domain-containing protein 6	NM_001044780.1
B9G0054	1.85	2.69	AKAP17A	A-kinase anchor protein 17A	NM_200682.2
B22G0637	1.85	3.06	MALT1	Mucosa-associated lymphoid tissue lymphoma translocation protein 1	NM_152976.1
B22G1040	1.85	2.31	MAP1S	Microtubule-associated protein 1S	XM_021473552.2
A25G0184	1.85	3.80	RIC8A	Synembryn-A	XM_005162988.5

B25G0801	1.85	3.14	CCDC77	Coiled-coil domain-containing protein 77	XM_005170662.5
B19G0534	1.85	27.20	TINAGL1	Tubulointerstitial nephritis antigen-like	NM_001044977.2
B14G0017	1.85	3.43	RTASE	Probable RNA-directed DNA polymerase from transposon BS	XM_068220297.1
A15G0053	1.85	10.35	DGKD	Diacylglycerol kinase delta	XM_068215329.1
B8G0667	1.85	2.37	REXO1	RNA exonuclease 1 homolog	NM_001126416.1
A16G0603	1.85	3.99	ZFY1	Zinc finger Y-chromosomal protein 1	NM_001328215.1
B16G0150	1.85	0.23	G5714_015676	A0A7J6C7V5_9TELE Uncharacterized protein	XM_009292662.4
A13G0500	1.85	8.19	PCNX2	Pecanex-like protein 2	NM_001013529.2
A3G0765	1.85	2.06	ARMC5	Armadillo repeat-containing protein 5	XM_693324.9
A20G0492	1.85	5.81			XM_005160586.4
B25G0208	1.85	1.02	IGKV2-29	Immunoglobulin kappa variable 2-29	XM_068217596.1
A11G0577	1.85	1.79	BSN	Protein bassoon	NM_001080079.1
A21G0472	1.85	6.87	ZW10	kinetochore protein zw10 homolog	NM_200141.2
B20G0363	1.85	2.84	OCIAD2	OCIA domain-containing protein 2	NM_001044856.2
A16G0023	1.85	4.29	AZI2	5-azacytidine-induced protein 2	NM_214733.2
A20G0449	1.85	0.41	LD	Formin	XM_684477.7
B1G1035	1.85	0.18	RASD1	Dexamethasone-induced Ras-related protein 1	XM_001345161.7
A7G1207	1.85	2.09	INTU	Protein inturned	NM_001353932.1
B6G0941	1.85	2.04	LOC113103972	A0A6P6PL90_CARAU high mobility group protein HMGI-C-like	
B5G0202	1.85	0.19			
B12G0647	1.85	3.49	TUBGCP2	Gamma-tubulin complex component 2	NM_200122.1
B4G0766	1.85	0.77	DPX16_3573	A0A3N0XQZ7_ANAGA Zinc finger protein 271	XM_021475755.2
B2G0228	1.85	2.07	GPC5	Glypican-5	XM_005171430.5
B19G0397	1.85	1.52	TRIL	TLR4 interactor with leucine rich repeats	XM_009294188.3
B5G1099	1.84	2.35	PNPLA7	Patatin-like phospholipase domain-containing protein 7	XM_687184.9
A3G0415	1.84	4.18	DOCK7	Dedicator of cytokinesis protein 7	XM_068219443.1
A4G0056	1.84	0.22	POL	Retrovirus-related Pol polyprotein from transposon 412	
B22G0980	1.84	1.81	G5714_021948	A0A7J6BSH6_9TELE Ig-like domain-containing protein	XM_068219015.1
A25G0615	1.84	2.30	PHRF1	PHD and RING finger domain-containing protein 1	XM_002666868.6
A18G0067	1.84	4.54	FOXA1	Forkhead box protein A1	NM_131299.1
A23G0051	1.84	5.60	TRAM1L1	Translocating chain-associated membrane protein 1-like 1	NM_001014341.1
B21G0162	1.84	7.52	ALDH3A2	Aldehyde dehydrogenase family 3 member A2	NM_001328218.1
B14G0039	1.84	4.70	LOC113114415	A0A6P6QUI0_CARAU uncharacterized protein LOC113114415	
A4G0621	1.84	5.17	ANKS1B	Ankyrin repeat and sterile alpha motif domain-containing protein 1B	XM_017355052.3
B9G0800	1.84	1.56	INSIG2	Insulin-induced gene 2 protein	NM_212804.1

A7G0318	1.84	2.33	GGH	Gamma-glutamyl hydrolase	NM_213322.1
A11G0140	1.84	3.25	A0A672MYI9	A0A672MYI9_SINGR Uncharacterized protein	NM_131233.1
A10G0623	1.84	9.62	TACC1	Transforming acidic coiled-coil-containing protein 1	XM_068223947.1
A10G0105	1.84	2.13	ATPSCKMT	ATP synthase subunit C lysine N-methyltransferase	
A22G0707	1.84	5.68	KLC1	Kinesin light chain 1	NM_001083550.1
A7G1358	1.84	1.40	LPCAT4	Lysophospholipid acyltransferase LPCAT4	NM_205559.2
B8G0320	1.84	6.57	KDM2B	Lysine-specific demethylase 2B	NM_213167.2
B5G1093	1.84	1.89	FUT7	Alpha-(1,3)-fucosyltransferase 7	
A5G0222	1.84	4.18	TAOK3	threonine-protein kinase TAO3	XM_068221408.1
B1G1054	1.84	1.25	CD300LD	CMRF35-like molecule 5	XM_005160208.5
B16G0504	1.84	1.45	SCNM1	Sodium channel modifier 1	XM_005158161.5
A21G0184	1.84	1.90	CASP7	Caspase-7	NM_001386318.1
B3G0649	1.84	8.33	MLX	Max-like protein X	NM_001024223.2
A9G0871	1.84	2.31	G5714_004049	A0A7J6DBD3_9TELE Uncharacterized protein	
B6G0960	1.84	0.93	ADORA1	Adenosine receptor A1	XM_688840.6
B21G0750	1.84	1.33	ESM1	Endothelial cell-specific molecule 1	NM_001076741.1
B5G0457	1.84	2.26	RAD51D	DNA repair protein RAD51 homolog 4	NM_207076.2
B16G1076	1.84	4.67	CYB5R4	Cytochrome b5 reductase 4	XM_005173452.5
A6G0476	1.84	4.56	FARP2	FERM, ARHGEF and pleckstrin domain-containing protein 2	XM_009302421.3
A23G0090	1.84	2.39	FBXO30	F-box only protein 30	NM_200432.1
A3G1245	1.84	9.57	PRKCA	Protein kinase C alpha type	XM_009299810.4
A20G0424	1.84	6.27	ACBD3	Golgi resident protein GCP60	NM_001328207.1
A18G0126	1.84	0.00	PFLUV_G00196910	A0A6A5DVP0_PERFL HECT domain-containing protein	
A20G0614	1.84	4.07	BRF1	Transcription factor IIIB 90 kDa subunit	XM_068215387.1
B5G0897	1.84	1.41	FKBP15	FK506-binding protein 15	XM_068219191.1
B5G0207	1.84	2.65	TCTN2	Tectonic-2	XM_687198.9
B2G1027	1.84	1.74	TNK2	Activated CDC42 kinase 1	XM_005163683.4
A12G0730	1.84	2.34	CEP55	Centrosomal protein of 55 kDa	NM_001020762.1
B23G0713	1.84	0.72	HOXC12A	Homeobox protein Hox-C12a	NM_001110759.1
B10G0101	1.84	2.16	VLDLR	Very low-density lipoprotein receptor	NM_200923.2
A3G1045	1.84	3.18	SLC25A17	Peroxisomal membrane protein PMP34	NM_001099261.2
B23G0857	1.84	2.92	G5714_022276	A0A7J6BMP8_9TELE Uncharacterized protein	
B6G0382	1.84	0.59	EVPL	Envoplakin	NM_001118897.1
A24G0284	1.84	5.19	STAG1	Cohesin subunit SA-1	XM_687028.9
A25G0532	1.84	6.35	GALK2	N-acetylgalactosamine kinase	NM_001007432.2
B6G0789	1.84	7.14	GLYCTK	Glycerate kinase	NM_001316959.1
A17G0369	1.84	1.54	MIS18BP1	Mis18-binding protein 1	NM_001312886.1
B2G0190	1.84	13.58	CD244	Natural killer cell receptor 2B4	XM_017352350.3
A23G0098	1.84	3.56	RTASE	Probable RNA-directed DNA polymerase from transposon BS	
A22G0249	1.83	3.50	LOC107745969	A0A673HE92_9TELE Uncharacterized LOC107745969	NM_001386835.1
A9G0256	1.83	8.63	TFDP1	Transcription factor Dp-1	NM_213492.2
A23G0479	1.83	1.17	P2RY1	P2Y purinoceptor 1	XM_009296933.3
A24G0533	1.83	2.60	ANKHB	Progressive ankylosis protein homolog B	NM_001030259.2
A3G1008	1.83	2.21	LOC113044418	A0A6P6JKG8_CARAU ZW10 interactor-like isoform X2	XM_001340431.9

B1G0594	1.83	0.00	RTASE	Probable RNA-directed DNA polymerase from transposon BS	XM_068220297.1
A8G0360	1.83	3.30	FCHO1	F-BAR domain only protein 1	NM_001423857.1
A10G0177	1.83	5.73	ATG10	Ubiquitin-like-conjugating enzyme ATG10	XM_068223824.1
A22G0718	1.83	2.44	PEX6	Peroxisome assembly factor 2	XM_001332616.9
B2G1197	1.83	2.08	ACKR2	Atypical chemokine receptor 2	
A9G0394	1.83	3.35	DNAJC3	DnaJ homolog subfamily C member 3	NM_199610.2
A23G0509	1.83	1.06	LOC107716202	A0A673NMR2_9TELE Uncharacterized LOC107716202	XM_001334580.8
B19G0422	1.83	0.00	DPX16_5508	A0A3N0Z396_ANAGA Uncharacterized protein	
B8G0989	1.83	3.66	FAM102A	Protein FAM102A	XM_003199038.6
B25G0213	1.83	4.33	RASSF8	Ras association domain-containing protein 8	XM_009298065.4
A4G0756	1.83	0.13	ENDOUC	Uridylate-specific endoribonuclease C	NM_001044967.1
B6G0762	1.83	4.04	RAD18	E3 ubiquitin-protein ligase RAD18	NM_200137.1
B13G0740	1.83	3.55	PYROXD2	Pyridine nucleotide-disulfide oxidoreductase domain-containing protein 2	XM_009307117.4
A20G0270	1.83	7.69	DNAJC5	DnaJ homolog subfamily C member 5	XM_005160813.5
A4G0439	1.83	1.95	PTPRR	Receptor-type tyrosine-protein phosphatase R	XM_021475305.2
B12G0734	1.83	0.82	SLC2A11	Solute carrier family 2, facilitated glucose transporter member 11	
B20G0410	1.83	1.78	TTBK2	Tau-tubulin kinase 2	NM_001326291.1
A22G0480	1.83	0.09	GNA15	Guanine nucleotide-binding protein subunit alpha-15	XM_002667364.6
B21G0609	1.83	1.13	CCDC74B	Coiled-coil domain-containing protein 74B	NM_001282393.1
A9G0096	1.83	2.42	STK39	SPS1-related proline-alanine-rich protein kinase	XM_002667521.7
B22G0140	1.83	0.39	GIPC3	PDZ domain-containing protein GIPC3	NM_001045318.2
A10G0064	1.83	2.35	PDXP	Chronophin	NM_001128705.1
A21G0243	1.83	29.51	SPTAN1	Spectrin alpha chain, non-erythrocytic 1	XM_021468833.1
A11G0778	1.83	15.88	ITGA7	Integrin alpha-7	XM_021479923.2
A4G0822	1.83	3.35	TMTC1	Protein O-mannosyl-transferase TMTC1	XM_001921990.8
A25G0285	1.83	2.38	PARVA	Alpha-parvin	NM_001002872.2
A2G0855	1.83	2.05	TOX4	TOX high mobility group box family member 4	XM_021471257.2
A12G0043	1.83	4.65	DNAJB12	DnaJ homolog subfamily B member 12	NM_001089508.1
A13G0729	1.83	1.62	PRKG2	cGMP-dependent protein kinase 2	XM_017358846.3
B14G0231	1.83	1.14	G5714_014106	A0A7J6CDR0_9TELE Uncharacterized protein	
B25G0638	1.83	36.72	SEPTIN7	Septin-7	XM_021470541.2
A12G0625	1.83	8.55	PLEKHA1	Pleckstrin homology domain-containing family A member 1	XM_068224733.1
A16G0065	1.83	3.55	SLC9A1	hydrogen exchanger 1	XM_005173542.5
B24G0245	1.83	1.74	FASN	Fatty acid synthase	XM_677883.8
B7G0076	1.83	2.93	MPDZ	Multiple PDZ domain protein	XM_009303673.4
A2G0657	1.83	7.40	AMPH	Amphiphysin	XM_068223372.1

A13G0537	1.83	4.27	ARSI	Arylsulfatase I	NM_201496.1
B10G0184	1.82	4.37	HVCN1	Voltage-gated hydrogen channel 1	NM_001002346.1
A17G0352	1.82	14.38	EHD4	EH domain-containing protein 4	NM_001386837.1
A11G0563	1.82	5.95	OPSP	Parapinopsin	XM_678952.10
A5G0258	1.82	4.28	ATP8B4	Probable phospholipid-transporting ATPase IM	XM_021476431.2
A4G0258	1.82	10.12	LOC107752851	A0A673NF79_9TELE Uncharacterized LOC107752851	NM_001083816.2
B10G0561	1.82	1.22	SMYD4	SET and MYND domain-containing protein 4	NM_001076594.1
B9G0476	1.82	1.68	SH3BP4	SH3 domain-binding protein 4	NM_001123295.1
B8G0086	1.82	6.23	KANK3	KN motif and ankyrin repeat domain-containing protein 3	XM_068222975.1
A20G0563	1.82	0.09	MDN1	Midasin	XM_003200703.6
B4G0514	1.82	3.24	SORT1	Sortilin	XM_683991.10
B13G0890	1.82	4.16	LBH	Protein LBH	XM_005161933.5
A19G0306	1.82	13.72	LAPTM4B	Lysosomal-associated transmembrane protein 4B	NM_200098.2
B1G0579	1.82	2.85	ZNF827	Zinc finger protein 827	XM_005159977.5
A6G0674	1.82	0.97	TSFM	Elongation factor Ts, mitochondrial	NM_001080035.1
A23G0113	1.82	4.67	HDAC7	Histone deacetylase 7	XM_021470075.2
B14G0501	1.82	0.14	IL17B	Interleukin-17B	
B12G0210	1.82	9.50	FASN	Fatty acid synthase	XM_009306807.4
A20G0286	1.82	5.34	EHD3	EH domain-containing protein 3	XM_068215509.1
A6G0520	1.82	1.33	MTR	Methionine synthase	NM_001114914.1
A21G0570	1.82	98.89	HIF1A	Hypoxia-inducible factor 1-alpha	NM_001012371.2
B21G0426	1.82	6.68	NF2	Merlin (Fragment)	NM_212951.1
B23G0049	1.82	2.68	FBXW12	WD repeat-containing protein 12	NM_001122628.2
B19G0606	1.82	21.98	MACF1	Microtubule-actin cross-linking factor 1	XM_068215317.1
B16G0016	1.82	8.69	CRTC2	CREB-regulated transcription coactivator 2	XM_021467058.2
B13G0871	1.82	3.84	HS3ST1	Heparan sulfate glucosamine 3-O-sulfotransferase 1	NM_001040247.2
B15G0339	1.82	5.36	FLOT2	Flotillin-2	NM_200639.1
A16G0333	1.82	1.90	MAP3K5	Mitogen-activated protein kinase kinase kinase 5	XM_009292511.4
A25G0296	1.82	5.61	ADA2A	Adenosine deaminase 2-A	NM_001033717.2
A16G0568	1.82	1.70	NSMCE2	E3 SUMO-protein ligase NSE2	NM_001128718.1
B19G0104	1.82	10.72	TAP2	Antigen peptide transporter 2	NM_001006594.1
B11G0153	1.82	4.69	PTPRG	Receptor-type tyrosine-protein phosphatase gamma	
B2G1123	1.82	4.06	FZR1	Fizzy-related protein homolog	XM_005163252.5
B16G0984	1.82	5.20	TRAK1	Trafficking kinesin-binding protein 1	XM_009292048.4
A21G0477	1.82	8.73	NCAM1	Neural cell adhesion molecule 1	XM_005161138.5
A13G0603	1.82	1.27	GABRB1	Gamma-aminobutyric acid receptor subunit beta-1	NM_001145590.1
A14G0545	1.82	0.30	B3GNT2	N-acetyllactosaminide beta-1,3-N-acetylglucosaminyltransferase 2	XM_682219.7
B3G0586	1.82	8.35	FOXK1	Forkhead box protein K1	NM_199902.1
A25G0101	1.82	0.33	CMASB	N-acylneuraminate cytidylyltransferase B	NM_001258409.1
A6G0986	1.82	6.07	RALGAPB	Ral GTPase-activating protein subunit beta	XM_009302766.4

B6G0807	1.82	2.45	PDZRN3B	E3 ubiquitin-protein ligase PDZRN3-B	NM_001256392.1
A16G0408	1.82	1.81	PHC1	Polyhomeotic-like protein 1	XM_005158338.5
A12G0169	1.82	6.68	AATK	threonine-protein kinase LMTK1	XM_021472589.2
A1G1016	1.82	3.10	TMA16	Translation machinery-associated protein 16	NM_001177745.1
B14G0511	1.82	3.69	ELP1	Elongator complex protein 1	XM_684442.9
B3G0318	1.82	9.18	CREBBP	CREB-binding protein	NM_001365186.1
B2G1077	1.82	3.62	DIPK2A	Divergent protein kinase domain 2A	NM_001007436.1
B21G0712	1.82	3.99	UBAP2	Ubiquitin-associated protein 2	NM_001082838.2
A7G0383	1.82	5.26	DPX16_12502	A0A3N0Y0Z1_ANAGA Uncharacterized protein	
B4G0907	1.82	2.70	DPX16_3453	A0A3N0XKN3_ANAGA Zinc finger protein 569	XM_068219487.1
B2G0602	1.82	2.79	LOC107733888	A0A673KUV1_9TELE Zinc finger protein 667-like	XM_687018.8
B3G1035	1.82	5.92	DHX58	ATP-dependent RNA helicase DHX58	NM_001257157.1
A16G0193	1.81	0.13	LOC107666836	A0A671MI36_9TELE Uncharacterized LOC107666836	
B8G0557	1.81	6.47	RBM15	RNA-binding protein 15	XM_001335784.8
A15G0142	1.81	2.10	GAL3ST2	Galactose-3-O-sulfotransferase 2	XM_678858.10
B2G0941	1.81	11.62	PPP1R7	Protein phosphatase 1 regulatory subunit 7	NM_001037386.2
A13G0340	1.81	10.40	LOC113056869	A0A6P6L4T7_CARAU Palmitoyltransferase	XM_021472679.2
A5G0807	1.81	9.86	RUFY3	Protein RUFY3	XM_009301649.4
B14G0486	1.81	1.86	SLC39A8	Metal cation symporter ZIP8	
A3G0163	1.81	0.44	SPHK1	Sphingosine kinase 1	XM_002667539.7
A22G0616	1.81	4.75	PDGFB	Platelet-derived growth factor subunit B	NM_001423262.1
A23G0745	1.81	0.11	TGM1	Protein-glutamine gamma-glutamyltransferase K	XM_009297092.4
B13G0314	1.81	1.28	SUFU	Suppressor of fused homolog	NM_001145583.1
A2G1075	1.81	4.08	HECW1	E3 ubiquitin-protein ligase HECW1	XM_009298865.4
B3G0163	1.81	0.75	G5714_004003	A0A7J6DCC3_9TELE Uncharacterized protein	
B18G0825	1.81	3.03	CYP1A1	Cytochrome P450 1A1	NM_131879.2
A23G0310	1.81	3.75	FAM110B	Protein FAM110B	XM_009296762.4
A13G0556	1.81	6.90	RTASE	Probable RNA-directed DNA polymerase from transposon BS	
A18G0096	1.81	15.33	EST1	Liver carboxylesterase 1	XM_009293838.4
A23G0429	1.81	4.06	PLEKHM2	Pleckstrin homology domain-containing family M member 2	NM_001130783.1
B15G0833	1.81	0.63	CLDN8	Claudin-8	NM_001017799.2
B1G0853	1.81	6.24	PSEN2	Presenilin-2	NM_131514.2
B16G0520	1.81	5.68	BCAN	Brevican core protein	NM_001083813.2
B20G0426	1.81	2.35	HINT3	Adenosine 5'-monophosphoramidase HINT3	
B9G0823	1.81	5.84	CMKLR2	Chemerin-like receptor 2	XM_001343478.8
B14G0659	1.81	2.61	CSF1R	Macrophage colony-stimulating factor 1 receptor	NM_131672.1
B13G0817	1.81	4.40	SLC39A9-B	Zinc transporter ZIP9-B	XM_017358922.3
B2G0578	1.81	5.09	COP1	E3 ubiquitin-protein ligase COP1	XM_021478383.2
A8G0728	1.81	5.34	GOLGA1	Golgin subfamily A member 1	XM_005169080.5
A13G0173	1.81	3.12	LMBRD1	Lysosomal cobalamin transport escort protein LMBD1	XM_001921404.9

A3G0905	1.81	0.00	MYO1D	A0A4W5JNC1_9TELE Myosin ID	XM_068216580.1
A2G0130	1.81	2.52	ZBTB41	Zinc finger and BTB domain-containing protein 41	XM_001922667.8
B20G0282	1.81	2.12	GPR132	Probable G-protein coupled receptor 132	NM_001025547.2
B12G0351	1.81	3.11	BMPR1A	Bone morphogenetic protein receptor type-1A	NM_001004585.2
B2G0872	1.81	3.45	MLLT1	Protein ENL	NM_001040384.1
A4G0617	1.81	1.21	CRADD	Death domain-containing protein CRADD	NM_001006066.1
B12G0818	1.81	8.46	TAOK2	threonine-protein kinase TAO2	XM_009306405.4
B2G0584	1.81	1.21	CENPL	Centromere protein L	NM_001003878.2
B3G0272	1.81	0.57	TMEM238	Transmembrane protein 238	XM_002661091.7
B15G0341	1.81	7.69	CALN1	Calcium-binding protein 8	XM_021466346.2
B2G0599	1.81	3.71	TMEM53	Transmembrane protein 53	NM_001002637.1
B5G0394	1.81	5.78	SLC31A1	High affinity copper uptake protein 1	NM_205717.3
A4G0105	1.80	10.24	NLRP3	NACHT, LRR and PYD domains-containing protein 3	XM_017352412.3
A22G0544	1.80	7.51	RRBP1	Ribosome-binding protein 1	NM_199431.2
A7G1314	1.80	11.70	NDRG2	Protein NDRG2	NM_001008593.1
A1G0624	1.80	2.58	C3ORF70A	UPF0524 protein C3orf70 homolog A	NM_001126467.1
B5G0430	1.80	3.14	CCDC15	Coiled-coil domain-containing protein 15	XM_009301883.3
B13G0726	1.80	5.61	COL19A1	Collagen alpha-1(XIX) chain	XM_009307103.4
A17G0106	1.80	5.22	A0A673NF18	A0A673NF18_9TELE Uncharacterized protein	XM_068219469.1
A1G0796	1.80	6.84	RETREG2	Reticulophagy regulator 2	NM_001080014.1
A11G0227	1.80	2.04	BCL2L15	Bcl-2-like protein 15	NM_001005206.1
A20G0735	1.80	5.71	GTF2A1	Transcription initiation factor IIA subunit 1	NM_212657.1
A20G0221	1.80	1.15	DCBLD1	Discoidin, CUB and LCCL domain-containing protein 1	NM_001039977.2
A18G0431	1.80	1.43	LOC107583743	A0A672MFL0_SINGR Uncharacterized LOC107583743	NM_001044713.2
B11G0089	1.80	0.07	GHRH	Somatoliberin	XM_009306309.3
A5G0177	1.80	2.46	EHD2	EH domain-containing protein 2	NM_001128748.1
A17G0569	1.80	0.81	RYR2	Ryanodine receptor 2	XM_068214574.1
A6G0529	1.80	6.48	PDE6D	Retinal rod rhodopsin-sensitive cGMP 3',5'-cyclic phosphodiesterase subunit delta	NM_001002708.1
A4G0562	1.80	2.75	DCP1B	mRNA-decapping enzyme 1B	XM_021475124.2
A13G0521	1.80	1.40	TRIM62	E3 ubiquitin-protein ligase TRIM62	XM_005156935.5
B20G0598	1.80	8.33	KIFAP3	Kinesin-associated protein 3	NM_001004644.1
B6G0739	1.80	2.77	SFI1	Protein SFI1 homolog	XM_683537.9
B3G0387	1.80	3.97	PDGFA	Platelet-derived growth factor subunit A	XM_005163736.5
B19G0647	1.80	1.45	LOC113119461	A0A6P6RHS1_CARAU uncharacterized protein LOC113119461	
A15G0730	1.80	4.24	RIN2	Ras and Rab interactor 2	XM_068213677.1
A1G0536	1.80	1.57	CEP44	Centrosomal protein of 44 kDa	NM_001076736.2
A5G0645	1.80	1.56	TTC33	Tetratricopeptide repeat protein 33	NM_001002722.1
B11G0375	1.80	2.47	LOC113051950	A0A6P6KLF3_CARAU fibroin heavy chain-like isoform X1	XM_009306035.4
A4G0365	1.80	1.59	A0A673G440	A0A673G440_9TELE Uncharacterized protein	XM_017355493.3

B24G0233	1.80	4.92	SLC4A2	Anion exchange protein 2	NM_001114440.2
A21G0696	1.80	2.66	KIF4A	Chromosome-associated kinesin KIF4A	NM_200823.1
A13G0438	1.80	2.44	RET	Proto-oncogene tyrosine-protein kinase receptor Ret	
B3G0604	1.80	2.85	SMCR8A	Guanine nucleotide exchange protein smcr8a	NM_001353855.1
A12G0452	1.80	9.38	CSDC2	Cold shock domain-containing protein C2	NM_001002690.1
A11G0780	1.80	1.21	AVIL	Advillin	
B12G0315	1.80	5.42	DUSP3	Dual specificity protein phosphatase 3	XM_021472556.2
A24G0530	1.80	2.45	YAE1	Protein YAE1 homolog	NM_213142.1
B24G0310	1.80	0.66	A0A672MG29	A0A672MG29_SINGR SCY domain-containing protein	
B22G0779	1.80	5.24	CKS1B	Cyclin-dependent kinases regulatory subunit 1	
B19G0533	1.80	5.72	TRIO	Triple functional domain protein	XM_021468075.2
B11G0602	1.80	1.79	SYPL1	Synaptophysin-like protein 1	
A24G0610	1.80	0.51	PCOLCE2	Procollagen C-endopeptidase enhancer 2	NM_200460.1
A13G0023	1.80	1.13	NLRC3	NLR family CARD domain-containing protein 3	NM_001045009.3
A22G0346	1.80	11.33	ANKRD29	Ankyrin repeat domain-containing protein 29	
A8G0905	1.80	3.11	WRAP73	WD repeat-containing protein WRAP73	NM_001328537.1
B21G0207	1.79	15.22	DBN1	Drebrin	XM_009295616.4
A1G0810	1.79	1.50	EFNB2A	Ephrin-B2a	NM_131808.1
B10G0751	1.79	8.51	CAMK2B	calmodulin-dependent protein kinase type II subunit beta	XM_009305704.4
A4G1023	1.79	1.27	RERG	Ras-related and estrogen-regulated growth inhibitor	NM_001327837.1
B6G0787	1.79	1.26	TNFRSF9	Tumor necrosis factor receptor superfamily member 9	
B19G0026	1.79	2.69	NLRC3	NLR family CARD domain-containing protein 3	XM_017352412.3
B19G0305	1.79	0.86	SLC9A3	hydrogen exchanger 3	XM_005159457.5
B22G0741	1.79	1.30	HSD11B1L	Hydroxysteroid 11-beta-dehydrogenase 1-like protein	NM_200323.2
A22G0376	1.79	3.55	PIP5K1C	Phosphatidylinositol 4-phosphate 5-kinase type-1 gamma	XM_009296337.4
B12G0502	1.79	4.12	TRRAP	transcription domain-associated protein	XM_009306682.4
B19G0066	1.79	2.05	NLRC3	NLR family CARD domain-containing protein 3	XM_017352412.3
A3G0174	1.79	2.42	TRIM16	Tripartite motif-containing protein 16	XM_068213621.1
A12G0078	1.79	0.58	TRIM16	E3 ubiquitin-protein ligase TRIM16	NM_001328162.1
A15G0063	1.79	5.23	LOC113058807	A0A6P6LDZ7_CARAU cell adhesion molecule 2-like isoform X1	
A11G0298	1.79	0.60	IL10	Interleukin-10	NM_001030143.1
A18G0375	1.79	0.00			
B17G0161	1.79	3.07	LOC107665099	A0A671LEQ4_9TELE Antimicrobial peptide NK-lysin-like	
B16G0659	1.79	2.37	PON2	arylesterase 2	NM_212734.1
B23G0197	1.79	6.39	ZBTB46	Zinc finger and BTB domain-containing protein 46	XM_021473582.2

B23G0574	1.79	4.92			
B23G0131	1.79	2.77	RBM10	RNA-binding protein 10	XM_009296566.4
A11G0255	1.79	2.26	UBA3	NEDD8-activating enzyme E1 catalytic subunit	XM_068219295.1
B22G0965	1.79	4.13	G5714_021930	A0A7J6BW91_9TELE Uncharacterized protein	
A16G1001	1.79	1.30	LARS2	Probable leucine--tRNA ligase, mitochondrial	NM_001105701.1
A19G0688	1.79	5.96	RPRD2	Regulation of nuclear pre-mRNA domain-containing protein 2	NM_199922.1
B5G0290	1.79	5.04	ABHD10	Palmitoyl-protein thioesterase ABHD10, mitochondrial	XM_005165639.5
A2G0400	1.79	1.15	HCCS	Holocytochrome c-type synthase	NM_001002498.2
A21G0285	1.79	8.30	MMP11	Stromelysin-3	XM_689061.10
B4G0902	1.79	0.93	A0A673LUR3	A0A673LUR3_9TELE Uncharacterized protein	XM_017355348.3
B17G0180	1.79	0.92	G5714_016766	A0A7J6C439_9TELE Methyltransf_11 domain-containing protein	NM_001020545.1
B7G0939	1.79	4.30	TNFSF10	Tumor necrosis factor ligand superfamily member 10	NM_131843.2
A17G0002	1.79	3.45	TTC8	Tetratricopeptide repeat protein 8	NM_001040309.1
B5G0867	1.79	0.33	LOC113050030	A0A6P6K9G4_CARAU uncharacterized protein LOC113050030	
B8G0735	1.79	2.28	FPGT	Fucose-1-phosphate guanylyltransferase	NM_001020754.2
B22G0777	1.79	2.58	CPAMD8	C3 and PZP-like alpha-2-macroglobulin domain-containing protein 8	XM_003201057.6
B15G0792	1.79	2.03	LPAR5	Lysophosphatidic acid receptor 5	NM_001128797.1
B11G0112	1.79	1.66	THOP1	Thimet oligopeptidase	XM_068224269.1
B19G0797	1.79	11.39	SGK1	threonine-protein kinase Sgk1	NM_001077302.2
B5G0152	1.79	2.77	UGT2A1	UDP-glucuronosyltransferase 2A1	NM_001365233.1
A5G0180	1.79	5.01	LZTR1	Leucine-zipper-like transcriptional regulator 1	NM_001080605.2
A6G0954	1.79	0.28	SMPD2	Sphingomyelin phosphodiesterase 2	
A9G0778	1.79	5.65	AGPS	Alkyldihydroxyacetonephosphate synthase, peroxisomal	NM_200113.1
A23G0465	1.79	6.19	FMNL3	Formin-like protein 3	XM_009296931.4
A5G1046	1.79	4.91	ARHGEF12	Rho guanine nucleotide exchange factor 12	XM_009301886.4
A9G0578	1.78	3.88	HNMT	Histamine N-methyltransferase	NM_001003636.1
B25G0824	1.78	15.78	FRIH	Ferritin, heavy subunit	NM_001004562.1
A21G0527	1.78	6.19	SDF2	Stromal cell-derived factor 2	NM_200039.1
B20G0285	1.78	3.50	LBH	Protein LBH	
B10G0108	1.78	9.00	ERBIN	Erbin	XM_005155577.4
B11G0552	1.78	3.07			XM_694726.10
B22G0800	1.78	2.24	CHAF1A	Chromatin assembly factor 1 subunit A	NM_001045013.2
A7G1063	1.78	1.71	PELI3	E3 ubiquitin-protein ligase pellino homolog 3	XM_021477739.2
A23G0604	1.78	2.66	NEMP1	Nuclear envelope integral membrane protein 1	NM_001316731.1
B24G0447	1.78	3.62	WASF3	Actin-binding protein WASF3	NM_001080590.1
B3G1606	1.78	1.82	A0A672SDS1	A0A672SDS1_SINGR Uncharacterized protein	XM_009300963.4

A5G0499	1.78	6.50	MAN1B1	Endoplasmic reticulum mannosyl-oligosaccharide 1,2-alpha-mannosidase	NM_001099979.1
A22G0010	1.78	2.18	PRKAR2B	cAMP-dependent protein kinase type II-beta regulatory subunit	NM_212958.1
A20G0922	1.78	7.57	SLC16A10	Monocarboxylate transporter 10	XM_005160300.5
A14G0822	1.78	1.02	CYSLTR1	Cysteinyl leukotriene receptor 1	NM_001020648.1
B2G1314	1.78	4.87	STXA	Stonustoxin subunit alpha	XM_021475328.2
B15G0524	1.78	12.45	RFLNB	Refilin-B	NM_001386285.1
A12G0761	1.78	0.16	TECTA	Alpha-tectorin	
A1G0015	1.78	0.61	AXIN1	Axin-1	
A5G0304	1.78	2.00	RDH11	Retinol dehydrogenase 11	NM_001082916.2
B7G0547	1.78	4.62	TBC1D14	TBC1 domain family member 14	XM_005166439.5
B6G0911	1.78	3.95	ZC3H10	Zinc finger CCCH domain-containing protein 10	NM_207098.1
B23G0537	1.78	5.15	LARP4	La-related protein 4	XM_009296950.4
A7G0359	1.78	2.78	RELL1	RELT-like protein 1	NM_001201360.1
B10G0740	1.78	3.45	IL1B	Interleukin-1 beta	
B22G0881	1.78	19.84	CXCL12	Stromal cell-derived factor 1	NM_001320414.1
B17G0357	1.78	1.04	SHTN1	Shootin-1	NM_001198761.1
A11G0643	1.78	3.51	HES5	Transcription factor HES-5	
B6G0004	1.78	3.40	MARF1	Meiosis regulator and mRNA stability factor 1	XM_021476962.2
A8G0921	1.78	1.55	PLCH2	1-phosphatidylinositol 4,5-bisphosphate phosphodiesterase eta-2	NM_001347692.1
A16G0234	1.78	2.07	ETS1	c-ets-1	NM_001037375.1
B8G0883	1.78	4.03			
A2G0993	1.78	3.47	NCAPD2	Condensin complex subunit 1	
B7G0046	1.78	12.00	TRIM21	E3 ubiquitin-protein ligase TRIM21	
B4G0268	1.78	5.20	FBH1	F-box DNA helicase 1	XM_017355080.3
A5G0106	1.78	1.53	ELAC1	Zinc phosphodiesterase ELAC protein 1	NM_001003503.1
B7G0588	1.78	1.38	PTPMT1	Phosphatidylglycerophosphatase and protein-tyrosine phosphatase 1	NM_001080187.1
B9G0581	1.78	1.64	TRIM35	E3 ubiquitin-protein ligase TRIM35	
B9G0073	1.78	4.79	OFD1	Centriole and centriolar satellite protein ofd1	XM_009305014.4
A25G0243	1.78	6.67	FA2H	Fatty acid 2-hydroxylase	NM_001430959.1
B16G0644	1.78	2.93	MYPOP	Myb-related transcription factor, partner of profilin	XM_003200185.6
A12G0584	1.78	1.64	PKMYT1	Membrane-associated tyrosine- and threonine-specific cdc2-inhibitory kinase	NM_001098196.1
B5G0369	1.78	3.45	GNAQ	Guanine nucleotide-binding protein G(q) subunit alpha	NM_001144799.1
A4G0859	1.78	2.29	TFEC	Transcription factor EC	XM_068219644.1
A20G0423	1.78	1.42	NHSL1	NHS-like protein 1	XM_005160764.5
A7G0935	1.78	3.11	CCNB3	mitotic-specific cyclin-B3	NM_001076719.1
B3G1319	1.78	4.66	GID4	Glucose-induced degradation protein 4 homolog	XM_002661312.7
B2G0975	1.78	1.89	EVI5	Ecotropic viral integration site 5 protein homolog	XM_009297997.4
B5G0201	1.78	2.37	TOP3B	DNA topoisomerase 3-beta-1	XM_003198603.6
A21G0319	1.78	3.14	VAV2	Guanine nucleotide exchange factor VAV2	XM_017352942.3
A24G0368	1.77	3.24	PIGN	GPI ethanolamine phosphate transferase 1	NM_001310080.1

A8G0984	1.77	0.49	A0A173M1Y2	A0A173M1Y2_CYPCA Interleukin 17 receptor C2	
B19G0917	1.77	2.04	TRIM16	Tripartite motif-containing protein 16	NM_001430853.1
A2G0671	1.77	34.74	LOC107718023	A0A673NE78_9TELE Cystatin-like protein	
B10G0668	1.77	2.40	ADRB4C	Beta-4C adrenergic receptor	NM_001135134.1
B4G0546	1.77	4.67	GOLGB1	Golgin subfamily B member 1	XM_068220776.1
B3G1630	1.77	5.71	A0A673LZZ1	A0A673LZZ1_9TELE SGNH_hydro domain-containing protein	XM_068219418.1
A2G0483	1.77	2.77	LYRM4	LYR motif-containing protein 4	NM_001164241.1
B19G0462	1.77	2.79	TRPS1	Zinc finger transcription factor Trps1	NM_001177934.1
A19G0159	1.77	1.06	LOC113068247	A0A6P6MJW7_CARAU uncharacterized protein LOC113068247 isoform X1	
B7G0166	1.77	4.11	IRAG1	Inositol 1,4,5-triphosphate receptor associated 1	XM_021478033.2
A24G0548	1.77	2.41	POL	RNA-directed DNA polymerase from mobile element jockey	XM_068217874.1
A2G0286	1.77	1.80	SDR16C6	reductase family 16C member 6	NM_212878.2
A21G0739	1.77	8.33	LIMK1	LIM domain kinase 1	XM_005161446.5
B24G0571	1.77	2.99	MAG	Myelin-associated glycoprotein	XM_068219079.1
A2G1167	1.77	4.76	PIP5K1C	Phosphatidylinositol 4-phosphate 5-kinase type-1 gamma	NM_001365187.1
B23G0454	1.77	3.72	PLEKHM2	Pleckstrin homology domain-containing family M member 2	NM_001130783.1
A17G0650	1.77	0.19	GJB4	Gap junction beta-4 protein	XM_005169817.5
B19G0896	1.77	3.36	PDIK1L	threonine-protein kinase pdik1l	NM_001037700.2
A25G0418	1.77	0.96	RAD52	DNA repair protein RAD52 homolog	XM_005163057.5
B8G1054	1.77	2.53	PRR16	Protein Largen	NM_001110405.1
B11G0305	1.77	2.42	TAFA4	Chemokine-like protein TAFA-4	NM_001199491.1
A20G0191	1.77	2.47	RUNX2	Runt-related transcription factor 2	NM_212862.2
A15G0174	1.77	5.74	SIGLEC1	Sialoadhesin	XM_068213643.1
B18G0098	1.77	5.16	GLB1L2	Beta-galactosidase-1-like protein 2	XM_009293835.4
B24G0471	1.77	3.41	LOC107591247	arginine repetitive matrix protein 1-like	XM_005162726.5
B22G1093	1.77	0.59	CKB	Creatine kinase B-type	NM_001113598.1
A10G0547	1.77	1.21	PROSER1	Proline and serine-rich protein 1	XM_679230.10
B9G0423	1.77	1.06	AFF3	FMR2 family member 3	XM_009304671.3
B12G0613	1.77	1.86	HEATR1	HEAT repeat-containing protein 1	NM_199900.1
A5G0117	1.77	7.66	NIPBLA	Nipped-B-like protein A	NM_001246284.1
A21G0621	1.77	6.70	AFAP1L1	Actin filament-associated protein 1-like 1	XM_021468916.2
A17G0470	1.77	3.19	ABCG23	ABC transporter G family member 23	XM_681911.8
A22G0712	1.77	2.79	RCOR3	REST corepressor 3	XM_005161857.5
A5G0989	1.77	4.24	TIMM22	Mitochondrial import inner membrane translocase subunit Tim22	NM_001386807.1
B18G0346	1.77	3.75	PPP1R13L	RelA-associated inhibitor	XM_005173643.5
A21G0163	1.77	4.94	WDR41	WD repeat-containing protein 41	NM_001030261.2
A6G0829	1.77	5.00	RAP1GAP	Rap1 GTPase-activating protein 1	XM_068222049.1
A1G0844	1.77	1.37	PRKCB	Protein kinase C beta type	

A7G1206	1.77	3.06	CXCL8	Interleukin-8	
A18G0587	1.77	2.52	SLC66A2	Solute carrier family 66 member 2	XM_017352034.3
A2G0298	1.77	0.10	SET-1	Histone-lysine N-methyltransferase set-1	
A7G0882	1.77	1.21	FBXO42	F-box only protein 42	NM_001012366.1
A7G0753	1.77	7.24	PSTPIP1	Proline-serine-threonine phosphatase-interacting protein 1	NM_001423802.1
B2G0737	1.77	1.26	LRRC42	Leucine-rich repeat-containing protein 42	NM_199747.2
B7G0422	1.77	1.66	FJX1	Four-jointed box protein 1	XM_001337564.9
B2G1125	1.77	2.53	HORMAD1	HORMA domain-containing protein 1	NM_001039809.1
A23G0201	1.77	1.60	PPP1R3D	Protein phosphatase 1 regulatory subunit 3D	NM_001110412.2
B20G0664	1.77	1.26	NSL1	Kinetochore-associated protein NSL1 homolog	NM_001025521.2
B25G0116	1.77	2.99	NFAT5	Nuclear factor of activated T-cells 5	NM_199895.1
B2G0772	1.77	3.12	HLTF	Helicase-like transcription factor	XM_005163376.5
B21G0028	1.77	2.48	TRIM16	Tripartite motif-containing protein 16	XM_681071.8
B11G0095	1.77	0.18	SPATA1	Spermatogenesis-associated protein 1	XM_009306309.3
A22G0187	1.77	2.00	G5714_021436	A0A7J6BR50_9TELE Ig-like domain-containing protein	XM_068216431.1
A1G0933	1.76	22.97	CXCL8	Interleukin-8	XM_001342570.8
B23G0132	1.76	1.90	FRMD4B	FERM domain-containing protein 4B	XM_009296561.4
A9G0556	1.76	3.80	KLHL13	Kelch-like protein 13	XM_001923378.8
A25G0409	1.76	2.54	EDC3	Enhancer of mRNA-decapping protein 3	NM_001018126.2
B9G0850	1.76	3.27	CHN1	N-chimaerin	NM_213000.1
B7G0456	1.76	2.11	TSHZ3	Teashirt homolog 3	XM_021477568.2
B4G0789	1.76	2.43	NLRC3	NLR family CARD domain-containing protein 3	XM_068218404.1
B22G1068	1.76	0.35	RTP3	Receptor-transporting protein 3	
B7G0255	1.76	3.09			
B8G0009	1.76	0.66			
B13G0387	1.76	1.55	PSD	PH and SEC7 domain-containing protein 1	NM_001111149.1
B3G0719	1.76	1.57	G5714_003489	A0A7J6D9P8_9TELE IG domain-containing protein	XR_001798090.3
A16G0868	1.76	7.25	M6PR	Cation-dependent mannose-6-phosphate receptor	NM_213205.1
B25G0523	1.76	1.07	LRRC29	Leucine-rich repeat-containing protein 29	
A11G0218	1.76	1.46	EFNA2	Ephrin-A2	XM_021479950.2
B3G0020	1.76	9.04	FAM234B	Protein FAM234B	XM_005171506.5
B7G0015	1.76	26.85	PTPRD	Receptor-type tyrosine-protein phosphatase delta	XM_017354405.2
A2G0580	1.76	5.22	RNF31	E3 ubiquitin-protein ligase RNF31	XM_009298565.4
B7G0887	1.76	5.14	PIK3C3	Phosphatidylinositol 3-kinase catalytic subunit type 3	NR_137316.1
A4G0156	1.76	0.00	G5714_016288	A0A7J6C9Y5_9TELE Uncharacterized protein	
B20G0373	1.76	2.53	SH3RF1	E3 ubiquitin-protein ligase SH3RF1	NM_001045487.2
B4G0129	1.76	3.15	TBC1D30	TBC1 domain family member 30	XM_021475403.2
A18G0542	1.76	1.04			NM_001037580.2

B15G0332	1.76	4.87	KIAA0100	Protein KIAA0100	
B14G0046	1.76	0.61	CLCF1	Cardiotrophin-like cytokine factor 1	XM_685641.9
B1G0895	1.76	0.54	ACY3.2	N-acyl-aromatic-L-amino acid amidohydrolase (carboxylate-forming) B	NM_001002347.1
B8G0429	1.76	4.99	PGM5	Phosphoglucomutase-like protein 5	NM_001126396.1
B15G0374	1.76	2.82	PHLDB1	Pleckstrin homology-like domain family B member 1	XM_068213700.1
B22G0754	1.76	12.70	TCF3	Transcription factor E2-alpha	NM_001172683.1
B15G0239	1.76	4.27	SLC37A4	Glucose-6-phosphate exchanger SLC37A4	NM_214738.1
A4G0400	1.76	52.35	EDSP	Epidermal differentiation-specific protein	
A18G0658	1.76	2.25	PDP2	[Pyruvate dehydrogenase [acetyl-transferring]]-phosphatase 2, mitochondrial	NM_131625.2
B4G0777	1.76	0.84	G5714_004447	A0A7J6D5J8_9TELE Uncharacterized protein	XM_021468205.2
A20G0425	1.76	1.09	CCR6	C-C chemokine receptor type 6	NM_001099991.1
A20G0401	1.76	1.20	ARMC2	Armadillo repeat-containing protein 2	XM_678490.8
B2G0652	1.76	6.44	COLEC12	Collectin-12	NM_001122840.1
B13G0261	1.76	1.77	TTC31	Tetratricopeptide repeat protein 31	NM_001327775.1
A19G0243	1.75	3.82	KPNA6	Importin subunit alpha-7	XM_001923886.7
A12G0408	1.75	9.22	POLR2A	DNA-directed RNA polymerase II subunit RPB1	XM_005156225.5
B17G0162	1.75	1.96	ROHU_025877	A0A498ME30_LABRO Calcium-responsive transcription factor isoform X1	
A1G0699	1.75	5.84	LOC113087903	A0A6P6NR94_CARAU uncharacterized protein LOC113087903	XM_068217696.1
A4G0468	1.75	10.44	CPNE8	Copine-8	XM_005164936.5
B8G0979	1.75	1.80	CTU1	Cytoplasmic tRNA 2-thiolation protein 1	NM_200130.1
A14G0009	1.75	1.36	HOX71	Homeobox protein XHOX-7.1 (Fragment)	NM_131273.1
B17G0692	1.75	0.84	GPR132	Probable G-protein coupled receptor 132	XM_021467526.2
B3G0930	1.75	2.48	G5714_003616	A0A7J6DA00_9TELE Uncharacterized protein	XM_021474111.2
A1G0223	1.75	2.52	TAF5L	CBP-associated factor-associated factor 65 kDa subunit 5L	NM_199852.1
B2G0001	1.75	8.19	PDE4DIP	Myomegalin	XM_068215035.1
A21G0017	1.75	5.51	ZER1	Protein zer-1 homolog	NM_212901.1
A20G0074	1.75	3.32	MIA3	Transport and Golgi organization protein 1 homolog	XM_005160894.5
A25G0773	1.75	2.04	INSC	Protein inscuteable homolog	XM_068217723.1
A16G0449	1.75	5.35	PDIA3	Protein disulfide-isomerase A3	NM_212905.1
A21G0079	1.75	2.22	TOR1B	Torsin-1B	NM_001200015.1
B6G0013	1.75	0.71	S1PR1	Sphingosine 1-phosphate receptor 1	
A10G0892	1.75	3.39	NLRC3	Protein NLRC3	XM_068222163.1
A1G0330	1.75	2.27	ITPRIP	Inositol 1,4,5-trisphosphate receptor-interacting protein	XM_001334343.8
B6G0461	1.75	2.23	LRRC8C	Volume-regulated anion channel subunit LRRC8C	NM_001326544.1

B6G0994	1.75	8.69	ZNFX1	NFX1-type zinc finger-containing protein 1	XM_021477327.2
B13G0718	1.75	1.75	SYT16	Synaptotagmin-16	NM_001144794.1
B7G0513	1.75	1.73	GAREM1	GRB2-associated and regulator of MAPK protein 1	NM_200169.2
A21G0011	1.75	7.55	STARD4	StAR-related lipid transfer protein 4	NM_001017679.1
B21G0665	1.75	7.10	ULK1	threonine-protein kinase ULK1	XM_002665925.7
A25G0614	1.75	9.28	IRF7	Interferon regulatory factor 7	NM_200677.2
A21G0618	1.75	1.15	FOXI1	Forkhead box protein I1	NM_198917.2
A5G0094	1.75	1.31	TNC	Tenascin	XM_009301159.4
B3G0770	1.75	7.90	IGHV3-33	Immunoglobulin heavy variable 3-33	
A4G0389	1.75	2.45	NLRC3	Protein NLRC3	XM_068220884.1
B12G0520	1.75	1.73	MINPP1	Multiple inositol polyphosphate phosphatase 1	NM_201100.1
B24G0066	1.75	3.47	HARBI1	Putative nuclease HARBI1	
B18G0212	1.75	4.07	RTASE	Probable RNA-directed DNA polymerase from transposon BS	
A2G1116	1.75	21.32	TLE5	TLE family member 5	NM_001312685.1
A24G0189	1.75	5.70	ATG9A	Autophagy-related protein 9A	NM_001320078.1
B12G0098	1.75	6.94	WBP2	WW domain-binding protein 2	NM_001020544.2
A8G0473	1.75	1.60	ZNF335	Zinc finger protein 335	XM_005167099.5
B16G0777	1.75	0.66	CXCR3	C-X-C chemokine receptor type 3	
B6G0894	1.75	8.35	OSGIN2	Oxidative stress-induced growth inhibitor 2	NM_200186.1
A7G0675	1.75	3.99	SMAD3	Mothers against decapentaplegic homolog 3	NM_131571.2
B3G1247	1.75	5.80	HDAC5	Histone deacetylase 5	XM_680567.10
A5G1224	1.75	2.78	MED13L	Mediator of RNA polymerase II transcription subunit 13-like	NM_001083838.2
B11G0701	1.75	0.59	ARF4	ADP-ribosylation factor 4	
A20G0504	1.75	3.38			XM_005160568.5
B7G1230	1.75	7.28	LOC113105865	A0A6P6PQG9_CARAU zinc finger protein 883-like	
B20G0133	1.75	3.03	LOC113121234	A0A6P6RQP4_CARAU uncharacterized protein LOC113121234 isoform X1	NM_001190382.2
A8G0033	1.75	5.94	MFSD14A	Hippocampus abundant transcript 1 protein	XM_003199034.6
A9G0672	1.75	11.13	COL4A1	Collagen alpha-1(IV) chain	XM_688948.10
A9G0358	1.75	5.09	MORC3	MORC family CW-type zinc finger protein 3	NM_001089366.1
B12G0506	1.75	5.20	TECPR1	Tectonin beta-propeller repeat-containing protein 1	XM_009306680.4
B23G0214	1.75	14.86	NDRG3	Protein NDRG3	NM_199797.1
B5G0719	1.75	11.58	ZFR	Zinc finger RNA-binding protein	NM_199558.2
B13G0218	1.74	3.14	VWC2	Brorin	XM_021471168.2
A5G0030	1.74	2.08	LOC107574520	A0A672LAZ5_SINGR Uncharacterized LOC107574520	
B25G0506	1.74	5.21	ARNTL	Aryl hydrocarbon receptor nuclear translocator-like protein 1	XM_005163022.5
A4G0873	1.74	1.40	UTP20	Small subunit processome component 20 homolog	XM_009300013.4
A3G1102	1.74	2.63	TBC1D16	TBC1 domain family member 16	NM_001006076.2
A11G0065	1.74	0.39	G5714_012129	A0A7J6CMH1_9TELE Uncharacterized protein	XM_003199409.6
A7G0646	1.74	2.61	ZDHHC1	Palmitoyltransferase ZDHHC1	NM_001423785.1
B25G0713	1.74	8.69	DGKZ	Diacylglycerol kinase zeta	XM_005174290.5

B2G1271	1.74	1.44	LOC113046181	A0A6P6JST0_CARAU gastrula zinc finger protein XlCGF8.2DB-like	XM_009300998.4
A9G0722	1.74	3.63	SI:DKEY-18L1.1	von Willebrand factor A domain-containing protein 8	NM_001128338.1
A9G0415	1.74	2.21	PLEKHM3	Pleckstrin homology domain-containing family M member 3	XM_021474190.2
B7G1069	1.74	3.87	AXL	Tyrosine-protein kinase receptor UFO	XM_009303014.4
B24G0240	1.74	7.53	RALYL	RNA-binding Raly-like protein	XM_009297569.4
B14G0166	1.74	2.63	CNOT7	CCR4-NOT transcription complex subunit 7	NM_001077255.1
B5G0654	1.74	0.58	DMRT2	Doublesex- and mab-3-related transcription factor 2	NM_130952.1
A13G0764	1.74	2.20	TIMM23	Mitochondrial import inner membrane translocase subunit Tim23	XM_009307544.4
B1G1077	1.74	3.50	PIGR	Polymeric immunoglobulin receptor	XM_021475896.2
A7G0785	1.74	3.19	GFOD2	Glucose-fructose oxidoreductase domain-containing protein 2	NM_001077746.2
B7G1150	1.74	4.33	TNFSF12	Tumor necrosis factor ligand superfamily member 12	NM_001076607.1
A7G1084	1.74	0.58	KVM5	Ig kappa chain V region Mem5 (Fragment)	NM_131649.2
B6G0365	1.74	6.37	SETD5	Histone-lysine N-methyltransferase SETD5	XM_693742.9
B6G0414	1.74	12.18	LOC107710016	A0A673N461_9TELE Uncharacterized LOC107710016	NM_001083876.1
B18G0588	1.74	4.30	NUDT4B	Diphosphoinositol polyphosphate phosphohydrolase NUDT4B	NM_001310061.1
B22G0196	1.74	1.71	GGH	Gamma-glutamyl hydrolase	NM_001111175.1
A8G0548	1.74	22.87	LOC113093952	A0A6P6P2X0_CARAU endochitinase A-like isoform X2	
A9G0670	1.74	5.46	IRS2	Insulin receptor substrate 2	XM_695654.10
B3G0501	1.74	32.92	HBB	Hemoglobin cathodic subunit beta	
A15G0710	1.74	1.18	RCSD1	CapZ-interacting protein	
B17G0232	1.74	2.90	MIA2	Melanoma inhibitory activity protein 2	XM_021467325.2
A24G0657	1.74	0.03	F13A1	Coagulation factor XIII A chain	XM_681557.9
B23G0426	1.74	3.81	VPS13D	Intermembrane lipid transfer protein VPS13D	XM_021469946.2
B23G0584	1.74	3.66	RBP7	Retinoid-binding protein 7	
B22G0267	1.74	5.72	LOC113040261	A0A6P6J2U5_CARAU uncharacterized protein LOC113040261	XM_021470864.2
B3G0246	1.74	4.24	EVPL	Envoplakin	XM_001922038.7
B20G0425	1.74	0.68	STX11	Syntaxin-11	NM_200862.1
B3G0044	1.74	1.56	LOC113090245	A0A6P6NSY2_CARAU gastrula zinc finger protein XlCGF57.1-like	XM_068218685.1
B12G0103	1.74	2.55	DOCK1	Dedicator of cytokinesis protein 1	NM_001110011.1
A17G0092	1.74	0.68	KCNK5	Potassium channel subfamily K member 5	NM_001037401.2
A21G0295	1.74	1.78	LOC113064092	A0A6P6M106_CARAU uncharacterized protein LOC113064092	NM_001130782.1

B2G0842	1.74	4.44	CHD7	Chromodomain-helicase-DNA-binding protein 7	XM_692864.9
A5G0410	1.74	2.32	GAS2L2.S	GAS2-like protein 2B	XM_695912.10
A9G0411	1.74	5.98	KLF7	Krueppel-like factor 7	NM_001044766.2
A4G0601	1.74	4.96	DEF6	Differentially expressed in FDCP 6 homolog	XM_009300326.4
A17G0679	1.74	11.14	EMILIN1	EMILIN-1	NM_001045342.2
B4G0106	1.74	3.33	ATXN7L1	Ataxin-7-like protein 1	XM_003198215.6
A18G0404	1.74	2.25	CFDP1	Craniofacial development protein 1	NM_001100035.1
B7G0633	1.74	2.47	ZDHHC1	Palmitoyltransferase ZDHHC1	NM_001423785.1
B5G1206	1.74	4.77	GAB3	GRB2-associated-binding protein 3	XM_001337679.9
B3G0626	1.74	4.47	ARHGAP23	Rho GTPase-activating protein 23	XM_003198148.6
B3G0185	1.73	0.46			
A4G0494	1.73	2.71	VEZT	Vezatin	NM_001114575.2
B23G0243	1.73	3.03	RYBPA	RING1 and YY1-binding protein A	NM_201317.2
B3G1015	1.73	2.46	ATAD5	ATPase family AAA domain-containing protein 5	XM_003198109.5
B15G0115	1.73	7.06	PGM2L1	Glucose 1,6-bisphosphate synthase	NM_001098485.1
B14G0031	1.73	2.61	NUDT6	Nucleoside diphosphate-linked moiety X motif 6	NM_001089522.2
B11G0605	1.73	0.94	ZBTB40	Zinc finger and BTB domain-containing protein 40	NM_001040387.1
A13G0300	1.73	5.26	MATN3	Matrilin-3	NM_001017691.1
A3G0574	1.73	3.58	ABCC1	Multidrug resistance-associated protein 1	XM_001341859.8
A19G0064	1.73	10.19	H2-Q10	H-2 class I histocompatibility antigen, Q10 alpha chain	NM_001110118.1
B3G1461	1.73	0.45	G5714_002892	A0A7J6D7Y6_9TELE Uncharacterized protein	XM_021470951.2
A24G0569	1.73	1.46	RTASE	Probable RNA-directed DNA polymerase from transposon BS	
A3G0736	1.73	2.82	STRADA	STE20-related kinase adapter protein alpha	XM_021469637.2
A7G0609	1.73	3.83			
A3G0565	1.73	8.91	RETREG3	Reticulophagy regulator 3	XM_689279.9
A25G0070	1.73	37.20	FADS2	Acyl-CoA 6-desaturase	NM_131645.2
A3G0356	1.73	4.88	AATK	threonine-protein kinase LMTK1	XM_001344016.9
A1G0467	1.73	8.80	ATP2A2	endoplasmic reticulum calcium ATPase 2	XM_679135.9
B18G0752	1.73	1.61	CUZD1	CUB and zona pellucida-like domain-containing protein 1	
B8G0688	1.73	4.02	SEC22BB	Vesicle-trafficking protein SEC22b-B	NM_201302.2
A11G0116	1.73	1.24	DAZAP1	DAZ-associated protein 1	
B17G0641	1.73	2.31	MBOAT2	Lysophospholipid acyltransferase 2	XM_694203.7
B18G0775	1.73	1.17	INTS4	Integrator complex subunit 4	XM_017351982.3
A12G0280	1.73	4.39	ATRNL1	Attractin-like protein 1	XM_692563.10
B13G0271	1.73	0.65			XM_017358765.3
A6G0942	1.73	0.86	SPDEF	SAM pointed domain-containing Ets transcription factor	XM_001343041.6
B16G0994	1.73	1.82	ENTPD3	Ectonucleoside triphosphate diphosphohydrolase 3	NM_001083581.1
B14G0012	1.73	3.31	ZNF518B	Zinc finger protein 518B	XM_005173052.5

B7G0956	1.73	1.18	ADAMTS17	A disintegrin and metalloproteinase with thrombospondin motifs 17	XM_021477710.2
B12G0161	1.73	9.27	AATK	threonine-protein kinase LMTK1	XM_678332.10
A25G0125	1.73	1.53	SLC25A44	Solute carrier family 25 member 44	NM_001007350.2
A16G0075	1.73	0.27	SERPINB1	Leukocyte elastase inhibitor	NM_001109730.1
B7G1257	1.73	0.44	MRC2	C-type mannose receptor 2	NM_001328058.1
A12G0687	1.73	2.85	SLC16A9	Monocarboxylate transporter 9	NM_001003552.1
B21G0361	1.73	2.81	NRG2	Pro-neuregulin-2, membrane-bound isoform	NM_001099254.2
B9G0247	1.73	4.04	MCM3AP	Germinal-center associated nuclear protein	XM_068223663.1
A7G0450	1.73	0.96	FJX1	Four-jointed box protein 1	XM_001337564.9
B16G0646	1.73	2.25	ZNF236	Zinc finger protein 236	XM_005158156.3
B21G0580	1.73	1.69	PRC1	Protein regulator of cytokinesis 1	NM_001002684.2
A1G1115	1.73	7.85	MXD4	Max dimerization protein 4	XM_682878.10
B4G0722	1.73	1.83	LOC113066586	A0A6P6MC70_CARAU zinc finger protein OZF-like	NM_001115109.1
A4G0497	1.73	6.41	NR2C1-A	Nuclear receptor subfamily 2 group C member 1-A	XM_005164817.5
B21G0767	1.73	3.40	F2R	Proteinase-activated receptor 1	XM_001339441.9
A4G0923	1.73	2.93	TBC1D30	TBC1 domain family member 30	XM_021475403.2
A21G0581	1.73	2.08	PCNX3	Pecanex-like protein 3	NM_001328187.1
A9G0069	1.73	12.10	PFKL	ATP-dependent 6-phosphofructokinase, liver type	XM_693543.9
A21G0479	1.73	4.75	CADM1	Cell adhesion molecule 1	NM_001113551.2
B17G0685	1.73	3.99	CCDC88C	Protein Daple	NM_001423870.1
B3G1159	1.73	8.12	SMURF2	E3 ubiquitin-protein ligase SMURF2	NM_001114426.1
A3G0746	1.72	2.28	KCNH6	Potassium voltage-gated channel subfamily H member 6	NM_212837.1
B20G0846	1.72	13.84	IPO13	Importin-13	XM_002665780.7
A23G0795	1.72	1.69	SCUBE3	Signal peptide, CUB and EGF-like domain-containing protein 3	XM_021469709.2
B17G0276	1.72	4.95	RRAGD	Ras-related GTP-binding protein D	NM_001099424.2
A16G0129	1.72	0.05	MYLK3	Myosin light chain kinase 3	
B3G0116	1.72	0.10	LOC113039826	A0A6P6J2C4_CARAU zinc finger protein 345-like	XM_068218685.1
B14G0024	1.72	6.40	TRIM16	Tripartite motif-containing protein 16	XM_681071.8
A22G0060	1.72	1.49	PPARD	Peroxisome proliferator-activated receptor delta	XM_068216510.1
A5G1131	1.72	6.81	FBXW5	WD repeat-containing protein 5	NM_213319.2
A16G0058	1.72	1.64	KLF10	Krueppel-like factor 10	NM_001386438.1
B3G0623	1.72	0.98	G5714_003393	A0A7J6DAD3_9TELE Uncharacterized protein	
B14G0795	1.72	1.95	YRDC	Threonylcarbamoyl-AMP synthase	XM_009307784.4
B18G0364	1.72	1.12	SIX4	Homeobox protein SIX4	NM_131720.2
A2G0382	1.72	2.68	ATR	threonine-protein kinase ATR	XM_691071.9
B18G0849	1.72	2.35	SLC27A2	Long-chain fatty acid transport protein 2	NM_001025299.1
B14G0212	1.72	13.50	ARHGEF6	Rho guanine nucleotide exchange factor 6	XM_005173222.5
A22G0682	1.72	2.73	B3GNT7	UDP-GlcNAc:betaGal beta-1,3-N-acetylglucosaminyltransferase 7	XM_009296510.4

B5G0480	1.72	3.22	AKAP10	A-kinase anchor protein 10, mitochondrial	XM_005165576.5
B14G0226	1.72	1.41	INPPL1B	Phosphatidylinositol 3,4,5-trisphosphate 5-phosphatase 2B	NM_001039135.2
B5G0585	1.72	10.09	ERAP2	Endoplasmic reticulum aminopeptidase 2	NM_001123052.1
A4G0924	1.72	11.94	GNS	N-acetylglucosamine-6-sulfatase	NM_001030208.1
B13G0671	1.72	3.56	HHLA2	HERV-H LTR-associating protein 2	NM_001328217.1
B16G0393	1.72	1.10	PIGV	GPI mannosyltransferase 2	NM_001200060.1
A23G0561	1.72	2.62	TMEM201	Transmembrane protein 201	NM_001110466.1
B7G0459	1.72	0.72	CCNE1	S-specific cyclin-E1	NM_130995.1
B6G0456	1.72	5.80	TGFBR3	Transforming growth factor beta receptor type 3	NM_001311172.2
A11G0474	1.72	4.27	MAL	Myelin and lymphocyte protein	XM_005169333.5
B6G0063	1.72	6.59	TPRA1	Transmembrane protein adipocyte-associated 1 homolog	NM_001030251.1
A11G0331	1.72	2.29	TRIB2	Tribbles homolog 2	XM_021479801.2
B5G0193	1.72	2.47	POLE	DNA polymerase epsilon catalytic subunit A	NM_001128523.1
A3G1304	1.72	1.44	LOC107675477	A0A671KBP6_9TELE Gastrula zinc finger protein XlCGF57.1-like	XM_009303226.4
A11G0510	1.72	3.27	COL6A1	Collagen alpha-1(VI) chain	NM_001312677.1
B7G0881	1.72	4.90	SHROOM4	Protein Shroom4	XM_068222593.1
B14G0240	1.72	6.02	DLG3	Disks large homolog 3	XM_009291192.4
A8G0582	1.72	1.07	CCSER1	Serine-rich coiled-coil domain-containing protein 1	XM_021478459.2
A21G0705	1.72	4.13	GRK6	G protein-coupled receptor kinase 6	XM_684691.9
A5G0895	1.71	5.99	THBS4B	Thrombospondin-4-B	NM_001114424.2
A16G0022	1.71	0.62			
A21G0780	1.71	2.84	PCYOX1L	Prenylcysteine oxidase-like	XM_009295681.4
A10G0071	1.71	0.73	GRK5	G protein-coupled receptor kinase 5	NM_001173503.1
A20G0636	1.71	0.88	NME6	Nucleoside diphosphate kinase 6	
B13G0701	1.71	3.84	MAP4K5	Mitogen-activated protein kinase kinase kinase kinase 5	NM_001014366.2
A13G0544	1.71	1.75	SPRN	Shadow of prion protein	NM_001128710.1
A17G0782	1.71	18.74	VSNL1	Visinin-like protein 1	NM_001327942.1
A19G0582	1.71	9.99	ATP9B	Probable phospholipid-transporting ATPase IIB	XM_068215165.1
A16G0959	1.71	2.64	ARHGEF2	Rho guanine nucleotide exchange factor 2	XM_684678.10
B3G0564	1.71	2.21	INTS1	Integrator complex subunit 1	XM_003198152.6
A4G1044	1.71	3.07	TINF2	TERF1-interacting nuclear factor 2	XM_021475187.2
B18G0155	1.71	1.75	VWA7	von Willebrand factor A domain-containing protein 7	XM_003200478.6
B3G1201	1.71	3.17	BAIAP2L2	Brain-specific angiogenesis inhibitor 1-associated protein 2-like protein 2	XM_021473970.2
B5G0885	1.71	38.53	IFITM3	Interferon-induced transmembrane protein 3	NM_001110287.1
A19G0591	1.71	6.35	PLEC	Plectin	NM_001100032.1
B14G0745	1.71	0.97	LTC4S	Leukotriene C4 synthase	XM_003199867.6
B2G0296	1.71	4.36	EFR3A	Protein EFR3 homolog A	NM_200827.1
B17G0512	1.71	0.87	E2F2	Transcription factor E2F2	XM_068214603.1
B9G0722	1.71	5.86	ING1	Inhibitor of growth protein 1	NM_001430945.1
B23G0610	1.71	6.22	OSBPL1A	Oxysterol-binding protein-related protein 1	XM_005162340.5
A7G1013	1.71	1.88	SNX15	Sorting nexin-15	NM_001111237.1

B3G1358	1.71	3.87	G5714_002996	A0A7J6D893_9TELE Uncharacterized protein	XM_009299865.4
B5G1157	1.71	2.38	CCHCR1	Coiled-coil alpha-helical rod protein 1	XM_690528.9
B20G0510	1.71	24.95	ID2	DNA-binding protein inhibitor ID-2	XM_005160630.3
B14G0397	1.71	3.65	INTS5	Integrator complex subunit 5	XM_687970.8
B16G0922	1.71	2.22	LAG3	Lymphocyte activation gene 3 protein	
B17G0140	1.71	1.22	ZBTB2	Zinc finger and BTB domain-containing protein 2	NM_200160.1
A20G0490	1.71	6.86	G5714_019663	A0A7J6BX15_9TELE Uncharacterized protein	NM_001386316.1
A7G1335	1.71	4.24	TCB1	Transposable element Tcb1 transposase	XM_021471168.2
A24G0467	1.71	3.31	KLHL15	Kelch-like protein 15	NM_001003580.2
A2G0912	1.71	10.30	KEAP1A	Kelch-like ECH-associated protein 1A	NM_182864.2
A17G0222	1.71	1.10	PLEKHH1	Pleckstrin homology domain-containing family H member 1	NM_001077519.2
B15G0555	1.71	2.14	PITPNM3	Membrane-associated phosphatidylinositol transfer protein 3	XM_005157661.5
A5G1220	1.71	4.34	LMAN2L	VIP36-like protein	NM_205725.2
A24G0479	1.71	2.45	CD226	CD226 antigen	NM_001080646.1
A2G0534	1.71	3.22	MSL2	E3 ubiquitin-protein ligase MSL2	NM_001163298.1
B17G0477	1.71	0.83	QRSL1	Glutamyl-tRNA(Gln) amidotransferase subunit A, mitochondrial	NM_001118898.1
B25G0861	1.71	3.44	MYO9AB	Unconventional myosin-IXAb	NM_001423784.1
B3G0678	1.70	4.88	SLC16A6	Monocarboxylate transporter 7	NM_001365238.1
B10G0224	1.70	42.91	NT5C2	Cytosolic purine 5'-nucleotidase	NM_001004549.2
B25G0692	1.70	2.85	POLG	DNA polymerase subunit gamma-1	XM_068217497.1
B7G0438	1.70	0.31	PRR5L	Proline-rich protein 5-like	XM_021477950.2
B25G0399	1.70	1.25	ALKBH3	Alpha-ketoglutarate-dependent dioxygenase alkB homolog 3	NM_001003511.2
B4G0020	1.70	2.06	MRAP2B	Melanocortin-2 receptor accessory protein 2B	
B19G0635	1.70	2.40	TRIB1	Tribbles homolog 1	XM_009294329.4
B24G0003	1.70	1.11	AHRR	Aryl hydrocarbon receptor repressor	NM_001035265.1
B12G0693	1.70	5.56	TBC1D12	TBC1 domain family member 12	XM_005169448.5
B12G0398	1.70	13.33	ZBTB4	Zinc finger and BTB domain-containing protein 4	XM_001339110.7
A13G0067	1.70	3.07	ITGA9	Integrin alpha-9	XR_011016140.1
A24G0332	1.70	3.85	MTMR6	Myotubularin-related protein 6	NM_199791.1
A23G0524	1.70	4.17	TRIO	Triple functional domain protein	XM_009296957.4
B21G0318	1.70	1.60	PGAP2	Post-GPI attachment to proteins factor 2	XM_005161301.5
B2G0582	1.70	1.12	DARS2	Aspartate--tRNA ligase, mitochondrial	XM_694712.9
A6G0014	1.70	4.66	FDX2	Ferredoxin-2, mitochondrial	
A6G0887	1.70	2.38	RBL1	Retinoblastoma-like protein 1	XM_021476946.2
A23G0744	1.70	0.02	TGM1	Protein-glutamine gamma-glutamyltransferase K	XM_009297092.4
B8G0694	1.70	2.30	MPEG1	Macrophage-expressed gene 1 protein	NM_001020586.2
A9G0450	1.70	0.41	LOC106524126	A0A2I4BZT1_9TELE formimidoyltransferase-cyclodeaminase-like isoform X2	NM_001076645.1

B11G0654	1.70	1.27	ZGC:112255	Uncharacterized protein C1orf50 homolog	
B21G0849	1.70	3.34	PHYHD1	Phytanoyl-CoA dioxygenase domain-containing protein 1	NM_001007445.1
B8G0178	1.70	9.01	GGH	Gamma-glutamyl hydrolase	NM_001256222.1
A9G0229	1.70	4.63	SEC22A	Vesicle-trafficking protein SEC22a	XM_021478609.2
A23G0883	1.70	4.17	CAMTA2	Calmodulin-binding transcription activator 2	XM_005170596.5
A5G0296	1.70	0.87	CMKLR1	Chemerin-like receptor 1	XM_005165199.5
A15G0771	1.70	5.91	RTASE	Probable RNA-directed DNA polymerase from transposon BS	
B16G0227	1.70	2.12	SEMA4A	Semaphorin-4A	XM_009292609.4
B19G0800	1.70	7.32	LOC113119229	A0A6P6REY2_CARAU uncharacterized protein LOC113119229	
A19G0474	1.70	1.89	TCB2	Transposable element Tcb2 transposase	XM_068220572.1
B16G0884	1.70	0.72	ABCB1	ATP-dependent translocase ABCB1	XM_001922682.8
A10G0304	1.70	1.83	NF1	Neurofibromin	XM_005169189.5
A24G0568	1.70	1.97	G5714_000125	A0A7J6DH20_9TELE Uncharacterized protein	
B5G0400	1.70	4.01	PRR14L	Protein PRR14L	XM_009301882.4
A7G1138	1.70	8.74	ABHD2A	Monoacylglycerol lipase ABHD2	NM_200914.1
B9G0470	1.70	0.76	LYPD6B	PLAUR domain-containing protein 6B	XM_068223388.1
A4G0998	1.70	3.69	SMP	Schwann cell myelin protein	XM_068219777.1
B13G0343	1.70	3.94	PRDX3	Thioredoxin-dependent peroxide reductase, mitochondrial	XM_005156949.5
A22G0018	1.70	9.45	CROCC	Rootletin	XM_068216596.1
A15G0628	1.70	4.24	DIABLO	Diablo IAP-binding mitochondrial protein	NM_200346.1
A7G1054	1.70	4.25	CORO1B	Coronin-1B	NM_001109707.1
B25G0267	1.70	1.17	SIGIRR	Single Ig IL-1-related receptor	NM_001098250.1
B7G0569	1.70	1.48	HARBI1	Putative nuclease HARBI1	NM_001003734.1
A18G0727	1.70	5.75	RSF1	Remodeling and spacing factor 1	XM_001921640.7
A25G0320	1.70	7.11	VAC14	Protein VAC14 homolog	NM_001004650.1
B24G0563	1.70	9.07	RDH10A	Retinol dehydrogenase 10-A	NM_001080583.2
B12G0665	1.70	5.81	SLC16A9	Monocarboxylate transporter 9	NM_001003552.1
B6G0686	1.70	5.10	PFKFB4	fructose-2,6-bisphosphatase 4	NM_001430986.1
B5G0612	1.70	2.82	ARSK	Arylsulfatase K	XM_068221239.1
B5G0791	1.70	1.60	CDC42EP2	Cdc42 effector protein 2	XM_005165306.5
A21G0566	1.70	9.64	MARK2	threonine-protein kinase MARK2	XM_009295510.4
B13G0748	1.70	2.04	MORN4	MORN repeat-containing protein 4	
B6G0187	1.70	2.54	USP40	Ubiquitin carboxyl-terminal hydrolase 40	XM_001921318.8
B17G0445	1.70	6.28	MTFR1L	Mitochondrial fission regulator 1-like	NM_199884.1
A17G0608	1.70	2.24	HMBOX1	Homeobox-containing protein 1	XM_005169831.5
B3G0498	1.70	2.76	MPG	DNA-3-methyladenine glycosylase	NM_001082850.2
A12G0340	1.70	8.95			NM_131709.1
B7G0574	1.70	1.18	ZNF408	Zinc finger protein 408	NM_001328275.1
B20G0137	1.69	2.24	SPRTN	DNA-dependent metalloprotease SPRTN	NM_001423865.1
B24G0709	1.69	13.74	CYB5A	Cytochrome b5	NM_213135.2
B11G0269	1.69	2.68	COL9A1	Collagen alpha-1(IX) chain	XM_021479797.2

A1G0097	1.69	0.57	CD300LF	CMRF35-like molecule 1	
B1G0713	1.69	0.32	G5714_000553	A0A7J6DGN6_9TELE Uncharacterized protein	NM_001145236.1
A20G0706	1.69	8.37	SUCO	SUN domain-containing ossification factor	XM_681409.10
A6G0999	1.69	6.15	ARHGAP9	Rho GTPase-activating protein 9	XM_005172127.5
A11G0427	1.69	1.81	DDB_G0282555	Beta-lactamase-like protein 3	XM_021472492.2
B15G0367	1.69	0.24	POU2AF2	POU class 2 homeobox associating-factor 2	XM_068213695.1
B2G0259	1.69	2.03	KLHL24	Kelch-like protein 24	NM_001077340.1
B22G0852	1.69	1.67	VASN	Vasorin	NM_001326685.1
B22G0692	1.69	4.19	MARCHF2	E3 ubiquitin-protein ligase MARCHF2	NM_001044790.2
B3G0042	1.69	1.03	LOC113097005	A0A6P6PBS8_CARAU zinc finger protein 845-like	XM_068218685.1
B18G0144	1.69	1.87	PGRP-SC2	Peptidoglycan-recognition protein SC2	
A7G0597	1.69	51.39	A0A5Q2U986	A0A5Q2U986_CYPCA Complement system-related protein CD59-2	NM_001326385.1
B2G0885	1.69	5.62	PLA2G4A	Cytosolic phospholipase A2	NM_131295.2
A6G0179	1.69	2.73	DZIP3	E3 ubiquitin-protein ligase DZIP3	
A20G0806	1.69	1.37	SPRTN	DNA-dependent metalloprotease SPRTN	NM_001423865.1
A2G0287	1.69	2.33	RTASE	Probable RNA-directed DNA polymerase from transposon BS	XM_068220297.1
A19G0525	1.69	0.55	CMTM8	CKLF-like MARVEL transmembrane domain-containing protein 8	XR_011007564.1
A7G0632	1.69	6.04	CHD9	Chromodomain-helicase-DNA-binding protein 9	XM_068218460.1
B6G0673	1.69	1.70	ESPL1	Separin	
B13G0663	1.69	2.57	MAP3K4	Mitogen-activated protein kinase kinase kinase 4	XM_005156608.5
A15G0580	1.69	2.51	TUBD1	Tubulin delta chain	NM_001002093.1
A19G0408	1.69	1.71	LOC107696596	A0A671P070_9TELE Uncharacterized LOC107696596	NM_001430907.1
B13G0641	1.69	0.24	MACROD2	ADP-ribose glycohydrolase MACROD2	
A4G0874	1.69	2.72	UTP20	Small subunit processome component 20 homolog	XM_009300013.4
B22G0289	1.69	2.52	CD244	Natural killer cell receptor 2B4	XM_068216416.1
A21G0831	1.69	2.43	TMLHE	Trimethyllysine dioxygenase, mitochondrial	XM_677739.7
A20G0905	1.69	2.52	AKAP7	A-kinase anchor protein 7 isoform gamma	
A21G0807	1.69	1.37	GPR34	Probable G-protein coupled receptor 34	
B9G0241	1.69	3.06	ZNF148	Zinc finger protein 148	NM_001080180.2
A8G0860	1.69	20.03	EGR3	Early growth response protein 3	NM_001002647.1
A25G0524	1.69	0.54	NRG4	Pro-neuregulin-4, membrane-bound isoform	
A22G0469	1.69	2.36	SHC2	SHC-transforming protein 2	XM_017353158.3
A10G0645	1.69	0.25	CXADR	Coxsackievirus and adenovirus receptor homolog	
A2G0979	1.69	2.56	P4HA1	Prolyl 4-hydroxylase subunit alpha-1	XM_021471285.2
B20G0951	1.69	2.67	MGA	MAX gene-associated protein	XM_068215584.1
A5G1027	1.69	1.13	TMEM132E	Transmembrane protein 132E	NM_001302234.1

A2G0507	1.69	2.78	NR2F6	Nuclear receptor subfamily 2 group F member 6	NM_205557.1
A10G0322	1.69	4.95	NIPBLB	Nipped-B-like protein B	NM_001161447.2
A14G0077	1.69	6.28	MED12	Mediator of RNA polymerase II transcription subunit 12	XM_009291037.4
B15G0902	1.69	2.02	SLC12A9	Solute carrier family 12 member 9	NM_001430896.1
A22G0525	1.69	6.59	CLCC1	Chloride channel CLIC-like protein 1	NM_001423847.1
B7G0380	1.69	3.73	CITED1	p300-interacting transactivator 1	
B2G0010	1.69	14.15	TRIM16	Tripartite motif-containing protein 16	XM_681071.8
A10G0581	1.69	4.52			
A23G0223	1.69	2.45	PLXNB1	Plexin-B1	XM_001920948.8
A22G0703	1.69	5.01	ACY1A	Aminoacylase-1A	NM_001272080.1
B25G0754	1.69	1.32	SI:DKEY-261E22.4	Overexpressed in colon carcinoma 1 protein homolog	NM_001302783.1
A10G0684	1.69	11.31	PCYOX1	Prenylcysteine oxidase 1	NM_001017626.1
B5G0600	1.68	1.65	MSH3	DNA mismatch repair protein Msh3	XM_009301782.4
A11G0306	1.68	1.88	MDFI	MyoD family inhibitor	XM_005155820.5
A25G0809	1.68	6.23	ADAM10	Disintegrin and metalloproteinase domain-containing protein 10	NM_200420.1
B3G1529	1.68	1.44	A0A673M8N8	A0A673M8N8_9TELE Uncharacterized protein	XM_068220660.1
B7G0752	1.68	9.47	VPS13C	Intermembrane lipid transfer protein VPS13C	XM_021471934.2
A7G1108	1.68	2.33	MPPED2	Metallophosphoesterase MPPED2	NM_001017735.2
B15G0811	1.68	1.22	LOC113079229	A0A6P6NE16_CARAU gastrula zinc finger protein XlCGF7.1-like	XM_003198534.6
B20G0261	1.68	3.20	MEP1B	Meprin A subunit beta	NM_001076621.2
B14G0090	1.68	3.57	SLIT3	Slit homolog 3 protein	NM_131736.3
A5G0840	1.68	1.43	POLK	DNA polymerase kappa	XM_068221512.1
B23G0113	1.68	8.46	UTRN	Utrophin	XM_021469867.2
A9G0629	1.68	3.37	NRP2	Neuropilin-2	NM_212966.1
A17G0466	1.68	3.51	ZMPSTE24	CAAX prenyl protease 1 homolog	NM_199892.1
A2G0191	1.68	2.07	RIPK2	threonine-protein kinase 2	NM_194411.2
B22G1015	1.68	7.24			
B24G0134	1.68	6.82	IGKV1-39	Immunoglobulin kappa variable 1-39	NM_001045408.1
A20G0760	1.68	1.03	ZFY2	Zinc finger Y-chromosomal protein 2	XM_005160411.4
A8G0827	1.68	5.06	UBE2D4	Ubiquitin-conjugating enzyme E2 D4	NM_001089453.1
B3G0892	1.68	2.26	ALDH16A1	Aldehyde dehydrogenase family 16 member A1	NM_001079954.2
A6G0545	1.68	11.59	PDE4B	cAMP-specific 3',5'-cyclic phosphodiesterase 4B	XM_068218329.1
B10G0134	1.68	3.44	CDC37L1	Hsp90 co-chaperone Cdc37-like 1	NM_001109826.2
A14G0445	1.68	2.63	CLP1	Polyribonucleotide 5'-hydroxyl-kinase Clp1	NM_001346238.1
B5G0682	1.68	1.56	CXCL11.6	C-X-C motif chemokine 11-6	
A1G0198	1.68	14.24	PGAM1	Phosphoglycerate mutase 1	NM_201300.2
B3G1355	1.68	7.02	SLC9A3R1	H(+) exchange regulatory cofactor NHE-RF1	XM_005174539.5
A16G0406	1.68	1.34	GSTK1	Glutathione S-transferase kappa 1	NM_001040300.1

A14G0468	1.68	8.63	RNF44	RING finger protein 44	NM_001076624.1
B13G0662	1.68	3.33	MAP3K4	Mitogen-activated protein kinase kinase kinase 4	NM_212946.1
A21G0819	1.68	0.24	PLSCR2	Phospholipid scramblase 2	XM_017355832.3
A12G0218	1.68	14.11	FASN	Fatty acid synthase	XM_009306807.4
A22G0348	1.68	8.01	PLEKHB2	Pleckstrin homology domain-containing family B member 2	NM_201002.1
A17G0405	1.68	2.48	FAM149B1	Protein FAM149B1	XM_005158524.5
A8G0489	1.68	1.09	ATXN7L2	Ataxin-7-like protein 2	NM_001002655.2
B18G0279	1.68	3.76	POL	RNA-directed DNA polymerase from mobile element jockey	XM_021472984.2
A14G0807	1.68	5.90	ST3GAL5	Lactosylceramide alpha-2,3-sialyltransferase	NM_199516.2
B18G0338	1.68	8.00	ITPKC	Inositol-trisphosphate 3-kinase C	NM_001305471.1
A6G0314	1.68	3.92	AGAP1	Arf-GAP with GTPase, ANK repeat and PH domain-containing protein 1	XM_021471740.2
B16G0052	1.68	3.98	ZNRF2	E3 ubiquitin-protein ligase znrf2	NM_001076728.1
B4G0552	1.68	4.71	KDM7A	Lysine-specific demethylase 7A	NM_001346151.1
A13G0016	1.68	1.00	LRRC27	Leucine-rich repeat-containing protein 27	NM_001386434.1
B24G0073	1.68	5.23	IGKV4-1	Immunoglobulin kappa variable 4-1	NM_001045408.1
B22G1036	1.68	1.37	E1301_TTI019070	A0A5A9NQU6_9TELE Uncharacterized protein	XR_011010925.1
A4G0683	1.68	9.10	UBE2H	Ubiquitin-conjugating enzyme E2 H	NM_201489.1
B4G0316	1.68	7.73	BRAF	threonine-protein kinase B-raf	NM_205744.4
B25G0348	1.68	11.14	EFCAB10	EF-hand calcium-binding domain-containing protein 10	XM_001332830.9
B12G0713	1.68	6.24	KANSL1	KAT8 regulatory NSL complex subunit 1	
B24G0519	1.67	1.11	SKIDA1	DACH domain-containing protein 1	XM_001342808.8
B3G0160	1.67	0.71	LOC113096987	A0A6P6P9V7_CARAU uncharacterized protein LOC113096987	
B16G0202	1.67	0.16	FAM83A	Protein FAM83A	XM_001340240.8
A25G0143	1.67	3.18	PTDSS2	Phosphatidylserine synthase 2	NM_001423812.1
B7G1215	1.67	8.18	CLCN3	Cl(-) exchange transporter 3	XM_003198891.6
B16G0852	1.67	9.12	SNTB1	Beta-1-syntrophin	NM_001002175.1
A15G0030	1.67	12.13	ARAP1	Arf-GAP with Rho-GAP domain, ANK repeat and PH domain-containing protein 1	XM_021466541.2
B24G0715	1.67	2.47	LY86	Lymphocyte antigen 86	XM_009297218.4
B17G0890	1.67	1.88	DDHD1	Phospholipase DDHD1	XM_009293324.4
A18G0615	1.67	2.90	TMTC2	Protein O-mannosyl-transferase TMTC2	XM_068214883.1
B24G0282	1.67	1.97	FRRS1	Ferric-chelate reductase 1	XM_017353998.3
B10G0555	1.67	3.34	PHLDB2	Pleckstrin homology-like domain family B member 2	XM_021479426.2
A7G0292	1.67	3.25	SLC22A23	Solute carrier family 22 member 23	XM_694062.10
A8G0086	1.67	1.46	NAIF1	Nuclear apoptosis-inducing factor 1	XM_009303707.4
A7G0839	1.67	1.35	TRAPPC14	Trafficking protein particle complex subunit 14	NM_001423811.1
A15G0861	1.67	2.19	P2RY12	P2Y purinoceptor 12	
B5G0504	1.67	2.64	CNTRL	Centriolin	XM_068221551.1
A14G0024	1.67	2.20	AURKB	Aurora kinase B	NM_212566.2

A19G0089	1.67	4.00	TENT5A	Terminal nucleotidyltransferase 5A	NM_001045170.2
B20G0220	1.67	1.23	G5714_019436	A0A7J6BWG1_9TELE Uncharacterized protein	NM_001025491.1
B2G0839	1.67	6.05	RALA	Ras-related protein Ral-A	NM_001007321.1
A4G0973	1.67	3.40	FAR1	Fatty acyl-CoA reductase 1	NM_001083089.1
B1G0676	1.67	7.11	SSRP1	FACT complex subunit SSRP1	XM_068221595.1
B19G0737	1.67	6.38	MGAT1	Alpha-1,3-mannosyl-glycoprotein 2-beta-N-acetylglucosaminyltransferase	NM_001079971.2
B15G0033	1.67	1.72	IGSF10	Immunoglobulin superfamily member 10	XM_009291391.4
B7G0787	1.67	2.25	CCDC102A	Coiled-coil domain-containing protein 102A	NM_205552.1
B3G1085	1.67	5.23	G5714_003759	A0A7J6DAJ2_9TELE Uncharacterized protein	NM_199815.1
A13G0411	1.67	3.25	B3GAT2	Galactosylgalactosylxylosylprotein 3-beta-glucuronosyltransferase 2	NM_001002523.3
B14G0155	1.67	4.75	SORBS1	Sorbin and SH3 domain-containing protein 1	XM_021472816.2
B24G0729	1.67	2.09	PIEZO2	Piezo-type mechanosensitive ion channel component 2	XM_021470255.2
A4G0530	1.67	5.96	KDM7A	Lysine-specific demethylase 7A	NM_001346151.1
B21G0436	1.67	0.37	OVOL2	Transcription factor Ovo-like 2	XM_003200839.6
B4G0245	1.67	0.34	TMPRSS9	Transmembrane protease serine 9	NM_200145.2
B15G0299	1.67	2.22	KBTBD8	Kelch repeat and BTB domain-containing protein 8	NM_001079948.2
B5G1015	1.67	4.27	TNC	Tenascin	NM_001312916.1
B19G0899	1.67	4.00	ROHU_022315	A0A498N5C3_LABRO Uncharacterized protein	
B7G0665	1.67	6.82	MAP2K5	Dual specificity mitogen-activated protein kinase kinase 5	NM_001114317.1
B13G0788	1.67	5.27	DPX16_10015	A0A3N0XWX6_ANAGA AN1-type zinc finger protein 3 (Fragment)	NM_213234.1
B13G0270	1.67	2.33	EMX1	Homeobox protein EMX1	XM_001339704.7
B21G1008	1.67	2.08	AFF4	FMR2 family member 4	
A7G0061	1.67	3.91	TRIM25	ISG15 ligase TRIM25	NM_001002723.2
B9G0412	1.67	1.38	EEF1AKMT1	EEF1A lysine methyltransferase 1	NM_207087.1
A7G0713	1.67	10.69	NAP1L4	Nucleosome assembly protein 1-like 4	XM_068222285.1
B4G0138	1.67	1.92	ZSCAN29	Zinc finger and SCAN domain-containing protein 29	NM_001013331.3
A2G1008	1.67	0.32	CCL12	C-C motif chemokine 12	
B17G0646	1.67	7.45	ADAM17	Disintegrin and metalloproteinase domain-containing protein 17	XM_005158718.5
A22G0589	1.67	0.19	CD276	CD276 antigen homolog	
A5G0755	1.67	2.84	CDS1	Phosphatidate cytidylyltransferase 1	XM_001341863.8
A10G0376	1.66	1.41	PRR11	Proline-rich protein 11	XM_690659.9
A5G0820	1.66	4.10	DAB2	Disabled homolog 2	XM_021471673.2
B10G0414	1.66	4.03	VPS51	Vacuolar protein sorting-associated protein 51 homolog	NM_001042735.1
B19G0488	1.66	0.62	CD164L2	CD164 sialomucin-like 2 protein	XM_005159772.5
B16G0765	1.66	2.87	ZFY1	Zinc finger Y-chromosomal protein 1	NM_001170836.1

A18G0466	1.66	4.16	SMAD6	Mothers against decapentaplegic homolog 6	NM_001045051.2
A16G0825	1.66	1.82			
B17G0228	1.66	2.64	PRORP	Mitochondrial ribonuclease P catalytic subunit	XM_678197.9
B1G1157	1.66	5.29	LOC113099797	A0A6P6PII8_CARAU zinc finger protein 709-like	XM_017357165.3
B20G0087	1.66	8.11	LAMA4	Laminin subunit alpha-4	NM_001039065.2
A19G0477	1.66	0.94	TRPS1	Zinc finger transcription factor Trps1	XM_009294450.4
A20G0599	1.66	6.40	KDR	Vascular endothelial growth factor receptor 2	NM_001024653.2
A1G0523	1.66	3.29	STOX2	Storkhead-box protein 2	XM_001922863.8
A17G0179	1.66	4.29	TMED8	Protein TMED8	XM_017351636.3
A5G0215	1.66	1.75	CDCA2	Cell division cycle-associated protein 2	XM_005168613.5
B13G0764	1.66	2.72	CPEB3	Cytoplasmic polyadenylation element-binding protein 3	XM_003199688.5
A8G0409	1.66	1.51	MAD2L2	Mitotic spindle assembly checkpoint protein MAD2B	NM_001017595.1
B5G1204	1.66	1.87	AMER1	APC membrane recruitment protein 1	NM_001346277.1
B25G0281	1.66	9.65	SCAMP5	Secretory carrier-associated membrane protein 5	NM_205547.1
B10G0225	1.66	1.80	SKA1	Spindle and kinetochore-associated protein 1	NM_001002592.1
A3G0269	1.66	5.80	NFIL3	Nuclear factor interleukin-3-regulated protein	NM_001197065.1
B8G0990	1.66	1.59	NAIF1	Nuclear apoptosis-inducing factor 1	XM_009303707.4
A3G0630	1.66	0.48	DUSP3	Dual specificity protein phosphatase 3	NM_001044307.1
A6G0901	1.66	17.59	ESYT1	Extended synaptotagmin-1	XM_694639.10
B1G0862	1.66	2.69	KIF16B	Kinesin-like protein KIF16B	XM_009294326.4
B16G0423	1.66	2.94	FBXL4	LRR-repeat protein 4	NM_001007315.2
A1G0666	1.66	2.64	RTASE	Probable RNA-directed DNA polymerase from transposon BS	
A15G0556	1.66	1.40	ARHGAP32	Rho GTPase-activating protein 32	XM_685829.10
B19G0799	1.66	10.35	JOCKEY\POL	RNA-directed DNA polymerase from mobile element jockey	XR_011011165.1
A5G1139	1.66	2.36	ARHGEF15	Rho guanine nucleotide exchange factor 15	XM_017356341.3
A21G0717	1.66	1.86	SIAH2L	E3 ubiquitin-protein ligase Siah2	NM_200427.2
A12G0628	1.66	1.85	PAAT	ATPase PAAT	XM_694146.9
B3G0266	1.66	6.23	TMEM100	Transmembrane protein 100	
B5G0831	1.66	2.73	TAF1	Transcription initiation factor TFIID subunit 1	NM_001044785.1
B10G0610	1.66	1.84	AKAP1	A-kinase anchor protein 1, mitochondrial	XM_021472362.2
B5G1199	1.66	2.97	ZC3H12B	Probable ribonuclease ZC3H12B	XM_021471586.2
A13G0819	1.66	6.75	WDR11	WD repeat-containing protein 11	NM_001030177.1
A22G0483	1.66	0.65	C2CD4C	C2 calcium-dependent domain-containing protein 4C	XM_005170537.5
A15G0071	1.66	2.26	AASDHPPT	L-aminoadipate-semialdehyde dehydrogenase-phosphopantetheinyl transferase	NM_001033729.1
B9G0740	1.66	4.61			
A5G0876	1.66	1.49	ARSK	Arylsulfatase K	XM_068221239.1

B7G1054	1.66	0.54	IGK	Immunoglobulin kappa light chain	
A21G0212	1.66	2.73	BDP1	Transcription factor TFIIIB component B" homolog	NM_001430814.1
A13G0433	1.66	2.32	NEUROG3	Neurogenin-3	NM_212794.1
A10G0745	1.66	2.12	CERCAM	Probable inactive glycosyltransferase 25 family member 3	XM_009305170.4
B4G0517	1.66	3.40	LRRC17	Leucine-rich repeat-containing protein 17	NM_205539.1
B15G0624	1.66	3.37	NF1	Neurofibromin	XM_009291769.4
B14G0029	1.66	2.65	TRIM25	ISG15 ligase TRIM25	XM_681071.8
A3G0332	1.66	1.89	QTRT1	Queuine tRNA-ribosyltransferase catalytic subunit 1	NM_201010.1
B9G0601	1.65	2.17	PIGA	Phosphatidylinositol N-acetylglucosaminyltransferase subunit A	NM_001126446.1
A1G0047	1.65	0.90	LOC113099797	A0A6P6PII8_CARAU zinc finger protein 709-like	XM_017357165.3
B15G0710	1.65	0.45	A0A672KZL4	A0A672KZL4_SINGR Lymphotoxin alpha (TNF superfamily, member 1)	NM_001024821.1
B3G1454	1.65	2.77	G5714_002892	A0A7J6D7Y6_9TELE Uncharacterized protein	XM_021471347.2
B1G0623	1.65	1.97	G5714_000629	A0A7J6DGV8_9TELE C3H1-type domain-containing protein	XM_021471222.2
B18G0824	1.65	0.22	CCBE1	Collagen and calcium-binding EGF domain-containing protein 1	
B23G0064	1.65	1.44	LOC113046763	A0A6P6JUE8_CARAU gastrula zinc finger protein XlCGF57.1-like isoform X1	XM_068220671.1
B2G0070	1.65	2.47	LOC113045253	A0A6P6JPU2_CARAU uncharacterized protein LOC113045253	XM_005163642.5
B6G0260	1.65	3.45	WDSUB1	WD repeat, SAM and U-box domain-containing protein 1	NM_001077769.1
B7G0671	1.65	0.29	PAQR5B	Membrane progestin receptor gamma-B	NM_001003573.2
B4G0118	1.65	3.32	SLC13A4	Solute carrier family 13 member 4	XM_005164503.5
B20G0178	1.65	2.18	PTGER2	Prostaglandin E2 receptor EP2 subtype	
B23G0047	1.65	0.99	WNT7A	Protein Wnt-7a	XM_691422.7
B9G0639	1.65	4.80	EHHADH	Peroxisomal bifunctional enzyme	NM_207068.1
B3G1525	1.65	1.90	LOC113039841	A0A6P6J106_CARAU gastrula zinc finger protein XlCGF7.1-like	NM_001114486.1
B16G0428	1.65	0.91	CALHM6	Calcium homeostasis modulator protein 6	NM_001079661.1
B11G0245	1.65	2.34	ADORA1	Adenosine receptor A1	XM_683725.10
B4G0817	1.65	5.76			
B14G0140	1.65	17.37	ACSL4	Long-chain-fatty-acid--CoA ligase 4	NM_200649.1
A23G0151	1.65	0.88	BMP7	Bone morphogenetic protein 7	NM_001077146.2
A4G0423	1.65	1.12	DPX16_1179	A0A3N0XUS6_ANAGA Zinc finger protein 569	XM_021475732.2
B21G0792	1.65	2.26	CENPT	Centromere protein T	XM_005161005.5
B18G0659	1.65	16.07	MICAL3A	Protein-methionine sulfoxide oxidase mical3a	XM_021473237.1

B2G0025	1.65	8.43	KCNJ10	ATP-sensitive inward rectifier potassium channel 10	XM_009298954.4
B25G0084	1.65	2.13	ANO9	Anoctamin-9	XM_001921933.8
A3G0951	1.65	15.57	VAT1	Synaptic vesicle membrane protein VAT-1 homolog	NM_001007051.3
A3G0520	1.65	1.75	B9D1	B9 domain-containing protein 1	NM_001024373.1
A7G0967	1.65	2.91	POP7	Ribonuclease P protein subunit p20	NM_001006004.1
A2G0508	1.65	2.65	OCLN	Occludin	NM_001080004.1
B2G1126	1.65	6.12	ALDH9A1B	4-trimethylaminobutyraldehyde dehydrogenase B	XM_005163231.5
A20G0502	1.65	5.31	SERTAD2	SERTA domain-containing protein 2	NM_001075116.2
B16G1042	1.65	3.83	ARHGEF2	Rho guanine nucleotide exchange factor 2	XM_684678.10
B4G0813	1.65	1.38	A0A671LA65	A0A671LA65_9TELE Uncharacterized protein	XM_068220406.1
B13G0660	1.65	0.65	ERLEC1	Endoplasmic reticulum lectin 1	
A24G0445	1.65	2.72	PREX2	Phosphatidylinositol 3,4,5-trisphosphate-dependent Rac exchanger 2 protein	XM_001923322.6
B5G0872	1.65	2.61	MS4A4A	Membrane-spanning 4-domains subfamily A member 4A	
B25G0377	1.65	1.13	ETFBKMT	Electron transfer flavoprotein beta subunit lysine methyltransferase	
B15G0823	1.65	0.13	ROHU_029833	A0A498LYM0_LABRO Paired box Pax-3 isoform X3	
B16G1070	1.65	1.44	HACE1	E3 ubiquitin-protein ligase HACE1	NM_001423976.1
B24G0587	1.65	1.15	GPR17	cysteinyl leukotriene receptor	XM_021470286.2
B23G0595	1.65	7.38	DVL1	Segment polarity protein dishevelled homolog DVL-1	XM_017353751.3
B5G0054	1.65	3.11	DHX33	ATP-dependent RNA helicase DHX33	NM_001100057.2
A1G0723	1.65	4.00	TXNL4B	Thioredoxin-like protein 4B	NM_001290146.1
B25G0568	1.65	0.88	OLFML1	Olfactomedin-like protein 1	XM_001342657.8
A22G0197	1.65	2.05	G5714_021483	A0A7J6BT36_9TELE Uncharacterized protein	XM_021469564.2
A20G0616	1.65	60.39	CKB	Creatine kinase B-type	NM_173222.2
B4G0853	1.65	1.58	A0A672MEL5	A0A672MEL5_SINGR Uncharacterized protein	XM_017355341.3
B23G0890	1.65	4.01	SCML2	Sex comb on midleg-like protein 2	XM_002666560.7
B7G0230	1.65	2.36	MATCAP	Microtubule-associated tyrosine carboxypeptidase	XM_680865.9
B16G0531	1.65	9.87	SYT11	Synaptotagmin-11	NM_001162531.1
A12G0318	1.65	0.88	DHX8	ATP-dependent RNA helicase DHX8	XM_002663947.6
B12G0332	1.65	4.62	ZNF438	Zinc finger protein 438	NM_001077161.1
A20G0562	1.65	1.68	CASP8AP2	CASP8-associated protein 2	NM_001014334.2
B3G0747	1.64	0.48	IGHV3-74	Immunoglobulin heavy variable 3-74	XM_021474793.1
A1G0328	1.64	6.41	HERC4	Probable E3 ubiquitin-protein ligase HERC4	XM_005160118.5
B3G0046	1.64	2.26	LOC113096977	A0A6P6PAM1_CARAU gastrula zinc finger protein XlCGF26.1-like	XM_068220071.1
B2G0250	1.64	3.31	ALG3	Dol-P-Man:Man(5)GlcNAc(2)-PP-Dol alpha-1,3-mannosyltransferase	XM_068224138.1
A11G0370	1.64	1.81	MAFB	Transcription factor MafB	XM_005172881.5

B15G0477	1.64	3.60			XM_005157583.5
B16G1118	1.64	2.88	TTK	Dual specificity protein kinase Ttk	XM_005157833.5
A9G0796	1.64	3.18	CIR1	Corepressor interacting with RBPJ 1	XM_021478817.2
B21G0997	1.64	135.92	BHMT	Betaine--homocysteine S-methyltransferase 1	NM_001012480.2
B12G0335	1.64	5.70	ARHGAP12	Rho GTPase-activating protein 12	XM_068224534.1
B21G0939	1.64	0.98	DPX16_23144	A0A3N0XKH6_ANAGA Uncharacterized protein	
A17G0022	1.64	14.90	VMO1	Vitelline membrane outer layer protein 1 homolog	
B13G0148	1.64	1.92	NCOA4	Nuclear receptor coactivator 4	NM_001037688.2
A23G0896	1.64	3.18	MEPCE	7SK snRNA methylphosphate capping enzyme	XM_001921694.6
A6G0675	1.64	4.98	ITPR1	Inositol 1,4,5-trisphosphate receptor type 1	XM_021474613.2
B3G0390	1.64	6.46	LILRA2	Leukocyte immunoglobulin-like receptor subfamily A member 2	XM_068218085.1
A11G0333	1.64	2.35	PNP	Purine nucleoside phosphorylase	XM_005155831.5
A3G0299	1.64	2.55	PDGFA	Platelet-derived growth factor subunit A	NM_001076757.1
A22G0556	1.64	2.87	YJU2B	Probable splicing factor YJU2B	NM_205595.2
A22G0726	1.64	2.70	SENP5	Sentrin-specific protease 5	NM_001080197.2
B19G0362	1.64	6.99	KAT2B	Histone acetyltransferase KAT2B	NM_001045034.1
B25G0318	1.64	3.13	ICE2	Little elongation complex subunit 2	
A7G0526	1.64	1.17	ORC6	Origin recognition complex subunit 6	NM_212671.2
A1G0257	1.64	10.49	OSBP	Oxysterol-binding protein 1	XM_068220270.1
B3G1324	1.64	3.24	FAM20C	threonine protein kinase FAM20C	XM_009299891.4
B24G0740	1.64	3.50	TGFBR1	TGF-beta receptor type-1	NM_001115059.1
B8G0278	1.64	4.52	BICD1	Protein bicaudal D homolog 1	NM_001083548.1
B21G0409	1.64	4.99	STX5	Syntaxin-5	NM_199630.2
B2G0242	1.64	0.86	CCL2	C-C motif chemokine 2	
B13G0622	1.64	8.52	NDUFAF1	Complex I intermediate-associated protein 30, mitochondrial	XM_021480617.2
A19G0764	1.64	3.30	RRAGC	Ras-related GTP-binding protein C	NM_001100068.1
B18G0721	1.64	11.28	PPP6R2	threonine-protein phosphatase 6 regulatory subunit 2	XM_003200421.6
B14G0449	1.64	3.88	MGAT4BQ9UQ53	Alpha-1,3-mannosyl-glycoprotein 4-beta-N-acetylglucosaminyltransferase B	NM_001077724.1
B24G0254	1.64	2.66	TRDMT1	tRNA (cytosine(38)-C(5))-methyltransferase	NM_001018143.1
A5G1032	1.64	4.08	RASA4	Ras GTPase-activating protein 4	XM_005171995.4
A8G0039	1.64	2.07	FABP1B.1	Fatty acid binding protein 1-B.1	NM_001024651.2
A15G0596	1.64	6.10	HIP1	Huntingtin-interacting protein 1	XM_005157432.5
A3G1314	1.64	36.76	ENDOD1	Endonuclease domain-containing 1 protein	
B17G0581	1.64	2.17	BAHD1	Bromo adjacent homology domain-containing 1 protein	XM_021473211.2
B4G0405	1.64	0.26	G5714_004900	A0A7J6D601_9TELE Uncharacterized protein	XR_011011502.1
B20G0548	1.64	1.45	LACE1B	Lactation elevated protein 1 homolog B	NM_001025482.2

B4G0901	1.64	1.00	A0A672M7M2	A0A672M7M2_SINGR Uncharacterized protein	XM_068219897.1
B8G0770	1.64	2.62	CDKN2C	Cyclin-dependent kinase 4 inhibitor C	NM_001199994.1
A14G0034	1.64	1.56	SPATA5	Ribosome biogenesis protein SPATA5	XM_021481297.2
A18G0643	1.64	3.33	CHKA	Choline kinase alpha	NM_001100012.2
A13G0856	1.64	3.39	PERP	p53 apoptosis effector related to PMP-22	XM_017358861.3
A16G0936	1.64	0.29	MAL2	Protein MAL2	NM_001135977.2
B22G1084	1.64	8.62	ZGC:77082	4-hydroxy-2-oxoglutarate aldolase, mitochondrial	NM_212870.1
B7G0491	1.64	2.73	AGTR1	Type-1 angiotensin II receptor	
B18G0587	1.64	5.15	EEA1	Early endosome antigen 1	XM_003200437.6
A25G0284	1.64	1.82	MICAL2B	[F-actin]-monooxygenase mical2b	NM_001271913.2
B14G0359	1.64	2.30	BCORL1	BCL-6 corepressor-like protein 1	XM_005173317.5
B4G0821	1.64	0.81	A0A671MNT3	A0A671MNT3_9TELE Uncharacterized protein	XM_068215354.1
B17G0165	1.64	76.22	A0A673M5Y4	A0A673M5Y4_9TELE Saposin B-type domain-containing protein	
A16G0894	1.64	2.20	CEP192	Centrosomal protein of 192 kDa	XM_009292114.4
A2G1117	1.64	5.55	GNA11	Guanine nucleotide-binding protein subunit alpha-11	NM_001007773.1
A13G0244	1.64	1.41	MEMO1	Protein MEMO1	NM_212813.2
A7G1148	1.64	6.05	DAGLA	Diacylglycerol lipase-alpha	XM_692781.9
B4G0238	1.64	1.88	HCFC2	Host cell factor 2	NM_001327839.1
B21G0165	1.64	4.32	UNC119	Protein unc-119 homolog A	NM_001040394.2
B8G0424	1.64	5.00	UPB1	Beta-ureidopropionase	NM_199616.1
B22G0234	1.64	2.82	LOC113040299	A0A6P6J4M9_CARAU uncharacterized protein LOC113040299 isoform X3	XM_068217738.1
A17G0279	1.64	2.90	DNAJC27	DnaJ homolog subfamily C member 27	NM_001007162.2
A7G0280	1.64	3.01	ZNF319	Zinc finger protein 319	XM_001923303.8
B11G0096	1.64	1.13	RPF1	Ribosome production factor 1	NM_001328553.2
B4G0710	1.64	2.70	POL	Retrovirus-related Pol polyprotein from transposon opus	
A9G0685	1.63	3.32	LOC113075922	A0A6P6N5I4_CARAU uncharacterized protein LOC113075922 isoform X1	
B12G0611	1.63	4.32	PRDM9	Histone-lysine N-methyltransferase PRDM9	NM_200902.1
A12G0756	1.63	0.19	CA14	Carbonic anhydrase 14	
B15G0905	1.63	7.53			
B22G0987	1.63	2.28	DAG1	Dystroglycan 1	XM_009296487.4
B9G0767	1.63	2.76	SI:DKEY-18L1.1	von Willebrand factor A domain-containing protein 8	NM_001128338.1
B6G0212	1.63	4.05	ALS2	Alsin	XM_005165740.5
A3G0278	1.63	1.50	PTGER1	Prostaglandin E2 receptor EP1 subtype	NM_001166333.1
A5G0946	1.63	1.17	TOR2A	Torsin-2A	NM_001034180.2
A9G0814	1.63	14.20	CYBRD1	Plasma membrane ascorbate-dependent reductase CYBRD1	NM_001020500.2
B11G0059	1.63	0.90	LIMA1	LIM domain and actin-binding protein 1	XM_021479917.2
A25G0179	1.63	1.84	IFTAP	Intraflagellar transport-associated protein	XM_005162985.5
A19G0460	1.63	11.25	PPT1	Palmitoyl-protein thioesterase 1	

B1G0223	1.63	10.51	TTYH3	Protein tweety homolog 3	NM_001037387.3
B25G0609	1.63	3.79	ZNRF1	E3 ubiquitin-protein ligase znrf1	NM_200870.1
B11G0176	1.63	5.10	G5714_012076	A0A7J6CPH5_9TELE SCY domain-containing protein	NM_001099982.2
A24G0407	1.63	4.35	USF3	Basic helix-loop-helix domain-containing protein USF3	XM_005162733.5
B5G0642	1.63	1.47	SSBP2	Single-stranded DNA-binding protein 2	NM_001110378.1
A19G0448	1.63	0.80	DPX16_7623	A0A3N0YR52_ANAGA AT-hook DNA-binding motif-containing protein 1	
A21G0723	1.63	11.87	DNTS_024005	A0A553MVC4_9TELE Uncharacterized protein	
B21G0925	1.63	3.26	LOC113071098	A0A6P6MV42_CARAU gastrula zinc finger protein XlCGF57.1-like	XM_021468955.2
A4G0095	1.63	1.58	A0A672NKX7	A0A672NKX7_SINGR Uncharacterized protein	XM_017355826.3
A12G0542	1.63	2.67	RTASE	Probable RNA-directed DNA polymerase from transposon BS	XM_068219861.1
A18G0545	1.63	5.47	EEA1	Early endosome antigen 1	XM_003200437.6
B5G0572	1.63	2.66	PPEF2	threonine-protein phosphatase with EF-hands 2	XM_009301816.4
A18G0807	1.63	2.33	GATM	Glycine amidinotransferase, mitochondrial	NM_199531.1
A8G0507	1.63	3.30	SEMA3F	Semaphorin-3F	XM_005167031.5
B4G0061	1.63	3.10	ARID2	AT-rich interactive domain-containing protein 2	XM_005164457.5
B4G0827	1.63	1.54	TCB1	Transposable element Tcb1 transposase	XM_021471168.2
B6G0028	1.63	2.19			
A5G1163	1.63	1.49	CDH1	Cadherin-1	XM_068222149.1
B2G0969	1.63	1.71	G5714_001606	A0A7J6DCN5_9TELE Uncharacterized protein	NM_001365136.1
B13G0578	1.63	3.54	PGAM1	Phosphoglycerate mutase 1	XM_068218720.1
B22G0435	1.63	2.24	LORF2	LINE-1 retrotransposable element ORF2 protein	
A5G0275	1.63	6.91	GIT2	ARF GTPase-activating protein GIT2	NM_001126453.1
B4G0872	1.63	0.87	MS4A4A	Membrane-spanning 4-domains subfamily A member 4A	XM_003199992.6
A6G0597	1.63	3.48	UHMK1	threonine-protein kinase Kist	NM_001076659.2
A9G0558	1.63	0.94	WARS2	Tryptophan--tRNA ligase, mitochondrial	NM_001013323.2
A2G0988	1.63	3.80	YEATS2	YEATS domain-containing protein 2	XM_002660895.7
B18G0805	1.63	6.73	LRCH3	DISP complex protein LRCH3	XM_005173610.5
A2G0640	1.63	4.16	CACNB2	Voltage-dependent L-type calcium channel subunit beta-2	NM_001423728.1
A11G0773	1.63	1.70	NCKAP1L	Nck-associated protein 1-like	NM_001002590.1
B21G0794	1.63	0.61	LOC113039228	A0A6P6IYT3_CARAU urokinase plasminogen activator surface receptor-like	XM_068216177.1
A15G0423	1.63	3.75	MNT	Max-binding protein MNT	XM_009291752.4
A17G0197	1.63	0.61	KIF26A	Kinesin-like protein KIF26A	XM_005158961.5
A22G0173	1.63	0.51	CD84	SLAM family member 5	XM_068216406.1
B20G0117	1.63	4.06	ARID1B	AT-rich interactive domain-containing protein 1B	XM_005160356.5
A18G0013	1.63	5.69	P2RY1	P2Y purinoceptor 1	XM_001343319.8
A23G0499	1.63	8.37	MIP	Lens fiber major intrinsic protein	NM_001003534.1
B24G0090	1.63	2.72	KATNIP	Katanin-interacting protein	XM_009297662.4

A23G0372	1.62	1.05	TNFRSF1A	Tumor necrosis factor receptor superfamily member 1A	XM_009296781.4
A9G0628	1.62	0.26	LOC113053619	A0A6P6KQK0_CARAU uncharacterized protein LOC113053619	XM_682096.6
A25G0616	1.62	5.03	RASSF8	Ras association domain-containing protein 8	XM_009298064.4
A21G0787	1.62	0.93	MSXD	Homeobox protein MSH-D	NM_131276.3
B3G0124	1.62	1.61	LOC113074991	A0A6P6N523_CARAU gastrula zinc finger protein XlCGF57.1-like isoform X1	XM_021475634.2
B16G0709	1.62	4.30	SHC1	SHC-transforming protein 1	XM_017351494.3
B1G0164	1.62	3.64	MEP1B	Meprin A subunit beta	XM_021471111.2
A4G0392	1.62	1.19	LOC107573893	A0A672MGK6_SINGR Gastrula zinc finger protein XlCGF57.1-like	XM_068220087.1
A17G0598	1.62	2.38	ALKBH1	Nucleic acid dioxygenase ALKBH1	XM_005158674.5
B2G1043	1.62	5.13	CABLES1	CDK5 and ABL1 enzyme substrate 1	NM_001105665.1
B1G0693	1.62	1.01	PTGDR2	Prostaglandin D2 receptor 2	
B3G1528	1.62	1.88	A0A673M8N8	A0A673M8N8_9TELE Uncharacterized protein	XM_068220660.1
B6G0534	1.62	10.80	CLDN22	Claudin-22	
B9G0792	1.62	2.42	A0A672VAH6	A0A672VAH6_STRHB Uncharacterized protein	
B10G0609	1.62	1.81	NF1	Neurofibromin	XM_005169190.5
[truncated: 2,544,968 more chars]
