# Supplementary material for: A telomere-to-telomere genome assembly of koi carp (Cyprinus carpio) using long reads and Hi-C technology
Source: Gigascience. 2025 Aug 29;14:giaf087. doi: 10.1093/gigascience/giaf087 (PMC12395963; doi:10.1093/gigascience/giaf087)
Supplement: giaf087_Supplemental_Files [file giaf087_supplemental_files.zip › Supplementary Figure.pdf]

Supplementary Figures

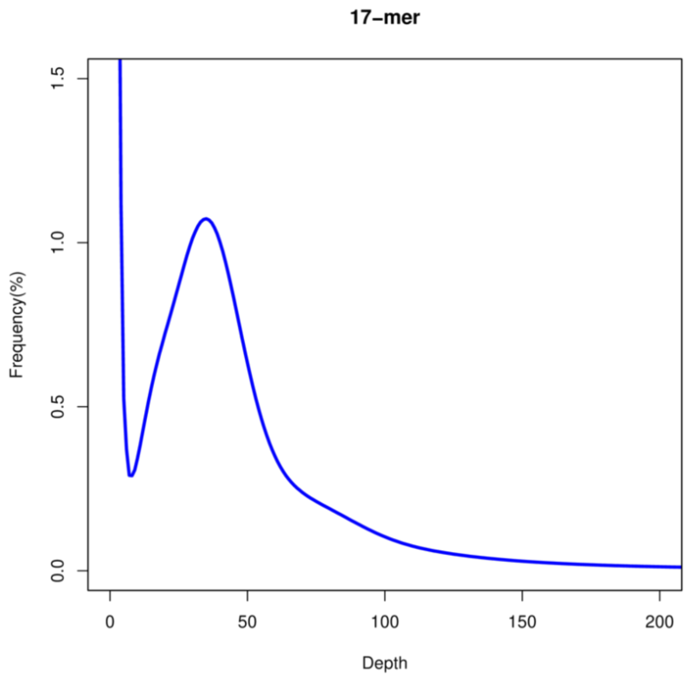

Supplementary Fig. 1 17 bp-mer estimation of the genome size. The X-axis represents the sequencing depth. The Y-axis is the proportion that represents the frequency at that depth divided by the total frequency of all depths.

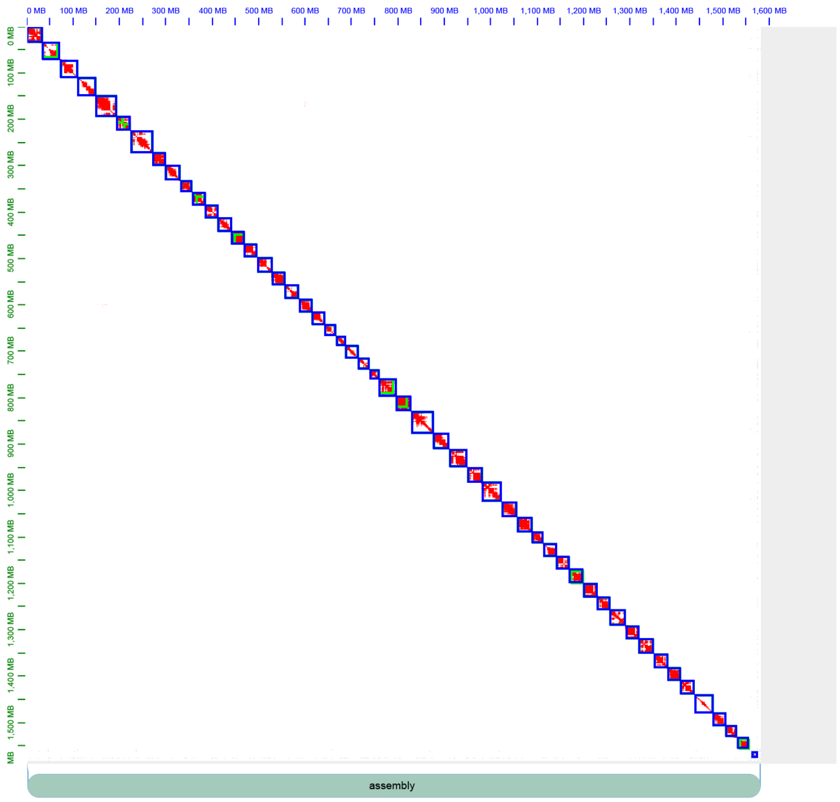

Supplementary Fig. 2 Contact map of Hi-C interaction for the koi carp genome assembly. The sequences anchored on chromosomes are shown in the plot; green and blue boxes represent contigs and chromosomes, respectively.

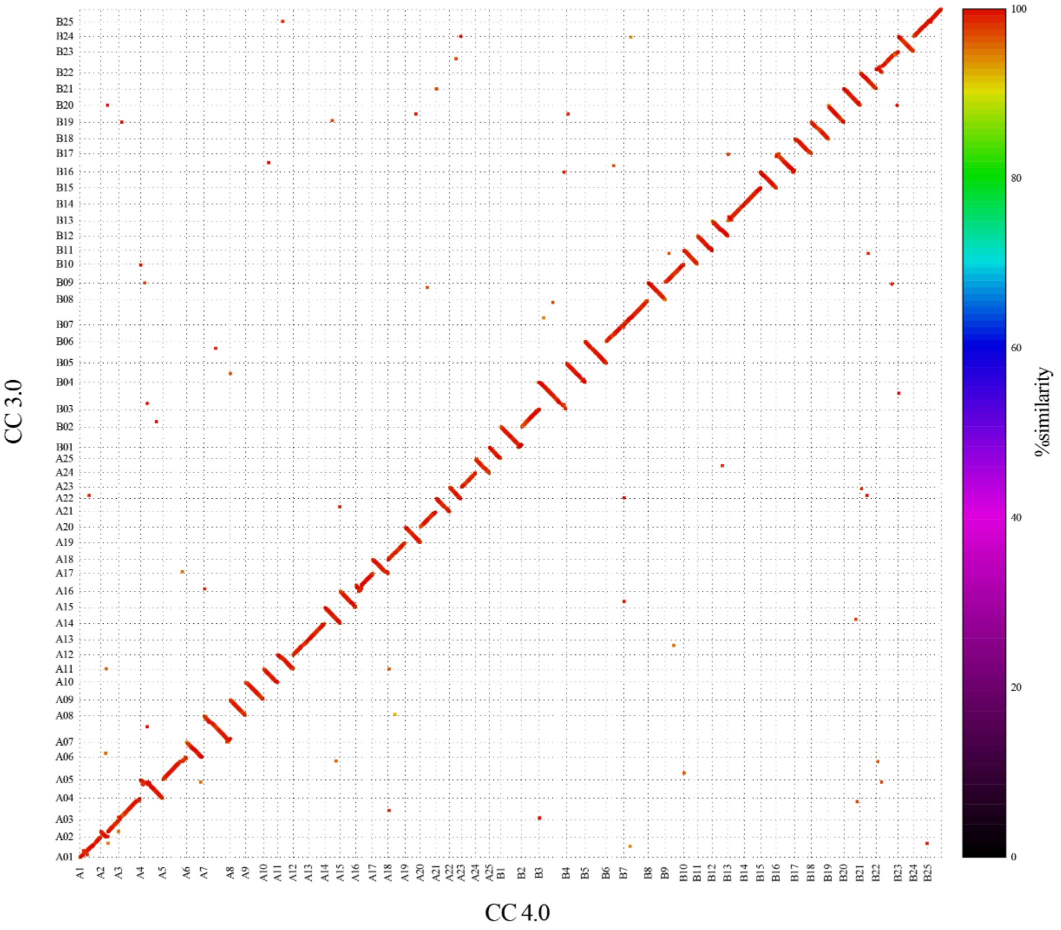

Supplementary Fig. 3. Comparison of the CC 3.0 and CC 4.0 using MUMmer. The X-axis and Y-axis represent the chromosomes of CC 4.0 and CC 3.0, respectively.

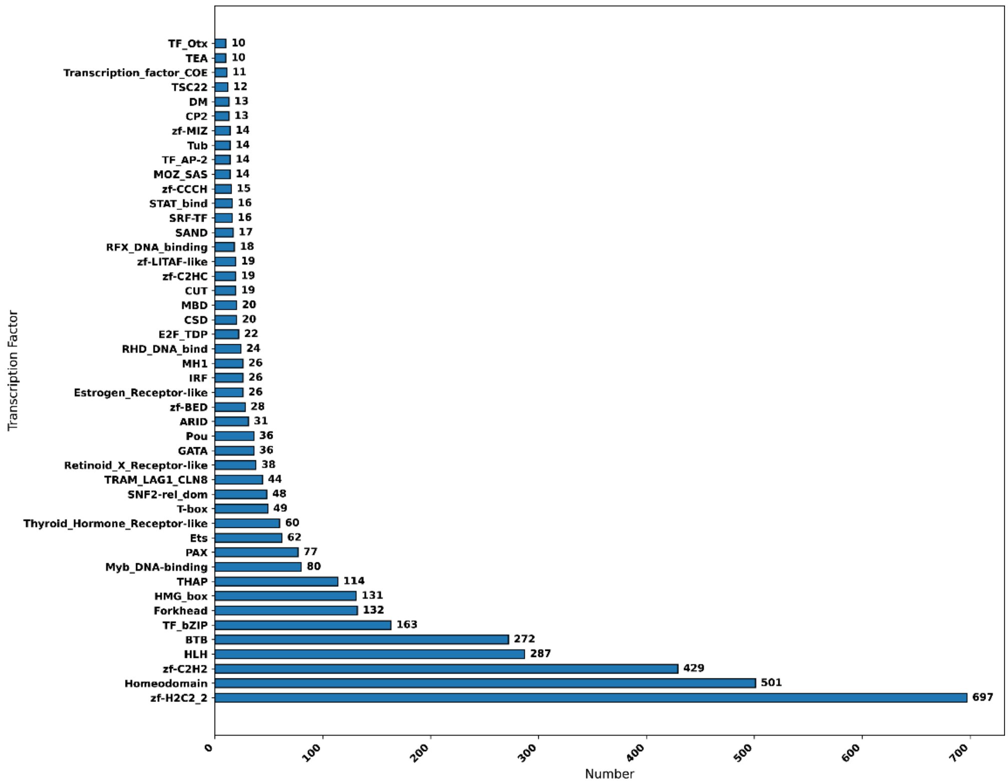

Supplementary Fig. 4. Distribution of transcription factor counts. The horizontal bar chart displays the number of transcription factors with ⩾10 copies. Each bar's length corresponds to the TF count, with numerical values labeled to the right of each bar.

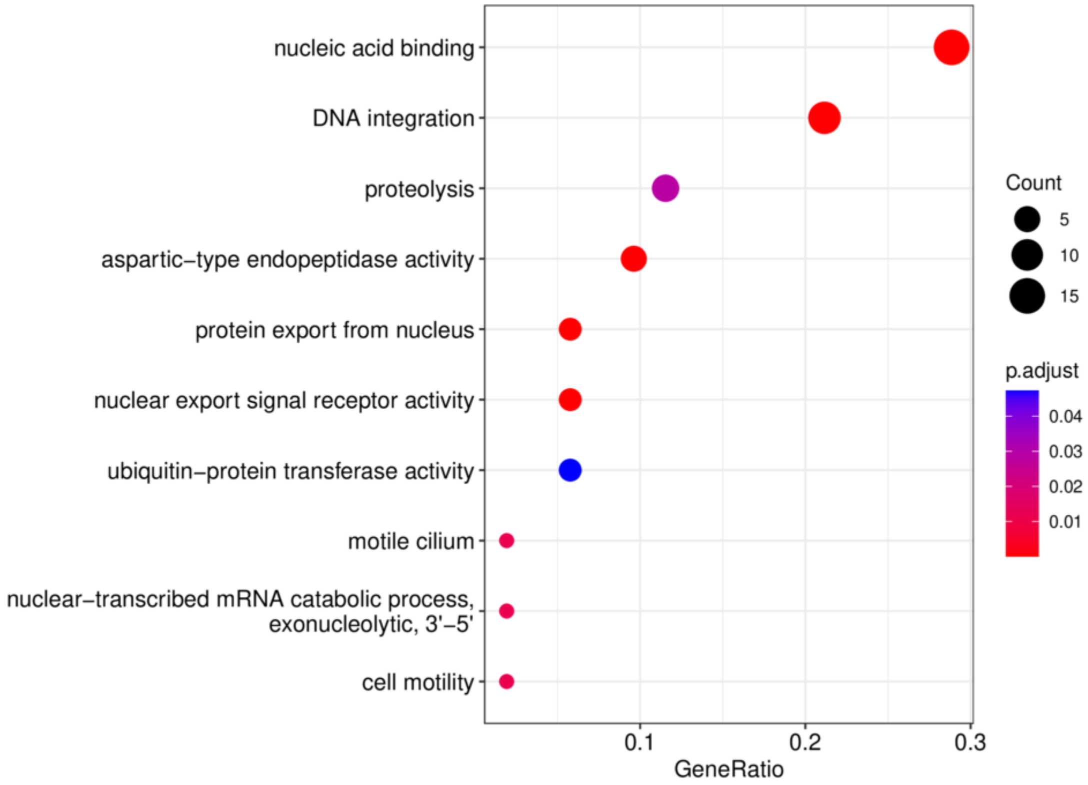

Supplementary Fig. 5 GO enrichment analysis of 95 genes in the centromeric regions. Gene ratio (x-axis) is the percentage of the number of genes present in this GO term over the total number of genes in this category. A larger size of a circle's diameter represents a higher gene number.
