# Supplementary material for: A telomere-to-telomere genome assembly of koi carp (Cyprinus carpio) using long reads and Hi-C technology
Source: Gigascience. 2025 Aug 29;14:giaf087. doi: 10.1093/gigascience/giaf087 (PMC12395963; doi:10.1093/gigascience/giaf087)
Supplement: giaf087_GIGA-D-24-00530_Original_Submission [file giaf087_giga-d-24-00530_original_submission.pdf]

GigaScience

A telomere-to-telomere genome assembly of koi carp (Cyprinus carpio) using long reads and Hi-C technology

--Manuscript Draft--

**Manuscript Number:**	GIGA-D-24-00530
**Full Title:**	A telomere-to-telomere genome assembly of koi carp (Cyprinus carpio) using long reads and Hi-C technology
**Article Type:**	Data Note
**Funding Information:**	
**Abstract:**	Background: The common carp (Cyprinus carpio) is a key species in global freshwater aquaculture. One of its variants, koi carp is particularly prized for its aesthetic appeal. However, the lack of a high-quality genome has limited genetic research and breeding efforts for common carp and koi carp.<br>Findings: In this study, we present a gap-free genome for the Taisho Sansyoku koi carp strain (C. carpio). The assembly achieved a total size of 1555.86 Mb with a contig N50 of 30.45 Mb, comprising 50 gap-free pseudochromosomes ranging in length from 20.70 to 49.02 Mb. The BUSCO completeness score reached 99.20% and the GCI score was 85.82, indicating a high level of genome integrity and accuracy. Notably, 83 out of 100 telomeres were detected, resulting in 33 chromosomes possessing complete telomeres. Comparative genomic analysis showed that the expanded gene families and unique genes play important roles in various biological traits, such as energy metabolism, endocrine regulation, cell proliferation, and immune response, potentially related to multiple metabolic diseases and health conditions. The positively selected genes are linked to various biological processes, such as the metalloendopeptidase activity, which plays a significant role in the central nervous system and is associated with diseases.<br>Conclusions: The gap-free genome assembly of koi carp fills a critical gap in the understanding of this species and its adaptations. It provides an invaluable resource for molecular-guided breeding and genetic enhancement strategies, underscoring the importance of common carp and koi carp in aquaculture and ecological research.
**Corresponding Author:**	Yongchao Niu, PhD<br>Biozeron Shenzhen Inc.<br>Shenzhen, CHINA
**Corresponding Author Secondary Information:**	
**Corresponding Author's Institution:**	Biozeron Shenzhen Inc.
**Corresponding Author's Secondary Institution:**	
**First Author:**	Yongchao Niu, PhD
**First Author Secondary Information:**	
**Order of Authors:**	Yongchao Niu, PhD
	Jiandong Yuan
	Jiang Li
	Jun Yong
	Xuewu Liao
	Huijuan Guo
**Order of Authors Secondary Information:**	
**Additional Information:**	
**Question**	**Response**

Powered by Editorial Manager® and ProduXion Manager® from Aries Systems Corporation

Are you submitting this manuscript to a special series or article collection?	No
**Experimental design and statistics**<br><br>Full details of the experimental design and statistical methods used should be given in the Methods section, as detailed in our Minimum Standards Reporting Checklist. Information essential to interpreting the data presented should be made available in the figure legends.<br><br>Have you included all the information requested in your manuscript?	Yes
**Resources**<br><br>A description of all resources used, including antibodies, cell lines, animals and software tools, with enough information to allow them to be uniquely identified, should be included in the Methods section. Authors are strongly encouraged to cite Research Resource Identifiers (RRIDs) for antibodies, model organisms and tools, where possible.<br><br>Have you included the information requested as detailed in our Minimum Standards Reporting Checklist?	Yes
**Availability of data and materials**<br><br>All datasets and code on which the conclusions of the paper rely must be either included in your submission or deposited in publicly available repositories (where available and ethically appropriate), referencing such data using a unique identifier in the references and in the "Availability of Data and Materials" section of your manuscript.<br><br>Have you have met the above requirement as detailed in our Minimum	Yes

Standards Reporting Checklist?	
GigaScience has policies and guidelines in place for the use of generative AI-writing tools such as ChatGPT. If you have used such writing tools to assist with writing the manuscript this must be declared and cited in the text. Authors should not list AI-writing tools and other AI-assisted technologies as an author or co-author and should acknowledge that they are fully responsible for text generated or refined by AI-writing tools.<p><br><br>A summary of use (particularly in the introduction or among methods) needs to be included at the end of the paper, and the outputs should also be included as a supplementary file hosted in GigaDB or other open repositories. Please read our guidelines for more information. <p><br><br>By submitting to GigaScience, you are aware of the journal's AI-writing tools policy, and if you have declared use of such tools below, you have acknowledged this where appropriate in your manuscript and have made a summary of use and outputs available. <p><br><b>AI-assisted writing tools have been used in the preparation of this manuscript?	No

Powered by Editorial Manager® and ProduXion Manager® from Aries Systems Corporation

DATANOTE

A telomere-to-telomere genome assembly of koi carp (*Cyprinus carpio*) using long reads and Hi-C technology

Jiandong Yuan [1*†], Jiang Li [2†], Jun Yong[3†], Xuewu Liao [1], Huijuan Guo[3], Yongchao Niu [2*]

1 Suxin Koi Farm, Suzhou 215000, China

2 Biozeron Shenzhen Inc., Shenzhen 518000, China

3 Geekgene Technology Co. Ltd., Beijing 100091, China

＊Correspondence address. Suxin Koi Farm, Suzhou 215000, China. E-mail: yuan@suxinkoi.com; Biozeron Shenzhen Inc. Shenzhen 518000, China. E-mail: niuyongchao@biozeron.com.

[†] These authors contributed equally to this work.

Abstract

Background: The common carp (*Cyprinus carpio*) is a key species in global freshwater aquaculture. One of its variants, koi carp is particularly prized for its aesthetic appeal. However, the lack of a high-quality genome has limited genetic research and breeding efforts for common carp and koi carp.

Findings: In this study, we present a gap-free genome for the Taisho Sansyoku koi carp strain (*C. carpio*). The assembly achieved a total size of 1555.86 Mb with a contig N50 of 30.45 Mb, comprising 50 gap-free pseudochromosomes ranging in length from 20.70 to 49.02 Mb. The BUSCO completeness score reached 99.20% and the GCI score was 85.82, indicating a high level of genome integrity and accuracy. Notably, 83 out of 100 telomeres were detected, resulting

in 33 chromosomes possessing complete telomeres. Comparative genomic analysis showed that the expanded gene families and unique genes play important roles in various biological traits, such as energy metabolism, endocrine regulation, cell proliferation, and immune response, potentially related to multiple metabolic diseases and health conditions. The positively selected genes are linked to various biological processes, such as the metalloendopeptidase activity, which plays a significant role in the central nervous system and is associated with diseases.

Conclusions: The gap-free genome assembly of koi carp fills a critical gap in the understanding of this species and its adaptations. It provides an invaluable resource for molecular-guided breeding and genetic enhancement strategies, underscoring the importance of common carp and koi carp in aquaculture and ecological research.

Key words: common carp, koi carp, telomere-to-telomere, genome, positively selected gene

Data Description

Context

Common carp (*Cyprinus carpio*) is one of the most economically significant species, accounting for up to 10% (over 3 million metric tons) of global freshwater aquaculture production [1]. It is mainly cultured in Europe and Asia with a cultural history of several thousand years and has been introduced into most parts of the world. Known for being environmentally friendly, common carp are primarily omnivorous filter-feeders, requiring less fish meal and fish oil compared to other aquaculture species such as salmon and shrimp [2]. In addition to serving as a food source, one of the common carp variant, koi carp, is highly prized as an ornamental fish, renowned for its

vibrant colors and patterns. *C. carpio* originated from the hybridization of a Barbinae-like species and an undetermined donor species, followed by a whole genome duplication event approximately 12.4 million years ago [3]. It is believed that their genome duplication is responsible for species divergence and biodiversity [4]. As an ideal model for studying the structural and functional adaptations in polyploid vertebrates, koi carp provides valuable insights into successful speciation and the evolutionary dynamics of polyploidy in animals, making it a critical species for both aquaculture and ecological research. Notably, it is also regarded as an alternative vertebrate model to zebrafish.

Over the past decade, various *C. carpio* genome resources have been developed. The genome of *C. carpio* (strain Songpu) was first decoded in 2014, marking the beginning of common carp genomics research [2]. In 2019, chromosome-level reference genomes of Yellow River carp, Hebao red carp, and German Mirror carp were generated [3]. Moreover, the availability of *Poropuntius huangchuchieni* genome provides a diploid progenitor-like reference genome for the allotetraploid *C. carpio* [5]. In 2021, the genome of common carp var. 'Songpu' was updated [6], followed by the availability of the latest *C. carpio* genome (CC 3.0) in 2023 obtained via Pacific Biosciences (PacBio) high-fidelity (HiFi) reads [7]. Intensive culture conditions make farmed common carp vulnerable to various pathogens, leading to high mortality rates and significant economic losses in the carp culture industry. Hence, *C. carpio* has been continuously studied in terms of its immunology and disease resistance [8-11]. *De novo* genome assembly is a fundamental and powerful tool. Currently, advances in sequencing and assembly algorithms make telomere-to-telomere (T2T) genome assembly feasible, enabling comprehensive genome identification. Some important species, such as humans [12], sheep [13, 14], rice [15], maize [16],

and sorghum [17], have successively released T2T-level genomes. The considerations and methodologies for executing T2T assembly have been thoroughly summarized [18, 19]. However, the assembly of the *C. carpio* genome to a comparable level has yet to be reported. To address this gap, we integrated PacBio HiFi sequencing, Oxford Nanopore Technologies (ONT) ultralong sequencing, and chromosomal conformational capture (Hi-C) technology to assemble a high-quality gap-free genome assembly for koi carp (strain Taisho Sansyoku; Fig. 1). Moreover, the characteristics of centromeric regions were investigated, and genomic evolution analyses were performed. This study on the *C. carpio* genome provides a valuable resource for the molecular-guided breeding and genetic improvement of the common carp and koi carp.

Methods

Sample collection

We collected a healthy koi carp (Taisho Sansyoku) from Suzhou City, Jiangsu Province, China for DNA sequencing, RNA-seq, and Iso-seq. Genomic DNA was extracted from a muscle sample. To improve genome annotation, scale, and fin tissues were prepared for RNA-seq. In addition, RNA from eighteen tissues, including eye, tail, white scalp, red scalp, brain, black scale, white scale, red scale, heart, blood, liver, bubble, essence, spleen, bile, kidney, muscle, gill, and intestines, were equally pooled together for Iso-seq. All samples were frozen in liquid nitrogen and stored at -80°C for preservation and subsequent analysis.

Sequencing and filtering

For HiFi sequencing, SMRTbell target size libraries were constructed according to PacBio's standard protocol (Pacific Biosciences) using the 15-kb preparation solutions. The sequencing was conducted in circular consensus sequencing (CCS) mode on the PacBio Revio platform (RRID:SCR_017990) at Grandomics Biosciences (Wuhan, China). The generated subreads were processed using SMRTLink version 8.0.0 [20] with the following parameters: "-minPasses 3 -minPredictedAccuracy 0.99 -minLength 500".

For ONT sequencing, ONT ultra-long insert libraries were obatinted using the Oxford Nanopore SQK-LSK109 kit and sequenced on the PromethION (RRID:SCR_017987) platform at Grandomics Biosciences (Wuhan, China). The ONT data underwent processing using NanoFilt version 2.8.020 (RRID:SCR_016966) [21] with a quality threshold of 7.

Hi-C libraries based on *Dpn*II restriction enzymes were prepared for Hi-C sequencing, as previously described [22]. These libraries were sequenced on the MGISEQ-2000 platform, generating paired-end 150 bp reads. Clean Hi-C data were obtained using fastp version 0.19.5 (RRID:SCR_016962) [23] with parameters set as "--length_required 50 -w 8". In addition, about 1.5 μg DNA was used to construct an approximately 350 bp insert size DNA library. According to the standard manufacturer's instructions, the quantified library was sequenced on the Illumina NovaSeq platform (Illumina, CA, USA).

The total RNA was extracted using TRIzol reagent in an RNAprep Pure Tissue Kit and processed according to the protocol provided by the manufacturer. Subsequently, the RNA purity and concentration were assessed using Nanodrop and Qubit, where only high-quality RNA samples were selected for cDNA synthesis in both bulk RNA-seq and Iso-seq experiments. For Iso-Seq, sequencing libraries were prepared using the SMRTbell Template Prep Kit 2.0 from

Pacific Biosciences and sequenced on the PacBio Sequel II platform. Libraries for bulk RNA-seq were sequenced on an MGISEQ-2000 instrument, generating 150 bp paired-end reads. The libraries for bulk RNA-seq were sequenced on an MGISEQ-2000 instrument, producing 150 bp paired-end reads. The raw reads of Iso-Seq were preprocessed using SMRTLink version 8.0.0 [20]. Iso-Seq CCS reads were derived from the subreads with specific parameters: minimum subread length = 50, maximum subread length = 15,000, minimum number of passes = 3, and minimum predicted accuracy = 0.99. The quality of RNA-seq data was examined using fastp version 0.19.5 (RRID:SCR_016962) [23] with the parameters set as "-w 8 -l 50".

Genome size estimation

To estimate the genome size and heterozygosity of the koi carp, a similar method in the study of the largemouth bass genome [24] was applied. Jellyfish version 2.1.3 (RRID: SCR_005491) [25] was used to analyze the *k*-mer depth distribution curve with a *k*-mer size of 17. The genome size was calculated using the formula G = (Ktotal - Kerror) / D, where Ktotal represents the total count of k-mers, Kerror is the total count of low-frequency k-mers (frequency ⩽ 3) likely due to sequencing errors, G is the genome size, and D is the k-mer depth [26].

Genome assembly and Hi-C scaffolding

To assemble a T2T reference genome, a combination of methods and sequencing reads was utilized. Initially, ultra-long ONT reads were processed using NextDenovo version 2.5.2 (RRID: SCR_025033) [27] for downstream gap-filling analysis. Primary contigs were generated with Hifiasm version 0.19.6 (RRID: SCR_021069) [28] using the command: "hifiasm -o Carp -t32 --ul ul.fq.gz --h1 Hi-C_clean_1.fq.gz --h2 Hi-C_clean_2.fq.gz HiFi-reads.fq.gz". Contigs

were then polished with NextPolish2 version 0.2.0 [29] using HiFi reads. Hi-C clean data was aligned to the polished contigs for scaffolding using Bowtie2 version 2.2.9 (RRID: SCR_016368) [30]. Low-quality reads were eliminated using the HiC-Pro pipeline (RRID: SCR_017643) [31] with default parameters. Valid reads were utilized to anchor chromosomes with Juicer version 1.6 (RID: SCR_017226) [32] and 3d-DNA pipeline version 180419 (RRID: SCR_017227) [33]. An additional error correction step was carried out with Juicebox version 2.13.07 (RRID: SCR_021172) [34] according to the interaction signal. Following a similar approach used in the goose T2T genome study [35], gaps within the assembled genome were filled using quartet_gapfiller.py from quarTeT version v1.1.1 (RRID: SCR_025258) [36], utilizing preassembled contigs generated from NextDenovo version 2.5.2 (RRID: SCR_025033) [27]. As recommended, the specific parameters used were "-f 5000 -l 1000 -i 40 -m 1000000 -t 20". In addition, we applied the LR_Gapcloser (RRID: SCR_017021) [37] program to close the remained gaps in the assembled chromosomes, referring to the methods described in the gap-free genome of *Neosalanx taihuensis* [38]. To enhance genome quality, Winnowmap version 2.03 (RRID: SCR_025349) [39] was used to align HiFi reads to the chromosomes, followed by filtering to exclude secondary alignments and excessive clipping with the 'falconc bam-filter-clipped' tool. Finally, racon version 1.5.0 (RRID: SCR_017642) [40] was performed for further polishing with the filtered alignments.

The completeness of the genome assembly was assessed utilizing Benchmarking Universal Single-Copy Orthologs (BUSCO) version 5.5.0 (RRID: SCR_015008) [41] with the actinopterygii_odb10 database, which includes 3,640 orthologs. The quality value (QV) was evaluated by Merqury program version 1.3 (RRID: SCR_022964) [42] with 17-mer. Furthermore,

short reads were aligned to the genome using bwa version 0.7.17-r1188 (RRID: SCR_010910) [43], while long reads from ONT and HiFi were aligned with Minimap2 version 2.24-r1122 (RRID: SCR_018550) [44]. In addition, the Genome Continuity Inspector (GCI) was assessed using GCI version 1.0 [45]. For collinearity analysis, the two genomes were compared using MUMmer4 version 4.0.0rc1 (RRID: SCR_018171) [46] with parameters of "-t 30 -p mummer --mum -g 1000 -c 90 -l 40".

Genome annotations

Tandem Repeats Finder version 4.10 (RRID: SCR_022065) [47] was used to identify the tandem repeat elements. For interspersed repetitive sequences, a combined approach of *de novo* prediction and known repeat searching was employed. RepeatModeler version 1.0.8 (RRID: SCR_015027) [48] and LTR_FINDER version 1.0.6 (RRID: SCR_015247) [49] were used to predict *de novo* repeat sequences. Subsequently, RepeatMasker version 4.0.7 (RRID: SCR_012954) [50] was applied to screen the koi carp genome against the combined *de novo* transposable element library. Additionally, RepeatMasker version 4.0.7 (RRID: SCR_012954) [50] and RepeatProteinMask version 4.0.6 were employed to search the Repbase database (RRID: SCR_021169) [51] to identify known transposable element repeats. The annotation of noncoding RNAs in the koi carp genome utilized the same method as in the largemouth bass genome study [24].

Telomeric sequences within the koi carp genome assembly were screened using quarTeT version v1.1.1 (RRID: SCR_025258) [36] with the "-c animal" option, following a method similar to that described in the study of the near-complete sheep assembly [52]. The telomere

repeat monomer identified by the TeloExplorer module in quarTeT program was “AACCCT”. Centromeres were identified using both the quarTeT version v1.1.1 (RRID: SCR_025258) [36] and Centromics methods (https://github.com/ShuaiNIEgithub/Centromics). The results from quarTeT were given priority. If the TRcoverage of a chromosome's centromere was less than 40% or TEcoverage was less than 80% in the quarTeT identification results, it was considered an unreliable result. In such cases, the centromere region was determined using the Centromics method.

The gene prediction process employed a comprehensive strategy that integrated transcriptome-based, homology-based, and *ab initio* prediction methods. Initially, RNA-seq clean reads and PacBio full-length CCS reads were assembled using Trinity version 2.11.0 (RRID: SCR_013048) [53], with the parameters ‘--max_memory 200G --CPU 40 --min_contig_length 200 --genome_guided_bam merged_sorted.bam --full_cleanup --min_kmer_cov 4 --min_glue 4 --bfly_opts '-V 5 --edge-thr=0.1 --stderr' --genome_guided_max_intron 10000 --long_reads ccs.fa’, yielded 289,634 transcripts with a N50 size of 2,826. The assembled transcripts were then aligned to the assembly using Program to Assemble Spliced Alignment (PASA) version 2.4.1 (RRID: SCR_014656) [54], generating gene structures from valid transcript alignments (PASA-set). Additionally, RNA-seq clean reads were mapped to the assembly via Hisat2 version 2.0.1 (RRID: SCR_015530) [55]. Subsequently, Stringtie version 1.2.2 (RRID: SCR_016323) [56] and TransDecoder version 5.7.1 (RRID: SCR_017647) were employed to assemble the transcripts and identify candidate coding regions, resulting in the creation of gene models (Stringtie-set). Homologous genomes from seven assemblies, including four common carps (hebaored, germanmirror, huanghe, Songpu;

ensenbl_release-111), *Carassius auratus* (ensenbl_release-111), *Danio rerio* (ensenbl_release-111), and *Poropuntius huangchuchieni* (https://ngdc.cncb.ac.cn/gwh/Assembly/10299/show) were downloaded and used as queries to search against the assembly using GeMoMa version 1.9 (RRID: SCR_017646) [57]. These homology predictions were referred to as "Homology-set". For *ab initio* prediction, helixer [58] was employed to predict coding regions in the soft-masked genome. The gene models from these three sources were subsequently merged using EvidenceModeler version 2.1.0 (RRID: SCR_014659) [59], with different weight parameters assigned to evidence from different sources (10 for PASA-set, 5 for Stringtie-set, 5 for Homology-set, and 1 for *ab initio* gene prediction). Finally, the generated gene models underwent further refinement with PASA version 2.4.1 (RRID: SCR_014656) [54] to obtain untranslated regions and alternative splicing variation information.

The integrated gene set was translated into amino-acid sequences and annotated using various databases. Diamond version 0.9.30 (RRID: SCR_009457) [60] with an E-value cutoff of 1e-05 was used to compare the protein against four public databases, including NCBI non-redundant protein sequence database, SwissProt [61], Kyoto Encyclopedia of Genes and Genomes (KEGG) [62], Translation of European Molecular Biology Laboratory. Gene ontology (GO) terms of these genes were identified using InterProScan version 5.59-91.0 (RRID: SCR_005829) [63].

Gene expression analysis was conducted following the method used in the blister beetles transcriptome [64]. Transcription factor (TF) prediction was done using AnimalTFDB version 4.0 [65].

Gene families and phylogenomic analysis

Protein sequences for six vertebrate animals, including *C. auratus, D. rerio, P. huangchuchieni*, *Oryzias latipes*, *Sinocyclocheilus grahami* and *Homo sapiens,* were obtained from public databases. The gene families were defined using Treefam (RRID: SCR_013401) [66]. For genes with alternative splicing variants, the longest transcripts were selected to represent the genes. Blastp version 2.7.1+ (RRID: SCR_001010) [67] with an E-value cutoff of 1e-5 was utilized to identify the best-hit protein for each sequence. Hcluster_sg with the parameter "-w 10 -s 0.34" was employed to identify one-to-one orthologous proteins among the seven species under study. A total of 241 single-copy gene families across these species were aligned using muscle version 3.8.1551(RRID: SCR_011812) [68]. Coding sequences were extracted from each single-copy gene family and concatenated to create a supergene for each species. The supergene data was then used to construct the phylogenetic tree via iqtree2 version 2.2.2.7 [69], with the parameters set as "-m MFP -B 1000".

The divergence time among seven species was estimated using the MCMCtree version 4.4 as implemented in the Phylogenetic Analysis of Maximum Likelihood (PAML) package (RRID: SCR_014932) [70], with the JC69 nucleotide substitution model and an independent rates clock. Three fossil calibration times from the TimeTree database (RRID: SCR_021162) [71] were used for calibration: 1) *C. carpio – C.auratus* 10.1 - 61.0 Mya; 2) *D. rerio - O. latipes* 180.0 - 251.5 Mya ; 3) *C. carpio - P. huangchuchieni* 81.0 - 124.7 Mya. Changes in gene family size along the phylogenetic tree were analyzed by CAFE version 4.2.1 (RRID: SCR_005983) [72].

Pathway enrichment of koi carp-specific genes, as well as genes in the expansion gene families, was conducted using KOBAS version 2.0.12 (RRID: SCR_006350) [73].

Positive selection genes

We applied a similar approach as previously reported [74] to identify positively selected genes (PSGs) within the koi carp genome. In brief, the branch-site model available in the PAML package was utilized based on the phylogenetic tree. The koi carp served as the foreground branch, while *C. auratus*, *S. grahami*, *P. huangchuch*, and *D. rerio* were designated as background branches. The null model used in the branch-site test assumed that the Ka/Ks ratios for all codons across all branches were ≤1, whereas the alternative model indicated that the foreground branch contained codons evolving with Ka/Ks >1. We conducted a maximum likelihood ratio test (LRT) to evaluate these two models. The *p*-value was derived from the chi-square distribution with 1 degree of freedom (df = 1). Subsequently, *p* values underwent adjustment for multiple comparisons using the false discovery rate (FDR) method. Genes were classified as positively selected with an FDR threshold of less than 0.05. Additionally, we required that at least one amino acid site exhibit a high probability of positive selection (Bayes probability >95%). Genes failing to satisfy this criterion at any amino acid site were designated as false positives and consequently excluded from further consideration. GO enrichment was conducted using clusterProfiler version 4.2.2 (RRID: SCR_016884) [75].

Results

T2T genome assembly and completeness evaluation

The assembly of the Taisho Sansyoku genome was achieved through the integration of diverse sequencing technologies, including Illumina whole-genome short reads, PacBio HiFi, ONT ultra-long reads, and Hi-C sequencing. In total, 70.05 Gb (~43.31×coverage) of Illumina whole-genome short reads, 223.46 Gb (~138.17×coverage) of PacBio HiFi reads, 252.59 Gb of ONT ultra-long reads (~156.18×coverage), and 219.26 Gb (~135.57×coverage) of Hi-C data (Supplementary Table S1) were generated. Notably, the N50 length surpassed 15 kb for HiFi reads and 59 kb for ONT reads (Supplementary Table S1). Through *k*-mer analysis of WGS reads, the estimated koi carp genome size was 1.62 Gb with a heterozygosity level of 0.45% (Supplementary Table S2 and Supplementary Fig. S1).

Independent assembly of the ONT reads using NextDenovo resulted in a total length of 2.00 Gb with an N50 length of 12.79 Mb (Supplementary Table S3). Furthermore, integration of ONT and HiFi reads via hifiasm yielded a total size of 1.58 Gb with an N50 length of 29.44 Mb, providing a more continuous assembly. The hifiasm initial assembly served as the backbone for scaffolding contigs into 50 pseudochromosomes using Hi-C data. Our results revealed that 34 pseudochromosomes were composed solely of a single contig, while 22 gaps were distributed across the remaining 16 pseudochromosomes (Supplementary Table S4 and Supplementary Fig. S2). After gap filling and polishing, the final koi carp assembly (termed CC 4.0) achieved a total size of 1555.86 Mb with an N50 of 30.45 Mb, comprising 50 gap-free pseudochromosomes ranging in length from 20.70 to 49.02 Mb (Fig. 2A; Table 1).

To validate the accuracy and completeness of the gap-free koi carp CC 4.0 genome assembly, multiple strategies were implemented. Firstly, the Hi-C heatmap demonstrated a high degree of consistency across all pseudochromosomes, confirming the precision in sequencing, ordering,

and orientation of contigs (Fig. 2B). Based on collinearity analysis, the koi carp CC 4.0 genome basically has a syntenic relationship and good coverage with common carp CC 3.0 genome (Supplementary Fig. S3). Secondly, all 22 gaps were successfully closed, with both HiFi and ONT reads achieving a 100% genome alignment rate, while WGS reads demonstrated a rate of 99.79%. Thirdly, the Merqury-estimated quality value of the koi carp CC 4.0 genome was 47.95, attesting to the high accuracy of the assembly (Table 1). Furthermore, 83 out of 100 telomeres were detected, resulting in 33 T2T pseudomolecules for the entire genome (Fig. 2C and Supplementary Table S5). The GCI score for the koi carp CC 4.0 genome was 85.82, comparable to the human T2T (CHM13) genome's GCI score of 87.04, indicating that the assembly quality of the koi carp CC 4.0 genome meets the current standards for T2T assembly [45]. Lastly, the Benchmarking Universal Single-Copy Orthologs (BUSCO) evaluation revealed that the koi carp CC 4.0 genome successfully identified 99.20% of the 3,640 actinopterygii gene set (Table 1). The koi carp CC 4.0 genome completeness was higher than that of the recently reported twenty-one cyprinid genomes (average 95.60%, from 91.7 to 96.6%) and the common carp CC 3.0 genome [7, 76]. Overall, these validations affirm the superior quality and reliability of the koi carp CC 4.0 genome assembly.

Annotation of repetitive elements and protein-coding genes

Approximately 696.41 Mb of the assembled koi carp CC 4.0 genome was classified as repetitive sequences, representing 44.76% of the genome (Table 1; Supplementary Table S6). The percentage of repetitive sequences was higher than the previously reported (31.3%-43.40%) [2, 3, 7]. Among the interspersed repetitive sequences, the majority consisted of DNA transposons,

making up 25.94% of the genome. (Supplementary Table S7). The long terminal repeat (LTR) and long interspersed nuclear elements classes accounted for 11.25% and 11.15% of the genome, respectively (Supplementary Table S7). Additionally, 39,065 noncoding RNAs were annotated, including 4,026 microRNAs, 24,096 transfer RNAs, 3,249 small nuclear RNAs, and 7,694 ribosomal RNAs (Supplementary Table S8).

Using a combined prediction strategy, a total of 50,187 protein-coding genes were identified, with an average of 8.87 exons per gene (Table 1). BUSCO assessment demonstrated 97.77% completeness with only 1.13% missing genes, indicating robust gene annotation (Fig. 3A). The length distribution of messenger RNA, coding sequences, exons, and introns among related species supported the reliability of the annotation results (Fig. 3B). Of the predicted genes, 49,326 (98.36%) contained at least one conserved functional domain, and 36,887 (73.50%) genes showed detectable transcriptional activity (FPKM$\geqslant$1) (Supplementary Table S9; Supplementary Table S10). In addition, 3,918 TFs were predicted across 77 types, surpassing the count in the CC 3.0 genome (3,812) [7]. The top 10 TF families with the highest gene counts were zf-H2C2_2, Homeodomain, zf-C2H2, HLH, BTB, TF-bZIP, Forkhead, HMG_box, THAP, and Myc_DNA binding (Supplementary Fig. S4). These findings affirmed the completeness and accuracy of gene prediction in the koi carp CC 4.0 genome.

The characteristics of centromeric regions

The centromeric sequences of the 50 pseudochromosomes in the koi carp CC 4.0 genome were assembled, with an average length of 748,298.76 bp (Table 2). The longest centromeric region measured 1,877,250 bp on pseudochromosome A5, while the shortest measured 30,001 bp on

pseudochromosome A14. Centromeric regions typically exhibited higher repeat sequence density and lower gene density (Fig. 1C). In summary, the average percentage of repetitive sequences in centromeric regions was 95.10%, with a total of 95 genes located in the centromeric regions. The genes located in the centromeric regions exhibited significant enrichment in ten GO terms, including DNA integration, nucleic acid binding, protein export from nucleus, nuclear export signal receptor activity, aspartic-type endopeptidase activity, motile cilium, nuclear-transcribed mRNA catabolic process, exonucleolytic, 3'-5', cell motility, proteolysis, and ubiquitin-protein transferase activity (Supplementary Fig. S5). The centromeric regions of different pseudochromosomes exhibited varying proportions of repeat elements. The centromere regions of 39 pseudochromosomes were mainly composed of satellite and simple repeat, while the remaining 11 pseudochromosomes were mainly composed of DNA transposons and LTR (Table 2).

Phylogenetic relationship analysis

The protein-coding genes from six vertebrate species, including *P.huangchuchieni*, *C.auratus*, *D.rerio*, *O.latipes*, *S.grahami*, and *H.sapiens*, were clustered into 18,442 gene families together with the protein-coding genes of koi carp CC 4.0 genome (Supplementary Table S11; Supplementary Table S12). Among these, 12,320 gene families were shared among *P.huangchuchieni*, *C.auratus*, *S.grahami*, and *C. carpio* (Fig. 4A). Additionally, 245 gene families with 589 genes were identified as specific to *C. carpio* when compared to the other six species (Fig. 4B). Of these koi carp-specific genes, 545 (92.53%) had functional annotations (Supplementary Table S13). These specific genes were significantly enriched in six pathways:

"Fructose and mannose metabolism", "Caffeine metabolism", "Phosphatidylinositol signaling system", "Thyroid hormone signaling pathway", "AMPK signaling pathway", and "Glycerolipid metabolism" (Fig. 4C).

A phylogenetic tree was constructed using 241 single-copy orthologous genes, with *H. sapiens* as the outgroup (Fig. 4D). The estimated divergence time between *C. carpio* and *C. auratus* was approximately 34.7 million years ago (MYA). Compared to the most recent common ancestor (MRCA), koi carp exhibited 87 expansion and 66 contraction events in gene families ($p <= 0.05$). The expanded gene families in common carp included 1,420 genes and were primarily enriched in 13 pathways, such as "Olfactory transduction", "RNA degradation", "NOD-like receptor signaling pathway", "Neuroactive ligand-receptor interaction", "Necroptosis", "Mineral absorption", "Ferroptosis", "Complement and coagulation cascades", "Glycerophospholipid metabolism", "TNF signaling pathway", "Apoptosis", "Gap junction", and "Cholinergic synapse" (Fig. 4C). Notably, the immune genes in koi carp identified by genome-wide association analysis was reported to involve in several immune response-related pathways, including NOD-like receptor signaling pathway [10].

Positively selected genes

A total of 3,438 one-to-one orthologous gene sets in five teleost fish (*C. auratus*, *C.carpio*, *S. grahami*, *P. huangchuchieni*, and *D. rerio*) were analyzed for positive selection gene (PSG) detection analysis. Ultimately, 124 genes were identified as PSGs (Supplementary Table S14). These PSGs were linked to various biological processes, including binding (GO:0005488; 58 genes), cellular process (GO:0009987; 46 genes), catalytic activity (GO:0003824, 43 genes),

single-organism process (GO:0044699; 40 genes), and others (Fig. 5A). GO enrichment analysis revealed that these genes were significantly associated with enzyme activities like metallopeptidase activity, metalloendopeptidase activity, methyltransferase activity, and RNA helicase activity, and so on (Fig. 5B). The metalloendopeptidase has been reported to play a significant role in the central nervous system and has been associated with various diseases including breast cancer, prostate cancer, and essential hypertension [77-80].

Conclusions

The first T2T genome assembly of koi carp was achieved using PacBio HiFi reads, ONT ultra-long sequencing, and Hi-C technologies, characterized by high completeness and accuracy. A total of 50 pseudochromosomes were assembled, with 33 meeting the T2T standard. The assembly predicted 696.41 Mb of repetitive sequences and identified 50,187 protein-coding genes. In addition, 3,918 TFs were predicted. Comparative genomics analysis revealed 589 genes specific to koi carp. Moreover, 87 expansion and 66 contraction events were obtained. Evolutionary analysis suggested that metalloendopeptidase activity may be crucial for koi carp. This dataset serves as a valuable resource for future genetic breeding research in koi carp and common carp.

Abbreviations

BLAST: Basic Local Alignment Search Tool; BUSCO: Benchmarking Universal Single-Copy Orthologs; CCS: circular consensus sequencing; Gb: gigabase pairs; GCI: Genome Continuity Inspector; GO: Gene Ontology; Hi-C: High-Throughput Chromosome Conformation Capture;

HiFi: High-Fidelity; KEGG: Kyoto Encyclopedia of Genes and Genomes; LINE: long interspersed nuclear element; LTR: long terminal repeat; Mb: megabase pairs; MRCA: most recent common ancestor; MYA: million years ago; PSG: positively selected gene; QV: quality value; RNA-seq: RNA sequencing; PASA: Program to Assemble Spliced Alignments; WGD: whole genome duplication; WGS: whole-genome sequencing; T2T: telomere-to-telomere

Tables

Table 1. Statistics for the common carp and koi carp genome assembly.

Genomic feature	Koi carp (CC 4.0)	Common carp (CC 3.0)
Total size (Mb)	1555.86	1579.38
Number of chromosomes	50	50
**Gap number**	**0**	**1,089**
Chromosome N50 (Mb)	30.45	28.32
GC content (%)	37.20	37.20
Protein-coding genes number	50,187	55,981
Repetitive sequences (%)	44.76	43.40
Genome BUSCOs (%)	99.20	99.10
GCI score	85.82	NA
WGS reads mapping rate (%)	99.79	NA
ONT reads mapping rate (%)	100.00	NA
HiFi reads mapping rate (%)	100.00	NA
Quality value	47.95	NA

Note: the common carp CC 3.0 was downloaded from Genome Warehouse database under accession GWHBHRW00000000. NA means not avaiable.

Table 2. The characteristic of centromeric regions of the koi carp CC 4.0 assembly.

Chr	Start	End	Length	Gene number	Total	Trf	Satellite	Simple repeat	DNA transposons	LTR
A1	6,983,780	7,145,651	161,872	2	81.31	60.02	0.00	0.00	13.97	54.67
A2	10,552,693	11,217,077	664,385	0	99.80	97.66	34.62	81.27	0.17	10.08
A3	31,879,419	33,636,405	1,756,987	15	94.48	47.82	0.52	0.01	23.15	19.92
A4	21,620,000	21,850,000	230,001	0	99.51	91.05	38.95	68.48	0.20	19.43
A5	32,599,339	34,476,588	1,877,250	4	95.23	78.50	37.86	38.32	19.57	18.10
A6	11,508,992	12,370,832	861,841	0	97.97	93.41	32.03	76.06	1.69	18.41

A7	11,516,427	13,371,720	1,855,294	5	97.97	92.18	32.58	34.23	4.61	12.75
A8	24,955,543	25,632,915	677,373	2	98.40	92.83	10.21	81.02	1.14	5.10
A9	29,694,335	30,547,340	853,006	4	90.68	74.76	73.54	0.00	9.05	6.00
A10	22,177,277	22,829,639	652,363	0	99.55	77.31	32.70	84.61	0.16	5.35
A11	4,746,905	5,590,589	843,685	0	96.67	69.23	69.71	29.75	0.32	0.52
A12	17,378,483	18,406,913	1,028,431	2	95.07	84.22	46.46	50.69	8.00	3.01
A13	4,680,000	4,900,000	220,001	0	90.67	89.23	78.71	11.37	0.00	0.13
A14	23,400,000	23,430,000	30,001	0	94.82	91.60	63.58	0.00	1.36	0.00
A15	19,963,423	21,668,624	1,705,202	2	95.22	82.73	42.88	51.19	8.10	5.78
A16	14,555,504	14,717,133	161,630	1	88.13	21.87	0.00	0.00	86.60	1.64
A17	3,716,890	4,150,698	433,809	3	99.97	62.40	1.99	0.00	0.26	29.53
A18	11,933,667	12,590,459	656,793	2	95.02	89.14	27.61	78.28	2.76	9.08
A19	2,597,145	3,177,452	580,308	2	96.10	94.22	21.03	87.82	1.42	6.54
A20	23,703,082	24,668,971	965,890	5	88.97	69.01	66.65	0.01	8.92	14.15
A21	16,706,975	17,545,549	838,575	1	95.78	88.81	16.58	82.56	3.99	8.95
A22	19,320,532	19,979,681	659,150	0	98.32	96.21	39.26	79.33	0.77	10.22
A23	1,348,620	2,293,930	945,311	1	100.00	98.81	57.69	52.65	0.00	17.76
A24	16,939,420	17,124,316	184,897	4	88.30	7.58	0.00	0.00	48.20	42.52
A25	18,285,858	19,188,655	902,798	0	97.68	91.44	69.84	24.54	0.86	7.48
B1	15,950,000	16,440,000	490,001	0	99.88	95.39	17.92	53.81	0.00	0.00
B2	22,023,874	22,670,773	646,900	6	90.32	72.71	73.80	0.00	11.34	5.13
B3	35,614,767	35,952,947	338,181	0	97.14	91.61	88.11	17.69	5.52	1.09
B4	33,360,418	33,527,013	166,596	0	99.39	97.34	0.00	0.00	0.33	0.00
B5	11,034,427	12,148,201	1,113,775	1	94.60	83.31	72.53	9.66	7.56	6.22
B6	16,740,439	17,090,269	349,831	1	96.16	93.15	0.00	0.00	1.17	15.35
B7	12,778,416	13,657,366	878,951	1	99.24	71.75	29.25	74.78	0.70	14.21
B8	2,758,449	3,284,545	526,097	2	93.21	86.27	55.03	49.18	2.79	14.59
B9	4,331,995	4,452,008	120,014	0	75.44	42.01	0.00	0.00	30.85	36.93
B10	2,079,927	2,213,557	133,631	0	95.15	78.41	0.54	50.58	5.73	50.44
B11	21,120,000	22,240,000	1,120,001	0	99.59	99.48	38.50	81.92	0.09	3.06
B12	15,879,559	17,438,428	1,558,870	6	95.57	85.19	39.74	67.93	8.58	9.30
B13	25,361,200	26,195,054	833,855	4	86.95	56.30	50.37	11.58	18.10	12.22
B14	23,790,000	24,620,000	830,001	1	94.93	12.26	13.52	79.35	0.76	18.71
B15	11,292,907	11,974,689	681,783	3	97.13	2.80	11.85	0.00	12.58	1.83
B16	24,380,000	25,210,000	830,001	1	99.77	98.92	38.71	84.91	0.19	7.52
B17	23,480,000	24,130,000	650,001	1	96.47	77.13	62.70	43.82	0.18	9.51
B18	10,339,546	11,415,040	1,075,495	8	89.51	59.19	44.49	0.59	24.08	17.50
B19	26,221,041	26,766,840	545,800	1	98.22	90.31	72.13	30.39	2.66	5.52
B20	4,550,000	4,900,000	350,001	0	98.63	98.56	27.54	95.27	0.13	0.00
B21	8,310,000	9,300,000	990,001	0	97.21	2.16	50.79	74.63	0.00	7.16
B22	36,566,575	37,323,917	757,343	1	97.09	92.48	23.27	67.10	5.81	22.69
B23	1,540,000	2,230,000	690,001	0	93.82	0.15	21.82	81.62	0.26	7.16
B24	16,763,174	17,679,191	916,018	3	94.99	84.22	37.99	54.93	10.27	7.39
B25	1,437,545	2,512,481	1,074,937	0	98.86	96.59	39.80	76.90	2.90	1.04

Additional Files

Supplementary Table S1. Summary of the data sequenced by multiple technologies.

Supplementary Table S2. *k*-mer analysis.

Supplementary Table S3. The statistics of initial assembly.

Supplementary Table S4. The statistics of the anchored chromosome length.

Supplementary Table S5. The identified telomeres in CC 4.0 assembly.

Supplementary Table S6. General statistics of repeats in CC 4.0 assembly.

Supplementary Table S7. The summary of interspersed repeat contents in CC 4.0 assembly.

Supplementary Table S8. Non-coding RNAs in CC 4.0 assembly.

Supplementary Table S9. Summary of gene function annotation.

Supplementary Table S10. The gene expression matrix.

Supplementary Table S11. The data sources of six vertebrate genomes.

Supplementary Table S12. Statistics for the orthologous gene families of seven species genomes.

Supplementary Table S13. The list of koi carp-specific genes.

Supplementary Table S14. The list of 124 positive selection genes.

Author Contributions

Jiandong Yuan, Jun Yong, and Yongchao Niu designed this study; Jun Yong, Huijuan Guo and Xuewu Liao collected the samples and performed the experiments; Jiang Li and Yongchao Niu performed the data analysis; Jiandong Yuan, Jiang Li, and Yongchao Niu wrote the first draft of

the manuscript. All other authors proofread and revised the manuscript. All authors read and approved the final manuscript.

Funding

This study was supported by Suxin Koi Farm.

Data Availability

The raw sequencing data that support the findings of this study have been deposited into the CNGB Sequence Archive (CNSA) of China National GeneBank DataBase (CNGBdb) with accession number CNP0006400.

Competing Interests

The authors declare that they have no competing interests.

Acknowledgements

We thank every project that provides funding and material support for the study. We also thank each author for their ideas and skills in study design, experimentation, data collection, data analysis, and manuscript writing.

Ethics statement

This study was carried out in accordance with the recommendations of the care and use of animals for scientific purposes set up by the Animal Care and Use Committee of Chinese

Academy of Fishery Sciences (ACUC-CAFS).

References

1. Bostock, J., et al., *Aquaculture: global status and trends.* Philosophical transactions of the Royal Society of London. Series B, Biological sciences, 2010. **365**(1554): p. 2897-2912.
2. Xu, P., et al., *Genome sequence and genetic diversity of the common carp, Cyprinus carpio.* Nature Genetics, 2014. **46**(11): p. 1212-1219.
3. Xu, P., et al. *The allotetraploid origin and asymmetrical genome evolution of the common carp Cyprinus carpio*. Nature communications, 2019. **10**, 4625 DOI: 10.1038/s41467-019-12644-1.
4. Ren, R., et al., *Widespread Whole Genome Duplications Contribute to Genome Complexity and Species Diversity in Angiosperms.* Molecular plant, 2018. **11**(3): p. 414-428.
5. Chen, L., et al., *Chromosome-level genome of Poropuntius huangchuchieni provides a diploid progenitor-like reference genome for the allotetraploid Cyprinus carpio.* Molecular ecology resources, 2021. **21**(5): p. 1658-1669.
6. Li, J.-T., et al., *Parallel subgenome structure and divergent expression evolution of allo-tetraploid common carp and goldfish.* Nature Genetics, 2021. **53**(10): p. 1493-1503.
7. Chen, L., et al. *Evolutionary divergence of subgenomes in common carp provides insights into speciation and allopolyploid success*. Fundamental research, 2024. **4**, 589-602 DOI: 10.1016/j.fmre.2023.06.011.
8. Zhang, Y., et al. *Identification of common carp innate immune genes with whole-genome sequencing and RNA-Seq data*. Journal of integrative bioinformatics, 2011. **8**, 169 DOI: 10.2390/biecoll-jib-2011-169.
9. Verma, D.K., et al., *Transcriptome analysis reveals immune pathways underlying resistance in the common carp Cyprinus carpio against the oomycete Aphanomyces invadans.* Genomics, 2021. **113**(1, Part 2): p. 944-956.
10. Jiang, Y., et al., *Genomic features of common carp that are relevant for resistance against Aeromonas hydrophila infection.* Aquaculture, 2022. **547**: p. 737512.
11. Wang, J., Q. Zhou, and Y. Jiang, *Genome-wide analysis of common carp (Cyprinus carpio) mucin genes and their roles in mucosal immune response following the Aeromonas hydrophila infection.* Comparative Immunology Reports, 2024. **7**: p. 200167.
12. Nurk, S., et al., *The complete sequence of a human genome.* Science (New York, N.Y.), 2022. **376**(6588): p. 44-53.
13. Luo, L.-Y., et al., *Telomere-to-telomere sheep genome assembly reveals new variants associated with wool fineness trait*. 2024, bioRxiv.
14. You, X., et al., *A near complete genome assembly of the East Friesian sheep genome.* Scientific Data, 2024. **11**(1): p. 762.
15. Shang, L., et al., *A complete assembly of the rice Nipponbare reference genome.* Molecular plant, 2023. **16**(8): p. 1232-1236.
16. Chen, J., et al., *A complete telomere-to-telomere assembly of the maize genome.* Nature genetics, 2023. **55**(7): p. 1221-1231.
17. Li, M., et al. *Telomere-to-telomere genome assembly of sorghum*. Scientific data, 2024. **11**, 835 DOI: 10.1038/s41597-024-03664-8.
18. Li, H. and R. Durbin, *Genome assembly in the telomere-to-telomere era.* Nature Reviews Genetics, 2024. **25**(9): p. 658-670.

19. Garg, V., et al., *Unlocking plant genetics with telomere-to-telomere genome assemblies.* Nature Genetics, 2024.
20. Chin, C., et al., *Nonhybrid, finished microbial genome assemblies from long-read SMRT sequencing data.* Nature Methods, 2013. **10**: p. 563-569.
21. De Coster, W., et al., *NanoPack: visualizing and processing long-read sequencing data.* Bioinformatics, 2018. **34**: p. 2666 - 2669.
22. Belton, J.-M., et al., *Hi-C: a comprehensive technique to capture the conformation of genomes.* Methods, 2012. **58 3**: p. 268-76.
23. Chen, S., et al., *fastp: an ultra-fast all-in-one FASTQ preprocessor.* Bioinformatics, 2018. **34**(17): p. i884-i890.
24. Sun, C., et al., *Chromosome-level genome assembly for the largemouth bass Micropterus salmoides provides insights into adaptation to fresh and brackish water.* Molecular ecology resources, 2021. **21**(1): p. 301-315.
25. Marçais, G. and C. Kingsford, *A fast, lock-free approach for efficient parallel counting of occurrences of k-mers.* Bioinformatics, 2011. **27 6**: p. 764-70.
26. Liu, B., et al., *Estimation of genomic characteristics by analyzing k-mer frequency in de novo genome projects.* arXiv: Genomics, 2013.
27. Hu, J., et al., *NextDenovo: an efficient error correction and accurate assembly tool for noisy long reads.* Genome Biology, 2024. **25**(1): p. 107.
28. Cheng, H., et al., *Haplotype-resolved de novo assembly using phased assembly graphs with hifiasm.* Nature Methods, 2021. **18**(2): p. 170-175.
29. Hu, J., et al., *NextPolish2:a repeat-aware polishing tool for genomes assembled using HiFi long reads*. 2023, bioRxiv.
30. Langmead, B. and S.L. Salzberg, *Fast gapped-read alignment with Bowtie 2.* Nature Methods, 2012. **9**(4): p. 357-359.
31. Servant, N., et al., *HiC-Pro: An optimized and flexible pipeline for Hi-C data processing.* Genome Biology, 2015. **16**.
32. Durand, N., et al., *Juicer Provides a One-Click System for Analyzing Loop-Resolution Hi-C Experiments.* Cell Systems, 2016. **3**: p. 95-98.
33. Dudchenko, O., et al., *De novo assembly of the Aedes aegypti genome using Hi-C yields chromosome-length scaffolds.* Science, 2017. **356**: p. eaal3327.
34. Durand, N.C., et al., *Juicebox Provides a Visualization System for Hi-C Contact Maps with Unlimited Zoom.* Cell systems, 2016. **3**(1): p. 99-101.
35. Zhao, H., et al. *Telomere-to-telomere genome assembly of the goose Anser cygnoides*. Scientific data, 2024. **11**, 741 DOI: 10.1038/s41597-024-03567-8.
36. Lin, Y., et al., *quarTeT: a telomere-to-telomere toolkit for gap-free genome assembly and centromeric repeat identification.* Horticulture Research, 2023.
37. Xu, G.-C., et al., *LR_Gapcloser: a tiling path-based gap closer that uses long reads to complete genome assembly.* GigaScience, 2018. **8**.
38. Zhou, Y., et al. *Gap-free genome assembly of Salangid icefish Neosalanx taihuensis*. Scientific data, 2023. **10**, 768 DOI: 10.1038/s41597-023-02677-z.
39. Jain, C., et al., *Long-read mapping to repetitive reference sequences using Winnowmap2.* Nature methods, 2022. **19**(6): p. 705-710.
40. Vaser, R., et al., *Fast and accurate de novo genome assembly from long uncorrected reads.* Genome research, 2017. **27 5**: p. 737-746.

41. Seppey, M., M. Manni, and E.M. Zdobnov, *BUSCO: Assessing Genome Assembly and Annotation Completeness.* Methods in molecular biology (Clifton, N.J.), 2019. **1962**: p. 227-245.
42. Rhie, A., et al., *Merqury: reference-free quality, completeness, and phasing assessment for genome assemblies.* Genome Biology, 2020. **21**.
43. Li, H. and R. Durbin, *Fast and accurate short read alignment with Burrows–Wheeler transform.* Bioinformatics, 2009. **25**(14): p. 1754-1760.
44. Li, H., *Minimap2: pairwise alignment for nucleotide sequences.* Bioinformatics, 2018. **34**(18): p. 3094-3100.
45. Chen, Q., et al., *GCI: a continuity inspector for complete genome assembly.* Bioinformatics, 2024. **40**(11).
46. Marçais, G., et al., *MUMmer4: A fast and versatile genome alignment system.* PLoS Computational Biology, 2018. **14**.
47. Benson, G., *Tandem repeats finder: a program to analyze DNA sequences.* Nucleic acids research, 1999. **27 2**: p. 573-80.
48. Flynn, J.M., et al., *RepeatModeler2 for automated genomic discovery of transposable element families.* Proceedings of the National Academy of Sciences of the United States of America, 2020. **117**(17): p. 9451-9457.
49. Xu, Z. and H. Wang, *LTR_FINDER: an efficient tool for the prediction of full-length LTR retrotransposons.* Nucleic Acids Research, 2007. **35**: p. W265 - W268.
50. Chen, N., *Using RepeatMasker to Identify Repetitive Elements in Genomic Sequences.* Current Protocols in Bioinformatics, 2004. **5**.
51. Bao, W., K.K. Kojima, and O. Kohany, *Repbase Update, a database of repetitive elements in eukaryotic genomes.* Mobile DNA, 2015. **6**.
52. You, X., et al. *A near complete genome assembly of the East Friesian sheep genome*. Scientific data, 2024. **11**, 762 DOI: 10.1038/s41597-024-03581-w.
53. Grabherr, M.G., et al., *Full-length transcriptome assembly from RNA-Seq data without a reference genome.* Nature biotechnology, 2011. **29 7**: p. 644-52.
54. Haas, B., *Improving the Arabidopsis genome annotation using maximal transcript alignment assemblies.* Nucleic Acids Research, 2003. **31**: p. 5654-5666.
55. Kim, D., B. Langmead, and S.L. Salzberg, *HISAT: a fast spliced aligner with low memory requirements.* Nature Methods, 2015. **12**(4): p. 357-360.
56. Kovaka, S., et al., *Transcriptome assembly from long-read RNA-seq alignments with StringTie2.* Genome Biology, 2019. **20**(1): p. 278.
57. Jens, et al., *GeMoMa: Homology-Based Gene Prediction Utilizing Intron Position Conservation and RNA-seq Data.* Methods in Molecular Biology, 2019.
58. Holst, F., et al., *Helixer–de novo Prediction of Primary Eukaryotic Gene Models Combining Deep Learning and a Hidden Markov Model.* bioRxiv, 2023.
59. Haas, B.J., et al., *Automated eukaryotic gene structure annotation using EVidenceModeler and the Program to Assemble Spliced Alignments.* Genome Biology, 2008. **9**(1): p. R7.
60. Buchfink, B., C. Xie, and D.H. Huson, *Fast and sensitive protein alignment using DIAMOND.* Nature Methods, 2015. **12**(1): p. 59-60.
61. Bairoch, A. and R. Apweiler, *The SWISS-PROT protein sequence data bank and its supplement TrEMBL in 1999.* Nucleic Acids Research, 1999. **27**(1): p. 49-54.
62. Kanehisa, M. and S. Goto, *KEGG: Kyoto Encyclopedia of Genes and Genomes.* Nucleic Acids Research, 2000. **28**(1): p. 27-30.
63. Jones, P., et al., *InterProScan 5: genome-scale protein function classification.* Bioinformatics, 2014. **30**(9): p. 1236-1240.

64. Wu, Y.-M., et al. *Investigation of sex expression profiles and the cantharidin biosynthesis genes in two blister beetles*. PloS one, 2023. **18**, e0290245 DOI: 10.1371/journal.pone.0290245.
65. Shen, W.-K., et al., *AnimalTFDB 4.0: a comprehensive animal transcription factor database updated with variation and expression annotations.* Nucleic acids research, 2023. **51**(D1): p. D39-D45.
66. Li, H., et al., *TreeFam: a curated database of phylogenetic trees of animal gene families.* Nucleic acids research, 2006. **34**(Database issue): p. D572-80.
67. Altschul, S.F., et al., *Basic local alignment search tool.* Journal of Molecular Biology, 1990. **215**(3): p. 403-410.
68. Edgar, R.C., *Muscle5: High-accuracy alignment ensembles enable unbiased assessments of sequence homology and phylogeny.* Nature Communications, 2022. **13**(1): p. 6968.
69. Minh, B.Q., et al., *IQ-TREE 2: New Models and Efficient Methods for Phylogenetic Inference in the Genomic Era.* Molecular Biology and Evolution, 2019. **37**: p. 1530 - 1534.
70. Yang, Z., *PAML: a program package for phylogenetic analysis by maximum likelihood.* Computer applications in the biosciences : CABIOS, 1997. **13 5**: p. 555-6.
71. Hedges, S.B., J.T. Dudley, and S. Kumar, *TimeTree: a public knowledge-base of divergence times among organisms.* Bioinformatics, 2006. **22 23**: p. 2971-2.
72. Bie, T.D., et al., *CAFE: a computational tool for the study of gene family evolution.* Bioinformatics, 2006. **22 10**: p. 1269-71.
73. Xie, C., et al., *KOBAS 2.0: a web server for annotation and identification of enriched pathways and diseases.* Nucleic acids research, 2011. **39**(Web Server issue): p. W316-22.
74. Wang, Y., et al., *Genomic insights into the seawater adaptation in Cyprinidae.* BMC Biology, 2024. **22**(1): p. 87.
75. Wu, T., et al., *clusterProfiler 4.0: A universal enrichment tool for interpreting omics data.* The Innovation, 2021. **2**.
76. Xu, M.-R.-X., et al., *Maternal dominance contributes to subgenome differentiation in allopolyploid fishes.* Nature Communications, 2023. **14**.
77. Ding, J., et al. *Membrane metalloendopeptidase (MME) is positively correlated with systemic lupus erythematosus and may inhibit the occurrence of breast cancer*. PloS one, 2023. **18**, e0289960 DOI: 10.1371/journal.pone.0289960.
78. Cheng, C.-Y., et al., *Membrane metalloendopeptidase suppresses prostate carcinogenesis by attenuating effects of gastrin-releasing peptide on stem/progenitor cells.* Oncogenesis, 2020. **9**(3): p. 38.
79. Moskalenko, M., et al., *Polymorphisms of the matrix metalloproteinase genes are associated with essential hypertension in a Caucasian population of Central Russia.* Scientific Reports, 2021. **11**(1): p. 5224.
80. Cervellini, I., et al., *Membrane metallo-endopeptidase is dispensable for repair after nerve injury.* Glia, 2019. **67**(10): p. 1990-2000.

Figures and Legends

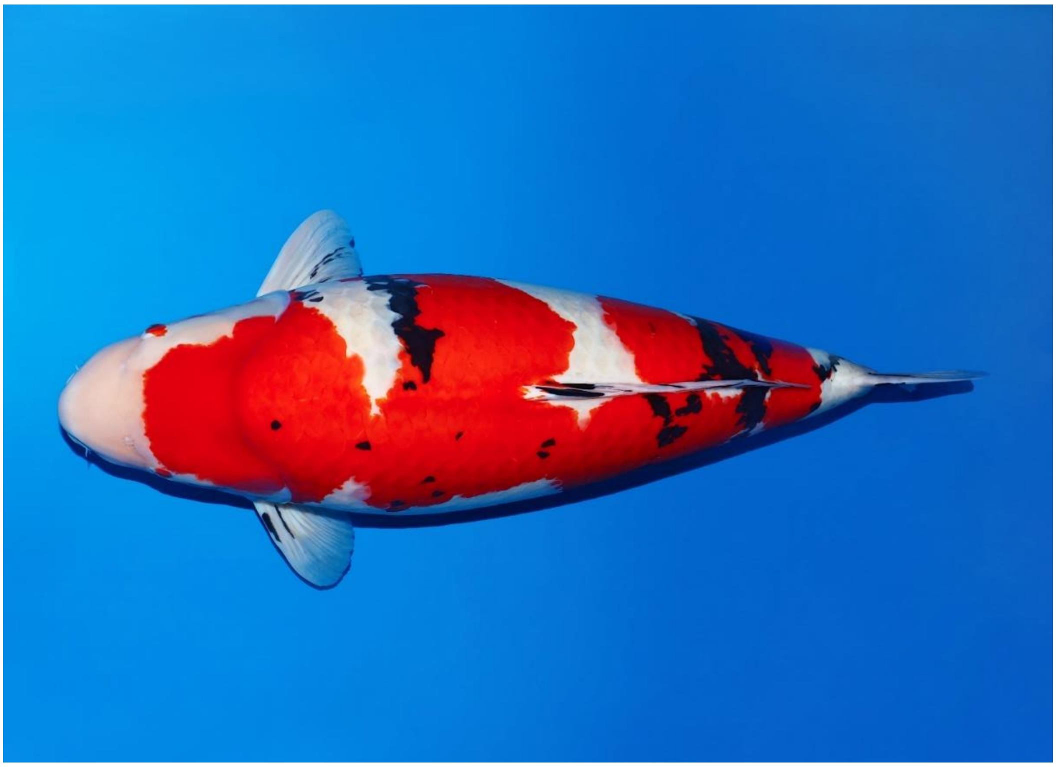

Fig. 1 | The koi carp strain Taisho Sansyoku in this study.

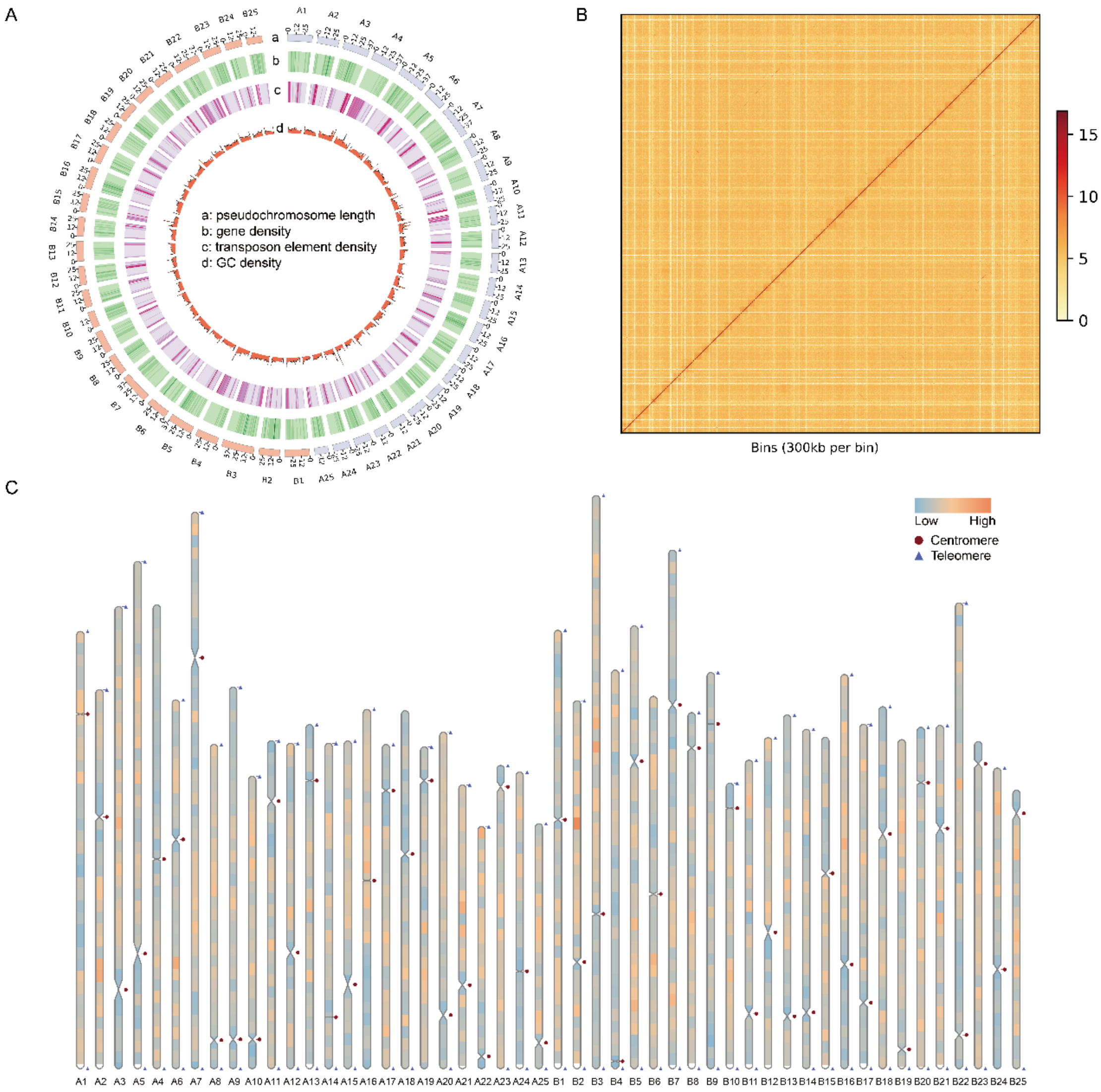

Fig. 2 | Genomic characteristics of the koi carp (CC 4.0) genome. A, Circos plot showing the characterization of CC 4.0 genome. From outside to inside: a, The length of pseudo-chromosome in the size of Mb. b, gene density in 1-Mb sliding windows. c, percentage of transposon elements in 1 Mb sliding windows. d, GC content in non-overlapping 1Mb windows. **B**, Intensity signal heat map of the Hi-C chromosome interaction. The colour block illuminates the intensity of interaction from yellow (low) to red (high). **C**, Telomere and centromere detection map. Triangles and circles represent telomeres and centromere within the CC 4.0 assembled

chromosomes. The orange color represents regions with high gene density, while the sky blue color represents regions with low gene density.

A

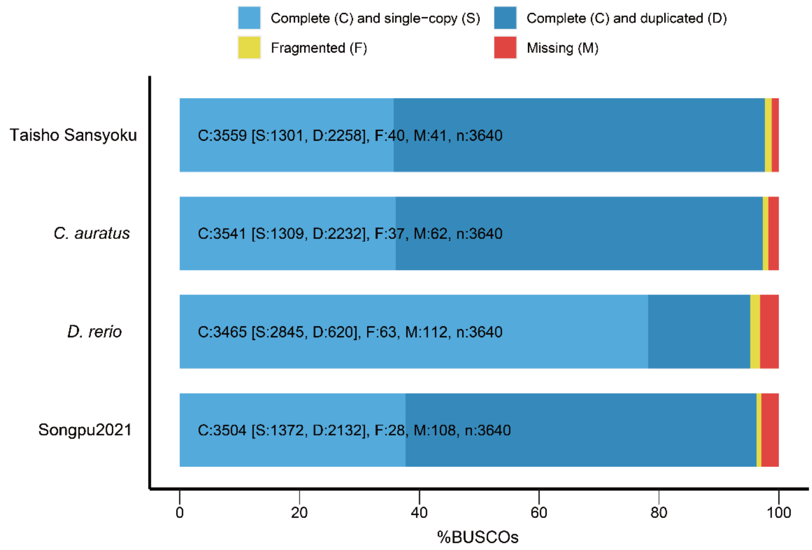

B

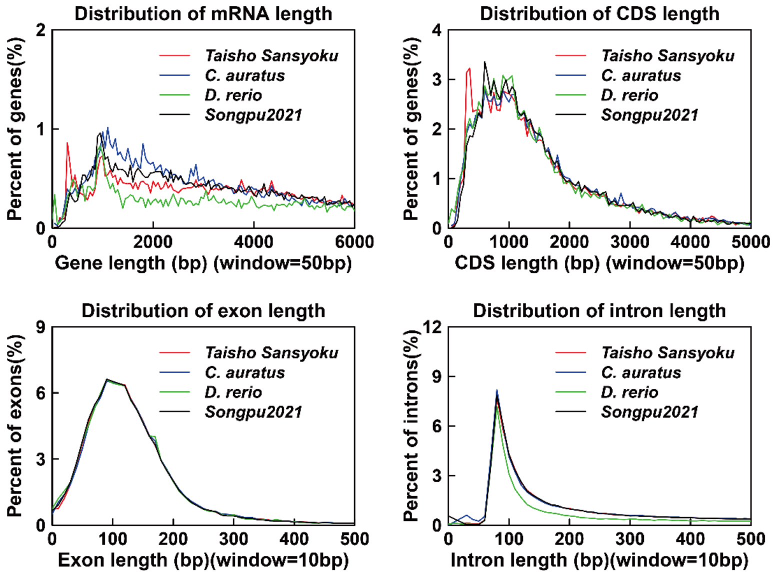

Fig. 3 | The high-quality of gene annotation. A, BUSCO assessments of Taisho Sansyoku, *C. auratus*, *D. rerio*, and Songpu2021. **B,** The composition of gene elements in the koi carp CC 4.0 genome compared to the other three genomes.

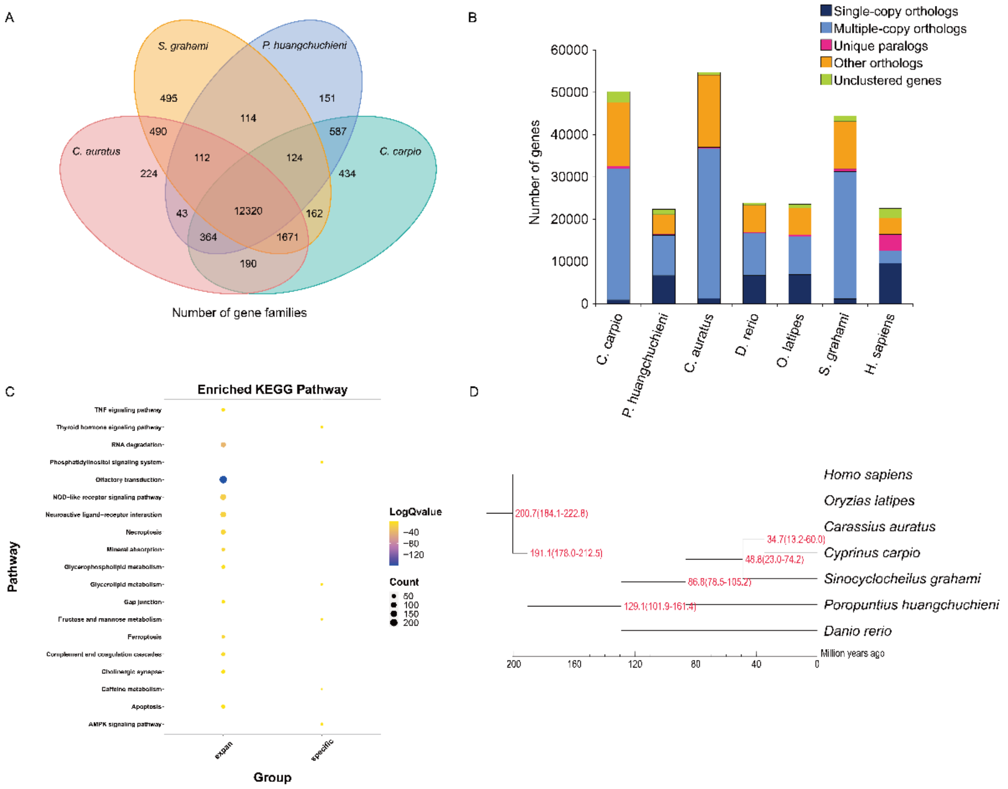

Fig. 4 | Evolution of the koi carp (CC 4.0) genome. A, Venn diagram of orthologous gene families in four genomes. The numbers represent quantities of gene families. **B,** Number of orthologous genes in seven species. **C,** Enrichment analysis of KEGG signaling pathway of specific genes and expansion gene families belongs to the CC 4.0 assembly. The size of the dots in the graph indicates the number of genes enriched in the pathway. The color indicates the significant Qvalue of the pathway. **D,** Phylogenetic tree constructed using conserved house-keeping proteins from seven species.

A

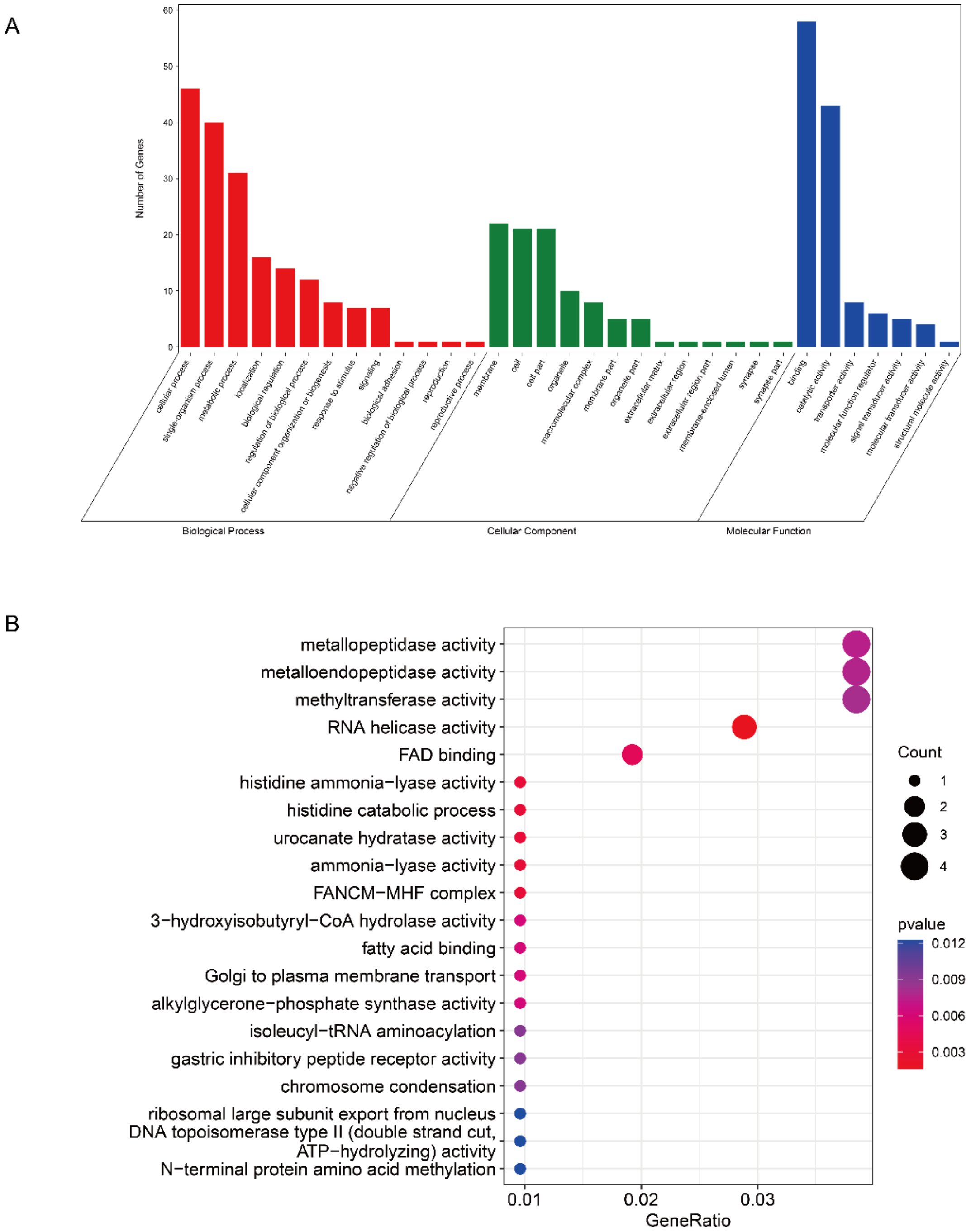

Fig. 5 | The GO function of PSGs in koi carp (CC 4.0) genome. A, WEGO (Web Gene Ontology Annotation Plotting) plot showing GO distribution of PSGs. **B,** GO enrichment analysis of PSGs. The bubble size indicates the gene number of a biological process GO term, with color maps the *p*value of the enrichment analysis. GeneRatio: number of DEGs annotated to the GO category/total number of DEGs.

Figure 1

Click here to access/download;Figure;Figure 1.jpg

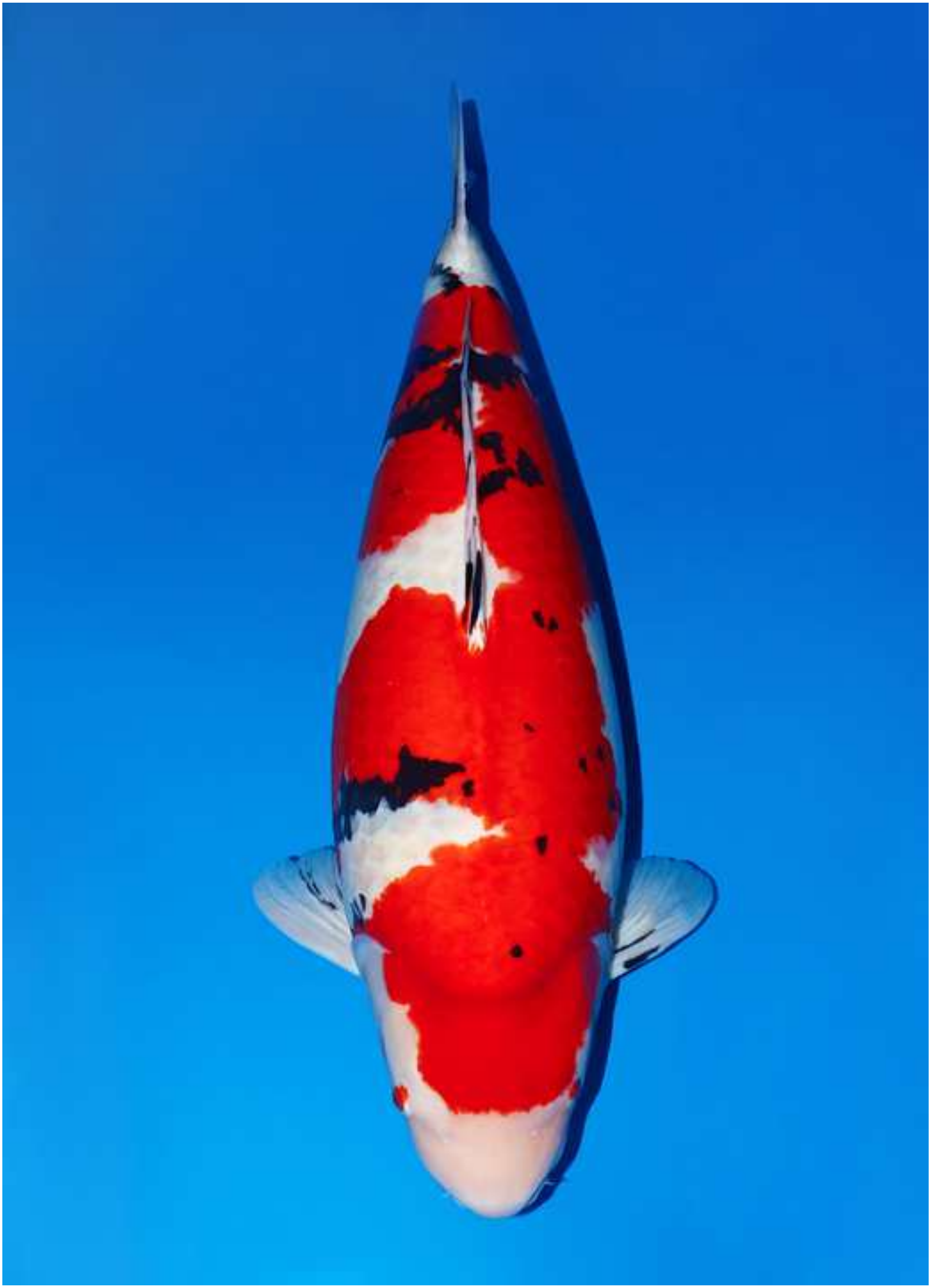

Figure 2

Click here to access/download;Figure;Figure 2.pdf

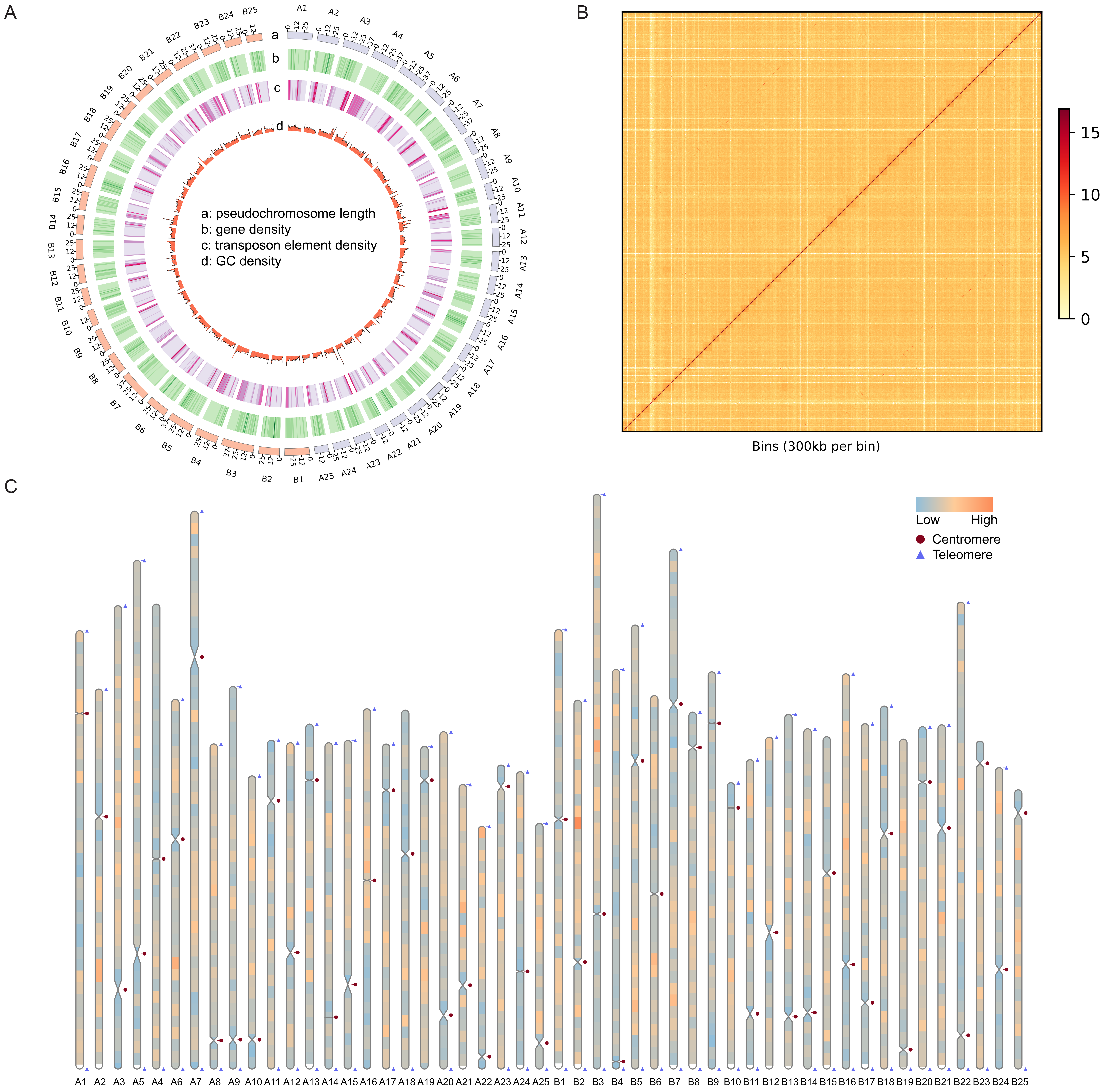

Click here to access/download;Figure;Figure 3.pdf

A

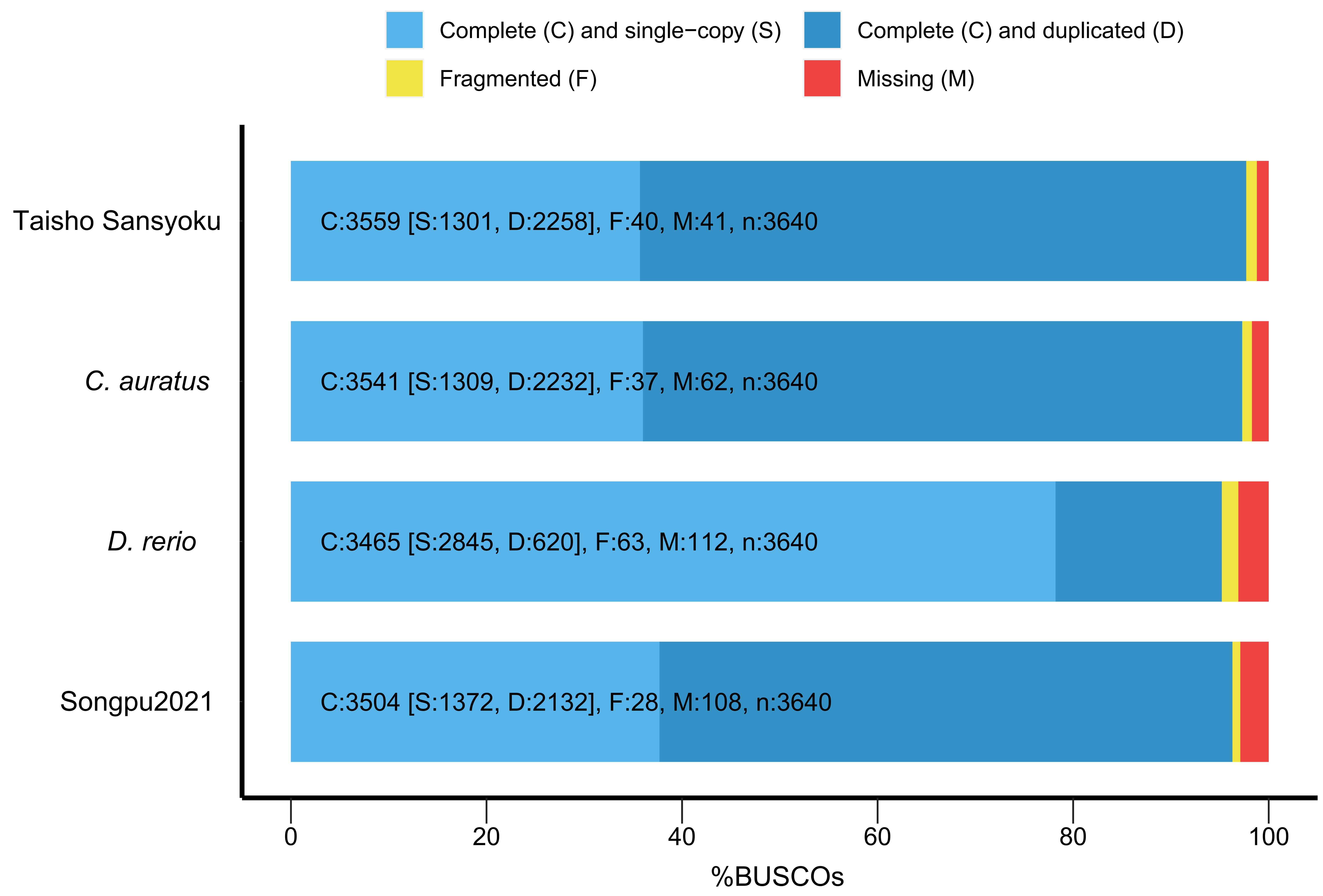

B

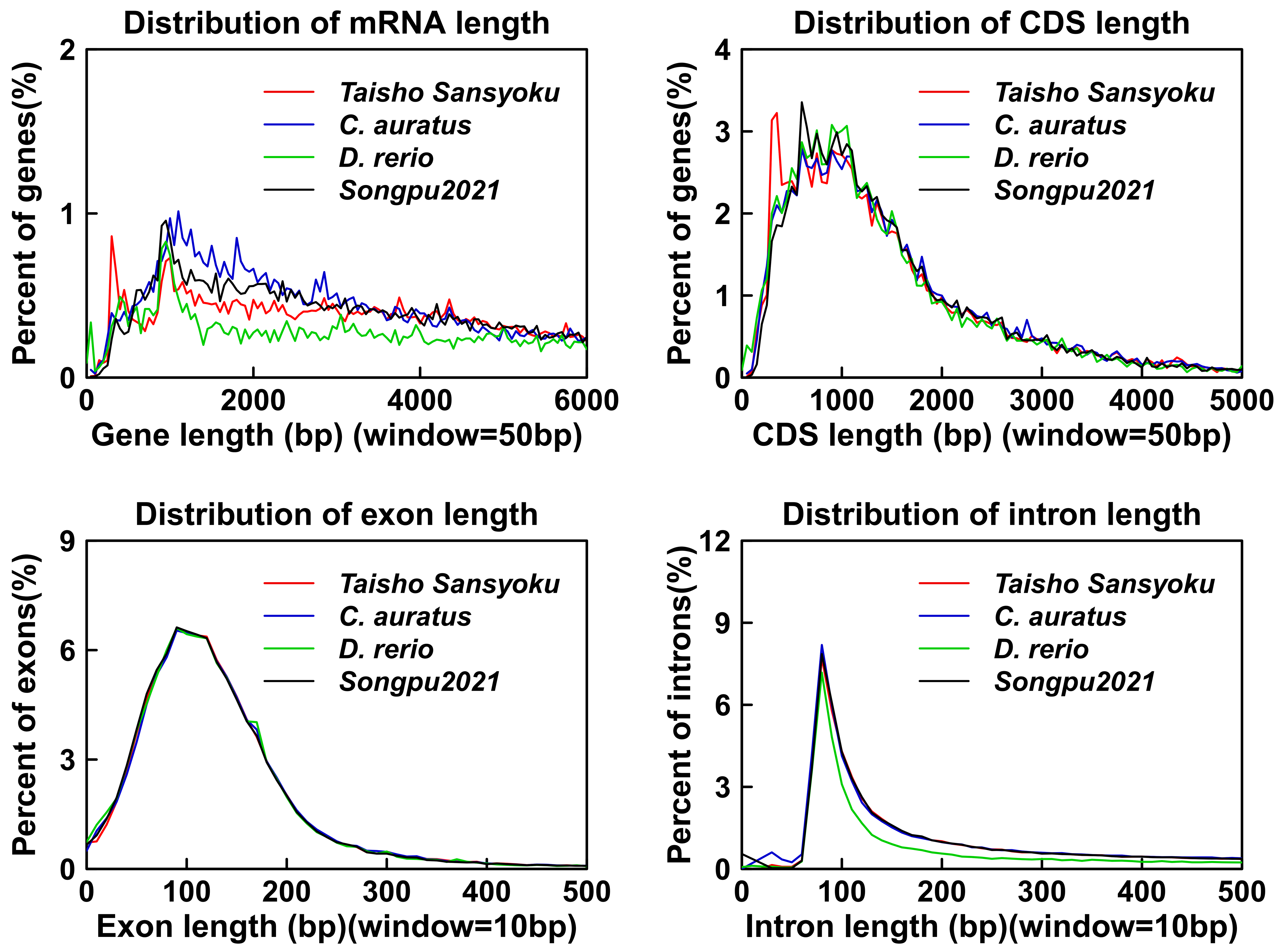

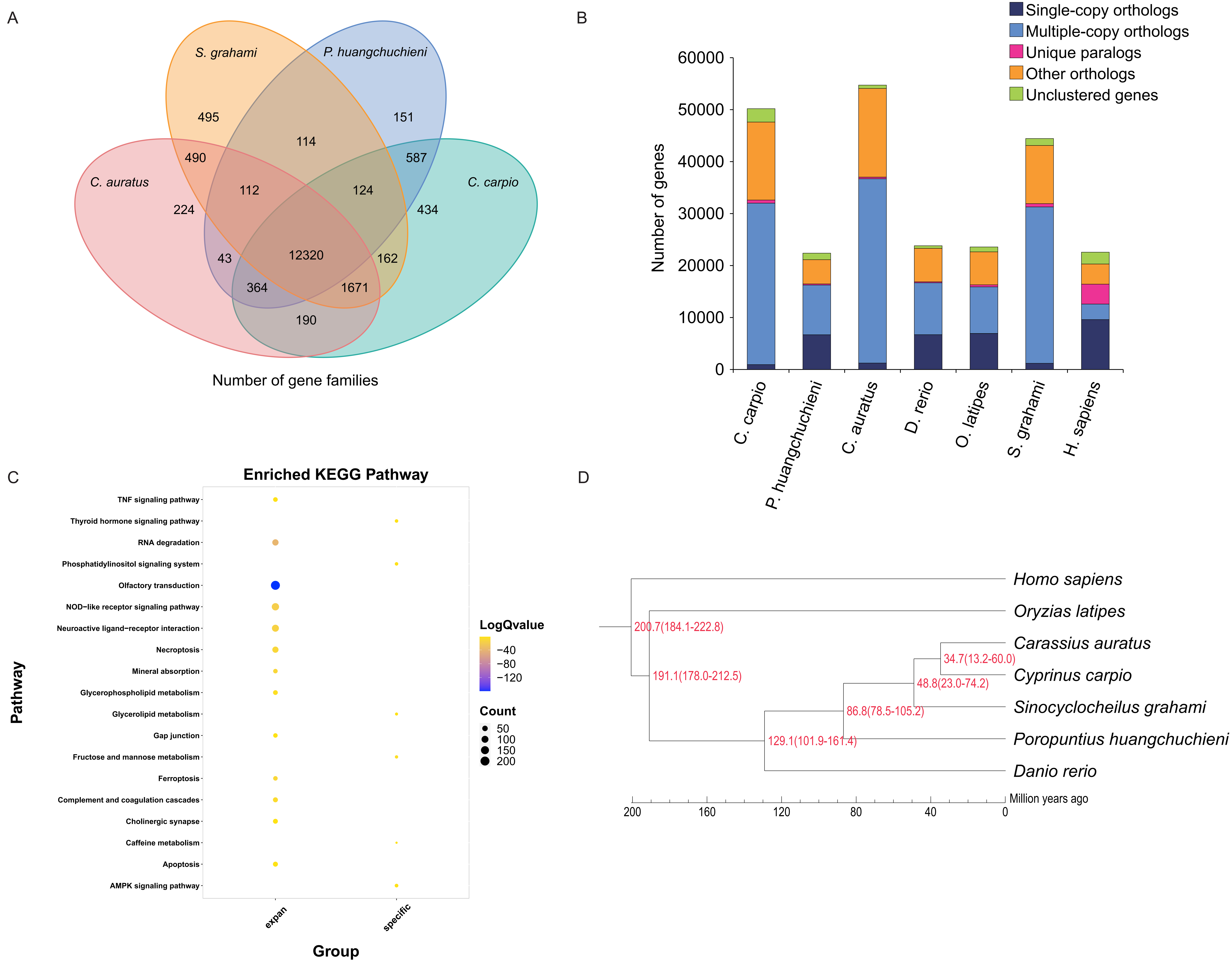
A
S. grahami
P. huangchuchieni
C. auratus
C. carpio
495
151
114
490
587
112
124
224
434
43
12320
162
364
1671
190
Number of gene families
B
Single-copy orthologs
Multiple-copy orthologs
Unique paralogs
Other orthologs
Unclustered genes
Number of genes
60000
50000
40000
30000
20000
10000
0
C. carpio
P. huangchuchieni
C. auratus
D. rerio
O. latipes
S. grahami
H. sapiens
C
Enriched KEGG Pathway
Pathway
TNF signaling pathway
Thyroid hormone signaling pathway
RNA degradation
Phosphatidylinositol signaling system
Olfactory transduction
NOD−like receptor signaling pathway
Neuroactive ligand−receptor interaction
Necroptosis
Mineral absorption
Glycerophospholipid metabolism
Glycerolipid metabolism
Gap junction
Fructose and mannose metabolism
Ferroptosis
Complement and coagulation cascades
Cholinergic synapse
Caffeine metabolism
Apoptosis
AMPK signaling pathway
expan
specific
Group
LogQvalue
−40
−80
−120
Count
50
100
150
200
D
Homo sapiens
Oryzias latipes
Carassius auratus
Cyprinus carpio
Sinocyclocheilus grahami
Poropuntius huangchuchieni
Danio rerio
200.7(184.1-222.8)
191.1(178.0-212.5)
34.7(13.2-60.0)
48.8(23.0-74.2)
86.8(78.5-105.2)
129.1(101.9-161.4)
200
160
120
80
40
0
Million years ago

Figure 5

Click here to access/download;Figure;Figure 5.pdf

A

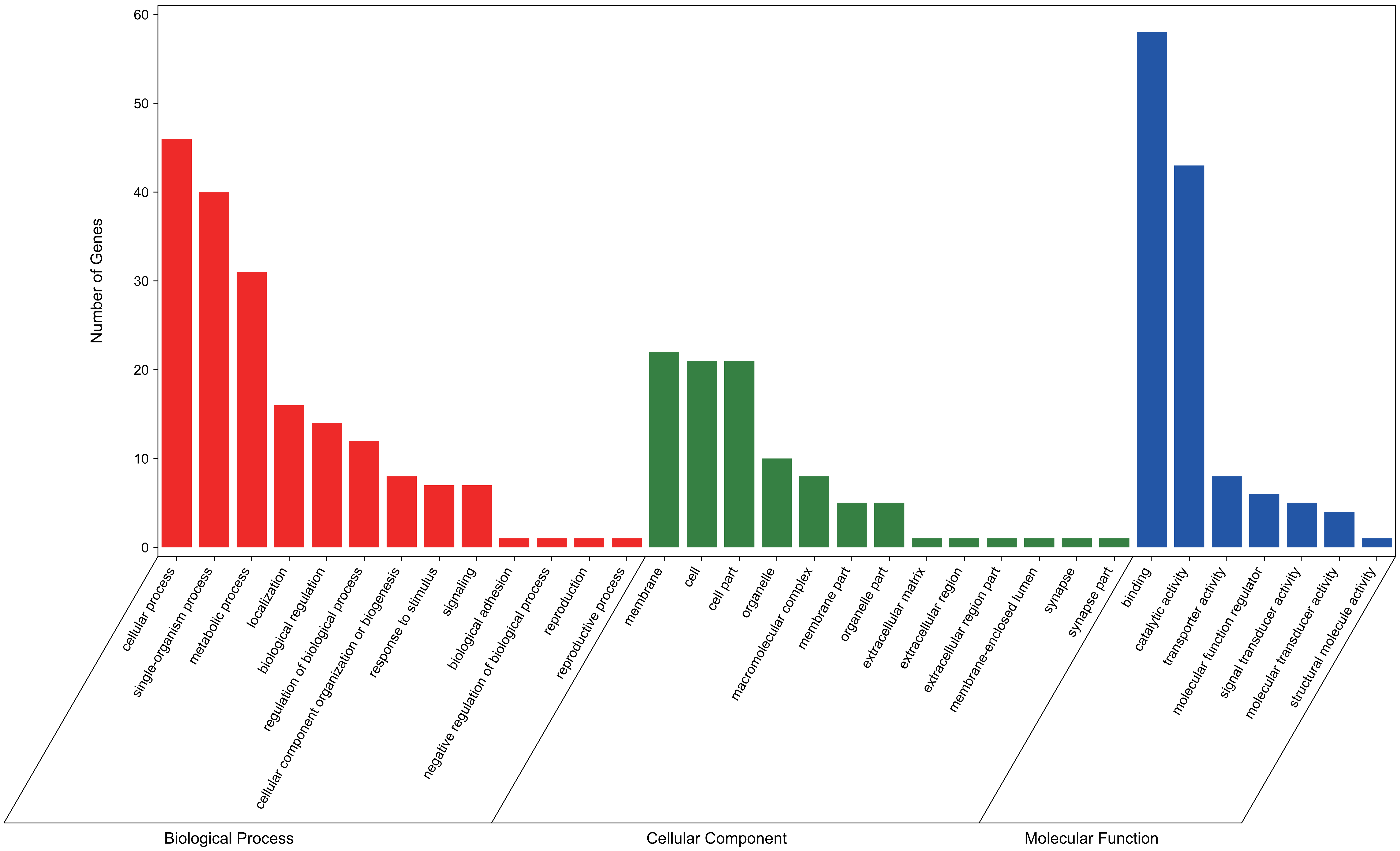

B

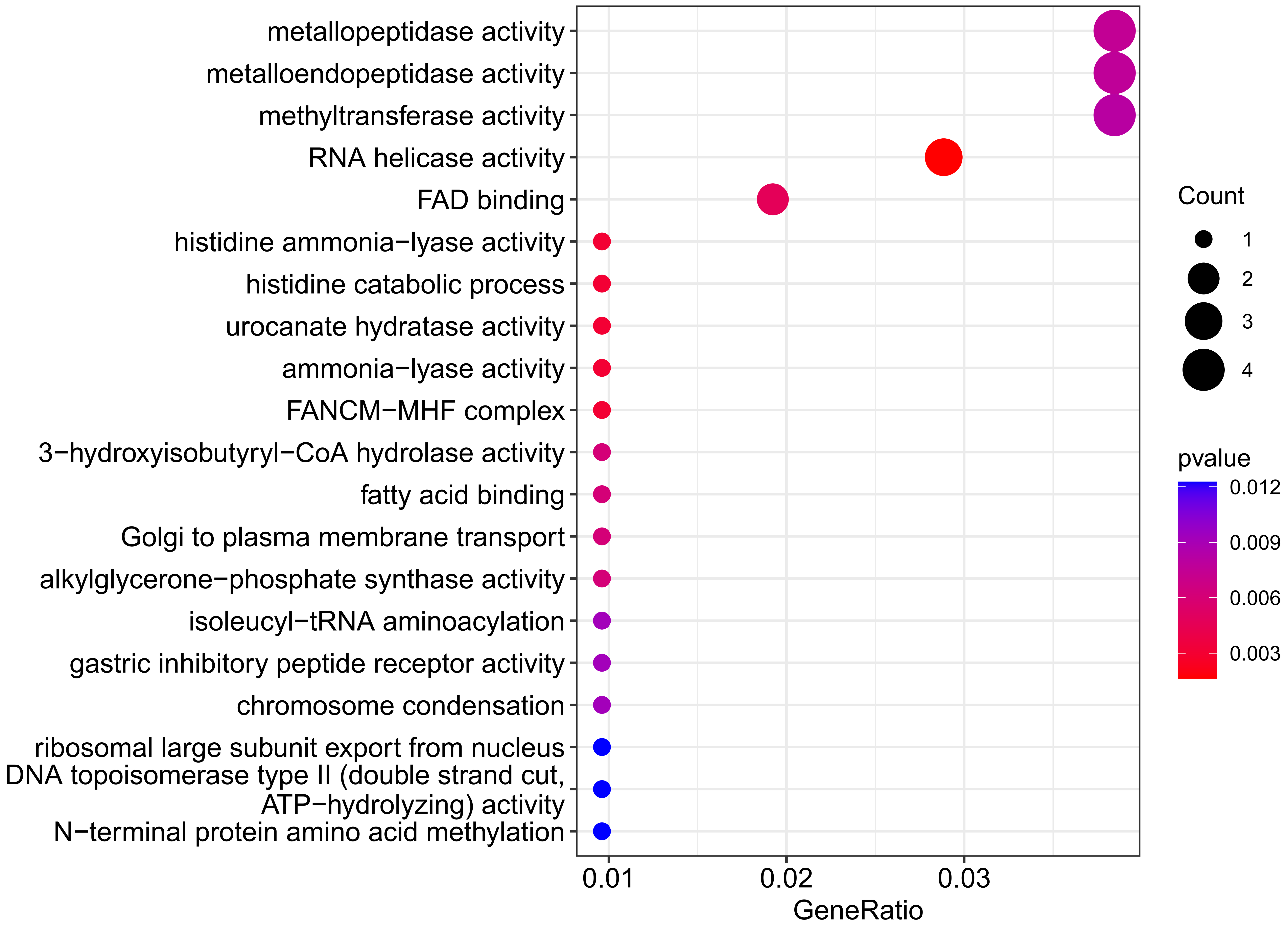

Supplementary Figures

Click here to access/download
Supplementary Material
Supplementary Figure.docx

Supplementary Tables

Click here to access/download
Supplementary Material
Supplementary Table.xlsx
