# Supplementary material for: A telomere-to-telomere genome assembly of koi carp (Cyprinus carpio) using long reads and Hi-C technology
Source: Gigascience. 2025 Aug 29;14:giaf087. doi: 10.1093/gigascience/giaf087 (PMC12395963; doi:10.1093/gigascience/giaf087)
Supplement: giaf087_GIGA-D-24-00530_Revision_1 [file giaf087_giga-d-24-00530_revision_1.pdf]

GigaScience

A telomere-to-telomere genome assembly of koi carp (Cyprinus carpio) using long reads and Hi-C technology

--Manuscript Draft--

Manuscript Number:	GIGA-D-24-00530R1
Full Title:	A telomere-to-telomere genome assembly of koi carp (Cyprinus carpio) using long reads and Hi-C technology
Article Type:	Data Note
Funding Information:	
Abstract:	Background: The common carp (Cyprinus carpio) is a key species in global freshwater aquaculture. One of its variants, koi carp is particularly prized for its aesthetic appeal. However, the lack of a high-quality genome has limited genetic research and breeding efforts for common carp and koi carp.<br>Findings: In this study, we present a gap-free genome for the Taisho Sansyoku koi carp strain (C. carpio). The assembly achieved a total size of 1555.86 Mb with a contig N50 of 30.45 Mb, comprising 50 gap-free pseudochromosomes ranging in length from 20.70 to 49.02 Mb. The BUSCO completeness score reached 99.20% and the GCI score was 85.82, indicating a high level of genome integrity and accuracy. Notably, 83 out of 100 telomeres were detected, resulting in 33 chromosomes possessing complete telomeres. Comparative genomic analysis showed that the expanded gene families and unique genes play important roles in various biological traits, such as energy metabolism, endocrine regulation, cell proliferation, and immune response, potentially related to multiple metabolic diseases and health conditions. The positively selected genes are linked to various biological processes, such as the metalloendopeptidase activity, which plays a significant role in the central nervous system and is associated with diseases.<br>Conclusions: The koi carp genome assembly (CC 4.0) fills a critical gap in the understanding of common carp and its adaptations. It provides an invaluable resource for molecular-guided breeding and genetic enhancement strategies, underscoring the importance of common carp and koi carp in aquaculture and ecological research.
Corresponding Author:	Yongchao Niu, PhD<br>Biozeron Shenzhen Inc.<br>Shenzhen, CHINA
Corresponding Author Secondary Information:	
Corresponding Author's Institution:	Biozeron Shenzhen Inc.
Corresponding Author's Secondary Institution:	
First Author:	Yongchao Niu, PhD
First Author Secondary Information:	
Order of Authors:	Yongchao Niu, PhD
	Jiandong Yuan
	Jiang Li
	Jun Yong
	Xuewu Liao
	Huijuan Guo
Order of Authors Secondary Information:	
Response to Reviewers:	Reviewer reports:<br><br>Reviewer #1: Yuan and colleagues have sequenced and assembled the koi carp

Powered by Editorial Manager® and ProduXion Manager® from Aries Systems Corporation

genome using data generated by several different advanced technologies, including PacBio HiFi, ONT ultralong sequences and Hi-C data. The sequencing data covered the genome over 470 times, resulting in a high-quality assembly (telomer-to-telomer assemblies for over 30 chromosomes, a few gaps, high BUSCO value, etc.). They have investigated the centromeric regions of the assembled pseudochromosomes and analyzed their genic and repeat content.

They have also analyzed the expanded gene families, unique genes and positively selected genes in the koi carp genome.

The authors propose that these data will provide "an invaluable resource for molecular-guided breeding and genetic enhancement strategies, underscoring the importance of common carp and koi carp in aquaculture and ecological research".

The Title covers the essence of the MS well. The flow of the Abstract is clear.
RE: Thanks for your positive comments on our work.

The Data Description section is concise and logical, but lacks important data published recently (see e.g. https://doi.org/10.1186/s12915-023-01806-9 and https://doi.org/10.1016/j.aquaculture.2024.741075). Due credit should be given to all others, who produced important and relevant data through the analysis of the common carp genome and transcriptome.
RE: Thanks for your comments. In the revised manuscript, we have included this two important papers in the introductions. Please find the details in lines 71-75 of the revised manuscript.

The Methods section is detailed, the sequencing and bioinformatic approaches that were taken by the authors are appropriate.
The Results are supported by two tables, five figures - four of which contain multiple panels - 14 Supplementary Tables and five Supplementary Figures. The description of data is logical, albeit sometimes potentially misleading (see below).
The Conclusions are succinct, but not complete and they do not always place the results into context well.
RE: Thanks for your comments. In line with your recommendation, the Conclusions has been supplemented with the previously omitted results. Please find more details in lines 428-440 of the revised manuscript. The description of data has been revised in the point-by-point responses below.

In summary, this Referee recommends a thorough revision of the MS prior to acceptance for publication in Gigascience.

Major criticisms:

A) In the opinion of this referee, the two most important aspects of this study are: (i) taking an important step towards the pan genome of common carp by improving the quality of the assembly; and (ii) revealing the genetic/genomic factors that led to the evolution of the colored koi carp varieties. In the current version of the MS, the authors do perform comparisons to emphasize the first aspect, but not the second. That leads to mis-interpretation of the data and wrong conclusions. Therefore, the MS should be revised to correct these errors and to help the reader to grasp the second advance better.
RE: Thanks for your comment. The manuscript, designed as a Data Note-type paper, focuses on the analytical aspects of constructing the T2T genome rather than focusing on biological stories. Its structure closely resembles that of the high-quality Chiridota heheva genome recently published in Gigascience (Pu, Zhou et al. 2024), prioritizing detailed analysis and data presentation. We appreciated your comments, so we have made efforts to improve the second aspect that you have mentioned. We conducted phylogenetic analysis and characterized positively selected genes in our study. Furthermore, a comprehensive genome-wide comparative analysis between CC 4.0 and Songpu2021 was newly generated, which identifying extensive genomic variations (lines 374-388). These efforts provided readers with certain analytical directions and ideas to the genetic and genomic factors underlying the evolution of the colored koi carp varieties.

Powered by Editorial Manager® and ProduXion Manager® from Aries Systems Corporation

	Reference :<br>Pu, Y., Zhou, Y., Liu, J. & Zhang, H. A high-quality chromosomal genome assembly of the sea cucumber Chiridota heheva and its hydrothermal adaptation. GigaScience 13(2024).<br><br>B) Terminology: The koi carp genome is referred to by several names in the MS, including 'gap-free genome assembly of koi carp', 'Taysho Sensyoku genome', 'final koi carp assembly (CC4.0)', 'gap-free koi carp CC4.0 genome assembly', and 'koi carp CC4.0 genome'. In the referee's opinion, the same name should be used throughout; 'koi carp genome assembly (CC4.0).<br>RE: Thanks for your valuable suggestion. We revised the manuscript to standardize the naming of the koi carp genome assembly as “koi carp genome assembly (CC4.0)” as suggested to avoid any confusion.<br><br>C) Phylogenetic relationship analysis (P16, L348-351): When the koi carp gene set is compared to those of six other vertebrates, genes that can only be found in the koi carp gene set cannot be called 'koi carp-specific'. The correct term here would be 'common carp-specific'. In order to find genes that are unique to koi carp the gene content of CC4.0 must be compared to CC3.0. The same refers to expanded and contracted gene families.<br>RE: We appreciate your attention to the accuracy and precision of our descriptions. We have revised the relevant sections in our revised manuscript.<br><br>D) Number formats: English uses decimal points and thousand separators in numbers, not the other way around. Therefore, all numbers in the tables and supplementary tables must be reformatted accordingly. Moreover, numbers in all tables must be flushed to the decimals to help the readers grasp their magnitude.<br>RE: Thanks for your comments. We have carefully revised all tables and supplementary tables to adhere to English numerical formatting standards, using decimal points and comma thousand separators (e.g., "748,299" instead of "748299") and aligning numbers by decimal points for clarity.<br><br>Additional criticisms (in the order of their appearance, not importance):<br>* Page 1, Line 15 vs. P2, L29-30: As correctly noted at the first appearance, koi carp is a variety of common carp. The second sentence must be corrected accordingly.<br>RE: Thanks for your comment. We changed “this species” to “common carp”. Please see lines 29-30 of the revised manuscript.<br><br>* P4, L79-80: The sex of the individual used for sequencing must be indicated here.<br>RE: Thanks for your comment. A 6-year-old female koi was used for sequencing in this study, the information has been added into the revised manuscript (line 96).<br><br>* P4, L81-84: Is there a specific reason, why the gonad RNA was not used for transcriptomics?<br>RE: Thanks for your comment. During the sampling process, the quality of the gonad RNA sample was suboptimal, so the tissue sample was not utilized. In this study, transcriptome data was predominantly employed for the annotation of gene sets. The annotation of gene sets encompasses not only transcriptome - derived sources but also homologous annotations and de novo annotations. The gene set annotation in this study is of high quality, as evidenced by a BUSCO score of 97.77. In the Songpu2011 genome study, gonad samples were not utilized either. Therefore, we consider that the absence of gonad samples may not affect the annotation quality.<br><br>* P12, L245: The correct term is 'positively selected genes'. This must be corrected throughout the MS.<br>RE: Thanks for your reminder. We corrected this term throughout the revised manuscript.<br><br>* P14, L307 - P15, L312: How do these data compare to those of CC3.0?<br>RE: Thanks for your comment. The main types of repeat sequences in CC 4.0 are consistent with those in CC 3.0. DNA transposons are the dominant transposable

	elements, and long terminal repeats (LTRs) and long interspersed nuclear elements (LINEs) have comparable proportions within the genome.<br>Regarding noncoding RNAs, although the CC 3.0 paper mentions that "RNAmmer and tRNAScan were used for rRNA and tRNA prediction, respectively", neither the main text nor its attachments present the relevant results. Consequently, we are unable to conduct a comparison.<br><br>* P15, L327-329: It is unnecessary to use two decimals in these numbers.<br>RE: We have deleted two decimal decimals in these numbers. Please find more details in line 354 of the revised manuscript.<br><br>* P15, L326 - P16, L341: How do the koi carp centromeres compare to other cyprinid or teleost centromeres? If not available they should be compared to mammalian centromeres.<br>RE: Given that telomere-to-telomere genomes of the closely related species goldfish and zebrafish remain unpublished, we compared our results with those of the Yangtze finless porpoise, rice, human, and macaque. Please find more details in lines 356-358, 365-372 of the revised manuscript.<br><br>* P16, L331: Erroneous pointer. Please correct to: (Fig.2C).<br>RE: Thanks, revised.<br><br>* P17, L358: What proportion of these expansions and contractions are present in the common carp genome (CC3.0)?<br>RE: Thank you for your comment. In the study of the CC 3.0 genome, the authors did not perform an analysis of gene family expansions and contractions. The CC 3.0 genome study mainly concentrated on aspects such as high - fidelity genome assembly, subgenome structural evolution, homoeologous expression, and the response to thermal stress. Consequently, the proportion of gene family expansions and contractions in the CC 3.0 genome cannot be obtained.<br><br>* P17, L371: What proportion of these 124 genes are positively selected in CC3.0?<br>RE: Similar to the previous question, the authors also did not carry out a positive selection analysis on CC 3.0 genome.<br><br>* P18, L382: Actually, this is the first T2T genome assembly for the whole species that happens to be a koi variety.<br>RE: Thank you for your reminder. In the revised manscript, we have replaced "koi carp" with "common carp". Please find more details in line 427 of the revised manuscript.<br><br>* P18, L382-390: Centromeric regions and positively selected genes must be mentioned in the Conclusion.<br>RE: Thank you for your reminder. The results regarding centromeric regions and positively selected genes have been presented in the conclusion of the revised manuscript. Please find more details in lines 429-432, 436-438 of the revised manuscript.<br><br><br>* Reference list: The list contains over a dozen incomplete citations that must be completed. This referee recommends that title should not be fully italicized, so that the italicized Latin name would stand out according to the convention. Also, the formatting of reference list must be unified.<br>RE: Thank you for your reminder. We have addressed the reference list issues in the revised manuscript.<br><br>* Figure 3, Panel A: CC3.0 and CC4.0 should be next to each other.<br>RE：In panel A of figure 3, the primary species compared are CC 4.0 and Songpu 2021. The species order has been revised in accordance with your suggestion. A detailed comparison between CC 4.0 and CC 3.0 is presented in Table 1.<br><br>* Supplementary Figure S1, Legend: Grammatical error. Please correct 'divide' to 'divideD'.<br>RE: Revised.

* Supplementary Figure S3, Legend: Please correct 'abscissa and ordinate' to 'X-axis and Y-axis'.
RE: Revised.

* Supplementary Figure S4: The (grammatically incorrect) title must be removed from the top of the figure. All essential info must be provided by the legend.
* Supplementary Figure S4, Legend: Instead of 'blue and green boxes', the figure contains only blue bars. The legend must be revised to reflect the content of the illustration.
* Ibid: Grammatical error ('gene families belongs').
RE: Supplementary Figure S4 has been revised with supplementary annotations, and additional interpretive details have been incorporated.

* Supplementary Figure S5, Legend: The legend must clearly indicate which GO category (Molecular Function, Cellular Component, or Biological Process) is shown on the illustration. The last sentence must be revised to indicate 'diameter of circle' instead of GO term.
RE: We understand the significance of GO term classification. In our study, GO terms were ranked in ascending order of P.adjusted values to emphasize the most statistically significant functional categories, as previously demonstrated in Figure S15 of the mouse T2T genome study (Liu, Li et al. 2024).
The final sentence has been revised in accordance with your recommendations.

Reference: Liu, J. et al. The complete telomere-to-telomere sequence of a mouse genome. Science. 386, 1141-1146 (2024).

Suggestion at the discretion of the authors:

* Table 1: This is a nice, informative table. The authors should consider extending it with data from earlier version of common carp genome assembly with years indicated to visualize the progression of the field.
RE: Thanks for your comment. We have incorporated relevant statistics regarding the Songpu2021 genome into Table 1.

Reviewer #2: In this paper, the authors describe T2T genome assembly of an ornamental-colored common carp (Cyprinus carpio) strain, Taisho Sanshoku. The assembly achieved a total size of 1555.86 Mb with a contig N50 of 30.45 Mb, comprising 50 gap-free pseudochromosomes. The BUSCO completeness score reached 99.20%. In this assembly, 83% of telomeres were detected, suggesting that 33 chromosomes possess complete telomeres. The findings are relatively descriptive; however, these data are useful for researchers in this field and suitable for publication in this journal after revisions. Several essential concerns should be addressed.

1. The authors did not explain what is "Taisho Sanshoku" koi carp strain. It is a Nishikigoi strain that was established in the 19th century in Niigata Prefecture in Japan. A review written by EK Balon describes the origin of them. The authors should add an explanation of this strain and its origin in the introduction and refer to this.

Balon, Eugene K. "The common carp, Cyprinus carpio: its wild origin, domestication in aquaculture, and selection as colored Nishikigoi." Guelph Ichthyology Reviews 3 (1995).

RE: Thank you for your reminder. In the revised manuscript, we have offered supplementary elucidations with respect to the pertinent characteristics of "Taisho Sanshoku" koi carp strain. Please find the details in lines 84-89 of the revised manuscript.

2. It is better to discuss allotetraploidy in common carp and goldfish, a species very closely related to common carp. The following papers that investigated allotetraploid in goldfish would help the understudying for readers.

Zelin Chen et al., Science Advances 2019 Vol 5, Issue 6 DOI: 10.1126/sciadv.aav0547

Powered by Editorial Manager® and ProduXion Manager® from Aries Systems Corporation

	Tetsuo Kon et al., Current Biology 2020 doi.org/10.1016/j.cub.2020.04.034<br>Jing Luo et al., Science Advances 2020 Vol 6, Issue 22 DOI: 10.1126/sciadv.aaz7677<br><br>RE: Thanks for your valuable suggestions. We have included these papers and added the supplementary description in the revised manuscript. Please see the details in lines 48-56 of the revised manuscript.<br><br><br>3. The authors compared their new assembly (CC 4.0) and previously published assembly (CC 3.0) in Table 1. In addition, it is better to compare the previous assembly shown in Ref 6 (Li et al. 2021). This comparison would highlight the progress of this paper.<br>RE: Thank you for your nice suggestion. In the revised manuscript, we have incorporated the detailed comparison results from Ref 6 (Li et al., 2021) into Table 1. Please find the details in Table 1 of the revised manuscript.<br><br><br>4. The authors should compare them and discuss the differences between Taisho Sanshoku and Songupu strains.<br>RE: Thank you for your nice suggest. We conducted comparative analysis of Taisho Sanshoku and Songupu strands at the whole genome level. Please find more details in lines 236-248, 375-387 of the revised manuscript.<br><br><br>5. The gene annotation file (GFF3 file) shows gene IDs (i.e. B23G0250) that the authors defined in this paper. These gene codes should link the gene names and gene symbols. This list should be added in a new Supplementary Table.<br>RE: Thank you for your comment. The gene symbol file, named "CC4.0 gene symbol.xls", has been uploaded to FigShare at the following link: https://figshare.com/s/93779280afb6ad32eafb.<br><br><br>6. It is useful to add gene symbols in the GFF3 file. It is useful for readers.<br>RE: Thank you for your comment. The GFF file containing symbol information has been uploaded to FigShare at the following link: https://figshare.com/s/93779280afb6ad32eafb (The file is named "Carp.add.symbol.gff3.gz").<br><br>7. At least the gene annotation file (gff3) and genome assembly file (fasta file) should be deposited on the public database such as Figshare. It should show in "Data Availability".<br>RE: Thank you for your comment. The gene annotation file (in GFF3 format) and the genome assembly file (in FASTA format) are now accessible on FigShare at the following link: https://figshare.com/s/93779280afb6ad32eafb.
**Additional Information:**	
**Question**	**Response**
Are you submitting this manuscript to a special series or article collection?	No
**Experimental design and statistics**<br><br>Full details of the experimental design and statistical methods used should be given in the Methods section, as detailed in our Minimum Standards Reporting Checklist. Information essential to interpreting the data presented should be made available in the figure legends.	Yes

Powered by Editorial Manager® and ProduXion Manager® from Aries Systems Corporation

Have you included all the information requested in your manuscript?	
**Resources**<br><br>A description of all resources used, including antibodies, cell lines, animals and software tools, with enough information to allow them to be uniquely identified, should be included in the Methods section. Authors are strongly encouraged to cite Research Resource Identifiers (RRIDs) for antibodies, model organisms and tools, where possible.<br><br>Have you included the information requested as detailed in our Minimum Standards Reporting Checklist?	Yes
**Availability of data and materials**<br><br>All datasets and code on which the conclusions of the paper rely must be either included in your submission or deposited in publicly available repositories (where available and ethically appropriate), referencing such data using a unique identifier in the references and in the “Availability of Data and Materials” section of your manuscript.<br><br>Have you have met the above requirement as detailed in our Minimum Standards Reporting Checklist?	Yes
GigaScience has policies and guidelines in place for the use of generative AI-writing tools such as ChatGPT. If you have used such writing tools to assist with writing the manuscript this must be declared and cited in the text. Authors should not list AI-writing tools and other AI-assisted technologies as an author or co-author and should acknowledge that they are fully responsible for text generated or refined by AI-writing tools.<p>	No

A summary of use (particularly in the introduction or among methods) needs to be included at the end of the paper, and the outputs should also be included as a supplementary file hosted in GigaDB or other open repositories. Please read our guidelines for more information. <p><br><br>By submitting to GigaScience, you are aware of the journal's AI-writing tools policy, and if you have declared use of such tools below, you have acknowledged this where appropriate in your manuscript and have made a summary of use and outputs available. <p><br><b>AI-assisted writing tools have been used in the preparation of this manuscript?	

Powered by Editorial Manager® and ProduXion Manager® from Aries Systems Corporation

Manuscript

Click here to access/download;Manuscript;Revised manuscript.docx

DATANOTE

A telomere-to-telomere genome assembly of koi carp (*Cyprinus carpio*) using long reads and Hi-C technology

Jiandong Yuan [1*†], Jiang Li [2†], Jun Yong[3†], Xuewu Liao [1], Huijuan Guo[3], Yongchao Niu [2*]

1 Suxin Koi Farm, Suzhou 215000, China

2 Biozeron Shenzhen Inc., Shenzhen 518000, China

3 Geekgene Technology Co. Ltd., Beijing 100091, China

*Correspondence address. Suxin Koi Farm, Suzhou 215000, China. E-mail: yuan@suxinkoi.com; Biozeron Shenzhen Inc. Shenzhen 518000, China. E-mail: niuyongchao@biozeron.com.

[†] These authors contributed equally to this work.

Abstract

Background: The common carp (*Cyprinus carpio*) is a key species in global freshwater aquaculture. One of its variants, koi carp is particularly prized for its aesthetic appeal. However, the lack of a high-quality genome has limited genetic research and breeding efforts for common carp and koi carp.

Findings: In this study, we present a gap-free genome for the Taisho Sansyoku koi carp strain (*C. carpio*). The assembly achieved a total size of 1555.86 Mb with a contig N50 of 30.45 Mb, comprising 50 gap-free pseudochromosomes ranging in length from 20.70 to 49.02 Mb. The BUSCO completeness score reached 99.20% and the GCI score was 85.82, indicating a high level of genome integrity and accuracy. Notably, 83 out of 100 telomeres were detected, resulting in 33

chromosomes possessing complete telomeres. Comparative genomic analysis showed that the expanded gene families and unique genes play important roles in various biological traits, such as energy metabolism, endocrine regulation, cell proliferation, and immune response, potentially related to multiple metabolic diseases and health conditions. The positively selected genes are linked to various biological processes, such as the metalloendopeptidase activity, which plays a significant role in the central nervous system and is associated with diseases.

Conclusions: The koi carp genome assembly (CC 4.0) fills a critical gap in the understanding of common carp and its adaptations. It provides an invaluable resource for molecular-guided breeding and genetic enhancement strategies, underscoring the importance of common carp and koi carp in aquaculture and ecological research.

Key words: common carp, koi carp, telomere-to-telomere, genome, positively selected gene

Data Description

Context

Common carp (*Cyprinus carpio*) is one of the most economically significant species, accounting for up to 10% (over 3 million metric tons) of global freshwater aquaculture production [1]. It is mainly cultured in Europe and Asia with a cultural history of several thousand years and has been introduced into most parts of the world. Known for being environmentally friendly, common carp are primarily omnivorous filter-feeders, requiring less fish meal and fish oil compared to other aquaculture species such as salmon and shrimp [2]. In addition to serving as a food source, one of the common carp variants, koi carp, is highly prized as an ornamental fish, renowned for its vibrant

colors and patterns. *C. carpio* originated from the hybridization of a Barbinae-like species and an undetermined donor species, followed by a whole genome duplication (WGD) event approximately 12.4 million years ago [3]. It is believed that their genome duplication is responsible for species divergence and biodiversity [4]. Common carp and goldfish (*Carassius auratus*) are evolutionarily closely related, both being allotetraploid species that have undergone WGD events. The allotetraploid nature of these species has been extensively characterized. In 2019, Chen *et al.* reported the *de novo* assembly of the goldfish genome and elucidated the evolutionary trajectories of genes subsequent to WGD [5]. Similarly, Kon *et al.* demonstrated balanced homoeolog expression and symmetric subgenomes in allotetraploid fish, thereby highlighting the crucial role of genomic plasticity in the establishment of allopolyploidy [6]. A landmark study on subgenomic evolution was further explored in allotetraploid fish, revealing a transition from asymmetrical to balanced genomic diversification during rediploidization [7]. As an ideal model for studying the structural and functional adaptations in polyploid vertebrates, koi carp provides valuable insights into successful speciation and the evolutionary dynamics of polyploidy in animals, making it a critical species for both aquaculture and ecological research. Notably, it is also regarded as an alternative vertebrate model to zebrafish.

Over the past decade, various *C. carpio* genome resources have been developed. The genome of *C. carpio* (strain Songpu) was first decoded in 2014, marking the beginning of common carp genomics research [2]. In 2019, chromosome-level reference genomes of Yellow River carp, Hebao red carp, and German Mirror carp were generated [3]. Moreover, the availability of *Poropuntius huangchuchieni* genome provides a diploid progenitor-like reference genome for the allotetraploid *C. carpio* [8]. In 2021, the genome of common carp var. ‘Songpu’ was updated [9], followed by

the availability of the latest *C. carpio* genome (CC 3.0) in 2023 obtained via Pacific Biosciences (PacBio) high-fidelity (HiFi) reads [10]. Intensive culture conditions make farmed common carp vulnerable to various pathogens, leading to high mortality rates and significant economic losses in the carp culture industry. Hence, *C. carpio* has been continuously studied in terms of its immunology and disease resistance [11-14]. The association between genetic variations and phenotypic diversity among different common carp strains has been studied. Wang *et al*. found genetic variations related to traits like scale reduction and high growth rate and identified new candidate genes [15]. Shi *et al*. detected single nucleotide polymorphisms (SNPs) linked to skin color variation across different carp strains [16]. *De novo* genome assembly is a fundamental and powerful tool. Currently, advances in sequencing and assembly algorithms make telomere-to-telomere (T2T) genome assembly feasible, enabling comprehensive genome identification. Some important species, such as humans [17], sheep [18, 19], rice [20], maize [21], and sorghum [22], have successively released T2T-level genomes. The considerations and methodologies for executing T2T assembly have been thoroughly summarized [23, 24]. However, the assembly of the *C. carpio* genome to a comparable level has yet to be reported. To address this gap, we integrated PacBio HiFi sequencing, Oxford Nanopore Technologies (ONT) ultralong sequencing, and chromosomal conformational capture (Hi-C) technology to assemble a high-quality gap-free genome assembly for koi carp (strain Taisho Sansyoku; Fig. 1). "Taisho Sanshoku" is a Nishikigoi strain established in the 19th century in Niigata Prefecture in Japan, which is a significant variety in the selective breeding of colored carp. This strain is characterized by its combination of red, black, and white colors, with all three colors being very deep and black streaks on paired fins being permissible. It is an important part of the koi carp breeding and has a certain position in the

ornamental fish market. Armed with the Taisho Sansyoku genome assembly, the characteristics of centromeric regions were investigated, and genomic evolution analyses were performed. This study on the *C. carpio* genome provides a valuable resource for the molecular-guided breeding and genetic improvement of the common carp and koi carp.

Methods

Sample collection

We collected a healthy 6-year-old female koi carp (Taisho Sansyoku) from Suzhou City, Jiangsu Province, China for DNA sequencing, RNA-seq, and Iso-seq. Genomic DNA was extracted from a muscle sample. To improve genome annotation, scale, and fin tissues were prepared for RNA-seq. In addition, RNA from eighteen tissues, including eye, tail, white scalp, red scalp, brain, black scale, white scale, red scale, heart, blood, liver, bubble, essence, spleen, bile, kidney, muscle, gill, and intestines, were equally pooled together for Iso-seq. All samples were frozen in liquid nitrogen and stored at -80°C for preservation and subsequent analysis.

Sequencing and filtering

For HiFi sequencing, SMRTbell target size libraries were constructed according to PacBio's standard protocol (Pacific Biosciences) using the 15-kb preparation solutions. The sequencing was conducted in circular consensus sequencing (CCS) mode on the PacBio Revio platform (RRID:SCR_017990) at Grandomics Biosciences (Wuhan, China). The generated subreads were processed using SMRTLink version 8.0.0 [25] with the following parameters: "-minPasses 3 -minPredictedAccuracy 0.99 -minLength 500".

For ONT sequencing, ONT ultra-long insert libraries were obatinted using the Oxford Nanopore SQK-LSK109 kit and sequenced on the PromethION (RRID:SCR_017987) platform at Grandomics Biosciences (Wuhan, China). The ONT data underwent processing using NanoFilt version 2.8.020 (RRID:SCR_016966) [26] with a quality threshold of 7.

Hi-C libraries based on *Dpn*II restriction enzymes were prepared for Hi-C sequencing, as previously described [27]. These libraries were sequenced on the MGISEQ-2000 platform, generating paired-end 150 bp reads. Clean Hi-C data were obtained using fastp version 0.19.5 (RRID:SCR_016962) [28] with parameters set as "--length_required 50 -w 8". In addition, about 1.5 μg DNA was used to construct an approximately 350 bp insert size DNA library. According to the standard manufacturer's instructions, the quantified library was sequenced on the Illumina NovaSeq platform (Illumina, CA, USA).

The total RNA was extracted using TRIzol reagent in an RNAprep Pure Tissue Kit and processed according to the protocol provided by the manufacturer. Subsequently, the RNA purity and concentration were assessed using Nanodrop and Qubit, where only high-quality RNA samples were selected for cDNA synthesis in both bulk RNA-seq and Iso-seq experiments. For Iso-Seq, sequencing libraries were prepared using the SMRTbell Template Prep Kit 2.0 from Pacific Biosciences and sequenced on the PacBio Sequel II platform. Libraries for bulk RNA-seq were sequenced on an MGISEQ-2000 instrument, generating 150 bp paired-end reads. The libraries for bulk RNA-seq were sequenced on an MGISEQ-2000 instrument, producing 150 bp paired-end reads. The raw reads of Iso-Seq were preprocessed using SMRTLink version 8.0.0 [25]. Iso-Seq CCS reads were derived from the subreads with specific parameters: minimum subread length = 50, maximum subread length = 15,000, minimum number of passes = 3, and minimum predicted

accuracy = 0.99. The quality of RNA-seq data was examined using fastp version 0.19.5 (RRID:SCR_016962) [28] with the parameters set as "-w 8 -l 50".

Genome size estimation

To estimate the genome size and heterozygosity of the koi carp, a similar method in the study of the largemouth bass genome [29] was applied. Jellyfish version 2.1.3 (RRID: SCR_005491) [30] was used to analyze the *K*-mer depth distribution curve with a *K*-mer size of 17. The genome size was calculated using the formula G = (Ktotal - Kerror) / D, where Ktotal represents the total count of *K*-mers, Kerror is the total count of low-frequency *K*-mers (frequency ⩽ 3) likely due to sequencing errors, G is the genome size, and D is the k-mer depth [31].

Genome assembly and Hi-C scaffolding

To assemble a T2T reference genome, a combination of methods and sequencing reads was utilized. Initially, ultra-long ONT reads were processed using NextDenovo version 2.5.2 (RRID: SCR_025033) [32] for downstream gap-filling analysis. Primary contigs were generated with Hifiasm version 0.19.6 (RRID: SCR_021069) [33] using the command: "hifiasm -o Carp -t32 --ul ul.fq.gz --h1 Hi-C_clean_1.fq.gz --h2 Hi-C_clean_2.fq.gz HiFi-reads.fq.gz". Contigs were then polished with NextPolish2 version 0.2.0 [34] using HiFi reads. Hi-C clean data was aligned to the polished contigs for scaffolding using Bowtie2 version 2.2.9 (RRID: SCR_016368) [35]. Low-quality reads were eliminated using the HiC-Pro pipeline (RRID: SCR_017643) [36] with default parameters. Valid reads were utilized to anchor chromosomes with Juicer version 1.6 (RID: SCR_017226) [37] and 3d-DNA pipeline version 180419 (RRID: SCR_017227) [38]. An additional error correction step was carried out with Juicebox version 2.13.07

(RRID: SCR_021172) [39] according to the interaction signal. Following a similar approach used in the goose T2T genome study [40], gaps within the assembled genome were filled using quartet_gapfiller.py from quarTeT version v1.1.1 (RRID: SCR_025258) [41], utilizing preassembled contigs generated from NextDenovo version 2.5.2 (RRID: SCR_025033) [32]. As recommended, the specific parameters used were "-f 5000 -l 1000 -i 40 -m 1000000 -t 20". In addition, we applied the LR_Gapcloser (RRID: SCR_017021) [42] program to close the remained gaps in the assembled chromosomes, referring to the methods described in the gap-free genome of *Neosalanx taihuensis* [43]. To enhance genome quality, Winnowmap version 2.03 (RRID: SCR_025349) [44] was used to align HiFi reads to the chromosomes, followed by filtering to exclude secondary alignments and excessive clipping with the 'falconc bam-filter-clipped' tool. Finally, Racon version 1.5.0 (RRID: SCR_017642) [45] was performed for further polishing with the filtered alignments.

The completeness of the genome assembly was assessed utilizing Benchmarking Universal Single-Copy Orthologs (BUSCO) version 5.5.0 (RRID: SCR_015008) [46] with the actinopterygii_odb10 database, which includes 3,640 orthologs. The quality value (QV) was evaluated by Merqury program version 1.3 (RRID: SCR_022964) [47] with 17-mer. Furthermore, short reads were aligned to the genome using Bwa version 0.7.17-r1188 (RRID: SCR_010910) [48], while long reads from ONT and HiFi were aligned with Minimap2 version 2.24-r1122 (RRID: SCR_018550) [49]. In addition, the Genome Continuity Inspector (GCI) was assessed using GCI version 1.0 [50]. For collinearity analysis, the two genomes were compared using MUMmer4 version 4.0.0rc1 (RRID: SCR_018171) [51] with parameters of "-t 30 -p mummer --mum -g 1000 -c 90 -l 40".

Genome annotations

Tandem Repeats Finder version 4.10 (RRID: SCR_022065) [52] was used to identify the tandem repeat elements. For interspersed repetitive sequences, a combined approach of *de novo* prediction and known repeat searching was employed. RepeatModeler version 1.0.8 (RRID: SCR_015027) [53] and LTR_FINDER version 1.0.6 (RRID: SCR_015247) [54] were used to predict *de novo* repeat sequences. Subsequently, RepeatMasker version 4.0.7 (RRID: SCR_012954) [55] was applied to screen the koi carp genome against the combined *de novo* transposable element library. Additionally, RepeatMasker version 4.0.7 (RRID: SCR_012954) [55] and RepeatProteinMask version 4.0.6 were employed to search the Repbase database (RRID: SCR_021169) [56] to identify known transposable element repeats. The annotation of noncoding RNAs in the koi carp genome utilized the same method as in the largemouth bass genome study [29].

Telomeric sequences within the koi carp genome assembly were screened using quarTeT version v1.1.1 (RRID: SCR_025258) [41] with the "-c animal" option, following a method similar to that described in the study of the near-complete sheep assembly [57]. The telomere repeat monomer identified by the TeloExplorer module in quarTeT program was “AACCCT”. Centromeres were identified using both the quarTeT version v1.1.1 (RRID: SCR_025258) [41] and Centromics methods (https://github.com/ShuaiNIEgithub/Centromics). The results from quarTeT were given priority. If the TRcoverage of a chromosome's centromere was less than 40% or TEcoverage was less than 80% in the quarTeT identification results, it was considered an unreliable result. In such cases, the centromere region was determined using the Centromics method.

The gene prediction process employed a comprehensive strategy that integrated transcriptome-based, homology-based, and *ab initio* prediction methods. Initially, RNA-seq clean reads and

PacBio full-length CCS reads were assembled using Trinity version 2.11.0 (RRID: SCR_013048) [58], with the parameters '--max_memory 200G --CPU 40 --min_contig_length 200 --genome_guided_bam merged_sorted.bam --full_cleanup --min_kmer_cov 4 --min_glue 4 --bfly_opts '-V 5 --edge-thr=0.1 --stderr' --genome_guided_max_intron 10000 --long_reads ccs.fa', yielded 289,634 transcripts with a N50 size of 2,826. The assembled transcripts were then aligned to the assembly using Program to Assemble Spliced Alignment (PASA) version 2.4.1 (RRID: SCR_014656) [59], generating gene structures from valid transcript alignments (PASA-set). Additionally, RNA-seq clean reads were mapped to the assembly via Hisat2 version 2.0.1 (RRID: SCR_015530) [60]. Subsequently, Stringtie version 1.2.2 (RRID: SCR_016323) [61] and TransDecoder version 5.7.1 (RRID: SCR_017647) were employed to assemble the transcripts and identify candidate coding regions, resulting in the creation of gene models (Stringtie-set). Homologous genomes from seven assemblies, including four common carps (hebaored, germanmirror, huanghe, Songpu; ensenbl_release-111), *Carassius auratus* (ensenbl_release-111), *Danio rerio* (ensenbl_release-111), and *Poropuntius huangchuchieni* (https://ngdc.cncb.ac.cn/gwh/Assembly/10299/show) were downloaded and used as queries to search against the assembly using GeMoMa version 1.9 (RRID: SCR_017646) [62]. These homology predictions were referred to as "Homology-set". For *ab initio* prediction, Helixer [63] was employed to predict coding regions in the soft-masked genome. The gene models from these three sources were subsequently merged using EvidenceModeler version 2.1.0 (RRID: SCR_014659) [64], with different weight parameters assigned to evidence from different sources (10 for PASA-set, 5 for Stringtie-set, 5 for Homology-set, and 1 for *ab initio* gene prediction). Finally, the generated gene models underwent further refinement with PASA version

2.4.1 (RRID: SCR_014656) [59] to obtain untranslated regions and alternative splicing variation information.

The integrated gene set was translated into amino-acid sequences and annotated using various databases. Diamond version 0.9.30 (RRID: SCR_009457) [65] with an E-value cutoff of 1e-05 was used to compare the protein against four public databases, including NCBI non-redundant protein sequence database, SwissProt [66], Kyoto Encyclopedia of Genes and Genomes (KEGG) [67], Translation of European Molecular Biology Laboratory. Gene ontology (GO) terms of these genes were identified using InterProScan version 5.59-91.0 (RRID: SCR_005829) [68].

Gene expression analysis was conducted following the method used in the blister beetles transcriptome [69]. Transcription factor (TF) prediction was done using AnimalTFDB version 4.0 [70].

Identification of variations

Genome alignment between the CC 4.0 genome and the Songpu2021 genome was carried out using the NUCmer program of MUMmer4 version 4.0.0rc1 (RRID: SCR_018171) [51]. The parameter settings were ‘‘--mum -g 1000 -c 90 -l 40’’. Subsequently, the delta-filter program was employed to identify alignment blocks with the parameter setting ‘‘-1 -l 1000’’. The show-snps program was utilized to detect SNPs and insertions/deletions (InDels) with the settings ‘‘-Clr -x 1 -T’’. Based on the genic regions overlapping with these variations, we annotated the identified variations using ANNOVAR version 2020-06-07. These variations were classified into seven categories: intergenic region, intronic region, exonic region, 2 kb upstream and downstream regions, 3′ UTR, and 5′ UTR.

Moreover, Assemblytics (http://assemblytics.com/) was used to detect structural variants (SVs) larger than 50 bp. SVs whose positions overlapped with potential expression regulatory regions (the ±2 kb flanking regions of a gene, as analyzed in this study) or the coding sequence (CDS) of reference genes were designated as "SV-genes", while the remaining genes were labeled as "nonSV-genes".

Gene families and phylogenomic analysis

Protein sequences for six vertebrate animals, including *C. auratus*, *D. rerio*, *P. huangchuchieni*, *Oryzias latipes*, *Sinocyclocheilus grahami* and *Homo sapiens*, were obtained from public databases. The gene families were defined using Treefam (RRID: SCR_013401) [71]. For genes with alternative splicing variants, the longest transcripts were selected to represent the genes. Blastp version 2.7.1+ (RRID: SCR_001010) [72] with an E-value cutoff of 1e-5 was utilized to identify the best-hit protein for each sequence. Hcluster_sg with the parameter "-w 10 -s 0.34" was employed to identify one-to-one orthologous proteins among the seven species under study. A total of 241 single-copy gene families across these species were aligned using muscle version 3.8.1551(RRID: SCR_011812) [73]. Coding sequences were extracted from each single-copy gene family and concatenated to create a supergene for each species. The supergene data was then used to construct the phylogenetic tree via iqtree2 version 2.2.2.7 [74], with the parameters set as "-m MFP -B 1000".

The divergence time among seven species was estimated using the MCMCtree version 4.4 as implemented in the Phylogenetic Analysis of Maximum Likelihood (PAML) package (RRID: SCR_014932) [75], with the JC69 nucleotide substitution model and an independent rates

clock. Three fossil calibration times from the TimeTree database (RRID: SCR_021162) [76] were used for calibration: 1) *C. carpio – C.auratus* 10.1 - 61.0 Mya; 2) *D. rerio - O. latipes* 180.0 - 251.5 Mya ; 3) *C. carpio - P. huangchuchieni* 81.0 - 124.7 Mya. Changes in gene family size along the phylogenetic tree were analyzed by CAFE version 4.2.1 (RRID: SCR_005983) [77]. Pathway enrichment of koi carp-specific genes, as well as genes in the expansion gene families, was conducted using KOBAS version 2.0.12 (RRID: SCR_006350) [78].

Positively selected genes

We applied a similar approach as previously reported [79] to identify positively selected genes (PSGs) within the koi carp genome. In brief, the branch-site model available in the PAML package was utilized based on the phylogenetic tree. The koi carp served as the foreground branch, while *C. auratus*, *S. grahami*, *P. huangchuch*, and *D. rerio* were designated as background branches. The null model used in the branch-site test assumed that the Ka/Ks ratios for all codons across all branches were $\leqslant 1$, whereas the alternative model indicated that the foreground branch contained codons evolving with Ka/Ks >1. We conducted a maximum likelihood ratio test (LRT) to evaluate these two models. The *p*-value was derived from the chi-square distribution with 1 degree of freedom (df = 1). Subsequently, *p* values underwent adjustment for multiple comparisons using the false discovery rate (FDR) method. Genes were classified as positively selected with an FDR threshold of less than 0.05. Additionally, we required that at least one amino acid site exhibit a high probability of positive selection (Bayes probability >95%). Genes failing to satisfy this criterion at any amino acid site were designated as false positives and consequently excluded from further

consideration. GO enrichment was conducted using clusterProfiler version 4.2.2 (RRID: SCR_016884) [80].

Results

T2T genome assembly and completeness evaluation

The assembly of the CC 4.0 was achieved through the integration of diverse sequencing technologies, including Illumina whole-genome short reads, PacBio HiFi, ONT ultra-long reads, and Hi-C sequencing. In total, 70.05 Gb (~43.31× coverage) of Illumina whole-genome short reads, 223.46 Gb (~138.17× coverage) of PacBio HiFi reads, 252.59 Gb of ONT ultra-long reads (~156.18× coverage), and 219.26 Gb (~135.57× coverage) of Hi-C data (Supplementary Table S1) were generated. Notably, the N50 length surpassed 15 kb for HiFi reads and 59 kb for ONT reads (Supplementary Table S1). Through *k*-mer analysis of WGS reads, the estimated CC 4.0 genome size was 1.62 Gb with a heterozygosity level of 0.45% (Supplementary Table S2 and Supplementary Fig. S1).

Independent assembly of the ONT reads using NextDenovo resulted in a total length of 2.00 Gb with an N50 length of 12.79 Mb (Supplementary Table S3). Furthermore, integration of ONT and HiFi reads via hifiasm yielded a total size of 1.58 Gb with an N50 length of 29.44 Mb, providing a more continuous assembly. The hifiasm initial assembly served as the backbone for scaffolding contigs into 50 pseudochromosomes using Hi-C data. Our results revealed that 34 pseudochromosomes were composed solely of a single contig, while 22 gaps were distributed across the remaining 16 pseudochromosomes (Supplementary Table S4 and Supplementary Fig. S2). After gap filling and polishing, the CC 4.0 genome achieved a total size of 1555.86 Mb with

an N50 of 30.45 Mb, comprising 50 gap-free pseudochromosomes ranging in length from 20.70 to 49.02 Mb (Fig. 2A; Table 1).

To validate the accuracy and completeness of the CC 4.0 genome assembly, multiple strategies were implemented. Firstly, the Hi-C heatmap demonstrated a high degree of consistency across all pseudochromosomes, confirming the precision in sequencing, ordering, and orientation of contigs (Fig. 2B). Based on collinearity analysis, the CC 4.0 genome basically has a syntenic relationship and good coverage with the CC 3.0 genome (Supplementary Fig. S3). Secondly, all 22 gaps were successfully closed, with both HiFi and ONT reads achieving a 100% genome alignment rate, while WGS reads demonstrated a rate of 99.79%. Thirdly, the Merqury-estimated quality value of the CC 4.0 genome was 47.95, attesting to the high accuracy of the assembly (Table 1). Furthermore, 83 out of 100 telomeres were detected, resulting in 33 T2T pseudomolecules for the entire genome (Fig. 2C and Supplementary Table S5). The GCI score for the CC 4.0 genome was 85.82, comparable to the human T2T (CHM13) genome's GCI score of 87.04, indicating that the assembly quality of the CC 4.0 genome meets the current standards for T2T assembly [50]. Lastly, the Benchmarking Universal Single-Copy Orthologs (BUSCO) evaluation revealed that the CC 4.0 genome successfully identified 99.20% of the 3,640 actinopterygii gene set (Table 1). The CC 4.0 genome completeness was higher than that of the recently reported twenty-one cyprinid genomes (average 95.60%, from 91.7 to 96.6%) and the CC 3.0 genome [10, 81]. Overall, these validations affirm the superior quality and reliability of the CC 4.0 genome assembly.

Annotation of repetitive elements and protein-coding genes

Approximately 696.41 Mb of the assembled CC 4.0 genome was classified as repetitive sequences, representing 44.76% of the genome (Table 1; Supplementary Table S6). The percentage of repetitive sequences was higher than the previously reported (31.3%-43.40%) [2, 3, 10]. Among the interspersed repetitive sequences, the majority consisted of DNA transposons, making up 25.94% of the genome. (Supplementary Table S7). The long terminal repeat (LTR) and long interspersed nuclear elements classes accounted for 11.25% and 11.15% of the genome, respectively (Supplementary Table S7). Additionally, 39,065 noncoding RNAs were annotated, including 4,026 microRNAs, 24,096 transfer RNAs, 3,249 small nuclear RNAs, and 7,694 ribosomal RNAs (Supplementary Table S8).

Using a combined prediction strategy, a total of 50,187 protein-coding genes were identified, with an average of 8.87 exons per gene (Table 1). BUSCO assessment demonstrated 97.77% completeness with only 1.13% missing genes, indicating robust gene annotation (Fig. 3A). The length distribution of messenger RNA, coding sequences, exons, and introns among related species supported the reliability of the annotation results (Fig. 3B). Of the predicted genes, 49,326 (98.36%) contained at least one conserved functional domain, and 36,887 (73.50%) genes showed detectable transcriptional activity (FPKM$\geqslant$1) (Supplementary Table S9; Supplementary Table S10). In addition, 3,918 TFs were predicted across 77 types, surpassing the count in the CC 3.0 genome (3,812) [10]. The top 10 TF families with the highest gene counts were zf-H2C2_2, Homeodomain, zf-C2H2, HLH, BTB, TF-bZIP, Forkhead, HMG_box, THAP, and Myc_DNA binding (Supplementary Fig. S4). These findings affirmed the completeness and accuracy of gene prediction in the CC 4.0 genome.

The characteristics of centromeric regions

The centromeric sequences of the 50 pseudochromosomes in the CC 4.0 genome were assembled, with an average length of 748,299 bp (Table 2). The longest centromeric region measured 1,877,250 bp on pseudochromosome A5, while the shortest measured 30,001 bp on pseudochromosome A14. Both mean and maximal lengths of koi carp centromeres were significantly shorter than those of the Yangtze finless porpoise (mean: 1,500,346 bp; maximum: 10,904,684 bp) [82]. Centromeric regions typically exhibited higher repeat sequence density and lower gene density (Fig. 2C). In summary, the average percentage of repetitive sequences in centromeric regions was 95.10%, with a total of 95 genes located in the centromeric regions. The genes located in the centromeric regions exhibited significant enrichment in ten GO terms, including DNA integration, nucleic acid binding, protein export from nucleus, nuclear export signal receptor activity, aspartic-type endopeptidase activity, motile cilium, nuclear-transcribed mRNA catabolic process, exonucleolytic, 3'-5', cell motility, proteolysis, and ubiquitin-protein transferase activity (Supplementary Fig. S5). In the T2T genome research on rice, genes in the centromere region were also enriched in the GO term of nucleic acid binding [83]. This suggested that the function of centromeres might highly conserved among eukaryotes. In many eukaryotes, centromeres were composed of tandemly repeated DNA sequences known as satellite DNA. As previously reported, satellite repeats constitute the primary centromeric repeat class in both human and macaque genomes [84, 85]. Within the centromeric regions of the CC 4.0 assembly, the predominant repetitive sequence classes included satellite and simple repeats, followed by DNA transposons and LTRs (Table 2).

Genomic variations between CC 4.0 and Songpu2021

In the regions of synteny between CC 4.0 and Songpu2021, 17,822,292 SNPs and 5,555,326 InDels were identified. Most of these variants were distributed in intronic (52.50% for SNPs, 52.85% for InDels) and intergenic (35.44% for SNPs, 36.26% for InDels) regions. Conversely, only 3.42% of SNPs and 1.68% of InDels resided within exonic regions (Supplementary Table S11). High-quality genome assemblies facilitated comprehensive SV analysis. A total of 179,321 SVs with an average size of 1108.25 bp were detected. Of which, 63,568 (35.45%) resided within potential expression regulatory domains or CDS of reference genes, termed "SV-genes". These genes were referred to as "SV-genes". GO enrichment analysis revealed significant over-representation of SV-genes in four functional categories: DNA integration, 2-oxoglutarate-dependent dioxygenase activity, nucleic acid binding, and proteolysis (Fig. 4A). By leveraging RNA-seq data, we found that SVs exerted negative effects on gene expression in scale tissue, whereas no such impacts were observed in fin tissue (Fig. 4B). These genomic variations serve as a comprehensive repository for subsequent investigations in both fundamental and applied studies of koi carp.

Phylogenetic relationship analysis

The protein-coding genes from six vertebrate species, including *P.huangchuchieni*, *C.auratus*, *D.rerio*, *O.latipes*, *S.grahami*, and *H.sapiens*, were clustered into 18,442 gene families together with the protein-coding genes of CC 4.0 genome (Supplementary Table S12; Supplementary Table S13). Among these, 12,320 gene families were shared among *P.huangchuchieni*, *C.auratus*, *S.grahami*, and *C. carpio* (Fig. 5A). Additionally, 245 gene families with 589 genes were identified as specific to common carp when compared to the other six species (Fig. 5B). Of these common

carp-specific genes, 545 (92.53%) had functional annotations (Supplementary Table S14). These specific genes were significantly enriched in six pathways: "Fructose and mannose metabolism", "Caffeine metabolism", "Phosphatidylinositol signaling system", "Thyroid hormone signaling pathway", "AMPK signaling pathway", and "Glycerolipid metabolism" (Fig. 5C).

A phylogenetic tree was constructed using 241 single-copy orthologous genes, with *H. sapiens* as the outgroup (Fig. 5D). The estimated divergence time between *C. carpio* and *C. auratus* was approximately 34.7 million years ago (MYA). Compared to the most recent common ancestor (MRCA), common carp exhibited 87 expansion and 66 contraction events in gene families ($p <= 0.05$). The expanded gene families in common carp included 1,420 genes and were primarily enriched in 13 pathways, such as "Olfactory transduction", "RNA degradation", "NOD-like receptor signaling pathway", "Neuroactive ligand-receptor interaction", "Necroptosis", "Mineral absorption", "Ferroptosis", "Complement and coagulation cascades", "Glycerophospholipid metabolism", "TNF signaling pathway", "Apoptosis", "Gap junction", and "Cholinergic synapse" (Fig. 5C). Notably, the immune genes in common carp identified by genome-wide association analysis was reported to involve in several immune response-related pathways, including NOD-like receptor signaling pathway [13].

Positively selected genes

A total of 3,438 one-to-one orthologous gene sets in five teleost fish (*C. auratus*, *C.carpio*, *S. grahami*, *P. huangchuchieni*, and *D. rerio*) were analyzed for positively selected gene (PSG) detection analysis. Ultimately, 124 genes were identified as PSGs (Supplementary Table S15). These PSGs were linked to various biological processes, including binding (GO:0005488; 58

genes), cellular process (GO:0009987; 46 genes), catalytic activity (GO:0003824, 43 genes), single-organism process (GO:0044699; 40 genes), and others (Fig. 6A). GO enrichment analysis revealed that these genes were significantly associated with enzyme activities like metallopeptidase activity, metalloendopeptidase activity, methyltransferase activity, and RNA helicase activity, and so on (Fig. 6B). The metalloendopeptidase has been reported to play a significant role in the central nervous system and has been associated with various diseases including breast cancer, prostate cancer, and essential hypertension [86-89].

Conclusions

The first T2T genome assembly of common carp was achieved using PacBio HiFi reads, ONT ultra-long sequencing, and Hi-C technologies, characterized by high completeness and accuracy. A total of 50 pseudochromosomes were assembled, with 33 meeting the T2T standard. All 50 centromeres in the CC 4.0 genome were predicted, with an average length of 748,299 bp, typically showing higher repeat sequence density and lower gene density. Genes in centromeric regions were significantly enriched in ten GO terms related to DNA and protein processes. The assembly predicted 696.41 Mb of repetitive sequences and identified 50,187 protein-coding genes. In addition, 3,918 TFs were predicted. Comparative genomics analysis revealed 589 genes specific to koi carp. Moreover, 87 expansion and 66 contraction events were obtained. Evolutionary analysis suggested that metalloendopeptidase activity may be crucial for koi carp. A total of 124 PSGs were identified in common carp, which were associated with various biological processes and enzyme activities, such as metallopeptidase activity. This dataset serves as a valuable resource for future genetic breeding research in koi carp and common carp.

Abbreviations

BLAST: Basic Local Alignment Search Tool; BUSCO: Benchmarking Universal Single-Copy Orthologs; CCS: circular consensus sequencing; Gb: gigabase pairs; GCI: Genome Continuity Inspector; GO: Gene Ontology; Hi-C: High-Throughput Chromosome Conformation Capture; HiFi: High-Fidelity; InDels: insertions/deletions; KEGG: Kyoto Encyclopedia of Genes and Genomes; LINE: long interspersed nuclear element; LTR: long terminal repeat; Mb: megabase pairs; MRCA: most recent common ancestor; MYA: million years ago; PASA: Program to Assemble Spliced Alignments; PSG: positively selected gene; QV: quality value; RNA-seq: RNA sequencing; SNPs: single nucleotide polymorphisms; SVs: structural variants; WGD: whole genome duplication; WGS: whole-genome sequencing; T2T: telomere-to-telomere

Tables

Table 1. Statistics for the common carp and koi carp genome assembly.

**Genomic feature**	**CC 4.0**	**CC 3.0**	**Songpu2021**
Total size (Mb)	1555.86	1579.38	1531.01
Number of chromosomes	50	50	50
**Gap number**	**0**	**1,089**	**22,301**
Chromosome N50 (Mb)	30.45	28.32	30.48
GC content (%)	37.20	37.20	37.00
Protein-coding genes number	50,187	55,981	41,939
Repetitive sequences (%)	44.76	43.40	40.09
Genome BUSCOs (%)	99.20	99.10	98.96
GCI score	85.82	NA	NA
WGS reads mapping rate (%)	99.79	NA	NA
ONT reads mapping rate (%)	100.00	NA	NA
HiFi reads mapping rate (%)	100.00	NA	NA
Quality value	47.95	NA	NA

Note: The CC 3.0 genome was downloaded from Genome Warehouse database under accession GWHBHRW00000000. The Songpu2021 genome was retrieved from the NCBI database under the accession number GCA_018340385.1. NA means not avaiable.

Table 2. The characteristic of centromeric regions of the koi carp CC 4.0 assembly.

Chr	Start	End	Length	Gene number	Total	Trf	Satellite	Simple repeat	DNA transposons	LTR
A1	6,983,780	7,145,651	161,872	2	81.31	60.02	0.00	0.00	13.97	54.67
A2	10,552,693	11,217,077	664,385	0	99.80	97.66	34.62	81.27	0.17	10.08
A3	31,879,419	33,636,405	1,756,987	15	94.48	47.82	0.52	0.01	23.15	19.92
A4	21,620,000	21,850,000	230,001	0	99.51	91.05	38.95	68.48	0.20	19.43
A5	32,599,339	34,476,588	1,877,250	4	95.23	78.50	37.86	38.32	19.57	18.10
A6	11,508,992	12,370,832	861,841	0	97.97	93.41	32.03	76.06	1.69	18.41
A7	11,516,427	13,371,720	1,855,294	5	97.97	92.18	32.58	34.23	4.61	12.75
A8	24,955,543	25,632,915	677,373	2	98.40	92.83	10.21	81.02	1.14	5.10
A9	29,694,335	30,547,340	853,006	4	90.68	74.76	73.54	0.00	9.05	6.00
A10	22,177,277	22,829,639	652,363	0	99.55	77.31	32.70	84.61	0.16	5.35
A11	4,746,905	5,590,589	843,685	0	96.67	69.23	69.71	29.75	0.32	0.52
A12	17,378,483	18,406,913	1,028,431	2	95.07	84.22	46.46	50.69	8.00	3.01
A13	4,680,000	4,900,000	220,001	0	90.67	89.23	78.71	11.37	0.00	0.13
A14	23,400,000	23,430,000	30,001	0	94.82	91.60	63.58	0.00	1.36	0.00
A15	19,963,423	21,668,624	1,705,202	2	95.22	82.73	42.88	51.19	8.10	5.78
A16	14,555,504	14,717,133	161,630	1	88.13	21.87	0.00	0.00	86.60	1.64
A17	3,716,890	4,150,698	433,809	3	99.97	62.40	1.99	0.00	0.26	29.53
A18	11,933,667	12,590,459	656,793	2	95.02	89.14	27.61	78.28	2.76	9.08
A19	2,597,145	3,177,452	580,308	2	96.10	94.22	21.03	87.82	1.42	6.54
A20	23,703,082	24,668,971	965,890	5	88.97	69.01	66.65	0.01	8.92	14.15
A21	16,706,975	17,545,549	838,575	1	95.78	88.81	16.58	82.56	3.99	8.95
A22	19,320,532	19,979,681	659,150	0	98.32	96.21	39.26	79.33	0.77	10.22
A23	1,348,620	2,293,930	945,311	1	100.00	98.81	57.69	52.65	0.00	17.76
A24	16,939,420	17,124,316	184,897	4	88.30	7.58	0.00	0.00	48.20	42.52
A25	18,285,858	19,188,655	902,798	0	97.68	91.44	69.84	24.54	0.86	7.48
B1	15,950,000	16,440,000	490,001	0	99.88	95.39	17.92	53.81	0.00	0.00
B2	22,023,874	22,670,773	646,900	6	90.32	72.71	73.80	0.00	11.34	5.13
B3	35,614,767	35,952,947	338,181	0	97.14	91.61	88.11	17.69	5.52	1.09
B4	33,360,418	33,527,013	166,596	0	99.39	97.34	0.00	0.00	0.33	0.00
B5	11,034,427	12,148,201	1,113,775	1	94.60	83.31	72.53	9.66	7.56	6.22
B6	16,740,439	17,090,269	349,831	1	96.16	93.15	0.00	0.00	1.17	15.35
B7	12,778,416	13,657,366	878,951	1	99.24	71.75	29.25	74.78	0.70	14.21
B8	2,758,449	3,284,545	526,097	2	93.21	86.27	55.03	49.18	2.79	14.59
B9	4,331,995	4,452,008	120,014	0	75.44	42.01	0.00	0.00	30.85	36.93

B10	2,079,927	2,213,557	133,631	0	95.15	78.41	0.54	50.58	5.73	50.44
B11	21,120,000	22,240,000	1,120,001	0	99.59	99.48	38.50	81.92	0.09	3.06
B12	15,879,559	17,438,428	1,558,870	6	95.57	85.19	39.74	67.93	8.58	9.30
B13	25,361,200	26,195,054	833,855	4	86.95	56.30	50.37	11.58	18.10	12.22
B14	23,790,000	24,620,000	830,001	1	94.93	12.26	13.52	79.35	0.76	18.71
B15	11,292,907	11,974,689	681,783	3	97.13	2.80	11.85	0.00	12.58	1.83
B16	24,380,000	25,210,000	830,001	1	99.77	98.92	38.71	84.91	0.19	7.52
B17	23,480,000	24,130,000	650,001	1	96.47	77.13	62.70	43.82	0.18	9.51
B18	10,339,546	11,415,040	1,075,495	8	89.51	59.19	44.49	0.59	24.08	17.50
B19	26,221,041	26,766,840	545,800	1	98.22	90.31	72.13	30.39	2.66	5.52
B20	4,550,000	4,900,000	350,001	0	98.63	98.56	27.54	95.27	0.13	0.00
B21	8,310,000	9,300,000	990,001	0	97.21	2.16	50.79	74.63	0.00	7.16
B22	36,566,575	37,323,917	757,343	1	97.09	92.48	23.27	67.10	5.81	22.69
B23	1,540,000	2,230,000	690,001	0	93.82	0.15	21.82	81.62	0.26	7.16
B24	16,763,174	17,679,191	916,018	3	94.99	84.22	37.99	54.93	10.27	7.39
B25	1,437,545	2,512,481	1,074,937	0	98.86	96.59	39.80	76.90	2.90	1.04

Additional Files

Supplementary Table S1. Summary of the data sequenced by multiple technologies.

Supplementary Table S2. *K*-mer analysis.

Supplementary Table S3. The statistics of initial assembly.

Supplementary Table S4. The statistics of the anchored chromosome length.

Supplementary Table S5. The identified telomeres in CC 4.0 assembly.

Supplementary Table S6. General statistics of repeats in CC 4.0 assembly.

Supplementary Table S7. The summary of interspersed repeat contents in CC 4.0 assembly.

Supplementary Table S8. Non-coding RNAs in CC 4.0 assembly.

Supplementary Table S9. Summary of gene function annotation.

Supplementary Table S10. The gene expression matrix.

Supplementary Table S11. The categories of SNPs and InDels with CC 4.0 as reference.

Supplementary Table S12. The data sources of six vertebrate genomes.

Supplementary Table S13. Statistics for the orthologous gene families of seven species genomes.

Supplementary Table S14. The list of koi carp-specific genes.

Supplementary Table S15. The list of 124 positive selection genes.

Author Contributions

Jiandong Yuan, Jun Yong, and Yongchao Niu designed this study; Jun Yong, Huijuan Guo and Xuewu Liao collected the samples and performed the experiments; Jiang Li and Yongchao Niu performed the data analysis; Jiandong Yuan, Jiang Li, and Yongchao Niu wrote the first draft of the manuscript. All other authors proofread and revised the manuscript. All authors read and approved the final manuscript.

Funding

This study was supported by Suxin Koi Farm.

Data Availability

The raw sequencing data that support the findings of this study have been deposited into the CNGB Sequence Archive (CNSA) of China National GeneBank DataBase (CNGBdb) with accession number CNP0006400. Genomic assembly sequences (FASTA files), gene annotation files (GFF3), functional annotations, and variant results are publicly accessible via figshare (https://figshare.com/s/93779280afb6ad32eafb).

Competing Interests

The authors declare that they have no competing interests.

Acknowledgements

We thank every project that provides funding and material support for the study. We also thank each author for their ideas and skills in study design, experimentation, data collection, data analysis, and manuscript writing.

Ethics statement

This study was carried out in accordance with the recommendations of the care and use of animals for scientific purposes set up by the Animal Care and Use Committee of Chinese Academy of Fishery Sciences (ACUC-CAFS).

References

1. Bostock, J., et al., Aquaculture: global status and trends. Philosophical transactions of the Royal Society of London. Series B, Biological sciences, 2010. **365**(1554): p. 2897-2912.
2. Xu, P., et al., Genome sequence and genetic diversity of the common carp*, Cyprinus carpio.* Nature genetics, 2014. **46**(11): p. 1212-1219.
3. Xu, P., et al. The allotetraploid origin and asymmetrical genome evolution of the common carp Cyprinus carpio. Nature communications, 2019. **10**, 4625.
4. Ren, R., et al., Widespread Whole Genome Duplications Contribute to Genome Complexity and Species Diversity in Angiosperms. Molecular plant, 2018. **11**(3): p. 414-428.
5. Chen, Z., et al. *De novo* assembly of the goldfish (*Carassius auratus*) genome and the evolution of genes after whole-genome duplication. Science advances, 2019. **5**.
6. Kon, T., et al., The Genetic Basis of Morphological Diversity in Domesticated Goldfish. Current biology : CB, 2020. **30**(12): p. 2260-2274.e6.
7. Luo, J., et al. From asymmetrical to balanced genomic diversification during rediploidization: Subgenomic evolution in allotetraploid fish. Science advances, 2020. **6**, eaaz7677.
8. Chen, L., et al., Chromosome-level genome of Poropuntius huangchuchieni provides a diploid progenitor-like reference genome for the allotetraploid Cyprinus carpio. Molecular ecology resources, 2021. **21**(5): p. 1658-1669.
9. Li, J.-T., et al., Parallel subgenome structure and divergent expression evolution of allo-tetraploid common carp and goldfish. Nature Genetics, 2021. **53**(10): p. 1493-1503.

10. Chen, L., et al. Evolutionary divergence of subgenomes in common carp provides insights into speciation and allopolyploid success. Fundamental research, 2024. **4**, 589-602.
11. Zhang, Y., et al. Identification of common carp innate immune genes with whole-genome sequencing and RNA-Seq data. Journal of integrative bioinformatics, 2011. **8**, 169.
12. Verma, D.K., et al., Transcriptome analysis reveals immune pathways underlying resistance in the common carp Cyprinus carpio against the oomycete Aphanomyces invadans. Genomics, 2021. **113**(1, Part 2): p. 944-956.
13. Jiang, Y., et al., Genomic features of common carp that are relevant for resistance against Aeromonas hydrophila infection. Aquaculture, 2022. **547**: p. 737512.
14. Wang, J., Q. Zhou, and Y. Jiang, Genome-wide analysis of common carp (Cyprinus carpio) mucin genes and their roles in mucosal immune response following the Aeromonas hydrophila infection. Comparative Immunology Reports, 2024. **7**: p. 200167.
15. Wang, M., et al., Asymmetric and parallel subgenome selection co-shape common carp domestication. BMC Biology, 2024. **22**(1): p. 4.
16. Shi, X., et al., Genome-wide association study reveals candidate genes critical for skin pigmentation in common carp (Cyprinus carpio) strains including koi. Aquaculture, 2024. **590**: p. 741075.
17. Nurk, S., et al., *T*he complete sequence of a human genome. Science, 2022. **376**(6588): p. 44-53.
18. Luo, L.-Y., et al., Telomere-to-telomere sheep genome assembly reveals new variants associated with wool fineness trait. 2024, bioRxiv.
19. You, X., et al., A near complete genome assembly of the East Friesian sheep genome. Scientific Data, 2024. **11**(1): p. 762.
20. Shang, L., et al., A complete assembly of the rice Nipponbare reference genome. Molecular plant, 2023. **16**(8): p. 1232-1236.
21. Chen, J., et al., A complete telomere-to-telomere assembly of the maize genome. Nature genetics, 2023. **55**(7): p. 1221-1231.
22. Li, M., et al. Telomere-to-telomere genome assembly of sorghum. Scientific data, 2024. **11**, 835.
23. Li, H. and R. Durbin, Genome assembly in the telomere-to-telomere era. Nature Reviews Genetics, 2024. **25**(9): p. 658-670.
24. Garg, V., et al., Unlocking plant genetics with telomere-to-telomere genome assemblies. Nature Genetics, 2024. **56**, 1788–1799
25. Chin, C., et al., Nonhybrid, finished microbial genome assemblies from long-read SMRT sequencing data. Nature Methods, 2013. **10**: p. 563-569.
26. De Coster, W., et al., NanoPack: visualizing and processing long-read sequencing data. Bioinformatics, 2018. **34**: p. 2666 - 2669.
27. Belton, J.-M., et al., Hi-C: a comprehensive technique to capture the conformation of genomes. Methods, 2012. **58 3**: p. 268-76.
28. Chen, S., et al., fastp: an ultra-fast all-in-one FASTQ preprocessor. Bioinformatics, 2018. **34**(17): p. i884-i890.
29. Sun, C., et al., Chromosome-level genome assembly for the largemouth bass Micropterus salmoides provides insights into adaptation to fresh and brackish water. Molecular ecology resources, 2021. **21**(1): p. 301-315.
30. Marçais, G. and C. Kingsford, A fast, lock-free approach for efficient parallel counting of occurrences of k-mers. Bioinformatics, 2011. **27 6**: p. 764-70.
31. Liu, B., et al., Estimation of genomic characteristics by analyzing k-mer frequency in de novo genome projects. arXiv: Genomics, 2013.
32. Hu, J., et al., NextDenovo: an efficient error correction and accurate assembly tool for noisy long reads. Genome Biology, 2024. **25**(1): p. 107.

33. Cheng, H., et al., Haplotype-resolved de novo assembly using phased assembly graphs with hifiasm. Nature Methods, 2021. **18**(2): p. 170-175.
34. Hu, J., et al., NextPolish2:a repeat-aware polishing tool for genomes assembled using HiFi long reads. 2023, bioRxiv.
35. Langmead, B. and S.L. Salzberg, Fast gapped-read alignment with Bowtie 2. Nature Methods, 2012. **9**(4): p. 357-359.
36. Servant, N., et al., HiC-Pro: An optimized and flexible pipeline for Hi-C data processing. Genome Biology, 2015. **16**.
37. Durand, N., et al., Juicer Provides a One-Click System for Analyzing Loop-Resolution Hi-C Experiments. Cell Systems, 2016. **3**: p. 95-98.
38. Dudchenko, O., et al., *De novo* assembly of the Aedes aegypti genome using Hi-C yields chromosome-length scaffolds. Science, 2017. **356**: p. eaal3327.
39. Durand, N.C., et al., Juicebox Provides a Visualization System for Hi-C Contact Maps with Unlimited Zoom. Cell systems, 2016. **3**(1): p. 99-101.
40. Zhao, H., et al. Telomere-to-telomere genome assembly of the goose Anser cygnoides. Scientific data, 2024. **11**, 741.
41. Lin, Y., et al., quarTeT: a telomere-to-telomere toolkit for gap-free genome assembly and centromeric repeat identification. Horticulture Research, 2023. 10(8):uhad127.
42. Xu, G.-C., et al., LR_Gapcloser: a tiling path-based gap closer that uses long reads to complete genome assembly. GigaScience, 2018. **8**. giy157.
43. Zhou, Y., et al. Gap-free genome assembly of Salangid icefish Neosalanx taihuensis. Scientific data, 2023. **10**, 768.
44. Jain, C., et al., Long-read mapping to repetitive reference sequences using Winnowmap2. Nature methods, 2022. **19**(6): p. 705-710.
45. Vaser, R., et al., Fast and accurate de novo genome assembly from long uncorrected reads. Genome research, 2017. **27 5**: p. 737-746.
46. Seppey, M., M. Manni, and E.M. Zdobnov, BUSCO: Assessing Genome Assembly and Annotation Completeness. Methods in molecular biology, 2019. **1962**: p. 227-245.
47. Rhie, A., et al., Merqury: reference-free quality, completeness, and phasing assessment for genome assemblies. Genome Biology, 2020. **21**.
48. Li, H. and R. Durbin, Fast and accurate short read alignment with Burrows–Wheeler transform. Bioinformatics, 2009. **25**(14): p. 1754-1760.
49. Li, H., Minimap2: pairwise alignment for nucleotide sequences. Bioinformatics, 2018. **34**(18): p. 3094-3100.
50. Chen, Q., et al., GCI: a continuity inspector for complete genome assembly. Bioinformatics, 2024. **40**(11).
51. Marçais, G., et al., MUMmer4: A fast and versatile genome alignment system. PLoS Computational Biology, 2018. **14**.
52. Benson, G., Tandem repeats finder: a program to analyze DNA sequences. Nucleic acids research, 1999. **27 2**: p. 573-80.
53. Flynn, J.M., et al., RepeatModeler2 for automated genomic discovery of transposable element families. Proceedings of the National Academy of Sciences of the United States of America, 2020. **117**(17): p. 9451-9457.
54. Xu, Z. and H. Wang, LTR_FINDER: an efficient tool for the prediction of full-length LTR retrotransposons. Nucleic Acids Research, 2007. **35**: p. W265 - W268.
55. Chen, N., Using RepeatMasker to Identify Repetitive Elements in Genomic Sequences. Current Protocols in Bioinformatics, 2004. **5**.

56. Bao, W., K.K. Kojima, and O. Kohany, Repbase Update, a database of repetitive elements in eukaryotic genomes. Mobile DNA, 2015. **6**.
57. You, X., et al. A near complete genome assembly of the East Friesian sheep genome. Scientific data, 2024. **11**, 762.
58. Grabherr, M.G., et al., Full-length transcriptome assembly from RNA-Seq data without a reference genome. Nature biotechnology, 2011. **29 7**: p. 644-52.
59. Haas, B., Improving the Arabidopsis genome annotation using maximal transcript alignment assemblies. Nucleic Acids Research, 2003. **31**: p. 5654-5666.
60. Kim, D., B. Langmead, and S.L. Salzberg, HISAT: a fast spliced aligner with low memory requirements. Nature Methods, 2015. **12**(4): p. 357-360.
61. Kovaka, S., et al., Transcriptome assembly from long-read RNA-seq alignments with StringTie2. Genome Biology, 2019. **20**(1): p. 278.
62. Jens, et al., GeMoMa: Homology-Based Gene Prediction Utilizing Intron Position Conservation and RNA-seq Data. Methods in Molecular Biology, 2019.
63. Holst, F., et al., Helixer–de novo Prediction of Primary Eukaryotic Gene Models Combining Deep Learning and a Hidden Markov Model. bioRxiv, 2023.
64. Haas, B.J., et al., Automated eukaryotic gene structure annotation using EVidenceModeler and the Program to Assemble Spliced Alignments. Genome Biology, 2008. **9**(1): p. R7.
65. Buchfink, B., C. Xie, and D.H. Huson, Fast and sensitive protein alignment using DIAMOND. Nature Methods, 2015. **12**(1): p. 59-60.
66. Bairoch, A. and R. Apweiler, The SWISS-PROT protein sequence data bank and its supplement TrEMBL in 1999. Nucleic Acids Research, 1999. **27**(1): p. 49-54.
67. Kanehisa, M. and S. Goto, KEGG: Kyoto Encyclopedia of Genes and Genomes. Nucleic Acids Research, 2000. **28**(1): p. 27-30.
68. Jones, P., et al., InterProScan 5: genome-scale protein function classification. Bioinformatics, 2014. **30**(9): p. 1236-1240.
69. Wu, Y.-M., et al. Investigation of sex expression profiles and the cantharidin biosynthesis genes in two blister beetles. PloS one, 2023. **18**, e0290245.
70. Shen, W.-K., et al., AnimalTFDB 4.0: a comprehensive animal transcription factor database updated with variation and expression annotations. Nucleic acids research, 2023. **51**(D1): p. D39-D45.
71. Li, H., et al., TreeFam: a curated database of phylogenetic trees of animal gene families. Nucleic acids research, 2006. **34**(Database issue): p. D572-80.
72. Altschul, S.F., et al., Basic local alignment search tool. Journal of Molecular Biology, 1990. **215**(3): p. 403-410.
73. Edgar, R.C., Muscle5: High-accuracy alignment ensembles enable unbiased assessments of sequence homology and phylogeny. Nature Communications, 2022. **13**(1): p. 6968.
74. Minh, B.Q., et al., IQ-TREE 2: New Models and Efficient Methods for Phylogenetic Inference in the Genomic Era. Molecular Biology and Evolution, 2019. **37**: p. 1530 - 1534.
75. Yang, Z., PAML: a program package for phylogenetic analysis by maximum likelihood. Computer applications in the biosciences : CABIOS, 1997. **13 5**: p. 555-6.
76. Hedges, S.B., J.T. Dudley, and S. Kumar, TimeTree: a public knowledge-base of divergence times among organisms. Bioinformatics, 2006. **22 23**: p. 2971-2.
77. Bie, T.D., et al., CAFE: a computational tool for the study of gene family evolution. Bioinformatics, 2006. **22 10**: p. 1269-71.
78. Xie, C., et al., KOBAS 2.0: a web server for annotation and identification of enriched pathways and diseases. Nucleic acids research, 2011. **39**: p. W316-22.

79. Wang, Y., et al., Genomic insights into the seawater adaptation in Cyprinidae. BMC Biology, 2024. **22**(1): p. 87.
80. Wu, T., et al., clusterProfiler 4.0: A universal enrichment tool for interpreting omics data. The Innovation, 2021. **2**.
81. Xu, M.-R.-X., et al., Maternal dominance contributes to subgenome differentiation in allopolyploid fishes. Nature Communications, 2023. **14**.
82. Yin, D., et al., Telomere-to-telomere gap-free genome assembly of the endangered Yangtze finless porpoise and East Asian finless porpoise. GigaScience, 2024. **13**: p. giae067.
83. Song, J.-M., et al., Two gap-free reference genomes and a global view of the centromere architecture in rice. Molecular Plant, 2021. **14**(10): p. 1757-1767.
84. Zhang, S., et al., Integrated analysis of the complete sequence of a macaque genome. Nature, 2025.
85. Altemose, N., et al., Complete genomic and epigenetic maps of human centromeres. Science, 2022. **376**(6588): p. eabl4178.
86. Ding, J., et al. Membrane metalloendopeptidase (MME) is positively correlated with systemic lupus erythematosus and may inhibit the occurrence of breast cancer. PloS one, 2023. **18**, e0289960.
87. Cheng, C.-Y., et al., Membrane metalloendopeptidase suppresses prostate carcinogenesis by attenuating effects of gastrin-releasing peptide on stem/progenitor cells. Oncogenesis, 2020. **9**(3): p. 38.
88. Moskalenko, M., et al., Polymorphisms of the matrix metalloproteinase genes are associated with essential hypertension in a Caucasian population of Central Russia. Scientific Reports, 2021. **11**(1): p. 5224.
89. Cervellini, I., et al., Membrane metallo-endopeptidase is dispensable for repair after nerve injury. Glia, 2019. **67**(10): p. 1990-2000.

Figures and Legends

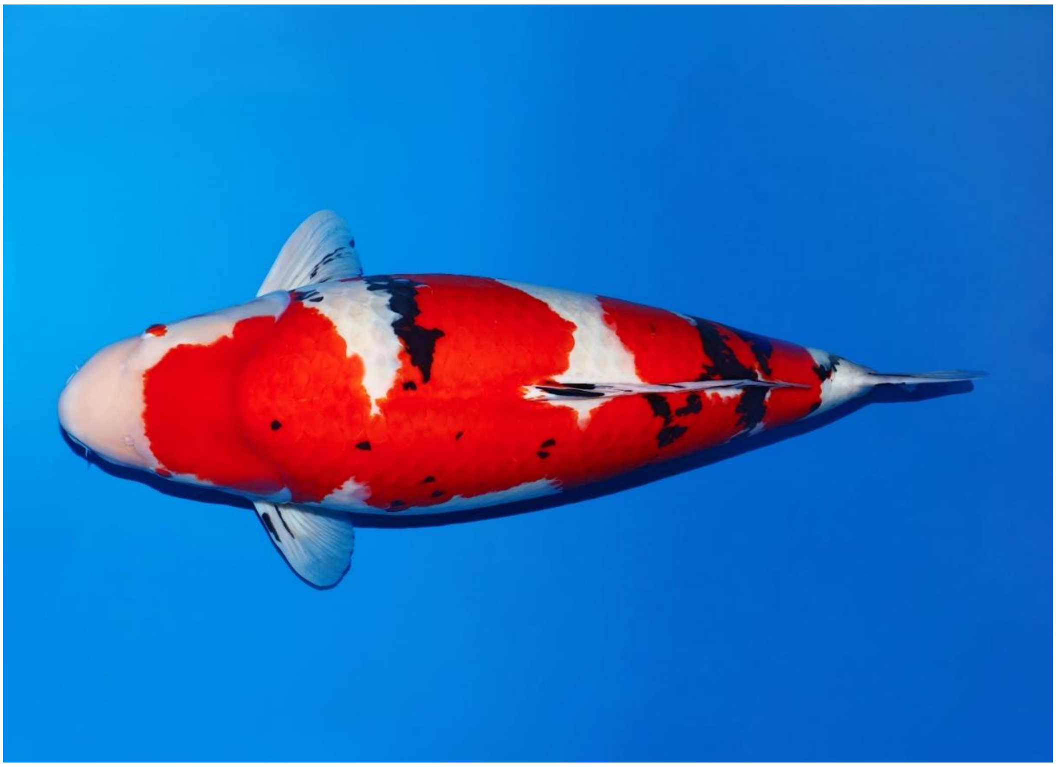

Fig. 1 | The koi carp strain Taisho Sansyoku in this study.

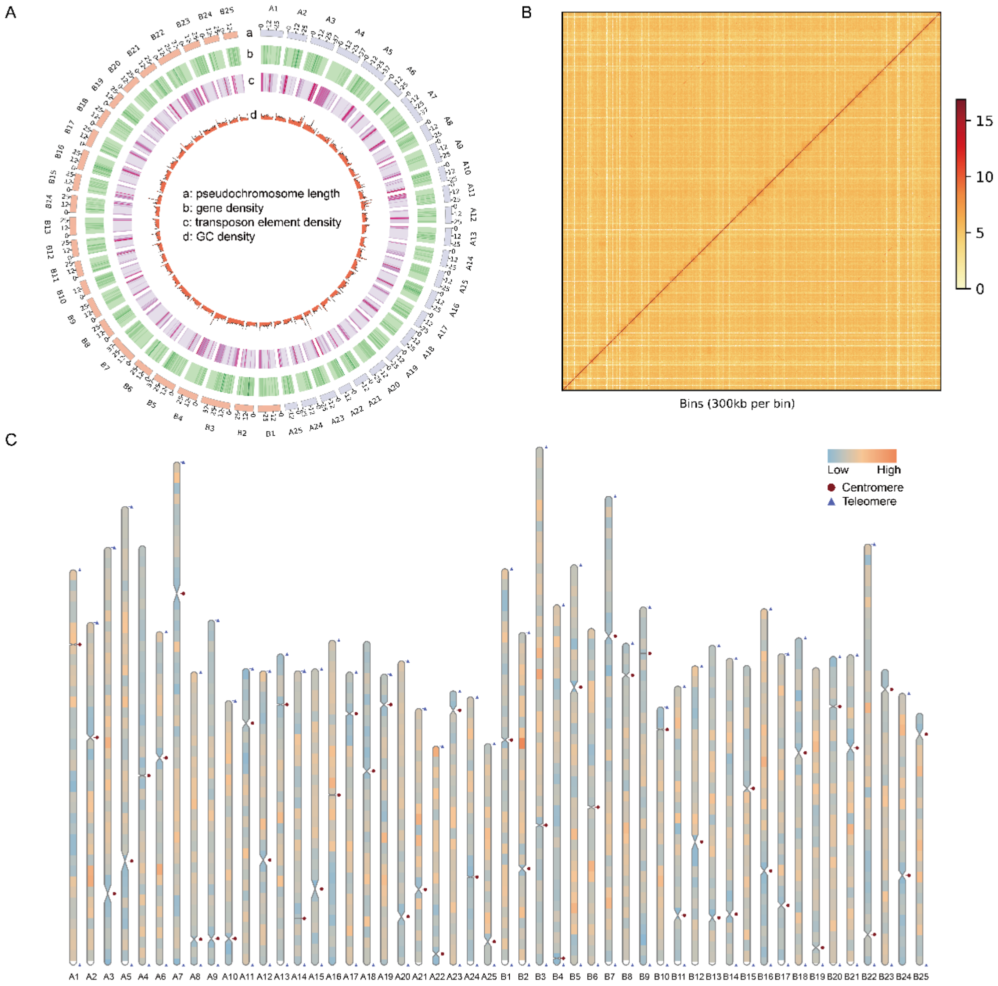

Fig. 2 | Genomic characteristics of the CC 4.0 genome. A, Circos plot showing the characterization of CC 4.0 genome. From outside to inside: a, The length of pseudo-chromosome in the size of Mb. b, gene density in 1-Mb sliding windows. c, percentage of transposon elements in 1 Mb sliding windows. d, GC content in non-overlapping 1Mb windows. **B**, Intensity signal heat map of the Hi-C chromosome interaction. The colour block illuminates the intensity of interaction from yellow (low) to red (high). **C**, Telomere and centromere detection map. Triangles and circles represent telomeres and centromere within the CC 4.0 assembled chromosomes. The orange color represents regions with high gene density, while the sky blue color represents regions with low gene density.

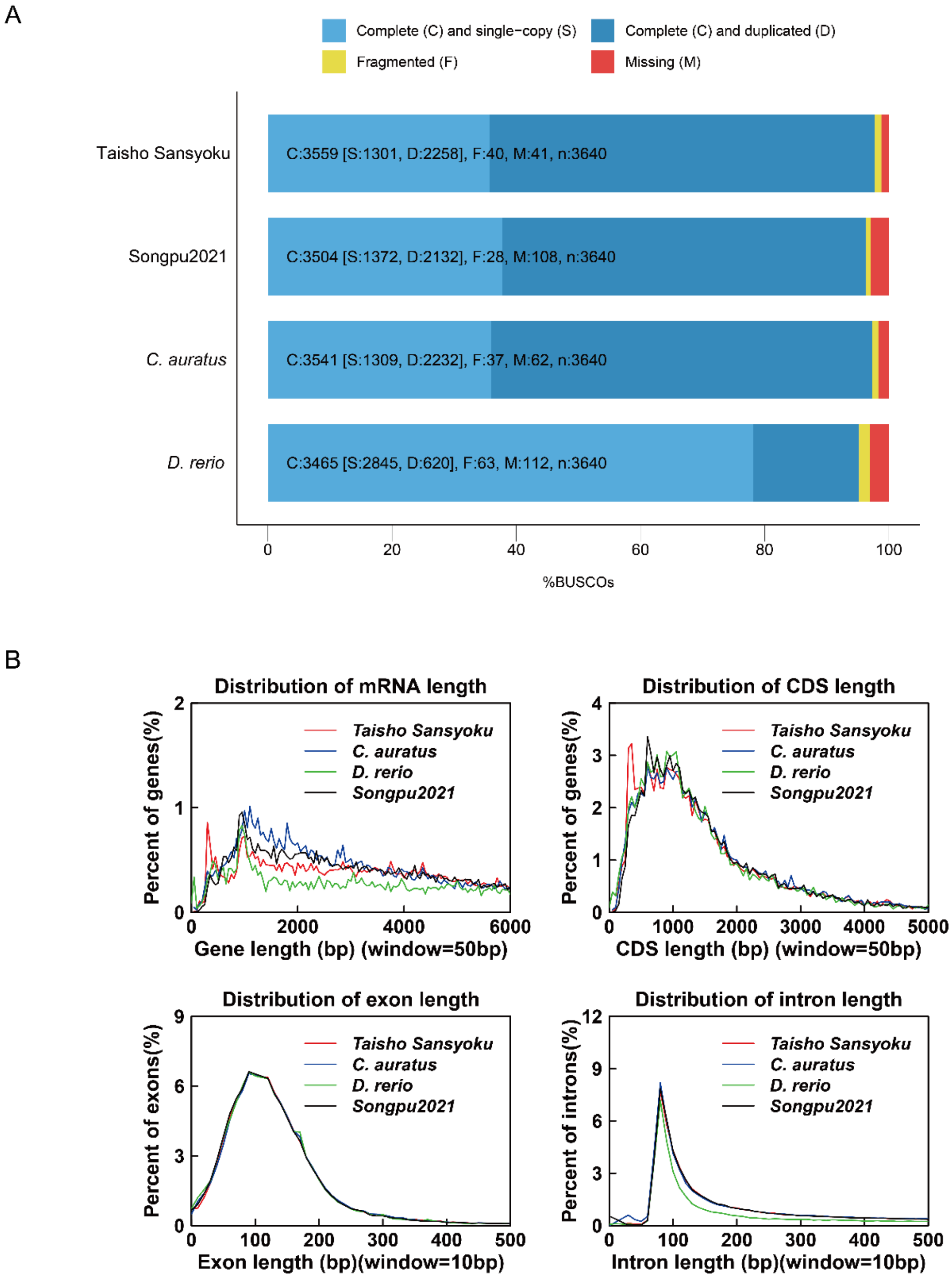

Fig. 3 | The high-quality of gene annotation. A, BUSCO assessments of Taisho Sansyoku, *C. auratus*, *D. rerio,* and Songpu2021. **B,** The composition of gene elements in the koi carp CC 4.0 genome compared to the other three genomes.

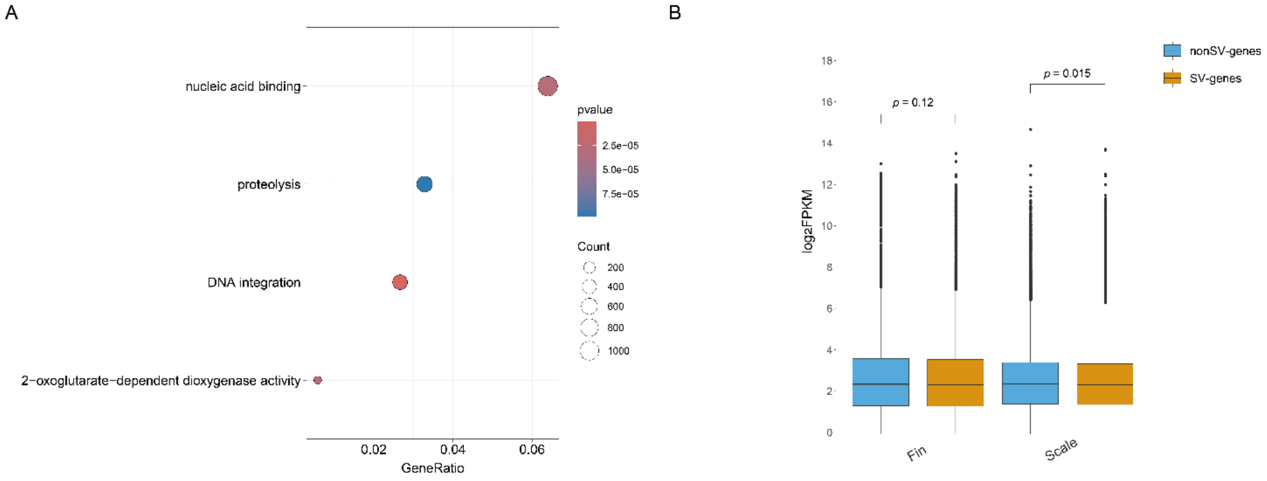

Fig. 4 | Comprehensive landscape of SVs between CC 4.0 and Songpu2021. A, GO enrichment analysis of SV-genes. The bubble size indicates the gene number of a biological process GO term, with color maps the *p*value of the enrichment analysis. GeneRatio: number of genes annotated to the GO category/total number of genes. **B,** The expression difference between genes with and without structural variants in multiple tissues. The statistical method used was the Wilcoxon test.

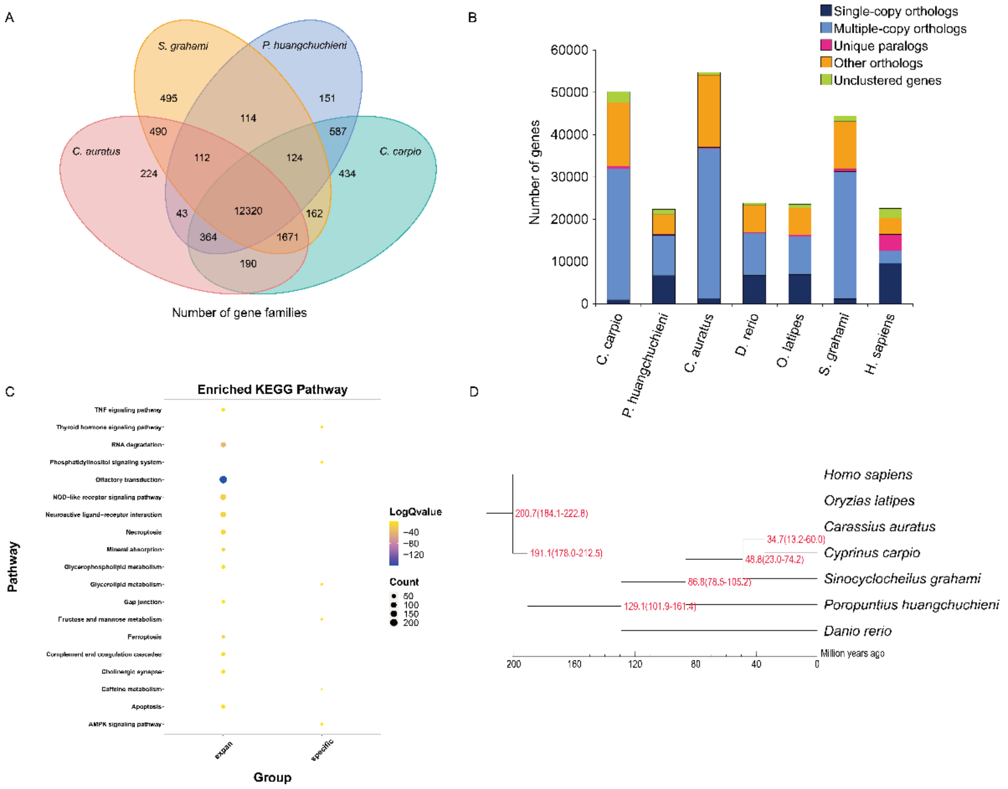

Fig. 5 **| Evolution of the koi carp (CC 4.0) genome. A,** Venn diagram of orthologous gene families in four genomes. The numbers represent quantities of gene families. **B,** Number of orthologous genes in seven species. **C,** Enrichment analysis of KEGG signaling pathway of specific genes and expansion gene families belongs to the CC 4.0 assembly. The size of the dots in the graph indicates the number of genes enriched in the pathway. The color indicates the significant Qvalue of the pathway. **D,** Phylogenetic tree constructed using conserved house-keeping proteins from seven species.

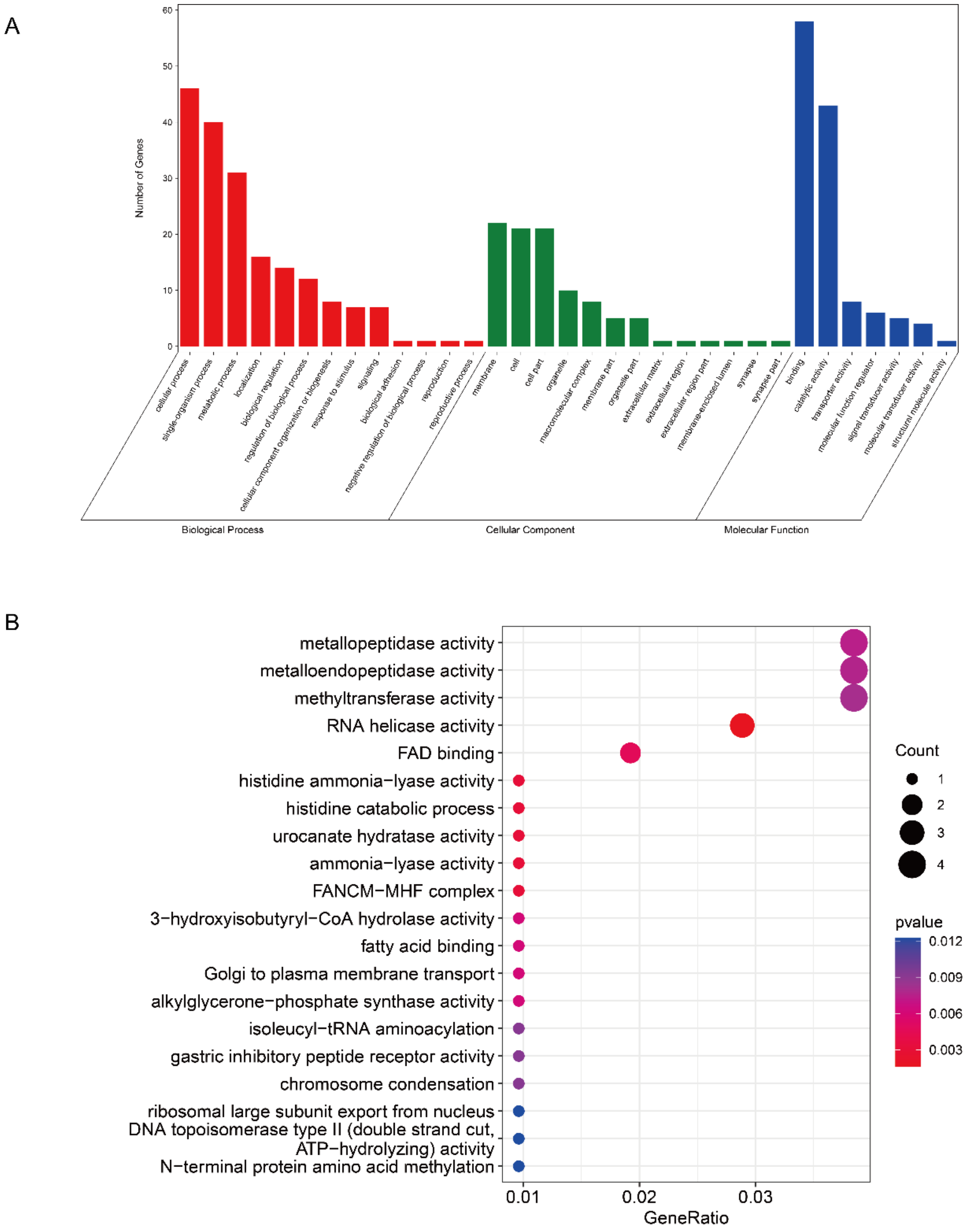

Fig. 6 | The GO function of PSGs in CC 4.0 genome. A, Web Gene Ontology Annotation Plotting plot showing GO distribution of PSGs. **B,** GO enrichment analysis of PSGs. The bubble size indicates the gene number of a biological process GO term, with color maps the *p*value of the enrichment analysis. GeneRatio: number of genes annotated to the GO category/total number of genes.

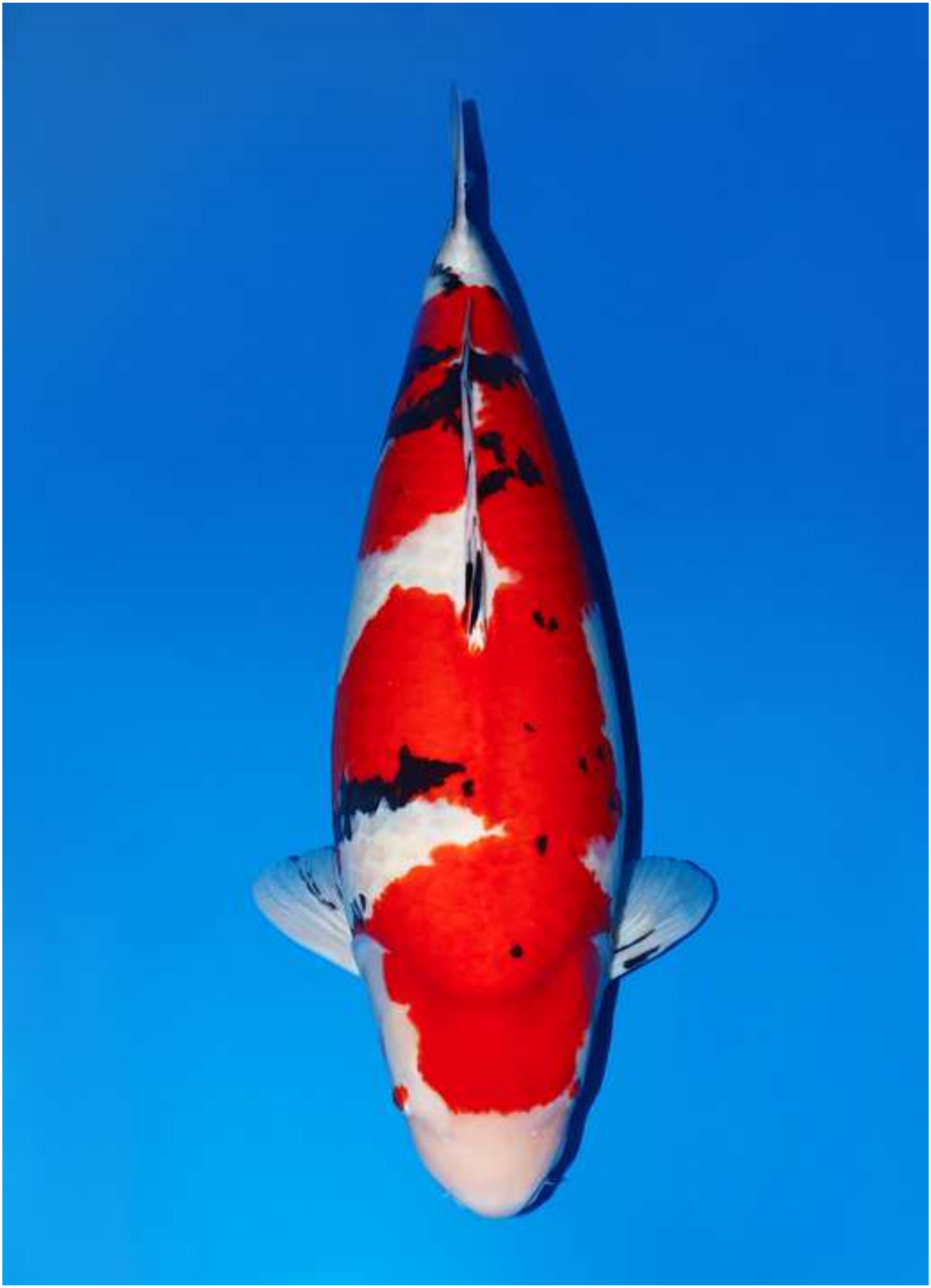

Click here to access/download;Figure;Figure 2.pdf

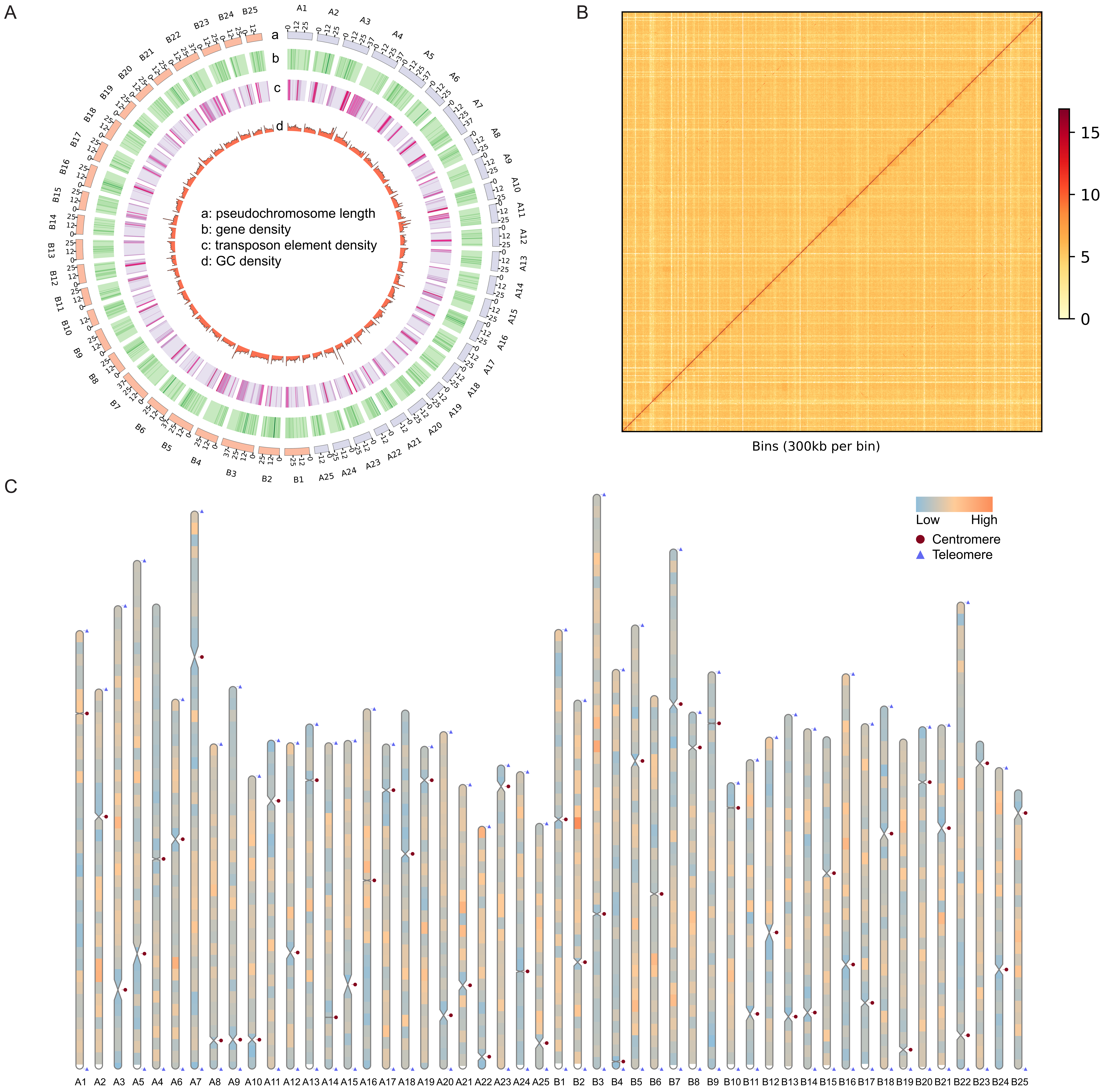

Figure_3
Click here to access/download;Figure;Figure 3.pdf

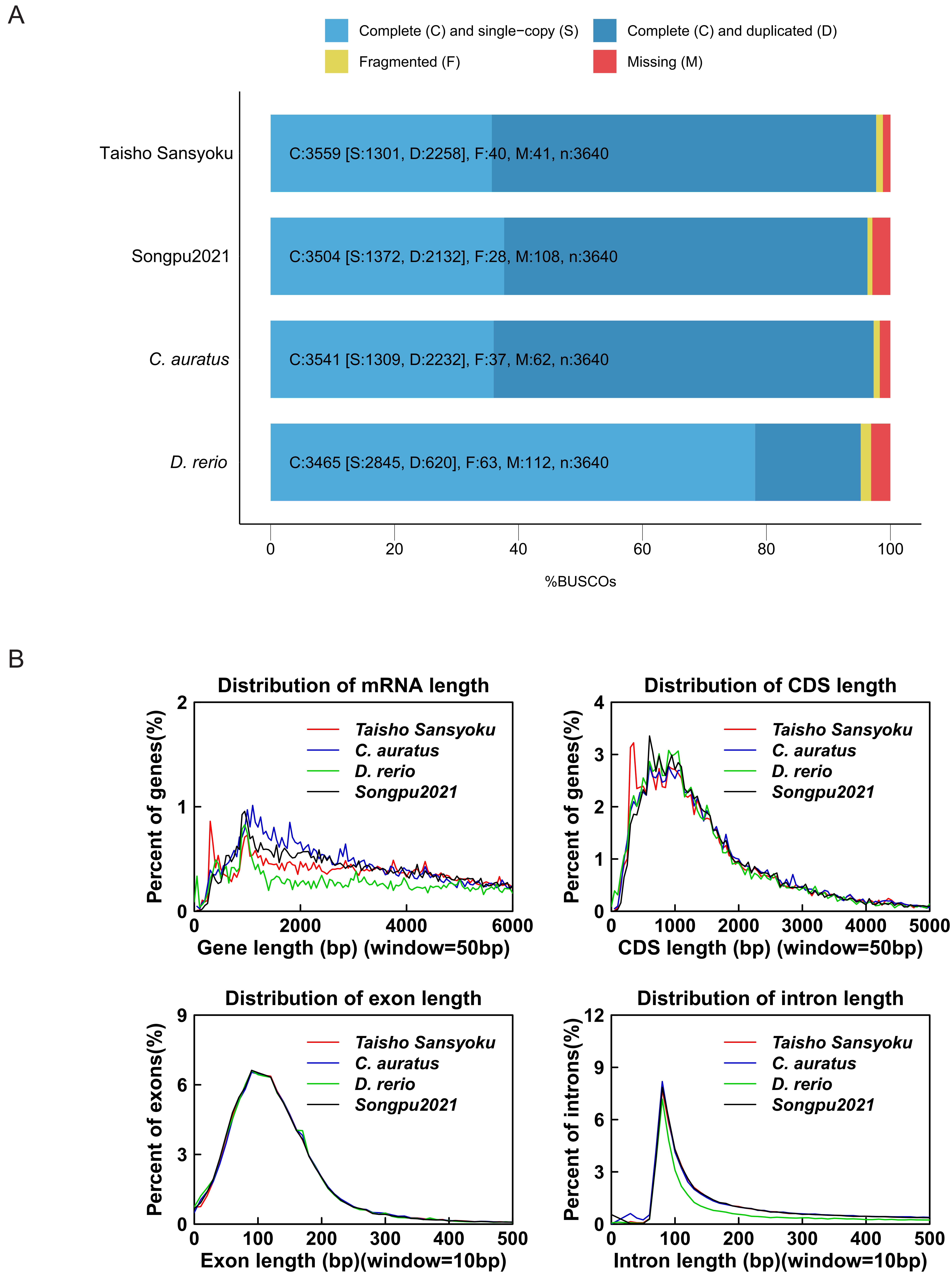

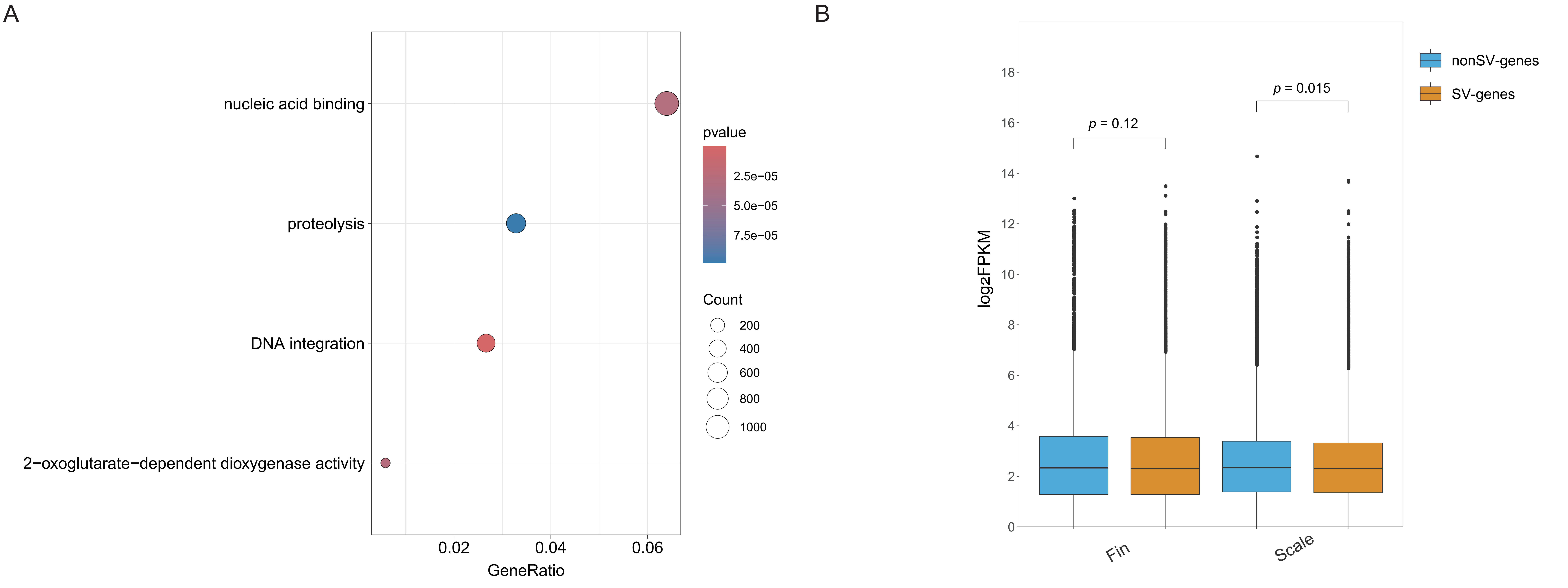
A
nucleic acid binding
proteolysis
DNA integration
2−oxoglutarate−dependent dioxygenase activity
0.02
0.04
0.06
GeneRatio
pvalue
2.5e−05
5.0e−05
7.5e−05
Count
200
400
600
800
1000
B
log2FPKM
0
2
4
6
8
10
12
14
16
18
p = 0.12
p = 0.015
Fin
Scale
nonSV-genes
SV-genes

Figure_5
Click here to access/download;Figure;Figure 5.pdf

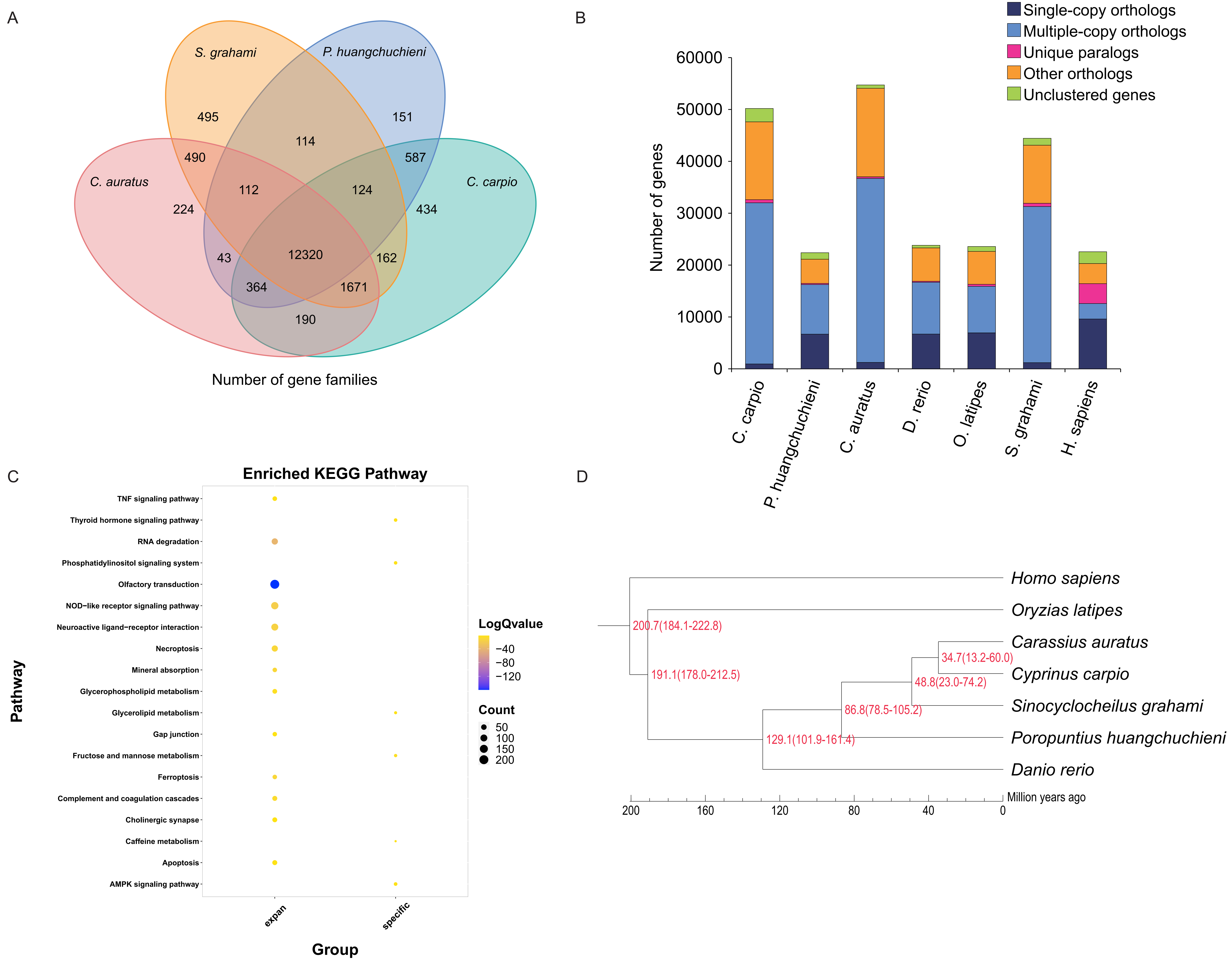

Click here to access/download;Figure;Figure 6.pdf

A

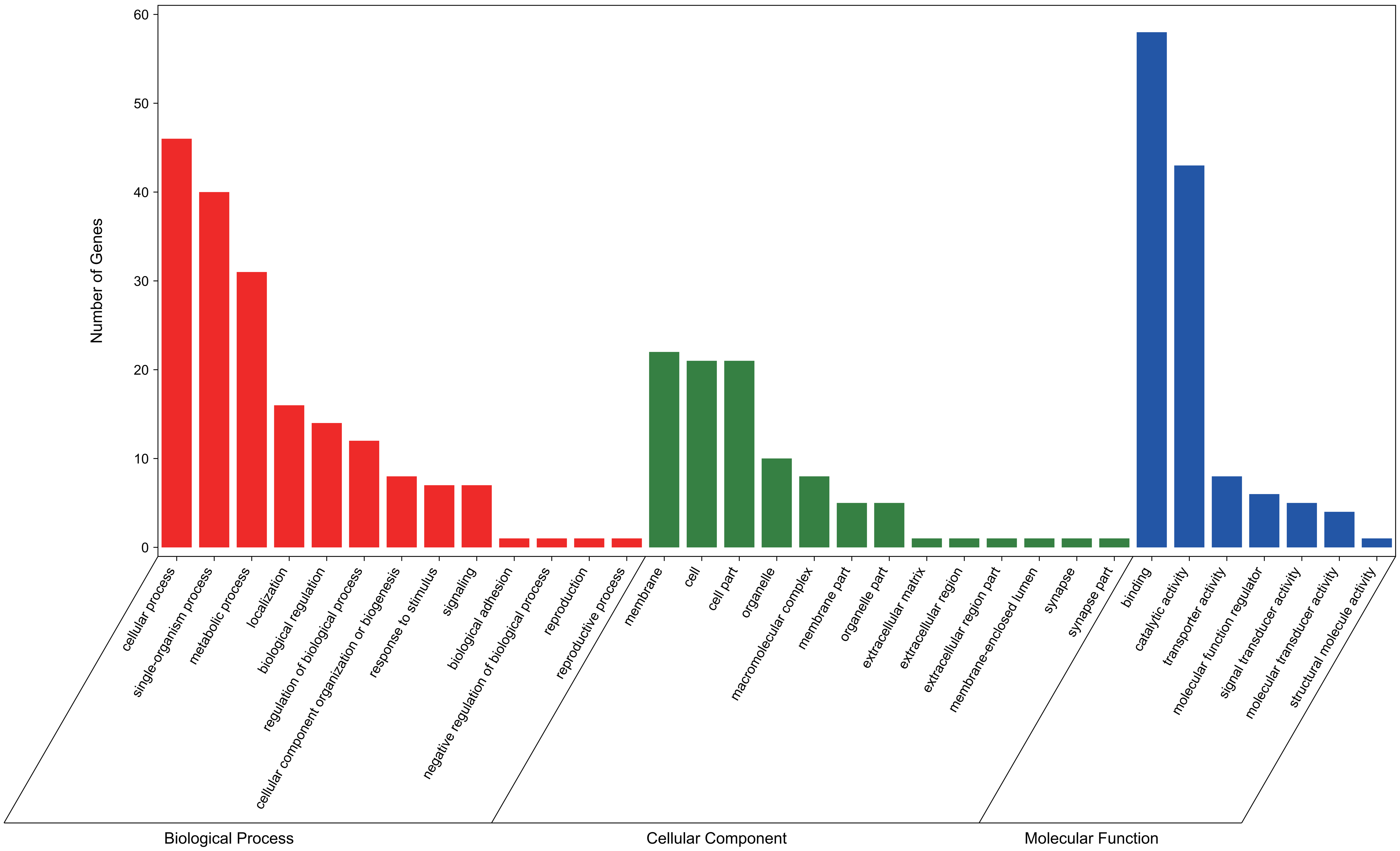

B

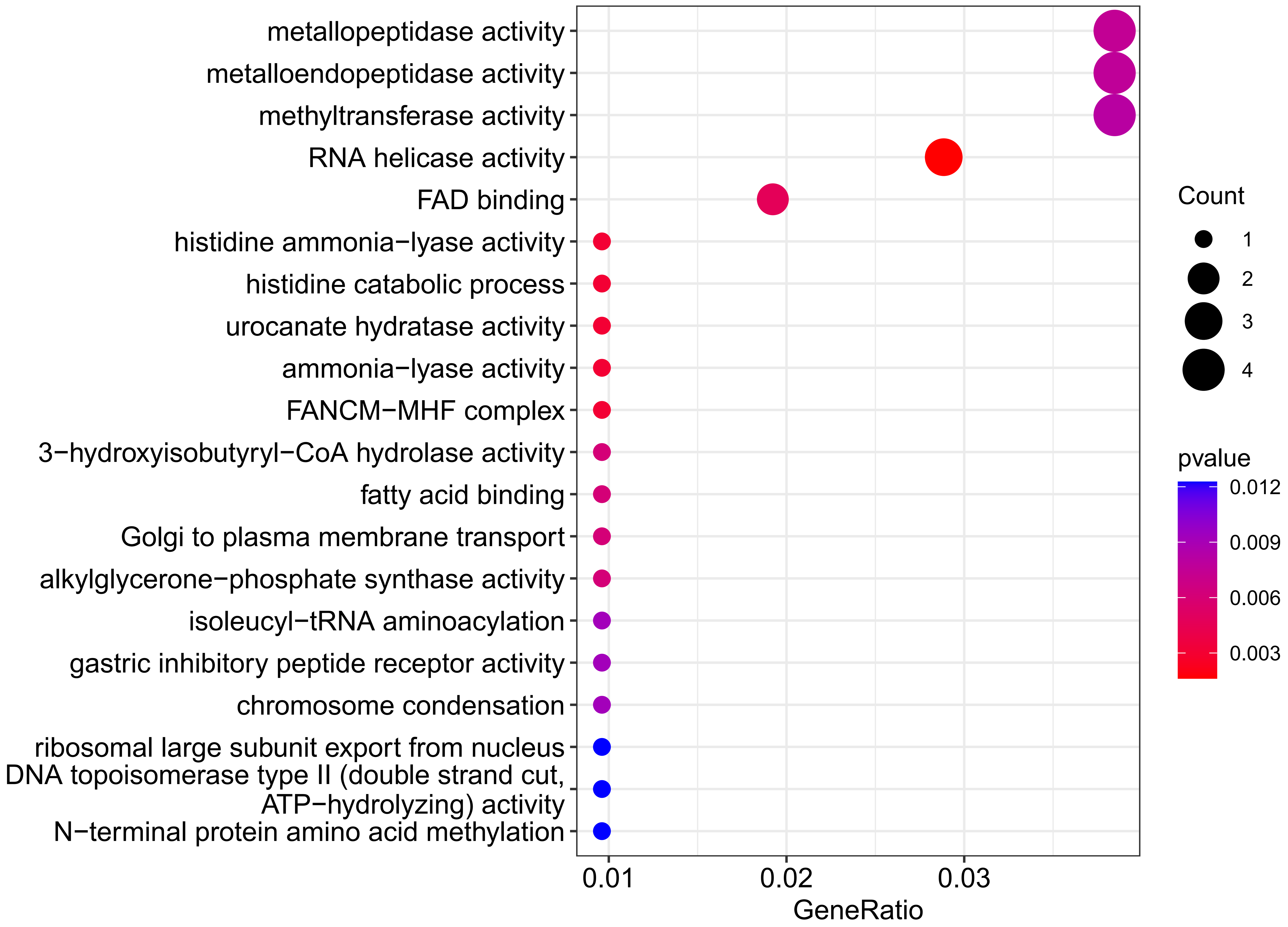

Supplementary Figures

Click here to access/download
Supplementary Material
Revised Supplementary Figure.docx

Click here to access/download
Supplementary Material
Revised Supplementary Table.xlsx

 Click here to access/download;Personal Cover;Cover Letter.docx

Dear Editor,

We sincerely thank you and the reviewers for your valuable comments and instructive advice, which help us to improve and revise our manuscript (Manuscript Number: GIGA-D-24-00530, Title: A telomere-to-telomere genome assembly of koi carp (*Cyprinus carpio*) using long reads and Hi-C technology). We have made detailed changes accordingly. Our point-by-point responses to the reviewers' comments are provided for your consideration. For ease of reviewing, all the significant changes in the revised manuscript have been highlighted in red. The list of our point-to-point responses is enclosed below with the reviewers' comments reproduced. We hope the revised manuscript can be accepted by the journal of *GigaScience*.

Thank you very much for your help!

Best regards,

Yongchao Niu
