# Supplementary material for: A telomere-to-telomere genome assembly of koi carp (Cyprinus carpio) using long reads and Hi-C technology
Source: Gigascience. 2025 Aug 29;14:giaf087. doi: 10.1093/gigascience/giaf087 (PMC12395963; doi:10.1093/gigascience/giaf087)
Supplement: giaf087_GIGA-D-24-00530_Revision_3 [file giaf087_giga-d-24-00530_revision_3.pdf]

GigaScience

A telomere-to-telomere genome assembly of koi carp (Cyprinus carpio) using long reads and Hi-C technology

--Manuscript Draft--

Manuscript Number:	GIGA-D-24-00530R3
Full Title:	A telomere-to-telomere genome assembly of koi carp (Cyprinus carpio) using long reads and Hi-C technology
Article Type:	Data Note
Funding Information:	
Abstract:	Background: The common carp (Cyprinus carpio) is a key species in global freshwater aquaculture. One of its variants, the koi carp, is particularly prized for its aesthetic appeal. However, lacking a high-quality genome has limited genetic research and breeding efforts for common carp and koi carp.<br>Findings: This study presents a gap-free genome for the Taisho Sansyoku koi carp strain (C. carpio). The assembly achieved a total size of 1555.86 Mb with a contig N50 of 30.45 Mb, comprising 50 gap-free pseudochromosomes ranging in length from 20.70 to 49.02 Mb. The BUSCO completeness score reached 99.20%, and the GCI score was 85.82, indicating high genome integrity and accuracy. Notably, 83 out of 100 telomeres were detected, resulting in 33 chromosomes possessing complete telomeres. Comparative genomic analysis showed that the expanded gene families and unique genes play essential roles in various biological traits, such as energy metabolism, endocrine regulation, cell proliferation, and immune response, potentially related to multiple metabolic diseases and health conditions. The positively selected genes are linked to various biological processes, such as the metalloendopeptidase activity, which plays a significant role in the central nervous system and is associated with diseases.<br>Conclusions: The koi carp genome assembly (CC 4.0) fills a critical gap in understanding common carp's biology and adaptation. It provides an invaluable resource for molecular-guided breeding and genetic enhancement strategies, underscoring the importance of common carp and koi carp in aquaculture and ecological research.
Corresponding Author:	Yongchao Niu, PhD<br>Biozeron Shenzhen Inc.<br>Shenzhen, CHINA
Corresponding Author Secondary Information:	
Corresponding Author's Institution:	Biozeron Shenzhen Inc.
Corresponding Author's Secondary Institution:	
First Author:	Yongchao Niu, PhD
First Author Secondary Information:	
Order of Authors:	Yongchao Niu, PhD
	Jiandong Yuan
	Jiang Li
	Jun Yong
	Xuewu Liao
	Huijuan Guo
Order of Authors Secondary Information:	
Response to Reviewers:	Reviewer reports:<br>Reviewer #1: The authors have revised their MS again, but still left several problems

Powered by Editorial Manager® and ProduXion Manager® from Aries Systems Corporation

identified earlier by this referee uncorrected. This referee is wondering, why several issues remained to be unsolved over two rounds of revision despite assurances given by the authors about the opposite. This referee has no other choice than returning the MS and asking for the corrections for the third time.
In order to help the authors, the remaining issues are now broken down to an itemized point-by-point list containing more detailed explanations regarding the tasks.
Re: Thank you for your comments and assistance. We believe that the latest updated version has addressed all the issues you mentioned.

* Using the term 'Taisho Sensyoku genome': This referee requested that the same name should be used for the koi carp genome assembly throughout the MS. The authors agreed and claimed that 'koi carp genome assembly (CC 4.0)' is now used everywhere. Yet, the second revised version still contains 'Taisho Sensyoku genome' (P4, L88) and 'BUSCO assessment of Taisho Sensyoku' (P34, L739).
Re: Revised.

* Numbers in Supplementary Tables: This referee noticed earlier that numbers in many of these tables contained decimal commas and periods as thousand indicators, whereas English language is using decimal points and commas as thousand indicators. The authors claimed that all these have been corrected. In reality, most supplementary tables (Supp. Tables S1-S11) of the second revised version still contain these errors.
Re: Thank you for your comment. We suspect that the Excel software may automatically adjusts delimiters according to the regional settings of the operating system by defult. For example: If the system region is set to "China", Excel will be used a comma as the thousands separator. If the setting is "Germany", a period or space will be used as the thousands separator, and a comma will serve as the decimal point.
To avoid this situation, in the updated version, we provide a PDF version of the supplementary tables.
Now, the thousands separator should be the comma "," and the decimal points should be the period ".".

* Aligning numbers to the decimal point in Tables and Supp. Tables: This referee requested repeatedly that numbers in many of these tables should be aligned to the decimal points, as this allows the reader to grasp their magnitude quicker. contained decimal commas and periods as thousand indicators. Although the authors claimed that this has been done, numbers in both Tables and most supplementary tables (Supp. Tables S1-S11) are still not aligned properly.
Re: The numbers were aligned to the decimal point in Tables and supplementary tables.

* Supplementary Tables S13: Please eliminate the duplication (genes genes) from the top of the last column.
Re: Revised.

* Supplementary Tables S14 and S15: Please replace 'Nr' with 'NCBI non-redundant (protein) database' on the top of the second column of both Tables.
Re: Revised.
* P21, L364: Please replace the word 'genome research on rice' with 'genome assembly of rice'.
Re: Revised.

* P22, L383: Please remove the word 'tissue' from this sentence. The revised sentence should read as follows: '…negative effects on gene expression in scales, whereas no such impacts were observed in fins…'.
Re: Revised.

* P22, L388-389: The term 'including' typically indicates an incomplete list of a larger group. Here, all the sex species are listed, therefore, the sentence should read as follows: '… species (P. huangchuchieni, C. auratus, D. rerio, O. latipes, S. grahami, and H. sapiens) were…'.
Re: Revised.

Powered by Editorial Manager® and ProduXion Manager® from Aries Systems Corporation

	* P24, L430: The term 'DNA and protein processes' is very general. The authors should replace it with more specific collective term(s).<br>Re: The sentence was revised by “Genes in centromeric regions were significantly enriched in ten GO terms, including DNA integration, nucleic acid binding, protein export from nucleus, and nuclear export signal receptor activity.”.<br><br>* List of References: This referee requested that all references on the list must be complete with unique IDs. The authors claimed that it has been done. Yet, BioRxiv references #18 and #62 do not have doi IDs, whereas #31 cannot be found with this title on arXiv.<br>Re: Thanks for your comments. #18, #31 and #62 were revised as follows:<br>18. Luo L, Wu H, Zhao L, et al. Telomere-to-telomere sheep genome assembly identifies variants associated with wool fineness. Nat Genet. 2025;57(1):218-230.<br>31. Liu, B., Shi Y., Yuan J., et al. Estimation of genomic characteristics by analyzing k mer frequency in de novo genome projects. arXiv:1308.2012v2 [q-bio.GN]. doi: https://doi.org/10.48550/arXiv.1308.2012<br>62. Holst F, Bolger A, Günther C, et al. Helixer–de novo Prediction of Primary Eukaryotic Gene Models Combining Deep Learning and a Hidden Markov Model. bioRxiv:2023.02.06.527280. doi: https://doi.org/10.1101/2023.02.06.527280
**Additional Information:**	
**Question**	**Response**
Are you submitting this manuscript to a special series or article collection?	No
**Experimental design and statistics**<br><br>Full details of the experimental design and statistical methods used should be given in the Methods section, as detailed in our Minimum Standards Reporting Checklist. Information essential to interpreting the data presented should be made available in the figure legends.<br><br>Have you included all the information requested in your manuscript?	Yes
**Resources**<br><br>A description of all resources used, including antibodies, cell lines, animals and software tools, with enough information to allow them to be uniquely identified, should be included in the Methods section. Authors are strongly encouraged to cite Research Resource Identifiers (RRIDs) for antibodies, model organisms and tools, where possible.<br><br>Have you included the information	Yes

requested as detailed in our Minimum Standards Reporting Checklist?	
**Availability of data and materials**<br><br>All datasets and code on which the conclusions of the paper rely must be either included in your submission or deposited in publicly available repositories (where available and ethically appropriate), referencing such data using a unique identifier in the references and in the “Availability of Data and Materials” section of your manuscript.<br><br>Have you have met the above requirement as detailed in our Minimum Standards Reporting Checklist?	Yes
GigaScience has policies and guidelines in place for the use of generative AI-writing tools such as ChatGPT. If you have used such writing tools to assist with writing the manuscript this must be declared and cited in the text. Authors should not list AI-writing tools and other AI-assisted technologies as an author or co-author and should acknowledge that they are fully responsible for text generated or refined by AI-writing tools.<p><br><br>A summary of use (particularly in the introduction or among methods) needs to be included at the end of the paper, and the outputs should also be included as a supplementary file hosted in GigaDB or other open repositories. Please read our guidelines for more information. <p><br><br>By submitting to GigaScience, you are aware of the journal's AI-writing tools policy, and if you have declared use of such tools below, you have acknowledged this where appropriate in your manuscript and have made a summary of use and outputs available. <p><br><b>AI-assisted writing tools have been used in the preparation of this manuscript?	No

Manuscript Click here to access/download;Manuscript;Final version manuscript 0704.docx

DATANOTE

A telomere-to-telomere genome assembly of koi carp (*Cyprinus carpio*) using long reads and Hi-C technology

Jiandong Yuan [1*†], Jiang Li [2†], Jun Yong[3†], Xuewu Liao [1], Huijuan Guo[3], Yongchao Niu [2*]

1 Suxin Koi Farm, Suzhou 215000, China

2 Biozeron Shenzhen Inc., Shenzhen 518000, China

3 Geekgene Technology Co. Ltd., Beijing 100091, China

*Correspondence address. Suxin Koi Farm, Suzhou 215000, China. E-mail: yuan@suxinkoi.com; Biozeron Shenzhen Inc. Shenzhen 518000, China. E-mail: niuyongchao@biozeron.com.

[†] These authors contributed equally to this work.

ORCID IDs: Yongchao Niu [0000-0002-9774-5417]; Jiandong Yuan [0009-0004-4214-9767]; Jiang Li [0000-0003-2099-8165]; Jun Yong [0000-0002-3770-2108]; Xuewu Liao [0009-0001-9256-5687]; Huijuan Guo [0009-0006-0440-4354]

Abstract

Background: The common carp (*Cyprinus carpio*) is a key species in global freshwater aquaculture. One of its variants, the koi carp, is particularly prized for its aesthetic appeal. However, lacking a high-quality genome has limited genetic research and breeding efforts for common carp and koi carp.

Findings: This study presents a gap-free genome for the Taisho Sansyoku koi carp strain (*C. carpio*). The assembly achieved a total size of 1555.86 Mb with a contig N50 of 30.45 Mb, comprising 50 gap-free pseudochromosomes ranging in length from 20.70 to 49.02 Mb. The

BUSCO completeness score reached 99.20%, and the GCI score was 85.82, indicating high genome integrity and accuracy. Notably, 83 out of 100 telomeres were detected, resulting in 33 chromosomes possessing complete telomeres. Comparative genomic analysis showed that the expanded gene families and unique genes play essential roles in various biological traits, such as energy metabolism, endocrine regulation, cell proliferation, and immune response, potentially related to multiple metabolic diseases and health conditions. The positively selected genes are linked to various biological processes, such as the metalloendopeptidase activity, which plays a significant role in the central nervous system and is associated with diseases.

Conclusions: The koi carp genome assembly (CC 4.0) fills a critical gap in understanding common carp's biology and adaptation. It provides an invaluable resource for molecular-guided breeding and genetic enhancement strategies, underscoring the importance of common carp and koi carp in aquaculture and ecological research.

Key words: common carp, koi carp, telomere-to-telomere, genome, positively selected gene

Data Description

Context

Common carp (*Cyprinus carpio*, NCBI: txid7962) is one of the most economically significant species, accounting for up to 10% (over 3 million metric tons) of global freshwater aquaculture production [1]. It is mainly cultured in Europe and Asia, with a cultural history of several thousand years, and has been introduced into most parts of the world. Known for being environmentally friendly, common carp are primarily omnivorous filter-feeders, requiring less fish meal and fish oil

than other aquaculture species such as salmon and shrimp [2]. In addition to serving as a food source, one of the common carp variants, koi carp, is highly prized as an ornamental fish, renowned for its vibrant colors and patterns. *C. carpio* originated from the hybridization of a Barbinae-like species and an undetermined donor species, followed by a whole genome duplication (WGD) event approximately 12.4 million years ago [3]. Their genome duplication is believed responsible for species divergence and biodiversity [4]. Common carp and goldfish (*Carassius auratus*) are evolutionarily closely related, both being allotetraploid species that have undergone WGD events. The allotetraploid nature of these species has been extensively characterized. In 2019, Chen *et al*. reported the goldfish genome's *de novo* assembly and elucidated the genes' evolutionary trajectories following the WGD [5]. Similarly, Kon *et al*. demonstrated balanced homoeolog expression and symmetric subgenomes in allotetraploid fish, thereby highlighting the crucial role of genomic plasticity in establishing allopolyploidy [6]. A landmark study on subgenomic evolution was further explored in allotetraploid fish, revealing a transition from asymmetrical to balanced genomic diversification during rediploidization [7]. As an ideal model for studying polyploid vertebrates' structural and functional adaptations, koi carp provides valuable insights into successful speciation and the evolutionary dynamics of polyploidy in animals, making it a critical species for aquaculture and ecological research. Notably, it is also regarded as an alternative vertebrate model to zebrafish.

Over the past decade, various *C. carpio* genome resources have been developed. The genome of *C. carpio* (strain Songpu) was first decoded in 2014, marking the beginning of common carp genomics research [2]. In 2019, chromosome-level reference genomes of Yellow River carp, Hebao red carp, and German Mirror carp were generated [3]. Moreover, the availability of the *Poropuntius*

huangchuchieni genome provides a diploid progenitor-like reference genome for the allotetraploid *C. carpio* [8]. In 2021, the genome of common carp var. 'Songpu' was updated (termed Songpu 2021) [9], followed by the availability of the latest *C. carpio* genome (termed CC 3.0) in 2023, obtained via Pacific Biosciences (PacBio) high-fidelity (HiFi) reads [10]. Intensive culture conditions make farmed common carp vulnerable to various pathogens, leading to high mortality rates and significant economic losses in the carp culture industry. Hence, *C. carpio* has been continuously studied regarding immunology and disease resistance [11-14]. The association between genetic variations and phenotypic diversity among common carp strains has been studied. Wang *et al*. found genetic variations related to traits like scale reduction and high growth rate and identified new candidate genes [15]. Shi *et al*. detected single-nucleotide polymorphisms (SNPs) linked to skin color variation across carp strains [16]. The *de novo* genome assembly is a fundamental and powerful tool. Currently, advances in sequencing and assembly algorithms make telomere-to-telomere (T2T) genome assembly feasible, enabling comprehensive genome identification. Some important species, such as humans [17], sheep [18, 19], rice [20], maize [21], and sorghum [22], have successively released T2T-level genomes. The considerations and methodologies for executing T2T assembly have been thoroughly summarized [23, 24]. However, the assembly of the *C. carpio* genome to a comparable level has yet to be reported. To address this gap, we integrated PacBio HiFi sequencing, Oxford Nanopore Technologies (ONT) ultralong sequencing, and chromosomal conformational capture (Hi-C) technology to assemble a high-quality gap-free genome assembly for koi carp (strain Taisho Sansyoku; Fig. 1). "Taisho Sanshoku" is a Nishikigoi strain established in the 19th century in Niigata Prefecture in Japan, which is a significant variety in the selective breeding of colored carp. This strain is characterized by its

combination of red, black, and white colors, with all three colors being intense, and black streaks on paired fins being permissible. It is an integral part of the koi carp breeding and has a specific position in the ornamental fish market. Armed with the koi carp genome assembly (CC 4.0), the characteristics of centromeric regions were investigated, and genomic evolution analyses were performed. This study on the *C. carpio* genome provides a valuable resource for the molecular-guided breeding and genetic improvement of the common carp and koi carp.

Methods

Sample collection

We collected a healthy 6-year-old female koi carp (Taisho Sansyoku) from Suzhou City, Jiangsu Province, China for DNA sequencing, RNA-seq, and Iso-seq. Genomic DNA was extracted from a muscle sample. To improve genome annotation, scale, and fin tissues were prepared for RNA-seq. In addition, RNA from eighteen tissues, including eye, tail, white scalp, red scalp, brain, black scale, white scale, red scale, heart, blood, liver, bubble, essence, spleen, bile, kidney, muscle, gill, and intestines, were equally pooled together for Iso-seq. All samples were frozen in liquid nitrogen and stored at -80°C for preservation and subsequent analysis.

Sequencing and filtering

For HiFi sequencing, SMRTbell target size libraries were constructed according to PacBio's standard protocol (Pacific Biosciences) using the 15-kb preparation solutions. The sequencing was conducted in circular consensus sequencing (CCS) mode on the PacBio Revio platform (RRID: SCR_017990) at Grandomics Biosciences (Wuhan, China). The generated subreads were

processed using SMRTLink version 8.0.0 [25] with the following parameters: "-minPasses 3 -minPredictedAccuracy 0.99 -minLength 500".

For ONT sequencing, ONT ultra-long insert libraries were obtained using the Oxford Nanopore SQK-LSK109 kit and sequenced on the PromethION (RRID: SCR_017987) platform at Grandomics Biosciences (Wuhan, China). The ONT data underwent processing using NanoFilt version 2.8.020 (RRID: SCR_016966) [26] with a quality threshold of 7.

As previously described, Hi-C libraries based on *Dpn*II restriction enzymes were prepared for Hi-C sequencing [27]. These libraries were sequenced on the MGISEQ-2000 platform, generating paired-end 150 bp reads. Clean Hi-C data were obtained using fastp version 0.19.5 (RRID: SCR_016962) [28] with parameters set as "--length_required 50 -w 8". In addition, about 1.5 μg of DNA was used to construct an approximately 350 bp insert size DNA library. According to the standard manufacturer's instructions, the quantified library was sequenced on the Illumina NovaSeq platform (Illumina, CA, USA).

The total RNA was extracted using TRIzol reagent in an RNAprep Pure Tissue Kit and processed according to the protocol provided by the manufacturer. Subsequently, the RNA purity and concentration were assessed using Nanodrop and Qubit, where only high-quality RNA samples were selected for cDNA synthesis in both bulk RNA-seq and Iso-seq experiments. For Iso-Seq, sequencing libraries were prepared using the SMRTbell Template Prep Kit 2.0 from Pacific Biosciences and sequenced on the PacBio Sequel II platform. Libraries for bulk RNA-seq were sequenced on an MGISEQ-2000 instrument, generating 150 bp paired-end reads. The libraries for bulk RNA-seq were sequenced on an MGISEQ-2000 instrument, producing 150 bp paired-end reads. The raw reads of Iso-Seq were preprocessed using SMRTLink version 8.0.0 [25]. Iso-Seq

CCS reads were derived from the subreads with specific parameters: minimum subread length = 50, maximum subread length = 15,000, minimum number of passes = 3, and minimum predicted accuracy = 0.99. The quality of RNA-seq data was examined using fastp version 0.19.5 (RRID: SCR_016962) [28] with the parameters set as "-w 8 -l 50".

Genome size estimation

To estimate the genome size and heterozygosity of the koi carp, a similar method in the study of the largemouth bass genome [29] was applied. Jellyfish version 2.1.3 (RRID: SCR_005491) [30] was used to analyze the *K*-mer depth distribution curve with a *K*-mer size of 17. The genome size was calculated using the formula G = (Ktotal - Kerror) / D, where Ktotal represents the total count of *K*-mers, Kerror is the total count of low-frequency *K*-mers (frequency ⩽ 3) likely due to sequencing errors, G is the genome size, and D is the k-mer depth [31].

Genome assembly and Hi-C scaffolding

To assemble a T2T reference genome, a combination of methods and sequencing reads was utilized. Initially, ultra-long ONT reads were processed using NextDenovo version 2.5.2 (RRID: SCR_025033) [32] for downstream gap-filling analysis. Primary contigs were generated with Hifiasm version 0.19.6 (RRID: SCR_021069) [33] using the command: "hifiasm -o Carp -t32 --ul ul.fq.gz --h1 Hi-C_clean_1.fq.gz --h2 Hi-C_clean_2.fq.gz HiFi-reads.fq.gz". Using HiFi reads, contigs were polished with NextPolish2 version 0.2.0 [34]. Hi-C clean data was aligned to the polished contigs for scaffolding using Bowtie2 version 2.2.9 (RRID: SCR_016368) [35]. Low-quality reads were eliminated using the HiC-Pro pipeline (RRID: SCR_017643) [36] with default parameters. Valid reads were utilized to anchor chromosomes with Juicer version 1.6

(RID: SCR_017226) [37] and 3d-DNA pipeline version 180419 (RRID: SCR_017227) [38]. According to the interaction signal, an additional error correction was performed with Juicebox version 2.13.07 (RRID: SCR_021172) [39]. Following a similar approach used in the goose T2T genome study [40], gaps within the assembled genome were filled using quartet_gapfiller.py from quarTeT version v1.1.1 (RRID: SCR_025258) [41], utilizing preassembled contigs generated from NextDenovo version 2.5.2 (RRID: SCR_025033) [32]. As recommended, the specific parameters used were "-f 5000 -l 1000 -i 40 -m 1000000 -t 20". In addition, we applied the LR_Gapcloser (RRID: SCR_017021) [42] program to close the remaining gaps in the assembled chromosomes, referring to the methods described in the gap-free genome of *Neosalanx taihuensis* [43]. To enhance genome quality, Winnowmap version 2.03 (RRID: SCR_025349) [44] was used to align HiFi reads to the chromosomes, followed by filtering to exclude secondary alignments and excessive clipping with the 'falconc bam-filter-clipped' tool. Finally, Racon version 1.5.0 (RRID: SCR_017642) [45] was performed for further polishing with the filtered alignments.

The completeness of the genome assembly was assessed utilizing Benchmarking Universal Single-Copy Orthologs (BUSCO) version 5.5.0 (RRID: SCR_015008) [46] with the actinopterygii_odb10 database, which includes 3,640 orthologs. The quality value (QV) was evaluated by the Merqury program version 1.3 (RRID: SCR_022964) [47] with 17-mer. Furthermore, short reads were aligned to the genome using BWA version 0.7.17-r1188 (RRID: SCR_010910) [48], while long reads from ONT and HiFi were aligned with Minimap2 version 2.24-r1122 (RRID: SCR_018550) [49]. In addition, the Genome Continuity Inspector (GCI) was assessed using GCI version 1.0 [50]. For collinearity analysis, the two genomes were compared

using MUMmer4 version 4.0.0rc1 (RRID: SCR_018171) [51] with parameters of "-t 30 -p mummer --mum -g 1000 -c 90 -l 40".

Genome annotations

Tandem Repeats Finder version 4.10 (RRID: SCR_022065) [52] was used to identify the tandem repeat elements. A combined approach of *de novo* prediction and known repeat searching was employed for interspersed repetitive sequences. RepeatModeler version 1.0.8 (RRID: SCR_015027) [53] and LTR_FINDER version 1.0.6 (RRID: SCR_015247) [54] were used to predict *de novo* repeat sequences. Subsequently, RepeatMasker version 4.0.7 (RRID: SCR_012954) [55] was applied to screen the koi carp genome against the combined *de novo* transposable element library. Additionally, RepeatMasker version 4.0.7 (RRID: SCR_012954) [55] and RepeatProteinMask version 4.0.6 were employed to search the Repbase database (RRID: SCR_021169) [56] to identify known transposable element repeats. The annotation of noncoding RNAs in the koi carp genome utilized the same method as in the largemouth bass genome study [29].

Telomeric sequences within the koi carp genome assembly were screened using the quarTeT version v1.1.1 (RRID: SCR_025258) [41] with the "-c animal" option, following a method similar to that described in the study of the near-complete sheep assembly [19]. The telomere repeat monomer identified by the TeloExplorer module in the quarTeT program was "AACCCT". Centromeres were determined using both the quarTeT version v1.1.1 (RRID: SCR_025258) [41] and Centromics methods [57]. The results from quarTeT were given priority. If the TR coverage of a chromosome's centromere was less than 40% or TE coverage was less than 80% in the quarTeT

identification results, it was considered unreliable. In such cases, the centromere region was determined using the Centromics method.

The gene prediction process employed a comprehensive strategy integrating transcriptome-based, homology-based, and *ab initio* prediction methods. Initially, RNA-seq clean reads and PacBio full-length CCS reads were assembled using Trinity version 2.11.0 (RRID: SCR_013048) [58], with the parameters '--max_memory 200G --CPU 40 --min_contig_length 200 --genome_guided_bam merged_sorted.bam --full_cleanup --min_kmer_cov 4 --min_glue 4 --bfly_opts '-V 5 --edge-thr=0.1 --stderr' --genome_guided_max_intron 10000 --long_reads ccs.fa', yielded 289,634 transcripts with a N50 size of 2,826. The assembled transcripts were then aligned to the assembly using Program to Assemble Spliced Alignment (PASA) version 2.4.1 (RRID: SCR_014656) [59], generating gene structures from valid transcript alignments (PASA-set). Additionally, RNA-seq clean reads were mapped to the assembly via Hisat2 version 2.0.1 (RRID: SCR_015530) [60]. Subsequently, Stringtie version 1.2.2 (RRID: SCR_016323) [61] and TransDecoder version 5.7.1 (RRID: SCR_017647) were employed to assemble the transcripts and identify candidate coding regions, resulting in the creation of gene models (Stringtie-set). Homologous genomes from seven assemblies, including four common carps (hebaored, germanmirror, huanghe, Songpu; ensenbl_release-111), *Carassius auratus* (ensenbl_release-111), *Danio rerio* (ensenbl_release-111), and *Poropuntius huangchuchieni* [62], were downloaded and used as queries to search against the assembly using GeMoMa version 1.9 (RRID: SCR_017646) [63]. These homology predictions were referred to as "Homology-set". For *ab initio* prediction, Helixer [64] was employed to predict coding regions in the soft-masked genome. The gene models from these three sources were subsequently merged using EvidenceModeler version 2.1.0

(RRID: SCR_014659) [65], with different weight parameters assigned to evidence from different sources (10 for PASA-set, 5 for Stringtie-set, 5 for Homology-set, and 1 for *ab initio* gene prediction). Finally, the generated gene models underwent further refinement with PASA version 2.4.1 (RRID: SCR_014656) [59] to obtain untranslated regions and alternative splicing variation information.

The integrated gene set was translated into amino-acid sequences and annotated using various databases. Diamond version 0.9.30 (RRID: SCR_009457) [66] with an E-value cutoff of 1e-05 was used to compare the protein against four public databases, including NCBI non-redundant protein sequence database, SwissProt [67], Kyoto Encyclopedia of Genes and Genomes (KEGG) [68], Translation of European Molecular Biology Laboratory. Gene ontology (GO) terms of these genes were identified using InterProScan version 5.59-91.0 (RRID: SCR_005829) [69].

Gene expression analysis was conducted following the method used in the blister beetles transcriptome [70]. Transcription factor (TF) prediction was done using AnimalTFDB version 4.0 [71].

Identification of variations

Genome alignment between the CC 4.0 genome and the Songpu2021 genome was carried out using the NUCmer program of MUMmer4 version 4.0.0rc1 (RRID: SCR_018171) [51]. The parameter settings were ‘‘--mum -g 1000 -c 90 -l 40’’. Subsequently, the delta-filter program was employed to identify alignment blocks with the parameter setting ‘‘-1 -l 1000’’. The show-snps program was utilized to detect SNPs and insertions/deletions (InDels) with the settings ‘‘-Clr -x 1 -T’’. Based on the genic regions overlapping with these variations, we annotated the identified variations using

ANNOVAR version 2020-06-07. These variations were classified into seven categories: intergenic region, intronic region, exonic region, 2 kb upstream and downstream regions, 3′ UTR, and 5′ UTR. Moreover, Assemblytics [72] was used to detect structural variants (SVs) larger than 50 bp. SVs whose positions overlapped with potential expression regulatory regions (the ±2 kb flanking regions of a gene, as analyzed in this study) or the coding sequence (CDS) of reference genes were designated as “SV-genes”, while the remaining genes were labeled as “nonSV-genes”.

Gene families and phylogenomic analysis

Protein sequences for six vertebrate animals, including *C. auratus*, *D. rerio*, *P. huangchuchieni*, *Oryzias latipes*, *Sinocyclocheilus grahami* and *Homo sapiens*, were obtained from public databases. The gene families were defined using Treefam (RRID: SCR_013401) [73]. The longest transcripts were selected for genes with alternative splicing variants to represent the genes. Blastp version 2.7.1+ (RRID: SCR_001010) [74] with an E-value cutoff of 1e-5 was utilized to identify the best-hit protein for each sequence. Hcluster_sg with the parameter “-w 10 -s 0.34” was employed to identify one-to-one orthologous proteins among the seven species under study. A total of 241 single-copy gene families across these species were aligned using Muscle version 3.8.1551(RRID: SCR_011812) [75]. Coding sequences were extracted from each single-copy gene family and concatenated to create a supergene for each species. The supergene data was then used to construct the phylogenetic tree via iqtree2 version 2.2.2.7 [76], with the parameters "-m MFP -B 1000".

The divergence time among seven species was estimated using the MCMCtree version 4.4 as implemented in the Phylogenetic Analysis of Maximum Likelihood (PAML) package

(RRID: SCR_014932) [77], with the JC69 nucleotide substitution model and an independent rates clock. Three fossil calibration times from the TimeTree database (RRID: SCR_021162) [78] were used for calibration: 1) *C. carpio – C. auratus* 10.1 - 61.0 Mya; 2) *D. rerio - O. latipes* 180.0 - 251.5 Mya ; 3) *C. carpio - P. huangchuchieni* 81.0 - 124.7 Mya. Changes in gene family size along the phylogenetic tree were analyzed by CAFE version 4.2.1 (RRID: SCR_005983) [79]. Pathway enrichment of koi carp-specific genes and genes in the expansion gene families was conducted using KOBAS version 2.0.12 (RRID: SCR_006350) [80].

Positively selected genes

We applied a similar approach as previously reported [81] to identify positively selected genes (PSGs) within the koi carp genome. In brief, the branch-site model available in the PAML package was utilized based on the phylogenetic tree. The koi carp served as the foreground branch, while *C. auratus*, *S. grahami*, *P. huangchuch,* and *D. rerio* were designated as background branches. The null model used in the branch-site test assumed that the Ka/Ks ratios for all codons across all branches were ≤1, whereas the alternative model indicated that the foreground branch contained codons evolving with Ka/Ks >1. We conducted a maximum likelihood ratio test (LRT) to evaluate these two models. The *p*-value was derived from the chi-square distribution with 1 degree of freedom (df = 1). Subsequently, *p-values* underwent adjustment for multiple comparisons using the false discovery rate (FDR) method. Genes were classified as positively selected with an FDR threshold of less than 0.05. Additionally, we required that at least one amino acid site exhibit a high probability of positive selection (Bayes probability >95%). Genes failing to satisfy this criterion at any amino acid site were designated false positives and consequently excluded from further

consideration. GO enrichment was conducted using clusterProfiler version 4.2.2 (RRID: SCR_016884) [82].

Results

T2T genome assembly and completeness evaluation

The assembly of the CC 4.0 was achieved through the integration of diverse sequencing technologies, including Illumina whole-genome short reads, PacBio HiFi, ONT ultra-long reads, and Hi-C sequencing. In total, 70.05 Gb (~43.31× coverage) of Illumina whole-genome short reads, 223.46 Gb (~138.17× coverage) of PacBio HiFi reads, 252.59 Gb of ONT ultra-long reads (~156.18× coverage), and 219.26 Gb (~135.57× coverage) of Hi-C data (Supplementary Table S1) were generated. Notably, the N50 length surpassed 15 kb for HiFi reads and 59 kb for ONT reads (Supplementary Table S1). Through *k*-mer analysis of WGS reads, the estimated CC 4.0 genome size was 1.62 Gb with a heterozygosity level of 0.45% (Supplementary Table S2 and Supplementary Fig. S1).

Independent assembly of the ONT reads using NextDenovo resulted in a total length of 2.00 Gb with an N50 length of 12.79 Mb (Supplementary Table S3). Furthermore, integrating ONT and HiFi reads via hifiasm yielded a total size of 1.58 Gb with an N50 length of 29.44 Mb, providing a more continuous assembly. The hifiasm initial assembly served as the backbone for scaffolding contigs into 50 pseudochromosomes using Hi-C data. Our results revealed that 34 pseudochromosomes were composed solely of a single contig, while 22 gaps were distributed across the remaining 16 pseudochromosomes (Supplementary Table S4 and Supplementary Fig. S2). After gap filling and polishing, the CC 4.0 genome achieved a total size of 1555.86 Mb with

an N50 of 30.45 Mb, comprising 50 gap-free pseudochromosomes ranging in length from 20.70 to 49.02 Mb (Fig. 2A; Table 1).

Multiple strategies were implemented to validate the accuracy and completeness of the CC 4.0 genome assembly. Firstly, the Hi-C heatmap demonstrated a high degree of consistency across all pseudochromosomes, confirming the precision in sequencing, ordering, and orientation of contigs (Fig. 2B). Based on collinearity analysis, the CC 4.0 genome has a syntenic relationship and good coverage with the CC 3.0 genome (Supplementary Fig. S3). Secondly, all 22 gaps were successfully closed, with both HiFi and ONT reads achieving a 100% genome alignment rate, while WGS reads demonstrated a rate of 99.79%. Thirdly, the Merqury-estimated quality value of the CC 4.0 genome was 47.95, attesting to the high accuracy of the assembly (Table 1). Furthermore, 83 out of 100 telomeres were detected, resulting in 33 T2T pseudomolecules for the entire genome (Fig. 2C and Supplementary Table S5). The GCI score for the CC 4.0 genome was 85.82, comparable to the human T2T (CHM13) genome's GCI score of 87.04, indicating that the assembly quality of the CC 4.0 genome meets the current standards for T2T assembly [50]. Lastly, the Benchmarking Universal Single-Copy Orthologs (BUSCO) evaluation revealed that the CC 4.0 genome successfully identified 99.20% of the 3,640 actinopterygii gene set (Table 1). The CC 4.0 genome completeness was higher than that of the recently reported twenty-one cyprinid genomes (average 95.60%, from 91.7 to 96.6%) and the CC 3.0 genome [10, 83]. Overall, these validations affirm the superior quality and reliability of the CC 4.0 genome assembly.

Annotation of repetitive elements and protein-coding genes

Approximately 696.41 Mb of the assembled CC 4.0 genome was classified as repetitive sequences, representing 44.76% of the genome (Table 1; Supplementary Table S6). The percentage of repetitive sequences was higher than previously reported (31.3%-43.40%) [2, 3, 10]. Most interspersed repetitive sequences consisted of DNA transposons, making up 25.94% of the genome. (Supplementary Table S7). The long terminal repeat (LTR) and long interspersed nuclear elements classes accounted for 11.25% and 11.15% of the genome, respectively (Supplementary Table S7). Additionally, 39,065 noncoding RNAs were annotated, including 4,026 microRNAs, 24,096 transfer RNAs, 3,249 small nuclear RNAs, and 7,694 ribosomal RNAs (Supplementary Table S8).

Using a combined prediction strategy, a total of 50,187 protein-coding genes were identified, with an average of 8.87 exons per gene (Table 1). BUSCO assessment demonstrated 97.77% completeness with only 1.13% missing genes, indicating robust gene annotation (Fig. 3A). The length distribution of messenger RNA, coding sequences, exons, and introns among related species supported the reliability of the annotation results (Fig. 3B). Of the predicted genes, 49,326 (98.36%) contained at least one conserved functional domain, and 36,887 (73.50%) genes showed detectable transcriptional activity (FPKM ≥ 1) (Supplementary Table S9; Supplementary Table S10). In addition, 3,918 TFs were predicted across 77 types, surpassing the count in the CC 3.0 genome (3,812) [10]. The top 10 TF families with the highest gene counts were zf-H2C2_2, Homeodomain, zf-C2H2, HLH, BTB, TF-bZIP, Forkhead, HMG_box, THAP, and Myc_DNA binding (Supplementary Fig. S4). These findings affirmed the completeness and accuracy of gene prediction in the CC 4.0 genome.

The characteristics of centromeric regions

The centromeric sequences of the 50 pseudochromosomes in the CC 4.0 genome were assembled, with an average length of 748,299 bp (Table 2). The longest centromeric region measured 1,877,250 bp on pseudochromosome A5, while the shortest measured 30,001 bp on pseudochromosome A14. Both mean and maximal lengths of koi carp centromeres were significantly shorter than those of the Yangtze finless porpoise (mean: 1,500,346 bp; maximum: 10,904,684 bp) [84]. Centromeric regions typically exhibited higher repeat sequence density and lower gene density (Fig. 2C). The average percentage of repetitive sequences in centromeric regions was 95.10%. Ninety-five genes were annotated in the centromeric regions. The genes located in the centromeric regions exhibited significant enrichment in ten GO terms, including DNA integration, nucleic acid binding, protein export from nucleus, nuclear export signal receptor activity, aspartic-type endopeptidase activity, motile cilium, nuclear-transcribed mRNA catabolic process, exonucleolytic, 3'-5', cell motility, proteolysis, and ubiquitin-protein transferase activity (Supplementary Fig. S5). In the T2T genome assembly of rice, genes in the centromere region were also enriched in the GO term of nucleic acid binding [85]. This suggests that the function of centromeres might be highly conserved among eukaryotes. In many eukaryotes, centromeres were composed of tandemly repeated DNA sequences known as satellite DNA. As previously reported, satellite repeats constitute human and macaque genomes' primary centromeric repeat class [86, 87]. Within the centromeric regions of the CC 4.0 assembly, the predominant repetitive sequence classes included satellite and simple repeats, followed by DNA transposons and LTRs (Table 2).

Genomic variations between CC 4.0 and Songpu2021

In the regions of synteny between CC 4.0 and Songpu2021, 17,822,292 SNPs and 5,555,326 InDels were identified. Most of these variants were distributed in intronic (52.50% for SNPs, 52.85% for InDels) and intergenic (35.44% for SNPs, 36.26% for InDels) regions. Conversely, only 3.42% of SNPs and 1.68% of InDels resided within exonic regions (Supplementary Table S11). High-quality genome assemblies facilitated comprehensive SV analysis. A total of 179,321 SVs with an average size of 1108.25 bp were detected. Of which, 63,568 (35.45%) resided within potential expression regulatory domains or CDS of reference genes, herein termed "SV-genes". GO enrichment analysis revealed significant over-representation of SV-genes in four functional categories: DNA integration, 2-oxoglutarate-dependent dioxygenase activity, nucleic acid binding, and proteolysis (Fig. 4A). By leveraging RNA-seq data, we found that SVs exerted negative effects on gene expression in scales, whereas no such impacts were observed in fins (Fig. 4B). These genomic variations serve as a comprehensive repository for subsequent investigations in both fundamental and applied studies of koi carp.

Phylogenetic relationship analysis

The protein-coding genes from six vertebrate species (*P. huangchuchieni*, *C. auratus*, *D. rerio*, *O. latipes*, *S. grahami*, and *H. sapiens*) were clustered into 18,442 gene families together with the protein-coding genes of the CC 4.0 genome (Supplementary Table S12; Supplementary Table S13). Among these, 12,320 gene families were shared among *P. huangchuchieni*, *C. auratus*, *S. grahami*, and *C. carpio* (Fig. 5A). Additionally, 245 gene families with 589 genes were identified as specific to common carp when compared to the other six species (Fig. 5B). Of these common carp-specific genes, 545 (92.53%) had functional annotations (Supplementary Table S14). These specific genes

were significantly enriched in six pathways: "Fructose and mannose metabolism", "Caffeine metabolism", "Phosphatidylinositol signaling system", "Thyroid hormone signaling pathway", "AMPK signaling pathway", and "Glycerolipid metabolism" (Fig. 5C).

A phylogenetic tree was constructed using 241 single-copy orthologous genes, with *H. sapiens* as the outgroup (Fig. 5D). The estimated divergence time between *C. carpio* and *C. auratus* was approximately 34.7 million years ago (MYA). Compared to the most recent common ancestor (MRCA), common carp exhibited 87 expansions and 66 contractions in gene families ($p <= 0.05$). The expanded gene families in common carp included 1,420 genes and were primarily enriched in 13 pathways, such as "Olfactory transduction", "RNA degradation", "NOD-like receptor signaling pathway", "Neuroactive ligand-receptor interaction", "Necroptosis", "Mineral absorption", "Ferroptosis", "Complement and coagulation cascades", "Glycerophospholipid metabolism", "TNF signaling pathway", "Apoptosis", "Gap junction", and "Cholinergic synapse" (Fig. 5C). Notably, the immune genes in common carp identified by genome-wide association analysis was reported to involve in several immune response-related pathways, including NOD-like receptor signaling pathway [13].

Positively selected genes

A total of 3,438 one-to-one orthologous gene sets in five teleost fish (*C. auratus*, *C. carpio*, *S. grahami*, *P. huangchuchieni*, and *D. rerio*) were analyzed for positively selected gene (PSG) detection analysis. Ultimately, 124 genes were identified as PSGs (Supplementary Table S15). These PSGs were linked to various biological processes, including binding (GO:0005488; 58 genes), cellular process (GO:0009987; 46 genes), catalytic activity (GO:0003824, 43 genes),

single-organism process (GO:0044699; 40 genes), and others (Fig. 6A). GO enrichment analysis revealed that these genes were significantly associated with enzyme activities like metallopeptidase activity, metalloendopeptidase activity, methyltransferase activity, and RNA helicase activity, and so on (Fig. 6B). The metalloendopeptidase has been reported to play a significant role in the central nervous system. It has been associated with various diseases, including breast cancer, prostate cancer, and essential hypertension [88-91].

Conclusions

Common carp's first T2T genome assembly was achieved using PacBio HiFi reads, ONT ultra-long sequencing, and Hi-C technologies, characterized by high completeness and accuracy. A total of 50 pseudochromosomes were assembled, with 33 meeting the T2T standard. All 50 centromeres in the CC 4.0 genome were predicted, with an average length of 748,299 bp, typically showing higher repeat sequence and lower gene density. Genes in centromeric regions were significantly enriched in ten GO terms including DNA integration, nucleic acid binding, protein export from nucleus, and nuclear export signal receptor activity. The assembly predicted 696.41 Mb of repetitive sequences and identified 50,187 protein-coding genes. In addition, 3,918 TFs were predicted. Comparative genomics analysis revealed 589 genes specific to koi carp. Moreover, 87 expansion and 66 contraction events were obtained. Evolutionary analysis suggested that metalloendopeptidase activity may be crucial for koi carp. 124 PSGs were identified in common carp, which were associated with various biological processes and enzyme activities, such as metallopeptidase activity. This dataset is valuable for future genetic breeding research in koi carp and common carp.

Abbreviations

BLAST: Basic Local Alignment Search Tool; BUSCO: Benchmarking Universal Single-Copy Orthologs; CCS: circular consensus sequencing; CDS: coding sequence; Gb: gigabase pairs; GCI: Genome Continuity Inspector; GO: Gene Ontology; Hi-C: High-Throughput Chromosome Conformation Capture; HiFi: High-Fidelity; InDels: insertions/deletions; Kb: kilobase pairs; KEGG: Kyoto Encyclopedia of Genes and Genomes; LINE: long interspersed nuclear element; LTR: long terminal repeat; Mb: megabase pairs; MRCA: most recent common ancestor; MYA: million years ago; NCBI: National Center for Biotechnology Information; ONT: Oxford Nanopore Technologies; PacBio: Pacific Biosciences; PASA: Program to Assemble Spliced Alignments; PSG: positively selected gene; QV: quality value; RNA-seq: RNA sequencing; SNPs: single nucleotide polymorphisms; SVs: structural variants; WGD: whole genome duplication; WGS: whole-genome sequencing; T2T: telomere-to-telomere; TRF: Tandem Repeats Finder

Tables

Table 1. Statistics for the common carp and koi carp genome assembly.

Genomic feature	CC 4.0	CC 3.0	Songpu2021
Total size (Mb)	1,555.86	1,579.38	1,531.01
Number of chromosomes	50	50	50
Gap number	0	1,089	22,301
Chromosome N50 (Mb)	30.45	28.32	30.48
GC content (%)	37.20	37.20	37.00
Protein-coding genes number	50,187	55,981	41,939
Repetitive sequences (%)	44.76	43.40	40.09
Genome BUSCOs (%)	99.20	99.10	98.96
GCI score	85.82	NA	NA
WGS reads mapping rate (%)	99.79	NA	NA
ONT reads mapping rate (%)	100.00	NA	NA
HiFi reads mapping rate (%)	100.00	NA	NA
Quality value	47.95	NA	NA

Note: The CC 3.0 genome was downloaded from the Genome Warehouse database under accession GWHBHRW00000000. The Songpu2021 genome was retrieved from the NCBI database under the accession number GCA_018340385.1. NA means not available.

Table 2. The characteristics of centromeric regions of the koi carp CC 4.0 assembly.

Chr	Start	End	Length	Gene number	Total repeats (%)	Tandem repeats (%)	Satellite (%)	Simple repeats (%)	DNA transposons (%)	LTR (%)
A1	6,983,780	7,145,651	161,872	2	81.31	60.02	0.00	0.00	13.97	54.67
A2	10,552,693	11,217,077	664,385	0	99.80	97.66	34.62	81.27	0.17	10.08
A3	31,879,419	33,636,405	1,756,987	15	94.48	47.82	0.52	0.01	23.15	19.92
A4	21,620,000	21,850,000	230,001	0	99.51	91.05	38.95	68.48	0.20	19.43
A5	32,599,339	34,476,588	1,877,250	4	95.23	78.50	37.86	38.32	19.57	18.10
A6	11,508,992	12,370,832	861,841	0	97.97	93.41	32.03	76.06	1.69	18.41
A7	11,516,427	13,371,720	1,855,294	5	97.97	92.18	32.58	34.23	4.61	12.75
A8	24,955,543	25,632,915	677,373	2	98.40	92.83	10.21	81.02	1.14	5.10
A9	29,694,335	30,547,340	853,006	4	90.68	74.76	73.54	0.00	9.05	6.00
A10	22,177,277	22,829,639	652,363	0	99.55	77.31	32.70	84.61	0.16	5.35
A11	4,746,905	5,590,589	843,685	0	96.67	69.23	69.71	29.75	0.32	0.52
A12	17,378,483	18,406,913	1,028,431	2	95.07	84.22	46.46	50.69	8.00	3.01
A13	4,680,000	4,900,000	220,001	0	90.67	89.23	78.71	11.37	0.00	0.13
A14	23,400,000	23,430,000	30,001	0	94.82	91.60	63.58	0.00	1.36	0.00
A15	19,963,423	21,668,624	1,705,202	2	95.22	82.73	42.88	51.19	8.10	5.78
A16	14,555,504	14,717,133	161,630	1	88.13	21.87	0.00	0.00	86.60	1.64
A17	3,716,890	4,150,698	433,809	3	99.97	62.40	1.99	0.00	0.26	29.53
A18	11,933,667	12,590,459	656,793	2	95.02	89.14	27.61	78.28	2.76	9.08
A19	2,597,145	3,177,452	580,308	2	96.10	94.22	21.03	87.82	1.42	6.54
A20	23,703,082	24,668,971	965,890	5	88.97	69.01	66.65	0.01	8.92	14.15
A21	16,706,975	17,545,549	838,575	1	95.78	88.81	16.58	82.56	3.99	8.95
A22	19,320,532	19,979,681	659,150	0	98.32	96.21	39.26	79.33	0.77	10.22
A23	1,348,620	2,293,930	945,311	1	100.00	98.81	57.69	52.65	0.00	17.76
A24	16,939,420	17,124,316	184,897	4	88.30	7.58	0.00	0.00	48.20	42.52
A25	18,285,858	19,188,655	902,798	0	97.68	91.44	69.84	24.54	0.86	7.48
B1	15,950,000	16,440,000	490,001	0	99.88	95.39	17.92	53.81	0.00	0.00

B2	22,023,874	22,670,773	646,900	6	90.32	72.71	73.80	0.00	11.34	5.13
B3	35,614,767	35,952,947	338,181	0	97.14	91.61	88.11	17.69	5.52	1.09
B4	33,360,418	33,527,013	166,596	0	99.39	97.34	0.00	0.00	0.33	0.00
B5	11,034,427	12,148,201	1,113,775	1	94.60	83.31	72.53	9.66	7.56	6.22
B6	16,740,439	17,090,269	349,831	1	96.16	93.15	0.00	0.00	1.17	15.35
B7	12,778,416	13,657,366	878,951	1	99.24	71.75	29.25	74.78	0.70	14.21
B8	2,758,449	3,284,545	526,097	2	93.21	86.27	55.03	49.18	2.79	14.59
B9	4,331,995	4,452,008	120,014	0	75.44	42.01	0.00	0.00	30.85	36.93
B10	2,079,927	2,213,557	133,631	0	95.15	78.41	0.54	50.58	5.73	50.44
B11	21,120,000	22,240,000	1,120,001	0	99.59	99.48	38.50	81.92	0.09	3.06
B12	15,879,559	17,438,428	1,558,870	6	95.57	85.19	39.74	67.93	8.58	9.30
B13	25,361,200	26,195,054	833,855	4	86.95	56.30	50.37	11.58	18.10	12.22
B14	23,790,000	24,620,000	830,001	1	94.93	12.26	13.52	79.35	0.76	18.71
B15	11,292,907	11,974,689	681,783	3	97.13	2.80	11.85	0.00	12.58	1.83
B16	24,380,000	25,210,000	830,001	1	99.77	98.92	38.71	84.91	0.19	7.52
B17	23,480,000	24,130,000	650,001	1	96.47	77.13	62.70	43.82	0.18	9.51
B18	10,339,546	11,415,040	1,075,495	8	89.51	59.19	44.49	0.59	24.08	17.50
B19	26,221,041	26,766,840	545,800	1	98.22	90.31	72.13	30.39	2.66	5.52
B20	4,550,000	4,900,000	350,001	0	98.63	98.56	27.54	95.27	0.13	0.00
B21	8,310,000	9,300,000	990,001	0	97.21	2.16	50.79	74.63	0.00	7.16
B22	36,566,575	37,323,917	757,343	1	97.09	92.48	23.27	67.10	5.81	22.69
B23	1,540,000	2,230,000	690,001	0	93.82	0.15	21.82	81.62	0.26	7.16
B24	16,763,174	17,679,191	916,018	3	94.99	84.22	37.99	54.93	10.27	7.39
B25	1,437,545	2,512,481	1,074,937	0	98.86	96.59	39.80	76.90	2.90	1.04

Note: Some repeat elements may partly include another element domain. LTR, long terminal repeat.

Additional Files

Supplementary Table S1. Summary of the data sequenced by multiple technologies.

Supplementary Table S2. *K*-mer analysis.

Supplementary Table S3. The statistics of the initial assembly.

Supplementary Table S4. The statistics of the anchored chromosome length.

Supplementary Table S5. The identified telomeres in CC 4.0 assembly.

Supplementary Table S6. General statistics of repeats in CC 4.0 assembly.

Supplementary Table S7. The summary of interspersed repeat contents in CC 4.0 assembly.

Supplementary Table S8. Non-coding RNAs in CC 4.0 assembly.

Supplementary Table S9. Summary of gene function annotation.

Supplementary Table S10. The gene expression matrix.

Supplementary Table S11. The categories of SNPs and InDels with CC 4.0 as reference.

Supplementary Table S12. The data sources of six vertebrate genomes.

Supplementary Table S13. Statistics for the orthologous gene families of seven species genomes.

Supplementary Table S14. The list of koi carp-specific genes.

Supplementary Table S15. The list of 124 positive selection genes.

Author Contributions

Jiandong Yuan, Jun Yong, and Yongchao Niu designed this study; Jun Yong, Huijuan Guo, and Xuewu Liao collected the samples and performed the experiments; Jiang Li and Yongchao Niu performed the data analysis; Jiandong Yuan, Jiang Li, and Yongchao Niu wrote the first draft of

the manuscript. All other authors proofread and revised the manuscript. All authors read and approved the final manuscript.

Funding

This study was supported by Suxin Koi Farm.

Data Availability

The genomic and transcriptomic sequence data generated in this study are available under the BioProject accession: PRJNA1268753. The raw sequencing data that support this study's findings also have been deposited into the CNGB Sequence Archive (CNSA) of China National GeneBank DataBase (CNGBdb) with accession number CNP0006400. All additional supporting data are available in the GigaScience repository, GigaDB [92].

Competing Interests

The authors declare that they have no competing interests.

Acknowledgements

We thank every project that provides funding and material support for the study. We also thank each author for their ideas and skills in study design, experimentation, data collection, data analysis, and manuscript writing.

Ethics statement

This study was carried out according to the recommendations for the care and use of animals for scientific purposes set up by the Animal Care and Use Committee of the Chinese Academy of Fishery Sciences (ACUC-CAFS).

References

1. Bostock J, McAndrew B, Richards R, et al. Aquaculture: global status and trends. Philos Trans R Soc Lond B Biol Sci 2010;365(1554): 2897-2912.

2. Xu P, Zhang X, Wang X, et al. Genome sequence and genetic diversity of the common carp, Cyprinus carpio. Nat Genet 2014;46(11):1212-9.

3. Xu P, Xu J, Liu G, et al. The allotetraploid origin and asymmetrical genome evolution of the common carp Cyprinus carpio. Nat Commun 2019;10(1):4625.

4. Ren R, Wang H, Guo C, Zhang N, et al. Widespread Whole Genome Duplications Contribute to Genome Complexity and Species Diversity in Angiosperms. Mol Plant. 2018;11(3):414-428.

5. Chen Z, Omori Y, Koren S, et al. De novo assembly of the goldfish (Carassius auratus) genome and the evolution of genes after whole-genome duplication. Sci Adv. 2019;5(6):eaav0547.

6. Kon T, Omori Y, Fukuta K, Wada H, et al. The Genetic Basis of Morphological Diversity in Domesticated Goldfish. Curr Biol. 2020;30(12):2260-2274.e6.

7. Luo J, Chai J, Wen Y, et al. From asymmetrical to balanced genomic diversification during rediploidization: Subgenomic evolution in allotetraploid fish. Sci Adv. 2020;6(22):eaaz7677.

8. Chen L, Li B, Chen B, et al. Chromosome-level genome of Poropuntius huangchuchieni provides a diploid progenitor-like reference genome for the allotetraploid Cyprinus carpio. Mol Ecol Resour. 2021;21(5):1658-1669.

9. Li JT, Wang Q, Huang Yang MD, et al. Parallel subgenome structure and divergent expression evolution of allo-

tetraploid common carp and goldfish. Nat Genet. 2021;53(10):1493-1503.

10. Chen L, Li C, Li B, et al. Evolutionary divergence of subgenomes in common carp provides insights into speciation and allopolyploid success. Fundam Res. 2023;4(3):589-602.

11. Zhang Y, Stupka E, Henkel CV, Jansen HJ, et al. Identification of common carp innate immune genes with whole-genome sequencing and RNA-Seq data. J Integr Bioinform. 2011;8(2):169.

12. Verma DK, Peruzza L, Trusch F, et al. Transcriptome analysis reveals immune pathways underlying resistance in the common carp Cyprinus carpio against the oomycete Aphanomyces invadans. Genomics. 2021;113(1 Pt 2):944-956.

13. Jiang Y, Yu M, Dong C, et al. Genomic features of common carp that are relevant for resistance against Aeromonas hydrophila infection. Aquaculture. 2022; 547:737512.

14. Wang J, Zhou Q, Jiang Y. Genome-wide analysis of common carp (Cyprinus carpio) mucin genes and their roles in mucosal immune response following the Aeromonas hydrophila infection. Comparative Immunology Reports. 2024; 7:200167.

15. Wang M, Li X, Wang C, et al. Asymmetric and parallel subgenome selection co-shape common carp domestication. BMC Biol. 2024;22(1):4.

16. Shi X, Zhu W, Guo J, et al. Genome-wide association study reveals candidate genes critical for skin pigmentation in common carp (Cyprinus carpio) strains including koi. Aquaculture. 2024; 590:741075.

17. Nurk S, Koren S, Rhie A, et al. The complete sequence of a human genome. Science. 2022; 376(6588):44-53.

18. Luo L, Wu H, Zhao L, et al. Telomere-to-telomere sheep genome assembly identifies variants associated with wool fineness. Nat Genet. 2025;57(1):218-230.

19. You X, Fang Q, Chen C, et al. Author Correction: A near complete genome assembly of the East Friesian sheep genome. Sci Data. 2024;11(1):947.

20. Shang L, He W, Wang T, et al., A complete assembly of the rice Nipponbare reference genome. Mol Plant. 2023;16(8):1232-1236.

21. Chen J, Wang Z, Tan K, et al. A complete telomere-to-telomere assembly of the maize genome. Nat Genet. 2023;55(7):1221-1231.

22. Li M, Chen C, Wang H, et al. Telomere-to-telomere genome assembly of sorghum. Sci Data. 2024;11(1):835.

23. Li H, Durbin R. Genome assembly in the telomere-to-telomere era. Nat Rev Genet. 2024;25(9):658-670.

24. Garg V, Bohra A, Mascher M, et al. Unlocking plant genetics with telomere-to-telomere genome assemblies. Nat Genet. 2024;56(9):1788-1799.

25. Chin CS, Alexander DH, Marks P, et al. Nonhybrid, finished microbial genome assemblies from long-read SMRT

sequencing data. Nat Methods. 2013;10(6):563-9.

26. De Coster W, D'Hert S, Schultz DT, et al. NanoPack: visualizing and processing long-read sequencing data. Bioinformatics. 2018;34(15):2666-2669.

27. Belton JM, McCord RP, Gibcus JH, et al. Hi-C: a comprehensive technique to capture the conformation of genomes. Methods. 2012;58(3):268-76.

28. Chen S, Zhou Y, Chen Y, et al. fastp: an ultra-fast all-in-one FASTQ preprocessor. Bioinformatics. 2018;34(17):i884-i890.

29. Sun C, Li J, Dong J, Niu Y, et al. Chromosome-level genome assembly for the largemouth bass Micropterus salmoides provides insights into adaptation to fresh and brackish water. Mol Ecol Resour. 2021;21(1):301-315.

30. Marçais G, Kingsford C. A fast, lock-free approach for efficient parallel counting of occurrences of k-mers. Bioinformatics. 2011;27(6):764-70.

31. Liu, B., Shi Y., Yuan J., et al. Estimation of genomic characteristics by analyzing k mer frequency in de novo genome projects. arXiv:1308.2012v2 [q-bio.GN]. doi: https://doi.org/10.48550/arXiv.1308.2012

32. Hu J, Wang Z, Sun Z, et al. NextDenovo: an efficient error correction and accurate assembly tool for noisy long reads. Genome Biol. 2024;25(1):107.

33. Cheng H, Concepcion GT, Feng X, et al. Haplotype-resolved de novo assembly using phased assembly graphs with hifiasm. Nat Methods. 2021;18(2):170-175.

34. Hu J, Wang Z, Liang F, et al. NextPolish2: A Repeat-aware Polishing Tool for Genomes Assembled Using HiFi Long Reads. Genomics Proteomics Bioinformatics. 2024;22(1):qzad009.

35. Langmead B, Salzberg SL. Fast gapped-read alignment with Bowtie 2. Nat Methods. 2012;9(4):357-9.

36. Servant N, Varoquaux N, Lajoie BR, et al. HiC-Pro: An optimized and flexible pipeline for Hi-C data processing. Genome Biol. 2015;16:259.

37. Durand NC, Shamim MS, Machol I, et al. Juicer Provides a One-Click System for Analyzing Loop-Resolution Hi-C Experiments. Cell Syst. 2016 Jul;3(1):95-8.

38. Dudchenko O, Batra SS, Omer AD, et al. De novo assembly of the Aedes aegypti genome using Hi-C yields chromosome-length scaffolds. Science. 2017;356(6333):92-95.

39. Durand NC, Robinson JT, Shamim MS, et al. Juicebox Provides a Visualization System for Hi-C Contact Maps with Unlimited Zoom. Cell Syst. 2016 Jul;3(1):99-101.

40. Zhao H, Zhou H, Sun G, et al. Telomere-to-telomere genome assembly of the goose Anser cygnoides. Sci Data. 2024;11(1):741.

41. Lin Y, Ye C, Li X, et al. quarTeT: a telomere-to-telomere toolkit for gap-free genome assembly and centromeric repeat identification. Hortic Res. 2023;10(8):uhad12.

42. Xu GC, Xu TJ, Zhu R, et al. LR_Gapcloser: a tiling path-based gap closer that uses long reads to complete genome assembly. Gigascience. 2019;8(1):giy157.

43. Zhou Y, Zhang X, Jian J, et al. Gap-free genome assembly of Salangid icefish Neosalanx taihuensis. Sci Data. 2023;10(1):768.

44. J Jain C, Rhie A, Hansen NF, et al. Long-read mapping to repetitive reference sequences using Winnowmap2. Nat Methods. 2022;19(6):705-710.

45. Vaser R, Sović I, Nagarajan N, et al., Fast and accurate de novo genome assembly from long uncorrected reads. Genome Res. 2017;27(5):737-746.

46. Seppey M, Manni M, Zdobnov EM. BUSCO: Assessing Genome Assembly and Annotation Completeness. Methods Mol Biol. 2019;1962:227-245.

47. Rhie A, Walenz BP, Koren S, et al. Merqury: reference-free quality, completeness, and phasing assessment for genome assemblies. Genome Biol. 2020;21(1):245.

48. Li H, Durbin R. Fast and accurate short read alignment with Burrows-Wheeler transform. Bioinformatics. 2009;25(14):1754-60.

49. Li H. Minimap2: pairwise alignment for nucleotide sequences. Bioinformatics. 2018;34(18):3094-3100.

50. Chen Q, Yang C, Zhang G, et al. GCI: a continuity inspector for complete genome assembly. 2024;40(11):btae633.

51. Marçais G, Delcher AL, Phillippy AM, et al. MUMmer4: A fast and versatile genome alignment system. PLoS Comput Biol. 2018;14(1):e1005944.

52. Benson G. Tandem repeats finder: a program to analyze DNA sequences. Nucleic Acids Res. 1999;27(2):573-80.

53. Flynn JM, Hubley R, Goubert C, et al. RepeatModeler2 for automated genomic discovery of transposable element families. Proc Natl Acad Sci U S A. 2020;117(17):9451-9457.

54. Xu Z, Wang H. LTR_FINDER: an efficient tool for the prediction of full-length LTR retrotransposons. Nucleic Acids Res. 2007;35(Web Server issue):W265-8.

55. Tarailo-Graovac M, Chen N. Using RepeatMasker to identify repetitive elements in genomic sequences. Curr Protoc Bioinformatics. 2009;Chapter 4:4.10.1-4.10.14.

56. Bao W, Kojima KK, Kohany O. Repbase Update, a database of repetitive elements in eukaryotic genomes. Mob DNA. 2015;6:11.

57. Centromics. https://github.com/ShuaiNIEgithub/Centromics. Accessed 3 Jul 2025.

58. Grabherr MG, Haas BJ, Yassour M, et al. Full-length transcriptome assembly from RNA-Seq data without a reference genome. Nat Biotechnol. 2011;29(7):644-52.

59. Haas BJ, Delcher AL, Mount SM, et al. Improving the Arabidopsis genome annotation using maximal transcript alignment assemblies. Nucleic Acids Res. 2003;31(19):5654-66.

60. Kim D, Langmead B, Salzberg SL. HISAT: a fast spliced aligner with low memory requirements. Nat Methods. 2015;12(4):357-60.

61. Kovaka S, Zimin AV, Pertea GM, et al. Transcriptome assembly from long-read RNA-seq alignments with StringTie2. Genome Biol. 2019;20(1):278.

62. GWHAOPL00000000. https://ngdc.cncb.ac.cn/gwh/Assembly/10299/show. Accessed 3 Jul 2025.

63. Keilwagen J, Hartung F, Grau J. GeMoMa: Homology-Based Gene Prediction Utilizing Intron Position Conservation and RNA-seq Data. Methods Mol Biol. 2019;1962:161-177.

64. Holst F, Bolger A, Günther C, et al. Helixer–de novo Prediction of Primary Eukaryotic Gene Models Combining Deep Learning and a Hidden Markov Model. bioRxiv:2023.02.06.527280. doi: https://doi.org/10.1101/2023.02.06.527280

65. Haas BJ, Salzberg SL, Zhu W, et al. Automated eukaryotic gene structure annotation using EVidenceModeler and the Program to Assemble Spliced Alignments. Genome Biol. 2008;9(1):R7.

66. Buchfink B, Xie C, Huson DH. Fast and sensitive protein alignment using DIAMOND. Nat Methods. 2015;12(1):59-60.

67. Bairoch A, Apweiler R. The SWISS-PROT protein sequence data bank and its supplement TrEMBL in 1999. Nucleic Acids Res. 1999;27(1):49-54.

68. Kanehisa M, Goto S. KEGG: kyoto encyclopedia of genes and genomes. Nucleic Acids Res. 2000;28(1):27-30.

69. Jones P, Binns D, Chang HY, et al. InterProScan 5: genome-scale protein function classification. Bioinformatics. 2014;30(9):1236-40.

70. Wu YM, Li JR, Li J, Guo T. Investigation of sex expression profiles and the cantharidin biosynthesis genes in two blister beetles. PLoS One. 2023;18(8):e0290245.

71. Shen WK, Chen SY, Gan ZQ, et al. AnimalTFDB 4.0: a comprehensive animal transcription factor database updated with variation and expression annotations. Nucleic Acids Res. 2023;51(D1):D39-D45.

72. Assemblytics. http://assemblytics.com. Accessed 3 Jul 2025.

73. Li H, Coghlan A, Ruan J, et al. TreeFam: a curated database of phylogenetic trees of animal gene families. Nucleic Acids Res. 2006;34(Database issue):D572-80.

74. Altschul SF, Gish W, Miller W, et al. Basic local alignment search tool. J J Mol Biol. 1990;215(3):403-10.

75. Edgar RC. Muscle5: High-accuracy alignment ensembles enable unbiased assessments of sequence homology and phylogeny. Nat Commun. 2022;13(1):6968.

76. Minh BQ, Schmidt HA, Chernomor O, Schrempf D, et al. IQ-TREE 2: New Models and Efficient Methods for Phylogenetic Inference in the Genomic Era. Mol Biol Evol. 2020;37(5):1530-1534.

77. Yang Z. PAML: a program package for phylogenetic analysis by maximum likelihood. Comput Appl Biosci. 1997;13(5):555-6.

78. Hedges SB, Dudley J, Kumar S. TimeTree: a public knowledge-base of divergence times among organisms. Bioinformatics. 2006;22(23):2971-2.

79. De Bie T, Cristianini N, Demuth JP, et al. CAFE: a computational tool for the study of gene family evolution. Bioinformatics. 2006;22(10):1269-71.

80. Xie C, Mao X, Huang J, Ding Y, et al. KOBAS 2.0: a web server for annotation and identification of enriched pathways and diseases. Nucleic Acids Res. 2011;39(Web Server issue):W316-22.

81. Wang Y, Zhang X, Wang J, et al. Genomic insights into the seawater adaptation in Cyprinidae. BMC Biol. 2024;22(1):87.

82 Wu T, Hu E, Xu S, Chen M, et al. clusterProfiler 4.0: A universal enrichment tool for interpreting omics data. Innovation (Camb). 2021;2(3):100141.

83. Xu MR, Liao ZY, Brock JR, et al. Maternal dominance contributes to subgenome differentiation in allopolyploid fishes. Nat Commun. 2023;14(1):8357.

84. Yin D, Chen C, Lin D, et al. Telomere-to-telomere gap-free genome assembly of the endangered Yangtze finless porpoise and East Asian finless porpoise. Gigascience. 2024;13:giae067.

85. Song JM, Xie WZ, Wang S, et al. Two gap-free reference genomes and a global view of the centromere architecture in rice. Mol Plant. 2021;14(10):1757-1767.

86. Zhang S, Xu N, Fu L, et al. Integrated analysis of the complete sequence of a macaque genome. Nature. 2025;640(8059):714-721.

87. Altemose N, Logsdon GA, Bzikadze AV, et al. Complete genomic and epigenetic maps of human centromeres. Science. 2022;376(6588):eabl4178.

88. Ding J, Li C, Shu K, et al. Membrane metalloendopeptidase (MME) is positively correlated with systemic lupus erythematosus and may inhibit the occurrence of breast cancer. PLoS One. 2023;18(8):e0289960.

89. Cheng CY, Zhou Z, Stone M, et al. Membrane metalloendopeptidase suppresses prostate carcinogenesis by

attenuating effects of gastrin-releasing peptide on stem/progenitor cells. Oncogenesis. 2020;9(3):38.
90. Moskalenko M, Ponomarenko I, Reshetnikov E, et al. Polymorphisms of the matrix metalloproteinase genes are associated with essential hypertension in a Caucasian population of Central Russia. Sci Rep. 2021;11(1):5224.
91. Cervellini I, Galino J, Zhu N, et al. Membrane metallo-endopeptidase is dispensable for repair after nerve injury. Glia. 2019;67(10):1990-2000.
92. Yuan J, Li J, Yong J, et al. Supporting data for "A telomere-to-telomere genome assembly of koi carp (Cyprinus carpio) using long reads and Hi-C technology" GigaScience Database. 2025. https://doi.org/10.5524/102721.

Figures and Legends

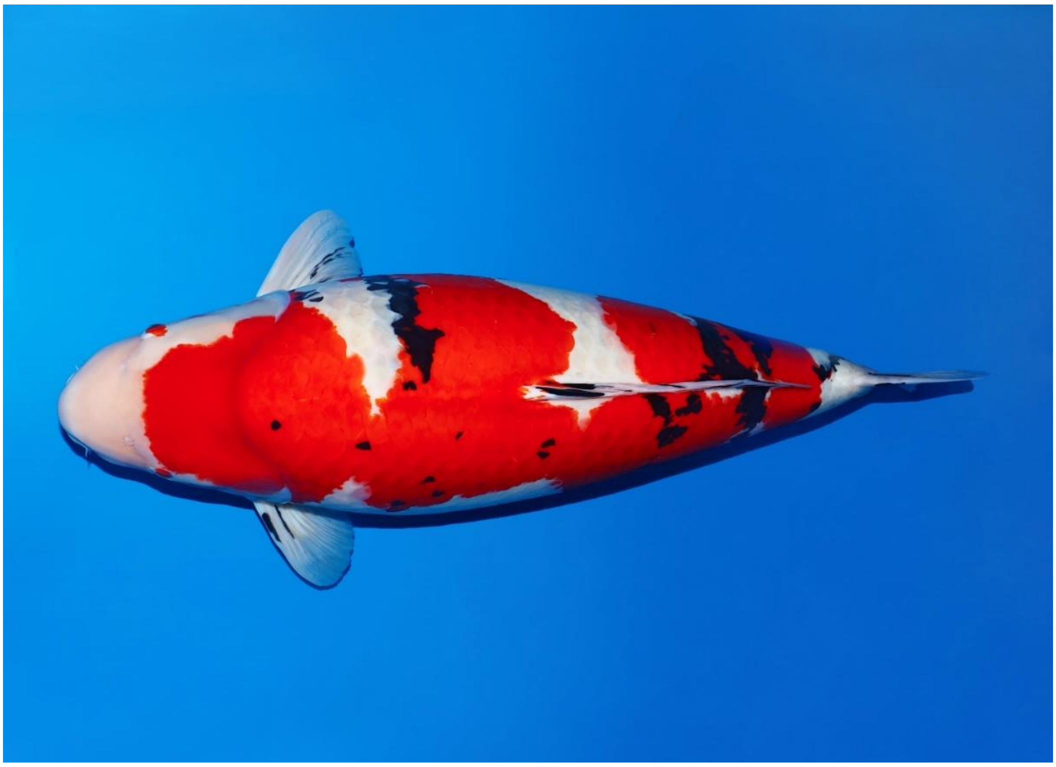

Fig. 1 | The koi carp strain Taisho Sansyoku in this study.

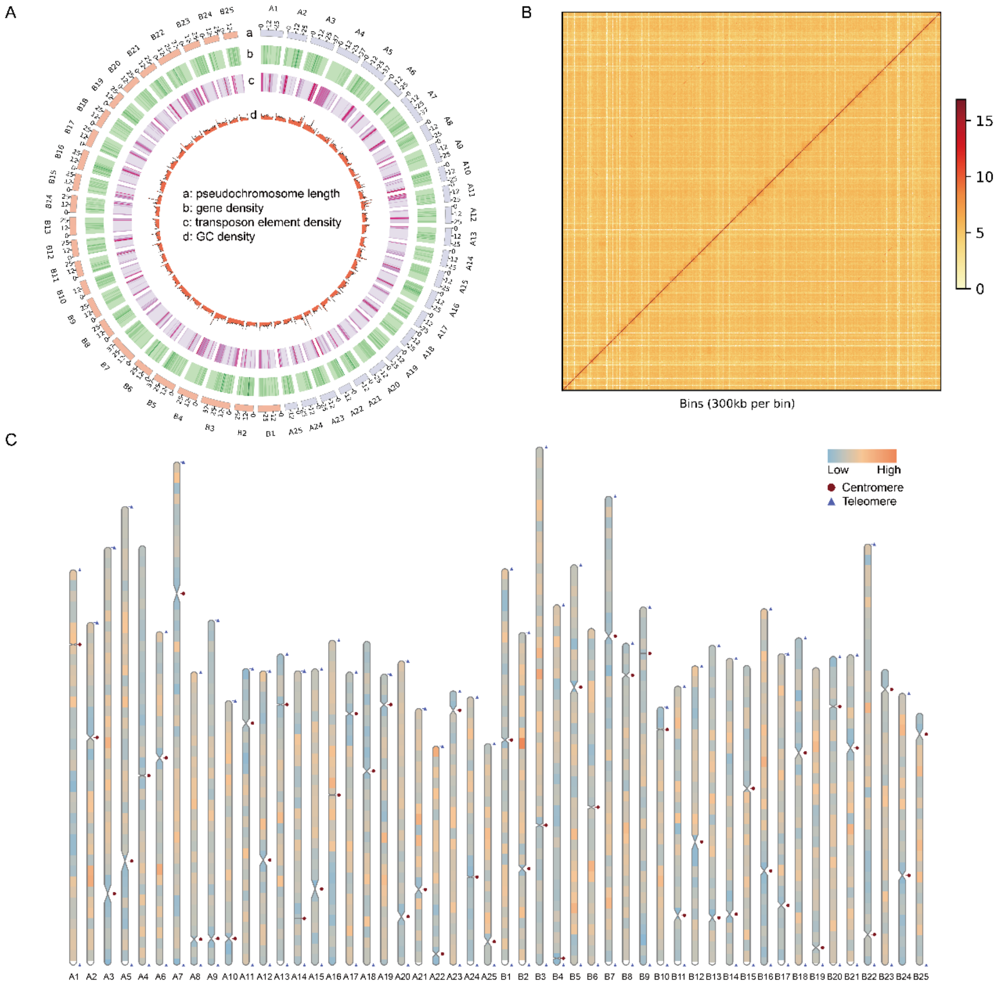

Fig. 2 | Genomic characteristics of the CC 4.0 genome. A, Circos plot showing the characterization of CC 4.0 genome. From outside to inside: a, The length of pseudo-chromosome in the size of Mb. b, gene density in 1-Mb sliding windows. c, percentage of transposon elements in 1 Mb sliding windows. d, GC content in non-overlapping 1Mb windows. **B**, Intensity signal heat map of the Hi-C chromosome interaction. The colour block illuminates the intensity of interaction from yellow (low) to red (high). **C**, Telomere and centromere detection map. Triangles and circles represent telomeres and centromere within the CC 4.0 assembled chromosomes. The orange color represents regions with high gene density, while the sky blue color represents regions with low gene density.

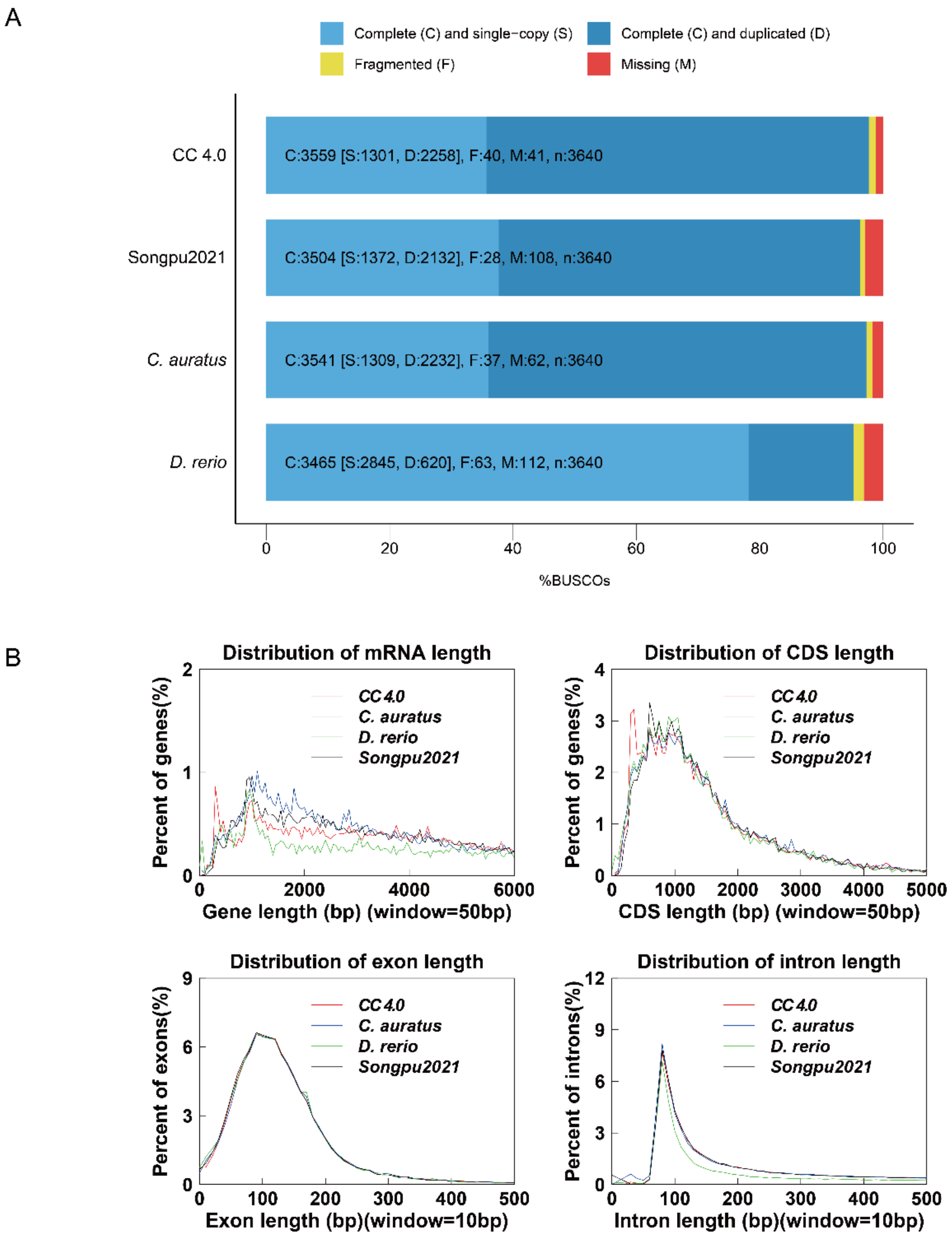

Fig. 3 | The high quality of gene annotation. A, BUSCO assessments of the koi carp genome assembly (CC 4.0), *C. auratus*, *D. rerio*, and Songpu2021. **B,** The composition of gene elements in the koi carp CC 4.0 genome compared to the other three genomes. The 'Songpu2021' refers to the common carp genome assembly retrieved from the NCBI database under the accession number GCA_018340385.1.

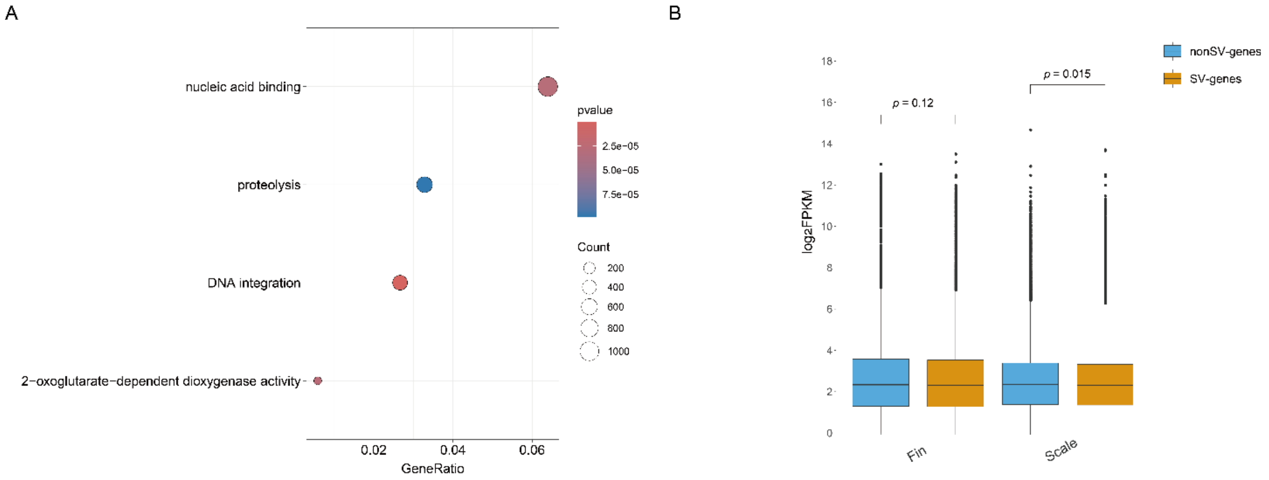

Fig. 4 | Comprehensive landscape of SVs between CC 4.0 and Songpu2021. A, GO enrichment analysis of SV-genes. The bubble size indicates the gene number of a biological process GO term, with color maps the *p*value of the enrichment analysis. GeneRatio: number of genes annotated to the GO category/total number of genes. **B,** The expression difference between genes with and without structural variants in multiple tissues. The statistical method used was the Wilcoxon test.

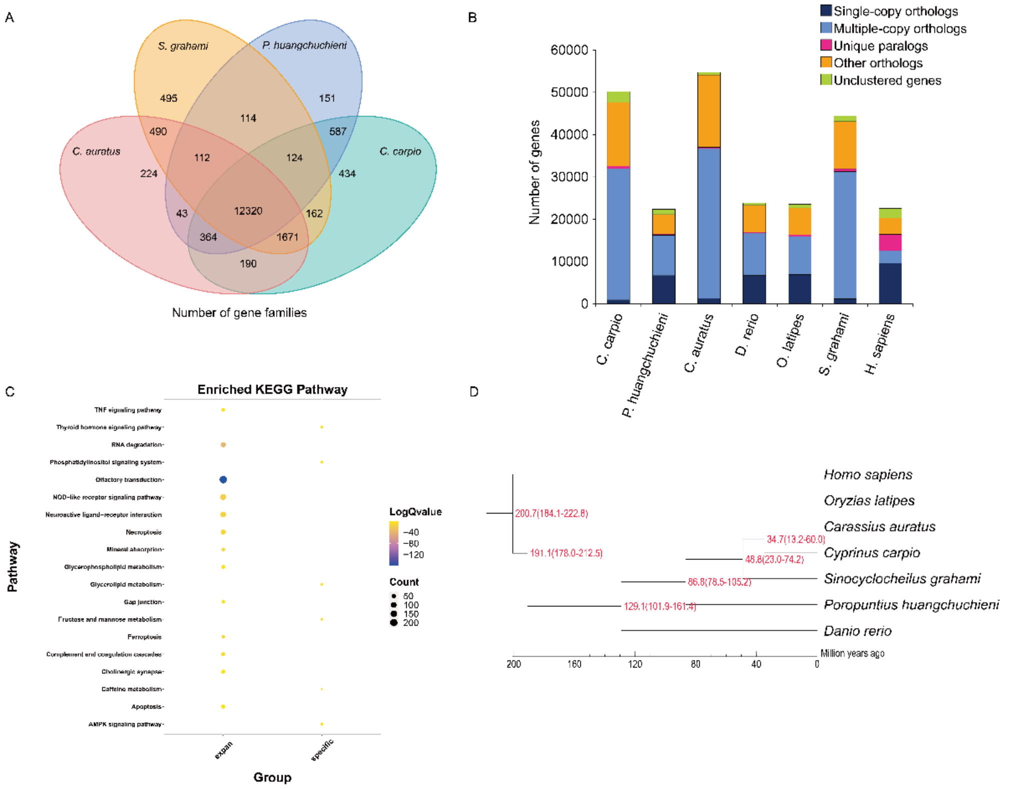

Fig. 5 | Evolution of the koi carp (CC 4.0) genome. A, Venn diagram of orthologous gene families in four genomes. The numbers represent quantities of gene families. **B,** Number of orthologous genes in seven species. **C,** Enrichment analysis of KEGG signaling pathway of specific genes and expansion gene families belongs to the CC 4.0 assembly. The size of the dots in the graph indicates the number of genes enriched in the pathway. The color indicates the significant Q-value of the pathway. **D,** Phylogenetic tree constructed using conserved house-keeping proteins from seven species.

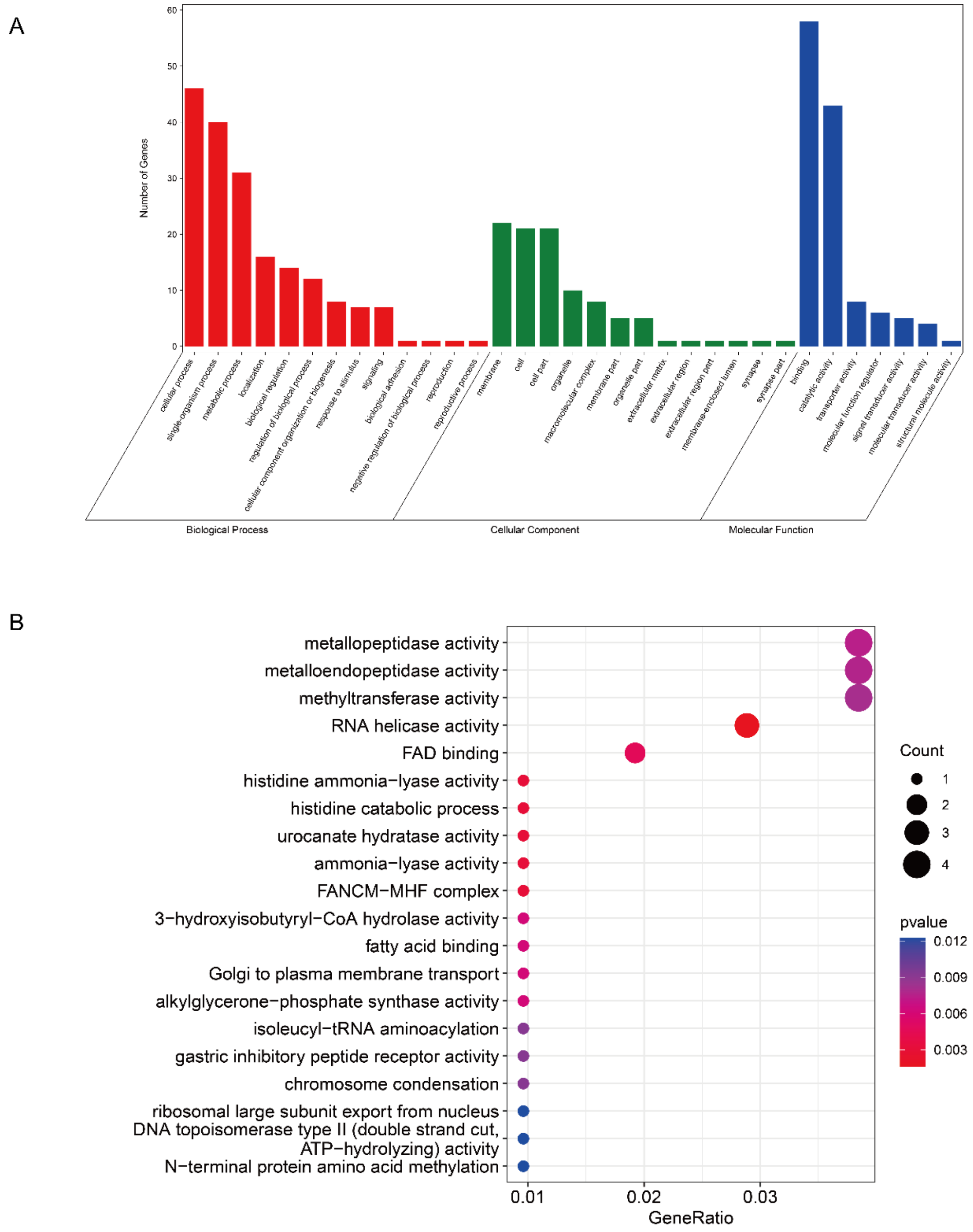

Fig. 6 | The GO function of PSGs in CC 4.0 genome. A, Web Gene Ontology Annotation Plotting plot showing GO distribution of PSGs. **B,** GO enrichment analysis of PSGs. The bubble size indicates the gene number of a biological process GO term, with color maps the *p*value of the enrichment analysis. GeneRatio: number of genes annotated to the GO category/total number of genes.

Figure 1

Click here to access/download;Figure;Figure 1.jpg

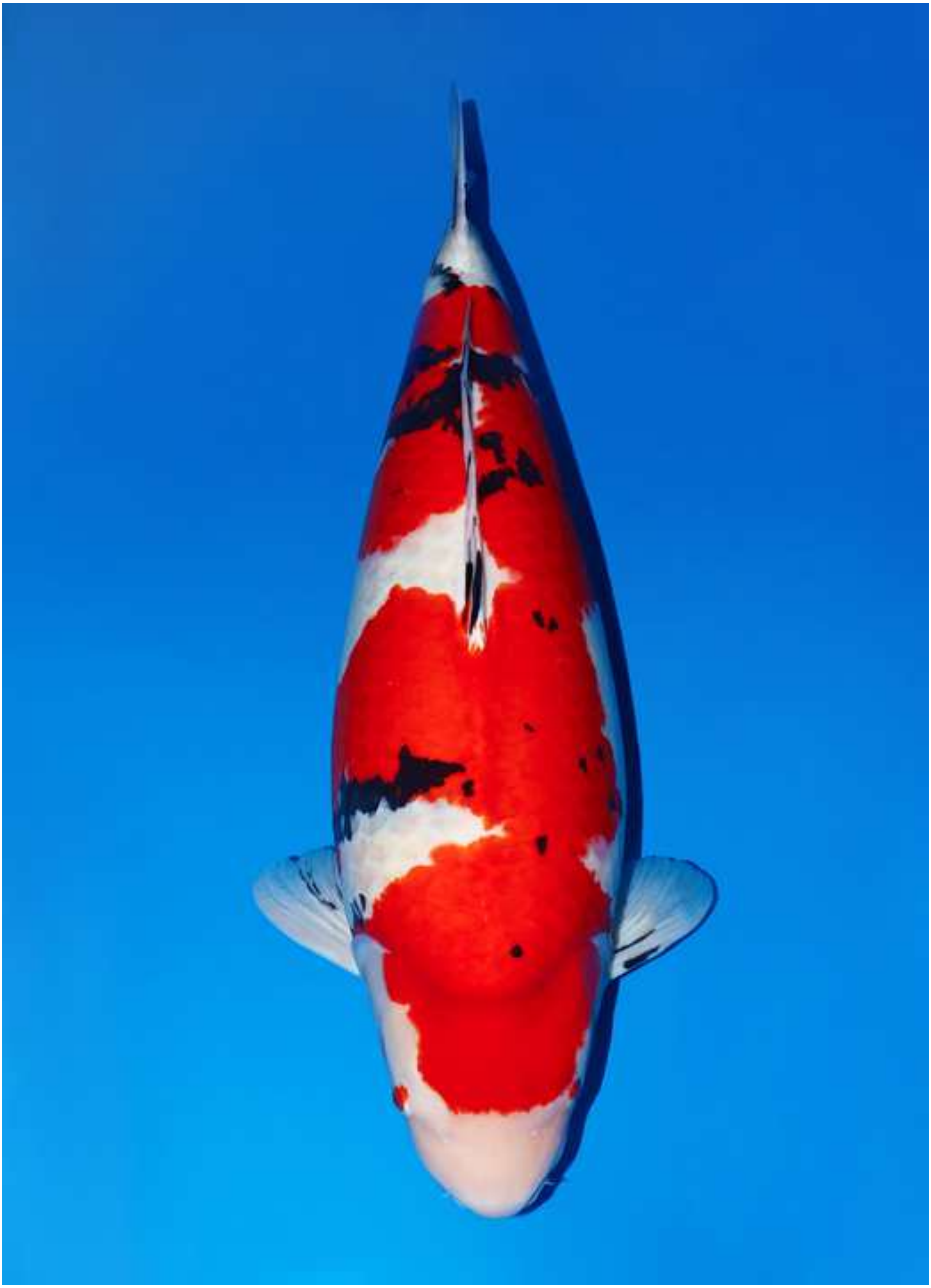

Figure 2

Click here to access/download;Figure;Figure 2.pdf

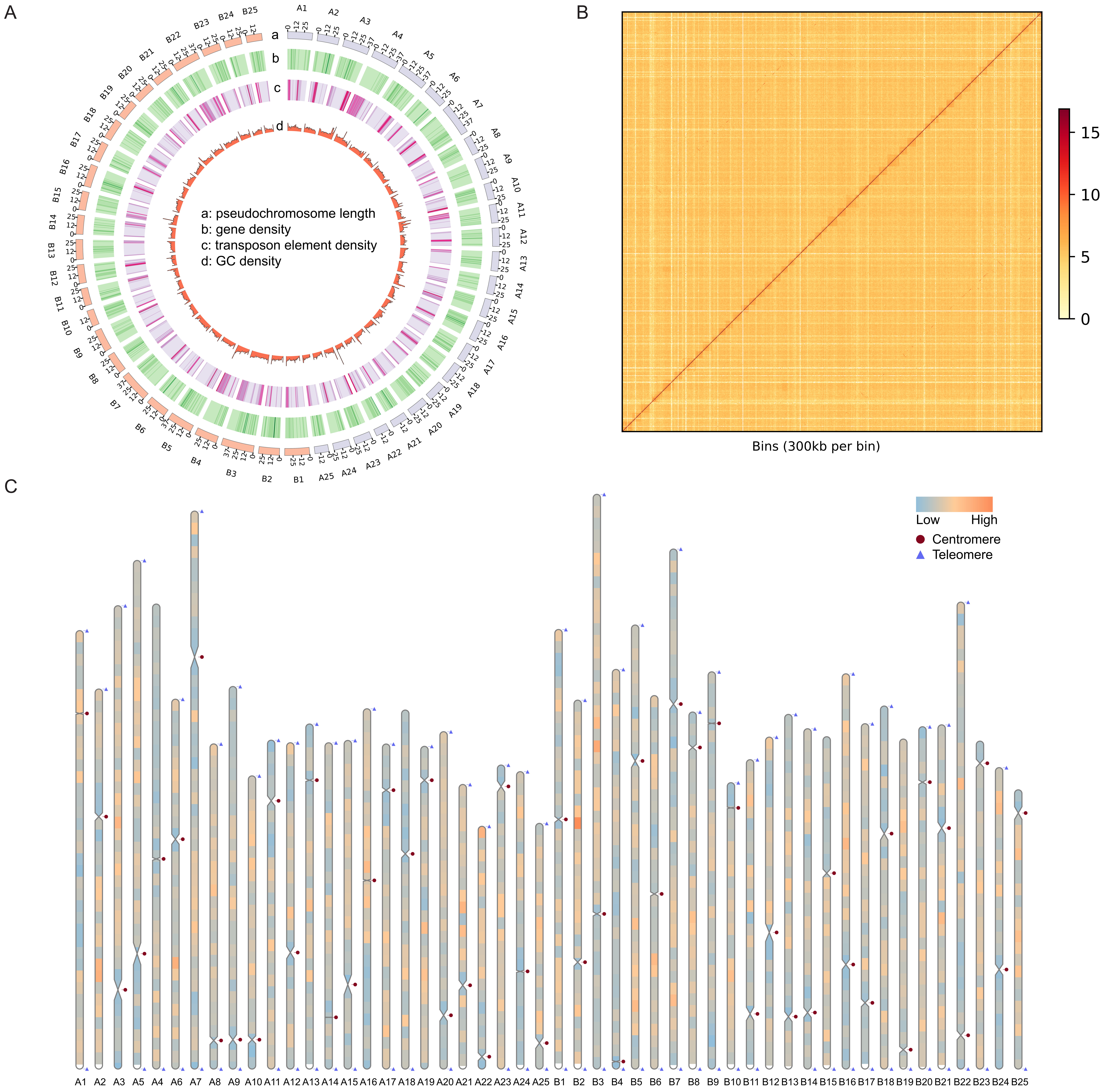

Figure 3
Click here to access/download;Figure;Figure 3.pdf

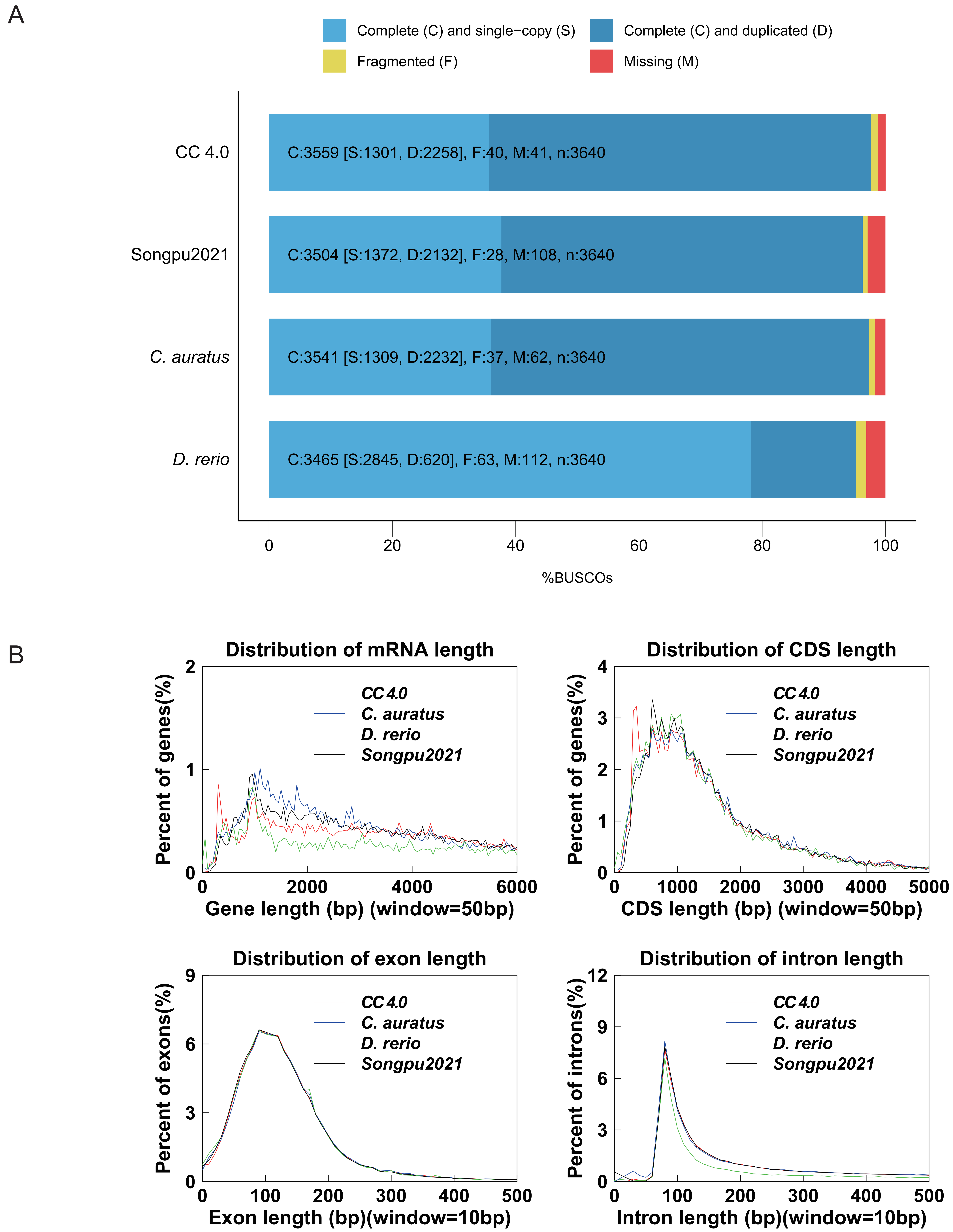

Click here to access/download;Figure;Figure 4.pdf

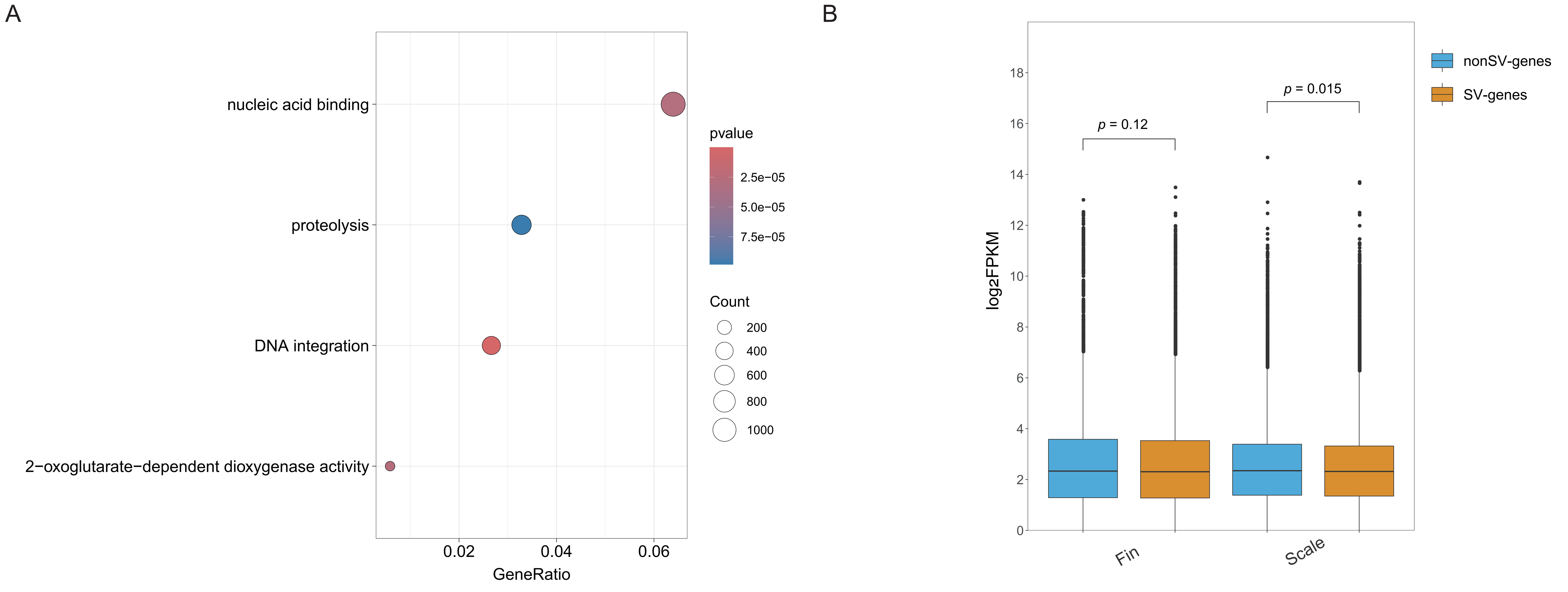

Figure 5

Click here to access/download;Figure;Figure 5.pdf

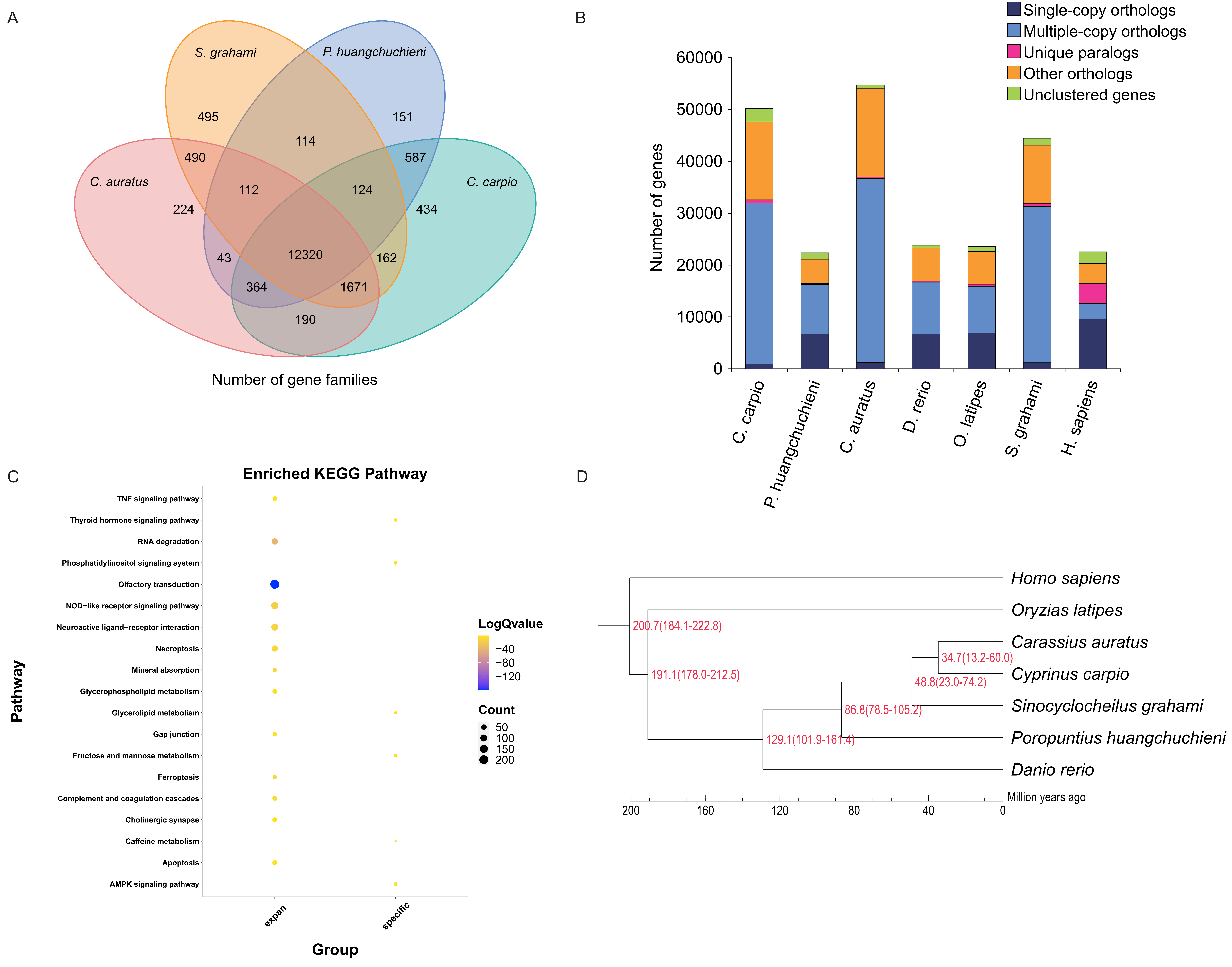

Figure 6

Click here to access/download;Figure;Figure 6.pdf

A

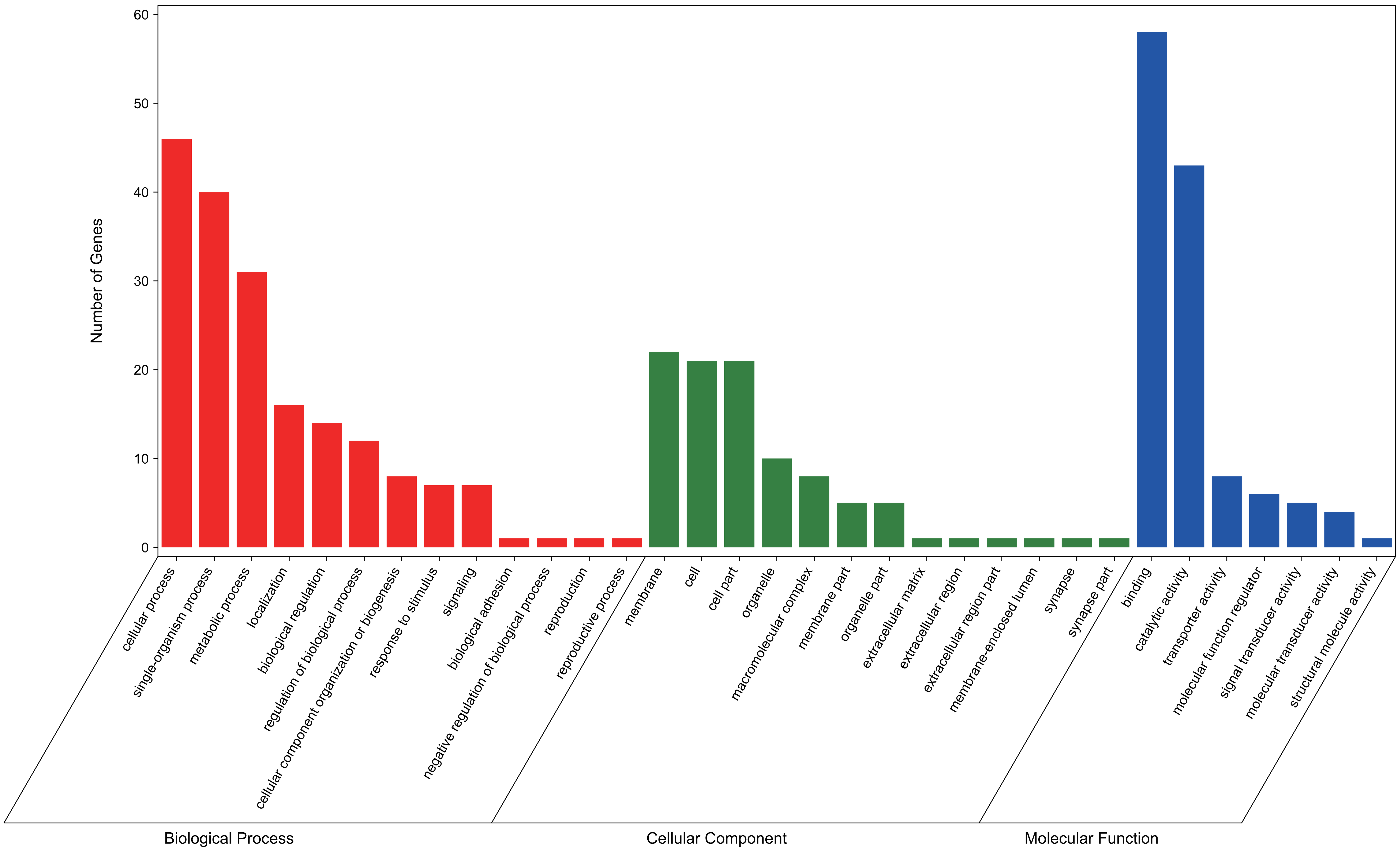

B

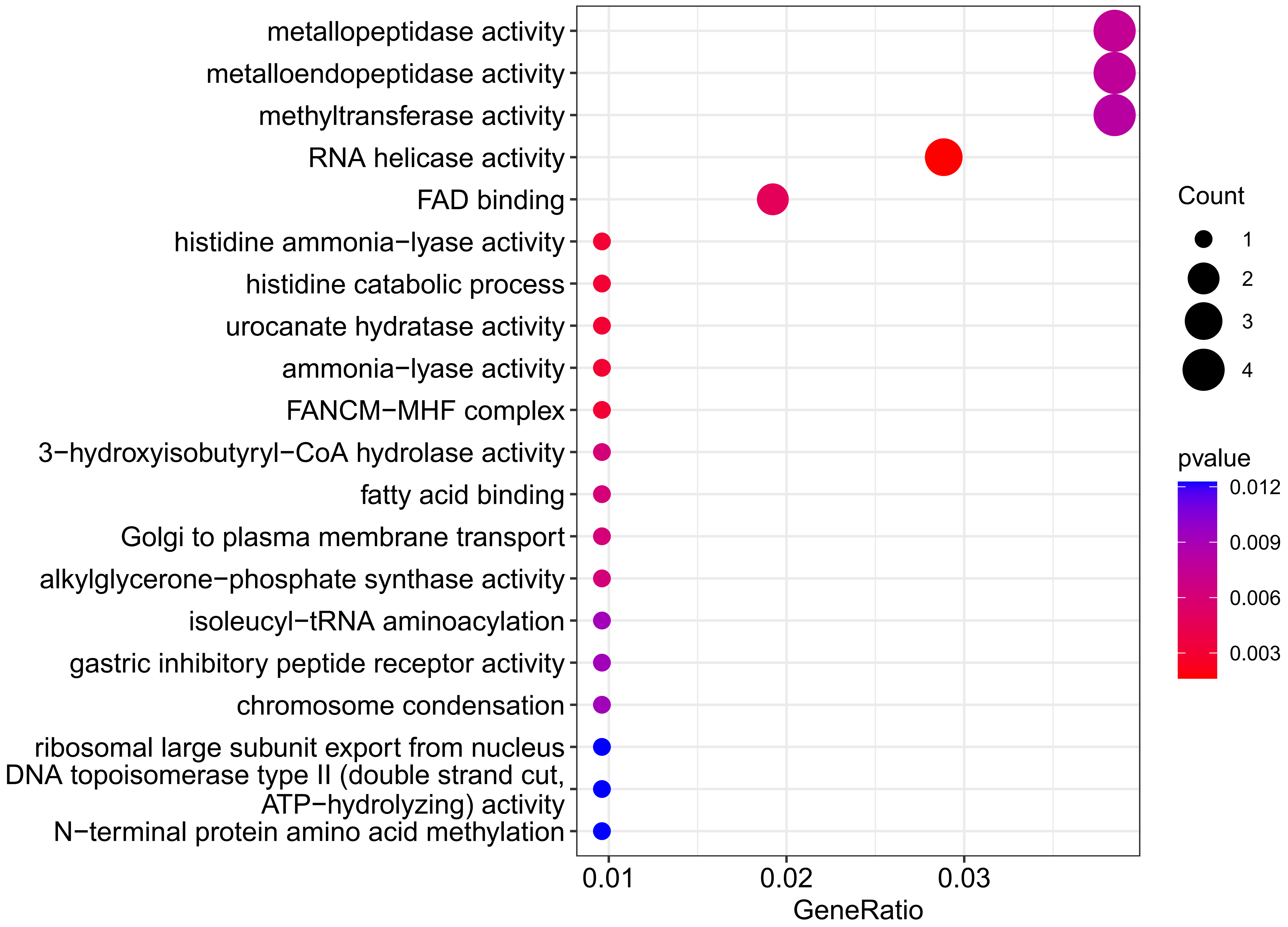

Supplementary tables

Click here to access/download
Supplementary Material
Revised Supplementary Table.pdf

Supplementary figures

Click here to access/download
Supplementary Material
Supplementary Figure.pdf
