# Supplementary material for: A telomere-to-telomere genome assembly of koi carp (Cyprinus carpio) using long reads and Hi-C technology
Source: Gigascience. 2025 Aug 29;14:giaf087. doi: 10.1093/gigascience/giaf087 (PMC12395963; doi:10.1093/gigascience/giaf087)
Supplement: giaf087_Authors_Response_To_Reviewer_Comments_original_submission [file giaf087_authors_response_to_reviewer_comments_original_submission.pdf]

Author's Response To Reviewer Comments

Reviewer reports:

Reviewer #1: Yuan and colleagues have sequenced and assembled the koi carp genome using data generated by several different advanced technologies, including PacBio HiFi, ONT ultralong sequences and Hi-C data. The sequencing data covered the genome over 470 times, resulting in a high-quality assembly (telomer-to-telomer assemblies for over 30 chromosomes, a few gaps, high BUSCO value, etc.). They have investigated the centromeric regions of the assembled pseudochromosomes and analyzed their genic and repeat content.

They have also analyzed the expanded gene families, unique genes and positively selected genes in the koi carp genome.

The authors propose that these data will provide "an invaluable resource for molecular-guided breeding and genetic enhancement strategies, underscoring the importance of common carp and koi carp in aquaculture and ecological research".

The Title covers the essence of the MS well. The flow of the Abstract is clear.
RE: Thanks for your positive comments on our work.

The Data Description section is concise and logical, but lacks important data published recently (see e.g. https://doi.org/10.1186/s12915-023-01806-9 and https://doi.org/10.1016/j.aquaculture.2024.741075). Due credit should be given to all others, who produced important and relevant data through the analysis of the common carp genome and transcriptome.
RE: Thanks for your comments. In the revised manuscript, we have included this two important papers in the introductions. Please find the details in lines 71-75 of the revised manuscript.

The Methods section is detailed, the sequencing and bioinformatic approaches that were taken by the authors are appropriate.
The Results are supported by two tables, five figures - four of which contain multiple panels - 14 Supplementary Tables and five Supplementary Figures. The description of data is logical, albeit sometimes potentially misleading (see below).
The Conclusions are succinct, but not complete and they do not always place the results into context well.
RE: Thanks for your comments. In line with your recommendation, the Conclusions has been supplemented with the previously omitted results. Please find more details in lines 428-440 of the revised manuscript. The description of data has been revised in the point-by-point responses below.

In summary, this Referee recommends a thorough revision of the MS prior to acceptance for publication in Gigascience.

Major criticisms:

A) In the opinion of this referee, the two most important aspects of this study are: (i) taking an important step towards the pan genome of common carp by improving the quality of the assembly; and (ii) revealing the genetic/genomic factors that led to the evolution of the colored koi carp varieties. In the current version of the MS, the authors do perform comparisons to emphasize the first aspect, but not the second. That leads to mis-interpretation of the data and wrong conclusions. Therefore, the MS should be revised to correct these errors and to help the reader to grasp the second advance better.
RE: Thanks for your comment. The manuscript, designed as a Data Note-type paper, focuses on the analytical aspects of constructing the T2T genome rather than focusing on biological stories. Its structure closely resembles that of the high-quality Chiridota heheva genome recently published in Gigascience (Pu, Zhou et al. 2024), prioritizing detailed analysis and data presentation. We appreciated your comments, so we have made efforts to improve the second aspect that you have mentioned. We conducted phylogenetic analysis and characterized positively selected genes in our study. Furthermore, a comprehensive genome-

wide comparative analysis between CC 4.0 and Songpu2021 was newly generated, which identifying extensive genomic variations (lines 374-388). These efforts provided readers with certain analytical directions and ideas to the genetic and genomic factors underlying the evolution of the colored koi carp varieties.

Reference：

Pu, Y., Zhou, Y., Liu, J. & Zhang, H. A high-quality chromosomal genome assembly of the sea cucumber Chiridota heheva and its hydrothermal adaptation. GigaScience 13(2024).

B) Terminology: The koi carp genome is referred to by several names in the MS, including 'gap-free genome assembly of koi carp', 'Taysho Sensyoku genome', 'final koi carp assembly (CC4.0)', 'gap-free koi carp CC4.0 genome assembly', and 'koi carp CC4.0 genome'. In the referee's opinion, the same name should be used throughout; 'koi carp genome assembly (CC4.0).
RE: Thanks for your valuable suggestion. We revised the manuscript to standardize the naming of the koi carp genome assembly as “koi carp genome assembly (CC4.0)” as suggested to avoid any confusion.

C) Phylogenetic relationship analysis (P16, L348-351): When the koi carp gene set is compared to those of six other vertebrates, genes that can only be found in the koi carp gene set cannot be called 'koi carp-specific'. The correct term here would be 'common carp-specific'. In order to find genes that are unique to koi carp the gene content of CC4.0 must be compared to CC3.0. The same refers to expanded and contracted gene families.
RE: We appreciate your attention to the accuracy and precision of our descriptions. We have revised the relevant sections in our revised manuscript.

D) Number formats: English uses decimal points and thousand separators in numbers, not the other way around. Therefore, all numbers in the tables and supplementary tables must be reformatted accordingly. Moreover, numbers in all tables must be flushed to the decimals to help the readers grasp their magnitude.
RE: Thanks for your comments. We have carefully revised all tables and supplementary tables to adhere to English numerical formatting standards, using decimal points and comma thousand separators (e.g., "748,299" instead of "748299") and aligning numbers by decimal points for clarity.

Additional criticisms (in the order of their appearance, not importance):
* Page 1, Line 15 vs. P2, L29-30: As correctly noted at the first appearance, koi carp is a variety of common carp. The second sentence must be corrected accordingly.
RE: Thanks for your comment. We changed “this species” to “common carp”. Please see lines 29-30 of the revised manuscript.

* P4, L79-80: The sex of the individual used for sequencing must be indicated here.
RE: Thanks for your comment. A 6-year-old female koi was used for sequencing in this study, the information has been added into the revised manuscript (line 96).

* P4, L81-84: Is there a specific reason, why the gonad RNA was not used for transcriptomics?
RE: Thanks for your comment. During the sampling process, the quality of the gonad RNA sample was suboptimal, so the tissue sample was not utilized. In this study, transcriptome data was predominantly employed for the annotation of gene sets. The annotation of gene sets encompasses not only transcriptome - derived sources but also homologous annotations and de novo annotations. The gene set annotation in this study is of high quality, as evidenced by a BUSCO score of 97.77. In the Songpu2011 genome study, gonad samples were not utilized either. Therefore, we consider that the absence of gonad samples may not affect the annotation quality.

* P12, L245: The correct term is 'positively selected genes'. This must be corrected throughout the MS.
RE: Thanks for your reminder. We corrected this term throughout the revised manuscript.

* P14, L307 - P15, L312: How do these data compare to those of CC3.0?

RE: Thanks for your comment. The main types of repeat sequences in CC 4.0 are consistent with those in CC 3.0. DNA transposons are the dominant transposable elements, and long terminal repeats (LTRs) and long interspersed nuclear elements (LINEs) have comparable proportions within the genome.
Regarding noncoding RNAs, although the CC 3.0 paper mentions that "RNAmmer and tRNAScan were used for rRNA and tRNA prediction, respectively", neither the main text nor its attachments present the relevant results. Consequently, we are unable to conduct a comparison.

* P15, L327-329: It is unnecessary to use two decimals in these numbers.
RE: We have deleted two decimal decimals in these numbers. Please find more details in line 354 of the revised manuscript.

* P15, L326 - P16, L341: How do the koi carp centromeres compare to other cyprinid or teleost centromeres? If not available they should be compared to mammalian centromeres.
RE: Given that telomere-to-telomere genomes of the closely related species goldfish and zebrafish remain unpublished, we compared our results with those of the Yangtze finless porpoise, rice, human, and macaque. Please find more details in lines 356-358, 365-372 of the revised manuscript.

* P16, L331: Erroneous pointer. Please correct to: (Fig.2C).
RE: Thanks, revised.

* P17, L358: What proportion of these expansions and contractions are present in the common carp genome (CC3.0)?
RE: Thank you for your comment. In the study of the CC 3.0 genome, the authors did not perform an analysis of gene family expansions and contractions. The CC 3.0 genome study mainly concentrated on aspects such as high - fidelity genome assembly, subgenome structural evolution, homoeologous expression, and the response to thermal stress. Consequently, the proportion of gene family expansions and contractions in the CC 3.0 genome cannot be obtained.

* P17, L371: What proportion of these 124 genes are positively selected in CC3.0?
RE: Similar to the previous question, the authors also did not carry out a positive selection analysis on CC 3.0 genome.

* P18, L382: Actually, this is the first T2T genome assembly for the whole species that happens to be a koi variety.
RE: Thank you for your reminder. In the revised manscript, we have replaced "koi carp" with "common carp". Please find more details in line 427 of the revised manuscript.

* P18, L382-390: Centromeric regions and positively selected genes must be mentioned in the Conclusion.
RE: Thank you for your reminder. The results regarding centromeric regions and positively selected genes have been presented in the conclusion of the revised manuscript. Please find more details in lines 429-432, 436-438 of the revised manuscript.

* Reference list: The list contains over a dozen incomplete citations that must be completed. This referee recommends that title should not be fully italicized, so that the italicized Latin name would stand out according to the convention. Also, the formatting of reference list must be unified.
RE: Thank you for your reminder. We have addressed the reference list issues in the revised manuscript.

* Figure 3, Panel A: CC3.0 and CC4.0 should be next to each other.
RE：In panel A of figure 3, the primary species compared are CC 4.0 and Songpu 2021. The species order has been revised in accordance with your suggestion. A detailed comparison between CC 4.0 and CC 3.0 is presented in Table 1.

* Supplementary Figure S1, Legend: Grammatical error. Please correct 'divide' to 'divideD'.
RE: Revised.

* Supplementary Figure S3, Legend: Please correct 'abscissa and ordinate' to 'X-axis and Y-axis'.

RE: Revised.

* Supplementary Figure S4: The (grammatically incorrect) title must be removed from the top of the figure. All essential info must be provided by the legend.
* Supplementary Figure S4, Legend: Instead of 'blue and green boxes', the figure contains only blue bars. The legend must be revised to reflect the content of the illustration.
* Ibid: Grammatical error ('gene families belongs').
RE: Supplementary Figure S4 has been revised with supplementary annotations, and additional interpretive details have been incorporated.

* Supplementary Figure S5, Legend: The legend must clearly indicate which GO category (Molecular Function, Cellular Component, or Biological Process) is shown on the illustration. The last sentence must be revised to indicate 'diameter of circle' instead of GO term.
RE: We understand the significance of GO term classification. In our study, GO terms were ranked in ascending order of P.adjusted values to emphasize the most statistically significant functional categories, as previously demonstrated in Figure S15 of the mouse T2T genome study (Liu, Li et al. 2024).
The final sentence has been revised in accordance with your recommendations.

Reference: Liu, J. et al. The complete telomere-to-telomere sequence of a mouse genome. Science. 386, 1141-1146 (2024).

Suggestion at the discretion of the authors:

* Table 1: This is a nice, informative table. The authors should consider extending it with data from earlier version of common carp genome assembly with years indicated to visualize the progression of the field.
RE: Thanks for your comment. We have incorporated relevant statistics regarding the Songpu2021 genome into Table 1.

Reviewer #2: In this paper, the authors describe T2T genome assembly of an ornamental-colored common carp (Cyprinus carpio) strain, Taisho Sanshoku. The assembly achieved a total size of 1555.86 Mb with a contig N50 of 30.45 Mb, comprising 50 gap-free pseudochromosomes. The BUSCO completeness score reached 99.20%. In this assembly, 83% of telomeres were detected, suggesting that 33 chromosomes possess complete telomeres. The findings are relatively descriptive; however, these data are useful for researchers in this field and suitable for publication in this journal after revisions. Several essential concerns should be addressed.

1. The authors did not explain what is "Taisho Sanshoku" koi carp strain. It is a Nishikigoi strain that was established in the 19th century in Niigata Prefecture in Japan. A review written by EK Balon describes the origin of them. The authors should add an explanation of this strain and its origin in the introduction and refer to this.

Balon, Eugene K. "The common carp, Cyprinus carpio: its wild origin, domestication in aquaculture, and selection as colored Nishikigoi." Guelph Ichthyology Reviews 3 (1995).

RE: Thank you for your reminder. In the revised manuscript, we have offered supplementary elucidations with respect to the pertinent characteristics of "Taisho Sanshoku" koi carp strain. Please find the details in lines 84-89 of the revised manuscript.

2. It is better to discuss allotetraploidy in common carp and goldfish, a species very closely related to common carp. The following papers that investigated allotetraploid in goldfish would help the understudying for readers.

Zelin Chen et al., Science Advances 2019 Vol 5, Issue 6 DOI: 10.1126/sciadv.aav0547
Tetsuo Kon et al., Current Biology 2020 doi.org/10.1016/j.cub.2020.04.034
Jing Luo et al., Science Advances 2020 Vol 6, Issue 22 DOI: 10.1126/sciadv.aaz7677

RE: Thanks for your valuable suggestions. We have included these papers and added the supplementary description in the revised manuscript. Please see the details in lines 48-56 of the revised manuscript.

3. The authors compared their new assembly (CC 4.0) and previously published assembly (CC 3.0) in Table 1. In addition, it is better to compare the previous assembly shown in Ref 6 (Li et al. 2021). This comparison would highlight the progress of this paper.
RE: Thank you for your nice suggestion. In the revised manuscript, we have incorporated the detailed comparison results from Ref 6 (Li et al., 2021) into Table 1. Please find the details in Table 1 of the revised manuscript.

4. The authors should compare them and discuss the differences between Taisho Sanshoku and Songupu strains.
RE: Thank you for your nice suggest. We conducted comparative analysis of Taisho Sanshoku and Songupu strands at the whole genome level. Please find more details in lines 236-248, 375-387 of the revised manuscript.

5. The gene annotation file (GFF3 file) shows gene IDs (i.e. B23G0250) that the authors defined in this paper. These gene codes should link the gene names and gene symbols. This list should be added in a new Supplementary Table.
RE: Thank you for your comment. The gene symbol file, named "CC4.0 gene symbol.xls", has been uploaded to FigShare at the following link: https://figshare.com/s/93779280afb6ad32eafb.

6. It is useful to add gene symbols in the GFF3 file. It is useful for readers.
RE: Thank you for your comment. The GFF file containing symbol information has been uploaded to FigShare at the following link: https://figshare.com/s/93779280afb6ad32eafb (The file is named "Carp.add.symbol.gff3.gz").

7. At least the gene annotation file (gff3) and genome assembly file (fasta file) should be deposited on the public database such as Figshare. It should show in "Data Availability".
RE: Thank you for your comment. The gene annotation file (in GFF3 format) and the genome assembly file (in FASTA format) are now accessible on FigShare at the following link: https://figshare.com/s/93779280afb6ad32eafb.

Editorial Manager®

Cookie Preference Center

We use cookies which are necessary to make our site work. We may also use additional cookies to analyze, improve and personalize our content and your digital experience. For more information, see our Cookie Policy.

You may choose not to allow some types of cookies. However, blocking some types may impact your experience of our site and the services we are able to offer. See the different category headings below to find out more or change your settings.

Allow all

Manage Consent Preferences

Strictly Necessary Cookies

Always active

These cookies are necessary for the website to function and cannot be switched off in our systems. They are usually only set in response to actions made by you which amount to a request for services, such as setting your privacy preferences, logging in or filling in forms. You can set your browser to block or alert you about these cookies, but some parts of the site will not then work.

Functional Cookies

These cookies enable the website to provide enhanced functionality and personalisation. They may be set by us or by third party providers whose services we have added to our pages. If you do not allow these cookies then some or all of these services may not function properly.

Performance Cookies

These cookies allow us to count visits and traffic sources so we can measure and improve the performance of our site. They help us to know which pages are the most and least popular and see how visitors move around the site.

Targeting Cookies

These cookies may be set through our site by our advertising partners. They may be used by those companies to build a profile of your interests and show you relevant adverts on other sites. If you do not allow these cookies, you will experience less targeted advertising.

Cookie List

Clear

Apply Cancel

Consent Leg.Interest

Confirm my choices

Powered by onetrust
