# Supplementary material for: A telomere-to-telomere genome assembly of koi carp (Cyprinus carpio) using long reads and Hi-C technology
Source: Gigascience. 2025 Aug 29;14:giaf087. doi: 10.1093/gigascience/giaf087 (PMC12395963; doi:10.1093/gigascience/giaf087)
Supplement: giaf087_Authors_Response_To_Reviewer_Comments_Revision_1 [file giaf087_authors_response_to_reviewer_comments_revision_1.pdf]

Author's Response To Reviewer Comments

Reviewer reports:
Reviewer #1: The authors have revised their MS by taking care of some of the issues identified earlier by this referee. Unfortunately, several issues remained to be unsolved despite assurances given by the authors I their replies.

Uncorrected problems identified in the previous round:
RE: We sincerely appreciate your thorough review, which has enabled us to produce a more precise manuscript. We are confident that this collaborative process will yield a publication-ready version that meets the academic standards.

* B) Terminology: The koi carp genome is referred to by several names in the MS, including 'gap-free genome assembly of koi carp', 'Taisho Sensyoku genome', 'final koi carp assembly (CC4.0)', 'gap-free koi carp CC4.0 genome assembly', and 'koi carp CC4.0 genome'. In the referee's opinion, the same name should be used throughout; 'koi carp genome assembly (CC4.0).
Authors' reply: Thanks for your valuable suggestion. We revised the manuscript to standardize the naming of the koi carp genome assembly as "koi carp genome assembly (CC4.0)" as suggested to avoid any confusion.
Referee's reply: The term 'Taisho Sensyoku' still appears on several panels of Figure 3. Also, Songpu2021 must be replaced or at least explained in Figures 3&4, as it is not abvious for the non-expert reader that this means the second genome assembly for common carp.
RE: The term ‘Taisho Sensyoku’ in Figure 3 has been replaced with ‘CC 4.0’. The designation ‘Songpu2021’ was explained in lines 65 and 465–466 of the revised manuscript. Additionally, an explanation of “The ‘Songpu2021’ refers to the common carp genome assembly retrieved from the NCBI database under the accession number GCA_018340385.1.”, has been incorporated into the legend of Figure 3. Please find more details in lines 743-745 of the revised manuscript.

* D) This referee requested that all numbers in the tables and supplementary tables must be reformatted according to the rules of English grammar (i.e., decimal points and commas as thousand separators, not the other way around). Moreover, numbers in all tables must be flushed to the decimals to help the readers grasp their magnitude.
Authors' reply: "We have carefully revised all tables and supplementary tables to adhere to English numerical formatting standards, using
decimal points and comma thousand separators (e.g., "748,299" instead of "748299") and aligning numbers by decimal points for clarity."
Referee's reply: In reality, the first request, but not the second has been complied with for Tables 1&2. Unfortunately, neither has been complied with for the Supplementary Tables.
RE: Thank you for your feedback. The tables have been revised as requested.

* Reference list: The list contains over a dozen incomplete citations that must be completed. This referee recommends that title should not be fully italicized, so that the italicized Latin name would stand out according to the convention. Also, the formatting of reference list must be unified.
Authors' reply: Thank you for your reminder. We have addressed the reference list issues in the revised manuscript.
Referee's reply: Despite the above, the List of References still contains nearly a dozen incomplete references (see e.g., 5, 18, 31, 34, 36, 47, 50, 51, 55). The references are not uniformly formatted (no need for a 'p.' in front of page numbers). The position of the year is unusual in the references> it is typically either behind the authors' names or at the very end of the citation. Latin species names are not italicized.
RE: We appreciate your guidance on the formatting issues in the references. The references list has been carefully checked and revised.

Newly introduced or newly discovered problems:
* General: The revised version of the MS still contains several grammatical errors and typos, and it is very

difficult to read. Therefore, the revised version should be carefully screened for these or alternatively sent to a professional language-editing and copyediting service before re-submission.
RE: Thank you for your suggestion. The manuscript has been thoroughly revised to correct grammatical and spelling errors. We have contacted the editorial office of GigaScience, and they will provide more professional language editing services to ensure the linguistic accuracy of the final published version.

* P2, L29-30: Grammatical error. Please correct to "… understanding of the BIOLOGY and adaptation of common carp."
RE: Thanks for your suggestions. We have modified the sentence. Please find more details in lines 29-30 of our revised manuscript.

* P16, L348-350 vs. Supp. Table S13: According to the text, there are 589 carp-specific genes in 245 families. On the other hand, Supp. Table S13 mentions 2,578 such genes in 245 families. Which number is correct?
RE: We apologize for the mistake. The value “589” is correct. We have updated it in Table S13.

* P18, L381-382: Redundant sentences, as SV genes have already been defined on P12, L246-247.
RE: Thanks for your comments. We have removed the sentence “These genes were referred to as ‘SV-genes’.”

* The List of Abbreviations is incomplete: quite a few abbreviations (e.g., GCI, ONT, TRF) are missing.
RE: Thanks for your reminder. We have supplemented the missing list of abbreviations in our revised article by adding abbreviations for CDS, GCI, Kb, NCBI, ONT, PacBio, and TRF. Please find more details in lines 441-450 of our revised manuscript.

* Table 2: The current version of the table is very difficult to understand. Several columns are without metrics, these must be added to the first row. Numbers in rows #6 - #11 are presumably percentages without indicating the point of reference. Detailed explanation for every category must be provided as notes below the table.
RE: Thanks for your comments. The header of Table 2 has been revised, and explanatory notes have been added under the table.

* Supp. Tables S4 vs. S5: Chromosomes in Supp. Table S4 are listed as 1-50, whereas those in S5 are listed as A1-25 and B1-25. Please unify throughout the MS.
RE: We apologize for any inconvenience caused. The chromosome IDs in Table S4 have been revised to ensure consistency across Tables S4, S5, and Figure 2 in the main text.

* Supp. Tables S10, S14 and S15: These gene IDs are not useful in their current format. Transcripts should be mapped to RefSeq or another publicly available database to allow the reader to search for the genes, not only the proteins.
RE: Thanks for your comments. To provide more useful information, we assign gene symbols and corresponding descriptions based on the protein sequence alignment results. Additionally, as you suggested, we used Blast software to align each gene’s nucleic acid sequence with zebrafish nucleic acid sequences from the NCBI RefSeq database (https://ftp.ncbi.nlm.nih.gov/refseq/D_rerio/) and incorporated the best alignment hits into Tables S10, S14, and S15.

Cookie Preference Center

We use cookies which are necessary to make our site work. We may also use additional cookies to analyze, improve and personalize our content and your digital experience. For more information, see our Cookie Policy.

You may choose not to allow some types of cookies. However, blocking some types may impact your experience of our site and the services we are able to offer. See the different category headings below to find out more or change your settings.

Allow all

Manage Consent Preferences

Strictly Necessary Cookies

Always active

These cookies are necessary for the website to function and cannot be switched off in our systems. They are usually only set in response to actions made by you which amount to a request for services, such as setting your privacy preferences, logging in or filling in forms. You can set your browser to block or alert you about these cookies, but some parts of the site will not then work.

Functional Cookies

These cookies enable the website to provide enhanced functionality and personalisation. They may be set by us or by third party providers whose services we have added to our pages. If you do not allow these cookies then some or all of these services may not function properly.

Performance Cookies

These cookies allow us to count visits and traffic sources so we can measure and improve the performance of our site. They help us to know which pages are the most and least popular and see how visitors move around the site.

Targeting Cookies

These cookies may be set through our site by our advertising partners. They may be used by those companies to build a profile of your interests and show you relevant adverts on other sites. If you do not allow these cookies, you will experience less targeted advertising.

Cookie List

Clear

Apply Cancel
Consent Leg.Interest

Confirm my choices

Powered by onetrust
