# Supplementary material for: A telomere-to-telomere genome assembly of koi carp (Cyprinus carpio) using long reads and Hi-C technology
Source: Gigascience. 2025 Aug 29;14:giaf087. doi: 10.1093/gigascience/giaf087 (PMC12395963; doi:10.1093/gigascience/giaf087)
Supplement: giaf087_Authors_Response_To_Reviewer_Comments_Revision_2 [file giaf087_authors_response_to_reviewer_comments_revision_2.pdf]

Author's Response To Reviewer Comments

Reviewer reports:
Reviewer #1: The authors have revised their MS again, but still left several problems identified earlier by this referee uncorrected. This referee is wondering, why several issues remained to be unsolved over two rounds of revision despite assurances given by the authors about the opposite. This referee has no other choice than returning the MS and asking for the corrections for the third time.
In order to help the authors, the remaining issues are now broken down to an itemized point-by-point list containing more detailed explanations regarding the tasks.
Re: Thank you for your comments and assistance. We believe that the latest updated version has addressed all the issues you mentioned.

* Using the term 'Taisho Sensyoku genome': This referee requested that the same name should be used for the koi carp genome assembly throughout the MS. The authors agreed and claimed that 'koi carp genome assembly (CC 4.0)' is now used everywhere. Yet, the second revised version still contains 'Taisho Sensyoku genome' (P4, L88) and 'BUSCO assessment of Taisho Sensyoku' (P34, L739).
Re: Revised.

* Numbers in Supplementary Tables: This referee noticed earlier that numbers in many of these tables contained decimal commas and periods as thousand indicators, whereas English language is using decimal points and commas as thousand indicators. The authors claimed that all these have been corrected. In reality, most supplementary tables (Supp. Tables S1-S11) of the second revised version still contain these errors.
Re: Thank you for your comment. We suspect that the Excel software may automatically adjusts delimiters according to the regional settings of the operating system by defult. For example: If the system region is set to "China", Excel will be used a comma as the thousands separator. If the setting is "Germany", a period or space will be used as the thousands separator, and a comma will serve as the decimal point.
To avoid this situation, in the updated version, we provide a PDF version of the supplementary tables. Now, the thousands separator should be the comma "," and the decimal points should be the period ".".

* Aligning numbers to the decimal point in Tables and Supp. Tables: This referee requested repeatedly that numbers in many of these tables should be aligned to the decimal points, as this allows the reader to grasp their magnitude quicker. contained decimal commas and periods as thousand indicators. Although the authors claimed that this has been done, numbers in both Tables and most supplementary tables (Supp. Tables S1-S11) are still not aligned properly.
Re: The numbers were aligned to the decimal point in Tables and supplementary tables.

* Supplementary Tables S13: Please eliminate the duplication (genes genes) from the top of the last column.
Re: Revised.

* Supplementary Tables S14 and S15: Please replace 'Nr' with 'NCBI non-redundant (protein) database' on the top of the second column of both Tables.
Re: Revised.
* P21, L364: Please replace the word 'genome research on rice' with 'genome assembly of rice'.
Re: Revised.

* P22, L383: Please remove the word 'tissue' from this sentence. The revised sentence should read as follows: '…negative effects on gene expression in scales, whereas no such impacts were observed in fins…'.
Re: Revised.

* P22, L388-389: The term 'including' typically indicates an incomplete list of a larger group. Here, all the sex species are listed, therefore, the sentence should read as follows: '… species (P. huangchuchieni, C.

auratus, D. rerio, O. latipes, S. grahami, and H. sapiens) were…'.
Re: Revised.

* P24, L430: The term 'DNA and protein processes' is very general. The authors should replace it with more specific collective term(s).
Re: The sentence was revised by "Genes in centromeric regions were significantly enriched in ten GO terms, including DNA integration, nucleic acid binding, protein export from nucleus, and nuclear export signal receptor activity.".

* List of References: This referee requested that all references on the list must be complete with unique IDs. The authors claimed that it has been done. Yet, BioRxiv references #18 and #62 do not have doi IDs, whereas #31 cannot be found with this title on arXiv.
Re: Thanks for your comments. #18, #31 and #62 were revised as follows:
18. Luo L, Wu H, Zhao L, et al. Telomere-to-telomere sheep genome assembly identifies variants associated with wool fineness. Nat Genet. 2025;57(1):218-230.
31. Liu, B., Shi Y., Yuan J., et al. Estimation of genomic characteristics by analyzing k mer frequency in de novo genome projects. arXiv:1308.2012v2 [q-bio.GN]. doi: https://doi.org/10.48550/arXiv.1308.2012
62. Holst F, Bolger A, Günther C, et al. Helixer–de novo Prediction of Primary Eukaryotic Gene Models Combining Deep Learning and a Hidden Markov Model. bioRxiv:2023.02.06.527280. doi: https://doi.org/10.1101/2023.02.06.527280

Cookie Preference Center

We use cookies which are necessary to make our site work. We may also use additional cookies to analyze, improve and personalize our content and your digital experience. For more information, see our Cookie Policy.

You may choose not to allow some types of cookies. However, blocking some types may impact your experience of our site and the services we are able to offer. See the different category headings below to find out more or change your settings.

Allow all

Manage Consent Preferences

Strictly Necessary Cookies

Always active

These cookies are necessary for the website to function and cannot be switched off in our systems. They are usually only set in response to actions made by you which amount to a request for services, such as setting your privacy preferences, logging in or filling in forms. You can set your browser to block or alert you about these cookies, but some parts of the site will not then work.

Functional Cookies

These cookies enable the website to provide enhanced functionality and personalisation. They may be set by us or by third party providers whose services we have added to our pages. If you do not allow these cookies then some or all of these services may not function properly.

Performance Cookies

These cookies allow us to count visits and traffic sources so we can measure and improve the performance of our site. They help us to know which pages are the most and least popular and see how visitors move around the site.

Targeting Cookies

These cookies may be set through our site by our advertising partners. They may be used by those companies to build a profile of your interests and show you relevant adverts on other sites. If you do not allow these cookies, you will experience less targeted advertising.

Cookie List

Clear

Apply Cancel
Consent Leg.Interest

Confirm my choices

Powered by onetrus
