# Supplementary material for: A telomere-to-telomere genome assembly of koi carp (Cyprinus carpio) using long reads and Hi-C technology
Source: Gigascience. 2025 Aug 29;14:giaf087. doi: 10.1093/gigascience/giaf087 (PMC12395963; doi:10.1093/gigascience/giaf087)
Supplement: giaf087_Reviewer_1_Report_Original_Submission — László Orbán, Ph.D. -- 1/6/2025 [file giaf087_reviewer_1_report_original_submission.pdf]

Reviewer Report

Title: A telomere-to-telomere genome assembly of koi carp (Cyprinus carpio) using long reads and Hi-C technology

Version: Original Submission **Date:** 1/6/2025

Reviewer name: LÃ¡szlÃ³ OrbÃ¡n, Ph.D.

Reviewer Comments to Author:

Yuan and colleagues have sequenced and assembled the koi carp genome using data generated by several different advanced technologies, including PacBio HiFi, ONT ultralong sequences and Hi-C data. The sequencing data covered the genome over 470 times, resulting in a high-quality assembly (telomer-to-telomer assemblies for over 30 chromosomes, a few gaps, high BUSCO value, etc.). They have investigated the centromeric regions of the assembled pseudochromosomes and analyzed their genic and repeat content.They have also analyzed the expanded gene families, unique genes and positively selected genes in the koi carp genome.The authors propose that these data will provide "an invaluable resource for molecular-guided breeding and genetic enhancement strategies, underscoring the importance of common carp and koi carp in aquaculture and ecological research".The Title covers the essence of the MS well. The flow of the Abstract is clear.The Data Description section is concise and logical, but lacks important data published recently (see e.g. https://doi.org/10.1186/s12915-023-01806-9 and https://doi.org/10.1016/j.aquaculture.2024.741075). Due credit should be given to all others, who produced important and relevant data through the analysis of the common carp genome and transcriptome.The Methods section is detailed, the sequencing and bioinformatic approaches that were taken by the authors are appropriate.The Results are supported by two tables, five figures - four of which contain multiple panels - 14 Supplementary Tables and five Supplementary Figures. The description of data is logical, albeit sometimes potentially misleading (see below).The Conclusions are succinct, but not complete and they do not always place the results into context well.In summary, this Referee recommends a thorough revision of the MS prior to acceptance for publication in Gigascience.Major criticisms:A) In the opinion of this referee, the two most important aspects of this study are: (i) taking an important step towards the pan genome of common carp by improving the quality of the assembly; and (ii) revealing the genetic/genomic factors that led to the evolution of the colored koi carp varieties. In the current version of the MS, the authors do perform comparisons to emphasize the first aspect, but not the second. That leads to mis-interpretation of the data and wrong conclusions. Therefore, the MS should be revised to correct these errors and to help the reader to grasp the second advance better.B) Terminology: The koi carp genome is referred to by several names in the MS, including 'gap-free genome assembly of koi carp', 'Taysho Sensyoku genome', 'final koi carp assembly (CC4.0)', 'gap-free koi carp CC4.0 genome assembly', and 'koi carp CC4.0 genome'. In the referee's opinion, the same name should be used throughout; 'koi carp genome assembly (CC4.0).C) Phylogenetic relationship analysis (P16, L348-351): When the koi carp gene set is compared to those of six other vertebrates, genes that can only be found in the koi carp gene set cannot be called 'koi carp-specific'. The correct term here would be 'common carp-specific'. In order to find genes that are unique

to koi carp the gene content of CC4.0 must be compared to CC3.0. The same refers to expanded and contracted gene families.D) Number formats: English uses decimal points and thousand separators in numbers, not the other way around. Therefore, all numbers in the tables and supplementary tables must be reformatted accordingly. Moreover, numbers in all tables must be flushed to the decimals to help the readers grasp their magnitude.Additional criticisms (in the order of their appearance, not importance):* Page 1, Line 15 vs. P2, L29-30: As correctly noted at the first appearance, koi carp is a variety of common carp. The second sentence must be corrected accordingly.* P4, L79-80: The sex of the individual used for sequencing must be indicated here.* P4, L81-84: Is there a specific reason, why the gonad RNA was not used for transcriptomics?* P12, L245: The correct term is 'positively selected genes'. This must be corrected throughout the MS.* P14, L307 - P15, L312: How do these data compare to those of CC3.0?* P15, L327-329: It is unnecessary to use two decimals in these numbers.* P15, L326 - P16, L341: How do the koi carp centromeres compare to other cyprinid or teleost centromeres? If not available they should be compared to mammalian centromeres.* P16, L331: Erroneous pointer. Please correct to: (Fig.2C).* P17, L358: What proportion of these expansions and contractions are present in the common carp genome (CC3.0)?* P17, L371: What proportion of these 124 genes are positively selected in CC3.0?* P18, L382: Actually, this is the first T2T genome assembly for the whole species that happens to be a koi variety.* P18, L382-390: Centromeric regions and positively selected genes must be mentioned in the Conclusion.* Reference list: The list contains over a dozen incomplete citations that must be completed. This referee recommends that title should not be fully italicized, so that the italicized Latin name would stand out according to the convention. Also, the formatting of reference list must be unified.* Figure 3, Panel A: CC3.0 and CC4.0 should be next to each other.* Supplementary Figure S1, Legend: Grammatical error. Please correct 'divide' to 'divideD'.* Supplementary Figure S3, Legend: Please correct 'abscissa and ordinate' to 'X-axis and Y-axis'.* Supplementary Figure S4: The (grammatically incorrect) title must be removed from the top of the figure. All essential info must be provided by the legend.* Supplementary Figure S4, Legend: Instead of 'blue and green boxes', the figure contains only blue bars. The legend must be revised to reflect the content of the illustration.* Ibid: Grammatical error ('gene families belongs').* Supplementary Figure S5, Legend: The legend must clearly indicate which GO category (Molecular Function, Cellular Component, or Biological Process) is shown on the illustration. The last sentence must be revised to indicate 'diameter of circle' instead of GO term.Suggestion at the discretion of the authors:* Table 1: This is a nice, informative table. The authors should consider extending it with data from earlier version of common carp genome assembly with years indicated to visualize the progression of the field.

Level of Interest

Please indicate how interesting you found the manuscript: Choose an item.

Quality of Written English

Please indicate the quality of language in the manuscript: Choose an item.

Declaration of Competing Interests

Please complete a declaration of competing interests, considering the following questions:

- Have you in the past five years received reimbursements, fees, funding, or salary from an organisation that may in any way gain or lose financially from the publication of this manuscript, either now or in the future?
- Do you hold any stocks or shares in an organisation that may in any way gain or lose financially from the publication of this manuscript, either now or in the future?
- Do you hold or are you currently applying for any patents relating to the content of the manuscript?
- Have you received reimbursements, fees, funding, or salary from an organization that holds or has applied for patents relating to the content of the manuscript?
- Do you have any other financial competing interests?
- Do you have any non-financial competing interests in relation to this paper?

If you can answer no to all of the above, write 'I declare that I have no competing interests' below. If your reply is yes to any, please give details below.

I declare that I have no competing interests

I agree to the open peer review policy of the journal. I understand that my name will be included on my report to the authors and, if the manuscript is accepted for publication, my named report including any attachments I upload will be posted on the website along with the authors' responses. I agree for my report to be made available under an Open Access Creative Commons CC-BY license (http://creativecommons.org/licenses/by/4.0/). I understand that any comments which I do not wish to be included in my named report can be included as confidential comments to the editors, which will not be published.

Choose an item.

To further support our reviewers, we have joined with Publons, where you can gain additional credit to further highlight your hard work (see: https://publons.com/journal/530/gigascience). On publication of this paper, your review will be automatically added to Publons, you can then choose whether or not to claim your Publons credit. I understand this statement.

Yes Choose an item.
