# Supplementary material for: A telomere-to-telomere genome assembly of koi carp (Cyprinus carpio) using long reads and Hi-C technology
Source: Gigascience. 2025 Aug 29;14:giaf087. doi: 10.1093/gigascience/giaf087 (PMC12395963; doi:10.1093/gigascience/giaf087)
Supplement: giaf087_Reviewer_1_Report_Revision_1 — László Orbán, Ph.D. -- 4/20/2025 [file giaf087_reviewer_1_report_revision_1.pdf]

Reviewer Report

Title: A telomere-to-telomere genome assembly of koi carp (Cyprinus carpio) using long reads and Hi-C technology

Version: Revision 1 **Date:** 4/20/2025

Reviewer name: LÃ¡szlÃ³ OrbÃ¡n, Ph.D.

Reviewer Comments to Author:

The authors have revised their MS by taking care of some of the issues identified earlier by this referee. Unfortunately, several issues remained to be unsolved despite assurances given by the authors I their replies.Uncorrected problems identified in the previous round:* B) Terminology: The koi carp genome is referred to by several names in the MS,including 'gap-free genome assembly of koi carp', 'Taisho Sensyoku genome', 'final koi carp assembly (CC4.0)', 'gap-free koi carp CC4.0 genome assembly', and 'koi carp CC4.0 genome'. In the referee's opinion, the same name should be used throughout; 'koi carp genome assembly (CC4.0).Authors' reply: Thanks for your valuable suggestion. We revised the manuscript to standardize the naming of the koi carp genome assembly as "koi carp genome assembly (CC4.0)" as suggested to avoid any confusion.Referee's reply: The term 'Taisho Sensyoku' still appears on several panels of Figure 3. Also, Songpu2021 must be replaced or at least explained in Figures 3&4, as it is not abvious for the non-expert reader that this means the second genome assembly for common carp.* D) This referee requested that all numbers in the tables and supplementary tables must be reformatted according to the rules of English grammar (i.e., decimal points and commas as thousand separators, not the other way around). Moreover, numbers in all tables must be flushed to the decimals to help the readers grasp their magnitude.Authors' reply: "We have carefully revised all tables and supplementary tables to adhere to English numerical formatting standards, usingdecimal points and comma thousand separators (e.g., "748,299" instead of "748299") and aligning numbers by decimal points for clarity."Referee's reply: In reality, the first request, but not the second has been complied with for Tables 1&2. Unfortunately, neither has been complied with for the Supplementary Tables.* Reference list: The list contains over a dozen incomplete citations that must be completed. This referee recommends that title should not be fully italicized, so that the italicized Latin name would stand out according to the convention. Also, the formatting of reference list must be unified.Authors' reply: Thank you for your reminder. We have addressed the reference list issues in the revised manuscript.Referee's reply: Despite the above, the List of References still contains nearly a dozen incomplete references (see e.g., 5, 18, 31, 34, 36, 47, 50, 51, 55). The references are not uniformly formatted (no need for a 'p.' in front of page numbers). The position of the year is unusual in the references> it is typically either behind the authors' names or at the very end of the citation. Latin species names are not italicized.Newly introduced or newly discovered problems:* General: The revised version of the MS still contains several grammatical errors and typos, and it is very difficult to read. Therefore, the revised version should be carefully screened for these or alternatively sent to a professional language-editing and copyediting service before re-submission.* P2, L29-30: Grammatical error. Please correct to "… understanding of the BIOLOGY and adaptation of common carp."* P16, L348-

350 vs. Supp. Table S13: According to the text, there are 589 carp-specific genes in 245 families. On the other hand, Supp. Table S13 mentions 2,578 such genes in 245 families. Which number is correct?* P18, L381-382: Redundant sentences, as SV genes have already been defined on P12, L246-247.* The List of Abbreviations is incomplete: quite a few abbreviations (e.g., GCI, ONT, TRF) are missing.* Table 2: The current version of the table is very difficult to understand. Several columns are without metrics, these must be added to the first row. Numbers in rows #6 - #11 are presumably percentages without indicating the point of reference. Detailed explanation for every category must be provided as notes below the table.* Supp. Tables S4 vs. S5: Chromosomes in Supp. Table S4 are listed as 1-50, whereas those in S5 are listed as A1-25 and B1-25. Please unify throughout the MS.* Supp. Tables S10, S14 and S15: These gene IDs are not useful in their current format. Transcripts should be mapped to RefSeq or another publicly available database to allow the reader to search for the genes, not only the proteins.

Level of Interest

Please indicate how interesting you found the manuscript: Choose an item.

Quality of Written English

Please indicate the quality of language in the manuscript: Choose an item.

Declaration of Competing Interests

Please complete a declaration of competing interests, considering the following questions:

- Have you in the past five years received reimbursements, fees, funding, or salary from an organisation that may in any way gain or lose financially from the publication of this manuscript, either now or in the future?
- Do you hold any stocks or shares in an organisation that may in any way gain or lose financially from the publication of this manuscript, either now or in the future?
- Do you hold or are you currently applying for any patents relating to the content of the manuscript?
- Have you received reimbursements, fees, funding, or salary from an organization that holds or has applied for patents relating to the content of the manuscript?
- Do you have any other financial competing interests?
- Do you have any non-financial competing interests in relation to this paper?

If you can answer no to all of the above, write 'I declare that I have no competing interests' below. If your reply is yes to any, please give details below.

I declare that I have no competing interests

I agree to the open peer review policy of the journal. I understand that my name will be included on my report to the authors and, if the manuscript is accepted for publication, my named report including any attachments I upload will be posted on the website along with the authors' responses. I agree for my report to be made available under an Open Access Creative Commons CC-BY license (http://creativecommons.org/licenses/by/4.0/). I understand that any comments which I do not wish to be included in my named report can be included as confidential comments to the editors, which will not be published.

Choose an item.

To further support our reviewers, we have joined with Publons, where you can gain additional credit to further highlight your hard work (see: https://publons.com/journal/530/gigascience). On publication of this paper, your review will be automatically added to Publons, you can then choose whether or not to claim your Publons credit. I understand this statement.

Yes Choose an item.
