# Supplementary material for: A telomere-to-telomere genome assembly of koi carp (Cyprinus carpio) using long reads and Hi-C technology
Source: Gigascience. 2025 Aug 29;14:giaf087. doi: 10.1093/gigascience/giaf087 (PMC12395963; doi:10.1093/gigascience/giaf087)
Supplement: giaf087_Reviewer_1_Report_Revision_2 — László Orbán, Ph.D. -- 5/23/2025 [file giaf087_reviewer_1_report_revision_2.pdf]

Reviewer Report

Title: A telomere-to-telomere genome assembly of koi carp (Cyprinus carpio) using long reads and Hi-C technology

Version: Revision 2 **Date:** 5/23/2025

Reviewer name: LÃ¡szlÃ³ OrbÃ¡n, Ph.D.

Reviewer Comments to Author:

The authors have revised their MS again, but still left several problems identified earlier by this referee uncorrected. This referee is wondering, why several issues remained to be unsolved over two rounds of revision despite assurances given by the authors about the opposite. This referee has no other choice than returning the MS and asking for the corrections for the third time.In order to help the authors, the remaining issues are now broken down to an itemized point-by-point list containing more detailed explanations regarding the tasks.* Using the term 'Taisho Sensyoku genome': This referee requested that the same name should be used for the koi carp genome assembly throughout the MS. The authors agreed and claimed that 'koi carp genome assembly (CC 4.0)' is now used everywhere. Yet, the second revised version still contains 'Taisho Sensyoku genome' (P4, L88) and 'BUSCO assessment of Taisho Sensyoku' (P34, L739).* Numbers in Supplementary Tables: This referee noticed earlier that numbers in many of these tables contained decimal commas and periods as thousand indicators, whereas English language is using decimal points and commas as thousand indicators. The authors claimed that all these have been corrected. In reality, most supplementary tables (Supp. Tables S1-S11) of the second revised version still contain these errors.* Aligning numbers to the decimal point in Tables and Supp. Tables: This referee requested repeatedly that numbers in many of these tables should be aligned to the decimal points, as this allows the reader to grasp their magnitude quicker. contained decimal commas and periods as thousand indicators. Although the authors claimed that this has been done, numbers in both Tables and most supplementary tables (Supp. Tables S1-S11) are still not aligned properly.* Supplementary Tables S13: Please eliminate the duplication (genes genes) from the top of the last column.* Supplementary Tables S14 and S15: Please replace 'Nr' with 'NCBI non-redundant (protein) database' on the top of the second column of both Tables.* P21, L364: Please replace the word 'genome research on rice' with 'genome assembly of rice'.* P22, L383: Please remove the word 'tissue' from this sentence. The revised sentence should read as follows: '…negative effects on gene expression in scales, whereas no such impacts were observed in fins…'.* P22, L388-389: The term 'including' typically indicates an incomplete list of a larger group. Here, all the sex species are listed, therefore, the sentence should read as follows: '… species (P. huangchuchieni, C. auratus, D. rerio, O. latipes, S. grahami, and H. sapiens) were…'.* P24, L430: The term 'DNA and protein processes' is very general. The authors should replace it with more specific collective term(s).* List of References: This referee requested that all references on the list must be complete with unique IDs. The authors claimed that it has been done. Yet, BioRxiv references #18 and #62 do not have doi IDs, whereas #31 cannot be found with this title on arXiv.

Level of Interest

Please indicate how interesting you found the manuscript: Choose an item.

Quality of Written English

Please indicate the quality of language in the manuscript: Choose an item.

Declaration of Competing Interests

Please complete a declaration of competing interests, considering the following questions:

- Have you in the past five years received reimbursements, fees, funding, or salary from an organisation that may in any way gain or lose financially from the publication of this manuscript, either now or in the future?
- Do you hold any stocks or shares in an organisation that may in any way gain or lose financially from the publication of this manuscript, either now or in the future?
- Do you hold or are you currently applying for any patents relating to the content of the manuscript?
- Have you received reimbursements, fees, funding, or salary from an organization that holds or has applied for patents relating to the content of the manuscript?
- Do you have any other financial competing interests?
- Do you have any non-financial competing interests in relation to this paper?

If you can answer no to all of the above, write 'I declare that I have no competing interests' below. If your reply is yes to any, please give details below.

I declare that I do not have competing interests.

I agree to the open peer review policy of the journal. I understand that my name will be included on my report to the authors and, if the manuscript is accepted for publication, my named report including any attachments I upload will be posted on the website along with the authors' responses. I agree for my report to be made available under an Open Access Creative Commons CC-BY license (http://creativecommons.org/licenses/by/4.0/). I understand that any comments which I do not wish to be included in my named report can be included as confidential comments to the editors, which will not be published.

Choose an item.

To further support our reviewers, we have joined with Publons, where you can gain additional credit to further highlight your hard work (see: https://publons.com/journal/530/gigascience). On publication of this paper, your review will be automatically added to Publons, you can then choose whether or not to claim your Publons credit. I understand this statement.

Yes Choose an item.
