# Supplementary material for: A telomere-to-telomere genome assembly of koi carp (Cyprinus carpio) using long reads and Hi-C technology
Source: Gigascience. 2025 Aug 29;14:giaf087. doi: 10.1093/gigascience/giaf087 (PMC12395963; doi:10.1093/gigascience/giaf087)
Supplement: giaf087_Reviewer_2_Report_Original_Submission — Yoshihiro Omori -- 2/4/2025 [file giaf087_reviewer_2_report_original_submission.pdf]

Reviewer Report

Title: A telomere-to-telomere genome assembly of koi carp (Cyprinus carpio) using long reads and Hi-C technology

Version: Original Submission **Date:** 2/4/2025

Reviewer name: Yoshihiro Omori

Reviewer Comments to Author:

In this paper, the authors describe T2T genome assembly of an ornamental-colored common carp (Cyprinus carpio) strain, Taisho Sanshoku. The assembly achieved a total size of 1555.86 Mb with a contig N50 of 30.45 Mb, comprising 50 gap-free pseudochromosomes. The BUSCO completeness score reached 99.20%. In this assembly, 83% of telomeres were detected, suggesting that 33 chromosomes possess complete telomeres. The findings are relatively descriptive; however, these data are useful for researchers in this field and suitable for publication in this journal after revisions. Several essential concerns should be addressed.1. The authors did not explain what is "Taisho Sanshoku" koi carp strain. It is a Nishikigoi strain that was established in the 19th century in Niigata Prefecture in Japan. A review written by EK Balon describes the origin of them. The authors should add an explanation of this strain and its origin in the introduction and refer to this.Balon, Eugene K. "The common carp, Cyprinus carpio: its wild origin, domestication in aquaculture, and selection as colored Nishikigoi." Guelph Ichthyology Reviews 3 (1995).2. It is better to discuss allotetraploidy in common carp and goldfish, a species very closely related to common carp. The following papers that investigated allotetraploid in goldfish would help the understudying for readers.Zelin Chen et al., Science Advances 2019 Vol 5, Issue 6 DOI: 10.1126/sciadv.aav0547Tetsuo Kon et al., Current Biology 2020 doi.org/10.1016/j.cub.2020.04.034Jing Luo et al., Science Advances 2020 Vol 6, Issue 22 DOI: 10.1126/sciadv.aaz76773. The authors compared their new assembly (CC 4.0) and previously published assembly (CC 3.0) in Table 1. In addition, it is better to compare the previous assembly shown in Ref 6 (Li et al. 2021). This comparison would highlight the progress of this paper.4. The authors should compare them and discuss the differences between Taisho Sanshoku and Songupu strains.5. The gene annotation file (GFF3 file) shows gene IDs (i.e. B23G0250) that the authors defined in this paper. These gene codes should link the gene names and gene symbols. This list should be added in a new Supplementary Table.6. It is useful to add gene symbols in the GFF3 file. It is useful for readers.7. At least the gene annotation file (gff3) and genome assembly file (fasta file) should be deposited on the public database such as Figshare. It should show in "Data Availability".

Level of Interest

Please indicate how interesting you found the manuscript: Choose an item.

Quality of Written English

Please indicate the quality of language in the manuscript: Choose an item.

Declaration of Competing Interests

Please complete a declaration of competing interests, considering the following questions:

- Have you in the past five years received reimbursements, fees, funding, or salary from an organisation that may in any way gain or lose financially from the publication of this manuscript, either now or in the future?
- Do you hold any stocks or shares in an organisation that may in any way gain or lose financially from the publication of this manuscript, either now or in the future?
- Do you hold or are you currently applying for any patents relating to the content of the manuscript?
- Have you received reimbursements, fees, funding, or salary from an organization that holds or has applied for patents relating to the content of the manuscript?
- Do you have any other financial competing interests?
- Do you have any non-financial competing interests in relation to this paper?

If you can answer no to all of the above, write 'I declare that I have no competing interests' below. If your reply is yes to any, please give details below.

I declare that I have no competing interests.

I agree to the open peer review policy of the journal. I understand that my name will be included on my report to the authors and, if the manuscript is accepted for publication, my named report including any attachments I upload will be posted on the website along with the authors' responses. I agree for my report to be made available under an Open Access Creative Commons CC-BY license (http://creativecommons.org/licenses/by/4.0/). I understand that any comments which I do not wish to be included in my named report can be included as confidential comments to the editors, which will not be published.

Choose an item.

To further support our reviewers, we have joined with Publons, where you can gain additional credit to further highlight your hard work (see: https://publons.com/journal/530/gigascience). On publication of this paper, your review will be automatically added to Publons, you can then choose whether or not to claim your Publons credit. I understand this statement.

Yes Choose an item.
